# How Wastewater Reflects Human Metabolism—Suspect Screening of Pharmaceutical Metabolites in Wastewater Influent

**DOI:** 10.1021/acs.est.4c00968

**Published:** 2024-05-24

**Authors:** Corina Meyer, Michael A. Stravs, Juliane Hollender

**Affiliations:** † Eawag: Swiss Federal Institute of Aquatic Science and Technology, Ueberlandstrasse 133, 8600 Duebendorf, Switzerland; ‡ Institute of Biogeochemistry and Pollutant Dynamics, ETH Zurich, Universitaetstrasse 16, 8092 Zurich, Switzerland

**Keywords:** drug metabolites, human pharmaceutical metabolism, suspect screening, wastewater, high resolution mass spectrometry, SIRIUS/CSI:FingerID, MetFrag, molecular network

## Abstract

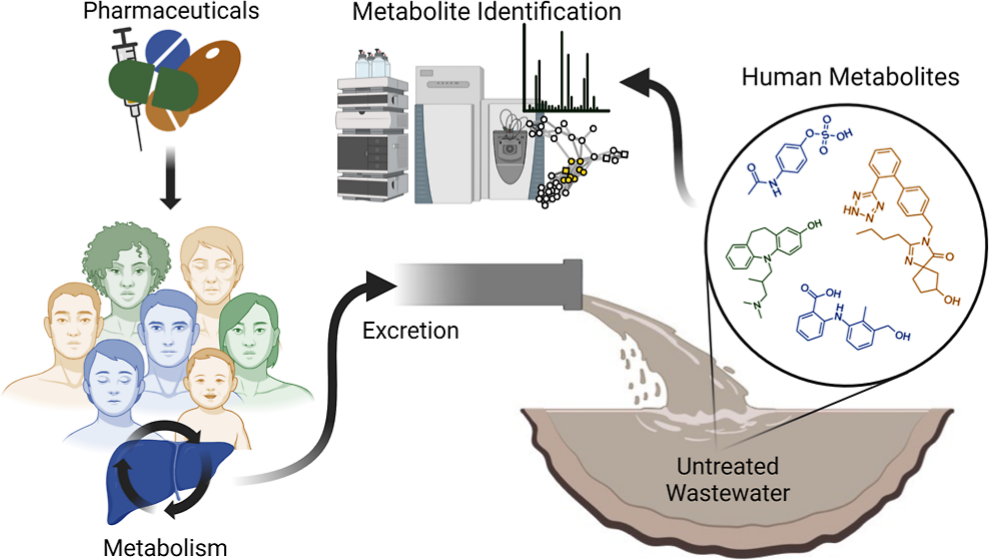

Pharmaceuticals and their human metabolites are contaminants of emerging concern in the aquatic environment. Most monitoring studies focus on a limited set of parent compounds and even fewer metabolites. However, more than 50% of the most consumed pharmaceuticals are excreted in higher amounts as metabolites than as parents, as confirmed by a literature analysis within this study. Hence, we applied a wide-scope suspect screening approach to identify human pharmaceutical metabolites in wastewater influent from three Swiss treatment plants. Based on consumption amounts and human metabolism data, a suspect list comprising 268 parent compounds and over 1500 metabolites was compiled. Online solid phase extraction combined with liquid chromatography coupled to high-resolution tandem mass spectrometry was used to analyze the samples. Data processing, annotation, and structure elucidation were achieved with various tools, including molecular networking as well as SIRIUS/CSI:FingerID and MetFrag for MS2 spectra rationalization. We confirmed 37 metabolites with reference standards and 16 by human liver S9 incubation experiments. More than 25 metabolites were detected for the first time in influent wastewater. Semiquantification with MS2Quant showed that metabolite to parent concentration ratios were generally lower compared to literature expectations, probably due to further metabolite transformation in the sewer system or limitations in the metabolite detection. Nonetheless, metabolites pose a large fraction to the total pharmaceutical contribution in wastewater, highlighting the need for metabolite inclusion in chemical risk assessment.

## Abstract 2

Several pharmaceutical metabolites were identified for the first time by a comprehensive suspect screening in wastewater influent. In combination with consumption and excretion data of pharmaceuticals, the relevance of the exposure of metabolites compared to their parents was investigated.

## 1 Introduction

Pharmaceuticals are becoming increasingly important for enhancing quality of life and decreasing mortality. With the treatment of age-related and chronic diseases as well as changes in clinical practices, worldwide consumption is constantly increasing.^1^ After excretion from the human body, pharmaceuticals and human metabolites thereof enter wastewater treatment plants (WWTPs). Wastewater monitoring can give an impression of a society’s consumption pattern and how it impacts the aquatic environment. However, current monitoring approaches are mostly based on target screening, which focus on a small set of parent pharmaceutical compounds and much less on metabolites.^2^

Metabolites of pharmaceuticals are formed in the human body in order to increase polarity and facilitate excretion. Metabolism is divided into phase I and phase II reactions. Phase I metabolism involves the introduction of a new functional group or the modification of an existing functional group in the molecule, often by oxidation or hydrolysis. Conjugation reactions with endogenous substances like sulfate, glucuronic acid or glutathione are classified as phase II metabolism and can occur directly at the parent compound or follow phase I reactions.^3^ Renal and fecal excretion remove unchanged parent drug and metabolites from the human body before they enter the sewer system and arrive in WWTPs.

The lack of reference standards for pharmaceutical metabolites impedes a broad target analysis in influent wastewater and receiving water bodies. However, high resolution tandem mass spectrometry (HRMS/MS) in combination with automated data processing enables suspect and nontarget screening approaches and has been successfully applied in previous environmental analytics.^4,5^ In short, a compiled suspect list comprising anticipated compounds from literature search or *in silico* prediction tools is checked against full-scan HRMS data based on exact mass. The resulting suspect hits are examined for plausibility, including isotope pattern, presence in background, ionization potential, MS2 fragmentation and retention time (RT). Obtained MS2 fragments can be compared to libraries like MassBank Europe,^6^ mzCloud,^7^ and NIST.^8^ Additionally, machine learning tools such as SIRIUS/CSI:FingerID^9,10^ or *in silico* prediction tools like MetFrag^11^ can be applied for rationalizing MS2 fragmentation. Furthermore, molecular networking, originally a tool implemented in metabolomics and natural product identification,^12^ well-known from the Global Natural Products Social Molecular Networking,^13^ has been extended into environmental analytics for the identification of related compounds.^14^ Its principle relies on the assumption that structurally similar compounds will share similar MS2 fragment ions. By clustering precursor ions with similar MS2 spectra together, unknown structures can quickly be identified if known structures are present in the same cluster.^13^ Since structures of metabolites and transformation products are generally similar to those of their parents,^15^ and reference standards of parent compounds are often available, molecular network strategies can be employed. Nonetheless, a reference standard is crucial for obtaining full confidence in the identification of a suspect hit. If reference material is not available, further confidence can be gained by comparing *in vitro* generated metabolites from their respective parent compounds, for example by employing human liver S9,^16^ with suspect hits.

In this study, we aimed to screen wastewater influent in a wide-scope manner for the presence of more than 250 parent pharmaceutical compounds and over 1500 human metabolites. Parent compounds were selected based on their consumption amounts in Switzerland, while the corresponding metabolites were compiled from human biomonitoring studies. Compounds for which a reference standard was available in-house were covered by a targeted approach, encompassing 132 parents and 77 metabolites. The remaining compounds were analyzed by suspect screening. The obtained samples from three Swiss WWTPs were processed with an analytical workflow optimized for matrix-rich samples, including online solid phase extraction (SPE), reversed phase separation and HRMS/MS detection after electrospray ionization (ESI). The suspect screening workflow encompassed machine learning (SIRIUS/CSI:FingerID^9,10^) and *in silico* prediction tools (MetFrag^11^), as well as molecular networking to gain further confidence in metabolite identification. Confirmation with reference material was carried out for candidates if possible. If no reference standard was available, confidence was increased by human liver S9 incubation experiments. Semiquantification was performed based on ionization efficiency predictions with the machine learning tool MS2Quant^17^ to determine metabolite to parent concentration ratios.

## 2 Materials & Methods

### 2.1 Chemicals and Solvents

Details on chemicals, including 91 isotope labeled internal standards (ILIS), target reference materials (216 parents, 89 metabolites), suspect reference materials (6 parents, 49 metabolites, 8 others), and solvents used in this study are given in SI-A2/5/6/9.

### 2.2 Wastewater Sample Collection

Wastewater influent samples originated from three Swiss WWTPs, encompassing Altenrhein, Neugut (Duebendorf) and Werdhoelzli (Zurich). They treat population equivalents of 83,000, 105,000 and 670,000 with industry contributions of 23%, 52%, and 30%, respectively. Influent wastewater samples were collected before the primary clarifier from Monday, February 28 to Friday, March 4, 2022, as 24 h composite samples. Sampling devices from MAXX (MAXX Mess-u. Probenahmetechnik GmbH, Germany) were preinstalled from the operators in Altenrhein and Werdhoelzli for flow-proportional sampling, while in Neugut, a MAXX TP5 C Active autosampler was positioned for time-proportional sampling. Collected samples were stored at 4 °C within the sampler. The samples were transferred to borosilicate glass bottles (previously annealed at 500 °C; 1 L bottles, Duran, Germany or SIMAX Kavalier, Czech Republic) within 10–72 h of sample collection, transported to the laboratory and prepared within 2 h. No impact of the duration until sample preparation on the results was observed.

### 2.3 Wastewater Sample Preparation

Triplicate 50 mL aliquots of wastewater were transferred into 50 mL centrifuge tubes (Corning, U.S.), followed by the addition of 20 μL of 1 mg/L ILIS solution. Samples were centrifuged at 3000 rcf and 4 °C for 10 min. The supernatant was transferred to 250 mL borosilicate bottles (previously annealed at 500 °C; 1 L bottles, Duran, Germany or SIMAX Kavalier, Czech Republic), and 120 mL of ultrapure water were added. The prepared samples were stored at 4 °C for up to 10 days until analysis. After analysis, prepared samples were stored at −20 °C and used later for spiking experiments with purchased reference standards. A schematic overview on the sample preparation is given by Figure SC6.

### 2.4 Human Liver S9 Incubation

The method for human liver S9 incubation was adapted from previous studies on phase I metabolite generation.^16,18^ In short, human liver S9 (Thermo Scientific, U.S.), containing six pooled human liver S9 fractions of mixed gender and reduced nicotinamide adenine dinucleotide phosphate solution were mixed with TRIS buffer at a pH of 7.4 in a 96-well plate. Standard solutions containing single parent compounds in organic solvent were added to individual wells. The incubation was conducted for 3 h at 37.5 °C under constant shaking and was stopped by the addition of cold methanol. Details can be found in SI-C2.3.

### 2.5 Online-SPE-HPLC-HRMS/MS Analysis

All samples were analyzed using automated online SPE followed by reversed phase liquid chromatography (LC) coupled via ESI to a high-resolution mass spectrometer (HRMS/MS). Sample aliquots of 20 mL were transferred into 20 mL headspace amber glass vials, before enrichment using a multilayer cartridge with reversed phase and ion exchange material. Chromatographic separation was achieved on a reversed-phase C18 column at 30 °C (Atlantis T3, 3 μm, 3.0·150 mm, Waters, U.S.). The HPLC system consisted of a PAL autosampler (CTC Analytics, Switzerland) and a Dionex UltiMate3000 RS pump (Thermo Fisher Scientific, U.S.). In the positive ionization mode, ultrapure water and methanol (each with 0.1% formic acid) were used as mobile phases, while the negative mode used 90:10 and 10:90 water/methanol mixtures with 5 mM ammonium formate. The flow rate was 300 μL/min.

Mass spectra were acquired separately in ESI positive and negative mode on a high resolution mass spectrometer (Q Exactive, Thermo Fisher Scientific, U.S.). Full scan MS1 acquisition (m/z 100–1000) was performed with a mass resolution of 140,000 at 200 m/z, followed by five data-dependent MS/MS scans with a resolution of 17,500 at 200 m/z using higher energy collision-induced dissociation and an isolation window of 1 Da. MS/MS acquisitions were triggered by m/z of suspect ions with normalized collision energies from 15 to 120, depending on m/z (see SI-C2.4). If no suspect was detected, the five most intense ions present in the MS1 spectrum were fragmented. For confirmation of suspects with reference materials, some samples were remeasured with adjusted MS/MS settings. More details about the analytical procedure and parameters of LC-HRMS/MS are available in SI-C2.4.

### 2.6 Target Quantification

TraceFinder 5.1 (Thermo Scientific, U.S.) was used for the quantification of 304 target analytes with an extraction window of 5 ppm. Sample analyte detections were confirmed by comparison of RT and MS/MS fragments from reference standards. Sample analytes were quantified with a linear calibration curve (1/X weighting) using peak area ratios of analyte and ILIS. If no structurally identical ILIS was available (80% of targets), an in-house R^19^ script (https://github.com/dutchjes/TFAnalyzeR/blob/master/RelativeRecoveryCalculation.R) was used to select an ILIS eluting at similar RT that resulted in the best relative recovery (close to 100%).^5^ Concentrations of compounds without structurally identical ILIS were corrected by their relative recoveries. Details on the quantification method and quality control are given in SI-C2.5, while target and internal standard chemicals are listed in SI-A2 and SI-A6.

### 2.7 Suspect Screening

#### 2.7.1 Suspect List

The suspect list was generated based on consumption amounts of pharmaceutical compounds in Switzerland from 2014 to 2016 available through the Swiss Federal Offices for the Environment. Pharmaceuticals with an average consumption of over 100 kg in these 3 years as well as those which showed a year-over-year increase in application of at least 10 kg were selected. For the resulting 268 prioritized compounds, the human phase I and phase II metabolites were searched in literature. Swiss Compendium^20^ and DrugBank^21^ served as a basis and were complemented by additional literature, leading to over 1500 metabolites. The complete suspect list is contained in SI-A1.

#### 2.7.2 Raw Data Preprocessing

In advance, five tools (Compound Discoverer, MZmine 3,^22^ MS-DIAL,^23^ SLAW,^24^ and enviMass^25^) were tested for the backbone processing steps peak picking, RT alignment, grouping of isotopologues and adducts to form components, gap filling, as well as grouping of components across samples, based on 49 ILIS signals on a small subdata set. Individual parameters for each tool were manually optimized, except for SLAW, where an automatized parameter optimization is included. The tools were evaluated methodically according to false positive and false negative rates as well as computation times, and compared in terms of additional annotation features (annotation with suspect list and MS2 libraries, molecular networking, neutral loss search, specific MS2 fragment search) and user-friendliness. Details on the procedure and the results are provided in SI-C1. Considering the aforementioned criteria, Compound Discoverer was assessed to be the most suitable. Correspondingly, raw data preprocessing for the complete data set was achieved with Compound Discoverer 3.3 (CD 3.3, Thermo Fisher Scientific, U.S.), with the mentioned backbone processing steps. Figure SC11 shows the applied CD 3.3 workflow, while Tables SC38–SC56 contain detailed parameter settings for each workflow node. After the preprocessing, additional annotation steps were performed, including mass list search with the generated suspect list, MS/MS spectra database comparison with mzCloud,^7^ EU MassBank^6^ and NIST^8^ libraries and molecular formula prediction.

#### 2.7.3 Suspect Filtering and Prioritization

Components obtained from raw data preprocessing were prioritized depending on the following criteria: (i) suspect list match based on exact mass; (ii) acquired MS2 spectrum; (iii) peak intensity > 10^5^; (iv) elution after the dead time (4 min ≤ RT ≤ 30 min); and (iv) sufficient peak shape by peak rating within Compound Discoverer and manual inspection. The resulting components were stored and MS2 spectra exported in the msp file format for further structural elucidation.

#### 2.7.4 MS2 Spectra Analysis

Processing and computational analysis of MS2 spectra relied on library match conducted within Compound Discoverer and three additional tools, which were used complementarily. The exported msp file containing MS2 spectra was directly used as input for SIRIUS/CSI:Finger ID 5.7, a machine learning-based tool for *in silico* compound identification from MS2 spectra^9,10^ and MetFrag, an *in silico* fragmenter based on bond energies.^11^ SIRIUS/CSI:FingerID was used directly with the graphical user interface, while MetFrag was set up on a local instance of the Galaxy server, an open source, web-based bioinformatics platform.^26^ The third tool, FISh scoring, was applied within CD 3.3. FISh scoring is only able to rationalize fragments based on an assigned putative molecular structure, here of the allocated suspect from the suspect list, while SIRIUS/CSI:FingerID computes a fragmentation tree which is used to predict molecular fingerprints that are searched against the entire PubChem database. In contrast, MetFrag was run with two local databases separately, on one hand the suspect list, on the other hand, PubChemLite,^27^ enabling benchmarking of the resulting FragmenterScore from the suspect list (see SI-A3).

#### 2.7.5 Molecular Network

A molecular network of the components with exact masses matching the suspect list was generated within CD 3.3. Parameters like cluster size, number of node links, number of matching fragments and spectral match were adapted to identify relevant clusters. Nodes and links data were exported in the json file format and visualized with the igraph package in R.^28,29^

#### 2.7.6 RT Prediction

RT prediction was performed based on the previous target screening. LogD_OW_ values of 369 compounds in positive and 81 in negative ionization mode at the respective pH of chromatography (2.7 for positive, 4.8 for negative) were predicted by JChem^30^ and plotted against the measured retention times in the influent samples. A linear model was established, allowing RT prediction of suspects based on their predicted logD_OW_ values. The two linear models are shown in Figures SC14 and SC15 and exhibit 95% confidence intervals of ±4.5 to 4.7 min and ±7.0 to 7.2 min.

#### 2.7.7 Confidence Communication

Identification confidence is given as confidence levels based on the level scheme by Schymanski et al.^31^ Moreover, the harmonized identification score by Alygizakis et al.^32^ was applied, with an adaptation to use the computed RT prediction model (see above) instead of calibrated RT indices,^33^ which were not available on the system. More details to the scoring system are given in SI-C2.11 and the R code is available on GitLab (https://gitlab.com/Corina_Meyer/confidence-score-suspect-identification-with-lc-msms).

#### 2.7.8 Suspect Confirmation

Reference materials of candidates where the structure was identified with sufficient confidence, either by comparison to library spectra or by MS2 spectra prediction in combination with molecular networking and RT prediction, were purchased if possible. Several samples were remeasured with adjusted MS/MS settings, together with spiked samples and reference standard solutions. Retention times and MS/MS fragments of the candidates in sample, spiked sample and standard were compared and visualized, using the R packages MSnbase^34^ and MSMSsim.^35^ A match between suspect and reference standard led to an increase of the confidence to level 1. If no match was observed, the confidence was decreased to level 4 if a unequivocal molecular formula was available, otherwise it was decreased to level 5.

For suspect hits with no commercially available reference material, an alternative strategy was applied to gain further confidence. Respective parent compounds were incubated with human liver S9 to form human metabolites *in vitro*(16) (see Section 2.4). The resulting raw data from the LC-HRMS/MS measurement were analyzed in CD 3.3 using the same preprocessing as for the wastewater samples. Formed metabolites were prioritized based on matching m/z (±5 ppm) and an extended RT window of ± half a minute due to the strong and differing matrix of wastewater and human liver S9 extracts. In case of a MS2 spectral match, the confidence level of the suspect was increased to level 2b, due to the obtained diagnostic evidence.

#### 2.7.9 Semiquantification

Semiquantification was performed differently for compounds in the positive and negative ionization mode. For suspects ionizing in the positive mode, MS2Quant, a machine learning model based on ionization efficiency data was applied.^17^ Structural fingerprints required for ionization efficiency value prediction were directly computed from the proposed chemical structure of the suspects. As calibrants, the previously analyzed targets were used, excluding 15 compounds for model assessment in the three different influent wastewater matrices. The comparison of predicted and measured concentrations revealed a deviation of less than a factor of 2 for 69% of the data points, and an over- or underestimation by a factor of less than four for 18%, while the two latest eluting compounds (13%) were underestimated by 1 order of magnitude. Overall, the model performed well for the test set in positive mode, which can also be attributed to the fact that compounds with high structural similarity were used as calibrants. To further analyze if pharmaceutical metabolites are in the scope for semiquantification with MS2Quant, a principal component analysis (PCA) with the 1191 training data set compounds and the identified suspects was conducted. For this purpose, one-dimensional and two-dimensional Pharmaceutical Data Exploration Laboratory descriptors^36^ were predicted. Figure SC17 shows the resulting PCA. It becomes visible that the suspects lie within the scope of MS2Quant. This finding suggests that semiquantification based on ionization efficiency is also reasonable for the metabolites. In combination with a previously published study, which showed better accuracy for the semiquantification of transformation products based on ionization efficiency than based on parent compounds or close eluting compounds,^37^ it can be concluded that MS2Quant is indeed suitable for the semiquantification of metabolites. More details can be found in SI-C2.12.

However, for compounds ionizing in the negative mode, no trained model was available within MS2Quant. Therefore, semiquantification of the four suspects in the negative ionization mode was conducted based on an external calibration curve in ultrapure water. Intensities of suspects in the samples were taken from the initial measurement because not all samples were remeasured for confirmation and degradation of compounds may have occurred during sample storage. To account for matrix effects, an ILIS close in RT and of similar structure was assigned to each suspect. Differing sensitivities were corrected by determining a sensitivity factor based on the measured average ILIS intensities in the samples and calibration standards. This approach is impaired by the unavailability of structurally identical ILISs and by the complexity of measurement sensitivity correction, leading to uncertainties in the semiquantification results. More details can be found in SI-C2.12, and the obtained concentrations of all compounds can be found in SI-A7.

## 3 Results and Discussion

### 3.1 Literature Analysis of Human Pharmaceutical Excretion for Suspect List Generation

To rationalize the relevance of human pharmaceutical metabolites in wastewater, a literature analysis of the 268 highly consumed parent pharmaceuticals was conducted. These compounds were evaluated in terms of their excretion as parents or metabolites from the human body. For 3% of the pharmaceuticals, pharmacokinetic parameters like distribution and metabolism cannot be applied, since they are not systemically absorbed into the body. For the absorbed pharmaceuticals, three categories are differentiated: (i) parents which experience no metabolism, (ii) parents which are metabolized, but more than half of the administered dose is excreted as unchanged parent compound, and (iii) parents for which at least half of the dose is excreted in the form of metabolites (Figure 2). The majority of pharmaceuticals were found to be excreted as metabolites, with more than half of them in higher amounts as metabolites than as parents. Phase II metabolites which are known to be cleaved back to the parent compounds or preceding phase I metabolites are reported significantly less than phase I metabolites. More details on the conducted literature analysis can be found in SI-B1.

**Figure 1:**
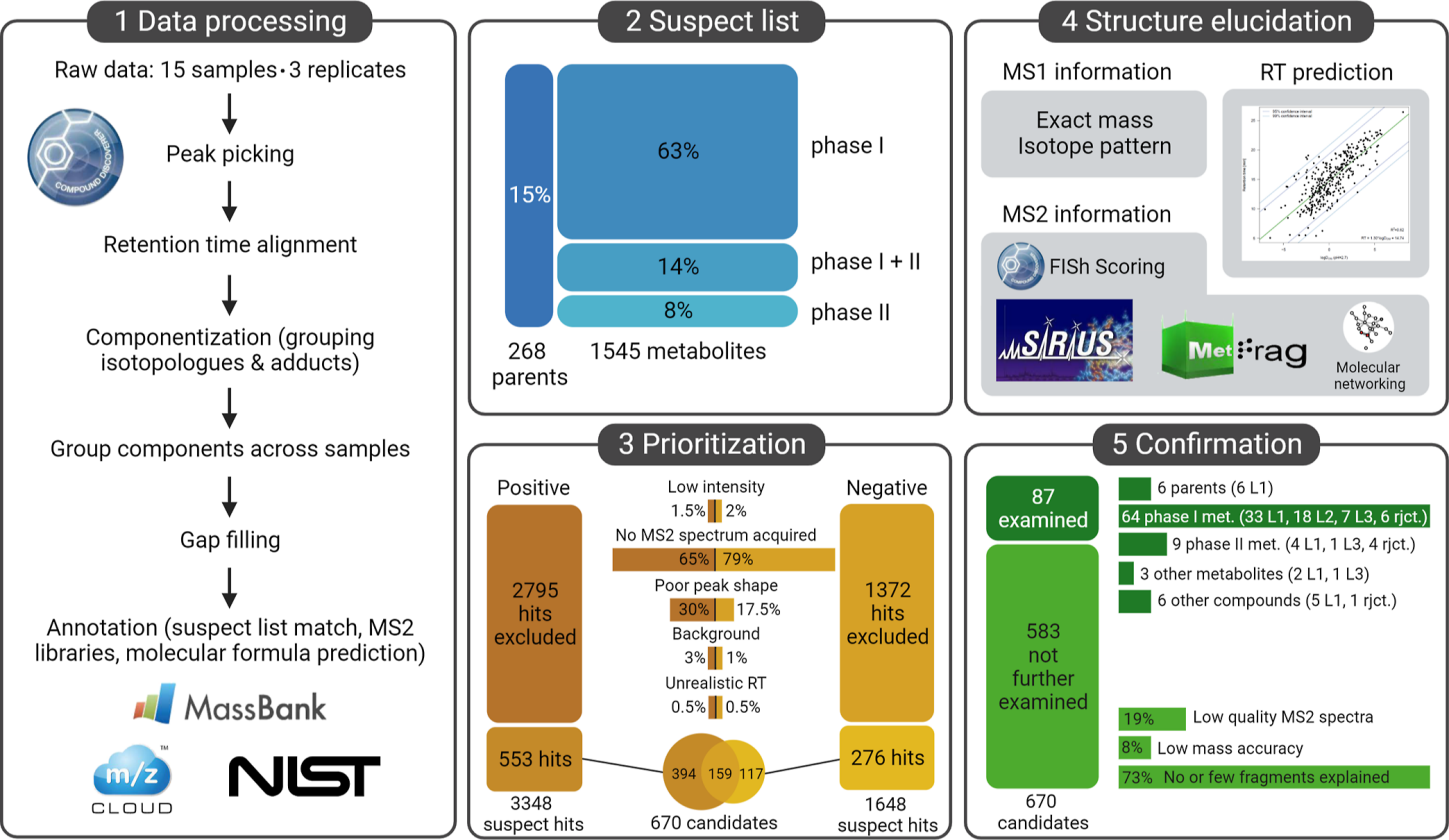
Data preprocessing, including annotation based on MS2 library matching, molecular formula prediction and suspect list matching was conducted (box 1). The compiled suspect list comprised 268 parent pharmaceutical compounds and 1547 metabolites (box 2). Suspect hits were prioritized (box 3) and subjected to structure elucidation using all available information from MS1 and MS2 spectra as well as from RT (box 4). The most promising candidates were compared to reference standards or *in vitro* generated metabolites for confirmation (box 5).

**Figure 2:**
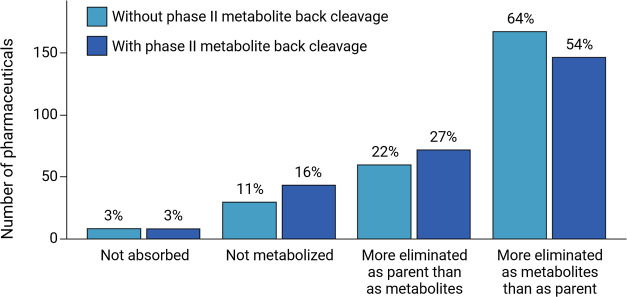
Elimination of 268 parent pharmaceutical compounds from the human body. More than 50% of the compounds are eliminated to a larger extent as metabolites than as parents.

Complementing the analysis of the parent to metabolite excretion, a suspect list with 1545 human metabolites was generated from literature search. The list encompasses the 268 parent pharmaceutical compounds (15%), phase I metabolites (63%), phase II metabolites (8%) and metabolites that have undergone a phase I followed by a phase II reaction (14%) (see Figure 1). The suspect list is publicly available on the NORMAN suspect list exchange (https://www.norman-network.com/?q=suspect-list-exchange, S113). No truncation of the suspect list based on the extent of human excretion, the LC retention or the ionization efficiency was performed, to avoid false negative findings. However, all suspect list compounds, except two, contain at least one oxygen or nitrogen atom, which facilitates ionization by ESI.

### 3.2 Target Screening

To evaluate the applicability of the analytical method for wide-scope suspect screening, target screening and quality control of 132 parents and 77 metabolites comprised in the suspect list was conducted. Additional 84 nonprioritized parents and 11 metabolites were included for a more reliable assessment. The limit of quantification was ≤1 ng/L for 80% of the targets, showing the high sensitivity of the analytical setup comprising online-SPE followed by LC-HRMS/MS analysis. In the 15 wastewater influent samples originating from three WWTPs, 99 prioritized parent pharmaceuticals and 51 metabolites were detected. Individual samples contained 134–141 targets. No appreciable difference in target detection was discovered between the WWTPs during dry weather conditions, indicating regular and widespread consumption of the targeted pharmaceuticals, which is in agreement with our selection criteria. Details on quality control and target screening can be found in SI-C2.5 and SI-A2.

### 3.3 Suspect Screening

#### 3.3.1 Suspect Filtering

Following data processing and prioritization of suspect components based on exact mass, 3348 hits in positive and 1648 hits in negative ionization mode were initially annotated (Figure 1). Subsequent application of exclusion criteria such as low intensity (1.5%/2% omitted in pos/neg mode, respectively), poor peak shape (30%/17.5%), missing MS2 spectra (65%/79%), background filtering (3%/1%), and unrealistic RT (0.5%/0.5%), led to a reduced list of 553 hits in positive and 276 hits in negative ionization mode, of which 159 were detected in both modes. The intensity distribution of the initially annotated suspects with and without acquired MS2 spectra (SI-C3) showed that components without an MS2 spectrum were significantly lower in intensity, indicating on average lower concentrations. Further examination of the remaining 670 unique hits based on molecular networking results, RT prediction, mass accuracy, and analysis with a combination of prediction tools (SIRIUS/CSI:FingerID, MetFrag, FISh Scoring), which included an automatic spectra quality filtering step, yielded a final selection of 87 annotated components.

#### 3.3.2 Suspect Confirmation

To increase the identification confidence of the 87 components, reference standards were purchased for the 51 most promising pharmaceutical parent and metabolite suspects. Overall, 6 parent compounds were confirmed along with 33 phase I metabolites (Level 1), while four phase I metabolites were rejected. For 27 phase I compounds, no reference standard could be purchased or were considered too expensive (Level 2a/3). Human liver S9 incubation led to further diagnostic evidence for 16 phase I metabolites (Level 2b). Four phase II metabolites were confirmed by reference standards and four were rejected. For one phase II metabolite, no reference material was available. The confirmed pharmaceutical metabolites (Levels 1–3) are summarized in Figure 4.

Nine out of 87 components initially presumed to be pharmaceutical metabolites actually were isobaric compounds originating from different sources. Information about the identification process of all compounds (including library and predicted MS2 spectra, molecular networking results and RT prediction) are given in SI-D. MS2 spectra of the confirmed suspects are available on MassBank Europe.^6^

#### 3.3.3 High-Quality Suspect Lists vs Comprehensive Databases

The 78 confirmed suspect components were checked for their presence in PubChem and PubChemLite for exposomics. One metabolite was not contained in PubChem while an additional 22 were not contained in PubChemLite. This means that 23 metabolites could have been missed by analyses based on PubChemLite alone. Moreover, the structures of 11 confirmed metabolites are present in PubChem, but not readily recognizable as pharmaceutical metabolites (e.g., by name or structure) without additional knowledge. Such results would be easily discarded in an approach solely based on databases and highlight the importance of a high-quality suspect list comprising prioritized compounds of interest, for example based on experimentally observed metabolites and transformation products.

#### 3.3.4 Incorporation of LC Information

To complement information from mass spectrometry, physicochemical properties influencing the RT in LC are useful in the identification of compounds. Overall, six suspect candidates had measured retention times lying outside the 99% confidence interval of the predicted retention times. For four of them, including two azithromycin metabolites, *N*-methylpregabalin and 3-hydroxy-cotinine, reference materials were available. However, only 3-hydroxy-cotinine could be confirmed. For the others, the RT of the standard was similar to the predicted RT, but more than 5 min away from the RT of the suspect hit.

For the *O*-dealkylated aliskiren metabolite and the *O*-demethylated and further oxidized metabolite with no available reference standards, human liver S9 incubation yielded no signals with matching exact mass at a similar RT. Moreover, lower retention times compared to the parent are expected, due to the increased polarity reflected in the lower logD_OW,pH2.7_ values, but the RTs for the two suspected metabolites in wastewater were higher than for the parent, leading to a rejection of the candidates. These results highlight the importance of combining all available information from an LC-HRMS/MS measurement. However, the RT prediction method applied in this work is relatively coarse, and the broad confidence intervals only allowed the rejection of few candidates. To further increase RT utility, RT indices,^33^ quantitative structure-(chromatographic) retention relationships (QSRR)^38^ or machine learning tools like MultiConditionRT^39^ can be applied.

#### 3.3.5 Molecular Networking

**Figure 3:**
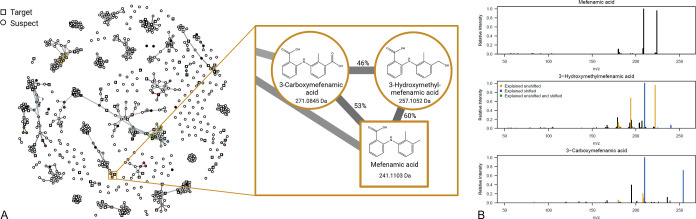
(A) Molecular network of the prioritized suspect hits in positive ionization mode. Colored dots indicate examined hits. The inset shows a cluster consisting of the parent mefenamic acid and two confirmed suspects, 3-carboxymefenamic acid and 3-hydroxymethylmefenamic acid. The numbers next to the connecting lines display the spectral similarity based on the number of matched fragments, including fragments shifted by the mass difference of parent and metabolite. (B) MS2 spectra of mefenamic acid and the two metabolites. Colors in the metabolite spectra indicate fragments that were either found directly in the parent MS2 spectrum or after shifting by the mass difference of parent and metabolite.

Molecular network strategies were useful in the identification of pharmaceutical metabolites in wastewater. One example is mefenamic acid, a nonsteroidal anti-inflammatory drug used to treat mild to moderate pain.^21^ Besides the targeted parent compound, two phase I metabolites were identified by molecular networking. Examination of the molecular network (see Figure 3) had revealed connections between the parent mefenamic acid and two additional components present in wastewater, which were, based on exact mass, suspected to be mefenamic acid metabolites. Further analysis with SIRIUS/CSI:FingerID and MetFrag for rationalizing MS2 spectra led to a putative identification of 3-hydroxymethylmefenamic acid and 3-carboxymefenamic acid. Confirmation of the hydroxylated metabolite was achieved by a reference standard (Level 1), while 3-carboxymefenamic acid was confirmed by human liver S9 incubation (Level 2b). The hydroxylated metabolite was detected in similar concentrations (around 500 ng/L) than the carboxylated metabolite (around 300 ng/L), which was previously reported in wastewater effluent,^40^ indicating the oxidative stability of 3-hydroxymethylmefenamic acid in the sewer system. This example shows that molecular network strategies can indeed be transferred from metabolomics to small molecule metabolite and transformation product identification. Moreover, 75% of the confirmed pharmaceutical metabolites (Levels 1–3) exhibited a connection in the molecular network to its parent or another metabolite originating from the same parent. This indicates that molecular networking is useful in the prioritization of components detected in wastewater.

#### 3.3.6 Limitations

We note that multiple factors can limit the scope of our suspect screening method. First, samples were stored up to 10 days at 4 °C, which can lead to depletion of some compounds. However, sample storage at −20 °C was deliberately avoided, since freezing and thawing processes can lead to pH changes, facilitating certain degradation reactions.^41^ Second, very small or very large metabolites with m/z ratios <100 or >1000 were not considered, since the scan range was set from 100 to 1000 m/z. Moreover, very small and polar compounds may not be retained by the sorbents utilized and can get lost during SPE. In addition, all compounds eluting within the dead time of the LC (first 4 min) were systematically excluded. The same applies to late eluting components (after 30 min). A rapid screening with the inclusion of those early and late eluting components led to only eight additional matches. Another limitation arises from missing MS2 spectra, where from the initially prioritized signals based on exact masses from the suspect list, 65% and 79% of components were excluded in the positive and negative ionization mode, respectively. As previously mentioned, this criterion affected low intensity and therefore low concentrated metabolites more strongly than intense signals (see Figure SC19). For those, the MS2 spectra might be noisy and not very characteristic. Nonetheless, some metabolites may be missed.

An additional complication arises from glucuronidated phase II metabolites. It has been shown that in-source fragmentation during ESI can occur,^42,43^ which can lead to the detection of parents or phase I metabolites at RTs of the glucuronides. However, no signals with the exact mass of the parent were found at other RTs. The fact that glucuronidated metabolites tend to break apart at the site of conjugation during fragmentation was used to specifically identify glucuronide and sulfate metabolites by neutral loss search in the positive and negative ionization mode.^44^ Besides neutral losses, specific MS2 fragment search for glucuronide and glutathione conjugates was conducted in the negative ionization mode (see SI-C2.13). Together, these approaches were able to further support the identification of paracetamol-sulfate (ESI+), tapentadol-*O*-sulfate (ESI+), diphenhydramine-*N*-glucuronide (ESI+) and lamotrigine-*N2*-glucuronide (ESI+), which were detected in all analyzed influent samples with concentrations ranging from 10 to 1000 ng/L. However, these approaches did not lead to additional prioritized signals, since the most relevant phase II metabolites were covered by the suspect list.

### 3.4 Newly Identified Pharmaceutical Metabolites

**Figure 4:**
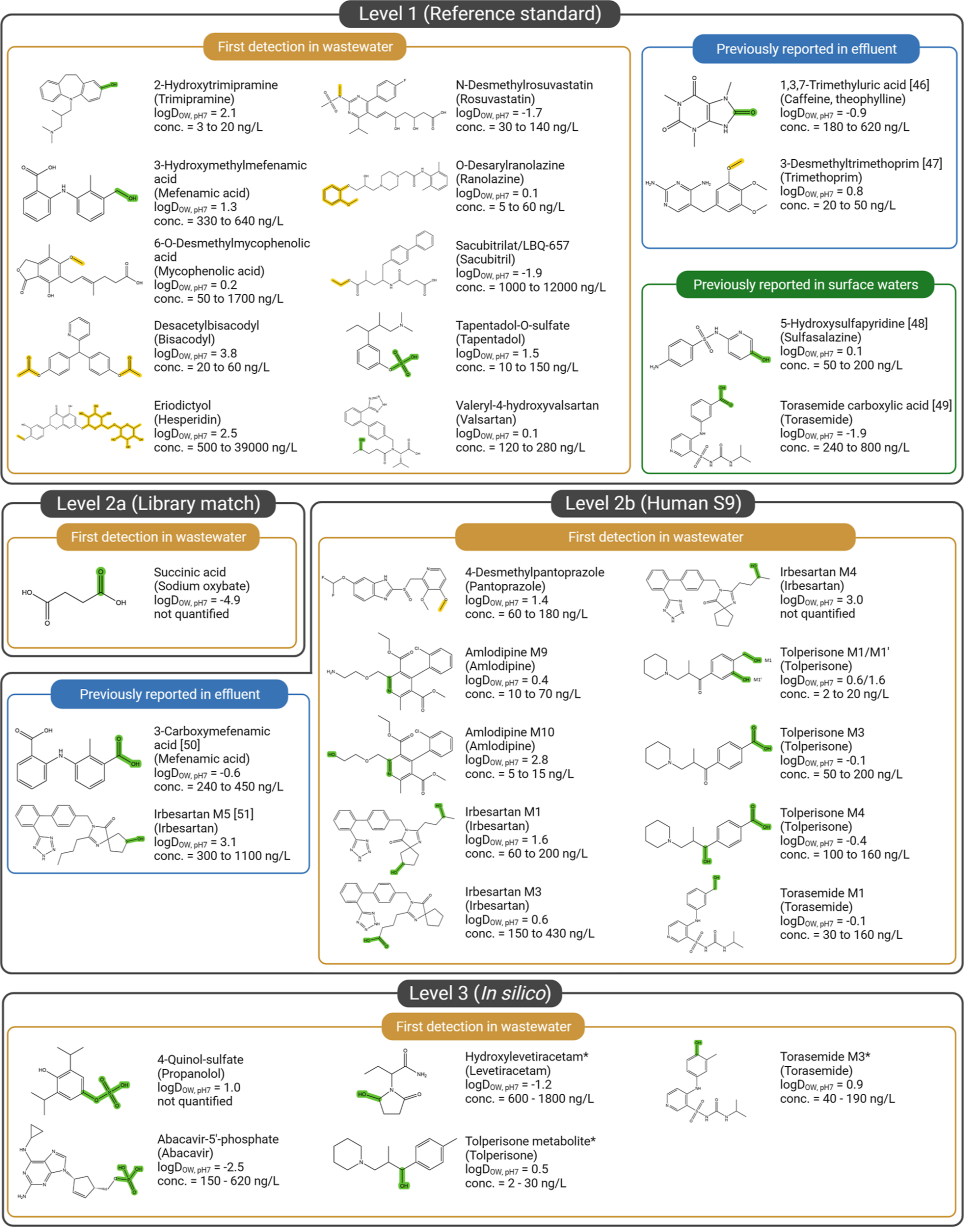
Confirmed pharmaceutical metabolites (levels 1–3) from suspect screening. Functional groups highlighted in green were added to the parent structure, groups highlighted in brown were removed during human metabolism. The name of the respective parent is given in parentheses. Compounds for which a human liver S9 incubation was conducted but the corresponding metabolite was not identified are marked with *. Chemical identifiers for all shown compounds can be found in SI-B3.^40,48−52^

By suspect screening, 64 pharmaceutical metabolites (Levels 1–3) were identified in addition to the 51 detected by target screening. Overall, prominent reaction types are *N*/*O*-dealkylations, dearylations or deacetylations (33%), followed by hydroxylations (21%), formation of carboxylic acids (8%), and ester hydrolyses (6%). A few of these suspects are not only found as a result of pharmaceutical consumption but also have another origin. This includes the metabolites hesperitin and eriodictyol, originating from hesperidin, the main flavonoid in citrus fruit peels;^45^ benzophenone, a UV blocker;^46^ and succinic acid, an endogenous metabolite in cellular respiration.^47^ Of the 64 metabolites, 26 are reported for the first time in wastewater (see Figure 4).

**Figure 5:**
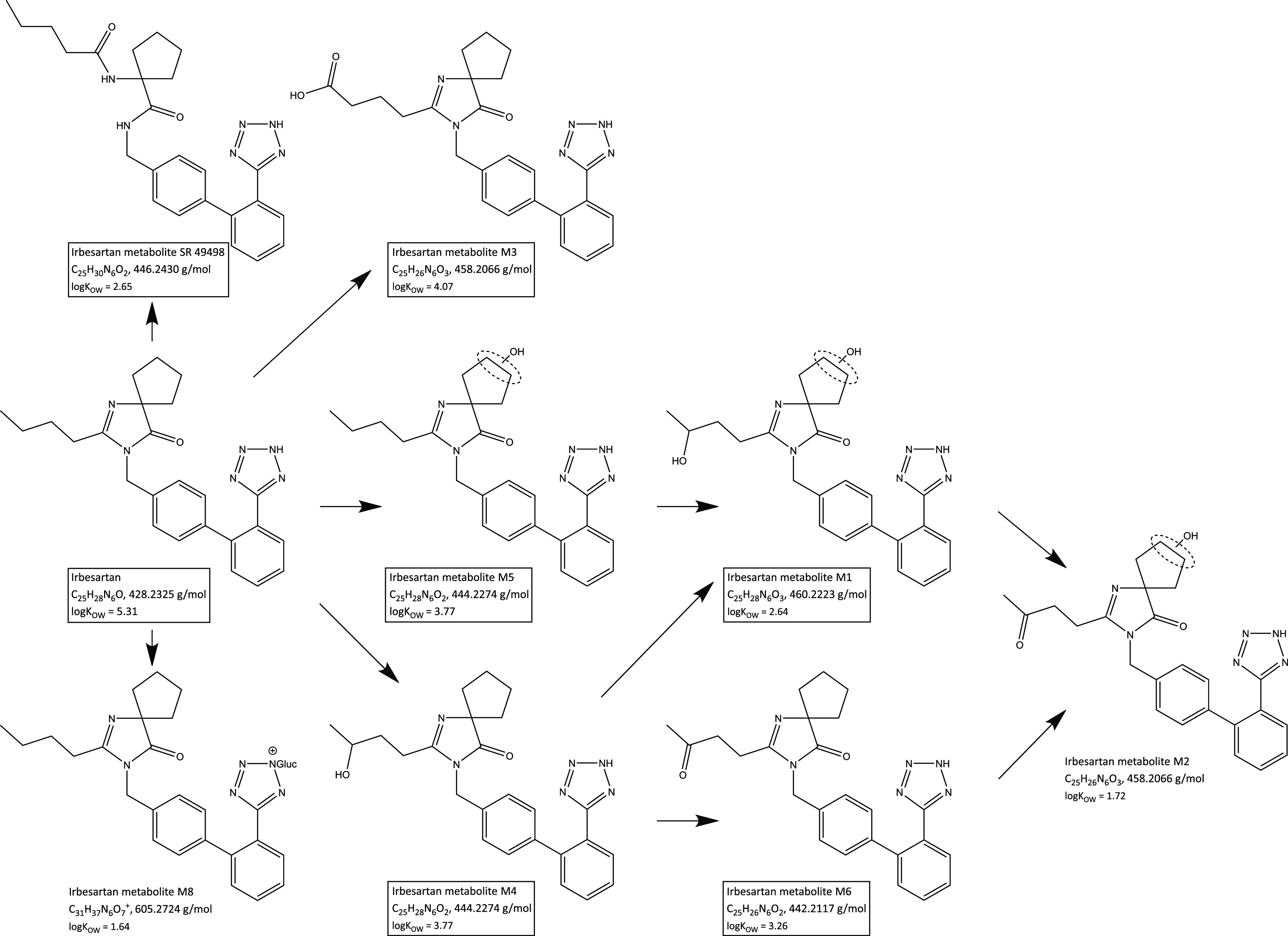
Metabolism scheme of irbesartan, adapted from reference (55). Framed compounds were detected in our study.

Several of the identified compounds are metabolites of sartans, a compound class used for the treatment of hypertension and cardiac insufficiency.^21^ Many of the sartans share a diphenyl group with a tetrazole ring in *ortho* position to the phenyl–phenyl bond as basic core, leading to the well-known phase I metabolite valsartan acid in human metabolism. Besides this metabolite covered in target screening, additional metabolites of the parents irbesartan, losartan and valsartan were found via suspect screening. To the best of our knowledge, we report here the detection of irbesartan metabolites M1 (Level 2b), M3 (Level 2b) and M4 (Level 2b) in wastewater for the first time. In addition, metabolite M5 (Level 2b), which was previously detected in wastewater effluent,^52^ and metabolites M6 (Level 3) and SR49498 (Level 2b), which were previously detected in wastewater influent,^53^ were identified. The highest concentrations were found for metabolite M5 in Altenrhein samples, amounting to over 1000 ng/L. However, all metabolites (see Figure 5) were detected in lower concentrations than the parent, exhibit higher polarity, and have no reported activity.^54^

**Figure 6:**
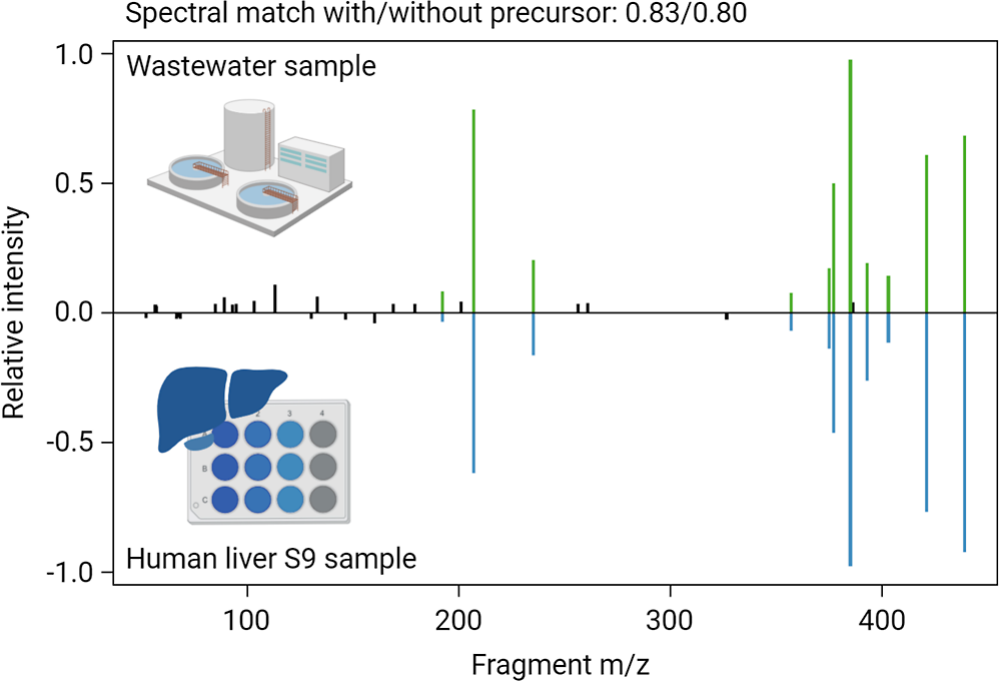
Mirror plot of losartan M2 MS2 spectra from a wastewater sample and a human liver S9 incubation sample. The dot product spectral similarity is indicated and matching fragments are colored.

In addition to the irbesartan metabolites, three losartan metabolites were detected in influent wastewater. These are losartan carboxylic acid and the hydroxylated metabolites M2 and M5. For the carboxylated metabolite, a reference standard was commercially available (Level 1), while the other two metabolites were confirmed based on human liver S9 incubation experiments (Level 2b). A mirror plot of the MS2 spectra of losartan metabolite M2 measured in wastewater and in a human liver S9 sample is displayed in Figure 6, showing a dot product spectral similarity of over 0.8. Besides metabolite M2, 15 additional metabolites could be confirmed by human liver S9 experiments, highlighting *in vitro* generation of metabolites as a useful tool for compound confirmation in cases where reference standards are not commercially available, are too expensive, or require complicated chemical synthesis.

Another confirmed (Level 1) sartan metabolite detected for the first time in influent wastewater is valeryl-4-hydroxyvalsartan. In all three treatment plants, similar concentrations of around 200 ng/L were detected. However, these concentrations are 2 orders of magnitude lower than of the parent valsartan.

### 3.5 Literature vs Monitored Metabolites/Parent Ratios

**Figure 7:**
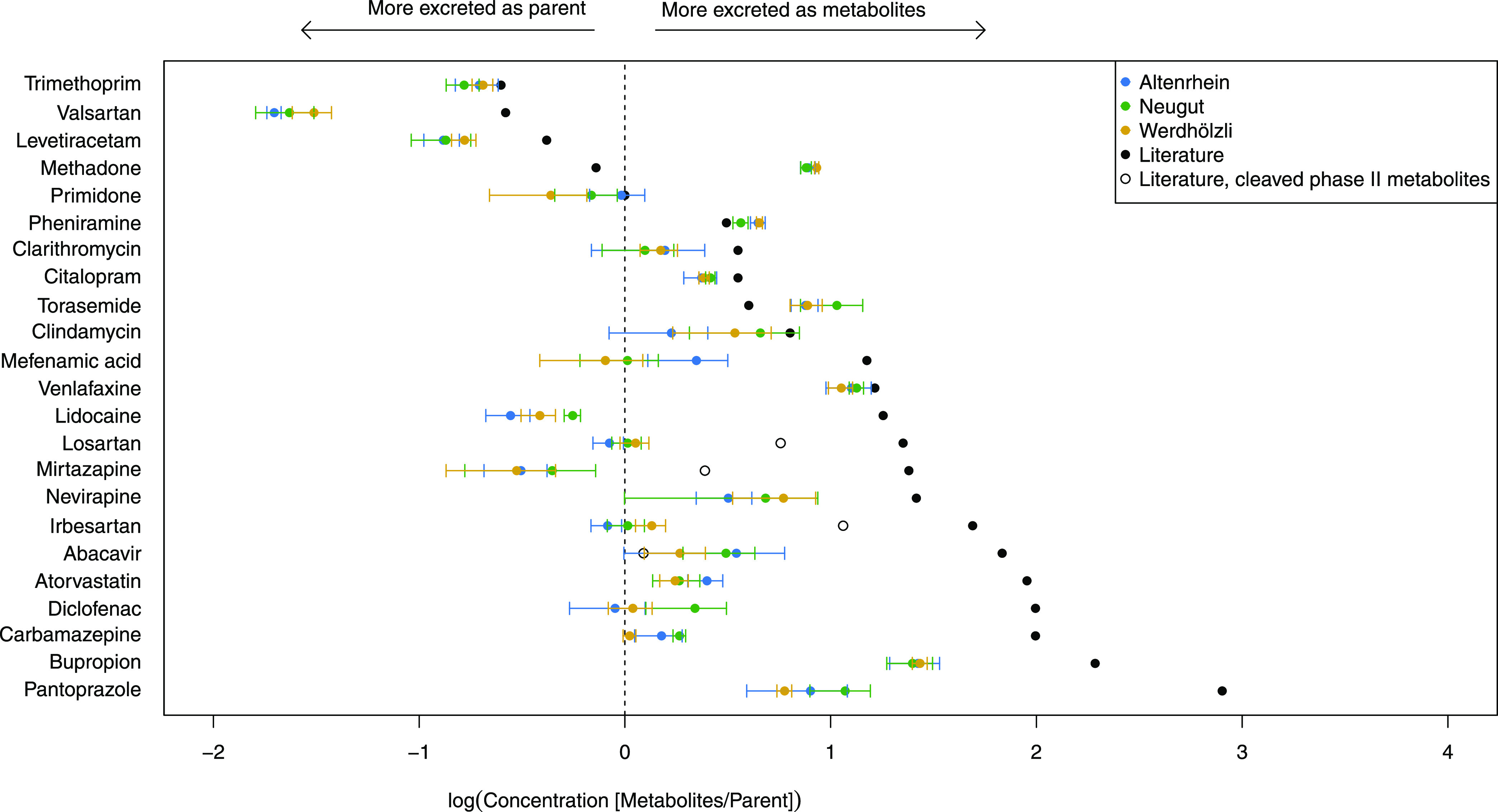
Ratio of total metabolite and parent concentrations. Black dots correspond to literature values, hollow black dots take back-cleavage of phase II metabolites to parents into account, while the colored dots refer to the measured concentrations in the three different WWTPs. Compounds for ratio calculation were selected based on the presence of the parent and the metabolites in wastewater influent.

Literature analysis in combination with target and suspect screening clearly revealed that metabolites need to be evaluated in addition to the parents, as they pose a large fraction to the total pharmaceutical contribution in wastewater. When looking at individual pharmaceuticals, many parents were detected at lower concentrations than the sum of their metabolites (compounds to the right of the dotted line in Figure 7). Still, due to the overall higher number of parent compounds than metabolites which were analyzed quantitatively and semiquantitaviely (222 vs 142), the summed concentrations of parents were by a factor 1.4-2.0 higher than the metabolites, depending on the WWTP.

From Figure 7, it is apparent that for almost all compounds, metabolite to parent concentrations are lower than what we would expect from literature. On one hand, metabolites below the LOD suffering from heavy matrix effects in influent wastewater or compounds too polar to be covered by the analytical method may be missed. On the other hand, further transformation of metabolites in the sewer system may occur, leading to a concentration underestimation of the metabolites. Methadone, pheniramine and torasemide are exceptions to this general trend that may be attributed to further transformation of the parent compounds in the sewer system.

Figure 7 covers four compounds (losartan, mirtazapine, irbesartan and abacavir) for which human phase II metabolites of the parent were reported. Assuming that all phase II metabolites are cleaved back to the parent upon arrival in the WWTP, the literature values move closer to the observed ratios. For losartan, mirtazapine and irbesartan, no phase II metabolites were detected in influent samples. But, for abacavir, a phase II metabolite was found. This detection shifts the measured concentration ratio of abacavir between the two literature values, while for the other three compounds, concentration ratios remain underestimated.

Another point to be considered is the uncertainty in the literature values. For example, genetic polymorphisms, which strongly depend on ethnicity, lead to different metabolic activities.^56^ Additionally, these literature values originate from analytical measurements with uncertainties. One example is pantoprazole, where 80% of an administered dose is excreted as metabolites in the urine. The remaining 20% are reported to be excreted via feces, but no information on whether this occurs as parent or as metabolites is available.^20,21^ For literature ratio calculation, it was assumed that 0.1% of the dose is excreted as parent. This may lead to a strong overestimation of the ratio and may explain the large difference observed between literature and measured ratios.

Metabolites are an alternative to the parent compounds for determining consumption of pharmaceuticals by wastewater-based epidemiology. This can be illustrated by the example of torasemide and its metabolite torasemide carboxylic acid, which were both detected in all three WWTPs. The average consumption of torasemide based on sales in Switzerland in the years 2014–2016 amounted to 925 kg.^57^ Using the detected concentrations and flow rates averaged over the 5 sampling days, excretion rates and the populations covered by the respective WWTP, yearly consumption amounts of 1215 ± 330 kg and 1135 ± 115 kg are calculated when torasemide or torasemide carboxylic acid are used as a basis, respectively. It becomes evident that the calculated consumptions are very similar. However, both approaches require assumptions. When estimating consumption of a compound via its metabolite, confounding sources such as improper disposal in the toilet can be excluded. However, excretion rates of specific metabolites are often unknown, impeding reliable calculations. Moreover, single metabolites are often present in lower concentrations, requiring sensitive methods like the one applied in this study to maintain adequate sensitivity. A last point to be considered is the sewer stability of parent and metabolites, since chemical transformations confound the calculations for both approaches.

This screening, based on a suspect list comprising more than 1500 pharmaceutical metabolites previously detected in urinary and fecal samples, revealed the presence of several previously overlooked metabolites in influent wastewater. This improves our knowledge of the initial stages of the environmental fate of pharmaceuticals, underscoring the need for monitoring and evaluating both parent compounds and their metabolites.

## Supporting Information Available

Literature analysis of pharmaceuticals and their excretion as parent and metabolites; peak picking tool comparison; information on wastewater sample collection and preparation, analytical method, quality control, structure elucidation, RT prediction, confidence score calculation and semiquantification; LC amenability of suspect list compounds; exclusion criteria of suspect screening; summary of identified suspect screening compounds, including annotated MS2 spectra, RT prediction, molecular network results, and confirmation by reference standards; summary of nontarget screening compounds (PDF)
Suspect list; target screening results; suspect candidates; confidence scores; reference standards; ILISs; semiquantification results; information on sampled WWTPs; used solvents and chemicals (XLSX)

## Abbreviations

ESI+/−: electrospray ionization positive/negative ionization mode
HRMS/MS: high-resolution tandem mass spectrometry
ILIS: isotope labeled internal standard
LC: liquid chromatography
PCA: principal component analysis
RT: retention time
SPE: solid phase extraction
WWTP: wastewater treatment plant

## References

[ref1] González Peña O. I.; López Zavala M. A..; Cabral Ruelas H. Pharmaceuticals market, consumption trends and disease incidence are not driving the pharmaceutical research on water and wastewater. Int. J. Environ. Res. Public Health 2021, 18, 2532 10.3390/ijerph18052532. 33806343 PMC7967517

[ref2] Gago-Ferrero P.; Bletsou A. A.; Damalas D. E.; Aalizadeh R.; Alygizakis N. A.; Singer H. P.; Hollender J.; Thomaidis N. S. Wide-scope target screening of > 2000 emerging contaminants in wastewater samples with UPLC-Q-ToF-HRMS/MS and smart evaluation of its performance through the validation of 195 selected representative analytes. J. Hazard. Mater. 2020, 387, 121712 10.1016/j.jhazmat.2019.121712. 31784138

[ref3] Jennifer L. Drug Metabolism. Clinical Pharmacology—MSD Manual Professional Ed; MSD, 2022.

[ref4] Moschet C.; Piazzoli A.; Singer H.; Hollender J. Alleviating the reference standard dilemma using a systematic exact mass suspect screening approach with liquid chromatography-high resolution mass spectrometry. Anal. Chem. 2013, 85, 10312–10320. 10.1021/ac4021598. 24161211

[ref5] Kiefer K.; Müller A.; Singer H.; Hollender J. New relevant pesticide transformation products in groundwater detected using target and suspect screening for agricultural and urban micropollutants with LC-HRMS. Water Res. 2019, 165, 114972 10.1016/j.watres.2019.114972. 31450217

[ref6] Horai H.; Arita M.; Kanaya S.; Nihei Y.; Ikeda T.; Suwa K.; Ojima Y.; Tanaka K.; Tanaka S.; Aoshima K.; et al. MassBank: A public repository for sharing mass spectral data for life sciences. J. Mass Spectrom. 2010, 45, 703–714. 10.1002/jms.1777. 20623627

[ref7] mzCloud. A mass spectral database that assists in identifying compunds in life sciences, matabolomics, pharmaceutical research, toxicology, forensic investigations, environemnta analysis, food control, and industry. https://www.mzcloud.org (accessed March 15, 2023).

[ref8] U.S. Department of Commerce. NIST20: Updates to the NIST Tandem and Electron Ionization Spectral Libraries; National Institute of Standards and Technology, 2023.

[ref9] Dührkop K.; Fleischauer M.; Ludwig M.; Aksenov A. A.; Melnik A. V.; Meusel M.; Dorrestein P. C.; Rousu J.; Böcker S. SIRIUS 4: a rapid tool for turning tandem mass spectra into metabolite structure information. Nat. Methods 2019, 16, 299–302. 10.1038/s41592-019-0344-8. 30886413

[ref10] Dührkop K.; Shen H.; Meusel M.; Rousu J.; Böcker S. Searching molecular structure databases with tandem mass spectra using CSI:FingerID. Proc. Natl. Acad. Sci. U.S.A. 2015, 112, 12580–12585. 10.1073/pnas.1509788112. 26392543 PMC4611636

[ref11] Ruttkies C.; Schymanski E. L.; Wolf S.; Hollender J.; Neumann S. MetFrag relaunched: Incorporating strategies beyond in silico fragmentation. J. Cheminf. 2016, 8, 3 10.1186/s13321-016-0115-9. PMC4732001 26834843

[ref12] Perez De Souza L.; Alseekh S.; Brotman Y.; Fernie A. R. Network-based strategies in metabolomics data analysis and interpretation: from molecular networking to biological interpretation. Expert Rev. Proteomics 2020, 17, 243–255. 10.1080/14789450.2020.1766975. 32380880

[ref13] Aron A. T.; Gentry E. C.; McPhail K. L.; Nothias L. F.; Nothias-Esposito M.; Bouslimani A.; Petras D.; Gauglitz J. M.; Sikora N.; Vargas F.; et al. Reproducible molecular networking of untargeted mass spectrometry data using GNPS. Nat. Protoc. 2020, 15, 1954–1991. 10.1038/s41596-020-0317-5. 32405051

[ref14] Wu G.; Wang X.; Zhang X.; Ren H.; Wang Y.; Yu Q.; Wei S.; Geng J. Nontarget screening based on molecular networking strategy to identify transformation products of citalopram and sertraline in wastewater. Water Res. 2023, 232, 119509 10.1016/j.watres.2022.119509. 36801596

[ref15] Schollée J. E.; Schymanski E. L.; Stravs M. A.; Gulde R.; Thomaidis N. S.; Hollender J. Similarity of High-Resolution Tandem Mass Spectrometry Spectra of Structurally Related Micropollutants and Transformation Products. J. Am. Soc. Mass Spectrom. 2017, 28, 2692–2704. 10.1007/s13361-017-1797-6. 28952028

[ref16] Huber C.; Müller E.; Schulze T.; Brack W.; Krauss M. Improving the Screening Analysis of Pesticide Metabolites in Human Biomonitoring by Combining High-Throughput In Vitro Incubation and Automated LC-HRMS Data Processing. Anal. Chem. 2021, 93, 9149–9157. 10.1021/acs.analchem.1c00972. 34161736

[ref17] Sepman H.; Malm L.; Peets P.; MacLeod M.; Martin J.; Breitholtz M.; Kruve A. Bypassing the Identification: MS2Quant for Concentration Estimations of Chemicals Detected with Nontarget LC-HRMS from MS2 Data. Anal. Chem. 2023, 95, 12329–12338. 10.1021/acs.analchem.3c01744. 37548594 PMC10448440

[ref18] Johanning K.; Hancock G.; Escher B.; Adekola A.; Bernhard M. J.; Cowan-Ellsberry C.; Domoradzki J.; Dyer S.; Eickhoff C.; Embry M.; et al. Assessment of Metabolic Stability Using the Rainbow Trout (Oncorhynchus mykiss) Liver S9 Fraction. Curr. Protoc. Toxicol. 2012, 53, 1–14. 10.1002/0471140856.tx1410s53. 22896006

[ref19] R Core Team R. A Language and Environment for Statistical Computing, 2022. https://www.r-project.org/.

[ref20] HCI Solutions AG. Swiss Compendium. 2023. https://compendium.ch/ (accessed August 29, 2023).

[ref21] Wishart D. S.; Feunang Y. D.; Guo A. C.; Lo E. J.; Marcu A.; Grant J. R.; Sajed T.; Johnson D.; Li C.; Sayeeda Z.; et al. DrugBank 5.0: A major update to the DrugBank database for 2018. Nucleic Acids Res. 2018, 46, D1074–D1082. 10.1093/nar/gkx1037. 29126136 PMC5753335

[ref22] Pluskal T.; Castillo S.; Villar-Briones A.; Orešič M. MZmine 2: Modular framework for processing, visualizing, and analyzing mass spectrometry-based molecular profile data. BMC Bioinf. 2010, 11, 395 10.1186/1471-2105-11-395. PMC2918584 20650010

[ref23] Tsugawa H.; Cajka T.; Kind T.; Ma Y.; Higgins B.; Ikeda K.; Kanazawa M.; Vandergheynst J.; Fiehn O.; Arita M. MS-DIAL: Data-independent MS/MS deconvolution for comprehensive metabolome analysis. Nat. Methods 2015, 12, 523–526. 10.1038/nmeth.3393. 25938372 PMC4449330

[ref24] Delabriere A.; Warmer P.; Brennsteiner V.; Zamboni N. SLAW: A Scalable and Self-Optimizing Processing Workflow for Untargeted LC-MS. Anal. Chem. 2021, 93, 15024–15032. 10.1021/acs.analchem.1c02687. 34735114

[ref25] Loos M. enviMass Version 3.5 LC-HRMS Trend Detection Workflow - R Package, 2019.

[ref26] Jalili V.; Afgan E.; Gu Q.; Clements D.; Blankenberg D.; Goecks J.; Taylor J.; Nekrutenko A. The Galaxy platform for accessible, reproducible and collaborative biomedical analyses: 2020 update. Nucleic Acids Res. 2020, 48, W395–W402. 10.1093/nar/gkaa434. 32479607 PMC7319590

[ref27] Schymanski E. L.; Kondić T.; Neumann S.; Thiessen P. A.; Zhang J.; Bolton E. E. Empowering large chemical knowledge bases for exposomics: PubChemLite meets MetFrag. J. Cheminf. 2021, 13, 19 10.1186/s13321-021-00489-0. PMC7938590 33685519

[ref28] Yu G.; Chen Y.-s.; Guo Y.-c. Design of integrated system for heterogeneous network query terminal. J. Comput. Appl. 2009, 29, 2191–2193. 10.3724/SP.J.1087.2009.02191.

[ref29] Csárdi G.; Nepusz T.; Traag V.; Horvát S.; Zanini F.; Noom D.; Müller K. Igraph: Network Analysis and Visualization in R. 2023. https://cran.r-project.org/package=igraph. 10.1371/journal.pone.0355108 PMC13436765 42550872

[ref30] JChem for Office. https://chemaxon.com/jchem-for-office.

[ref31] Schymanski E. L.; Jeon J.; Gulde R.; Fenner K.; Ruff M.; Singer H. P.; Hollender J. Identifying small molecules via high resolution mass spectrometry: Communicating confidence. Environ. Sci. Technol. 2014, 48, 2097–2098. 10.1021/es5002105. 24476540

[ref32] Alygizakis N.; Lestremau F.; Gago-Ferrero P.; Gil-Solsona R.; Arturi K.; Hollender J.; Schymanski E. L.; Dulio V.; Slobodnik J.; Thomaidis N. S. Towards a harmonized identification scoring system in LC-HRMS/MS based non-target screening (NTS) of emerging contaminants. TrAC, Trends Anal. Chem. 2023, 159, 116944 10.1016/j.trac.2023.116944.

[ref33] Aalizadeh R.; Alygizakis N. A.; Schymanski E. L.; Krauss M.; Schulze T.; Ibáñez M.; McEachran A. D.; Chao A.; Williams A. J.; Gago-Ferrero P.; et al. Development and Application of Liquid Chromatographic Retention Time Indices in HRMS-Based Suspect and Nontarget Screening. Anal. Chem. 2021, 93, 11601–11611. 10.1021/acs.analchem.1c02348. 34382770

[ref34] Gatto L.; Lilley K. S. Msnbase-an R/Bioconductor package for isobaric tagged mass spectrometry data visualization, processing and quantitation. Bioinformatics 2012, 28, 288–289. 10.1093/bioinformatics/btr645. 22113085

[ref35] Schollée J. E. MSMSsim: functions for processing HRMS2 spectra from output from RMassBank, mainly for calculating spectral similarity. 2017. https://github.com/dutchjes/MSMSsim (accessed July 31, 2023).

[ref36] Yap C. W. PaDEL-descriptor: An open source software to calculate molecular descriptors and fingerprints. J. Comput. Chem. 2011, 32, 1466–1474. 10.1002/jcc.21707. 21425294

[ref37] Kruve A.; Kiefer K.; Hollender J. Benchmarking of the quantification approaches for the non-targeted screening of micropollutants and their transformation products in groundwater. Anal. Bioanal. Chem. 2021, 413, 1549–1559. 10.1007/s00216-020-03109-2. 33506334 PMC7921029

[ref38] Kaliszan R. Q. S. R. R. QSRR: Quantitative Structure-(Chromatographic) Retention Relationships. Chem. Rev. 2007, 107, 3212–3246. 10.1021/cr068412z. 17595149

[ref39] Souihi A.; Mohai M. P.; Palm E.; Malm L.; Kruve A. M.C.R. T. MultiConditionRT: Predicting liquid chromatography retention time for emerging contaminants for a wide range of eluent compositions and stationary phases. J. Chromatogr. A 2022, 1666, 462867 10.1016/j.chroma.2022.462867. 35139450

[ref40] Godejohann M.; Berset J. D.; Muff D. Non-targeted analysis of wastewater treatment plant effluents by high performance liquid chromatography-time slice-solid phase extraction-nuclear magnetic resonance/time-of-flight-mass spectrometry. J. Chromatogr. A 2011, 1218, 9202–9209. 10.1016/j.chroma.2011.10.051. 22098937

[ref41] Jewell K. S.; Wick A.; Ternes T. A. Comparisons between abiotic nitration and biotransformation reactions of phenolic micropollutants in activated sludge. Water Res. 2014, 48, 478–489. 10.1016/j.watres.2013.10.010. 24238259

[ref42] Yan Z.; Caldwell G. W.; Jones W. J.; Masucci J. A. Cone voltage induced in-source dissociation of glucuronides in electrospray and implications in biological analyses. Rapid Commun. Mass Spectrom. 2003, 17, 1433–1442. 10.1002/rcm.1071. 12820208

[ref43] Yuan L.; Sophia Xu X.; Ji Q. C. Challenges and recommendations in developing LC–MS/MS bioanalytical assays of labile glucuronides and parent compounds in the presence of glucuronide metabolites. Bioanalysis 2020, 12, 615–624. 10.4155/bio-2020-0055. 32441529

[ref44] Huber C.; Krauss M.; Reinstadler V.; Denicolò S.; Mayer G.; Schulze T.; Brack W.; Oberacher H. In silico deconjugation of glucuronide conjugates enhances tandem mass spectra library annotation of human samples. Anal. Bioanal. Chem. 2022, 414, 2629–2640. 10.1007/s00216-022-03899-7. 35080654 PMC8888480

[ref45] Mohammadi M.; Ramezani-Jolfaie N.; Lorzadeh E.; Khoshbakht Y.; Salehi-Abargouei A. Hesperidin, a major flavonoid in orange juice, might not affect lipid profile and blood pressure: A systematic review and meta-analysis of randomized controlled clinical trials. Phytother. Res. 2019, 33, 534–545. 10.1002/ptr.6264. 30632207

[ref46] Benzophenone. 2012. https://www.ncbi.nlm.nih.gov/books/NBK373188/ (accessed January 26, 2024).

[ref47] Tretter L.; Patocs A.; Chinopoulos C. Succinate, an intermediate in metabolism, signal transduction, ROS, hypoxia, and tumorigenesis. Biochim. Biophys. Acta, Bioenerg. 2016, 1857, 1086–1101. 10.1016/j.bbabio.2016.03.012. 26971832

[ref48] Choi Y.; Kim K.; Kim D.; Moon H. b.; Jeon J. Ny-Ålesund-oriented organic pollutants in sewage effluent and receiving seawater in the Arctic region of Kongsfjorden. Environ. Pollut. 2020, 258, 113792 10.1016/j.envpol.2019.113792. 31877466

[ref49] Nürenberg G.; Kunkel U.; Wick A.; Falås P.; Joss A.; Ternes T. A. Nontarget analysis: A new tool for the evaluation of wastewater processes. Water Res. 2019, 163, 114842 10.1016/j.watres.2019.07.009. 31323503

[ref50] Cui H.; Chang H.; Zheng H.; Wan Y. Determination and occurrence of sulfonamide transformation products in surface waters. Sci. Total Environ. 2021, 779, 146562 10.1016/j.scitotenv.2021.146562. 34030252

[ref51] Scheurer M.; Nödler K.; Schmid R.; Schaffer M. Vorkommen ausgewählter persistenter und mobiler organischer Spurenstoffe in Oberflächengewässern Niedersachsens. Vom Wasser 2023, 121, 10–18. 10.1002/vomw.202300001.

[ref52] Fabregat-Safont D.; Botero-Coy A. M.; Nieto-Juárez J. I.; Torres-Palma R. A.; Hernández F. Searching for pharmaceutically active products and metabolites in environmental waters of Peru by HRMS-based screening: Proposal for future monitoring and environmental risk assessment. Chemosphere 2023, 337, 139375 10.1016/j.chemosphere.2023.139375. 37391080

[ref53] Li R.; Liang C.; Svendsen S. B.; Kisielius V.; Bester K. Sartan blood pressure regulators in classical and biofilm wastewater treatment – Concentrations and metabolism. Water Res. 2023, 229, 119352 10.1016/j.watres.2022.119352. 36450176

[ref54] Opie L. H.; Pfeffer M. A. Drugs for the Heart, 7th ed.; Opie L. H., Bernard J. G., Braunwald E., Walker J., Eds.; Saunders, 2008.

[ref55] Chando T. J.; Everett D. W.; Kahle A. D.; Starrett A. M.; Vachharajani N.; Shyu W. C.; Kripalani K. J.; Barbhaiya R. H. Biotransformation of irbesartan in man. Drug Metab. Dispos. 1998, 26, 408–417. 9571222

[ref56] Zanger U. M.; Schwab M. Cytochrome P450 enzymes in drug metabolism: Regulation of gene expression, enzyme activities, and impact of genetic variation. Pharmacol. Ther. 2013, 138, 103–141. 10.1016/j.pharmthera.2012.12.007. 23333322

[ref57] Wetzel S. Analyse der Menge an pharmazeutischen Wirkstoffen, die in der Schweiz verkauft wurden (Jahre 2014–2016); IMS Health, 2017.

