# Supplementary material for: How Wastewater Reflects Human Metabolism—Suspect Screening of Pharmaceutical Metabolites in Wastewater Influent
Source: Environ Sci Technol. 2024 May 24;58(22):9828–39. doi: 10.1021/acs.est.4c00968 (PMC11154963; doi:10.1021/acs.est.4c00968)
Supplement: Supplementary file 1 — es4c00968_si_001.pdf [file es4c00968_si_001.pdf]

Supporting Information B, C and D (SI-B/C/D) to:

How Wastewater Reflects Human Metabolism - Suspect Screening of Pharmaceutical Metabolites in Untreated Wastewater

Corina Meyer[†,‡], Michael A. Stravs[†], Juliane Hollender[*,†,‡]

[†] Eawag: Swiss Federal Institute of Aquatic Science and Technology, Ueberlandstrasse 133, 8600, Duebendorf, Switzerland
[‡] Institute of Biogeochemistry and Pollutant Dynamics, Universitaetstrasse 16, ETH Zurich, 8092, Zurich, Switzerland

[*] Corresponding author: juliane.hollender@eawag.ch

Environmental Science & Technology, 2024

Supporting Information B: S2 - S19
Supporting Information C: S20 - S78
Supporting Information D: S79 - S577

Supporting Information B

Supporting Pages: S2 - S19
Supporting Tables: SI-B1

Contents

SI-B1 Literature Analysis **S4**

List of Tables

SI-B1 Excretion of pharmaceuticals and their metabolites from the human body. . . . S5

SI-B1 Literature Analysis

The aim of this literature analysis was to evaluate to which extent pharmaceuticals are excreted as parent compound or as metabolites from the human body into wastewater. A differentiation between phase I metabolites, direct phase II metabolites, and combined phase I and phase II metabolism was made, because the conjugated part of a phase II metabolite is easily cleaved in wastewater, resulting in either the parent or the previous phase I metabolite. This is especially true for glucuronides.[1] Table SI-B1 summarizes the results of the 268 pharmaceuticals previously prioritized based on their consumption amounts in Switzerland from 2014 - 2016 in alphabetical order. The results need to be considered with reservation, as they display averaged data. Differences in metabolism and excretion are observed depending on dose, mode of administration, drug-drug interactions or genetic variability, leading to varying metabolization rates. Moreover, data availability for some compounds was limited or contradictory, such that some values had to be estimated (indicated by footnote [e]). Some compounds are not meant to be resorbed in the human body. Therefore, the ADME parameters absorption, distribution, metabolism and excretion cannot be applied. These cases are indicated in the table. A further complication are pro-drugs. They are designed to be cleaved in the human body. In case this occurs to nearly 100%, the excretion percentages are given for the active pharmaceutical compound and not the prodrug (indicated by footnote [f]). For those that are not completely cleaved, the data refers to the prodrug and no special label is given. If available, information were taken from the DrugBank, a free-to-access, online database containing information on drugs and drug targets of the University of Alberta,[2] or Swiss Compendium, a reference to pharmaceutical products on the Swiss market.[3] Missing data were compiled from scientific publications. If available, the excretion pathway (urinary, fecal, both) is specified. For some compounds it was not possible to differentiate between phase I metabolites and phase I metabolites that have undergone an additional conjugation. If this is the case, a total value in the sixth column is provided.

Table SI-B1: Excretion of pharmaceuticals and their metabolites from the human body.

API	Excreted as parent [%]	Excreted as phase I metabolite [%]	Excreted as direct phase II metabolite (back cleavable to parent) [%]	Excreted as phase II metabolite (not back cleavable to parent) [%]	Phase I and conjugated Phase I metabolites [&]	Comment	Source
Abacavir	1.2[a]	45.8[a]	36[a]	0	NA	16% excreted in feces[d]	[4]
Abiraterone acetate[f]	77[b]	0	0	4[a]	NA		[3,5]
Acamprosate	90[a]	0.1[a]	0	0	NA		[2,3]
Acemetacin	5[c,e]	NA	5[c,e]	NA	90[c,e]		[2,3]
Acetazolamide	100[a]	0	0	0	NA		[6]
Acetylcysteine	33[c]	0	0	0	NA	Remaining dose is metabolized and incorporated into cellular pools	[2,3,7]
Acetylsalicylic acid	0	85[a]	0	10[a]	NA		[3]
Acyclovir	80[a]	16[a]	0	0	NA		[2,3]
Alginic acid	not resorbed, parameters do not apply						[2]
Aliskiren	80[b]	1.4	0	0	NA		[2,3]
Allopurinol	<10[a]	70[a]	0	0	NA	20% excreted in feces[d]	[3]
Amiodarone	<1[a]	90[a,e]	0	0	NA		[3,8]
Amisulpride	78[c]	19[c]	0	0	NA		[2]
Amitriptyline	2[a]	NA	0	NA	98[a,e]		[2]
Amlodipine	10[a]	90[c]	0	0	NA		[2]
Amoxicillin	70[a]	20[a]	0	0	NA		[3]
Apixaban	50[c]	7[c]	0	25[c]	NA		[2]
Apremilast	10[c]	NA	0	NA	90		[2,3]
Atazanavir	27[c]	NA	0	NA	65[c]		[3]
Atenolol	90[c]	8[c]	2[c]	0	NA		[2]
Atorvastatin	<1[a]	NA	0	NA	90[b,e]		[9]
Atovaquone	>90[b]	0	0	0	NA		[3]
Azathiprine	10[a]	NA	0	NA	40[a]	12.6% excreted in feces[d]	[3]

Azilsartan medoximil[f]	16[a]	74[c]	0	0	NA		[10]
Azithromycin	70[c,e]	30[c,e]	0	0	NA		[2,3]
Benserazide	0	88[c]	0	0	NA		[2,3]
Betahistine	0	85[a]	0	0	NA		[3]
Bezafibrate	50[a]	25[a]	20[a]	0	NA	3% excreted in feces[d]	[3]
Bilastine	94.8[c]	0	0	0	NA		[2]
Bisacodyl	0	67.2[c]	0	10.5[a]	NA		[2,3]
Bisoprolol	50[a]	50[a]	0	0	NA		[2,3]
Bupropion	0.5[a]	96.5[c]	0	0	NA		[2,3]
Caffeine	1[a]	99[a]	0	0	NA		[2,3]
Calcium dobesilate	90[c]	0	10[a]	0	NA		[3]
Canagliflozin	42.5[b]	7[b]	33.7[c]	0	NA		[2,3]
Candesartan cilexetil[f]	82[c]	17[c]	1[e]	0	NA		[3]
Capecitabine	2.9[a]	92.6[a]	0	0	NA	2.6% excreted in feces[d]	[3]
Carbamaezepine	<1[a]	NA	0	NA	99[c]		[2,3]
Carbasalate Calcium	0	90[a]	0	10[a]	NA		[3]
Carbidopa	17.5[a]	NA	0	NA	32.5[a]	50% excreted in feces[d]	[3,11]
Carbocisteine	60[a]	20[a,e]	10[a,e]	0	NA	0.3% excreted in feces[d]	[2,3,12]
Carvedilol	<2[a]	NA	0	NA	74[c]		[2,3]
Cefaclor	85[a]	0	0	0	NA		[2,3]
Cefazolin	100[a]	0	0	0	NA		[2,3]
Cefepime	85[a]	10.3[a]	0	0	NA		[2,3]
Cefpodoxime proxetil	80[a]	0	0	0	NA		[3]
Ceftriaxone	100[c]	0	0	0	NA		[3]
Cefuroxime	100[a]	0	0	0	NA		[3]
Cefuroxime axetil[f]	100[a]	0	0	0	NA		[3]
Celecoxib	<3[c]	NA	0	NA	81[c]		[2]
Cetirizine	70[c]	10[c]	0	0	NA		[2,3,13]
Chloral hydrate	0	NA	0	NA	100[c]		[2]
Chlorprothixene	40[c,e]	60[c,e]	0	0	NA		[2,3]

Chlorthalidone	50[a]	50[a,e]	0	0	NA	Details of metabolism not elucidated	3,14
Cinnarizine	<1[a]	99[c]	0	0	NA		3
Ciprofloxacin	69.7[c]	9.2[c]	9.6[c]	0	NA		3
Citalopram	22[c]	78[c,e]	0	0	NA		2,3
Clarithromycin	22[c]	78[c]	0	0	NA		15
Clavlanic acid	58.6[c]	24.4[c]	0	0	NA	17% exhaled	2,3
Clindamycin	13.6[c]	86.4[b]	0	0	NA		2,3
Clomethiazole	<1[a]	99[a]	0	0	NA		3,16
Clomipramine	2[a]	NA	0	NA	98[c]		2,3
Clopidogrel	0	96[c]	0	0	NA		2,3
Clozapine	<1[c,e]	NA	0	NA	80[c]		2,17
Cobicistat	35.2[c]	59[b]	0	0	NA		3
Codeine	10[a]	NA	50	NA	35		2
Colestipol	not resorbed, parameters do not apply						2
Colestyramine	not resorbed, parameters do not apply						2
Cyclosporin	<1	94[b]	0	0	NA		2
Dabigatran etexilate[f]	77[a]	0	4[a]	0	NA		3,18
Darunavir	48.9[c]	NA	0	NA	44.5[c]		3
Dexibuprofen	0.5[a]	NA	0.5[a]	NA	99[a]	1% excreted as ibuprofen and ibuprofen glucuronide, value arbitrarily divided by 2 to account for the unchanged parent compound and direct phase II metabolite	2
Dexlansoprazole	0	NA	0	NA	100[a,e]		19,20
Dextromethorphan	1[a]	NA	0	NA	99[a]		21,22
Diclofenac	1[a]	NA	0	NA	99[c]		3
Dihydrocodeine	23.9[a]	11.9[a]	26.5[a]	0	5[a]		23
Diltiazem	63[c]	NA	0	NA	35[a]		3
Dimethyl fumarate	0	17[c]	0	0	NA	80% exhaled	2

Diosmin	0	NA	0	NA	94[c]		[3]
Diphenhydramine	1[a]	NA	0	NA	60[a]		[2,3]
Dipyridamole	0	0	100[b]	0	NA		[2]
Disulfiram	15[b]	80	0	0	NA		[24]
Dolutegravir	54[b]	6.6[a]	18.9[a]	NA	5.5		[2,3]
Domperidone	11[c]	86[c]	0	0	NA		[3]
Doxycycline	100[c]	0	0	0	NA		[3]
Dronedarone	5[a,e]	NA	0	NA	85[c,e]		[2,3]
Duloxetine	2[b]	20[c]	0	68[a]	NA		[2,25]
Edoxaban	73[c]	2[c]	0	0	NA		[26]
Efavirenz	<1[a]	NA	0	NA	99[c,e]		[2,3,27,28]
Elvitegravir	31[b]	NA	0	NA	69[c]		[3]
Empagliflozin	57.9[c]	0	37.8[c]	0	NA		[3]
Emtricitabine	73[a]	9[a]	4[a]	0	NA	14% excreted in feces[d]	[2,3]
Enalapril	34[c]	60[c]	0	0	NA		[2,3]
Enoxaparin sodium	60[a]	40[a]	0	0	NA		[2,3]
Entacapone	85[b]	1[a]	14[a]	0	NA		[2,3]
Enzalutamide	0.5[c]	84.5[c]	0	0	NA		[2,3]
Eprosartan	98[c]	0	<2[a]	0	NA		[2,3]
Erdosteine	0	100	0	0	NA		[3]
Escitalopram	22[c]	78[c,e]	0	0	NA	Values from citalopram	[2,3]
Esomeprazole	1[a]	99[c]	0	0	NA		[2]
Ethosuximide	20[a]	65[a]	0	0	NA		[29]
Etodolac	1[a]	NA	13[a]	NA	58[a]	16% excreted in feces[d]	[2]
Etoricoxib	<2[c]	NA	0	NA	88[c]		[3]
Ezetimibe	69[b]	NA	9[a]	NA	11[c]		[2]
Febuxostat	15[c]	NA	31[a]	NA	48[c]		[2]
Fenofibrate	0	NA	0	NA	100[c]		[2]
Fexofenadine	91[c]	5.1	0	0	NA		[2]
Flavoxate	0	NA	0	NA	60[a]		[3]
Flecainide	42[a]	NA	0	NA	44[a]	5% excreted in feces[d]	[2]

Flucloxacillin	70[a]	10[a]	0	0	NA	Small amounts excreted in feces[d]	[3,30,31]
Fluconazole	80[a]	4.5[a]	6.5[a]	0	NA		[2,3]
Fluorouracil	<15[a]	0	0	0	NA	75% exhaled	[3]
Fluoxetine	11[c]	27[c]	7[a]	8[a]	NA		[32]
Fluvastatin	<2[b]	NA	0	NA	97[b]		[2,3]
Fluvoxamine	2[a]	92[a]	0	0	NA		[2]
Fosfomycin Trometamol	100[c]	0	0	0	NA		[2,3]
Furosemide	43[a]	20[c,e]	14[a]	0	NA		[3,33,34]
Gabapentin	99[a]	<1[a]	0	0	NA		[3]
Gemfibrozil	<2[a]	38[a]	30[a]	0	NA	6% excreted in feces[d]	[2,3]
Gliclazide	1[a]	NA	0	NA	80[c]		[2,3]
Guaifenesin	0	95[a]	0	0	NA		[2,3]
Hesperidin	10[a,e]	NA	0	NA	90[a,e]		[35]
Hydrochlorothiazide	100[a]	0	0	0	NA		[3]
Hydroxycarbamide	37[a]	60	0	0	NA		[2]
Hydroxychloroquine	25[a]	45[c]	0	0	NA	30% excreted via skin	[2,3]
Ibrutinib	1[b]	89[c]	0	0	NA		[3]
Ibuprofen	0.5[a]	NA	0.5[a]	NA	99[a]	1% excreted as ibuprofen and ibuprofen glucuronide, value arbitrarily divided by 2 to account for the unchanged parent compound and direct phase II metabolite	[2]
Iopamidol	99.9	< 0.1	0	0	NA		[3]
Iopromide	97[a]	0	0	0	NA		[3]
Irbesartan	<2[a]	92[c]	6[a]	0	NA		[2,3]
Isosorbide dinitrate	25[a]	70[a]	0	0	NA		[3]
Isotretinoin	64[b]	NA	0	NA	30[e]		[36]
Labetalol	<4[a]	NA	40[a,e]	NA	15[a,e]		[2,3,37]
Lacosamide	40[a]	52[a]	0	0	NA		[3]

Lactitol	not resorbed, parameters do not apply						2,3
Lamivudine	90[a]	<10[a]	0	0	NA		2,3
Lamotrigine	10[a]	4.14[a]	86[a]	0	NA	2% excreted in feces[d]	3
Lansoprazole	0	NA	0	NA	100[a,e]		19,20
Lercanidipine	0	100[c]	0	0	NA		3
Levetiracetam	66[a]	27.5[a]	0	0	NA		3
Levocarnitine	0	85[a]	0	0	NA	15% enters the carnitine body pool	3
Levofloxacin	87[a]	5[a]	0	0	NA	4% excreted in feces[d]	2,3
Lidocaine	5[a]	90[a]	0	0	NA		2
Lisdexamfetamine	2[a]	NA	0	NA	94[a]	0.3% excreted in feces[d]	3
Lisinopril	100[a]	0	0	0	NA		3
Lopinavir	22[c]	71[c]	0	0	NA		2,3
Losartan	4[a]	80[c,e]	10[a,e]	0	NA		2,3
Lymecycline	65[a]	0	0	0	NA	Not metabolized and most likely not fully resorbed due to formation of insoluble complexes with Ca, Mg, Fe and Al	2,38
Mebeverine	0	100[a]	0	0	NA		3
Mefenamic acid	<5[a]	NA	0	NA	75[c]		2,3,39
Melitracen	30[c,e]	70[c,e]	0	0	NA		2,3
Meropenem	70[a]	28[a]	0	0	NA	2% excreted in feces[d]	3
Mesalazine	80[c]	0	13[a]	0	NA	21-22% resorbed, rest excreted via feces	2,3
Metamizole sodium[f]	<5[a]	NA	0	NA	85[a]	10% excreted in feces[d]	2,3,40
Metformin	100[a]	0	0	0	NA		3
Methadone	58[c]	42[c]	0	0	NA		41,42
Methylephedrine	33[a]	67[a]	0	0	NA		2
Methylphenidate	< 1[a]	99[a]	0	0	NA		2
Metoclopramide	20[a]	NA	65[a]	NA	5[e]		2,43,44

Metoprolol	<5[a]	90[a]	0	0	NA		[2,3]
Metronidazole	20[a]	NA	0	NA	60[a]	10% excreted in feces[d]	[2,3]
Minocycline	37.5[c]	62.5[c]	0	0	NA		[3]
Mirabegron	59[c]	13.2[a]	6.8[a]	0	NA		[45]
Mirtazapine	4[a]	NA	25[a]	NA	71[c]		[46]
Moclobemide	1[a]	99[c]	0	0	NA		[3]
Morclofone	5[a,e]	NA	0	NA	95[a,e]		[3]
Morphine	10.9	NA	67.7	NA	20.8		[47]
Moxifloxacin	44[c]	0	52.5[c]	0	NA		[3]
Mycophenolate mofetil[f]	<1[a]	5[a]	87[a]	0	NA	6% excreted in feces[d]	[2]
Mycophenolic acid	3[a]	10[a,e]	>70[a,e]	0	NA		[2,3]
Naftidrofuryl	20[a]	80[a]	0	0	NA		[48,49]
Naloxone	0	10[a,e]	60[a,e]	0	NA		[2,3]
Naproxen	<1[a]	0	0	94[a]	NA	<5% excreted in feces[d]	[2]
Nebivolol	<1[c]	NA	30[a,e]	NA	50[c,e]		[2,3,50]
Nevirapine	<3[a]	NA	0	NA	78.3[a]	10.1% excreted in feces[d]	[3]
Nifedipine	<0.1[a]	99.9[c]	0	0	NA		[3]
Nikethamide	5[e]	95[e]	0	0	NA	Data from horses, human data not available	[51]
Nimesulide	2	0	0	77[c]	NA		[3]
Nintedanib	20.05[b]	NA	0	NA	74[b]		[2,3]
Nitrofurantoin	40[a]	1.2[a]	45[a]	0	NA		[2,3,52]
Norfloxacin	>70[c]	25[e]	0	0	NA		[3,53]
Olmesartan medoxomil[f]	100[c]	0	0	0	NA		[2,3]
Omeprazole	0	100[c]	0	0	NA		[2,3]
Orlistat	83[b]	14[b]	0	0	NA		[3]
Oxazepam	23[c]	0	75[a]	0	NA		[2,3,54]
Oxcarbazepine	<1[a]	30[a]	13[a]	49[a]	NA	4% excreted in feces[d]	[2]
Oxycodone	8[a]	18[a]	0	0	47[a]		[55]
Pantoprazole	0	NA	0	NA	80[a]	20% excreted in feces[d]	[2,3]
Paracetamol	<5[a]	0	90[a]	4[a]	NA		[3]

Paroxetine	<3[c]	NA	0	NA	97[c]		[2,3]
Penicillin G	70[a]	20[a]	0	0	NA		[3]
Penicillin V	26[a]	56[c]	0	0	NA		[56]
Pentoxifylline	<1[a]	92.5[a]	0	0	NA	5% excreted in feces[d]	[2,3]
Perindopril	8[a]	NA	5[a,e]	NA	85[a,e]		[3,57]
Pheniramine	24.3[a]	75.7[a,e]	0	0	NA		[2]
Phenobarbital	25[a]	NA	0	NA	75[a]		[3]
Phenolphthaleine	0	0	99.5[b]	0	NA	Data from rats, human data not available	[58]
Phenylephrine	16[a]	62[a]	0	8[a]	NA		[2]
Phenytoin	3[a]	NA	0	NA	95[b]		[2,3]
Pipamperone	20[a,e]	80[a,e]	0	0	NA		[3]
Piperacillin	90[c]	0	0	0	NA		[3]
Piracetam	95[a]	0	0	0	NA		[3]
Pirfenidone	<1[a]	99[c]	0	0	NA		[2,3]
Pravastatin	60[c,e]	40[c]	0	0	NA		[2,59]
Prednisolone	5[a,e]	NA	0	NA	95[a,e]		[2,60]
Prednisone	5[a,e]	NA	0	NA	95[a,e]		[2,60]
Pregabalin	98[a]	0.9[a]	0	0	NA		[3]
Primidone	40[a]	40[a]	0	0	NA		[2,3]
Proguanil	40[a]	60[a,e]	0	0	NA		[61]
Propafenone	<1[a]	NA	0	NA	90[c]		[3,62]
Propanolol	0	83[a]	17[a]	0	NA		[63]
Propofol	<0.3[a]	29[c]	70[c]	0	NA		[64]
Pseudoephedrine	85[a]	1	0	0	NA		[2,3]
Quetiapine	<5[c]	89[c]	0	0	NA		[3]
Quinine	20[a]	80[a]	0	0	NA		[2]
Raltegravir	59.8[c]	0	22.9[a]	0	NA		[65,66]
Ranitidine	90[c,e]	<10[a]	0	0	NA		[67]
Ranolazine	5[c]	NA	0	NA	93[c]		[2,3]
Rifampicin	15[c]	85[c]	0	0	NA		[68]

Rifaximin	96.65[b]	0.29[a]	0	0	NA		[2]
Ritonavir	37.3[c]	60.4[c]	0	0	NA		[2]
Rivaroxaban	43[c]	51[c]	0	0	NA		[2]
Rosuvastatin	90[b]	10[a]	0	0	NA		[3]
Saccharin	85[a]	0	0	0	NA		[69]
Sacubitril[f]	90[c]	<10[a]	0	0	NA		[2]
Senna	4[b]	95[b]	0	0	NA		[2]
Sertraline	13.2[b]	NA	0	NA	86.8[c]		[2,3]
Sevelamer	not resorbed, parameters do not apply						[2,3]
Sildenafil	<4[a]	89[c]	0	0	NA		[3]
Silymarin	5[a]	0	80[c]	0	NA		[70]
Simeticone	not resorbed, parameters do not apply						[2]
Simvastatin	10[c]	61[c]	0	0	NA		[71]
Sitagliptin	79[a]	14	<3	<1	NA		[2,3]
Sodium amidotrizoate	100[a]	0	0	0	NA		[2]
Sodium oxybate	5[a]	0	0	0	NA	95% exhaled	[2,3]
Sorbitol	barely resorbed, parameters do not apply						[2,3]
Sotalol	100[a]	0	0	0	NA		[3]
Spironolactone	0	63.4[c]	0	0	NA		[2,3]
Sucralfate	barely resorbed, parameters do not apply						[2,3]
Sulfamethoxazole	30[a]	0	54.5[a]	0	NA		[2]
Sulfasalazine	15[c]	NA	0	NA	80[c]		[2,3,72]
Tapentadol	3[a]	15[a]	70[a]	11[a]	NA		[2,3]
Tazobactam	80[a]	20[a]	0	0	NA		[2,3]
Telmisartan	97[b]	0	<1[a]	0	NA		[2,73]
Tenofovir alafenamide	75[a]	0	0	0	NA		[2,3]
Tenofovir disoproxil[f]	75[a]	0	0	0	NA		[2,3]
Terbinafine	0	93[c]	0	0	NA		[3]
Theophylline	10[a]	90[a,e]	0	0	NA		[2,3]
Ticagrelor	<1[c]	NA	0	NA	83.3[c]		[2,3]
Tolperisone	0.1[a]	97.9[a]	0	0	NA		[3]

Topiramate	75[a]	20[a]	0	0	NA		2,3
Torasemide	20[a]	80[c]	0	0	NA		2
Tramadol	30[a]	60[a]	0	0	NA	10% excreted in feces[d]	2
Tranexamic acid	95[a]	1.5	0	0	NA		2,3
Trazodone	<1[a]	NA	0	NA	74[a]	21% excreted in feces[d]	2
Trimethoprim	80[a]	20[a]	0	0	NA		2,3
Trimipramine	10[a]	90[a,e]	0	0	NA		3
Trospium	88.8[c]	2.2[a]	0	0	NA		2
Ursodeoxycholic acid	5[b]	NA	0	NA	83[b]		3
Valaciclovir	<1[a]	91[c]	0	0	NA		2,3
Valproic acid	<3[a]	45[a]	45[a]	0	NA		2,74
Valsartan	76[c]	20[c]	0	0	NA		2
Vancomycin	80[c]	0	0	0	NA		2,3,75
Venlafaxine	5[a]	56[a]	0	26[a]	NA		2
Verapamil	3.5[a]	70[a]	0	0	NA	≥16% excreted in feces[d]	2,3
Vigabatrin	80[a]	15[a]	0	0	NA		2
Vildagliptin	23[a]	61[c]	8[a]	0	NA		2,3,76
Zidovudine	14[a]	0	74[a]	0	5[a,e]		3
Zolpidem	0	93[c]	0	0	NA		3

a: Only/mostly renal excretion
b: Only/mostly fecal excretion
c: Renal and fecal excretion
d: Not further specified if as parent or as metabolites
e: Estimated value
f: Refers to the active pharmaceutical, not the prodrug

References

[1] Pérez, S.; Barceló, D. Application of advanced MS techniques to analysis and identification of human and microbial metabolites of pharmaceuticals in the aquatic environment. *TrAC - Trends in Analytical Chemistry* **2007**, *26*, 494–514.

[2] Wishart, D. S. et al. DrugBank 5.0: A major update to the DrugBank database for 2018. *Nucleic Acids Research* **2018**, *46*, D1074–D1082.

[3] HCI Solutions AG, Swiss Compendium. 2023; `https://compendium.ch/`.

[4] McDowell, J. A.; Chittick, G. E.; Ravitch, J. R.; Polk, R. E.; Kerkering, T. M.; Stein, D. S. Pharmacokinetics of [14C]abacavir, a human immunodeficiency virus type 1 (HIV-1) reverse transcriptase inhibitor, administered in a single oral dose to HIV-1-infected adults: A mass balance study. *Antimicrobial Agents and Chemotherapy* **1999**, *43*, 2855–2861.

[5] Benoist, G. E.; Hendriks, R. J.; Mulders, P. F.; Gerritsen, W. R.; Somford, D. M.; Schalken, J. A.; van Oort, I. M.; Burger, D. M.; van Erp, N. P. Pharmacokinetic Aspects of the Two Novel Oral Drugs Used for Metastatic Castration-Resistant Prostate Cancer: Abiraterone Acetate and Enzalutamide. *Clinical Pharmacokinetics* **2016**, *55*, 1369–1380.

[6] Alex, Y. Deranged Physiology - Acetazolamide. 2021; `https://derangedphysiology.com/main/cicm-primary-exam/required-reading/renal-system/Chapter023/acetazolamide`.

[7] Prescribers' Digital Reference, Acetylcysteine. `https://www.pdr.net/drug-summary/Acetylcysteine-acetylcysteine-668`.

[8] Latini, R.; Tognoni, G.; Kates, R. E. Clinical Pharmacokinetics of Amiodarone. **1984**, *156*, 136–156.

[9] Lennernäs, H. Clin Pharmacokinet 2003; 42 (13): 1141-1160. *Clin Pharmacokinet* **2003**, *42*, 1141–1160.

[10] Dudkowski, C.; Karim, A.; Zhao, Z.; Alonso, A. B.; Garg, D.; Preston, R. A. Single-Center Evaluation of the Pharmacokinetics and Safety of the Angiotensin II Receptor Antagonist Azilsartan Medoxomil in Mild to Moderate Hepatic Impairment. *Journal of Clinical Pharmacology* **2018**, *58*, 48–56.

[11] Indications, T. Summary of product characteristics. *Pharmaceutical Medicine* **2014**, 87–89.

[12] HPRA, Carbocisteine. **2023**,

[13] Lieberman, P.; Hernandez-Trujillo, V.; Lieberman, J.; Frew, A. J. Antihistamines: Anti-Allergic, Anti-Inflammatory Activities of Second-Generation Antihistamines. *Clinical Immunology: Principles and Practice Expert Consult: Online and Print* **2008**, 1317–1329.

[14] Singer, J. M.; Hare, M. J. O.; Rehrn, C. R.; Zarembo, J. E. Jeff M. Singer, Michael. *Analytical Profiles of Drug Substances* **1985**, *14*.

[15] Davey, P. G. The pharmacokinetics of clarithromycin and its 14-OH metabolite. *Journal of Hospital Infection* **1991**, *19*, 29–37.

[16] Wilby, M. J.; Hutchinson, P. J. The pharmacology of chlormethiazole: A potential neuroprotective agent? *CNS Drug Reviews* **2004**, *10*, 281–294.

[17] Thorn, C. F.; Müller, D. J.; Altman, R. B.; Klein, T. E. PharmGKB summary: Clozapine pathway, pharmacokinetics. *Pharmacogenetics and Genomics* **2018**, *28*, 214–222.

[18] Blech, S.; Ebner, T.; Ludwig-Schwellinger, E.; Stangier, J.; Roth, W. The metabolism and disposition of the oral direct thrombin inhibitor, dabigatran, in humans. *Drug Metabolism and Disposition* **2008**, *36*, 386–399.

[19] Damia, G.; Dlncalci, M. Clinical Pharmacokinetics of Altretamine. *Clinical Pharmacokinetics* **1995**, *28*, 439–448.

[20] Al-Zehouri, J.; El-Subbagh, H. I.; Al-Badr, A. A. Lansoprazole. *Analytical Profiles of Drug Substances and Excipients* **2001**, *28*, 117–151.

[21] Liang, I. E.; Boyer, E. W. *Haddad and Winchester's Clinical Management of Poisoning and Drug Overdose, Fourth Edition*, fourth edi ed.; Elsevier Inc., 2007; pp 773–779.

[22] Magarey, J. Dextromethorphan. 1996; `https://inchem.org/documents/pims/pharm/pim179.htm#SectionTitle:6.4Metabolism`.

[23] Hufschmid, E.; Theurillat, R.; Martin, U.; Thormann, W. Exploration of the metabolism of dihydrocodeine via determination of its metabolites in human urine using micellar electrokinetic capillary chromatography. *Journal of Chromatography B: Biomedical Sciences and Applications* **1995**, *668*, 159–170.

[24] Soghoian, S. Medscape - Disulfiram. 2022; `https://emedicine.medscape.com/article/814525-overview#a5`.

[25] Lantz, R. J.; Gillespie, T. A.; Rash, T. J.; Kuo, F.; Skinner, M.; Kuan, H. Y.; Knadler, M. P. Metabolism, excretion, and pharmacokinetics of duloxetine in healthy human subjects. *Drug Metabolism and Disposition* **2003**, *31*, 1142–1150.

[26] Parasrampuria, D. A.; Truitt, K. E. Pharmacokinetics and Pharmacodynamics of Edoxaban, a Non-Vitamin K Antagonist Oral Anticoagulant that Inhibits Clotting Factor Xa. *Clinical Pharmacokinetics* **2016**, *55*, 641–655.

[27] Ward, B.; Gorski, C.; Jones, D.; Hall, S.; Flockhart, D.; Desta, Z. The cytochrome P450 2B6 (CYP2B6) is the main catalyst of efavirenz primary and secondary metabolism: implication for HIV/AIDS therapy and utility of efavirenz as a substrate marker of CYP2B6 catalytic activity. *J Pharmacol Exp Ther.* **2003**, *306*, 287–300.

[28] Ogburn, E. T.; Jones, D. R.; Masters, A. R.; Xu, C.; Guo, Y.; Desta, Z. Efavirenz primary and secondary metabolism in vitro and in vivo: Identification of novel metabolic pathways and cytochrome P450 2A6 as the principal catalyst of efavirenz 7-hydroxylation. *Drug Metabolism and Disposition* **2010**, *38*, 1218–1229.

[29] Asconapé, J. J. Use of antiepileptic drugs in hepatic and renal disease. *Handbook of Clinical Neurology* **2014**, *119*, 417–432.

[30] Aurobindo, EMC - Flucloxacillin. `https://www.medicines.org.uk/emc/product/545/smpc#gref`.

[31] MedSafe, Flucloxacillin. **2019**, 1–11.

[32] Altamura, A. C.; Moro, A. R.; Percudani, M. Clinical Pharmacokinetics of Fluoxetine. *Clinical Pharmacokinetics* **1994**, *26*, 201–214.

[33] Huang, X.; Dorhout Mees, E.; Vos, P.; Hamza, S.; Braam, B. Everything we always wanted to know about furosemide but were afraid to ask. *American Journal of Physiology - Renal Physiology* **2016**, *310*, F958–F971.

[34] Pichette, V.; Du Souich, P. Role of the kidneys in the metabolism of furosemide: Its inhibition by probenecid. *Journal of the American Society of Nephrology* **1996**, *7*, 345–349.

[35] Ávilagálvez, M. Giménezbastida, J. A.; Gonzálezsarrías, A.; Espín, J. C. New insights into the metabolism of the flavanones eriocitrin and hesperidin: A comparative human pharmacokinetic study. *Antioxidants* **2021**, *10*, 1–20.

[36] Khoo, K.-C.; Reik, D.; Colburn, W. A. Pharmacokinetics of Isotretinoin Following a Single Oral Dose. *The Journal of Clinical Pharmacology* **1982**, *22*, 395–402.

[37] FDA, Labetalol. 2–12.

[38] Agwuh, K. N.; MacGowan, A. Pharmacokinetics and pharmacodynamics of the tetracyclines including glycylcyclines. *Journal of Antimicrobial Chemotherapy* **2006**, *58*, 256–265.

[39] Pfizer, Mefenamic acid.

[40] Bachmann, F.; Meyer zu Schwabedissen, H. E.; Duthaler, U.; Krähenbühl, S. Cytochrome P450 1A2 is the most important enzyme for hepatic metabolism of the metamizole metabolite 4-methylaminoantipyrine. *British Journal of Clinical Pharmacology* **2022**, *88*, 1885–1896.

[41] Inturrisi, C. E.; Colburn, A., Wayne; Kaiko, R. F.; Houde, R. W.; Foley, K. M. Pharmacokinetics and pharmacodynamics of methadone in patients with chronic pain. *Clinical Pharmacology & Therapeutics* **1987**, *41*, 392–401.

[42] Ferrari, A.; Coccia, C. P. R.; Bertolini, A.; Sternieri, E. Methadone - Metabolism, pharmacokinetics and interactions. *Pharmacological Research* **2004**, *50*, 551–559.

[43] FDA, Metoclopramide. **2017**,

[44] Shakhatreh, M.; Jehangir, A.; Malik, Z.; Parkman, H. P. Metoclopramide for the treatment of diabetic gastroparesis. *Expert Review of Gastroenterology and Hepatology* **2019**, *13*, 711–721.

[45] Takusagawa, S.; Van Lier, J. J.; Suzuki, K.; Nagata, M.; Meijer, J.; Krauwinkel, W.; Schaddelee, M.; Sekiguchi, M.; Miyashita, A.; Iwatsubo, T.; Van Gelderen, M.; Usui, T. Absorption, metabolism and excretion of [14C]mirabegron (YM178), a potent and selective β 3-adrenoceptor agonist, after oral administration to healthy male volunteers. *Drug Metabolism and Disposition* **2012**, *40*, 815–824.

[46] Timmer, C. J. Timmer, C. J., Ad Sitsen, J. M., & Delbressine, L. P. 2000. Clinical Pharmacokinetics of Mirtazapine. Clinical Pharmacokinetics, 38(6), 461474..pdf. **2000**, *38*, 461–474.

[47] Hasslesstrom, J.; Sawe, J. Morphine pharmacokinetics and metabolism in humans. Enterohepatic cycling and relative contribution of metabolites to active opioid concentrations. *Clin Pharmacokinetics* **2001**, *40*, 344–345.

[48] Harlev, C.; Houlind, K. C.; Dyrby, J.; Rai, A. Intermittent claudication. *Ugeskrift for laeger* **2021**, *183*, 967–977.

[49] MIMS, Naftidrofuryl. https://sso.mims.com/Account/acknowledge?email=corina.meyer@eawag.ch&country=CH&ssouserid=3841376&isManualVerify=False&returnUrl=https%3A%2F%2Fwww.mims.com%2Fmalaysia%2Fdrug%2Finfo%2Fnaftidrofuryl%3Fmtype%3Dgeneric.

[50] Fongemie, J.; Felix-Getzik, E. A Review of Nebivolol Pharmacology and Clinical Evidence. *Drugs* **2015**, *75*, 1349–1371.

[51] Delbeke, F. T.; Diergeneeskunde, F.; Gent, R. They investigating. 116–123.

[52] Hoener, B.-A.; Patterson, S. E. Nitrofurantoin disposition. *Clinical Pharmacaology & Therapeutics* **1981**, *29*, 808–816.

[53] Stein, G. E. Review of the bioavailability and pharmacokinetics of oral norfloxacin. *The American Journal of Medicine* **1987**, *82*, 18–21.

[54] Greenblatt, D. J. Clinical Pharmacokinetics of Oxazepam and Lorazepam. *Clinical Pharmacokinetics* **1981**, *6*, 89–105.

[55] Kwilas, A. R.; Donahue, R. N.; Tsang, K. Y.; Hodge, J. W. HHS Public Access. *Cancer Cell* **2015**, *2*, 1–17.

[56] Barza, M.; Weinstein, L. Pharmacokinetics of the Penicillins in Man. 1976.

[57] Devissaguet, J.; Ammoury, N.; Devissaguet, M.; Perret, L. Pharmacokinetics of perindopril and its metabolites in healthy volunteers. *Fundamental & Clinical Pharmacology* **1990**, *4*, 175–189.

[58] IARC Monographs, IARC Monograph on the Carcinogenic Risks to Humans. *International agency on research for cancer* **2000**, *76*, 387–415.

[59] Hatanaka, T. Clinical pharmacokinetics of pravastatin: Mechanisms of pharmacokinetic events. *Clinical Pharmacokinetics* **2000**, *39*, 397–412.

[60] Schijvens, A. M.; ter Heine, R.; de Wildt, S. N.; Schreuder, M. F. Pharmacology and pharmacogenetics of prednisone and prednisolone in patients with nephrotic syndrome. *Pediatric Nephrology* **2019**, *34*, 389–403.

[61] Ipsen, Product Monograph Therapeutic Product Monograph. *Toxicology* **2015**, 1–17.

[62] Siddoway, L. A.; Roden, D. M.; Woosley, R. L. Clinical pharmacology of propafenone: Pharmacokinetics, metabolism and concentration-response relations. *The American Journal of Cardiology* **1984**, *54*, 9D–12D.

[63] Walle, T.; Walle, U.; Olanoff, L. Quantitative account of propranolol metabolism in urine of normal man. *Drug Metabolism and Disposition* **1985**, *13*, 204–209.

[64] Sahinovic, M. M.; Struys, M. M.; Absalom, A. R. Clinical Pharmacokinetics and Pharmacodynamics of Propofol. *Clinical Pharmacokinetics* **2018**, *57*, 1539–1558.

[65] Kassahun, K.; McIntosh, I.; Cui, D.; Hreniuk, D.; Merschman, S.; Lasseter, K.; Azrolan, N.; Iwamoto, M.; Wagner, J. A.; Wenning, L. A. Metabolism and disposition in humans of raltegravir (MK-0518), an anti-AIDS drug targeting the human immunodeficiency virus 1 integrase enzyme. *Drug Metabolism and Disposition* **2007**, *35*, 1657–1663.

[66] Sawyer, J. Raltgravir. *Antimicrobe*

[67] Kortejärvi, H.; Yliperttula, M.; Dressman, J. B.; Junginger, H. E.; Midha, K. K.; Shah, V. P.; Barends, D. M. Biowaiver monographs for immediate release solid oral dosage forms: Ranitidine hydrochloride. *Journal of Pharmaceutical Sciences* **2005**, *94*, 1617–1625.

[68] Hartigan-Go, K. INCHEM - Rifampicin. 1990; `https://inchem.org/documents/pims/pharm/rifam.htm#SectionTitle:6.4Metabolism`.

[69] Weinborn, V.; Lehmkuhler, A. L.; Zyba, S. J.; Haskell, M. J.; Morel, F. B.; Zeilani, M.; Mitchell, A. E. Measurement of Saccharin and trans-Resveratrol Metabolites in Urine as Adherence Markers for Small Quantity Lipid-Based Nutrient Supplement Consumption. *Journal of Agricultural and Food Chemistry* **2021**, *69*, 1107–1114.

[70] Bijak, M. Silybin, a Major Bioactive Component of Milk Thistle (Silybum marianum L. Gaernt.)Chemistry, Bioavailability, and Metabolism. *Molecules* **2017**, *22*, 1–11.

[71] Cid-Conde, L.; Lopez-Castro, J. *Cardiovascular Risk Factors in Pathology*; 2020; Chapter Pharmacoki.

[72] AHSP, Sulfasalazine (Monograph). 2023; https://www.drugs.com/monograph/sulfasalazine.html.

[73] Stangier, J.; Schmid, J.; Türck, D.; Switek, H.; Verhagen, A.; Peeters, P. A.; van Marle, S. P.; Tamminga, W. J.; Sollie, F. A.; Jonkman, J. H. Absorption, metabolism, and excretion of intravenously and orally administered [14C]telmisartan in healthy volunteers. *Journal of Clinical Pharmacology* **2000**, *40*, 1312–1322.

[74] Ghodke-Puranik, Y.; Thorn, C. F.; Lamba, J. K.; Leeder, J. S.; Song, W.; Birnbaum, A. K.; Altman, R. B.; Klein, T. E. Valproic acid pathway: Pharmacokinetics and pharmacodynamics. *Pharmacogenetics and Genomics* **2013**, *23*, 236–241.

[75] Patel, S.; Preuss, C. V.; Bernice, F. StatPearls - Vancomycin. 2023; https://www.ncbi.nlm.nih.gov/books/NBK459263/#:~:text=Theeliminationhalf-lifeis,predominantlygetsexcretedinfeces.

[76] Bakkes, J.; Cheatle, M.; Mžavanadze, N.; Pintér, L.; Witt, R. G. Annex I:. *Keeping the Worlds Environment under Review* **2022**, 421–434.

Supporting Information C

Supporting Pages: S20 - S78
Supporting Figures: SI-C1 - SI-C19
Supporting Tables: SI-C1 - SI-C62

Contents

SI-C1 Peak Picking Tool Comparison **S25**
SI-C1.1 Introduction S25
SI-C1.2 Peak Picking Tools S29
SI-C1.2.1 Compound Discoverer S29
SI-C1.2.2 MZmine S33
SI-C1.2.3 MS-DIAL S39
SI-C1.2.4 SLAW S42
SI-C1.3 R Analysis S46
SI-C1.4 Results and Discussion S47

SI-C2 Materials & Methods **S48**
SI-C2.1 Wastewater Sample Collection S48
SI-C2.2 Wastewater Sample Preparation S48
SI-C2.3 Human Liver S9 Incubation S48
SI-C2.4 Online-SPE-HPLC-HRMS/MS S49
SI-C2.5 Quality Control S51
SI-C2.6 Processing with Compound Discoverer S55
SI-C2.7 Export of MS2 Information S65
SI-C2.8 *In Silico* and Machine Learning Tools S65
SI-C2.8.1 SIRIUS/CSI:FingerID S65
SI-C2.8.2 MetFrag S66
SI-C2.8.3 FISh Scoring S67
SI-C2.9 Molecular Networking S68
SI-C2.10 Retention Time Prediction S69
SI-C2.11 Confidence Score Calculation S70
SI-C2.12 Semiquantification S71
SI-C2.13 Phase II Metabolite Search: Glucuronide, Sulfate, Glutathione S73
SI-C2.14 Summarizing Information S74

SI-C3 Suspect Screening - Exclusion Criteria **S75**

List of Figures

SI-C1 Compound Discoverer workflow S29
SI-C2 MZmine workflow S33
SI-C3 MS-DIAL workflow S39
SI-C4 General workflow of SLAW, adapted from.[1] S42
SI-C5 Scatterplot of precision and recall of the analyzed tools. The F_1 isocost lines are given in gray. S47
SI-C6 Wastewater sample preparation scheme. S48
SI-C7 Relative recoveries in the influents of the three WWTPs Altenrhein, Neugut and Werdhoelzli. Compounds with and without own ILIS are depicted separately. . . S52
SI-C8 Absolute recoveries in the influents of the three WWTPs Altenrhein, Neugut and Werdhoelzli. Compounds with and without own ILIS are depicted separately. . . S53
SI-C9 Matrix factors in the influents of the three WWTPs Altenrhein, Neugut and Werdhoelzli. Compounds with and without own ILIS are depicted separately. . . S53
SI-C10 Matrix LOQs in the influents of the three WWTPs Altenrhein, Neugut and Werdhoelzli. S54
SI-C11 Compound Discoverer workflow. The node *Compound Class Scoring* was only applied in negative ionization mode. S55
SI-C12 Molecular network of positive ionization mode data. Colored dots indicate clusters which enabled the tentative identification of suspects. Black dots are other tentatively identified pharmaceuticals/pharmaceutical metabolites, gray dots indicate other compounds. S68
SI-C13 Molecular network of negative ionization mode data. Colored dots indicate clusters which enabled the tentative identification of suspects. Black dots are other tentatively identified pharmaceuticals/pharmaceutical metabolites, gray dots indicate other compounds. S69
SI-C14 Retention time prediction based on predicted $logD_{OW}$ values at chromatographic pH against measured retention time of 369 compounds in the positive ionization mode. S69
SI-C15 Retention time prediction based on predicted $logD_{OW}$ values at chromatographic pH against measured retention time of 81 compounds in the negative ionization mode. S70
SI-C16 Ratios of predicted (MS2Quant) and experimental concentrations of the 15 test compounds. The retention time in minutes is indicated next to the compound. . S72
SI-C17 Principal component analysis of the MS2Quant training set compounds and the quantified suspects based on 144 PaDEL descriptors. S72
SI-C18 Intensities of internal standards measured in the calibration curves of the two different measurement batches in the negative mode. Internal standards are ordered by increasing retention time. S73
SI-C19 Intensity distribution of components prioritized based on exact mass of the suspect list in the positive and negative ionization mode, respectively. For components in brown an MS2 spectrum was acquired, while green components do not have an MS2 spectrum. The dashed line indicates the intensity cutoff. S75

List of Tables

SI-C1 Chosen samples for peak picking tool comparison. S26
SI-C2 Chosen isotopically labeled internal standards for peak picking tool comparison. The retention time refers to the sample, which was used for the alignment. S27
SI-C3 Parameter specifications of the *Select Spectra* node in Compound Discoverer. . . S30
SI-C4 Parameter specifications of the *Align Retention Times (ChromAlign)* node in Compound Discoverer. S30
SI-C5 Parameter specifications of the *Detect Compounds* node in Compound Discoverer.S31
SI-C6 Parameter specifications of the *Group Compounds* node in Compound Discoverer.S32
SI-C7 Parameter specifications of the *Fill Gaps* node in Compound Discoverer. S32
SI-C8 Parameter specifications of the *Mark Background Compounds* node in Compound Discoverer. S32
SI-C9 Parameter specifications of the *HPLC presets* in MZmine. S34
SI-C10 Parameter specifications of the *Mass spectrometer presets* in MZmine. S34
SI-C11 Parameter specifications of the *Import MS data* module in MZmine. S34
SI-C12 Parameter specifications of the *Mass detection* module in MZmine. S35
SI-C13 Parameter specifications of the *ADAP Chromatogram Builder* module in MZmine.S35
SI-C14 Parameter specifications of the *Smoothing* module in MZmine. S35
SI-C15 Parameter specifications of the *Local minimum feature resolver* module in MZmine.S36
SI-C16 Parameter specifications of the *13C isotope filter (formerly: isotope grouper)* module in MZmine. S36
SI-C17 Parameter specifications of the *Isotopic peaks finder* module in MZmine. . . . S36
SI-C18 Parameter specifications of the *Join aligner* module in MZmine. S37
SI-C19 Parameter specifications of the *Feature list rows filter* module in MZmine. . . . S37
SI-C20 Parameter specifications of the *Peak finder (multithreaded)* module in MZmine. S38
SI-C21 Parameter specifications of the *Duplicate peak filter* module in MZmine. S38
SI-C22 Parameter specifications of the *Start up a project* step in MS-DIAL. S40
SI-C23 Parameter specifications of the *Data collection* step in MS-DIAL. S40
SI-C24 Parameter specifications of the *Peak detection* step in MS-DIAL. S41
SI-C25 Parameter specifications of the *MS2Dec* step in MS-DIAL. S41
SI-C26 Parameter specifications of the *Adduct* step in MS-DIAL. S41
SI-C27 Parameter specifications of the *Alignment* step in MS-DIAL. S41
SI-C28 Parameter specifications of the *Filtering* step in SLAW. S43
SI-C29 Parameter specifications of the *Grouping* step in SLAW. S43
SI-C30 Parameter specifications of the *Ion annotation* step in SLAW. S44
SI-C31 Parameter specifications of the *Optimization* step in SLAW. Values separated by an arrow indicate initial and optimized parameters, respectively. S44
SI-C32 Parameter specifications of the *Output format* in SLAW. S45
SI-C33 Parameter specifications of the *Peak picking* step in SLAW. Parameter specifications of SLAW. Values separated by an arrow indicate initial and optimized parameters, respectively. S45
SI-C34 Precision, recall and F_1 scores of the five different peak picking algorithms. The different tools are ordered along decreasing F_1 scores. S47
SI-C35 Time schedule of the online-SPE. S50
SI-C36 Time schedule of the liquid chromatography. S50
SI-C37 ESI-HRMS/MS settings. S50
SI-C38 Parameter specifications of the *Select Spectra* node in Compound Discoverer. . S56
SI-C39 Parameter specifications of the *Align Retention Times (ChromAlign)* node in Compound Discoverer. S56
SI-C40 Parameter specifications of the *Create Pattern Trace* node in Compound Discoverer.S57
SI-C41 Parameter specifications of the *Detect Compounds* node in Compound Discoverer.S57

SI-C42 Parameter specifications of the *Merge Features* node in Compound Discoverer. S58
SI-C43 Parameter specifications of the *Group Compounds* node in Compound Discoverer. S58
SI-C44 Parameter specifications of the *Fill Gaps* node in Compound Discoverer. . . . S58
SI-C45 Parameter specifications of the *Search mzCloud* node in Compound Discoverer. S59
SI-C46 Parameter specifications of the *Search mzVault* node in Compound Discoverer. S60
SI-C47 Parameter specifications of the *Predict Compositions* node in Compound Discoverer. S60
SI-C48 Parameter specifications of the *Search Mass Lists* node in Compound Discoverer. S61
SI-C49 Parameter specifications of the *Apply Spectral Distance* node in Compound Discoverer. S61
SI-C50 Parameter specifications of the *Apply mzLogic* node in Compound Discoverer. . S61
SI-C51 Parameter specifications of the *Calculate Mass Defect* node in Compound Discoverer. S62
SI-C52 Parameter specifications of the *Assign Compound Annotation* node in Compound Discoverer. S62
SI-C53 Parameter specifications of the *Generate Molecular Networks* node in Compound Discoverer. S63
SI-C54 Parameter specifications of the *Pattern Scoring* node in Compound Discoverer. S63
SI-C55 Parameter specifications of the *Search Neutral Losses* node in Compound Discoverer. S64
SI-C56 Parameter specifications of the *Compound Class Scoring* node in Compound Discoverer. This node was only applied in the negative ionization mode. S64
SI-C57 Parameter settings of SIRIUS/CSI:FingerID. S66
SI-C58 Parameter settings of MetFrag. S67
SI-C59 Parameter settings of FISh Scoring. S67
SI-C60 Identification point system for suspect and non-target HRMS/MS analysis. Adapted from.[2] S71
SI-C61 Specific MSn fragments of glucuronide.[3] S74
SI-C62 Specific MSn fragments of glutathione.[4] S74

SI-C1 Peak Picking Tool Comparison

SI-C1.1 Introduction

To achieve the best basis for a successful suspect screening, different data processing tools were compared for the acquired data set in advance. The tools are based on different peak picking, retention time alignment and componentization algorithms, which exhibit disparate weaknesses and strengths for different types of samples and matrices. To achieve an optimal processing for a given data set, it is worth to identify the most suitable algorithms and to optimize the respective input parameters. For the conducted comparison, four different tools were chosen, namely Compound Discoverer 3.3,[5] MZmine 3.1.0,[6] MS-DIAL 4.90,[7] and SLAW.[1] SLAW has three different underlying peak pickers implemented, of which centWave from XCMS[8] and FeatureFinderMetabo from openMS[9] were tested. The third algorithm, ADAP from MZmine, is already covered by testing MZmine itself. For Compound Discoverer, raw files served as input, while for the other tools the data were converted to the mzML format by ProteoWizard,[10] including centroiding of the data. Besides these four tools, we further evaluated enviMass,[11] but failed to obtain reasonable starting parameters for our system. Therefore, these data were not considered in the final analysis.

For comparison, a small subdataset consisting of 27 sample and three blank raw data files was chosen, representing the whole measurement period and different wastewater influent matrices. A list of these files is given in Table SI-C1. We screened the pre-processed data for 50 ILIS (see Table SI-C2) spiked to all samples and manually verified to consistently show a single chromatographic peak in the selected samples. The working hypothesis was that correct peak pre-processing would result in one, and only one, matched peak within a reasonable RT window and consistent intensity. The absence of a match was treated as a false negative, and matches beyond the first (indicating peaks picked from noise, peak splitting, or alignment problems) were counted as false positives. We note that these are not true statistical values, as e.g. the number of total observations considered is not constant across the methods, but sustain that our approach provides a concise evaluation that overlaps well with practitioners' intuition. The obtained values of true positives, false positives and false negatives were used to calculate the recall rate and the precision of each tool. The precision is defined as the number of true positives (TP), divided by the number of all positive results, including the false positives (FP):

$$precision = \frac{TP}{TP + FP} \quad . \tag{1}$$

By dividing the true positives by the number of all samples that should have been identified as positive, one obtains the recall, defined by

$$recall = \frac{TP}{TP + FN} \quad , \tag{2}$$

where FN are the false negatives. To measure the accuracy, these two values are combined in the F_1 score, given by

$$F_1\ score = 2 \cdot \frac{precision \cdot recall}{precision + recall} \quad . \tag{3}$$

For each tool, a simple standard workflow was chosen and most parameters were kept at the default value for the first run, except the m/z range and the inter-sample retention time shift, which were known from the experimental setup and the previous target screening, respectively.

The parameter settings were optimized by adapting a single parameter in each run and comparing it to the initial run, by calculating precision, recall and F_1 score. If an improvement was observed, the parameter was increased or decreased in the chosen direction as long as no significant change was observed anymore. In case of an impairment, the parameter was changed into the other direction. This procedure was repeated for a few parameters, which were expected to have the biggest influence on the quality of the peak picking. SLAW has an integrated parameter optimization, which made the previously described procedure unnecessary. In the following Section SI-C1.2, the working principle of the different algorithms and the workflow are described in detail. The optimized input parameters are given, as well as an explanation of the followup analysis of the resulting peak tables with R.[12]

Table SI-C1: Chosen samples for peak picking tool comparison.

WWTP	Day	Date	Replicates	Sample type
Altenrhein	Tuesday	2022/03/01	R1-R3	24 h composite sample
	Thursday	2022/03/03	R1-R3	24 h composite sample
	Monday-Friday	2022/02/28-03/04	R1-R3	Mixed QC sample
Neugut	Tuesday	2022/03/01	R1-R3	24 h composite sample
	Thursday	2022/03/03	R1-R3	24 h composite sample
	Monday-Friday	2022/02/28-03/04	R1-R3	Mixed QC sample
Werdhoelzli	Tuesday	2022/03/01	R1-R3	24 h composite sample
	Thursday	2022/03/03	R1-R3	24 h composite sample
	Monday-Friday	2022/02/28-03/04	R1-R3	Mixed QC sample

Table SI-C2: Chosen isotopically labeled internal standards for peak picking tool comparison. The retention time refers to the sample, which was used for the alignment.

Compound	Adduct	CAS-No.	Monoisotopic mass [g/mol]	Molecular formula	RT [min]
4-Acetamidoantipyrine-D3	$[M+H]^+$	342821-66-3	248.1353	$C_{13}H_{12}\ {}^2H_3N_3O_2$	12.88
4-Aminoantipyrine-D3	$[M+H]^+$	68229-55-0	206.1247	$C_{11}H_{10}\ {}^2H_3N_3O$	10.76
4-Formylaminoantipyrine-D3	$[M+H]^+$	-	234.1196	$C_{12}H_{10}\ {}^2H_3N_3O_2$	12.74
Aliskiren-D6	$[M+H]^+$	-	557.4311	$C_{30}H_{47}\ {}^2H_6N_3O_6$	18.53
Amisulpride-D5	$[M+H]^+$	1216626-17-3	374.2036	$C_{17}H_{22}\ {}^2H_5N_3O_4S$	11.92
Amphetamine-D6	$[M+H]^+$	73758-26-6	141.1425	$C_9H_7\ {}^2H_6N$	12.00
Atazanavir-D5	$[M+H]^+$	1132747-14-8	709.4211	$C_{38}H_{47}\ {}^2H_5N_6O_7$	21.58
Atenolol acid-D5	$[M+H]^+$	1215404-47-9	272.1784	$C_{14}H_{16}\ {}^2H_5NO_4$	12.00
Atenolol-D7	$[M+H]^+$	1202864-50-3	273.2070	$C_{14}H_{15}\ {}^2H_7N_2O_3$	9.76
Azoxystrobin-D4	$[M+H]^+$	1346606-39-0	407.1419	$C_{22}H_{13}\ {}^2H_4N_3O_5$	20.07
Benzoylecgonine-D3	$[M+H]^+$	115732-68-8	292.1502	$C_{16}H_{16}\ {}^2H_3NO_4$	13.97
Bosentan-D4	$[M+H]^+$	1065472-77-6	555.2090	$C_{27}H_{25}\ {}^2H_4N_5O_6S$	20.88
Cocaine-D3	$[M+H]^+$	65266-73-1	306.1659	$C_{17}H_{18}\ {}^2H_3NO_4$	13.83
Dextrorphan-D3	$[M+H]^+$	524713-57-3	260.1968	$C_{17}H_{20}\ {}^2H_3NO$	13.8
Dihydrocodeine-D6	$[M+H]^+$	-	301.1678	$C_{18}H_{17}\ {}^2H_6NO_3$	10.37
EDDP-D3	$[M+H]^+$	-	280.2019	$C_{20}H_{20}\ {}^2H_3N$	16.18
Ephedrine-D3	$[M+H]^+$	548786-03-4	168.1342	$C_{10}H_{12}\ {}^2H_3NO$	10.96
Fexofenadine-D6	$[M+H]^+$	548783-71-7	507.3256	$C_{32}H_{33}\ {}^2H_6NO_4$	17.55
Flecainide-D4	$[M+H]^+$	-	418.1629	$C_{17}H_{16}\ {}^2H_4N_2O_3F_6$	16.27
Ketamine-D4	$[M+H]^+$	-	241.1171	$C_{13}H_{12}\ {}^2H_4NOCl$	13.33
Ketoprofen-D3	$[M+H]^+$	159490-55-8	257.1131	$C_{16}H_{11}\ {}^2H_3O_3$	19.93
Lidocaine-D10	$[M+H]^+$	851528-09-1	244.2360	$C_{14}H_{12}\ {}^2H_{10}N_2O$	12.85
Losartan-D4	$[M+H]^+$	1030937-27-9	426.1873	$C_{22}H_{19}\ {}^2H_4N_6OCl$	19.48
Methadone-D9	$[M+H]^+$	1435933-74-6	318.2658	$C_{21}H_{18}\ {}^2H_9NO$	17.77
Methamphetamine-D5	$[M+H]^+$	60124-88-1	154.1518	$C_{10}H_{10}\ {}^2H_5N$	12.24
Midazolam-D4	$[M+H]^+$	-	329.1033	$C_{18}H_9\ {}^2H_4N_3ClF$	16.30
Naloxone-D5	$[M+H]^+$	1261079-38-2	332.1784	$C_{19}H_{16}\ {}^2H_5NO_4$	10.41
N-Desvenlafaxine-D3	$[M+H]^+$	-	266.2074	$C_{16}H_{22}\ {}^2H_3NO_2$	15.85

Oseltamivir acid-D3	$[M+H]^+$	1242184-43-5	287.1924	$C_{14}H_{21}\,^{2}H_3N_2O_4$	14.20
Oseltamivir-D5	$[M+H]^+$	-	317.2363	$C_{16}H_{23}\,^{2}H_5N_2O_4$	16.56
Pantoprazole-D3	$[M+H]^+$	922727-37-5	386.0940	$C_{16}H_{12}\,^{2}H_3N_3O_4SF_2$	17.48
Phenazone-D3 (antipyrine-D3)	$[M+H]^+$	-	191.1138	$C_{11}H_9\,^{2}H_3N_2O$	14.40
Prednisolone-D8	$[M+H]^+$	-	368.2439	$C_{21}H_{20}\,^{2}H_8O_5$	19.07
Prednisone-D7	$[M+H]^+$	-	365.2220	$C_{21}H_{19}\,^{2}H_7O_5$	18.41
Ranitidine-D6	$[M+H]^+$	-	320.1789	$C_{13}H_{16}\,^{2}H_6N_4O_3S$	9.81
Rivastigmine-D6	$[M+H]^+$	194930-04-6	256.2058	$C_{14}H_{16}\,^{2}H_6N_2O_2$	13.75
Saquinavir-D9	$[M+H]^+$	1356355-11-7	679.4408	$C_{38}H_{41}\,^{2}H_9N_6O_5$	19.10
Sitagliptin-D4	$[M+H]^+$	-	411.1432	$C_{16}H_{11}\,^{2}H_4N_5OF_6$	14.28
Sulpiride-D3	$[M+H]^+$	124020-27-5	344.1598	$C_{15}H_{20}\,^{2}H_3N_3O_4S$	9.56
Telmisartan-D3	$[M+H]^+$	1189889-44-8	517.2557	$C_{33}H_{27}\,^{2}H_3N_4O_2$	19.00
Tipranavir-D4	$[M+H]^+$	-	606.2313	$C_{31}H_{29}\,^{2}H_4N_2O_5SF_3$	22.12
Torasemide-D7	$[M+H]^+$	1189375-06-1	355.1695	$C_{16}H_{13}\,^{2}H_7N_4O_3S$	17.06
Trazodone-D6	$[M+H]^+$	1181578-48-2	377.1889	$C_{19}H_{16}\,^{2}H_6N_5OCl$	15.21
Trimethoprim-D9	$[M+H]^+$	1189460-62-5	299.1944	$C_{14}H_9\,^{2}H_9N_4O_3$	12.35
Trimipramine-D3	$[M+H]^+$	-	297.2284	$C_{20}H_{23}\,^{2}H_3N_2$	18.08
Valsartan acid-D4	$[M+H]^+$	-	270.1055	$C_{14}H_6\,^{2}H_4N_4O_2$	17.41
Vardenafil-D5	$[M+H]^+$	1189685-70-8	493.2520	$C_{23}H_{27}\,^{2}H_5N_6O_4S$	17.17
Venlafaxine-D6	$[M+H]^+$	1062606-12-5	283.2418	$C_{17}H_{21}\,^{2}H_6NO_2$	15.70
Vildagliptin-13C-15N	$[M+H]^+$	1044741-01-6	309.2085	$C_{12}\,^{13}C_5H_{25}N_2\,^{15}NO_2$	9.90
Zolpidem-D6	$[M+H]^+$	959605-90-4	313.2061	$C_{19}H_{15}\,^{2}H_6N_3O$	14.40

SI-C1.2 Peak Picking Tools

SI-C1.2.1 Compound Discoverer

Compound Discoverer (CD) is the commercial software for suspect and non-target screening of Thermo Fisher Scientific. The workflow can be assembled manually and in a flexible manner from workflow nodes. These nodes encompass peak picking, alignment of retention time, gap filling, suspect list and MS2 database searches, composition prediction, neutral loss and specific fragment searches, molecular networking, and several statistical evaluations. The resulting merged feature list can be exported and used for further data analysis. Underlying algorithms of the different nodes are not published. In this project, the latest released version CD 3.3 was applied.

For this comparison, a simple linear workflow consisting of seven nodes was assembled. After file input and spectra selection, the retention times of the different files are aligned according to an indicated reference file. The detected compounds are then grouped and gaps filled, before background compounds based on the given blank files are marked. For all utilized tools which required the indication of a retention time alignment reference file, the first replicate of the mixed quality control sample of Werdhoelzli was used. The optimized parameters of the workflow are given in Tables SI-C3 to SI-C8.

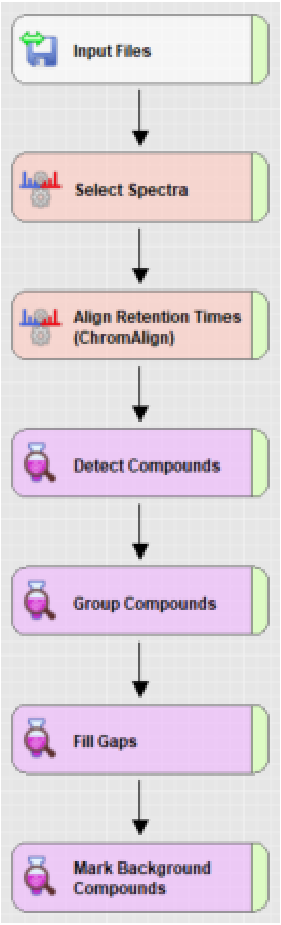

Figure SI-C1: Compound Discoverer workflow

Table SI-C3: Parameter specifications of the *Select Spectra* node in Compound Discoverer.

**1. Spectrum Properties Filter**	
Lower RT Limit	0
Upper RT Limit	0
First Scan	0
Last Scan	0
Ignore Specified Scans	
Lowest Charge State	0
Highest Charge State	0
Min. Precursor Mass	100 Da
Max. Precursor Mass	1000 Da
Total Intensity Threshold	0
Minimum Peak Count	1
**2. Scan Event Filters**	
Mass Analyzer	Any
MS Order	Any
Activation Type	Any
Min Collision Energy	0
Max. Collision Energy	1000
Scan Type	Any
Polarity Mode	Any
MS1 Mass Range	
FAIMS CV	
**3. Peak Filters**	
S/N Threshold (FT-only)	1.5
**4. Replacements for Unrecognized Properties**	
Unrecognized Charge Replacements	1
Unrecognized Mass Analyzer Replacements	FTMS
Unrecognized MS Order Replacements	MS2
Unrecognized Activation Type Replacements	CID
Unrecognized Polarity Replacements	+
Unrecognized MS Resolution@200 Replacements	140000
Unrecognized MSn Resolution@200 Replacements	17500
**5. General Settings**	
Precursor Selection	Use MS(n-1) Precursor
Use Isotope Pattern in Precursor Reevaluation	True
Provide Profile Spectra	Automatic
Store Chromatograms	False

Table SI-C4: Parameter specifications of the *Align Retention Times (ChromAlign)* node in Compound Discoverer.

**1. General Settings**	
Reference File	Werdhoelzli, mixed QC sample, first replicate

Table SI-C5: Parameter specifications of the *Detect Compounds* node in Compound Discoverer.

**1. General Settings**	
Mass Tolerance [ppm]	5 ppm
Min. Peak Intensity	10000
Min. # Scans per Peak	5
Use Most Intense Isotope Only	True
**2. Trace Detection**	
Max. Number of Gaps to Correct	2
Min. Number of Adjacent Non-Zeros	2
**3. Peak Detection**	
Chromatographic S/N Threshold	1.5
Remove Baseline	FALSE
Gap Ratio Threshold	0.35
Max. Peak Width [min]	1
Min. Relative Valley Depth	0.1
**4. Isotope Pattern Detection**	
Group Isotopes for	Br; Cl
Use Peak Quality for Isotope Grouping	True
Filter out Features with Bad Peaks Only	True
Zig-Zag Index Threshold	0.2
Jaggedness Threshold	0.4
Modality Threshold	0.9
Remove Potentially False Positive Isotopes	True
**5. Compound Detection**	
Ions	$[2M+H]^{+1}$; $[M+2H]^{+2}$; $[M+H]^{+1}$; $[M+Na]^{+1}$; $[M+NH_4]^{+1}$
Base Ions	$[M+H]^{+1}$
Remove Singlets	True
**6. AcquireX Settings**	
Detect Persistent Background Ions	False

Table SI-C6: Parameter specifications of the *Group Compounds* node in Compound Discoverer.

**1. General Settings**	
Mass Tolerance	5 ppm
RT Tolerance [min]	1
Align Peaks	False
Preferred Ions	$[M+H]^{+1}$
Area Integration	Most Common Ion
**2. Peak Rating Contributions**	
Area Contribution	3
CV Contribution	10
FWHM to Base Contribution	5
Jaggedness Contribution	5
Modality Contribution	5
Zig-Zag Index Contribution	5
**3. Peak Rating Filter**	
Peak Rating Threshold	0
Number of Files	0

Fill Gaps:

Table SI-C7: Parameter specifications of the *Fill Gaps* node in Compound Discoverer.

**1. General Settings**	
Mass Tolerance	5 ppm
S/N Threshold	1.5
Use Real Peak Detection	True
Apply Restrictive Gap Filling	True

Mark Background Compounds:

Table SI-C8: Parameter specifications of the *Mark Background Compounds* node in Compound Discoverer.

**1. General Settings**	
Max. Sample/Blank	5
Max. Blank/Sample	0
Hide Background	True

SI-C1.2.2 MZmine

MZmine is an open-source data processing tool for data generated by coupling separation to mass spectrometry. Besides data from liquid chromatography, it is also able to process gas chromatography or ion mobility data. Processing starts with mass detection in MS1 and MS2 spectra and continues with the ADAP chromatogram builder, which constructs extracted ion chromatograms (EICs) for masses that have been continuously detected in MS1 over a certain duration of time. In a next step, a smoothing of the EICs is applied, followed by isotopologue grouping, retention time alignment and gap filling. MZmine3 also enables downstream annotation, including molecular formula prediction, MS2 library search and direct interfaces to SIRIUS[13] and GNPS.[14] More details on MZmine and its underlying algorithms can be found in the respective publications.[6,15]

In MZmine, different data processing modules can be combined to a workflow. These modules encompass raw data processing, peak detection, peak list alignment, peak identification, visualization and statistical analysis. Figure SI-C2 shows the applied workflow, while the Tables SI-C9 to SI-C21 summarize the parameter settings of the different modules.

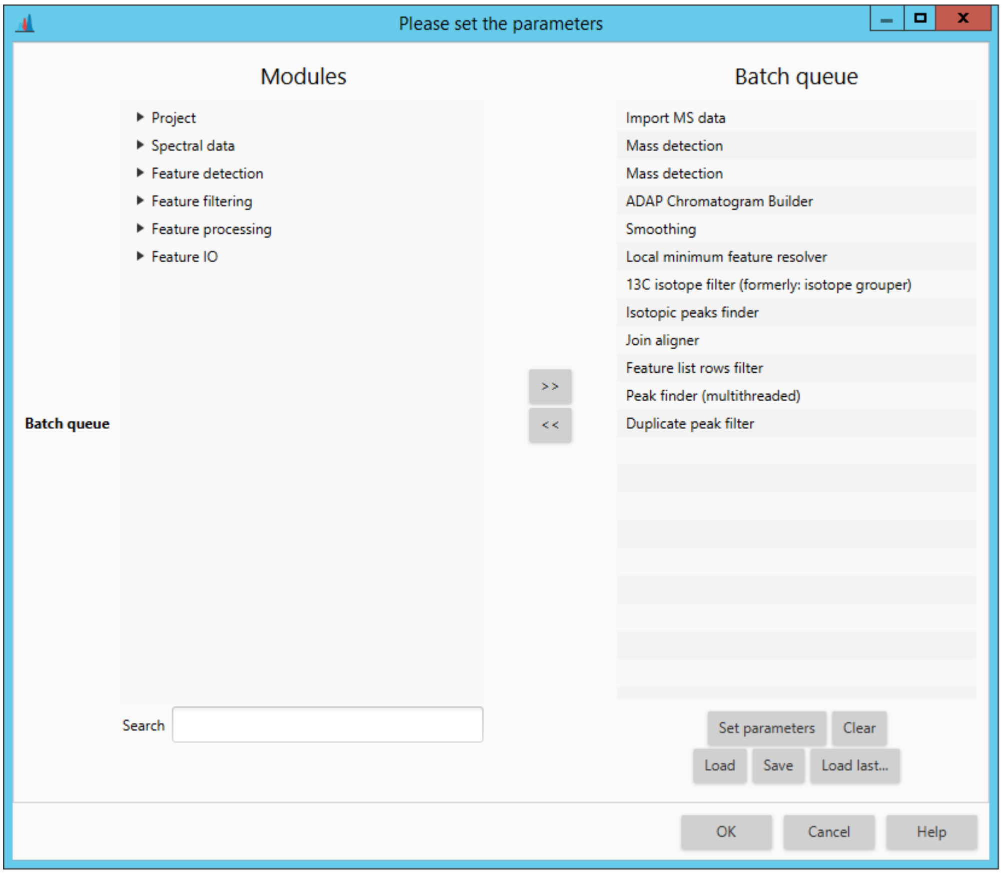

Figure SI-C2: MZmine workflow

Table SI-C9: Parameter specifications of the *HPLC presets* in MZmine.

	☑ HPLC<br>☐ UHPLC
Stable ionization across samples	☑
Crop retention time	5.00 - 30.00 min.
Max peaks in chromatogram	15
Min samples per alignment	2
Min # of data points	5
Approximate feature width	0.5 absolute (min)
Intra-sample RT tolerance	0.080 absolute (min)
Inter-sample RT tolerance	1.000 absolute (min)
Only keep features with 13C	☐
Original feature list	KEEP
Export path	☐

Table SI-C10: Parameter specifications of the *Mass spectrometer presets* in MZmine.

	☑ Orbitrap<br>☐ TOF<br>☐ Ion mobility<br>Positive
MS1 noise level	1.0E4
MS2 noise level	3.0E3
Minimum feature intensity	5.0E4
Scan to scan m/z tolerance	0.0020 m/z or 10.0000 ppm
Feature to feature m/z tolerance	0.0010 m/z or 3.0000 ppm
Sample to sample m/z tolerance	0.0015 m/z or 5.0000 ppm

Table SI-C11: Parameter specifications of the *Import MS data* module in MZmine.

File names	30 mzML files, see Table SI-C1
Advanced import	☐
Spectral library files	none

Table SI-C12: Parameter specifications of the *Mass detection* module in MZmine.

Raw data files	Those created by previous batch step
Scans	MS level: 1
Scan types (IMS)	All scan types
Mass detector	Auto
Output netCDF filename (optional)	☐

Raw data files	Those created by previous batch step
Scans	MS level: 2
Scan types (IMS)	All scan types
Mass detector	Auto
Output netCDF filename (optional)	☐

Table SI-C13: Parameter specifications of the *ADAP Chromatogram Builder* module in MZmine.

Raw data files	Those created by previous batch step
Scans	Retention time: 5.00 - 30.00 min<br>MS level: 1
Min group size in # of scans	5
Group intensity threshold	1.0E4
Min highest intensity	5.0E4
Scan to scan accuracy (m/z)	0.0020 m/z or 10.0000 ppm
Suffix	chroms

Table SI-C14: Parameter specifications of the *Smoothing* module in MZmine.

Feature lists	Those created by previous batch step		
Smoothing algorithm	Savitzky Golay	Retention time smoothing<br>Mobility smoothing	☑ 5<br>☐
Original feature list	KEEP		
Suffix	sm		

Table SI-C15: Parameter specifications of the *Local minimum feature resolver* module in MZmine.

<table>
<tr><td>Feature lists</td><td colspan="3">Those created by previous batch step</td></tr>
<tr><td>Suffix</td><td colspan="3">r</td></tr>
<tr><td>Original feature list</td><td colspan="3">KEEP</td></tr>
<tr><td rowspan="6">MS/MS scan pairing</td><td rowspan="6">☑</td><td>Retention time tolerance</td><td>1.500</td></tr>
<tr><td>MS1 to MS2 precursor tolerance (m/z)</td><td>0.0020 m/z or 10.0000 ppm</td></tr>
<tr><td>Limit by RT edges</td><td>☑</td></tr>
<tr><td>Combine MS/MS spectra (TIMS)</td><td>☐</td></tr>
<tr><td>Lock to feature mobility range</td><td>☑</td></tr>
<tr><td>Minimum merged intensity (IMS)</td><td>☐</td></tr>
<tr><td>Dimension</td><td colspan="3">Retention time</td></tr>
<tr><td>Chromatographic threshold</td><td colspan="3">20.0%</td></tr>
<tr><td>Minimum search range RT/Mobility (absolute)</td><td colspan="3">0.500</td></tr>
<tr><td>Minimum relative height</td><td colspan="3">0.0%</td></tr>
<tr><td>Minimum absolute height</td><td colspan="3">5.0E4</td></tr>
<tr><td>Min ratio of peak top/edge</td><td colspan="3">2.00</td></tr>
<tr><td>Peak duration range (min/mobility)</td><td colspan="3">0.00 - 15.00</td></tr>
<tr><td>Min # of data points</td><td colspan="3">5</td></tr>
</table>

Table SI-C16: Parameter specifications of the *13C isotope filter (formerly: isotope grouper)* module in MZmine.

Feature lists	Those created by previous batch step
Name suffix	deiso
m/z tolerance	0.0015 m/z or 3.0000 ppm
Retention time tolerance	0.080 absolute (min)
Mobility tolerance	☐
Monotonic shape	☑
Maximum charge	2
Representative isotope	Most intense
Never remove feature with MS2	☑
Original feature list	KEEP

Table SI-C17: Parameter specifications of the *Isotopic peaks finder* module in MZmine.

Feature lists	Those created by previous batch step
Chemical elements	H, C, N, O, S, Cl, Br
m/z tolerance	0.0015 m/z or 3.0000 ppm
Maximum charge of isotope m/z	1
Search in scans	SINGLE MOST INTENSE

Table SI-C18: Parameter specifications of the *Join aligner* module in MZmine.

Feature lists	Those created by previous batch step
Feature list name	Aligned feature list
m/z tolerance	0.0015 m/z or 5.0000 ppm
Weight for m/z	3
Retention time tolerance	1.000 absolute (min)
Weight for RT	1
Mobility tolerance	☐
Mobility weight	1.000
Require same charge state	☐
Require same ID	☐
Compare isotope pattern	☐
Compare spectra similarity	☐
Original feature list	KEEP

Table SI-C19: Parameter specifications of the *Feature list rows filter* module in MZmine.

Feature lists	Those created by previous batch step		
Name suffix	peak		
Minimum features in a row (abs or%)	☑ 2.000		
Minimum features in an isotope pattern	☐		
Validate 13C isotope pattern	☑	m/z tolerance<br><br>Max charge<br>Estimate minimum carbon<br>Remove if 13C<br>Exclude isotopes	0.0010 m/z or 3.0000 ppm<br>2<br>☑<br>☑<br>O
m/z	☐		
Retention time	☐		
Features duration range	☐		
Chromatographic FWHM	☐		
Charge	☐		
Kendrick mass defect	☐		
Parameter	No parameters defined		
Only identified?	☐		
Text in identity	☐		
Text in comment	☐		
Keep or remove rows	Keep rows that match all criteria		
Feature with MS2 scan	☐		
Never remove feature with MS2	☑		
Reset the feature number ID	☐		
Mass defect	☐		
Original feature list	KEEP		

Table SI-C20: Parameter specifications of the *Peak finder (multithreaded)* module in MZmine.

Feature lists	Those created by previous batch step
Name suffix	gaps
Intensity tolerance	20.0%
m/z tolerance	0.0020 m/z or 10.0000 ppm
Retention time tolerance	0.080 absolute (min)
Minimum data points	1
Original feature list	KEEP

Table SI-C21: Parameter specifications of the *Duplicate peak filter* module in MZmine.

Feature lists	Those created by previous batch step
Name suffix	dup
Filter mode	NEW AVERAGE
m/z tolerance	0.0005 m/z or 1.5000 ppm
RT tolerance	0.350 absolute (min)
Require same identification	☐
Original feature list	KEEP

SI-C1.2.3 MS-DIAL

MS-DIAL, an abbreviation for Mass Spectrometry - Data Independent AnaLysis software, is an universal open-source program for untargeted metabolomics, with focus on data-independent acquisition (DIA) data, but can also be applied to data-dependent acquisition (DDA) data. In the following, only those parts of the algorithm are described in detail, which are relevant for centroided DDA data. The peak detection algorithm starts with a smoothing method with respect to retention time and accurate mass. The peak detection itself is based on differential calculus and noise estimations. For noise evaluation, three threshold values are estimated, namely the maximum amplitude difference between two adjacent points, as well as the maxima of the first and second derivatives, respectively. For the values below 5% of each maximum, medians of amplitude differences, first derivatives and second derivatives are calculated and serve as threshold values for peak detection. They are called amplitude filter (AF), first-order derivative filter (FF) and second-order derivative filter (SF). The peak edges are recognized when amplitude and first-order derivative both exceed AF and FF in two adjacent points, while the peak top is recognized by a sign change of the first-order derivative and when the second-order derivative is less than the SF. After peak detection, peak spotting is conducted. This term refers to the visualization method of MS-DIAL based on retention time and MS1 data axes. For each mass slice of 0.1 m/z with a default step size of 0.05 m/z, the base peak chromatogram is formed, allowing all data points to belong to two adjacent slices. Each data point of the base peak chromatogram has its scan number, retention time, base peak m/z, and base peak intensity. To these base peak chromatograms, the peak detection algorithm as described above is applied, and detected peak tops are shown as spots. Two spots with the same retention time and close m/z value in adjacent bins are merged, keeping the one with higher intensity.

The peak alignment is based on the Joint Aligner, also implemented in MZmine, consisting of four major steps. In the first step, a reference peak table is generated, where a user-defined reference file is used as basis. Information of each sample peak table is inserted to the reference peak table. In a second step, each peak in the sample data is associated with the reference peak list. Afterwards, the aligned peaks are filtered based on several criteria. For example, if peak intensities of all samples in a row are missing, the alignment information is removed, as well as if the percentage of filled peaks is less than the user-defined peak count filter. Optionally, quality control samples can be included in the filtering. In the last step, missing values are interpolated. More detailed information on MS-DIAL and its underlying algorithms can be found in the respective publication.[7]

In contrast to Compound Discoverer and MZmine, MS-DIAL does not allow a manual assembly of nodes or processing modules into a workflow. Rather, it is a given succession of algorithms, whose performance can be influenced by the parameter settings. The workflow starts with the start-up of the project and giving the analysis file paths. Then the parameter settings for data collection, peak detection, MS2 deconvolution, which is especially relevant for DIA data, identification, adducts, alignment, mobility, if ion mobility data were measured, and isotope tracking can be chosen (see Figure SI-C3). The optimized parameters are given in Tables SI-C22 to SI-C27.

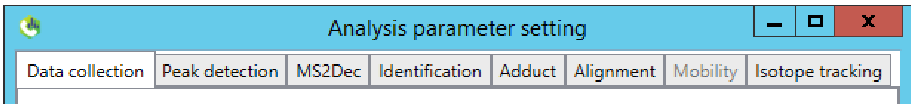

Figure SI-C3: MS-DIAL workflow

Start up a project:

Table SI-C22: Parameter specifications of the *Start up a project* step in MS-DIAL.

Ionization type	☑ Soft ionization (LC/MS, LC/MS/MS, or precursor-oriented GC/MS/MS<br>☐ Hard ionization (GC/MS)
Separation type	☑ Chromatography (GC, LC, CE, or SFC)<br>☐ Ion mobility (now coupled with liquid chromatography)
MS method type	☑ Conventional LC/MS or data dependent MS/MS<br>☐ SWATH-MS or conventional All-ions method<br>☐ All-ions with multiple CEs (cycled like 0V-10V-40V)
Data type (MS1)	☐ Profile data<br>☑ Centroid data
Data type (MS2)	☐ Profile data<br>☑ Centroid data
Ion mode	☑ Positive ion mode<br>☐ Negative ion mode
Target omics	☑ Metabolomics<br>☐ Lipidomics

Table SI-C23: Parameter specifications of the *Data collection* step in MS-DIAL.

**Mass accuracy (centroid parameter)**	
MS1 tolerance	0.002 Da
MS2 tolerance	0.0025 Da
**Data collection parameters**	
Retention time begin	5 min
Retention time end	30 min
MS1 mass range begin	100 Da
MS2 mass range end	1000 Da
MS/MS mass range begin	50 Da
MS/MS mass range end	1000 Da
**Isotope recognition**	
Maximum charged number	2
Consider Cl and Br elements	☑
**Multithreading**	
Number of threads	1
Execute retention time corrections	☐

Table SI-C24: Parameter specifications of the *Peak detection* step in MS-DIAL.

**Peak detection parameters**	
Minimum peak height	5000 amplitude
Mass slice width	0.002 Da
Smoothing method	Savitzky-Golay filter
Smoothing level	3 scans
Minimum peak width	5 scans
Exclusion mass list	

Table SI-C25: Parameter specifications of the *MS2Dec* step in MS-DIAL.

**Deconvolution parameters**	
Sigma window value	0.5
MS/MS abundance cut off	0 amplitude
Exclude after precursor ion	☑
Keep the isotopic ions until	0.5 Da
Keep the isotopic ions w/o MS2Dec	☐

Table SI-C26: Parameter specifications of the *Adduct* step in MS-DIAL.

Adduct	$[M+H]^+$, $[M+NH_4]^+$, $[M+Na]^+$, $[M+H-H_2O]^+$, $[M+H-2H_2O]^+$, $[2M+H]^+$, $[M+2H]^{2+}$

Table SI-C27: Parameter specifications of the *Alignment* step in MS-DIAL.

**Alignment parameters setting**	
Result name	alignmentResult_2022_8_2_Werdhoelzli_SSC_R1
Reference file	Werdhoelzli, mixed sample, first replicate
Retention time tolerance	1 min
MS1 tolerance	0.001 Da
Retention time factor	0.5
MS1 factor	0.5
Peak count filter	0%
N% detected in at least one group	0%
Remove features based on blank information	☐
Gap filling by compulsion	☑

SI-C1.2.4 SLAW

SLAW is a scalable workflow for processing untargeted LC-MS data. It encompasses different state-of-the-art peak picking algorithms, automated parameter optimization, sample alignment, gap filling and extraction of MS2 data and isotopic patterns across all samples. Since the outcome of untargeted data processing is sensitive to a multitude of parameters, SLAW starts its workflow with a parameter optimization, applied to a subset of indicated quality control samples or a subset of randomly selected samples. For this purpose, a region of the acquisition mass range containing the most intense features and their isotopes is selected, to accelerate optimization. The best parameter combination is identified by a method derived from the response surface model. The peak picking itself is performed by one of the three implemented peak picking algorithms: FeatureFinderMetabo from openMS,[9] centWave from XCMS[8] or ADAP from MZmine.[15] The parameter optimization operates with all algorithms. The following retention time alignment implemented in SLAW detects a set of 150 reproducible reference peaks that are used to determine the alignment parameters. With locally optimized RANSAC,[16] the retention times of all sample peak tables are corrected. These time-corrected sample peak tables are then aligned to a master peak table by density-based clustering. After alignment, gap filling is performed by data recursion. Afterwards, isotopic patterns are extracted from the sample, using recursion with the raw data. To perform adducts and fragment annotation, the strategy implemented in CliqueMS[17] is adopted, which extracts ion chromatograms for all the detected peaks, builds a similarity network based on cosine similarity, and searches for cliques to identify related metabolites. The resulting sets of cliques are annotated by the InterpretMSSpectrum package.[18] Based on the precursor m/z and retention time, all collected MS2 spectra are finally assigned to the feature table. By an implementation of ClustMS,[19] representative MS2 spectra with the highest precursor intensity are extracted. The final results are represented in tables and text files, allowing further processing.

In contrast to all previously described tools, SLAW is command-line based. Since it encompasses tools coded in different languages, everything is assembled in a container and is available on DockerHub and on SingularityHub. More detailed information on SLAW can be found in the respective publication.[1]

Similar to MS-DIAL, the workflow is based on a series of algorithms and cannot be assembled manually, as for Compound Discoverer or MZmine. The general workflow is depicted in Figure SI-C4. The parameters resulting from the automated method optimization are given in Tables SI-C28 to SI-C33.

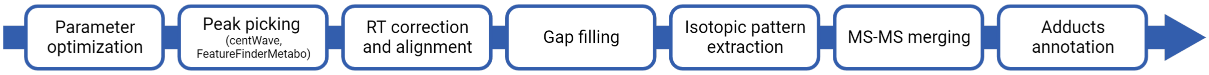

Figure SI-C4: General workflow of SLAW, adapted from.[1]

Table SI-C28: Parameter specifications of the *Filtering* step in SLAW.

**Filtering**	
fold_blank	priority: HIGH<br>value: 3
frac_qc	priority: HIGH<br>value: 0

Table SI-C29: Parameter specifications of the *Grouping* step in SLAW.

**Grouping**	
alpha	priority: LOW<br>value: 0.1
dmz	priority: LOW<br>value: 0.01
drt	priority: HIGH<br>value: 0.5
extracted_quantity	priority: HIGH<br>value: intensity
num_references	priority: LOW<br>value: 150
ppm	priority: HIGH<br>value: 15

Table SI-C30: Parameter specifications of the *Ion annotation* step in SLAW.

**Ion annotation**	
adducts_negative	priority: HIGH<br>value: NONE
adducts_positive	priority: HIGH<br>value: '[M+H]+', '[M+2H]2+', '[M+Na]+', '[M+K]+', '[M+NH4]+', '[M+2Na-H]+', '[2M+H]+', '[2M+2H]2+', '[2M+H+Na]2+', '[2M+Na]+', '[2M+2Na-H]+', '[M+2H-NH3]2+', '[M+H-H2O]+', '[M+2H-H2O]2+', '[M+3H]3+', '[M+CH3COONa+H]+', [M+CH3COONa+Na]+', '[M+CH3COONa+NH4]+'
dmz	priority: HIGH<br>value: 0.01
main_adducts_negative	priority: HIGH<br>value: NONE
main_adducts_positive	priority: HIGH<br>value: '[M+H]+', '[M+2H]2+', '[M+Na]+', '[M+NH4]+'
max_charge	priority: LOW<br>value: 3
max_isotopes	priority: LOW<br>value: 4
min_filter	priority: HIGH<br>value: 2
num_files	priority: HIGH<br>value: 50
polarity	priority: ESSENTIAL<br>value: positive
ppm	priority: HIGH<br>value: 15

Table SI-C31: Parameter specifications of the *Optimization* step in SLAW. Values separated by an arrow indicate initial and optimized parameters, respectively.

**Optimization**	
files_used	priority: LOW<br>value: 3
initial_estimation	priority: LOW<br>value: false
need_optimization	value: true $\rightarrow$ false
noise_threshold	priority: LOW<br>value: 500
num_iterations	priority: LOW<br>value: 5
number_of_points	priority: LOW<br>value: 30

Table SI-C32: Parameter specifications of the *Output format* in SLAW.

**Output format**	
ms1	priority: LOW<br>value: gap-filled data matrix
ms2	priority: LOW<br>value: fused mgf

Table SI-C33: Parameter specifications of the *Peak picking* step in SLAW. Parameter specifications of SLAW. Values separated by an arrow indicate initial and optimized parameters, respectively.

**Peak picking**		
algorithm	value: CENTWAVE	
noise_level_ms1	priority: ESSENTIAL<br>value: 0	
noise_level_ms2	priority: ESSENTIAL<br>value: 0	
peaks_deconvolution	SN	priority: HIGH<br>value: 3 → 6.0
	coefficient_area_ threshold	priority: LOW<br>value: 50
	ms2_mz_tol	priority: LOW<br>value: 0.1
	ms2_rt_tol	priority: LOW<br>value: 0.1
	noise_level	priority: HIGH<br>value: 0
	peak_width	priority: ESSENTIAL<br>value: 0.02-0.1 →<br>0.1800-0.3349
	peak_width_fac	priority: HIGH<br>value: 0.2 → 0.0640
	rt_wavelet	priority: ESSENTIAL<br>value: 0.0005-0.005 →<br>0.0005-0.0055
peaktable_filter	priority: LOW<br>value: absolute_intensity top 30000	
traces_construction	dmz	priority: ESSENTIAL<br>value: 0.007 → 0.0022
	min_scan	priority: ESSENTIAL<br>value: 15 → 7.7216
	num_outliers	priority: LOW<br>value: 5
	ppm	priority: ESSENTIAL<br>value: 15 → 5.171

SI-C1.3 R Analysis

After processing the chosen subdataset with the different peak picking tools, the resulting peak tables are exported as csv or txt files, which are read by R.[12] These peak tables are compared to the list of 50 isotopically labeled internal standards (ILISs), whose mass to charge ratios and retention times (RTs) are known from the previous target screening. If a picked feature lies within the given m/z range of ±3 ppm and the RT range of ±0.5 min, it is considered as true positive for each sample the feature was found in. This means that in total 1350 true positives can be achieved, which is given by the 50 ILISs multiplied by the 27 analyzed samples. To ensure that the peak picking is of sufficient quality, a filtering based on the intensity is incorporated. Since all samples were analyzed in triplicates, one can assume that the intensity of a single ILIS is correct, if at least one of the other two replicates shows a similar intensity. As cutoff, a deviation of less than 20% was chosen. In case one of the three replicates deviates more than the given cutoff value, it is discarded and the number of true positives reduced by one, while the number of false negatives is increased by one. If all three replicates differ from each other, they are all considered as false negatives. If no feature for an ILIS within the given m/z and RT limits was found, the number of false negatives increases by 27. In case multiple hits for one ILIS were found, the one with the highest median intensity was considered as the true positive, while the other features were interpreted as false positives. The number of false positives was increased by the number of samples this additional feature was found in. By summing up the true positives, false positives and false negatives and applying Equations 1 to 3, one obtains precision, recall and F_1 score.

SI-C1.4 Results and Discussion

The resulting precision, recall and F_1 scores of the five different peak picking algorithms, after optimization of the input parameters, are given in Table SI-C34 and Figure SI-C5. It becomes visible that MZmine has the highest achievable precision of 100%. Of all tools, Compound Discoverer exhibits the highest recall rate of 88.2%. When combining precision and recall in the F_1 score, SLAW run with centWave achieves the highest value of 90.9%, followed by 88.8% by SLAW run with FFM and Compound Discoverer with 85.3%. Regarding the processing time of the different peak picking tools, MZmine and SLAW with run times below four hours are best, followed by Compound Discoverer with a processing time of less than 24 h, while the execution of the final optimized MS-DIAL workflow took more than 24 h. Considering these aspects in combination with the other functionalities available in the tools, like compound annotation by molecular formula prediction, suspect list search, MS2 library search, neutral loss and characteristic fragment search, molecular networking, as well as the user friendliness, Compound Discoverer was selected as the tool for further data processing.

Table SI-C34: Precision, recall and F_1 scores of the five different peak picking algorithms. The different tools are ordered along decreasing F_1 scores.

	**Precision**	**Recall**	**F_1 score**
SLAW (centWave)	0.962	0.861	0.909
SLAW (FFM)	0.961	0.826	0.888
Compound Discoverer	0.825	0.882	0.853
MZmine	1.000	0.657	0.793
MS-DIAL	0.768	0.673	0.717

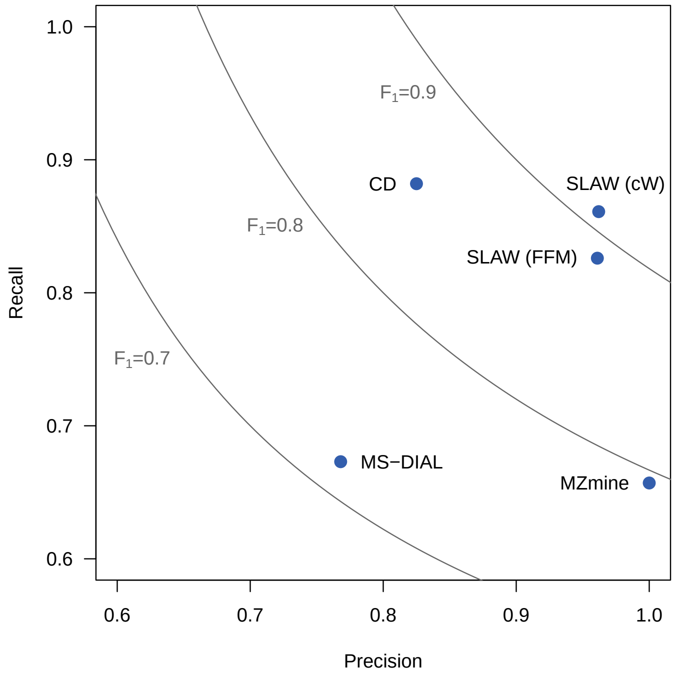

Figure SI-C5: Scatterplot of precision and recall of the analyzed tools. The F_1 isocost lines are given in gray.

SI-C2 Materials & Methods

SI-C2.1 Wastewater Sample Collection

Wastewater influent samples originated from three Swiss wastewater treatment plants, including Altenrhein, Neugut (Duebendorf) and Werdhoelzli (Zurich). They have population equivalents of 83,000, 105,000 and 670,000 and industry contributions of 23%, 52% and 30%, respectively. Untreated wastewater samples were collected from Monday, February 28 to Friday, March 4, 2022, as 24 hours composite samples before the primary clarifier. For Neugut and Werdhoelzli, the time period ranged from midnight to midnight, while in Altenrhein the sampling time spanned from 7 am to 7 am. Sampling devices from MAXX were pre-installed from the operators in Altenrhein and Werdhoelzli, allowing flow-proportional sampling. In Neugut, a MAXX autosampler TP5 C Active was positioned for time-proportional sampling of the influent. Collected samples were stored at 4 °C within the sampler. The samples were transferred maximally 72 hours after finished collection into borosilicate glass bottles (previously annealed at 500 °C; 1 L bottles, Duran, Germany or SIMAX Kavalier, Czech Republic) and transported to the laboratory. Samples were stored at 4 °C for maximally two hours until sample preparation.

SI-C2.2 Wastewater Sample Preparation

Triplicates of 50 mL wastewater were transferred into 50 mL centrifuge tubes (Corning, U.S.), followed by addition of 20 µL of 1 mg/L isotope labeled internal standard solution. Centrifugation was conducted at 3000 rcf and 4 °C for 10 minutes. The supernatant was transferred to 250 mL borosilicate bottles (previously annealed at 500 °C; Duran, Germany or SIMAX Kavalier, Czech Republic) and 120 mL of ultrapure water were added. The prepared samples were stored at 4 °C for maximally 10 days until analysis. After analysis, samples were stored at −20 °C and later used for spiking experiments with purchased reference standards. Figure SI-C6 displays the sample preparation. The same preparation was applied for the quality control samples. For details on quality control see Section SI-C2.5

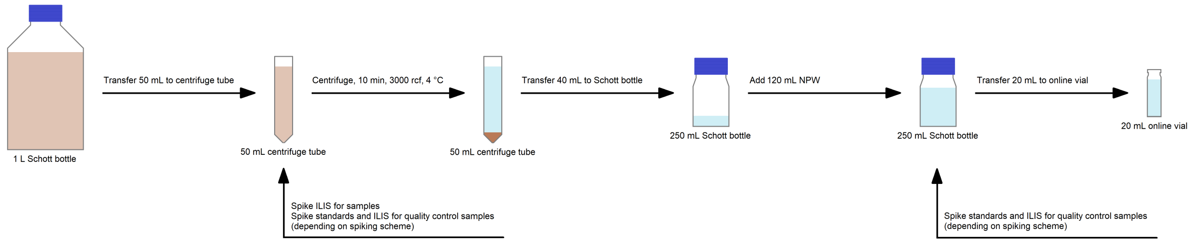

Figure SI-C6: Wastewater sample preparation scheme.

SI-C2.3 Human Liver S9 Incubation

The method for human liver S9 incubation was adapted from previous studies in phase I metabolite generation.[20,21] The experiment was conducted in a 96-well plate, containing three replicates of the incubation of each pharmaceutical compound, one standard control without human liver S9, six negative control replicates (human liver S9, coenzyme NADPH, without pharmaceutical) and three positive control replicates with 7-ethoxycoumarin. A 1 mg/mL standard solution of each pharmaceutical compound in organic solvent (EtOH, MeOH or ACN) was prepared. Into each well representing an incubation replicate or a standard control, 2.1 µL of standard solution were added into 200 µL of 0.05 M TRIS buffer (pH 7.4). Afterwards, 20 µL of pooled human

liver S9 (Thermo Scientific, U.S., containing six pooled human liver S9 fractions of mixed gender at a concentration of 20 mg/ml), were added to the wells. The well plate was placed in an incubator (Labwit Scientific, ZWYR-D2401, Australia) and incubated for 5 min, before 15 µL of 20 mM reduced nicotinamide adenine dinucleotide phosphate (NADPH) solution was added. The incubation was conducted for 3 h at 37.5 °C under constant shaking and stopped by the addition of −20 °C cold methanol (1:1 incubation volume). The content of each well was transferred to 1.5 mL glass vials for storage at −20 °C until analysis. For HPLC-HRMS/MS analysis, 40 µL were transferred to a 20 mL headspace amber glass vials and diluted with 20 mL ultrapure water. The settings were the same as for the analysis of wastewater samples.

SI-C2.4 Online-SPE-HPLC-HRMS/MS

To prepare the online solid phase extraction (SPE) column, a stainless steel SPE cartridge (20 x 2.1 mm, BGB Analytik AG, Switzerland) was manually filled in flow direction with a mixture of 9 mg Oasis HLB (∅ = 30 µm, Waters, U.S.) and a mix of 9 mg ion exchangers, including Strata X-AW (∅ = 33 µm), Strata X-CW (∅ = 25 µm, Phenomenex, U.S.) and Isolute ENV+ (∅ = 90 µm, Biotage, Wales), in the ration 1:1:1.5. The time schedule of the online-SPE is given in Table SI-C35. The gradient of the loading pump comprises an ammonium acetate solution (2 mM in ultrapure water) and acetonitrile. The elution of the sample was performed with a Dionex UltiMate3000 RS pump (Thermo Fisher Scientific, U.S.), while the sample loading was conducted with the dispenser syringe. Acetonitrile was used for flushing the sample loop and the SPE cartridge after every sample. Chromatographic separation was achieved with a reversed-phase C18 column at 30 °C (Atlantis® T3, 3 µm, 3.0 x 150 mm, Waters, U.S.). In the positive ionization mode, ultrapure water and methanol, both with 0.1% formic acid were used as mobile phases A and B, respectively. For the negative ionization mode, mobile phase A was composed of 90%/10% water/methanol and mobile phase B of 10%/90% water/methanol, both with 5 mM ammonium formate. Table SI-C36 summarizes the applied LC gradient, while Table SI-C37 contains the mass spectrometer (Q Exactive, Thermo Fisher Scientific) settings. To maximize the number of MS2 spectra, the suspect list was used as inclusion list. A normalized collision energy (NCE) for each compound was calculated based on the following formula and rounded to the nearest number divisible by five lying between 15 and 120:

$$NCE = \begin{cases} \mathrm{m/z} \cdot (-0.41) + 160 & \mathrm{m/z} < 350 \\ 15 & \mathrm{m/z} > 350 \end{cases}, \tag{4}$$

where m/z is the mass to charge ratio of the $[M{+}H]^+$ or the $[M{-}H]^-$ adduct, respectively.

Table SI-C35: Time schedule of the online-SPE.

**Time** [min]	**Ammonium acetate solution** [µL/min]	**Acetonitrile** [µL/min]	**SPE step**
0.0	200		
0.1		4000	
1.1		4000	Elution of the sample
1.2	4000		from the cartridge
6.7	4000		and washing of the loop
6.8	400		
7.3	400		
7.4		400	
12.5		400	Loading of the new sample
12.6	400		into the loop and conditioning
18.4	400		of the cartridge
18.5	1270		
34.5	1270		
34.7	200		Enrichment of the new sample
35.0	200		

Table SI-C36: Time schedule of the liquid chromatography.

**Time** [min]	**Eluent A** [µL/min]	**Eluent B** [µL/min]
0.0	260	40
5.0	260	40
20.0	15	285
29.0	15	285
29.5	260	40
35.0	260	40

Table SI-C37: ESI-HRMS/MS settings.

Ionization source	Spray voltage (positive/negative mode)	+4/-3
	Sheath gas flow rate (nitrogen)	40 L/min
	Auxiliary gas flow rate (nitrogen)	15 L/min
	Transfer capillary temperature	320°C
	Auxiliary gas heater temperature	50°C
MS1	Mass resolution	140,000
	Scan range (m/z)	100 - 1,000
	AGC target	100,000
	Maximal injection time	100 ms
MS2	Mass resolution	17,500
	AGC target	10,000
	Maximal injection time	50 ms

SI-C2.5 Quality Control

For quality control, absolute and relative recoveries, matrix factors and limits of quantification (LOQ) were calculated for each target compound. As control samples, volume proportional mix samples of the five 24 h influent composite samples for each WWTP were taken. Four different spiking schemes of standards and internal standards were applied in triplicates each and are listed below.

- Scheme A: Spiked standards and ILIS after sample preparation
- Scheme B: Spiked standards and ILIS before sample preparation
- Scheme C: Spiked only ILIS before sample preparation
- Scheme D: Spiked only ILIS before sample preparation into ultrapure water

The relative recovery can be calculated based on spiking schemes B and C:

$$\text{relative recovery [\%]} = \frac{c_{\text{scheme B}} - c_{\text{scheme C}}}{\text{theoretically spiked amount}} \cdot 100 \quad , \tag{5}$$

where c corresponds to the concentration of the respective compound. In all cases, the theoretically spiked amount corresponds to 100 ng/L. It can be used to evaluate how well the internal standards can correct matrix effects. For the calculation of the absolute recovery, the same schemes can be used, but the peak area A instead of the concentration is required and instead of the theoretically spiked amount, the average area $\overline{A}$ of the corresponding calibration standard is inserted:

$$\text{absolute recovery [\%]} = \frac{A_{\text{scheme B}} - A_{\text{scheme C}}}{\bar{A}_{\text{calibration standard}}} \cdot 100 \quad . \tag{6}$$

The resulting value includes all effects, such as matrix effects, instrument contamination and variability and can be used to evaluate the overall performance of the method. For the matrix factor calculation, two different cases have to be differentiated. If the compound of interest has an own ILIS, the following equation has to be applied:

$$\text{matrix factor}_{\text{own ILIS}} = \frac{A(\text{ILIS})_{\text{scheme A}}}{\bar{A}(\text{ILIS})_{\text{calibration series}}} \quad , \tag{7}$$

where $\overline{A}$ corresponds to the average area of the respective ILIS in all calibration solutions from 0.1 ng/L up to 2500 ng/L. If a target compound has no own internal standard, the calculation of the matrix factor is based on the following equation:

$$\text{matrix factor}_{\text{no own ILIS}} = \frac{A_{\text{scheme A}} - A_{\text{scheme C}}}{\bar{A}_{\text{calibration standard}}} \quad , \tag{8}$$

whereat the area refers to the corresponding standard. The matrix factor can be used to draw conclusions about matrix effects, such as ion suppression or enhancement. Furthermore, the calculation of the matrix limit of quantification (LOQ), which is the lowest analyte concentration that can be detected quantitatively in a sample with the given matrix, is based on the LOQ of nanopure water (NPW). It corresponds to the lowest concentration in the calibration series, where a peak with a signal to noise ratio larger than 1:10 and a reasonable peak shape is observed. Furthermore, the matrix factor is required for the calculation:

$$\text{matrix LOQ} = \frac{\text{LOQ}_{\text{NPW}}}{\text{matrix factor}} \quad . \tag{9}$$

The obtained relative and absolute recoveries, matrix factors and LOQs for the three different WWTPs are listed in SI-B2 and shown in Figures SI-C7 to SI-C10. Average relative recoveries for compounds with own ILIS range from 95% to 103% and show smaller variance than the compounds without own ILIS. Although over 80 ILIS were used for the analysis, they are not sufficient to correct the matrix effects of all targeted compounds. This is especially true for early and late eluting targets, where only few ILIS are available. Concentrations of compounds without own ILIS were correspondingly corrected by the chosen ILIS and relative recoveries. Absolute recoveries show matrix effects before correction by ILIS and are correspondingly more pronounced. Considering the high matrix load of wastewater influent and that online-SPE enriches not only the analytes but also part of the matrix, ion suppression is expected for most compounds. On the contrary, a few compounds show strong ion enhancement due to the matrix enrichment, such that absolute recoveries of up to 400% are reached. Similar trends as for the absolute recoveries are also observed for the matrix factors. Despite stronger matrix effects stemming from matrix enrichment from the online-SPE, LOQs below 0.5 ng/L are achieved for most targeted compounds, meaning that the applied method is sensitive and well suited for the detection of the often low concentrated human metabolites in untreated wastewater.

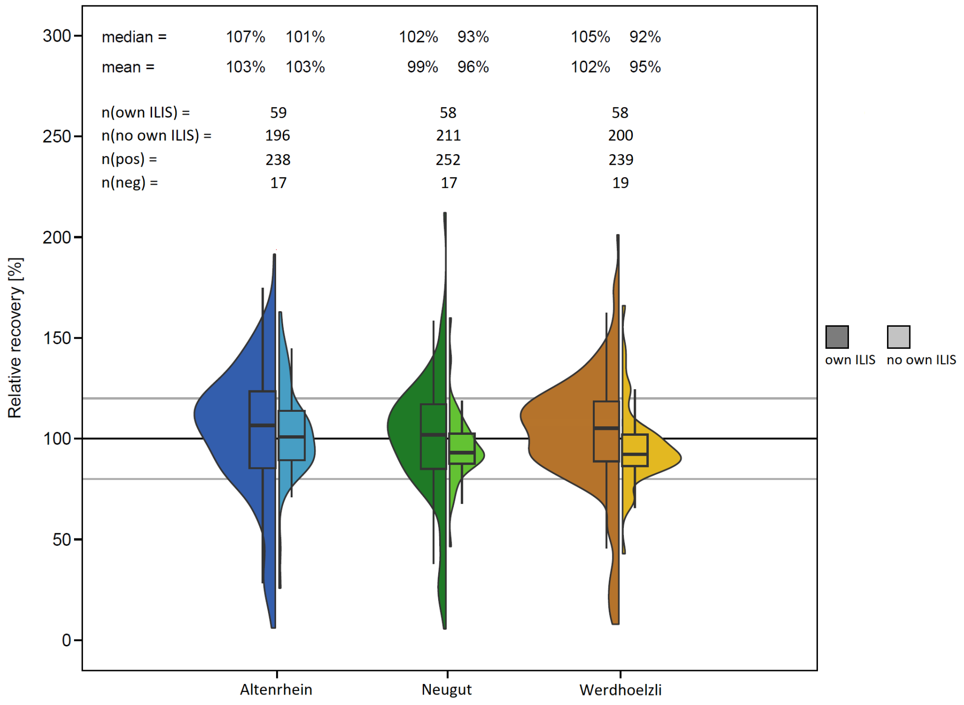

Figure SI-C7: Relative recoveries in the influents of the three WWTPs Altenrhein, Neugut and Werdhoelzli. Compounds with and without own ILIS are depicted separately.

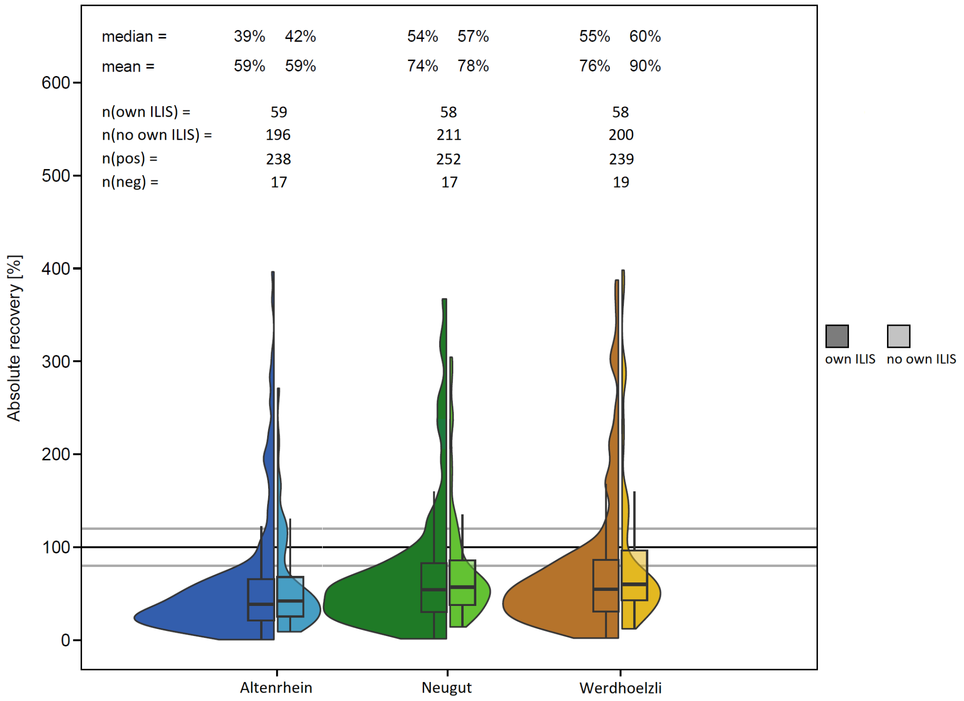

Figure SI-C8: Absolute recoveries in the influents of the three WWTPs Altenrhein, Neugut and Werdhoelzli. Compounds with and without own ILIS are depicted separately.

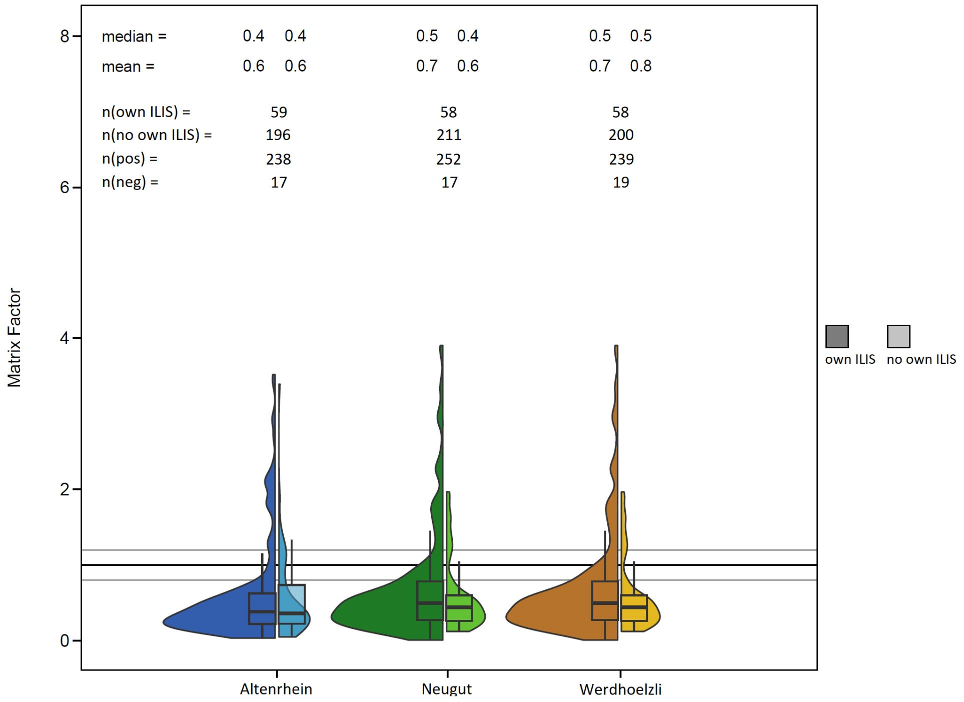

Figure SI-C9: Matrix factors in the influents of the three WWTPs Altenrhein, Neugut and Werdhoelzli. Compounds with and without own ILIS are depicted separately.

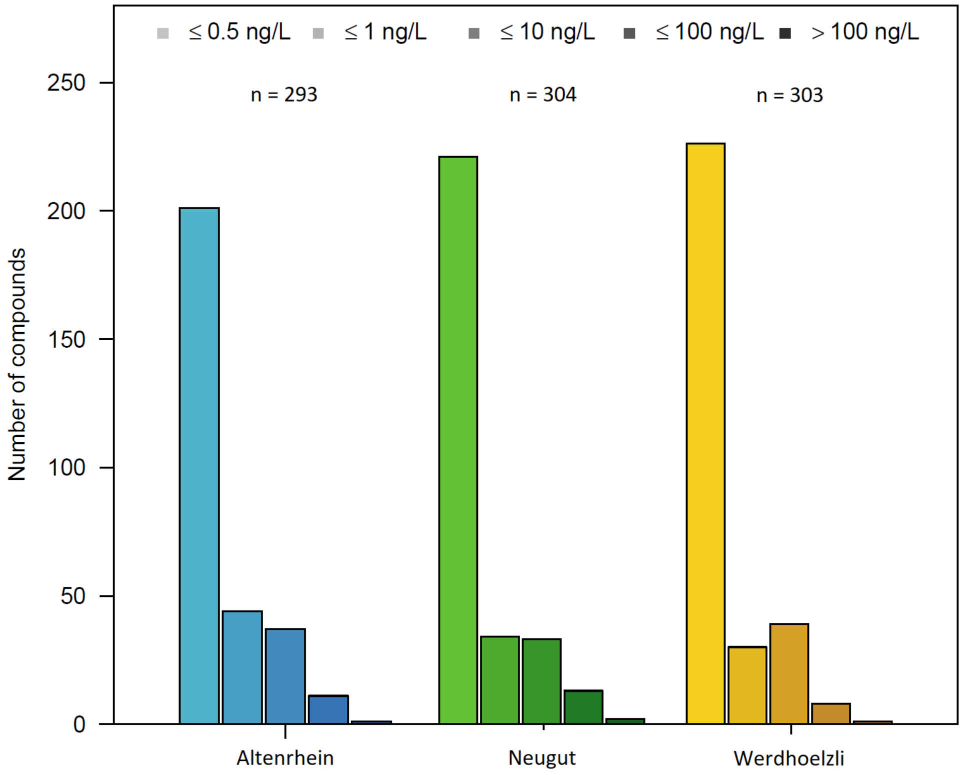

Figure SI-C10: Matrix LOQs in the influents of the three WWTPs Altenrhein, Neugut and Werdhoelzli.

SI-C2.6 Processing with Compound Discoverer

Unknown data processing and initial data interpretation was performed with Compound Discoverer (Thermo Fisher Scientific, U.S.)[5] for positive and negative mode separately. The backbone of the workflow is based on spectra selection, retention time alignment, compound detection and grouping and gap filling. Annotation is on one hand built on library match with mzCloud[22] and the mzVault node, including MassBank[23] and NIST[24] spectral libraries. On the other hand, the suspect list of pharmaceuticals and their human metabolites was used for assignment, as well as the predict compositions node. Molecular networking was included to gain additional confidence in metabolite identification based on spectral similarity of parent and metabolite. For the non-targeted approach, the neutral losses search and the compound class scoring nodes were included. They enable the search for specific neutral losses, corresponding for example to sulfate or glucuronide conjugates or the identification of fragments characteristic for glucuronide or glutathione conjugates. The applied workflow is depicted in Figure SI-C11 and the parameter settings for each workflow node can be found in Tables SI-C38 to SI-C56.

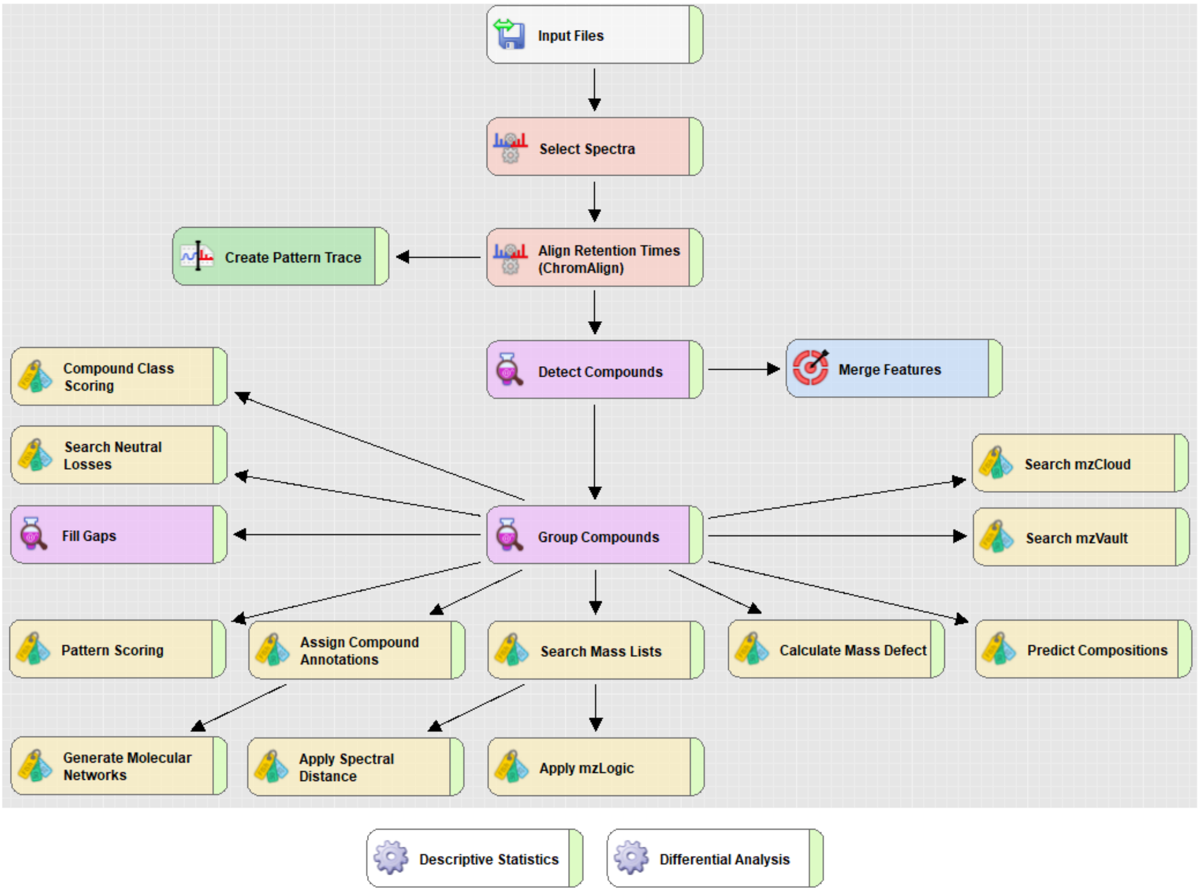

Figure SI-C11: Compound Discoverer workflow. The node *Compound Class Scoring* was only applied in negative ionization mode.

Table SI-C38: Parameter specifications of the *Select Spectra* node in Compound Discoverer.

**1. Spectrum Properties Filter**	
Lower RT Limit	0
Upper RT Limit	0
First Scan	0
Last Scan	0
Ignore Specified Scans	
Lowest Charge State	0
Highest Charge State	0
Min. Precursor Mass	100 Da
Max. Precursor Mass	1000 Da
Total Intensity Threshold	0
Minimum Peak Count	1
**2. Scan Event Filters**	
Mass Analyzer	Any
MS Order	Any
Activation Type	Any
Min. Collision Energy	0
Max. Collision Energy	1000
Scan Type	Any
Polarity Mode	Any
MS1 Mass Range	
FAIMS CV	
**3. Peak Filters**	
S/N Threshold (FT-only)	1.5
**4. Replacements for Unrecognized Properties**	
Unrecognized Charge Replacements	1
Unrecognized Mass Analyzer Replacements	FTMS
Unrecognized MS Order Replacements	MS2
Unrecognized Activation Type Replacements	CID
Unrecognized Polarity Replacements	+/-
Unrecognized MS Resolution@200 Replacements	140000
Unrecognized MSn Resolution@200 Replacements	17500
**5. General Settings**	
Precursor Selection	Use MS(n-1) Precursor
Use Isotope Pattern in Precursor Reevaluation	True
Provide Profile Spectra	Automatic
Store Chromatograms	False

Table SI-C39: Parameter specifications of the *Align Retention Times (ChromAlign)* node in Compound Discoverer.

**1. General Settings**	
Reference File	Neugut, Wednesday, influent, second replicate

Table SI-C40: Parameter specifications of the *Create Pattern Trace* node in Compound Discoverer.

**1. General Settings**	
Isotope Ratios	Cl Br
Mass Tolerance	5 ppm
Intensity Tolerance	30
MS Order	MS1
Polarity	+/-
Custom Label	1Cl Trace

Table SI-C41: Parameter specifications of the *Detect Compounds* node in Compound Discoverer.

**1. General Settings**	
Mass Tolerance	5 ppm
Min. Peak Intensity	5000
Min. # Scans per Peak	5
Use Most Intense Isotope Only	True
**2. Trace Detection**	
Max. Number of Gaps to Correct	2
Min. Number of Adjacent Non-Zeros	2
**3. Peak Detection**	
Chromatographic S/N Threshold	1.5
Remove Baseline	FALSE
Gap Ratio Threshold	0.35
Max. Peak Width [min]	1
Min. Relative Valley Depth	0.1
**4. Isotope Pattern Detection**	
Group Isotopes for	Br; Cl
Use Peak Quality for Isotope Grouping	True
Filter out Features with Bad Peaks Only	True
Zig-Zag Index Threshold	0.2
Jaggedness Threshold	0.4
Modality Threshold	0.9
Remove Potentially False Positive Isotopes	True
**5. Compound Detection**	
Ions	$[2M+H]^{+1}$; $[M+2H]^{+2}$; $[M+H]^{+1}$; $[M+Na]^{+1}$; $[M+NH_4]^{+1}$
	$[2M-H]^{-1}$; $[M-2H]^{-2}$; $[M-H]^{-1}$; $[M-H-H_2O]^{-1}$
Base Ions	$[M+H]^{+1}$ / $[M-H]^{-1}$
Remove Singlets	True
**6. AcquireX Settings**	
Detect Persistent Background Ions	False

Table SI-C42: Parameter specifications of the *Merge Features* node in Compound Discoverer.

**1. Peak Consolidation**	
Mass Tolerance	5 ppm
RT Tolerance [min]	1

Table SI-C43: Parameter specifications of the *Group Compounds* node in Compound Discoverer.

**1. General Settings**	
Mass Tolerance	5 ppm
RT Tolerance [min]	1
Align Peaks	False
Preferred Ions	$[M+H]^{+1}$ / $[M\text{-}H]^{-1}$
Area Integration	Most Common Ion
**2. Peak Rating Contributions**	
Area Contribution	3
CV Contribution	10
FWHM to Base Contribution	5
Jaggedness Contribution	5
Modality Contribution	5
Zig-Zag Index Contribution	5
**3. Peak Rating Filter**	
Peak Rating Threshold	0
Number of Files	0

Table SI-C44: Parameter specifications of the *Fill Gaps* node in Compound Discoverer.

**1. General Settings**	
Mass Tolerance	5 ppm
S/N Threshold	1.5
Use Real Peak Detection	True

Table SI-C45: Parameter specifications of the *Search mzCloud* node in Compound Discoverer.

**1. General Settings**	
Compound Classes	All
Precursor Mass Tolerance	10 ppm
FT Fragment Mass Tolerance	10 ppm
IT Fragment Mass Tolerance	0.4 Da
Library	Autoprocessed; Reference
Post Processing	Recalibrated
Max. # Results	10
Annotate Matching Fragments	True
Search MSn Tree	False
**2. DDA Search**	
Identity Search	HighChem HighRes
Match Activation Type	True
Match Activation Energy	Match with Tolerance
Activation Energy Tolerance	50
Apply Intensity Threshold	True
Similarity Search	Confidence Forward
Match Factor Threshold	50
**3. DIA Search**	
Use DIA Scans for Search	False
Max. Isolation Width [Da]	500
Match Activation Type	False
Match Activation Energy	Any
Activation Energy Tolerance	100
Apply Intensity Threshold	True
Match Factor Threshold	20

Table SI-C46: Parameter specifications of the *Search mzVault* node in Compound Discoverer.

**1. Search Settings**	
mzVault Library	MassBank, NIST
Compound Classes	All
Match Ion Activation Type	True
Match Ion Activation Energy	Match with Tolerance
Ion Activation Energy Tolerance	50
Match Ionization Method	True
Apply Intensity Treshhold	True
Remove Precursor Ion	True
Precursor Mass Tolerance	10 ppm
FT Fragment Mass Tolerance	10 ppm
IT Fragment Mass Tolerance	0.4 Da
Match Analyzer Type	False
Search Algorithm	HighChem HighRes
Match Factor Threshold	50
Max. # Results	10
RT Tolerance [min]	2
Use Retention Time	False

Table SI-C47: Parameter specifications of the *Predict Compositions* node in Compound Discoverer.

**1. Prediction Settings**	
Mass Tolerance	5 ppm
Min. Element Counts	C H
Max. Element Counts	C90 H190 Br3 Cl4 F6 N10 O18 P3 S5
Min. RDBE	0
Max. RDBE	40
Min. H/C	0.1
Max. H/C	3.5
Max. # Candidates	10
Max. # Internal Candidates	500
**2. Patterns Matching**	
Intensity Tolerance [%]	30
Intensity Threshold [%]	0.1
S/N Threshold	3
Min. Spectral Fit [%]	30
Min. Pattern Cov. [%]	50
Use Dynamic Recalibration	True
**3. Fragment Matching**	
Use Fragment Matching	True
Mass Tolerance	5 ppm
S/N threshold	3

Table SI-C48: Parameter specifications of the *Search Mass Lists* node in Compound Discoverer.

**1. Search Settings**	
Mass Lists	Suspect list of pharmaceuticals and their human metabolites
Use Retention Time	False
RT Tolerance [min]	1
Mass Tolerance	5 ppm

Table SI-C49: Parameter specifications of the *Apply Spectral Distance* node in Compound Discoverer.

**1. Pattern Matching**	
Mass Tolerance	5 ppm
Intensity Tolerance [%]	30
Intensity Threshold [%]	0.1
S/N Threshold	3
Use Dynamic Recalibration	True

Table SI-C50: Parameter specifications of the *Apply mzLogic* node in Compound Discoverer.

**1. Search Settings**	
FT Fragment Mass Tolerance	10 ppm
IF Fragment Mass Tolerance	0.4 Da
Max. # Compounds	0
Max. # mzCloud Similarity Results	10
Match Factor Threshold	30

Table SI-C51: Parameter specifications of the *Calculate Mass Defect* node in Compound Discoverer.

**1. Mass Defect**	
Fractional Mass	True
Standard Mass Defect	True
Relative Mass Defect	True
Kendrick Mass Defect	True
Nominal Mass Rounding	Round
**2. Kendrick Formula**	
Formula 1	C2 H4 O
Formula 2	C3 H6 O2
Formula 3	C H2
Formula 4	C2 F4
Formula 5	C2 F3 O

Table SI-C52: Parameter specifications of the *Assign Compound Annotation* node in Compound Discoverer.

**1. General Settings**	
Mass Tolerance	5 ppm
**2. Data Sources**	
Data Source #1	MassList Search
Data Source #2	mzCloud Search
Data Source #3	mzVault Search
Data Source #4	Predict Compositions
Data Source #5	
Data Source #6	
Data Source #7	
**3. Scoring Rules**	
Use mzLogic	True
Use Spectral Distance	True
SFit Threshold	20
SFit Range	20
**4. Reprocessing**	
Clear Names	False

Table SI-C53: Parameter specifications of the *Generate Molecular Networks* node in Compound Discoverer.

**1. Spectral Similarity**	
Use Full MSn Tree	True
Match Mass Shift	True
Match Transformations	True
Variate Transformations	False
S/N Threshold	3
Mass Tolerance	5 ppm
Min. Fragment m/z	50
**2. Transformations**	
Phase I	
Phase II	
Others	
Max. # Phase II	2
Max. # All Steps	5
**3. Applied View Filters**	
Require Transformation	False
Require MSn	True
Min. MSn Score	50
Min. MSn Coverage	50
Min. Fragments	2
**4. Applied Threshold**	
Require Transformation	False
Require MSn	False
Min. MSn Score	20
Min. MSn Coverage	20
Min. Fragments	0

Table SI-C54: Parameter specifications of the *Pattern Scoring* node in Compound Discoverer.

**1. General Settings**	
Isotope Patterns	Cl; Br
Mass Tolerance	5 ppm
Intensity Tolerance [%]	30
SN threshold	3
Min. Spectral Fit [%]	15
Preferred Ions	$[M+H]^{+1}$ / $[M-H]^{-1}$

Table SI-C55: Parameter specifications of the *Search Neutral Losses* node in Compound Discoverer.

**1. General Settings**	
Neutral Losses	Glucuronic acid (C6 H10 O7, 194.04);<br>Glucuronide (C6 H8 O6, 176.03);<br>Sulfate (O3 S, 79.96)
High Acc. Mass Tolerance	5 ppm
Low Acc. Mass Tolerance	0.5 Da
S/N Threshold	3
Use DIA Scans for Search	False

Table SI-C56: Parameter specifications of the *Compound Class Scoring* node in Compound Discoverer. This node was only applied in the negative ionization mode.

**1. General Settings**	
Compound Classes	Glutathione, glucuronide
S/N Threshold	50
High Acc. Mass Tolerance	5 ppm
Low Acc. Mass Tolerance	0.5 Da
Use Full MS Tree	True
Allow DIA Scoring	True

SI-C2.7 Export of MS2 Information

The MS2 spectra of the remaining components were exported as a mzVault library from Compound Discoverer. This library was then opened with mzVault (Thermo Fisher Scientific, U.S.)[25] and exported in the msp file format, which serves as input for further data analysis tools.

SI-C2.8 *In Silico* and Machine Learning Tools

Three different *in silico* and machine learning tools were combined to increase the confidence in component annotation based on the suspect list. These tools encompass SIRIUS/CSI:FingerID,[13,26,27] MetFrag[28] and FISh Scoring used within Compound Discoverer. The following subsections specify the application and the parameter settings.

SI-C2.8.1 SIRIUS/CSI:FingerID

SIRIUS[13] in combination with CSI:FingerID[26,27] was applied with the graphical user interface. The msp file generated from Compound Discoverer and mzVault was used as input. The processing parameters were set as specified in Table SI-C57.

Table SI-C57: Parameter settings of SIRIUS/CSI:FingerID.

**SIRIUS**	
Instrument	Orbitrap
Filter by isotope pattern	☑
MS2 mass accuracy (ppm)	5
MS/MS isotope scorer	IGNORE
Candidates stored	10
Min candidates per ion stored	1
Use DB formulas only	PubChem
Possible ionizations	[M+H]+/[M-H]-
Tree timeout	0
Compound timeout	0
Use heuristic above m/z	300
Use heuristic only above m/z	650
H	0 - inf
C	0 - inf
N	0 - inf
O	0 - inf
P	0 - inf
B	0 - auto ☑
Si	0 - 0 ☐
S	0 - auto ☑
Cl	0 - auto ☑
Se	0 - auto ☑
Br	0 - auto ☑
F	0 - 0
I	0 - 0
**Predict FPs**	
Fallback adducts	[M+H]+/[M-H]-
Score threshold	☑
**Search DBs**	
Search DBs	PubChem
Tag lipids	☑

SI-C2.8.2 MetFrag

MetFrag[28] was run on a local instance of Galaxy, an open source web-based platform for scientific workflows, data integration and analysis.[29,30] It was applied with two different local databases, encompassing the suspect list and PubChemLite for Exposomics.[31] The msp file generated from Compound Discoverer and mzVault was used as input together with the two local data bases in the csv file format. The processing parameters were set as specified in Table SI-C58.

Table SI-C58: Parameter settings of MetFrag.

**MetFrag**	
MSP file	Output from Compound Discoverer and mzVault
Choose compound database	Local database (csv)
Local database of compounds	Suspect list, PubChemLite for Exposomics
Relative mass deviation for database search (ppm)	10.0
Fragment peak match relative mass deviation (ppm)	5.0
Fragment peak match absolute mass deviation (Da)	0.001
Polarity	Positive/Negative
Schema	Generic MSP
Choose how additional metadata columns are extracted	Extra metadata columns from MSP parameters
Suspect list	Do not include suspect list
MetFrag score types	FragmenterScore, OfflineMetFusionScore
MetFrag score weights	1.0, 1.0
How to handle adducts	Select from list<br>[M+H]+, [M+NH4]+, [M+Na]+/[M-H]-
Skip invalid or undefined adduct types	No
Minimum number of MS/MS peaks	0
Output the MetFrag command line call	Yes
Preprocessing filters	None
Postprocessing filter	None

SI-C2.8.3 FISh Scoring

FISh Scoring was applied within Compound Discoverer to selected entries in the compound table in order to explain fragments in MS2 spectra based on *in silico* fragmentation prediction. The algorithm attempts to match the fragment structures of a list of expected fragments to the observed fragments, based on the previously annotated structure. The FISh coverage score is then calculated as follows, where the used fragments represent the sum of matched and unmatched fragments:

$$FISh\, coverage\, score = \frac{\#\, matched\, fragments}{\#\, used\, fragments} \cdot 100 \quad . \tag{10}$$

Table SI-C59 summarizes the FISh Scoring settings.

Table SI-C59: Parameter settings of FISh Scoring.

**FISh Scoring**	
Annotate full spectrum tree	☑
Use general rules	☑
Use fragmentation libraries	☑
Allow aromatic cleavage	☑
Max. depth	5
High accuracy mass tolerance	5 ppm
Low accuracy mass tolerance	0.5 Da
S/N threshold	3

SI-C2.9 Molecular Networking

Molecular networking was applied to get further confidence in the identification of human metabolites. MS2 spectra are put into a network-shaped map based on spectral similarity, which suggests structural similarity. Since a parent and its metabolites are structurally related, a clustering of parent-metabolite pairs is expected.[32,33] Molecular networking was conducted within Compound Discoverer for components with an exact mass matching the suspect list, whereby no transformation reactions were included. The detailed parameters are given in Table SI-C53. For visualization of the molecular network, the links and nodes were exported in the json file format and pictured with the igraph package[34] within R.[12] The obtained molecular networks from positive and negative ionization are shown in Figures SI-C12 and SI-C13.

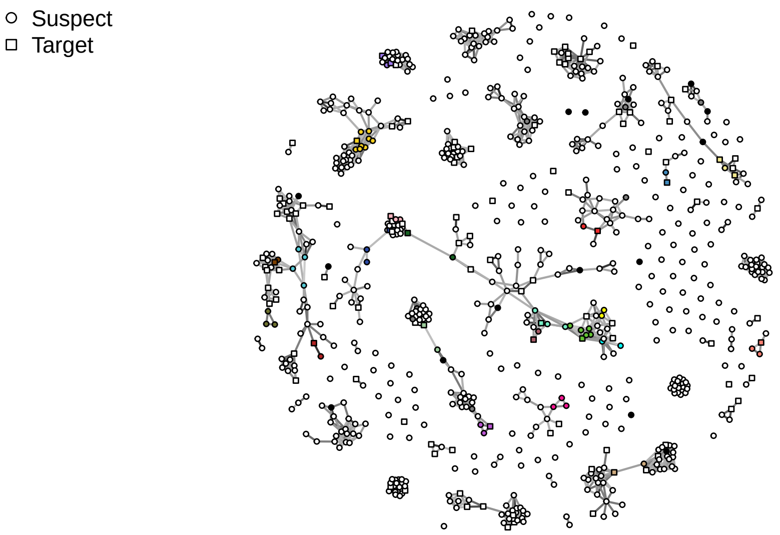

Figure SI-C12: Molecular network of positive ionization mode data. Colored dots indicate clusters which enabled the tentative identification of suspects. Black dots are other tentatively identified pharmaceuticals/pharmaceutical metabolites, gray dots indicate other compounds.

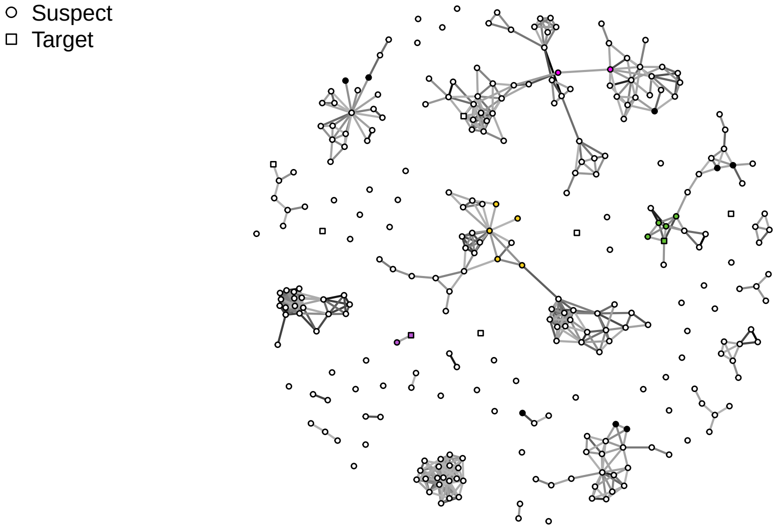

Figure SI-C13: Molecular network of negative ionization mode data. Colored dots indicate clusters which enabled the tentative identification of suspects. Black dots are other tentatively identified pharmaceuticals/pharmaceutical metabolites, gray dots indicate other compounds.

SI-C2.10 Retention Time Prediction

Retention time prediction was achieved based on a linear regression of predicted $\log D_{OW}$ values[35] and measured retention times of 369 compounds in positive and 81 compounds in negative ionization mode. The linear regressions are displayed in Figures SI-C14 and SI-C15, respectively.

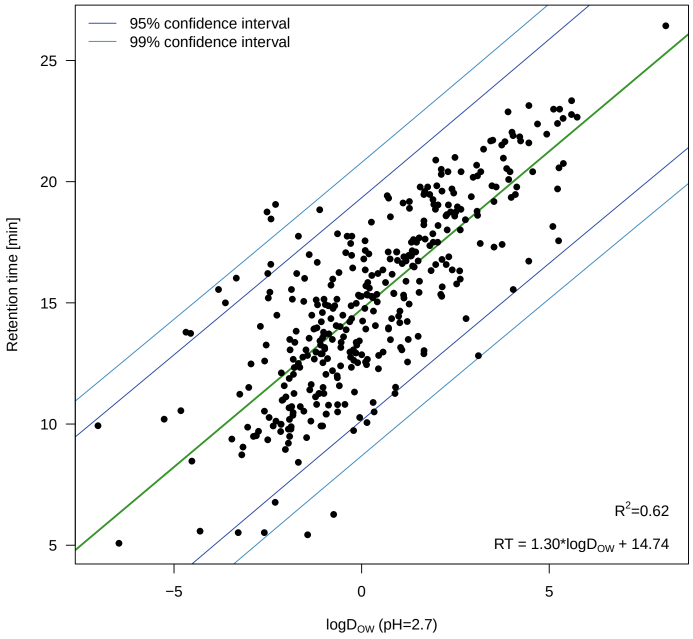

Figure SI-C14: Retention time prediction based on predicted $\log D_{OW}$ values at chromatographic pH against measured retention time of 369 compounds in the positive ionization mode.

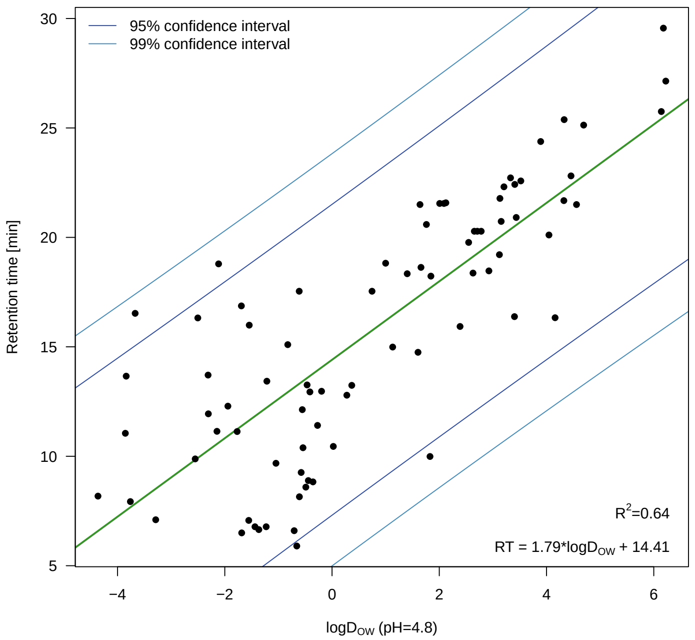

Figure SI-C15: Retention time prediction based on predicted $\log D_{OW}$ values at chromatographic pH against measured retention time of 81 compounds in the negative ionization mode.

SI-C2.11 Confidence Score Calculation

Besides the widely known and applied confidence levels[36] used for communicating the confidence of identification from suspect and non-target screening methods, an additional approach has recently been developed.[2] This approach is based on a score with values between 0 and 1, which takes mass accuracy, retention time information, isotopic fit and presence and number of MS2 fragments into account. Moreover, it considers whether the data were acquired in DDA or DIA mode. The score can be determined in an automated, concise and unambiguous manner and defined threshold values allow a translation of the score to the confidence levels. However, no official scripts are provided for the calculation of this score so far. Moreover, retention time information is incorporated via the retention time indices, which requires the measurement of 18 calibrants with the same chromatographic system as the samples.[37] Since these calibrants were not measured during this measurement campaign, retention time indices cannot be applied and retention time information had to be included in a different way. To do so, a linear relationship between $\log D_{OW}$ and retention of target compounds was established (see Section SI-C2.10). If the predicted retention time of a suspect lies within the 95% confidence interval given by the linear regression, the full amount of points is given, which equals to 0.15. In case the suspect compounds lies within the 99% confidence interval, half of the maximum possible points is given, which are 0.075. Suspects outside the 99% confidence interval receive no points. Table SI-C60 summarizes the adapted scoring system such that it was applicable to this study. The respective R code can be found on GitLab (https://gitlab.com/Corina_Meyer/confidence-score-suspect-identification-with-lc-msms).

Table SI-C60: Identification point system for suspect and non-target HRMS/MS analysis. Adapted from.[2]

Precursor ion (accuracy: 5 ppm, resolution: > 140,000)	mandatory
Retention time within 95% confidence interval of $logD_{OW}$ vs RT	0.15
Retention time within 99% confidence interval of $logD_{OW}$ vs RT	0.075
Retention time outside 99% confidence interval of $logD_{OW}$ vs RT	0
Isotopic fit, based on dot product	max. 0.2
Library spectrum available: Match of most intense fragment ion (without molecular ion)	0.2
All other fragment ions: number of experimental fragments normalized by the number of fragments in the library spectrum	max. 0.2
Penalizing if less than 2 fragments (without molecular ion) are in the MS2 spectrum	-0.1
In silico predicted fragment ions: Prediction of the ten most intense fragments (if less than 10 fragments in the spectrum, normalized to the number of fragments available)	max. 0.2

SI-C2.12 Semiquantification

For semiquantification, two different approaches were applied, depending on the ionization mode. For the positive mode, MS2Quant was applied,[38] using 122 calibrant compounds previously analyzed in target screening. Six of the calibration curve points (0.1 ng/L, 1 ng/L, 10 ng/L, 50 ng/L, 100 ng/L and 1000 ng/L) were used for each calibrant. Low concentration calibration curve points in which a compound was not detectable, were excluded. To account for matrix effects, the sample spiked with 100 ng/L before sample preparation was used in combination with the 100 ng/L calibration curve point to calculate a response factor, which was used to correct the other included calibration curve points. To test the applied procedure, 15 previously quantified targets, spanning the whole retention time range and previously not used as calibrants in the model, were evaluated. Figure SI-C16 shows the obtained concentration ratios between the predicted and the experimental concentrations. It becomes visible that the majority of the predicted concentrations differs only by a factor of two from the experimental concentrations. An exception are the two late eluting compounds etodolac and flufenamic acid, both containing a carboxylic acid functionality, where MS2Quant underestimates the concentration by about one order of magnitude. Semiquantification was performed separately for the three different WWTP matrices.

To further analyze if pharmaceutical metabolites are in the scope for semiquantification with MS2Quant, a principal component analysis (PCA) with the 1191 training data set compounds and the identified suspects was conducted. For this purpose, one-dimensional and two-dimensional Pharmaceutical Data Exploration Laboratory (PaDEL) descriptors[?] were applied. PaDEL descriptors with more than ten missing values per descriptor, near-zero variance and strongly correlating descriptors relating to the training data set compounds were removed, such that 144 descriptors remained.[38] Figure SI-C17 shows the resulting PCA. It becomes visible that the suspects lie within the scope of MS2Quant. This finding suggests that semiquantification based on ionization efficiency, which is applied within MS2Quant, is also reasonable for metabolites. In combination with a previously published study, which showed better accuracy for the semiquantification of transformation products based on ionization efficiency than based on parent compounds or close eluting compounds,[?] it can be concluded that MS2Quant is indeed suitable for the semiquantification of metabolites.

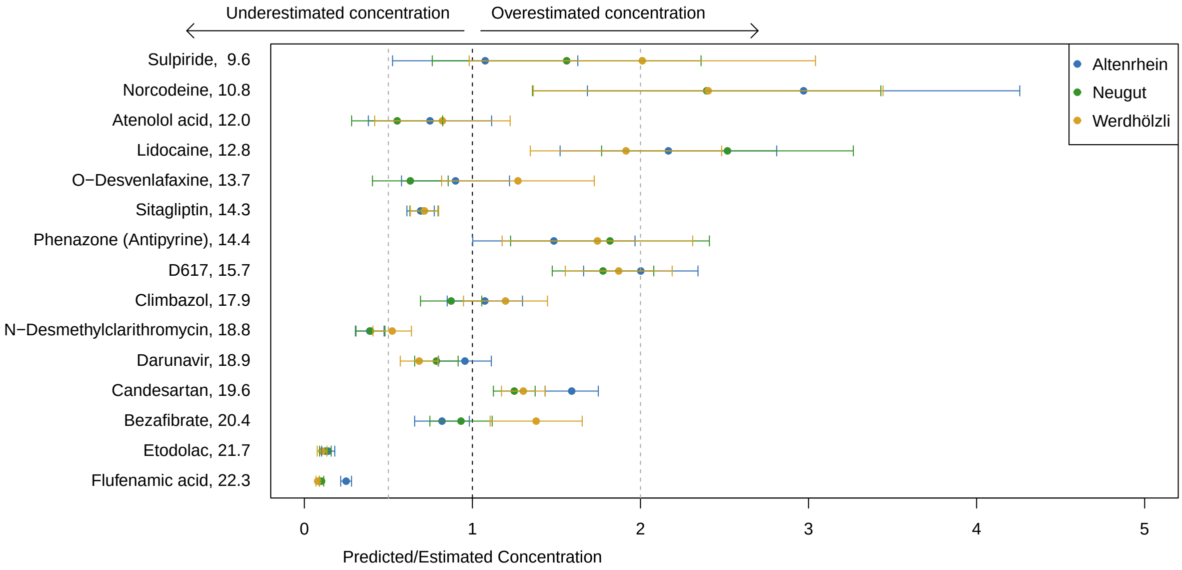

Figure SI-C16: Ratios of predicted (MS2Quant) and experimental concentrations of the 15 test compounds. The retention time in minutes is indicated next to the compound.

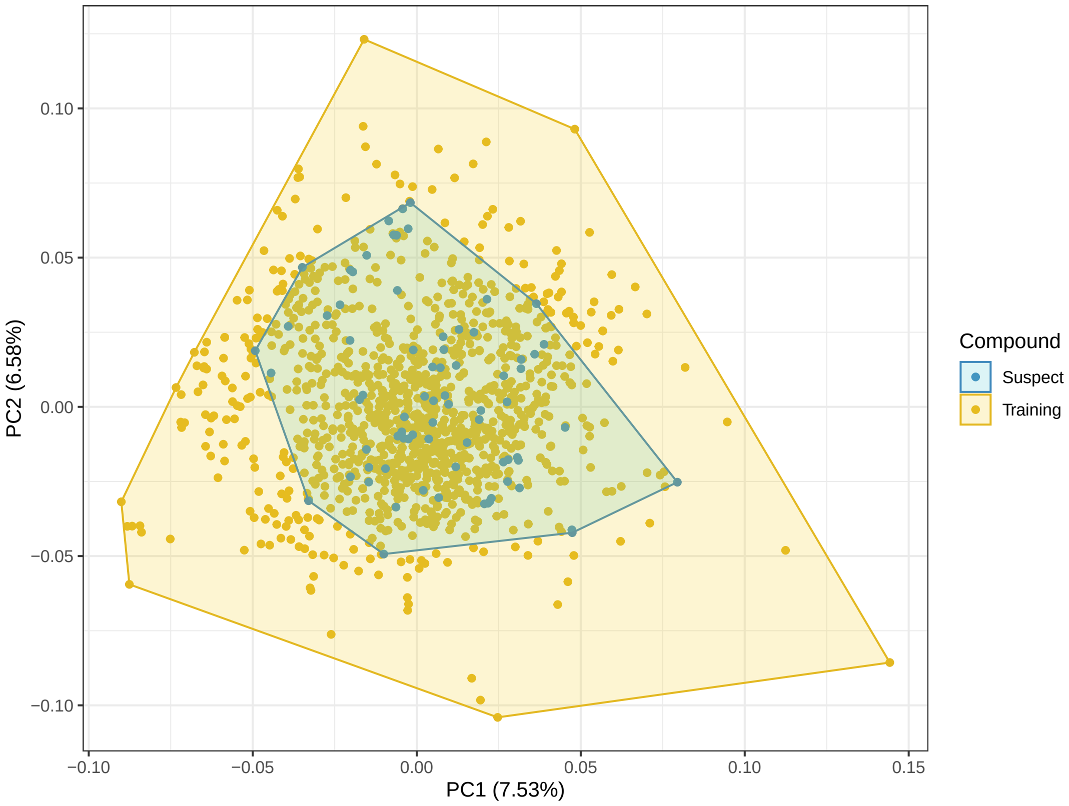

Figure SI-C17: Principal component analysis of the MS2Quant training set compounds and the quantified suspects based on 144 PaDEL descriptors.

At the time semiquantification was conducted, no model within MS2Quant was available for the negative ionization mode. Therefore, if a reference material was at hand, an external calibration curve in the range from 0.1 ng/L to 2500 ng/L was measured in the same batch as the confirmation measurements of the suspects with reference standards. Since only a few samples were remeasured in this batch and since the analytes might have degraded since the first

measurement, despite being stored at 20 °C, the intensities from the first measurement batch were taken for semiquantification. In addition to the matrix correction, this also necessitates a correction for the different sensitivities of the mass spectrometer during these two measurement campaigns. Matrix correction for individual compounds was achieved by assigning an internal standard that elutes in the same retention time range as the suspect and is, if possible, of similar structure. However, since no recovery experiments for these suspects were conducted, it cannot be evaluated if the assigned internal standard can adequately correct the matrix effects. For sensitivity correction, the measured intensities of the assigned internal standards in the calibration samples were compared between the first and second measurement campaign and a sensitivity correction factor was derived by dividing the mean intensity from the second measurement by the mean intensity from the first measurement. The highest two calibration curve points at 1000 and 2500 ng/L were excluded, as matrix suppression effects were observed for some internal standards. Dividing the concentrations derived from the external calibration curve by this factor, which already encompasses the matrix correction, allows for sensitivity correction. Figure SI-C18 displays the measured internal standard intensities in the calibration curves from the two measurement batches for the negative ionization mode.

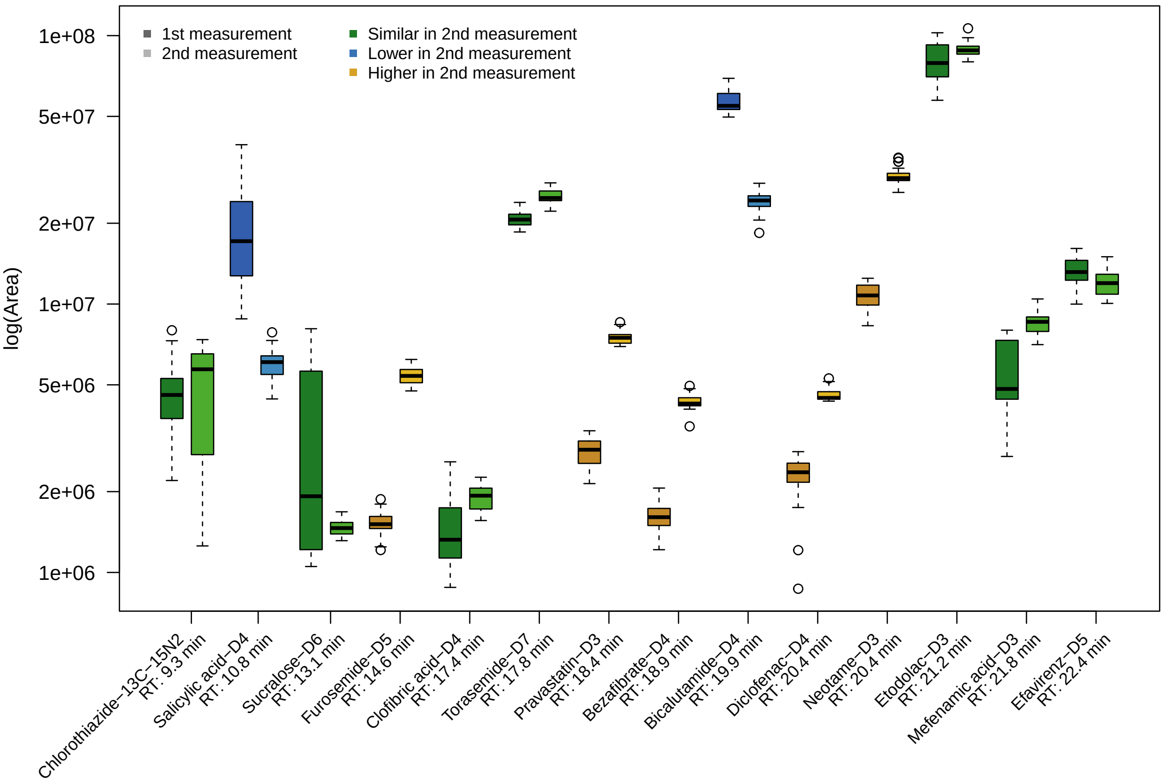

Figure SI-C18: Intensities of internal standards measured in the calibration curves of the two different measurement batches in the negative mode. Internal standards are ordered by increasing retention time.

SI-C2.13 Phase II Metabolite Search: Glucuronide, Sulfate, Glutathione

Phase II metabolism involves conjugation of a body's own group, for example glucuronide, sulfate, glutathione, methyl or acetyl, to a compound to be metabolized. Such conjugates lead during fragmentation to specific neutral losses and characteristic fragments. This fact can be used to screen MS2 spectra specifically for human phase II metabolites that were not previously addressed in the suspect screening. Neutral loss search was applied within Compound Discoverer for positive and negative ionization mode data to screen for glucuronide and sulfate conjugates.

The corresponding Δmasses are equal to 176.0321 Da and 79.9568 Da. In Table SI-C55 the parameter settings of the neutral loss node are specified. The compound class scoring node in Compound Discoverer for negative ionization mode data was used to screen for characteristic fragments of glucuronide and glutathione conjugates. These fragments are listed in Tables SI-C61 and SI-C62, respectively, while Table SI-C56 lists the parameter settings of the compound class scoring node.

Table SI-C61: Specific MSn fragments of glucuronide.[3]

Formula	m/z
$C_6H_7O_6^-$	175.0248
$C_5H_5O_3^-$	113.0244
$C_4H_3O_3^-$	99.0088
$C_5H_3O_2^-$	95.0139
$C_4H_5O_2^-$	85.0295
$C_2H_3O_3^-$	75.0088
$C_3H_3O_2^-$	71.0139

Table SI-C62: Specific MSn fragments of glutathione.[4]

Formula	m/z
$C_{10}H_{16}N_3O_6S^-$	306.0765
$C_{10}H_{14}N_3O_6^-$	272.0888
$C_{10}H_{12}N_3O_5^-$	254.0782
$C_9H_{12}N_3O_3^-$	210.0884
$C_8H_7N_2O_3^-$	179.0462
$C_5H_6NO_3S^-$	160.0074
$C_5H_7N_2O_3^-$	143.0462
$C_5H_6NO_3^-$	128.0353

SI-C2.14 Summarizing Information

The resulting information from Compound Discoverer, mzCloud library search, mzVault library search, SIRIUS/CSI:FingerID, MetFrag and FISh Scoring as well as molecular networking are summarized in SI-B3 and SI-D. The identified compounds are grouped into different categories, where the first four cover the suspect screening approach, and the last the non-targeted approach.

- Parent compounds prioritized by suspect list but not covered by target approach (SI-D1)
- Metabolites prioritized by suspect list that are identified with different levels of confidence but are not covered by target approach (SI-D2)
- Metabolites prioritized by suspect list that turned out to be human metabolites but not of the prioritized parent compounds (SI-D3)
- Compounds prioritized by suspect list that turned out to be neither parent pharmaceutical compounds nor human metabolites thereof (SI-D4)
- Candidates prioritized by neutral loss and/or characteristic fragment search (SI-D5)

SI-C3 Suspect Screening - Exclusion Criteria

Following data processing and prioritization of suspect components based on exact mass, 3348 hits in positive and 1648 hits in negative ionization mode were initially annotated. Subsequent application of exclusion criteria such as low intensity (1.5%/2% omitted in pos/neg mode, respectively), poor peak shape (30%/17.5%), missing MS2 spectra (65%/79%), background filtering (3%/1%), and unrealistic retention time (0.5%/0.5%), led to a reduced list of 553 hits in positive and 276 hits in negative ionization mode, of which 159 were detected in both modes. Figure SI-C19 displays the intensity distribution of the components prioritized based on exact mass of suspect list in the positive and negative ionization mode, respectively. The components are assigned into two groups, depending on the acquisition of an MS2 spectrum. It becomes visible that the intensities of components with acquired MS2 spectrum are higher in both modes, but that this effect is more pronounced in the positive ionization mode. Consequently, low intensity components were more likely to be neglected than high intensity components due to the missing MS2 information.

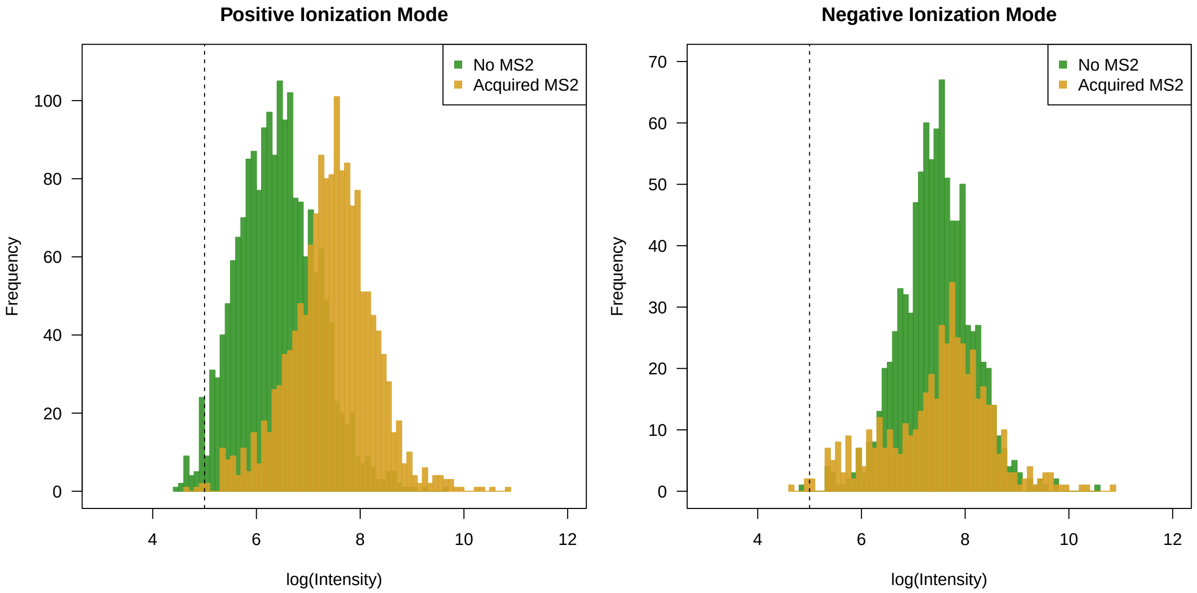

Figure SI-C19: Intensity distribution of components prioritized based on exact mass of the suspect list in the positive and negative ionization mode, respectively. For components in brown an MS2 spectrum was acquired, while green components do not have an MS2 spectrum. The dashed line indicates the intensity cutoff.

References

[1] Delabriere, A.; Warmer, P.; Brennsteiner, V.; Zamboni, N. SLAW: A Scalable and Self-Optimizing Processing Workflow for Untargeted LC-MS. *Analytical Chemistry* **2021**, *93*, 15024–15032.

[2] Alygizakis, N.; Lestremau, F.; Gago-Ferrero, P.; Gil-Solsona, R.; Arturi, K.; Hollender, J.; Schymanski, E. L.; Dulio, V.; Slobodnik, J.; Thomaidis, N. S. Towards a harmonized identification scoring system in LC-HRMS/MS based non-target screening (NTS) of emerging contaminants. *TrAC - Trends in Analytical Chemistry* **2023**, *159*, 1–11.

[3] Evich, M. G.; Mosley, J. D.; Ntai, I.; Cavallin, J. E.; Villeneuve, D. L.; Ankley, G. T.; Collette, T. W.; Ekman, D. R. Untargeted MSn-Based Monitoring of Glucuronides in Fish: Screening Complex Mixtures for Contaminants with Biological Relevance. *ACS ES and T Water* **2022**, *2*, 2481–2490.

[4] Dieckhaus, C. M.; Fernández-Metzler, C. L.; King, R.; Krolikowski, P. H.; Baillie, T. A. Negative ion tandem mass spectrometry for the detection of glutathione conjugates. *Chemical Research in Toxicology* **2005**, *18*, 630–638.

[5] Thermo Fisher Scientific, Compound Discoverer. 2021.

[6] Schmid, R. et al. Integrative analysis of multimodal mass spectrometry data in MZmine 3. *Nature Biotechnology* **2023**, *41*, 447–449.

[7] Tsugawa, H.; Cajka, T.; Kind, T.; Ma, Y.; Higgins, B.; Ikeda, K.; Kanazawa, M.; Vandergheynst, J.; Fiehn, O.; Arita, M. MS-DIAL: Data-independent MS/MS deconvolution for comprehensive metabolome analysis. *Nature Methods* **2015**, *12*, 523–526.

[8] Smith, C. A.; Want, E. J.; O'Maille, G.; Abagyan, R.; Siuzdak, G. XCMS: Processing mass spectrometry data for metabolite profiling using nonlinear peak alignment, matching, and identification. *Analytical Chemistry* **2006**, *78*, 779–787.

[9] Röst, H. L. et al. OpenMS: A flexible open-source software platform for mass spectrometry data analysis. 2016.

[10] Chambers, M. C. et al. A cross-platform toolkit for mass spectrometry and proteomics. 2012.

[11] Loos, M. enviMass version 3.5 LC-HRMS trend detection workflow - R package. 2019; `https://doi.org/10.5281/zenodo.1213098`.

[12] R Core Team, R: A Language and Environment for Statistical Computing. 2022; `https://www.R-project.org/`.

[13] Dührkop, K.; Fleischauer, M.; Ludwig, M.; Aksenov, A. A.; Melnik, A. V.; Meusel, M.; Dorrestein, P. C.; Rousu, J.; Böcker, S. SIRIUS 4: a rapid tool for turning tandem mass spectra into metabolite structure information. *Nature Methods* **2019**, *16*, 299–302.

[14] Wang, M. et al. Sharing and community curation of mass spectrometry data with Global Natural Products Social Molecular Networking. *Nature Biotechnology* **2016**, *34*, 828–837.

[15] Pluskal, T.; Castillo, S.; Villar-Briones, A.; Orešič, M. MZmine 2: Modular framework for processing, visualizing, and analyzing mass spectrometry-based molecular profile data. *BMC Bioinformatics* **2010**, *11*.

[16] Chum, O.; Matas, J.; Kittler, J. In *Pattern Recognition*; Goos, G., Hartmanis, J., van Leeuwen, J., Eds.; 2003; pp 236–243.

[17] Senan, O.; Aguilar-Mogas, A.; Navarro, M.; Capellades, J.; Noon, L.; Burks, D.; Yanes, O.; Guimera, R.; Sales-Pardo, M. CliqueMS: A computational tool for annotating in-source metabolite ions from LC-MS untargeted metabolomics data based on a coelution similarity network. *Bioinformatics* **2019**, *35*, 4089–4097.

[18] Jaeger, C.; Hoffmann, F.; Schmitt, C. A.; Lisec, J. Automated Annotation and Evaluation of In-Source Mass Spectra in GC/Atmospheric Pressure Chemical Ionization-MS-Based Metabolomics. *Analytical Chemistry* **2016**, *88*, 9386–9390.

[19] Frank, A. M.; Bandeira, N.; Shen, Z.; Tanner, S.; Briggs, S. P.; Smith, R. D.; Pevzner, P. A. Clustering millions of tandem mass spectra. *Journal of Proteome Research* **2008**, *7*, 113–122.

[20] Karla Johanning, et al. Assessment of Metabolic Stability Using the Rainbow Trout (Oncorhynchus mykiss) Liver S9 Fraction. *Current Protocols in Toxicology* **2012**, *53*, 1–14.

[21] Huber, C.; Müller, E.; Schulze, T.; Brack, W.; Krauss, M. Improving the Screening Analysis of Pesticide Metabolites in Human Biomonitoring by Combining High-ThroughputIn VitroIncubation and Automated LC-HRMS Data Processing. *Analytical Chemistry* **2021**, *93*, 9149–9157.

[22] HighChem LLC, mzCloud - Advanced Mass Spectral Database. `https://www.mzcloud.org/`.

[23] MassBank - High Quality Mass Spectral Database. `https://massbank.eu/MassBank/`.

[24] NIST Tandem Mass Spectral Library. `http://chemdata.nist.gov`.

[25] mzVault.

[26] Hoffmann, M. A.; Nothias, L.-F.; Ludwig, M.; Fleischauer, M.; Gentry, E. C.; Witting, M.; Dorrestein, P. C.; Dührkop, K.; Böcker, S. Assigning confidence to structural annotations from mass spectra with COSMIC. *bioRxiv* **2021**, 2021.03.18.435634.

[27] Ludwig, M.; Dührkop, K.; Böcker, S. Bayesian networks for mass spectrometric metabolite identification via molecular fingerprints. *Bioinformatics* **2018**, *34*, i333–i340.

[28] Ruttkies, C.; Schymanski, E. L.; Wolf, S.; Hollender, J.; Neumann, S. MetFrag relaunched: Incorporating strategies beyond in silico fragmentation. *Journal of Cheminformatics* **2016**, *8*, 1–16.

[29] Afgan, E. et al. The Galaxy platform for accessible, reproducible and collaborative biomedical analyses: 2016 update. *Nucleic Acids Research* **2016**, *44*, W3–W10.

[30] Blankenberg, D.; Coraor, N.; Von Kuster, G.; Taylor, J.; Nekrutenko, A. Integrating diverse databases into an unified analysis framework: A Galaxy approach. *Database* **2011**, *2011*, 1–9.

[31] Bolton, E. K. T. T. P. Z. J., Evan; Schymanski PubChemLite for Exposomics. 2023; `https://zenodo.org/record/7576412`.

[32] Vincenti, F.; Montesano, C.; Di Ottavio, F.; Gregori, A.; Compagnone, D.; Sergi, M.; Dorrestein, P. Molecular Networking: A Useful Tool for the Identification of New Psychoactive Substances in Seizures by LCHRMS. *Frontiers in Chemistry* **2020**, *8*, 1–9.

[33] Yu, J. S.; Nothias, L. F.; Wang, M.; Kim, D. H.; Dorrestein, P. C.; Kang, K. B.; Yoo, H. H. Tandem Mass Spectrometry Molecular Networking as a Powerful and Efficient Tool for Drug Metabolism Studies. *Analytical Chemistry* **2022**, *94*, 1456–1464.

[34] YU, G.; CHEN, Y.-s.; GUO, Y.-c. Design of integrated system for heterogeneous network query terminal. *Journal of Computer Applications* **2009**, *29*, 2191–2193.

[35] Chemaxon, JChem for Office. `https://chemaxon.com/jchem-for-office`.

[36] Schymanski, E. L.; Jeon, J.; Gulde, R.; Fenner, K.; Ruff, M.; Singer, H. P.; Hollender, J. Identifying small molecules via high resolution mass spectrometry: Communicating confidence. *Environmental Science and Technology* **2014**, *48*, 2097–2098.

[37] Aalizadeh, R. et al. Development and Application of Liquid Chromatographic Retention Time Indices in HRMS-Based Suspect and Nontarget Screening. *Analytical Chemistry* **2021**, *93*, 11601–11611.

[38] Sepman, H.; Malm, L.; Peets, P.; MacLeod, M.; Martin, J.; Breitholtz, M.; Kruve, A. Bypassing the Identification: MS2Quant for Concentration Estimations of Chemicals Detected with Nontarget LC-HRMS from MS2 Data. *Analytical Chemistry* **2023**,

Supporting Information D (SI-D) to:

How Wastewater Reflects Human Metabolism - Suspect Screening of Pharmaceutical Metabolites in Untreated Wastewater

Corina Meyer[†,‡], Michael A. Stravs[†], Juliane Hollender[*,†,‡]

[†] Eawag: Swiss Federal Institute of Aquatic Science and Technology, Ueberlandstrasse 133, 8600, Duebendorf, Switzerland
[‡] Institute of Biogeochemistry and Pollutant Dynamics, Universitaetstrasse 16, ETH Zurich, 8092, Zurich, Switzerland

[*] Corresponding author: juliane.hollender@eawag.ch

Environmental Science & Technology, 2024

Supporting Pages: S80 - S577
Supporting Figures: SI-D1 - SI-D514
Supporting Tables: SI-D1 - SI-D261

Contents

SI-D1 Parent Pharmaceutical Compounds **S83**
- SI-D1.1 Abacavir . . . S83
- SI-D1.2 Acyclovir . . . S88
- SI-D1.3 Lamivudine . . . S93
- SI-D1.4 Nevirapine . . . S98
- SI-D1.5 Pentoxifylline . . . S104
- SI-D1.6 Quinine . . . S110

SI-D2 Human Metabolites of Prioritized Pharmaceuticals **S115**
- SI-D2.1 Abacavir Metabolites . . . S115
  - SI-D2.1.1 Abacavir-5'-Carboxylate . . . S117
  - SI-D2.1.2 Abacavir-5'-Phosphate . . . S123
- SI-D2.2 Aliskiren Metabolites . . . S127
  - SI-D2.2.1 Aliskiren Metabolite M2 . . . S128
  - SI-D2.2.2 Aliskiren Metabolite M4 . . . S133
- SI-D2.3 Amlodipine Metabolites . . . S136
  - SI-D2.3.1 Amlodipine Metabolite M9 . . . S137
  - SI-D2.3.2 Amlodipine Metabolite M10 . . . S143
- SI-D2.4 Atorvastatin Metabolites . . . S148
  - SI-D2.4.1 ortho-Hydroxyatorvastatin . . . S149
  - SI-D2.4.2 para-Hydroxyatorvastatin . . . S155
- SI-D2.5 Azithromycin Metabolites . . . S161
  - SI-D2.5.1 Azithromycin-13-O-Descladinosyl-9-N-Desmethyl . . . S162
  - SI-D2.5.2 Desosaminylazithromycin . . . S167
- SI-D2.6 Caffeine and Theophylline Metabolites . . . S172
  - SI-D2.6.1 Paraxanthine and Theophylline . . . S174
  - SI-D2.6.2 Theobromine . . . S182
  - SI-D2.6.3 1-Methylxanthine . . . S188
  - SI-D2.6.4 3-Methylxanthine and 7-Methylxanthine . . . S194
  - SI-D2.6.5 1-Methyluric Acid . . . S206
  - SI-D2.6.6 1,7-Dimethyluric Acid . . . S212
  - SI-D2.6.7 1,3,7-Trimethyluric Acid . . . S218
- SI-D2.7 Hesperidin Metabolites . . . S224
  - SI-D2.7.1 Eriodictyol . . . S225
  - SI-D2.7.2 Hesperitin . . . S231
- SI-D2.8 Irbesartan Metabolites . . . S237
  - SI-D2.8.1 Irbesartan Metabolite M1 . . . S238
  - SI-D2.8.2 Irbesartan Metabolite M3 . . . S244
  - SI-D2.8.3 Irbesartan Metabolite M4 . . . S250
  - SI-D2.8.4 Irbesartan Metabolite M5 . . . S255
  - SI-D2.8.5 Irbesartan Metabolite M6 . . . S261
  - SI-D2.8.6 Irbesartan Metabolite SR49498 . . . S266
- SI-D2.9 Losartan Metabolites . . . S271
  - SI-D2.9.1 Losartan Carboxylic Acid . . . S272
  - SI-D2.9.2 Losartan Metabolite M2 . . . S278
  - SI-D2.9.3 Losartan Metabolite M5 . . . S283
- SI-D2.10 Mefenamic Acid Metabolites . . . S288
  - SI-D2.10.1 3-Carboxymefenamic Acid . . . S289
  - SI-D2.10.2 3-Hydroxymethylmefenamic Acid . . . S294
- SI-D2.11 Nevirapine Metabolites . . . S301

SI-D2.11.1 2-Hydroxynevirapine and 12-Hydroxynevirapine S302
SI-D2.11.2 3-Hydroxynevirapine and 8-Hydroxynevirapine S310
SI-D2.12 Tolperisone Metabolites S318
SI-D2.12.1 Tolperisone Metabolite S319
SI-D2.12.2 Tolperisone Metabolites M1 and M1' S323
SI-D2.12.3 Tolperisone Metabolite M3 . . . . S328
SI-D2.12.4 Tolperisone Metabolite M4 . . . . S334
SI-D2.13 Torasemide Metabolites S339
SI-D2.13.1 Torasemide Carboxylic Acid S340
SI-D2.13.2 Torasemide Metabolite M1 . . . . S347
SI-D2.13.3 Torasemide Metabolite M3 . . . . S352
SI-D2.14 Other Phase I Metabolites S357
SI-D2.14.1 2-Hydroxytrimipramine S357
SI-D2.14.2 3-Desmethyltrimethoprim and 4-Desmethyltrimethoprim S363
SI-D2.14.3 4-Desmethylpantoprazole S371
SI-D2.14.4 5-Fluorocytosine S377
SI-D2.14.5 5-Hydroxyomeprazole-Sulfone S382
SI-D2.14.6 5-Hydroxysulfapyridine S387
SI-D2.14.7 6-O-Desmethylmycophenolic Acid S393
SI-D2.14.8 8-Hydroxymirtazapine S398
SI-D2.14.9 14-Hydroxyclarithromycin S404
SI-D2.14.10 α-Hydroxymetoprolol S410
SI-D2.14.11 Benzophenone S416
SI-D2.14.12 Clindamycin-Sulfoxide S422
SI-D2.14.13 Desacetylbisacodyl S427
SI-D2.14.14 Hydroxylevetiracetam S432
SI-D2.14.15 Licarbazepine S436
SI-D2.14.16 N-Desmethylrosuvastatin S443
SI-D2.14.17 N-Methylpregabalin S448
SI-D2.14.18 O-Desarylranolazine S453
SI-D2.14.19 Phenylethylmalonamide (PEMA) S458
SI-D2.14.20 Sacubitrilat (LBQ-657) S464
SI-D2.14.21 Succinic acid S469
SI-D2.14.22 Valeryl-4-Hydroxyvalsartan S474
SI-D2.14.23 Zolpidem Carboxylic Acid S480
SI-D2.15 Other Phase II Metabolites S485
SI-D2.15.1 4-Hydroxypropanolol-Sulfate (HOPS) S485
SI-D2.15.2 4-Quinol-Sulfate S490
SI-D2.15.3 Diphenhydramine-N-Glucuronide S494
SI-D2.15.4 Lamotrigine-N2-Glucuronide S498
SI-D2.15.5 Paracetamol-Sulfate S503
SI-D2.15.6 Phenolic-Glucuronide S508
SI-D2.15.7 Phenylephrine-3-O-Sulfate S513
SI-D2.15.8 Tapentadol-O-Sulfate S518
SI-D2.15.9 Telmisartan-O-Acyl-Glucuronide S523

SI-D3 Metabolites of Non-Prioritized Compounds S527
SI-D3.1 3-Hydroxycotinine S527
SI-D3.2 2',3'-Anhydroinosine S532
SI-D3.3 Methylecgonine S535

SI-D4 Other Compounds S541

SI-D4.1 2-Acetylpyrazine S541
SI-D4.2 4-Hydroxybenzaldehyde S546
SI-D4.3 Hexa(methoxymethyl)melanine (HMMM) S551
SI-D4.4 Methyl Anthranilate S556
SI-D4.5 Piperine S562
SI-D4.6 Sulfurol S568

SI-D5 Non-Target Screening Compounds S573
SI-D5.1 Glucuronide Conjugates: Positive Ionization Mode S573
SI-D5.2 Glucuronide Conjugates: Negative Ionization Mode S573
SI-D5.3 Sulfate Conjugates: Positive Ionization Mode S573
SI-D5.4 Sulfate Conjugates: Negative Ionization Mode S573
SI-D5.5 Glutathione Conjugates: Negative Ionization Mode S575

SI-D1 Parent Pharmaceutical Compounds

SI-D1.1 Abacavir

Abacavir is an antiviral nucleoside reverse transcriptase inhibitor. It is used in combination with other antiretrovirals for the treatment of HIV. In Switzerland it is sold under the brand names Kivexa, Triumeq, Trizivir or Ziagen.[1]

Table SI-D1: Information on identifiers, chemical properties, detection and confidence of identification of abacavir.

IUPAC Name	[(1*S*,4*R*)-4-[2-amino-6-(cyclopropylamino)purin-9-yl]cyclopent-2-en-1-yl]methanol
Molecular formula	$C_{14}H_{18}N_6O$
Monoisotopic mass [g/mol]	286.1542
Adduct	$[M+H]^+$
Retention time [min]	11.9
SMILES	C1CC1NC2=C3C(=NC(=N2)N)N(C=N3)[C@@H]4C[C@@H](C=C4)CO
InChI	InChI=1S/C14H18N6O/c15-14-18-12(17-9-2-3-9)11-13(19-14)20(7-16-11)10-4-1-8(5-10)6-21/h1,4,7-10,21H,2-3,5-6H2,(H3,15,17,18,19)/t8-,10+/m1/s1
InChI-Key	MCGSCOLBFJQGHM-SCZZXKLOSA-N
CAS RN	136470-78-5
Detection frequency	100% (15/15 samples)
Detected in	Altenrhein, Monday-Friday<br>Neugut, Monday-Friday<br>Werdhölzli, Monday-Friday
Intensity	E7
Initial confidence level	level 2a
Initial confidence score	0.63
Final confidence level	level 1

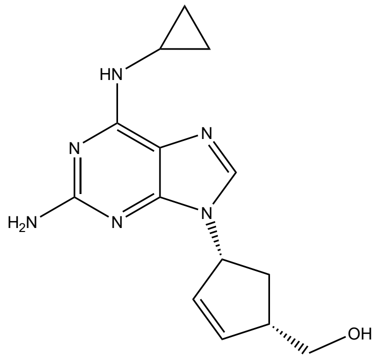

Figure SI-D1: Molecular structure of abacavir.

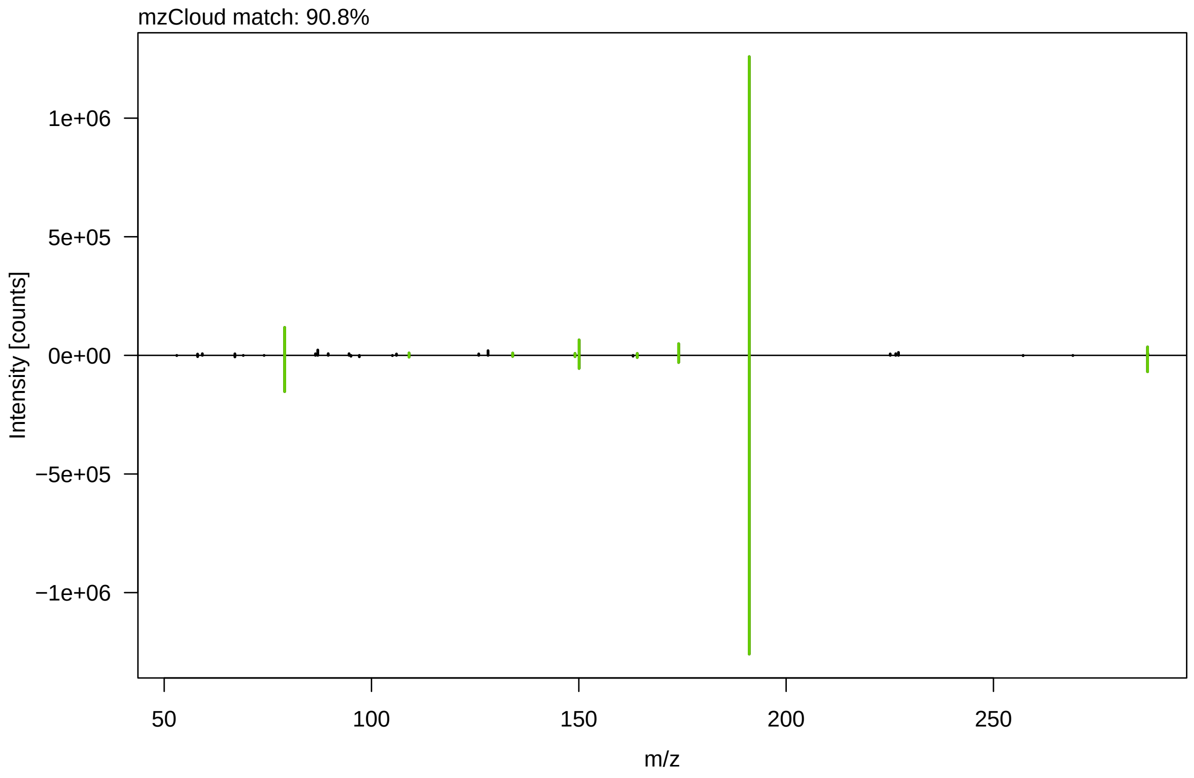

Figure SI-D2: Head to tail plot of measured MS2 spectrum against mzCloud library spectrum of abacavir. Matching fragments are highlighted in green.

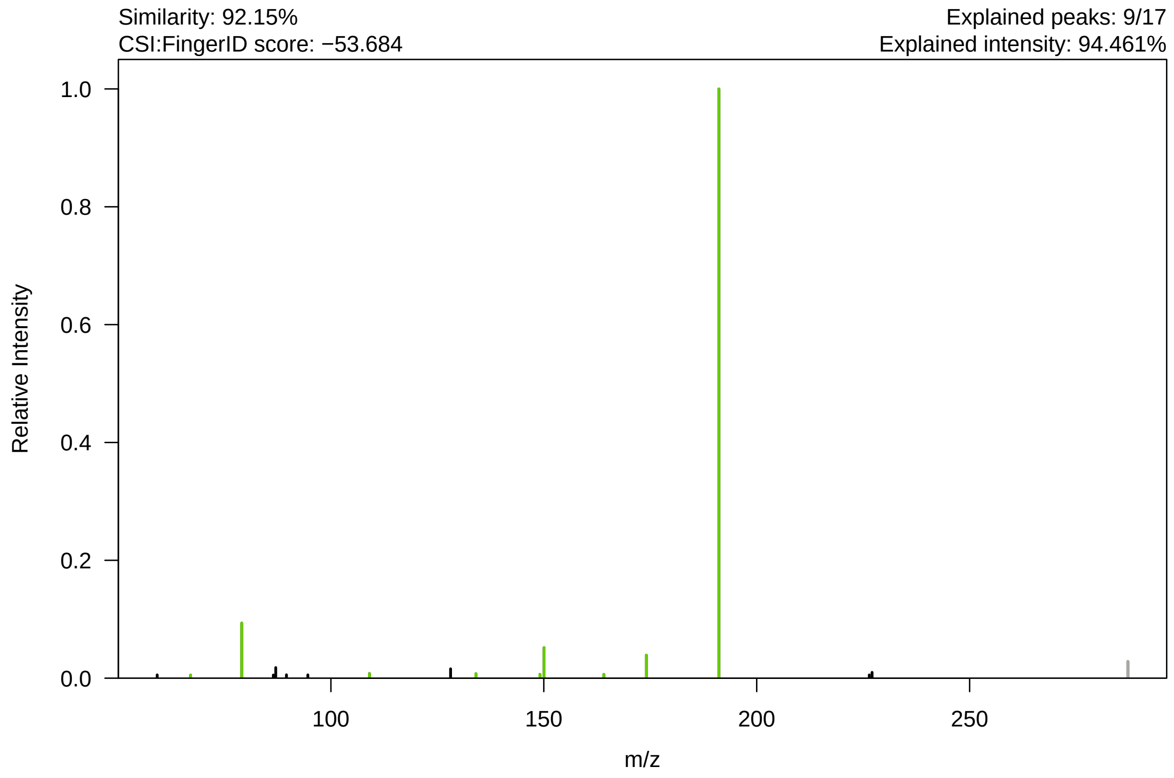

Figure SI-D3: Measured MS2 spectrum. Matching fragments with abacavir predicted by SIRIUS/CSI:FingerID are highlighted in green. The molecular ion in gray is not considered.

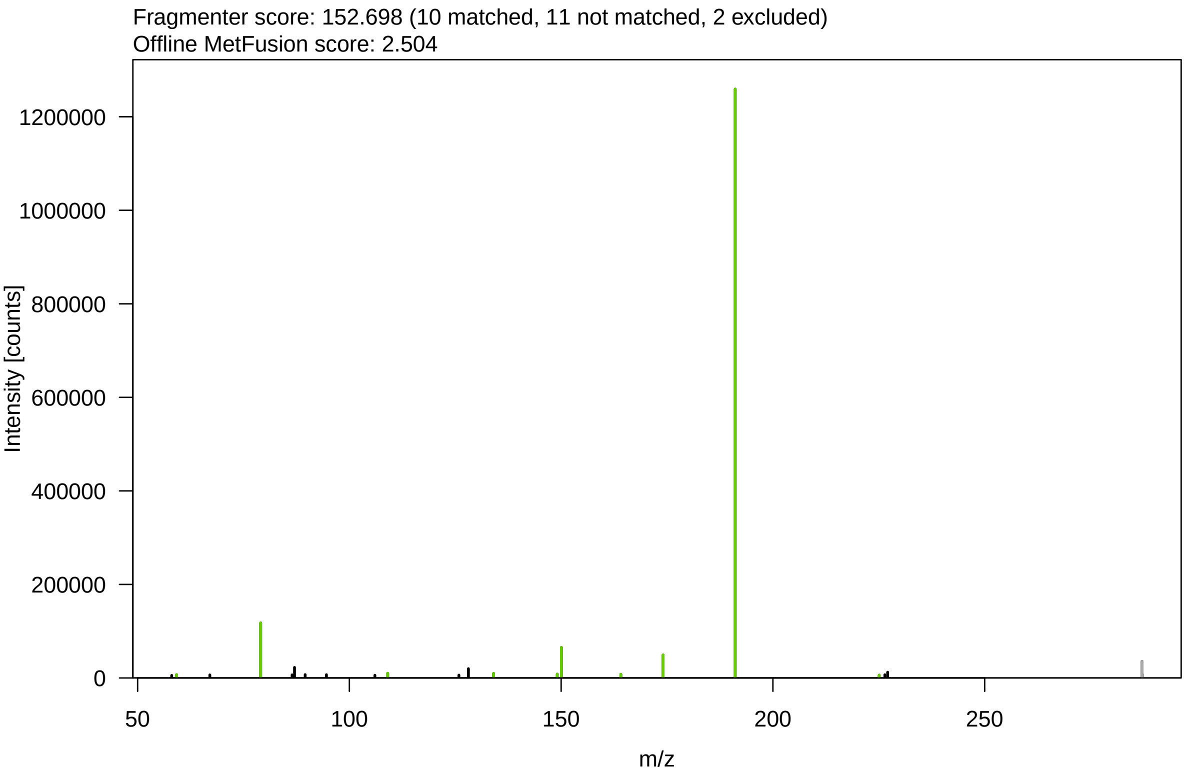

Figure SI-D4: Measured MS2 spectrum. Matching fragments with abacavir predicted by MetFrag are highlighted in green. The molecular ion in gray is not considered.

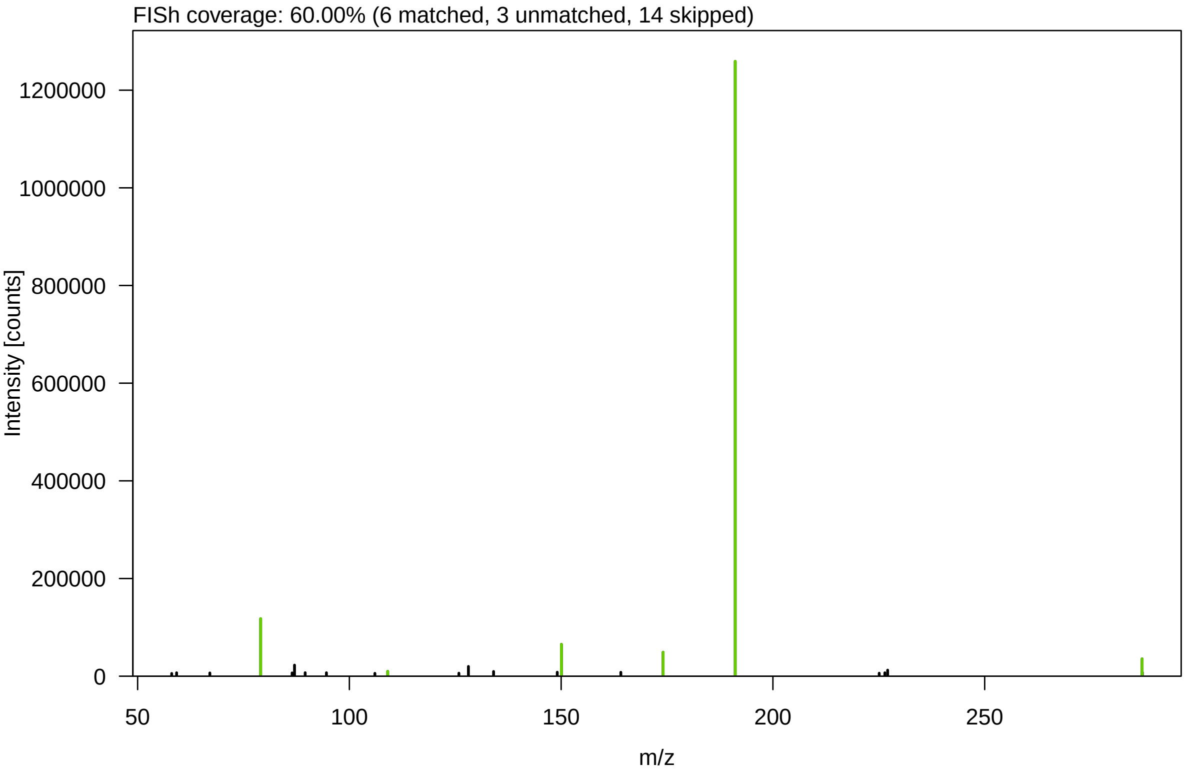

Figure SI-D5: Measured MS2 spectrum. Matching fragments with abacavir predicted by FISh Scoring are highlighted in green. Low intensity fragments are not considered and skipped.

Table SI-D2: Retention time prediction of abacavir.

Measured retention time [min]	11.9
Predicted $logD_{OW}$ (pH = 2.7)	-1.52
Predicted retention time [min]	12.8
Predicted retention time range (95% confidence interval) [min]	8.2-17.4
Predicted retention time range (99% confidence interval) [min]	6.7-18.8

Table SI-D3: Annotated MS2 spectrum of abacavir.

m/z	Relative Intensity	Annotation
58.0498	4.44	
59.2059	5.56	
67.0545	5.24	$C_5H_6 + H^+$
79.0541	93.43	$C_6H_6 + H^+$
86.4817	5.35	
87.0438	17.97	
89.5602	5.69	
94.5841	5.60	
106.0235	4.62	
109.0509	7.96	$C_4H_4N_4 + H^+$
125.8623	4.90	
128.1067	15.82	
134.0454	7.65	$C_5H_3N_5 + H^+$
149.0819	6.45	$C_7H_8N_4 + H^+$
150.0648	51.76	$C_5H_5N_6 + H^+$
164.0928	6.31	$C_7H_9N_5 + H^+$
174.0772	38.97	$C_8H_7N_5 + H^+$
191.1036	999.00	$C_8H_{10}N_6 + H^+$
225.1013	4.86	$C_{12}H_{10}N_5 + H^+$
226.4369	5.53	
227.0914	9.82	
287.1626	28.15	$C_{14}H_{18}N_6O$
287.2307	6.35	

A reference standard of abacavir was purchased. Figure SI-D6 shows the extracted ion chromatograms of this standard, the sample and the spiked sample, as well as a head to tail plot of the MS2 spectra of the standard and the sample. In addition, the most intense MS2 fragments in the sample and in the standard are displayed. It becomes visible that the retention times of the sample and the spiked sample are identical and the spectra similarity score between sample and standard is equal to 0.994. It can therefore be concluded that the suspected compound is indeed abacavir. Correspondingly, the identification confidence can be increased to level 1.

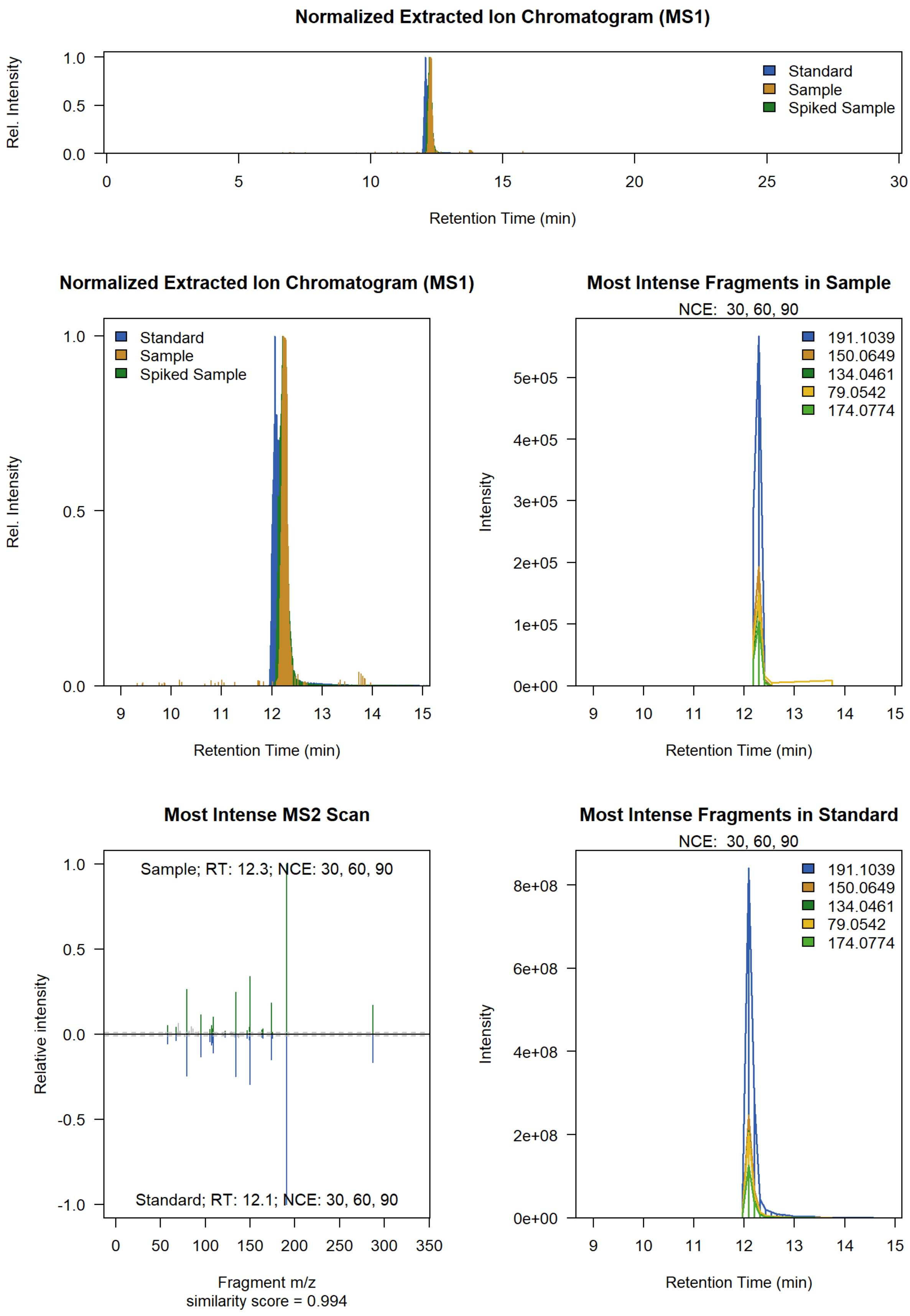

Figure SI-D6: Extracted ion chromatograms of abacavir in the reference standard, the sample and the spiked sample, as well as MS2 head to tail plot and most intense MS2 fragments in standard and sample.

SI-D1.2 Acyclovir

Acyclovir is a nucleotide analog antiviral used to treat *Herpes simplex*, *Varicella zoster* or *Herpes zoster*.[2] In Switzerland it is sold under the brand names Xorox, Zovirax, Aivision, Aviral, Virucalm or Virupos.[1] Besides being a parent compound, acyclovir is a metabolite of valacyclovir, resulting from the splitting of *L*-valine. Similar to acyclovir, valacyclovir is used to treat herpes infections.[2] In Switzerland it is sold under the name Valtrex.[1]

Table SI-D4: Information on identifiers, chemical properties, detection and confidence of identification of acyclovir.

IUPAC Name	2-amino-9-(2-hydroxyethoxymethyl)-1*H*-purin-6-one
Molecular formula	$C_8H_{11}N_5O_3$
Monoisotopic mass [g/mol]	225.0862
Adduct	$[M+H]^+$
Retention time [min]	7.6
SMILES	C1=NC2=C(N1COCCO)N=C(NC2=O)N
InChI	InChI=1S/C8H11N5O3/c9-8-11-6-5(7(15)12-8)10-3-13(6)4-16-2-1-14/h3,14H,1-2,4H2,(H3,9,11,12,15)
InChI-Key	MKUXAQIIEYXACX-UHFFFAOYSA-N
CAS RN	59277-89-3
Metabolite of	Parent<br>Valacyclovir
Detection frequency	100% (15/15 samples)
Detected in	Altenrhein, Monday-Friday<br>Neugut, Monday-Friday<br>Werdhölzli, Monday-Friday
Intensity	E6-E7
Initial confidence level	level 2a
Initial confidecne score	0.49
Final confidence level	level 1

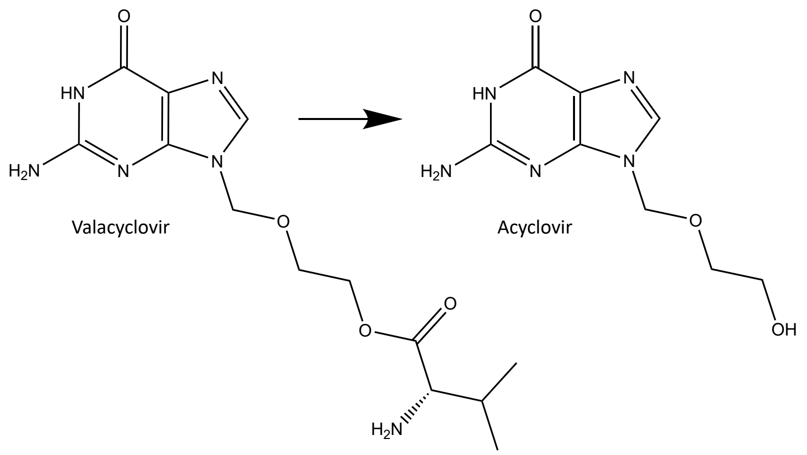

Figure SI-D7: Metabolism of valacyclovir to acyclovir.

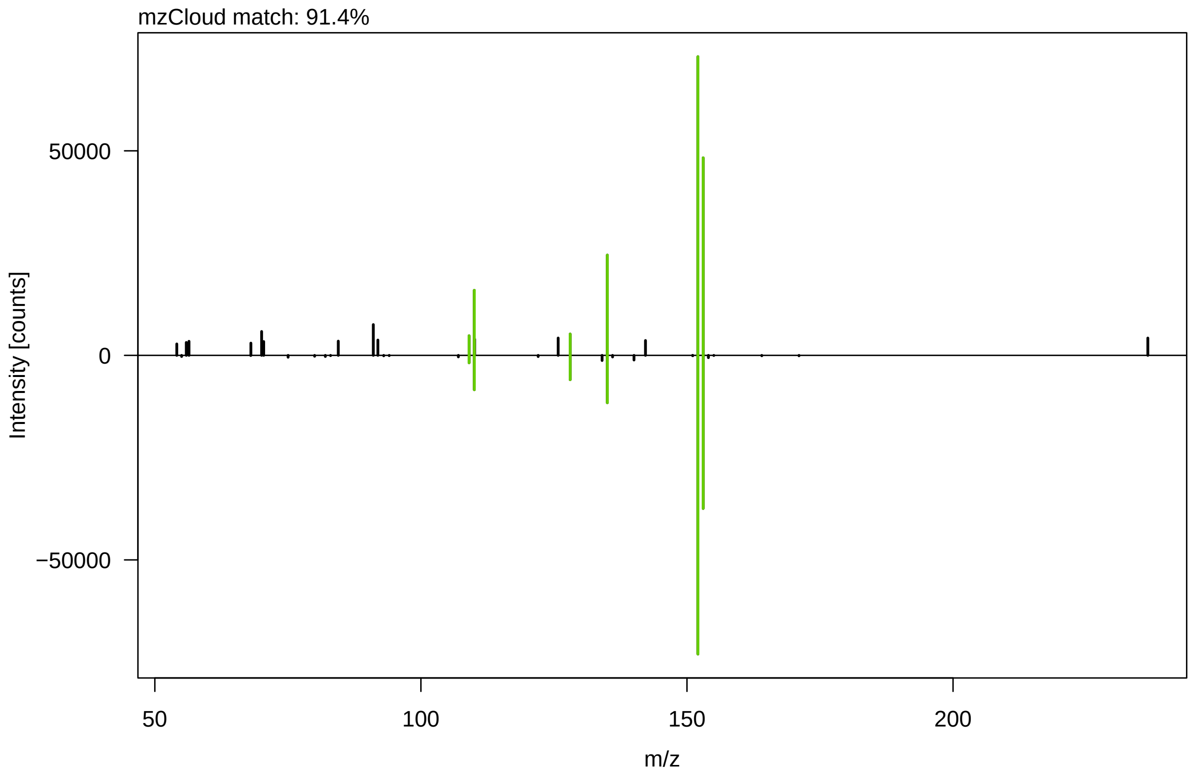

Figure SI-D8: Head to tail plot of measured MS2 spectrum against mzCloud library spectrum of acyclovir. Matching fragments are highlighted in green.

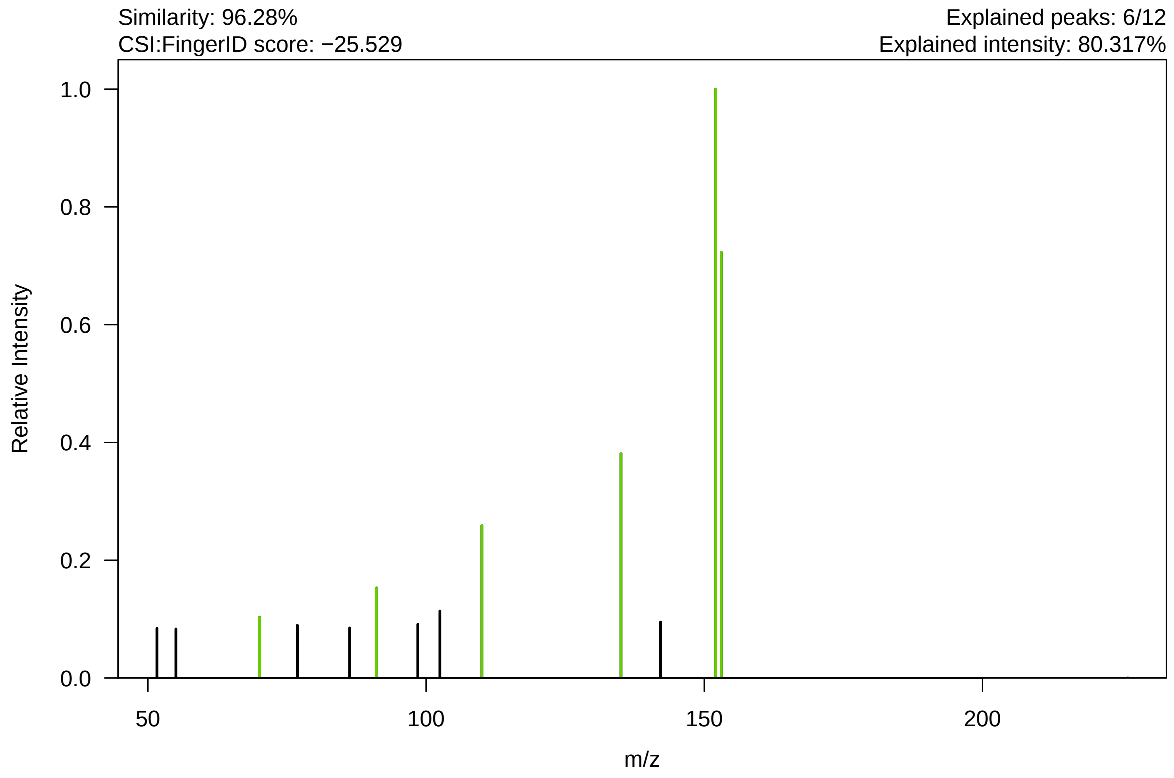

Figure SI-D9: Measured MS2 spectrum. Matching fragments with acyclovir predicted by SIRIUS/CSI:FingerID are highlighted in green.

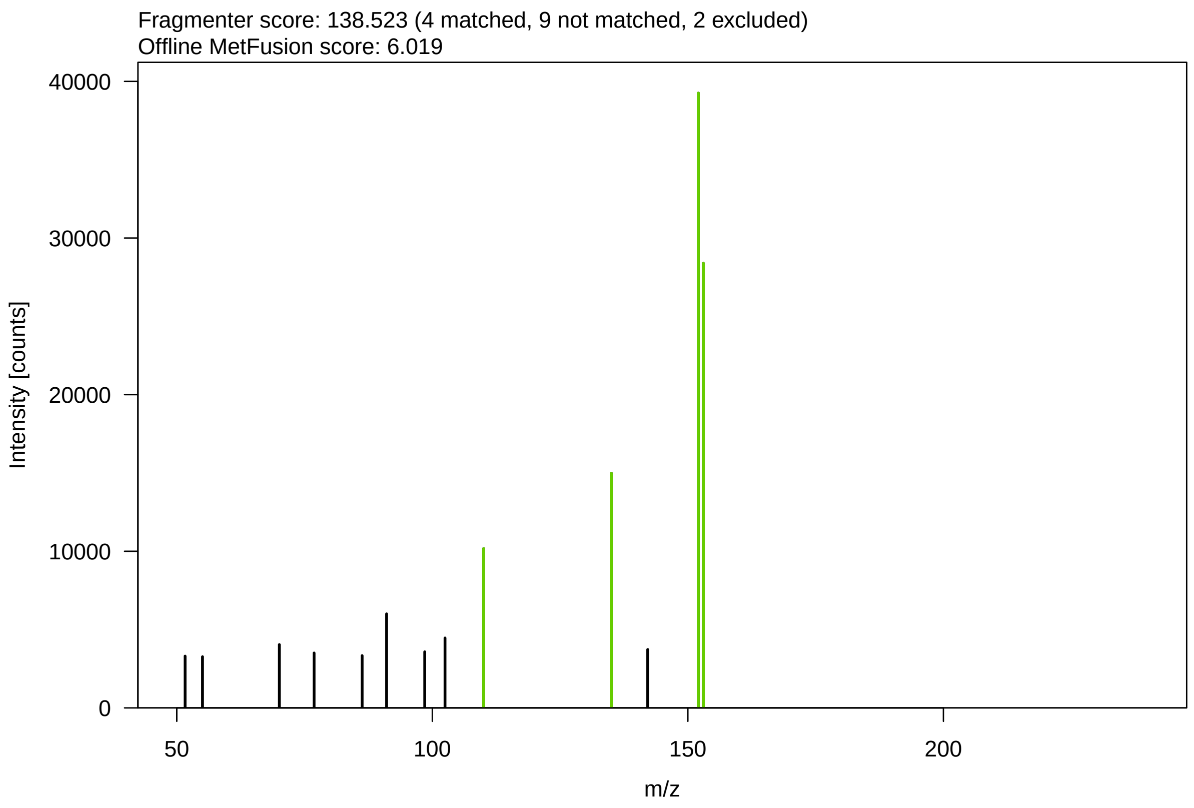

Figure SI-D10: Measured MS2 spectrum. Matching fragments with acyclovir predicted by MetFrag are highlighted in green.

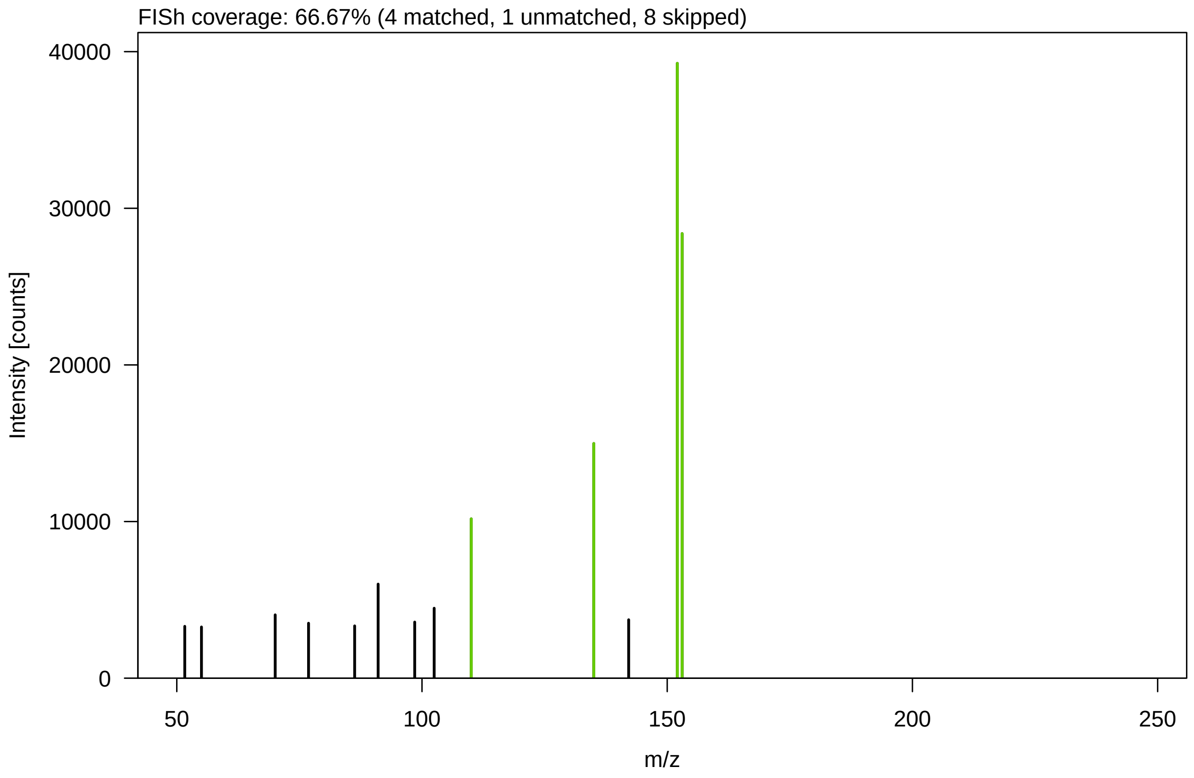

Figure SI-D11: Measured MS2 spectrum. Matching fragments with acyclovir predicted by FISh Scoring are highlighted in green. Low intensity fragments are not considered and skipped.

Table SI-D5: Retention time prediction of acyclovir.

Measured retention time [min]	7.6
Predicted $logD_{OW}$ (pH = 2.7)	-1.55
Predicted retention time [min]	12.7
Predicted retention time range (95% confidence interval) [min]	8.1-17.3
Predicted retention time range (99% confidence interval) [min]	6.7-18.8

Table SI-D6: Annotated MS2 spectrum of acyclovir.

m/z	Relative Intensity	Annotation
51.6257	84.24	
55.0355	83.19	
70.0652	102.89	$C_4H_7N + H^+$
76.8668	89.30	
86.2689	84.95	
91.0540	152.85	$C_7H_6 + H^+$
98.5141	91.18	
102.4832	113.66	
110.0348	258.87	$C_4H_3N_3O + H^+$
135.0298	381.30	$C_5H_2N_4O + H^+$
142.1410	94.90	
152.0568	999.00	$C_5H_5N_5O + H^+$
153.0404	722.39	$C_5H_4N_4O_2 + H^+$

A reference standard of acyclovir was purchased. Figure SI-D12 shows the extracted ion chromatograms of this standard, the sample and the spiked sample, as well as a head to tail plot of the MS2 spectra of the standard and the sample. In addition, the most intense MS2 fragments in the sample and in the standard are displayed. It becomes visible that the retention times of the sample and the spiked sample are identical and the spectra similarity score between sample and standard is equal to 0.749. Three of the most intense fragments of the sample are explained by the reference standard. It can therefore be concluded that the suspected compound is indeed acyclovir. Correspondingly,the identification confidence can be increased to level 1.

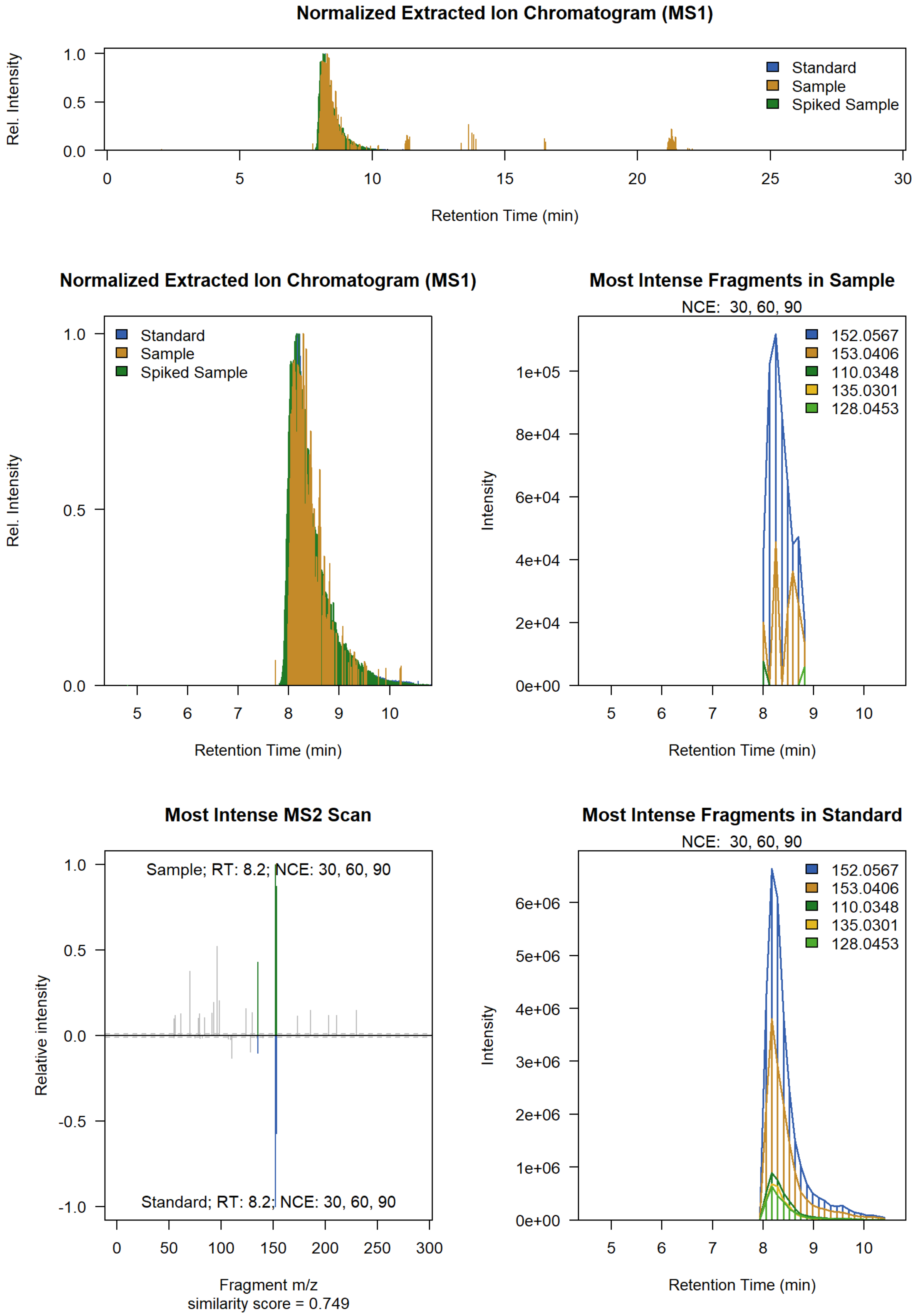

Figure SI-D12: Extracted ion chromatograms of acyclovir in the reference standard, the sample and the spiked sample, as well as MS2 head to tail plot and most intense MS2 fragments in standard and sample.

SI-D1.3 Lamivudine

Lamivudine is an inhibitor of the reverse transcriptase and is used to treat HIV and hepatitis B infections.[2] In Switzerland it is sold under the brand names Zeffix, Kivexa, Trizivir, Triumeq, Dovato, Delstrigo, Combivir and 3TC.[1]

Table SI-D7: Information on identifiers, chemical properties, detection and confidence of identification of lamivudine.

IUPAC Name	4-amino-1-[(2*R*,5*S*)-2-(hydroxymethyl)-1,3-oxathiolan-5-yl]pyrimidin-2-one
Molecular formula	$C_8H_{11}N_3O_3S$
Monoisotopic mass [g/mol]	229.0521
Adduct	$[M+H]^+$
Retention time [min]	7.0
SMILES	C1[C@H](O[C@H](S1)CO)N2C=CC(=NC2=O)N
InChI	InChI=1S/C8H11N3O3S/c9-5-1-2-11(8(13)10-5)6-4-15-7(3-12)14-6/h1-2,6-7,12H,3-4H2,(H2,9,10,13)/t6-,7+/m0/s1
InChI-Key	JTEGQNOMFQHVDC-NKWVEPMBSA-N
CAS RN	134678-17-4
Detection frequency	100% (15/15 samples)
Detected in	Altenrhein, Monday-Friday
	Neugut, Monday-Friday
	Werdhölzli, Monday-Friday
Intensity	E6-E7
Initial confidence level	level 2a
Initial confidence score	0.56
Final confidence level	level 1

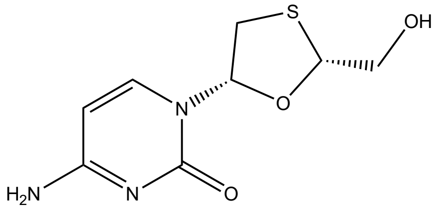

Figure SI-D13: Molecular structure of lamivudine.

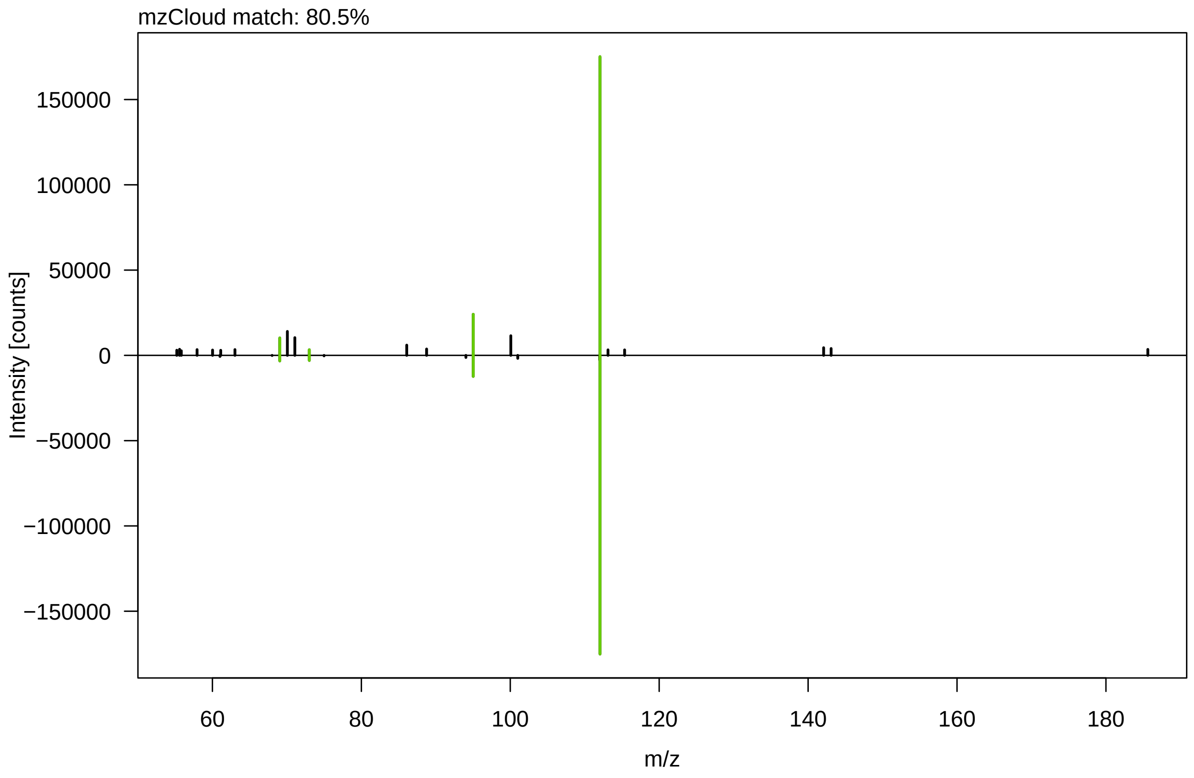

Figure SI-D14: Head to tail plot of measured MS2 spectrum against mzCloud library spectrum of lamivudine. Matching fragments are highlighted in green.

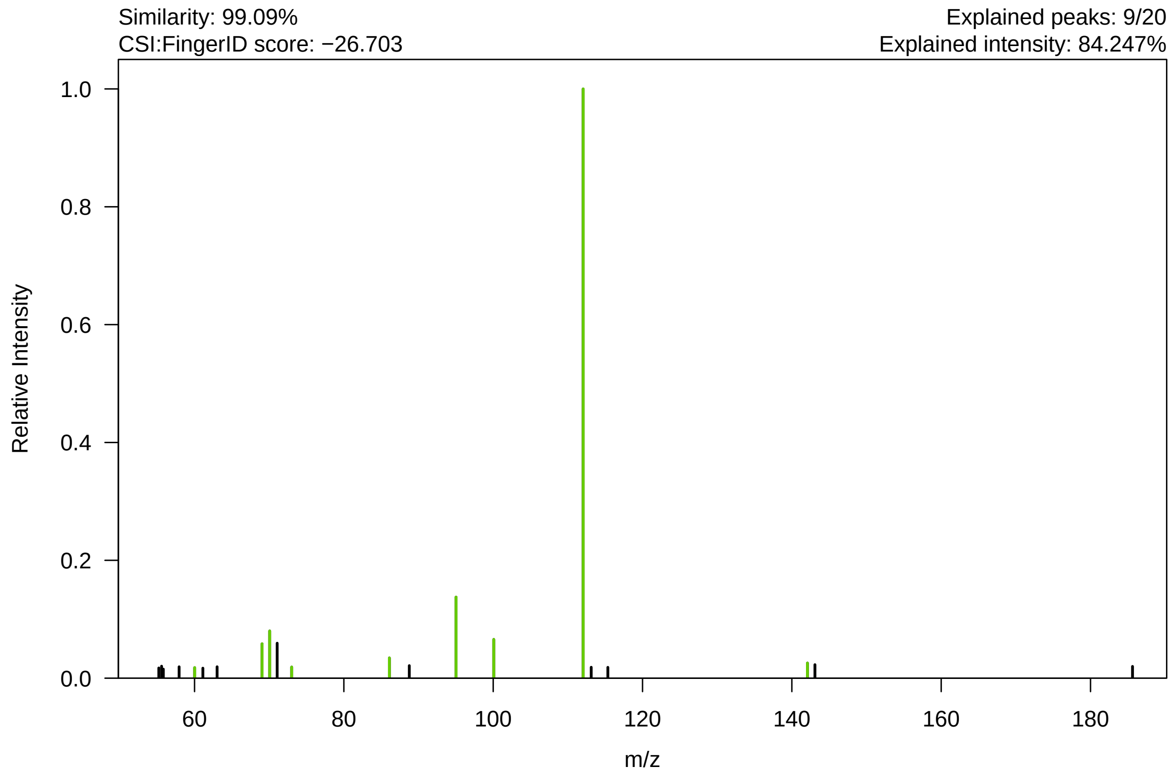

Figure SI-D15: Measured MS2 spectrum. Matching fragments with lamivudine predicted by SIRIUS/CSI:FingerID are highlighted in green.

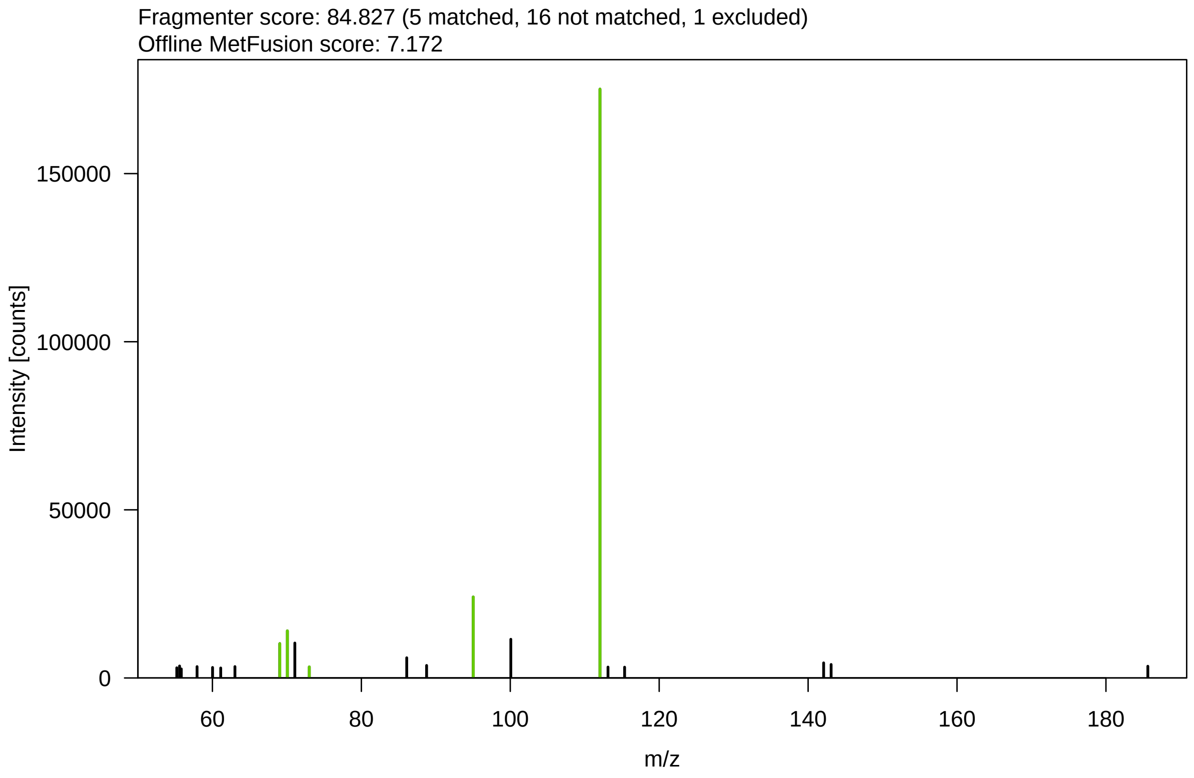

Figure SI-D16: Measured MS2 spectrum. Matching fragments with lamivudine predicted by MetFrag are highlighted in green.

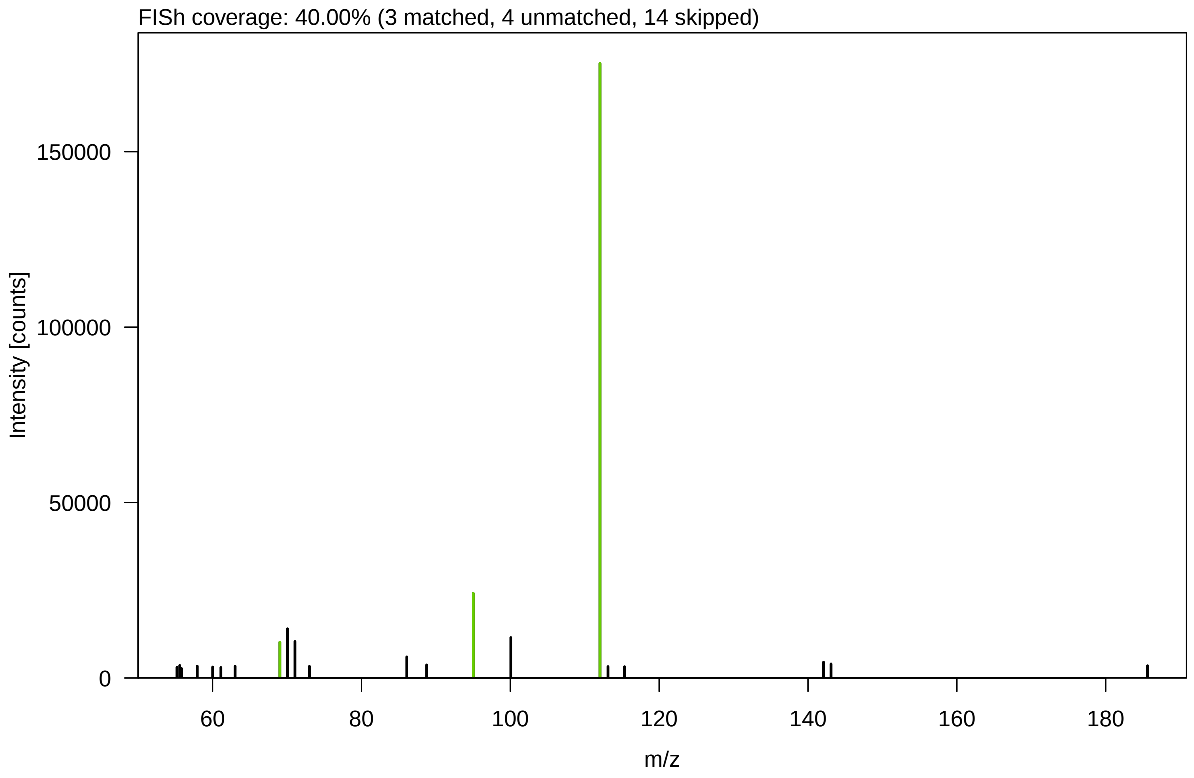

Figure SI-D17: Measured MS2 spectrum. Matching fragments with lamivudine predicted by FISh Scoring are highlighted in green. Low intensity fragments are not considered and skipped.

Table SI-D8: Retention time prediction of lamivudine.

Measured retention time [min]	7.0
Predicted $logD_{OW}$ (pH = 2.7)	-2.55
Predicted retention time [min]	11.4
Predicted retention time range (95% confidence interval) [min]	6.8-16.0
Predicted retention time range (99% confidence interval) [min]	5.4-17.5

Table SI-D9: Annotated MS2 spectrum of lamivudine.

m/z	Relative Intensity	Annotation
55.2124	17.26	
55.5859	20.34	
55.8188	15.37	
57.9360	19.27	
60.0176	17.88	$HN_3O + H^+$
61.1144	16.99	
63.0213	19.33	
69.0447	58.34	$C_3H_4N_2 + H^+$
70.0650	80.03	$C_4H_7N + H^+$
71.0683	59.29	
73.0106	18.86	$C_3H_4S + H^+$
86.0962	34.30	$C_5H_{11}N + H^+$
88.7625	21.26	
95.0238	137.42	$C_4H_2N_2O + H^+$
100.0756	65.66	$C_5H_9NO + H^+$
112.0504	999.00	$C_3H_5N_3O + H^+$
113.1266	18.49	
115.3560	18.32	
142.0854	25.61	$C_7H_{11}NO_2 + H^+$
143.0899	22.89	
185.6250	19.91	

A reference standard of lamivudine was purchased. Figure SI-D18 shows the extracted ion chromatograms of this standard, the sample and the spiked sample, as well as a head to tail plot of the MS2 spectra of the standard and the sample. In addition, the most intense MS2 fragments in the sample and in the standard are displayed. It becomes visible that the retention times of the sample and the spiked sample are identical and the spectra similarity score between sample and standard is equal to 0.987. The most intense fragments of the sample are explained by the reference standard. It can therefore be concluded that the suspected compound is indeed lamivudine. Correspondingly, the identification confidence can be increased to level 1.

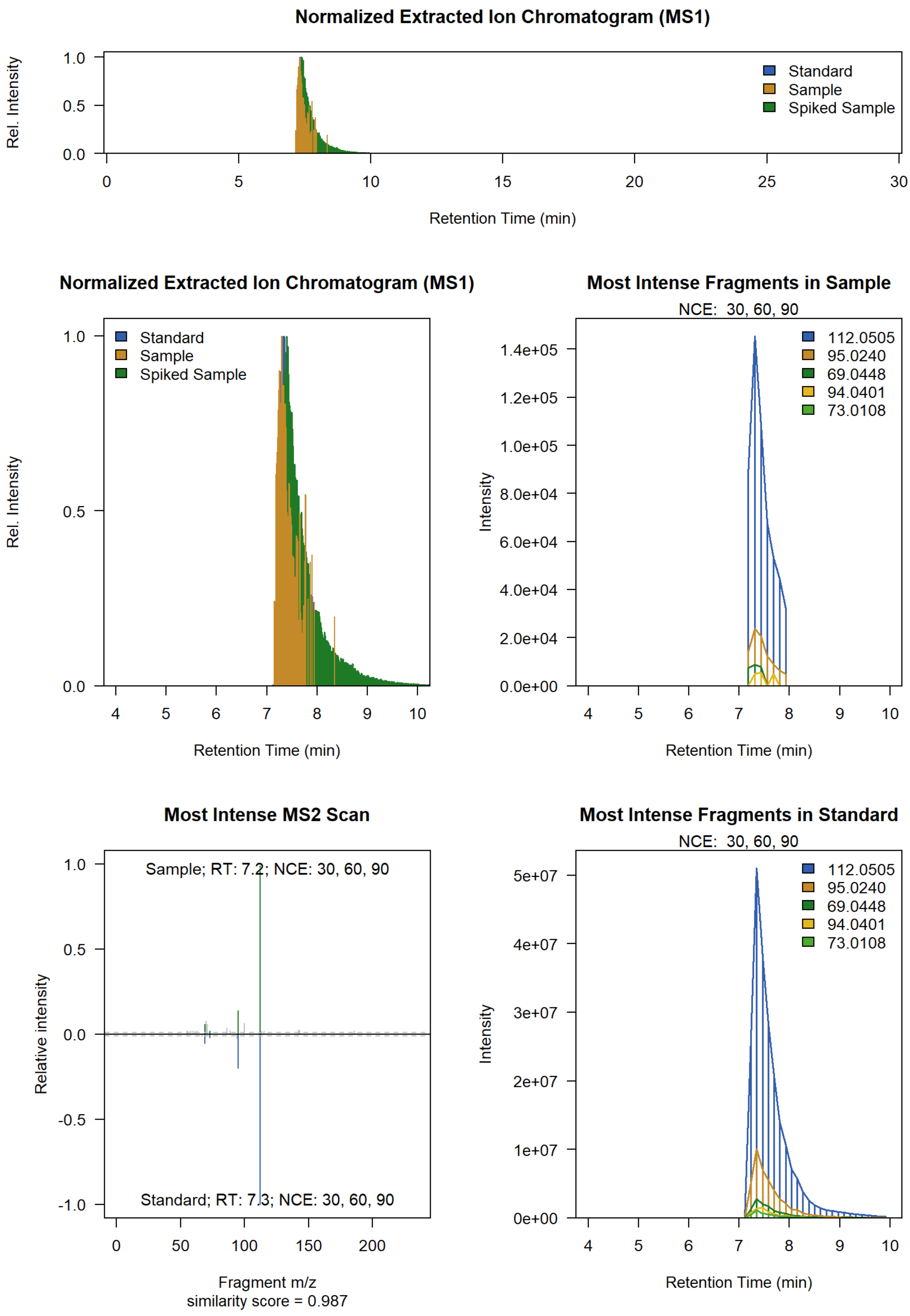

Figure SI-D18: Extracted ion chromatograms of lamivudine in the reference standard, the sample and the spiked sample, as well as MS2 head to tail plot and most intense MS2 fragments in standard and sample.

SI-D1.4 Nevirapine

Nevirapine is a non-nucleoside reverse transcriptase inhibitor, which is used in combination with other antiretrovirals for the treatment of HIV.[2] In Switzerland it is sold under the brand name Viramune.[1]

Table SI-D10: Information on identifiers, chemical properties, detection and confidence of identification of nevirapine.

IUPAC Name	2-cyclopropyl-7-methyl-2,4,9,15-tetrazatricyclo[9.4.0.0^{3,8}]pentadeca-1(11),3,5,7,12,14-hexaen-10-one
Molecular formula	$C_{15}H_{14}N_4O$
Monoisotopic mass [g/mol]	266.1168
Adduct	$[M+H]^+$
Retention time [min]	16.2
SMILES	CC1=C2C(=NC=C1)N(C3=C(C=CC=N3)C(=O)N2)C4CC4
InChI	InChI=1S/C15H14N4O/c1-9-6-8-17-14-12(9)18-15(20)11-3-2-7-16-13(11)19(14)10-4-5-10/h2-3,6-8,10H,4-5H2,1H3,(H,18,20)
InChI-Key	NQDJXKOVJZTUJA-UHFFFAOYSA-N
CAS RN	129618-40-2
Detection frequency	100% (15/15 samples)
Detected in	Altenrhein, Monday-Friday<br>Neugut, Monday-Friday<br>Werdhölzli, Monday-Friday
Intensity	E6-E7
Initial confidence level	level 2a
Initial confidence score	0.46
Final confidence level	level 1

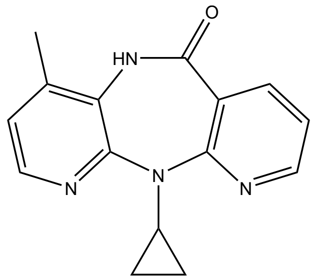

Figure SI-D19: Molecular structure of nevirapine.

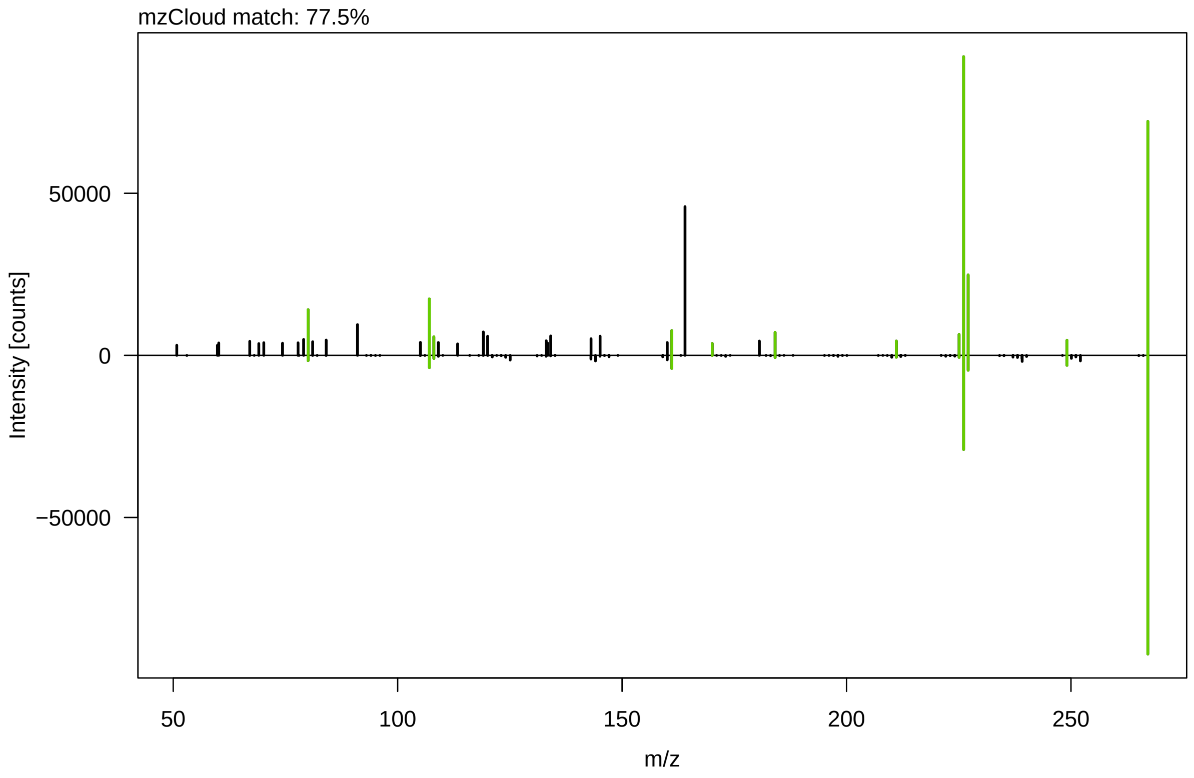

Figure SI-D20: Head to tail plot of measured MS2 spectrum against mzCloud library spectrum of nevirapine. Matching fragments are highlighted in green.

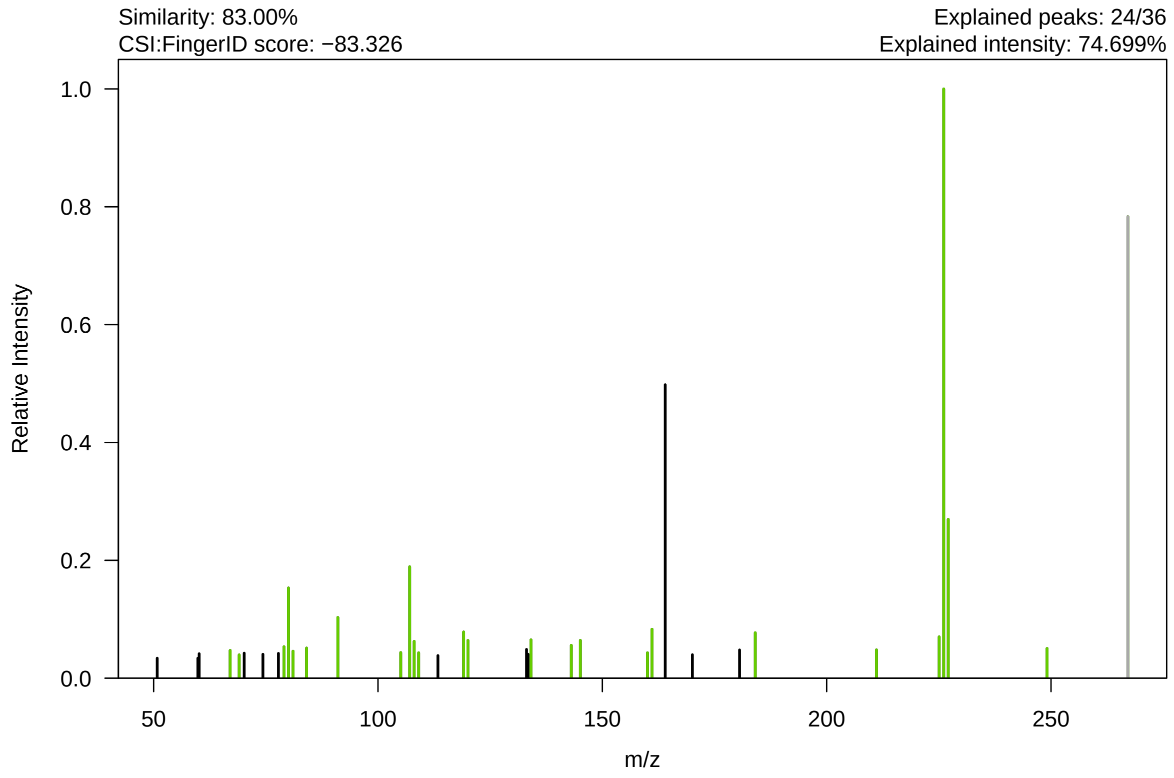

Figure SI-D21: Measured MS2 spectrum. Matching fragments with nevirapine predicted by SIRIUS/CSI:FingerID are highlighted in green. The molecular ion in gray is not considered.

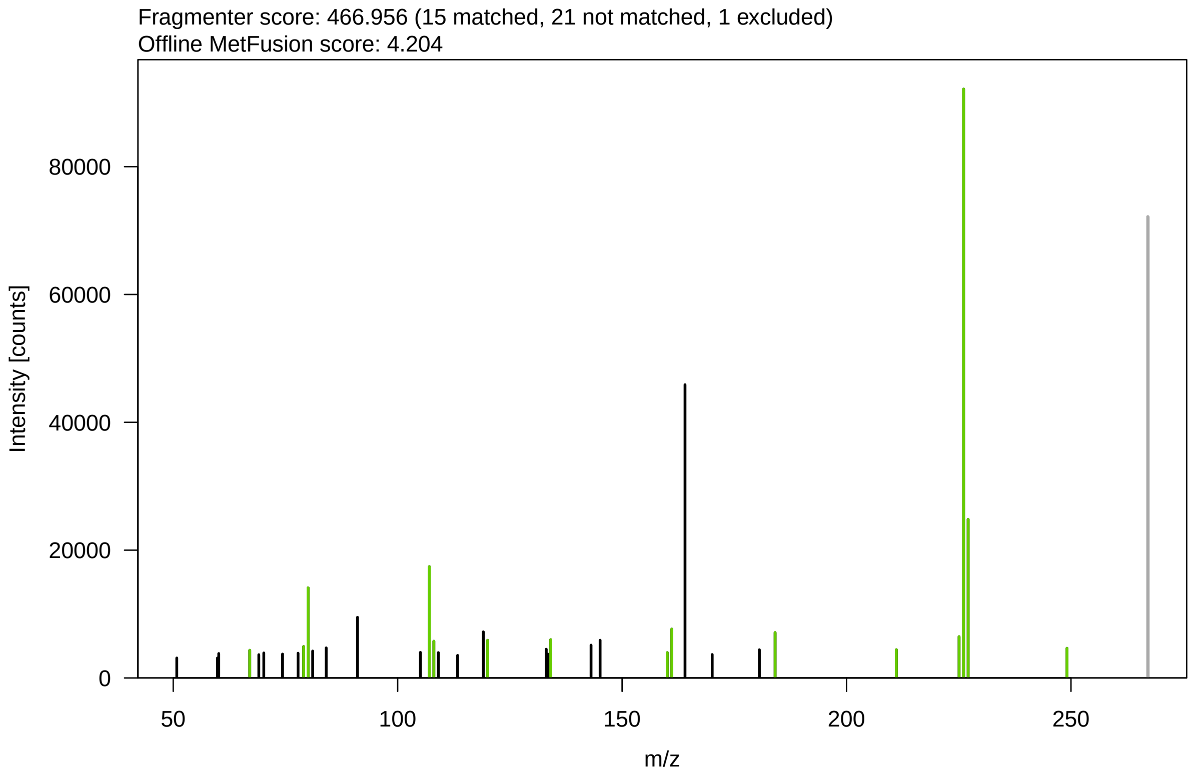

Figure SI-D22: Measured MS2 spectrum. Matching fragments with nevirapine predicted by MetFrag are highlighted in green. The molecular ion in gray is not considered.

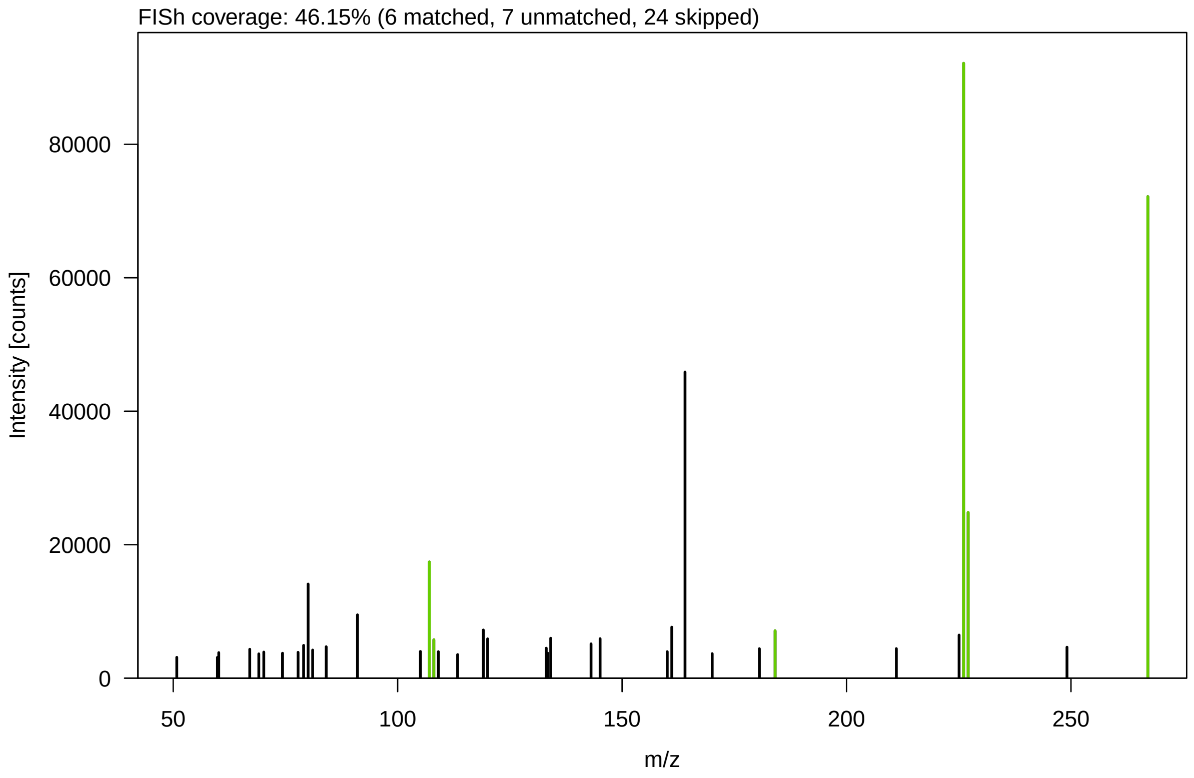

Figure SI-D23: Measured MS2 spectrum. Matching fragments with nevirapine predicted by FISh Scoring are highlighted in green. Low intensity fragments are not considered and skipped.

Table SI-D11: Retention time prediction of nevirapine.

Measured retention time [min]	16.2
Predicted $logD_{OW}$ (pH = 2.7)	1.85
Predicted retention time [min]	17.1
Predicted retention time range (95% confidence interval) [min]	12.5-21.7
Predicted retention time range (99% confidence interval) [min]	11.1-23.2

A reference standard of nevirapine was purchased. Figure SI-D24 shows the extracted ion chromatograms of this standard, the sample and the spiked sample, as well as a head to tail plot of the MS2 spectra of the standard and the sample. In addition, the most intense MS2 fragments in the sample and in the standard are displayed. It becomes visible that the retention times of the sample and the spiked sample are identical and the spectra similarity score between sample and standard is equal to 0.800. The most intense fragments of the sample are explained by the reference standard. It can therefore be concluded that the suspected compound is indeed nevirapine. Correspondingly, the identification confidence can be increased to level 1.

Table SI-D12: Annotated MS2 spectrum of nevirapine.

m/z	Relative Intensity	Annotation
50.7941	33.92	
59.8530	33.87	
60.1474	41.48	
67.0541	47.04	$C_5H_6 + H^+$
69.0698	39.52	
70.1818	42.46	
74.3546	40.57	
77.8106	42.05	
79.0538	53.33	$C_6H_6 + H^+$
80.0492	152.98	$C_5H_5N + H^+$
81.0698	45.74	$C_6H_8 + H^+$
84.0804	51.08	$C_5H_9N + H^+$
91.0538	102.92	$C_7H_6 + H^+$
105.0697	43.41	$C_8H_8 + H^+$
107.0602	188.85	$C_6H_6N_2 + H^+$
108.0676	62.38	$C_6H_7N_2 + H^+$
109.0653	43.04	$C_7H_8O + H^+$
113.3596	38.31	
119.0853	78.33	$C_9H_{10} + H^+$
120.0444	63.92	$C_7H_5NO + H^+$
133.1018	48.80	
133.4317	40.70	
134.0962	64.95	$C_9H_{11}N + H^+$
143.0854	55.77	$C_{11}H_{10} + H^+$
145.1003	64.10	$C_{11}H_{12} + H^+$
160.0627	43.04	$C_9H_7N_2O + H^+$
161.0705	82.96	$C_9H_8N_2O + H^+$
164.0163	497.55	
170.0750	39.78	
180.5843	47.88	
184.0874	77.03	$C_{11}H_9N_3 + H^+$
211.0971	47.99	$C_{12}H_{10}N_4 + H^+$
225.0754	70.09	$C_{12}H_8N_4O + H^+$
226.0845	999.00	$C_{12}H_9N_4O + H^+$
227.0927	269.18	$C_{12}H_{10}N_4O + H^+$
249.1132	50.40	$C_{15}H_{12}N_4 + H^+$
267.1238	782.45	$C_{15}H_{14}N_4O + H^+$

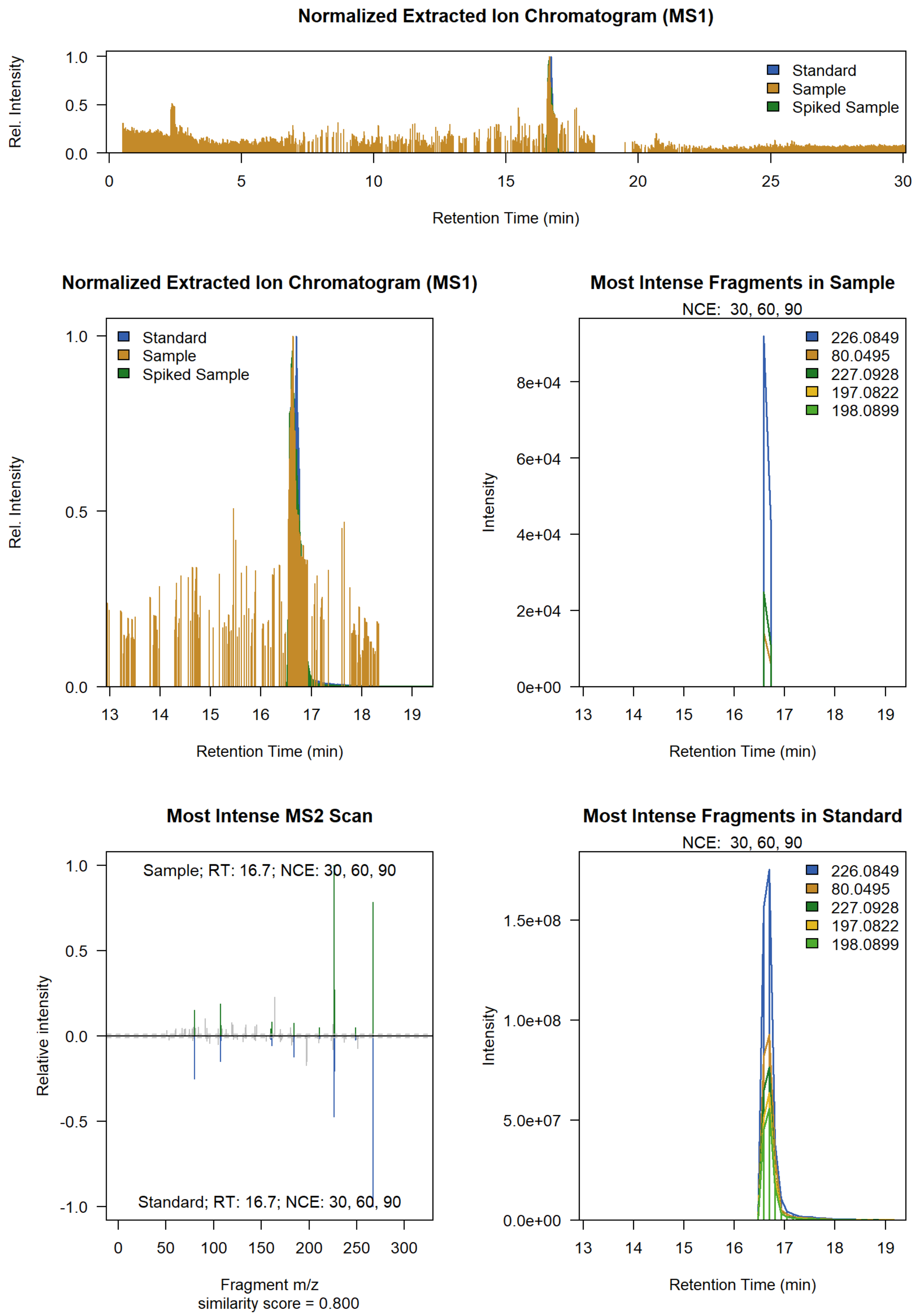

Figure SI-D24: Extracted ion chromatograms of nevirapine in the reference standard, the sample and the spiked sample, as well as MS2 head to tail plot and most intense MS2 fragments in standard and sample.

SI-D1.5 Pentoxifylline

Pentoxifylline is a synthetic dimethylxanthine derivative. It modulates the rheological properties of blood and has anti-oxidant and anti-inflammatory properties. It is used for the treatment of intermittent claudication for patients with peripheral arterial disease.[2]

Table SI-D13: Information on identifiers, chemical properties, detection and confidence of identification of pentoxifylline.

IUPAC Name	3,7-dimethyl-1-(5-oxohexyl)purine-2,6-dione
Molecular formula	$C_{13}H_{18}N_4O_3$
Monoisotopic mass [g/mol]	278.1379
Adduct	$[M+H]^+$
Retention time [min]	15.5
SMILES	CC(=O)CCCCN1C(=O)C2=C(N=CN2C)N(C1=O)C
InChI	InChI=1S/C13H18N4O3/c1-9(18)6-4-5-7-17-12(19)10-11(14-8-15(10)2)16(3)13(17)20/h8H,4-7H2,1-3H3
InChI-Key	BYPFEZZEUUWMEJ-UHFFFAOYSA-N
CAS RN	6493-05-6
Detection frequency	100% (15/15 samples)
Detected in	Altenrhein, Monday-Friday
	Neugut, Monday-Friday
	Werdhölzli, Monday-Friday
Intensity	E6-E7
Initial confidence level	level 2a
Initial confidecne score	0.50
Final confidence level	level 1

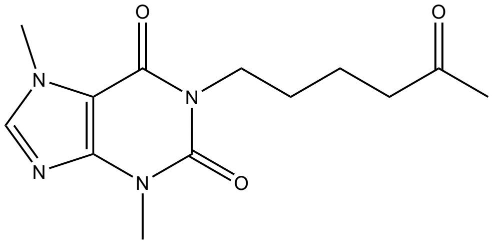

Figure SI-D25: Molecular structure of pentoxifylline.

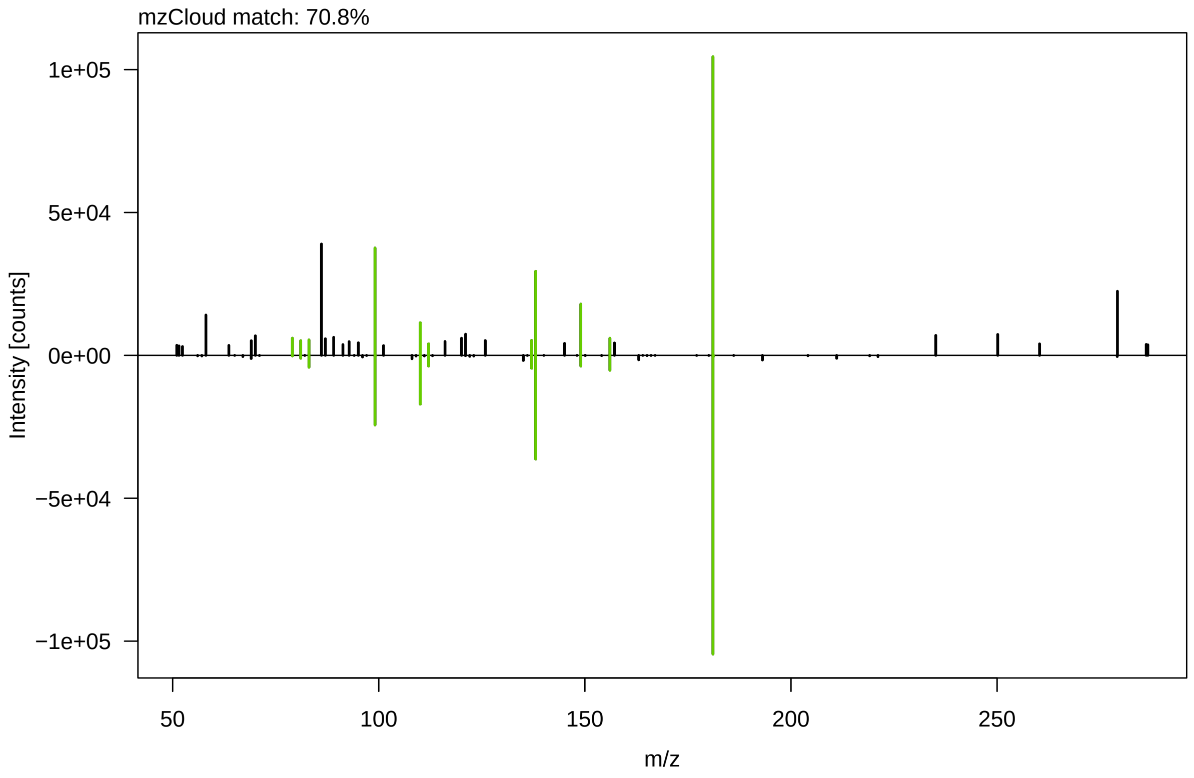

Figure SI-D26: Head to tail plot of measured MS2 spectrum against mzCloud library spectrum of pentoxifylline. Matching fragments are highlighted in green.

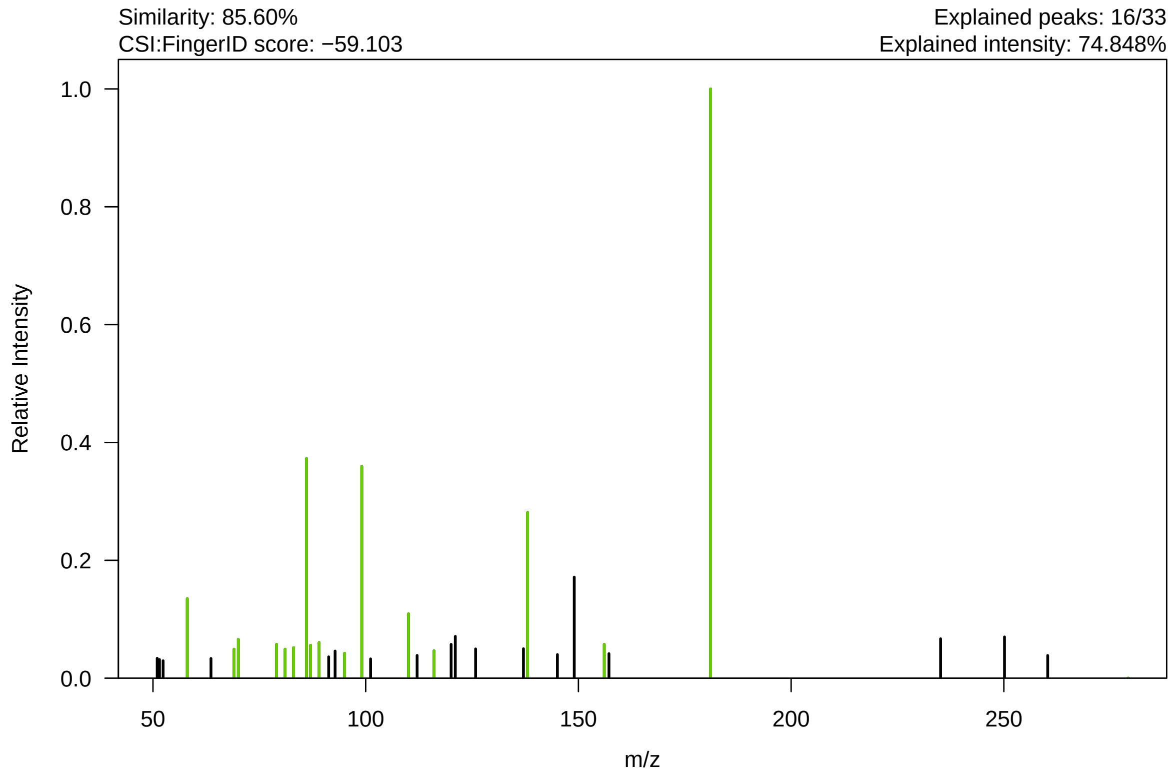

Figure SI-D27: Measured MS2 spectrum. Matching fragments with pentoxifylline predicted by SIRIUS/CSI:FingerID are highlighted in green.

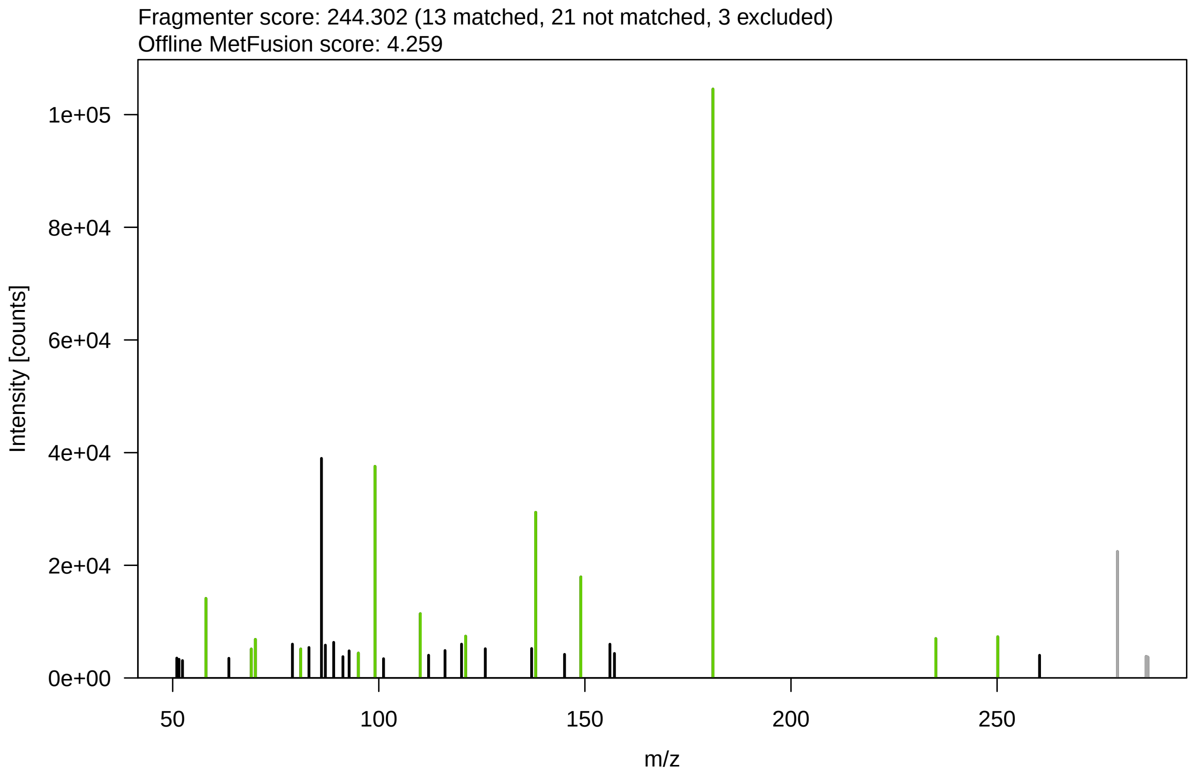

Figure SI-D28: Measured MS2 spectrum. Matching fragments with pentoxifylline predicted by MetFrag are highlighted in green. Fragments in gray are not considered.

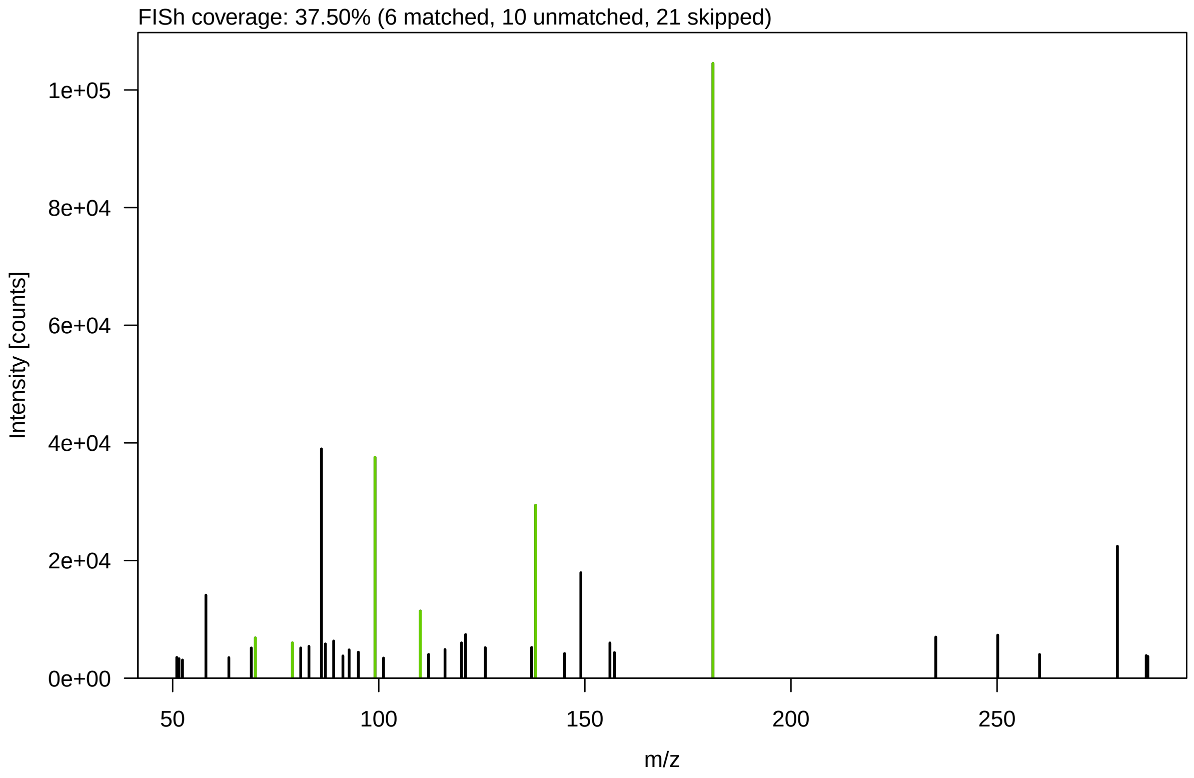

Figure SI-D29: Measured MS2 spectrum. Matching fragments with pentoxifylline predicted by FISh Scoring are highlighted in green. Low intensity fragments are not considered and skipped.

Table SI-D14: Retention time prediction of pentoxifylline.

Measured retention time [min]	15.5
Predicted $logD_{OW}$ (pH = 2.7)	0.23
Predicted retention time [min]	15.0
Predicted retention time range (95% confidence interval) [min]	10.4-19.6
Predicted retention time range (99% confidence interval) [min]	9.0-21.1

Table SI-D15: Annotated MS2 spectrum of pentoxifylline.

m/z	Relative Intensity	Annotation
50.9963	33.72	
51.5288	31.67	
52.3821	29.50	
58.0652	134.94	$C_3H_7N + H^+$
63.6312	33.37	
69.0699	49.10	$C_5H_8 + H^+$
70.0651	65.52	$C_4H_7N + H^+$
79.0541	57.38	$C_6H_6 + H^+$
81.0698	49.15	$C_6H_8 + H^+$
83.0601	51.70	$C_4H_6N_2 + H^+$
86.0964	372.52	$C_5H_{11}N + H^+$
87.0440	55.70	$C_4H_6O_2 + H^+$
89.0594	60.42	$C_4H_8O_2 + H^+$
91.3012	36.16	
92.8076	46.02	
95.0485	42.23	$C_4H_4N_3 + H^+$
99.0805	359.06	$C_6H_{10}O + H^+$
101.1539	32.68	
110.0714	109.08	$C_5H_7N_3 + H^+$
112.0861	38.56	
116.0708	46.55	$C_5H_9NO_2 + H^+$
120.0803	57.40	
121.0646	70.89	$C_6H_6N_3 + H^+$
125.8393	49.61	
137.0814	49.94	
138.0662	281.02	$C_6H_7N_3O + H^+$
145.0653	40.03	
149.0230	171.36	$C_6H_2N_3O_2 + H^+$
156.0767	57.28	$C_6H_9N_3O_2 + H^+$

Continued on next page

Table SI-D15: Annotated MS2 spectrum of pentoxifylline.(Continued)

157.1772	41.59	
181.0718	999.00	$C_7H_8N_4O_2 + H^+$
235.1303	66.80	$C_{12}H_{16}N_3O_2 + H^+$
250.1531	69.97	$C_{13}H_{19}N_3O_2 + H^+$
260.3033	38.52	
279.1936	214.39	
286.1785	36.67	
286.5694	35.32	

A reference standard of pentoxifylline was purchased. Figure SI-D30 shows the extracted ion chromatograms of this standard, the sample and the spiked sample, as well as a head to tail plot of the MS2 spectra of the standard and the sample. In addition, the most intense MS2 fragments in the sample and in the standard are displayed. It becomes visible that the retention times of the sample and the spiked sample are identical and the spectra similarity score between sample and standard is equal to 0.866. The most intense fragments of the sample are explained by the reference standard. It can therefore be concluded that the suspected compound is indeed pentoxifylline. Correspondingly, the identification confidence can be increased to level 1.

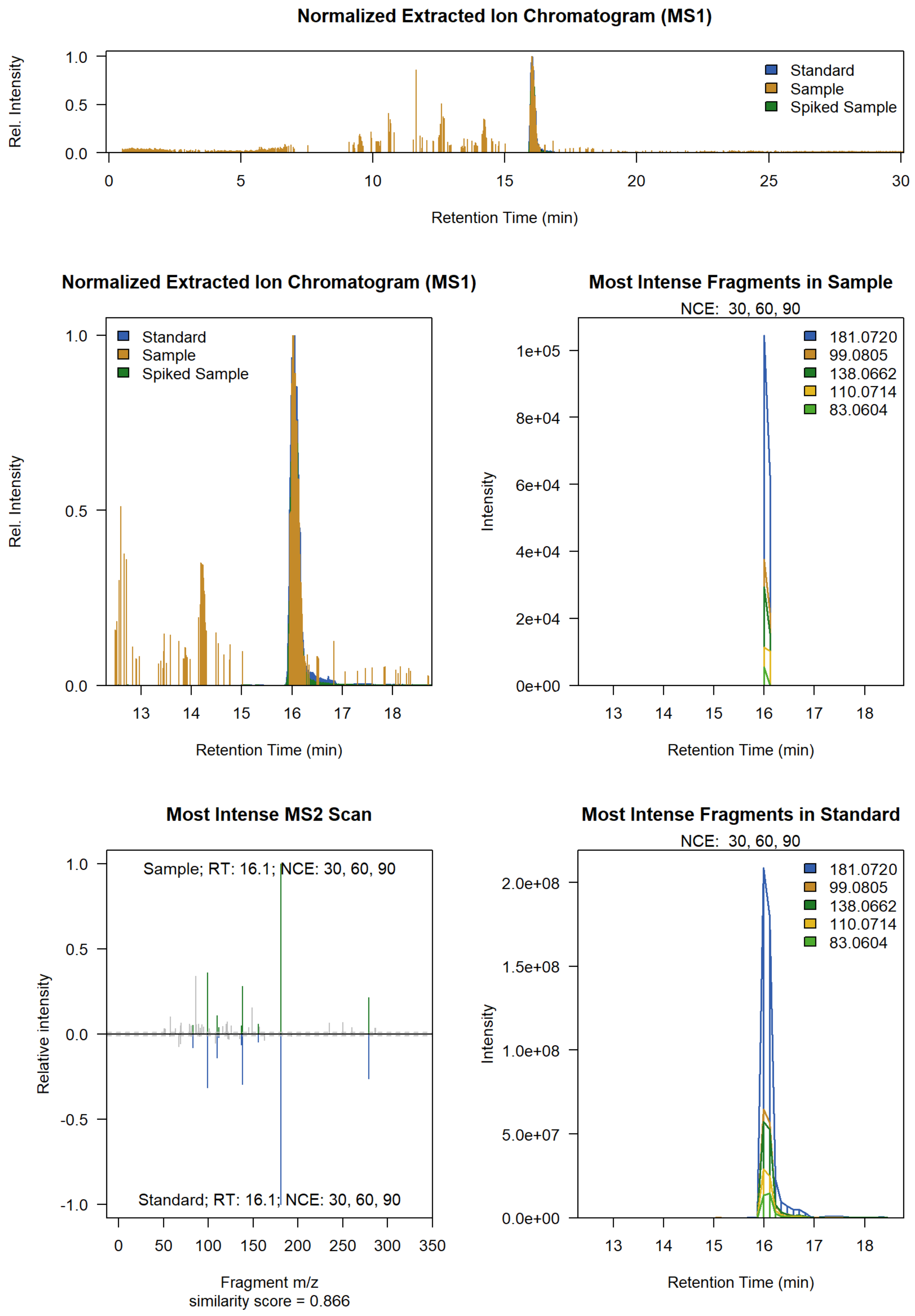

Figure SI-D30: Extracted ion chromatograms of pentoxifylline in the reference standard, the sample and the spiked sample, as well as MS2 head to tail plot and most intense MS2 fragments in standard and sample.

SI-D1.6 Quinine

Quinine is an alkaloid, used to treat malaria.[2] The diastereomeric compound quinidine, a class IA antiarrhythmic used to treat cardiac arrhythmia, is no longer commercially available in Switzerland.[3] As a consequence, it is much more likely that the suspect corresponds to quinine, rather than quinidine.

Table SI-D16: Information on identifiers, chemical properties, detection and confidence of identification of quinine.

IUPAC name	(*R*)-[(2*S*,4*S*,5*R*)-5-ethenyl-1-azabicyclo[2.2.2]octan-2-yl]-(6-methoxyquinolin-4-yl)methanol
Molecular formula	$C_{20}H_{24}N_2O_2$
Monoisotopic mass [g/mol]	324.1838
Adduct	$[M+H]^+$
Retention time [min]	13.4
SMILES	COC1=CC2=C(C=CN=C2C=C1)[C@H]([C@@H]3C[C@@H]4CCN3C[C@@H]4C=C)O
InChI	InChI=1S/C20H24N2O2/c1-3-13-12-22-9-7-14(13)10-19(22)20(23)16-6-8-21-18-5-4-15(24-2)11-17(16)18/h3-6,8,11,13-14,19-20,23H,1,7,9-10,12H2,2H3/t13-,14-,19-,20+/m0/s1
InChI-Key	LOUPRKONTZGTKE-WZBLMQSHSA-N
CAS RN	130-95-0
Detection frequency	100% (15/15 samples)
Detected in	Altenrhein, Monday-Friday
	Neugut, Monday-Friday
	Werdhölzli, Monday-Friday
Intensity	E7-E9
Initial confidence level	level 2a
Initial confidence score	0.50
Final confidence level	level 1

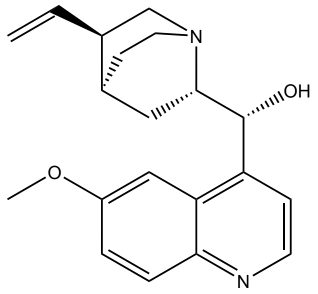

Figure SI-D31: Molecular structure of quinine.

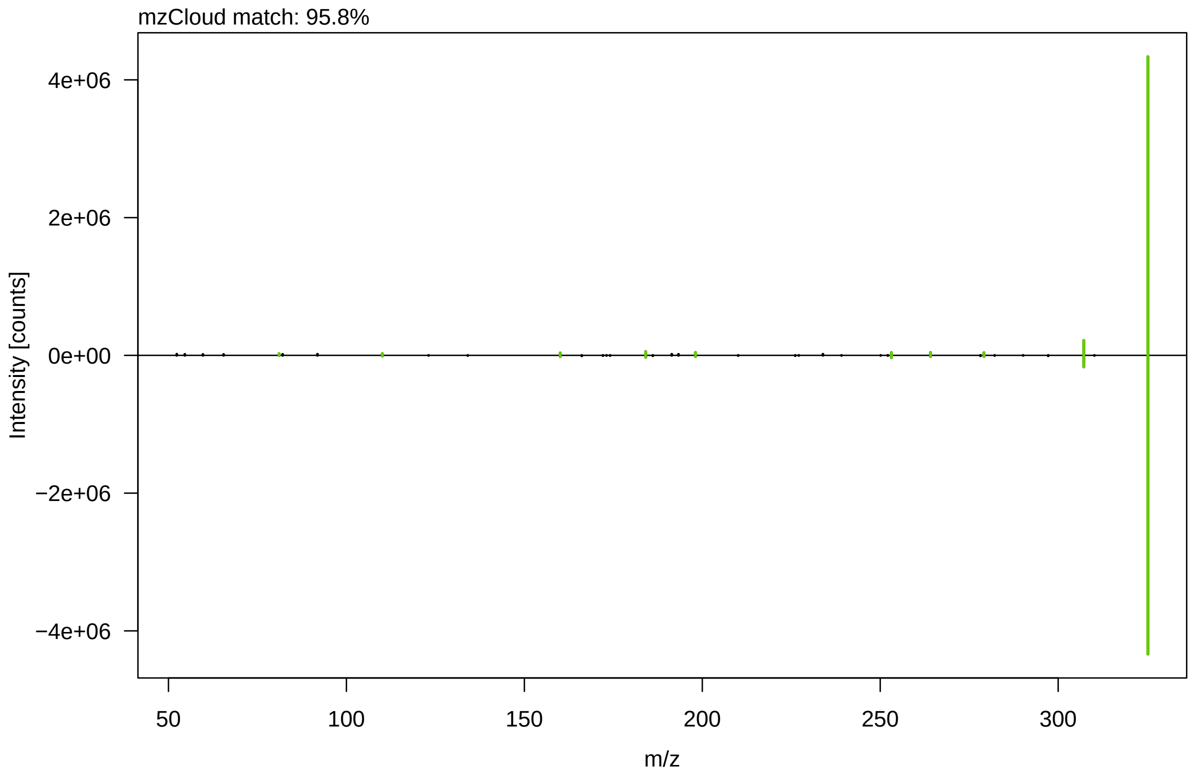

Figure SI-D32: Head to tail plot of measured MS2 spectrum against mzCloud library spectrum of quinine/quinidine. Matching fragments are highlighted in green.

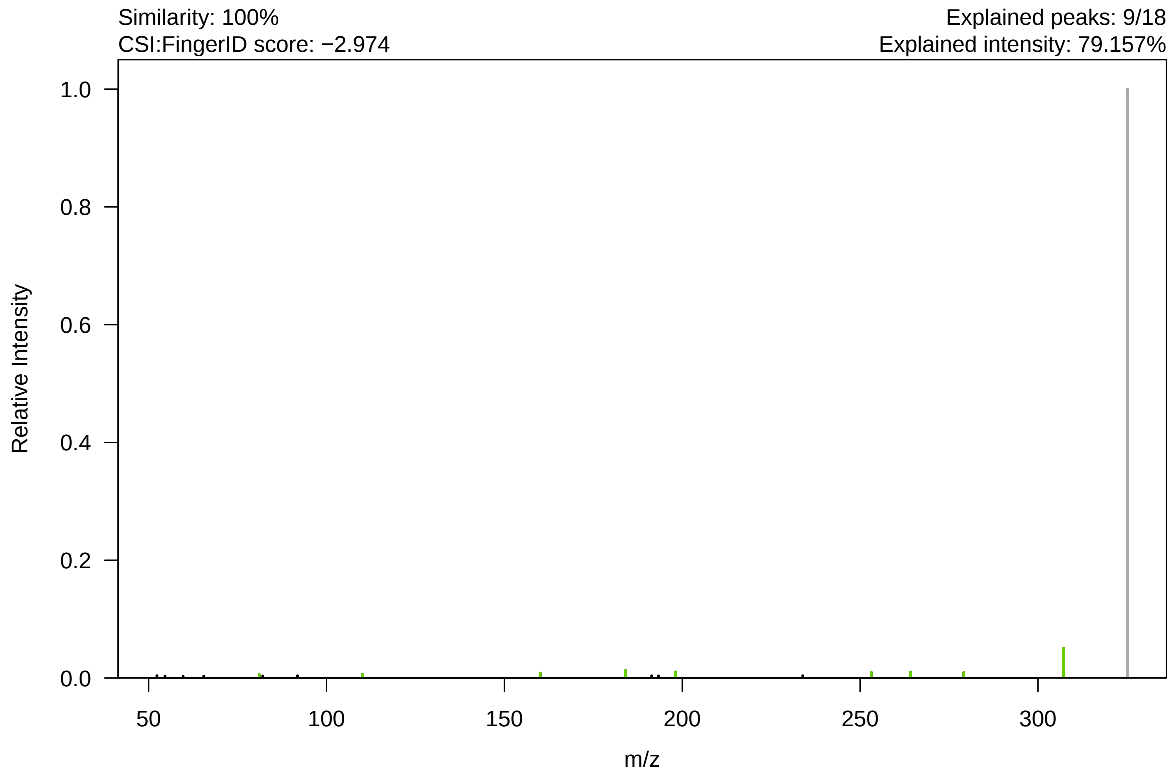

Figure SI-D33: Measured MS2 spectrum. Matching fragments with quinine/quinidine predicted by SIRIUS/CSI:FingerID are highlighted in green. The molecular ion in gray is not considered.

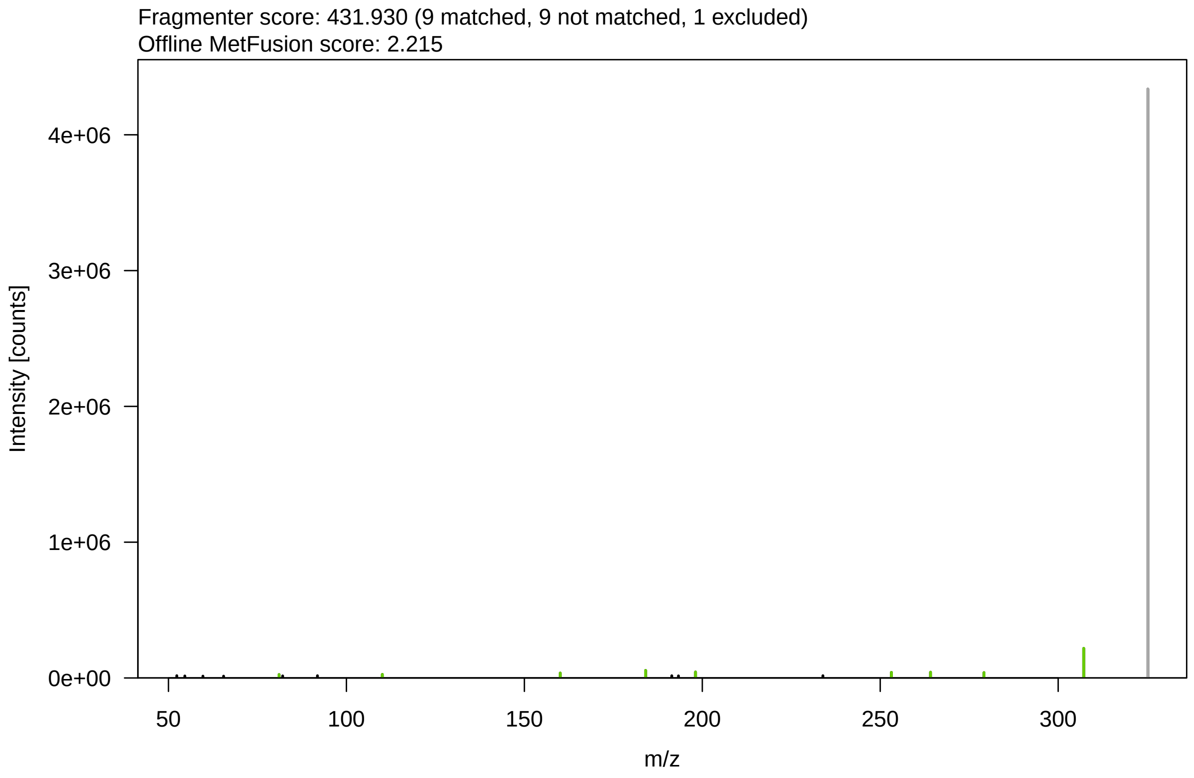

Figure SI-D34: Measured MS2 spectrum. Matching fragments with quinine/quinidine predicted by MetFrag are highlighted in green. The molecular ion in gray is not considered.

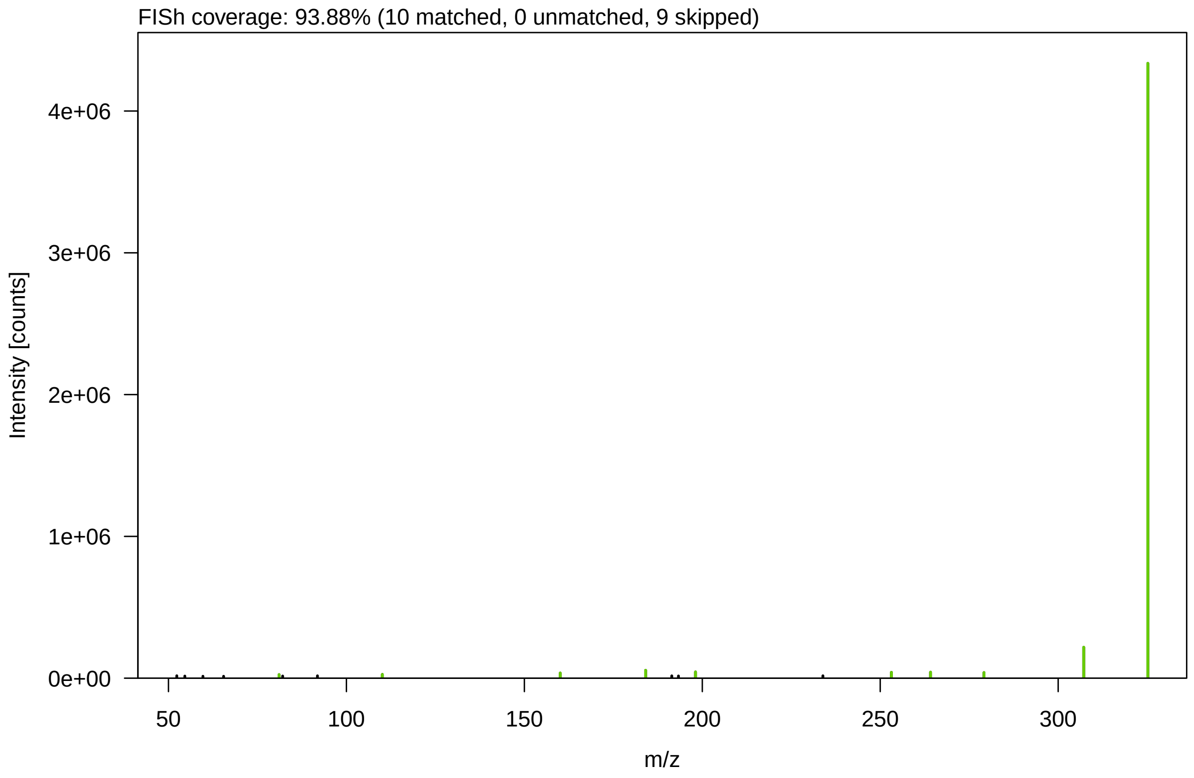

Figure SI-D35: Measured MS2 spectrum. Matching fragments with quinine/quinidine predicted by FISh Scoring are highlighted in green. Low intensity fragments are not considered and skipped.

Table SI-D17: Retention time prediction of quinine.

Measured retention time [min]	13.4
Predicted $logD_{OW}$ (pH = 2.7)	-2.97
Predicted retention time [min]	10.9
Predicted retention time range (95% confidence interval) [min]	6.3-15.5
Predicted retention time range (99% confidence interval) [min]	4.8-16.9

Table SI-D18: Annotated MS2 spectrum of quinine.

m/z	Relative Intensity	Annotation
52.3329	3.91	
54.5945	3.42	
59.6765	3.12	
65.4874	3.10	
81.0697	5.84	$C_6H_8 + H^+$
82.0822	3.50	
91.8583	3.83	
110.0963	6.12	$C_7H_{11}N + H^+$
160.0756	8.37	$C_{10}H_9NO + H^+$
184.0757	12.86	$C_{12}H_9NO + H^+$
191.4207	3.81	
193.3032	3.59	
198.0911	10.20	$C_{13}H_{11}NO + H^+$
233.8879	3.87	
253.1339	9.50	$C_{16}H_{16}N_2O + H^+$
264.1389	9.78	$C_{18}H_{17}NO + H^+$
279.1495	9.13	$C_{18}H_{18}N_2O + H^+$
307.1809	50.32	$C_{20}H_{22}N_2O + H^+$
325.1910	999.00	$C_{20}H_{24}N_2O_2 + H^+$

A reference standard of quinine was purchased. Figure SI-D36 shows the extracted ion chromatograms of this standard, the sample and the spiked sample, as well as a head to tail plot of the MS2 spectra of the standard and the sample. In addition, the most intense MS2 fragments in the sample and in the standard are displayed. It becomes visible that the retention times of the sample and the spiked sample are identical and the spectra similarity score between sample and standard is equal to 0.991. The vast majority of the sample fragments are explained by the reference standard. It can therefore be concluded that the suspected compound is indeed quinine. Correspondingly, the identification confidence can be increased to level 1.

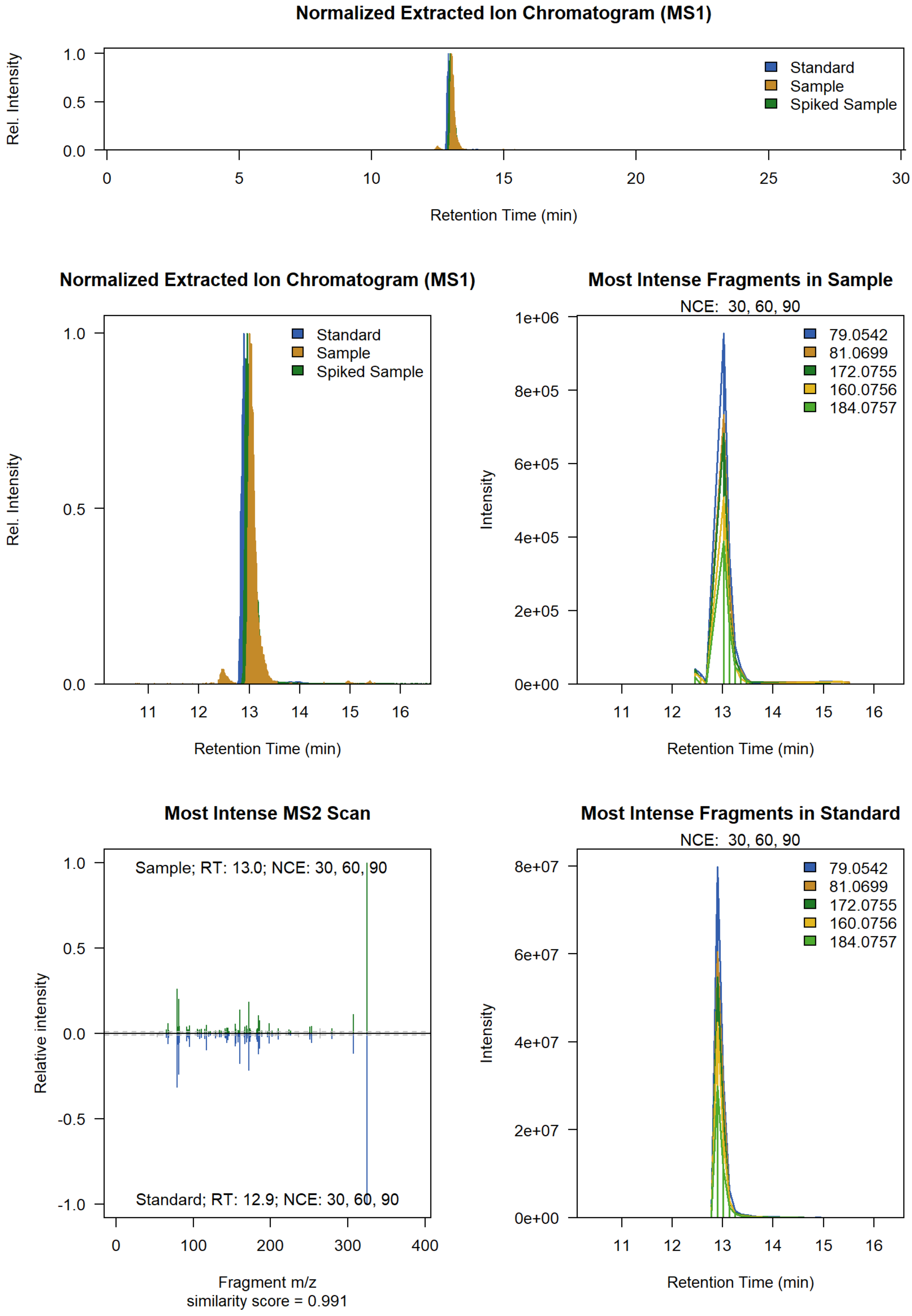

Figure SI-D36: Extracted ion chromatograms of quinine in the reference standard, the sample and the spiked sample, as well as MS2 head to tail plot and most intense MS2 fragments in standard and sample.

SI-D2 Human Metabolites of Prioritized Pharmaceuticals

SI-D2.1 Abacavir Metabolites

Abacavir is an antiviral nucleoside reverse transcriptase inhibitor. It is used in combination with other antiretrovirals for the treatment of HIV.[2,4] Besides the identification of the parent abacavir, two metabolites were found by suspect screening. The identified parent and metabolites are highlighted in the metabolism scheme of abacavir in Figure SI-D37. An excerpt of the molecular network of abacavir and one of its human metabolites, abacavir 5'-carboxylate, is shown in Figure SI-D38. The following subsections give more details on the identification of the individual metabolites.

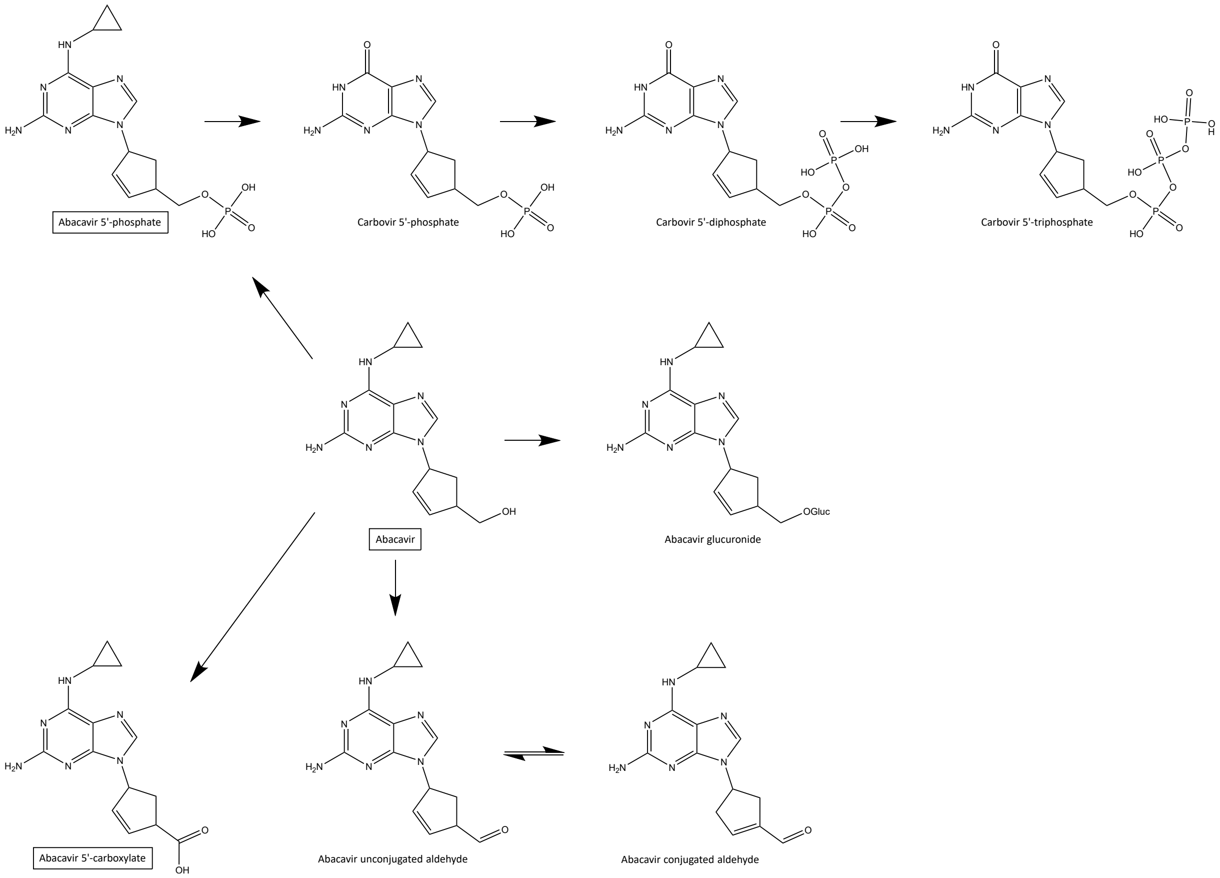

Figure SI-D37: Human metabolism of abacavir. Framed parent and metabolites were identified during suspect screening. Scheme adapted from.[4]

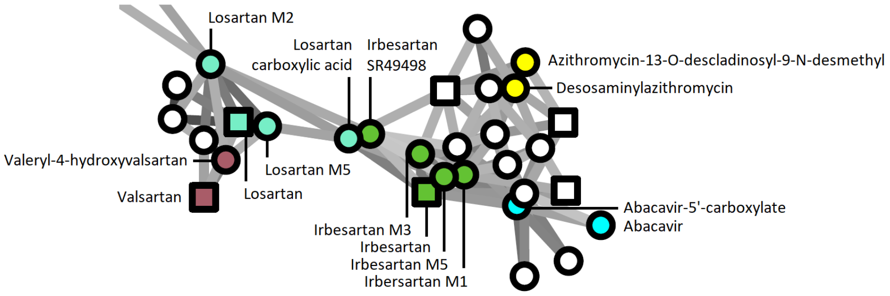

Figure SI-D38: Excerpt of the molecular network showing the abacavir cluster.

SI-D2.1.1 Abacavir-5'-Carboxylate

Table SI-D19: Information on identifiers, chemical properties, detection and confidence of identification of abacavir-5'-carboxylate.

IUPAC Name	(1*S*,4*R*)-4-[2-amino-6-(cyclopropylamino)purin-9-yl]cyclopent-2-ene-1-carboxylic acid
Molecular formula	$C_{14}H_{16}N_6O_2$
Monoisotopic mass [g/mol]	300.1335
Adduct	$[M+H]^+$
Retention time [min]	12.0
SMILES	C1CC1NC2=C3C(=NC(=N2)N)N(C=N3)[C@@H]4C[C@@H](C=C4)C(=O)O
InChI	InChI=1S/C14H16N6O2/c15-14-18-11(17-8-2-3-8)10-12(19-14)20(6-16-10)9-4-1-7(5-9)13(21)22/h1,4,6-9H,2-3,5H2,(H,21,22)(H3,15,17,18,19)/t7-,9+/m1/s1
InChI-Key	OCSMNHMMTKMVCP-APPZFPTMSA-N
CAS RN	384380-52-3
Metabolite of	Abacavir
Detection frequency	100% (15/15 samples)
Detected in	Altenrhein, Monday-Friday
	Neugut, Monday-Friday
	Werdhölzli, Monday-Friday
Intensity	E7-E8
Initial confidence level	level 3
Initial confidence score	0.47
Final confidence level	level 1

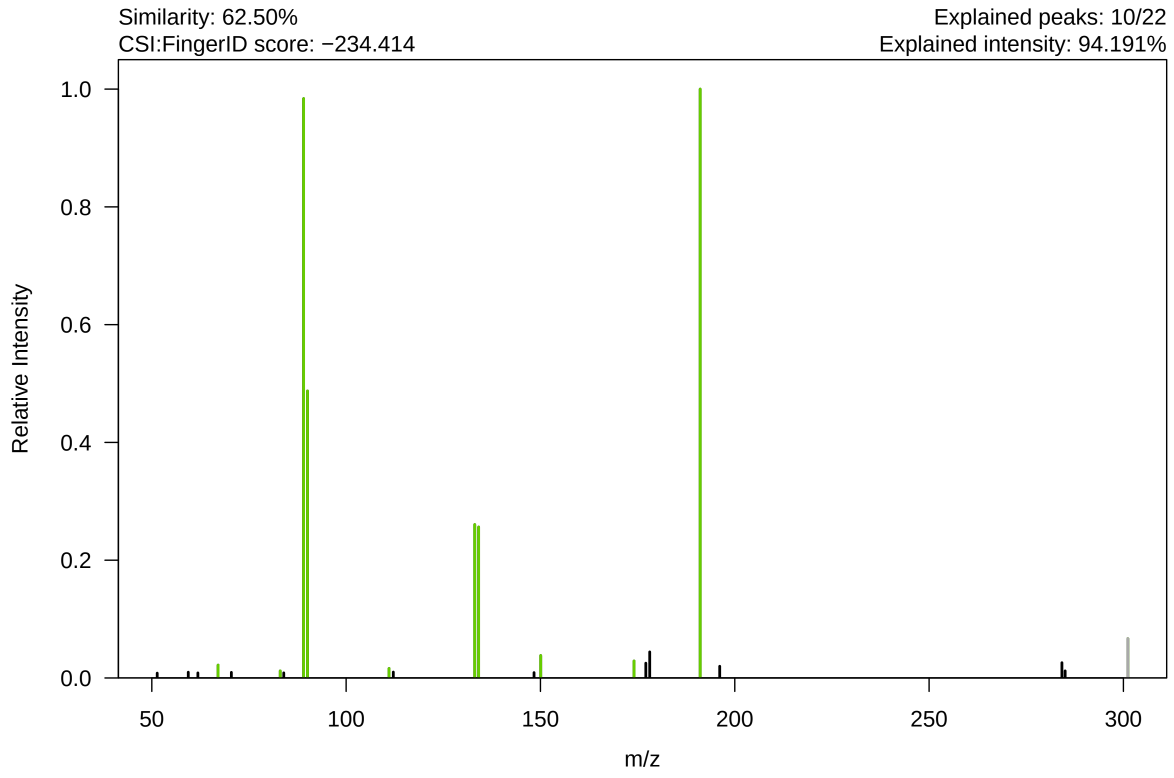

Figure SI-D39: Measured MS2 spectrum. Matching fragments with abacavir-5'-carboxylate predicted by SIRIUS/CSI:FingerID are highlighted in green. The molecular ion in gray is not considered.

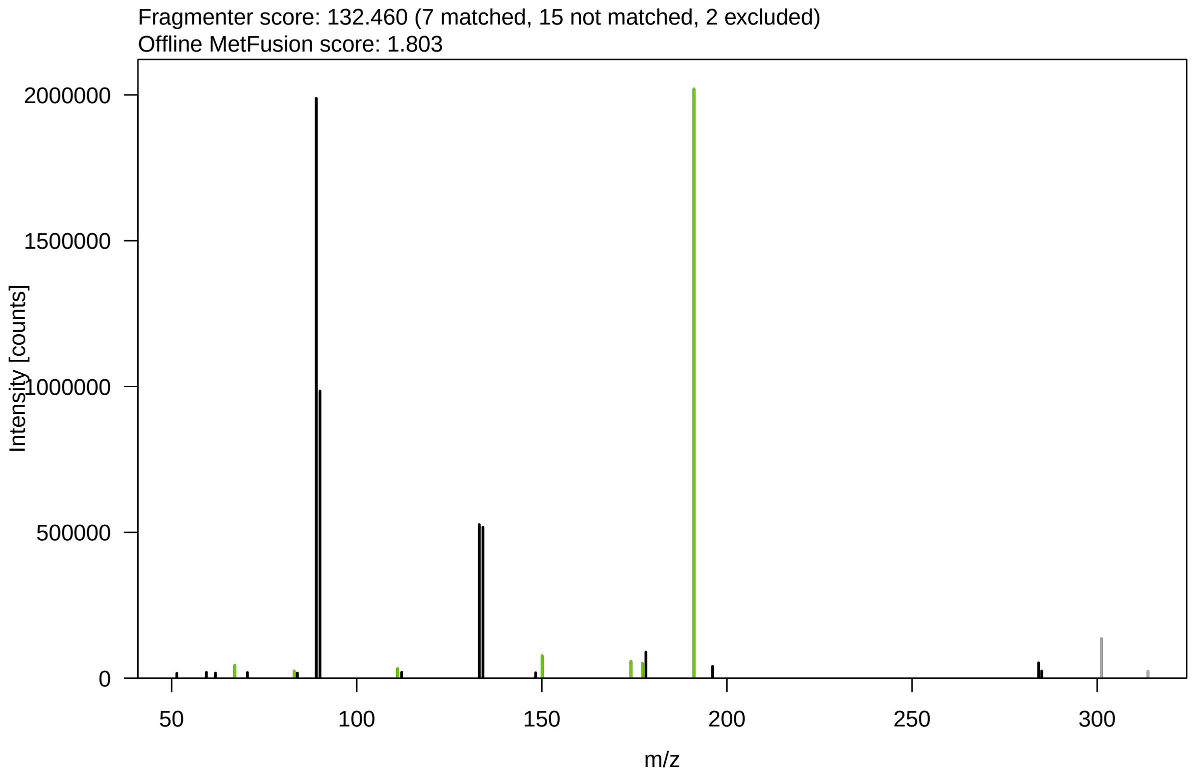

Figure SI-D40: Measured MS2 spectrum. Matching fragments with abacavir-5'-carboxylate predicted by MetFrag are highlighted in green. The molecular ion in gray is not considered.

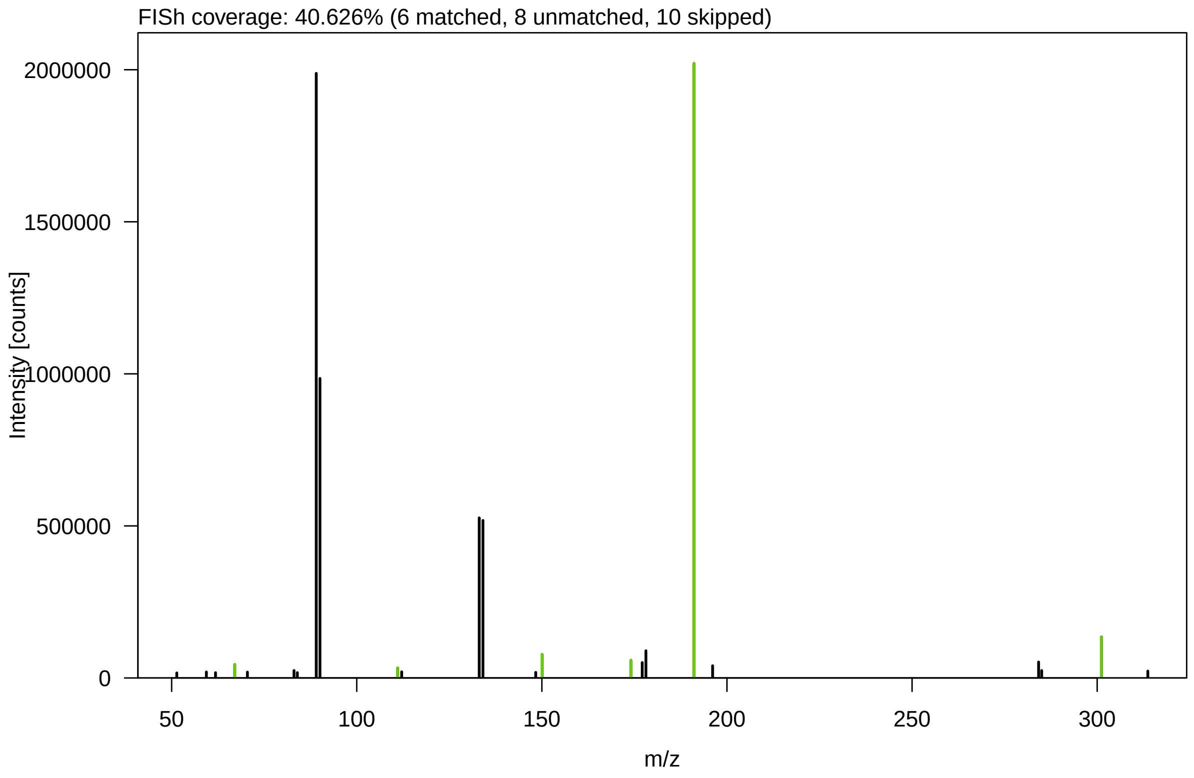

Figure SI-D41: Measured MS2 spectrum. Matching fragments with abacavir-5'-carboxylate predicted by FISh Scoring are highlighted in green. Low intensity fragments are not considered and skipped.

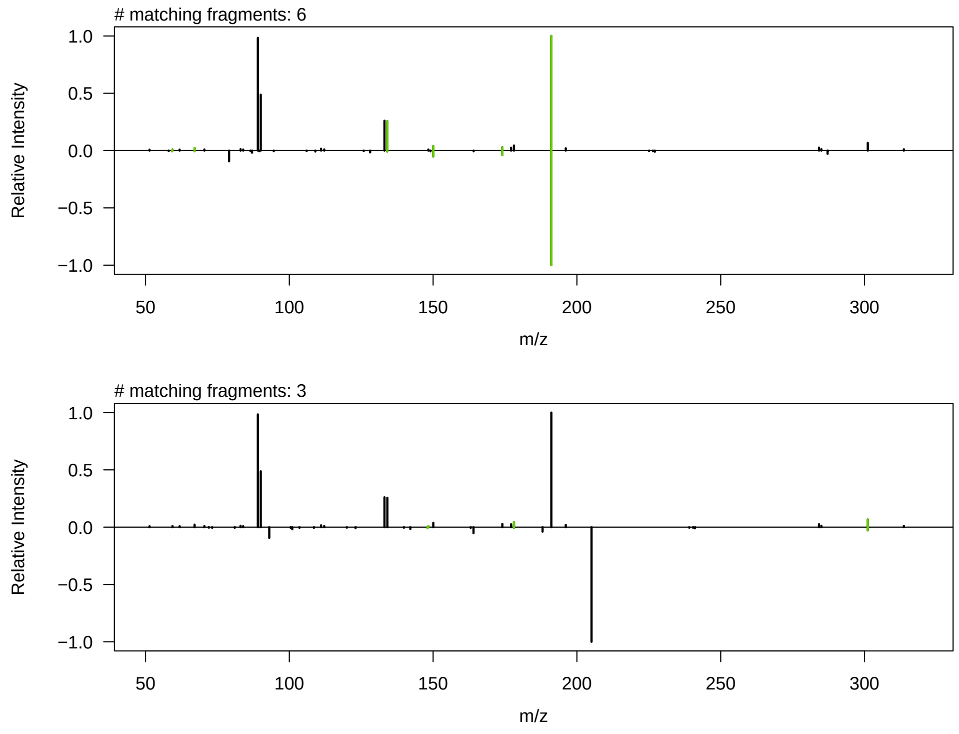

Figure SI-D42: Head to tail plots of abacavir-5'-carboxylate and abacavir. In the bottom plot, the mass spectrum of abacavir is shifted by the mass difference. Matching fragments are highlighted in green.

Table SI-D20: Molecular network results and retention time prediction of abacavir-5'-carboxylate.

Comparison with	Abacavir
MSn Score	87
Forward coverage	84
Reverse coverage	90
Forward match	27
Reverse match	18
ΔMass [g/mol]	13.9793
Measured retention time [min]	12.0
Predicted $logD_{OW}$ (pH = 2.7)	-1.25
Predicted retention time [min]	13.1
Predicted retention time range (95% confidence interval) [min]	8.5-17.7
Predicted retention time range (99% confidence interval) [min]	7.1-19.2

Table SI-D21: Annotated MS2 spectrum of abacavir-5'-carboxylate.

m/z	Relative Intensity	Annotation
51.3892	8.24	
59.3821	9.81	
61.8571	8.61	
67.0544	21.81	$C_5H_6 + H^+$
70.4700	9.52	
83.0491	12.07	$C_5H_6O + H^+$
83.9520	8.70	
89.0597	982.99	$C_2H_6N_3O + H^+$
90.0630	486.95	
111.0440	16.16	$C_6H_6O_2 + H^+$
112.1393	10.04	
133.0858	260.29	$C_4H_{10}N_3O_2 + H^+$
134.0892	256.09	$C_2H_9N_6O + H^+$
148.3484	9.11	
150.0648	38.13	$C_5H_5N_6 + H^+$
174.0775	28.83	$C_8H_7N_5 + H^+$
177.1124	25.14	$C_9H_{12}N_4 + H^+$
178.1152	44.25	
191.1038	999.00	$C_8H_{10}N_6 + H^+$
196.1262	19.89	
284.1794	25.95	
285.0082	12.08	
301.1410	66.77	$C_{14}H_{16}N_6O_2 + H^+$
313.6838	11.16	

A reference standard of abacavir-5'-carboxylate is commercially available. Figure SI-D43 shows the extracted ion chromatograms of this standard, the sample and the spiked sample, as well as a head to tail plot of the MS2 spectra of the standard and the sample. In addition, the most intense MS2 fragments in the sample and in the standard are displayed. It becomes visible that the retention times of the sample and the spiked sample are identical and several MS2 fragments besides the molecular ion between standard and sample match. It can therefore be concluded that the suspected compound is indeed abacavir-5'-carboxylate. Correspondingly, the identification confidence can be increased to level 1.

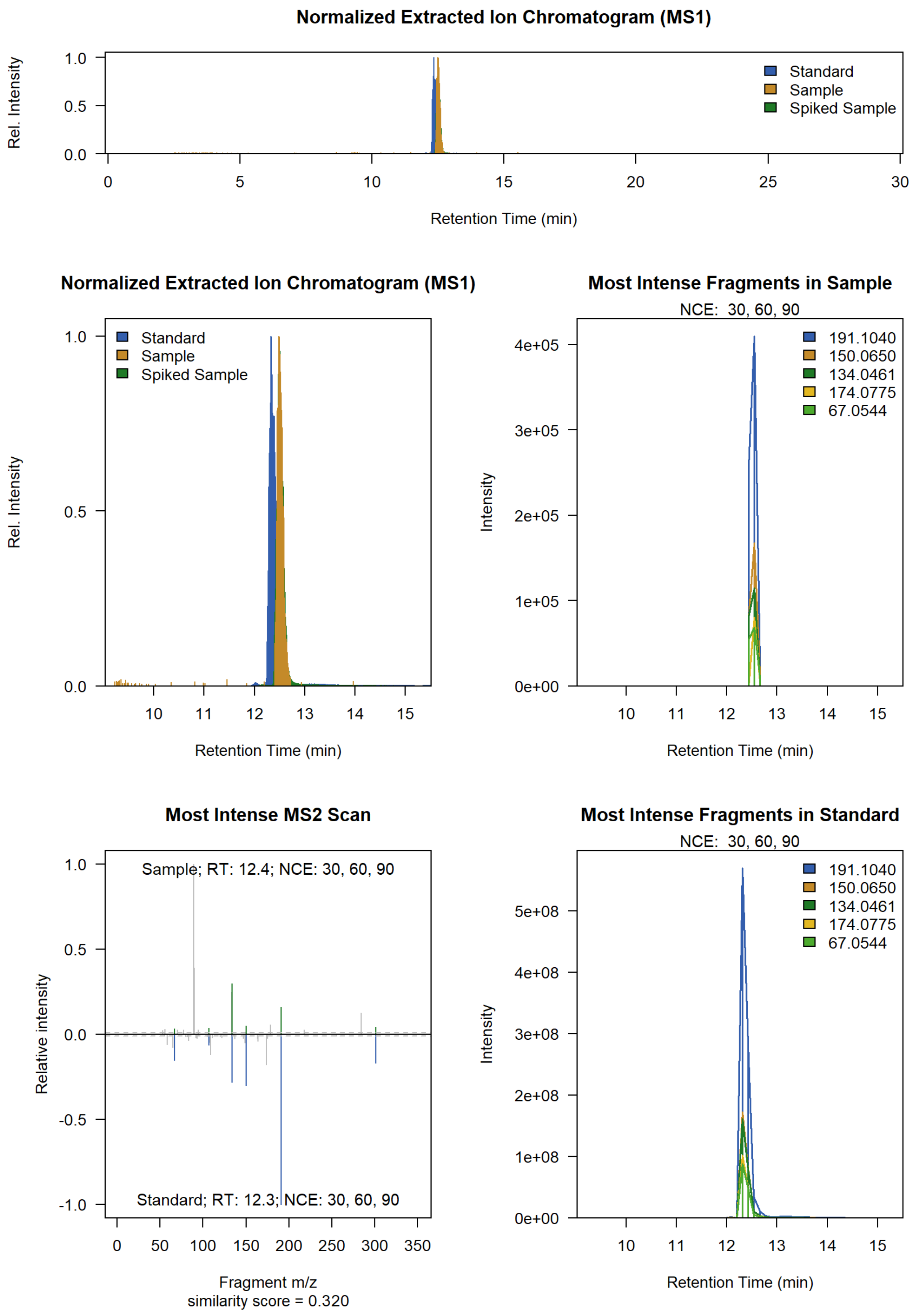

Figure SI-D43: Extracted ion chromatograms of abacavir-5'-carboxylate in the reference standard, the sample and the spiked sample, as well as MS2 head to tail plot and most intense MS2 fragments in standard and sample.

SI-D2.1.2 Abacavir-5'-Phosphate

Table SI-D22: Information on identifiers, chemical properties, detection and confidence of identification of abacavir-5'-phosphate.

IUPAC Name	[(1*S*,4*R*)-4-[2-amino-6-(cyclopropylamino)purin-9-yl]cyclopent-2-en-1-yl]methyl dihydrogen phosphate
Molecular formula	$C_{14}H_{19}N_6O_4P$
Monoisotopic mass [g/mol]	366.1205
Adduct	$[M+H]^+$
Retention time [min]	12.0
SMILES	C1CC1NC2=C3C(=NC(=N2)N)N(C=N3)[C@@H]4C[C@@H](C=C4)COP(=O)(O)O
InChI	InChI=1S/C14H19N6O4P/c15-14-18-12(17-9-2-3-9)11-13(19-14)20(7-16-11)10-4-1-8(5-10)6-24-25(21,22)23/h1,4,7-10H,2-3,5-6H2,(H2,21,22,23)(H3,15,17,18,19)/t8-,10+/m1/s1
InChI-Key	YQBOXVWMECPEJS-SCZZXKLOSA-N
CAS RN	136470-77-4
Metabolite of	Abacavir
Detection frequency	100% (15/15 samples)
Detected in	Altenrhein, Monday-Friday<br>Neugut, Monday-Friday<br>Werdhölzli, Monday-Friday
Intensity	E7
Initial confidence level	level 3
Initial confidence score	0.42
Final confidence level	level 3

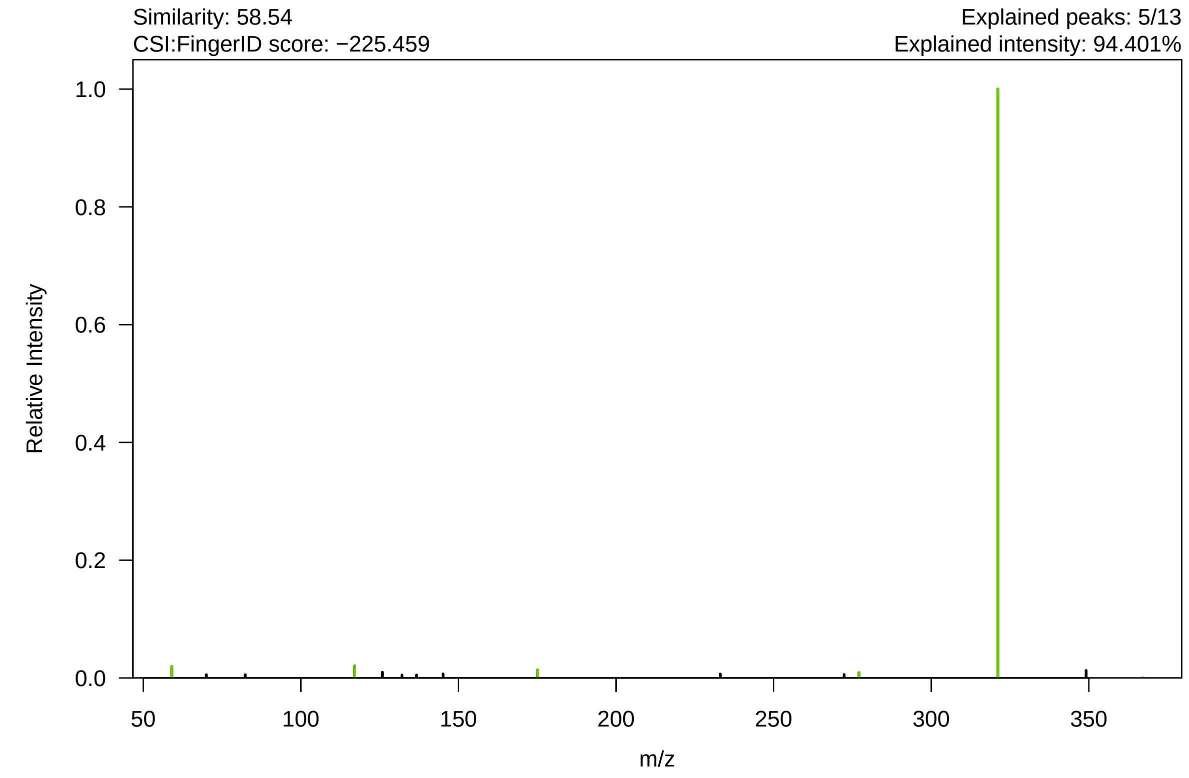

Figure SI-D44: Measured MS2 spectrum. Matching fragments with abacavir-5'-phosphate predicted by SIRIUS/CSI:FingerID are highlighted in green.

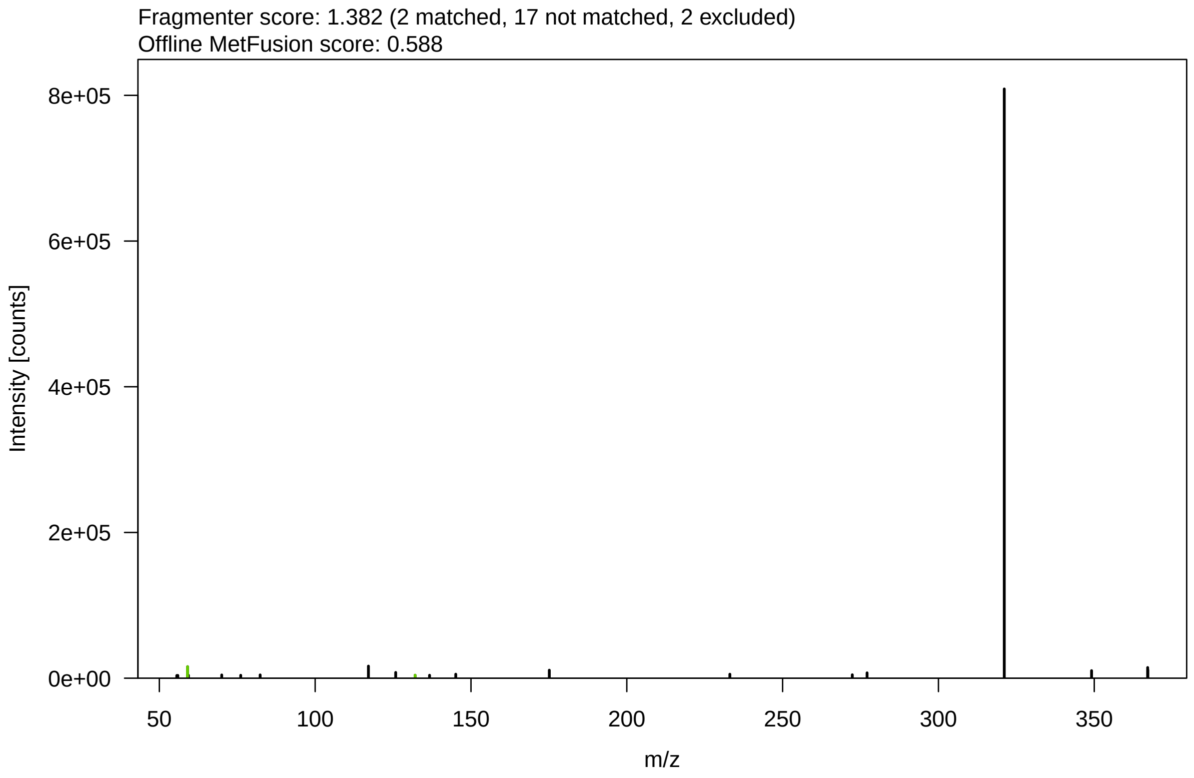

Figure SI-D45: Measured MS2 spectrum. Matching fragments with abacavir-5'-phosphate predicted by MetFrag are highlighted in green.

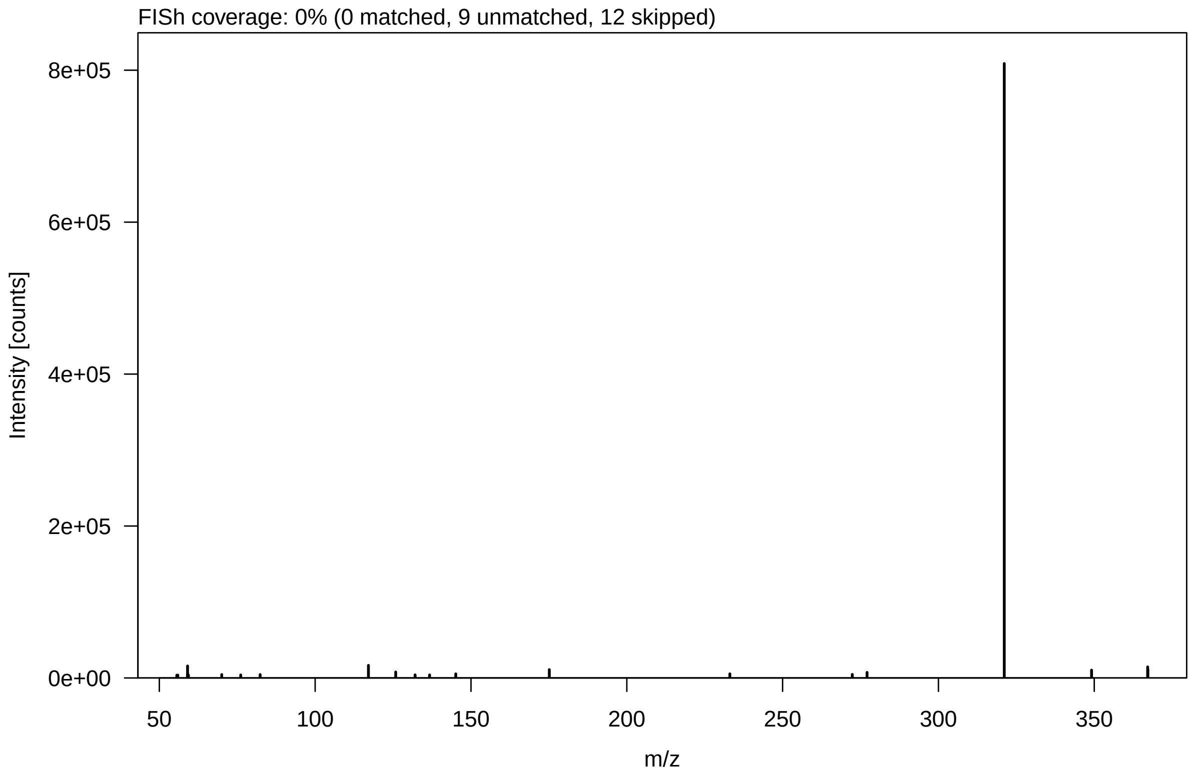

Figure SI-D46: Measured MS2 spectrum. None of the fragments could be rationalized by FISh Scoring. Low intensity fragments are not considered and skipped.

Table SI-D23: Retention time prediction of abacavir-5'-phosphate.

Measured retention time [min]	12.0
Predicted logD_{OW} (pH = 2.7)	-2.45
Predicted retention time [min]	11.6
Predicted retention time range (95% confidence interval) [min]	6.9-16.2
Predicted retention time range (99% confidence interval) [min]	5.5-17.6

Table SI-D24: Annotated MS2 spectrum of abacavir-5'-phosphate.

m/z	Relative Intensity	Annotation
55.5944	4.57	
55.9293	4.64	
59.0492	19.55	$C_3H_6O + H^+$
59.0519	5.68	
59.3416	4.92	
70.0183	5.52	
76.1108	4.95	
82.3454	5.57	
117.0908	20.50	$C_6H_{12}O_2 + H^+$
125.8400	9.74	
132.0681	5.06	$C_8H_7N_2 + H^+$
136.7062	5.00	
145.1156	6.77	
175.1321	13.60	$C_9H_{18}O_3 + H^+$
233.0698	6.75	
272.3564	5.78	
277.0974	9.06	$C_{11}H_{13}N_6OP + H^+$
321.1218	999.00	$C_{13}H_{17}N_6O_2P + H^+$
349.1394	12.78	
367.1415	18.04	$C_{14}H_{19}N_6O_4P + H^+$
367.1927	14.14	

A reference standard of abacavir-5'-phosphate was ordered, however, the product did not pass the quality control and led the manufacturer to the decision to not produce it anymore. Correspondingly, abacavir-5'-phosphate could neither be confirmed nor rejected. Therefore, the initial and final confidence levels are identical and are assigend to level 3.

SI-D2.2 Aliskiren Metabolites

Aliskiren is a non-peptide direct renin inhibitor used for the treatment of hypertension.[5] By suspect screening and molecular networking, two metabolites could be identified. The identified metabolites are highlighted in the metabolism scheme of aliskiren in Figure SI-D47. A panel of the molecular network of aliskiren is shown in Figure SI-D48. The following subsections give more details on the individual metabolites.

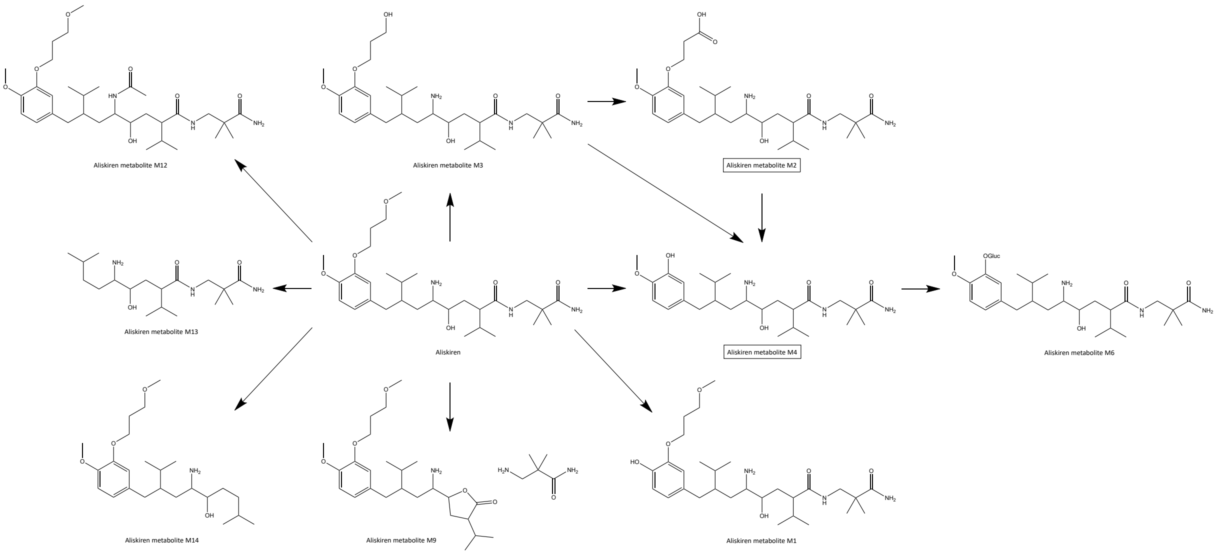

Figure SI-D47: Human metabolism of aliskiren. Framed metabolites were identified during suspect screening. Scheme adapted from.[5]

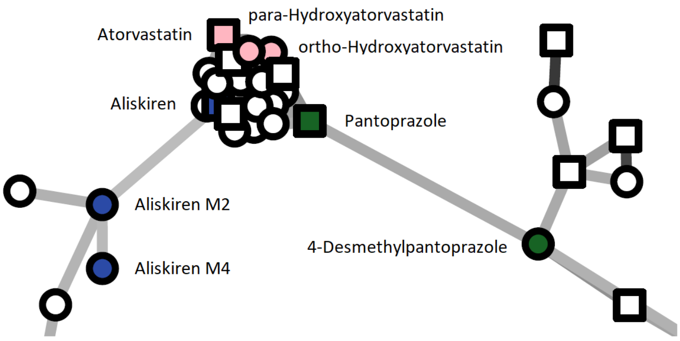

Figure SI-D48: Excerpt of the molecular network showing the aliskiren cluster.

SI-D2.2.1 Aliskiren Metabolite M2

Table SI-D25: Information on identifiers, chemical properties, detection and confidence of identification of aliskiren metabolite M2.

IUPAC Name	3-[5-[(2*S*,4*S*,5*S*,7*S*)-4-amino-7-[(3-amino-2,2-dimethyl-3-oxopropyl)carbamoyl]-5-hydroxy-8-methyl-2-propan-2-ylnonyl]-2-methoxyphenoxy]propanoic acid
Molecular formula	$C_{29}H_{49}N_3O_7$
Monoisotopic mass [g/mol]	551.3571
Adduct	$[M+H]^+$
Retention time [min]	22.1
SMILES	CC(C)[C@@H](CC1=CC(=C(C=C1)OC)OCCC(=O)O)C[C@@H]([C@H](C[C@@H](C(C)C)C(=O)NCC(C)(C)C(=O)N)O)N
InChI	InChI=1S/C29H49N3O7/c1-17(2)20(12-19-8-9-24(38-7)25(13-19)39-11-10-26(34)35)14-22(30)23(33)15-21(18(3)4)27(36)32-16-29(5,6)28(31)37/h8-9,13,17-18,20-23,33H,10-12,14-16,30H2,1-7H3,(H2,31,37)(H,32,36)(H,34,35)/t20-,21-,22-,23-/m0/s1
InChI-Key	WGLWFINTGGHHBQ-MLCQCVOFSA-N
CAS RN	949925-75-1
Metabolite of	Aliskiren
Detection frequency	100% (15/15 samples)
Detected in	Altenrhein, Monday-Friday<br>Neugut, Monday-Friday<br>Werdhölzli, Monday-Friday
Intensity	E6-E7
Initial confidence level	level 3
Initial confidence score	0.33
Final confidence level	level 4

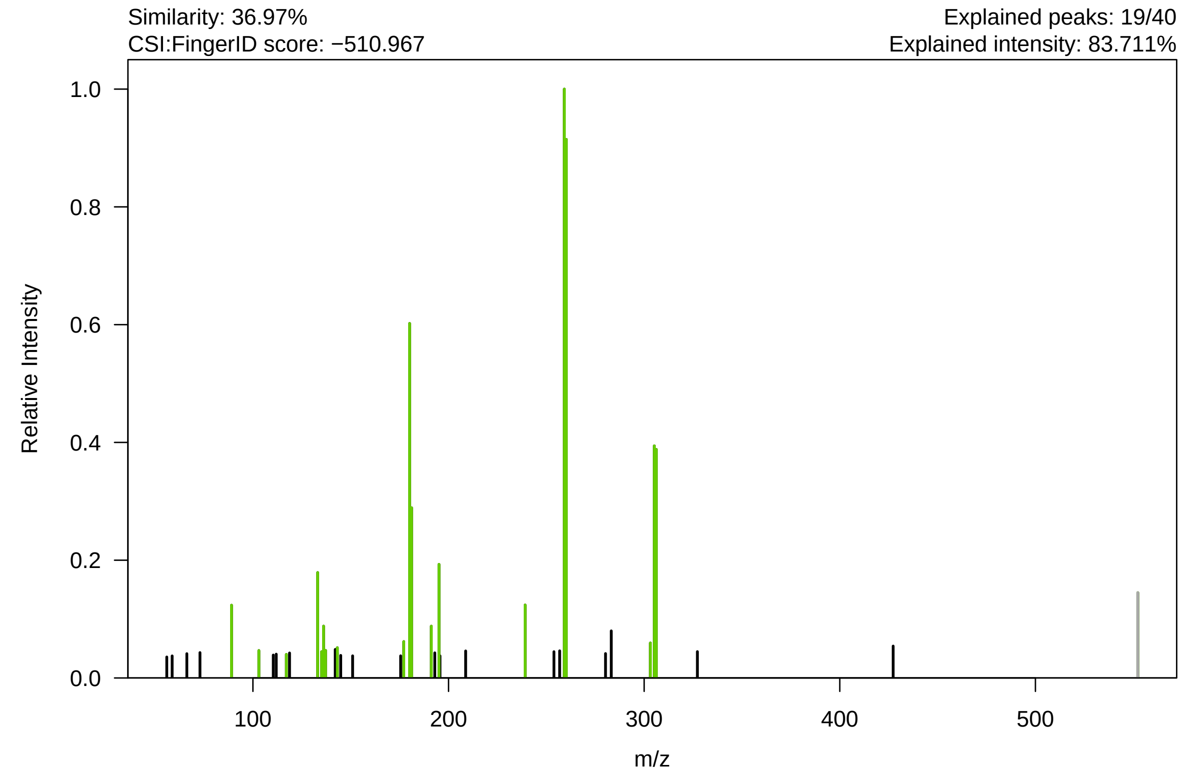

Figure SI-D49: Measured MS2 spectrum. Matching fragments with aliskiren metabolite M2 predicted by SIRIUS/CSI:FingerID are highlighted in green. The molecular ion in gray is not considered.

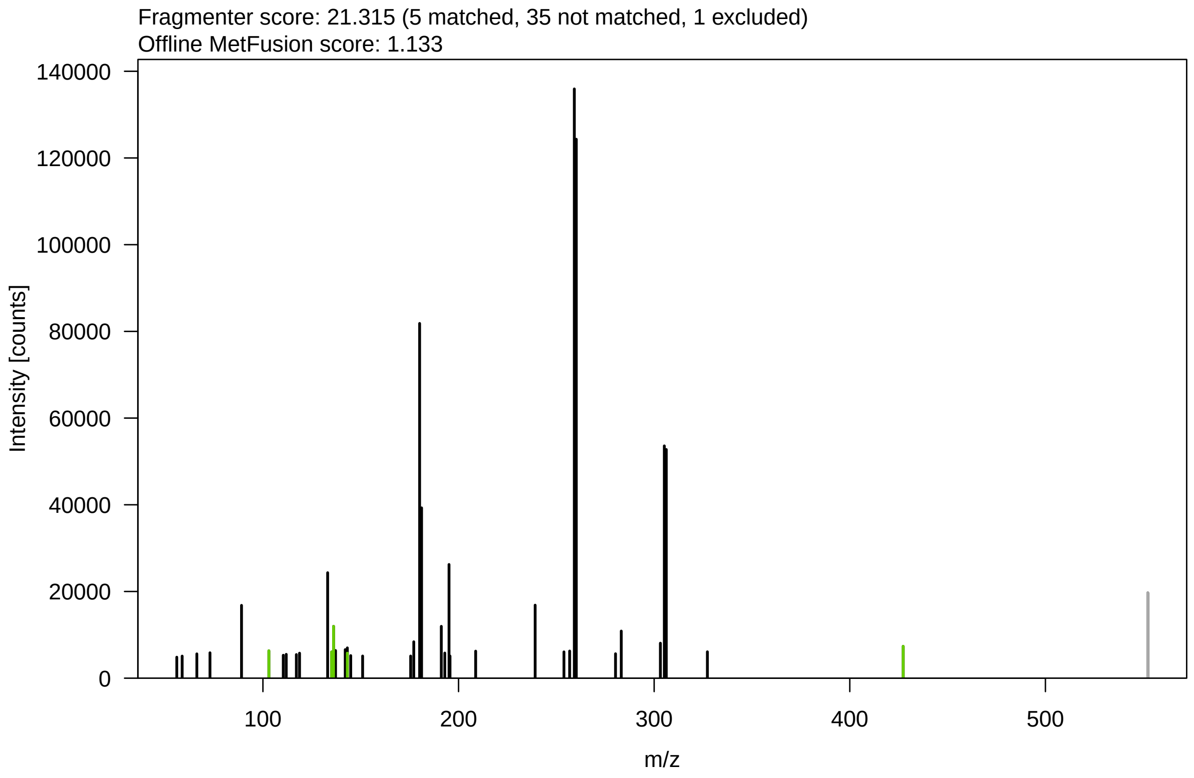

Figure SI-D50: Measured MS2 spectrum. Matching fragments with aliskiren metabolite M2 predicted by MetFrag are highlighted in green. The molecular ion in gray is not considered.

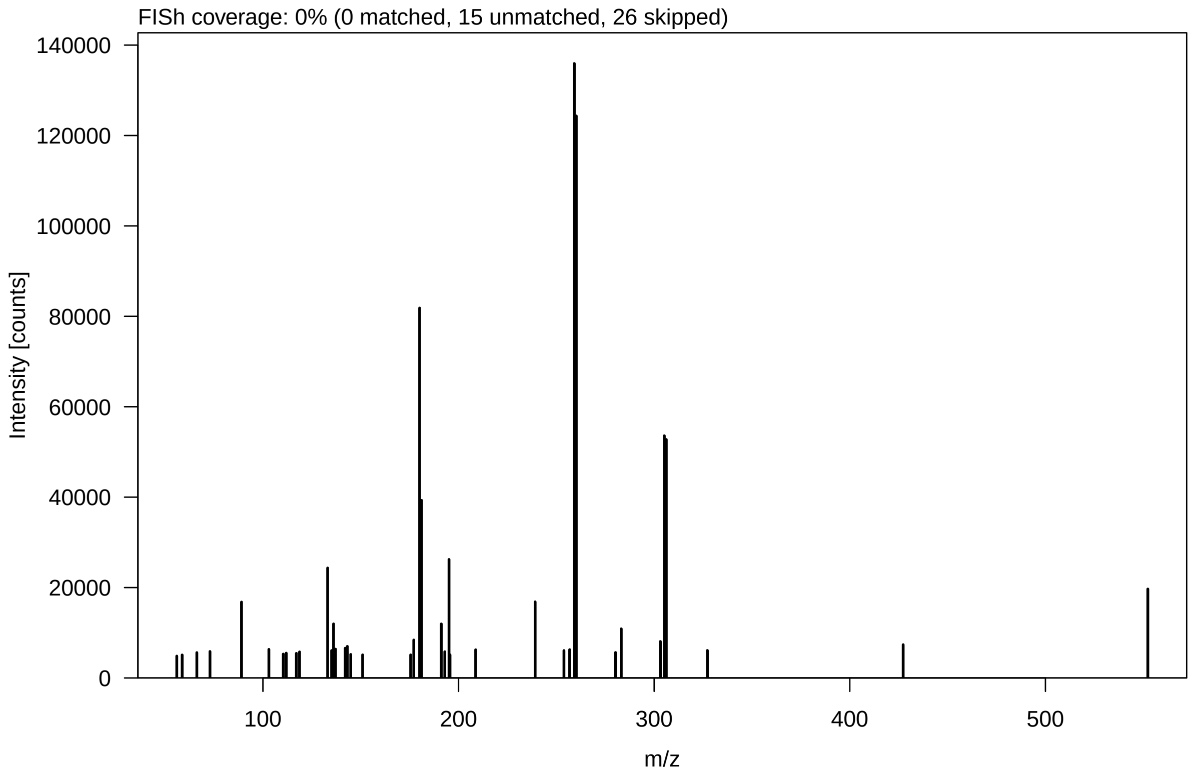

Figure SI-D51: Measured MS2 spectrum. None of the fragments could be rationalized by FISh Scoring. Low intensity fragments are not considered and skipped.

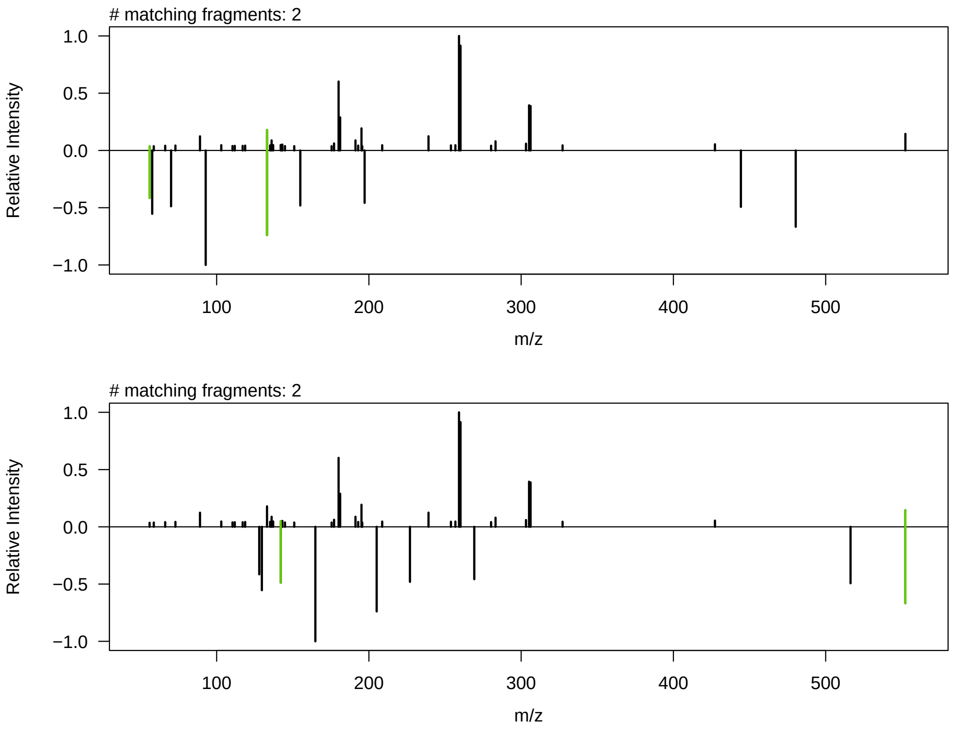

Figure SI-D52: Head to tail plots of aliskiren metabolite M2 and aliskiren metabolite M4. In the bottom plot, the mass spectrum of aliskiren metabolite M4 is shifted by the mass difference. Matching fragments are highlighted in green.

Table SI-D26: Molecular network results and retention time prediction of aliskiren metabolite M2.

Comparison with	Aliskiren metabolite M4
MSn Score	28
Forward coverage	50
Reverse coverage	6
Forward match	1
Reverse match	3
ΔMass [g/mol]	72.0212
Measured retention time [min]	22.1
Predicted $logD_{OW}$ (pH = 2.7)	-0.03
Predicted retention time [min]	14.7
Predicted retention time range (95% confidence interval) [min]	10.1-19.3
Predicted retention time range (99% confidence interval) [min]	8.7-20.8

Table SI-D27: Annotated MS2 spectrum of aliskiren metabolite M2.

m/z	Relative Intensity	Annotation
55.9624	35.60	
58.7128	37.43	
66.2154	41.24	
72.9221	43.06	
89.0597	123.44	$C_4H_8O_2 + H^+$
103.0389	46.53	$C_4H_6O_3 + H^+$
110.4043	38.89	
111.9110	40.41	
117.0911	40.03	$C_6H_{12}O_2 + H^+$
118.7178	42.41	
133.0859	178.81	$C_6H_{12}O_3 + H^+$
135.1162	44.60	$C_{10}H_{14} + H^+$
136.1122	87.94	$C_9H_{13}N + H^+$
137.1157	46.90	$C_6H_{16}O_3 + H^+$
142.0789	48.43	
143.1315	42.50	$C_8H_{16}NO + H^+$
143.1422	51.36	$C_9H_{18}O + H^+$
144.8800	38.28	
150.9654	37.58	
175.5206	37.56	
177.1114	61.74	$C_8H_{16}O_4 + H^+$
180.1018	601.44	$C_{10}H_{13}NO_2 + H^+$
181.1050	288.78	$C_7H_{16}O_5 + H^+$

Continued on next page

Table SI-D27: Annotated MS2 spectrum of aliskiren metabolite M2.(Continued)

191.1794	87.89	$C_{14}H_{22} + H^+$
192.9647	42.64	
195.1226	192.75	$C_8H_{18}O_5 + H^+$
195.6287	37.59	
208.7410	45.88	
239.1481	123.79	$C_{10}H_{22}O_6 + H^+$
253.8822	44.59	
256.8012	46.05	
259.1802	999.00	$C_{16}H_{22}N_2O + H^+$
260.1835	913.75	$C_{13}H_{25}NO_4 + H^+$
280.2478	41.41	
283.1744	79.90	
303.1714	59.34	$C_{17}H_{22}N_2O_3 + H^+$
305.1859	393.82	$C_{17}H_{24}N_2O_3 + H^+$
306.1888	387.71	$C_{14}H_{27}NO_6 + H^+$
327.2015	44.74	
427.2783	54.13	$C_{22}H_{38}N_2O_6 + H^+$
552.3527	144.62	$C_{29}H_{49}N_3O_7 + H^+$

No reference standard of aliskiren metabolite M2 was purchasable. Therefore, a human liver S9 incubation experiment with aliskiren was performed, to generate aliskiren metabolites *in vitro*. However, no compound with a precursor matching the one of aliskiren metabolite M2 was detectd within a retention time window of ±3 minutes of the suspect. Considering that the RT of the suspected candidate lies outside the 99% confidence interval of the predicted RT, the candidate is rejected and the confidence level decreased to level 4 due to the unequivocal molecular formula.

SI-D2.2.2 Aliskiren Metabolite M4

Table SI-D28: Information on identifiers, chemical properties, detection and confidence of identification of aliskiren metabolite M4.

IUPAC Name	(2*S*,4*S*,5*S*,7*S*)-5-amino-N-(3-amino-2,2-dimethyl-3-oxopropyl)-4-hydroxy-7-[(3-hydroxy-4-methoxyphenyl)methyl]-8-methyl-2-propan-2-ylnonanamide
Molecular formula	$C_{26}H_{45}N_3O_5$
Monoisotopic mass [g/mol]	479.3359
Adduct	$[M+H]^+$
Retention time [min]	22.3
SMILES	CC(C)[C@@H](CC1=CC(=C(C=C1)OC)O)C[C@@H]([C@H](C[C@@H](C(C)C)C(=O)NCC(C)(C)C(=O)N)O)N
InChI	InChI=1S/C26H45N3O5/c1-15(2)18(10-17-8-9-23(34-7)22(31)11-17)12-20(27)21(30)13-19(16(3)4)24(32)29-14-26(5,6)25(28)33/h8-9,11,15-16,18-21,30-31H,10,12-14,27H2,1-7H3,(H2,28,33)(H,29,32)/t18-,19-,20-,21-/m0/s1
InChI-Key	BCUNQZNYUMYGGS-TUFLPTIASA-N
CAS RN	949925-77-3
Metabolite of	Aliskiren
Detection frequency	100% (15/15 samples)
Detected in	Altenrhein, Monday-Friday<br>Neugut, Monday-Friday<br>Werdhölzli, Monday-Friday
Intensity	E8
Initial confidence level	level 3
Initial confidence score	0.18
Final confidence level	level 4

Due to the low quality MS2 spectrum, no SIRIUS/CSI:FingerID evaluation was possible.

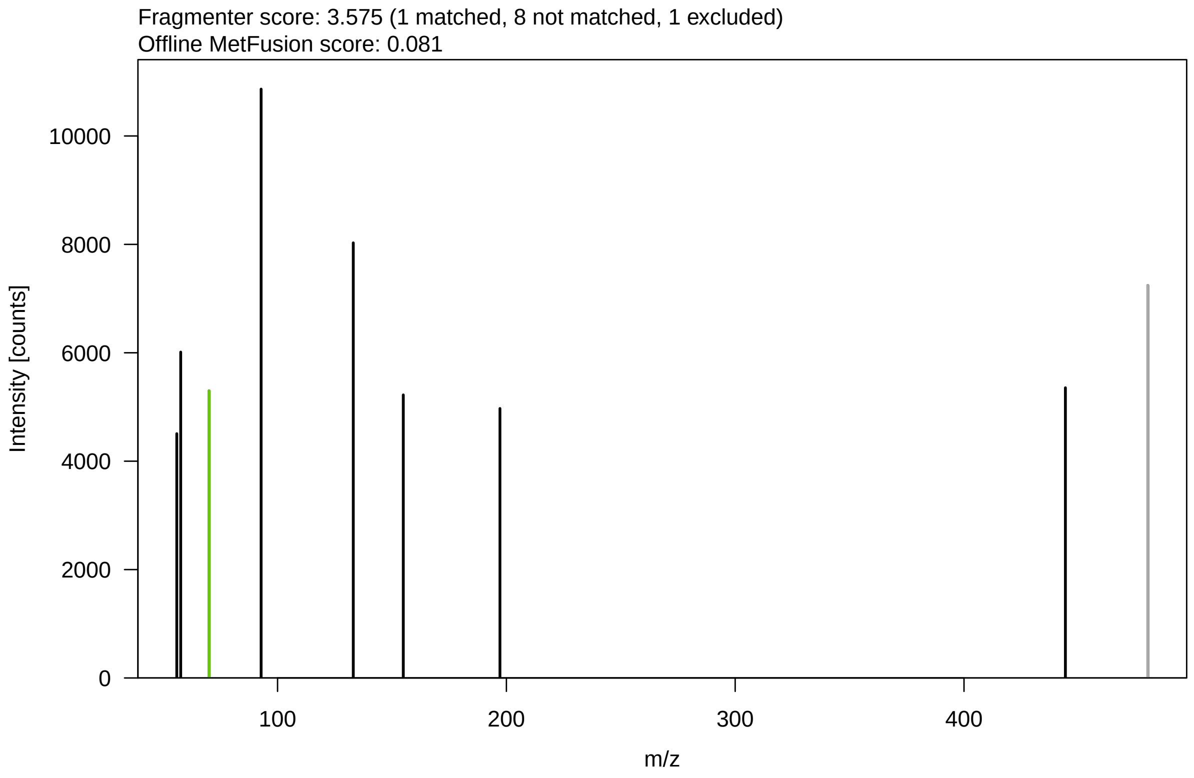

Figure SI-D53: Measured MS2 spectrum. Matching fragments with aliskiren metabolite M4 predicted by MetFrag are highlighted in green. The molecular ion in gray is not considered.

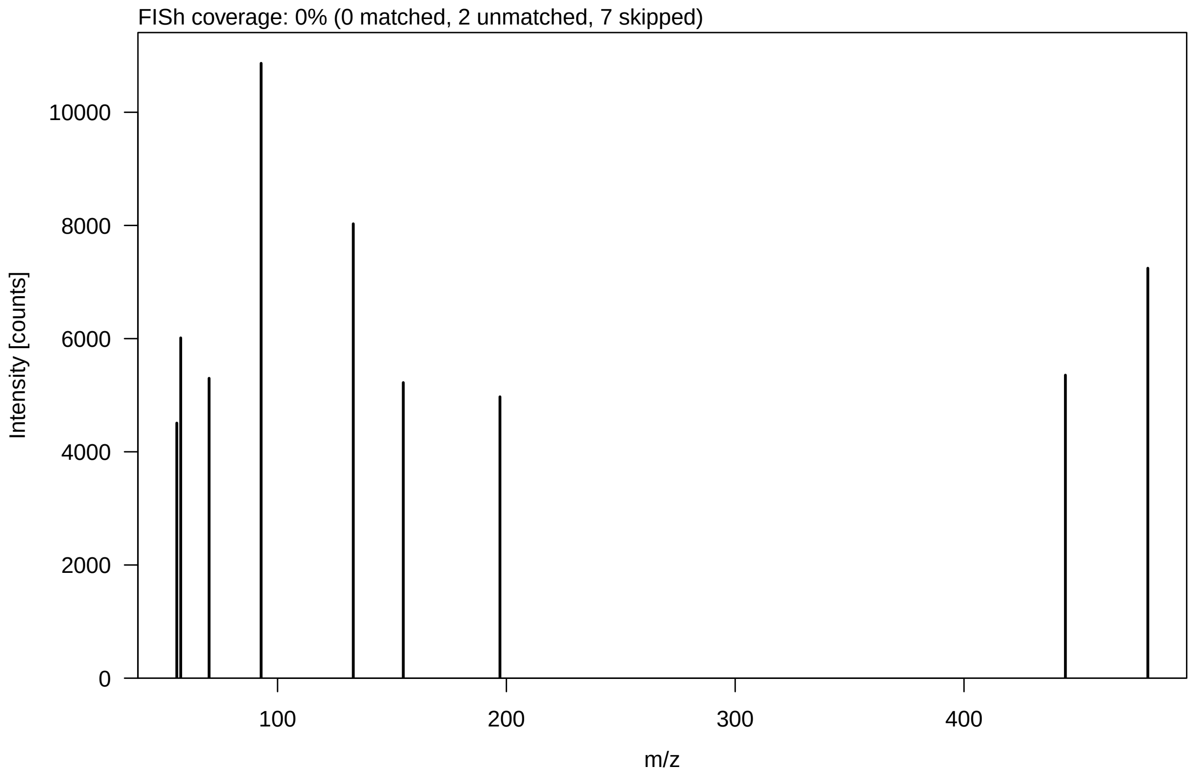

Figure SI-D54: Measured MS2 spectrum. None of the fragments could be rationlaized by FISh Scoring. Low intensity fragments are not considered and skipped.

Table SI-D29: Retention time prediction of aliskiren metabolite M4.

Measured retention time [min]	22.3
Predicted $logD_{OW}$ (pH = 2.7)	-0.07
Predicted retention time [min]	14.7
Predicted retention time range (95% confidence interval) [min]	10.1-19.2
Predicted retention time range (99% confidence interval) [min]	8.6-20.7

Table SI-D30: Annotated MS2 spectrum of aliskiren metabolite M4.

m/z	Relative Intensity	Annotation
55.9568	414.50	
57.6788	552.88	
70.0784	487.23	$C_5H_9 + H^+$
92.8105	999.00	
133.0856	738.24	
154.9429	480.24	
197.1904	457.16	
444.3139	492.37	
480.3345	666.06	$C_{26}H_{45}N_3O_5 + H^+$

No reference standard of aliskiren metabolite M4 was purchasable. Therefore, a human liver S9 incubation experiment with aliskiren was performed, to generate aliskiren metabolites *in vitro*. However, no compound with a precursor matching the one of aliskiren metabolite M4 was detectd within a retention time window of ±3 minutes of the suspect. Moreoever, the MS2 spectrum quality is low and the RT lies outside the 99% confidence interval of the predicted RT. Consequently, aliskiren metabolite M4 is rejected and the confidence level decreased to level 4 due to the unequivocal molecular formula.

SI-D2.3 Amlodipine Metabolites

Amlodipine is a dihydropyridine calcium channel blocker to treat hypertension and ischemic heart disease.[2,6] Metabolite M12 was covered in target screening, while the metabolites M9 and M10 werde detected by suspect screening and molecular networking. The identified metabolites are highlighted in the metabolism scheme of amlodipine in Figure SI-D55. An excerpt of the molecular network of the amlodipine metabolites is shown in Figure SI-D56. The following subsections give more details on the individual metabolites.

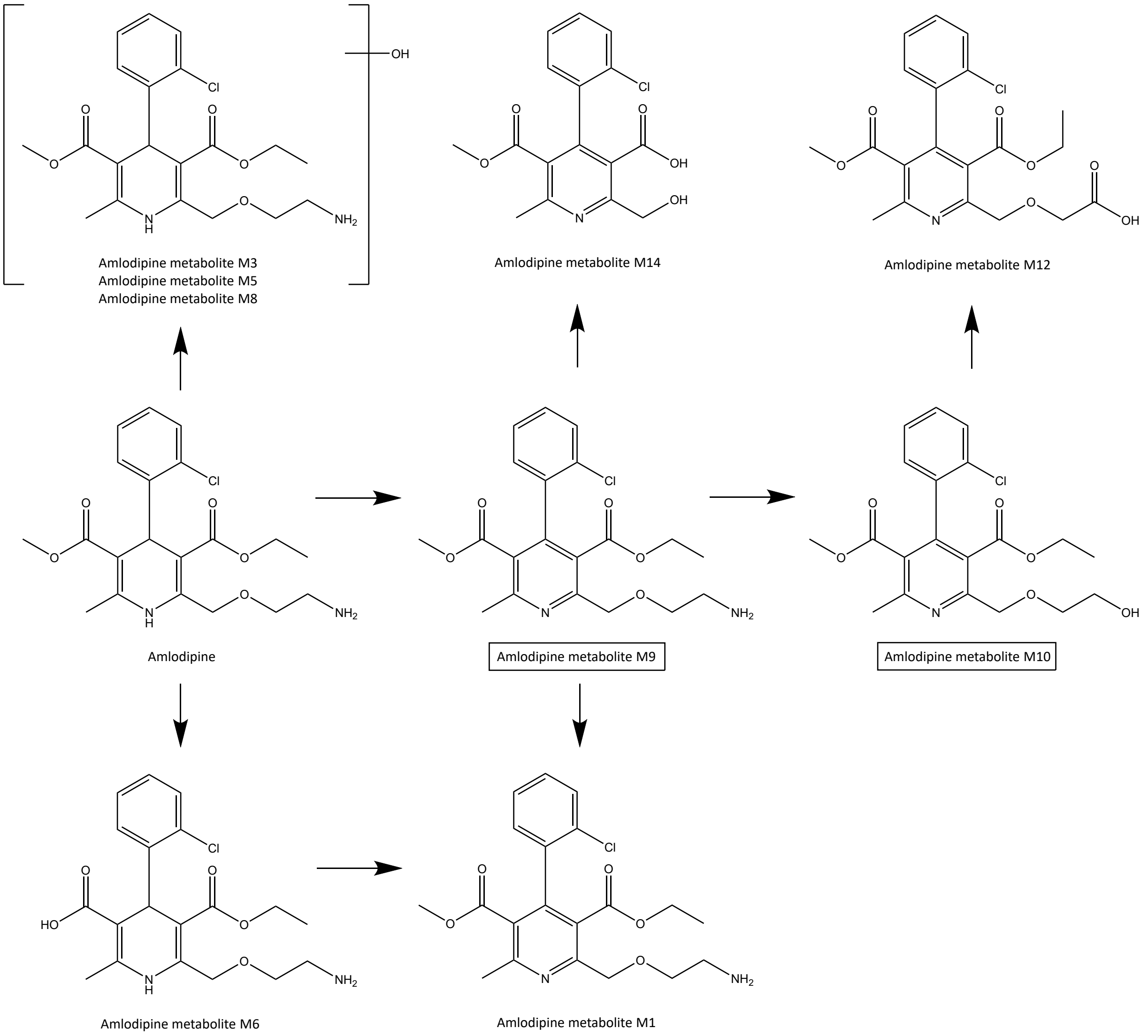

Figure SI-D55: Human metabolism of amlodipine. Framed metabolites were identified during suspect screening. Scheme adapted from.[6]

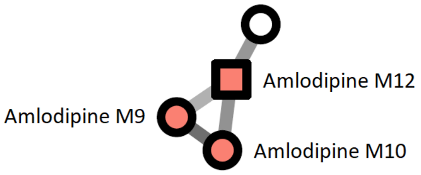

Figure SI-D56: Excerpt of the molecular network showing the amlodipine metabolites cluster.

SI-D2.3.1 Amlodipine Metabolite M9

Table SI-D31: Information on identifiers, chemical properties, detection and confidence of identification of amlodipine metabolite M9.

IUPAC Name	3-*O*-ethyl-5-*O*-methyl-2-(2-aminoethoxymethyl)-4-(2-chlorophenyl)-6-methylpyridine-3,5-dicarboxylate
Molecular formula	$C_{20}H_{23}ClN_2O_5$
Monoisotopic mass [g/mol]	406.1295
Adduct	$[M+H]^+$
Retention time [min]	16.1
SMILES	CCOC(=O)C1=C(C(=C(N=C1COCCN)C)C(=O)OC)C2=CC=CC=C2Cl
InChI	InChI=1S/C20H23ClN2O5/c1-4-28-20(25)18-15(11-27-10-9-22)23-12(2)16(19(24)26-3)17(18)13-7-5-6-8-14(13)21/h5-8H,4,9-11,22H2,1-3H3
InChI-Key	APZSGEHAFPIYQZ-UHFFFAOYSA-N
CAS RN	113994-41-5
Metabolite of	Amlodipine
Detection frequency	100% (15/15 samples)
Detected in	Altenrhein, Monday-Friday<br>Neugut, Monday-Friday<br>Werdhölzli, Monday-Friday
Intensity	E6-E7
Initial confidence level	level 3
Initial confidence score	0.40
Final confidence level	level 2b

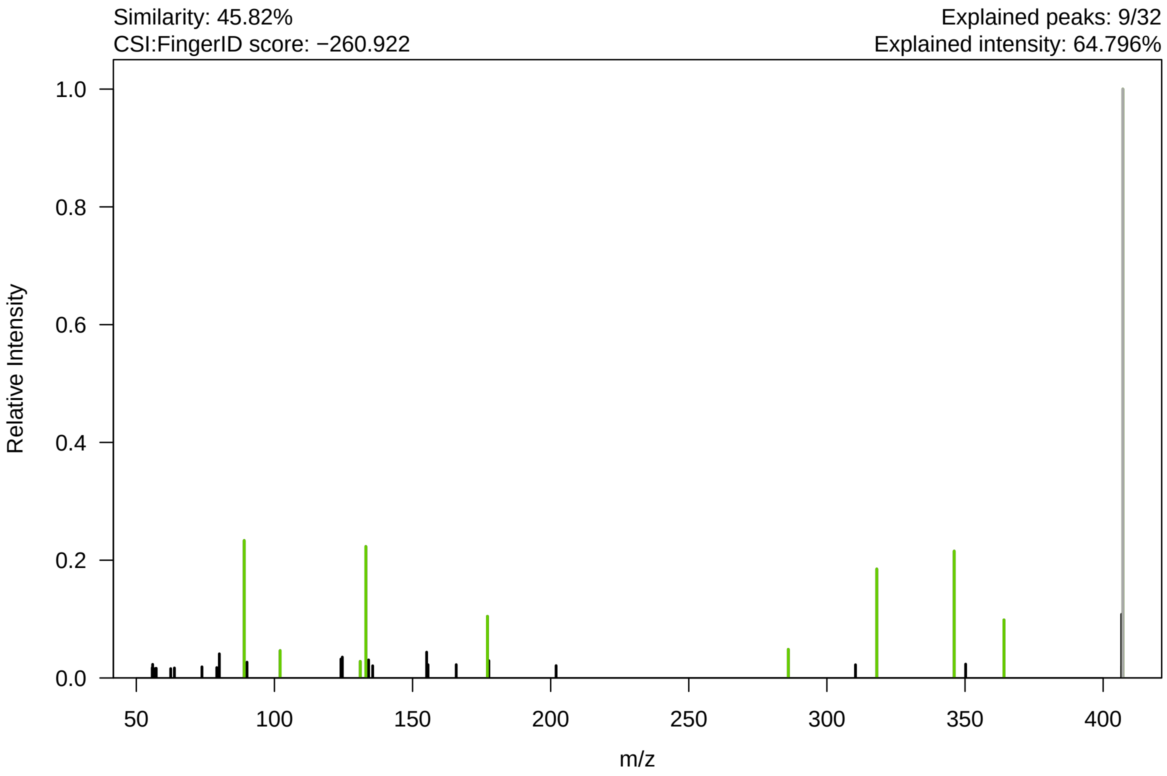

Figure SI-D57: Measured MS2 spectrum. Matching fragments with amlodipine metabolite M9 predicted by SIRIUS/CSI:FingerID are highlighted in green. The molecular ion in gray is not considered.

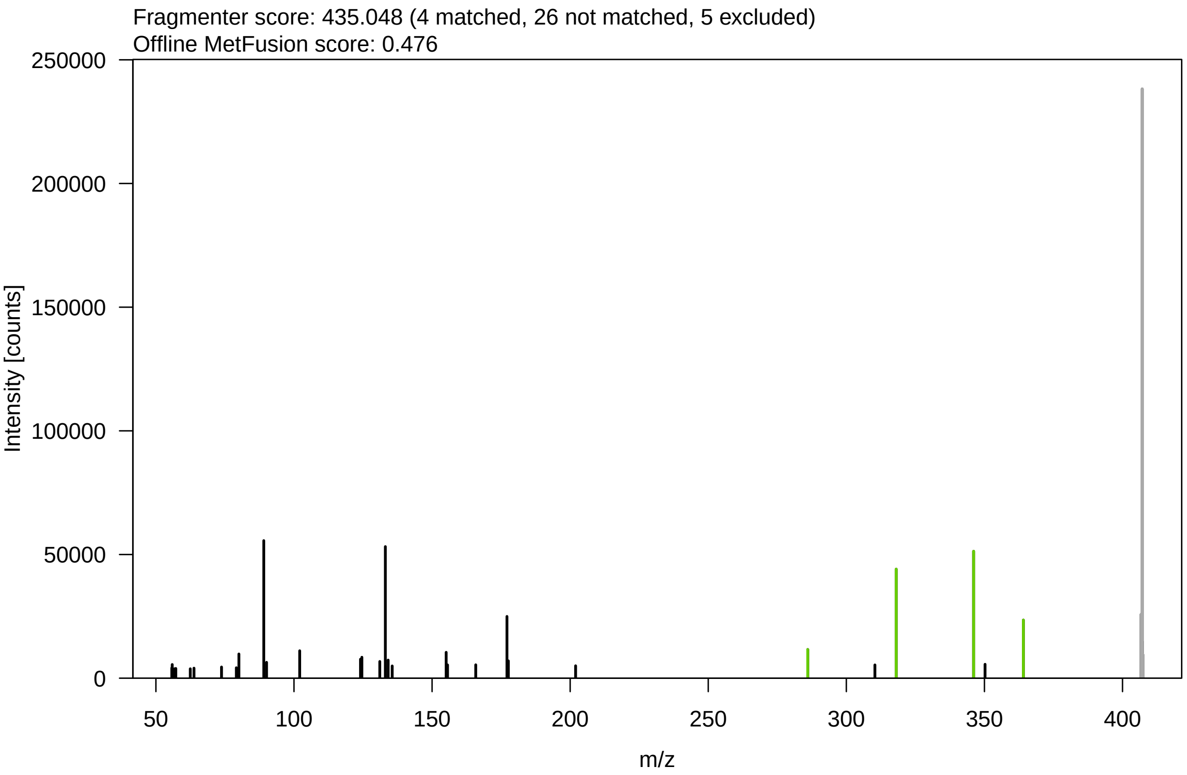

Figure SI-D58: Measured MS2 spectrum. Matching fragments with amlodipine metabolite M9 predicted by MetFrag are highlighted in green. The molecular ion in gray is not considered.

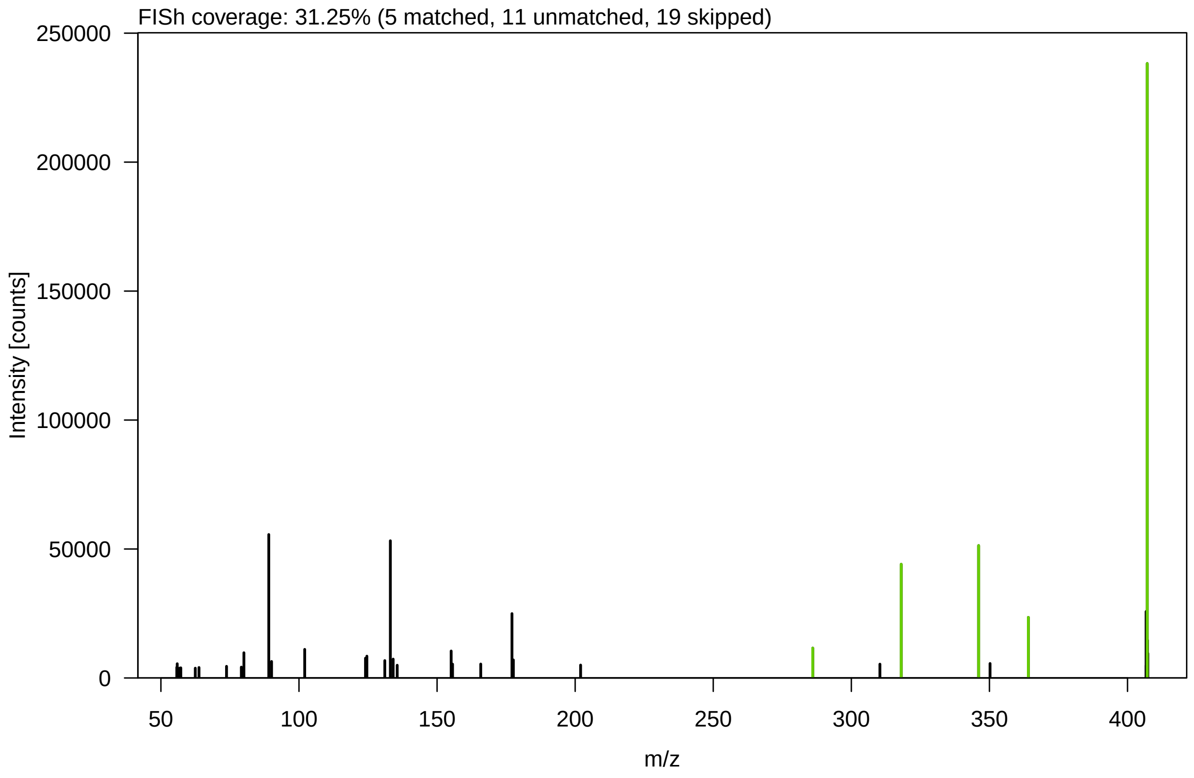

Figure SI-D59: Measured MS2 spectrum. Matching fragments with amlodipine metabolite M9 predicted by FISh Scoring are highlighted in green. Low intensity fragments are not considered and skipped.

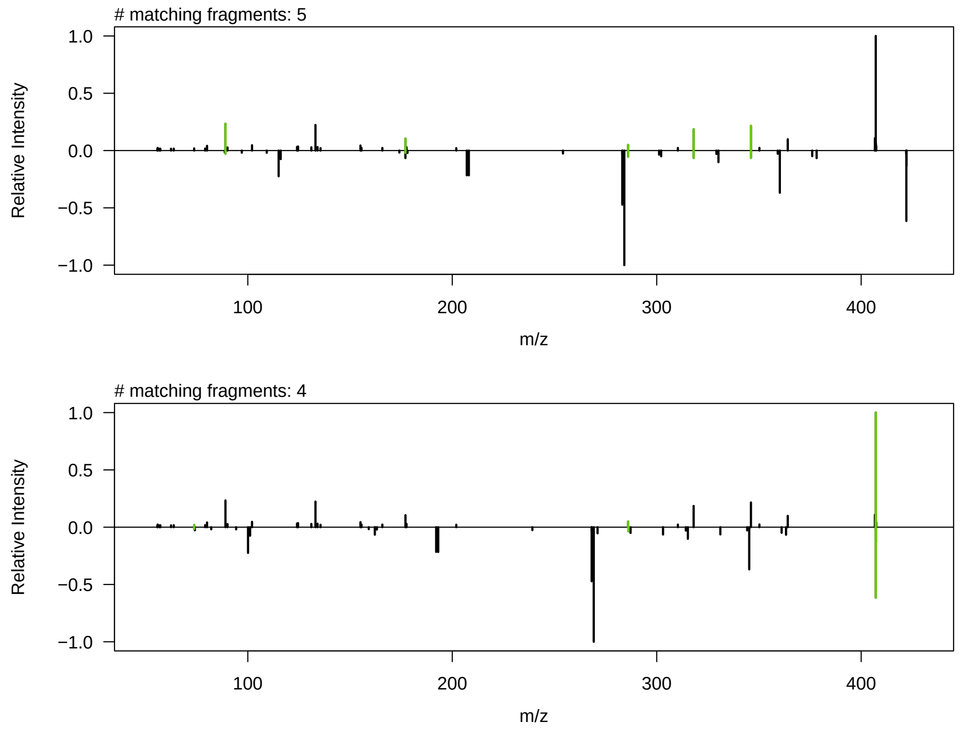

Figure SI-D60: Head to tail plots of amlodipine metabolite M9 and amlodipine metabolite M12. In the bottom plot, the mass spectrum of amlodipine metabolite M12 is shifted by the mass difference. Matching fragments are highlighted in green.

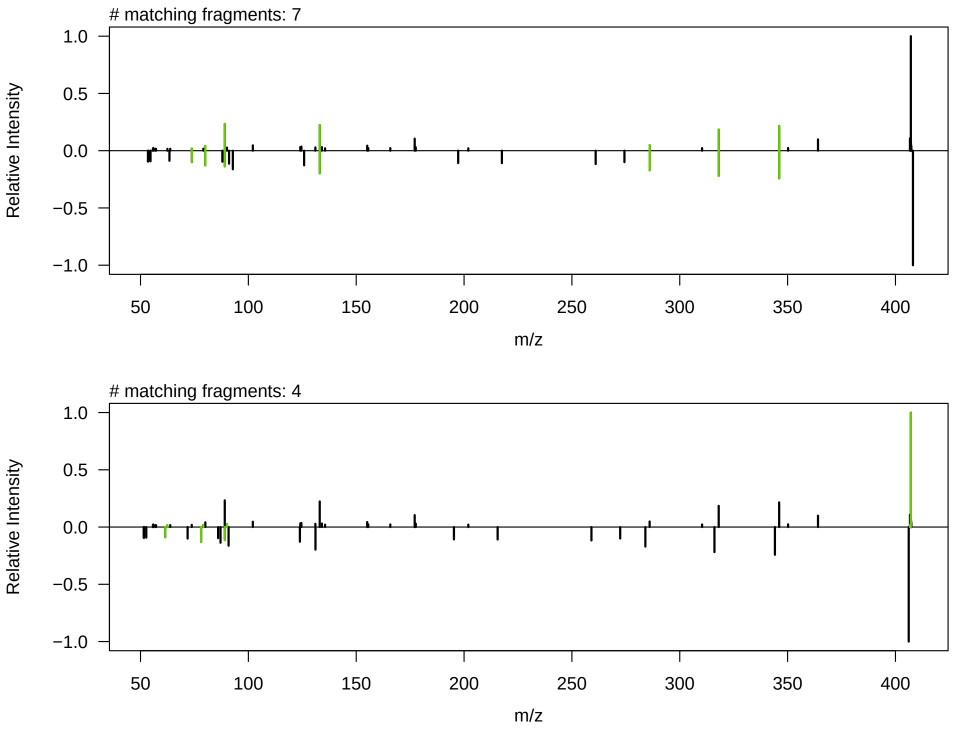

Figure SI-D61: Head to tail plots of amlodipine metabolite M9 and amlodipine metabolite M10. In the bottom plot, the mass spectrum of amlodipine metabolite M10 is shifted by the mass difference. Matching fragments are highlighted in green.

Table SI-D32: Molecular network results and retention time prediction of amlodipine metabolite M9.

Comparison with	Amlodipine metabolite M12
MSn Score	31
Forward coverage	38
Reverse coverage	25
Forward match	6
Reverse match	19
ΔMass [g/mol]	14.9633
Comparison with	Amlodipine metabolite M10
MSn Score	57
Forward coverage	31
Reverse coverage	83
Forward match	5
Reverse match	5
ΔMass [g/mol]	0.9841
Measured retention time [min]	16.1
Predicted $logD_{OW}$ (pH = 2.7)	-0.73
Predicted retention time [min]	13.8
Predicted retention time range (95% confidence interval) [min]	9.2-18.4
Predicted retention time range (99% confidence interval) [min]	7.7-19.8

Table SI-D33: Annotated MS2 spectrum of amlodipine metabolite M9.

m/z	Relative Intensity	Annotation
55.7500	17.25	
55.9090	23.12	
56.8122	16.08	
57.2034	16.44	
62.4478	15.91	
63.7908	16.99	
73.7600	18.92	
79.1224	17.63	
80.0541	41.00	
89.0596	233.20	$C_4H_8O_2 + H^+$
90.0634	26.84	
102.0672	46.46	$C_5H_9O_2 + H^+$
124.0807	32.16	
124.5822	35.46	
131.0706	28.27	$C_6H_{10}O_3 + H^+$
133.0859	223.04	$C_6H_{12}O_3 + H^+$
134.0890	30.66	
135.5600	20.70	
155.0986	43.83	
155.6008	22.52	
165.8060	22.66	
177.1118	104.63	$C_8H_{16}O_4 + H^+$
177.6136	29.36	
201.9668	20.97	
286.0262	48.61	$C_{15}H_8ClNO_3 + H^+$
310.3459	22.46	
318.0523	184.91	$C_{16}H_{12}ClNO_4 + H^+$
346.0840	215.25	$C_{18}H_{16}ClNO_4 + H^+$
350.2269	23.54	
364.0950	98.54	$C_{18}H_{18}ClNO_5 + H^+$
406.7224	108.08	
406.7683	20.09	
407.1357	999.00	$C_{20}H_{23}ClN_2O_5 + H^+$
407.2201	61.25	
407.3542	40.00	

No reference standard of amlodipine metabolite M9 was commercially available. Therefore, a human liver S9 incubation experiment with the parent amlodipine was performed, which led to the formation of amlodipine metabolite M9. Considering the spectral match of 0.502 (see Figure SI-D62) and the retention times of 16.1 and 16.2 minutes in the wastewater and the human liver S9 sample, respectively, further confidence could be gained that the detected feature in wastewater is amlodipine metabolite M9. Due to this diagnostic evidence, the final confidence level can be increased from level 3 to level 2b.

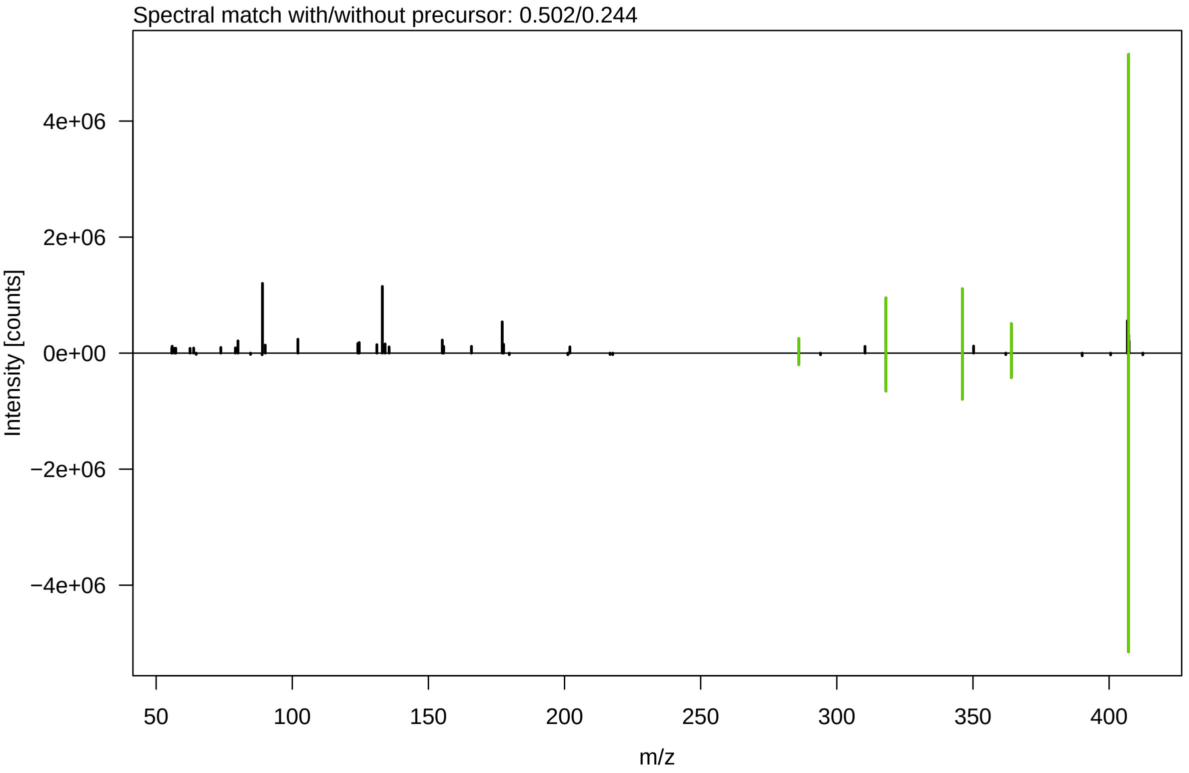

Figure SI-D62: Head to tail plot of amlodipine metabolite M9 in wastewater (top) and from human liver S9 incubation (bottom). Matching fragments are highlighted in green.

SI-D2.3.2 Amlodipine Metabolite M10

Table SI-D34: Information on identifiers, chemical properties, detection and confidence of identification of amlodipine metabolite M10.

IUPAC Name	3-ethyl-5-methyl-4-(2-chlorophenyl)-2-((2-hydroxyethoxy)methyl)-6-methylpyridine-3,5-dicarboxylate
Molecular formula	$C_{20}H_{22}ClNO_6$
Monoisotopic mass [g/mol]	407.1136
Adduct	$[M+H]^+$
Retention time [min]	19.3
SMILES	CCOC(C1=C(C2=CC=CC=C2Cl)C(C(OC)=O)=C(C)N=C1COCCO)=O
InChI	InChI=1S/C20H22ClNO6/c1-4-28-20(25)18-15(11-27-10-9-23)22-12(2)16(19(24)26-3)17(18)13-7-5-6-8-14(13)21/h5-8,23H,4,9-11H2,1-3H3
InChI-Key	GENKLQVQPHBQHA-UHFFFAOYSA-N
CAS RN	-
Metabolite of	Amlodipine
Detection frequency	100% (15/15 samples)
Detected in	Altenrhein, Monday-Friday<br>Neugut, Monday-Friday<br>Werdhölzli, Monday-Friday
Intensity	E6
Initial confidence level	level 3
Initial confidence score	0.41
Final confidence level	level 2b

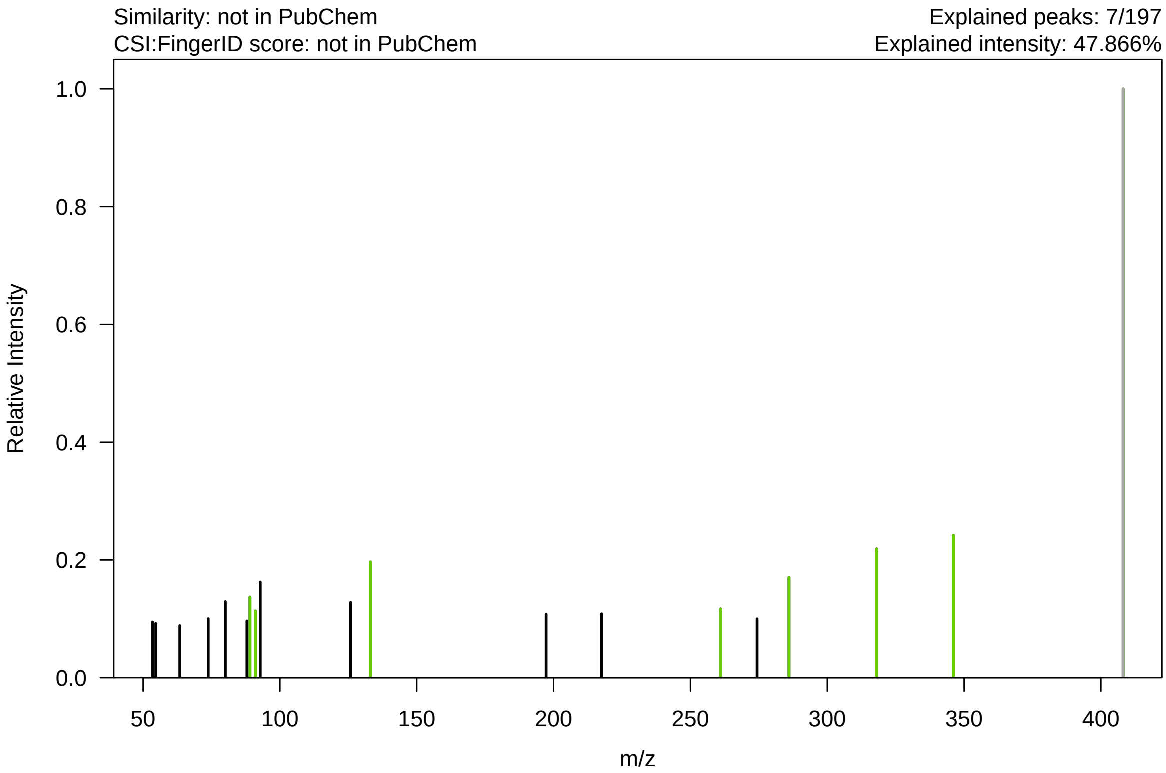

Figure SI-D63: Measured MS2 spectrum. Matching fragments with amlodipine metabolite M10 predicted by SIRIUS/CSI:FingerID are highlighted in green. The molecular ion in gray is not considered.

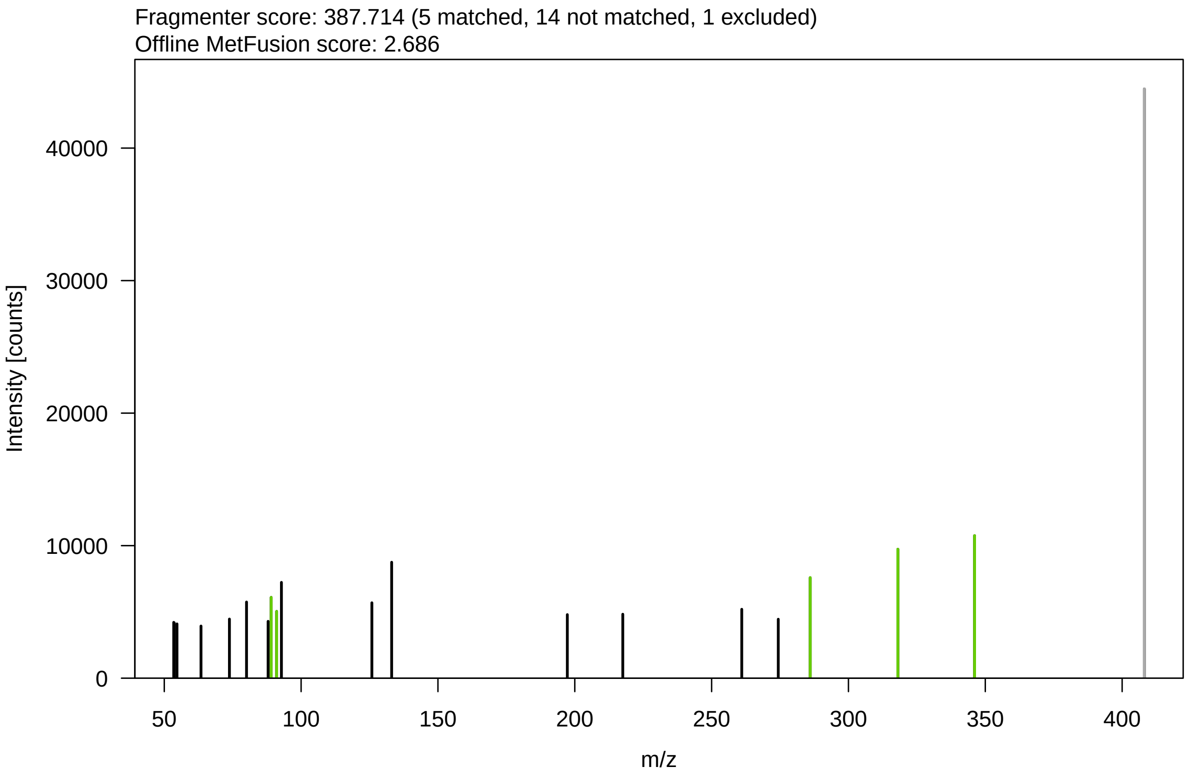

Figure SI-D64: Measured MS2 spectrum. Matching fragments with amlodipine metabolite M10 predicted by MetFrag are highlighted in green. The molecular ion in gray is not considered.

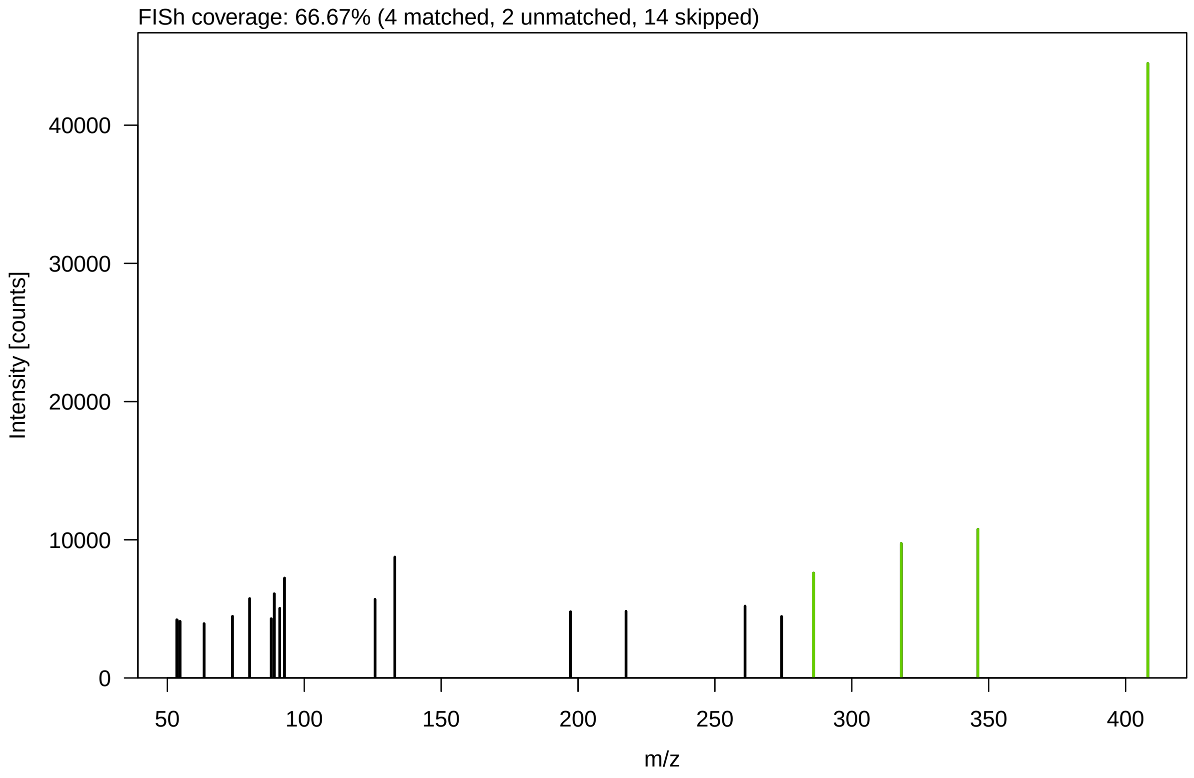

Figure SI-D65: Measured MS2 spectrum. Matching fragments with amlodipine metabolite M10 predicted by FISh Scoring are highlighted in green. Low intensity fragments are not considered and skipped.

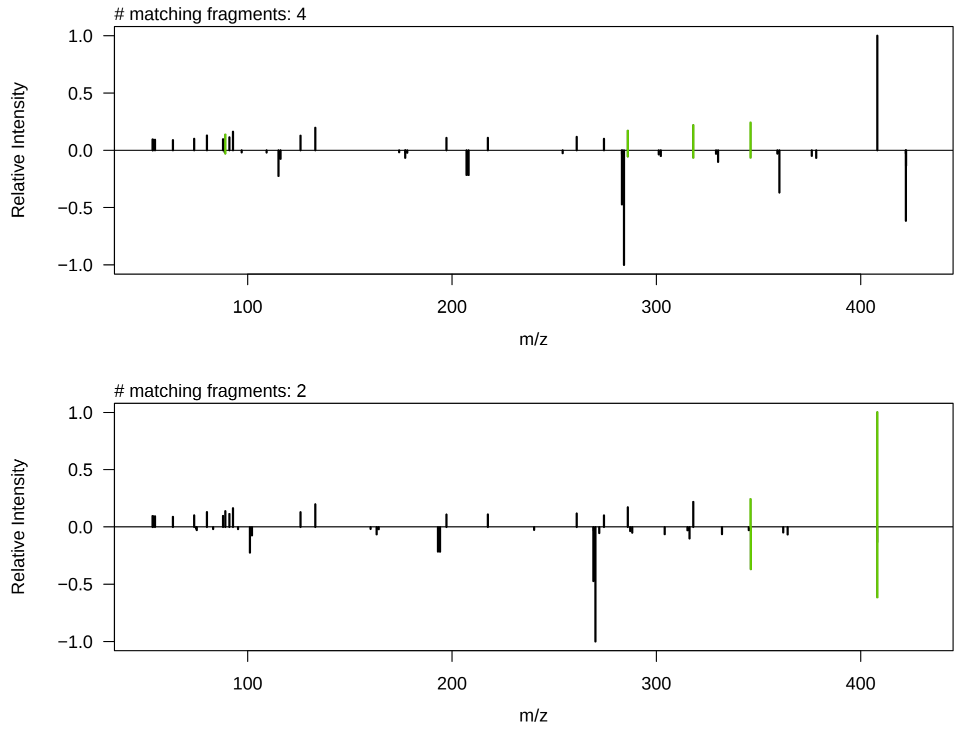

Figure SI-D66: Head to tail plots of amlodipine metabolite M10 and amlodipine metabolite M12. In the bottom plot, the mass spectrum of amlodipine metabolite M12 is shifted by the mass difference. Matching fragments are highlighted in green.

Table SI-D35: Molecular network results and retention time prediction of amlodipine metabolite M10.

Comparison with	Amlodipine metabolite M12
MSn Score	43
Forward coverage	67
Reverse coverage	19
Forward match	4
Reverse match	14
ΔMass [g/mol]	13.9792
Measured retention time [min]	19.3
Predicted $logD_{OW}$ (pH = 2.7)	2.41
Predicted retention time [min]	17.9
Predicted retention time range (95% confidence interval) [min]	13.3-22.5
Predicted retention time range (99% confidence interval) [min]	11.8-23.9

Table SI-D36: Annotated MS2 spectrum of amlodipine metabolite M10.

m/z	Relative Intensity	Annotation
53.4463	94.63	
53.6477	93.73	
54.6407	92.05	
63.4152	88.39	
73.8058	100.25	
80.0541	129.12	
87.9434	96.39	
89.0599	136.99	$C_4H_8O_2 + H^+$
91.0541	113.24	$C_7H_6 + H^+$
92.8077	162.42	
125.8551	127.82	
133.0864	196.55	$C_6H_{12}O_3 + H^+$
197.2743	107.73	
217.5359	108.43	
261.0156	116.81	$C_{10}H_9ClO_6 + H^+$
274.3533	99.96	
286.0257	170.46	$C_{15}H_8ClNO_3 + H^+$
318.0525	218.70	$C_{16}H_{12}ClNO_4 + H^+$
346.0829	241.68	$C_{18}H_{16}ClNO_4 + H^+$
408.1198	999.00	$C_{20}H_{22}ClNO_6 + H^+$

No reference standard of amlodipine metabolite M10 was commercially available. Therefore, a human liver S9 incubation experiment with the parent amlodipine was performed, which led to the formation of amlodipine metabolite M10. Considering the spectral match of 0.382 (see Figure SI-D67) and the retention times of 19.3 and 19.7 minutes in the wastewater and the human liver S9 sample, respectively, further confidence could be gained that the detected feature in wastewater is amlodipine metabolite M10. Due to this diagnostic evidence, the final confidence level can be increased from level 3 to level 2b.

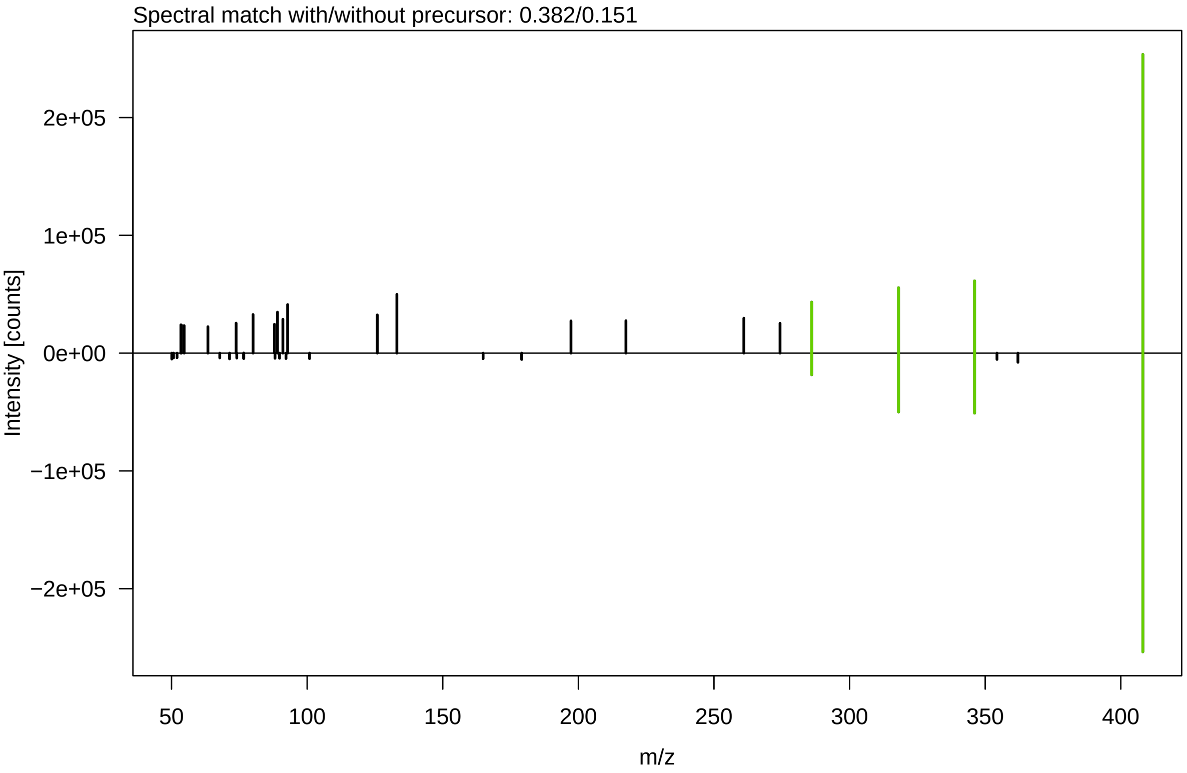

Figure SI-D67: Head to tail plot of amlodipine metabolite M10 in wastewater (top) and from human liver S9 incubation (bottom). Matching fragments are highlighted in green.

SI-D2.4 Atorvastatin Metabolites

Atorvastatin is a HMG-CoA reductase inhibitor. It is used to lower lipid levels and reduce the risk of cardiovascular disease including myocardial infarction and stroke.[2] Suspect screening and molecular networking enabled the identification of two hydroxylated human metabolites. The identified metabolites are highlighted in the metabolism scheme of atorvastatin in Figure SI-D68. A panel of the molecular network of the atrovastatin metabolites is shown in Figure SI-D69. The following subsections give more details on the individual metabolites.

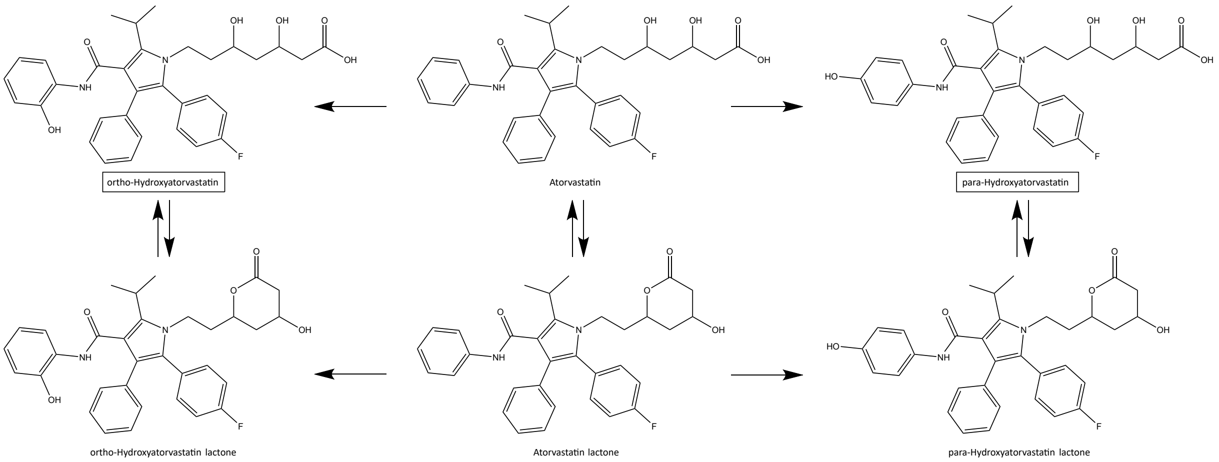

Figure SI-D68: Human metabolism of atorvastatin. Framed metabolites were identified during suspect screening. Scheme adapted from.[7]

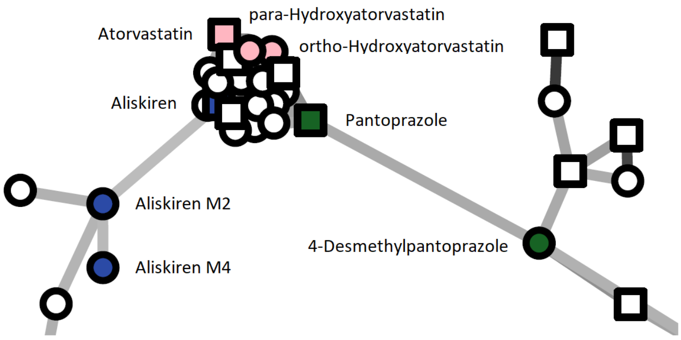

Figure SI-D69: Excerpt of the molecular network showing the atorvastatin cluster.

SI-D2.4.1 ortho-Hydroxyatorvastatin

Table SI-D37: Information on identifiers, chemical properties, detection and confidence of identification of ortho-hydroxytorasemide.

IUPAC Name	7-[2-(4-fluorophenyl)-4-[(2-hydroxyphenyl)carbamoyl]-3-phenyl-5-propan-2-ylpyrrol-1-yl]-3,5-dihydroxyheptanoic acid
Molecular formula	$C_{33}H_{35}FN_2O_6$
Monoisotopic mass [g/mol]	574.2479
Adduct	$[M+H]^+$
Retention time [min]	20.3
SMILES	CC(C)C1=C(C(=C(N1CCC(CC(CC(=O)O)O)O)C2=CC=C(C=C2)F)C3=CC=CC=C3)C(=O)NC4=CC=CC=C4O
InChI	InChI=1S/C33H35FN2O6/c1-20(2)31-30(33(42)35-26-10-6-7-11-27(26)39)29(21-8-4-3-5-9-21)32(22-12-14-23(34)15-13-22)36(31)17-16-24(37)18-25(38)19-28(40)41/h3-15,20,24-25,37-39H,16-19H2,1-2H3,(H,35,42)(H,40,41)
InChI-Key	CZBPKFICAYVHHM-UHFFFAOYSA-N
CAS RN	-
Metabolite of	Atorvastatin
Detection frequency	100% (15/15 samples)
Detected in	Altenrhein, Monday-Friday<br>Neugut, Monday-Friday<br>Werdhölzli, Monday-Friday
Intensity	E7
Initial confidence level	level 3
Initial confidence score	0.41
Final confidence level	level 1

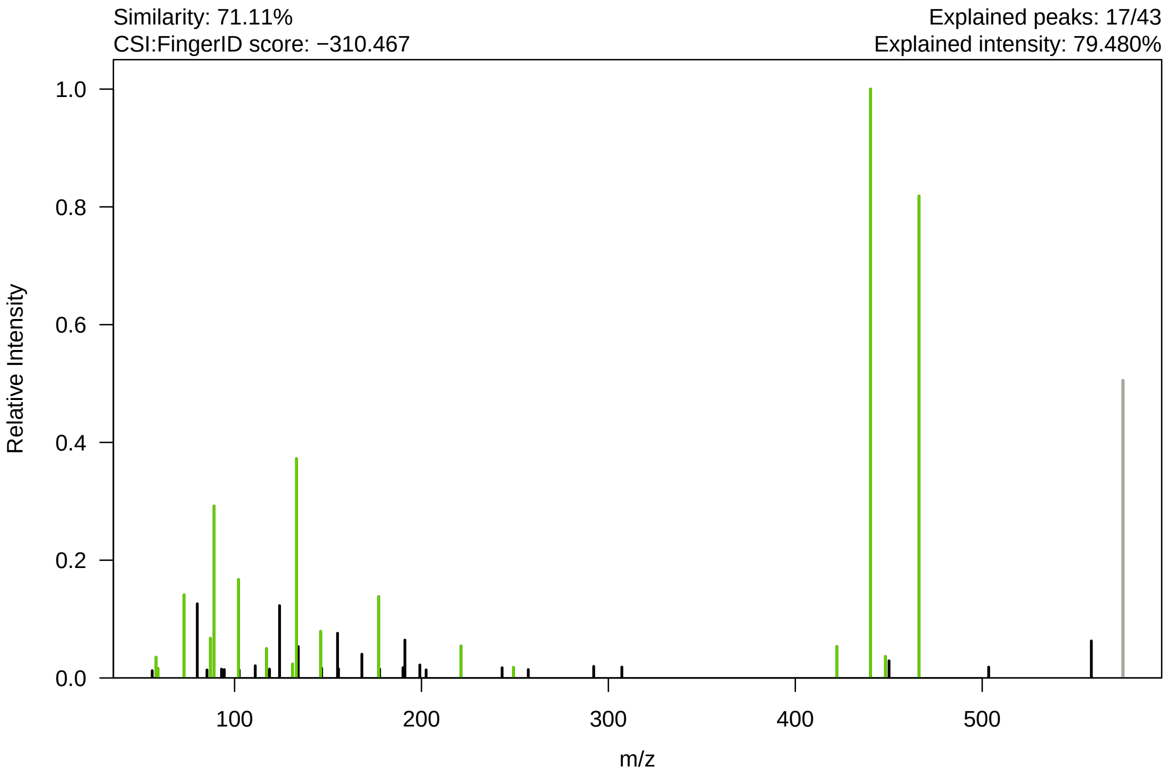

Figure SI-D70: Measured MS2 spectrum. Matching fragments with ortho-hydroxyatorvastatin predicted by SIRIUS/CSI:FingerID are highlighted in green. The molecular ion in gray is not considered.

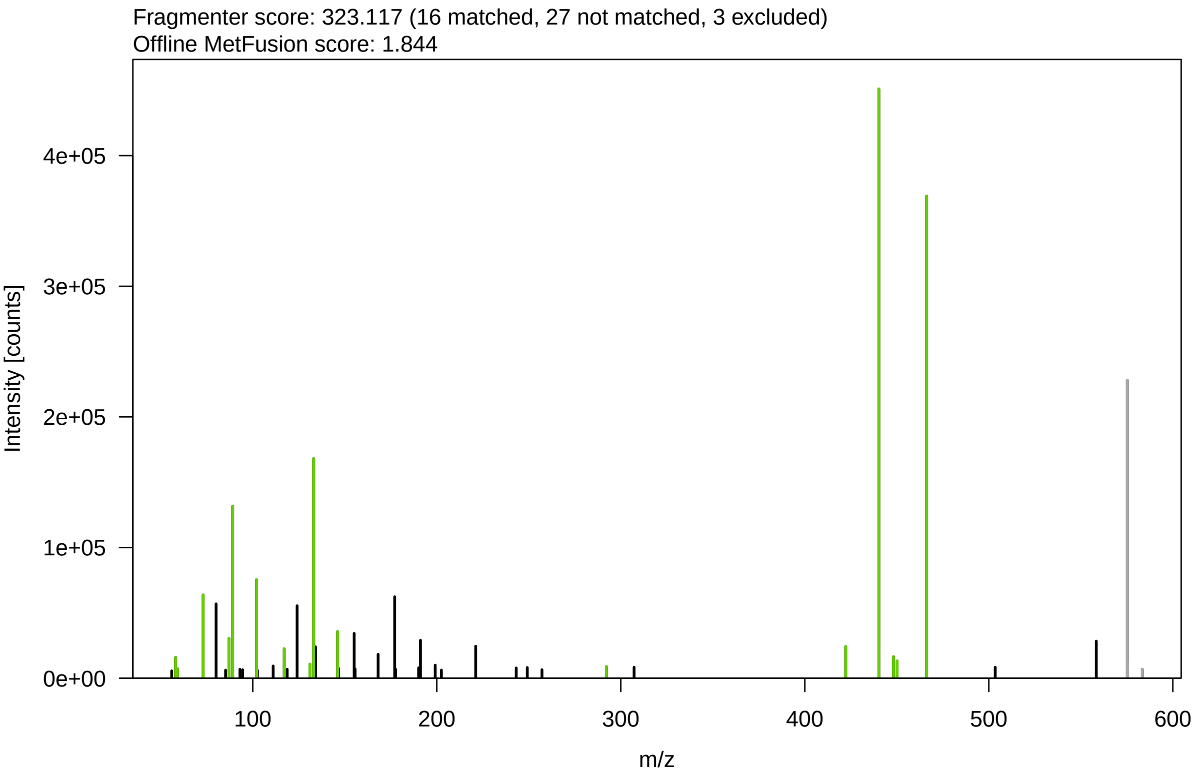

Figure SI-D71: Measured MS2 spectrum. Matching fragments with ortho-hydroxyatorvastatin predicted by MetFrag are highlighted in green. The molecular ion in gray is not considered.

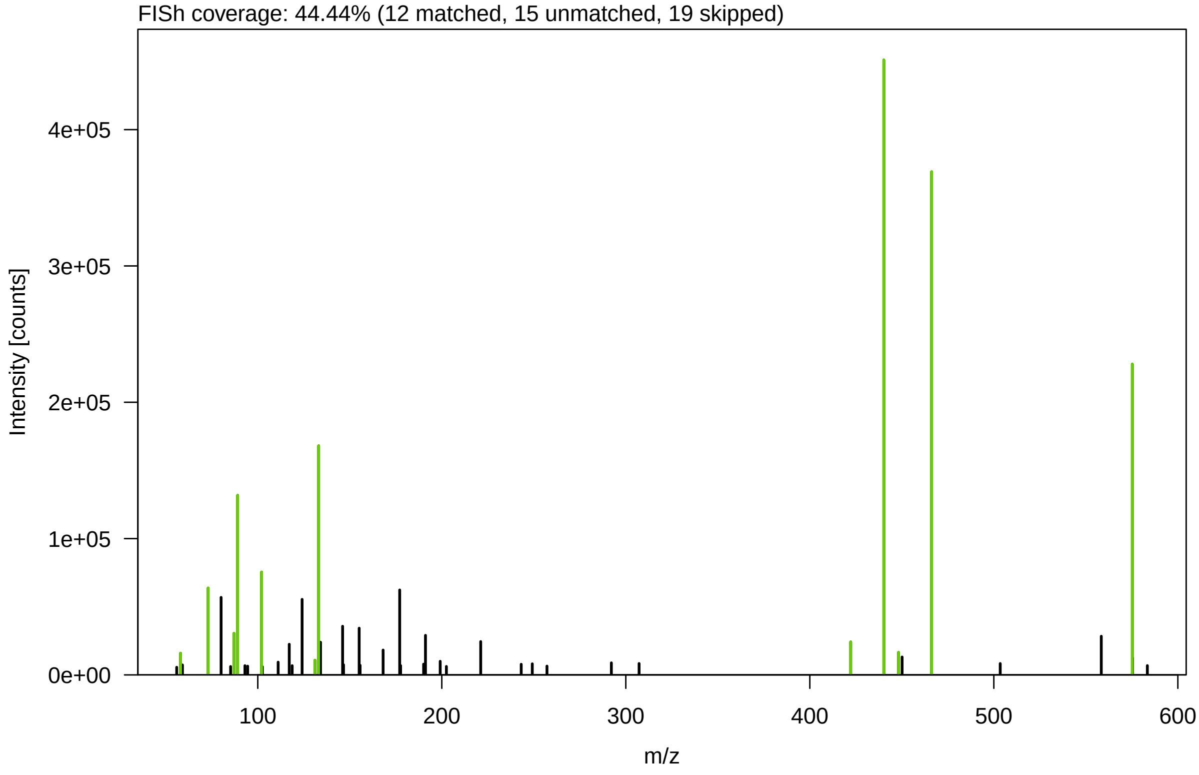

Figure SI-D72: Measured MS2 spectrum. Matching fragments with ortho-hydroxyatorvastatin predicted by FISh Scoring are highlighted in green. Low intensity fragments are not considered and skipped.

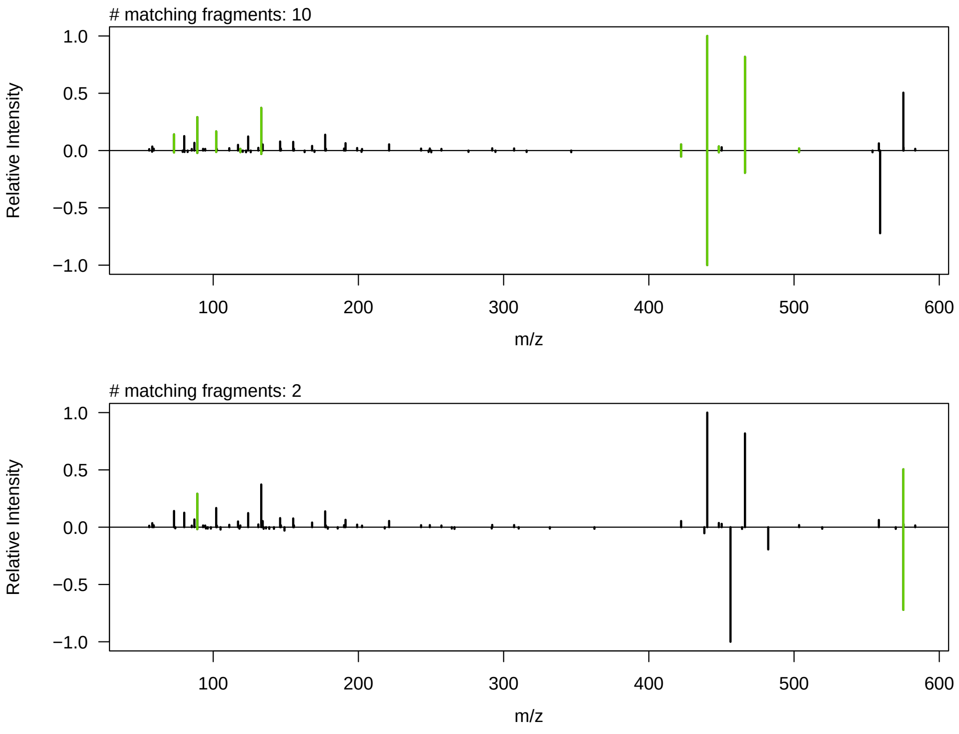

Figure SI-D73: Head to tail plots of ortho-hydroxyatorvastatin and atorvastatin. In the bottom plot, the mass spectrum of atorvastatin is shifted by the mass difference. Matching fragments are highlighted in green.

Table SI-D38: Molecular network results and retention time prediction of ortho-hydroxyatorvastatin.

Comparison with	Atorvastatin
MSn Score	48
Forward coverage	70
Reverse coverage	26
Forward match	16
Reverse match	7
ΔMass [g/mol]	15.9949
Measured retention time [min]	20.3
Predicted $logD_{OW}$ (pH = 2.7)	5.07
Predicted retention time [min]	21.3
Predicted retention time range (95% confidence interval) [min]	16.7-26.0
Predicted retention time range (99% confidence interval) [min]	15.3-27.4

Table SI-D39: Annotated MS2 spectrum of ortho-hydroxyatorvastatin.

m/z	Relative Intensity	Annotation
55.9370	12.43	
58.0416	35.22	$C_3H_5O + H^+$
59.0493	16.18	$C_3H_6O + H^+$
73.0284	141.07	$C_3H_4O_2 + H^+$
80.0544	125.95	
85.2336	13.61	
87.0440	67.43	$C_4H_6O_2 + H^+$
89.0597	291.66	$C_4H_8O_2 + H^+$
93.0235	15.03	
94.4940	14.24	
102.0675	166.99	$C_5H_9O_2 + H^+$
102.4912	13.37	
111.0730	20.72	
117.0910	49.82	$C_6H_{12}O_2 + H^+$
118.6883	15.01	
124.0807	122.75	
131.0695	23.71	$C_6H_{10}O_3 + H^+$
133.0859	372.18	$C_6H_{12}O_3 + H^+$
134.0894	53.13	
146.0935	79.02	$C_7H_{13}O_3 + H^+$
146.5959	16.43	
155.0990	75.93	
155.6011	15.62	

Continued on next page

Table SI-D39: Annotated MS2 spectrum of ortho-hydroxyatorvastatin.(Continued)

168.1070	40.37	
177.1121	137.98	$C_8H_{16}O_4 + H^+$
177.6127	15.14	
190.1192	17.47	
191.1274	64.26	
199.1256	22.12	
202.4927	13.72	
221.1386	54.18	$C_{10}H_{20}O_5 + H^+$
243.1509	17.31	
249.1701	18.00	$C_{12}H_{24}O_5 + H^+$
257.1546	14.29	
292.1496	19.62	$C_{20}H_{18}FN + H^+$
307.2113	18.51	
422.2117	53.54	$C_{29}H_{27}NO_2 + H^+$
440.2230	999.00	$C_{29}H_{29}NO_3 + H^+$
448.1905	36.45	$C_{27}H_{26}FNO_4 + H^+$
450.1560	29.08	$C_{25}H_{22}FN_2O_5 + H^+$
466.2023	817.42	$C_{27}H_{28}FNO_5 + H^+$
503.4649	18.48	
558.3921	62.84	
575.2540	504.82	$C_{33}H_{35}FN_2O_6 + H^+$
575.4059	27.49	
583.4230	15.11	

A reference standard of ortho-hydroxyatorvastatin is commercially available. Figure SI-D74 shows the extracted ion chromatograms of this standard, the sample and the spiked sample, as well as a head to tail plot of the MS2 spectra of the standard and the sample. The first eluting peak in the extracted ion chromatogram originates from an isobaric compound, para-hydroxyatorvastatin (see SI-D2.4.2). In addition, the most intense MS2 fragments in the sample and in the standard are displayed. It becomes visible that the retention times of the sample and the spiked sample are identical and several MS2 fragments between standard and sample match. It can therefore be concluded that the suspected compound is indeed ortho-hydroxyatorvastatin. Correspondingly, the identification confidence can be increased to level 1.

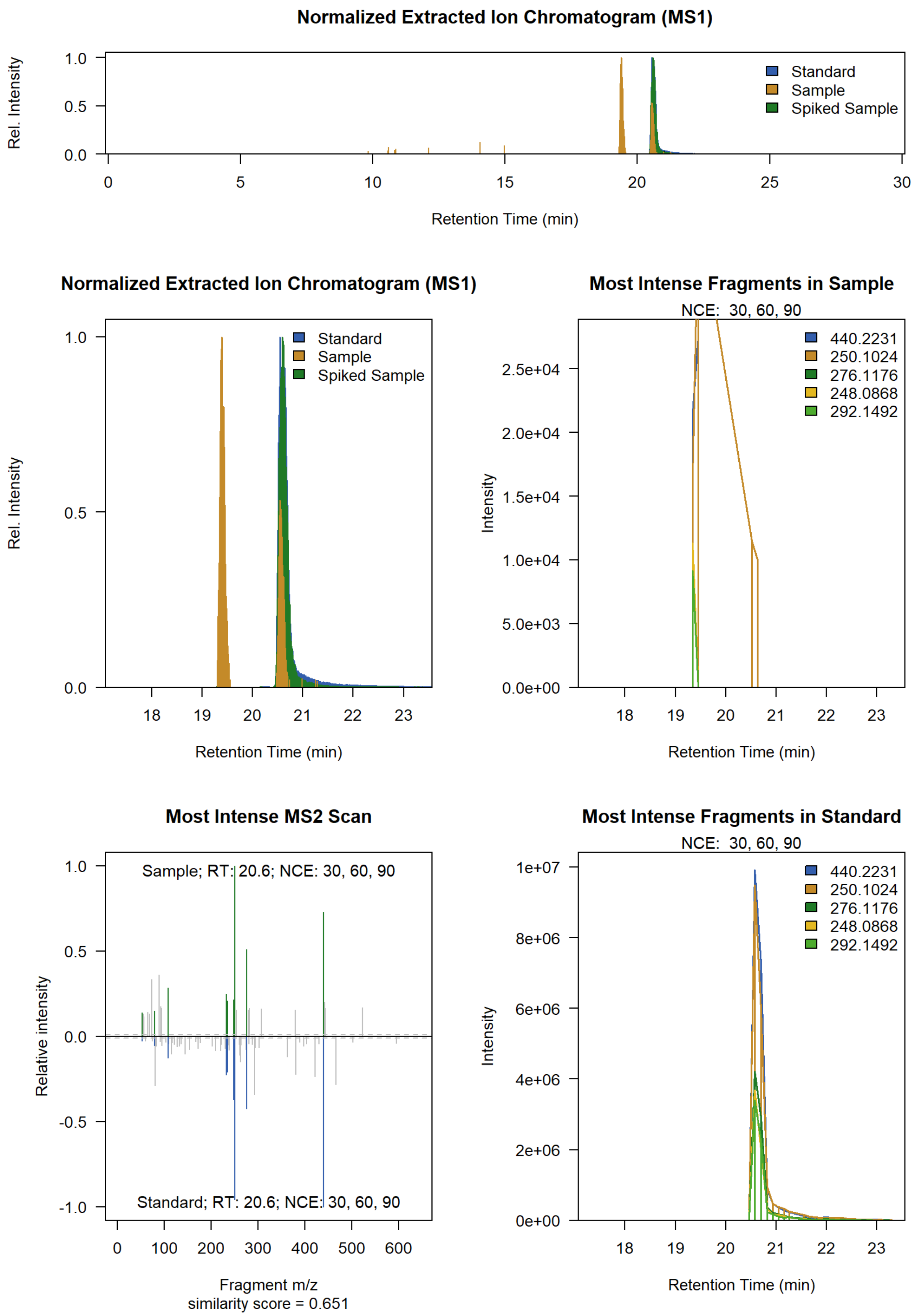

Figure SI-D74: Extracted ion chromatograms of ortho-hydroxyatorvastatin in the reference standard, the sample and the spiked sample, as well as MS2 head to tail plot and most intense MS2 fragments in standard and sample. The first eluting peak originates from para-hydroxyatorvastatin.

SI-D2.4.2 para-Hydroxyatorvastatin

Table SI-D40: Information on identifiers, chemical properties, detection and confidence of identification of para-Hydroxyatorvastatin.

IUPAC Name	7-[2-(4-fluorophenyl)-4-[(4-hydroxyphenyl)carbamoyl]-3-phenyl-5-propan-2-ylpyrrol-1-yl]-3,5-dihydroxyheptanoic acid
Molecular formula	$C_{33}H_{35}FN_2O_6$
Monoisotopic mass [g/mol]	574.2479
Adduct	$[M+H]^+$
Retention time [min]	19.1
SMILES	CC(C)C1=C(C(=C(N1CCC(CC(CC(=O)O)O)O)C2=CC=C(C=C2)F)C3=CC=CC=C3)C(=O)NC4=CC=C(C=C4)O
InChI	InChI=1S/C33H35FN2O6/c1-20(2)31-30(33(42)35-24-12-14-25(37)15-13-24)29(21-6-4-3-5-7-21)32(22-8-10-23(34)11-9-22)36(31)17-16-26(38)18-27(39)19-28(40)41/h3-15,20,26-27,37-39H,16-19H2,1-2H3,(H,35,42)(H,40,41)
InChI-Key	SOZOATLLFFVAPM-UHFFFAOYSA-N
CAS RN	-
Metabolite of	Atorvastatin
Detection frequency	100% (15/15 samples)
Detected in	Altenrhein, Monday-Friday<br>Neugut, Monday-Friday<br>Werdhölzli, Monday-Friday
Intensity	E7
Initial confidence level	level 3
Initial confidence score	0.42
Final confidence level	level 1

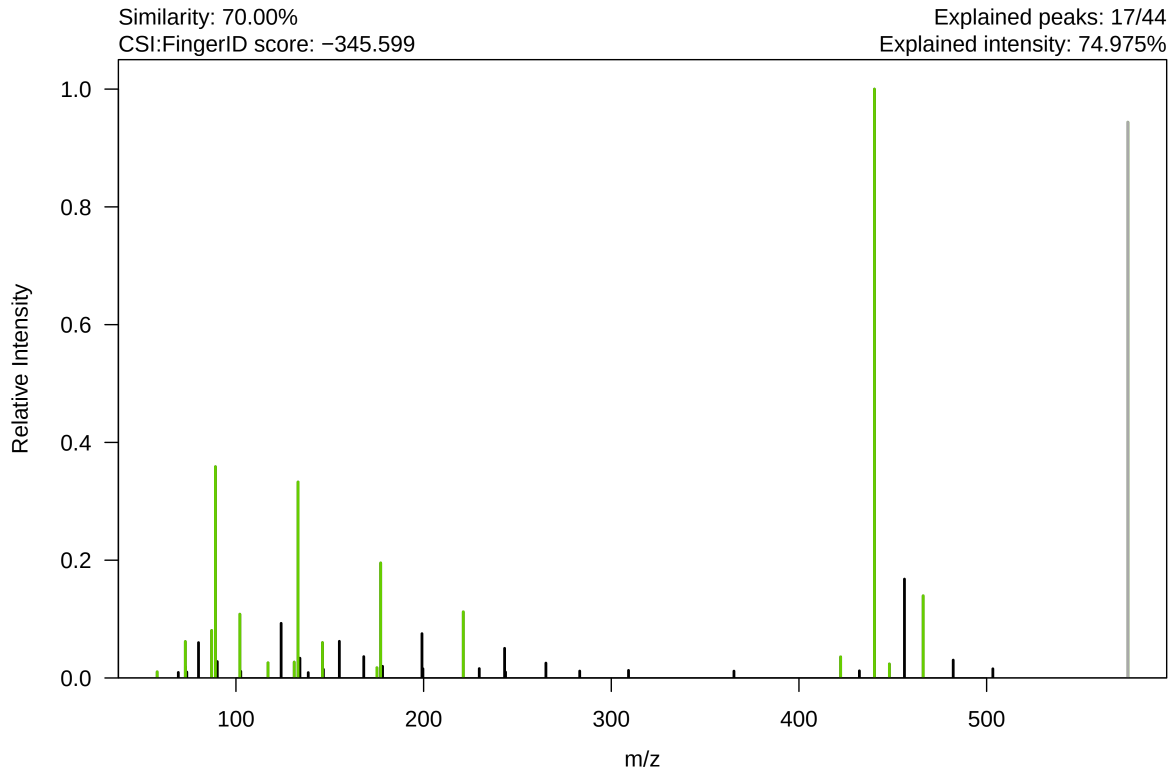

Figure SI-D75: Measured MS2 spectrum. Matching fragments with para-hydroxyatorvastatin predicted by SIRIUS/CSI:FingerID are highlighted in green. The molecular ion in gray is not considered.

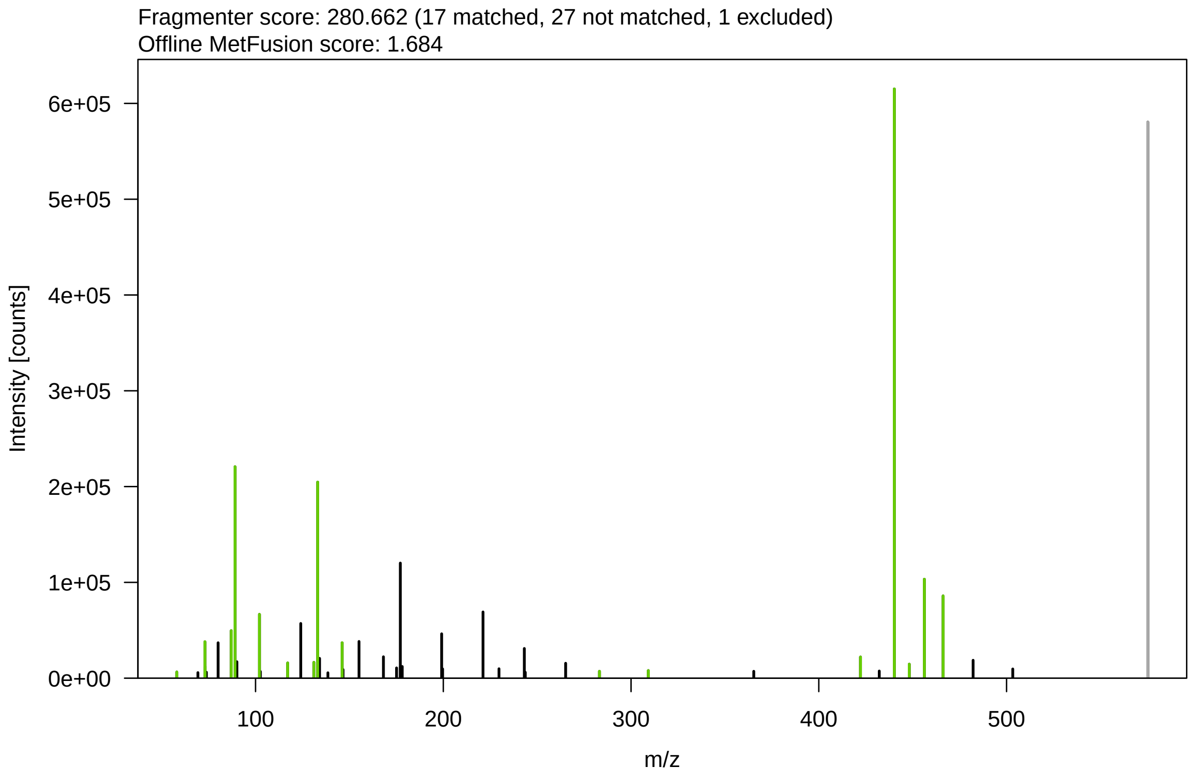

Figure SI-D76: Measured MS2 spectrum. Matching fragments with para-hydroxyatorvastatin predicted by MetFrag are highlighted in green. The molecular ion in gray is not considered.

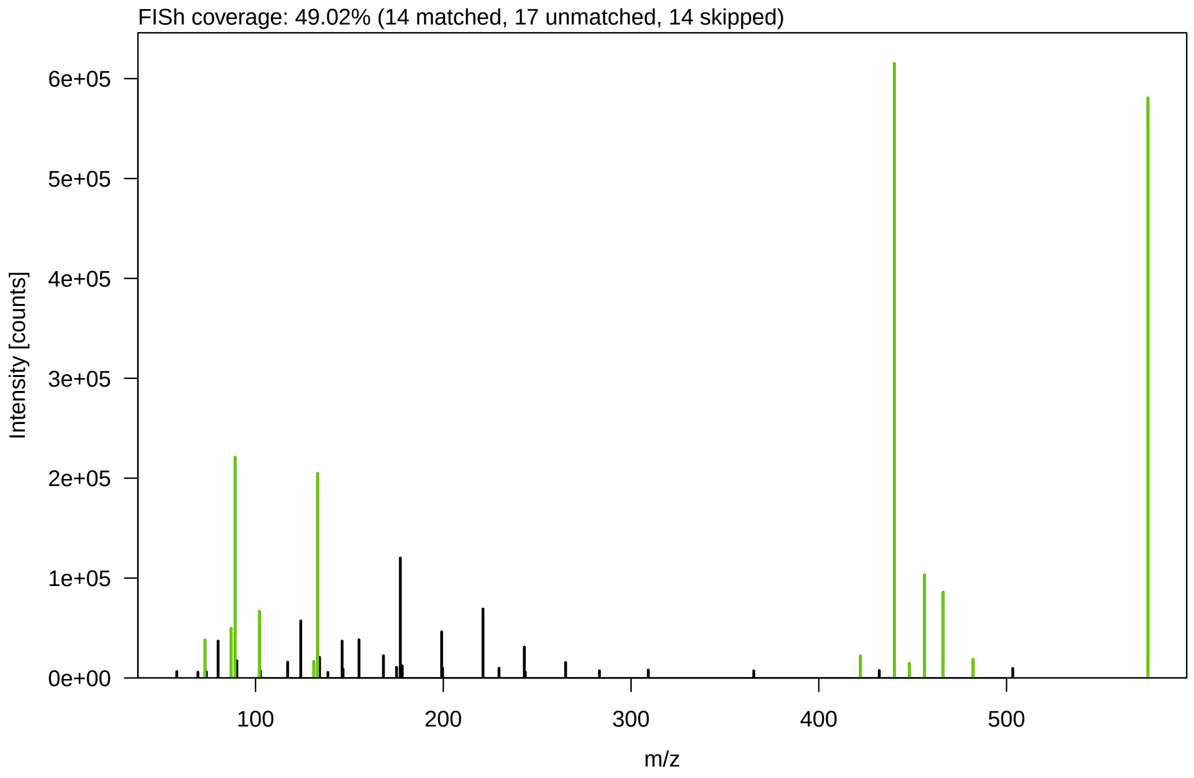

Figure SI-D77: Measured MS2 spectrum. Matching fragments with para-hydroxyatorvastatin predicted by FISh Scoring are highlighted in green. Low intensity fragments are not considered and skipped.

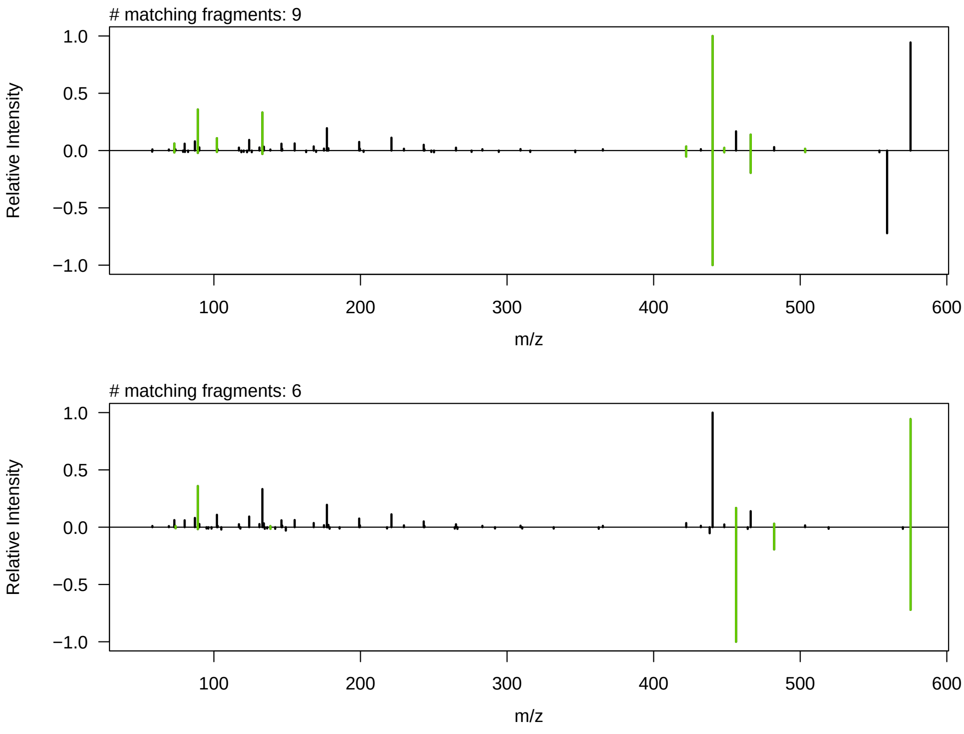

Figure SI-D78: Head to tail plots of para-hydroxyatorvastatin and atorvastatin. In the bottom plot, the mass spectrum of atorvastatin is shifted by the mass difference. Matching fragments are highlighted in green.

Table SI-D41: Molecular network results and retention time prediction of para-hydroxyatorvastatin.

Comparison with	Atorvastatin
MSn Score	57
Forward coverage	78
Reverse coverage	35
Forward match	18
Reverse match	18
ΔMass [g/mol]	15.9949
Measured retention time [min]	19.1
Predicted $logD_{OW}$ (pH = 2.7)	5.07
Predicted retention time [min]	21.3
Predicted retention time range (95% confidence interval) [min]	16.7-26.0
Predicted retention time range (99% confidence interval) [min]	15.3-27.4

Table SI-D42: Annotated MS2 spectrum of para-hydroxyatorvastatin.

m/z	Relative Intensity	Annotation
58.0416	10.48	$C_3H_5O + H^+$
69.2893	9.42	
73.0284	61.82	$C_3H_4O_2 + H^+$
73.0648	19.29	$C_4H_8O + H^+$
73.8111	10.11	
80.0543	60.01	
87.0440	80.60	$C_4H_6O_2 + H^+$
89.0597	358.49	$C_4H_8O_2 + H^+$
90.0629	27.86	
102.0676	108.18	$C_5H_9O_2 + H^+$
102.5694	11.28	
117.0910	25.80	$C_6H_{12}O_2 + H^+$
124.0805	92.73	
131.0702	26.96	$C_6H_{10}O_3 + H^+$
133.0859	332.41	$C_6H_{12}O_3 + H^+$
134.0893	33.58	
138.5269	9.12	
146.0935	60.09	$C_7H_{13}O_3 + H^+$
146.5963	14.63	$C_7H_{13}O_3 + H^+$
155.0991	62.23	
168.1074	36.23	
175.0963	17.36	$C_8H_{14}O_4 + H^+$
177.1121	195.16	$C_8H_{16}O_4 + H^+$

Continued on next page

Table SI-D42: Annotated MS2 spectrum of para-hydroxyatorvastatin.(Continued)

177.1266	19.30	
177.4427	9.28	
178.1149	19.78	
199.1250	75.21	
199.6270	15.72	
221.1382	112.23	$C_{10}H_{20}O_5 + H^+$
229.6520	15.94	
243.1523	50.32	
243.6531	10.04	
265.1641	25.29	
283.1765	11.74	$C_{15}H_{24}NO_4 + H^+$
309.1913	12.88	$C_{17}H_{26}NO_4 + H^+$
365.3628	11.67	
422.2116	35.89	$C_{26}H_{28}FNO_3 + H^+$
432.2097	12.13	
440.2229	999.00	$C_{26}H_{30}FNO_4 + H^+$
448.1934	23.78	$C_{27}H_{26}FNO_4 + H^+$
456.2170	167.80	$C_{26}H_{30}FNO_5 + H^+$
466.2027	139.48	$C_{27}H_{28}FNO_5 + H^+$
482.1979	30.34	$C_{30}H_{26}FN_2O_3 + H^+$
503.3172	15.57	
575.2545	942.78	$C_{33}H_{35}FN_2O_6 + H^+$

A reference standard of para-hydroxyatorvastatin is commercially available. Figure SI-D79 shows the extracted ion chromatograms of this standard, the sample and the spiked sample, as well as a head to tail plot of the MS2 spectra of the standard and the sample. The second eluting peak in the extracted ion chromatogram originates from an isobaric compound, ortho-hydroxyatorvastatin (see SI-D2.4.1). In addition, the most intense MS2 fragments in the sample and in the standard are displayed. It becomes visible that the retention times of the sample and the spiked sample are identical and several MS2 fragments between standard and sample match. It can therefore be concluded that the suspected compound is indeed para-hydroxyatorvastatin. Correspondingly, the identification confidence can be increased to level 1.

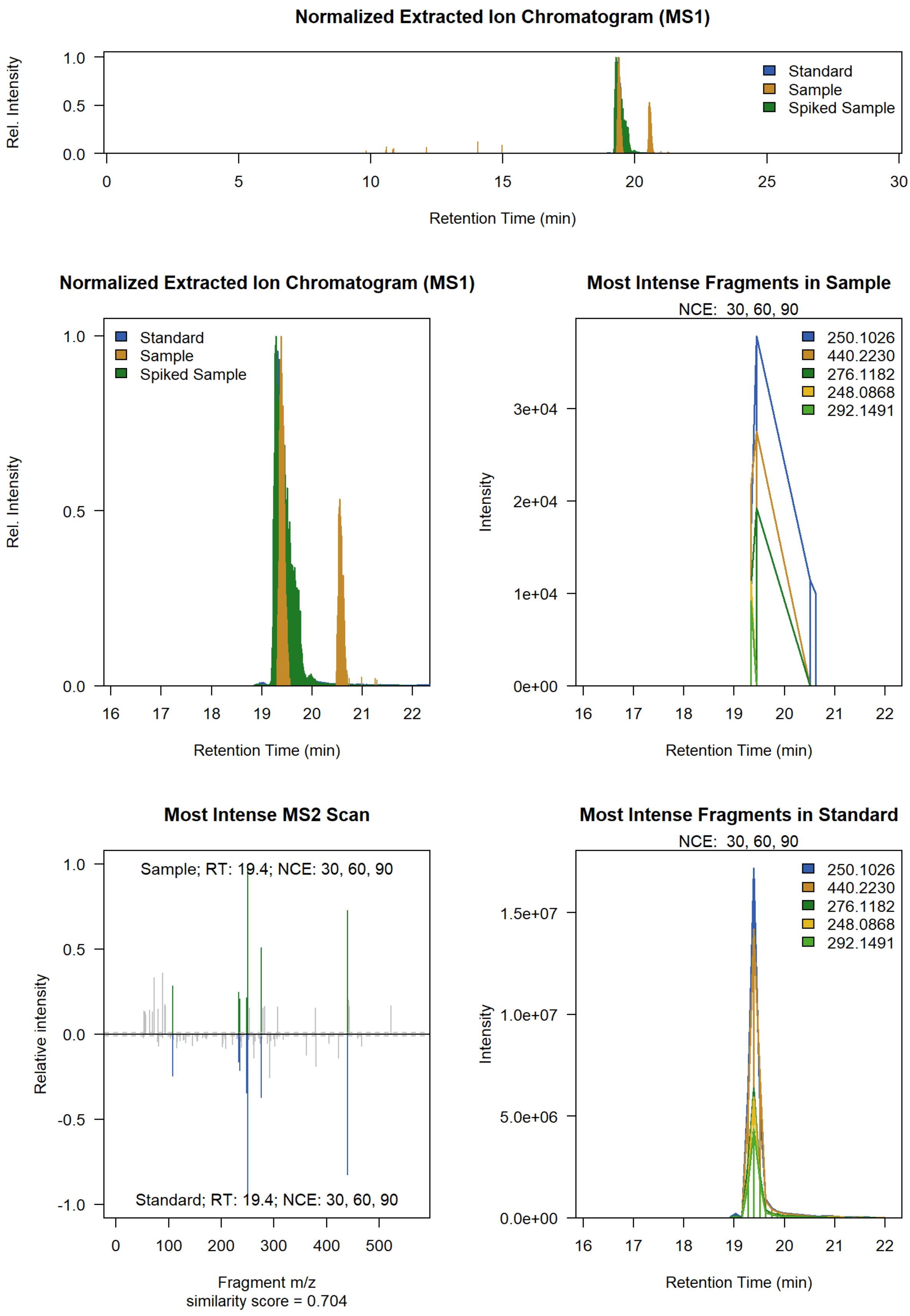

Figure SI-D79: Extracted ion chromatograms of para-hydroxyatorvastatin in the reference standard, the sample and the spiked sample, as well as MS2 head to tail plot and most intense MS2 fragments in standard and sample. The second eluting peak originates from ortho-hydroxyatorvastatin.

SI-D2.5 Azithromycin Metabolites

Azithromycin is a macrolide antibiotic. It is used to treat a wide variety of bacterial infections.[2] Suspect screening in combination with molecular networking enabled the identification of two human metabolites. The identified metabolites are highlighted in the metabolism scheme of azithromycin in Figure SI-D80. No metabolism studies of azithromycin were conducted in human but in ball python.[8] The data in the scheme therefore refer to the metabolism in ball python. A panel of the molecular network is shown in Figure SI-D81. The following subsections give more details on the individual metabolites.

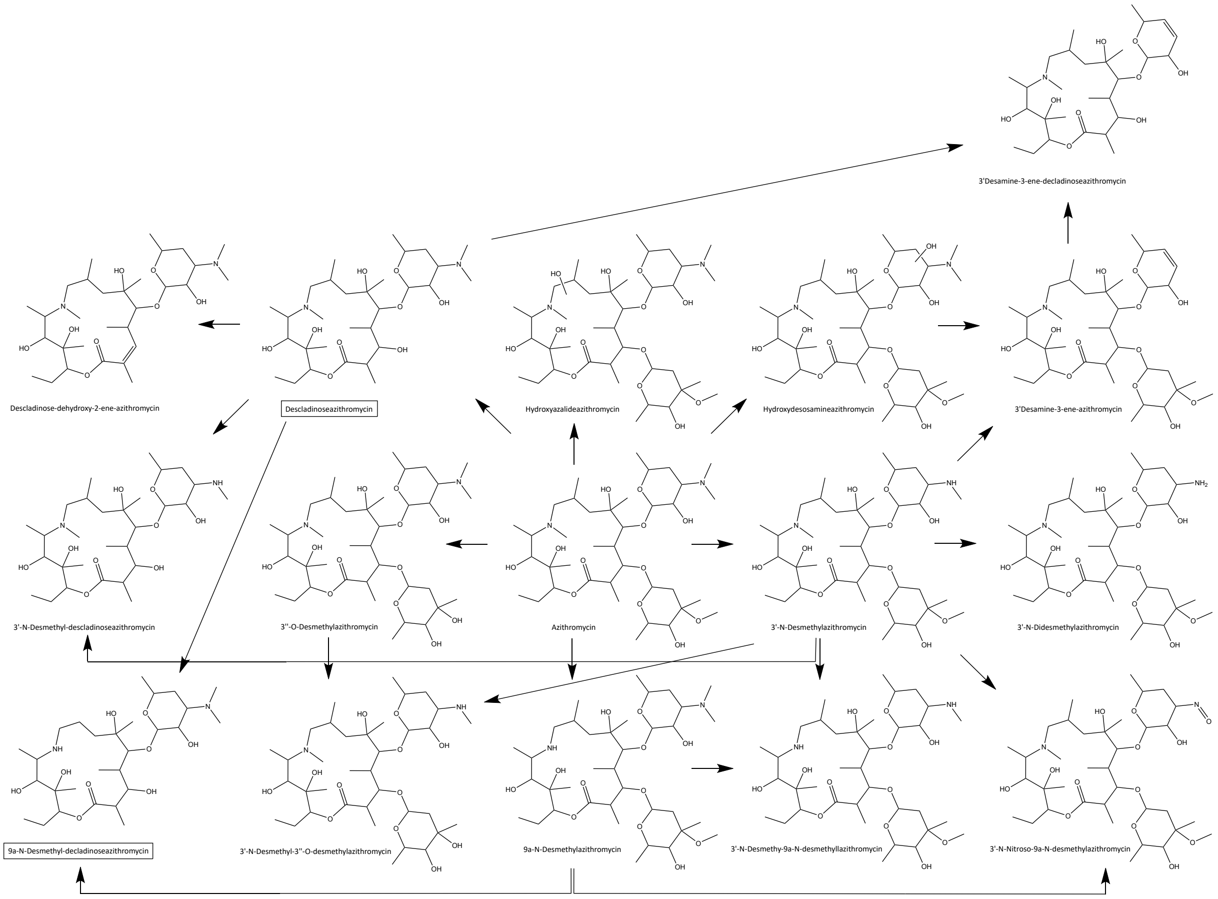

Figure SI-D80: Metabolism of azithromycin in ball python. Framed metabolites were identified during suspect screening. Scheme adapted from.[8]

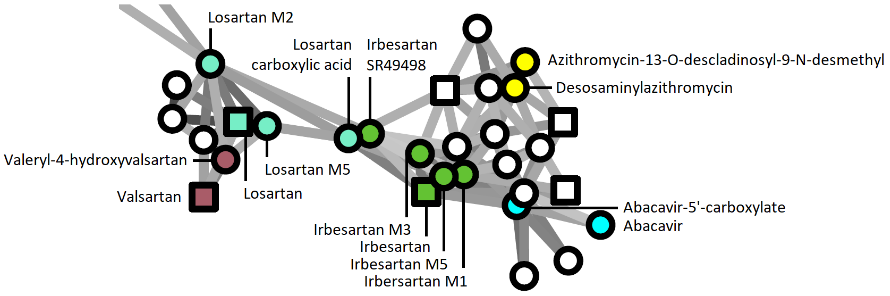

Figure SI-D81: Excerpt of the molecular network showing the azithromycin cluster.

SI-D2.5.1 Azithromycin-13-O-Descladinosyl-9-N-Desmethyl

Table SI-D43: Information on identifiers, chemical properties, detection and confidence of identification of azithromycin-13-O-descladinosyl-9-N-desmethyl.

IUPAC Name	(2*R*,3*S*,4*R*,5*R*,8*R*,10*R*,11*R*,12*S*,13*S*,14*R*)-11-[(2*S*,3*R*,4*S*,6*R*)-4-(dimethylamino)-3-hydroxy-6-methyloxan-2-yl]oxy-2-ethyl-3,4,10,13-tetrahydroxy-3,5,8,10,12,14-hexamethyl-1-oxa-6-azacyclopentadecan-15-one
Molecular formula	$C_{29}H_{56}N_2O_9$
Monoisotopic mass [g/mol]	576.3986
Adduct	$[M+H]^+$
Retention time [min]	24.2
SMILES	CC[C@@H]1[C@@]([C@@H]([C@H](NC[C@@H](C[C@@]([C@@H]([C@H]([C@@H]([C@H](C(=O)O1)C)O)C)O[C@H]2[C@@H]([C@H](C[C@H](O2)C)N(C)C)O)(C)O)C)C)O)(C)O
InChI	InChI=1S/C29H56N2O9/c1-11-21-29(8,37)24(34)19(6)30-14-15(2)13-28(7,36)25(17(4)22(32)18(5)26(35)39-21)40-27-23(33)20(31(9)10)12-16(3)38-27/h15-25,27,30,32-34,36-37H,11-14H2,1-10H3/t15-,16-,17+,18-,19-,20+,21-,22+,23-,24-,25-,27+,28-,29-/m1/s1
InChI-Key	LOMZTTMTKSVHAG-NHUOXGILSA-N
CAS RN	-
Metabolite of	Azithromycin
Detection frequency	100% (15/15 samples)
Detected in	Altenrhein, Monday-Friday<br>Neugut, Monday-Friday<br>Werdhölzli, Monday-Friday
Intensity	E6-E7
Initial confidence level	level 3
Initial confidence score	0.25
Final confidence level	level 4

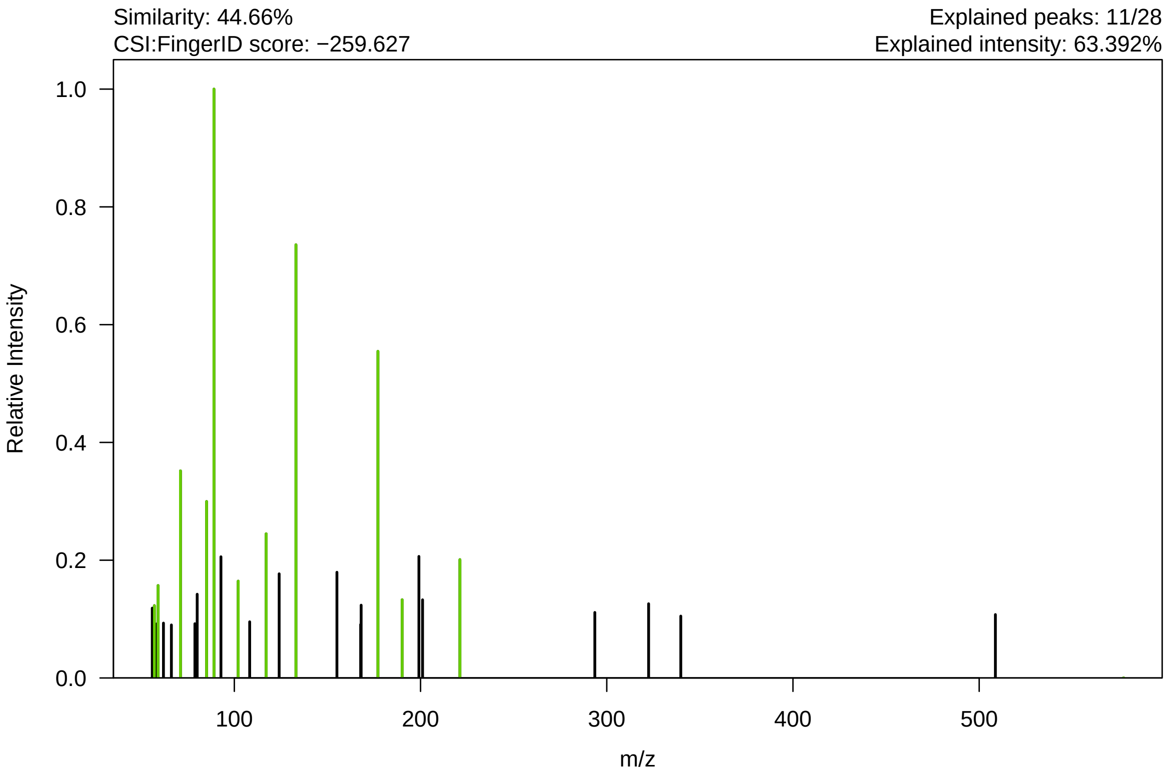

Figure SI-D82: Measured MS2 spectrum. Matching fragments with azithromycin-13-O-descladinosyl-9-N-desmethyl predicted by SIRIUS/CSI:FingerID are highlighted in green.

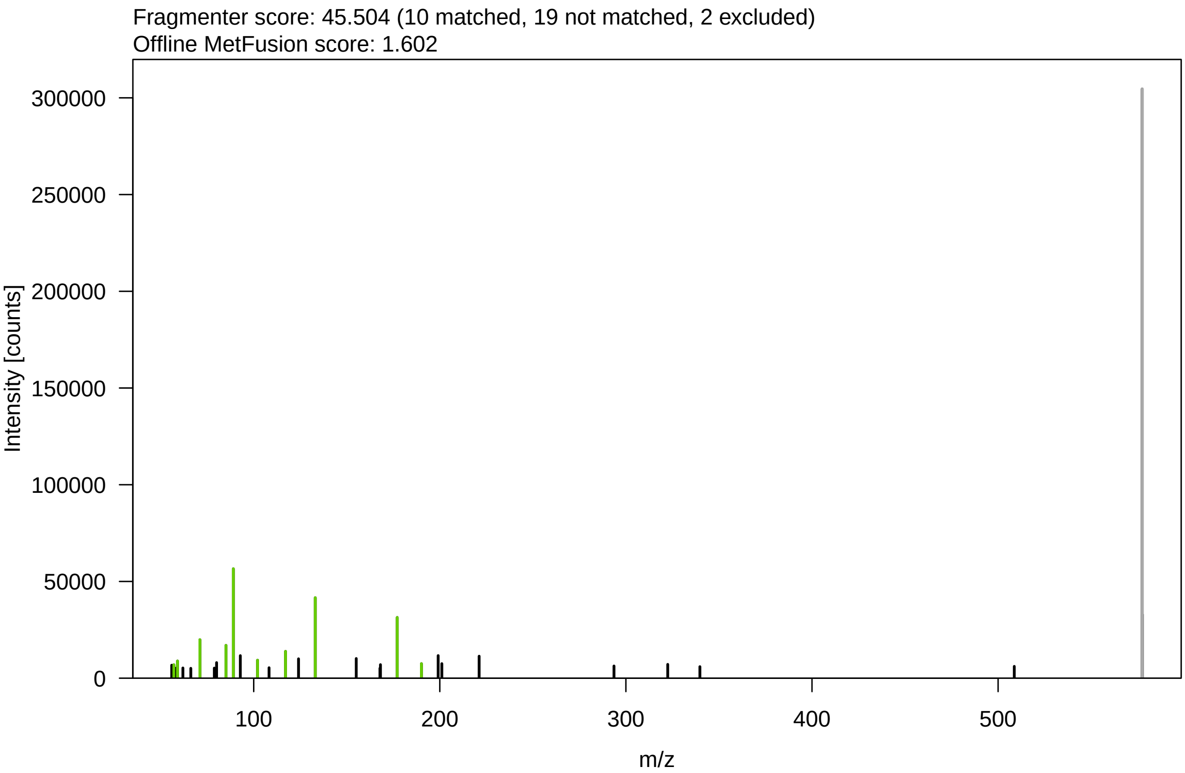

Figure SI-D83: Measured MS2 spectrum. Matching fragments with azithromycin-13-O-descladinosyl-9-N-desmethyl predicted by MetFrag are highlighted in green. The molecular ion in gray is not considered.

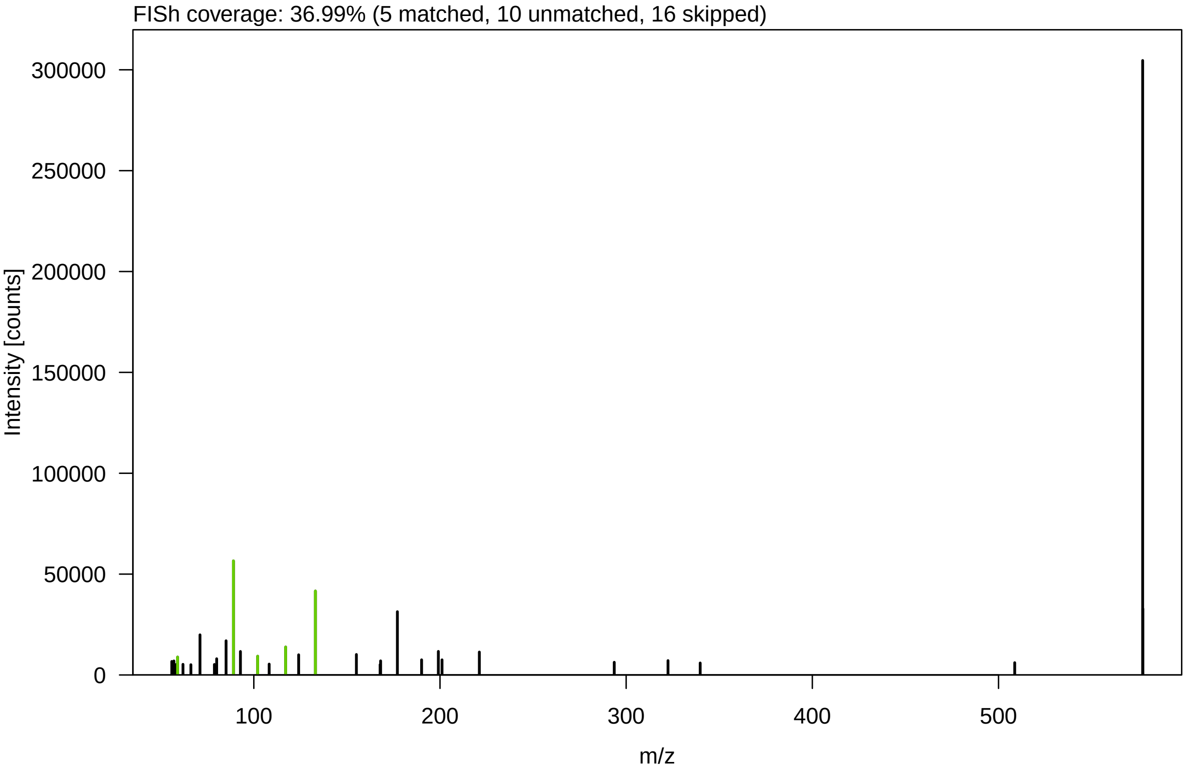

Figure SI-D84: Measured MS2 spectrum. Matching fragments with azithromycin-13-O-descladinosyl-9-N-desmethyl predicted by FISh Scoring are highlighted in green. Low intensity fragments are not considered and skipped.

Table SI-D44: Retention time prediction of azithromycin-13-O-descladinosyl-9-N-desmethyl.

Measured retention time [min]	24.2
Predicted $\log D_{OW}$ (pH = 2.7)	-5.78
Predicted retention time [min]	7.2
Predicted retention time range (95% confidence interval) [min]	2.6-11.8
Predicted retention time range (99% confidence interval) [min]	1.1-13.3

Table SI-D45: Annotated MS2 spectrum of azithromycin-13-O-descladinosyl-9-N-desmethyl.

m/z	Relative Intensity	Annotation
55.9391	22.01	
57.0702	22.79	$C_4H_8 + H^+$
58.2532	17.03	
59.0494	29.12	$C_3H_6O + H^+$
61.9495	17.29	
66.2050	16.71	
71.0855	65.25	$C_5H_{10} + H^+$
78.8374	17.10	
80.0542	26.38	
85.1010	55.59	$C_6H_{12} + H^+$
89.0596	185.49	$C_4H_8O_2 + H^+$
92.8137	38.18	
102.0674	30.50	$C_5H_9O_2 + H^+$
108.2457	17.70	
117.0908	45.43	$C_6H_{12}O_2 + H^+$
124.0807	32.82	
133.0858	136.47	$C_6H_{12}O_3 + H^+$
155.0989	33.30	
167.8377	16.85	
168.1058	22.94	
177.1120	102.90	$C_8H_{16}O_4 + H^+$
190.1194	24.65	$C_9H_{17}O_4 + H^+$
199.1246	38.29	
201.0868	24.63	
221.1379	37.28	$C_{10}H_{20}O_5 + H^+$
293.5975	20.64	
322.4786	23.38	
339.7577	19.50	
508.6971	19.98	
577.4219	999.00	$C_{29}H_{56}N_2O_9 + H^+$
577.5139	107.78	

A reference standard of azithromycin-13-O-descladinosyl-9-N-desmethyl is not commercially available, but of a structurally similar compound, azithromycin-13-O-descladinosyl-6-N-desmethyl, which was purchased. Figure SI-D85 shows the extracted ion chromatograms of this standard, the sample and the spiked sample. It becomes visible that the standard elutes more than ten minutes earlier than the compound suspected to be azithromycin-13-O-descladinosyl-6/9-N-desmethyl in the sample. Due to the high molecular weight of over 500 g/mol and the ring structure, a late elution was expected. However, the high polarity mediated by the presence of several hydroxy groups and two amine functioanlities, leads overall to an early elution. As a consequence of the non-matching retention time, azithromycin-13-O-descladinosyl-9-N-desmethyl cannot be confirmed and the identification confidence is decreased to level 4 due to the unequivocal molecular formula.

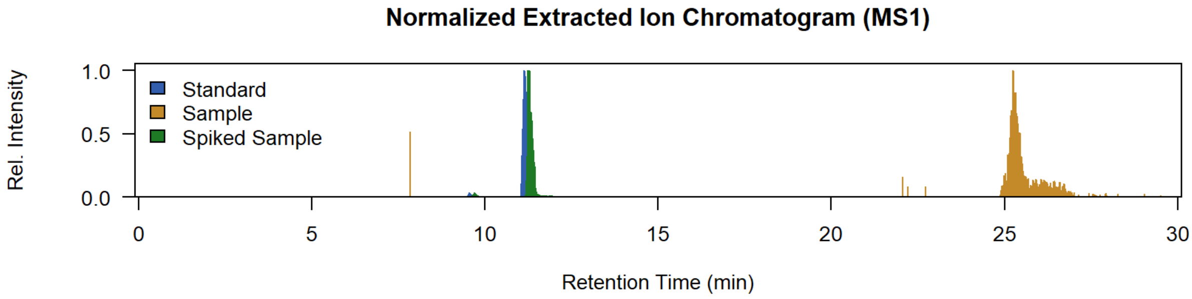

Figure SI-D85: Extracted ion chromatograms of azithromycin-13-O-descladinosyl-6/9-N-desmethyl in the reference standard, the sample and the spiked sample.

SI-D2.5.2 Desosaminylazithromycin

Table SI-D46: Information on identifiers, chemical properties, detection and confidence of identification of desosaminylazithromycin.

IUPAC Name	(2*R*,3*S*,4*R*,5*R*,8*R*,10*R*,11*R*,12*S*,13*S*,14*R*)-11-[(2*S*,3*R*,4*S*,6*R*)-4-(dimethylamino)-3-hydroxy-6-methyloxan-2-yl]oxy-2-ethyl-3,4,10,13-tetrahydroxy-3,5,6,8,10,12,14-heptamethyl-1-oxa-6-azacyclopentadecan-15-one
Molecular formula	$C_{30}H_{58}N_2O_9$
Monoisotopic mass [g/mol]	590.4142
Adduct	$[M+H]^+$
Retention time [min]	24.8
SMILES	CC[C@@H]1[C@@]([C@@H]([C@H](N(C[C@@H](C[C@@]([C@@H]([C@H]([C@@H]([C@H](C(=O)O1)C)O)C)O[C@H]2[C@@H]([C@H](C[C@H](O2)C)N(C)C)O)(C)O)C)C)C)O)(C)O
InChI	InChI=1S/C30H58N2O9/c1-12-22-30(8,38)25(35)20(6)32(11)15-16(2)14-29(7,37)26(18(4)23(33)19(5)27(36)40-22)41-28-24(34)21(31(9)10)13-17(3)39-28/h16-26,28,33-35,37-38H,12-15H2,1-11H3/t16-,17-,18+,19-,20-,21+,22-,23+,24-,25-,26-,28+,29-,30-/m1/s1
InChI-Key	PXDYILJJHOVNLO-NZMWSZMZSA-N
CAS RN	117693-41-1
Metabolite of	Azithromycin
Detection frequency	100% (15/15 samples)
Detected in	Altenrhein, Monday-Friday<br>Neugut, Monday-Friday<br>Werdhölzli, Monday-Friday
Intensity	E7
Initial confidence level	level 3
Initial confidence score	0.32
Final confidence level	level 4

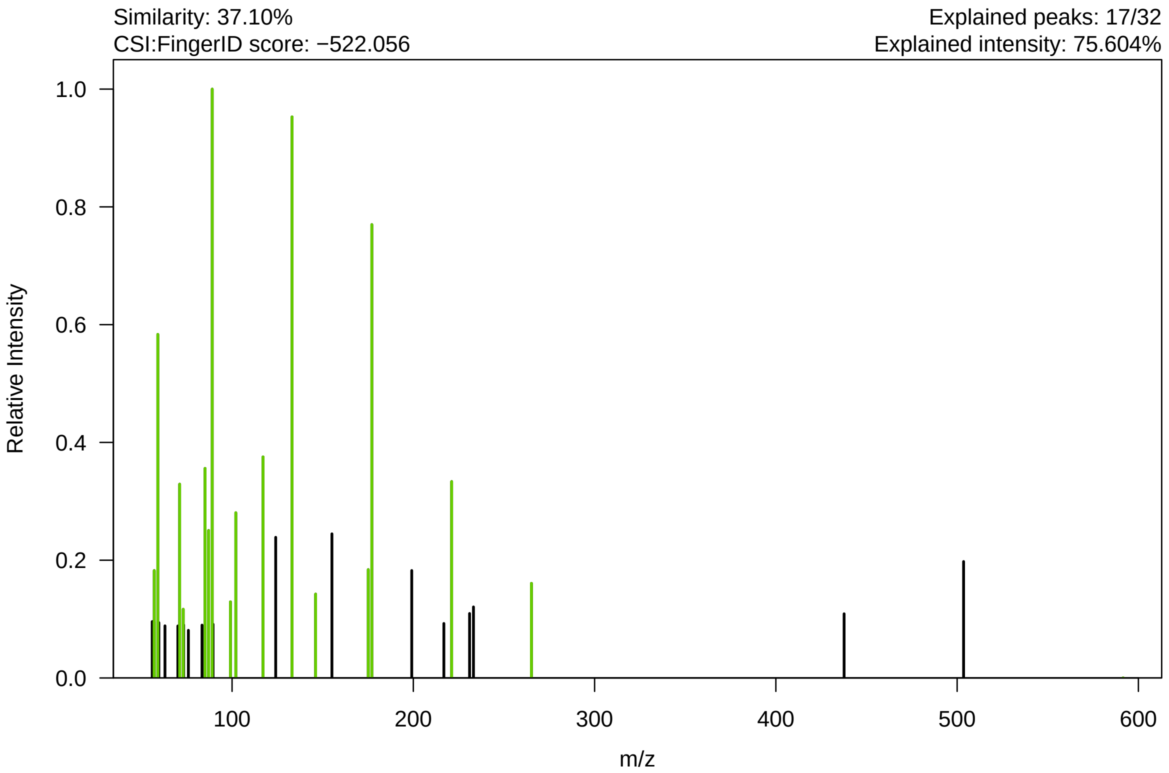

Figure SI-D86: Measured MS2 spectrum. Matching fragments with desosaminylazithromycin predicted by SIRIUS/CSI:FingerID are highlighted in green.

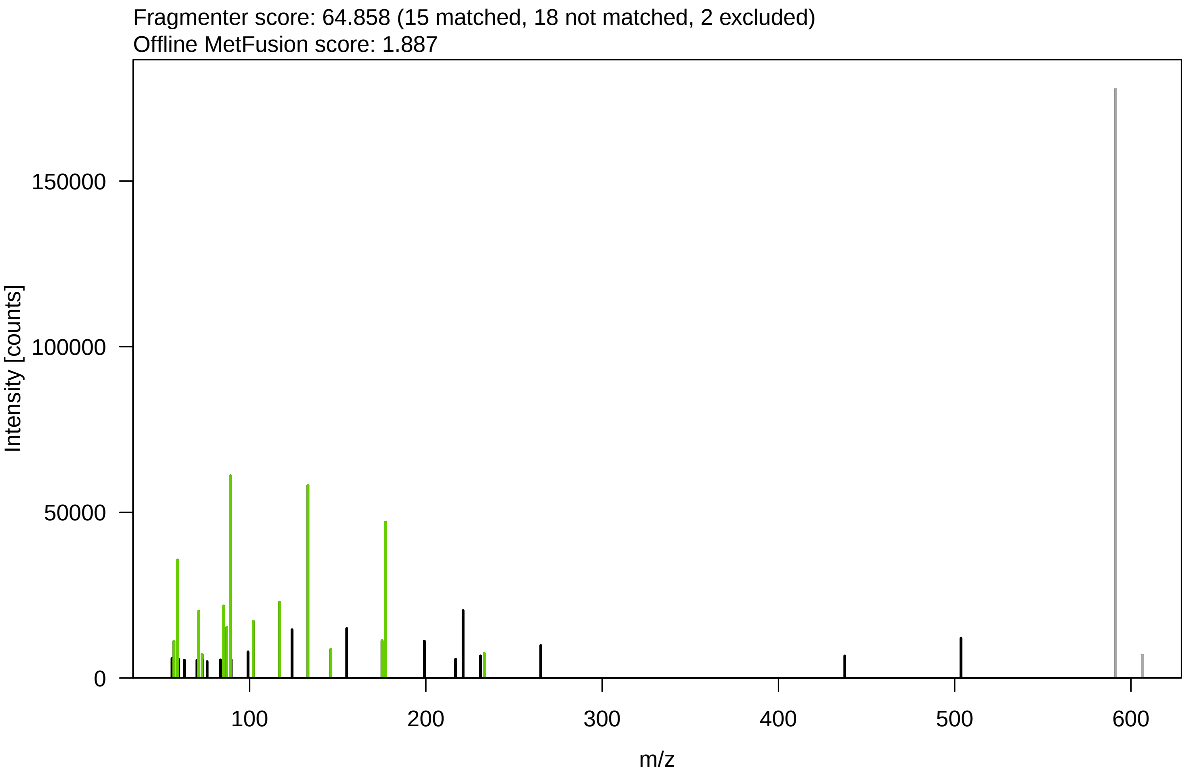

Figure SI-D87: Measured MS2 spectrum. Matching fragments with desosaminylazithromycin predicted by MetFrag are highlighted in green. The molecular ion in gray is not considered.

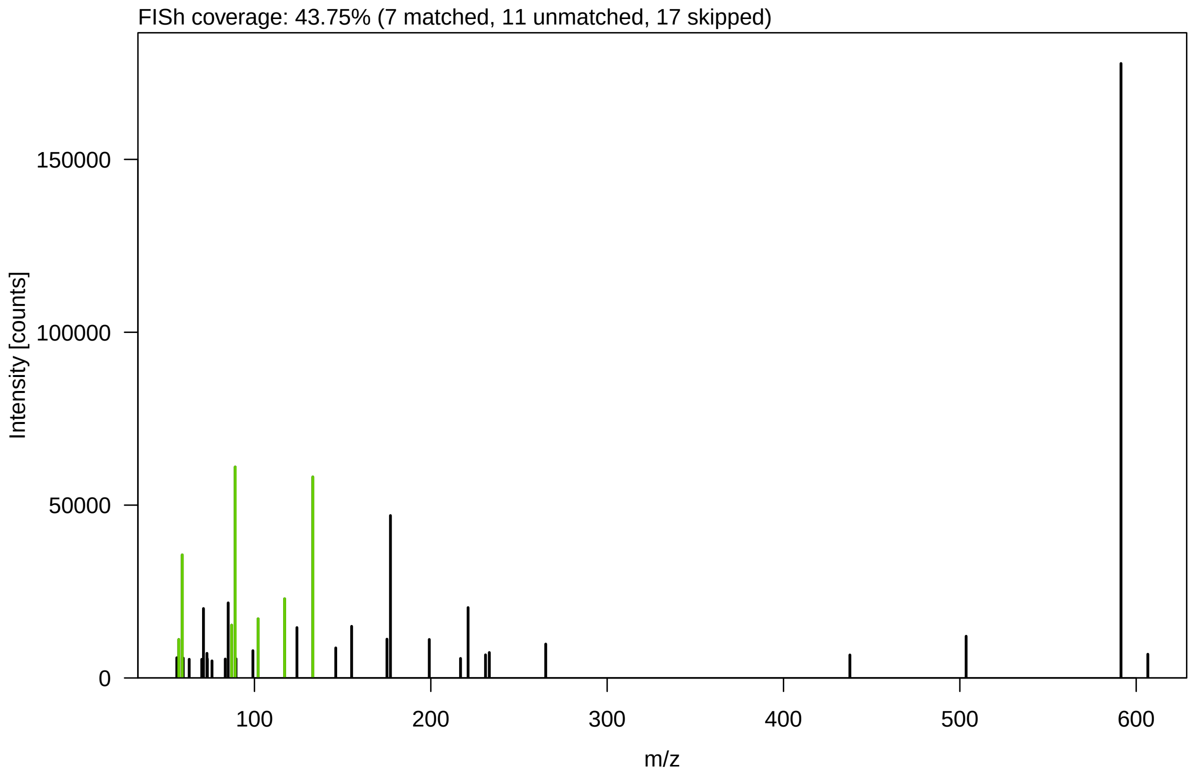

Figure SI-D88: Measured MS2 spectrum. Matching fragments with desosaminylazithromycin predicted by FISh Scoring are highlighted in green. Low intensity fragments are not considered and skipped.

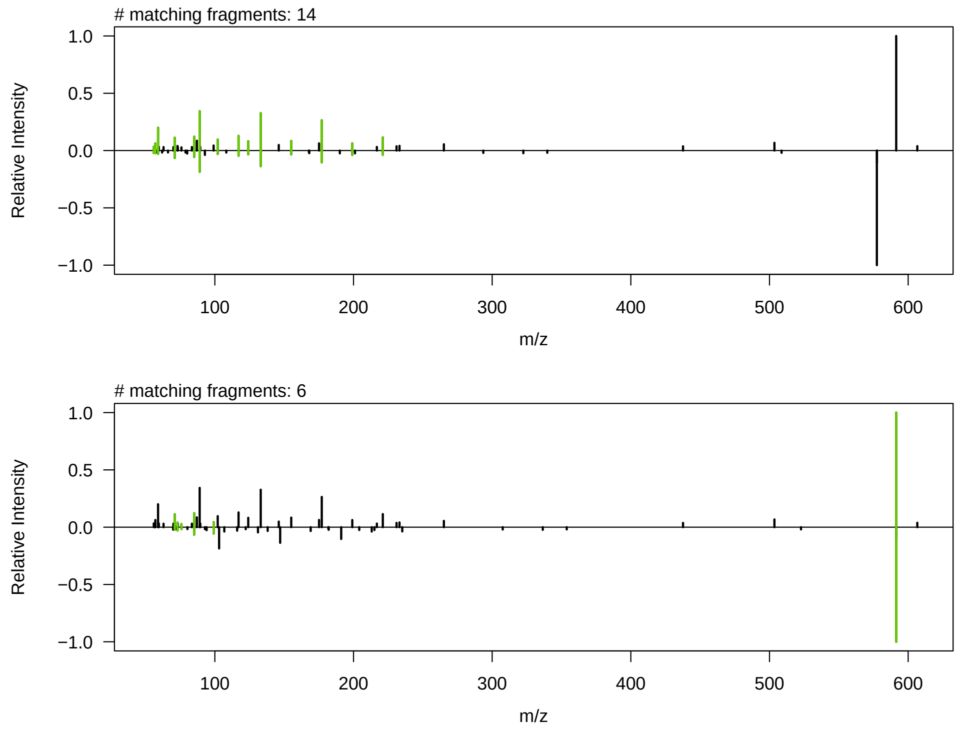

Figure SI-D89: Head to tail plots of desosaminylazithromycin and azithromycin-13-O-descladinosyl-9-N-desmethyl. In the bottom plot, the mass spectrum of azithromycin-13-O-descladinosyl-9-N-desmethyl is shifted by the mass difference. Matching fragments are highlighted in green.

Table SI-D47: Molecular network results and retention time prediction of desosaminylazithromycin.

Comparison with	Azithromycin-13-O-descladinosyl-6-N-desmethyl
MSn Score	74
Forward coverage	78
Reverse coverage	71
Forward match	57
Reverse match	34
ΔMass [g/mol]	14.0157
Measured retention time [min]	24.8
Predicted $logD_{OW}$ (pH = 2.7)	-5.66
Predicted retention time [min]	7.4
Predicted retention time range (95% confidence interval) [min]	2.7-12.0
Predicted retention time range (99% confidence interval) [min]	1.3-13.5

Table SI-D48: Annotated MS2 spectrum of desosaminylazithromycin.

m/z	Relative Intensity	Annotation
55.9501	32.87	
57.0338	32.87	$C_3H_4O + H^+$
57.0701	62.56	$C_4H_8 + H^+$
59.0495	200.13	$C_3H_6O + H^+$
59.6115	32.22	
62.9958	30.38	
70.0944	30.31	
71.0855	112.89	$C_5H_{10} + H^+$
73.0644	40.04	$C_4H_8O + H^+$
73.2982	30.88	
75.9155	27.77	
83.4364	30.79	
85.1011	122.08	$C_6H_{12} + H^+$
87.0438	85.89	$C_4H_6O_2 + H^+$
89.0596	342.97	$C_4H_8O_2 + H^+$
89.5245	31.29	
99.1169	44.36	$C_7H_{14} + H^+$
102.0675	96.25	$C_5H_9O_2 + H^+$
117.0908	128.77	$C_6H_{12}O_2 + H^+$
124.0807	81.92	
133.0859	326.81	$C_6H_{12}O_3 + H^+$
146.0943	48.86	$C_7H_{13}O_3 + H^+$

Continued on next page

Table SI-D48: Annotated MS2 spectrum of desosaminylazithromycin.(Continued)

155.0988	83.96	
175.1331	63.10	$C_9H_{18}O_3 + H^+$
177.1118	264.05	$C_8H_{16}O_4 + H^+$
199.1250	62.58	
216.8521	31.76	
221.1377	114.41	$C_{10}H_{20}O_5 + H^+$
231.0265	37.57	
233.1753	41.38	$C_{12}H_{24}O_4 + H^+$
265.1647	55.13	$C_{12}H_{24}O_6 + H^+$
437.6468	37.39	
503.5609	67.82	
591.3782	999.00	$C_{30}H_{58}N_2O_9$
606.5948	38.57	

A reference standard of desosaminylazithromycin is commercially available. Figure SI-D90 shows the extracted ion chromatograms of this standard, the sample and the spiked sample. It becomes visible that the standard elutes more than 10 minutes earlier than the compound suspected to be desosaminylazithromycin in the sample. Due to the high molecular weight of over 500 g/mol and the ring structure, a late elution was expected. However, the high polarity mediated by the presence of several hydroxy groups and two amine functioanlities, leads overall to an early elution. As a consequence of the non-matching retention time, desosaminylazithromycin cannot be confirmed and the identification confidence is decreased to level 4 due to the unequivocal molecular formula.

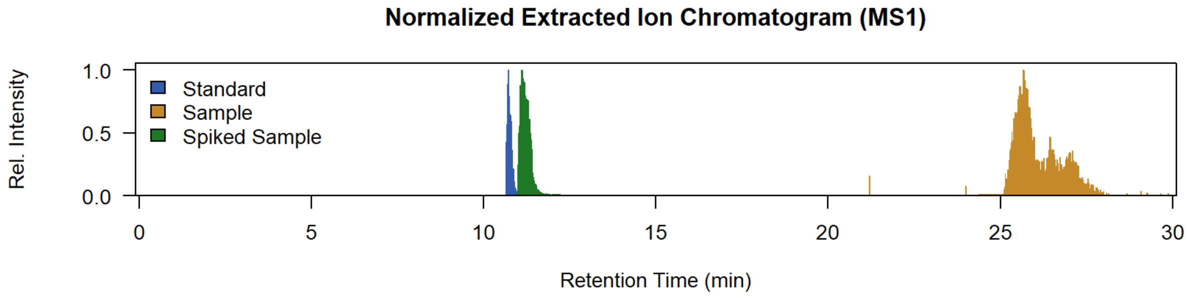

Figure SI-D90: Extracted ion chromatograms of desosaminylazithromycin in the reference standard, the sample and the spiked sample.

SI-D2.6 Caffeine and Theophylline Metabolites

Caffeine is a stimulant, present in tea, coffee and cola beverages, as well as in analgesic drugs and agents to increase alertness. In addition, it is used to prevent and treat pulmonary complications of premature birth.[2] Theophylline, a metabolite of caffeine, is itself used to treat symptoms of asthma and COPD.[2] Suspect screening together with molecular networking led to the identification of several caffeine/theophylline metabolites. The identified metabolites are highlighted in the metabolism scheme of caffeine and theophylline in Figure SI-D91. Excerpts of the molecular networks in positive and negative ionization mode are shown in Figures SI-D92 and SI-D93. The following subsections give more details on the individual metabolites.

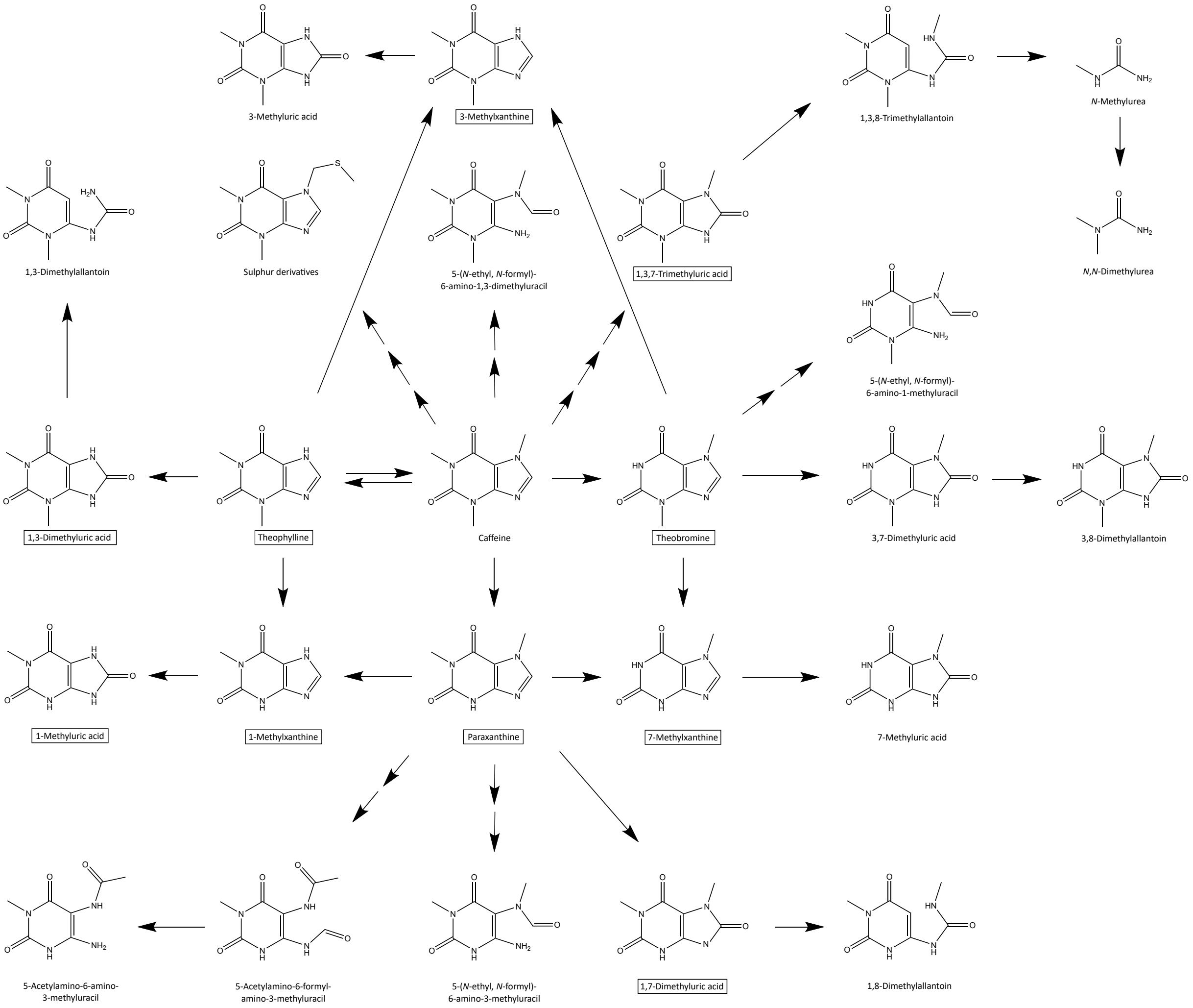

Figure SI-D91: Human metabolism of caffeine and theophylline. Framed metabolites were identified during suspect screening. Scheme adapted from.[9]

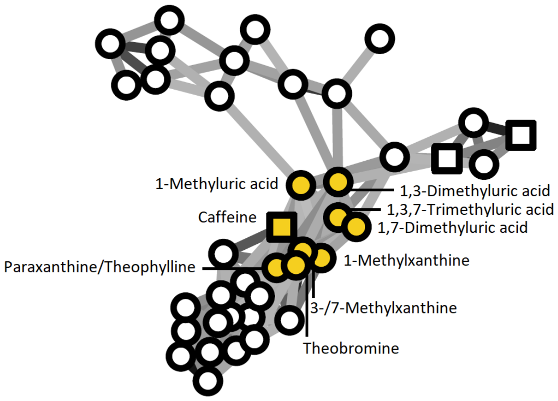

Figure SI-D92: Excerpt of the molecular network showing the caffeine/theophylline cluster in the positive ionization mode.

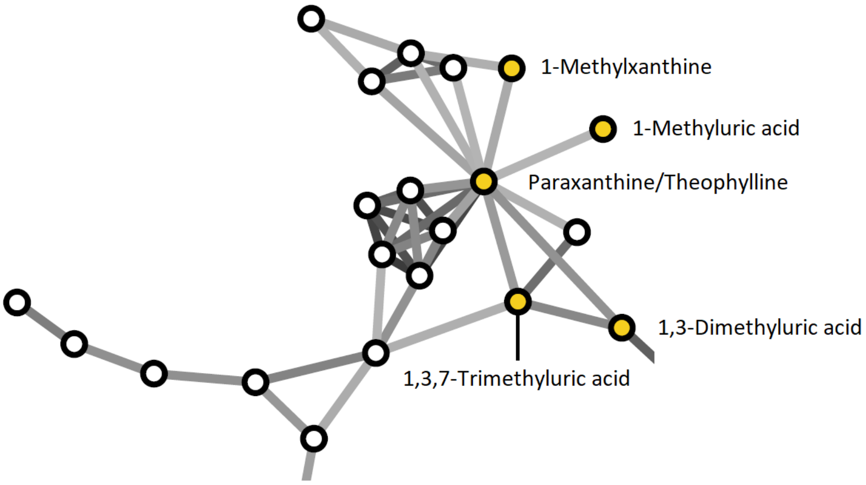

Figure SI-D93: Excerpt of the molecular network showing the caffeine/theophylline cluster in the negative ionization mode.

SI-D2.6.1 Paraxanthine and Theophylline

The chromatographic signal is a double peak, which Compound Discoverer was not able to separate. Therefore, the two compounds are discussed together in this subsection. However, for the confirmation measurements, a better chromatogrpahic resolution was achieved, enabling a separation of the two compounds. To make a semiquantification of the two compounds in the samples possible, 80% of the area was assigned to originate from paraxanthine and 20% from theophylline. This leads to a bias in the quantification approach, but since it is only semiquantitative, the error introduced is expected to be in the same range than the one from MS2Quant.

Table SI-D49: Information on identifiers, chemical properties, detection and confidence of identification of paraxanthine and theophylline.

IUPAC Name	1,7-dimethyl-3*H*-purine-2,6-dione
	1,3-dimethyl-7*H*-purine-2,6-dione
Molecular formula	$C_7H_8N_4O_2$
Monoisotopic mass [g/mol]	180.0647
Adduct	$[M+H]^+$
Retention time [min]	11.9
	12.3
SMILES	CN1C=NC2=C1C(=O)N(C(=O)N2)C
	CN1C2=C(C(=O)N(C1=O)C)NC=N2
InChI	InChI=1S/C7H8N4O2/c1-10-3-8-5-4(10)6(12)11(2)7(13)9-5/h3H,1-2H3,(H,9,13)
	InChI=1S/C7H8N4O2/c1-10-5-4(8-3-9-5)6(12)11(2)7(10)13/h3H,1-2H3,(H,8,9)
InChI-Key	QUNWUDVFRNGTCO-UHFFFAOYSA-N
	ZFXYFBGIUFBOJW-UHFFFAOYSA-N
CAS RN	611-59-6
	58-55-9
Metabolite of	Caffeine
	Parent compound
Detection frequency	100% (15/15 samples)
Detected in	Altenrhein, Monday-Friday
	Neugut, Monday-Friday
	Werdhölzli, Monday-Friday
Intensity	E9
Initial confidence level	level 2a
	level 2a
Initial confidence score	0.76
	0.59
Final confidence level	level 1
	level 1

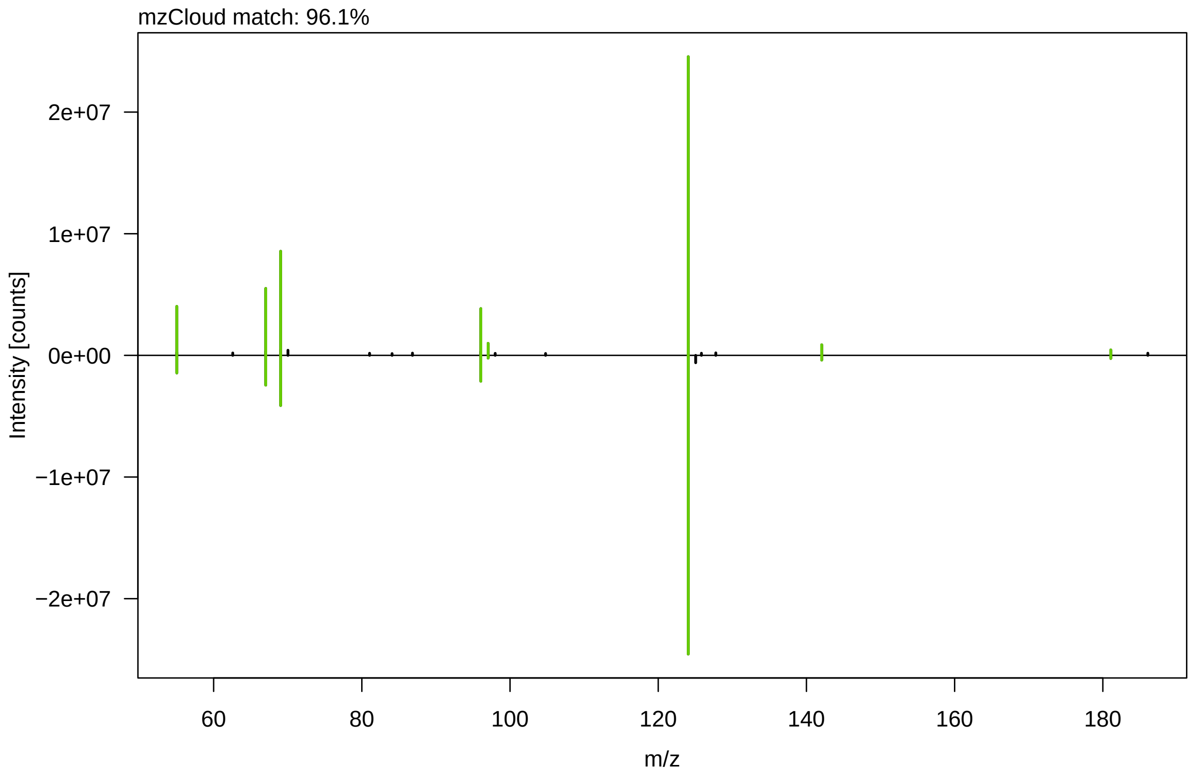

Figure SI-D94: Head to tail plot of measured MS2 spectrum against mzCloud library spectrum of paraxanthine. Matching fragments are highlighted in green.

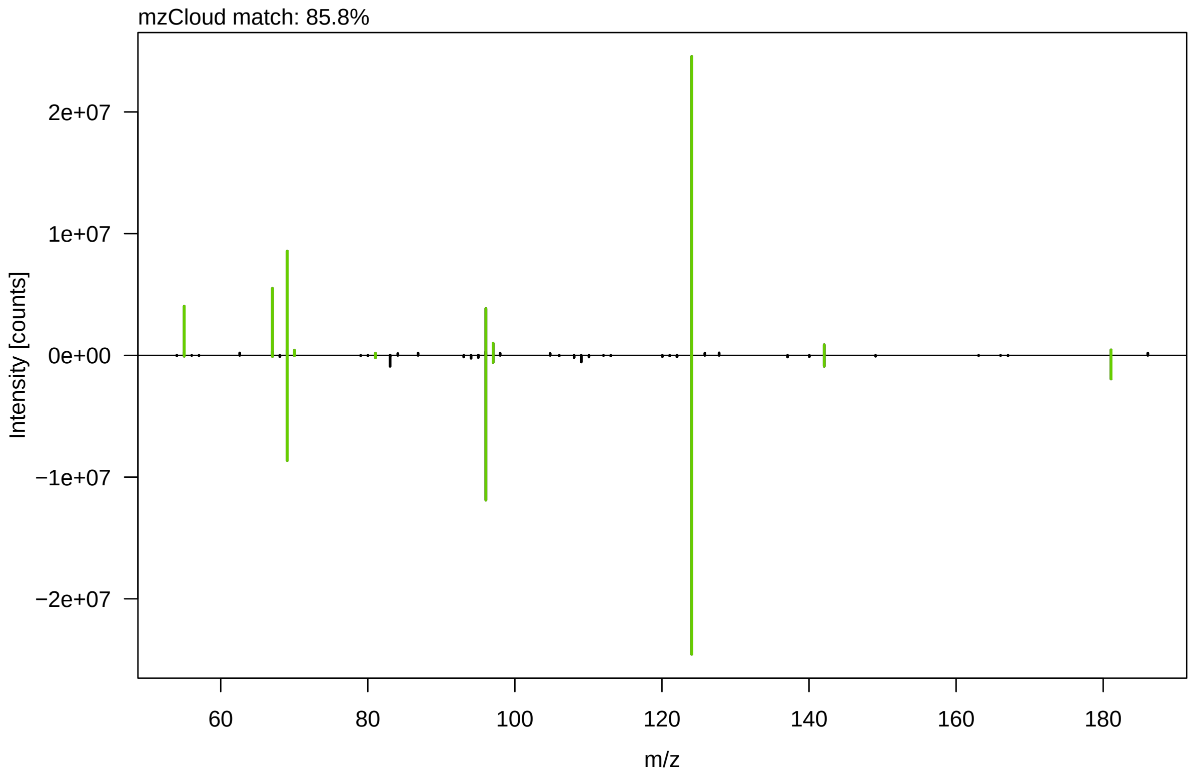

Figure SI-D95: Head to tail plot of measured MS2 spectrum against mzCloud library spectrum of theophylline. Matching fragments are highlighted in green.

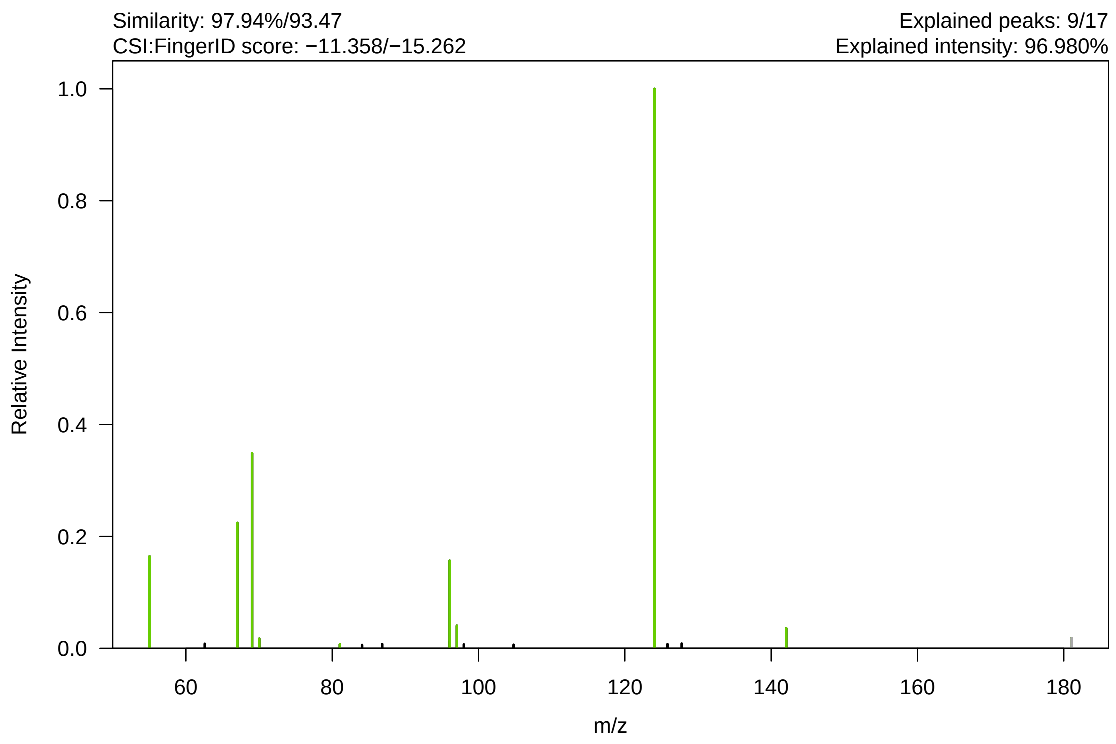

Figure SI-D96: Measured MS2 spectrum. Matching fragments with paraxanthine or theophylline predicted by SIRIUS/CSI:FingerID are highlighted in green. The molecular ion in gray is not considered. The scores refer to paraxanthine and theophylline, respectively.

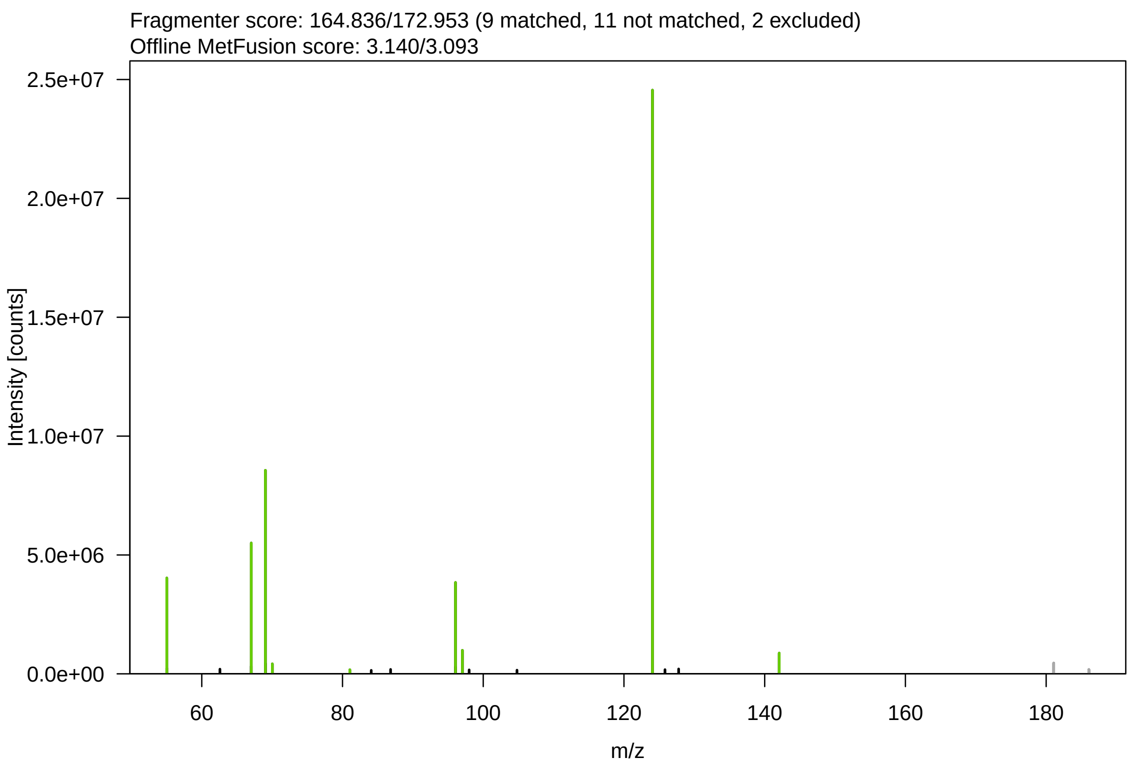

Figure SI-D97: Measured MS2 spectrum. Matching fragments with paraxanthine or theophylline predicted by MetFrag are highlighted in green. The molecular ion in gray is not considered. The scores refer to paraxanthine and theophylline, respectively.

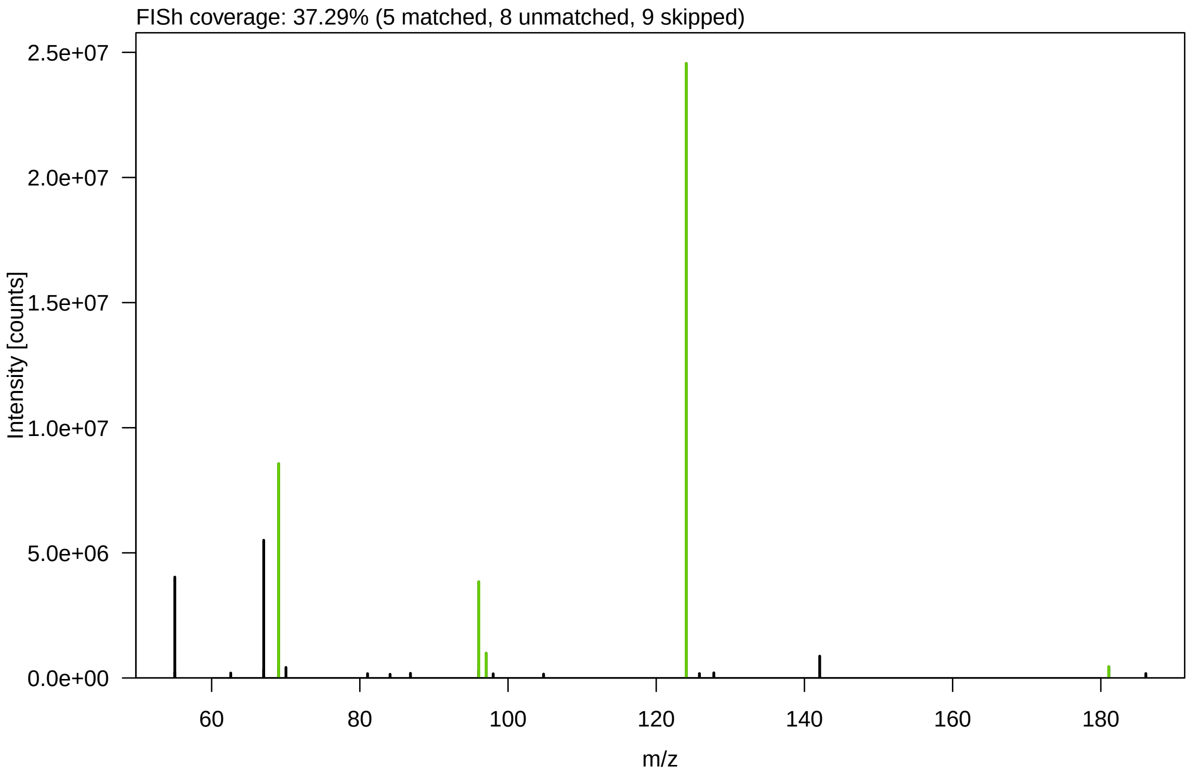

Figure SI-D98: Measured MS2 spectrum. Matching fragments with paraxanthine predicted by FISh Scoring are highlighted in green. Low intensity fragments are not considered and skipped.

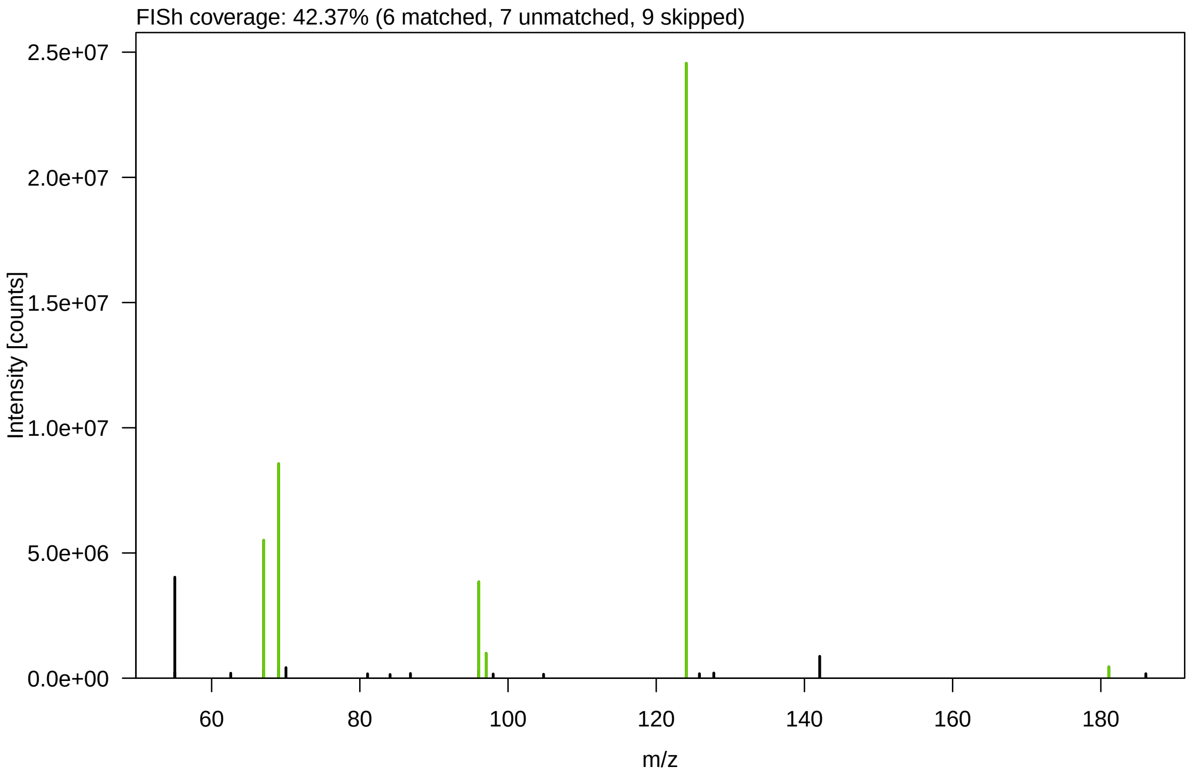

Figure SI-D99: Measured MS2 spectrum. Matching fragments with theophylline predicted by FISh Scoring are highlighted in green. Low intensity fragments are not considered and skipped.

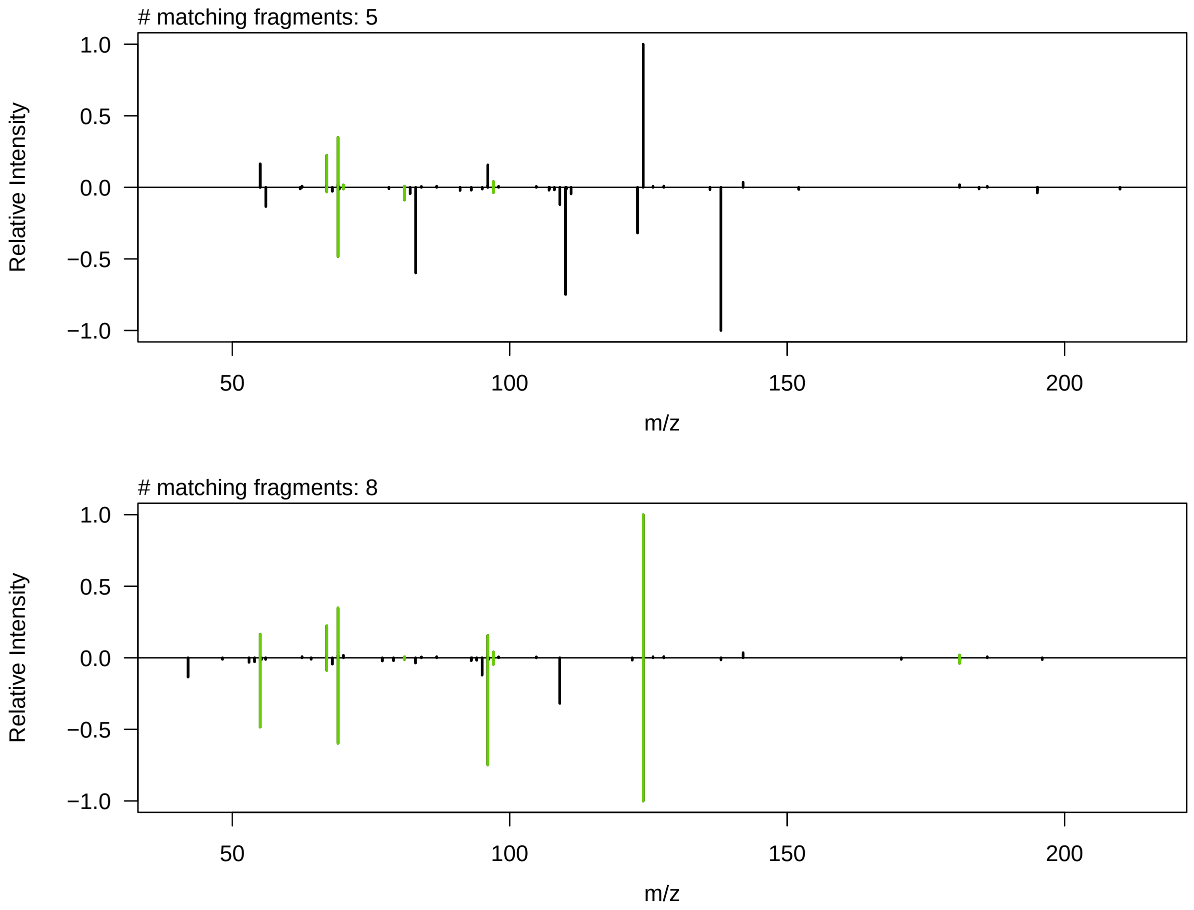

Figure SI-D100: Head to tail plots of paraxanthine/theophylline and caffeine. In the bottom plot, the mass spectrum of caffeine is shifted by the mass difference. Matching fragments are highlighted in green.

Table SI-D50: Molecular network results and retention time prediction of paraxanthine and theophylline.

Comparison with	Caffeine
MSn Score	62
Forward coverage	37
Reverse coverage	86
Forward match	22
Reverse match	42
ΔMass [g/mol]	14.0157
Measured retention time [min]	11.9
Predicted $\log D_{OW}$ (pH = 2.7)	0.25, -0.77
Predicted retention time [min]	15.3, 13.7
Predicted retention time range (95% confidence interval) [min]	10.5-19.6, 9.1-18.3
Predicted retention time range (99% confidence interval) [min]	9.0-21.1, 7.7-19.8

Table SI-D51: Annotated MS2 spectrum of paraxanthine and theophylline, respectively.

m/z	Relative Intensity	Annotation
55.0269	9.90	
55.0295	164.01	$C_2H_2N_2 + H^+$
62.5792	8.01	
67.0258	13.22	
67.0293	223.91	$C_3H_2N_2 + H^+$
69.0412	17.86	
69.0449	348.34	$C_3H_4N_2 + H^+$
70.0287	17.11	$C_3H_3NO + H^+$
81.0448	7.05	$C_4H_4N_2 + H^+$
84.0806	5.98	
86.8323	7.55	
96.0501	11.86	
96.0557	156.39	$C_4H_5N_3 + H^+$
97.0397	40.28	$C_4H_4N_2O + H^+$
97.9960	6.76	
104.7957	6.31	
124.0505	999.00	$C_5H_5N_3O + H^+$
125.8288	7.21	
127.7705	8.34	
142.0615	35.49	$C_5H_7N_3O_2 + H^+$
181.0725	18.29	$C_7H_8N_4O_2 + H^+$
186.0720	7.25	

Reference standards of paraxanthine and theophylline were purchased. Figures SI-D101 and SI-D102 show the extracted ion chromatograms of these standards, the sample and the spiked samples, as well as a head to tail plot of the MS2 spectra of the standard and the sample. In addition, the most intense MS2 fragments in the sample and in the standards are displayed. It becomes visible that for both compounds the retention times of the sample and the spiked sample are identical and that the spectra similarity score between sample and standard is equal to 0.996 for paraxanthine and equal to 0.932 for theophylline, respectively. The majority of the MS2 fragments in the samples can be explained by the respective reference standard. It can therefore be concluded that the suspected compounds are indeed paraxanthine and theophylline. Correspondingly, the identification confidences can be increased to level 1.

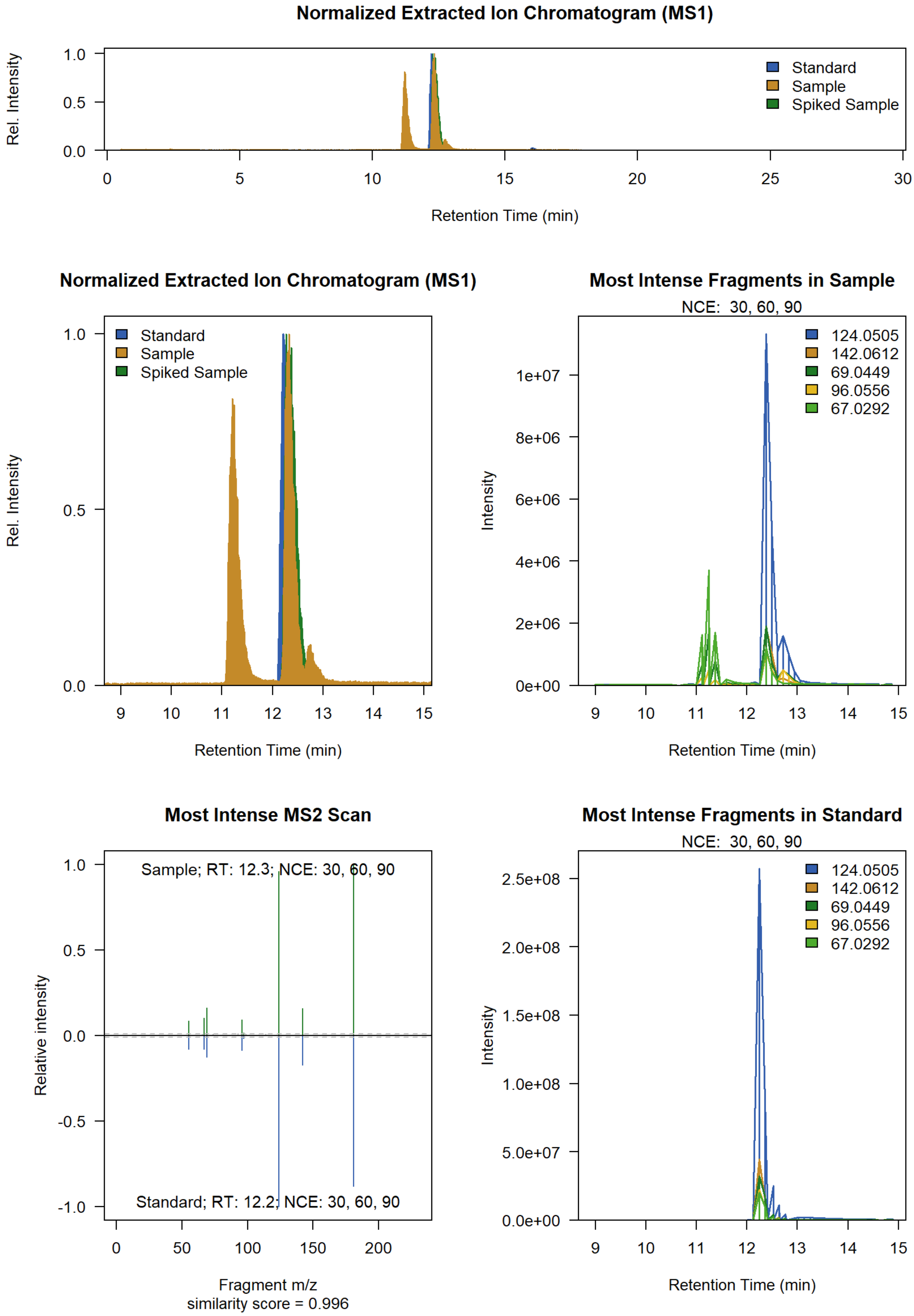

Figure SI-D101: Extracted ion chromatograms of paraxanthine in the reference standard, the sample and the spiked sample, as well as MS2 head to tail plot and most intense MS2 fragments in standard and sample. The first eluting peak originates from theobromine, the second from paraxanthine and the last from theophylline.

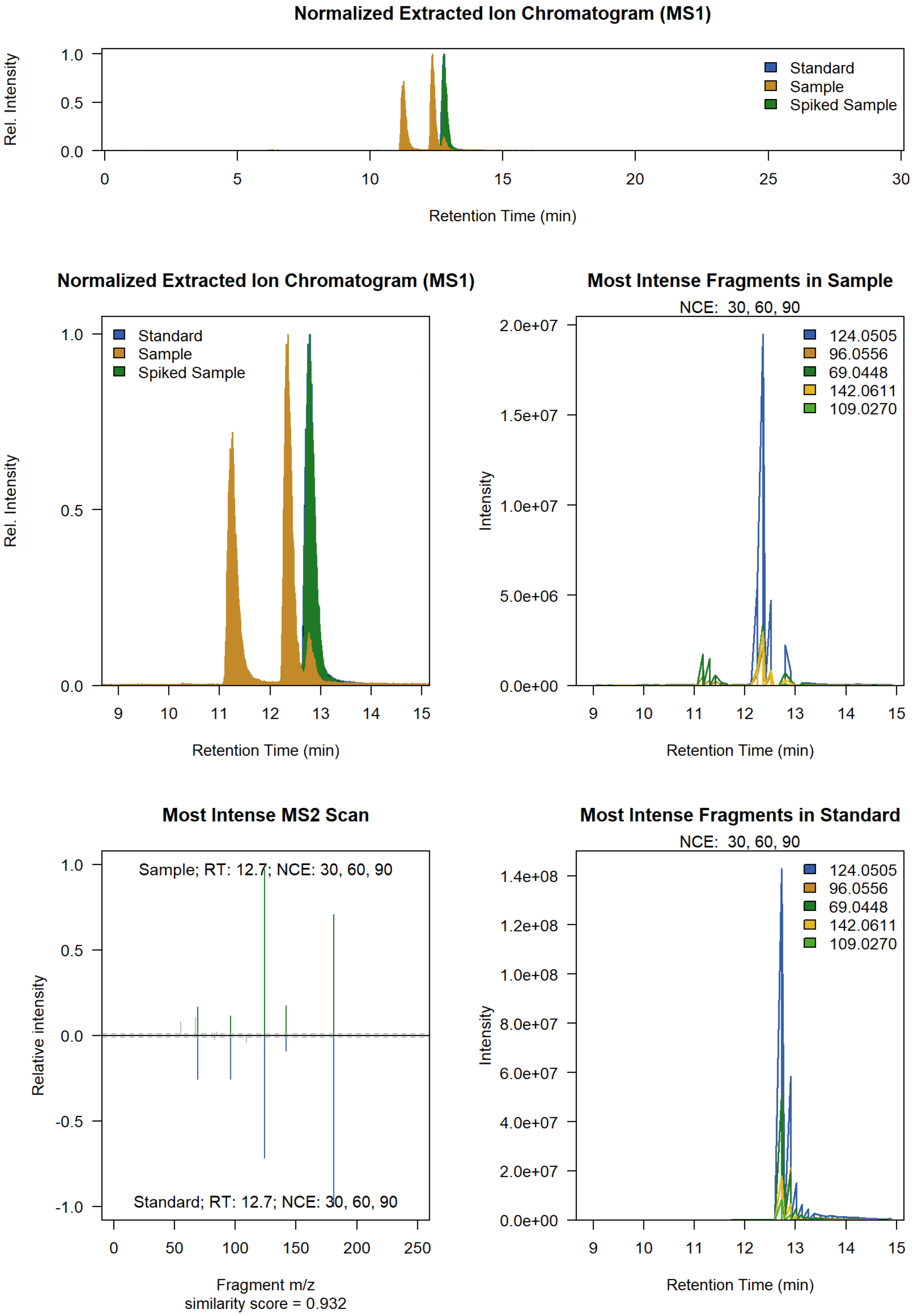

Figure SI-D102: Extracted ion chromatograms of theophylline in the reference standard, the sample and the spiked sample, as well as MS2 head to tail plot and most intense MS2 fragments in standard and sample. The first eluting peak originates from theobromine, the second from paraxanthine and the last from theophylline.

SI-D2.6.2 Theobromine

Table SI-D52: Information on identifiers, chemical properties, detection and confidence of identification of theobromine.

IUPAC Name	3,7-dimethylpurine-2,6-dione
Molecular formula	$C_7H_8N_4O_2$
Monoisotopic mass [g/mol]	180.0647
Adduct	$[M+H]^+$
Retention time [min]	10.8
SMILES	CN1C=NC2=C1C(=O)NC(=O)N2C
InChI	InChI=1S/C7H8N4O2/c1-10-3-8-5-4(10)6(12)9-7(13)11(5)2/h3H,1-2H3,(H,9,12,13)
InChI-Key	YAPQBXQYLJRXSA-UHFFFAOYSA-N
CAS RN	83-67-0
Metabolite of	Caffeine, theophylline
Detection frequency	100% (15/15 samples)
Detected in	Altenrhein, Monday-Friday<br>Neugut, Monday-Friday<br>Werdhölzli, Monday-Friday
Intensity	E9
Initial confidence level	level 2a
Initial confidence score	0.62
Final confidence level	level 1

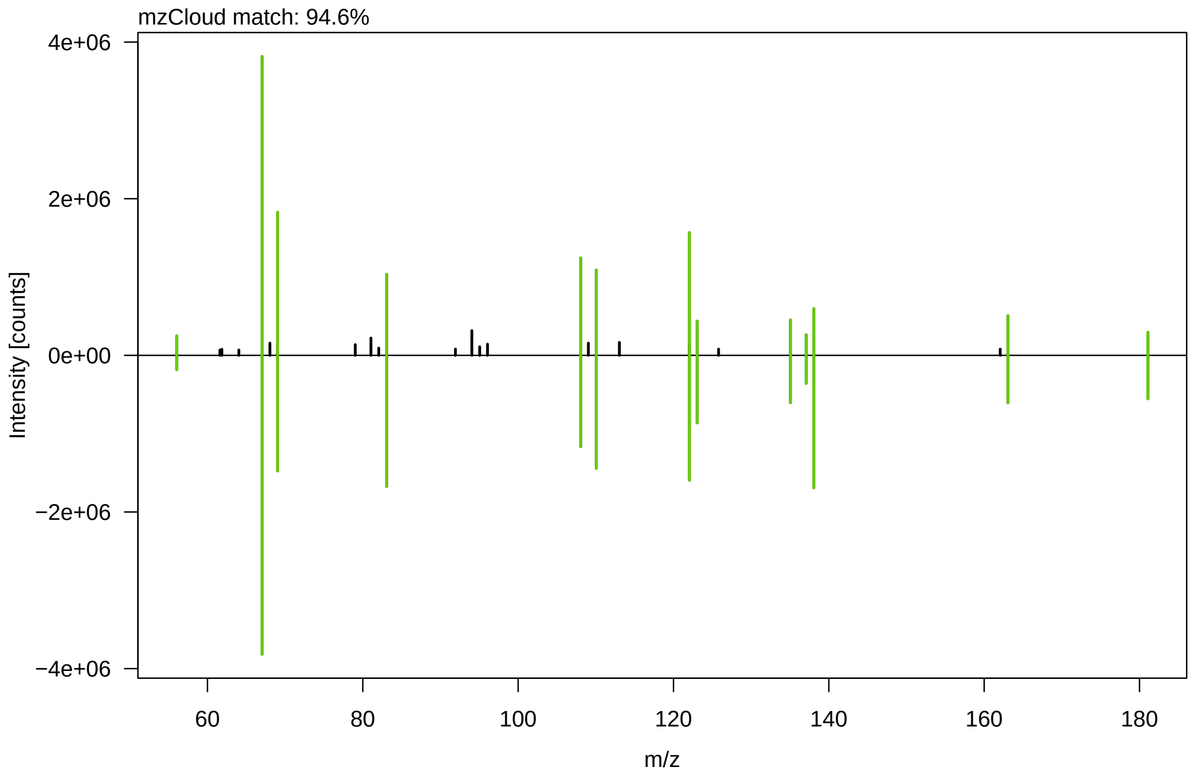

Figure SI-D103: Head to tail plot of measured MS2 spectrum against mzCloud library spectrum of theobromine. Matching fragments are highlighted in green.

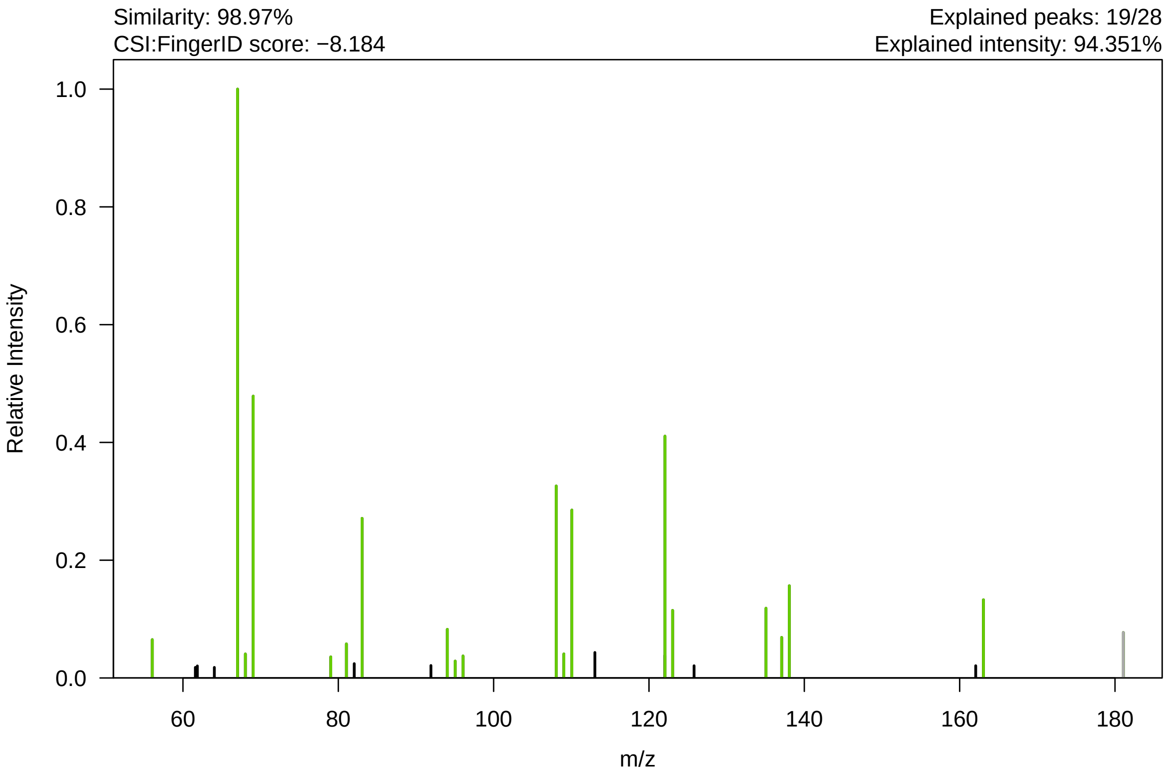

Figure SI-D104: Measured MS2 spectrum. Matching fragments with theobromine predicted by SIRIUS/CSI:FingerID are highlighted in green. The molecular ion in gray is not considered.

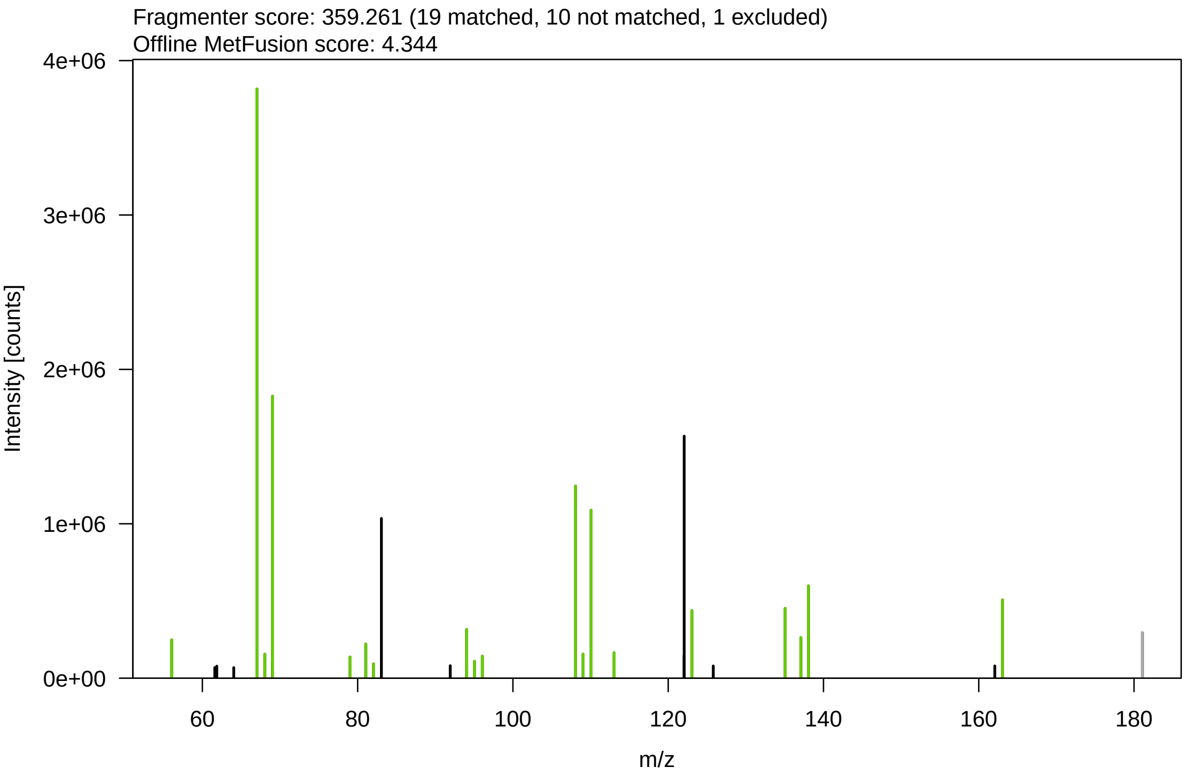

Figure SI-D105: Measured MS2 spectrum. Matching fragments with theobromine predicted by MetFrag are highlighted in green. The molecular ion in gray is not considered.

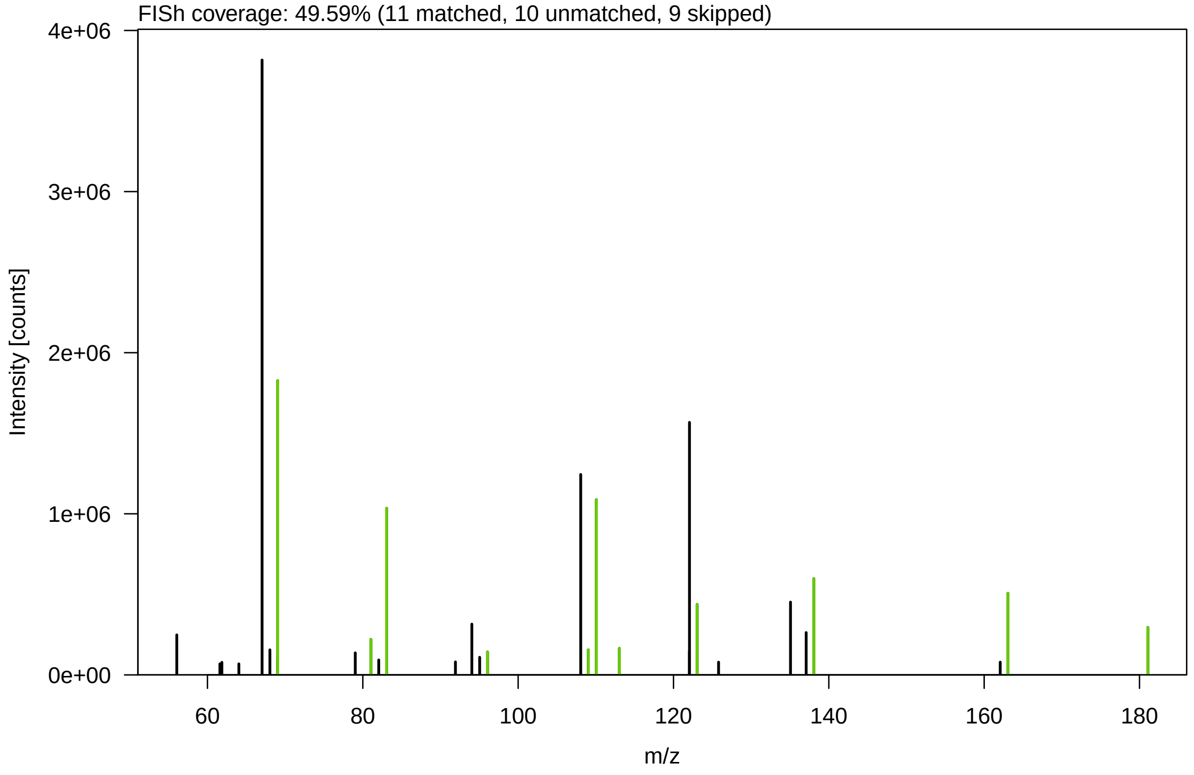

Figure SI-D106: Measured MS2 spectrum. Matching fragments with theobromine predicted by FISh Scoring are highlighted in green. Low intensity fragments are not considered and skipped.

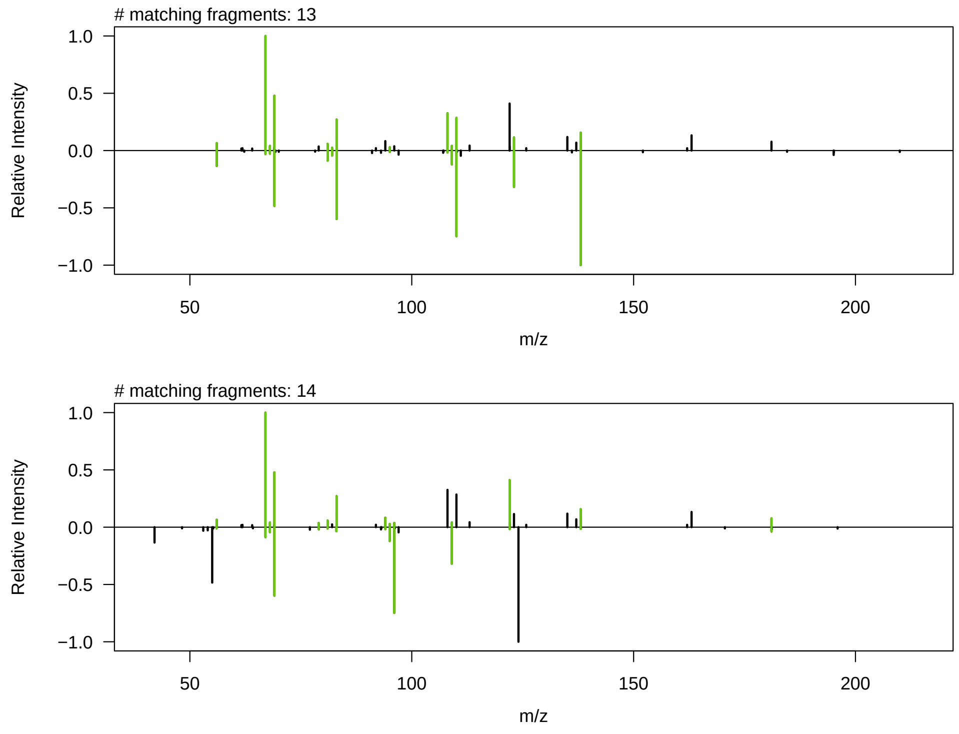

Figure SI-D107: Head to tail plots of theobromine and caffeine. In the bottom plot, the mass spectrum of caffeine is shifted by the mass difference. Matching fragments are highlighted in green.

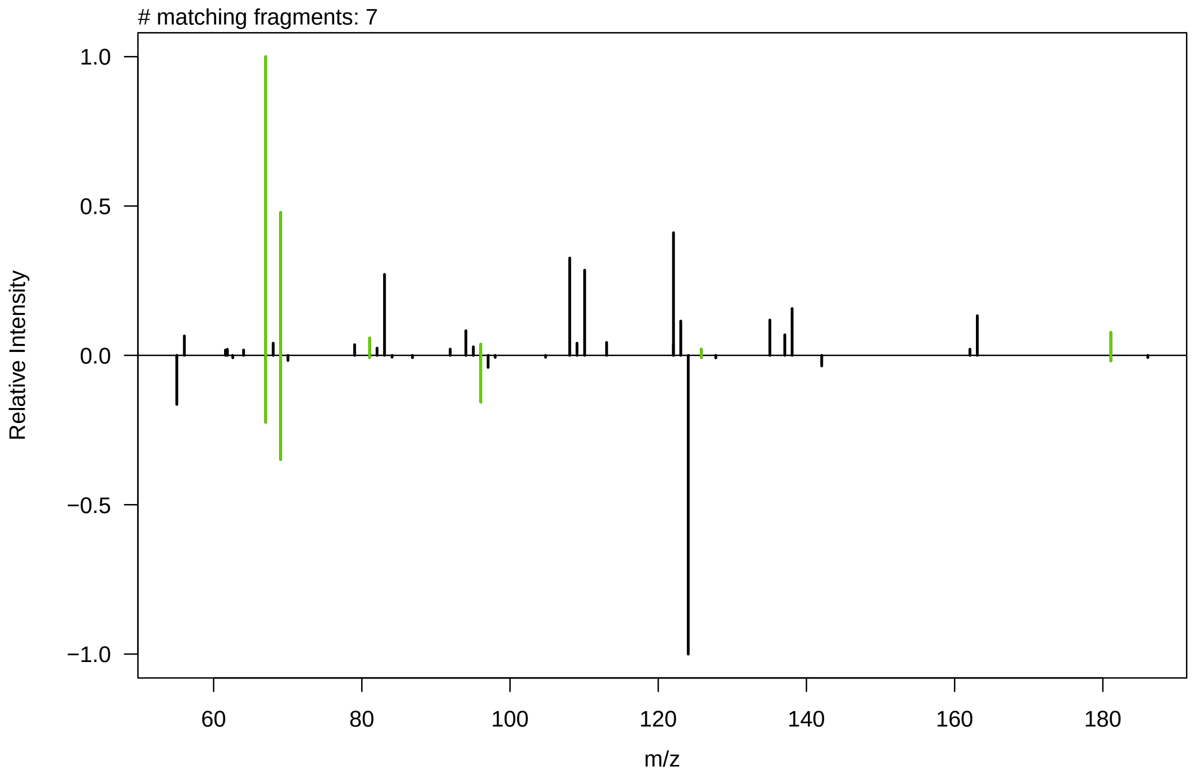

Figure SI-D108: Head to tail plot of the isomeric compounds theobromine and paraxanthine/theophylline. Matching fragments are highlighted in green.

Table SI-D53: Molecular network results and retention time prediction of theobromine.

Comparison with	Caffeine
MSn Score	69
Forward coverage	51
Reverse coverage	88
Forward match	62
Reverse match	43
ΔMass [g/mol]	14.0157
Comparison with	Paraxanthine/Theophylline
MSn Score	70
Forward coverage	63
Reverse coverage	77
Forward match	37
Reverse match	93
ΔMass [g/mol]	0
Measured retention time [min]	10.8
Predicted $\log D_{OW}$ (pH = 2.7)	-0.77
Predicted retention time [min]	13.7
Predicted retention time range (95% confidence interval) [min]	9.1-18.3
Predicted retention time range (99% confidence interval) [min]	7.7-19.8

Table SI-D54: Annotated MS2 spectrum of theobromine.

m/z	Relative Intensity	Annotation
56.0498	65.02	$C_3H_5N + H^+$
61.5990	17.95	
61.8541	20.38	
64.0416	17.91	
67.0293	999.00	$C_3H_2N_2 + H^+$
68.0370	40.72	$C_3H_3N_2 + H^+$
69.0412	25.55	
69.0449	478.10	$C_3H_4N_2 + H^+$
79.0289	35.80	$C_4H_2N_2 + H^+$
81.0448	57.87	$C_4H_4N_2 + H^+$
82.0521	24.28	$C_4H_5N_2 + H^+$
83.0604	270.82	$C_4H_6N_2 + H^+$
91.9261	21.12	
94.0399	82.54	$C_4H_3N_3 + H^+$
95.0477	28.62	$C_4H_4N_3 + H^+$
96.0557	37.35	$C_4H_5N_3 + H^+$
108.0558	325.67	$C_5H_5N_3 + H^+$
109.0392	40.80	$C_5H_4N_2O + H^+$
110.0713	284.82	$C_5H_7N_3 + H^+$
113.0345	43.22	$C_4H_4N_2O_2 + H^+$
122.0509	38.12	
122.0588	410.23	$C_5H_4N_4 + H^+$
123.0424	114.68	$C_5H_4N_3O + H^+$
125.8045	20.72	
135.0664	118.32	$C_6H_6N_4 + H^+$
137.0819	68.81	$C_6H_8N_4 + H^+$
138.0661	156.49	$C_6H_7N_3O + H^+$
162.0651	20.74	
163.0615	132.66	$C_7H_6N_4O + H^+$
181.0715	77.14	$C_7H_8N_4O_2 + H^+$

A reference standard of theobromine was purchased. Figure SI-D109 shows the extracted ion chromatograms of this standard, the sample and the spiked sample, as well as a head to tail plot of the MS2 spectra of the standard and the sample. In addition, the most intense MS2 fragments in the sample and in the standard are displayed. It becomes visible that the retention times of the sample and the spiked sample are identical and the spectra similarity score between sample and standard is equal to 0.996. The majority of the MS2 fragments in the sample can be explained by the reference standard. It can therefore be concluded that the suspected compound is indeed theobromine. Correspondingly, the identification confidence can be increased to level 1.

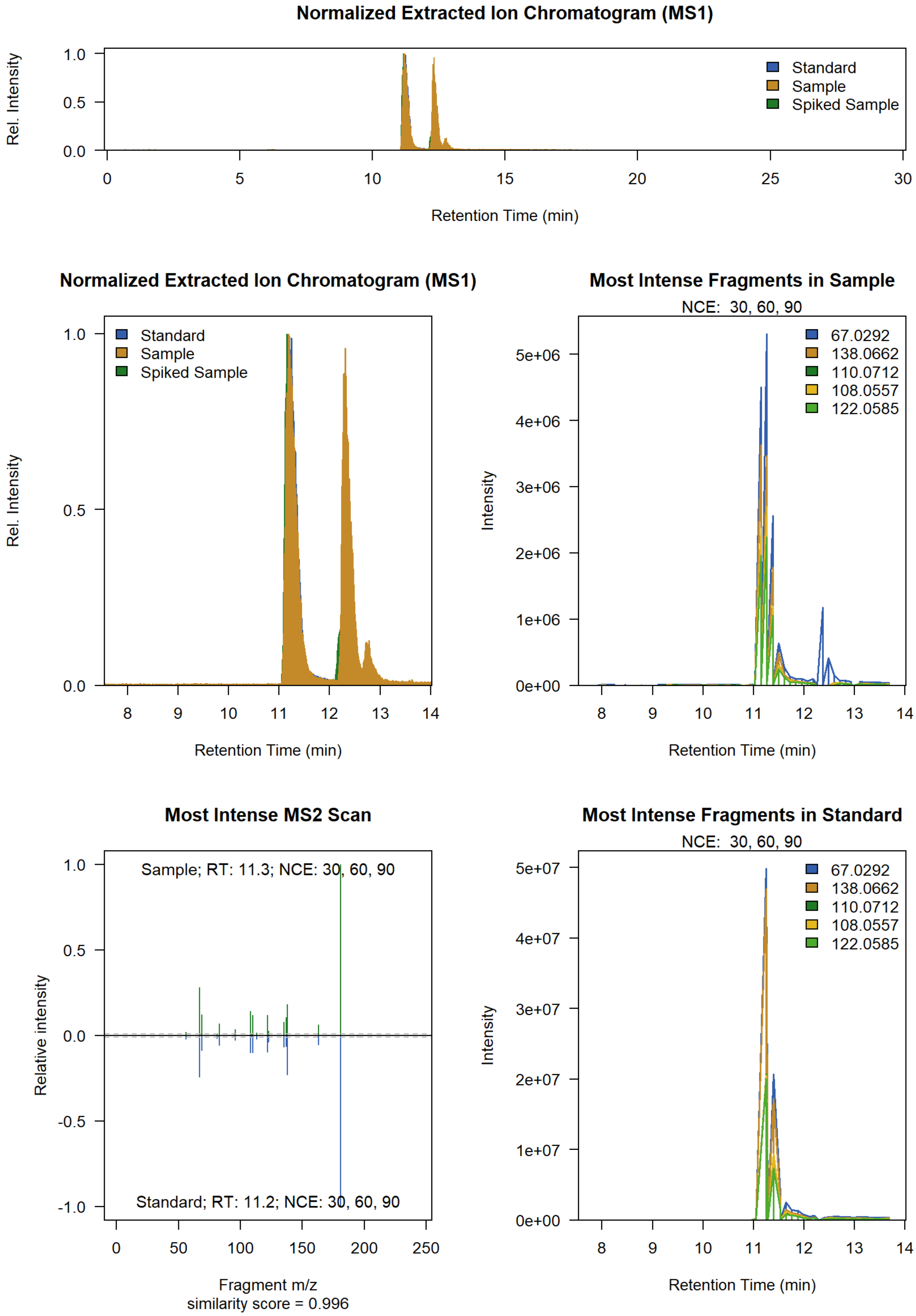

Figure SI-D109: Extracted ion chromatograms of theobromine in the reference standard, the sample and the spiked sample, as well as MS2 head to tail plot and most intense MS2 fragments in standard and sample. The first eluting peak originates from theobromine, the second from paraxanthine and the last from theophylline.

SI-D2.6.3 1-Methylxanthine

Table SI-D55: Information on identifiers, chemical properties, detection and confidence of identification of 1-methylxanthine.

IUPAC Name	1-methyl-3,7-dihydropurine-2,6-dione
Molecular formula	$C_6H_6N_4O_2$
Monoisotopic mass [g/mol]	166.0491
Adduct	$[M+H]^+$
Retention time [min]	10.3
SMILES	CN1C(=O)C2=C(NC1=O)N=CN2
InChI	InChI=1S/C6H6N4O2/c1-10-5(11)3-4(8-2-7-3)9-6(10)12/h2H,1H3,(H,7,8)(H,9,12)
InChI-Key	MVOYJPOZRLFTCP-UHFFFAOYSA-N
CAS RN	6136-37-4
Metabolite of	Caffeine, theophylline
Detection frequency	100% (15/15 samples)
Detected in	Altenrhein, Monday-Friday
	Neugut Monday-Friday
	Werdhölzli, Monday-Friday
Intensity	E9
Initial confidence level	level 2a
Inital confidence score	0.71
Final confidence level	level 1

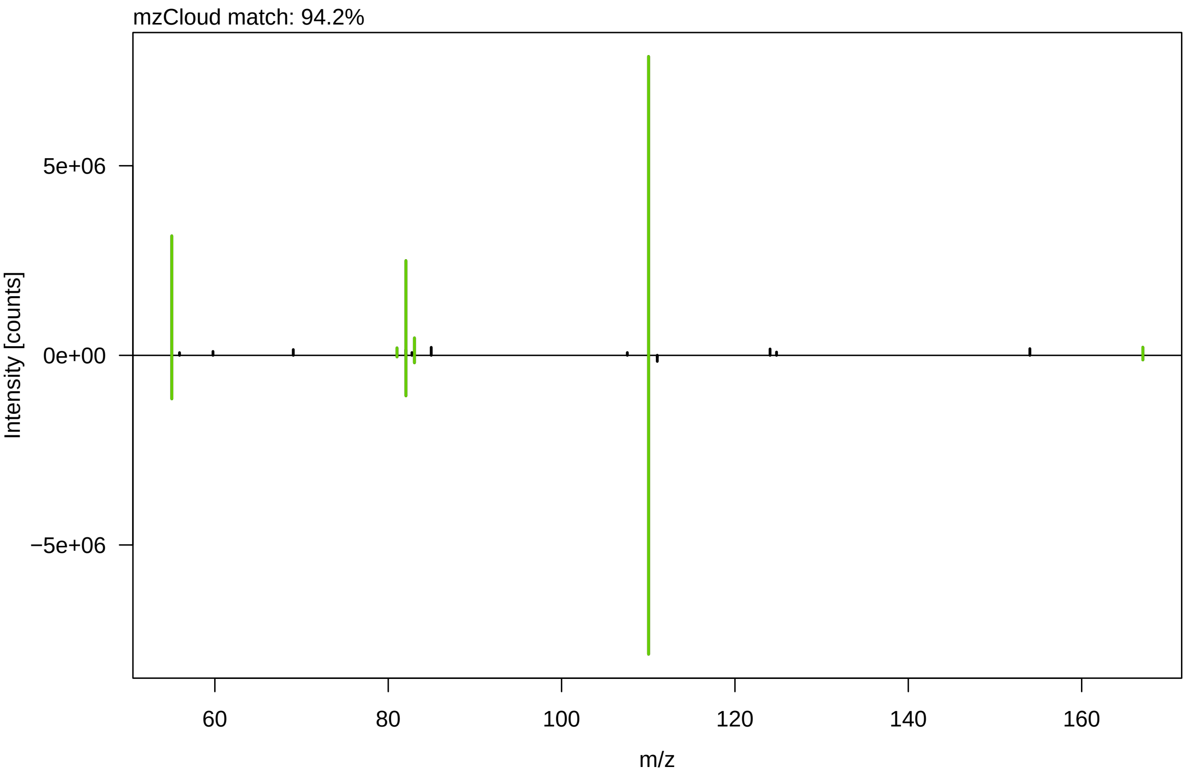

Figure SI-D110: Head to tail plot of measured MS2 spectrum against mzCloud library spectrum of 1-methylxanthine. Matching fragments are highlighted in green.

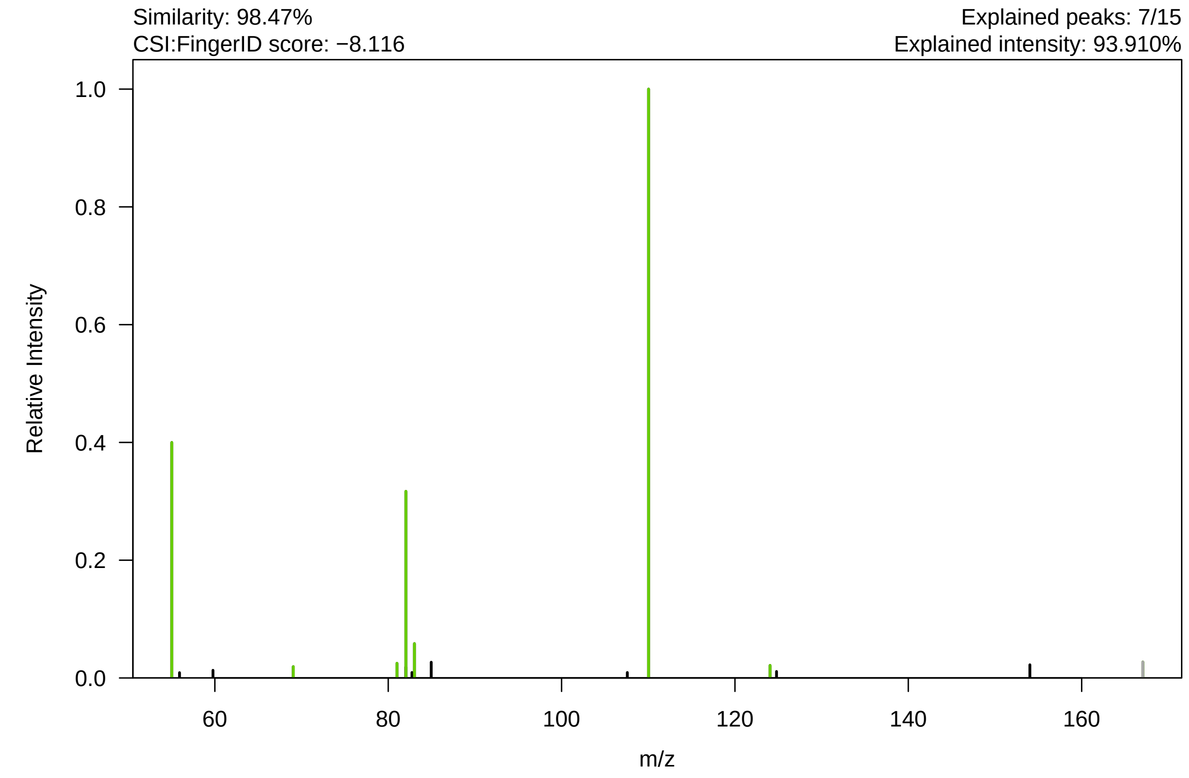

Figure SI-D111: Measured MS2 spectrum. Matching fragments with 1-methylxanthine predicted by SIRIUS/CSI:FingerID are highlighted in green. The molecular ion in gray is not considered.

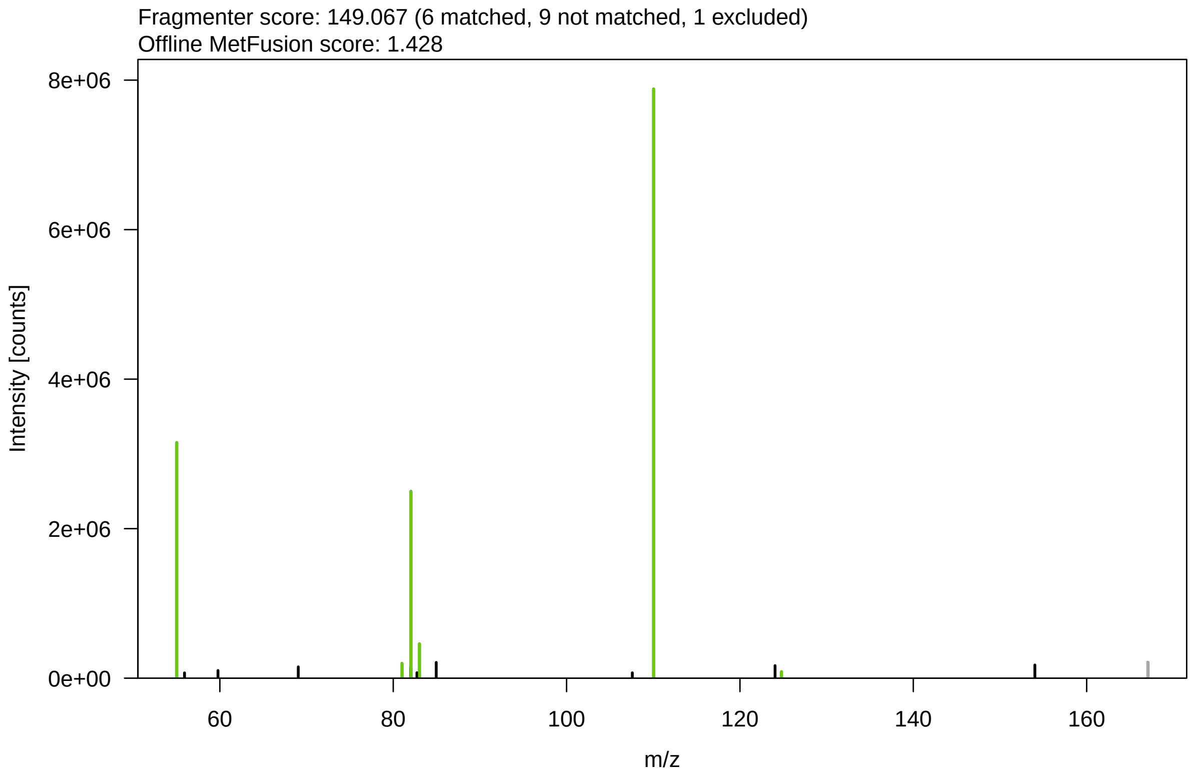

Figure SI-D112: Measured MS2 spectrum. Matching fragments with 1-methylxanthine predicted by MetFrag are highlighted in green. The molecular ion in gray is not considered.

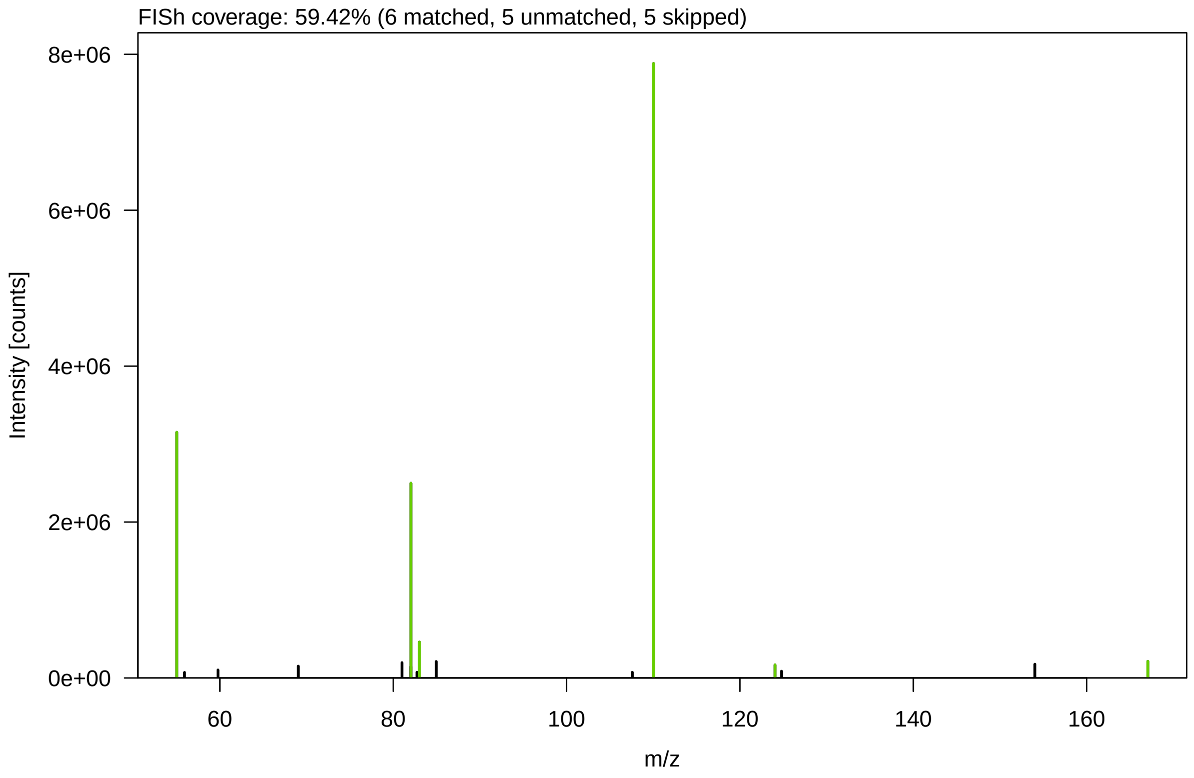

Figure SI-D113: Measured MS2 spectrum. Matching fragments with 1-methylxanthine predicted by FISh Scoring are highlighted in green. Low intensity fragments are not considered and skipped.

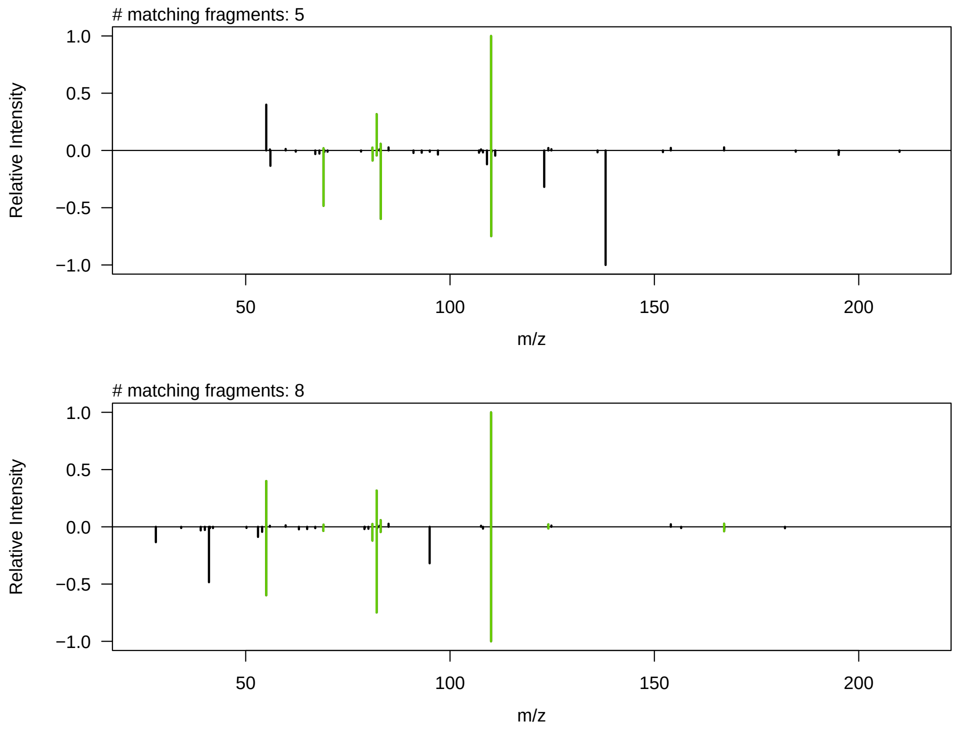

Figure SI-D114: Head to tail plots of 1-methylxanthine and caffeine. In the bottom plot, the mass spectrum of caffeine is shifted by the mass difference. Matching fragments are highlighted in green.

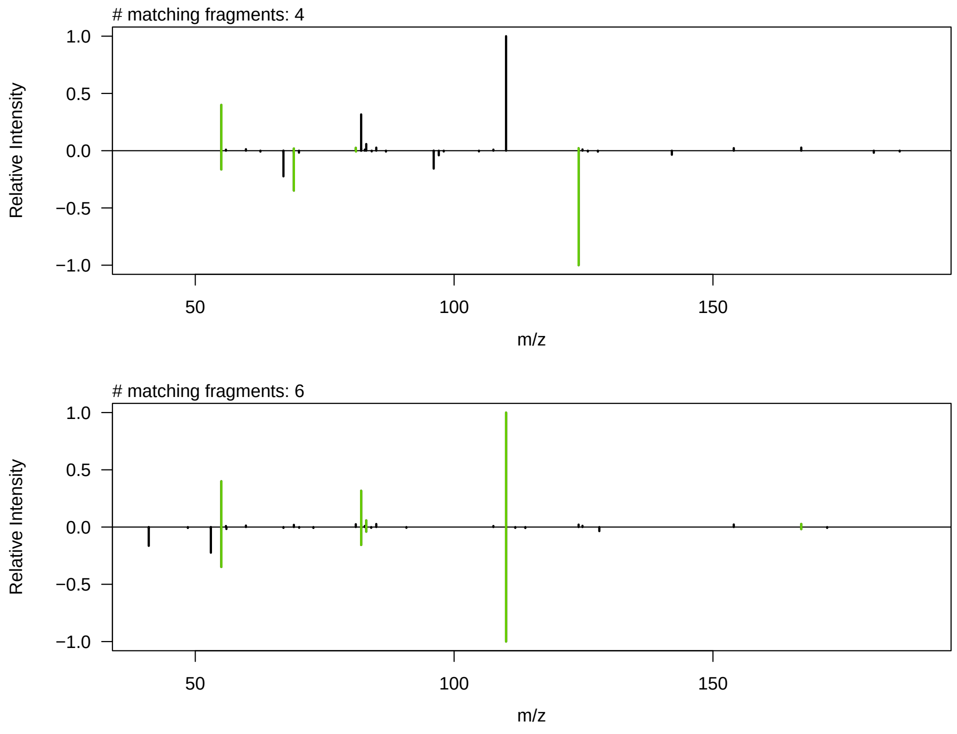

Figure SI-D115: Head to tail plots of 1-methylxanthine and paraxanthine/theophylline. In the bottom plot, the mass spectrum of paraxanthine/theophylline is shifted by the mass difference. Matching fragments are highlighted in green.

Table SI-D56: Molecular network results and retention time prediction of 1-methylxanthine.

Comparison with	Caffeine
MSn Score	55
Forward coverage	54
Reverse coverage	57
Forward match	37
Reverse match	28
ΔMass [g/mol]	28.0313
Comparison with	Paraxanthine/Theophylline
MSn Score	63
Forward coverage	64
Reverse coverage	63
Forward match	44
Reverse match	37
ΔMass [g/mol]	14.0157
Measured retention time [min]	10.3
Predicted $logD_{OW}$ (pH = 2.7)	0.02
Predicted retention time [min]	14.8
Predicted retention time range (95% confidence interval) [min]	10.2-19.4
Predicted retention time range (99% confidence interval) [min]	8.7-20.8

Table SI-D57: Annotated MS2 spectrum of 1-methylxanthine.

m/z	Relative Intensity	Annotation
55.0295	399.50	$C_2H_2N_2 + H^+$
55.9213	9.03	
59.7818	13.03	
69.0448	19.21	$C_3H_4N_2 + H^+$
81.0083	24.92	$C_3N_2O + H^+$
82.0352	18.49	
82.0400	316.69	$C_3H_3N_3 + H^+$
82.7357	9.31	
83.0241	58.29	$C_3H_2N_2O + H^+$
84.9595	26.77	
107.5835	9.25	
110.0349	999.00	$C_4H_3N_3O + H^+$
124.0504	21.32	$C_5H_5N_3O + H^+$
124.7974	11.00	
154.0243	22.38	
167.0564	27.05	$C_6H_6N_4O_2 + H^+$

A reference standard of 1-methylxanthine was purchased. Figure SI-D116 shows the extracted ion chromatograms of this standard, the sample and the spiked sample, as well as a head to tail plot of the MS2 spectra of the standard and the sample. In addition, the most intense MS2 fragments in the sample and in the standard are displayed. It becomes visible that the retention times of the sample and the spiked sample are identical and the spectra similarity score between sample and standard is equal to 0.997. The majority of the MS2 fragments in the sample can be explained by the reference standard. It can therefore be concluded that the suspected compound is indeed 1-methylxanthine. Correspondingly, the identification confidence can be increased to level 1.

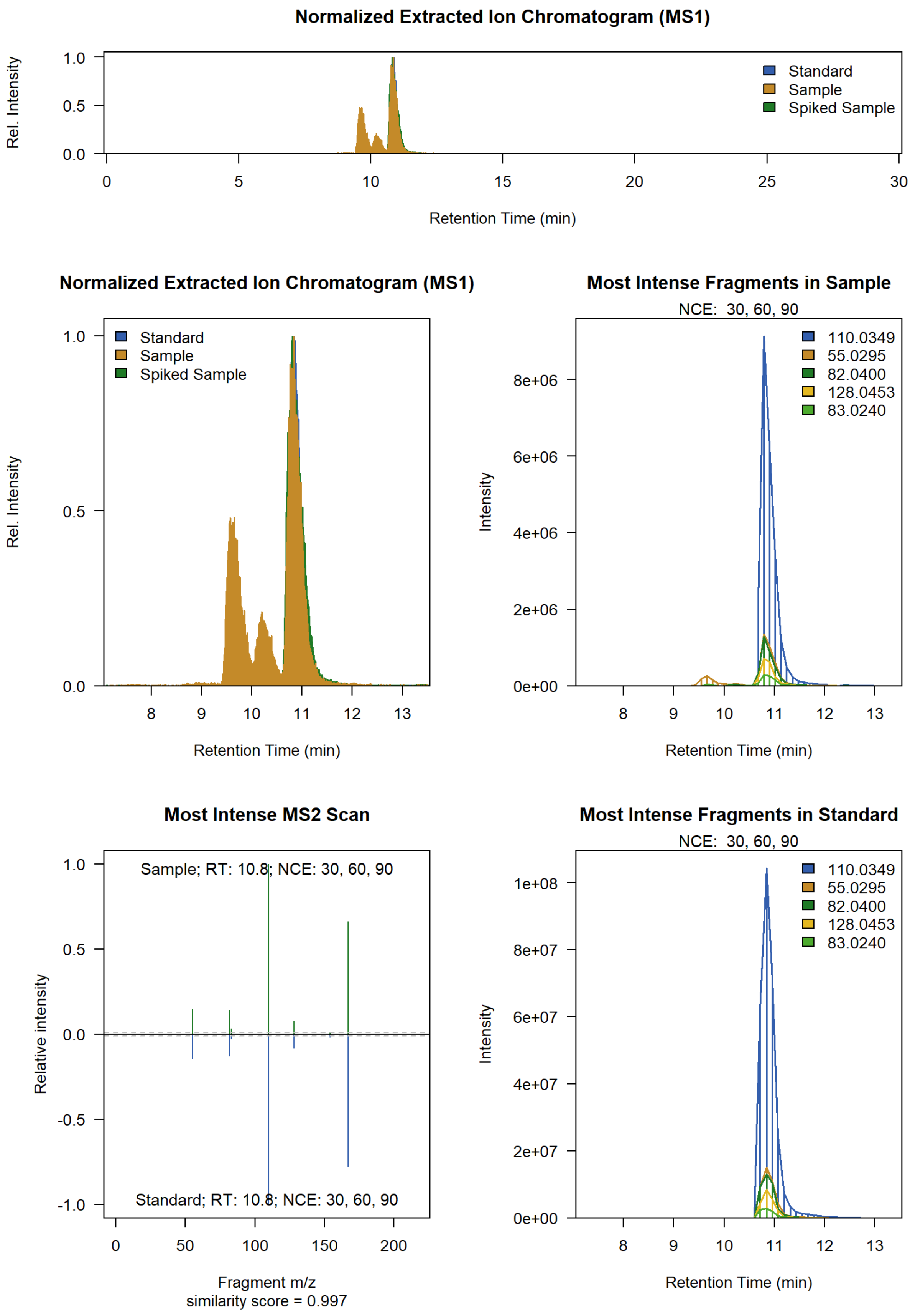

Figure SI-D116: Extracted ion chromatograms of 1-methylxanthine in the reference standard, the sample and the spiked sample, as well as MS2 head to tail plot and most intense MS2 fragments in standard and sample. The first eluting peak originates from 3-methylxanthine, the second from 7-methylxanthine and the last from 1-methylxanthine.

SI-D2.6.4 3-Methylxanthine and 7-Methylxanthine

The chromatographic signal is a double peak, which Compound Discoverer was not able to separate. Therefore, the two compounds are discussed together in this subsection. However, for the confirmation measurements, a better chromatogrpahic resolution was achieved, enabling a separation of the two compounds. A reference standard for 3-methylxanthine was not purchased. To make a semiquantification in the samples possible, 2/3 of the area was assigned to originate from 7-methylxanthine and 1/3 from 3-methylxanthine. This leads to a bias in the quantification approach, but since it is only semiquantitative, the error introduced is expected to be in the same range than the one from MS2Quant.

Table SI-D58: Information on identifiers, chemical properties, detection and confidence of identification of 3-methylxanthine and 7-methylxanthine.

IUPAC Name	3-methyl-7*H*-purine-2,6-dione
	7-methyl-3*H*-purine-2,6-dione
Molecular formula	$C_6H_6N_4O_2$
Monoisotopic mass [g/mol]	166.0491
Adduct	$[M+H]^+$
Retention time [min]	9.7
	9.5
SMILES	CN1C2=C(C(=O)NC1=O)NC=N2
	CN1C=NC2=C1C(=O)NC(=O)N2
InChI	InChI=1S/C6H6N4O2/c1-10-4-3(7-2-8-4)5(11)9-6(10)12/h2H,1H3,(H,7,8)(H,9,11,12)
	InChI=1S/C6H6N4O2/c1-10-2-7-4-3(10)5(11)9-6(12)8-4/h2H,1H3,(H2,8,9,11,12)
InChI-Key	GMSNIKWWOQHZGF-UHFFFAOYSA-N
	PFWLFWPASULGAN-UHFFFAOYSA-N
CAS RN	1076-22-8
	552-62-5
Metabolite of	Caffeine, theophylline
Detection frequency	100% (15/15 samples)
Detected in	Altenrhein, Monday-Friday
	Neugut, Monday-Friday
	Werdhölzli, Monday-Friday
Intensity	E8-E9
Initial confidence level	level 2a
	level 2a
Initial confidence score	0.53
	0.61
Final confidence level	level 2a
	level 1

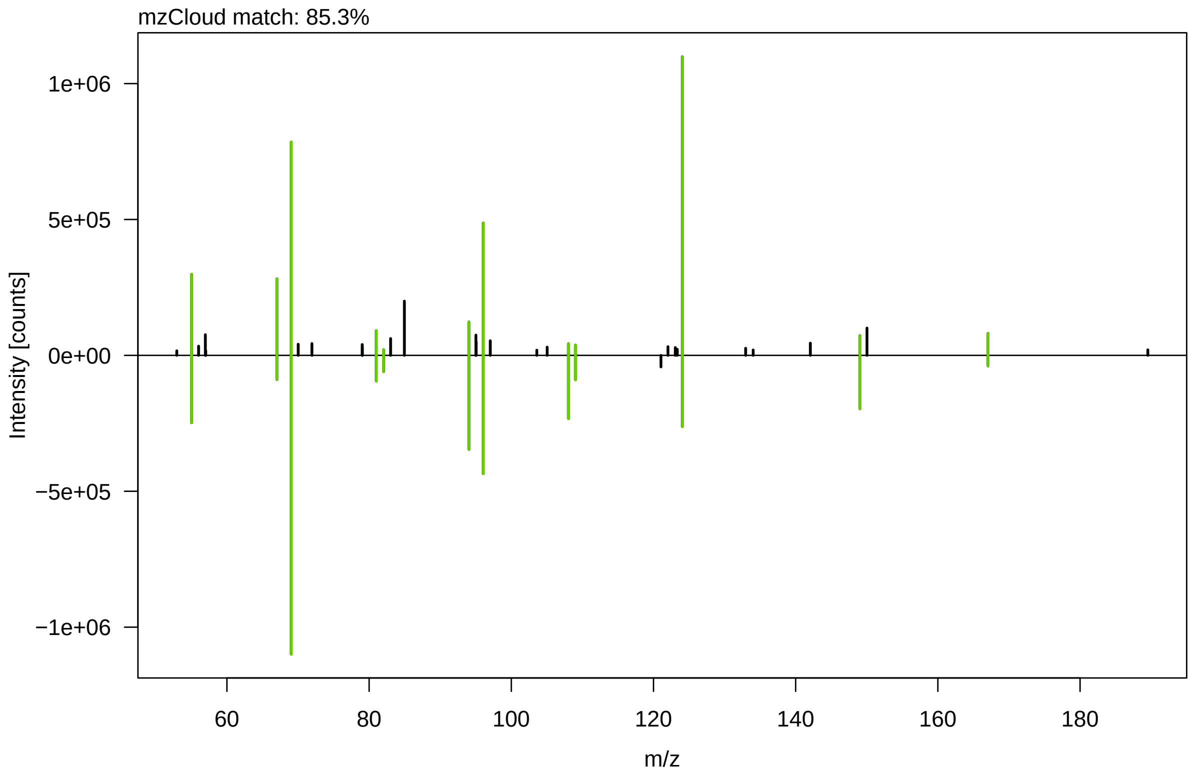

Figure SI-D117: Head to tail plot of measured MS2 spectrum against mzCloud library spectrum of 3-methylxanthine. Matching fragments are highlighted in green.

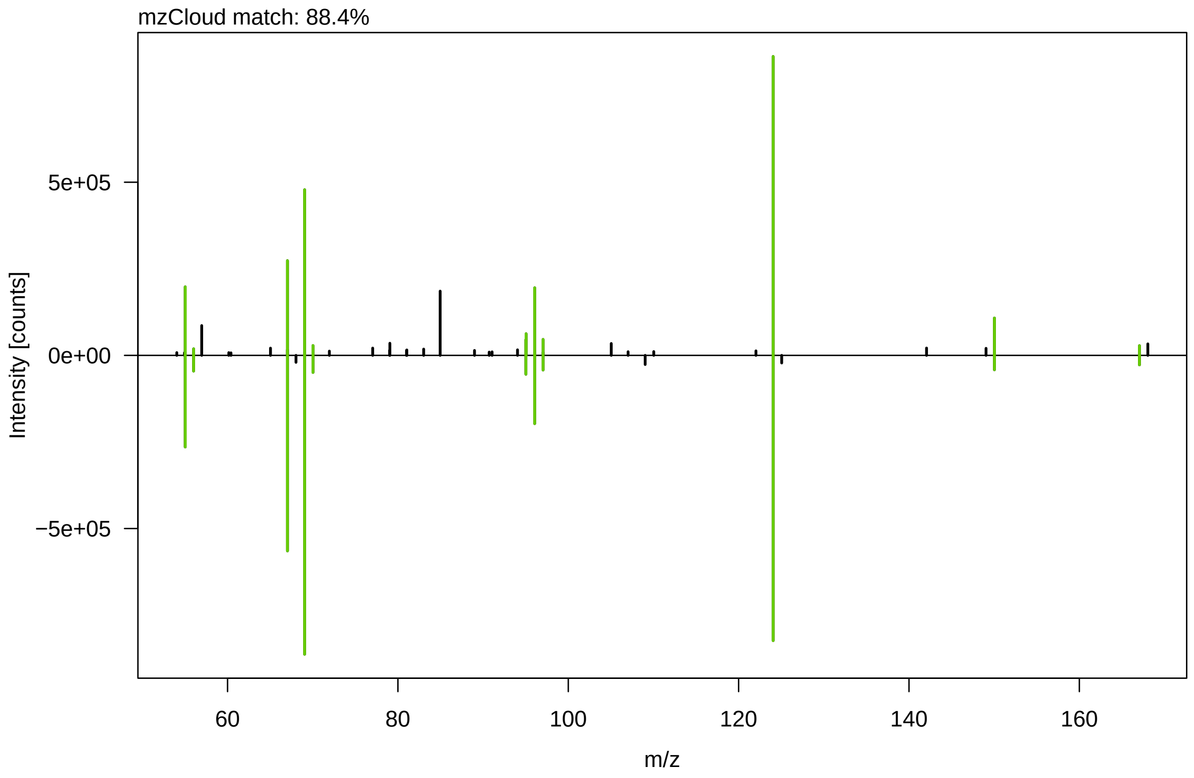

Figure SI-D118: Head to tail plot of measured MS2 spectrum against mzCloud library spectrum of 7-methylxanthine. Matching fragments are highlighted in green.

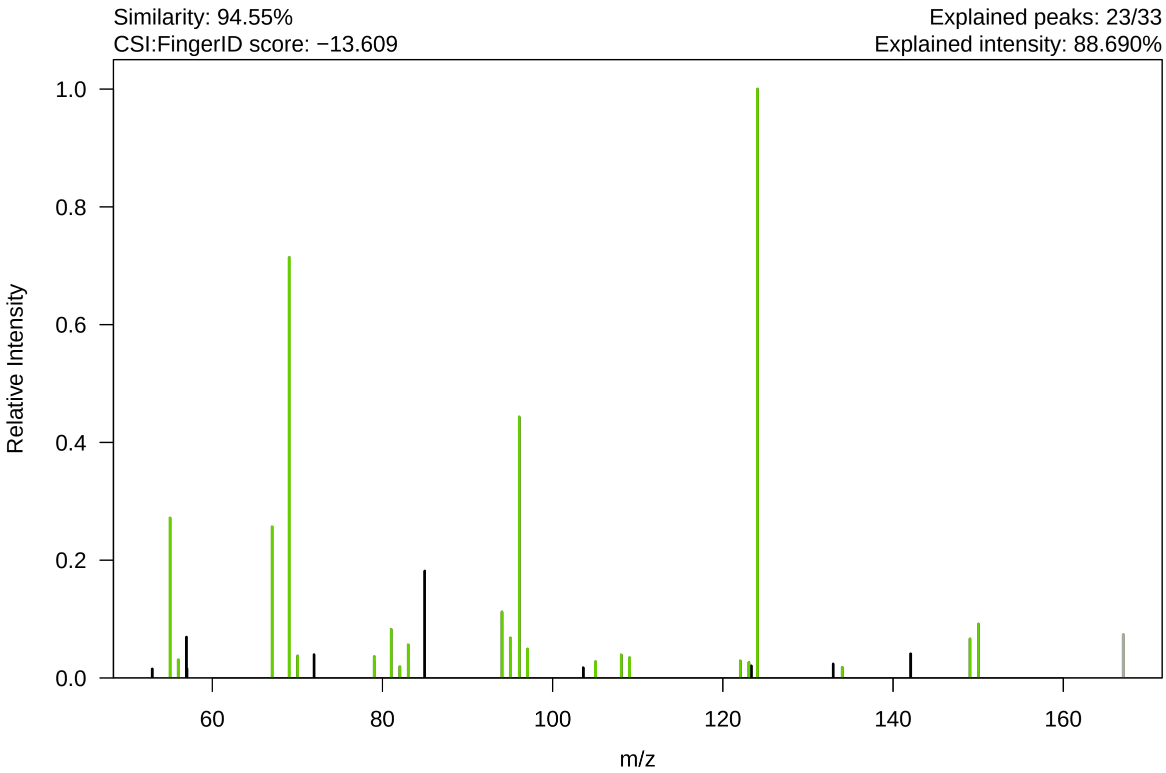

Figure SI-D119: Measured MS2 spectrum. Matching fragments with 3-methylxanthine predicted by SIRIUS/CSI:FingerID are highlighted in green. The molecular ion in gray is not considered.

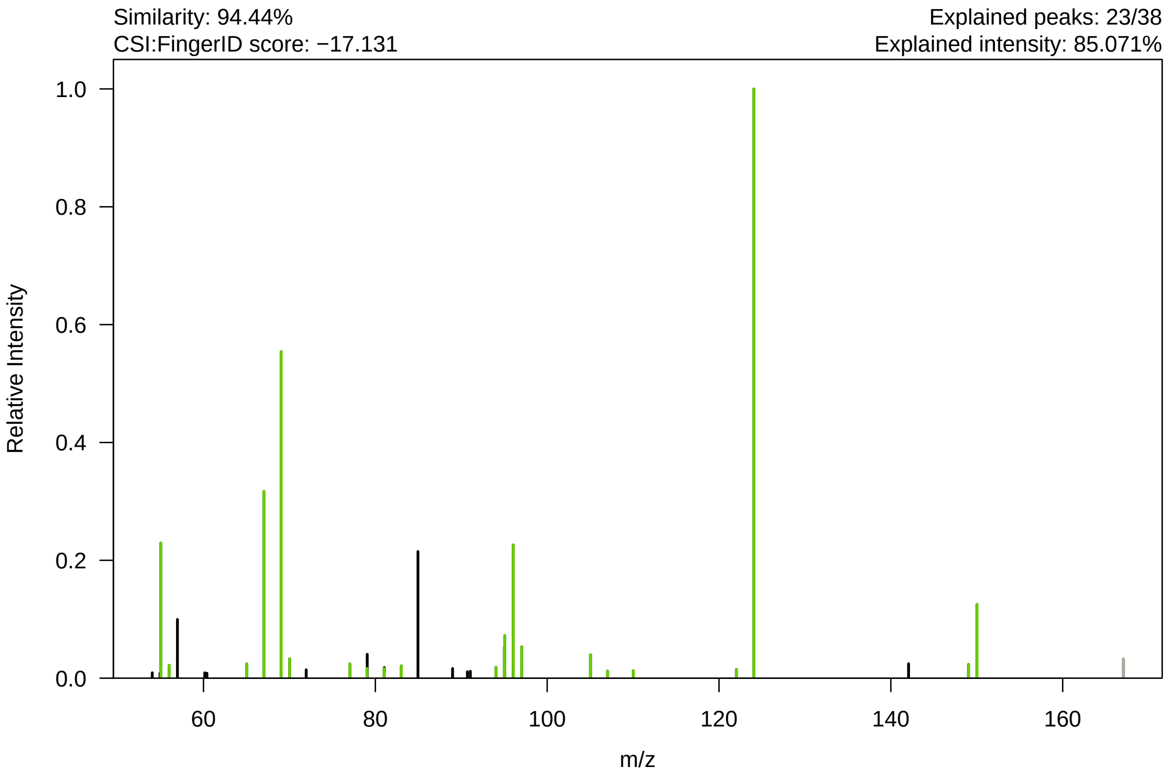

Figure SI-D120: Measured MS2 spectrum. Matching fragments with 7-methylxanthine predicted by SIRIUS/CSI:FingerID are highlighted in green. The molecular ion in gray is not considered.

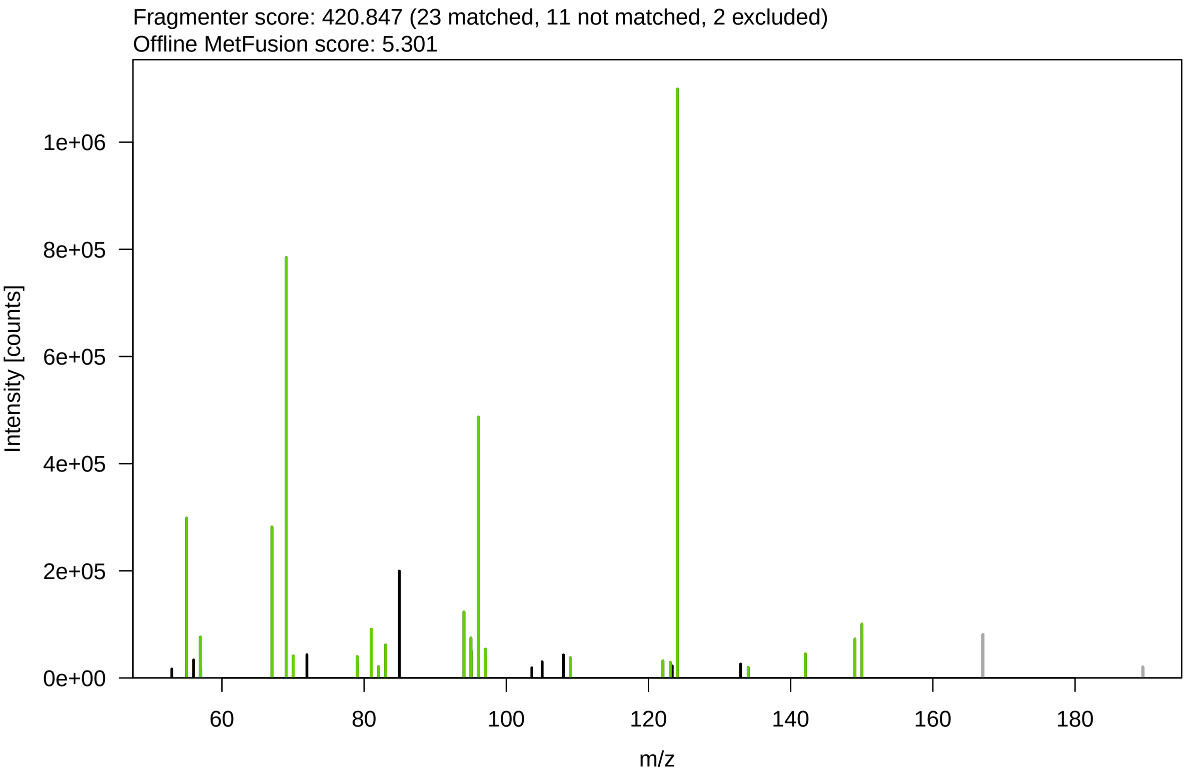

Figure SI-D121: Measured MS2 spectrum. Matching fragments with 3-methylxanthine predicted by MetFrag are highlighted in green. The molecular ion in gray is not considered.

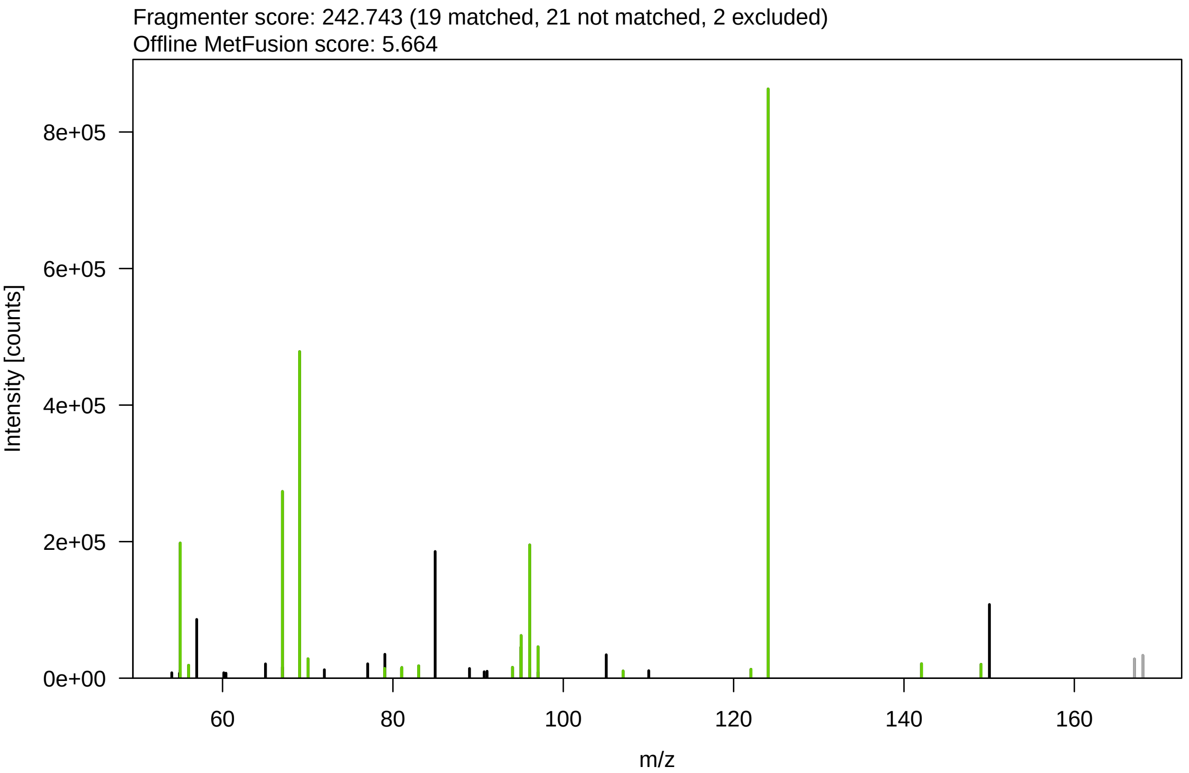

Figure SI-D122: Measured MS2 spectrum. Matching fragments with 7-methylxanthine predicted by MetFrag are highlighted in green. The molecular ion in gray is not considered.

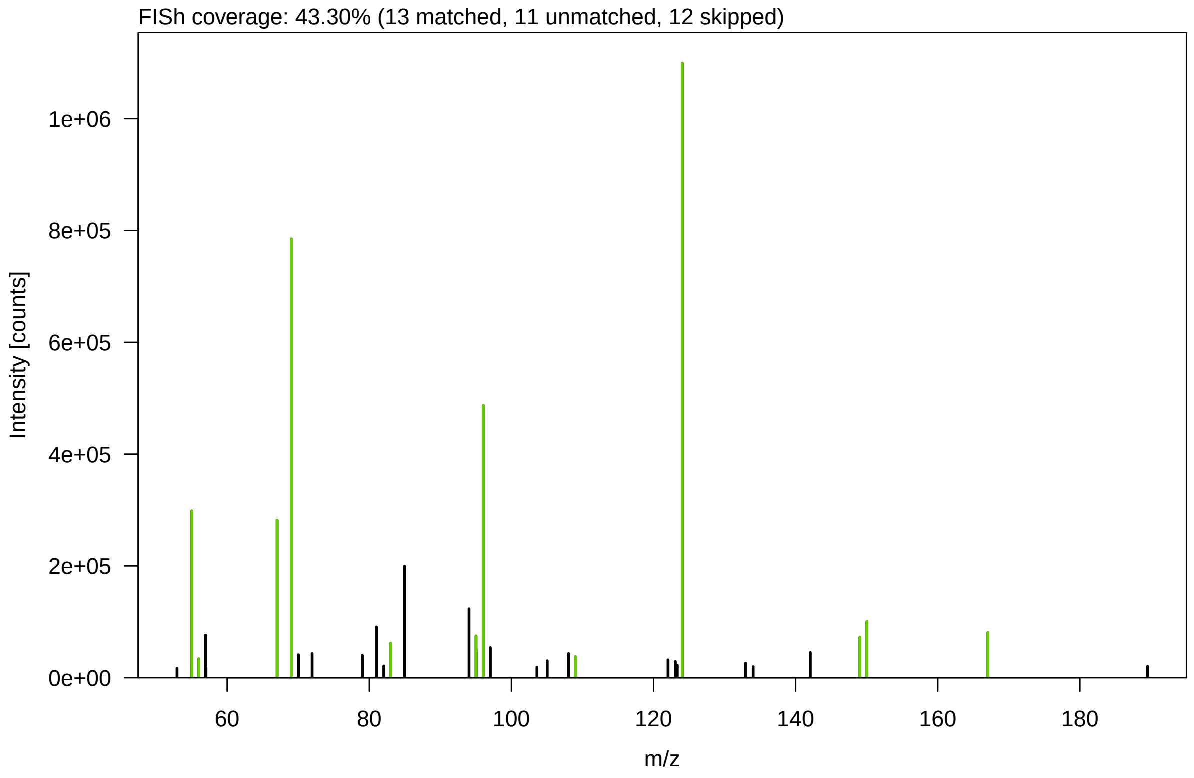

Figure SI-D123: Measured MS2 spectrum. Matching fragments with 3-methylxanthine predicted by FISh Scoring are highlighted in green. Low intensity fragments are not considered and skipped.

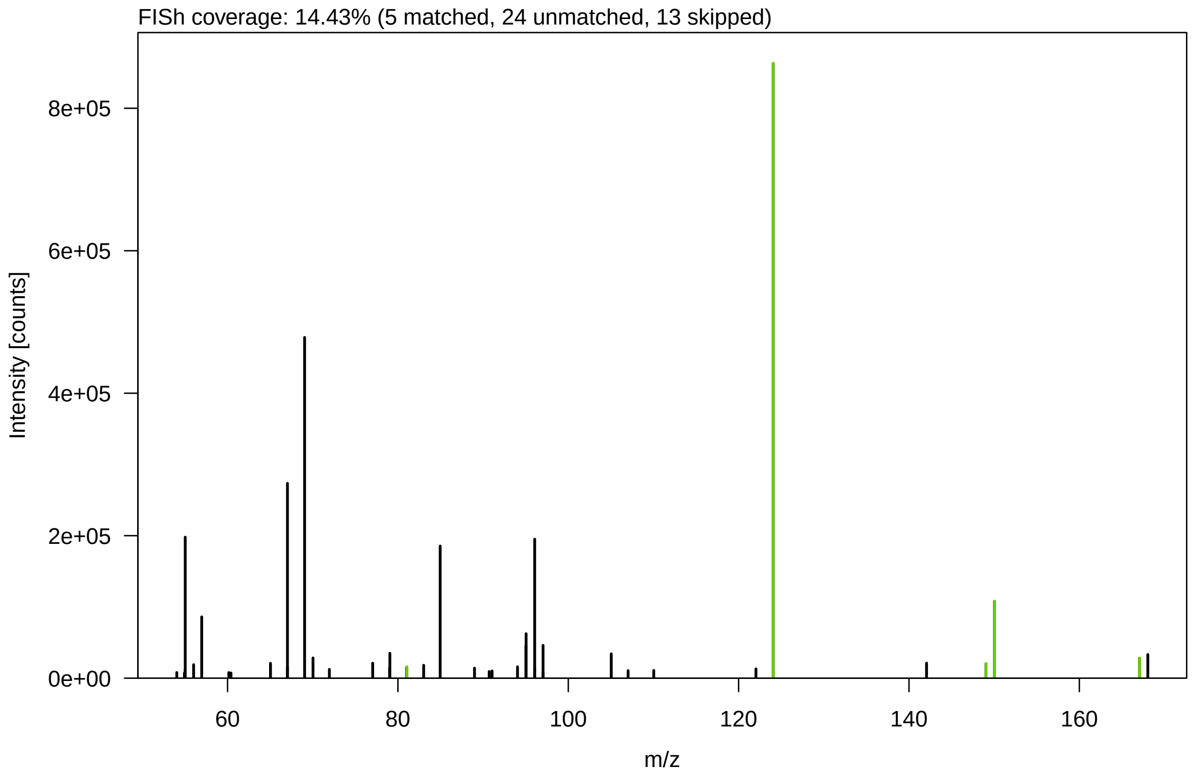

Figure SI-D124: Measured MS2 spectrum. Matching fragments with 7-methylxanthine predicted by FISh Scoring are highlighted in green. Low intensity fragments are not considered and skipped.

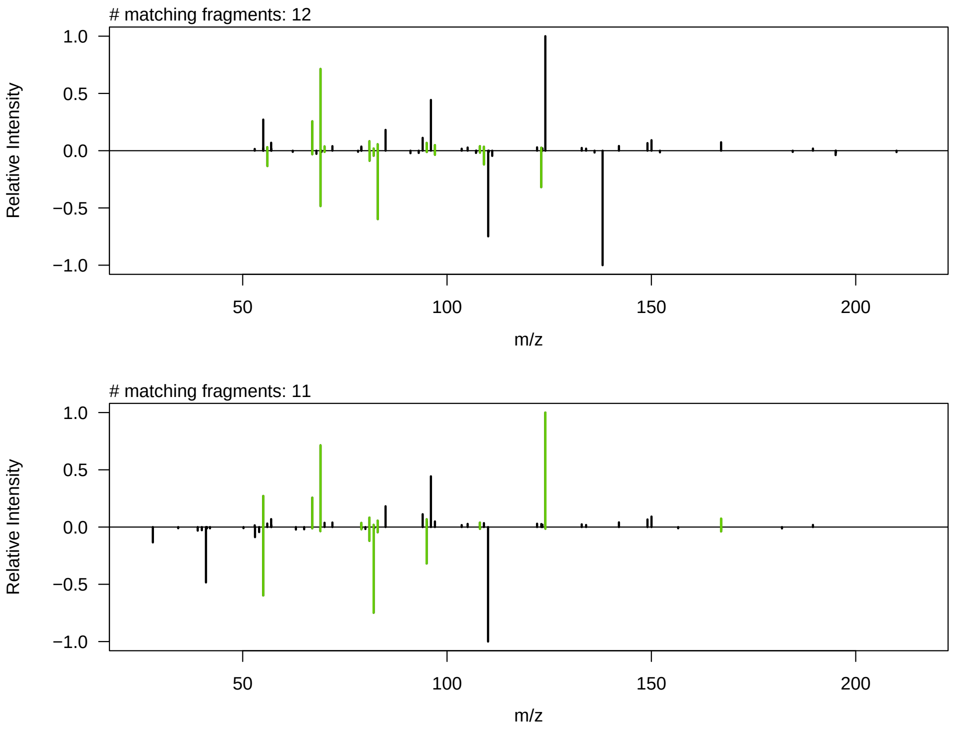

Figure SI-D125: Head to tail plots of 3-methylxanthine and caffeine. In the bottom plot, the mass spectrum of caffeine is shifted by the mass difference. Matching fragments are highlighted in green.

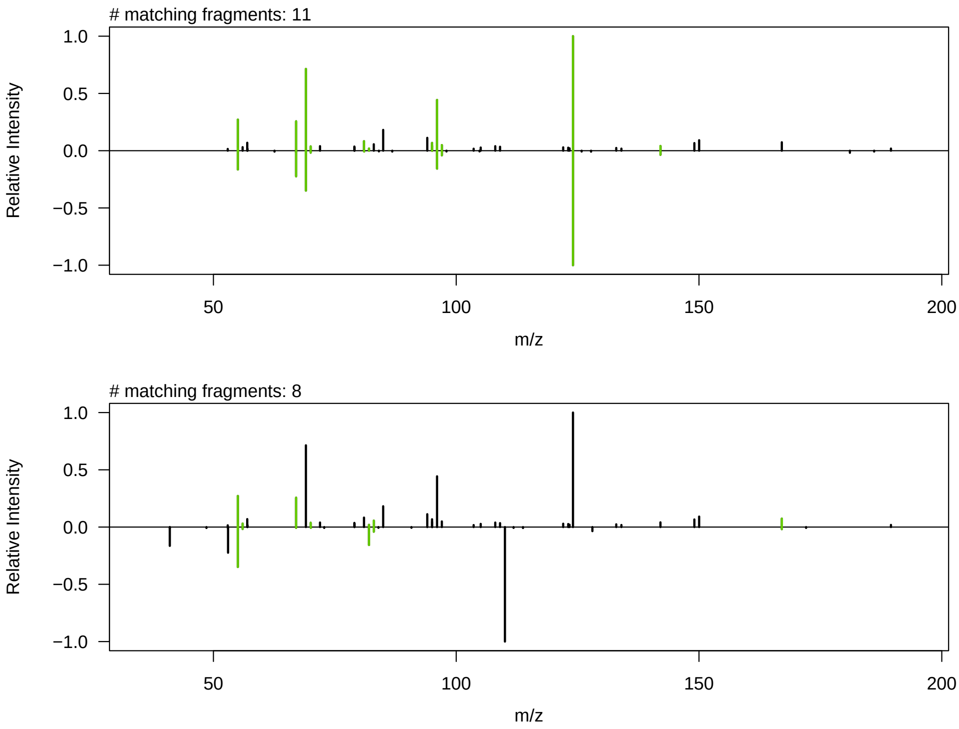

Figure SI-D126: Head to tail plots of 3-methylxanthine and paraxanthine/theophylline. In the bottom plot, the mass spectrum of paraxanthine/theophylline is shifted by the mass difference. Matching fragments are highlighted in green.

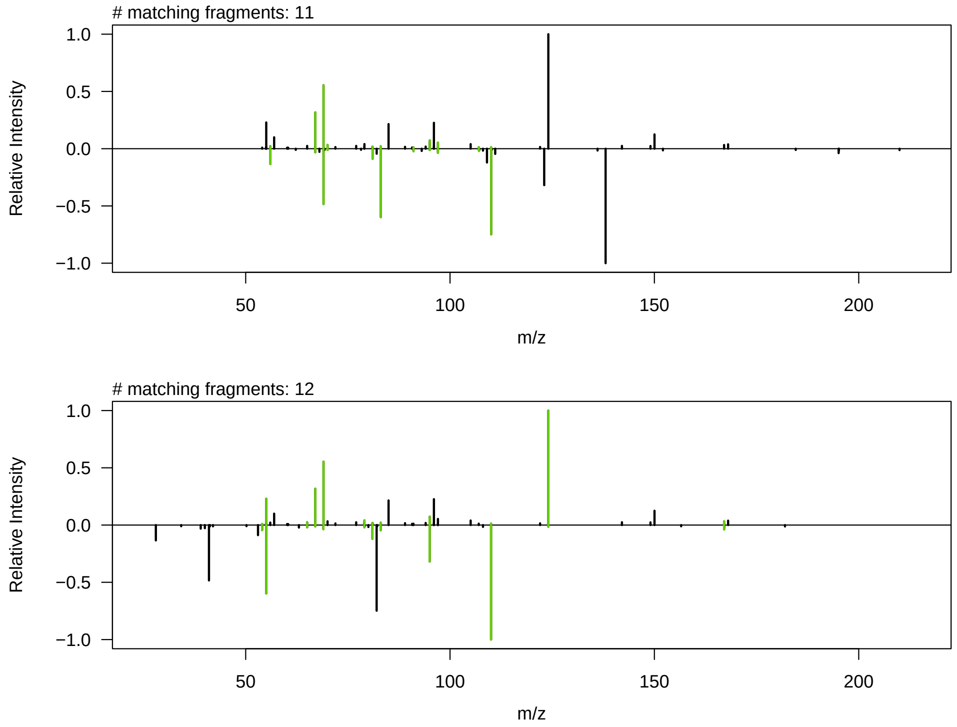

Figure SI-D127: Head to tail plots of 7-methylxanthine and caffeine. In the bottom plot, the mass spectrum of caffeine is shifted by the mass difference. Matching fragments are highlighted in green.

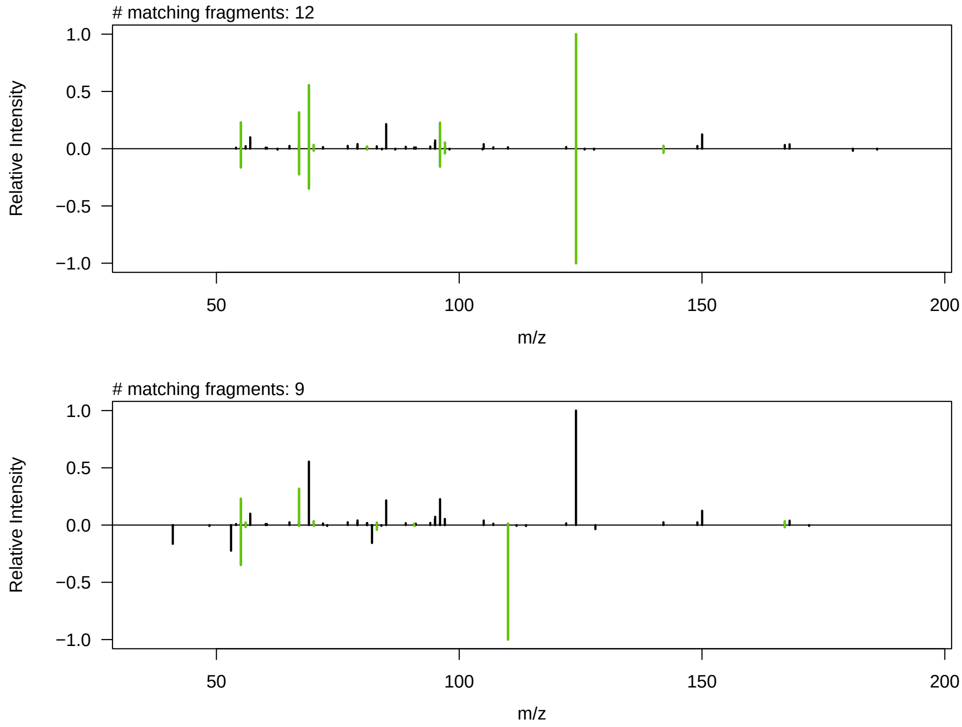

Figure SI-D128: Head to tail plots of 7-methylxanthine and paraxanthine/theophylline. In the bottom plot, the mass spectrum of paraxanthine/theophylline is shifted by the mass difference. Matching fragments are highlighted in green.

Table SI-D59: Molecular network results and retention time prediction of 3-methylxanthine.

Comparison with	Caffeine
MSn Score	52
Forward coverage	31
Reverse coverage	73
Forward match	60
Reverse match	36
ΔMass [g/mol]	28.0313
Comparison with	Paraxanthine/Theophylline
MSn Score	76
Forward coverage	62
Reverse coverage	90
Forward match	121
Reverse match	53
ΔMass [g/mol]	14.0157
Measured retention time [min]	9.7
Predicted $\log D_{OW}$ (pH = 2.7)	-0.99
Predicted retention time [min]	13.4
Predicted retention time range (95% confidence interval) [min]	8.8-18.0
Predicted retention time range (99% confidence interval) [min]	7.4-19.5

Table SI-D60: Molecular network results and retention time prediction of 7-methylxanthine.

Comparison with	Caffeine
MSn Score	52
Forward coverage	31
Reverse coverage	73
Forward match	60
Reverse match	36
ΔMass [g/mol]	28.0313
Comparison with	Paraxanthine/Theophylline
MSn Score	76
Forward coverage	62
Reverse coverage	90
Forward match	121
Reverse match	53
ΔMass [g/mol]	14.0157
Measured retention time [min]	9.5
Predicted $\log D_{OW}$ (pH = 2.7)	0.02
Predicted retention time [min]	14.7
Predicted retention time range (95% confidence interval) [min]	10.1-19.3
Predicted retention time range (99% confidence interval) [min]	8.7-20.8

Table SI-D61: Annotated MS2 spectrum of 3-methylxanthine.

m/z	Relative Intensity	Annotation
52.9462	15.31	
55.0272	21.65	
55.0295	271.23	$C_2H_2N_2 + H^+$
56.0134	30.79	$C_2HNO + H^+$
56.9650	69.31	
57.0211	15.62	$C_2H_2NO + H^+$
67.0293	256.27	$C_3H_2N_2 + H^+$
69.0448	713.30	$C_3H_4N_2 + H^+$
70.0287	37.36	$C_3H_3NO + H^+$
71.9520	39.55	
79.0290	36.26	$C_4H_2N_2 + H^+$
79.0540	29.07	
81.0083	82.56	$C_3N_2O + H^+$
82.0400	19.16	$C_3H_3N_3 + H^+$
83.0240	56.19	$C_3H_2N_2O + H^+$
84.9597	181.37	
94.0400	112.08	$C_4H_3N_3 + H^+$
95.0239	67.86	$C_4H_2N_2O + H^+$
95.0490	45.57	$C_6H_6O + H^+$
96.0556	442.63	$C_4H_5N_3 + H^+$
97.0398	48.94	$C_4H_4N_2O + H^+$
103.5923	17.35	
105.0448	27.63	$C_6H_4N_2 + H^+$
108.0431	39.24	$C_4H_3N_4 + H^+$
109.0271	34.35	$C_4H_2N_3O + H^+$
122.0350	29.12	$C_5H_3N_3O + H^+$
123.0665	26.35	$C_5H_6N_4 + H^+$
123.3410	20.56	
124.0505	999.00	$C_5H_5N_3O + H^+$
132.9614	23.80	
134.0218	17.98	$C_5HN_4O + H^+$
142.0611	41.01	$C_5H_7N_3O_2 + H^+$
149.0459	66.20	$C_6H_4N_4O + H^+$
150.0299	91.44	$C_6H_3N_3O_2 + H^+$
167.0562	73.47	$C_6H_6N_4O_2 + H^+$
189.5330	18.57	

,

Table SI-D62: Annotated MS2 spectrum of 7-methylxanthine.

m/z	Relative Intensity	Annotation
54.0382	9.16	
54.9367	8.39	
55.0269	13.07	
55.0295	229.10	$C_2H_2N_2 + H^+$
56.0134	21.91	$C_2HNO + H^+$
56.9652	99.57	
60.1440	9.15	
60.3975	8.42	
65.0386	24.19	$C_5H_4 + H^+$
67.0258	18.58	
67.0292	316.54	$C_3H_2N_2 + H^+$
69.0448	553.61	$C_3H_4N_2 + H^+$
70.0287	32.90	$C_3H_3NO + H^+$
71.9518	14.22	
77.0385	24.32	$C_6H_4 + H^+$
79.0288	16.72	$C_4H_2N_2 + H^+$
79.0541	40.46	
81.0084	16.53	$C_3N_2O + H^+$
81.0331	10.94	
81.0449	18.10	$C_4H_4N_2 + H^+$
83.0238	20.97	$C_3H_2N_2O + H^+$
84.9596	214.72	
88.9901	16.36	
90.7164	10.88	
91.0540	11.72	
94.0402	18.45	$C_4H_3N_3 + H^+$
95.0240	52.62	$C_4H_2N_2O + H^+$
95.0491	72.28	$C_6H_6O + H^+$
96.0500	16.18	
96.0556	225.91	$C_4H_5N_3 + H^+$
97.0342	10.09	
97.0393	53.30	$C_4H_4N_2O + H^+$
105.0447	39.65	$C_6H_4N_2 + H^+$
107.0238	12.20	$C_5H_2N_2O + H^+$
110.0354	12.66	$C_4H_3N_3O + H^+$
122.0345	15.03	$C_5H_3N_3O + H^+$

Continued on next page

Table SI-D62: Annotated MS2 spectrum of 7-methylxanthine.(Continued)

124.0504	999.00	$C_5H_5N_3O + H^+$
142.0608	24.50	$C_5H_7N_3O_2 + H^+$
149.0458	23.50	$C_6H_4N_4O + H^+$
150.0298	124.87	$C_6H_3N_3O_2 + H^+$
167.0564	32.43	$C_6H_6N_4O_2 + H^+$
168.0402	38.37	

A reference standard of 7-methylxanthine was purchased. Figure SI-D129 shows the extracted ion chromatograms of this standard, the sample and the spiked sample, as well as a head to tail plot of the MS2 spectra of the standard and the sample. In addition, the most intense MS2 fragments in the sample and in the standard are displayed. It becomes visible that the retention times of the sample and the spiked sample are identical and the spectra similarity score between sample and standard is equal to 0.515. The majority of the MS2 fragments in the sample can be explained by the reference standard. It can therefore be concluded that the suspected compound is indeed 7-methylxanthine. Correspondingly, the identification confidence can be increased to level 1.

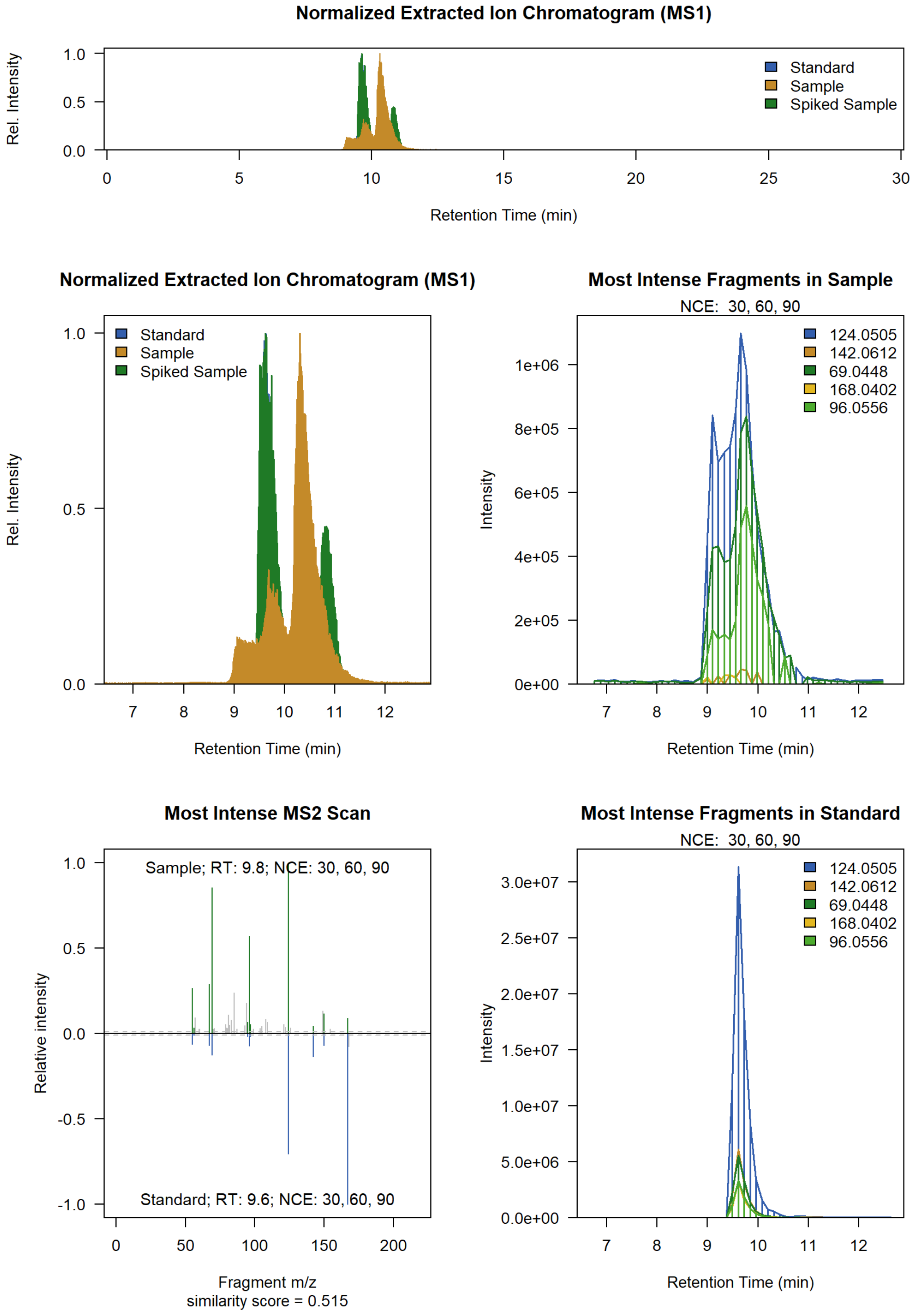

Figure SI-D129: Extracted ion chromatograms of 7-methylxanthine in the reference standard, the sample and the spiked sample, as well as MS2 head to tail plot and most intense MS2 fragments in standard and sample. The first eluting peak originates from 3-methylxanthine, the second from 7-methylxanthine and the last from 1-methylxanthine.

SI-D2.6.5 1-Methyluric Acid

Table SI-D63: Information on identifiers, chemical properties, detection and confidence of identification of 1-methyluric acid.

IUPAC Name	1-methyl-7,9-dihydro-3*H*-purine-2,6,8-trione
Molecular formula	$C_6H_6N_4O_3$
Monoisotopic mass [g/mol]	182.0440
Adduct	$[M+H]^+$
Retention time [min]	9.5
SMILES	CN1C(=O)C2=C(NC(=O)N2)NC1=O
InChI	InChI=1S/C6H6N4O3/c1-10-4(11)2-3(9-6(10)13)8-5(12)7-2/h1H3,(H,9,13)(H2,7,8,12)
InChI-Key	QFDRTQONISXGJA-UHFFFAOYSA-N
CAS RN	708-79-2
Metabolite of	Caffeine, theophylline
Detection frequency	100% (15/15 samples)
Detected in	Altenrhein, Monday-Friday<br>Neugut, Monday-Friday<br>Werdhölzli, Monday-Friday
Intensity	E7
Initial confidence level	level 2a
Initial confidence score	0.48
Final confidence level	level 1

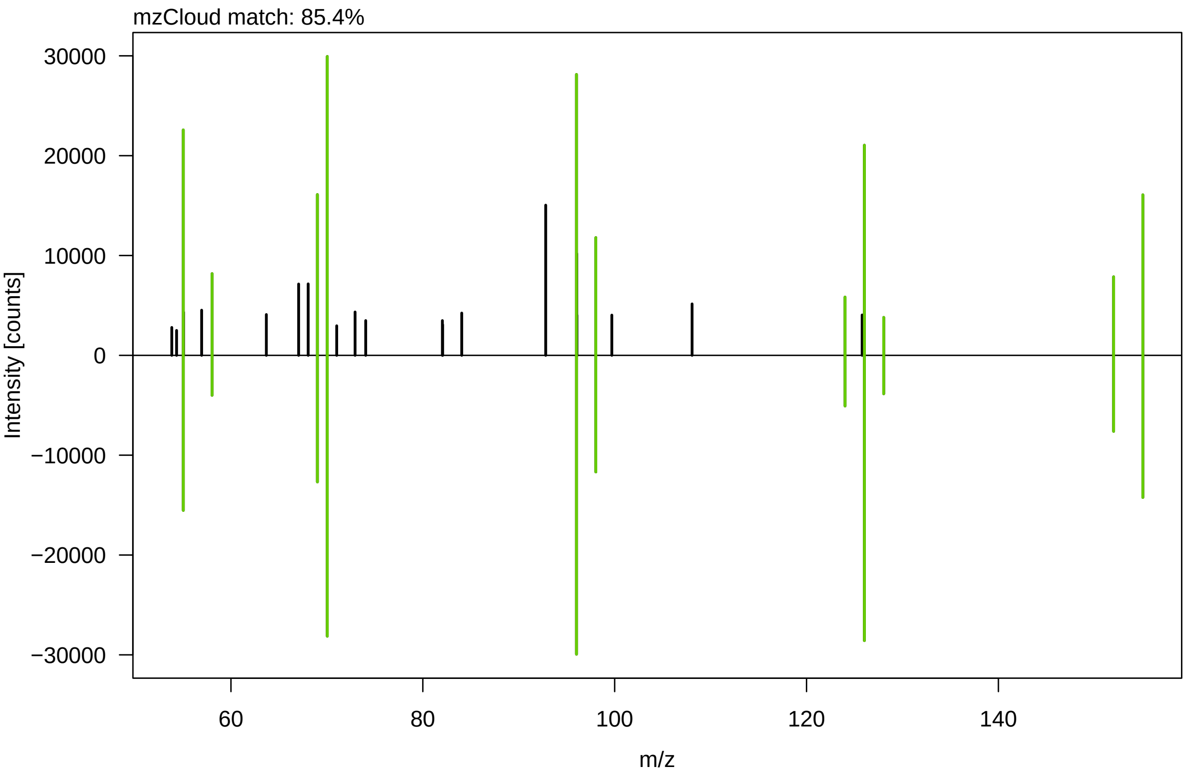

Figure SI-D130: Head to tail plot of measured MS2 spectrum against mzCloud library spectrum of 1-methyluric acid. Matching fragments are highlighted in green.

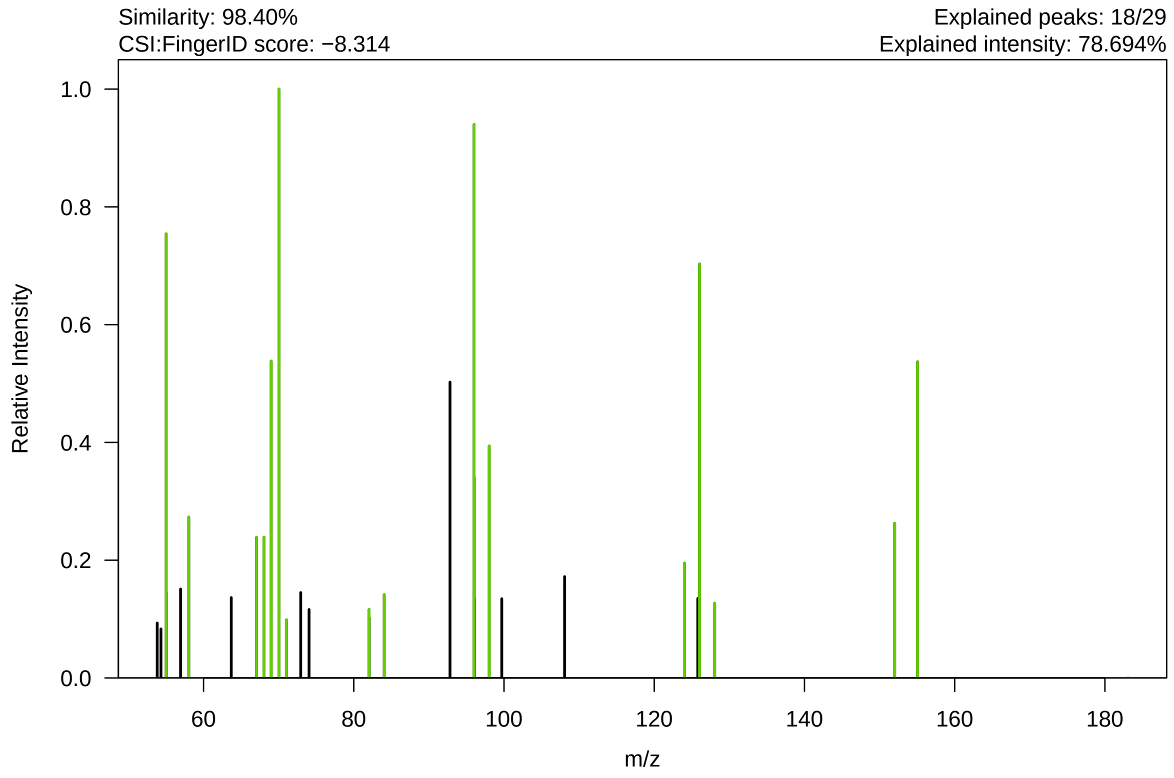

Figure SI-D131: Measured MS2 spectrum. Matching fragments with 1-methyluric acid predicted by SIRIUS/CSI:FingerID are highlighted in green.

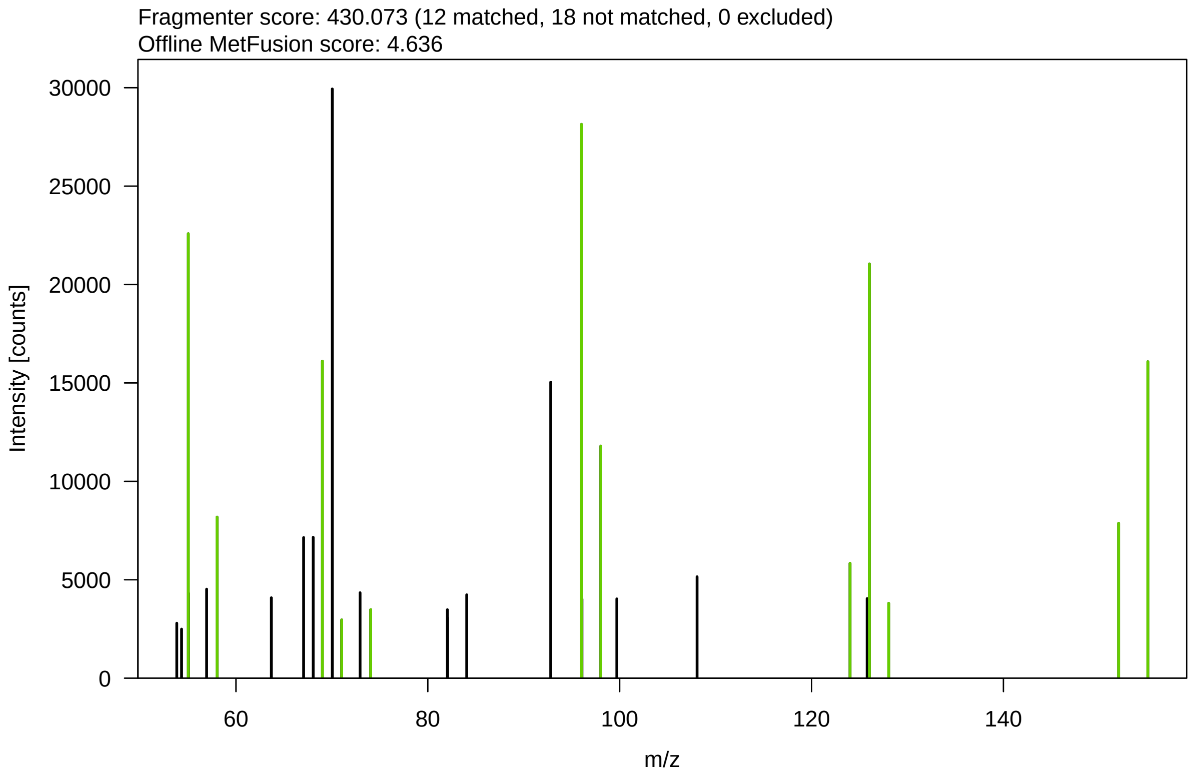

Figure SI-D132: Measured MS2 spectrum. Matching fragments with 1-methyluric acid predicted by MetFrag are highlighted in green.

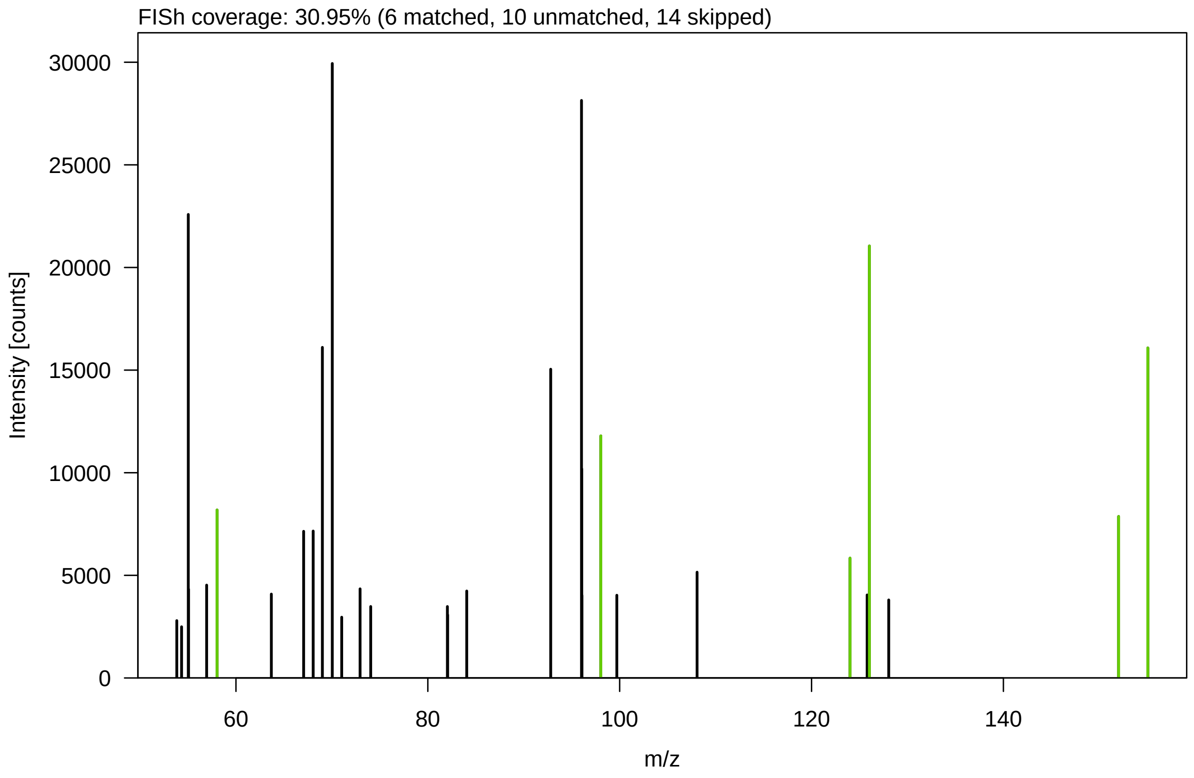

Figure SI-D133: Measured MS2 spectrum. Matching fragments with 1-methyluric acid predicted by FISh Scoring are highlighted in green. Low intensity fragments are not considered and skipped.

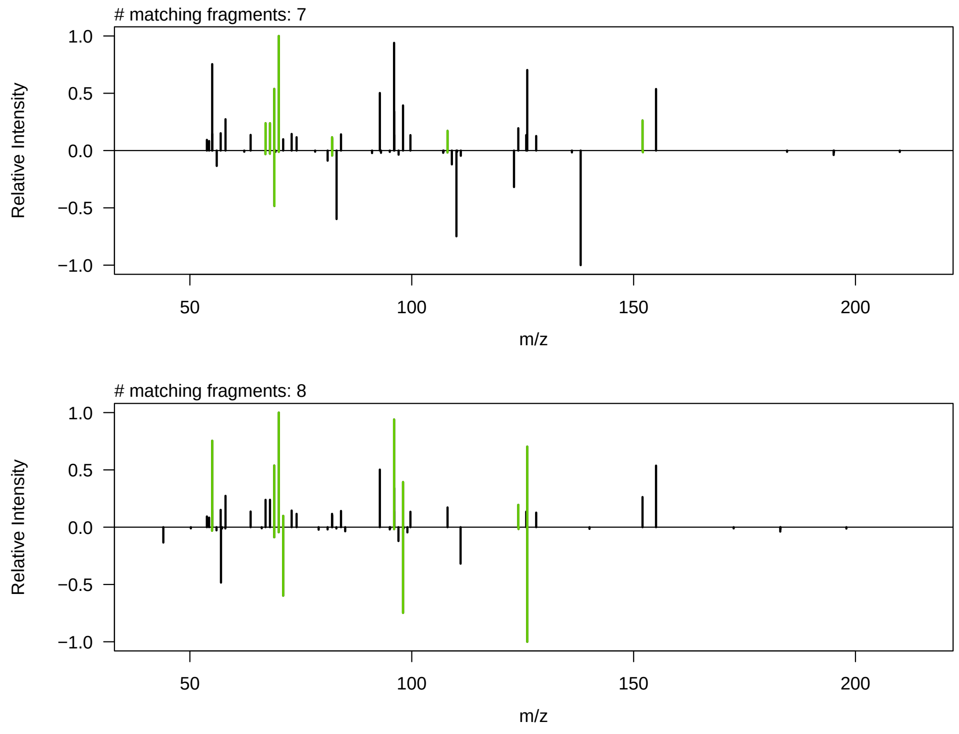

Figure SI-D134: Head to tail plots of 1-methyluric acid and caffeine. In the bottom plot, the mass spectrum of caffeine is shifted by the mass difference. Matching fragments are highlighted in green.

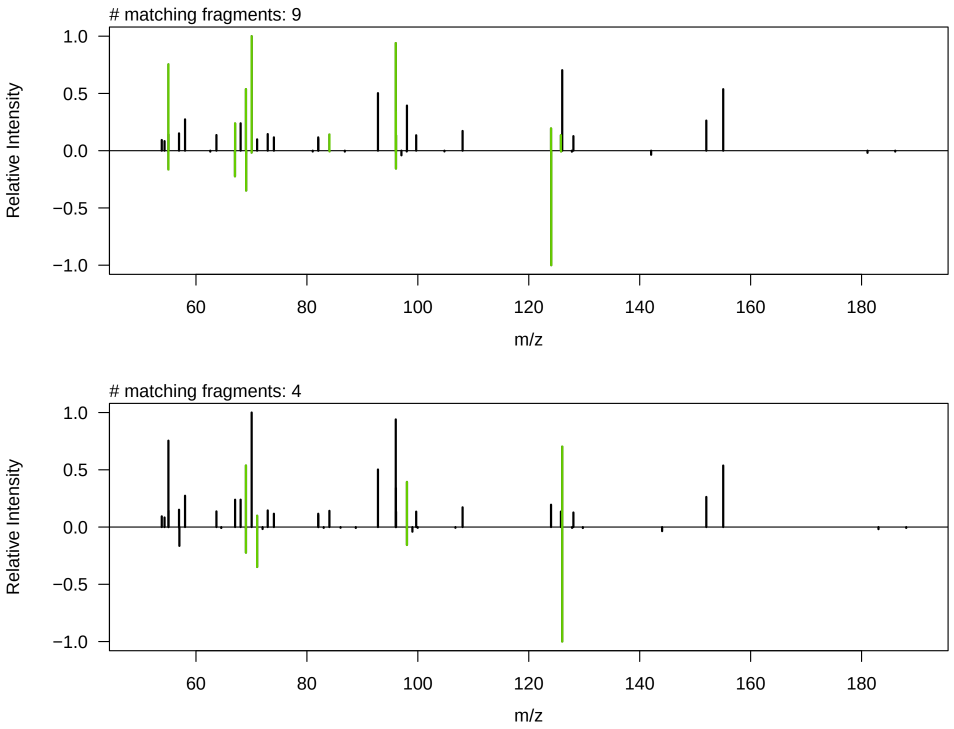

Figure SI-D135: Head to tail plots of 1-methyluric acid and paraxanthine/theophylline. In the bottom plot, the mass spectrum of paraxanthine/theophylline is shifted by the mass difference. Matching fragments are highlighted in green.

Table SI-D64: Molecular network results and retention time prediction of 1-methyluric acid.

Comparison with	Caffeine
MSn Score	30
Forward coverage	33
Reverse coverage	27
Forward match	14
Reverse match	13
ΔMass [g/mol]	12.0364
Comparison with	Paraxanthine/Theophylline
MSn Score	41
Forward coverage	27
Reverse coverage	55
Forward match	16
Reverse match	23
ΔMass [g/mol]	1.9793
Measured retention time [min]	9.5
Predicted $\log D_{OW}$ (pH = 2.7)	-1.32
Predicted retention time [min]	13.0
Predicted retention time range (95% confidence interval) [min]	8.4-17.6
Predicted retention time range (99% confidence interval) [min]	7.0-19.1

Table SI-D65: Annotated MS2 spectrum of 1-methyluric acid.

m/z	Relative Intensity	Annotation
53.8305	93.23	
54.3312	83.22	
55.0295	753.57	$C_2H_2N_2 + H^+$
55.0545	144.25	$C_4H_6 + H^+$
56.9424	150.96	
58.0291	273.09	$C_2H_3NO + H^+$
63.6889	136.38	
67.0543	238.45	$C_5H_6 + H^+$
68.0495	238.86	$C_4H_5N + H^+$
69.0083	537.47	$C_2N_2O + H^+$
70.0401	999.00	$C_2H_3N_3 + H^+$
71.0238	98.81	$C_2H_2N_2O + H^+$
72.9370	144.89	
74.0473	116.19	$C_2H_5N_2O + H^+$
82.0398	116.12	$C_3H_3N_3 + H^+$
82.0648	102.54	
84.0555	141.38	$C_3H_5N_3 + H^+$
92.8100	501.97	
96.0193	938.94	$C_3HN_3O + H^+$
96.0443	339.86	$C_5H_5NO + H^+$
96.0685	134.38	
98.0349	393.55	$C_3H_3N_3O + H^+$
99.7027	134.48	
108.0684	171.98	
124.0138	194.84	$C_4HN_3O_2 + H^+$
125.7966	134.98	
126.0301	702.35	$C_4H_3N_3O_2 + H^+$
128.0463	126.84	$C_4H_5N_3O_2 + H^+$
152.0091	262.58	$C_5HN_3O_3 + H^+$
155.0561	536.57	$C_5H_6N_4O_2 + H^+$

A reference standard of 1-methyluric acid was purchased. Figure SI-D136 shows the extracted ion chromatograms of this standard, the sample and the spiked sample, as well as a head to tail plot of the MS2 spectra of the standard and the sample. In addition, the most intense MS2 fragments in the sample and in the standard are displayed. It becomes visible that the retention times of the sample and the spiked sample are identical and the spectra similarity score between sample and standard is equal to 0.420. The majority of the MS2 fragments in the sample can be explained by the reference standard. It can therefore be concluded that the suspected compound is indeed 1-methyluric acid. Correspondingly, the identification confidence is increased to level 1.

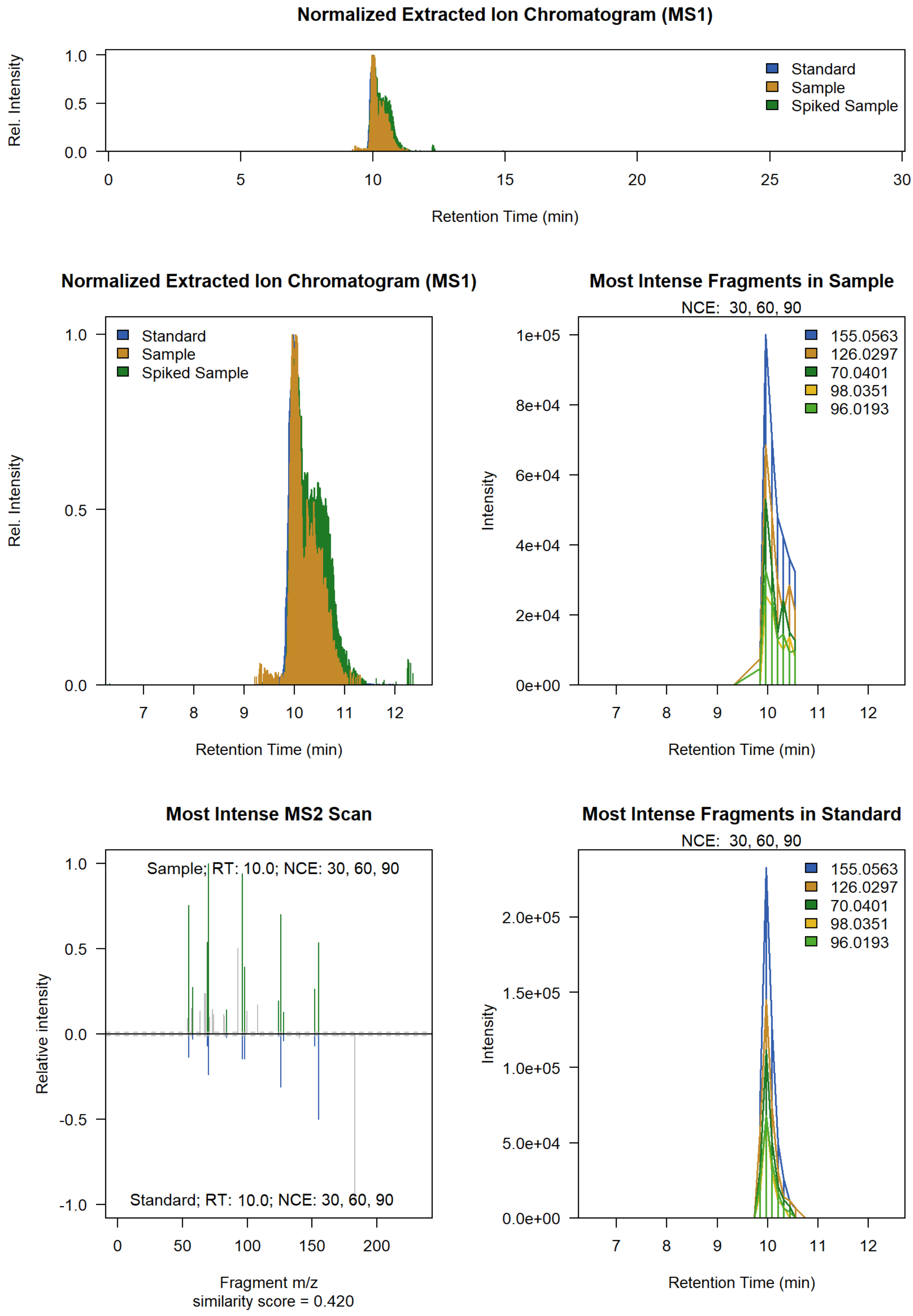

Figure SI-D136: Extracted ion chromatograms of 1-methyluric acid in the reference standard, the sample and the spiked sample, as well as MS2 head to tail plot and most intense MS2 fragments in standard and sample.

SI-D2.6.6 1,7-Dimethyluric Acid

Table SI-D66: Information on identifiers, chemical properties, detection and confidence of identification of 1,7-dimethyluric acid.

IUPAC Name	1,7-dimethyl-3,9-dihydropurine-2,6,8-trione
Molecular formula	$C_7H_8N_4O_3$
Monoisotopic mass [g/mol]	196.0596
Adduct	$[M+H]^+$
Retention time [min]	11.8
SMILES	CN1C2=C(NC1=O)NC(=O)N(C2=O)C
InChI	InChI=1S/C7H8N4O3/c1-10-3-4(8-6(10)13)9-7(14)11(2)5(3)12/h1-2H3,(H,8,13)(H,9,14)
InChI-Key	NOFNCLGCUJJPKU-UHFFFAOYSA-N
CAS RN	33868-03-0
Metabolite of	Theophylline, caffeine
Detection frequency	100% (15/15 samples)
Detected in	Altenrhein, Monday-Friday<br>Neugut, Monday-Friday<br>Werdhölzli, Monday-Friday
Intensity	E8
Initial confidence level	level 2a
Initial confidence score	0.69
Final confidence level	level 1

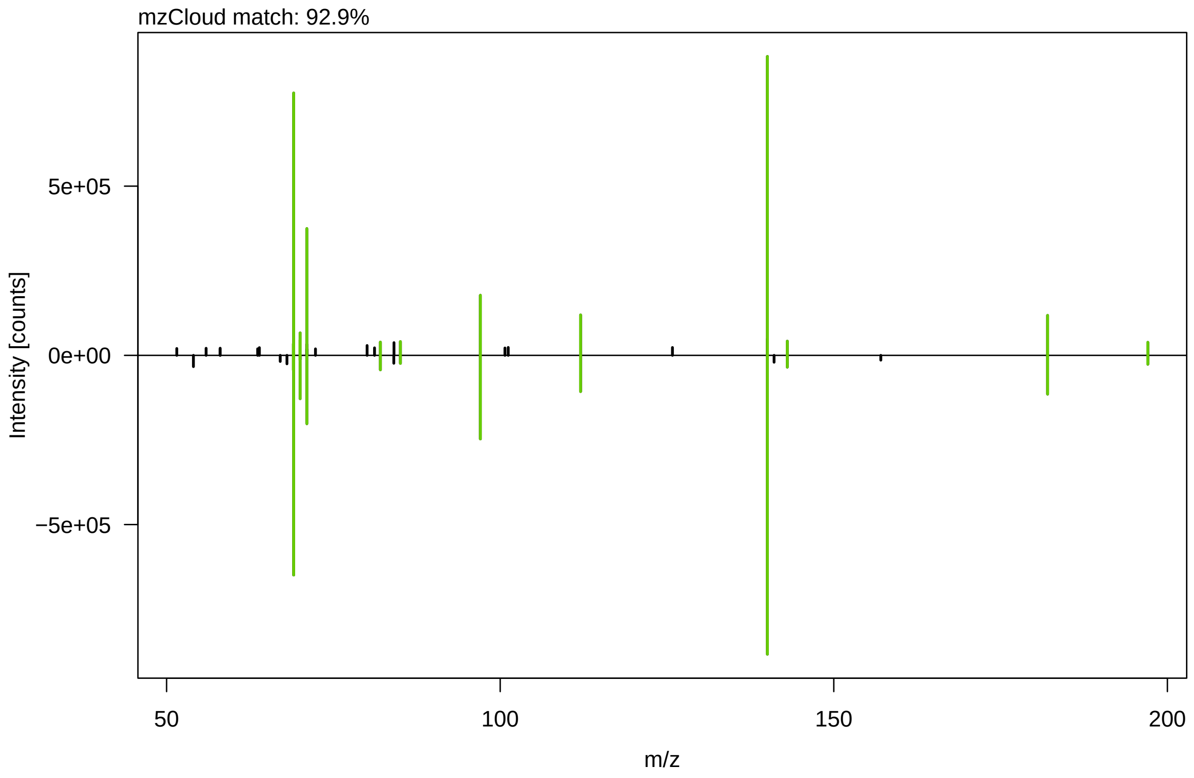

Figure SI-D137: Head to tail plot of measured MS2 spectrum against mzCloud library spectrum of 1,7-dimethyluric acid. Matching fragments are highlighted in green.

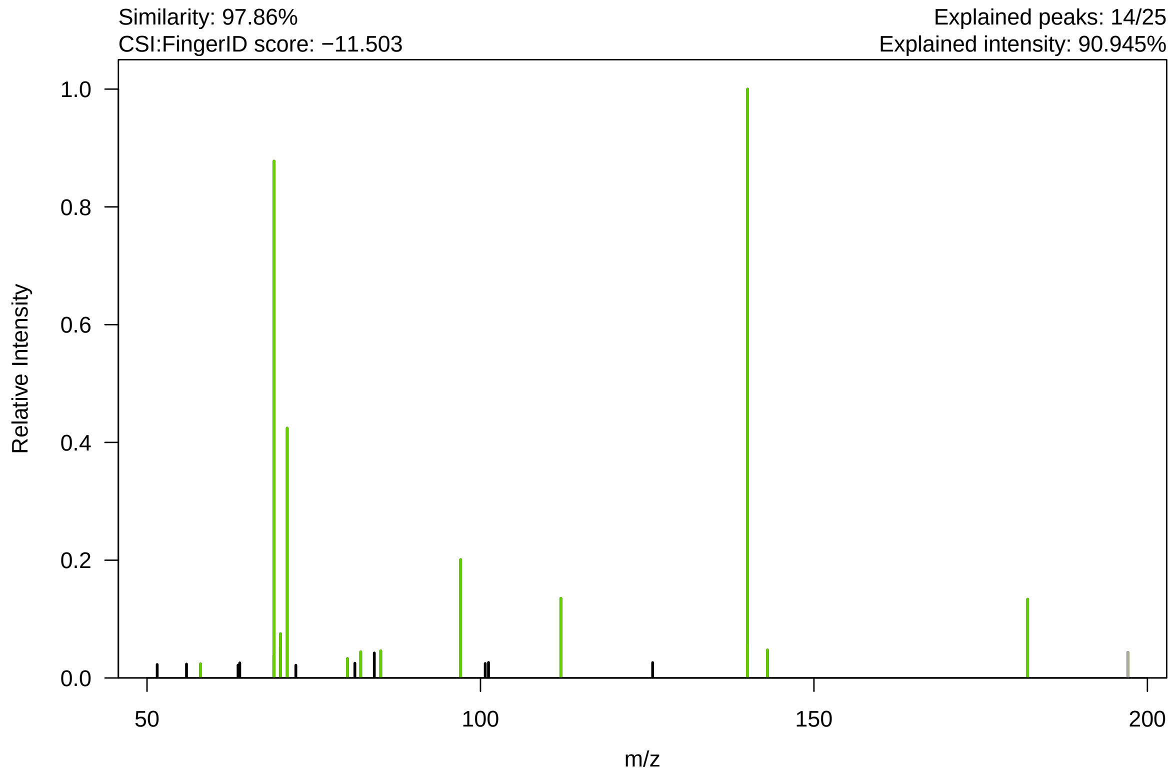

Figure SI-D138: Measured MS2 spectrum. Matching fragments with 1,7-dimethyluric acid predicted by SIRIUS/CSI:FingerID are highlighted in green. The molecular ion in gray is not considered.

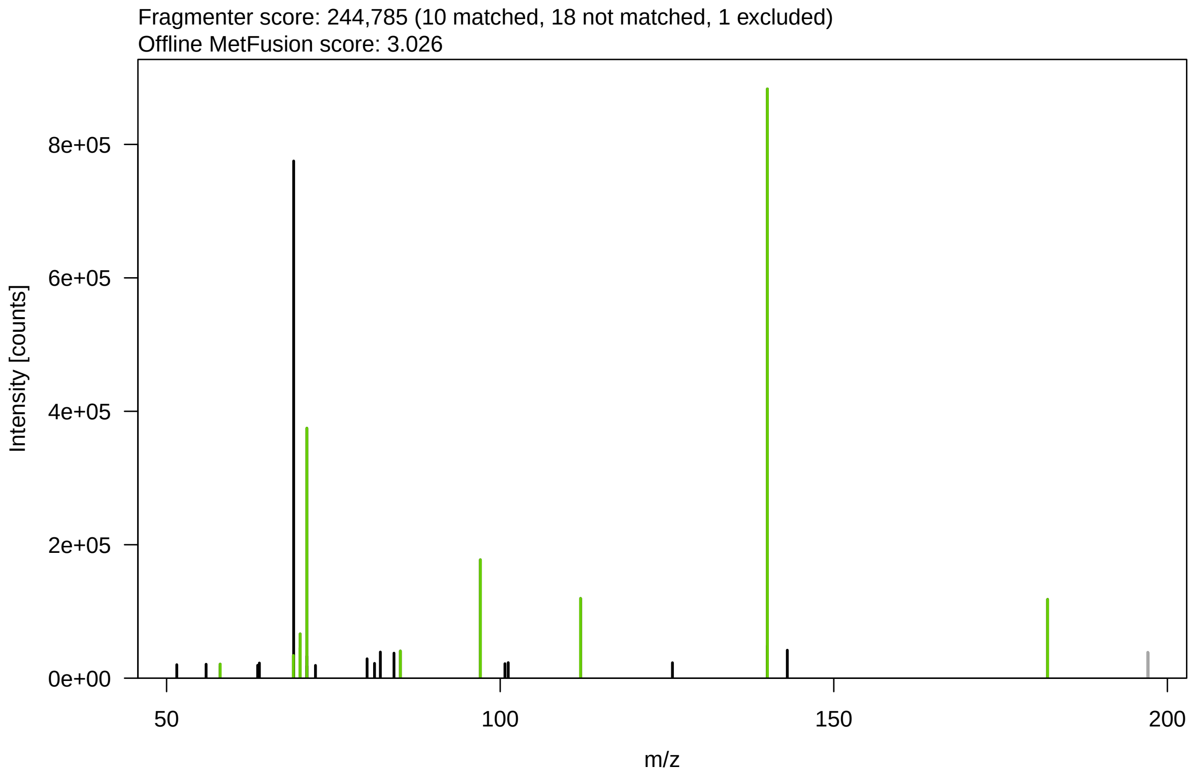

Figure SI-D139: Measured MS2 spectrum. Matching fragments with 1,7-dimethyluric acid predicted by MetFrag are highlighted in green. The molecular ion in gray is not considered.

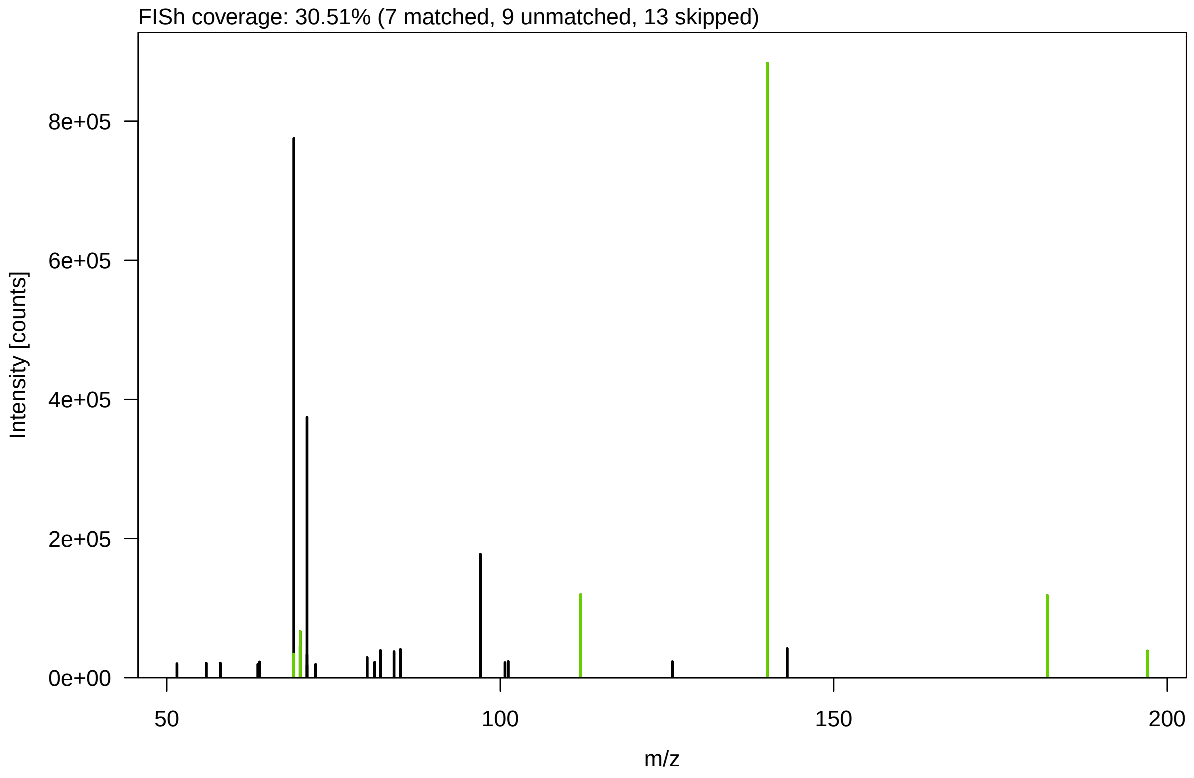

Figure SI-D140: Measured MS2 spectrum. Matching fragments with 1,7-dimethyluric acid predicted by FISh Scoring are highlighted in green. Low intensity fragments are not considered and skipped.

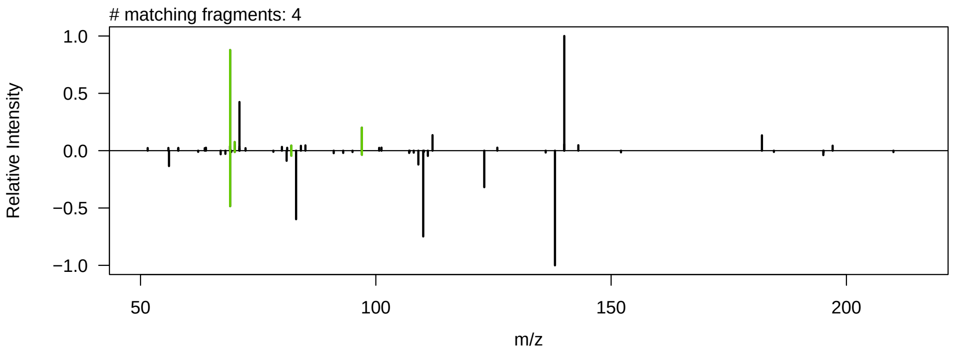

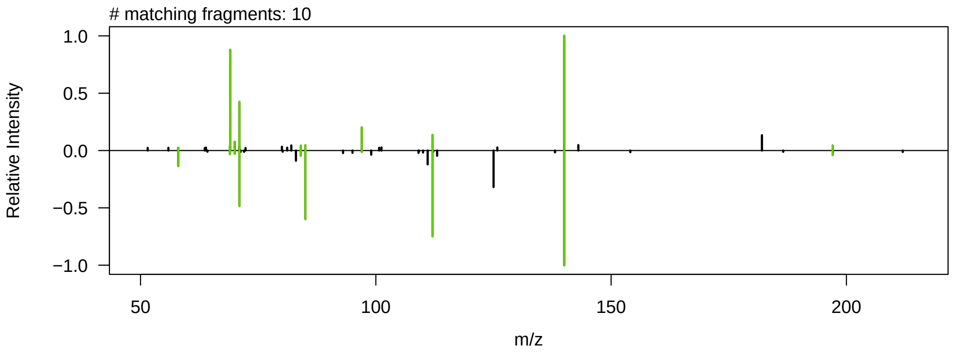

Figure SI-D141: Head to tail plots of 1,7-dimethyluric acid and caffeine. In the bottom plot, the mass spectrum of caffeine is shifted by the mass difference. Matching fragments are highlighted in green.

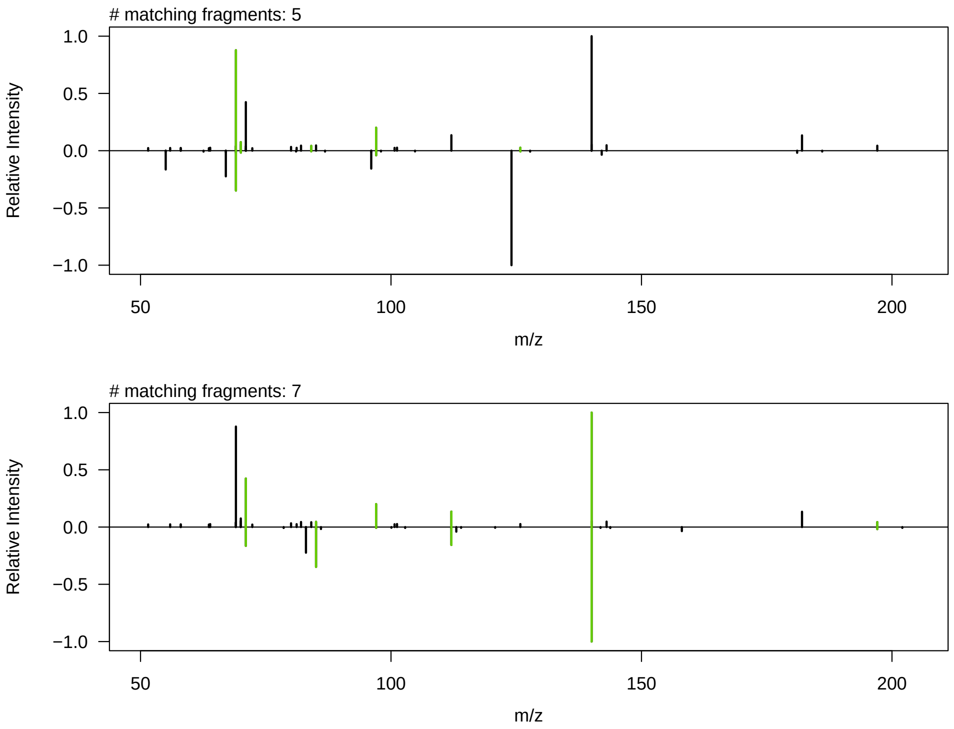

Figure SI-D142: Head to tail plots of 1,7-dimethyluric acid and paraxanthine/theophylline. In the bottom plot, the mass spectrum of paraxanthine/theophylline is shifted by the mass difference. Matching fragments are highlighted in green.

Table SI-D67: Molecular network results and retention time prediction of 1,7-dimethyluric acid.

Comparison with	Caffeine
MSn Score	49
Forward coverage	55
Reverse coverage	42
Forward match	27
Reverse match	25
ΔMass [g/mol]	1.9793
Comparison with	Paraxanthine/Theophylline
MSn Score	42
Forward coverage	41
Reverse coverage	42
Forward match	24
Reverse match	25
ΔMass [g/mol]	15.9949
Measured retention time [min]	11.8
Predicted logD_{OW} (pH = 2.7)	-1.10
Predicted retention time [min]	13.3
Predicted retention time range (95% confidence interval) [min]	8.7-17.9
Predicted retention time range (99% confidence interval) [min]	7.3-19.4

Table SI-D68: Annotated MS2 spectrum of 1,7-dimethyluric acid.

m/z	Relative Intensity	Annotation
51.5271	22.85	
55.9207	23.60	
58.0289	23.83	$C_2H_3NO + H^+$
58.0317	20.32	
63.6496	21.83	
63.9059	25.65	
69.0083	38.55	$C_2N_2O + H^+$
69.0448	876.79	$C_3H_4N_2 + H^+$
69.9923	51.74	$C_2H_1N_2O + H^+$
70.0162	75.08	$C_2HN_2O + H^+$
71.0204	37.38	
71.0240	423.78	$C_2H_2N_2O + H^+$
71.0279	20.52	
72.3152	21.62	
80.0494	32.84	$C_5H_5N + H^+$
81.1653	25.07	
82.0397	44.31	$C_3H_3N_3 + H^+$
84.0808	42.40	
85.0395	46.01	$C_3H_4N_2O + H^+$
97.0270	200.72	$C_3H_2N_3O + H^+$
100.7115	24.55	
101.2042	26.33	
112.0503	135.14	$C_4H_5N_3O + H^+$
125.8169	26.14	
140.0346	54.40	$C_6H_5NO_3 + H^+$
140.0454	999.00	$C_5H_5N_3O_2 + H^+$
143.0323	47.40	$C_4H_4N_3O_3 + H^+$
182.0431	133.57	$C_6H_5N_4O_3 + H^+$
197.0677	43.44	$C_7H_8N_4O_3 + H^+$

A reference standard of 1,7-dimethyluric acid was purchased. Figure SI-D143 shows the extracted ion chromatograms of this standard, the sample and the spiked sample, as well as a head to tail plot of the MS2 spectra of the standard and the sample. In addition, the most intense MS2 fragments in the sample and in the standard are displayed. It becomes visible that the retention times of the sample and the spiked sample are identical and the spectra similarity score between sample and standard is equal to 0.993. The majority of the MS2 fragments in the sample can be explained by the reference standard. It can therefore be concluded that the suspected compound is indeed 1,7-dimethyluric acid. Correspondingly, the identification confidence can be increased to level 1.

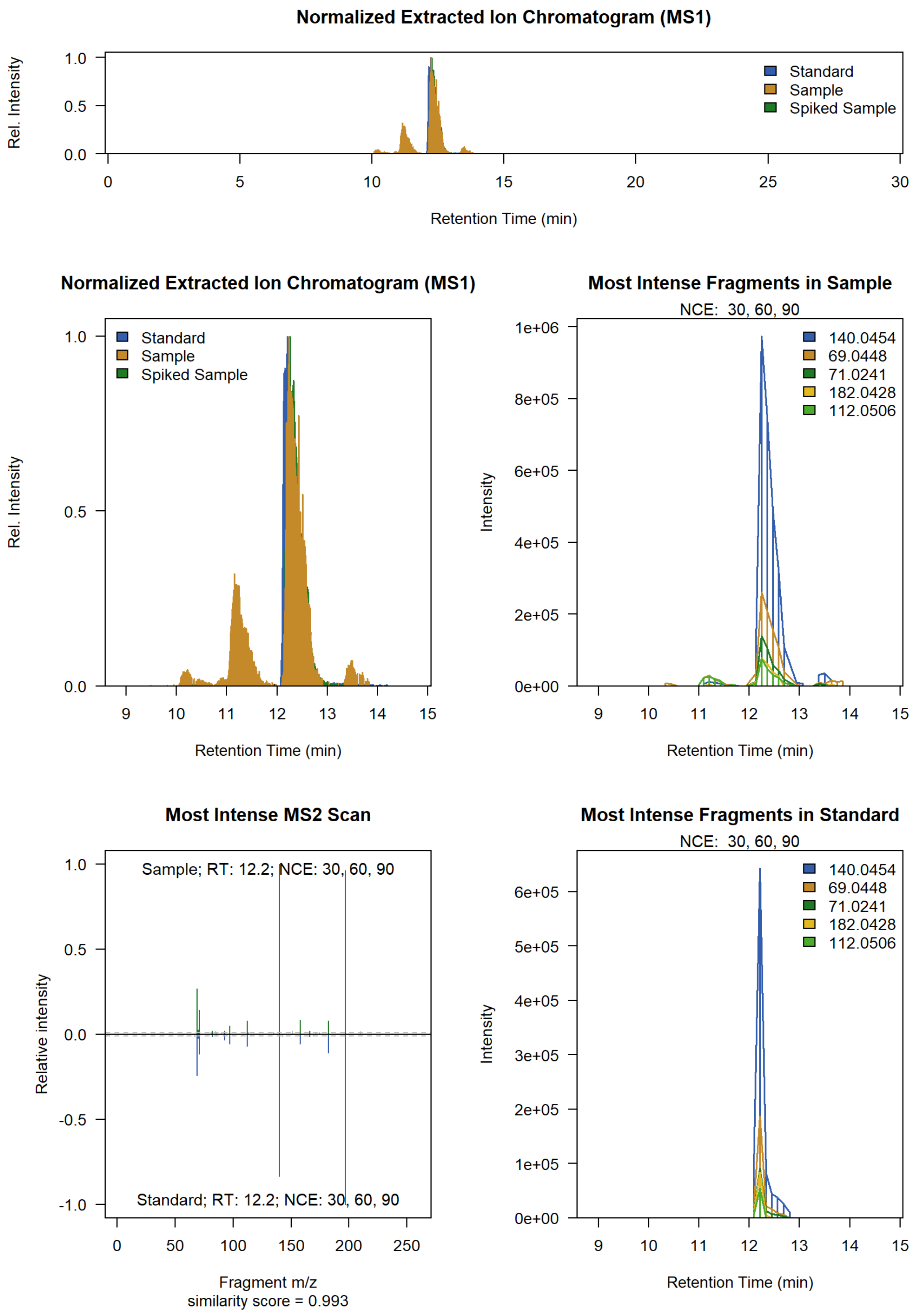

Figure SI-D143: Extracted ion chromatograms of 1,7-dimethyluric acid in the reference standard, the sample and the spiked sample, as well as MS2 head to tail plot and most intense MS2 fragments in standard and sample.

SI-D2.6.7 1,3,7-Trimethyluric Acid

Table SI-D69: Information on identifiers, chemical properties, detection and confidence of identification of 1,3,7-trimethyluric acid.

IUPAC Name	1,3,7-trimethyl-9*H*-purine-2,6,8-trione
Molecular formula	$C_8H_{10}N_4O_3$
Monoisotopic mass [g/mol]	210.0753
Adduct	$[M+H]^+$
Retention time [min]	12.5
SMILES	CN1C2=C(NC1=O)N(C(=O)N(C2=O)C)C
InChI	InChI=1S/C8H10N4O3/c1-10-4-5(9-7(10)14)11(2)8(15)12(3)6(4)13/h1-3H3,(H,9,14)
InChI-Key	BYXCFUMGEBZDDI-UHFFFAOYSA-N
CAS RN	5415-44-1
Metabolite of	Caffeine, theophylline
Detection frequency	100% (15/15 samples)
Detected in	Altenrhein, Monday-Friday<br>Neugut, Monday-Friday<br>Werdhölzli, Monday-Friday
Intensity	E7-E8
Initial confidence level	level 3
Initial confidence score	0.54
Final confidence level	level 1

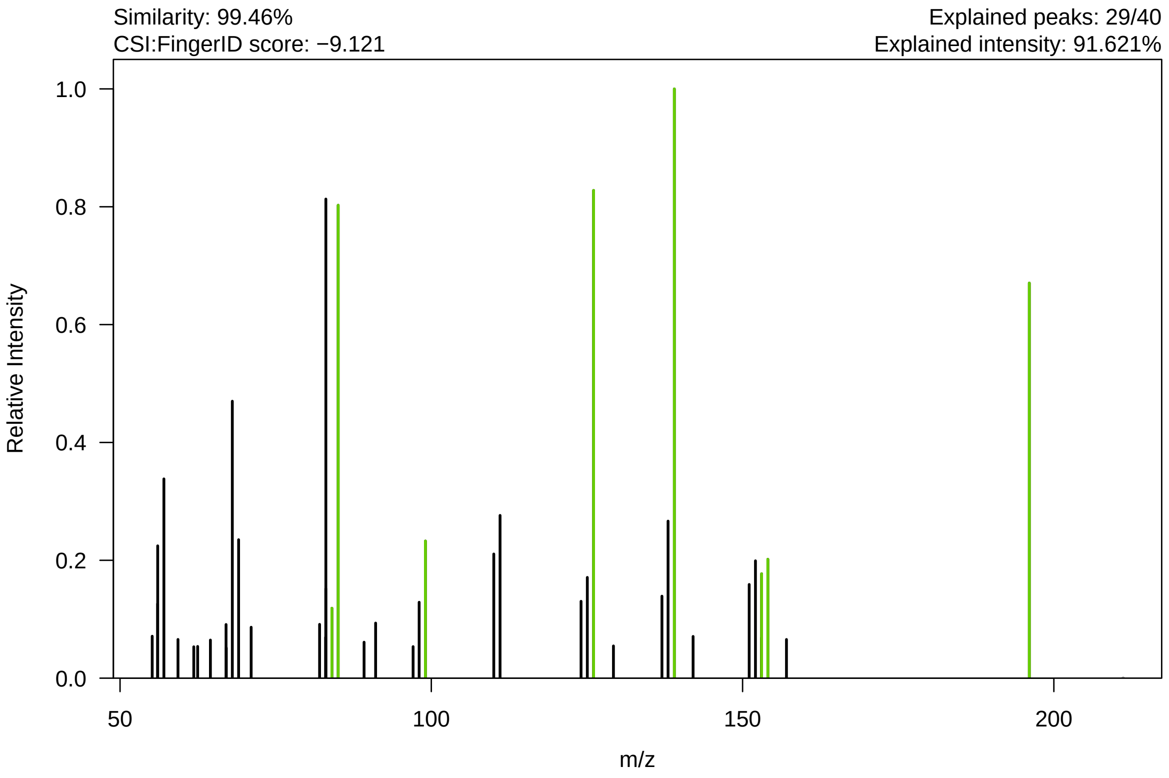

Figure SI-D144: Measured MS2 spectrum. Matching fragments with 1,3,7-trimethyluric acid predicted by SIRIUS/CSI:FingerID are highlighted in green.

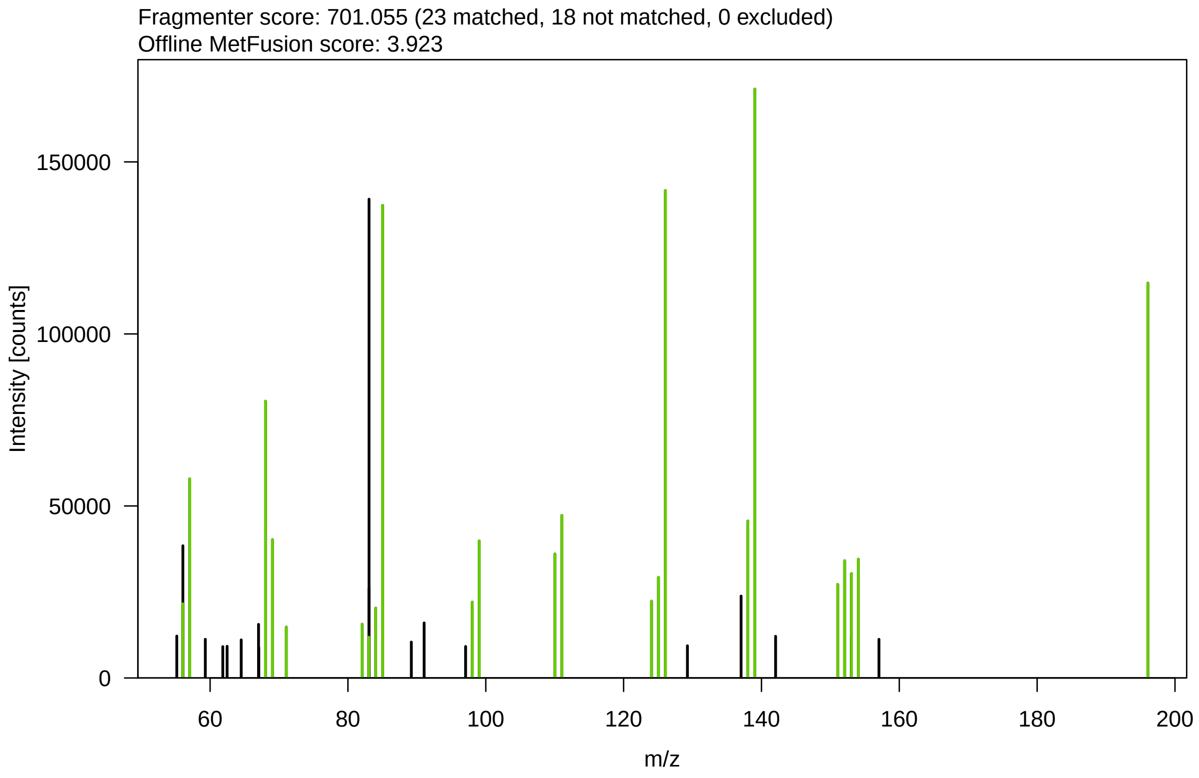

Figure SI-D145: Measured MS2 spectrum. Matching fragments with 1,3,7-trimethyluric acid predicted by MetFrag are highlighted in green.

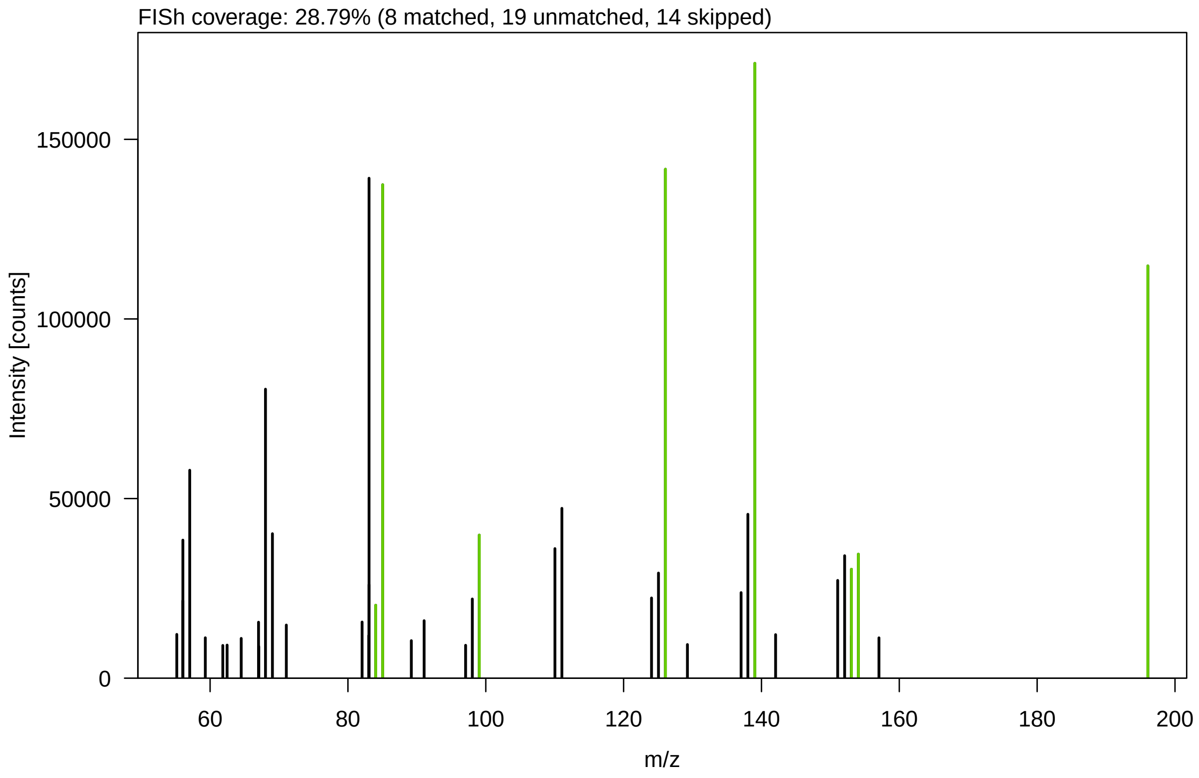

Figure SI-D146: Measured MS2 spectrum. Matching fragments with 1,3,7-trimethyluric acid predicted by FISh Scoring are highlighted in green. Low intensity fragments are not considered and skipped.

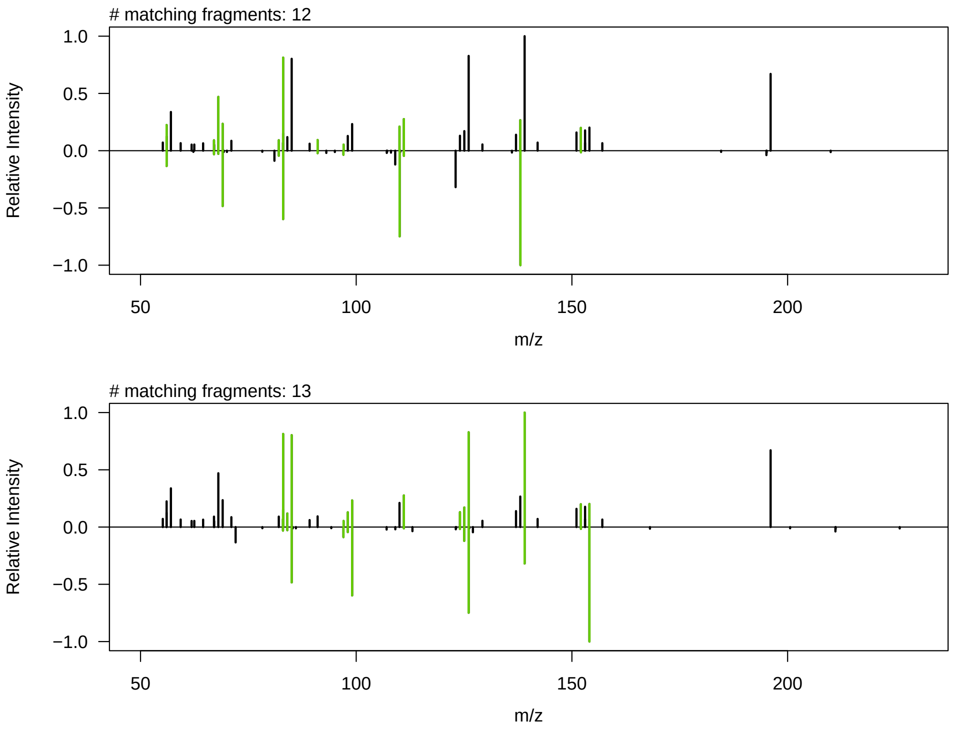

Figure SI-D147: Head to tail plots of 1,3,7-trimethyluric acid and caffeine. In the bottom plot, the mass spectrum of caffeine is shifted by the mass difference. Matching fragments are highlighted in green.

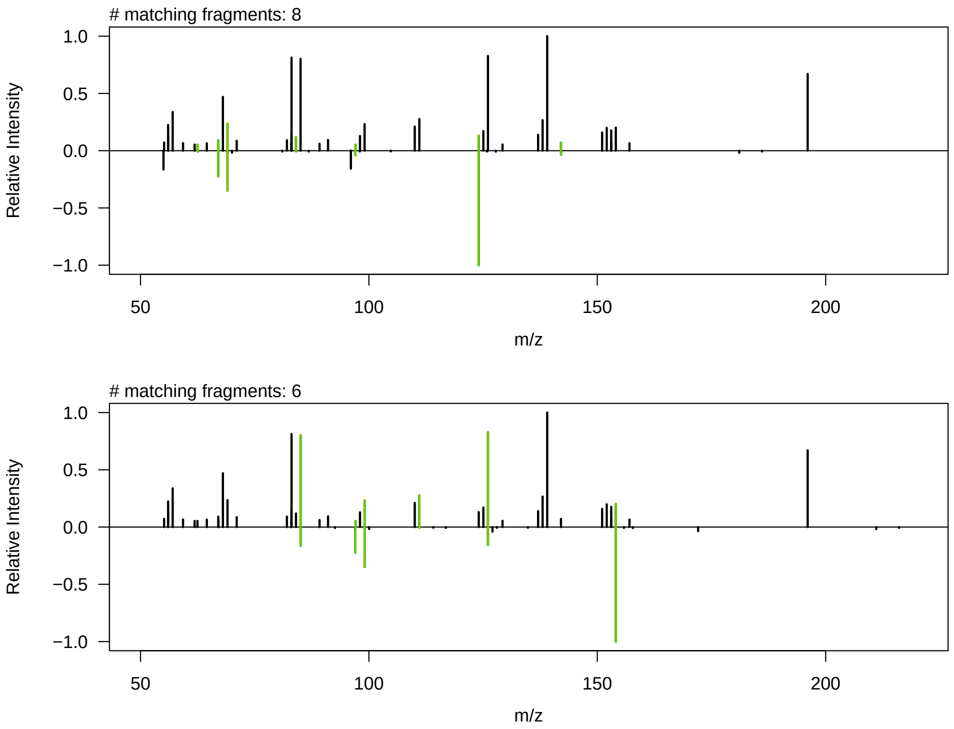

Figure SI-D148: Head to tail plots of 1,3,7-trimethyluric acid and paraxanthine/theophylline. In the bottom plot, the mass spectrum of paraxanthine/theophylline is shifted by the mass difference. Matching fragments are highlighted in green.

Table SI-D70: Molecular network results and retention time prediction of 1,3,7-Trimethyluric acid.

Comparison with	Caffeine
MSn Score	58
Forward coverage	63
Reverse coverage	53
Forward match	31
Reverse match	35
ΔMass [g/mol]	15.9949
Comparison with	Paraxanthine/Theophylline
MSn Score	45
Forward coverage	41
Reverse coverage	50
Forward match	24
Reverse match	33
ΔMass [g/mol]	30.0106
Measured retention time [min]	12.5
Predicted $logD_{OW}$ (pH = 2.7)	-0.87
Predicted retention time [min]	13.6
Predicted retention time range (95% confidence interval) [min]	9.0-18.2
Predicted retention time range (99% confidence interval) [min]	7.5-19.7

Table SI-D71: Annotated MS2 spectrum of 1,3,7-trimethyluric.

m/z	Relative Intensity	Annotation
55.1668	71.10	
56.0372	125.96	$C_2H_3N_2$≡H^+
56.0496	224.31	$C_3H_5N + H^+$
57.0451	337.85	$C_2H_4N_2 + H^+$
59.3064	65.63	
61.8437	53.18	
62.4689	53.74	
64.5150	64.60	
67.0291	90.97	$C_3H_2N_2 + H^+$
67.0693	51.37	
68.0370	469.53	$C_3H_3N_2 + H^+$
69.0447	234.71	$C_3H_4N_2 + H^+$
71.0604	86.32	$C_3H_6N_2 + H^+$
82.0521	91.21	$C_4H_5N_2 + H^+$
83.0241	69.26	$C_3H_2N_2O + H^+$
83.0477	151.90	
83.0603	812.28	$C_4H_6N_2 + H^+$
84.0317	118.58	$C_3H_3N_2O + H^+$

Continued on next page

Table SI-D71: Annotated MS2 spectrum of 1,3,7-trimethyluric.(Continued)

85.0396	801.76	$C_3H_4N_2O + H^+$
89.1976	60.96	
91.0540	93.38	
97.0751	53.46	$C_5H_8N_2 + H^+$
98.0475	128.67	$C_4H_5N_2O + H^+$
99.0552	232.52	$C_4H_6N_2O + H^+$
110.0346	210.52	$C_4H_3N_3O + H^+$
111.0428	275.92	$C_4H_4N_3O + H^+$
124.0501	130.29	$C_5H_5N_3O + H^+$
125.0583	170.78	$C_5H_6N_3O + H^+$
126.0662	826.92	$C_5H_7N_3O + H^+$
129.2564	54.62	
137.0452	139.01	$C_5H_4N_4O + H^+$
138.0298	266.26	$C_5H_3N_3O_2 + H^+$
139.0377	999.00	$C_5H_4N_3O_2 + H^+$
142.0513	70.73	
151.0613	158.83	$C_6H_6N_4O + H^+$
152.0694	198.94	$C_6H_7N_4O + H^+$
153.0417	68.33	
153.0537	177.04	$C_6H_6N_3O_2 + H^+$
154.0607	201.57	$C_6H_7N_3O_2 + H^+$
157.0489	65.57	
196.0590	669.89	$C_6H_7N_3O_2 + H^+$

A reference standard of 1,3,7-trimethyluric acid was purchased. Figure SI-D149 shows the extracted ion chromatograms of this standard, the sample and the spiked sample, as well as a head to tail plot of the MS2 spectra of the standard and the sample. In addition, the most intense MS2 fragments in the sample and in the standard are displayed. It becomes visible that the retention times of the sample and the spiked sample are identical and the spectra similarity score between sample and standard is equal to 0.991. The vast majority of the MS2 fragments in the sample can be explained by the reference standard. It can therefore be concluded that the suspected compound is indeed 1,3,7-trimethyluric acid. Correspondingly, the identification confidence can be increased to level 1.

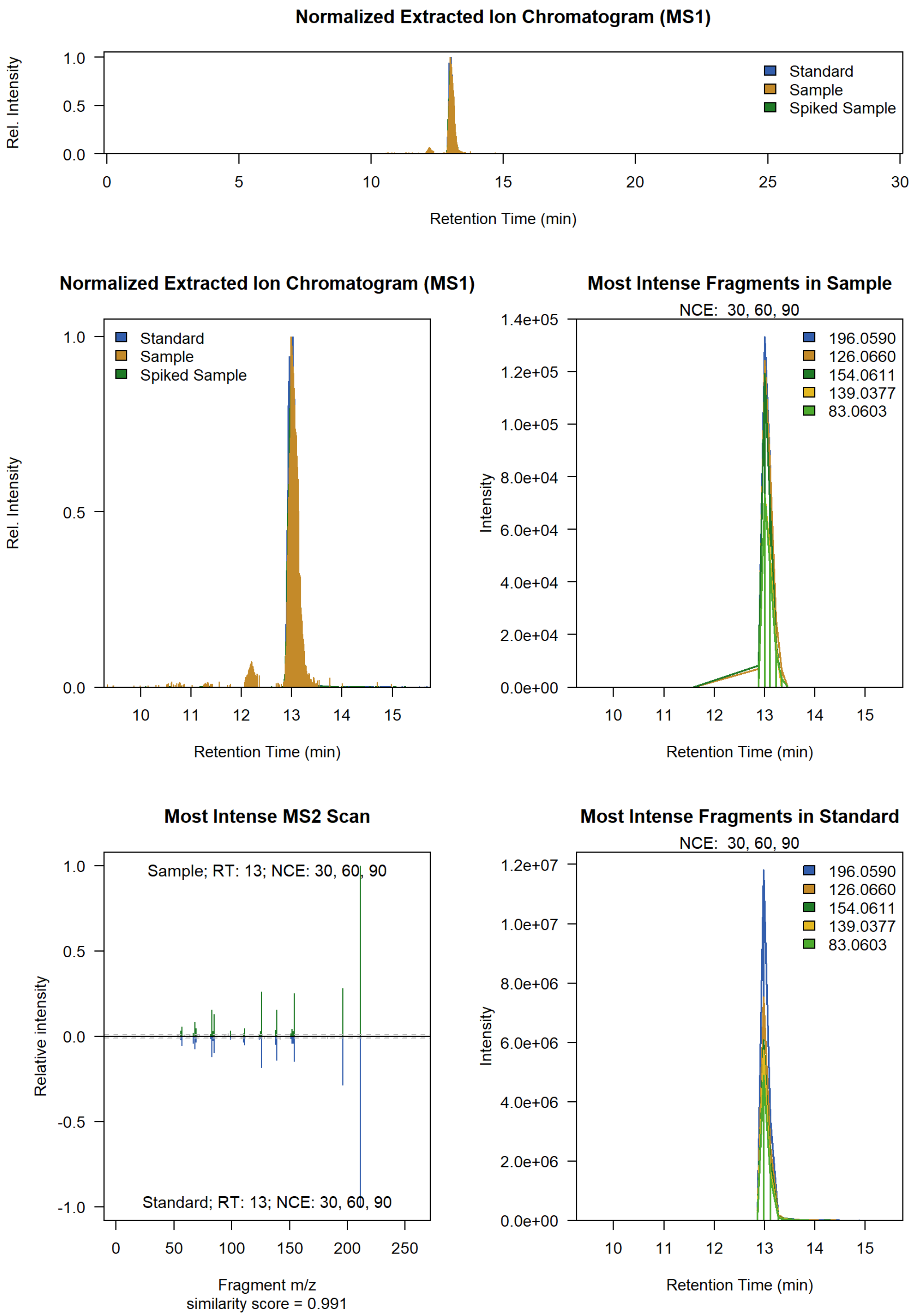

Figure SI-D149: Extracted ion chromatograms of 1,3,7-trimethyluric acid in the reference standard, the sample and the spiked sample, as well as MS2 head to tail plot and most intense MS2 fragments in standard and sample.

SI-D2.7 Hesperidin Metabolites

Hesperidin is a natural flavonoid found in a variety of dietary supplements. It is thought to have beneficial effects against blood vessel disorders and various other conditions.[2] It is the main flavonoid in orange and lemon peel.[10] The identified metabolites hesperitin and eriodictyol are highlighted in the metabolism scheme of hesperidin SI-D150. An excerpt of the molecular network in the negative ionization mode is shown in Figure SI-D151. The following subsections give more details on the individual metabolites.

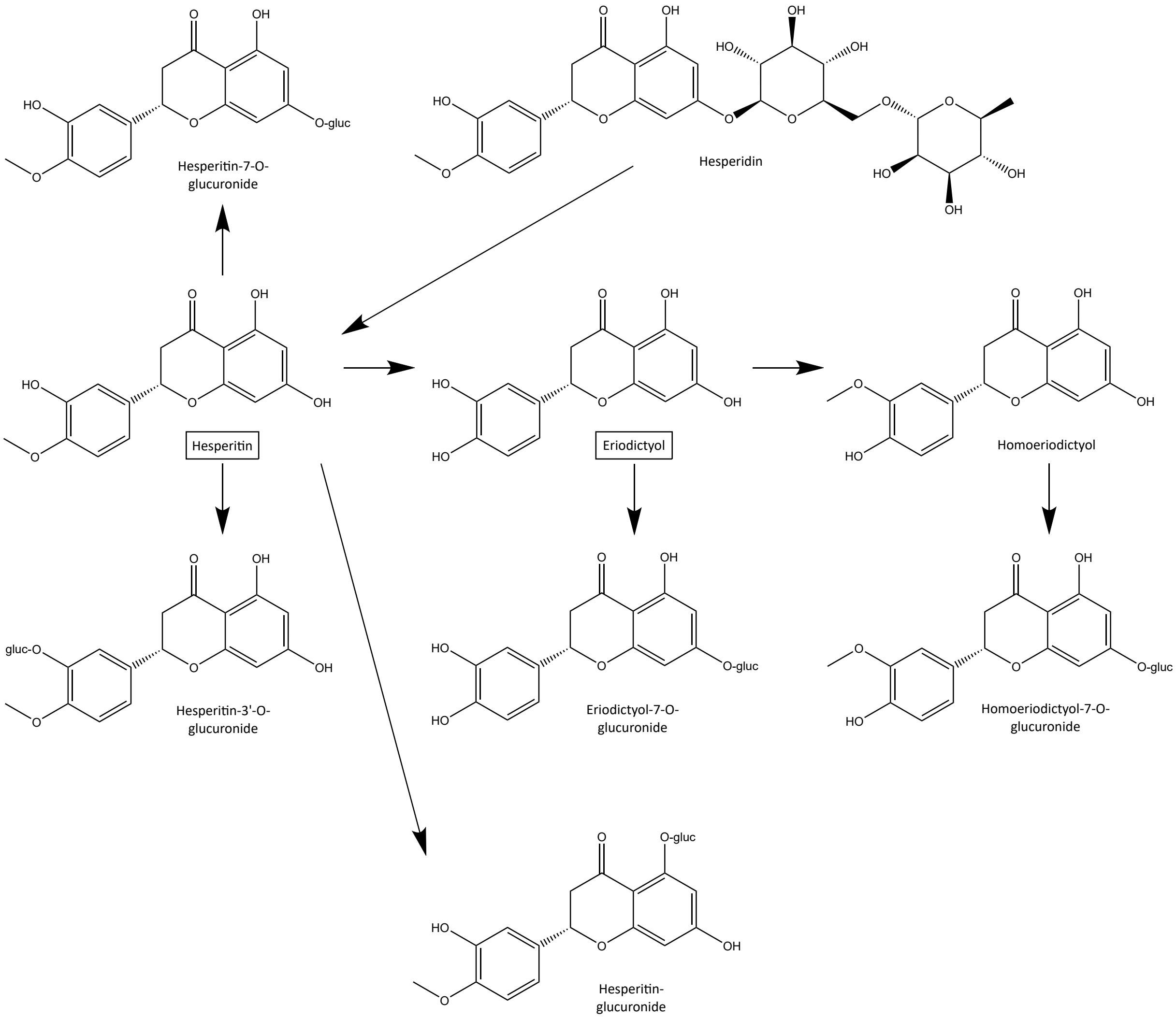

Figure SI-D150: Human metabolism of hesperidin. Framed metabolites were identified during suspect screening. Scheme adapted from.[11]

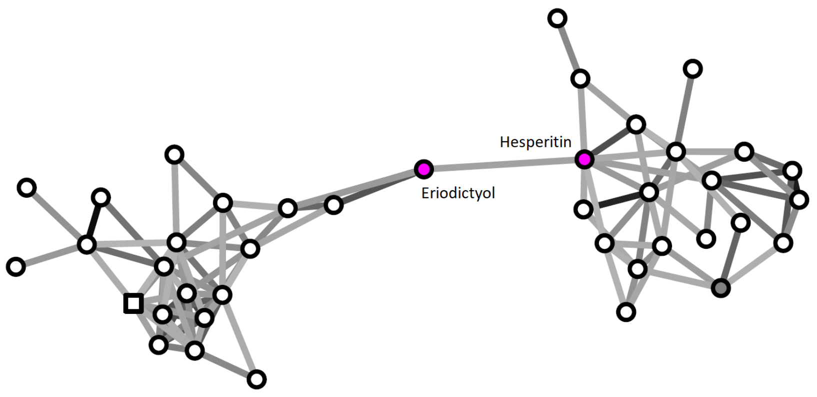

Figure SI-D151: Excerpt of the molecular network showing the hesperidin cluster.

SI-D2.7.1 Eriodictyol

Table SI-D72: Information on identifiers, chemical properties, detection and confidence of identification of eriodictyol.

IUPAC Name	(2*S*)-2-(3,4-Dihydroxyphenyl)-5,7-dihydroxy-2,3-dihydrochromen-4-one
Molecular formula	$C_{15}H_{12}O_6$
Monoisotopic mass [g/mol]	288.0634
Adduct	$[M\text{-}H]^-$
Retention time [min]	18.5
SMILES	C1[C@H](OC2=CC(=CC(=C2C1=O)O)O)C3=CC(=C(C=C3)O)O
InChI	InChI=1S/C15H12O6/c16-8-4-11(19)15-12(20)6-13(21-14(15)5-8)7-1-2-9(17)10(18)3-7/h1-5,13,16-19H,6H2/t13-/m0/s1
InChI-Key	SBHXYTNGIZCORC-ZDUSSCGKSA-N
CAS RN	552-58-9
Metabolite of	Hesperidin
Detection frequency	100% (15/15 samples)
Detected in	Altenrhein, Monday-Friday<br>Neugut, Monday-Friday<br>Werdhölzli, Monday-Friday
Intensity	E7
Initial confidence level	level 2a
Initial confidence score	0.58
Final confidence level	level 1

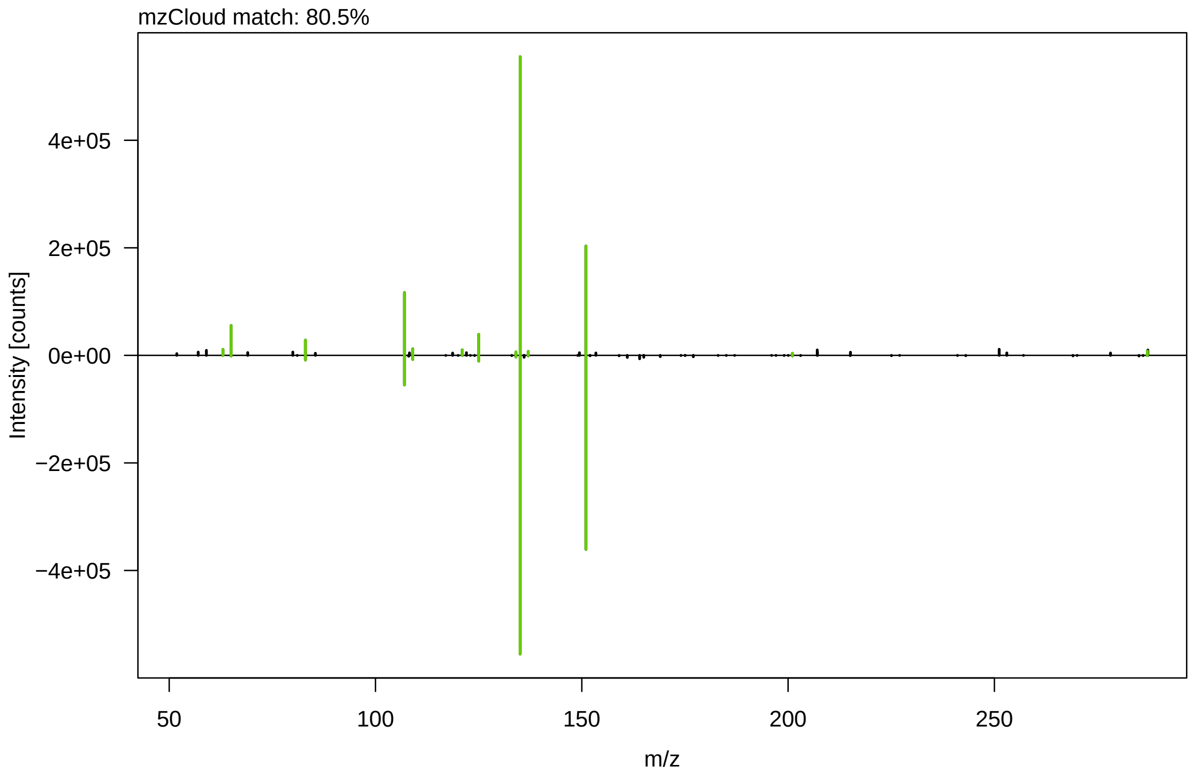

Figure SI-D152: Head to tail plot of measured MS2 spectrum against mzCloud library spectrum of eriodictyol. Matching fragments are highlighted in green.

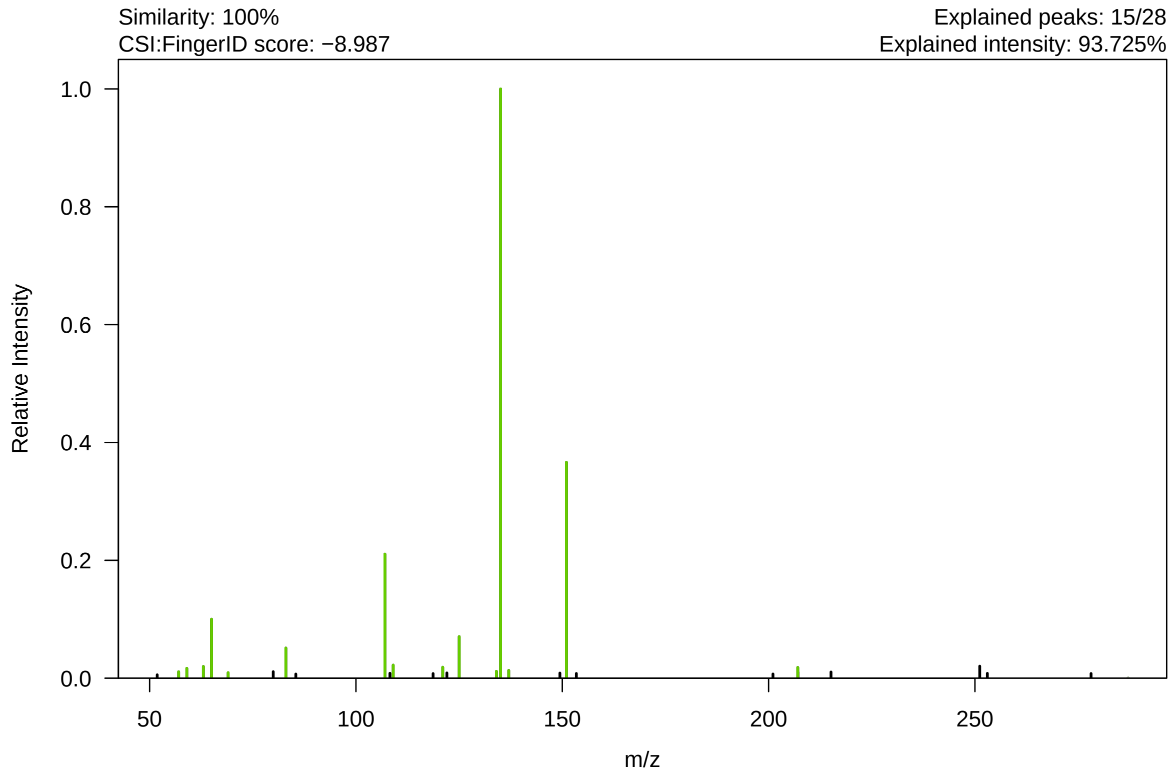

Figure SI-D153: Measured MS2 spectrum. Matching fragments with eriodictyol predicted by SIRIUS/CSI:FingerID are highlighted in green.

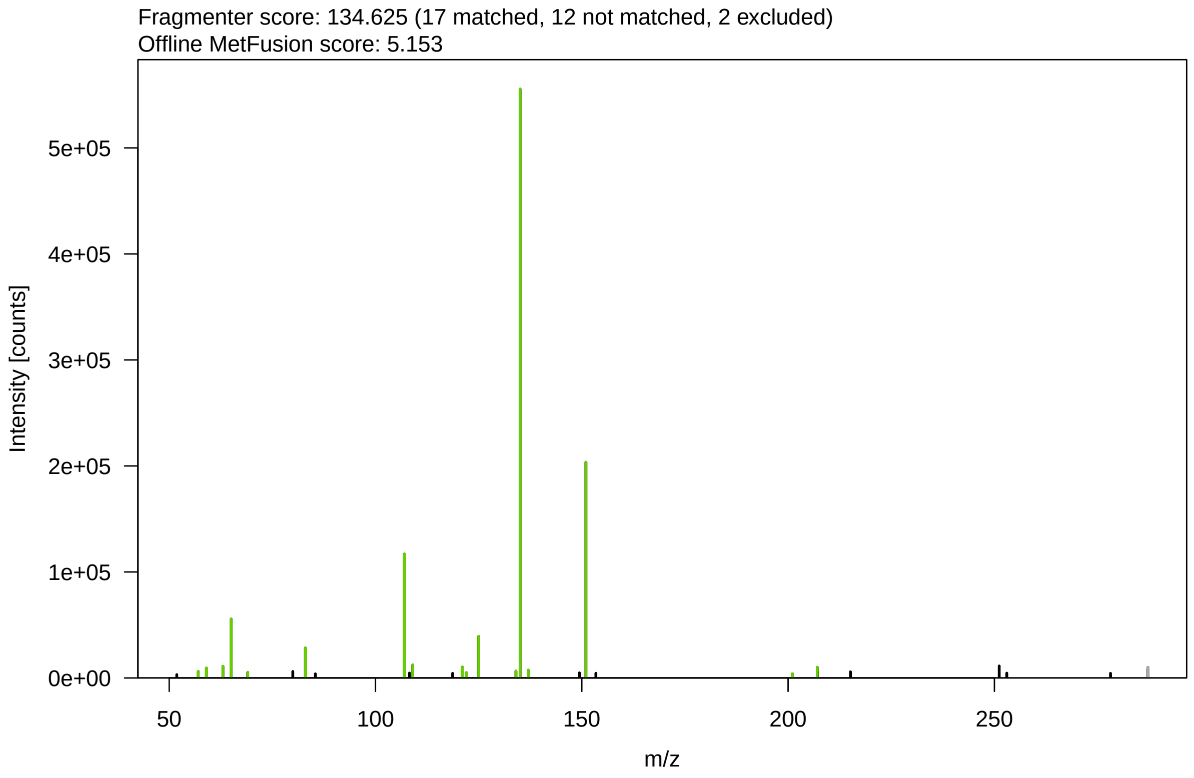

Figure SI-D154: Measured MS2 spectrum. Matching fragments with eriodictyol predicted by MetFrag are highlighted in green. The molecular ion in gray is not considered.

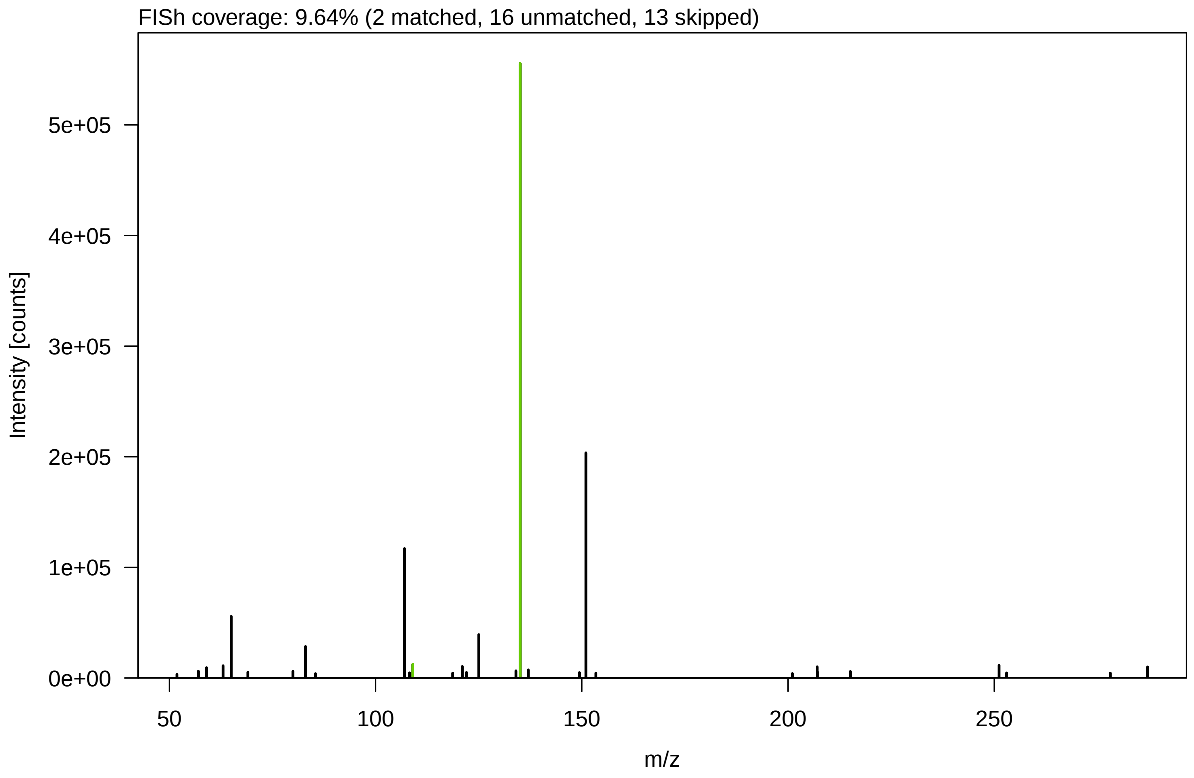

Figure SI-D155: Measured MS2 spectrum. Matching fragments with eriodictyol predicted by FISh Scoring are highlighted in green. Low intensity fragments are not considered and skipped.

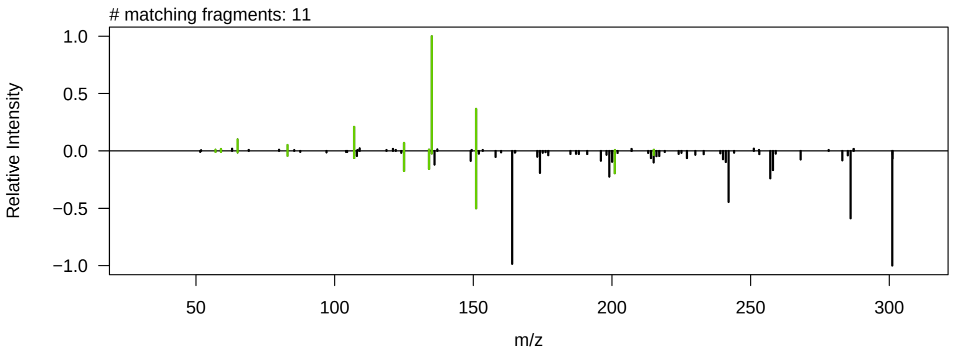

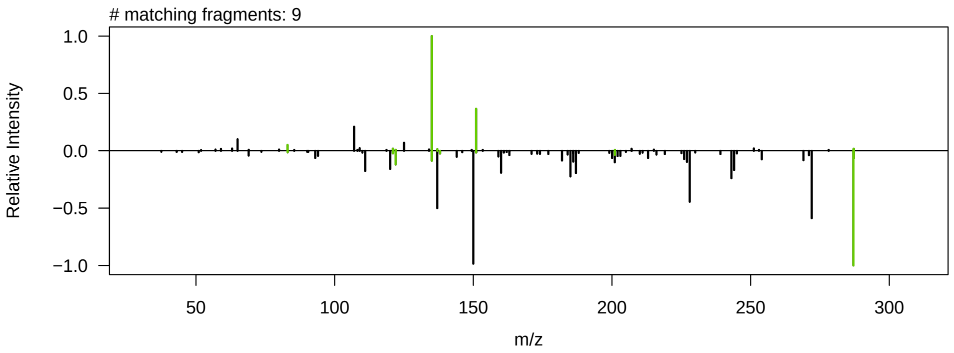

Figure SI-D156: Head to tail plots of eriodictyol and hesperitin. In the bottom plot, the mass spectrum of hesperitin is shifted by the mass difference. Matching fragments are highlighted in green.

Table SI-D73: Retention time prediction of eriodictyol.

Comparison with	Hesperitin
MSn Score	37
Forward coverage	51
Reverse coverage	24
Forward match	42
Reverse match	37
ΔMass [g/mol]	14.0157
Measured retention time [min]	17
Predicted $\log D_{OW}$ (pH = 4.8)	2.53
Predicted retention time [min]	18.9
Predicted retention time range (95% confidence interval) [min]	11.8-26.1
Predicted retention time range (99% confidence interval) [min]	9.5-28.4

Table SI-D74: Annotated MS2 spectrum of eriodictyol.

m/z	Relative Intensity	Annotation
51.8379	5.72	
57.0337	10.91	$C_3H_6O - H^-$
59.0129	16.74	$C_2H_4O_2 - H^-$
63.0231	19.87	$C_5H_4 - H^-$

Continued on next page

Table SI-D74: Annotated MS2 spectrum of eriodictyol.(Continued)

65.0025	100.06	$C_4H_2O - H^-$
69.0336	9.30	$C_4H_6O - H^-$
79.9570	11.04	
83.0138	51.11	$C_4H_4O_2 - H^-$
85.4290	7.05	
107.0140	210.34	$C_6H_4O_2 - H^-$
108.2296	8.36	
109.0297	22.16	$C_6H_6O_2 - H^-$
118.6953	7.82	
121.0295	18.63	$C_7H_6O_2 - H^-$
122.0381	8.94	$C_7H_7O_2 - H^-$
125.0246	70.45	$C_6H_6O_3 - H^-$
134.0375	11.62	$C_8H_7O_2 - H^-$
135.0453	999.00	$C_8H_8O_2 - H^-$
137.0251	13.20	$C_7H_6O_3 - H^-$
149.4222	8.72	
151.0036	366.01	$C_7H_4O_4 - H^-$
153.4116	7.98	
201.0577	7.14	$C_{12}H_{10}O_3 - H^-$
207.0665	18.19	$C_{11}H_{12}O_4 - H^-$
207.1029	9.94	
215.1196	10.50	
251.1647	20.43	
253.0075	8.19	
278.1428	7.90	
287.0957	14.51	
287.1871	17.95	

A reference standard of eriodictyol was purchased. Figure SI-D157 shows the extracted ion chromatograms of this standard, the sample and the spiked sample, as well as a head to tail plot of the MS2 spectra of the standard and the sample. In addition, the most intense MS2 fragments in the sample and in the standard are displayed. It becomes visible that the retention times of the sample and the spiked sample are identical and the spectra similarity score between sample and standard is equal to 0.935. The majority of the MS2 fragments in the sample can be explained by the reference standard. It can therefore be concluded that the suspected compound is indeed eriodictyol. Correspondingly, the identification confidence can be increased to level 1.

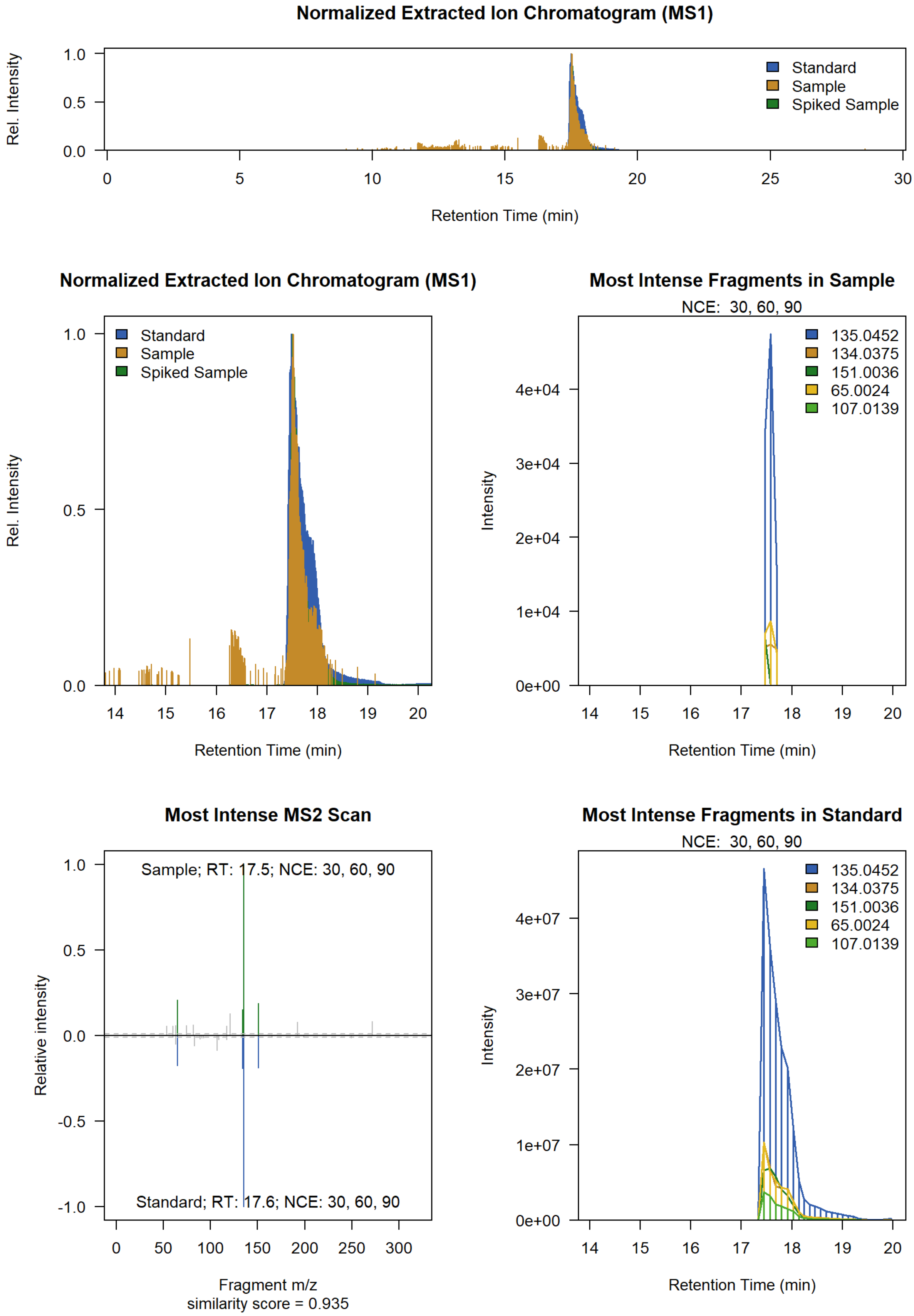

Figure SI-D157: Extracted ion chromatograms of eriodictyol in the reference standard, the sample and the spiked sample, as well as MS2 head to tail plot and most intense MS2 fragments in standard and sample.

SI-D2.7.2 Hesperitin

Table SI-D75: Information on identifiers, chemical properties, detection and confidence of identification of hesperitin.

IUPAC Name	5,7-dihydroxy-2-(3-hydroxy-4-methoxyphenyl)-2,3-dihydrochromen-4-one
Molecular formula	$C_{16}H_{14}O_6$
Monoisotopic mass [g/mol]	302.0790
Adduct	$[M+H]^+$
Retention time [min]	18.4
SMILES	COC1=C(C=C(C=C1)C2CC(=O)C3=C(C=C(C=C3O2)O)O)O
InChI	InChI=1S/C16H14O6/c1-21-13-3-2-8(4-10(13)18)14-7-12(20)16-11(19)5-9(17)6-15(16)22-14/h2-6,14,17-19H,7H2,1H3
InChI-Key	AIONOLUJZLIMTK-UHFFFAOYSA-N
CAS RN	520-33-2
Metabolite of	Hesperidin
Detection frequency	100% (15/15 samples)
Detected in	Altenrhein, Monday-Friday<br>Neugut, Monday-Friday<br>Werdhölzli, Monday-Friday
Intensity	E7-E8
Initial confidence level	level 2a
Initial confidence score	0.60
Final confidence level	level 1

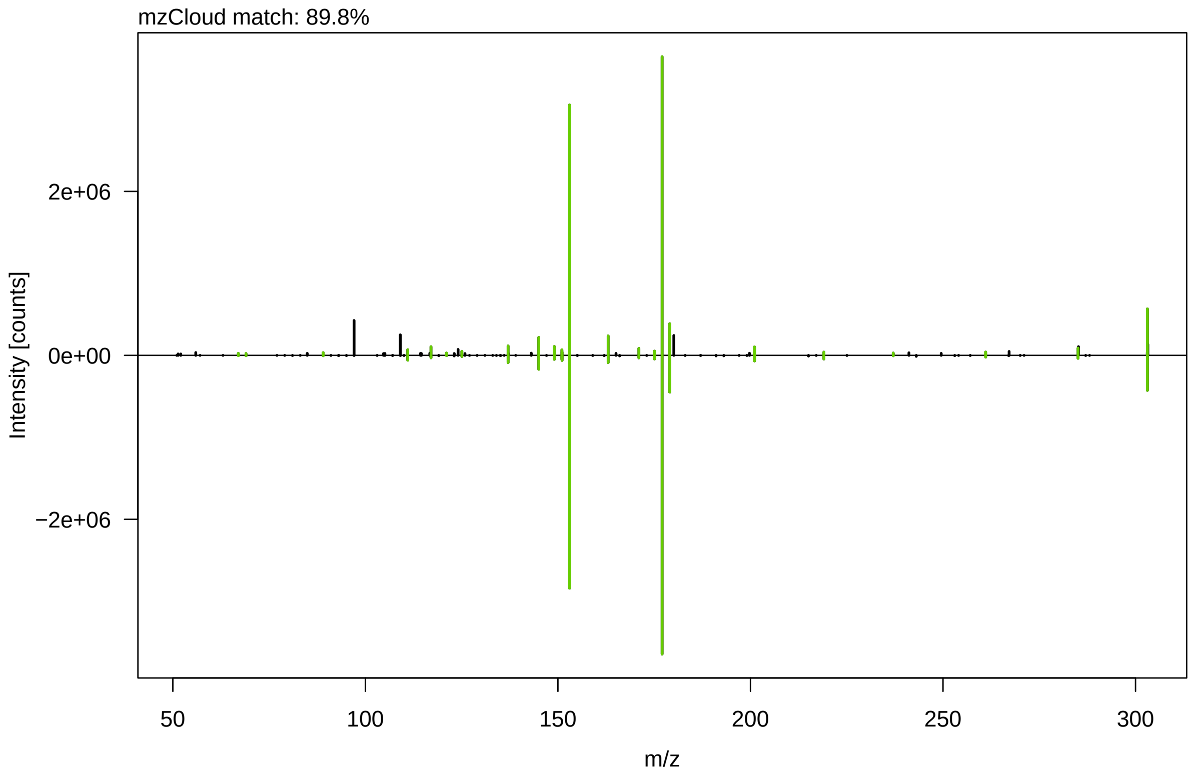

Figure SI-D158: Head to tail plot of measured MS2 spectrum against mzCloud library spectrum of hesperitin. Matching fragments are highlighted in green.

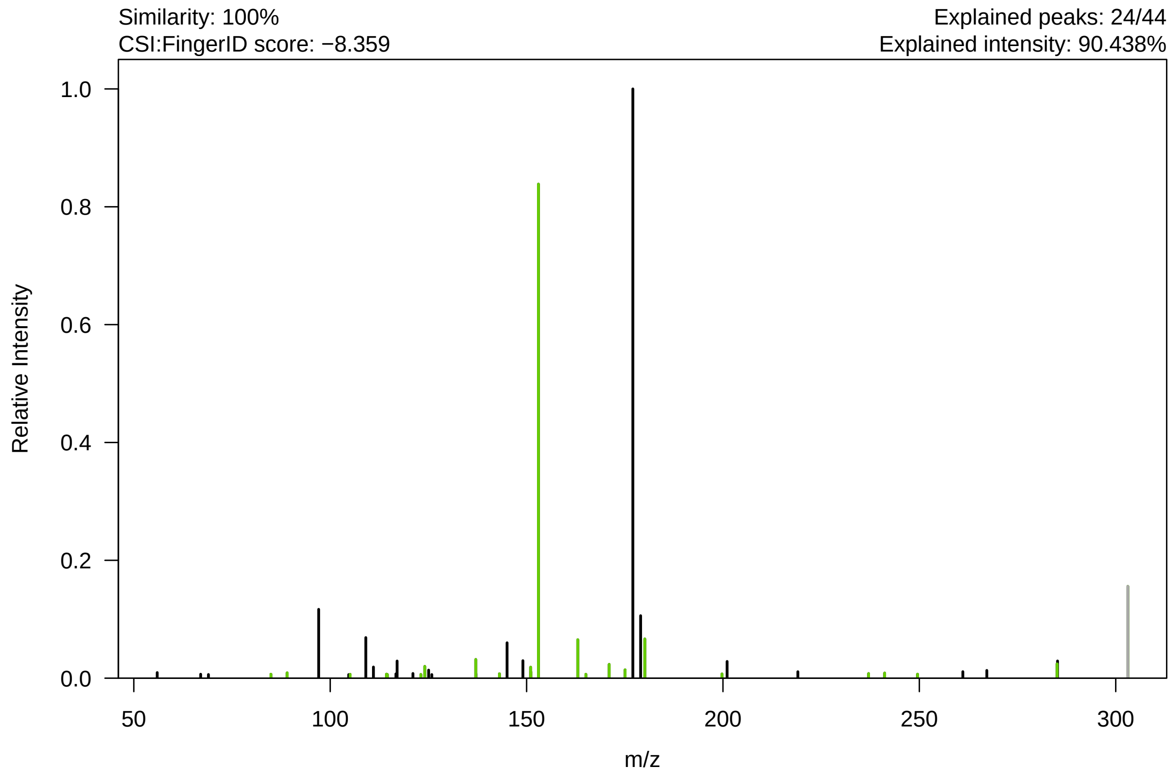

Figure SI-D159: Measured MS2 spectrum. Matching fragments with hesperitin predicted by SIRIUS/CSI:FingerID are highlighted in green. The molecular ion in gray is not considered.

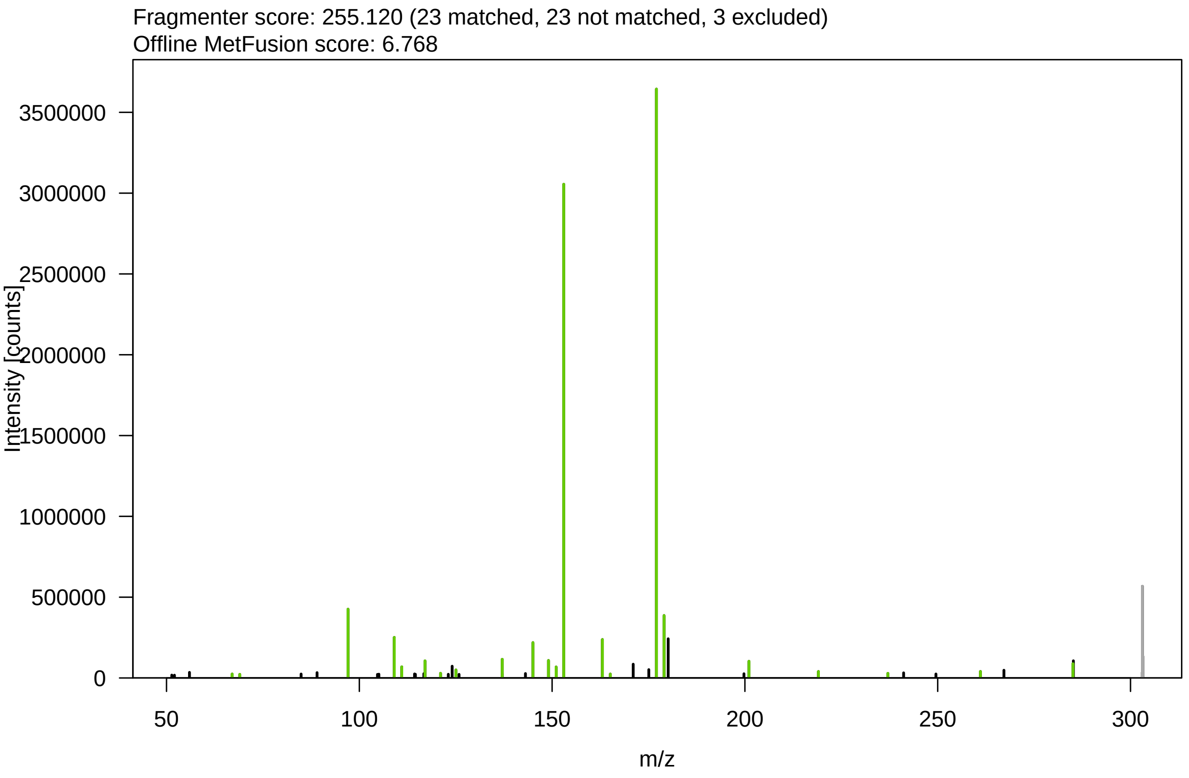

Figure SI-D160: Measured MS2 spectrum. Matching fragments with hesperitin predicted by MetFrag are highlighted in green. The molecular ion in gray is not considered.

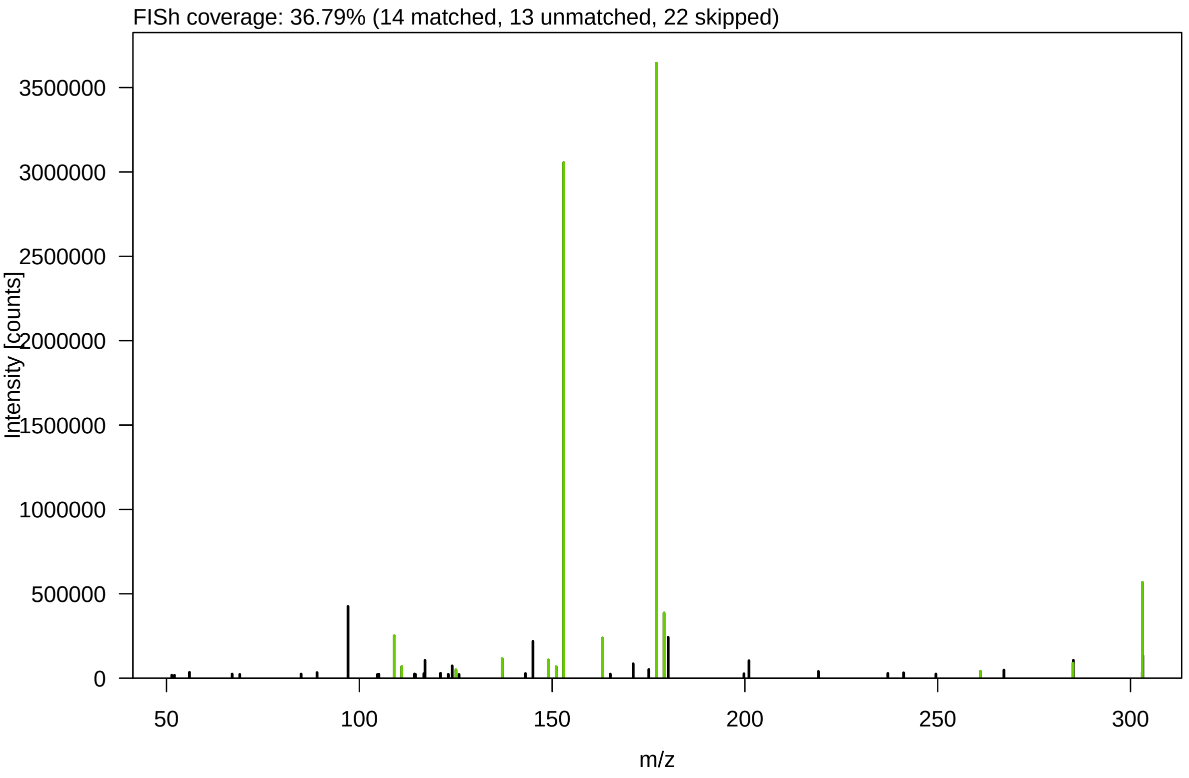

Figure SI-D161: Measured MS2 spectrum. Matching fragments with hesperitin predicted by FISh Scoring are highlighted in green. Low intensity fragments are not considered and skipped.

Table SI-D76: Retention time prediction of hesperitin.

Measured retention time [min]	18.4
Predicted $logD_{OW}$ (pH = 2.7)	2.68
Predicted retention time [min]	18.2
Predicted retention time range (95% confidence interval) [min]	13.6-22.8
Predicted retention time range (99% confidence interval) [min]	12.2-24.3

Table SI-D77: Annotated MS2 spectrum of hesperitin.

m/z	Relative Intensity	Annotation
51.3647	4.87	
52.0552	4.73	
55.9533	9.48	
67.0181	6.70	$C_4H_2O + H^+$
68.9970	6.19	$C_3O_2 + H^+$
84.9018	6.67	
89.0385	9.07	$C_7H_4 + H^+$
97.0649	116.67	$C_6H_8O + H^+$
104.7080	6.07	
105.0703	6.44	$C_8H_8 + H^+$
109.0648	68.80	$C_7H_8O + H^+$
111.0075	18.89	$C_5H_2O_3 + H^+$
114.3239	6.82	
114.5458	6.24	
116.7324	7.32	
117.0331	29.07	$C_8H_4O + H^+$
121.0647	7.94	$C_8H_8O + H^+$
123.0802	6.24	$C_8H_{10}O + H^+$
124.0754	19.95	
125.0591	13.67	$C_7H_8O_2 + H^+$
125.8633	6.11	
137.0596	31.66	$C_8H_8O_2 + H^+$
137.0952	7.26	
143.0853	7.51	
145.0284	59.99	$C_9H_4O_2 + H^+$
149.0596	29.63	$C_9H_8O_2 + H^+$
151.0391	18.66	$C_8H_6O_3 + H^+$
151.0750	10.78	$C_9H_{10}O_2 + H^+$
153.0181	837.58	$C_7H_4O_4 + H^+$

Continued on next page

Table SI-D77: Annotated MS2 spectrum of hesperitin.(Continued)

163.0387	65.20	$C_9H_6O_3 + H^+$
165.0919	6.66	$C_{10}H_{12}O_2 + H^+$
171.0287	23.38	
175.0755	14.16	
177.0544	999.00	$C_{10}H_8O_3 + H^+$
179.0334	105.90	$C_9H_6O_4 + H^+$
180.1017	66.48	
199.7489	7.15	
201.0546	28.36	$C_{12}H_8O_3 + H^+$
219.0649	10.91	$C_{12}H_{10}O_4 + H^+$
237.0771	7.78	
241.1585	8.68	
249.5402	6.75	
261.0759	11.03	$C_{14}H_{12}O_5 + H^+$
267.1750	13.12	
285.0755	24.30	$C_{16}H_{12}O_5 + H^+$
285.1851	29.13	
303.0519	9.39	
303.0862	155.55	$C_{16}H_{14}O_6 + H^+$
303.1955	36.68	

A reference standard of hesperitin was purchased. Figure SI-D162 shows the extracted ion chromatograms of this standard, the sample and the spiked sample, as well as a head to tail plot of the MS2 spectra of the standard and the sample. In addition, the most intense MS2 fragments in the sample and in the standard are displayed. It becomes visible that the retention times of the sample and the spiked sample are identical and the spectra similarity score between sample and standard is equal to 0.776. The majority of the MS2 fragments in the sample can be explained by the reference standard. It can therefore be concluded that the suspected compound is indeed hesperitin. Correspondingly, the identification confidence can be increased to level 1.

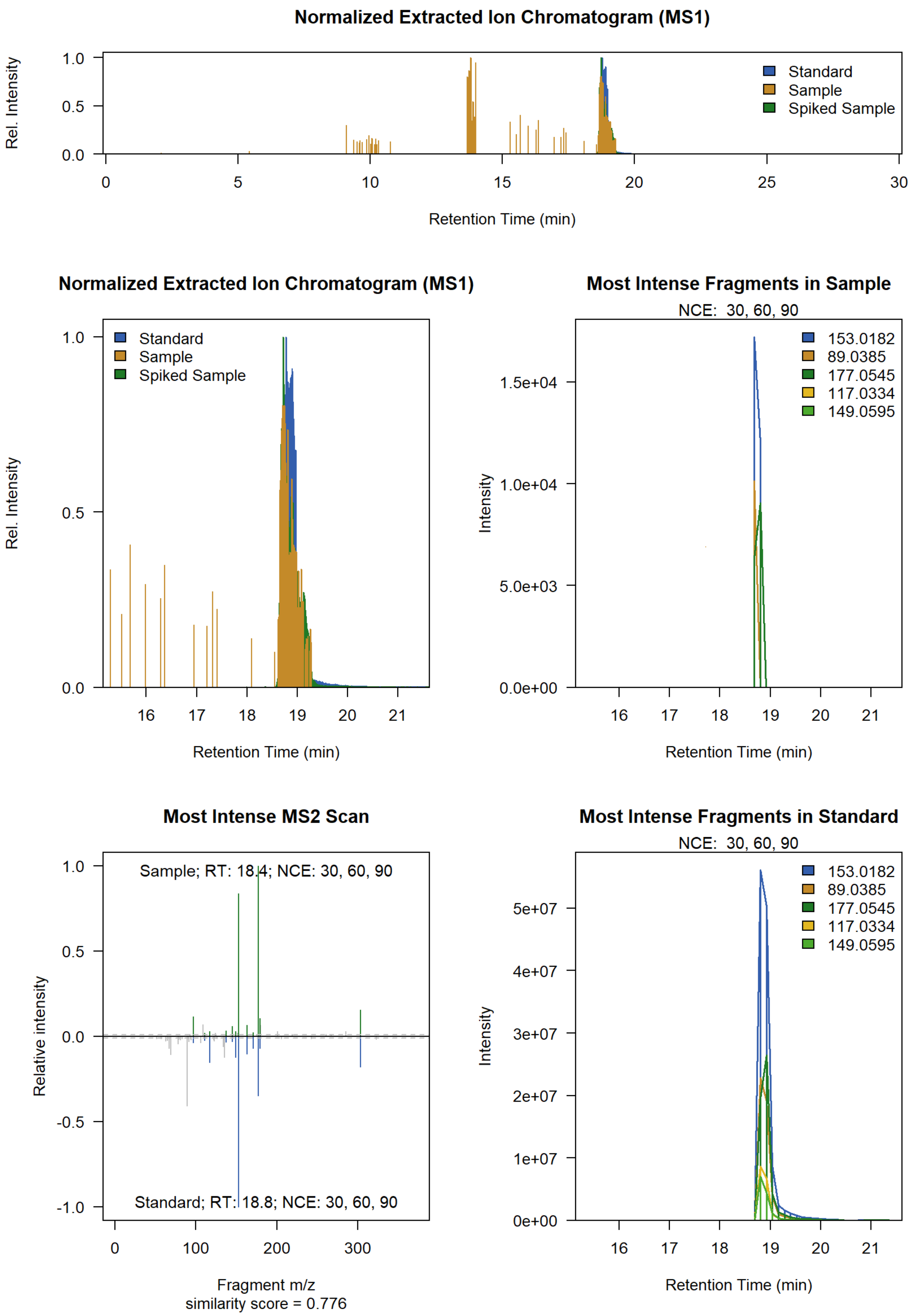

Figure SI-D162: Extracted ion chromatograms of hesperitin in the reference standard, the sample and the spiked sample, as well as MS2 head to tail plot and most intense MS2 fragments in standard and sample.

SI-D2.8 Irbesartan Metabolites

Irbesartan is an angiotesin II receptor blocker.[12] It is used to treat hypertension and congestive heart failure, as well as to delay progression of diabetic nephropathy.[2] With the aid of suspect screening combined with molecular networking, six human metabolites could be identified. The identified metabolites are highlighted in the metabolism scheme of irbesartan in Figure SI-D163. Ecerpts of the molecular networks in positive and negative ionization mode, respectively, are shown in Figures SI-D164 and SI-D165. The following subsections give more details on the individual metabolites.

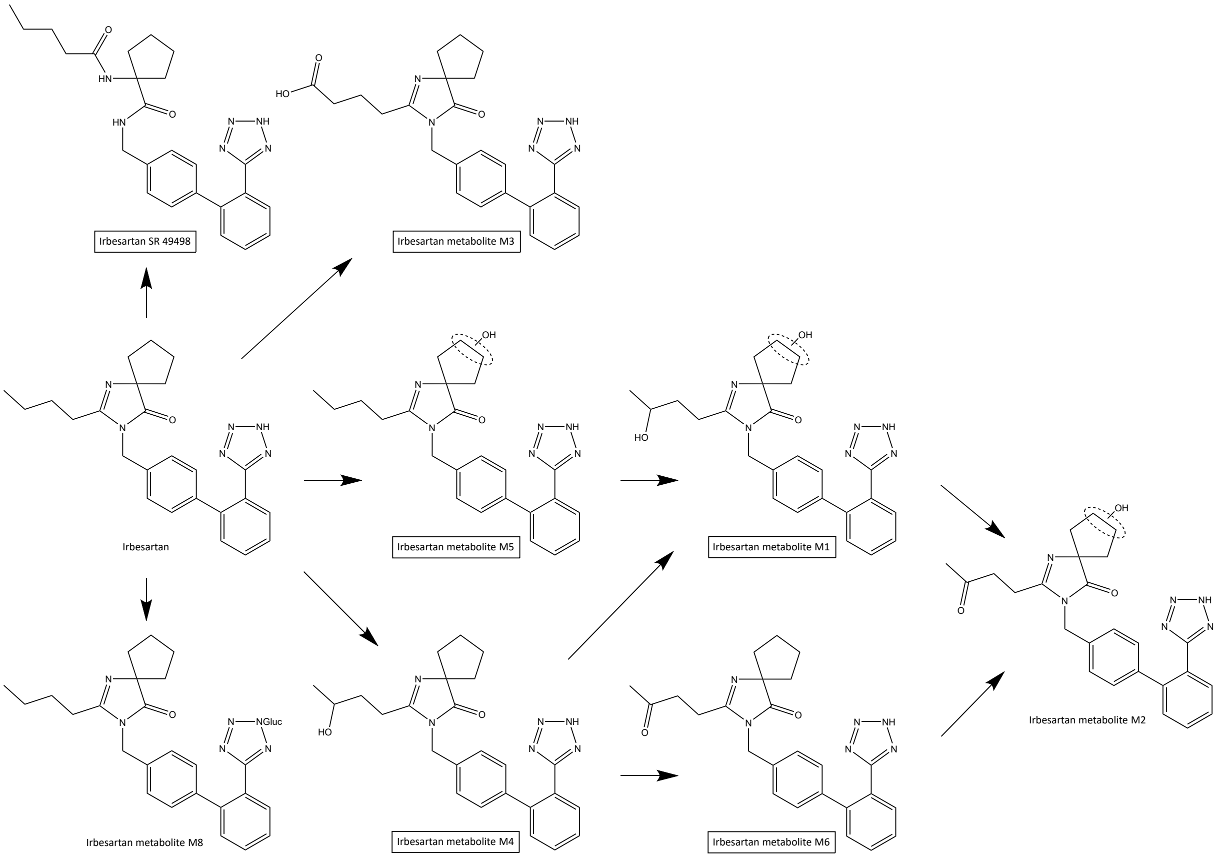

Figure SI-D163: Human metabolism of irbesartan. Framed metabolites were identified during suspect screening. Scheme adapted from.[12]

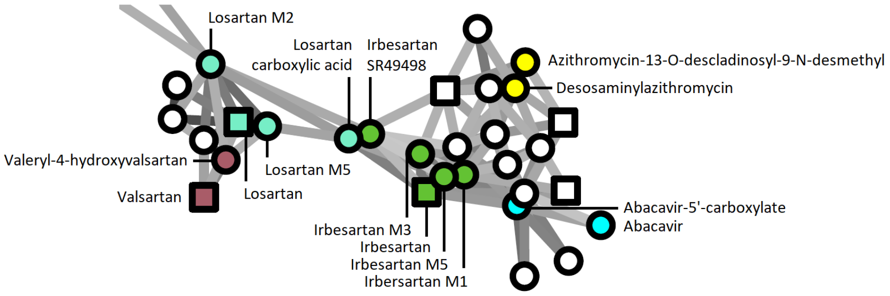

Figure SI-D164: Excerpt of the molecular network showing the irbesartan cluster in the positive ionization mode.

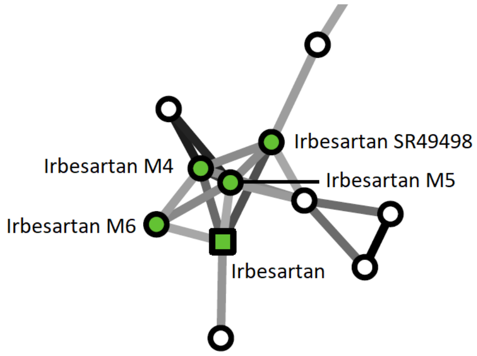

Figure SI-D165: Excerpt of the molecular network showing the irbesartan cluster in the negative ionization mode.

SI-D2.8.1 Irbesartan Metabolite M1

Table SI-D78: Information on identifiers, chemical properties, detection and confidence of identification of irbesartan metabolite M1.

IUPAC Name	8-hydroxy-2-(3-hydroxybutyl)-3-[[4-[2-(2*H*-tetrazol-5-yl)phenyl]phenyl]methyl]-1,3-diazaspiro[4.4]non-1-en-4-one
Molecular formula	$C_{25}H_{28}N_6O_3$
Monoisotopic mass [g/mol]	460.2223
Adduct	$[M+H]^+$
Retention time [min]	16.2
SMILES	CC(CCC1=NC2(CCC(C2)O)C(=O)N1CC3=CC=C(C=C3)C4=CC=CC=C4C5=NNN=N5)O
InChI	InChI=1S/C25H28N6O3/c1-16(32)6-11-22-26-25(13-12-19(33)14-25)24(34)31(22)15-17-7-9-18(10-8-17)20-4-2-3-5-21(20)23-27-29-30-28-23/h2-5,7-10,16,19,32-33H,6,11-15H2,1H3,(H,27,28,29,30)
InChI-Key	LIDZWACDVHJFLD-UHFFFAOYSA-N
CAS RN	208988-50-5
Metabolite of	Irbesartan
Detection frequency	100% (15/15 samples)
Detected in	Altenrhein, Monday-Friday<br>Neugut, Monday-Friday<br>Werdhölzli, Monday-Friday
Intensity	E7
Initial confidence level	level 3
Initial confidence score	0.40
Final confidence level	level 2b

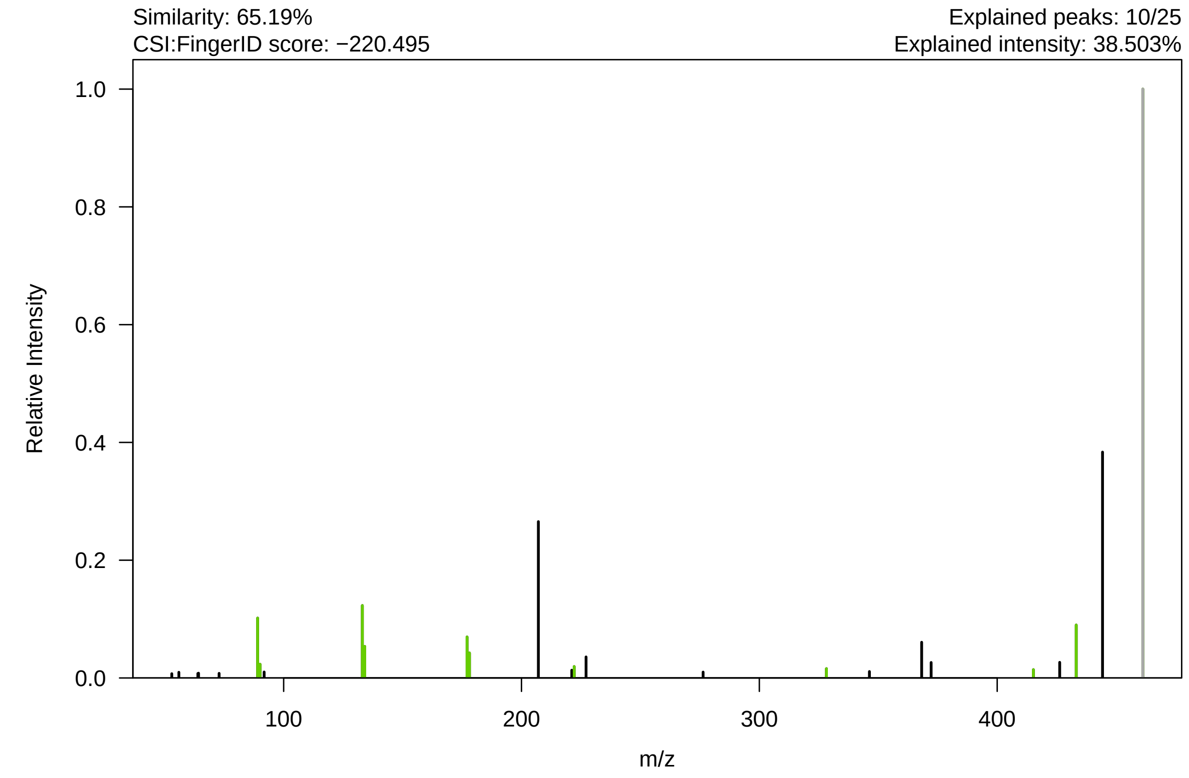

Figure SI-D166: Measured MS2 spectrum. Matching fragments with irbesartan metabolite M1 predicted by SIRIUS/CSI:FingerID are highlighted in green. The molecular ion in gray is not considered.

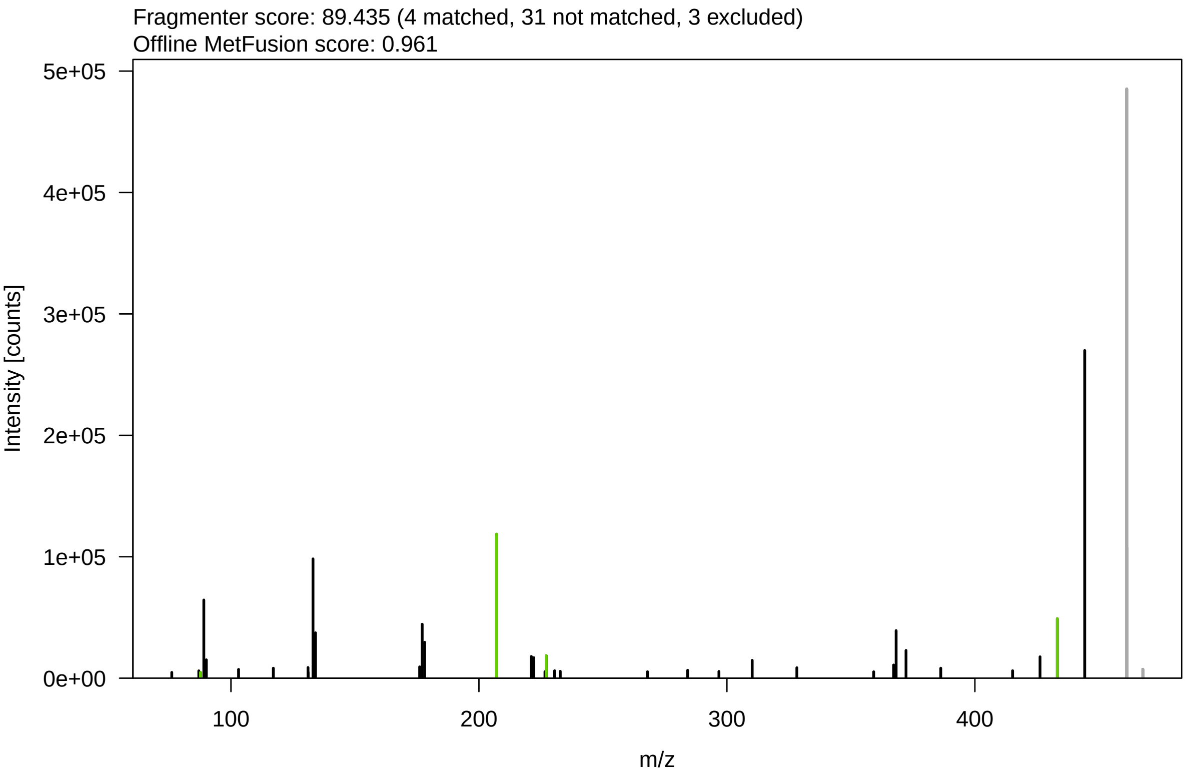

Figure SI-D167: Measured MS2 spectrum. Matching fragments with irbesartan metabolite M1 predicted by MetFrag are highlighted in green. The molecular ion in gray is not considered.

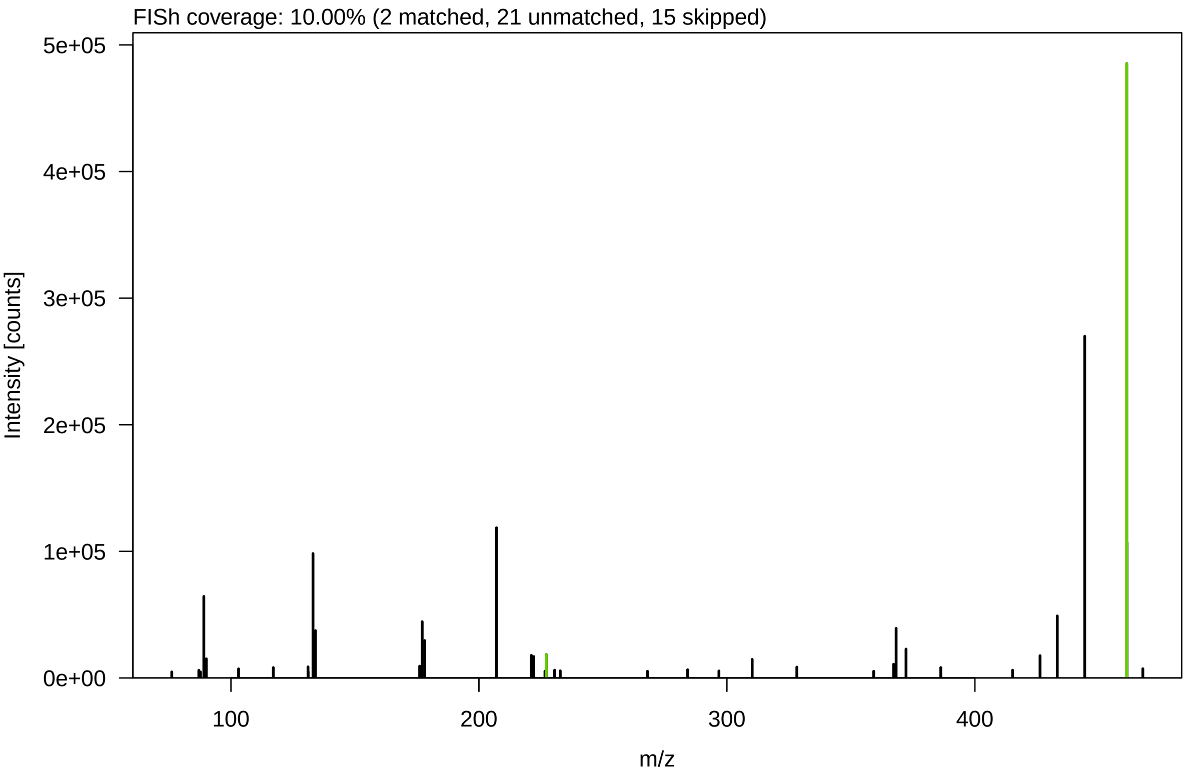

Figure SI-D168: Measured MS2 spectrum. Matching fragments with irbesartan metabolite M1 predicted by FISh Scoring are highlighted in green. Low intensity fragments are not considered and skipped.

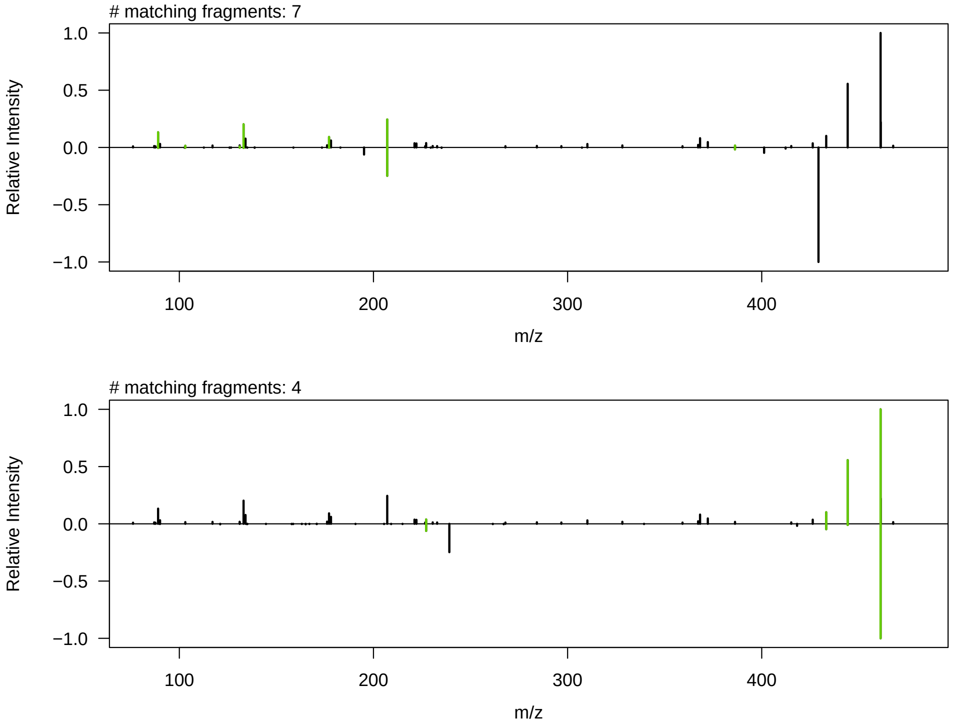

Figure SI-D169: Head to tail plots of irbesartan metabolite M1 and irbesartan. In the bottom plot, the mass spectrum of irbesartan is shifted by the mass difference. Matching fragments are highlighted in green.

Table SI-D79: Molecular network results and retention time prediction of irbesartan metabolite M1.

Comparison with	Irbesartan
MSn Score	64
Forward coverage	65
Reverse coverage	63
Forward match	33
Reverse match	25
ΔMass [g/mol]	31.9898
Measured retention time [min]	16.2
Predicted $logD_{OW}$ (pH = 2.7)	1.54
Predicted retention time [min]	16.7
Predicted retention time range (95% confidence interval) [min]	12.1-21.3
Predicted retention time range (99% confidence interval) [min]	10.7-22.8

Table SI-D80: Annotated MS2 spectrum of irbesartan metabolite M1.

m/z	Relative Intensity	Annotation
76.1380	9.82	
87.0441	12.47	$C_4H_6O_2 + H^+$
87.7146	9.87	
89.0596	132.53	$C_2H_6N_3O + H^+$
90.0629	31.20	
103.0754	14.86	$C_3H_8N_3O + H^+$
117.0908	16.89	$C_4H_{10}N_3O + H^+$
131.0697	18.10	$C_4H_8N_3O_2 + H^+$
131.1204	9.60	
133.0858	202.28	$C_4H_{10}N_3O_2 + H^+$
134.0896	76.99	$C_2H_9N_6O + H^+$
176.0996	19.22	$C_4H_{11}N_6O_2 + H^+$
177.1118	91.54	$C_6H_{14}N_3O_3 + H^+$
178.1158	60.87	$C_4H_{13}N_6O_2 + H^+$
207.0914	244.23	$C_{14}H_{10}N_2 + H^+$
221.1379	36.76	
222.1414	34.76	$C_6H_{17}N_6O_3 + H^+$
226.6237	11.07	
227.1390	38.20	$C_{11}H_{18}N_2O_3 + H^+$
230.5220	12.61	
232.7919	11.68	
267.9759	10.93	
284.1789	13.43	$C_{19}H_{23}O_2 + H^+$

Continued on next page

Table SI-D80: Annotated MS2 spectrum of irbesartan metabolite M1.(Continued)

296.7846	11.44	
310.1929	30.27	$C_{21}H_{25}O_2 + H^+$
328.2051	17.70	$C_{21}H_{27}O_3 + H^+$
359.1928	10.85	
367.2323	22.37	
368.2351	80.63	
372.2293	47.06	
386.2471	16.92	
415.2090	12.66	
426.2776	36.28	
433.2237	100.88	$C_{25}H_{28}N_4O_3 + H^+$
444.2880	555.55	
461.2283	999.00	$C_{25}H_{28}N_6O_3 + H^+$
461.3120	220.07	
467.7239	15.03	

Since no reference standard of irbesartan M1 is commercially available, an incubation experiment was conducted. The human liver S9 incubation of irbesartan led to the formation of irbesartan metabolite M1. Considering the spectral match of 0.413 (see Figure SI-D170) and the retention times of 16.2 and 16.4 minutes in the wastewater and the human liver S9 sample, respectively, further confidence could be gained that the detected feature in wastewater is irbesartan metabolite M1. Due to this diagnostic evidence, the final confidence level can be increased from level 3 to level 2b.

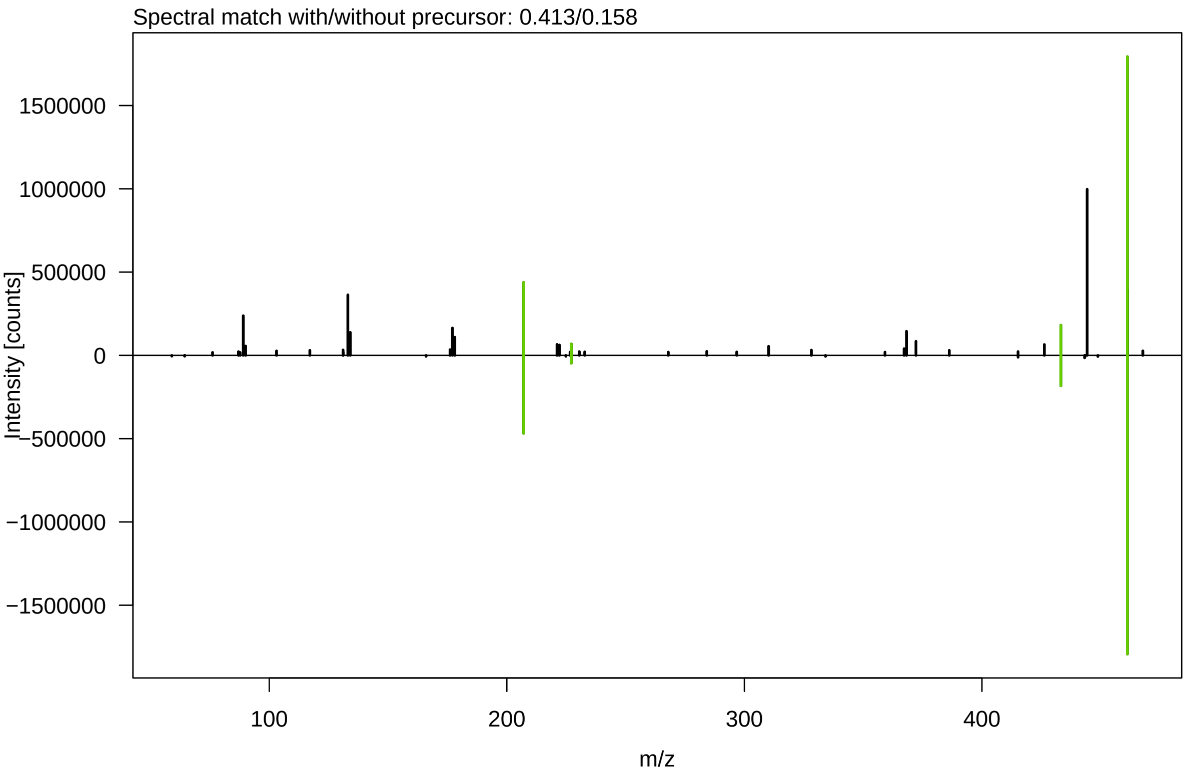

Figure SI-D170: Head to tail plot of irbesartan metabolite M1 in wastewater (top) and from human liver S9 incubation (bottom). Matching fragments are highlighted in green.

SI-D2.8.2 Irbesartan Metabolite M3

Table SI-D81: Information on identifiers, chemical properties, detection and confidence of identification of irbesartan metabolite M3.

IUPAC Name	4-[4-oxo-3-[[4-[2-(2*H*-tetrazol-5-yl)phenyl]phenyl]methyl]-1,3-diazaspiro[4.4]non-1-en-2-yl]butanoic acid
Molecular formula	$C_{25}H_{26}N_6O_3$
Monoisotopic mass [g/mol]	458.2066
Adduct	$[M+H]^+$
Retention time [min]	17.9
SMILES	C1CCC2(C1)C(=O)N(C(=N2)CCCC(=O)O)CC3=CC=C(C=C3)C4=CC=CC=C4C5=NNN=N5
InChI	InChI=1S/C25H26N6O3/c32-22(33)9-5-8-21-26-25(14-3-4-15-25)24(34)31(21)16-17-10-12-18(13-11-17)19-6-1-2-7-20(19)23-27-29-30-28-23/h1-2,6-7,10-13H,3-5,8-9,14-16H2,(H,32,33)(H,27,28,29,30)
InChI-Key	SQPSUXCOWHFKKF-UHFFFAOYSA-N
CAS RN	208923-63-1
Metabolite of	Irbesartan
Detection frequency	100% (15/15 samples)
Detected in	Altenrhein, Monday-Friday<br>Neugut, Monday-Friday<br>Werdhölzli, Monday-Friday
Intensity	E7
Initial confidence level	level 3
Initial confidence score	0.45
Final confidence level	level 2b

Based on the exact mass, two metabolites of irbesartan are possible, irbesartan metabolite M2 and irbesartan metabolite M3. With the aid of the MS2 fragments, a differentiation of the two metabolites is possible (see Figure SI-D171).

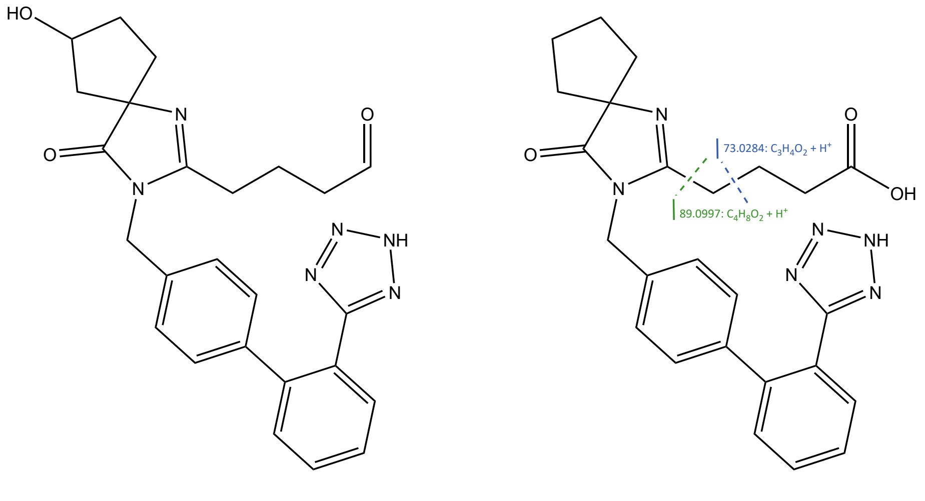

Irbesartan metabolite M2 Irbesartan metabolite M3

Figure SI-D171: Differentiation of irbesartan metabolite M2 and M3 based on two fragments. These two fragments can only be explained by the structure of irbesartan metabolite M3, but not by irbesartan metabolite M2.

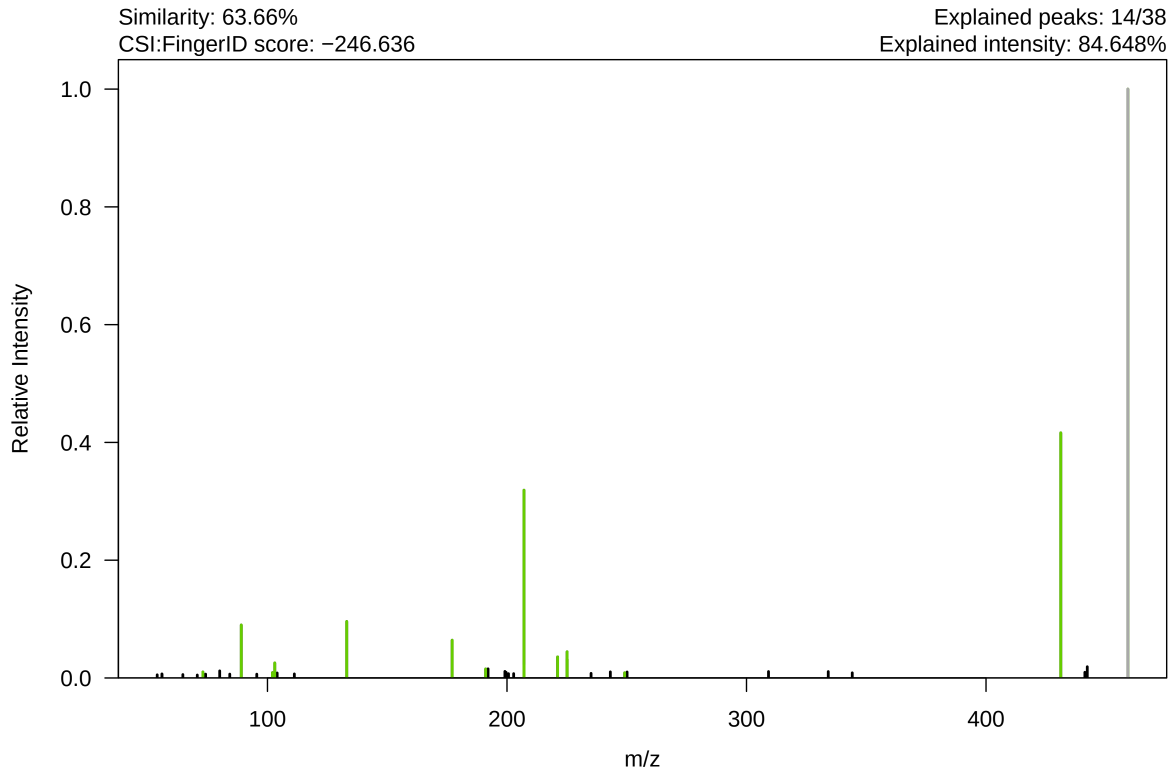

Figure SI-D172: Measured MS2 spectrum. Matching fragments with irbesartan metabolite M3 predicted by SIRIUS/CSI:FingerID are highlighted in green. The molecular ion in gray is not considered.

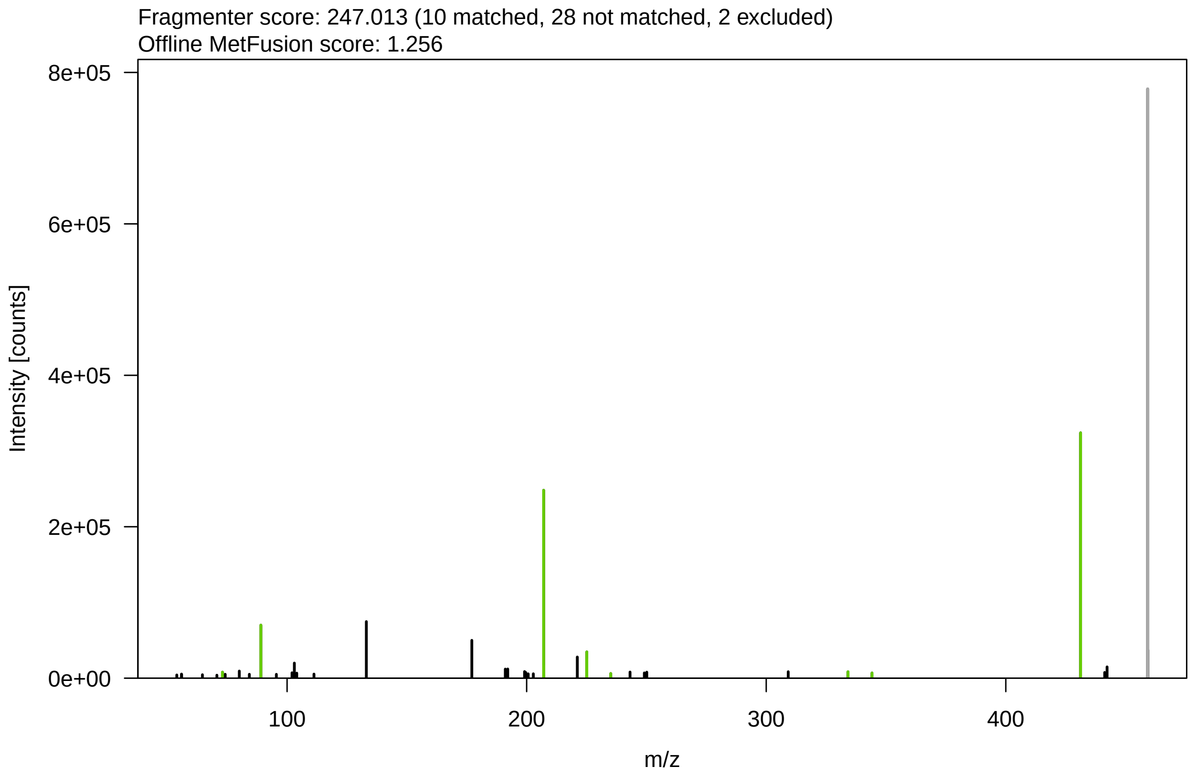

Figure SI-D173: Measured MS2 spectrum. Matching fragments with irbesartan metabolite M3 predicted by MetFrag are highlighted in green. The molecular ion in gray is not considered.

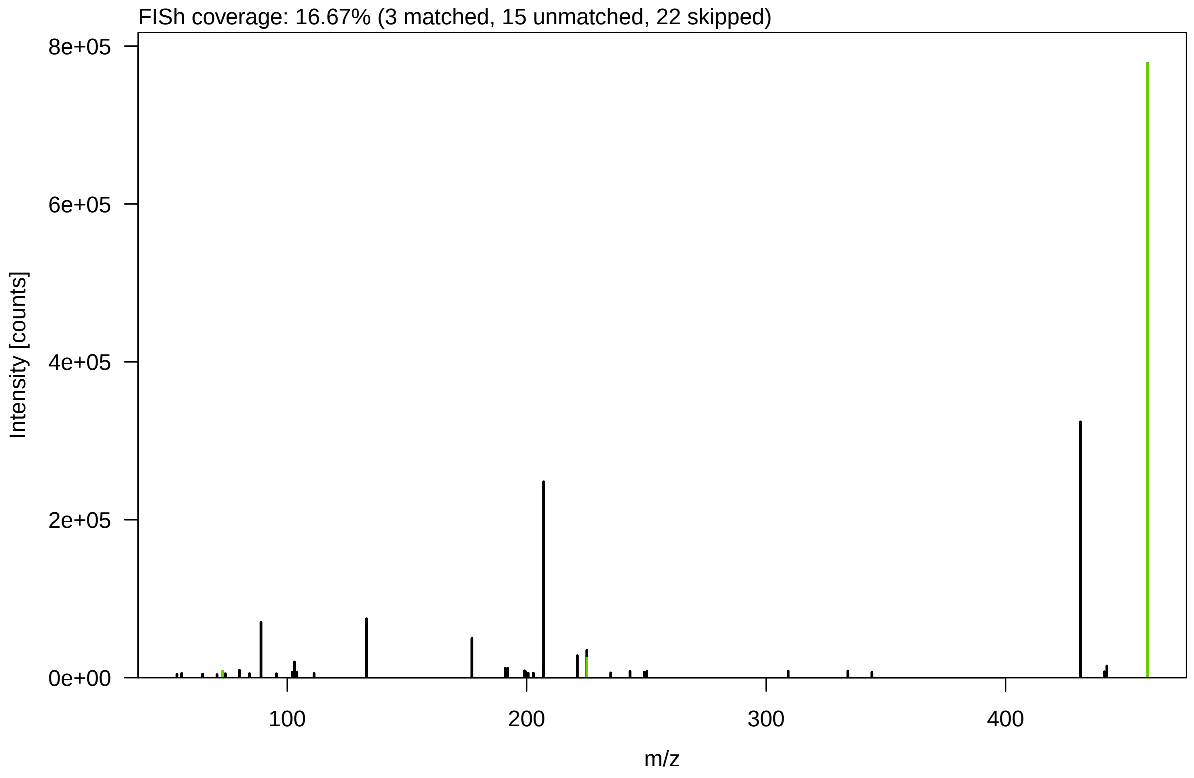

Figure SI-D174: Measured MS2 spectrum. Matching fragments with irbesartan metabolite M3 predicted by FISh Scoring are highlighted in green. Low intensity fragments are not considered and skipped.

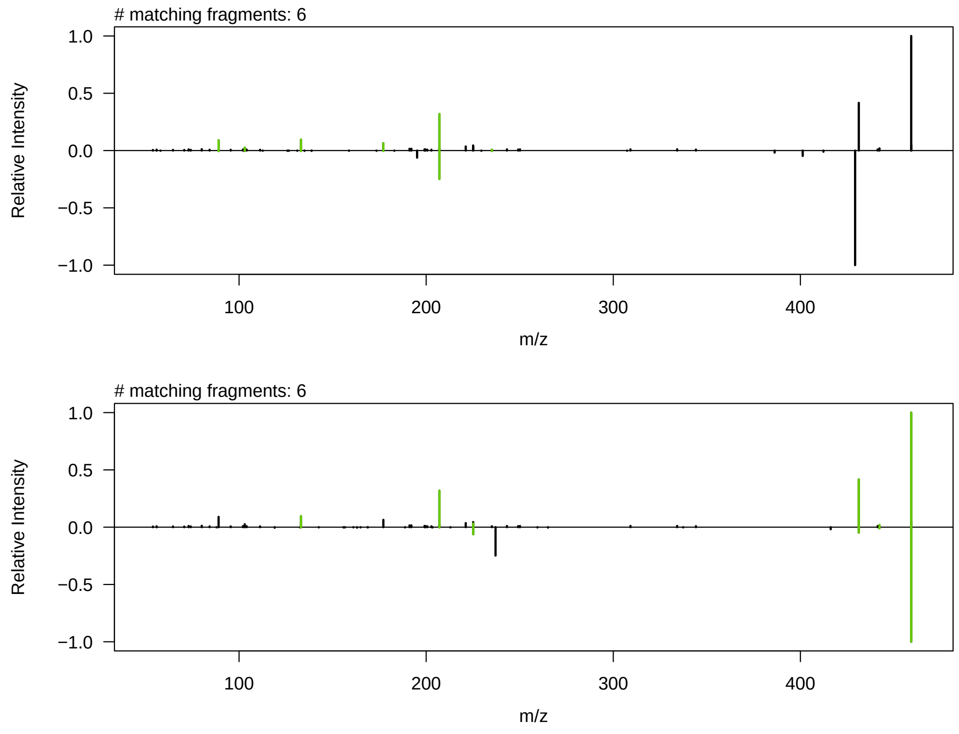

Figure SI-D175: Head to tail plots of irbesartan metabolite M3 and irbesartan. In the bottom plot, the mass spectrum of irbesartan is shifted by the mass difference. Matching fragments are highlighted in green.

Table SI-D82: Molecular network results and retention time prediction of irbesartan metabolite M3.

Comparison with	Irbesartan
MSn Score	53
Forward coverage	57
Reverse coverage	50
Forward match	29
Reverse match	9
ΔMass [g/mol]	29.9741
Measured retention time [min]	17.9
Predicted $logD_{OW}$ (pH = 2.7)	3.06
Predicted retention time [min]	18.7
Predicted retention time range (95% confidence interval) [min]	14.1-23.3
Predicted retention time range (99% confidence interval) [min]	12.7-24.8

Table SI-D83: Annotated MS2 spectrum of irbesartan metabolite M3.

m/z	Relative Intensity	Annotation
53.9611	5.40	
55.9293	6.93	
55.9383	6.24	
64.6654	5.82	
70.6944	5.02	
73.0284	10.23	$C_3H_4O_2 + H^+$
74.1657	6.56	
80.0543	12.06	
84.2521	6.60	
89.0597	89.96	$C_4H_8O_2 + H^+$
95.5331	6.51	
102.0672	9.14	
103.0389	25.58	$C_4H_6O_3 + H^+$
104.0420	8.51	
111.2199	6.89	
133.0857	95.90	$C_6H_{12}O_3 + H^+$
177.1121	64.11	$C_9H_{12}N_4 + H^+$
191.0912	15.38	$C_9H_{10}N_4O + H^+$
192.0948	15.54	
199.1262	11.18	
199.6270	9.63	
200.6308	7.35	
202.7861	7.41	

Continued on next page

Table SI-D83: Annotated MS2 spectrum of irbesartan metabolite M3.(Continued)

207.0917	318.57	$C_{14}H_{10}N_2 + H^+$
207.1115	22.04	$C_{11}H_{14}N_2O_2 + H^+$
221.1377	35.86	$C_{11}H_{16}N_4O + H^+$
225.1023	44.52	$C_{14}H_{12}N_2O + H^+$
225.1230	32.03	$C_{11}H_{16}N_2O_3 + H^+$
235.0976	7.85	$C_{14}H_{10}N_4 + H^+$
243.1526	10.39	
249.1327	8.91	
250.1372	10.19	
309.1824	10.94	
334.1299	10.91	$C_{20}H_{17}N_2O_3 + H^+$
344.1524	8.76	$C_{20}H_{17}N_5O + H^+$
431.2073	415.96	$C_{25}H_{26}N_4O_3 + H^+$
441.2729	9.69	
442.2733	19.10	
459.2140	999.00	$C_{25}H_{26}N_6O_3 + H^+$
459.2791	47.31	

Since no reference standard of irbesartan M3 is commercially available, an incubation experiment was conducted. The human liver S9 incubation of irbesartan led to the formation of irbesartan metabolite M3. Considering the spectral match of 0.698 (see Figure SI-D176) and the retention times of 17.9 and 18.0 minutes in the wastewater and the human liver S9 sample, respectively, further confidence could be gained that the detected feature in wastewater is irbesartan metabolite M3. Due to this diagnostic evidence, the final confidence level can be increased from level 3 to level 2b.

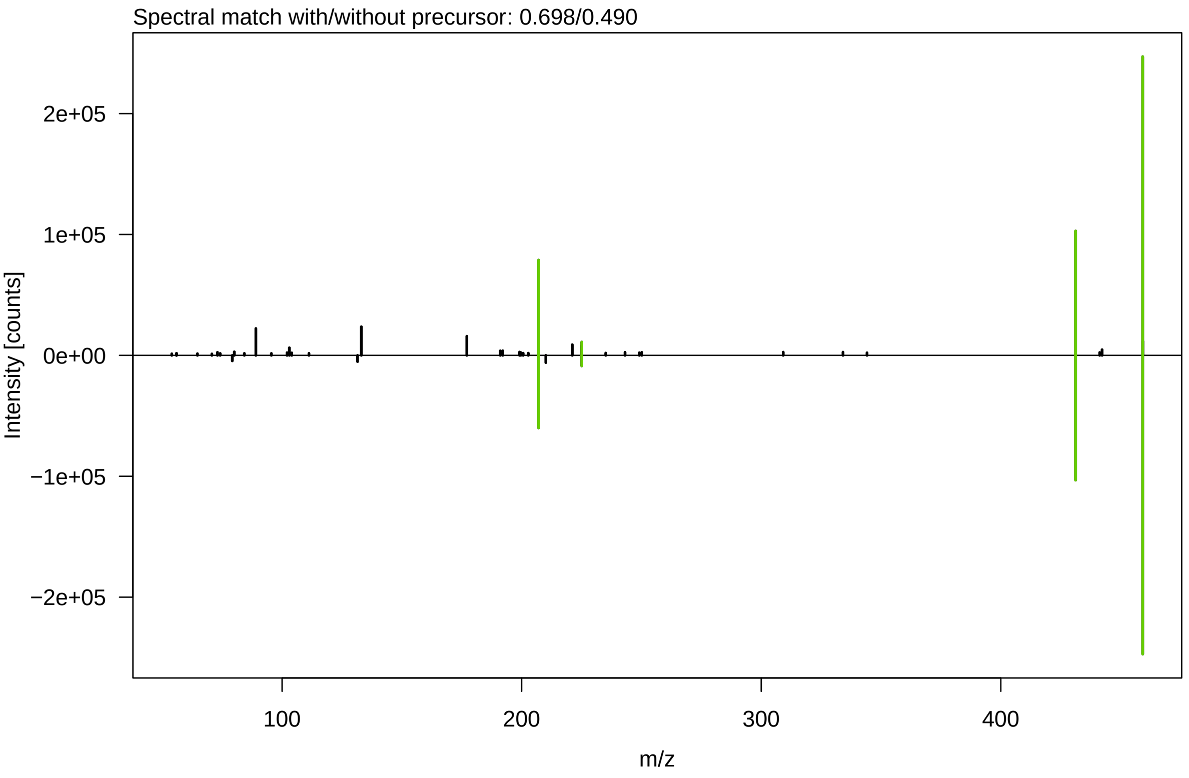

Figure SI-D176: Head to tail plot of irbesartan metabolite M3 in wastewater (top) and from human liver S9 incubation (bottom). Matching fragments are highlighted in green.

SI-D2.8.3 Irbesartan Metabolite M4

Table SI-D84: Information on identifiers, chemical properties, detection and confidence of identification of irbesartan metabolite M4.

IUPAC Name	2-(3-hydroxybutyl)-3-[[4-[2-(2*H*-tetrazol-5-yl)phenyl]phenyl]methyl]-1,3-diazaspiro[4.4]non-1-en-4-one
Molecular formula	$C_{25}H_{28}N_6O_2$
Monoisotopic mass [g/mol]	444.2274
Adduct	$[M\text{-}H]^-$
Retention time [min]	18.5
SMILES	CC(CCC1=NC2(CCCC2)C(=O)N1CC3=CC=C(C=C3)C4=CC=CC=C4C5=NNN=N5)O
InChI	InChI=1S/C25H28N6O2/c1-17(32)8-13-22-26-25(14-4-5-15-25)24(33)31(22)16-18-9-11-19(12-10-18)20-6-2-3-7-21(20)23-27-29-30-28-23/h2-3,6-7,9-12,17,32H,4-5,8,13-16H2,1H3,(H,27,28,29,30)
InChI-Key	YASLDPAAWWZGIP-UHFFFAOYSA-N
CAS RN	208923-64-2
Metabolite of	Irbesartan
Detection frequency	100% (15/15 samples)
Detected in	Altenrhein, Monday-Friday<br>Neugut, Monday-Friday<br>Werdhölzli, Monday-Friday
Intensity	E7
Initial confidence level	level 3
Initial confidence score	0.48
Final confidence level	level 2b

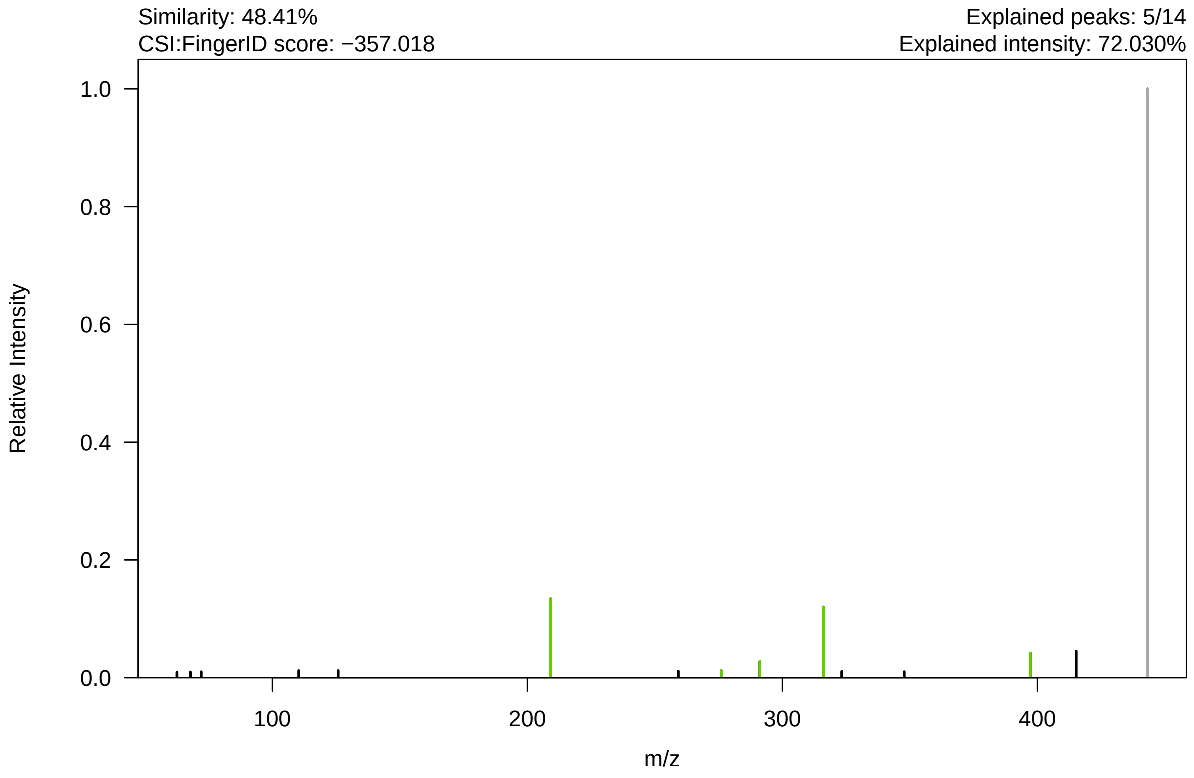

Figure SI-D177: Measured MS2 spectrum. Matching fragments with irbesartan metabolite M4 predicted by SIRIUS/CSI:FingerID are highlighted in green. The molecular ion in gray is not considered.

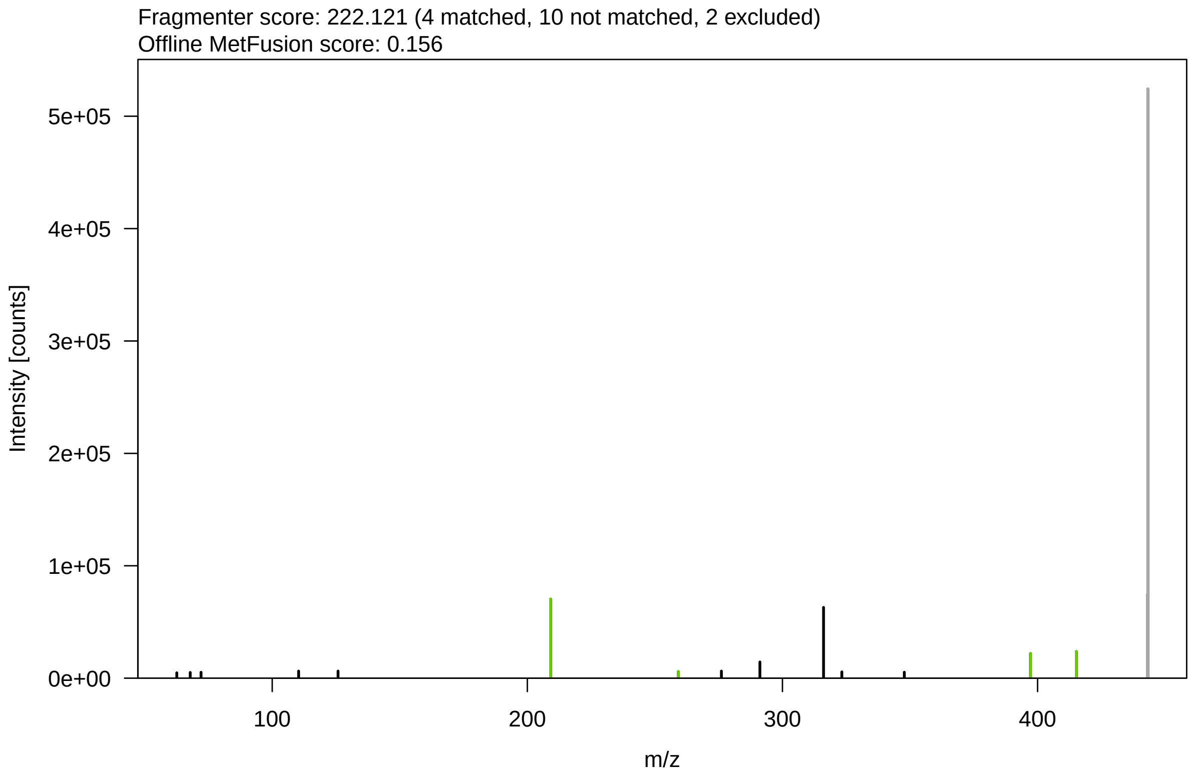

Figure SI-D178: Measured MS2 spectrum. Matching fragments with irbesartan metabolite M4 predicted by MetFrag are highlighted in green. The molecular ion in gray is not considered.

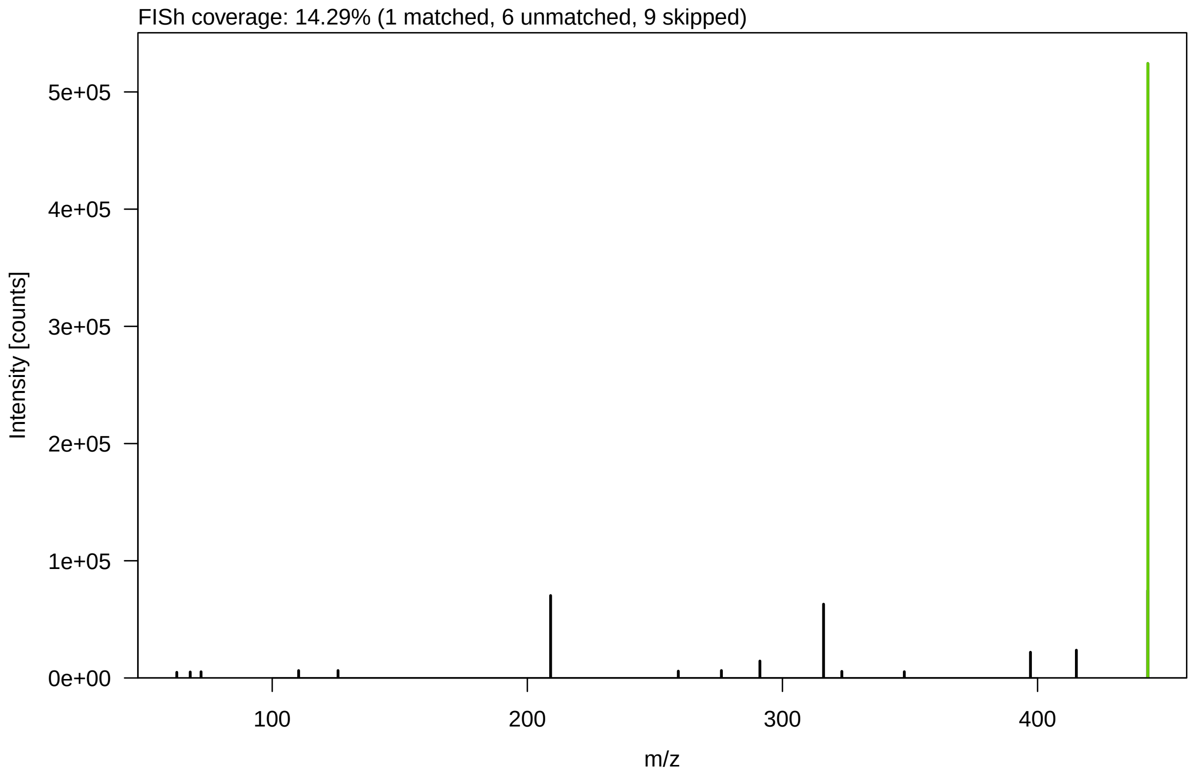

Figure SI-D179: Measured MS2 spectrum. Matching fragments with irbesartan metabolite M4 predicted by FISh Scoring are highlighted in green. Low intensity fragments are not considered and skipped.

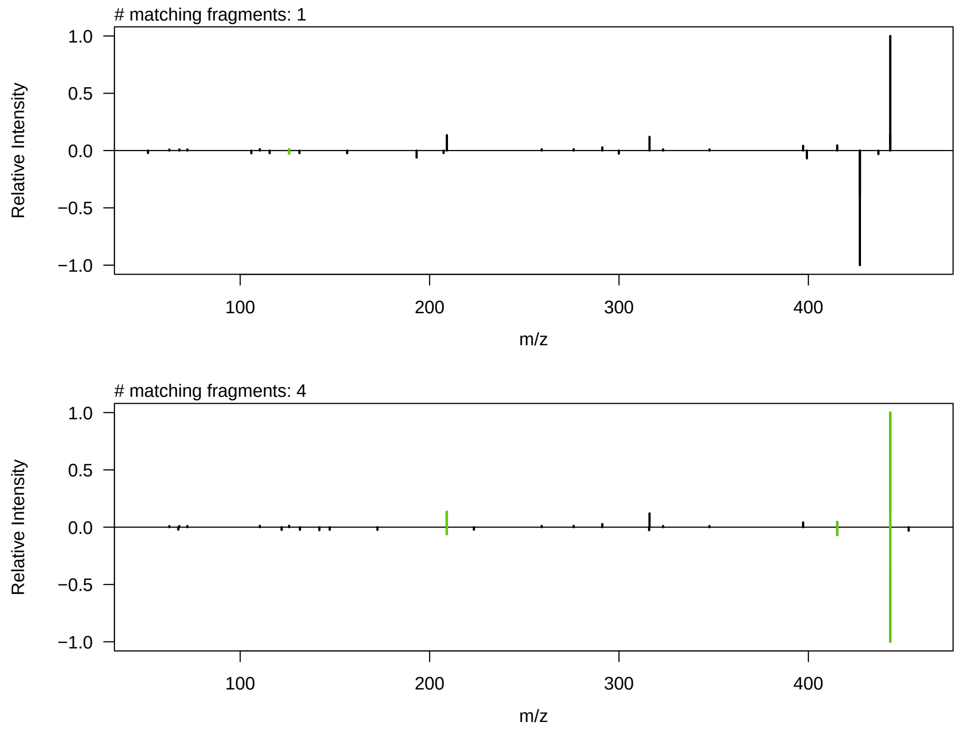

Figure SI-D180: Head to tail plots of irbesartan metabolite M4 and irbesartan. In the bottom plot, the mass spectrum of irbesartan is shifted by the mass difference. Matching fragments are highlighted in green.

Table SI-D85: Molecular network results and retention time prediction of irbesartan metabolite M4.

Comparison with	Irbesartan
MSn Score	59
Forward coverage	75
Reverse coverage	43
Forward match	3
Reverse match	3
ΔMass [g/mol]	15.9949
Measured retention time [min]	18.5
Predicted $logD_{OW}$ (pH = 4.8)	3.98
Predicted retention time [min]	21.5
Predicted retention time range (95% confidence interval) [min]	14.4-28.7
Predicted retention time range (99% confidence interval) [min]	12.0-31.0

Table SI-D86: Annotated MS2 spectrum of irbesartan metabolite M4.

m/z	Relative Intensity	Annotation
62.5952	9.19	
67.8648	9.71	
72.1005	10.08	
110.3584	12.02	
125.8040	12.08	
209.1296	134.01	$C_9H_{16}N_5O - H^-$
259.1831	11.11	$C_{16}H_{24}N_2O - H^-$
276.0789	12.07	$C_{16}H_{11}N_3O_2 - H^-$
291.1723	27.51	$C_{14}H_{22}N_5O_2 - H^-$
316.1112	119.98	$C_{19}H_{15}N_3O_2 - H^-$
323.2781	10.72	
347.7725	10.17	
397.2146	41.83	$C_{24}H_{26}N_6 - H^-$
415.2127	45.17	$C_{25}H_{28}N_4O_2 - H^-$
443.1718	142.36	
443.2223	999.00	$C_{25}H_{28}N_6O_2 - H^-$

Since no reference standard of irbesartan M4 is commercially available, an incubation experiment was conducted. The human liver S9 incubation of irbesartan led to the formation of irbesartan metabolite M4. Considering the spectral match of 0.709 (see Figure SI-D181) and the retention times of 18.5 and 18.7 minutes in the wastewater and the human liver S9 sample, respectively, further confidence could be gained that the detected feature in wastewater is irbesartan metabolite M4. Due to this diagnostic evidence, the final confidence level can be increased from level 3 to level 2b.

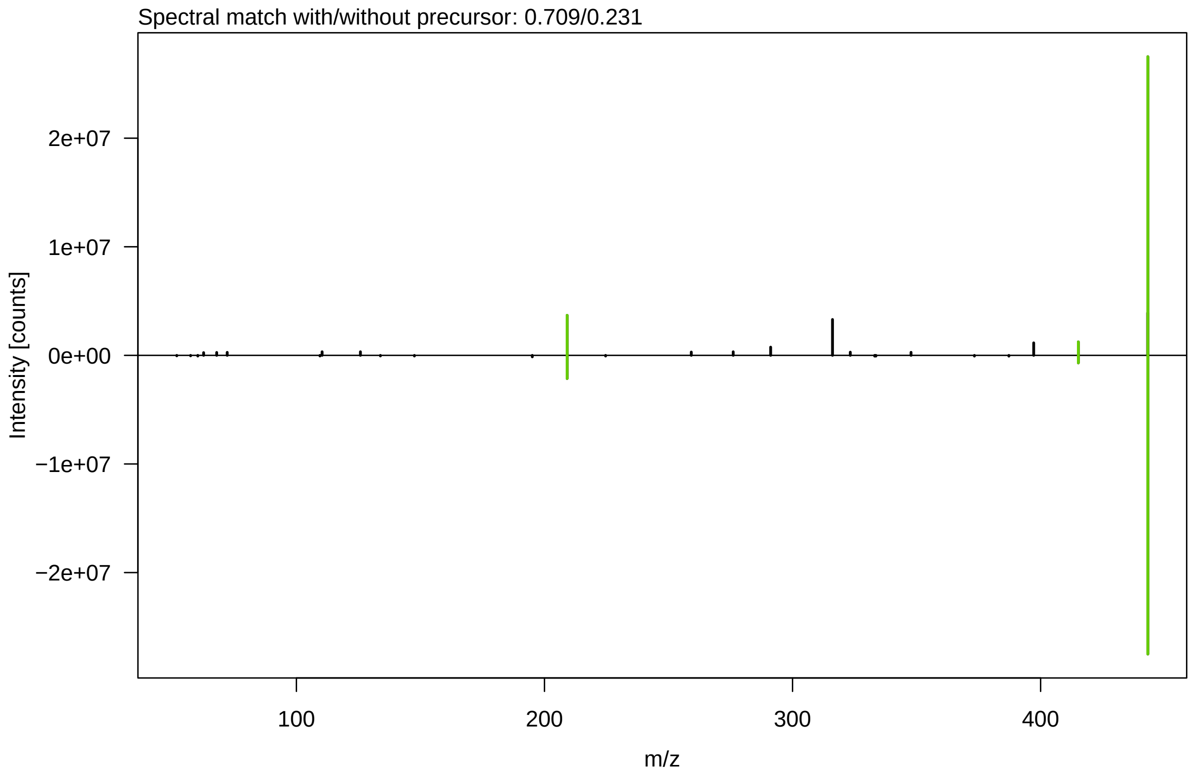

Figure SI-D181: Head to tail plot of irbesartan metabolite M4 in wastewater (top) and from human liver S9 incubation (bottom). Matching fragments are highlighted in green.

SI-D2.8.4 Irbesartan Metabolite M5

Table SI-D87: Information on identifiers, chemical properties, detection and confidence of identification of irbesartan metabolite M5.

IUPAC Name	2-butyl-8-hydroxy-3-[[4-[2-(2*H*-tetrazol-5-yl)phenyl]phenyl]methyl]-1,3-diazaspiro[4.4]non-1-en-4-one
Molecular formula	$C_{25}H_{28}N_6O_2$
Monoisotopic mass [g/mol]	444.2274
Adduct	$[M+H]^+$
Retention time [min]	17.9
SMILES	CCCCC1=NC2(CCC(C2)O)C(=O)N1CC3=CC=C(C=C3)C4=CC=CC=C4C5=NNN=N5
InChI	InChI=1S/C25H28N6O2/c1-2-3-8-22-26-25(14-13-19(32)15-25)24(33)31(22)16-17-9-11-18(12-10-17)20-6-4-5-7-21(20)23-27-29-30-28-23/h4-7,9-12,19,32H,2-3,8,13-16H2,1H3,(H,27,28,29,30)
InChI-Key	SGLAKMDXJPVHBY-UHFFFAOYSA-N
CAS RN	208988-52-7
Metabolite of	Irbesartan
Detection frequency	100% (15/15 samples)
Detected in	Altenrhein, Monday-Friday<br>Neugut, Monday-Friday<br>Werdhölzli, Monday-Friday
Intensity	E8
Initial confidence level	level 3
Initial confidence score	0.46
Final confidence level	level 2b

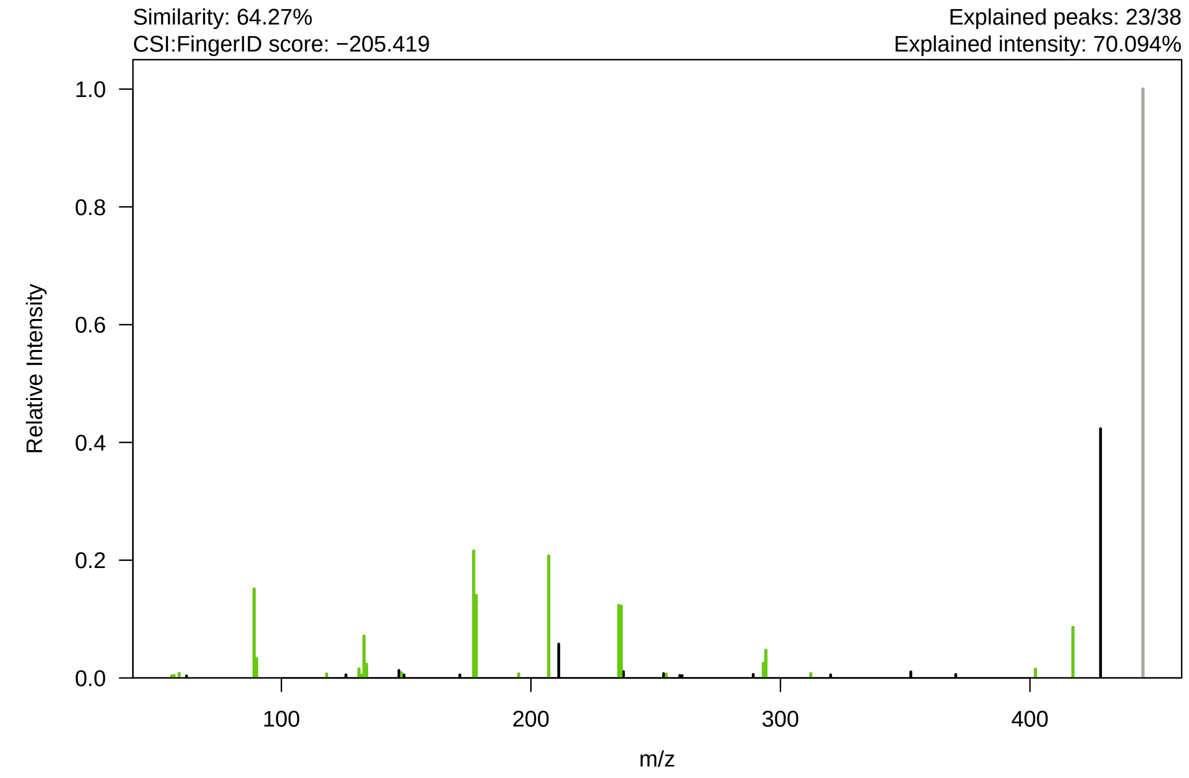

Figure SI-D182: Measured MS2 spectrum. Matching fragments with irbesartan metabolite M5 predicted by SIRIUS/CSI:FingerID are highlighted in green. The molecular ion in gray is not considered.

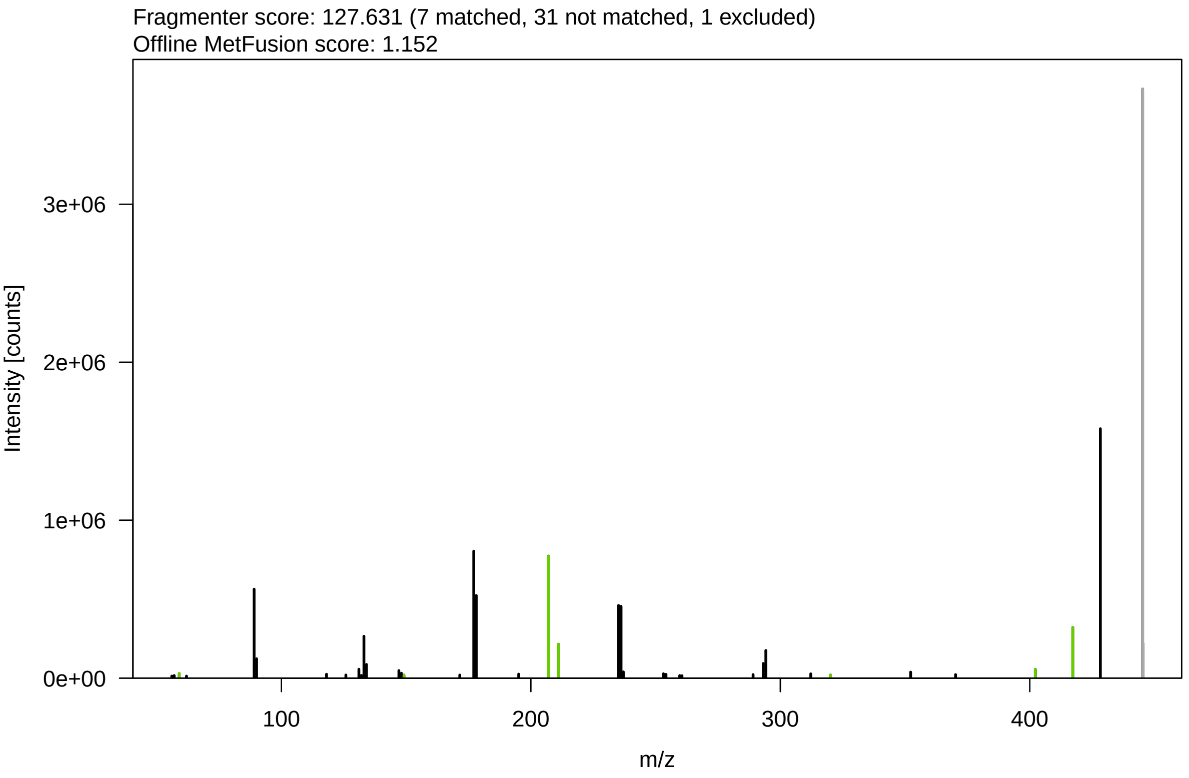

Figure SI-D183: Measured MS2 spectrum. Matching fragments with irbesartan metabolite M5 predicted by MetFrag are highlighted in green. The molecular ion in gray is not considered.

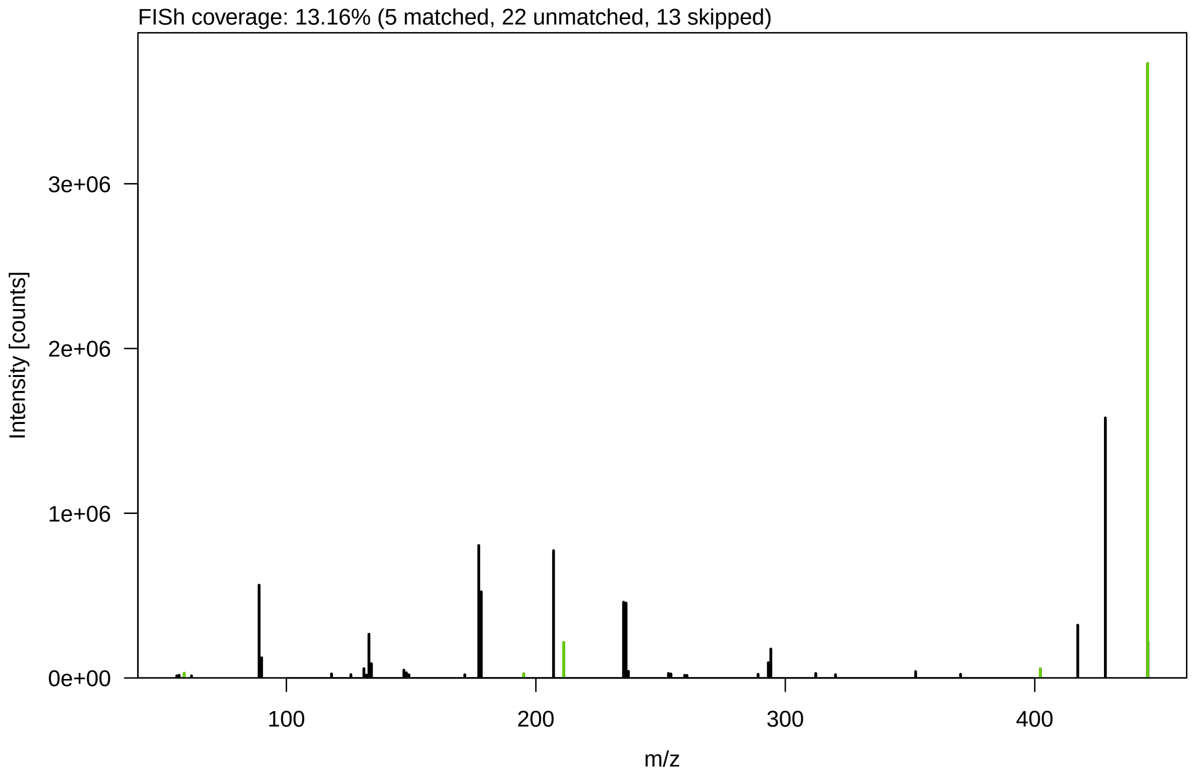

Figure SI-D184: Measured MS2 spectrum. Matching fragments with irbesartan metabolite M5 predicted by FISh Scoring are highlighted in green. Low intensity fragments are not considered and skipped.

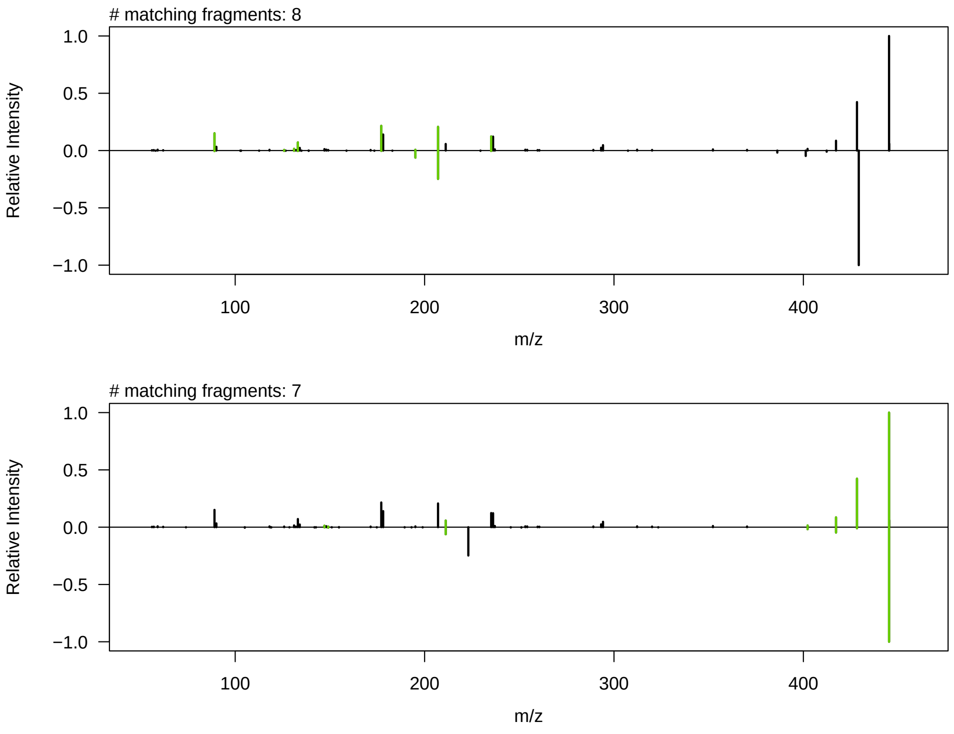

Figure SI-D185: Head to tail plots of irbesartan metabolite M5 and irbesartan. In the bottom plot, the mass spectrum of irbesartan is shifted by the mass difference. Matching fragments are highlighted in green.

Table SI-D88: Molecular network results and retention time prediction of irbesartan metabolite M5.

Comparison with	Irbesartan
MSn Score	57
Forward coverage	71
Reverse coverage	43
Forward match	36
Reverse match	33
ΔMass [g/mol]	15.9949
Measured retention time [min]	17.9
Predicted $logD_{OW}$ (pH = 2.7)	2.87
Predicted retention time [min]	18.5
Predicted retention time range (95% confidence interval) [min]	13.9-23.1
Predicted retention time range (99% confidence interval) [min]	12.4-24.5

Table SI-D89: Annotated MS2 spectrum of irbesartan metabolite M5.

m/z	Relative Intensity	Annotation
56.0603	3.60	$C_4H_7 + H^+$
57.0177	4.43	$N_4 + H^+$
59.0493	7.72	$CH_4N_3 + H^+$
61.9661	3.55	
89.0597	150.93	
90.0630	32.91	
118.0944	6.67	
125.8472	5.45	
131.0701	15.23	$C_4H_8N_3O_2 + H^+$
132.0739	4.77	
133.0859	71.18	$C_4H_{10}N_3O_2 + H^+$
134.0893	23.28	
147.1015	12.83	
148.1052	8.41	
149.1183	5.23	$C_{10}H_{14}N + H^+$
171.5067	5.37	
177.1120	215.40	$C_9H_{12}N_4 + H^+$
178.1153	140.15	
195.1232	6.86	$C_9H_{14}N_4O + H^+$
207.0915	206.95	$C_1H_{10}N_2 + H^+$
211.1441	57.56	$C_{11}H_{18}N_2O_2 + H^+$
235.1537	123.28	$C_{12}H_{18}N_4O + H^+$
236.1571	121.85	$C_{18}H_{19} + H^+$

Continued on next page

Table SI-D89: Annotated MS2 spectrum of irbesartan metabolite M5.(Continued)

237.0753	10.77	
253.1627	7.51	
254.1688	6.57	$C_{18}H_{21}O + H^{+}$
259.6609	4.44	
260.5878	4.15	
289.0932	6.04	
293.1944	24.91	$C_{15}H_{24}N_{4}O_{2} + H^{+}$
294.1987	47.04	$C_{21}H_{25}O + H^{+}$
312.2083	7.39	$C_{21}H_{27}O_{2} + H^{+}$
320.1128	5.40	$C_{17}H_{13}N_{5}O_{2} + H^{+}$
352.2419	10.37	
370.2562	6.03	
402.2176	14.92	$C_{25}H_{27}N_{3}O_{2} + H^{+}$
417.2289	85.89	$C_{25}H_{28}N_{4}O_{2} + H^{+}$
428.2934	422.89	
445.2343	999.00	$C_{25}H_{28}N_{6}O_{2} + H^{+}$
445.3182	59.20	

Since no reference standard of irbesartan M5 is commercially available, an incubation experiment was conducted. The human liver S9 incubation of irbesartan led to the formation of irbesartan metabolite M5. Considering the spectral match of 0.450 (see Figure SI-D186) and the retention times of 17.9 and 18.0 minutes in the wastewater and the human liver S9 sample, respectively, further confidence could be gained that the detected feature in wastewater is irbesartan metabolite M5. Due to this diagnostic evidence, the final confidence level can be increased from level 3 to level 2b.

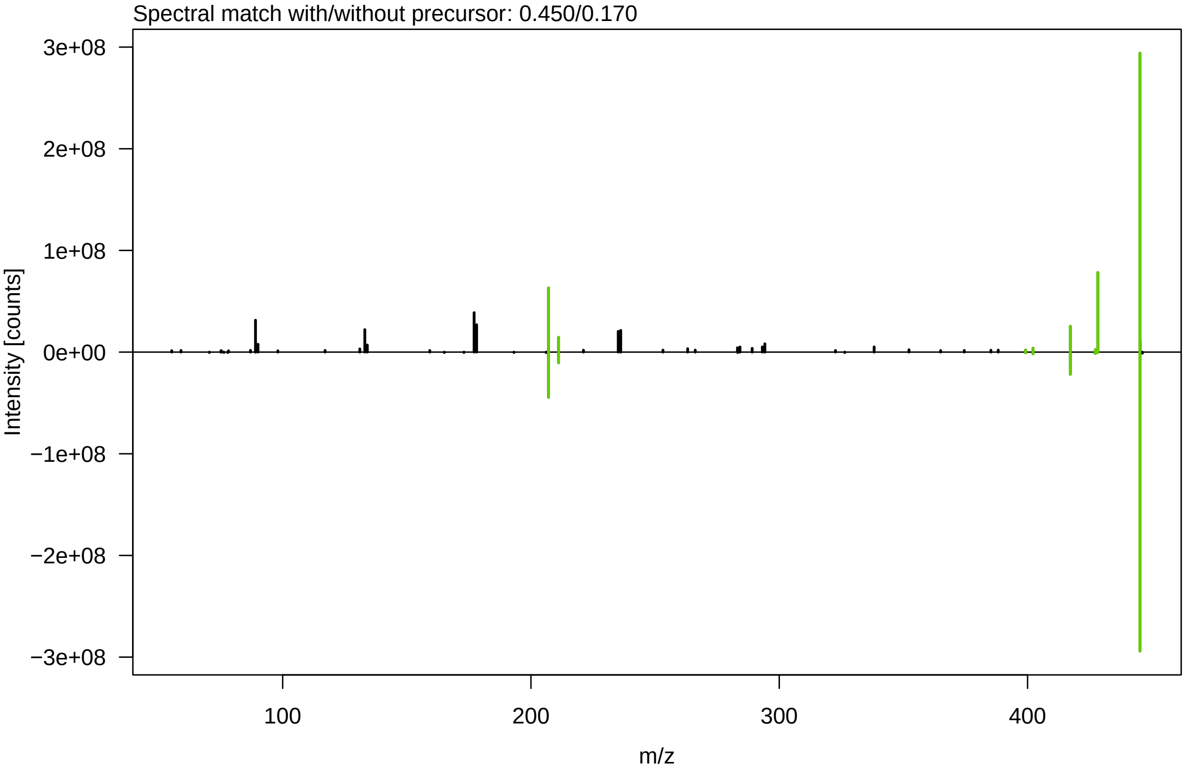

Figure SI-D186: Head to tail plot of irbesartan metabolite M5 in wastewater (top) and from human liver S9 incubation (bottom). Matching fragments are highlighted in green.

SI-D2.8.5 Irbesartan Metabolite M6

Table SI-D90: Information on identifiers, chemical properties, detection and confidence of identification of irbesartan metabolite M6.

IUPAC Name	2-(3-oxobutyl)-3-[[4-[2-(2*H*-tetrazol-5-yl)phenyl]phenyl]methyl]-1,3-diazaspiro[4.4]non-1-en-4-one
Molecular formula	$C_{25}H_{26}N_6O_2$
Monoisotopic mass [g/mol]	442.2117
Adduct	$[M\text{-}H]^-$
Retention time [min]	17.6
SMILES	CC(=O)CCC1=NC2(CCCC2)C(=O)N1CC3=CC=C(C=C3)C4=CC=CC=C4C5=NNN=N5
InChI	InChI=1S/C25H26N6O2/c1-17(32)8-13-22-26-25(14-4-5-15-25)24(33)31(22)16-18-9-11-19(12-10-18)20-6-2-3-7-21(20)23-27-29-30-28-23/h2-3,6-7,9-12H,4-5,8,13-16H2,1H3,(H,27,28,29,30)
InChI-Key	MIGTZGLTQNODIS-UHFFFAOYSA-N
CAS RN	208923-65-3
Metabolite of	Irbesartan
Detection frequency	100% (15/15 samples)
Detected in	Altenrhein, Monday-Friday<br>Neugut, Monday-Friday<br>Werdhölzli, Monday-Friday
Intensity	E7
Initial confidence level	level 3
Initial confidence score	0.41
Final confidence level	level 3

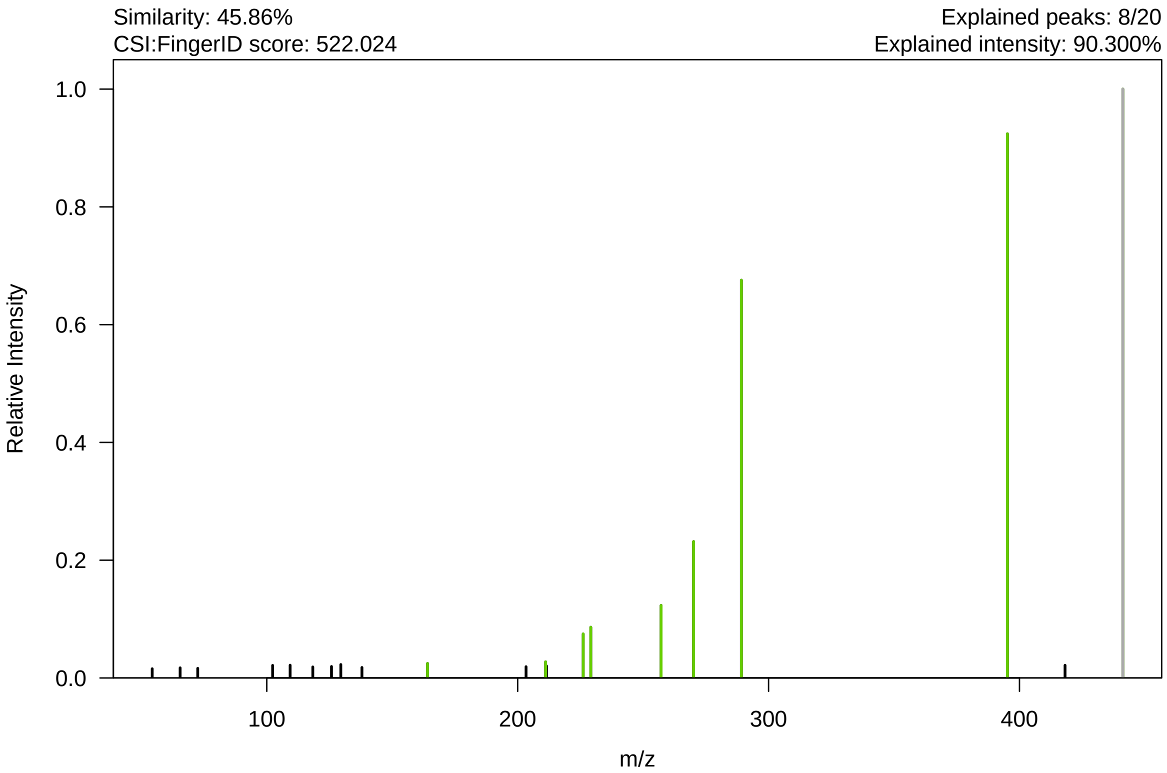

Figure SI-D187: Measured MS2 spectrum. Matching fragments with irbesartan metabolite M6 predicted by SIRIUS/CSI:FingerID are highlighted in green. The molecular ion in gray is not considered.

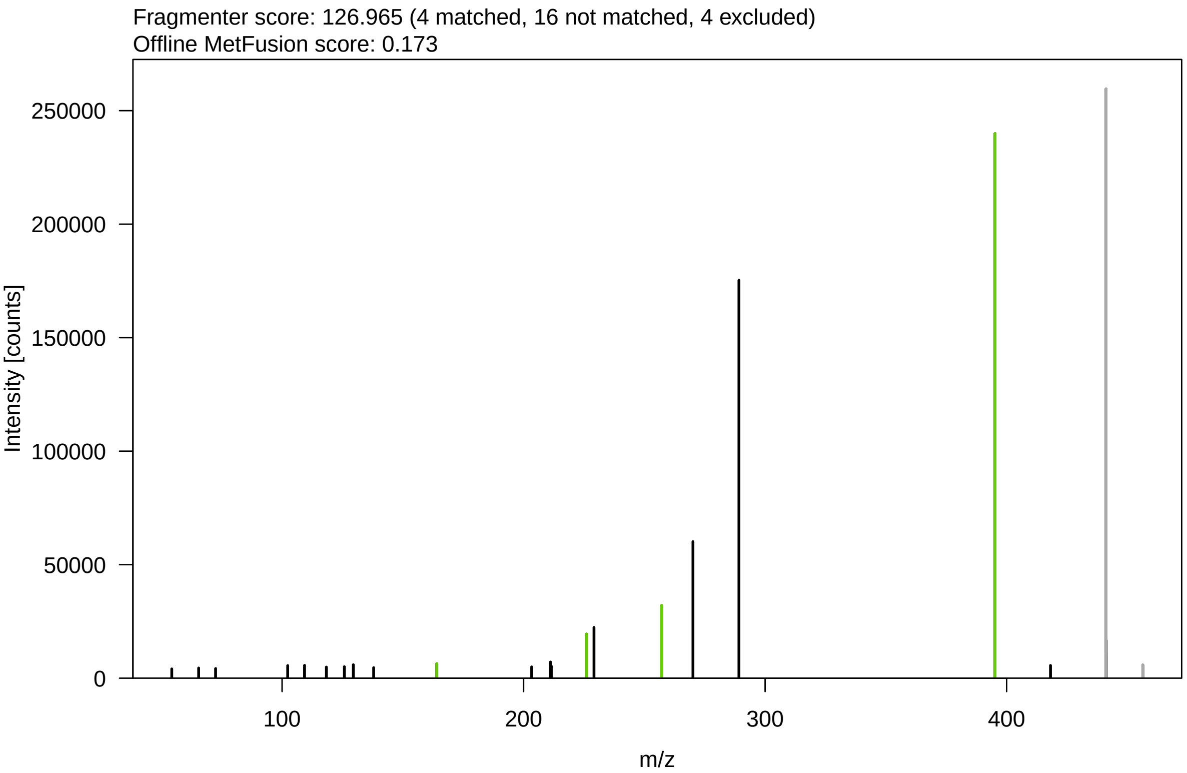

Figure SI-D188: Measured MS2 spectrum. Matching fragments with irbesartan metabolite M6 predicted by MetFrag are highlighted in green. The molecular ion in gray is not considered.

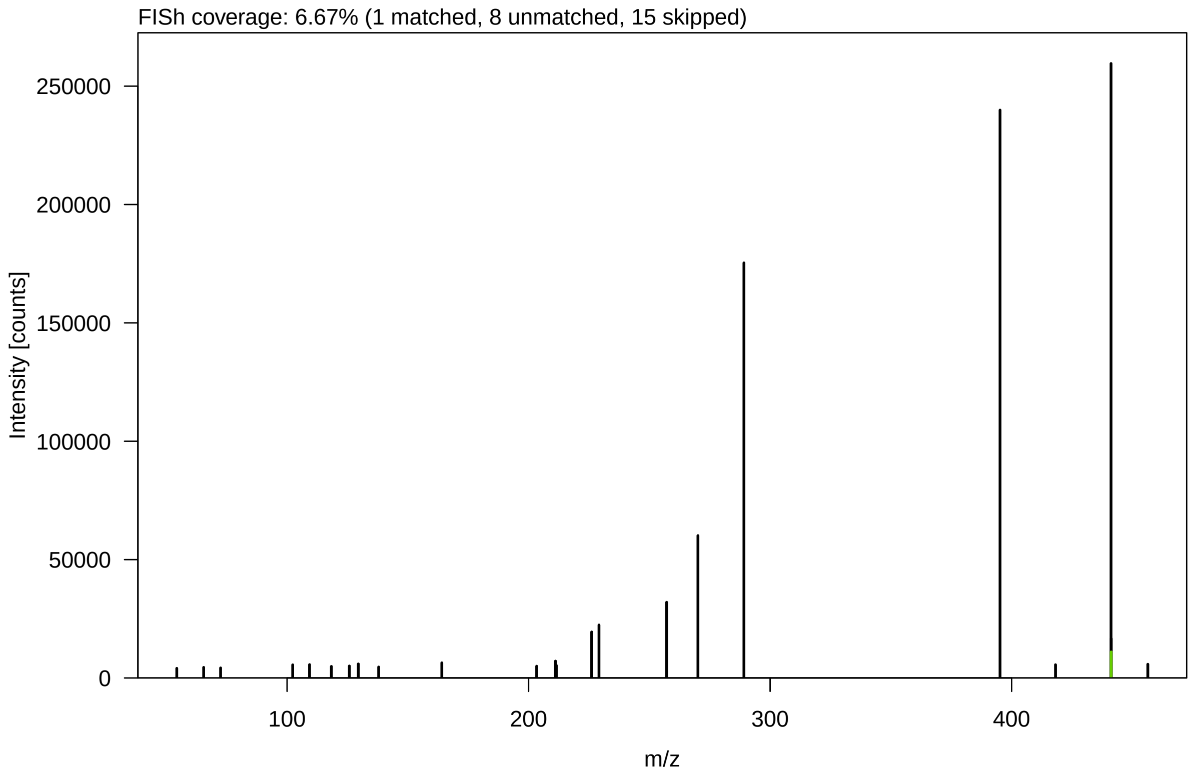

Figure SI-D189: Measured MS2 spectrum. Matching fragments with irbesartan metabolite M6 predicted by FISh Scoring are highlighted in green. Low intensity fragments are not considered and skipped.

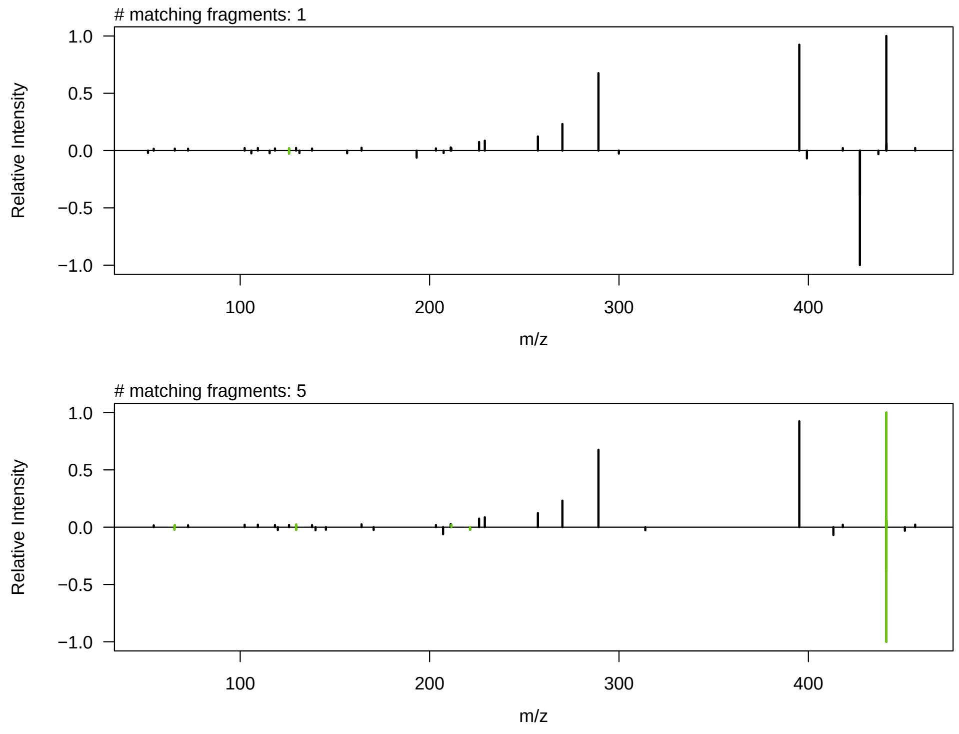

Figure SI-D190: Head to tail plots of irbesartan metabolite M6 and irbesartan. In the bottom plot, the mass spectrum of irbesartan is shifted by the mass difference. Matching fragments are highlighted in green.

Table SI-D91: Molecular network results and retention time prediction of irbesartan metabolite M6.

Comparison with	Irbesartan
MSn Score	35
Forward coverage	50
Reverse coverage	20
Forward match	2
Reverse match	3
ΔMass [g/mol]	13.9793
Measured retention time [min]	17.6
Predicted $logD_{OW}$ (pH = 4.8)	4.14
Predicted retention time [min]	21.8
Predicted retention time range (95% confidence interval) [min]	14.7-29.0
Predicted retention time range (99% confidence interval) [min]	12.3-31.3

Table SI-D92: Annotated MS2 spectrum of irbesartan metabolite M6.

m/z	Relative Intensity	Annotation
54.3344	15.71	
65.4650	17.25	
72.4791	16.36	
102.3356	21.36	
109.3054	21.72	
118.3496	18.79	
125.7958	19.52	
129.5027	22.84	
137.9258	17.83	
164.0715	24.64	$C_7H_9N_4O - H^-$
203.3408	19.17	
211.1343	27.47	$C_8H_{16}N_6O - H^-$
211.4780	20.51	
226.1232	74.81	$C_{13}H_{15}N_4 - H^-$
229.1437	86.13	$C_8H_{18}N_6O_2 - H^-$
257.1660	123.07	$C_{14}H_{20}N_5 - H^-$
270.1134	231.45	$C_{14}H_{15}N_4O_2 - H^-$
289.1558	674.83	$C_{14}H_{20}N_5O_2 - H^-$
395.1979	923.26	$C_{24}H_{24}N_6 - H^-$
418.1651	21.56	
441.1591	999.00	
441.2049	42.37	$C_{25}H_{26}N_6O_2 - H^-$
441.2492	64.22	
456.3899	22.30	

No reference standard of irbesartan metabolite M6 was purchasable. Therefore, a human liver S9 incubation experiment with irbesartan was performed, to generate irbesartan metabolites *in vitro*. However, no compound with a precursor matching the one of irbesartan metabolite M6 was detectd within a retention time window of ±3 minutes of the suspect.

SI-D2.8.6 Irbesartan Metabolite SR49498

Table SI-D93: Information on identifiers, chemical properties, detection and confidence of identification of irbesartan metabolite SR49498.

IUPAC Name	1-(pentanoylamino)-*N*-[[4-[2-(2*H*-tetrazol-5-yl)phenyl]phenyl]methyl]cyclopentane-1-carboxamide
Molecular formula	$C_{25}H_{30}N_6O_2$
Monoisotopic mass [g/mol]	446.2430
Adduct	$[M-H]^-$
Retention time [min]	18.7
SMILES	CCCCC(=O)NC1(CCCC1)C(=O)NCC2=CC=C(C=C2)C3=CC=CC=C3C4=NNN=N4
InChI	InChI=1S/C25H30N6O2/c1-2-3-10-22(32)27-25(15-6-7-16-25)24(33)26-17-18-11-13-19(14-12-18)20-8-4-5-9-21(20)23-28-30-31-29-23/h4-5,8-9,11-14H,2-3,6-7,10,15-17H2,1H3,(H,26,33)(H,27,32)(H,28,29,30,31)
InChI-Key	PAKGYCNZUGIDHV-UHFFFAOYSA-N
CAS RN	748812-53-5
Metabolite of	Irbesartan
Detection frequency	100% (15/15 samples)
Detected in	Altenrhein, Monday-Friday<br>Neugut, Monday-Friday<br>Werdhölzli, Monday-Friday
Intensity	E6
Initial confidence level	level 3
Initial confidence score	0.31
Final confidence level	level 2b

Due to the low intensity fragments, SIRIUS/CSI:FingerID computation was not possible.

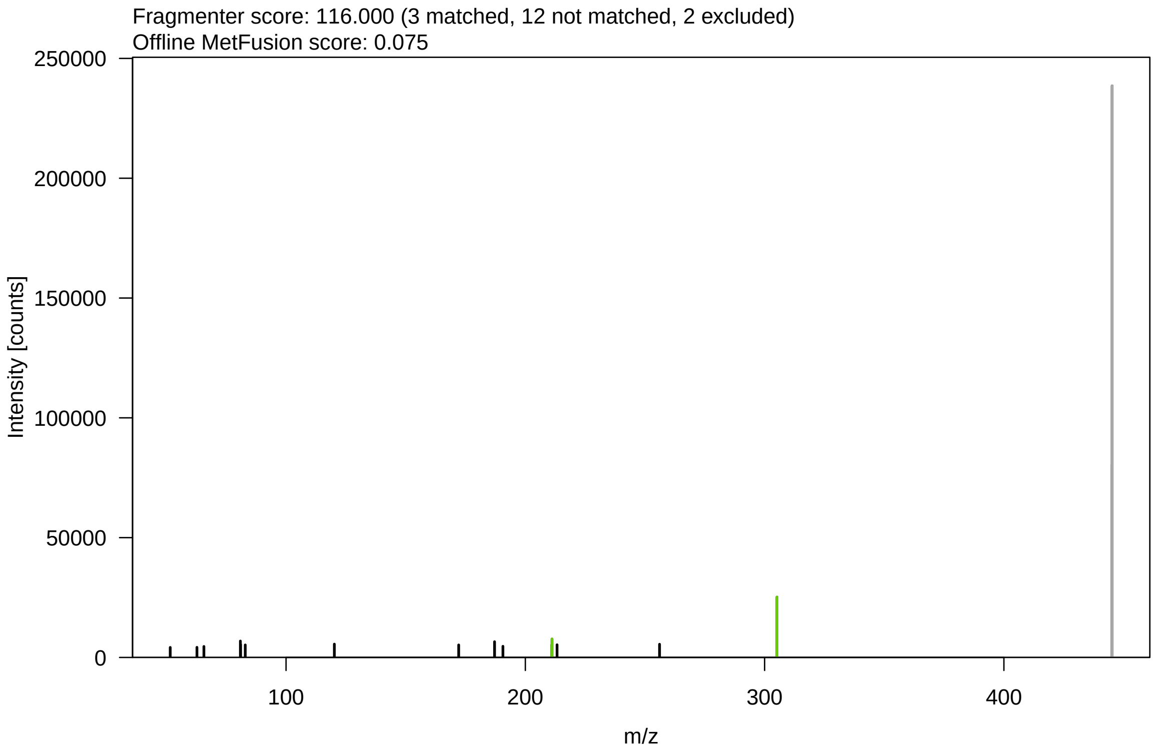

Figure SI-D191: Measured MS2 spectrum. Matching fragments with irbesartan metabolite SR49498 predicted by MetFrag are highlighted in green. The molecular ion in gray is not considered.

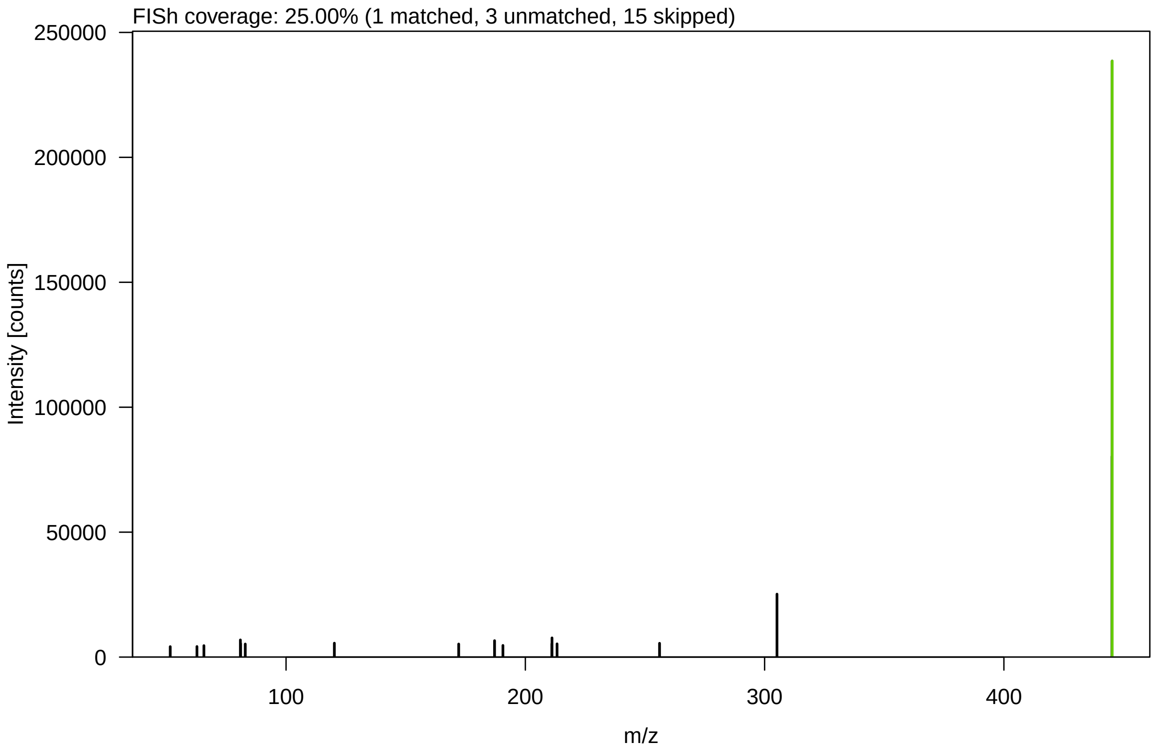

Figure SI-D192: Measured MS2 spectrum. Matching fragments with irbesartan metabolite SR49498 predicted by FISh Scoring are highlighted in green. Low intensity fragments are not considered and skipped.

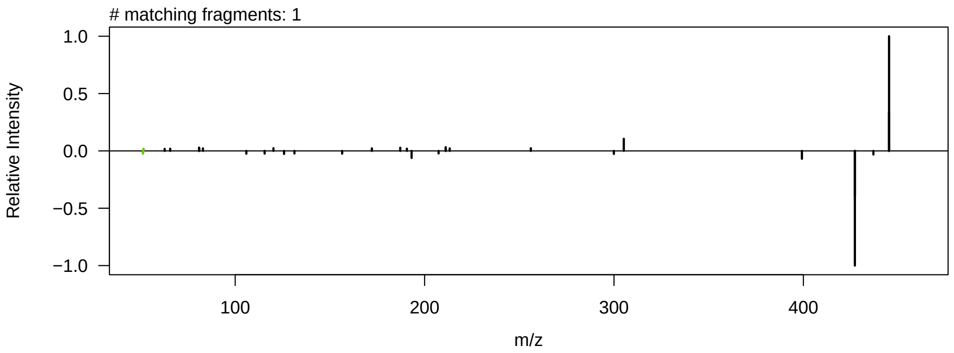

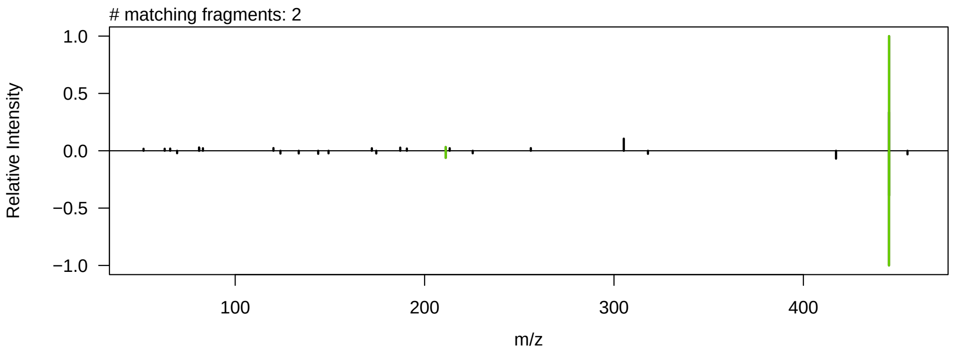

Figure SI-D193: Head to tail plots of irbesartan metabolite SR49498 and irbesartan. In the bottom plot, the mass spectrum of irbesartan is shifted by the mass difference. Matching fragments are highlighted in green.

Table SI-D94: Molecular network results and retention time prediction of irbesartan metabolite SR49498.

Comparison with	Irbesartan
MSn Score	69
Forward coverage	75
Reverse coverage	63
Forward match	3
Reverse match	5
ΔMass [g/mol]	18.0106
Measured retention time [min]	18.7
Predicted $logD_{OW}$ (pH = 4.8)	4.35
Predicted retention time [min]	22.2
Predicted retention time range (95% confidence interval) [min]	15.0-29.4
Predicted retention time range (99% confidence interval) [min]	12.7-31.7

Table SI-D95: Annotated MS2 spectrum of irbesartan metabolite SR49498.

m/z	Relative Intensity	Annotation
51.5971	17.51	
62.7626	17.70	
65.6664	19.20	
80.9166	28.74	
80.9922	22.76	
82.9539	21.88	
120.1875	23.30	
172.1489	21.89	
187.1340	27.51	
190.6316	19.51	
211.1002	22.36	$C_{14}H_{14}NO - H^{-}$
211.1456	32.14	$C_{11}H_{20}N_2O_2 - H^{-}$
213.2517	22.15	
256.0848	23.10	
305.1671	105.51	$C_{20}H_{22}N_2O - H^{-}$
445.1889	336.15	
445.2358	999.00	$C_{25}H_{30}N_6O_2 - H^{-}$

Since no reference standard of irbesartan SR49498 is commercially available, an incubation experiment was conducted. The human liver S9 incubation of irbesartan led to the formation of irbesartan metabolite SR49498. Considering the spectral match of 0.625 (see Figure SI-D194) and the retention times of 18.7 and 19.1 minutes in the wastewater and the human liver S9 sample, respectively, further confidence could be gained that the detected feature in wastewater is irbesartan metabolite SR49498. Due to this diagnostic evidence, the final confidence level can be increased from level 3 to level 2b.

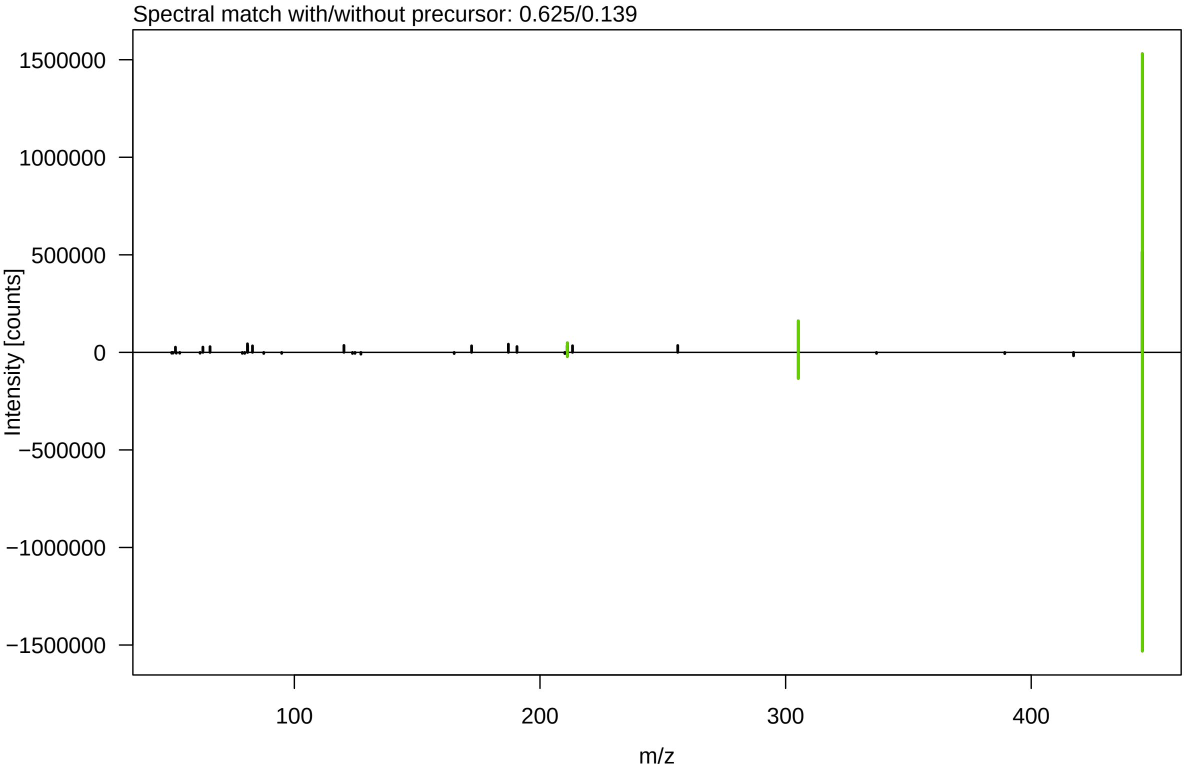

Figure SI-D194: Head to tail plot of irbesartan metabolite SR49498 in wastewater (top) and from human liver S9 incubation (bottom). Matching fragments are highlighted in green.

SI-D2.9 Losartan Metabolites

Losartan is as irbesartan an angiotesin II receptor blocker and belongs to the class of antihypertensive medications.[13] Moreover, it is used to treat diabetic nephropathy and to reduce the risk of stroke.[2] Three human metabolites could be identified by suspect screening. The identified metabolites are highlighted in the metabolism scheme of losartan in Figure SI-D195. A pane of the molecular network showing the losartan cluster is shown in Figure SI-D196. The following subsections give more details on the individual metabolites.

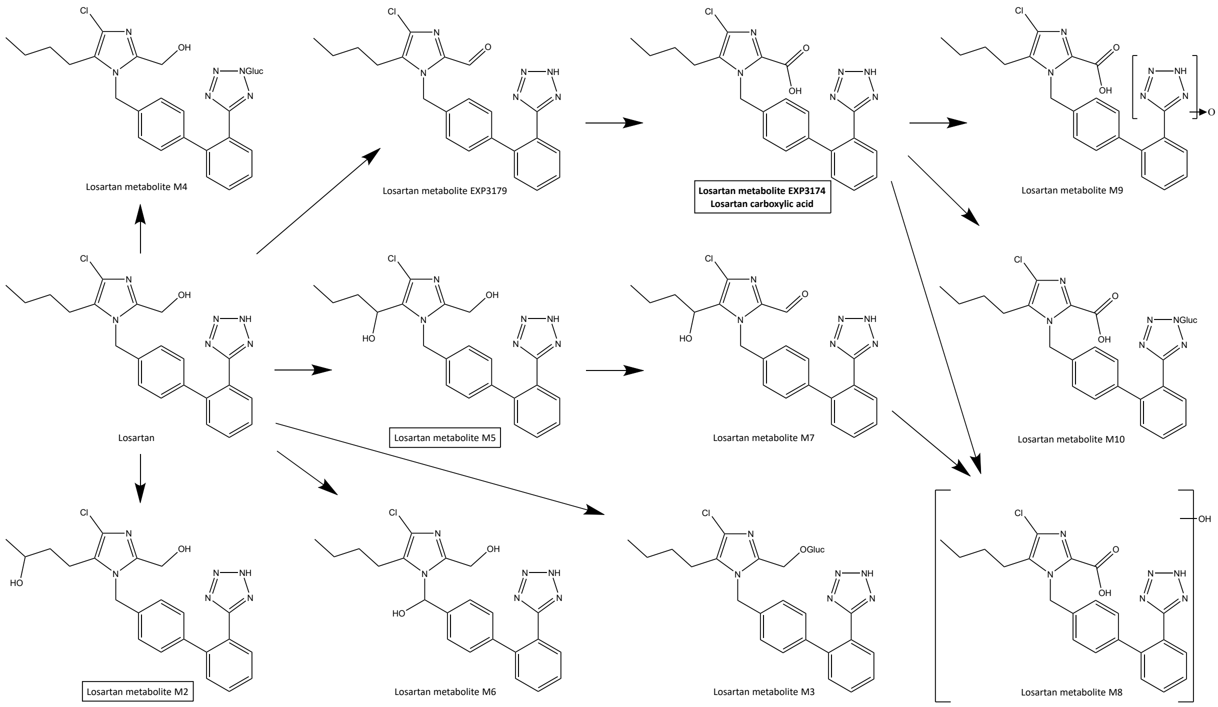

Figure SI-D195: Metabolism of losartan. Framed metabolites were identified during suspect screening. Scheme adapted from.[13]

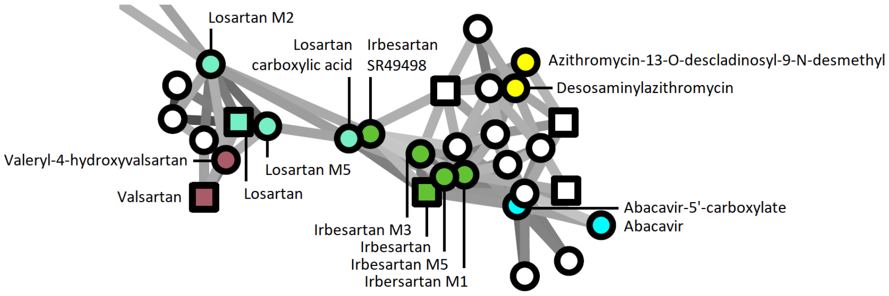

Figure SI-D196: Excerpt of the molecular network showing the losartan cluster.

SI-D2.9.1 Losartan Carboxylic Acid

Table SI-D96: Information on identifiers, chemical properties, detection and confidence of identification of losartan carboxylic acid.

IUPAC Name	2-butyl-5-chloro-3-[[4-[2-(2*H*-tetrazol-5-yl)phenyl]phenyl]methyl]imidazole-4-carboxylic acid
Molecular formula	$C_{22}H_{21}ClN_6O_2$
Monoisotopic mass [g/mol]	436.1415
Adduct	$[M+H]^+$
Retention time [min]	19.9
SMILES	CCCCC1=NC(=C(N1CC2=CC=C(C=C2)C3=CC=CC=C3C4=NNN=N4)C(=O)O)Cl
InChI	InChI=1S/C22H21ClN6O2/c1-2-3-8-18-24-20(23)19(22(30)31)29(18)13-14-9-11-15(12-10-14)16-6-4-5-7-17(16)21-25-27-28-26-21/h4-7,9-12H,2-3,8,13H2,1H3,(H,30,31)(H,25,26,27,28)
InChI-Key	ZEUXAIYYDDCIRX-UHFFFAOYSA-N
CAS RN	124750-92-1
Metabolite of	Losartan
Detection frequency	100% (15/15 samples)
Detected in	Altenrhein, Monday-Friday<br>Neugut, Monday-Friday<br>Werdhölzli, Monday-Friday
Intensity	E6-E7
Initial confidence level	level 3
Initial confidence score	0.33
Final confidence level	level 1

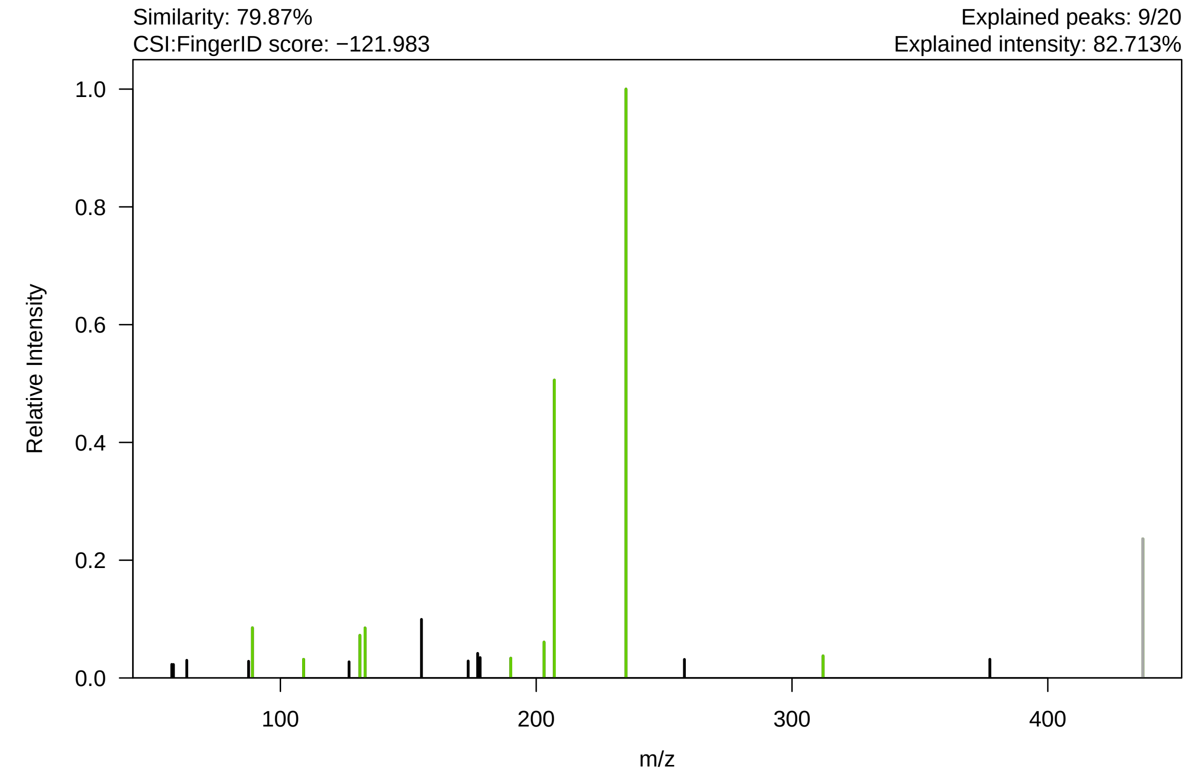

Figure SI-D197: Measured MS2 spectrum. Matching fragments with losartan carboxylic acid predicted by SIRIUS/CSI:FingerID are highlighted in green. The molecular ion in gray is not considered.

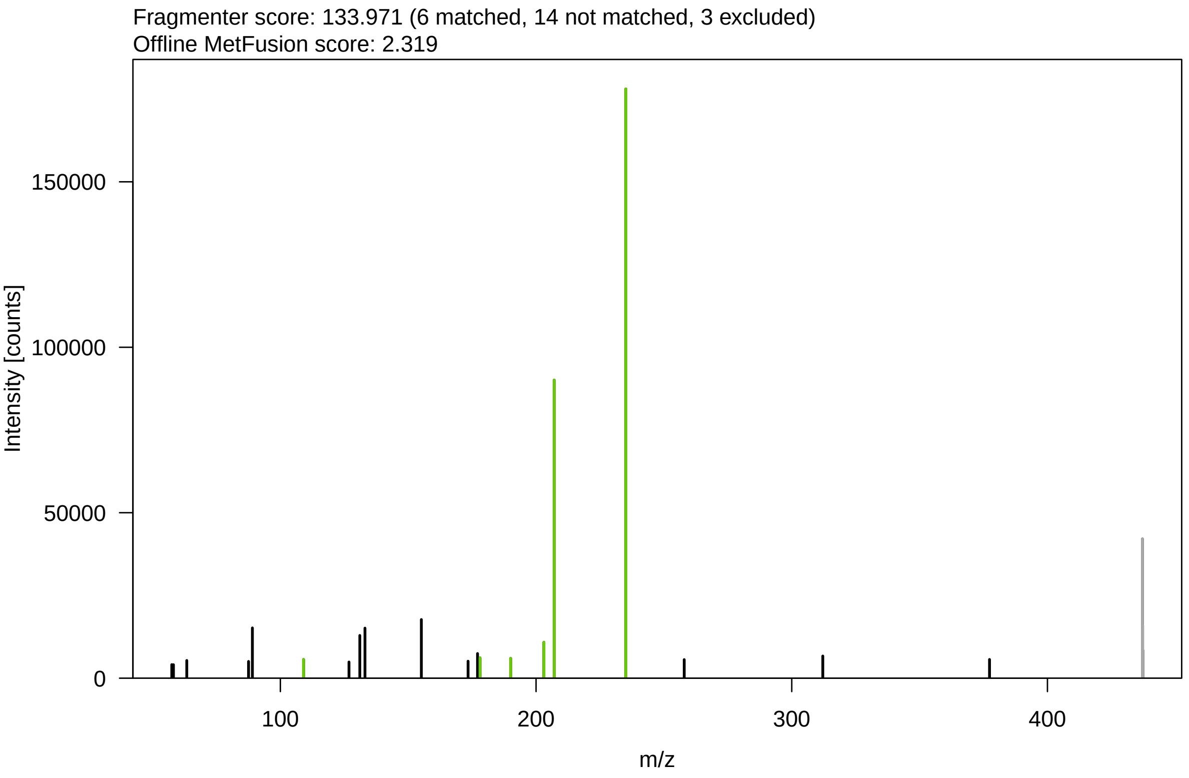

Figure SI-D198: Measured MS2 spectrum. Matching fragments with losartan carboxylic acid predicted by MetFrag are highlighted in green. The molecular ion in gray is not considered.

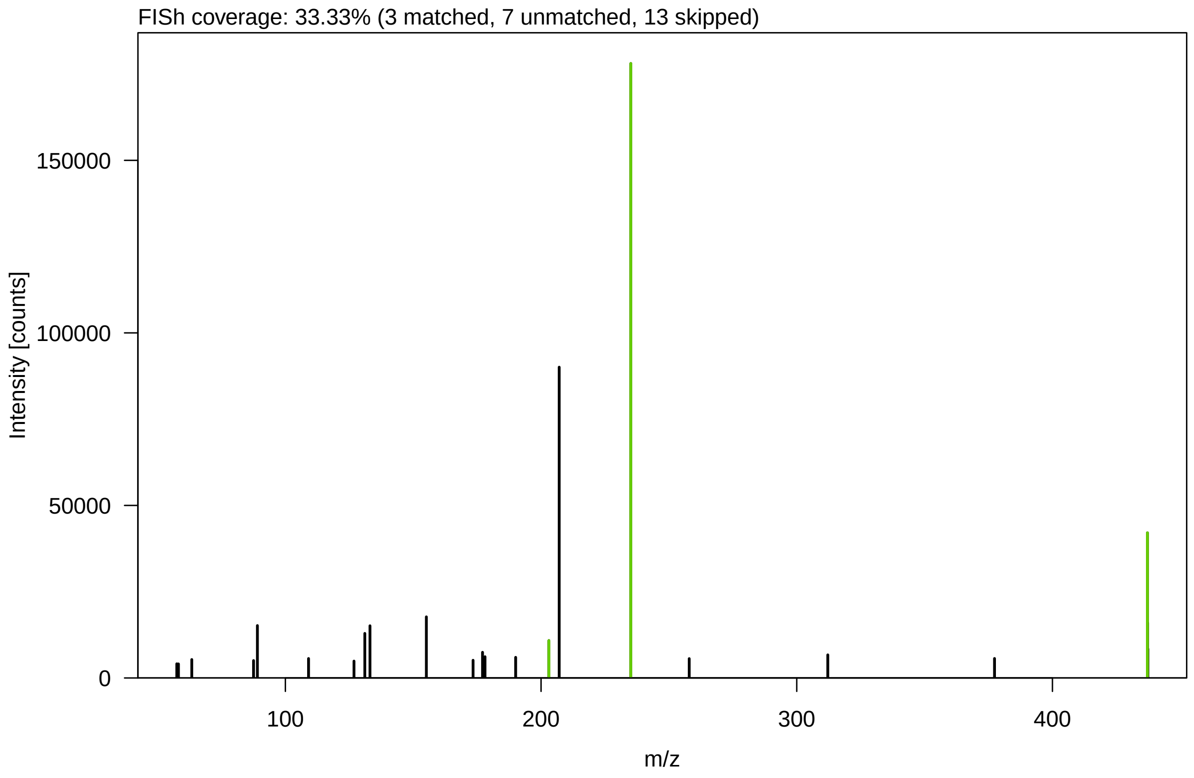

Figure SI-D199: Measured MS2 spectrum. Matching fragments with losartan carboxylic acid predicted by FISh Scoring are highlighted in green. Low intensity fragments are not considered and skipped.

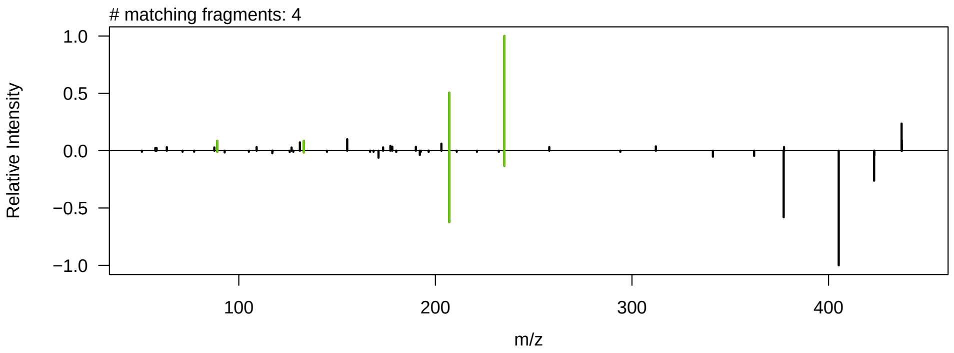

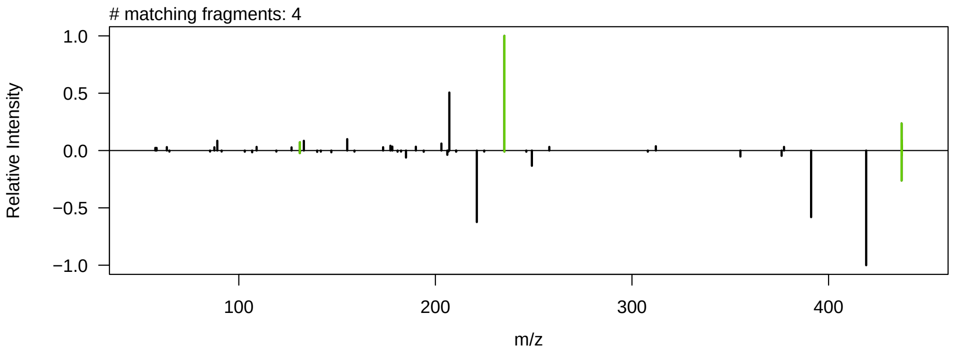

Figure SI-D200: Head to tail plots of losartan carboxylic acid and losartan. In the bottom plot, the mass spectrum of losartan is shifted by the mass difference. Matching fragments are highlighted in green.

Table SI-D97: Molecular network results and retention time prediction of losartan carboxylic acid.

Comparison with	Losartan
MSn Score	36
Forward coverage	28
Reverse coverage	44
Forward match	7
Reverse match	8
ΔMass [g/mol]	13.9793
Measured retention time [min]	19.9
Predicted $logD_{OW}$ (pH = 2.7)	5.2
Predicted retention time [min]	21.5
Predicted retention time range (95% confidence interval) [min]	16.8-26.1
Predicted retention time range (99% confidence interval) [min]	15.3-27.5

Table SI-D98: Annotated MS2 spectrum of losartan carboxylic acid.

m/z	Relative Intensity	Annotation
57.5338	23.02	
58.1859	22.86	
63.3955	29.98	
87.5605	28.33	
89.0594	85.20	$C_2H_6N_3O + H^+$
109.0636	31.64	$C_5H_6N_3 + H^+$
126.8336	27.44	
131.0702	72.44	$C_4H_8N_3O_2 + H^+$
133.0859	84.86	$C_4H_{10}N_3O_2 + H^+$
155.1428	99.38	
173.4047	28.78	
177.1121	41.73	
178.0783	34.57	$C_{14}H_9 + H^+$
190.0655	33.54	$C_{14}H_7N + H^+$
203.0582	60.83	$C_8H_{11}ClN_2O_2 + H^+$
207.0916	505.30	$C_{14}C_{10}N_2 + H^+$
235.0977	999.00	$C_8H_{15}ClN_4O_2 + H^+$
257.9441	31.46	
312.1437	37.56	$C_{16}H_{17}N_5O_2 + H^+$
377.3419	31.70	
437.1473	236.05	$C_{22}H_{21}ClN_6O_2 + H^+$
437.2230	89.66	
437.3049	47.34	

The human liver S9 incubation of losartan led to the formation of losartan carboxylic acid. Considering the spectral match of 0.637 (see Figure SI-D201) and the retention times of 19.9 and 20.1 minutes in the wastewater and the human liver S9 sample, respectively, further confidence could be gained that the detected feature in wastewater is losartan carboxylic acid. Due to this diagnostic evidence, the confidence level can be increased from level 3 to level 2b.

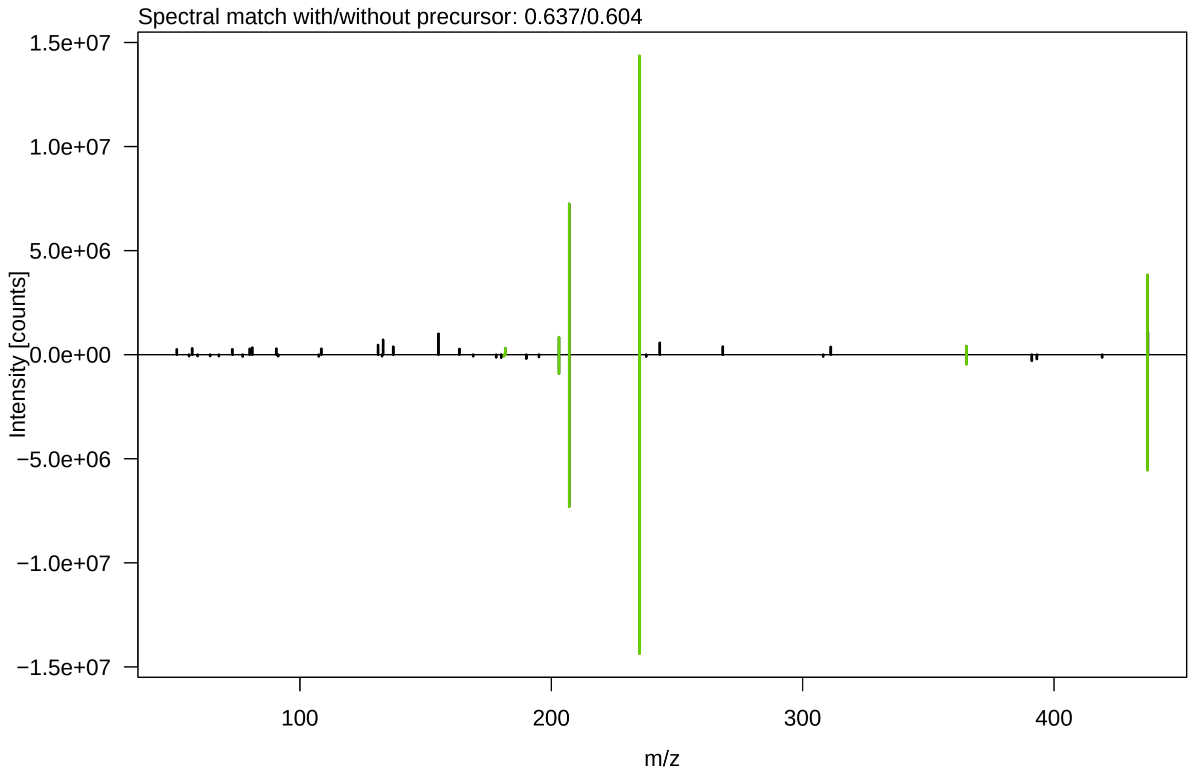

Figure SI-D201: Head to tail plot of losartan carboxylic acid in wastewater (top) and from human liver S9 incubation (bottom). Matching fragments are highlighted in green.

In addition to the human liver S9 incubation experiment, a reference standard of losartan carboxylic acid was purchased. Figure SI-D202 shows the extracted ion chromatograms of this standard, the sample and the spiked sample, as well as a head to tail plot of the MS2 spectra of the standard and the sample. In addition, the most intense MS2 fragments in the sample and in the standard are displayed. It becomes visible that the retention times of the sample and the spiked sample are identical and the spectra similarity score between sample and standard is equal to 0.855. The majority of the MS2 fragments in the sample can be explained by the reference standard. It can therefore be concluded that the suspected compound is indeed losartan carboxylic acid. Correspondingly, the identification confidence can be increased to level 1.

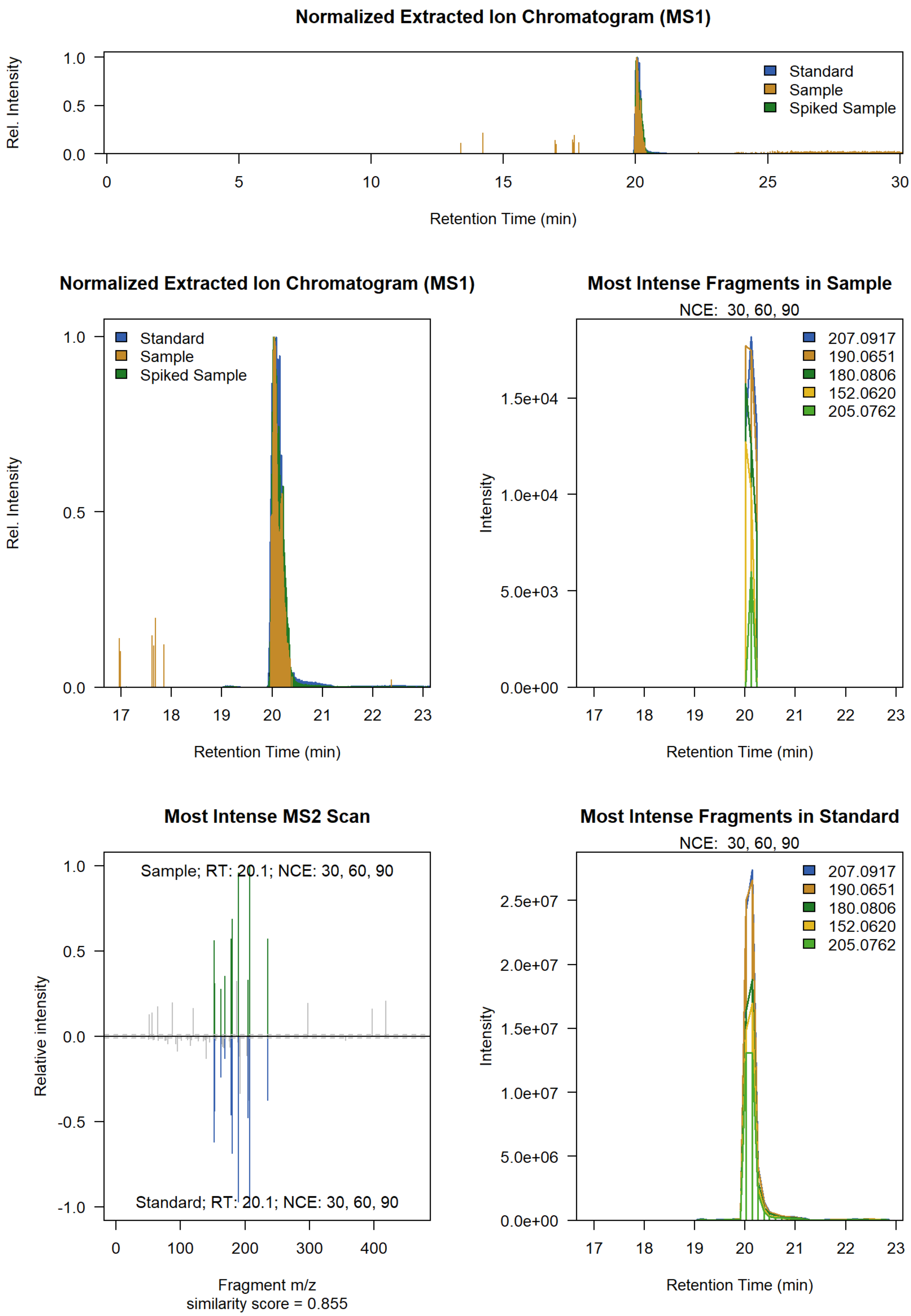

Figure SI-D202: Extracted ion chromatograms of losartan carboxylic acid in the reference standard, the sample and the spiked sample, as well as MS2 head to tail plot and most intense MS2 fragments in standard and sample.

SI-D2.9.2 Losartan Metabolite M2

Table SI-D99: Information on identifiers, chemical properties, detection and confidence of identification of losartan metabolite M2.

IUPAC Name	4-[4-chloro-5-(hydroxymethyl)-1-[[4-[2-(2*H*-tetrazol-5-yl)phenyl]phenyl]methyl]imidazol-2-yl]butan-2-ol
Molecular formula	$C_{22}H_{23}ClN_6O_2$
Monoisotopic mass [g/mol]	438.1571
Adduct	$[M+H]^+$
Retention time [min]	17.3
SMILES	CC(CCC1=NC(=C(N1CC2=CC=C(C=C2)C3=CC=CC=C3C4=NNN=N4)CO)Cl)O
InChI	InChI=1S/C22H23ClN6O2/c1-14(31)6-11-20-24-21(23)19(13-30)29(20)12-15-7-9-16(10-8-15)17-4-2-3-5-18(17)22-25-27-28-26-22/h2-5,7-10,14,30-31H,6,11-13H2,1H3,(H,25,26,27,28)
InChI-Key	RPKQQYYBEFCTLZ-UHFFFAOYSA-N
CAS RN	141675-57-2
Metabolite of	Losartan
Detection frequency	100% (15/15 samples)
Detected in	Altenrhein, Monday-Friday<br>Neugut, Monday-Friday<br>Werdhölzli, Monday-Friday
Intensity	E6-E7
Initial confidence level	level 3
Initial confidence score	0.41
Final confidence level	level 2b

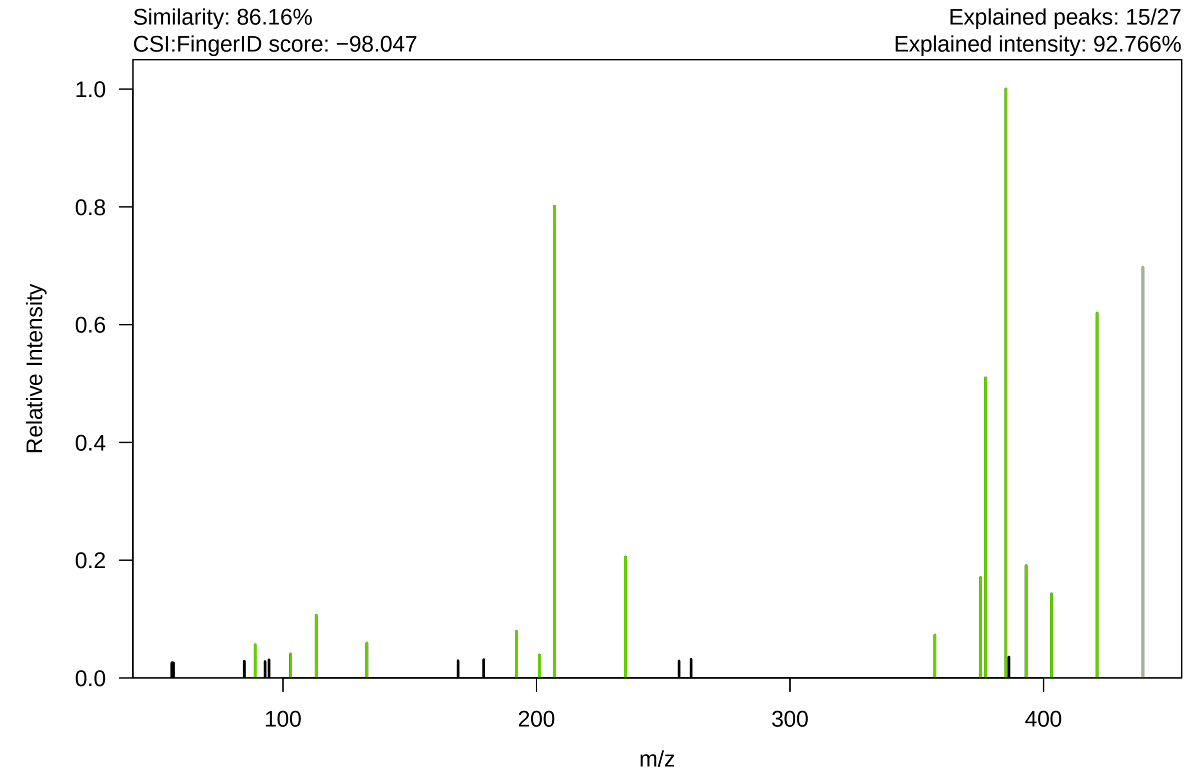

Figure SI-D203: Measured MS2 spectrum. Matching fragments with losartan metabolite M2 predicted by SIRIUS/CSI:FingerID are highlighted in green. The molecular ion in gray is not considered.

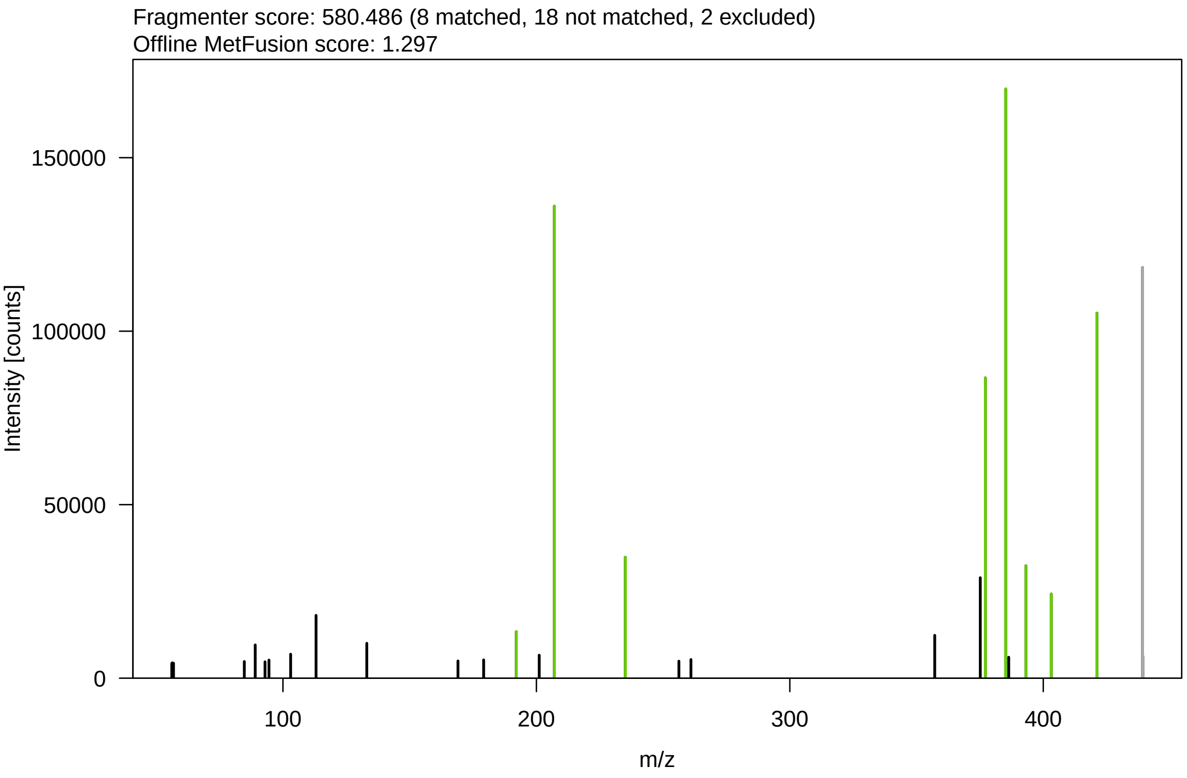

Figure SI-D204: Measured MS2 spectrum. Matching fragments with losartan metabolite M2 predicted by MetFrag are highlighted in green. The molecular ion in gray is not considered.

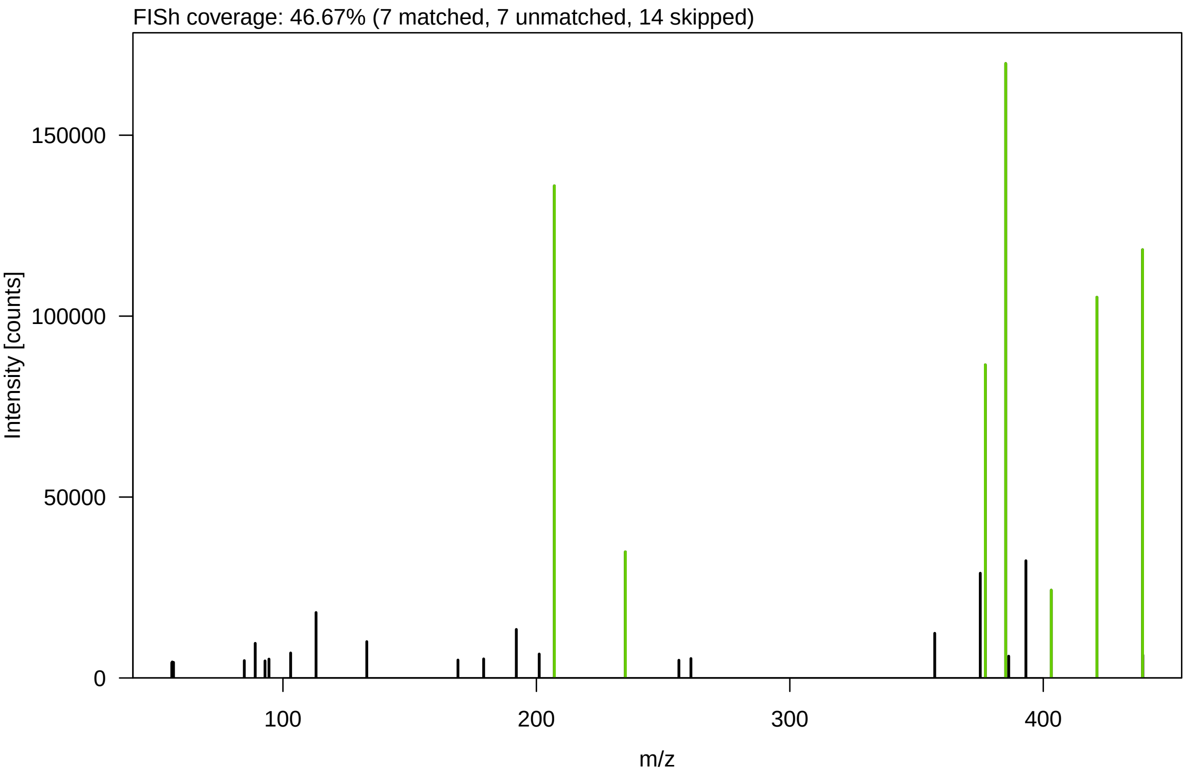

Figure SI-D205: Measured MS2 spectrum. Matching fragments with losartan metabolite M2 predicted by FISh Scoring are highlighted in green. Low intensity fragments are not considered and skipped.

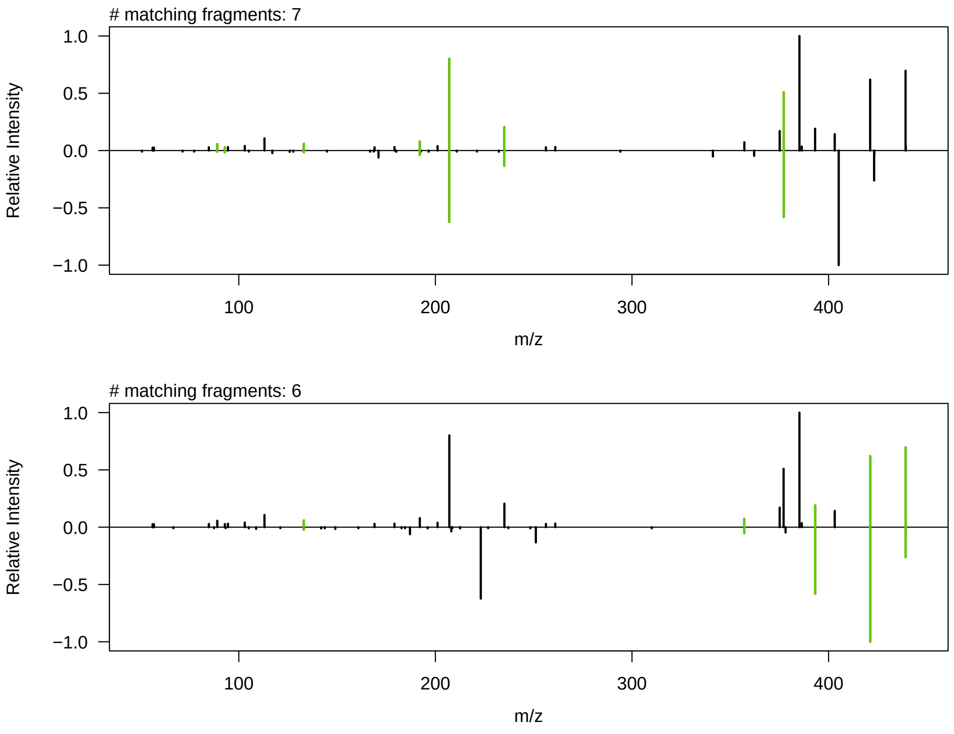

Figure SI-D206: Head to tail plots of losartan metabolite M2 and losartan. In the bottom plot, the mass spectrum of losartan is shifted by the mass difference. Matching fragments are highlighted in green.

Table SI-D100: Molecular network results and retention time prediction of losartan metabolite M2.

Comparison with	Losartan
MSn Score	52
Forward coverage	60
Reverse coverage	43
Forward match	15
Reverse match	13
ΔMass [g/mol]	15.9949
Measured retention time [min]	17.2
Predicted $logD_{OW}$ (pH = 2.7)	2.59
Predicted retention time [min]	18.1
Predicted retention time range (95% confidence interval) [min]	13.5-22.7
Predicted retention time range (99% confidence interval) [min]	12.0-24.2

Table SI-D101: Annotated MS2 spectrum of losartan metabolite M2.

m/z	Relative Intensity	Annotation
56.1407	24.79	
56.3282	26.03	
56.8203	25.40	
84.7598	28.22	
89.0595	56.34	
92.9197	27.74	
94.4807	30.68	
103.0391	40.63	
113.0594	106.42	
133.0858	59.12	
169.0530	29.13	
179.1946	31.00	
192.0812	78.91	$C_{14}H_9N + H^+$
201.1123	38.95	
207.0916	800.18	$C_{14}H_{10}N_2 + H^+$
235.0977	205.09	$C_{14}H_{10}N_4 + H^+$
256.2302	28.83	
260.9761	31.59	
357.1687	72.62	$C_{22}H_{20}N_4O + H^+$
375.1368	170.34	$C_{22}H_{19}ClN_4 + H^+$
377.1277	509.17	$C_{20}H_{17}ClN_6 + H^+$
385.1766	999.00	$C_{22}H_{20}N_6O + H^+$
386.3573	35.46	

Continued on next page

Table SI-D101: Annotated MS2 spectrum of losartan metabolite M2.(Continued)

393.1487	190.64	$C_{22}H_{21}ClN_4O + H^+$
403.1449	142.81	$C_{22}H_{19}ClN_6 + H^+$
421.1534	618.99	$C_{22}H_{21}ClN_6o + H^+$
439.1636	696.30	$C_{22}H_{23}ClN_6O_2 + H^+$
439.2764	37.37	

Since no reference standard of losartan metabolite M2 is commercially available, an incubation experiment was conducted. The human liver S9 incubation of losartan led to the formation of losartan metabolite M2. Considering the spectral match of 0.827 (see Figure SI-D207) and the retention times of 17.3 and 17.6 minutes in the wastewater and the human liver S9 sample, respectively, further confidence could be gained that the detected feature in wastewater is losartan metabolite M2. Due to this diagnostic evidence, the final confidence level can be increased from level 3 to level 2b.

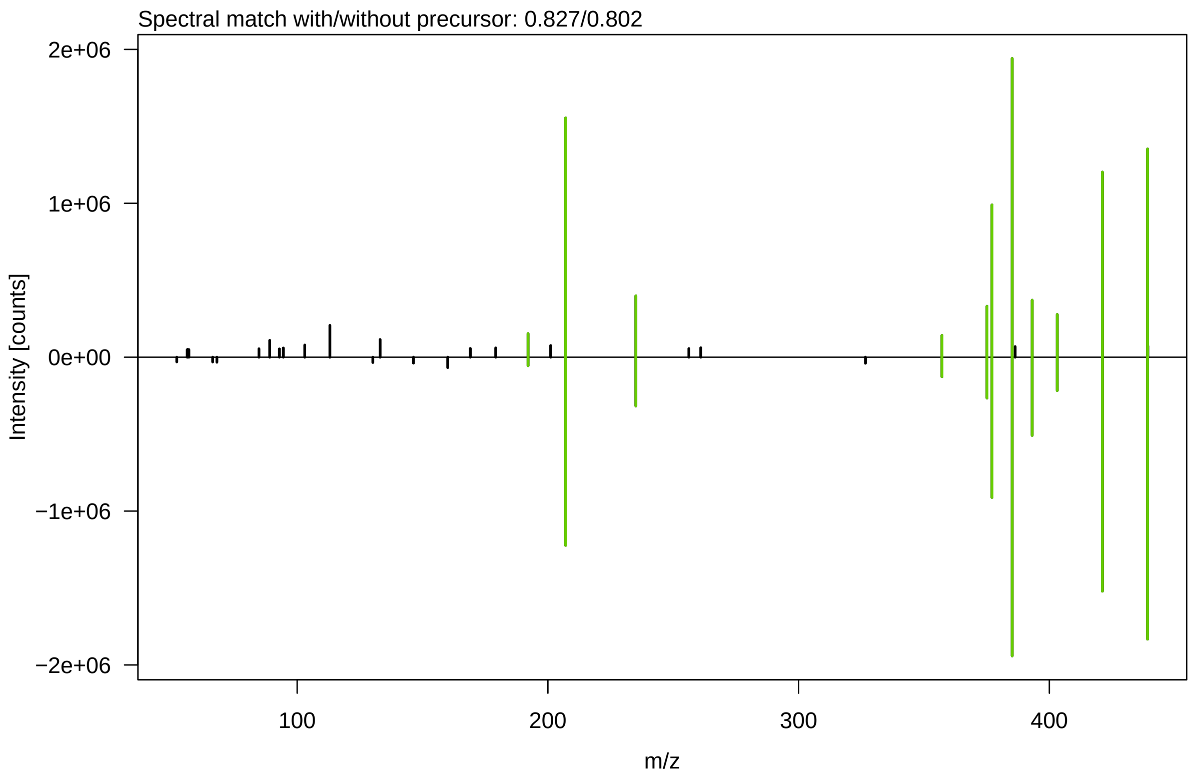

Figure SI-D207: Head to tail plot of losartan metabolite M2 in wastewater (top) and from human liver S9 incubation (bottom). Matching fragments are highlighted in green.

SI-D2.9.3 Losartan Metabolite M5

Table SI-D102: Information on identifiers, chemical properties, detection and confidence of identification of losartan metabolite M5.

IUPAC Name	1-[4-chloro-5-(hydroxymethyl)-1-[[4-[2-(2*H*-tetrazol-5-yl)phenyl]phenyl]methyl]imidazol-2-yl]butan-1-ol
Molecular formula	$C_{22}H_{23}ClN_6O_2$
Monoisotopic mass [g/mol]	438.1571
Adduct	$[M+H]^+$
Retention time [min]	18.4
SMILES	CCCC(C1=NC(=C(N1CC2=CC=C(C=C2)C3=CC=CC=C3C4=NNN=N4)CO)Cl)O
InChI	InChI=1S/C22H23ClN6O2/c1-2-5-19(31)22-24-20(23)18(13-30)29(22)12-14-8-10-15(11-9-14)16-6-3-4-7-17(16)21-25-27-28-26-21/h3-4,6-11,19,30-31H,2,5,12-13H2,1H3,(H,25,26,27,28)
InChI-Key	MGRMJQDIUBITKP-UHFFFAOYSA-N
CAS RN	141675-59-4
Metabolite of	Losartan
Detection frequency	100% (15/15 samples)
Detected in	Altenrhein, Monday-Friday<br>Neugut, Monday-Friday<br>Werdhölzli, Monday-Friday
Intensity	E7
Initial confidence level	level 3
Initial confidence score	0.39
Final confidence level	level 2b

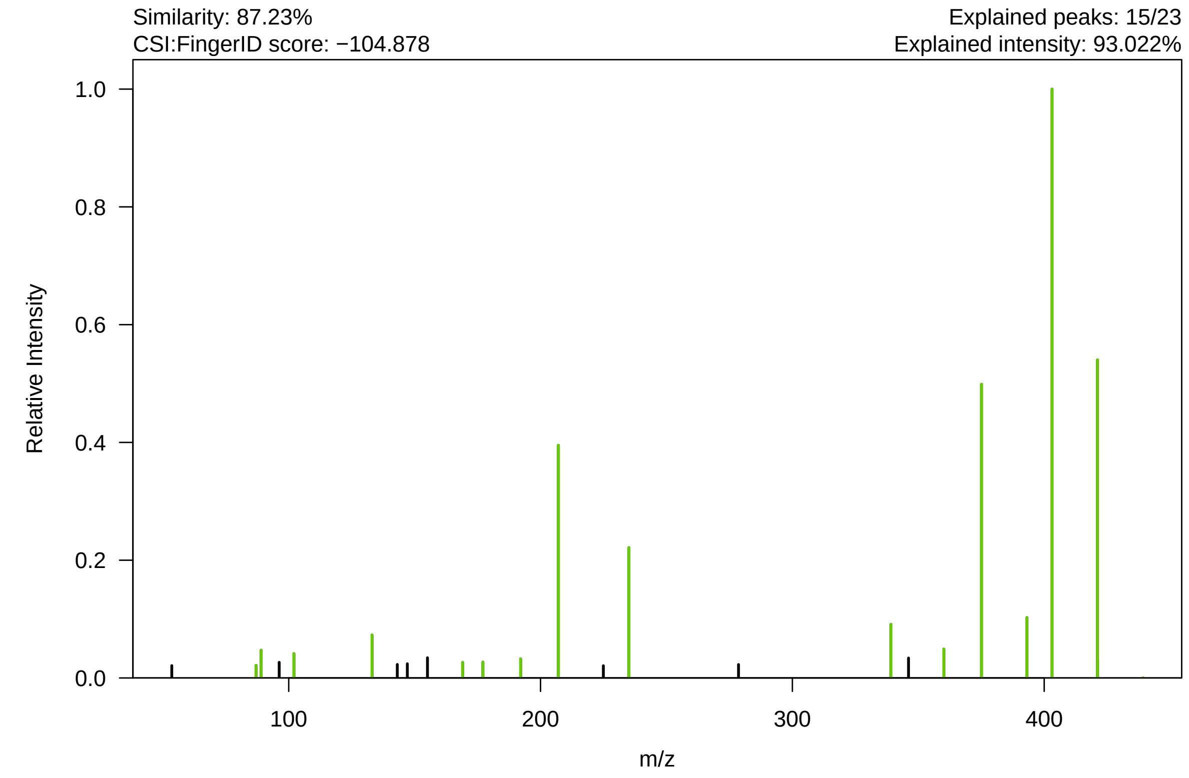

Figure SI-D208: Measured MS2 spectrum. Matching fragments with losartan metabolite M5 predicted by SIRIUS/CSI:FingerID are highlighted in green.

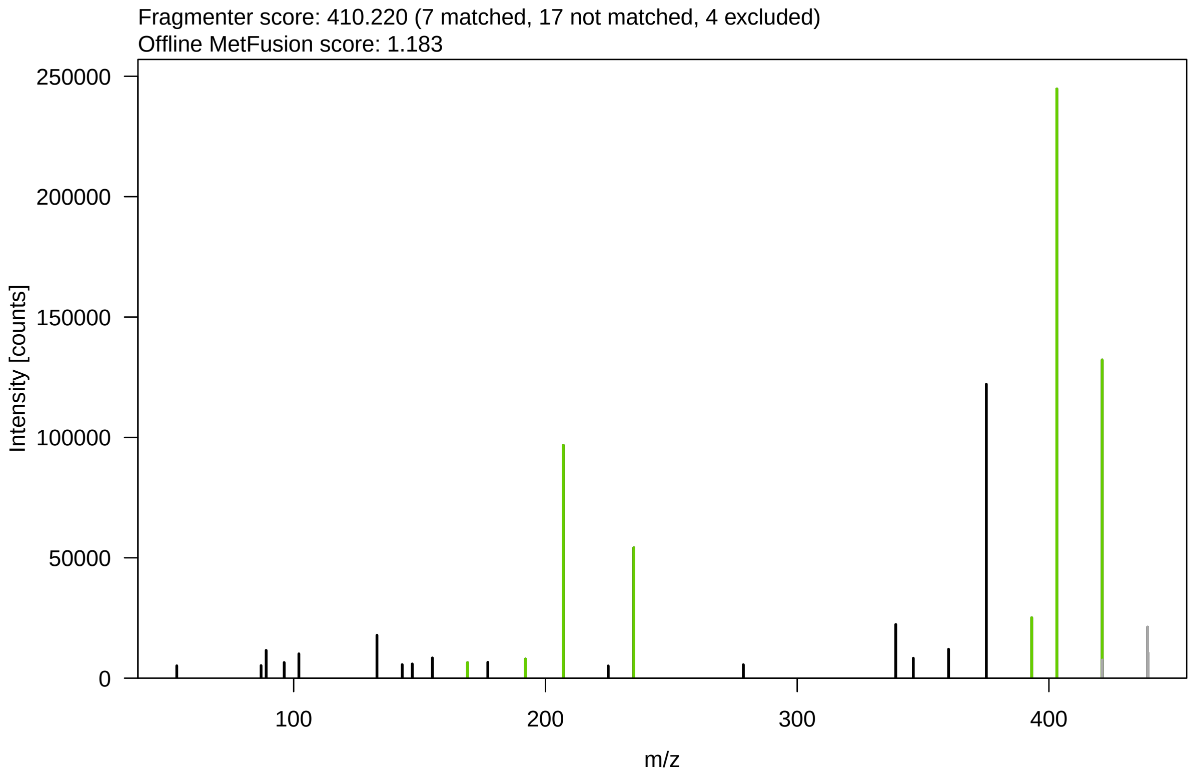

Figure SI-D209: Measured MS2 spectrum. Matching fragments with losartan metabolite M5 predicted by MetFrag are highlighted in green. The molecular ion in gray is not considered.

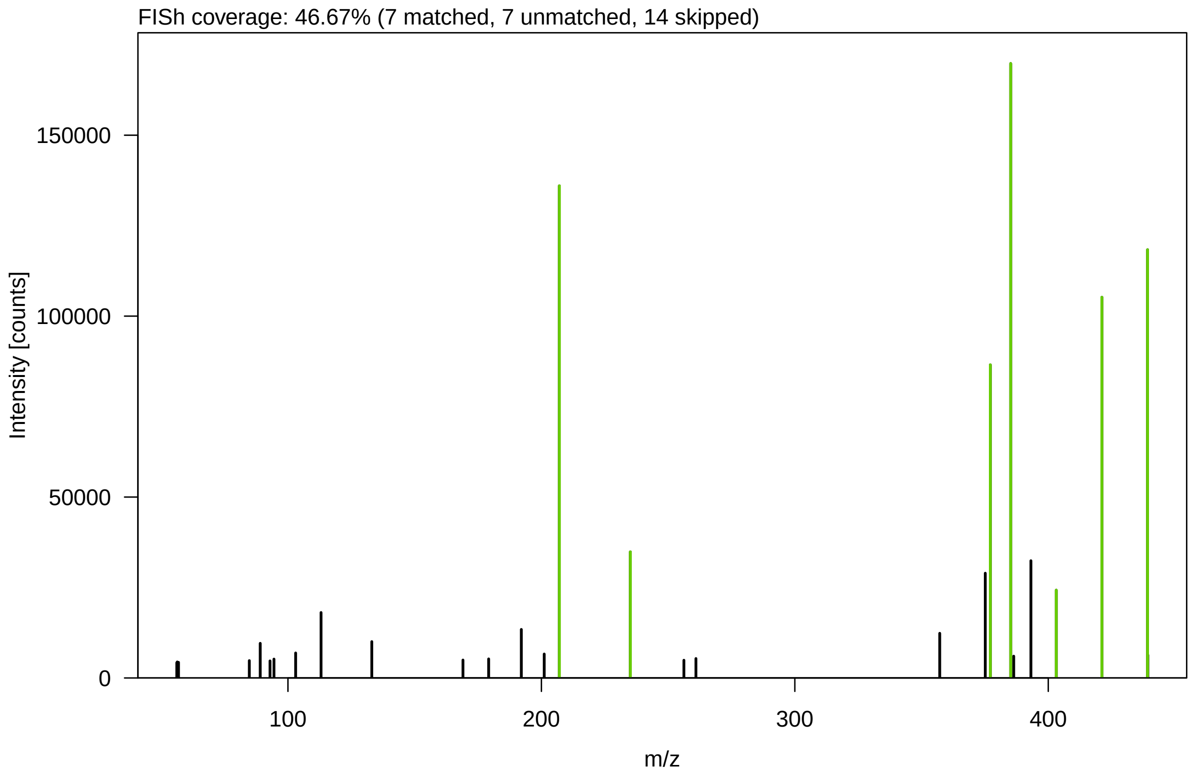

Figure SI-D210: Measured MS2 spectrum. Matching fragments with losartan metabolite M5 predicted by FISh Scoring are highlighted in green. Low intensity fragments are not considered and skipped.

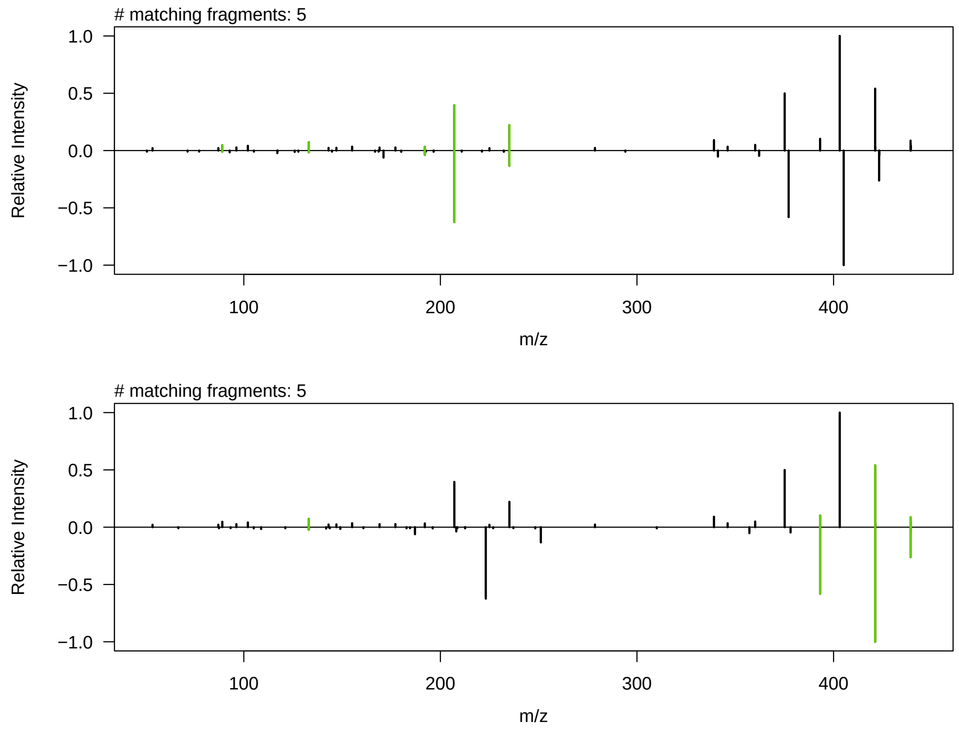

Figure SI-D211: Head to tail plots of losartan metabolite M5 and losartan. In the bottom plot, the mass spectrum of losartan is shifted by the mass difference. Matching fragments are highlighted in green.

Table SI-D103: Molecular network results and retention time prediction of losartan metabolite M5.

Comparison with	Losartan
MSn Score	46
Forward coverage	48
Reverse coverage	44
Forward match	12
Reverse match	11
ΔMass [g/mol]	15.9949
Measured retention time [min]	18.4
Predicted $logD_{OW}$ (pH = 2.7)	4.09
Predicted retention time [min]	20.1
Predicted retention time range (95% confidence interval) [min]	15.5-24.7
Predicted retention time range (99% confidence interval) [min]	14.0-26.1

Table SI-D104: Annotated MS2 spectrum of losartan metabolite M5.

m/z	Relative Intensity	Annotation
53.5676	21.01	
87.0440	21.34	
89.0598	47.12	
96.2239	26.52	
102.0677	41.31	
133.0857	72.87	
143.1158	22.95	
147.1027	24.17	
155.0989	34.35	
169.0526	26.32	$C_9H_4N_4 + H^+$
177.1121	26.96	
192.0814	32.50	$C_{14}H_9N + H^+$
207.0916	394.82	$C_{14}H_{10}N_2 + H^+$
224.9454	20.81	
235.0976	221.10	$C_{14}H_{10}N_4 + H^+$
278.6179	22.84	
339.1605	91.06	$C_{22}H_{18}N_4 + H^+$
346.1240	33.85	
360.1268	49.10	$C_{22}H_{18}ClN_3 + H^+$
375.1364	498.35	$C_{22}H_{19}ClN_4 + H^+$
393.1498	102.48	$C_{22}H_{21}ClN_4O + H^+$
403.1429	999.00	$C_{22}H_{19}ClN_6 + H^+$
421.1535	539.59	$C_{22}H_{21}ClN_6O + H^+$

Continued on next page

Table SI-D104: Annotated MS2 spectrum of losartan metabolite M5.(Continued)

421.2079	30.83
439.1773	86.53
439.2322	44.00
439.2822	42.87

Since no reference standard of losartan metabolite M5 is commercially available, an incubation experiment was conducted. The human liver S9 incubation of losartan led to the formation of a losartan metabolite M5. Considering the spectral match of 0.789 (see Figure SI-D212) and the retention times of 18.4 and 18.7 minutes in the wastewater and the human liver S9 sample, respectively, further confidence could be gained that the detected feature in wastewater is losartan metabolite M5. Due to this diagnostic evidence, the final confidence level can be increased from level 3 to level 2b.

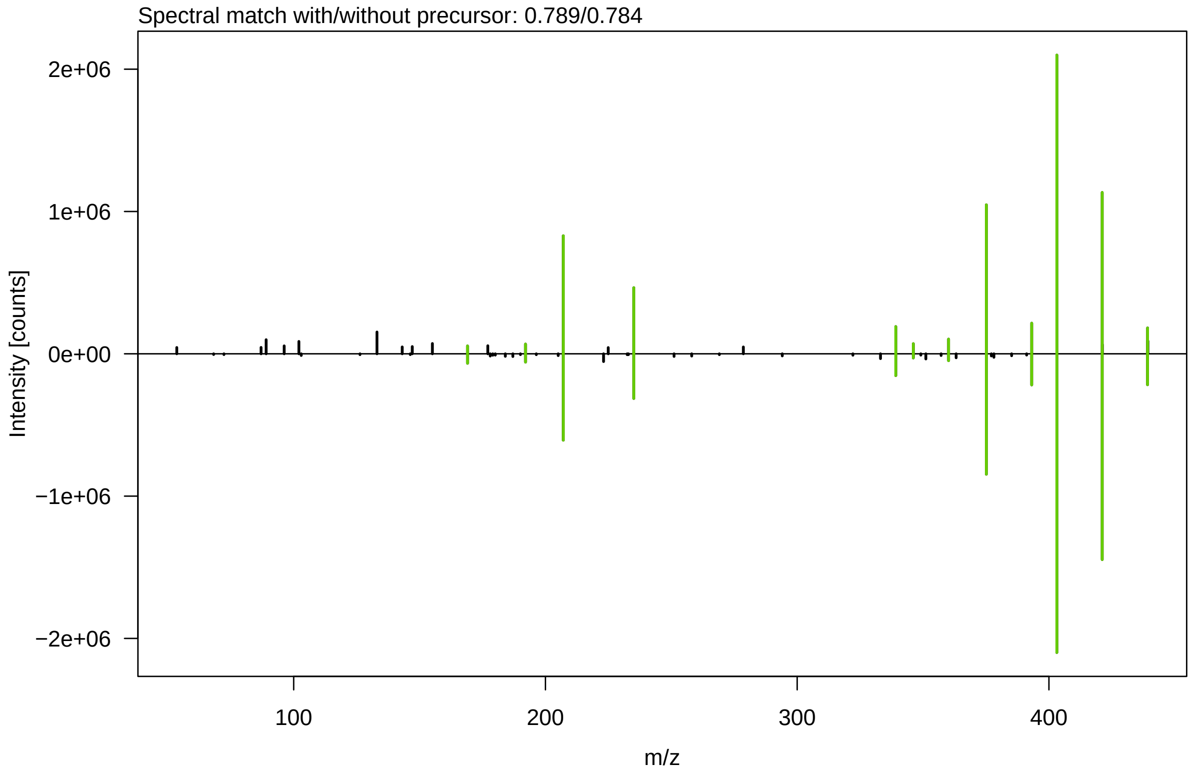

Figure SI-D212: Head to tail plot of losartan metabolite M5 in wastewater (top) and from human liver S9 incubation (bottom). Matching fragments are highlighted in green.

SI-D2.10 Mefenamic Acid Metabolites

Mefenamic acid is nonsteroidal anti-inflammatory drug, which is used to treat mild pain.[2] Suspect screening combined with molecular networking enabled the identification of two metabolites. The identified metabolites are highlighted in the metabolism scheme of mefenamic acid in Figure SI-D213. Excerpts of the molecular networks in the positive and negative ionization mode of the mefenamic acid cluster are shown in Figures SI-D214 and SI-D215. The following subsections give more details on the individual metabolites.

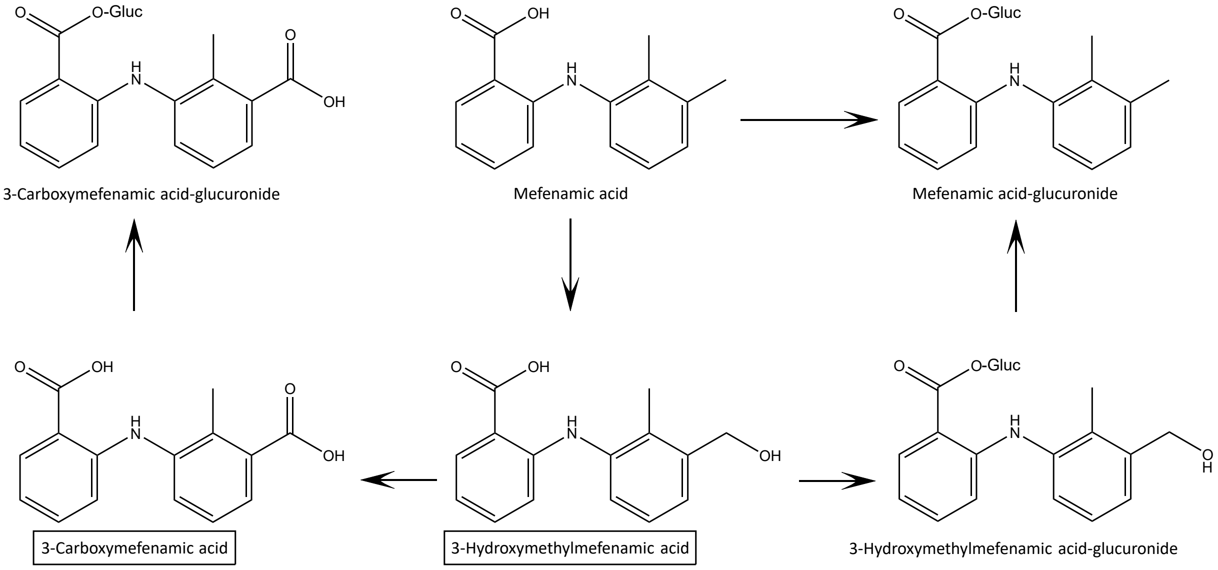

Figure SI-D213: Human metabolism of mefenamic acid. Framed metabolites were identified during suspect screening. Scheme drawn based on information from.[2]

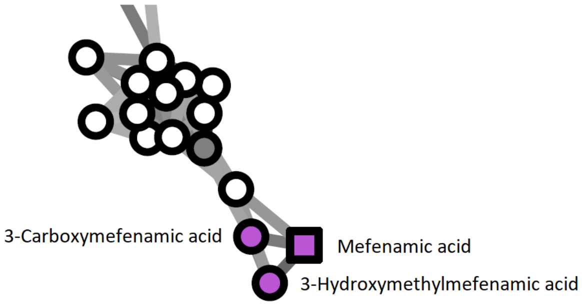

Figure SI-D214: Excerpt of the molecular network showing the mefenamic acid cluster in the positive ionization mode.

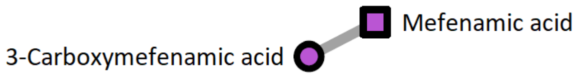

Figure SI-D215: Excerpt of the molecular network showing the mefenamic acid cluster in the negative ionization mode.

SI-D2.10.1 3-Carboxymefenamic Acid

Table SI-D105: Information on identifiers, chemical properties, detection and confidence of identification of 3-carboxymefenamic acid.

IUPAC Name	3-(2-carboxyanilino)-2-methylbenzoic acid
Molecular formula	$C_{15}H_{13}NO_4$
Monoisotopic mass [g/mol]	271.0845
Adduct	$[M+H]^+$
Retention time [min]	20.5
SMILES	CC1=C(C=CC=C1NC2=CC=CC=C2C(=O)O)C(=O)O
InChI	InChI=1S/C15H13NO4/c1-9-10(14(17)18)6-4-8-12(9)16-13-7-3-2-5-11(13)15(19)20/h2-8,16H,1H3,(H,17,18)(H,19,20)
InChI-Key	OOQQWHSTKBWQPB-UHFFFAOYSA-N
CAS RN	190379-82-9
Metabolite of	Mefenamic acid
Detection frequency	100% (15/15 samples)
Detected in	Altenrhein, Monday-Friday
	Neugut, Monday-Friday
	Werdhölzli, Monday-Friday
Intensity	E7-E8
Initial confidence level	level 3
Initial confidence score	0.49
Final confidence level	level 2b

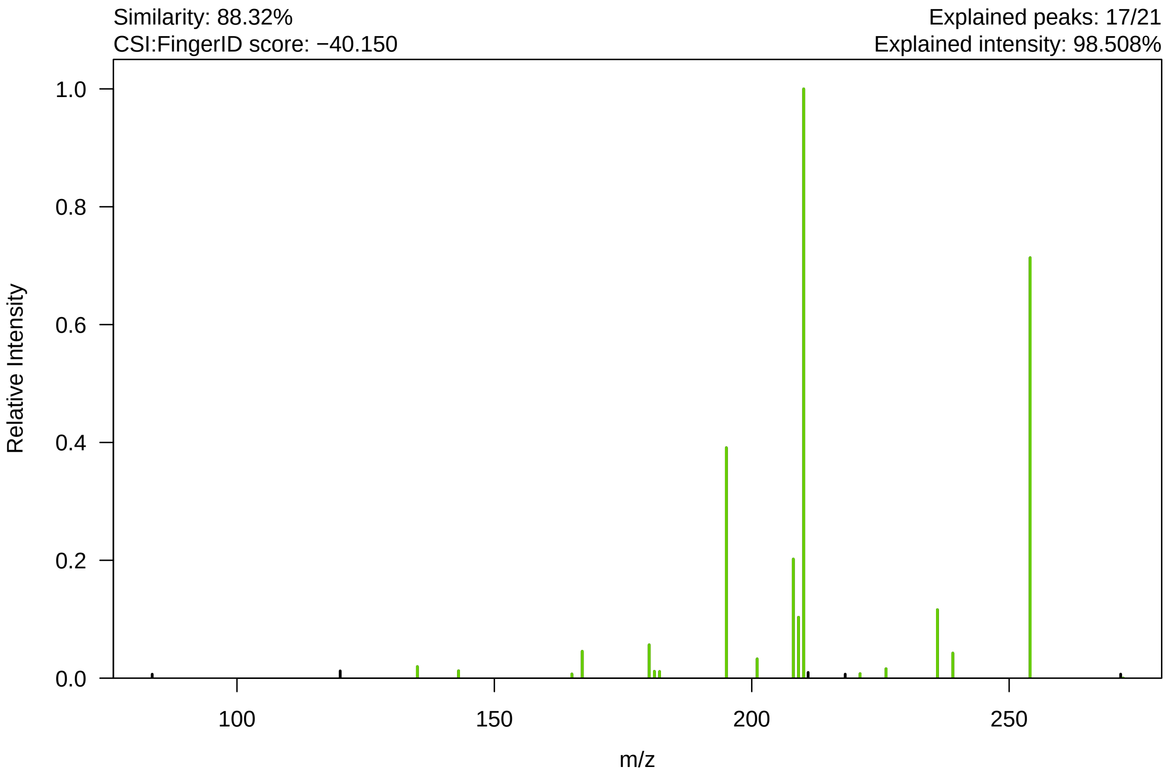

Figure SI-D216: Measured MS2 spectrum. Matching fragments with 3-carboxymefenamic acid predicted by SIRIUS/CSI:FingerID are highlighted in green.

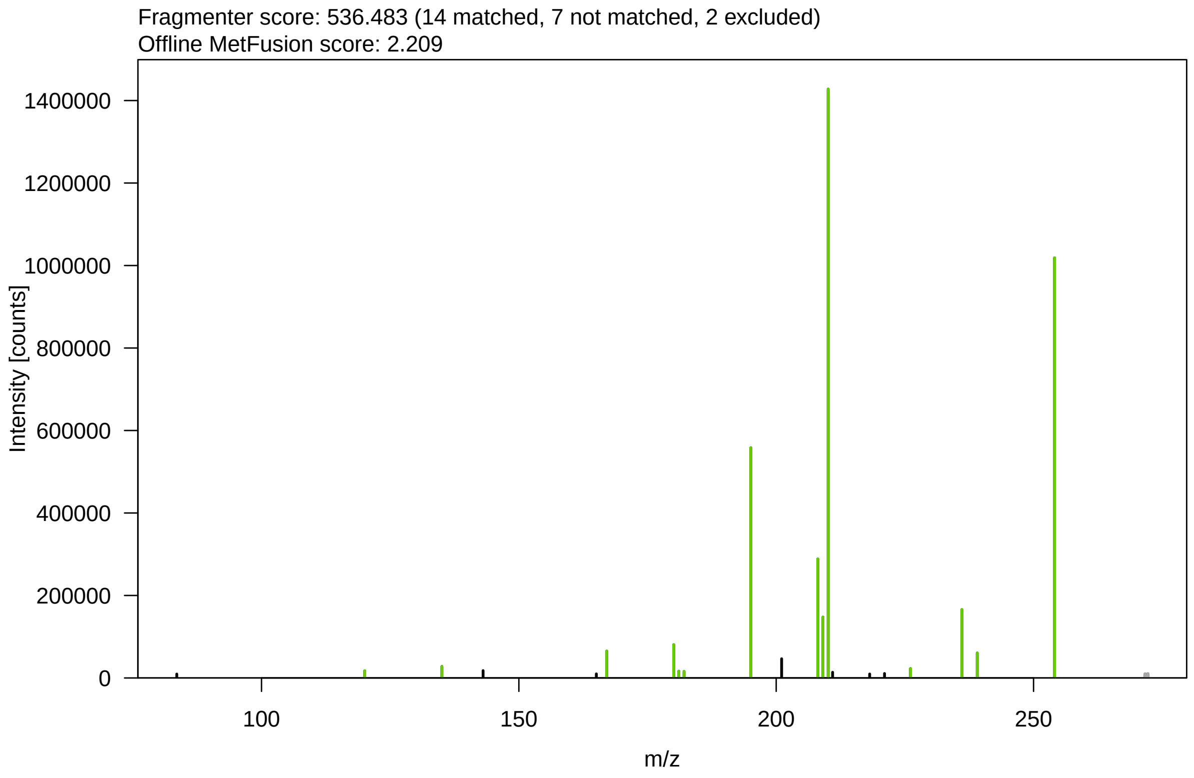

Figure SI-D217: Measured MS2 spectrum. Matching fragments with 3-carboxymefenamic acid predicted by MetFrag are highlighted in green. The molecular ion in gray is not considered.

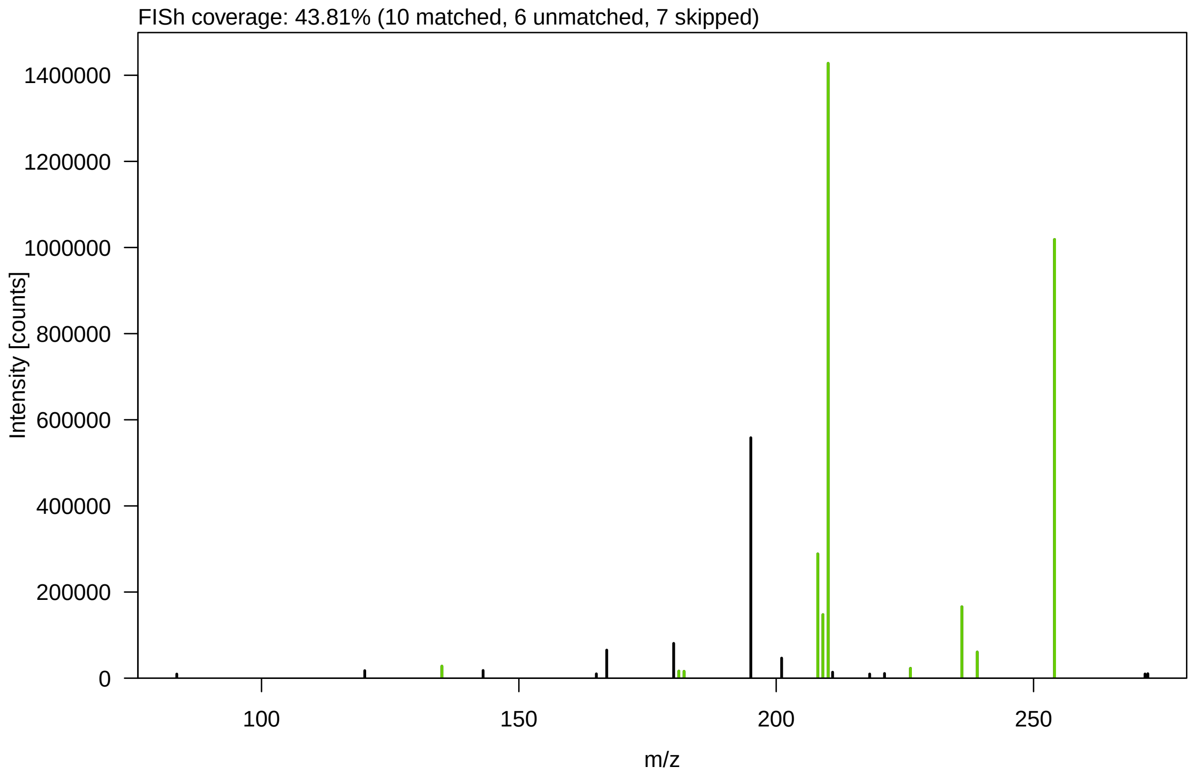

Figure SI-D218: Measured MS2 spectrum. Matching fragments with 3-carboxymefenamic acid predicted by FISh Scoring are highlighted in green. Low intensity fragments are not considered and skipped.

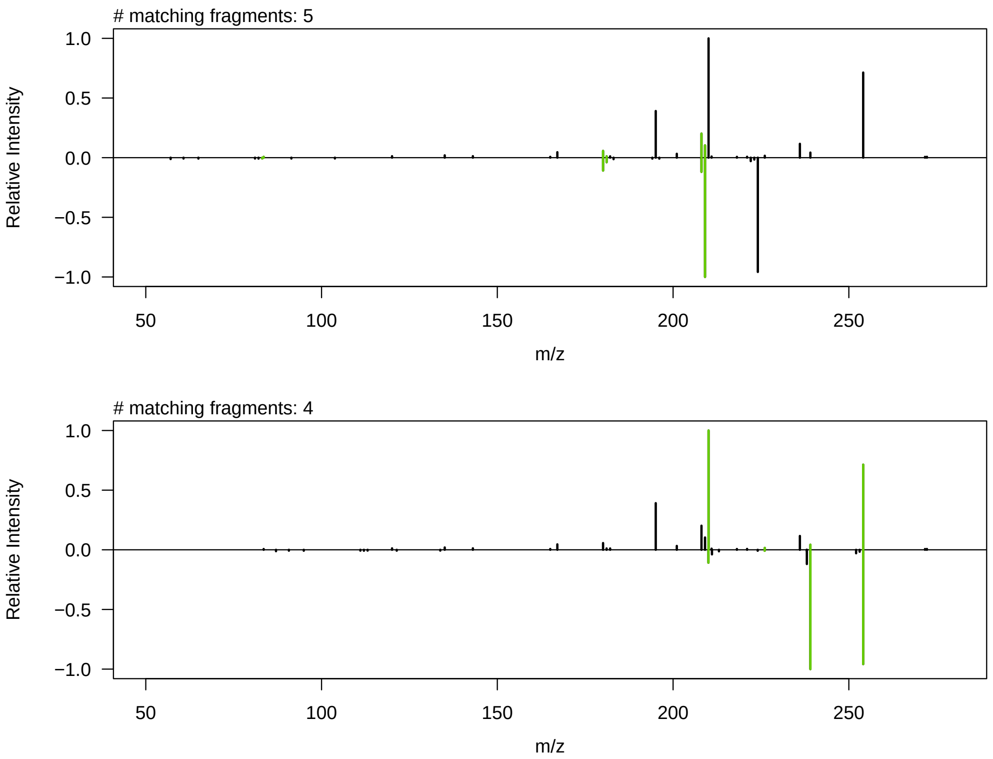

Figure SI-D219: Head to tail plots of 3-carboxymefenamic acid and mefenamic acid. In the bottom plot, the mass spectrum of mefenamic acid is shifted by the mass difference. Matching fragments are highlighted in green.

Table SI-D106: Molecular network results and retention time prediction of 3-carboxymefenamic acid.

Comparison with	Mefenamic acid
MSn Score	53
Forward coverage	71
Reverse coverage	34
Forward match	20
Reverse match	36
ΔMass [g/mol]	29.9742
Measured retention time [min]	20.5
Predicted $\log D_{OW}$ (pH = 2.7)	4.51
Predicted retention time [min]	20.6
Predicted retention time range (95% confidence interval) [min]	16.0-25.2
Predicted retention time range (99% confidence interval) [min]	14.5-26.7

Table SI-D107: Annotated MS2 spectrum of 3-carboxymefenamic acid.

m/z	Relative Intensity	Annotation
83.5349	6.68	
120.0555	12.24	$C_8H_7O + H^+$
135.0440	19.55	$C_8H_6O_2 + H^+$
143.0491	12.47	$C_{10}H_6O + H^+$
165.0555	7.00	$C_{12}H_6N + H^+$
167.0727	45.61	$C_{12}H_8N + H^+$
180.0808	56.51	$C_{13}H_9N + H^+$
181.0883	11.48	$C_{13}H_{10}N + H^+$
182.0961	11.18	$C_{13}H_{11}N + H^+$
195.0679	390.65	$C_{13}H_8NO + H^+$
201.0555	32.59	$C_{12}H_8O_3 + H^+$
208.0758	201.86	$C_{14}H_9NO + H^+$
209.0836	103.26	$C_{14}H_{19}NO + H^+$
210.0913	999.00	$C_{14}H_{11}NO + H^+$
210.9560	9.70	
218.1575	6.78	
221.0475	7.60	$C_{14}H_6NO_2 + H^+$
226.0863	15.98	$C_{14}H_{11}NO_2 + H^+$
236.0705	116.00	$C_{15}H_9NO + H^+$
239.0573	42.47	$C_{14}H_8NO_3 + H^+$
254.0810	712.76	$C_{15}H_{11}NO_3 + H^+$
271.6660	6.80	
272.2055	7.23	

The commercially available reference standard of 3-carboxymefenamic acid was considered as too expensive. Consequently, an incubation experiment was conducted. The human liver S9 incubation of mefenamic acid led to the formation of 3-carboxymefenamic acid. Considering the spectral match of 0.833 (see Figure SI-D220) and the retention times of 20.5 and 20.8 minutes in the wastewater and the human liver S9 sample, respectively, further confidence could be gained that the detected feature in wastewater is 3-carboxymefenamic acid. Due to this diagnostic evidence, the final confidence level can be increased from level 3 to level 2b.

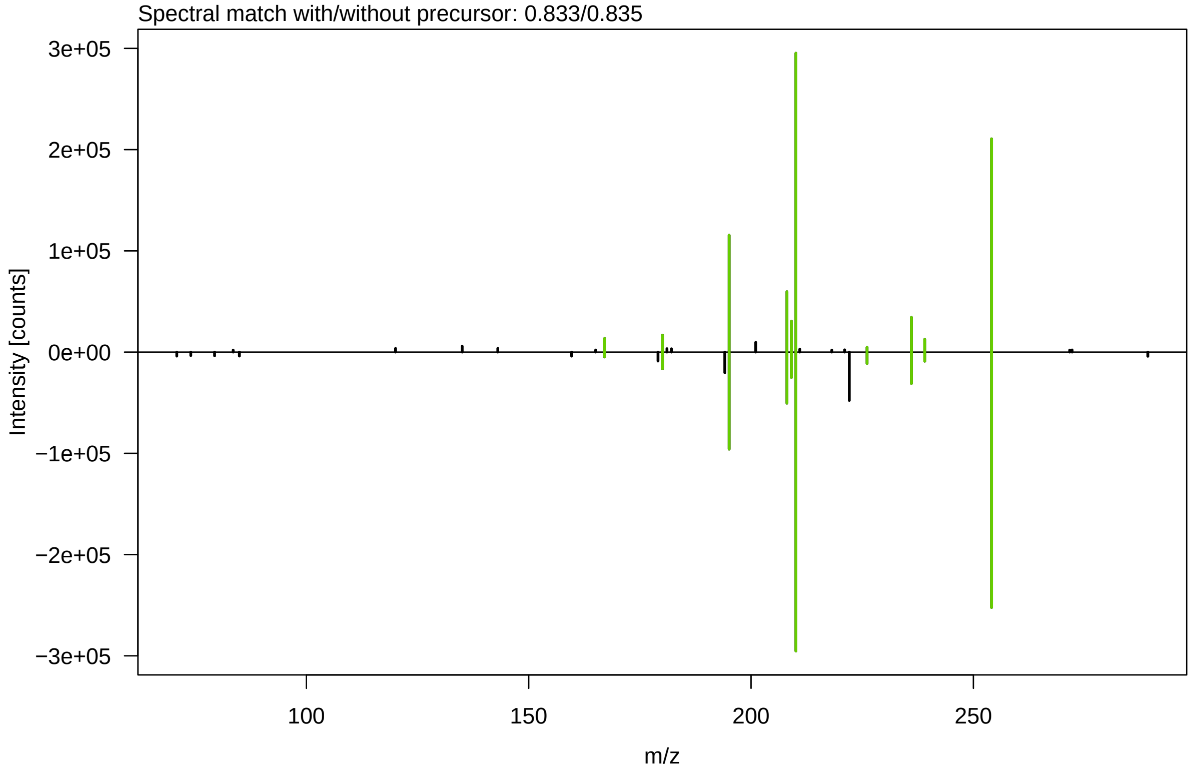

Figure SI-D220: Head to tail plot of 3-carboxymefenamic acid in wastewater (top) and from human liver S9 incubation (bottom). Matching fragments are highlighted in green.

SI-D2.10.2 3-Hydroxymethylmefenamic Acid

Table SI-D108: Information on identifiers, chemical properties, detection and confidence of identification of 3-hydroxymethylmefenamic acid.

IUPAC Name	52-[3-(hydroxymethyl)-2-methylanilino]benzoic acid
Molecular formula	$C_{15}H_{15}NO_3$
Monoisotopic mass [g/mol]	257.1052
Adduct	$[M+H]^+$
Retention time [min]	20.1
SMILES	CC1=C(C=CC=C1NC2=CC=CC=C2C(=O)O)CO
InChI	InChI=1S/C15H15NO3/c1-10-11(9-17)5-4-8-13(10)16-14-7-3-2-6-12(14)15(18)19/h2-8,16-17H,9H2,1H3,(H,18,19)
InChI-Key	QBONJEHEDCBRMZ-UHFFFAOYSA-N
CAS RN	5129-20-4
Metabolite of	Mefenamic acid
Detection frequency	100% (15/15 samples)
Detected in	Altenrhein, Monday-Friday
	Neugut, Monday-Friday
	Werdhölzli, Monday-Friday
Intensity	E7-E8
Initial confidence level	level 3
Initial confidence score	0.49
Final confidence level	level 1

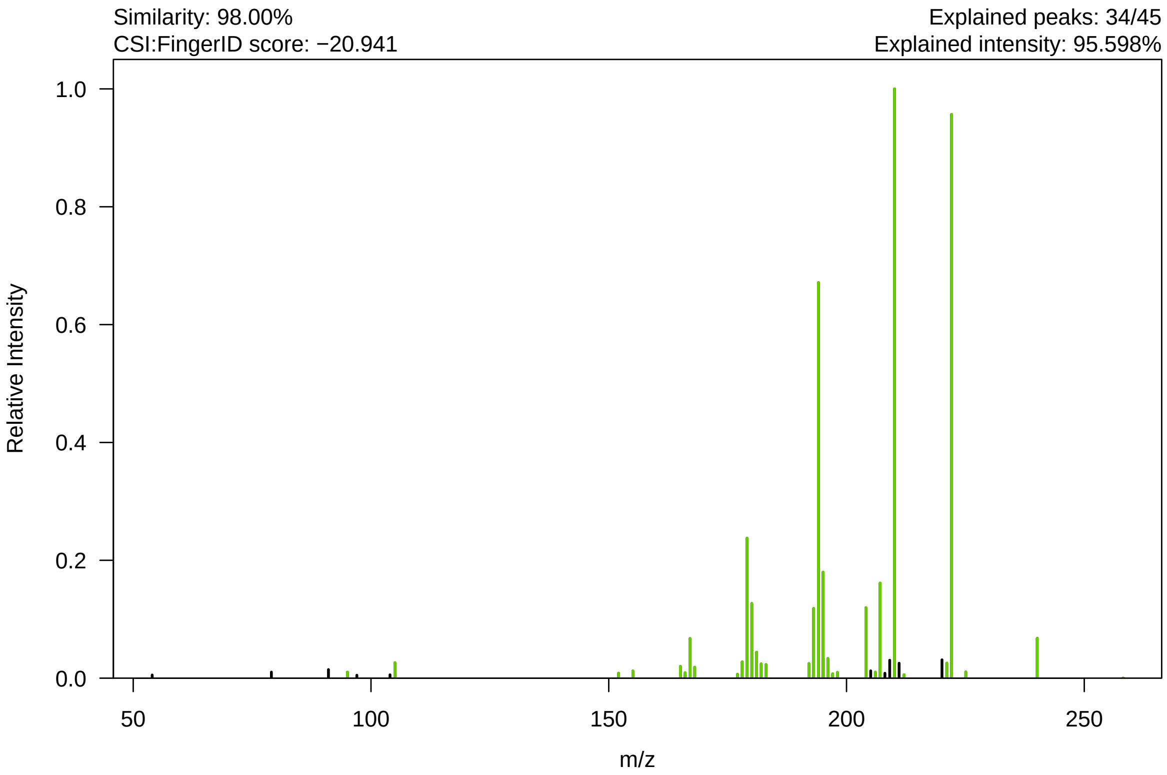

Figure SI-D221: Measured MS2 spectrum. Matching fragments with 3-hydroxymethylmefenamic acid predicted by SIRIUS/CSI:FingerID are highlighted in green.

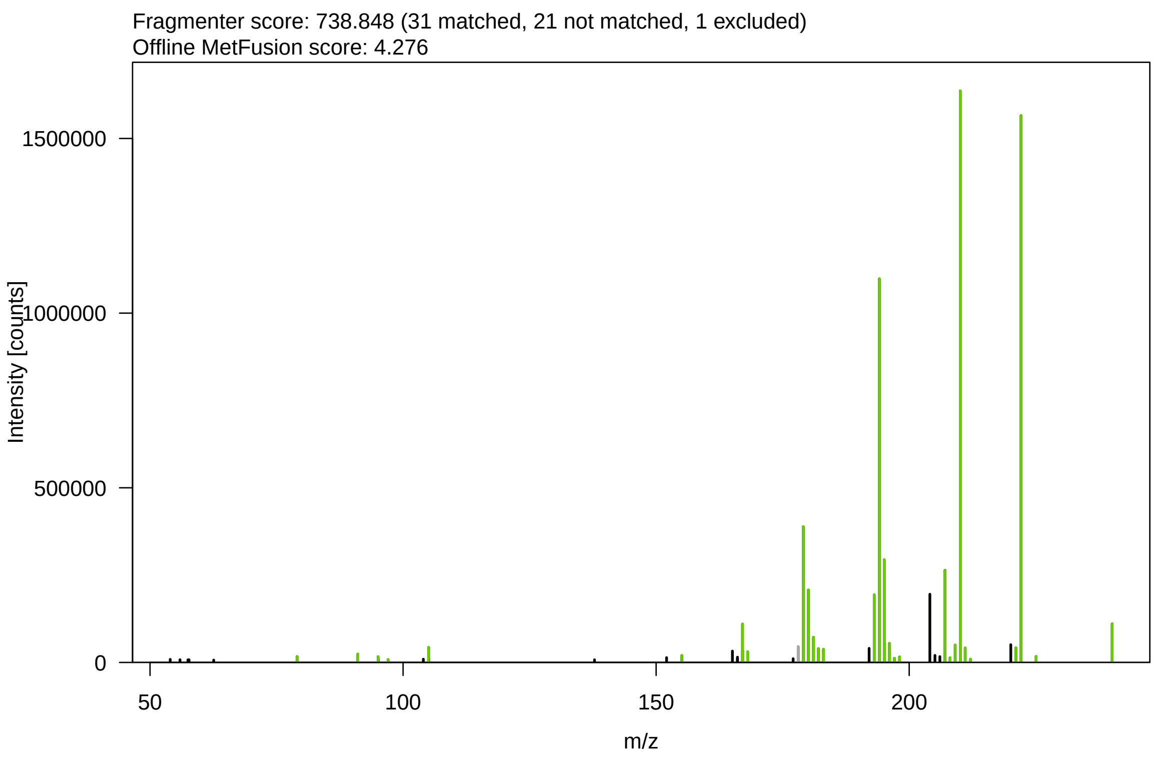

Figure SI-D222: Measured MS2 spectrum. Matching fragments with 3-hydroxymethylmefenamic acid predicted by MetFrag are highlighted in green.

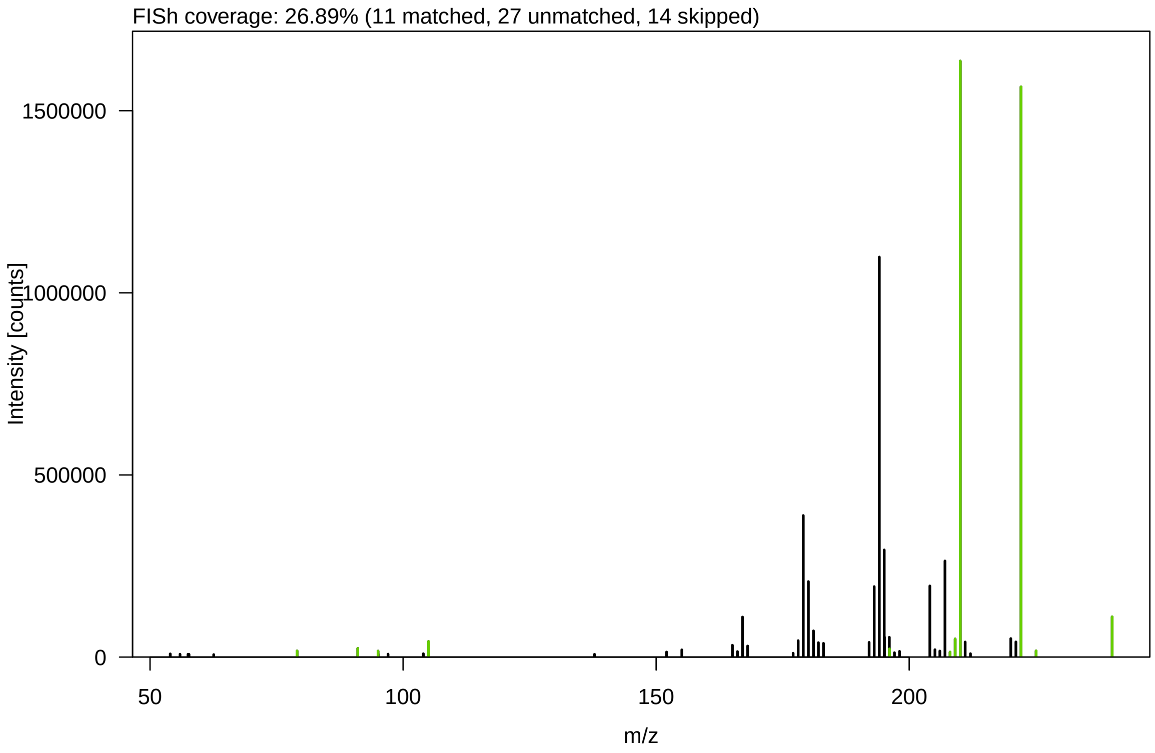

Figure SI-D223: Measured MS2 spectrum. Matching fragments with 3-hydroxymethylmefenamic acid predicted by FISh Scoring are highlighted in green. Low intensity fragments are not considered and skipped.

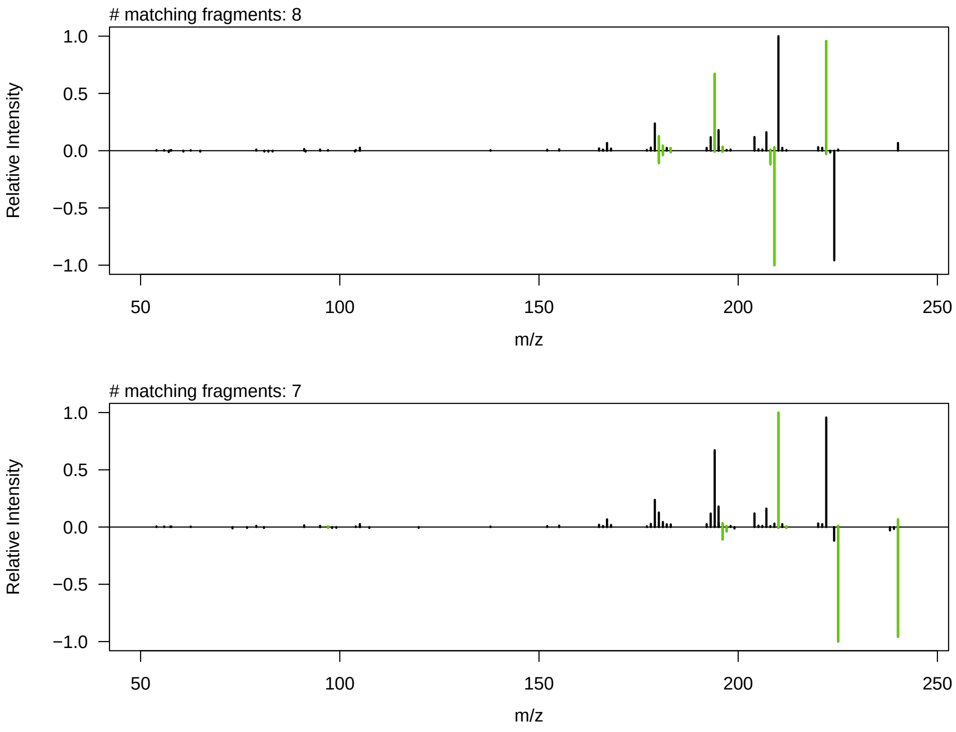

Figure SI-D224: Head to tail plots of 3-hydroxymethylmefenamic acid and mefenamic acid. In the bottom plot, the mass spectrum of mefenamic acid is shifted by the mass difference. Matching fragments are highlighted in green.

Table SI-D109: Molecular network results and retention time prediction of 3-hydroxymethylmefenamic acid.

Comparison with	Mefenamic acid
MSn Score	60
Forward coverage	82
Reverse coverage	38
Forward match	23
Reverse match	45
ΔMass [g/mol]	15.9949
Measured retention time [min]	20.1
Predicted $\log D_{OW}$ (pH = 2.7)	4.09
Predicted retention time [min]	20.1
Predicted retention time range (95% confidence interval) [min]	15.5-24.7
Predicted retention time range (99% confidence interval) [min]	14.0-26.1

Table SI-D110: Annotated MS2 spectrum of 3-hydroxymethylmefenamic acid.

m/z	Relative Intensity	Annotation
53.9916	5.40	
55.9256	4.83	
57.4905	4.64	
57.7071	4.67	
62.5823	4.20	
79.0542	10.34	$C_6H_6 + H^+$
91.0543	14.62	$C_7H_6 + H^+$
95.0489	10.11	$C_6H_6O + H^+$
95.0762	4.92	
97.0280	5.02	$C_5H_4O_2$ + H≡;
104.0023	5.67	
105.0335	26.16	$C_7H_4O + H^+$
105.0443	6.78	
137.8240	4.67	
152.0621	8.46	$C_{12}H_7 + H^+$
155.0731	12.13	$C_{11}H_8N + H^+$
165.0701	19.90	$C_{13}H_8 + H^+$
166.0643	9.25	$C_{12}H_7N + H^+$
166.0779	6.55	$C_{13}H_9 + H^+$
167.0728	67.11	$C_{12}H_8N + H^+$
167.0856	20.60	$C_{13}H_{10} + H^+$
168.0808	18.59	$C_{12}H_9N + H^+$
177.0696	6.56	$C_{14}H_8 + H^+$
178.0652	27.55	$C_{13}H_7N + H^+$
179.0727	237.25	$C_{13}H_8N + H^+$
180.0804	126.57	$C_{13}H_9N + H^+$
181.0883	43.97	$C_{13}H_{10}N + H^+$
182.0600	24.19	$C_{12}H_7NO + H^+$
183.0676	23.04	$C_{12}H_8NO + H^+$
192.0814	24.67	$C_{14}H_9N + H^+$
193.0886	118.13	$C_{14}H_{10}N + H^+$
194.0963	670.51	$C_{14}H_{11}N + H^+$
195.0678	179.59	$C_{13}H_8NO + H^+$
195.1046	33.46	$C_{14}H_{12}N + H^+$
196.0757	33.26	$C_{13}H_9NO + H^+$
196.1123	13.83	$C_{14}H_{13}N + H^+$

Continued on next page

Table SI-D110: Annotated MS2 spectrum of 3-hydroxymethylmefenamic acid.(Continued)

197.0837	7.37	$C_{13}H_{10}NO + H^+$
198.0920	9.63	$C_{13}H_{11}No + H^+$
204.0807	119.28	$C_{15}H_9N + H^+$
205.0882	12.38	
206.0597	10.19	$C_{14}H_7NO + H^+$
207.0677	161.15	$C_{14}H_8NO + H^+$
208.0756	8.34	$C_{14}H_9NO + H^+$
209.0835	30.41	$C_{14}H_{10}NO + H^+$
210.0913	999.00	$C_{14}H_{11}NO + H^+$
211.0628	25.31	$C_{13}H_8NO_2 + H^+$
212.1068	5.74	$C_{14}H_{13}NO + H^+$
220.0750	31.06	
221.0833	25.45	$C_{15}H_{10}NO + H^+$
222.0912	955.77	$C_{15}H_{11}NO + H^+$
225.0784	10.44	$C_{14}H_{10}NO_2 + H^+$
240.1018	67.50	$C_{15}H_{13}NO_2 + H^+$

The human liver S9 incubation of mefenamic acid led to the formation of a 3-hydroxymethylmefenamic acid. Considering the spectral match of 0.941 (see Figure SI-D220) and the retention times of 20.1 and 20.4 minutes in the wastewater and the human liver S9 sample, respectively, further confidence could be gained that the detected feature in wastewater is 3-hydroxymethylmefenamic acid. Due to this diagnostic evidence, the confidence level can be increased from level 3 to level 2b.

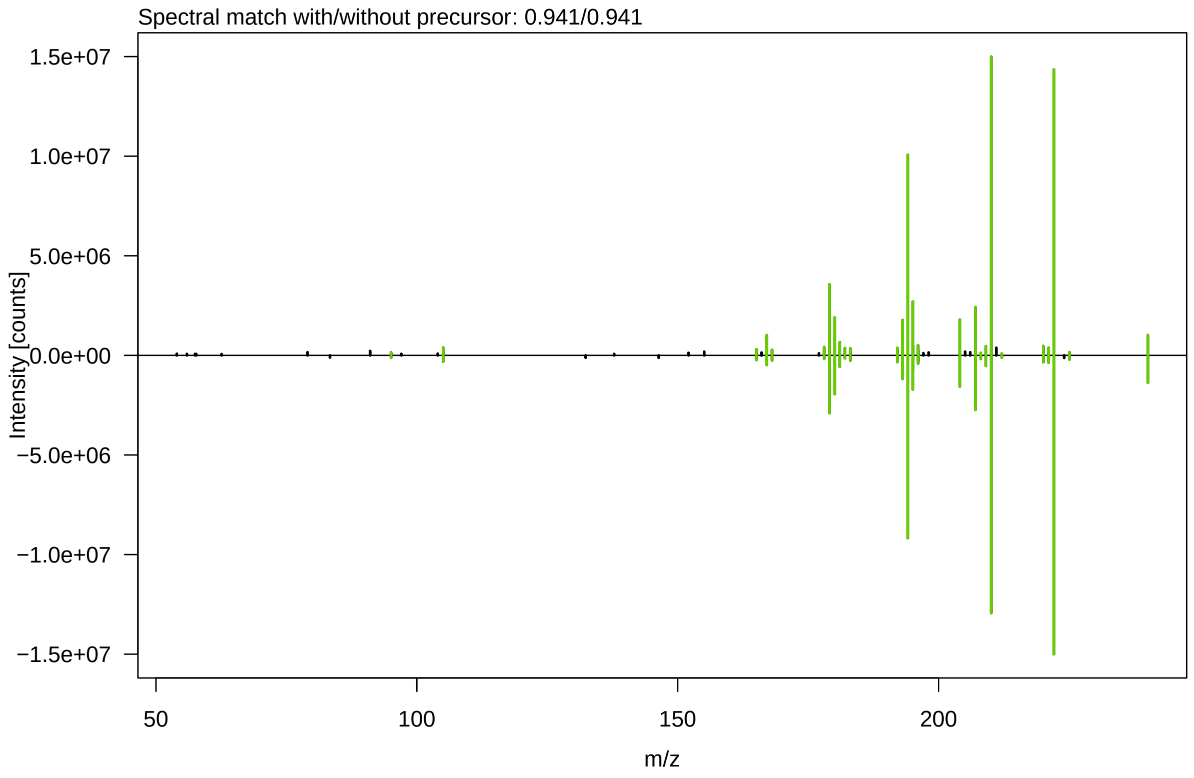

Figure SI-D225: Head to tail plot of 3-hydroxymethylmefenamic acid in wastewater (top) and from human liver S9 incubation (bottom). Matching fragments are highlighted in green.

In addition to the human liver S9 incubation experiment, a reference standard of 3-hydroxymethyl-mefenamic acid was purchased. Figure SI-D226 shows the extracted ion chromatograms of this standard, the sample and the spiked sample, as well as a head to tail plot of the MS2 spectra of the standard and the sample. In addition, the most intense MS2 fragments in the sample and in the standard are displayed. It becomes visible that the retention times of the sample and the spiked sample are identical and the spectra similarity score between sample and standard is equal to 0.991. The vast majority of the MS2 fragments in the sample can be explained by the reference standard. It can therefore be concluded that the suspected compound is indeed 3-hydroxymethylmefenamic acid. Correspondingly, the identification confidence can be increased to level 1.

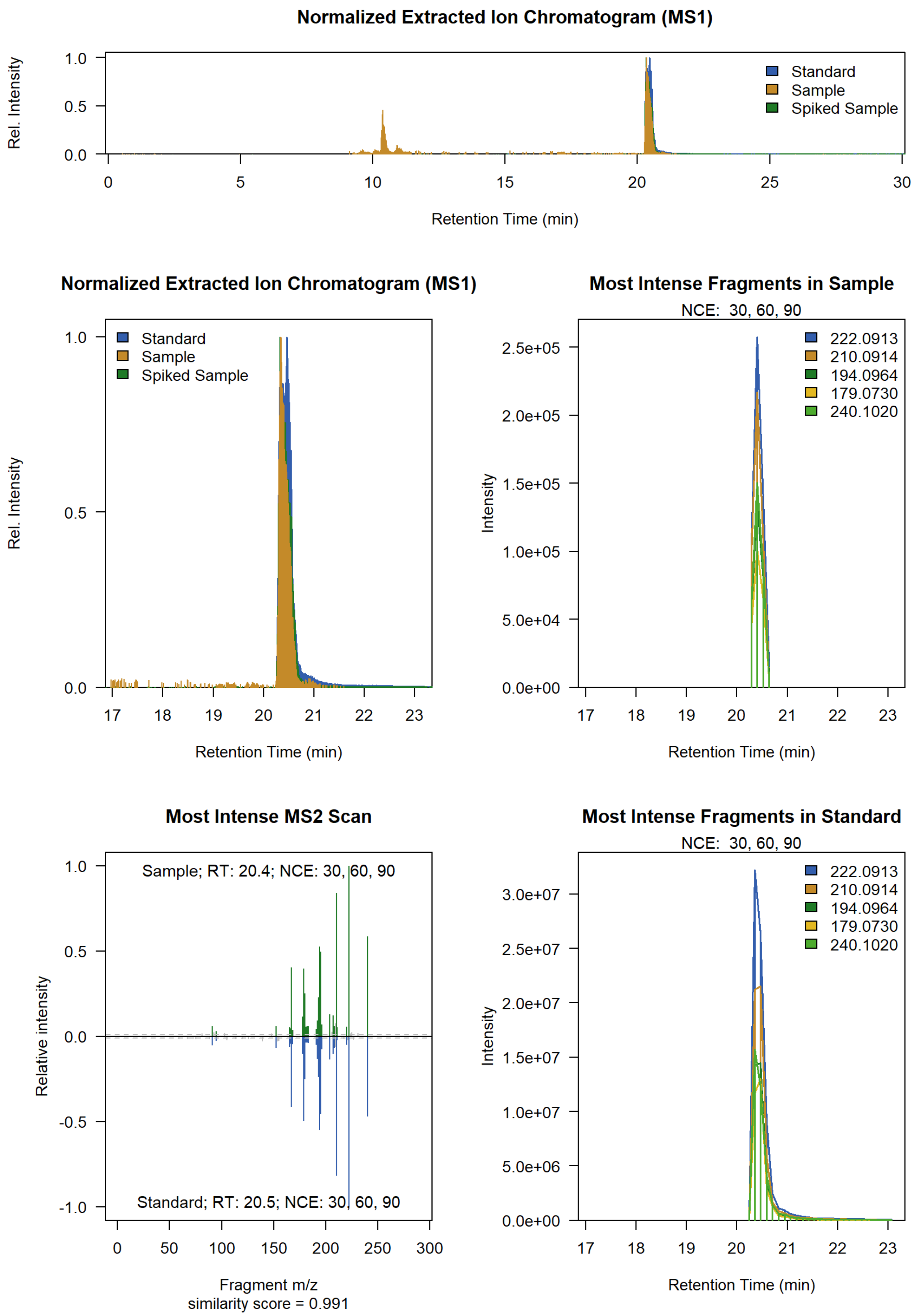

Figure SI-D226: Extracted ion chromatograms of 3-hydroxymethylmefenamic acid in the reference standard, the sample and the spiked sample, as well as MS2 head to tail plot and most intense MS2 fragments in standard and sample.

SI-D2.11 Nevirapine Metabolites

Nevirapine is a non-nucleoside reverse transcriptase inhibtor used in combination with nucleosdie analogues for the treatment of HIV.[2] Two signals potentially originating from two hydroxylated metabolites were detected in the wastewater samples by suspect screening and molecular networking (see Fiure SI-D228.) In literature, four hydroxynevirapine metabolites are reported from pharmacokinetic studies in humans. They are displayed in the metabolism scheme of nevirapine (Figure SI-D227). Based on *in silico* fragmentation, no clear differentiation between these four metabolites was possible. Therefore, the most abundant hydroxylated metabolite in plasma, 12-hydroxynevirapine,[14] and a second metabolite, 2-hydroxynevirapine were purchased. Based on the different predicted $\log K_{OW}$ of the four metabolites, it was hypthesized, that the earlier eluting signal originates from 2-hydrxynevirapine ($\log K_{OW}$ = 1.20[15]) and 12-hydroxynevirapine ($\log K_{OW}$ = 2.43[15]). The other two metabolites, 3-hydroxynevirapine and 8-hydroxynevirapine, which both have $\log K_{OW}$ values of 3.06,[15] are expected to contribute to the later eluting signal. Moreoever, SIRIUS/CSI:FingerID yielded better results for 2/12-hydroxynevirapine for the earlier eluting signal, and better for 3/8-hydroxynevirapine for the later eluting peak.

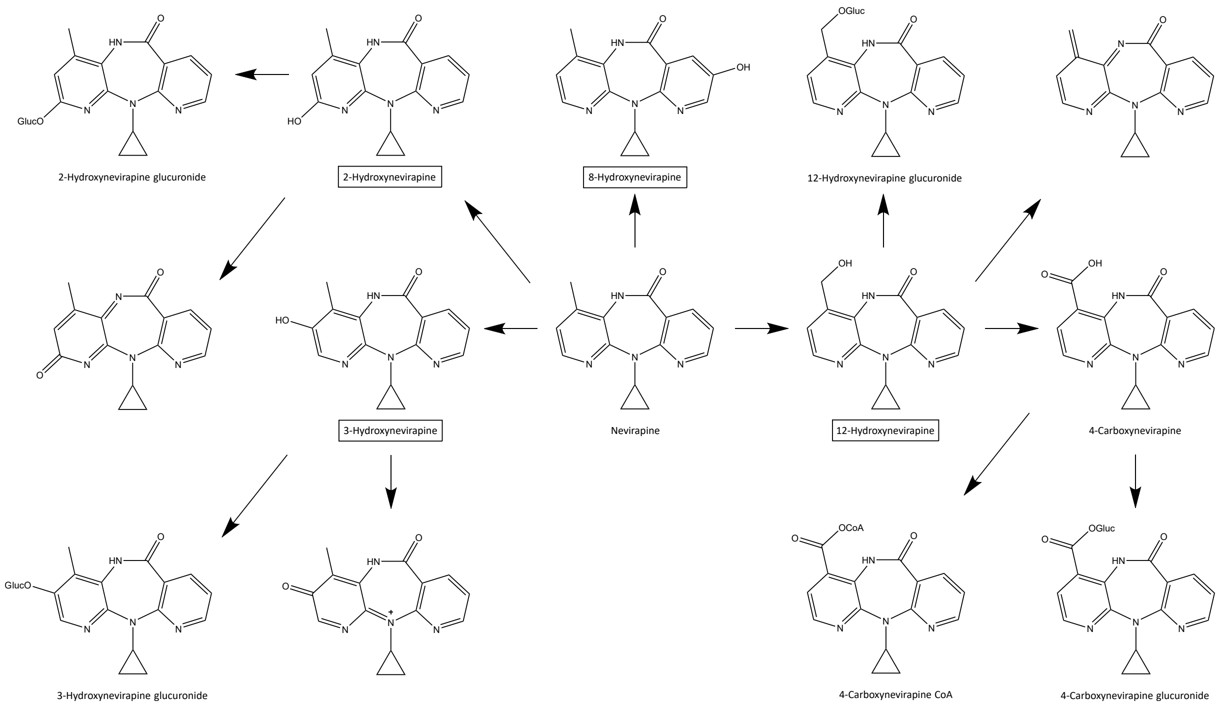

Figure SI-D227: Human metabolism of nevirapine. Framed metabolites were identified during suspect screening. Scheme adapted from.[14]

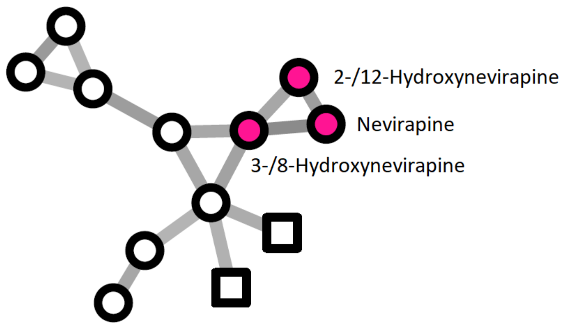

Figure SI-D228: Excerpt of the molecular network showing the nevirapine cluster.

SI-D2.11.1 2-Hydroxynevirapine and 12-Hydroxynevirapine

Table SI-D111: Information on identifiers, chemical properties, detection and confidence of identification of 2-hydroxynevirapine and 12-hydroxynevirapine.

IUPAC Name	2-cyclopropyl-7-methyl-2,4,9,15-tetrazatricyclo[9.4.0.0^{3,8}]pentadeca-1(11),3(8),6,12,14-pentaene-5,10-dione<br>2-cyclopropyl-7-(hydroxymethyl)-2,4,9,15-tetrazatricyclo[9.4.0.0^{3,8}]pentadeca-1(11),3,5,7,12,14-hexaen-10-one
Molecular formula	$C_{15}H_{14}N_4O_2$
Monoisotopic mass [g/mol]	282.1117
Adduct	$[M+H]^+$
Retention time [min]	14.9
SMILES	CC1=CC(=O)NC2=C1NC(=O)C3=C(N2C4CC4)N=CC=C3<br>C1CC1N2C3=C(C=CC=N3)C(=O)NC4=C(C=CN=C42)CO
InChI	InChI=1S/C15H14N4O2/c1-8-7-11(20)17-14-12(8)18-15(21)10-3-2-6-16-13(10)19(14)9-4-5-9/h2-3,6-7,9H,4-5H2,1H3,(H,17,20)(H,18,21)<br>InChI=1S/C15H14N4O2/c20-8-9-5-7-17-14-12(9)18-15(21)11-2-1-6-16-13(11)19(14)10-3-4-10/h1-2,5-7,10,20H,3-4,8H2,(H,18,21)
InChI-Key	LFZSOJABLBVGRJ-UHFFFAOYSA-N<br>SEBABOMFNCVZGF-UHFFFAOYSA-N
CAS RN	254889-31-1<br>133627-24-4
Metabolite of	Nevirapine
Detection frequency	100% (15/15 samples)
Detected in	Altenrhein, Monday-Friday<br>Neugut, Monday-Friday<br>Werdhölzli, Monday-Friday
Intensity	E9
Initial confidence level	level 3<br>level 3
Initial confidence score	0.45<br>0.45
Final confidence level	level 3<br>level 1

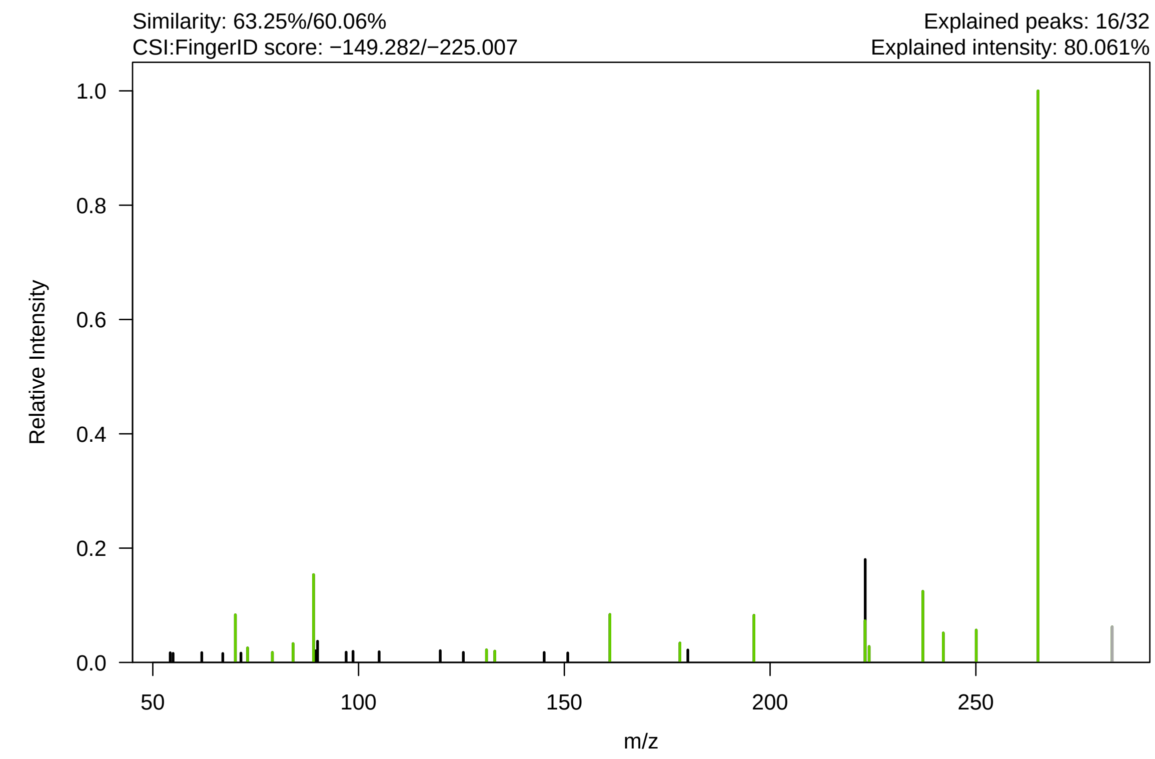

Figure SI-D229: Measured MS2 spectrum. Matching fragments with 2-hydroxynevirapine and 12-hydroxynevirapine predicted by SIRIUS/CSI:FingerID are highlighted in green. The molecular ion in gray is not considered.

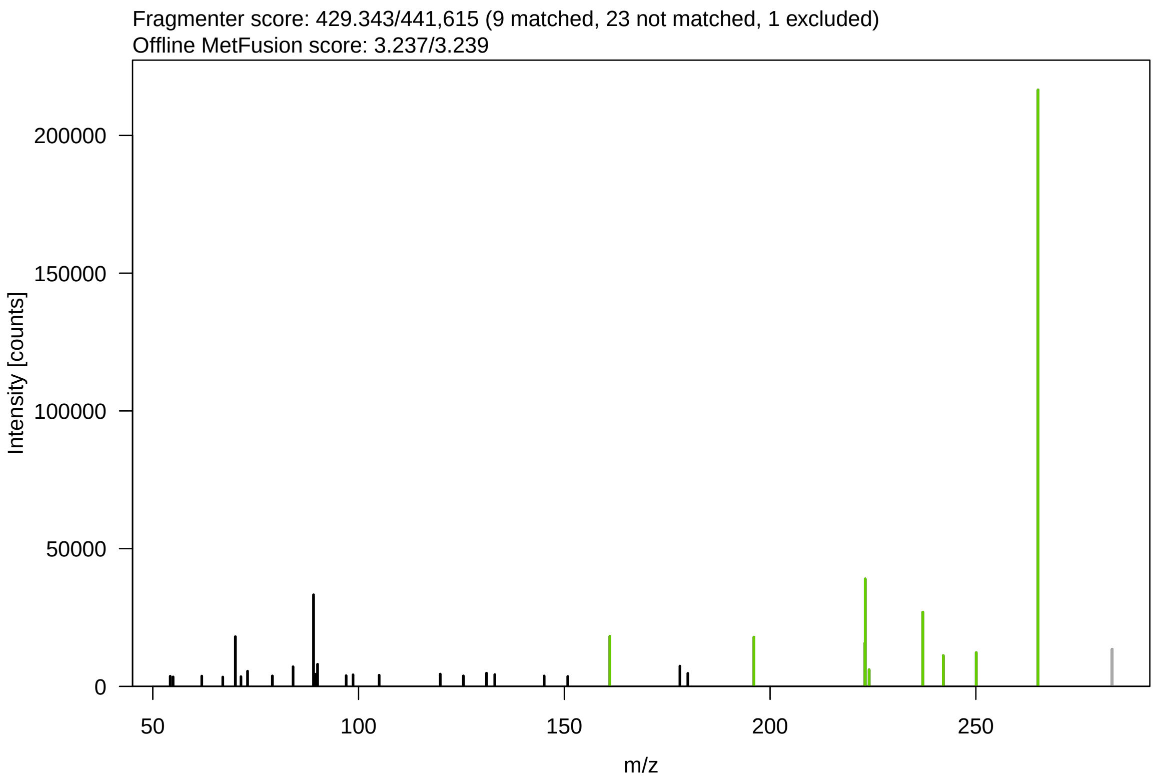

Figure SI-D230: Measured MS2 spectrum. Matching fragments with 2-hydroxynevirapine and 12-hydroxynevirapine predicted by MetFrag are highlighted in green. The molecular ion in gray is not considered.

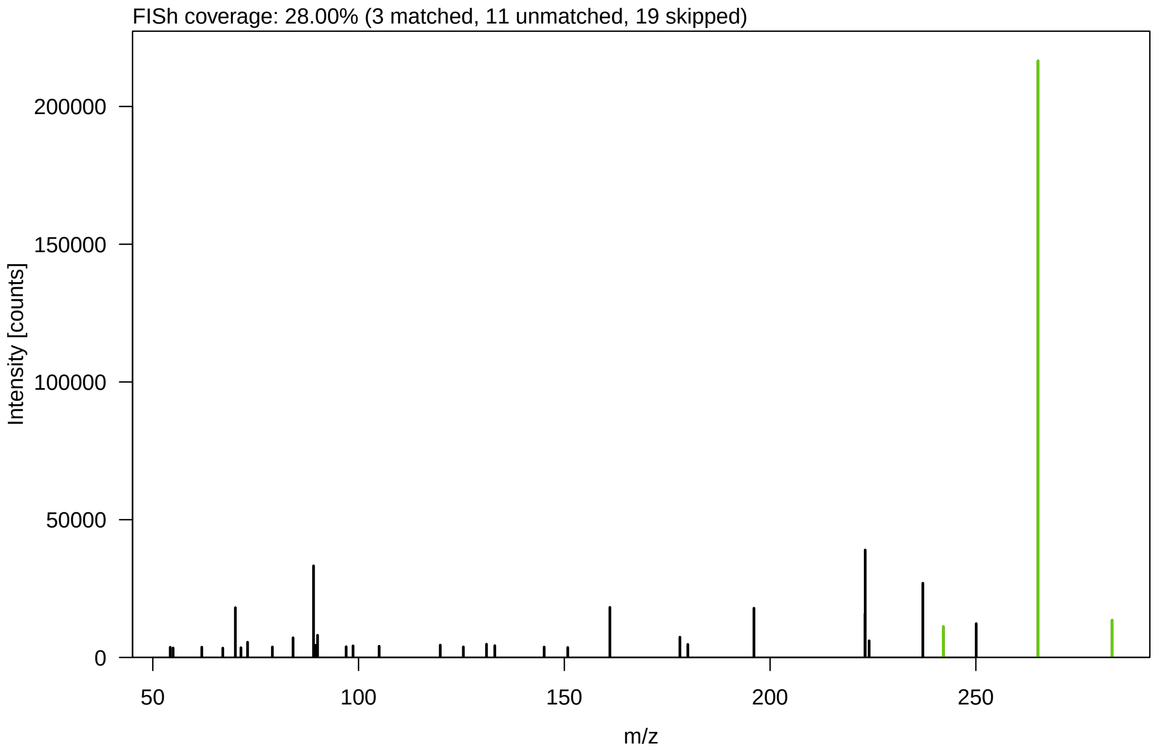

Figure SI-D231: Measured MS2 spectrum. Matching fragments with 2-hydroxynevirapine and 12-hydroxynevirapine predicted by FISh Scoring are highlighted in green. Low intensity fragments are not considered and skipped.

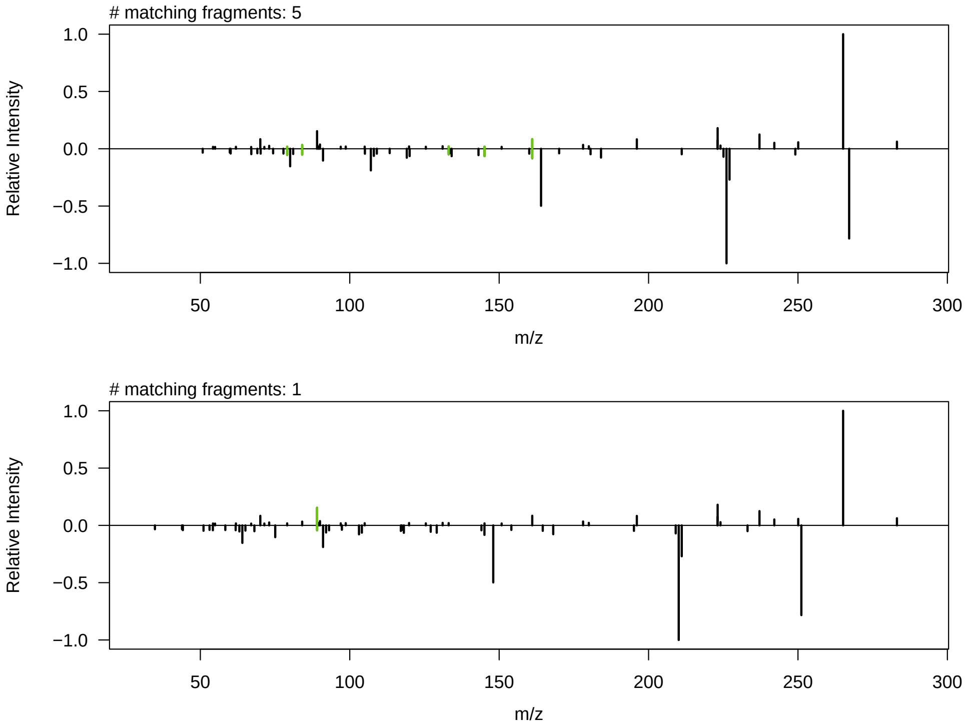

Figure SI-D232: Head to tail plots of 2/12-hydroxynevirapine and nevirapine. In the bottom plot, the mass spectrum of nevirapine is shifted by the mass difference. Matching fragments are highlighted in green.

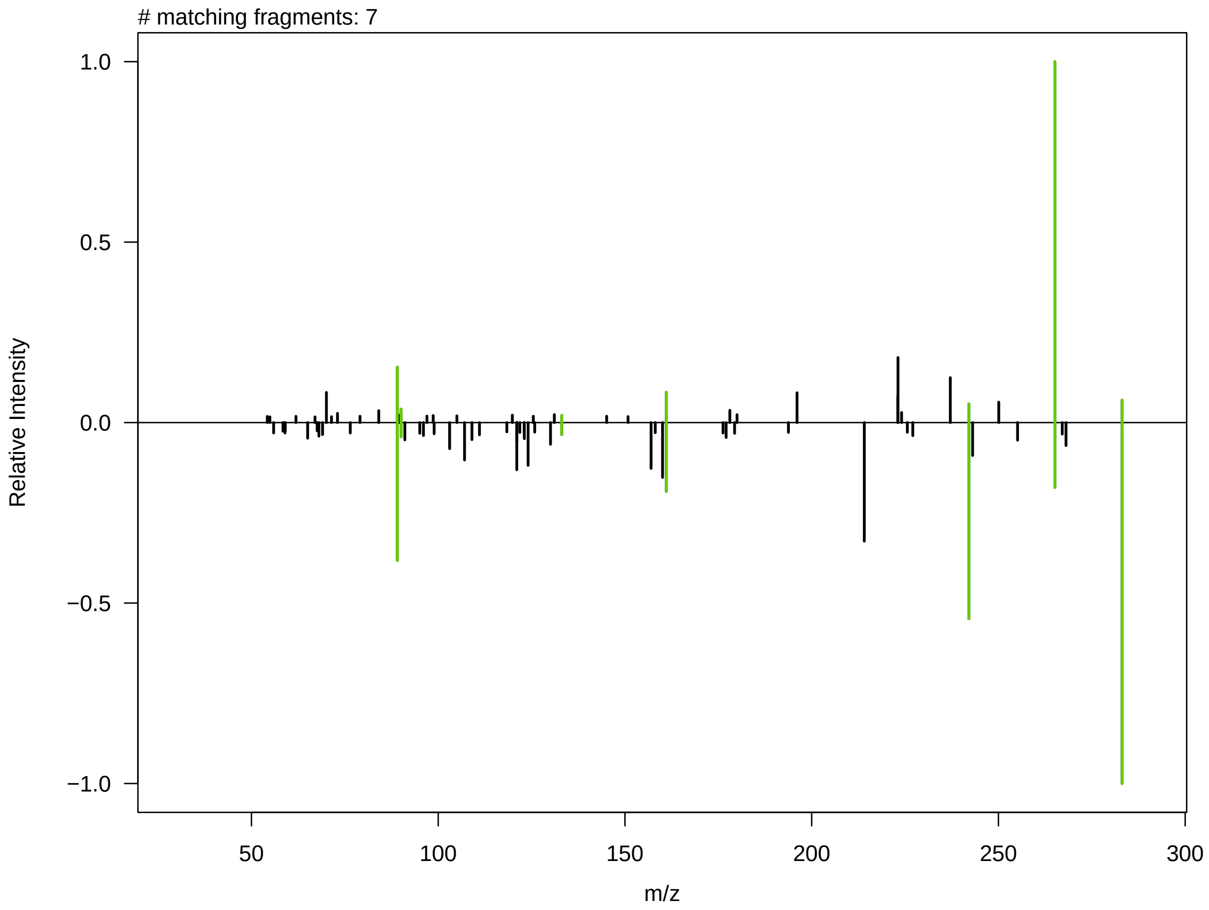

Figure SI-D233: Head to tail plot of 2/12-hydroxynevirapine and 3/8-hydroxynevirapine. Matching fragments are highlighted in green.

Table SI-D112: Molecular network results and retention time prediction of 2- and 12-hydroxynevirapine.

Comparison with	Nevirapine
MSn Score	35
Forward coverage	38
Reverse coverage	32
Forward match	5
Reverse match	8
ΔMass [g/mol]	15.9949
Comparison with	3/8-Hydroxynevirapine
MSn Score	46
Forward coverage	39
Reverse coverage	52
Forward match	15
Reverse match	13
ΔMass [g/mol]	0
Measured retention time [min]	14.9
Predicted $logD_{OW}$ (pH = 2.7)	-0.09
Predicted retention time [min]	14.6
Predicted retention time range (95% confidence interval) [min]	10.0-19.2
Predicted retention time range (99% confidence interval) [min]	8.6-20.7
Measured retention time [min]	14.9
Predicted $logD_{OW}$ (pH = 2.7)	1.07
Predicted retention time [min]	16.1
Predicted retention time range (95% confidence interval) [min]	11.5-20.7
Predicted retention time range (99% confidence interval) [min]	10.1-22-2

Table SI-D113: Annotated MS2 spectrum of 2/12-hydroxynevirapine.

m/z	Relative Intensity	Annotation
54.2349	16.97	
54.9309	15.86	
61.9041	17.23	
67.0080	15.69	
70.0652	83.31	$C_4H_7N + H^+$
71.4193	16.30	
73.0282	25.47	$C_3H_4O_2 + H^+$
79.0543	17.54	$C_6H_6 + H^+$
84.0807	32.90	$C_5H_9N + H^+$
89.0597	153.47	$C_4H_8O_2 + H^+$
89.5704	20.45	
90.0371	37.11	
96.9816	17.95	
98.6553	19.41	

Continued on next page

Table SI-D113: Annotated MS2 spectrum of 2/12-hydroxynevirapine.(Continued)

105.0020	18.76	
119.8482	20.64	
125.4616	17.64	
131.0855	22.08	$C_{10}H_{10} + H^+$
133.1010	19.77	$C_{10}H_{12} + H^+$
145.1004	17.49	
150.8321	16.64	
161.0709	83.85	$C_9H_8N_2O + H^+$
178.0857	33.97	$C_{10}H_{11}NO_2 + H^+$
180.0118	21.83	
196.0744	82.45	$C_{11}H_7N_4 + H^+$
223.0611	72.98	$C_{12}H_6N_4O + H^+$
223.1104	179.98	$C_{14}H_{12}N_3 + H^+$
224.0681	27.87	$C_{12}H_7N_4O + H^+$
237.1131	124.30	$C_{14}H_{12}N_4 + H^+$
242.0799	51.56	$C_{12}H_9N_4O_2 + H^+$
250.0852	56.60	$C_{14}H_9N_4O + H^+$
265.1082	999.00	$C_{15}H_{12}N_4O + H^+$
283.1178	62.21	$C_{25}H_{14}N_4O_2 + H^+$

Reference standards of 2-hydroxynevirapine and 12-hydroxynevirapine were purchased, since it was not possible to differentiate between the two compounds based on the previously shown results. Figures SI-D234 and SI-D235 show the extracted ion chromatograms of 2-hydroxynevirapine and 12-hydroxynevirapine reference standards, the sample and the spiked samples, as well as the head to tail plots of the MS2 spectra of the standard and the sample. In addition, the most intense MS2 fragments in the sample and in the standards are displayed. It becomes visible, that the retention times of 2-hydroxynevirapine and 12-hydroxynevirapine are identical, but that the MS2 fragments of 12-hydroxynevirapine match better with a score of 0.362. Several MS2 fragments in the sample can be explained by the reference standard of 12-hydroxynevirapine. Consequently, the majority of the signal intensity in the sample can be attributed to 12-hydroxynevirapine, but a contribution of 2-hydroxynevirapine cannot be excluded. The identification confidence of 12-hydroxynevirapine is increased to level 1, while the identification confidence of 2-hydroxynevirapine remains at level 3.

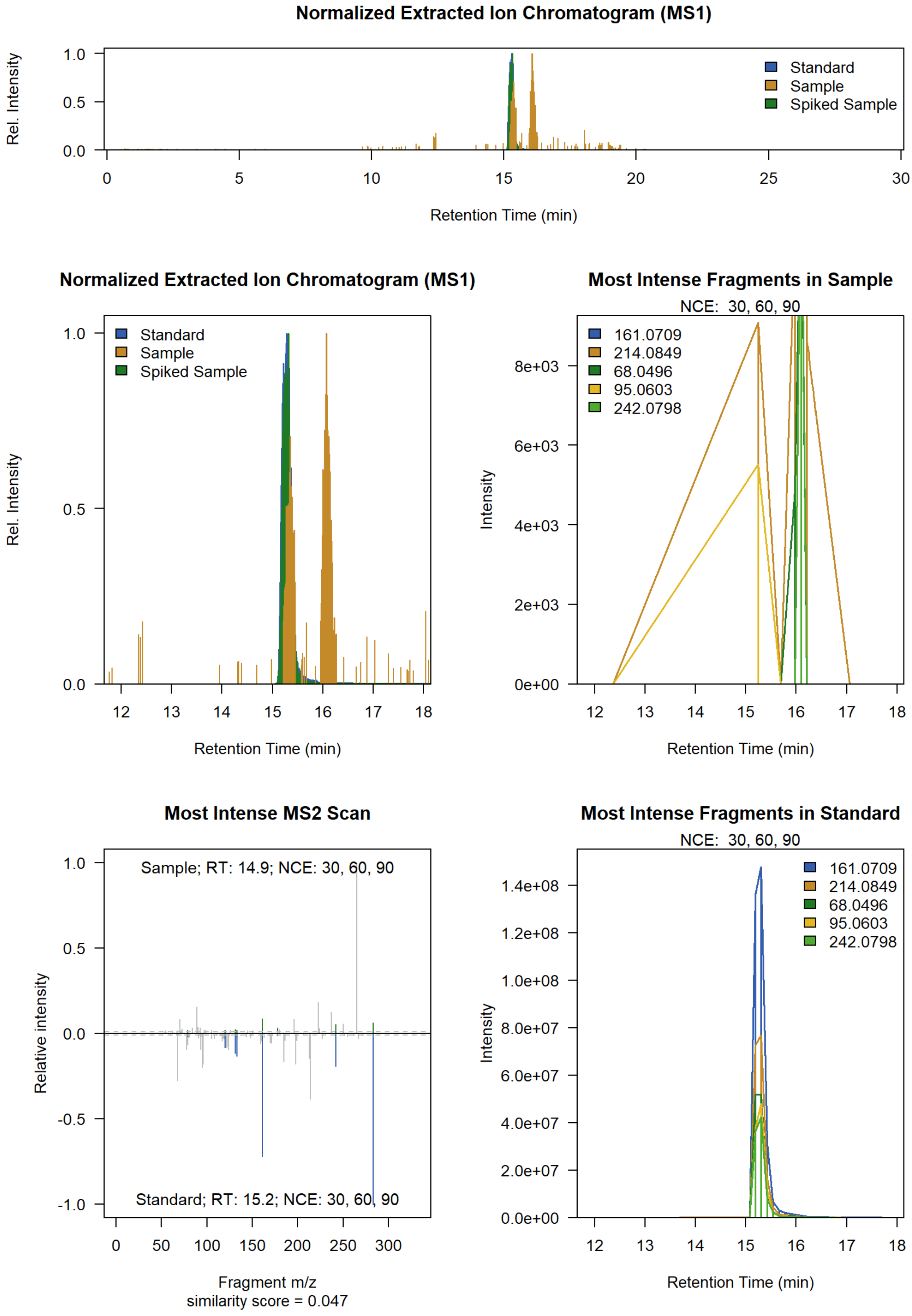

Figure SI-D234: Extracted ion chromatograms of 2-hydroxynevirapine in the reference standard, the sample and the spiked sample, as well as MS2 head to tail plot and most intense MS2 fragments in standard and sample.

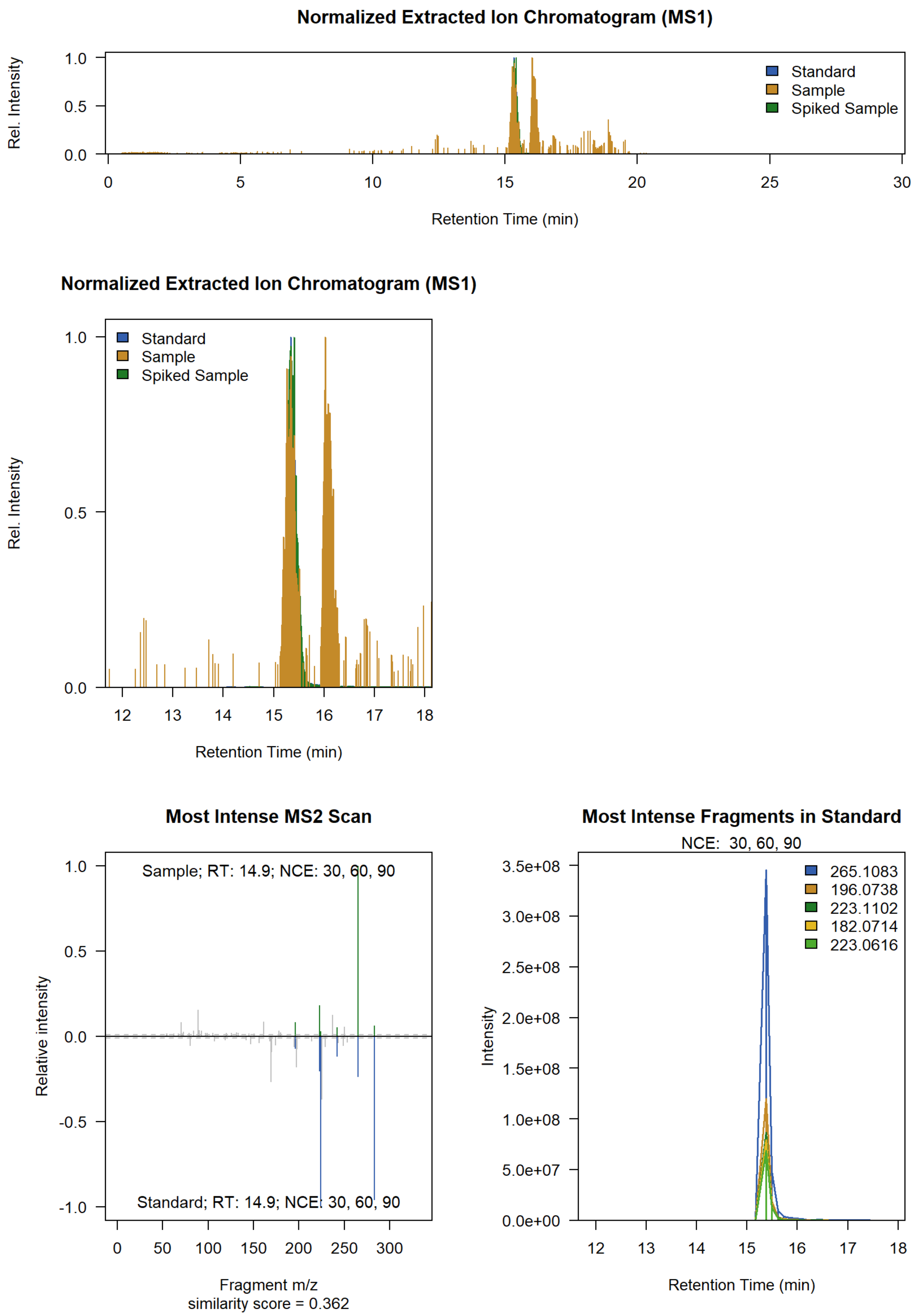

Figure SI-D235: Extracted ion chromatograms of 12-hydroxynevirapine in the reference standard, the sample and the spiked sample, as well as MS2 head to tail plot and most intense MS2 fragments in standard and sample.

SI-D2.11.2 3-Hydroxynevirapine and 8-Hydroxynevirapine

Table SI-D114: Information on identifiers, chemical properties, detection and confidence of identification of 3-hydroxynevirapine and 8-hydroxynevirapine.

IUPAC Name	2-cyclopropyl-6-hydroxy-7-methyl-2,4,9,15-tetrazatricyclo[9.4.0.0^{3,8}]pentadeca-1(11),3,5,7,12,14-hexaen-10-one<br>2-cyclopropyl-13-hydroxy-7-methyl-2,4,9,15-tetrazatricyclo[9.4.0.0^{3,8}]pentadeca-1(11),3,5,7,12,14-hexaen-10-one
Molecular formula	$C_{15}H_{14}N_4O_2$
Monoisotopic mass [g/mol]	282.1117
Adduct	$[M+H]^+$
Retention time [min]	15.6
SMILES	CC1=C2C(=NC=C1O)N(C3=C(C=CC=N3)C(=O)N2)C4CC4<br>CC1=C2C(=NC=C1)N(C3=C(C=C(C=N3)O)C(=O)N2)C4CC4
InChI	InChI=1S/C15H14N4O2/c1-8-11(20)7-17-14-12(8)18-15(21)10-3-2-6-16-13(10)19(14)9-4-5-9/h2-3,6-7,9,20H,4-5H2,1H3,(H,18,21)<br>InChI=1S/C15H14N4O2/c1-8-4-5-16-14-12(8)18-15(21)11-6-10(20)7-17-13(11)19(14)9-2-3-9/h4-7,9,20H,2-3H2,1H3,(H,18,21)
InChI-Key	DANIONWINZEYME-UHFFFAOYSA-N<br>DZPVEPLRIKDBFC-UHFFFAOYSA-N
CAS RN	174532-82-2<br>254889-32-2
Metabolite of	Nevirapine
Detection frequency	100% (15/15 samples)
Detected in	Altenrhein, Monday-Friday<br>Neugut, Monday-Friday<br>Werdhölzli, Monday-Friday
Intensity	E9
Initial confidence level	level 3<br>level 3
Initial confidence score	0.50<br>0.50
Final confidence level	level 3<br>level 3

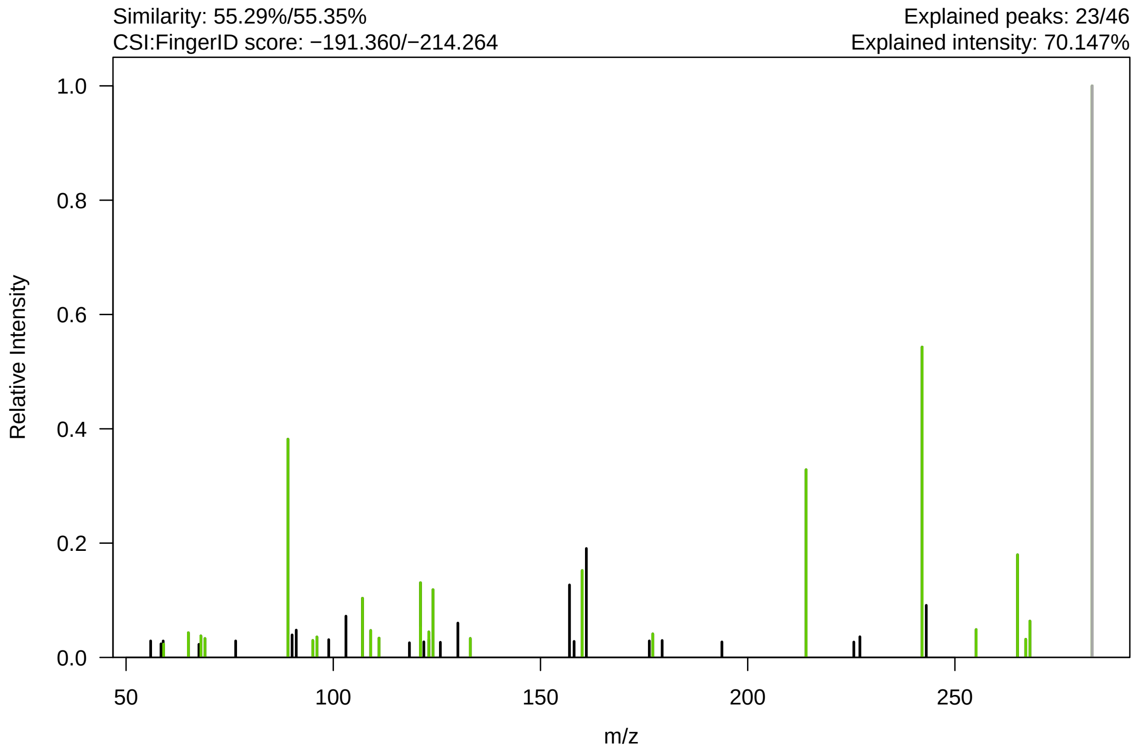

Figure SI-D236: Measured MS2 spectrum. Matching fragments with 3-hydroxynevirapine and 8-hydroxynevirapine predicted by SIRIUS/CSI:FingerID are highlighted in green. The molecular ion in gray is not considered.

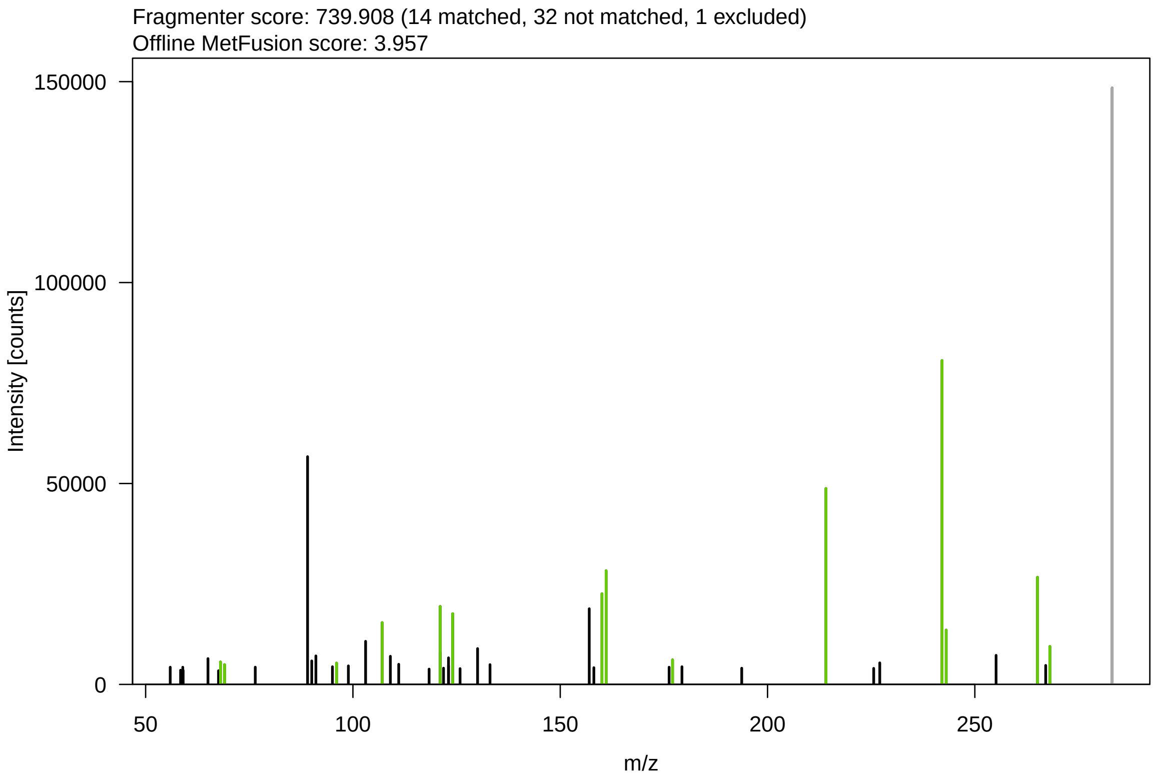

Figure SI-D237: Measured MS2 spectrum. Matching fragments with 3-hydroxynevirapine predicted by MetFrag are highlighted in green. The molecular ion in gray is not considered.

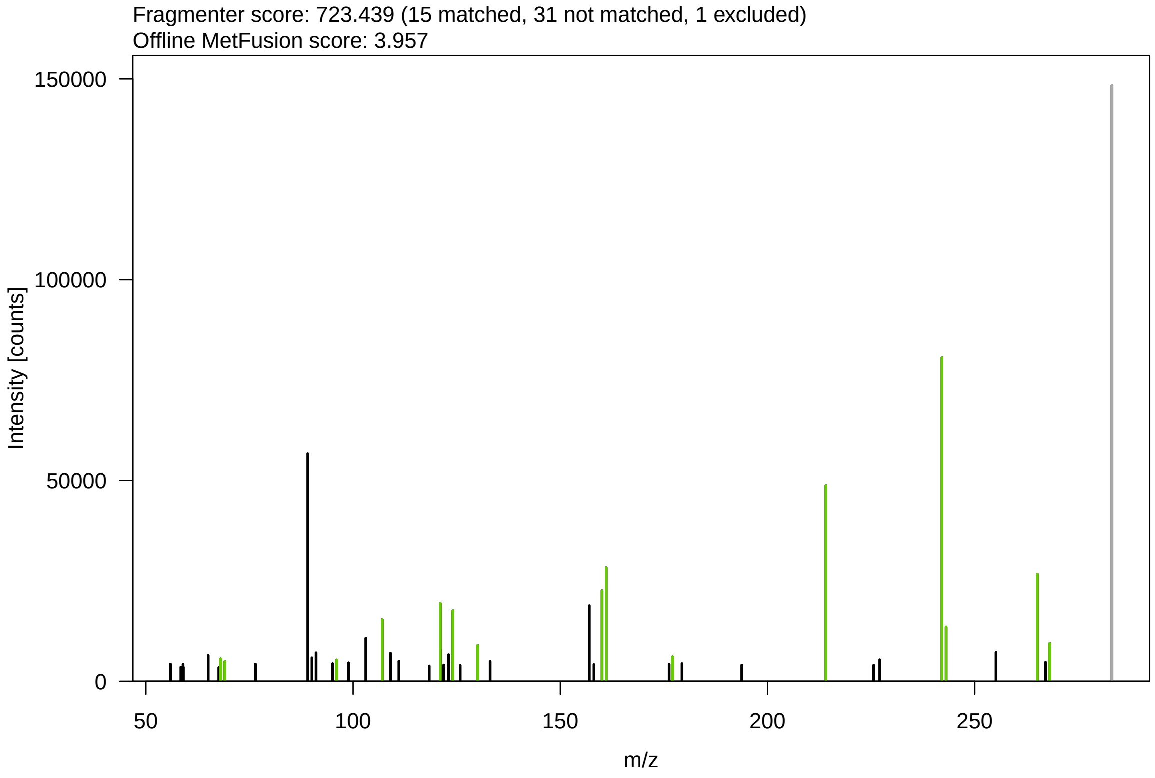

Figure SI-D238: Measured MS2 spectrum. Matching fragments with 8-hydroxynevirapine predicted by MetFrag are highlighted in green. The molecular ion in gray is not considered.

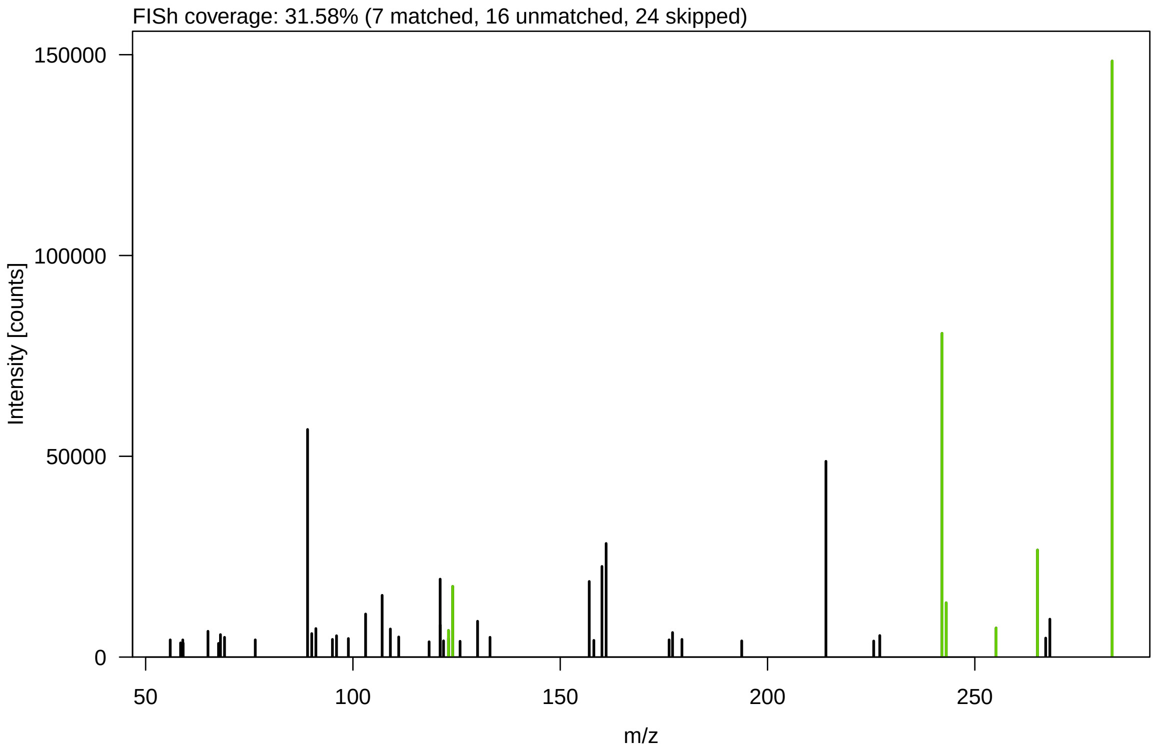

Figure SI-D239: Measured MS2 spectrum. Matching fragments with 3-hydroxynevirapine predicted by FISh Scoring are highlighted in green. Low intensity fragments are not considered and skipped.

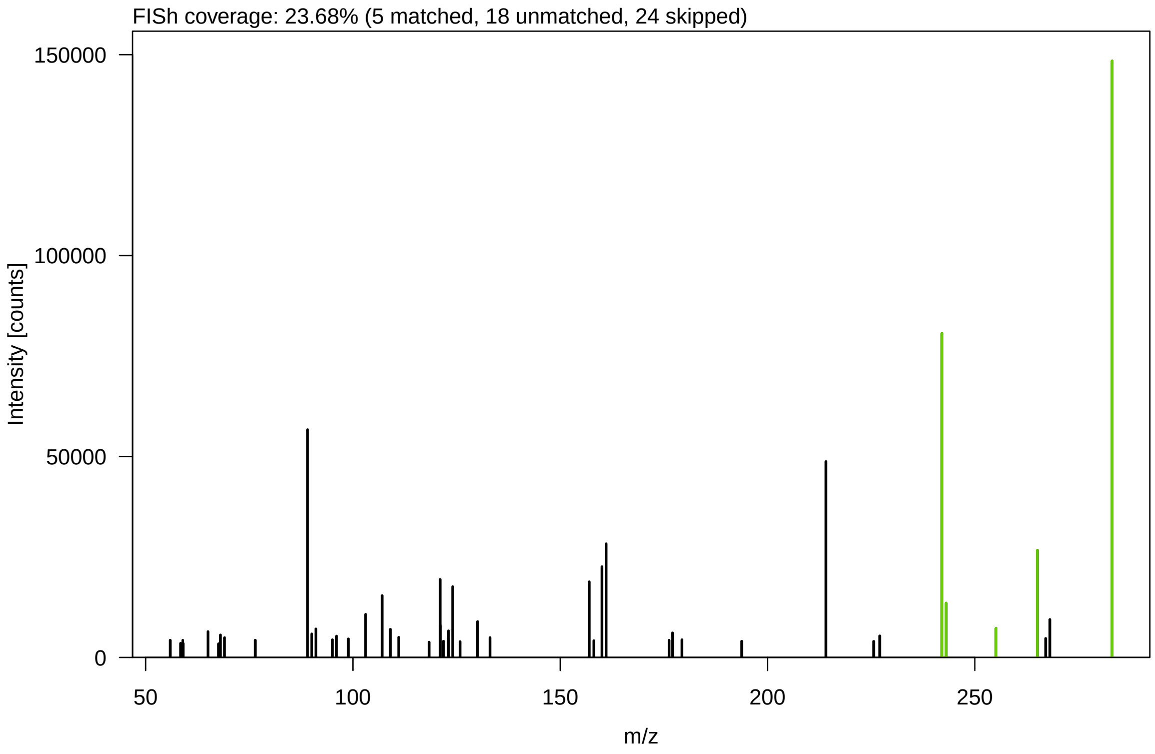

Figure SI-D240: Measured MS2 spectrum. Matching fragments with 8-hydroxynevirapine predicted by FISh Scoring are highlighted in green. Low intensity fragments are not considered and skipped.

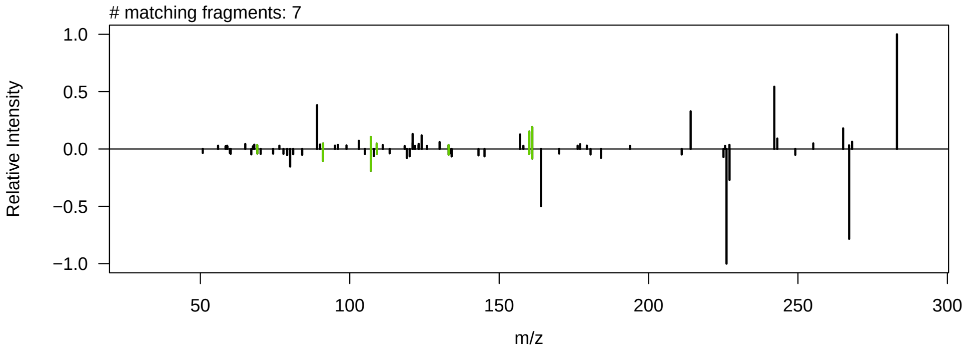

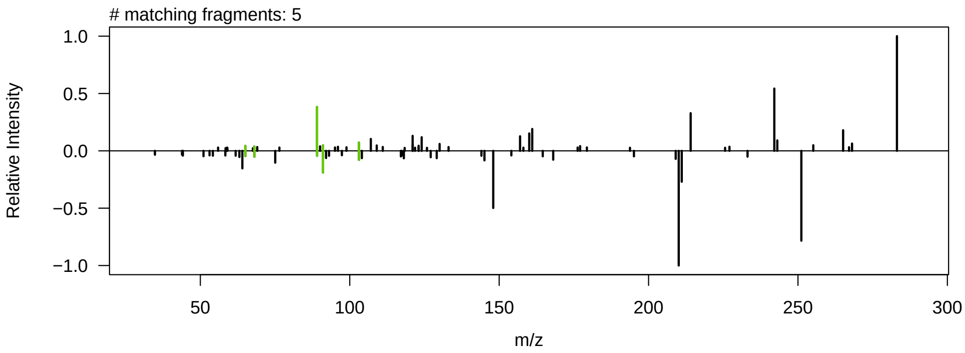

Figure SI-D241: Head to tail plots of 3/8-hydroxynevirapine and nevirapine. In the bottom plot, the mass spectrum of nevirapine is shifted by the mass difference. Matching fragments are highlighted in green.

Table SI-D115: Molecular network results and retention time prediction of 3- and 8-hydroxynevirapine.

Comparison with	Nevirapine
MSn Score	39
Forward coverage	46
Reverse coverage	32
Forward match	6
Reverse match	12
ΔMass [g/mol]	15.9949
Measured retention time [min]	15.6
Predicted $logD_{OW}$ (pH = 2.7)	1.41
Predicted retention time [min]	16.6
Predicted retention time range (95% confidence interval) [min]	12.0-21.2
Predicted retention time range (99% confidence interval) [min]	10.5-22.6
Measured retention time [min]	15.6
Predicted $logD_{OW}$ (pH = 2.7)	1.41
Predicted retention time [min]	16.6
Predicted retention time range (95% confidence interval) [min]	12.0-21.2
Predicted retention time range (99% confidence interval) [min]	10.5-22.6

Table SI-D116: Annotated MS2 spectrum of 3-hydroxynevirapine.

m/z	Relative Intensity	Annotation
55.9349	28.84	
58.4355	23.80	
58.9622	28.77	
59.0492	23.44	$C_3H_6O + H^+$
65.0388	43.25	$C_5H_4 + H^+$
67.5875	22.92	
68.0495	37.67	$C_4H_5N + H^+$
69.0335	33.11	$C_4H_4O + H^+$
76.4477	28.86	
89.0597	381.49	$C_4H_8O_2 + H^+$
90.0629	39.40	
91.0640	47.80	
95.0601	29.64	$C_5H_6N_2 + H^+$
96.0442	35.75	$C_5H_5NO + H^+$
98.9050	31.08	
103.0574	72.17	
107.0489	103.52	$C_7H_6O + H^+$
109.0285	47.10	$C_6H_4O_2 + H^+$
111.0440	33.75	$C_6H_6O_2 + H^+$

Continued on next page

Table SI-D116: Annotated MS2 spectrum of 3-hydroxynevirapine.(Continued)

118.3757	25.66	
121.0395	130.65	$C_6H_4N_2O + H^+$
121.0643	53.55	$C_8H_8O + H^+$
121.8529	27.25	
123.0444	31.46	
123.0546	44.60	$C_6H_6N_2O + H^+$
124.0632	118.48	$C_6H_7N_2O + H^+$
125.8419	26.48	
130.0650	60.08	
133.0649	33.15	$C_9H_8O + H^+$
156.9952	126.75	
158.1102	28.03	
160.0633	151.93	$C_9H_7N_2O + H^+$
161.0708	190.39	$C_9H_8N_2O + H^+$
176.2584	28.87	
177.0897	41.13	$C_{11}H_{12}O_2 + H^+$
179.3560	29.64	
193.7738	27.13	
214.0848	328.12	$C_{11}H_9N_4O + H^+$
225.6083	26.91	
227.0686	36.13	
242.0795	542.56	$C_{12}H_9N_4O_2 + H^+$
243.0866	91.05	$C_{12}H_{10}N_4O_2 + H^+$
255.1238	48.71	$C_{14}H_{14}N_4O + H^+$
265.1092	179.45	$C_{15}H_{12}N_4O + H^+$
267.0879	31.84	$C_{14}H_{10}N_4O_2 + H^+$
268.0953	63.55	$C_{14}H_{11}N_4O_2 + H^+$
283.1188	999.00	$C_{15}H_{14}N_4O_2 + H^+$

Table SI-D117: Annotated MS2 spectrum of 8-hydroxynevirapine.

m/z	Relative Intensity	Annotation
55.9349	28.84	
58.4355	23.80	
58.9622	28.77	
59.0492	23.44	$C_3H_6O + H^+$
65.0388	43.25	$C_5H_4 + H^+$
67.5875	22.92	
68.0495	37.67	$C_4H_5N + H^+$
69.0335	33.11	$C_4H_4O + H^+$
76.4477	28.86	
89.0597	381.49	$C_4H_8O_2 + H^+$
90.0629	39.40	
91.0640	47.80	
95.0601	29.64	$C_5H_6N_2 + H^+$
96.0442	35.75	$C_5H_5NO + H^+$
98.9050	31.08	
103.0574	72.17	
107.0489	103.52	$C_7H_6O + H^+$
109.0285	47.10	$C_6H_4O_2 + H^+$
111.0440	33.75	$C_6H_6O_2 + H^+$
118.3757	25.66	
121.0395	130.65	$C_6H_4N_2O + H^+$
121.0643	53.55	$C_8H_8O + H^+$
121.8529	27.25	
123.0444	31.46	
123.0546	44.60	$C_6H_6N_2O + H^+$
124.0632	118.48	$C_6H_7N_2O + H^+$
125.8419	26.48	
130.0650	60.08	$C_9H_7N + H^+$
133.0649	33.15	$C_9H_8O + H^+$
156.9952	126.75	
158.1102	28.03	
160.0633	151.93	$C_9H_7N_2O + H^+$
161.0708	190.39	$C_9H_8N_2O + H^+$
176.2584	28.87	
177.0897	41.13	$C_{11}H_{12}O_2 + H^+$
179.3560	29.64	

Continued on next page

Table SI-D117: Annotated MS2 spectrum of 8-hydroxynevirapine.(Continued)

193.7738	27.13	
214.0848	328.12	$C_{11}H_9N_4O + H^+$
225.6083	26.91	
227.0686	36.13	
242.0795	542.56	$C_{12}H_9N_4O_2 + H^+$
243.0866	91.05	$C_{12}H_{10}N_4O_2 + H^+$
255.1238	48.71	$C_{14}H_{14}N_4O + H^+$
265.1092	179.45	$C_{15}H_{12}N_4O + H^+$
267.0879	31.84	$C_{14}H_{10}N_4O_2 + H^+$
268.0953	63.55	$C_{14}H_{11}N_4O_2 + H^+$
283.1188	999.00	$C_{15}H_{14}N_4O_2 + H^+$

No reference standards for 3-hydroxynevirapine and 8-hydroxynevirapine were purchased and no human liver S9 incubation experiment was conducted. Consequently, the identification confidences for both compounds remain at level 3.

SI-D2.12 Tolperisone Metabolites

Tolperisone is a piperidine derivative and acts as a muscle relaxant.[16] Over the course of the suspect screening, four, respectively five, human metabolites could be identified with the aid of the molecular network. The identified metabolites are highlighted in the metabolism scheme of tolperisone in Figure SI-D242. An excerpt of the molecular network showing the tolperisone metabolites cluster is shown in Figure SI-D243. The following subsections give more details on the individual metabolites.

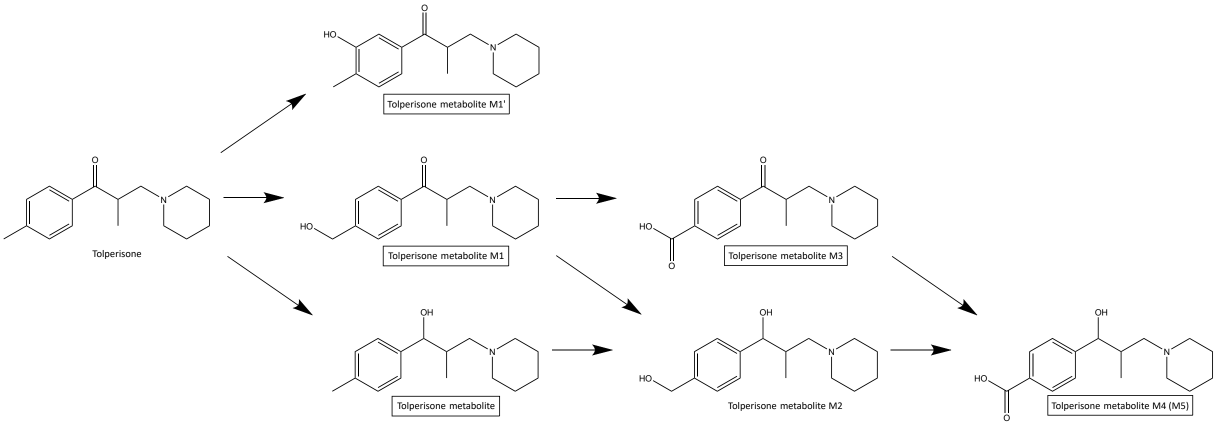

Figure SI-D242: Human metabolism of tolperisone. Framed metabolites were identified during suspect screening. Scheme adapted from.[16]

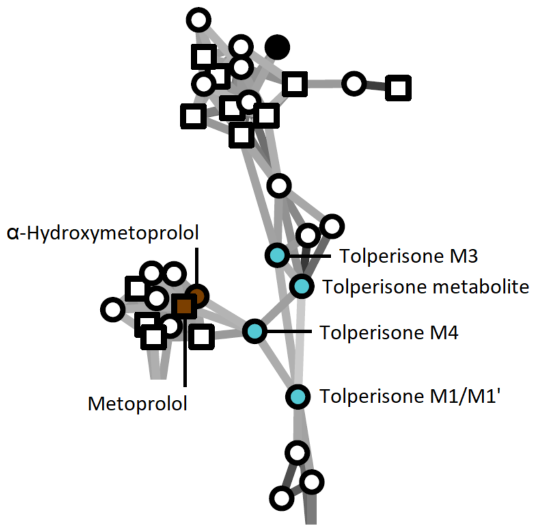

Figure SI-D243: Excerpt of the molecular network showing the tolperisone metabolites cluster.

SI-D2.12.1 Tolperisone Metabolite

Table SI-D118: Information on identifiers, chemical properties, detection and confidence of identification of tolperisone metabolite.

IUPAC Name	2-methyl-1-(4-methylphenyl)-3-piperidin-1-ylpropan-1-ol
Molecular formula	$C_{16}H_{25}NO$
Monoisotopic mass [g/mol]	247.1936
Adduct	$[M+H]^+$
Retention time [min]	11.3
SMILES	CC1=CC=C(C=C1)C(C(C)CN2CCCCC2)O
InChI	InChI=1S/C16H25NO/c1-13-6-8-15(9-7-13)16(18)14(2)12-17-10-4-3-5-11-17/h6-9,14,16,18H,3-5,10-12H2,1-2H3
InChI-Key	IDYRXAZRNVDYCR-UHFFFAOYSA-N
CAS RN	-
Metabolite of	Tolperisone
Detection frequency	100% (15/15 samples)
Detected in	Altenrhein, Monday-Friday
	Neugut, Monday-Friday
	Werdhölzli, Monday-Friday
Intensity	E6-E7
Initial confidence level	level 3
Initial confidence score	0.44
Final confidence level	level 3

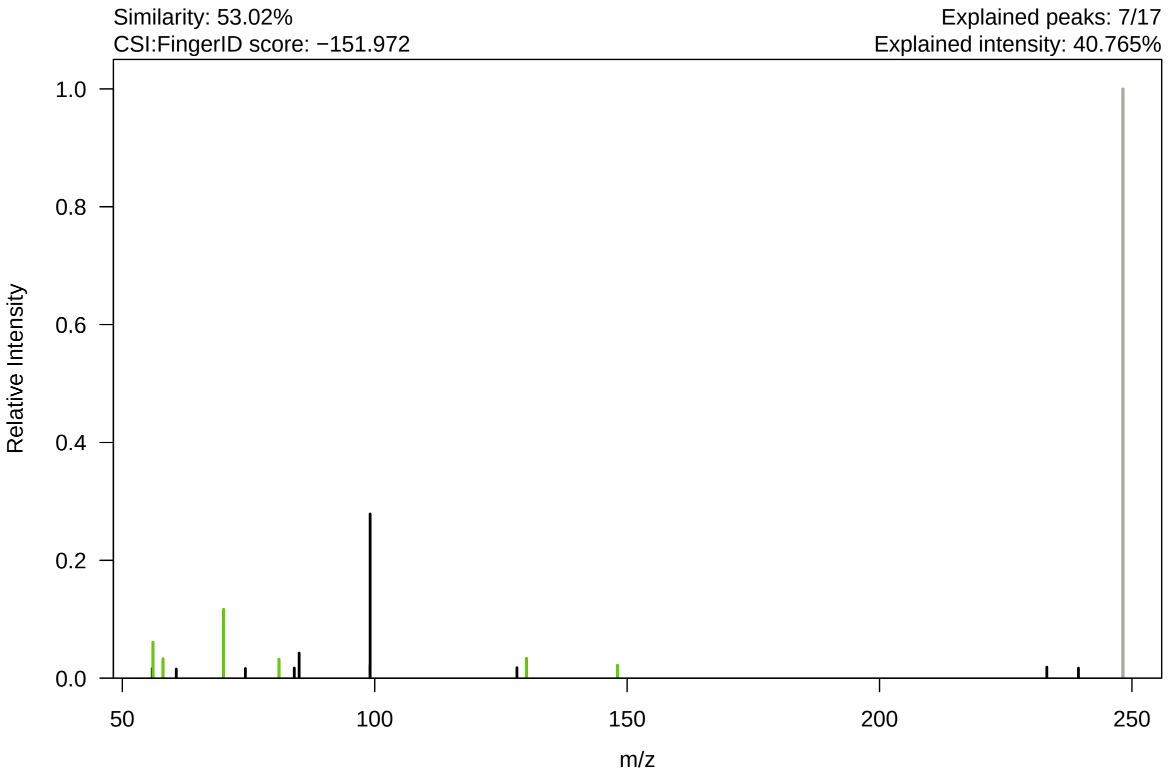

Figure SI-D244: Measured MS2 spectrum. Matching fragments with the tolperisone metabolite predicted by SIRIUS/CSI:FingerID are highlighted in green. The molecular ion in gray is not considered.

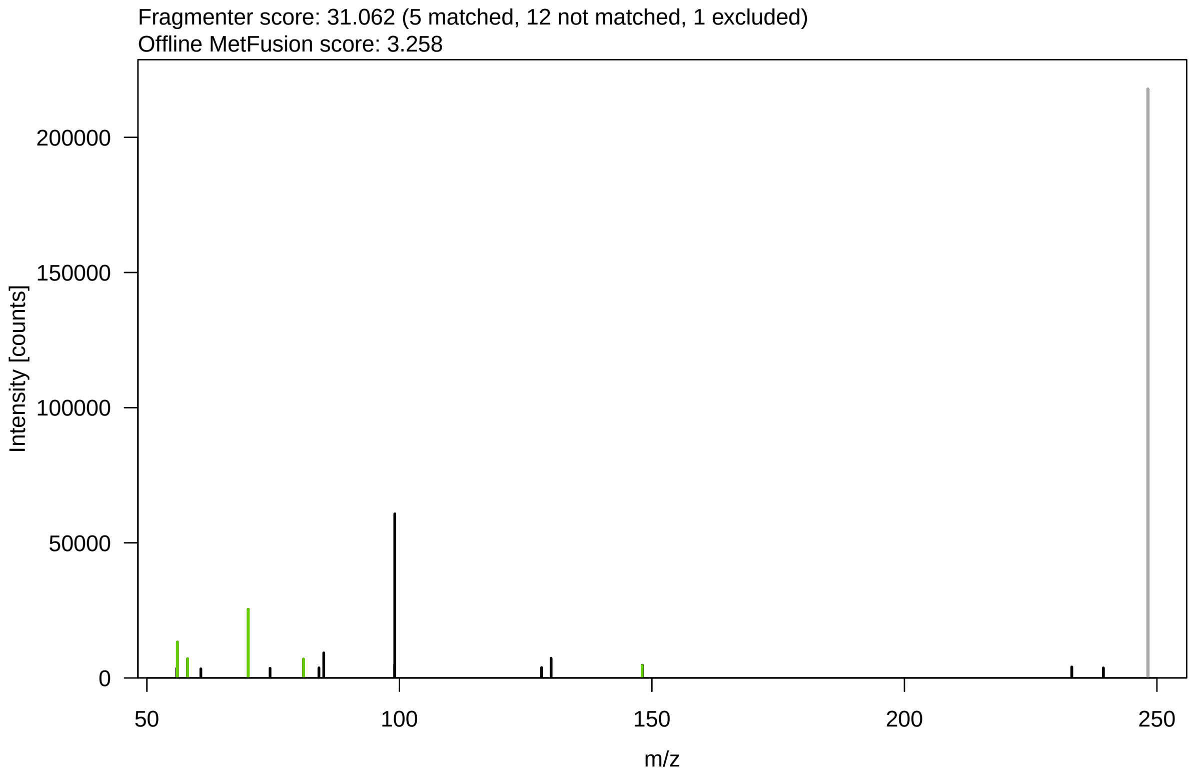

Figure SI-D245: Measured MS2 spectrum. Matching fragments with the tolperisone metabolite predicted by MetFrag are highlighted in green. The molecular ion in gray is not considered.

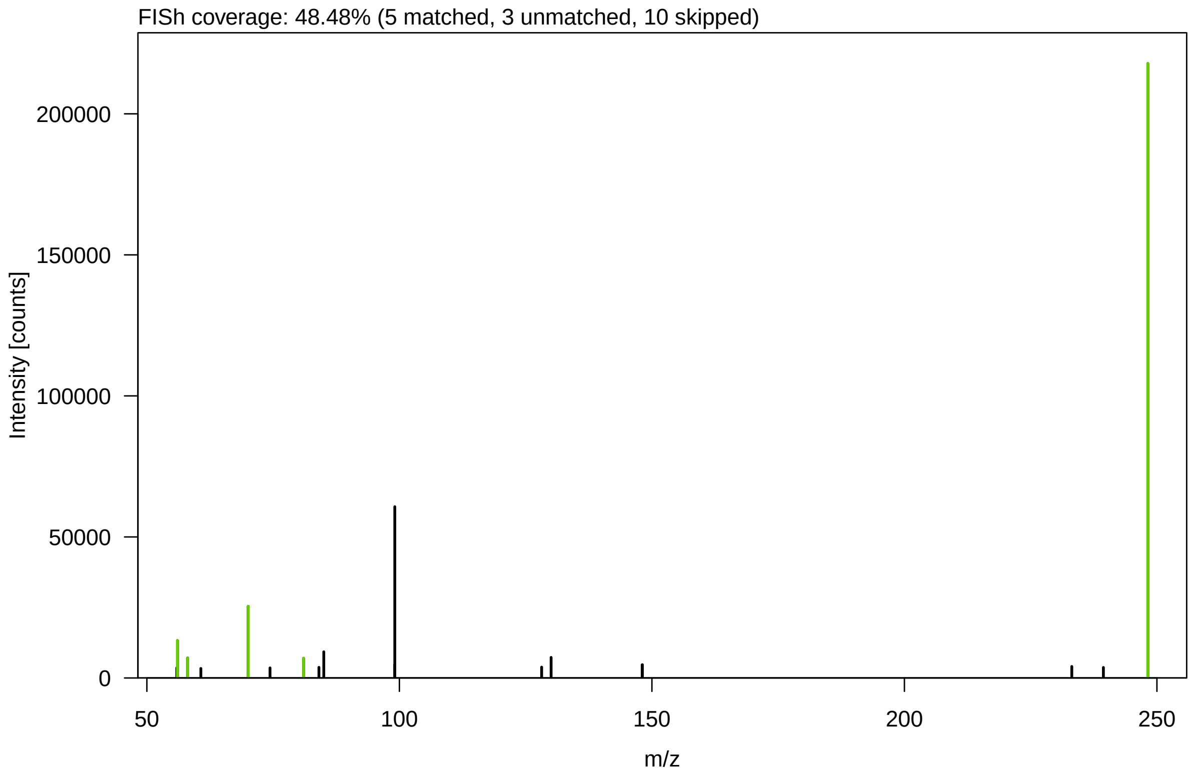

Figure SI-D246: Measured MS2 spectrum. Matching fragments with the tolperisone metabolite predicted by FISh Scoring are highlighted in green. Low intensity fragments are not considered and skipped.

Table SI-D119: Retention time prediction of the tolperisone metabolite.

Measured retention time [min]	11.3
Predicted logD_{OW} (pH = 2.7)	-0.31
Predicted retention time [min]	14.3
Predicted retention time range (95% confidence interval) [min]	9.7-18.9
Predicted retention time range (99% confidence interval) [min]	8.3-20.4

Table SI-D120: Annotated MS2 spectrum of the tolperisone metabolite.

m/z	Relative Intensity	Annotation
55.9209	16.43	
56.0496	60.86	$C_3H_5N + H^+$
58.0653	32.63	$C_3H_7N + H^+$
60.6873	15.49	
70.0652	116.46	$C_4H_7N + H^+$
74.3794	16.41	
81.0335	32.05	$C_5H_4O + H^+$
84.0681	17.27	
85.0282	42.61	
99.0855	22.22	
99.0916	278.48	
128.1702	17.64	
130.0411	33.45	$C_9H_5O + H^+$
148.0751	21.11	$C_9H_9NO + H^+$
148.1119	21.63	$C_{10}H_{13}N + H^+$
233.1421	18.72	
239.4053	17.11	
248.2006	999.00	$C_{16}H_{25}NO + H^+$

No reference standard of tolperisone metabolite was purchasable. Therefore, a human liver S9 incubation experiment with tolperisone was performed, to generate tolperisone metabolites *in vitro*. However, no compound with a precursor matching the one of tolperisone metabolite was detectd within a retention time window of ±3 minutes of the suspect. Correspondingly, the confidence of identification remains at level 3.

SI-D2.12.2 Tolperisone Metabolites M1 and M1’

Based on the obtained information, no differentiation between tolperisone metabolite M1 and M1’ was possible. Consequently, the two metabolites are discussed together.

Table SI-D121: Information on identifiers, chemical properties, detection and confidence of identification of tolperisone metabolites M1 and M1’.

IUPAC Name	1-[4-(hydroxymethyl)phenyl]-2-methyl-3-piperidin-1-ylpropan-1-one<br>1-(3-hydroxy-4-methylphenyl)-2-methyl-3-piperidin-1-ylpropan-1-one
Molecular formula	$C_{16}H_{23}NO_2$
Monoisotopic mass [g/mol]	261.1729
Adduct	$[M+H]^+$
Retention time [min]	11.3
SMILES	CC(CN1CCCCC1)C(=O)C2=CC=C(C=C2)CO<br>CC1=C(C=C(C=C1)C(=O)C(C)CN2CCCCC2)O
InChI	InChI=1S/C16H23NO2/c1-13(11-17-9-3-2-4-10-17)16(19)15-7-5-14(12-18)6-8-15/h5-8,13,18H,2-4,9-12H2,1H3<br>InChI=1S/C16H23NO2/c1-12-6-7-14(10-15(12)18)16(19)13(2)11-17-8-4-3-5-9-17/h6-7,10,13,18H,3-5,8-9,11H2,1-2H3
InChI-Key	MBFHFUBFALKDKH-UHFFFAOYSA-N<br>DPTIOTYHVFZYFS-UHFFFAOYSA-N
CAS RN	-<br>-
Metabolite of	Tolperisone
Detection frequency	100% (15/15 samples)
Detected in	Altenrhein, Monday-Friday<br>Neugut, Monday-Friday<br>Werdhölzli, Monday-Friday
Intensity	E6-E7
Initial confidence level	level 3
Initial confidence score	0.44
Final confidence level	level 2b

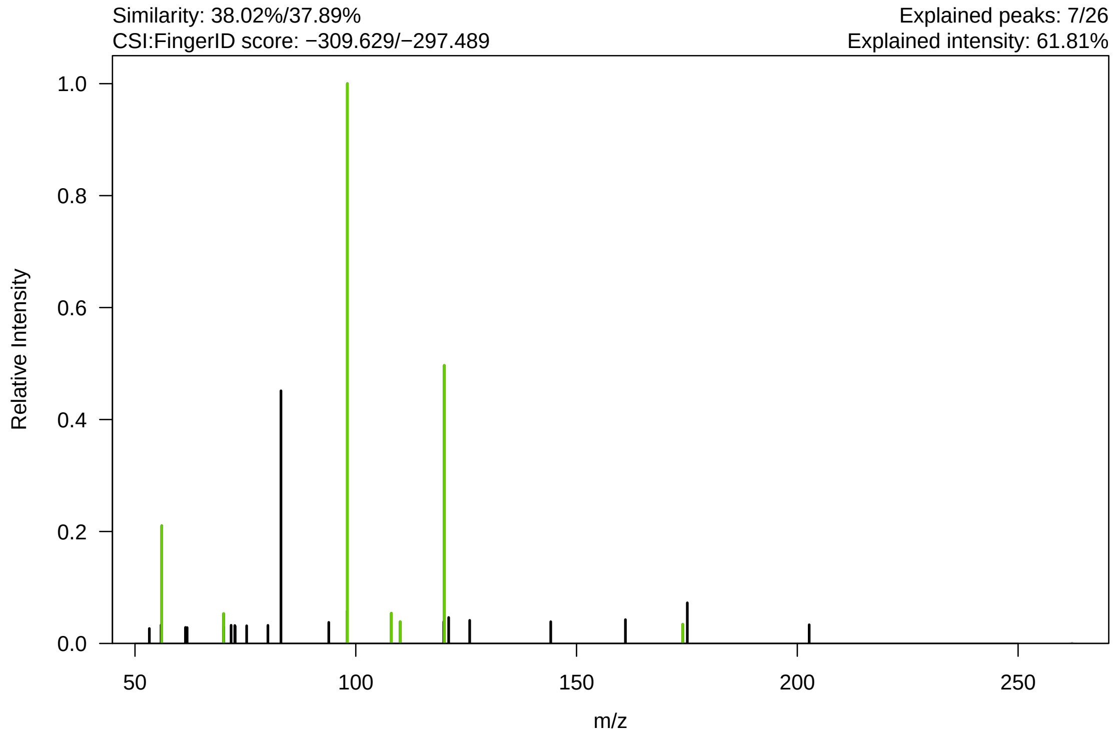

Figure SI-D247: Measured MS2 spectrum. Matching fragments with tolperisone metabolite M1 or M1' predicted by SIRIUS/CSI:FingerID are highlighted in green. The scores refer to tolperisone metabolite M1 and M1', respectively.

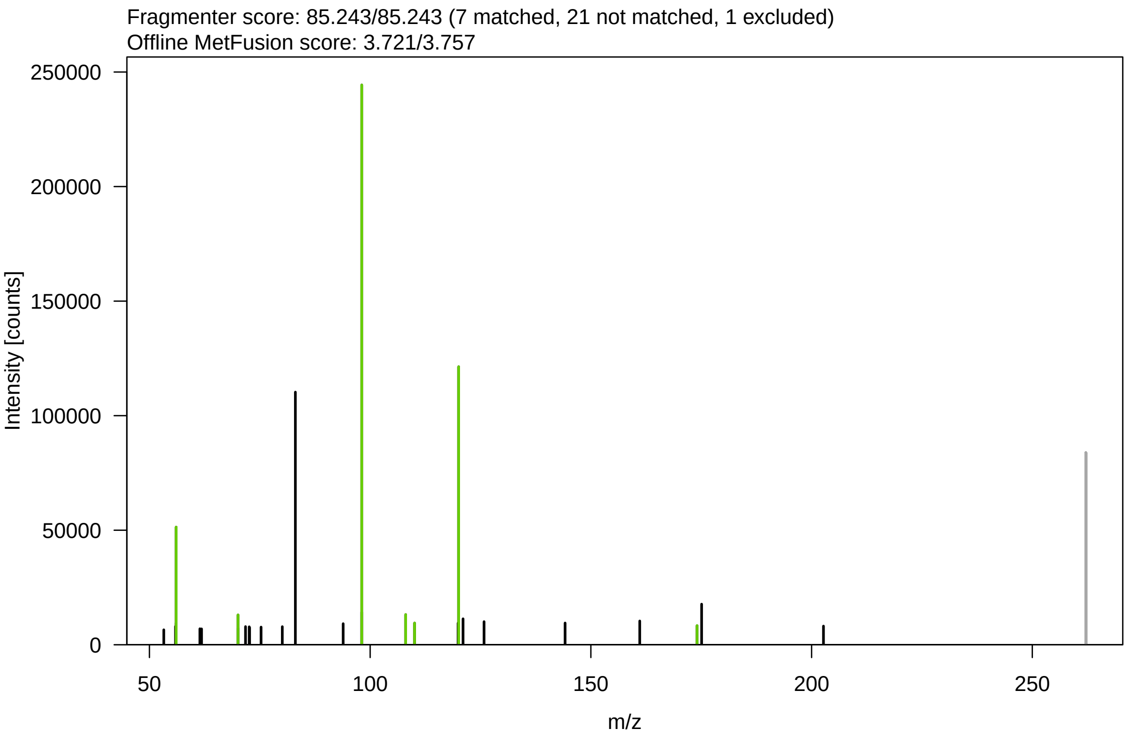

Figure SI-D248: Measured MS2 spectrum. Matching fragments with tolperisone metabolite M1 or M1' predicted by MetFrag are highlighted in green. The scores refer to tolperisone metabolite M1 and M1', respectively. The molecular ion in gray is not considered.

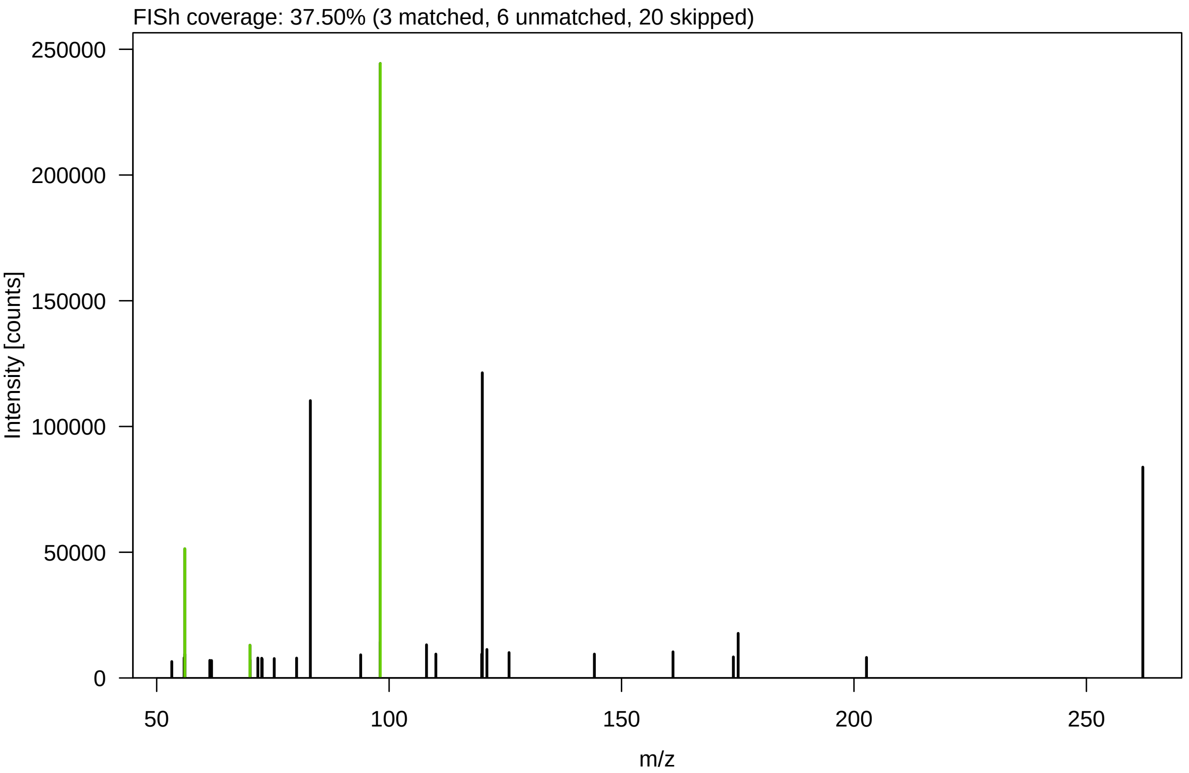

Figure SI-D249: Measured MS2 spectrum. Matching fragments with tolperisone metabolite M1 or M1' predicted by FISh Scoring are highlighted in green. Low intensity fragments are not considered and skipped.

Table SI-D122: Retention time prediction of tolperisone metabolites M1 and M1'.

Measured retention time [min]	11.3
Predicted logD_{OW} (pH = 2.7)	-1.21
Predicted retention time [min]	13.2
Predicted retention time range (95% confidence interval) [min]	8.6-17.8
Predicted retention time range (99% confidence interval) [min]	7.1-19.2
Measured retention time [min]	11.3
Predicted logD_{OW} (pH = 2.7)	-0.24
Predicted retention time [min]	14.4
Predicted retention time range (95% confidence interval) [min]	9.8-19.0
Predicted retention time range (99% confidence interval) [min]	8.4-20.5

Table SI-D123: Annotated MS2 spectrum of tolperisone metabolite M1 and M1', respectively.

m/z	Relative Intensity	Annotation
55.0269	9.90	
55.0295	164.01	$C_2H_2N_2 + H^+$
62.5792	8.01	
67.0258	13.22	
67.0293	223.91	$C_3H_2N_2 + H^+$
69.0412	17.86	
69.0449	348.34	$C_3H_4N_2 + H^+$
70.0287	17.11	$C_3H_3NO + H^+$
81.0448	7.05	$C_4H_4N_2 + H^+$
84.0806	5.98	
86.8323	7.55	
96.0501	11.86	
96.0557	156.39	$C_4H_5N_3 + H^+$
97.0397	40.28	$C_4H_4N_2O + H^+$
97.9960	6.76	
104.7957	6.31	
124.0505	999.00	$C_5H_5N_3O + H^+$
125.8288	7.21	
127.7705	8.34	
142.0615	35.49	$C_5H_7N_3O_2 + H^+$
181.0725	18.29	$C_7H_8N_4O_2 + H^+$
186.0720	7.25	

Since no reference standards of tolperisone metabolite M1 or M1' are commercially available, an incubation experiment was conducted. The human liver S9 incubation of tolperisone led to the formation of tolperisone metabolites M1 or M1'. Considering the spectral match of 0.294 (see Figure SI-D250) and the retention times of 11.3 and 11.5 minutes in the wastewater and the human liver S9 sample, respectively, further confidence could be gained that the detected feature in wastewater is tolperisone metabolite M1 or M1'. Due to this diagnostic evidence, the final confidence level can be increased from level 3 to level 2b.

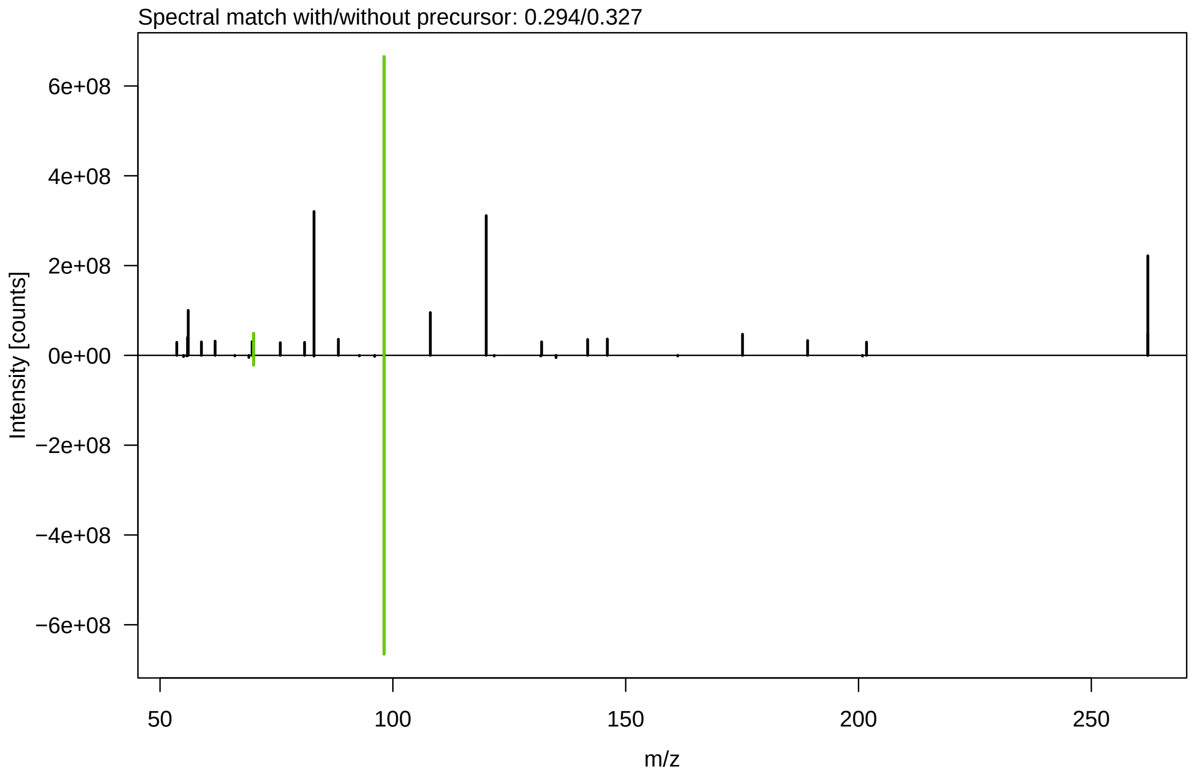

Figure SI-D250: Head to tail plot of tolperisone metabolite M1 or M1' in wastewater (top) and from human liver S9 incubation (bottom). Matching fragments are highlighted in green.

SI-D2.12.3 Tolperisone Metabolite M3

Table SI-D124: Information on identifiers, chemical properties, detection and confidence of identification of tolperisone metabolite M3.

IUPAC Name	4-(2-methyl-3-piperidin-1-ylpropanoyl)benzoic acid
Molecular formula	$C_{16}H_{21}NO_3$
Monoisotopic mass [g/mol]	275.1521
Adduct	$[M+H]^+$
Retention time [min]	12.3
SMILES	CC(CN1CCCCC1)C(=O)C2=CC=C(C=C2)C(=O)O
InChI	InChI=1S/C16H21NO3/c1-12(11-17-9-3-2-4-10-17)15(18)13-5-7-14(8-6-13)16(19)20/h5-8,12H,2-4,9-11H2,1H3,(H,19,20)
InChI-Key	DUFFTQXSWOMYLQ-UHFFFAOYSA-N
CAS RN	-
Metabolite of	Tolperisone
Detection frequency	100% (15/15 samples)
Detected in	Altenrhein, Monday-Friday<br>Neugut, Monday-Friday<br>Werdhölzli, Monday-Friday
Intensity	E7
Initial confidence level	level 3
Initial confidence score	0.49
Final confidence level	level 2b

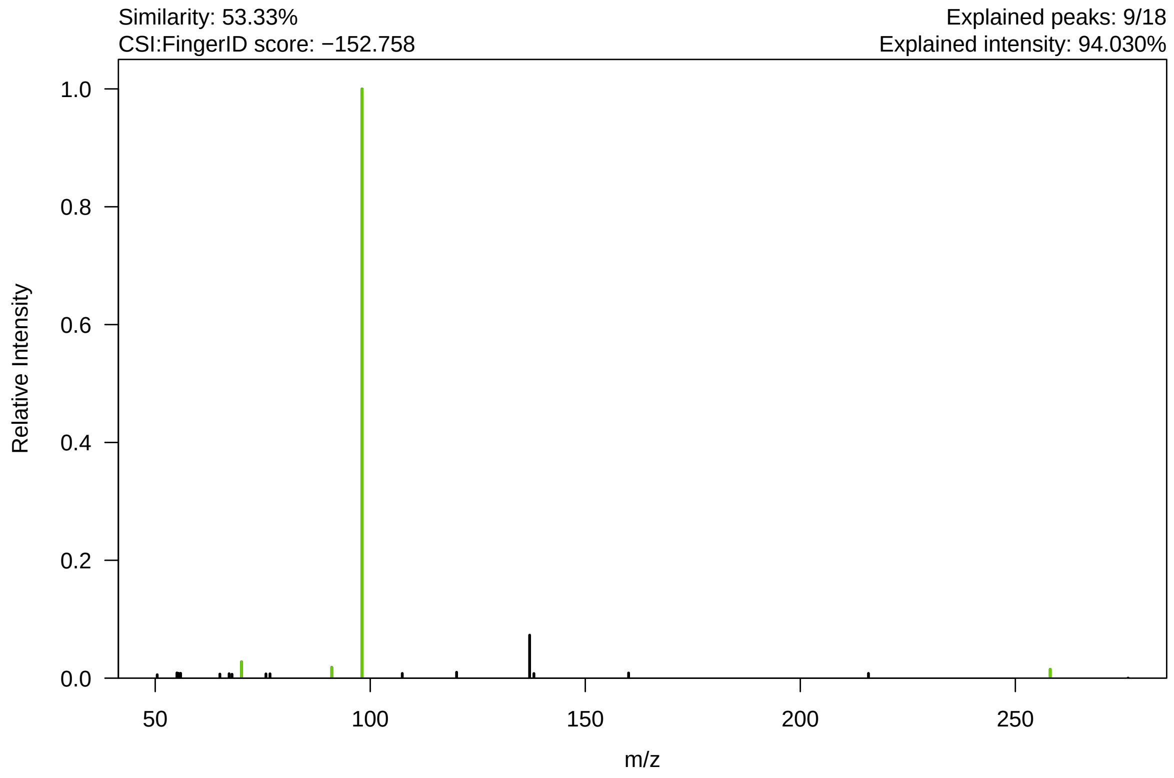

Figure SI-D251: Measured MS2 spectrum. Matching fragments with tolperisone metabolite M3 predicted by SIRIUS/CSI:FingerID are highlighted in green.

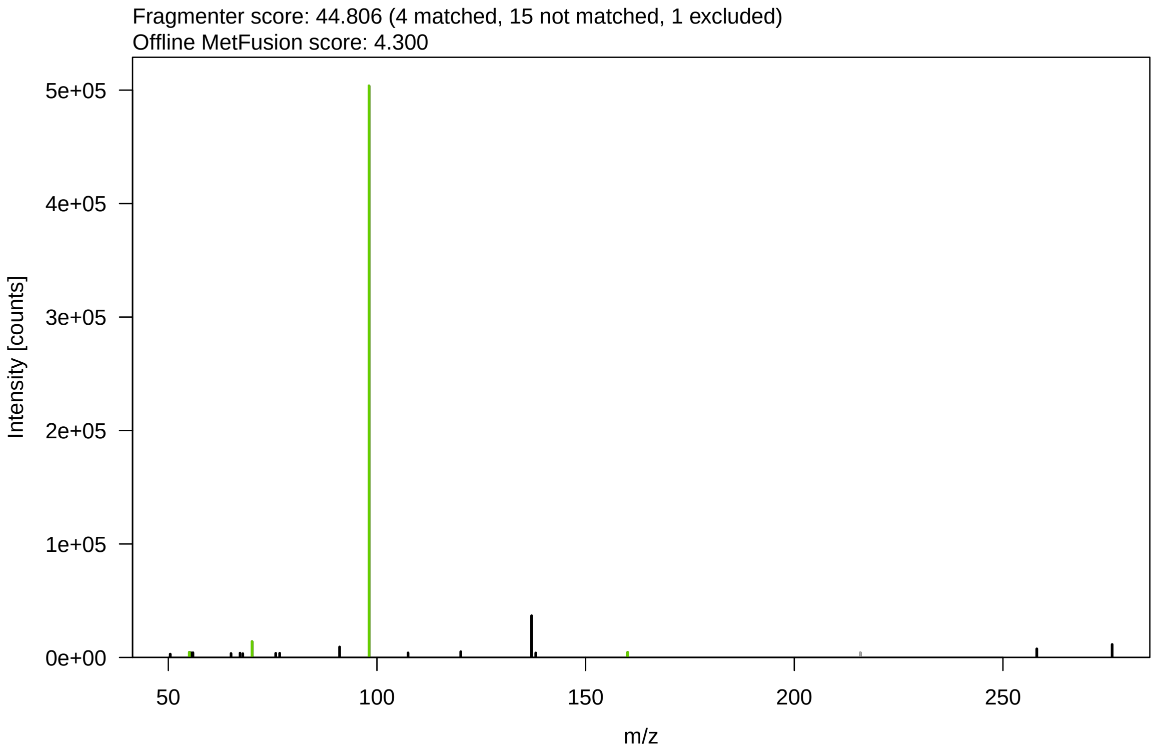

Figure SI-D252: Measured MS2 spectrum. Matching fragments with tolperisone metabolite M3 predicted by MetFrag are highlighted in green.

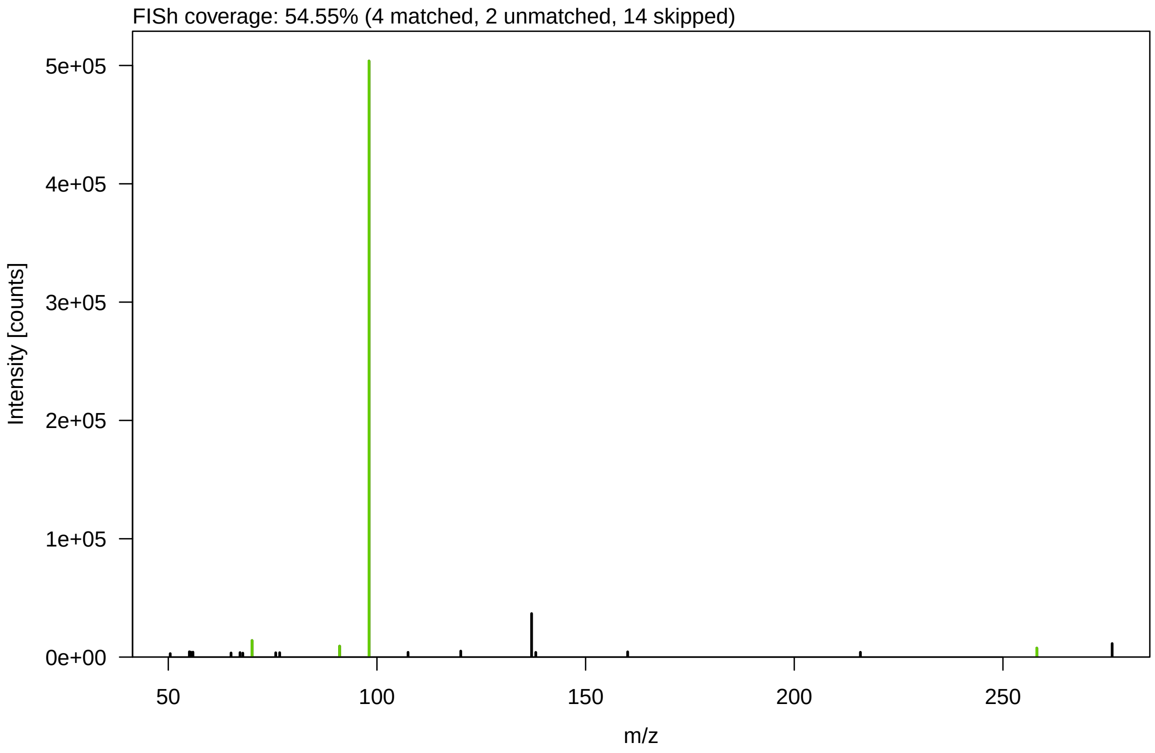

Figure SI-D253: Measured MS2 spectrum. Matching fragments with tolperisone metabolite M3 predicted by FISh Scoring are highlighted in green. Low intensity fragments are not considered and skipped.

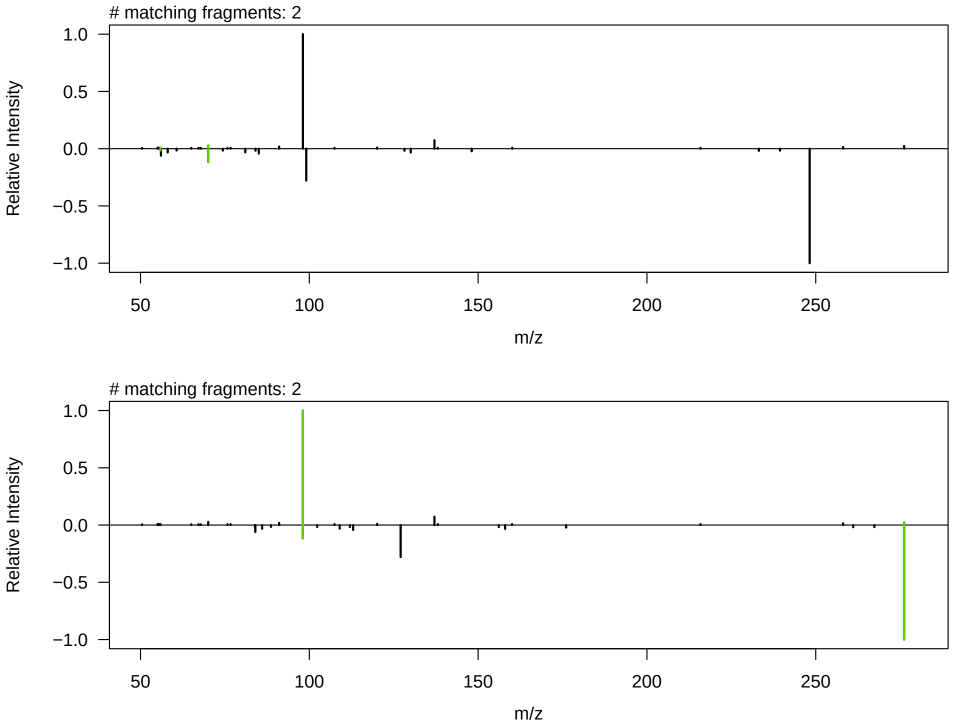

Figure SI-D254: Head to tail plots of tolperisone metabolite M3 and tolperisone metabolite. In the bottom plot, the mass spectrum of tolperisone metabolite is shifted by the mass difference. Matching fragments are highlighted in green.

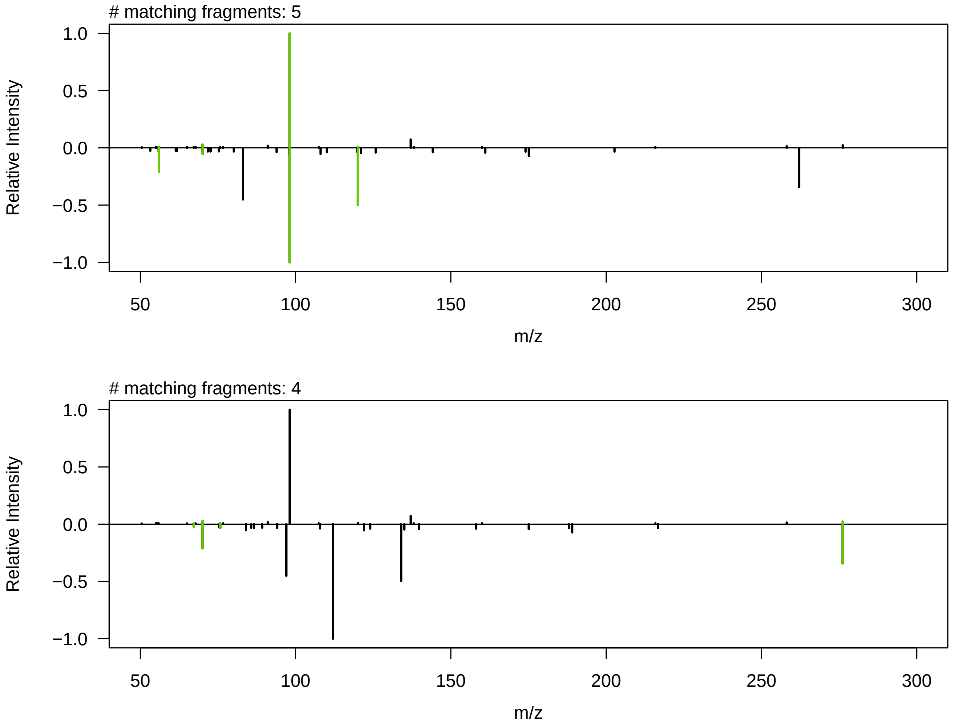

Figure SI-D255: Head to tail plots of tolperisone metabolite M3 and tolperisone metabolite M1. In the bottom plot, the mass spectrum of tolperisone metabolite M1 is shifted by the mass difference. Matching fragments are highlighted in green.

Table SI-D125: Molecular network results and retention time prediction of tolpersione metabolite M3.

Comparison with	Tolperisone metabolite
MSn Score	21
Forward coverage	27
Reverse coverage	15
Forward match	3
Reverse match	5
ΔMass [g/mol]	27.9585
Comparison with	Tolperisone metabolite M1/M1'
MSn Score	32
Forward coverage	38
Reverse coverage	27
Forward match	6
Reverse match	9
ΔMass [g/mol]	13.9796
Measured retention time [min]	12.3
Predicted $\log D_{OW}$ (pH = 2.7)	-0.59
Predicted retention time [min]	14.0
Predicted retention time range (95% confidence interval) [min]	9.4-18.6
Predicted retention time range (99% confidence interval) [min]	7.9-20.0

Table SI-D126: Annotated MS2 spectrum of tolperisone metabolite M3.

m/z	Relative Intensity	Annotation
50.4654	5.81	
55.0545	8.91	$C_4H_6 + H^+$
55.4067	8.32	
55.9062	8.28	
65.0352	6.93	
67.1873	7.52	
67.8561	6.63	
70.0651	27.70	$C_4H_7N + H^+$
75.7464	7.26	
76.6898	7.37	
91.0541	18.33	$C_7H_6 + H^+$
98.0964	999.00	$C_6H_{11}N + H^+$
107.4504	7.98	
120.0807	10.03	$C_8H_9N + H^+$
137.0597	72.94	$C_8H_8O_2 + H^+$
138.0544	7.86	$C_7H_7NO_2 + H^+$
160.0761	8.76	$C_{10}H_9NO + H^+$
215.8476	8.08	
258.1122	15.03	$C_{15}H_{15}NO_3 + H^+$
276.1704	22.66	$C_{16}H_{21}NO_3 + H^+$

Since no reference standard of tolperisone metabolite M3 is commercially available, an incubation experiment was conducted. The human liver S9 incubation of tolperisone led to the formation of tolperisone metabolite M3. Considering the spectral match of 0.809 (see Figure SI-D256) and the retention times of 12.3 and 12.4 minutes in the wastewater and the human liver S9 sample, respectively, further confidence could be gained that the detected feature in wastewater is tolperisone metabolite M3. Due to this diagnostic evidence, the final confidence level can be increased from level 3 to level 2b.

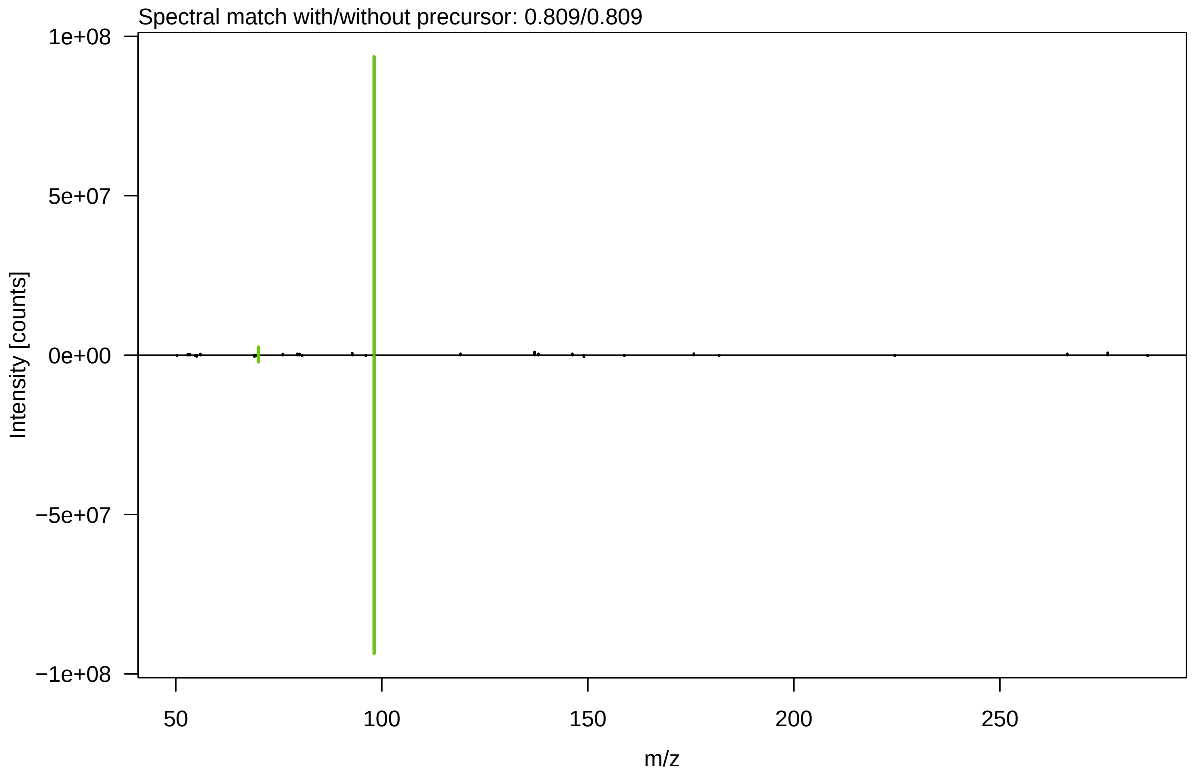

Figure SI-D256: Head to tail plot of tolperisone metabolite M3 in wastewater (top) and from human liver S9 incubation (bottom). Matching fragments are highlighted in green.

SI-D2.12.4 Tolperisone Metabolite M4

Table SI-D127: Information on identifiers, chemical properties, detection and confidence of identification of tolperisone metabolite M4.

IUPAC Name	4-(1-hydroxy-2-methyl-3-piperidin-1-ylpropyl)benzoic acid
Molecular formula	$C_{16}H_{23}NO_3$
Monoisotopic mass [g/mol]	277.1678
Adduct	$[M+H]^+$
Retention time [min]	11.1
SMILES	CC(CN1CCCCC1)C(C2=CC=C(C=C2)C(=O)O)O
InChI	InChI=1S/C16H23NO3/c1-12(11-17-9-3-2-4-10-17)15(18)13-5-7-14(8-6-13)16(19)20/h5-8,12,15,18H,2-4,9-11H2,1H3,(H,19,20)
InChI-Key	GQXHANPQZCYING-UHFFFAOYSA-N
CAS RN	-
Metabolite of	Tolperisone
Detection frequency	100% (15/15 samples)
Detected in	Altenrhein, Monday-Friday<br>Neugut, Monday-Friday<br>Werdhölzli, Monday-Friday
Intensity	E7
Initial confidence level	level 3
Initial confidence score	0.52
Final confidence level	level 2b

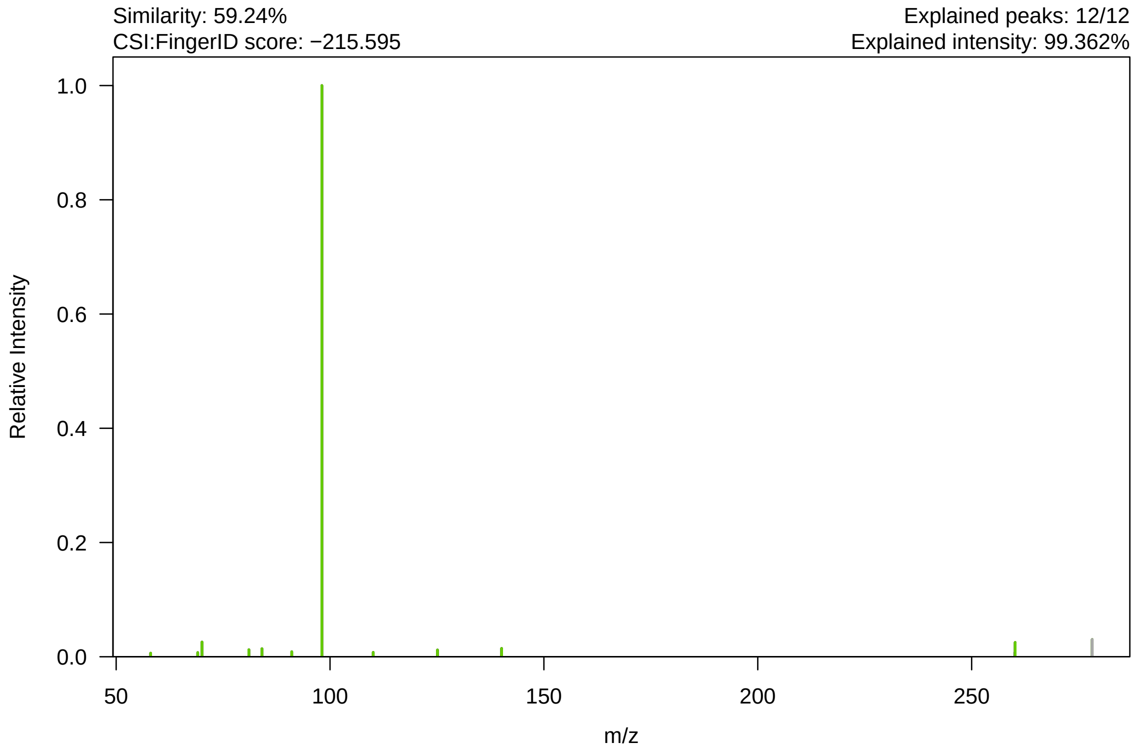

Figure SI-D257: Measured MS2 spectrum. Matching fragments with tolperisone metabolite M4 predicted by SIRIUS/CSI:FingerID are highlighted in green. The molecular ion in gray is not considered.

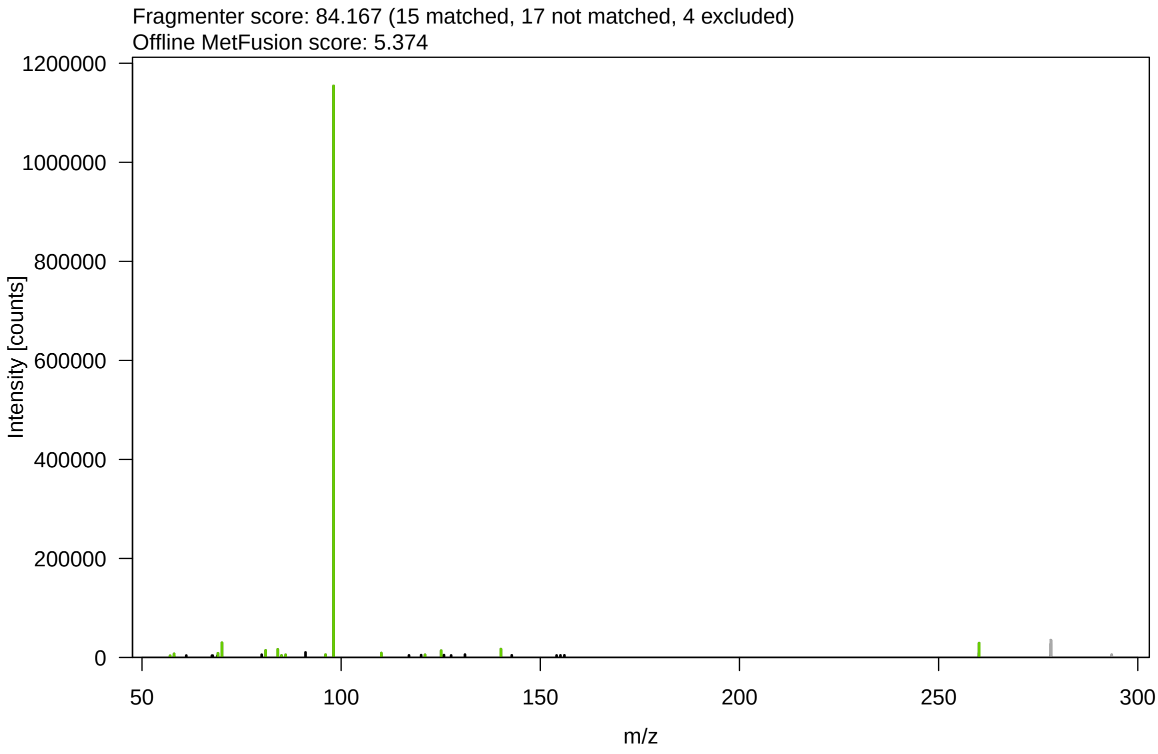

Figure SI-D258: Measured MS2 spectrum. Matching fragments with tolperisone metabolite M4 predicted by MetFrag are highlighted in green. The molecular ion in gray is not considered.

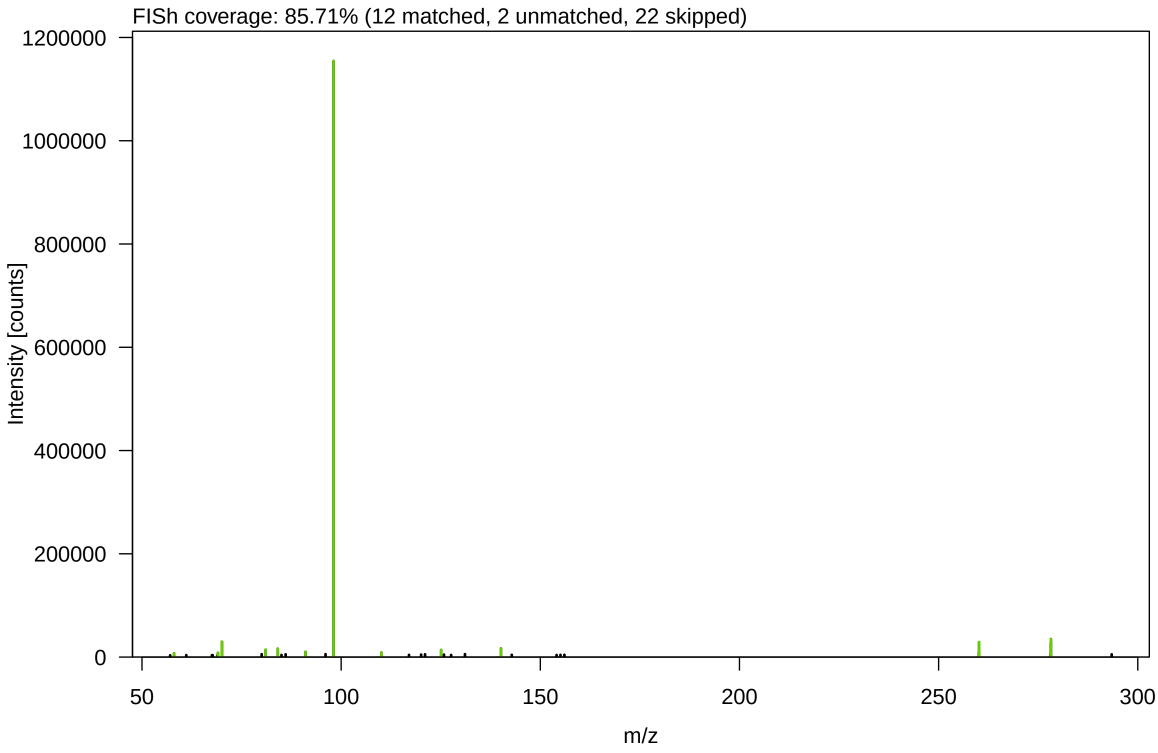

Figure SI-D259: Measured MS2 spectrum. Matching fragments with tolperisone metabolite M4 predicted by FISh Scoring are highlighted in green. Low intensity fragments are not considered and skipped.

Table SI-D128: Molecular network results and retention time prediction of tolpersione metabolite M4.

Comparison with	Tolperisone metabolite
MSn Score	38
Forward coverage	33
Reverse coverage	43
Forward match	11
Reverse match	6
ΔMass [g/mol]	29.9742
Comparison with	Tolperisone metabolite M3
MSn Score	33
Forward coverage	21
Reverse coverage	45
Forward match	3
Reverse match	5
ΔMass [g/mol]	2.0157
Measured retention time [min]	11.1
Predicted $logD_{OW}$ (pH = 2.7)	-1.05
Predicted retention time [min]	13.4
Predicted retention time range (95% confidence interval) [min]	8.8-18.0
Predicted retention time range (99% confidence interval) [min]	7.3-19.4

Table SI-D129: Annotated MS2 spectrum of tolperisone metabolite M4.

m/z	Relative Intensity	Annotation
57.0701	3.01	$C_4H_8 + H^+$
58.0654	6.26	$C_3H_7N + H^+$
58.1030	2.77	
61.1222	3.35	
67.5554	3.15	
67.7476	3.27	
68.9389	3.49	
69.0699	7.27	$C_5H_8 + H^+$
70.0653	25.73	$C_4H_7N + H^+$
80.0494	4.83	
81.0334	12.34	$C_5H_4O + H^+$
84.0807	14.12	$C_5H_9N + H^+$
85.0283	3.54	$C_4H_4O_2 + H^+$
86.0601	4.63	C_4H_7NO + H≡;
91.0543	8.84	$C_7H_6 + H^+$
96.0808	4.90	C_6H_9N + H≡;
98.0964	999.00	$C_6H_{11}N + H^+$
110.0962	7.68	$C_7H_{11}N + H^+$

Continued on next page

Table SI-D129: Annotated MS2 spectrum of tolperisone metabolite M4.(Continued)

117.0321	3.68	
120.0806	4.15	
121.0650	4.60	$C_8H_8O + H^+$
125.1199	11.87	$C_8H_{14}N + H^+$
125.8124	4.19	
127.6159	3.56	
131.0855	4.99	
140.1072	14.57	$C_8H_{13}NO + H^+$
142.8346	3.79	
154.0883	3.53	
155.0590	3.69	
156.0491	3.82	
260.1230	7.27	
260.1647	25.01	$C_{16}H_{21}NO_2 + H^+$
278.1081	3.83	
278.1396	23.96	
278.1764	30.20	$C_{16}H_{23}NO_3 + H^+$
293.4459	4.57	

Since no reference standard of tolperisone metabolite M4 is commercially available, an incubation experiment was conducted. The human liver S9 incubation of tolperisone led to the formation of tolperisone metabolite M4. Considering the spectral match of 0.795 (see Figure SI-D260) and the retention times of 11.1 and 11.4 minutes in the wastewater and the human liver S9 sample, respectively, further confidence could be gained that the detected feature in wastewater is tolperisone metabolite M4. Due to this diagnostic evidence, the final confidence level can be increased from level 3 to level 2b.

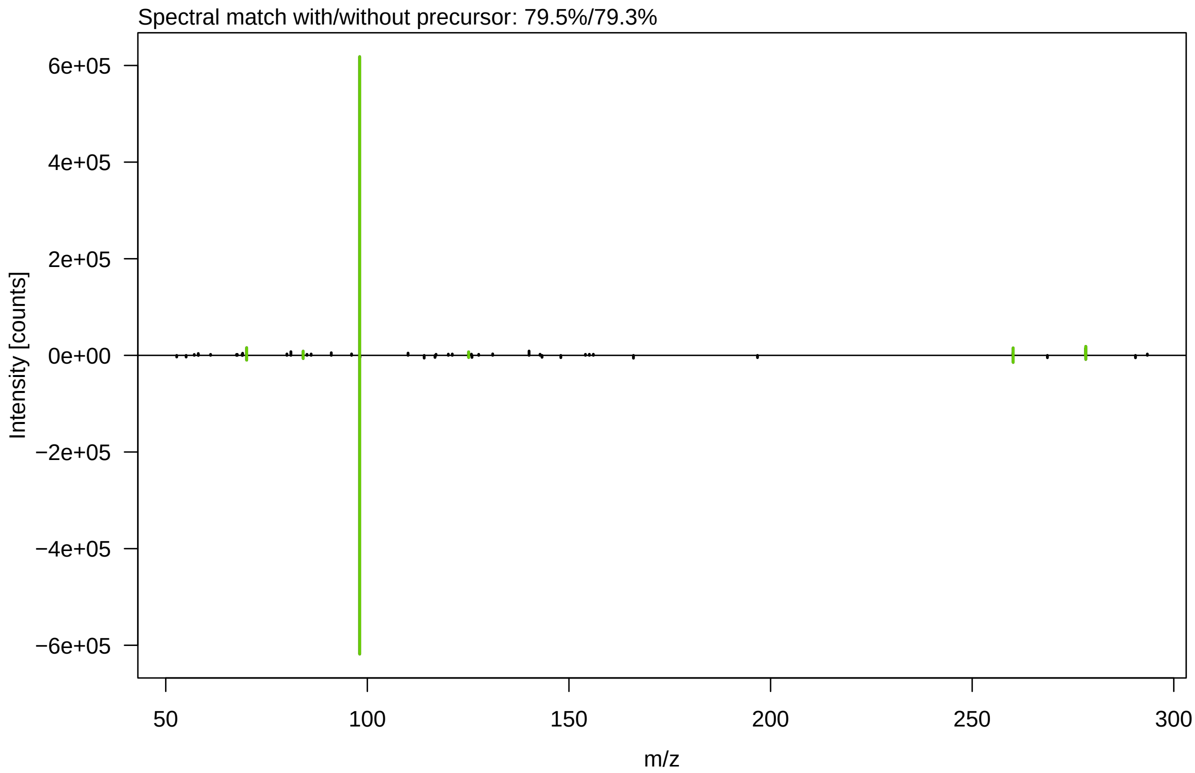

Figure SI-D260: Head to tail plot of tolperisone metabolite M4 in wastewater (top) and from human liver S9 incubation (bottom). Matching fragments are highlighted in green.

SI-D2.13 Torasemide Metabolites

Torasemide is an anilinopyridine sulphonylurea and acts as a diuretic.[17] During suspect screening, three human metabolites could be identified with the aid of molecular networking. The identified metabolites are highlighted in the metabolism scheme of torasemide in Figure SI-D261. An excerpt of the molecular network showing the torasemide metabolites cluster is shown in Figure SI-D262. The following subsections give more details on the individual metabolites.

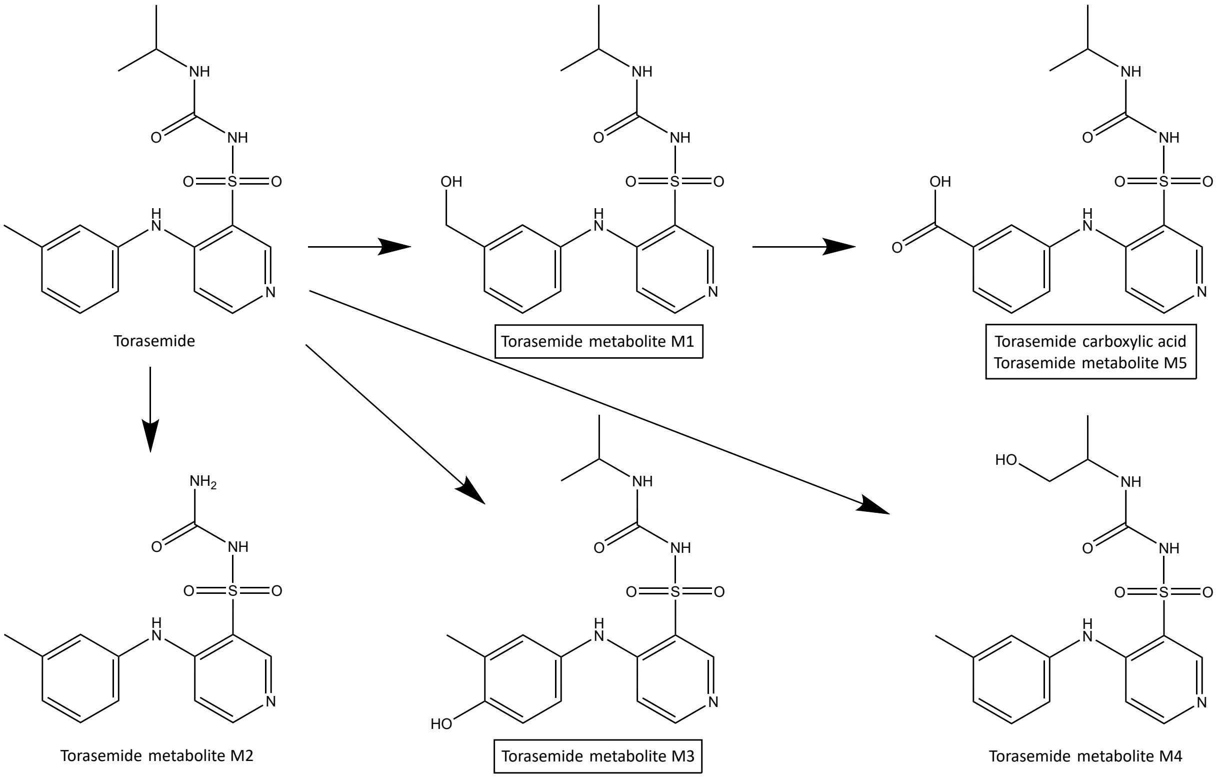

Figure SI-D261: Human metabolism of torasemide. Framed metabolites were identified during suspect screening. Scheme adapted from.[17]

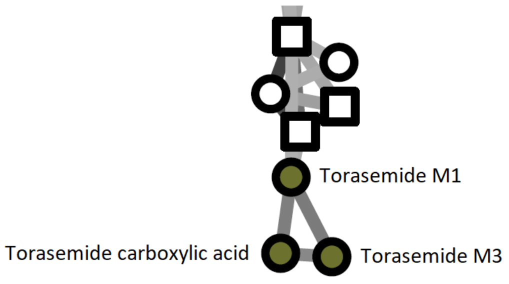

Figure SI-D262: Excerpt of the molecular network showing the torasemide metabolites cluster.

SI-D2.13.1 Torasemide Carboxylic Acid

Table SI-D130: Information on identifiers, chemical properties, detection and confidence of identification of torasemide carboxylic acid.

IUPAC Name	3-[[3-(propan-2-ylcarbamoylsulfamoyl)pyridin-4-yl]amino]benzoic acid
Molecular formula	$C_{16}H_{18}N_4O_5S$
Monoisotopic mass [g/mol]	378.0998
Adduct	$[M+H]^+$
Retention time [min]	14.4
SMILES	CC(C)NC(=O)NS(=O)(=O)C1=C(C=CN=C1)NC2=CC=CC(=C2)C(=O)O
InChI	InChI=1S/C16H18N4O5S/c1-10(2)18-16(23)20-26(24,25)14-9-17-7-6-13(14)19-12-5-3-4-11(8-12)15(21)22/h3-10H,1-2H3,(H,17,19)(H,21,22)(H2,18,20,23)
InChI-Key	PGPRBNDLCZQUST-UHFFFAOYSA-N
CAS RN	-
Metabolite of	Torasemide
Detection frequency	100% (15/15 samples)
Detected in	Altenrhein, Monday-Friday<br>Neugut, Monday-Friday<br>Werdhölzli, Monday-Friday
Intensity	E7
Initial confidence level	level 3
Initial confidence score	0.38
Final confidence level	level 1

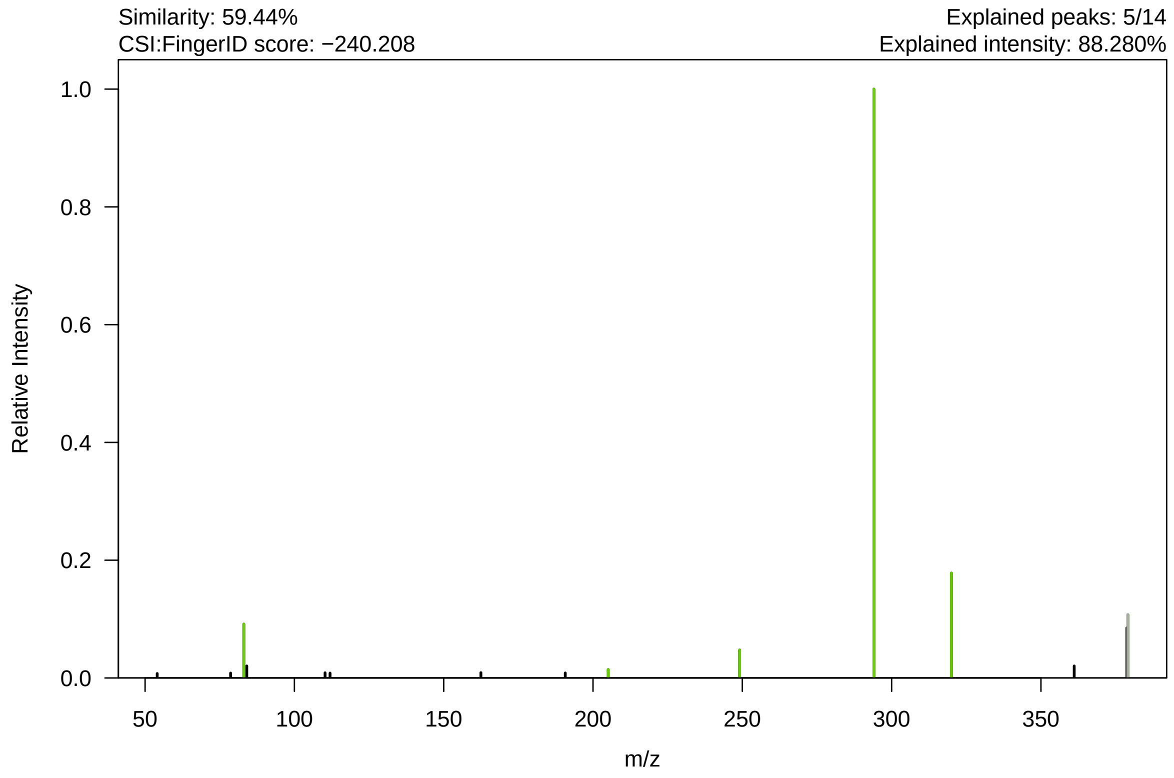

Figure SI-D263: Measured MS2 spectrum. Matching fragments with torasemide carboxylic acid predicted by SIRIUS/CSI:FingerID are highlighted in green. The molecular ion in gray is not considered.

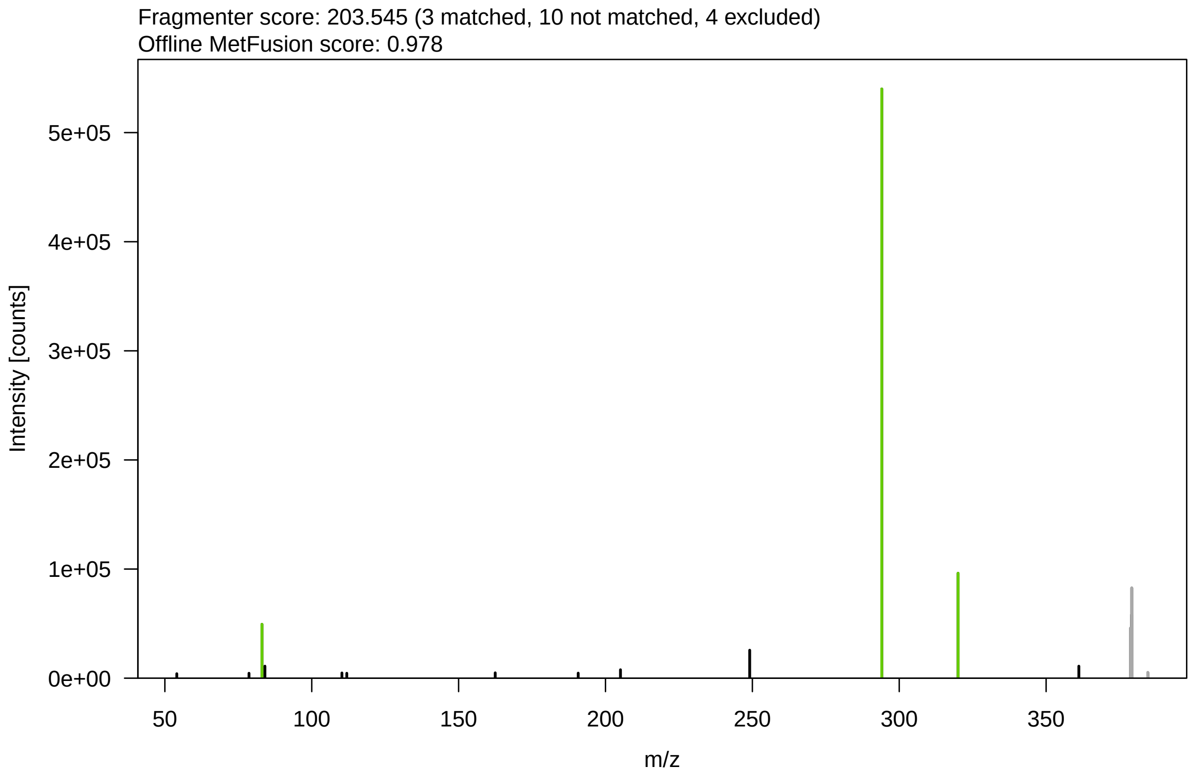

Figure SI-D264: Measured MS2 spectrum. Matching fragments with torasemide carboxylic acid predicted by MetFrag are highlighted in green. The molecular ion in gray is not considered.

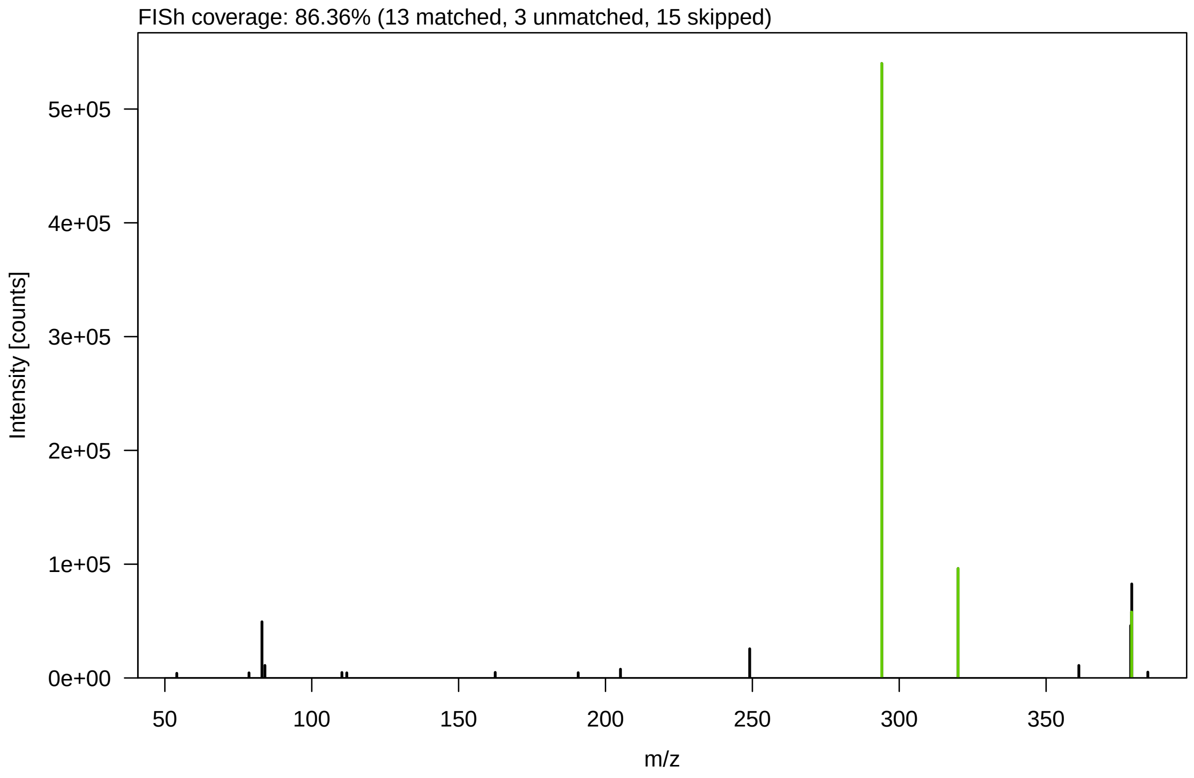

Figure SI-D265: Measured MS2 spectrum. Matching fragments with torasemide carboxylic acid predicted by FISh Scoring are highlighted in green.

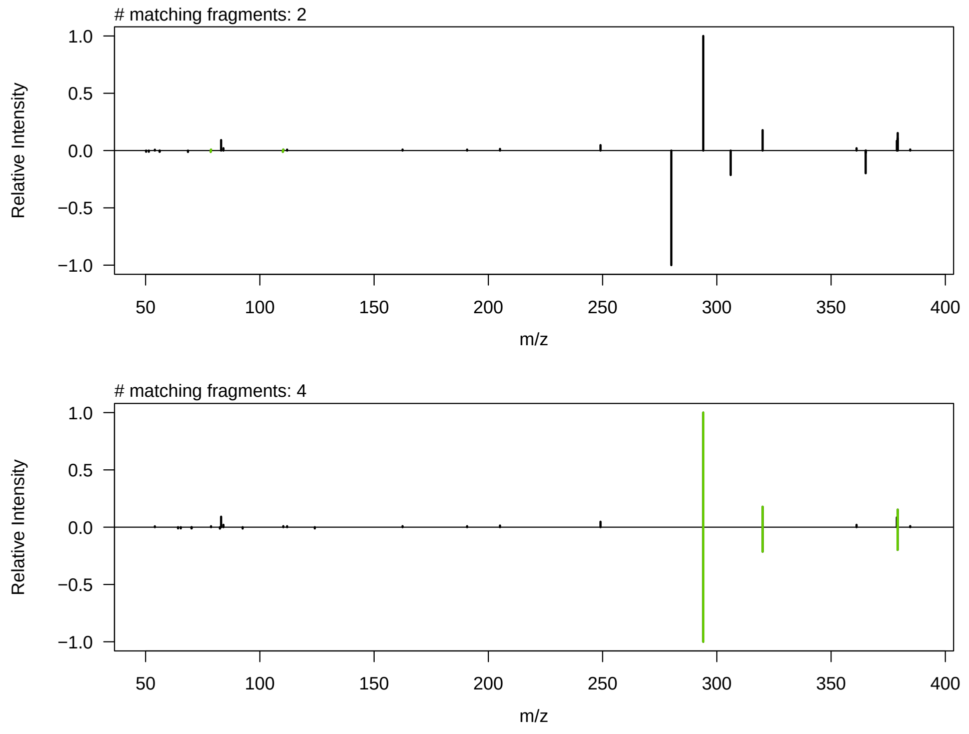

Figure SI-D266: Head to tail plots of torasemide carboxylic acid and torasemide metabolite M1. In the bottom plot, the mass spectrum of torasemide metabolite M1 is shifted by the mass difference. Matching fragments are highlighted in green.

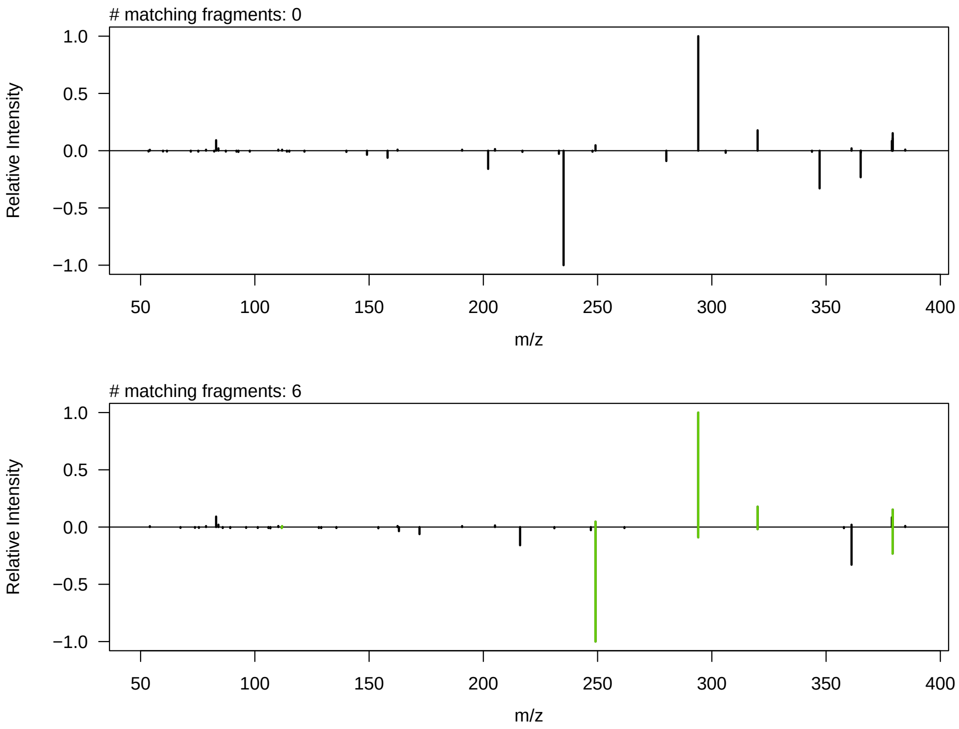

Figure SI-D267: Head to tail plots of torasemide carboxylic acid and torasemide metabolite M3. In the bottom plot, the mass spectrum of torasemide metabolite M3 is shifted by the mass difference. Matching fragments are highlighted in green.

Table SI-D131: Molecular network results and retention time prediction of torasemide caboxylic acid.

Comparison with	Torasemide metabolite M1
MSn Score	52
Forward coverage	71
Reverse coverage	33
Forward match	5
Reverse match	6
ΔMass [g/mol]	13.9793
Comparison with	Torasemide metabolite M3
MSn Score	47
Forward coverage	50
Reverse coverage	44
Forward match	5
Reverse match	8
ΔMass [g/mol]	13.9793
Measured retention time [min]	14.4
Predicted $\log D_{OW}$ (pH = 2.7)	0.22
Predicted retention time [min]	15.0
Predicted retention time range (95% confidence interval) [min]	10.4-19.6
Predicted retention time range (99% confidence interval) [min]	9.0-21.1

Table SI-D132: Annotated MS2 spectrum of torasemide carboxylic acid.

m/z	Relative Intensity	Annotation
54.0562	7.57	
78.6388	8.32	
83.0492	91.36	$C_5H_6O + H^+$
84.0526	20.40	
110.2696	8.85	
111.9318	8.33	
162.4556	9.05	
190.7096	8.54	
205.1003	14.24	$C_8H_{16}N_2O_2S + H^+$
249.0910	47.43	$C_9H_{16}N_2O_4S + H^+$
294.0541	999.00	$C_{12}H_{11}N_3O_4S + H^+$
320.0333	177.95	$C_{13}H_9N_3O_5S + H^+$
361.1415	20.37	
378.7804	85.13	
379.1061	107.29	$C_{16}H_{18}N_4O_5S + H^+$
379.1518	152.84	
384.6277	9.51	

The human liver S9 incubation of torasemide led to the formation of torasemide carboxylic acid. Considering the spectral match of 0.596 (see Figure SI-D268) and the retention times of 14.4 and 14.6 minutes in the wastewater and the human liver S9 sample, respectively, further confidence could be gained that the detected feature in wastewater is torasemide carboxylic acid. Due to this diagnostic evidence, the final confidence level can be increased from level 3 to level 2b.

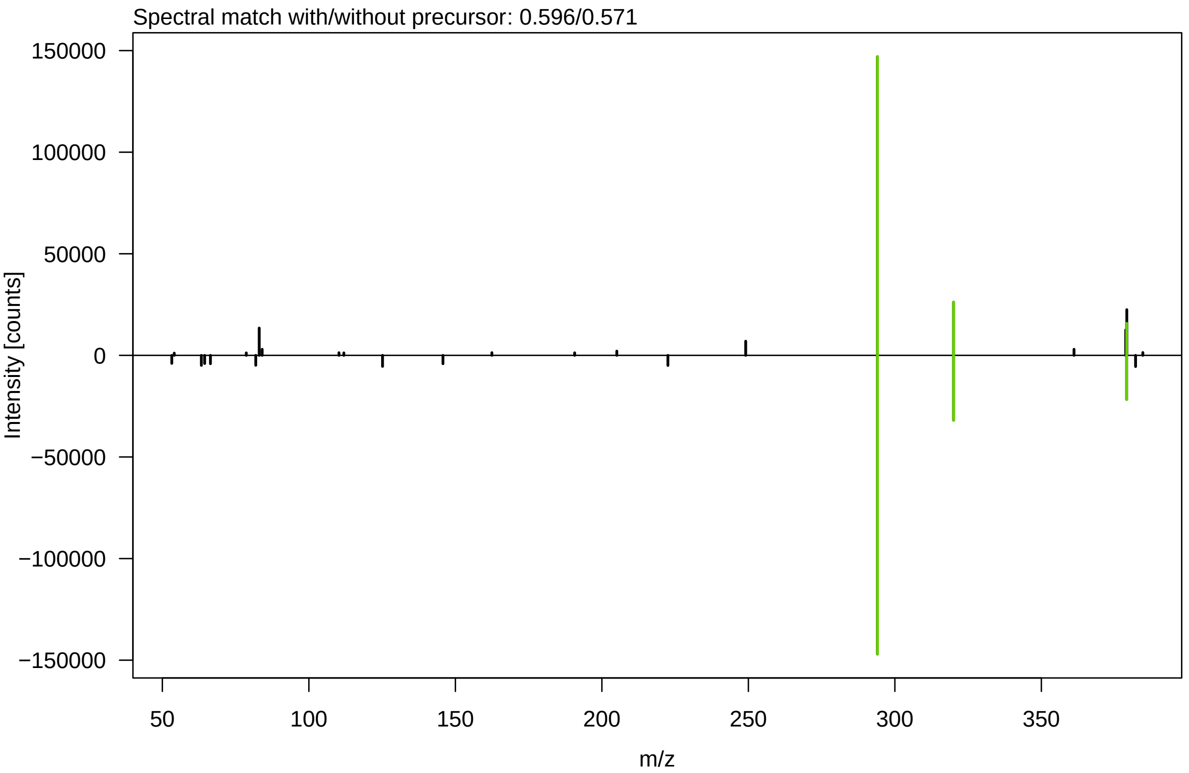

Figure SI-D268: Head to tail plot of torasemide carboxlic acid in wastewater (top) and from human liver S9 incubation (bottom). Matching fragments are highlighted in green.

In addition to the human liver S9 incubation experiment, a reference standard of torasemide carboxylic acid was purchased. Figure SI-D269 shows the extracted ion chromatograms of this standard, the sample and the spiked sample, as well as a head to tail plot of the MS2 spectra of the standard and the sample. In addition, the most intense MS2 fragments in the sample and in the standard are displayed. It becomes visible that the retention times of the sample and the spiked sample are identical and the spectra similarity score between sample and standard is equal to 0.755. Several MS2 fragments in the sample can be explained by the reference standard. It can therefore be concluded that the suspected compound is indeed torasemide carboxylic acid. Correspondingly, the identification confidence can be increased to level 1.

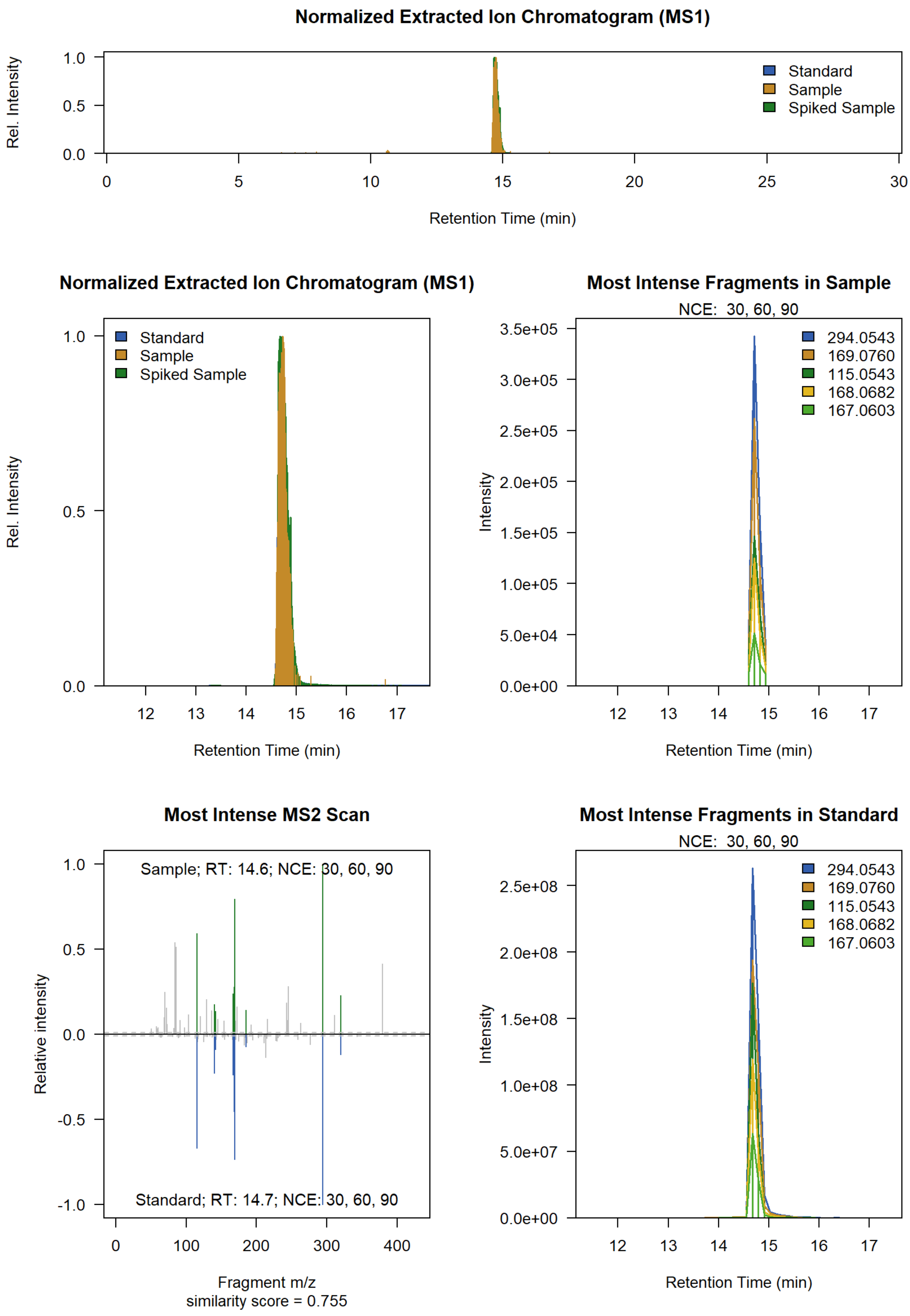

Figure SI-D269: Extracted ion chromatograms of torasemide carboxylic acid in the reference standard, the sample and the spiked sample, as well as MS2 head to tail plot and most intense MS2 fragments in standard and sample.

SI-D2.13.2 Torasemide Metabolite M1

Table SI-D133: Information on identifiers, chemical properties, detection and confidence of identification of torasemide metabolite M1.

IUPAC Name	1-[4-[3-(hydroxymethyl)anilino]pyridin-3-yl]sulfonyl-3-propan-2-ylurea
Molecular formula	$C_{16}H_{20}N_4O_4S$
Monoisotopic mass [g/mol]	364.1205
Adduct	$[M+H]^+$
Retention time [min]	13.6
SMILES	CC(C)NC(=O)NS(=O)(=O)C1=C(C=CN=C1)NC2=CC=CC(=C2)CO
InChI	InChI=1S/C16H20N4O4S/c1-11(2)18-16(22)20-25(23,24)15-9-17-7-6-14(15)19-13-5-3-4-12(8-13)10-21/h3-9,11,21H,10H2,1-2H3,(H,17,19)(H2,18,20,22)
InChI-Key	WCYVLAMJCQZUCR-UHFFFAOYSA-N
CAS RN	99300-68-2
Metabolite of	Torasemide
Detection frequency	100% (15/15 samples)
Detected in	Altenrhein, Monday-Friday<br>Neugut, Monday-Friday<br>Werdhölzli, Monday-Friday
Intensity	E6-E7
Initial confidence level	level 3
Initial confidence score	0.32
Final confidence level	level 2b

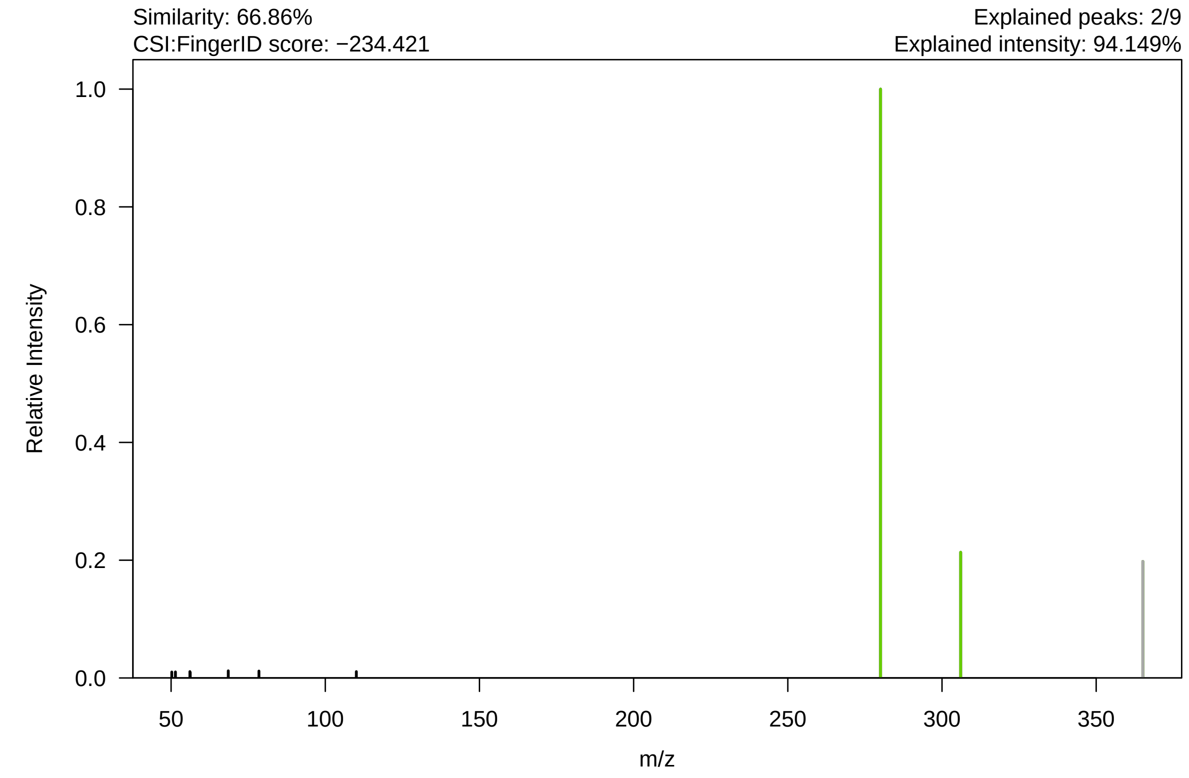

Figure SI-D270: Measured MS2 spectrum. Matching fragments with torasemide metabolite M1 predicted by SIRIUS/CSI:FingerID are highlighted in green. The molecular ion in gray is not considered.

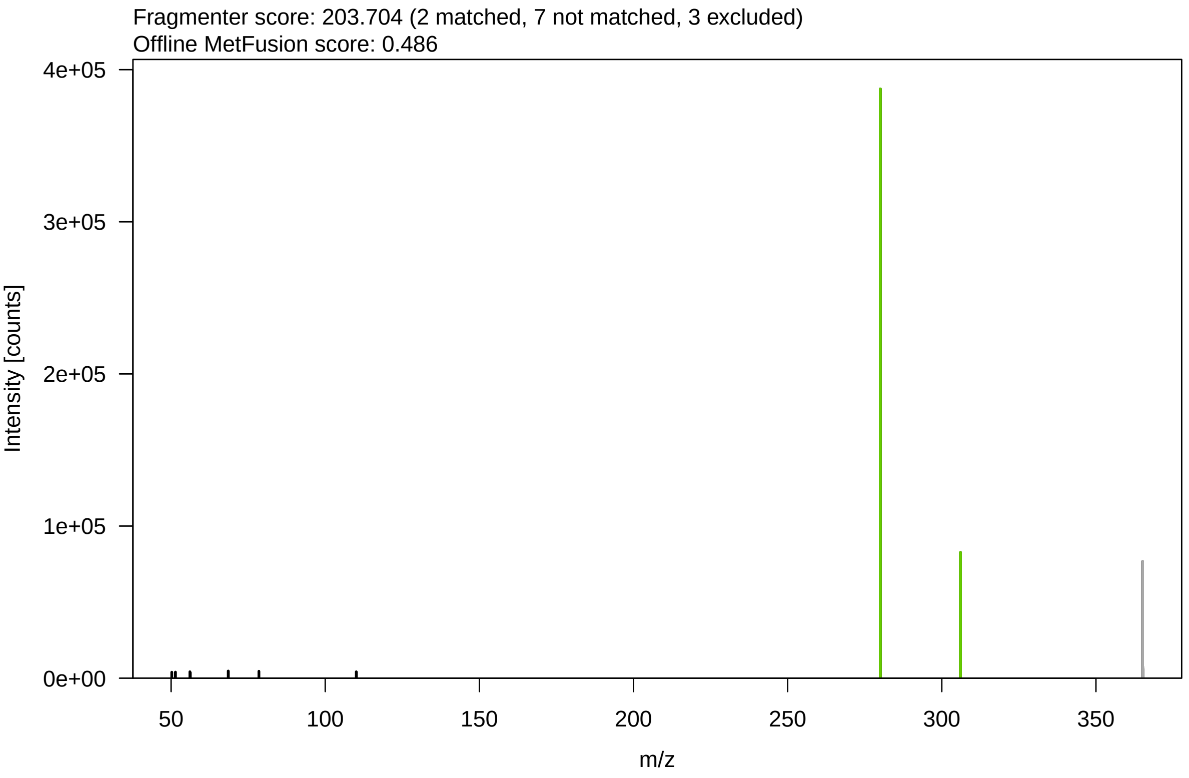

Figure SI-D271: Measured MS2 spectrum. Matching fragments with torasemide metabolite M1 predicted by MetFrag are highlighted in green. The molecular ion in gray is not considered.

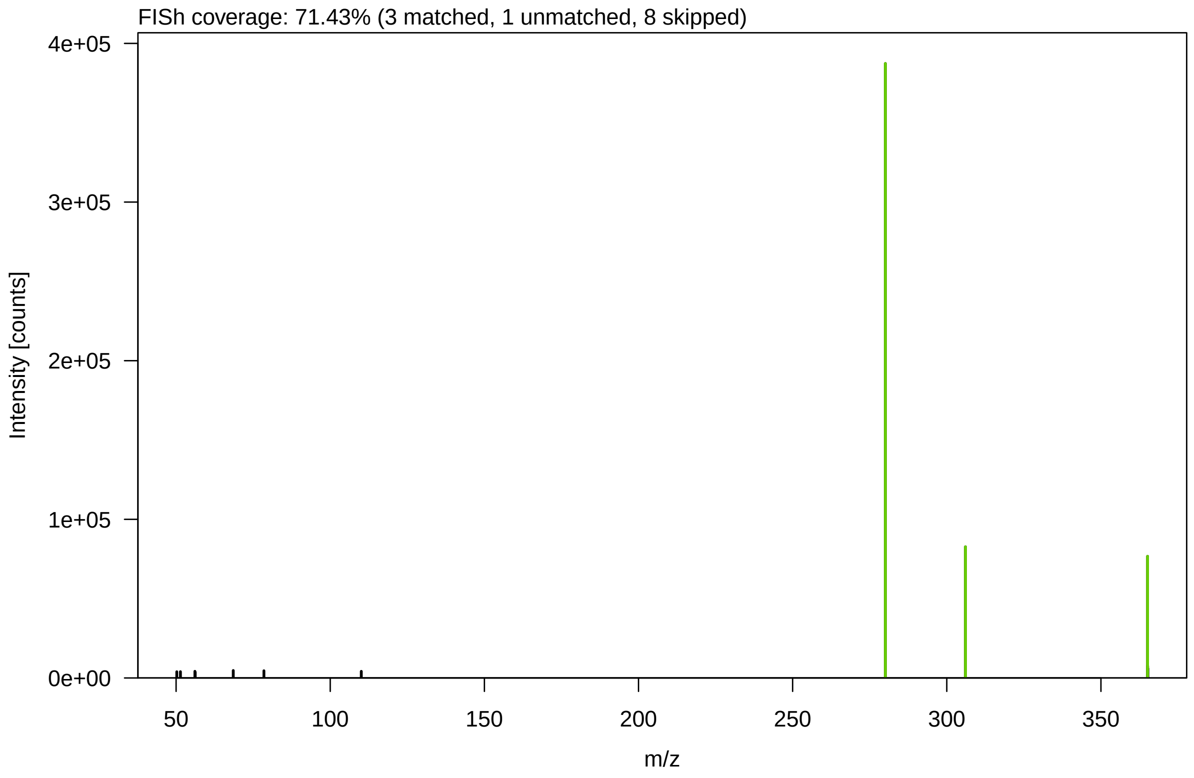

Figure SI-D272: Measured MS2 spectrum. Matching fragments with torasemide metabolite M1 predicted by FISh Scoring are highlighted in green.

Table SI-D134: Retention time prediction of torasemide metabolite M1.

Measured retention time [min]	13.6
Predicted $logD_{OW}$ (pH = 2.7)	-0.20
Predicted retention time [min]	14.5
Predicted retention time range (95% confidence interval) [min]	9.9-19.1
Predicted retention time range (99% confidence interval) [min]	8.4-20.5

Table SI-D135: Annotated MS2 spectrum of torasemide metabolite M1.

m/z	Relative Intensity	Annotation
50.2242	10.14	
51.4092	10.27	
56.0988	10.84	
56.2014	9.40	
68.5429	12.14	
78.4874	11.75	
110.0719	10.80	
280.0747	999.00	$C_{12}H_{13}N_3O_3S + H^+$

Continued on next page

Table SI-D135: Annotated MS2 spectrum of torasemide metabolite M1.(Continued)

306.0538	213.30	$C_{13}H_{11}N_3O_4S + H^+$
365.1255	197.82	$C_{16}H_{20}N_4O_4S + H^+$
365.1621	19.65	
365.2117	15.58	

The human liver S9 incubation of torasemide led to the formation of torasemide metabolite M1. Considering the spectral match of 0.906 (see Figure SI-D273) and the retention times of 13.6 and 13.9 minutes in the wastewater and the human liver S9 sample, respectively, further confidence could be gained that the detected feature in wastewater is torasemide metabolite M1. Due to this diagnostic evidence, the final confidence level can be increased from level 3 to level 2b.

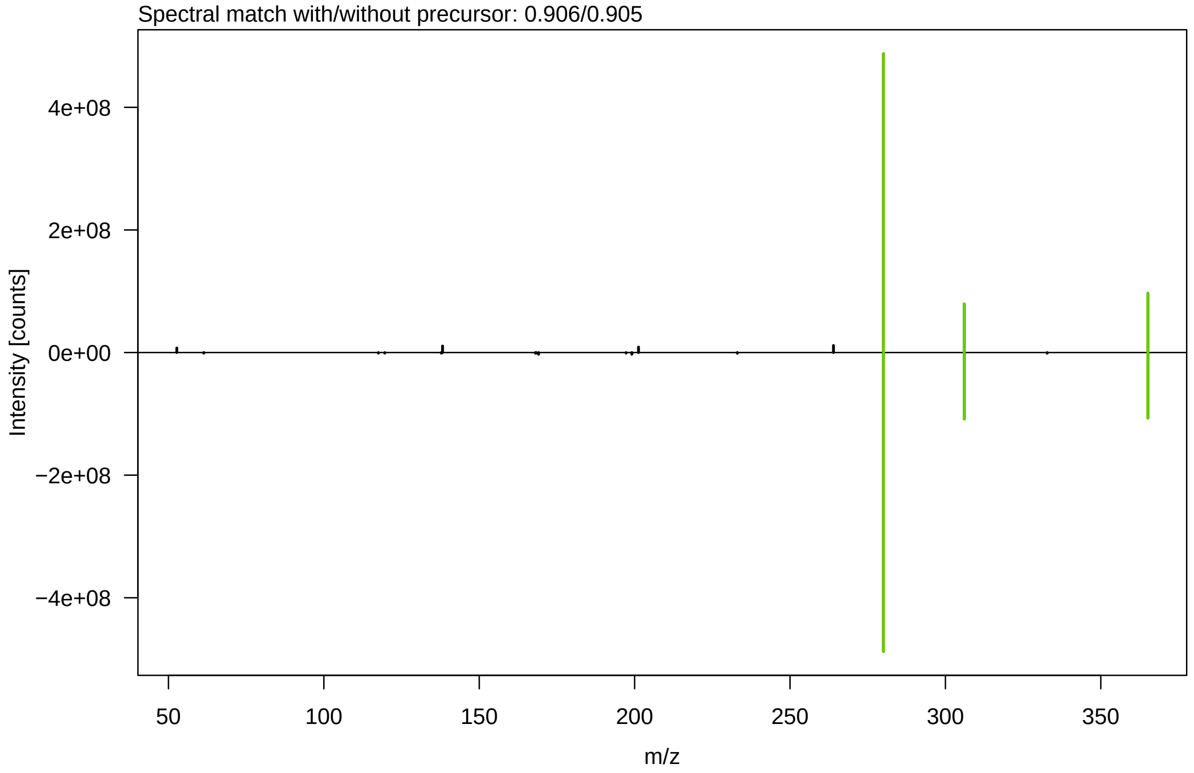

Figure SI-D273: Head to tail plot of torasemide metabolite M1 in wastewater (top) and from human liver S9 incubation (bottom). Matching fragments are highlighted in green.

In addition to the human liver S9 incubation experiment, a reference standard of torasemide metabolite M1 was purchased. Figure SI-D274 shows the extracted ion chromatograms of this standard, the sample and the spiked sample, as well as a head to tail plot of the MS2 spectra of the standard and the sample. In addition, the most intense MS2 fragments in the sample and in the standard are displayed. It becomes visible that the retention times of the sample and the spiked sample are identical and the spectra similarity score between sample and standard is equal to 0.678. The most intense MS2 fragment in the sample can be explained by the reference standard. It can therefore be concluded that the suspected compound is indeed torasemide metabolite M1. Correspondingly, the identification confidence can be increased to level 1.

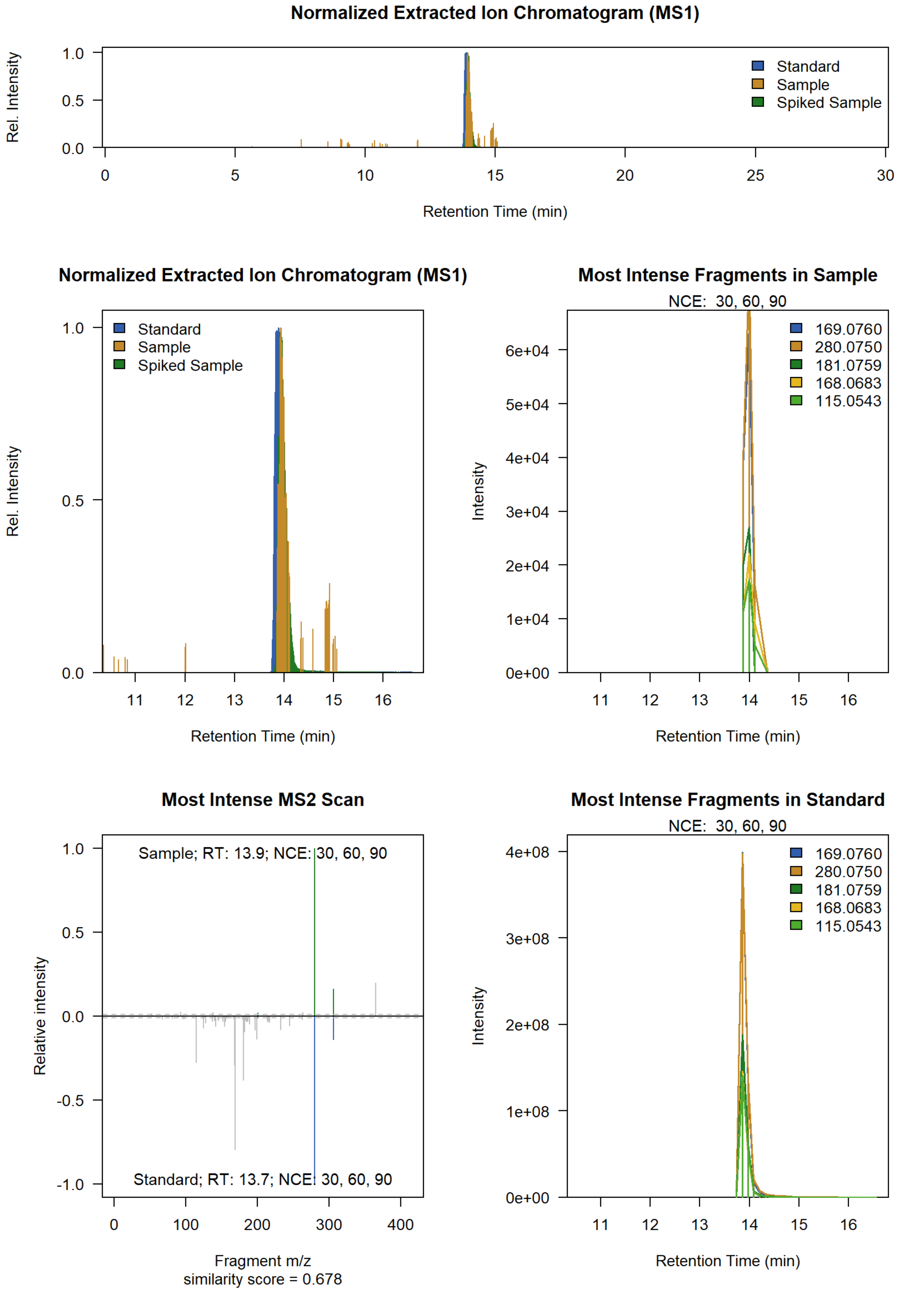

Figure SI-D274: Extracted ion chromatograms of torasemide metabolite M1 in the reference standard, the sample and the spiked sample, as well as MS2 head to tail plot and most intense MS2 fragments in standard and sample.

SI-D2.13.3 Torasemide Metabolite M3

Table SI-D136: Information on identifiers, chemical properties, detection and confidence of identification of torasemide metabolite M3.

IUPAC Name	1-[4-(4-hydroxy-3-methylanilino)pyridin-3-yl]sulfonyl-3-propan-2-ylurea
Molecular formula	$C_{16}H_{20}N_4O_4S$
Monoisotopic mass [g/mol]	364.1205
Adduct	$[M+H]^+$
Retention time [min]	14.0
SMILES	CC1=C(C=CC(=C1)NC2=C(C=NC=C2)S(=O)(=O)NC(=O)NC(C)C)O
InChI	InChI=1S/C16H20N4O4S/c1-10(2)18-16(22)20-25(23,24)15-9-17-7-6-13(15)19-12-4-5-14(21)11(3)8-12/h4-10,21H,1-3H3,(H,17,19)(H2,18,20,22)
InChI-Key	BJCCDWZGWOVSPR-UHFFFAOYSA-N
CAS RN	99300-67-1
Metabolite of	Torasemide
Detection frequency	100% (15/15 samples)
Detected in	Altenrhein, Monday-Friday<br>Neugut, Monday-Friday<br>Werdhölzli, Monday-Friday
Intensity	E7
Initial confidence level	level 3
Initial confidence score	0.42
Final confidence level	level 3

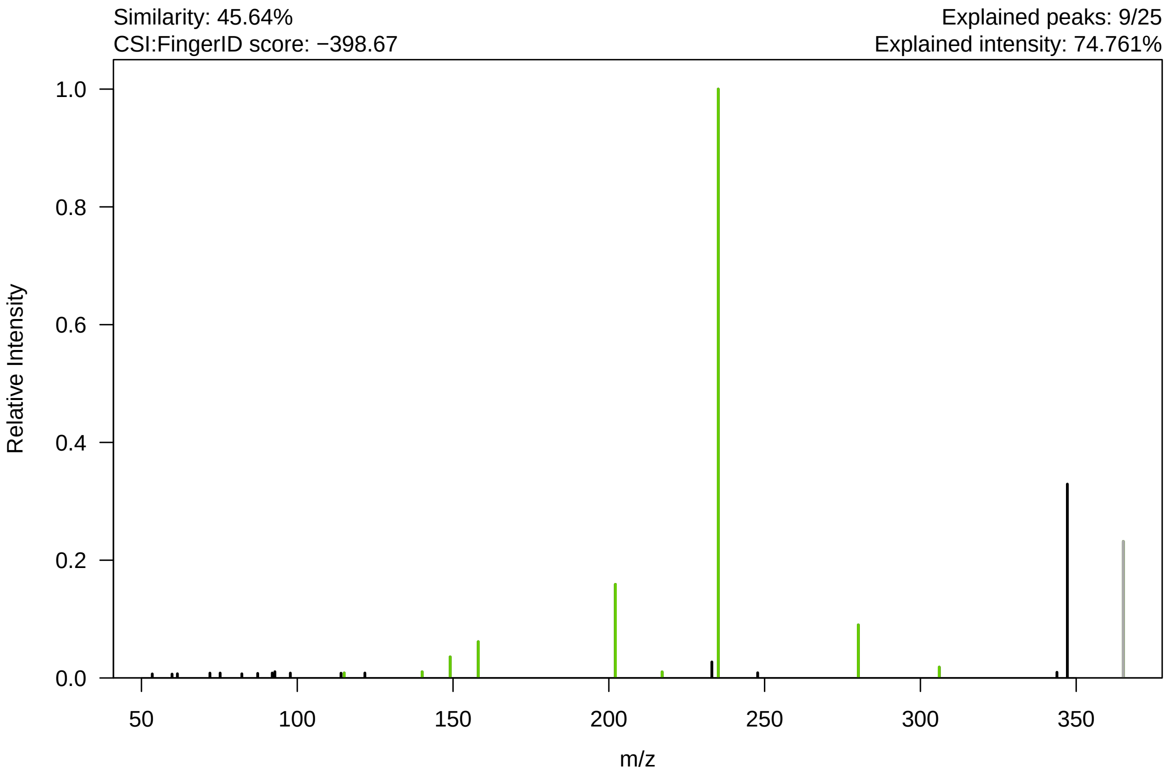

Figure SI-D275: Measured MS2 spectrum. Matching fragments with torasemide metabolite M3 predicted by SIRIUS/CSI:FingerID are highlighted in green. The molecular ion in gray is not considered.

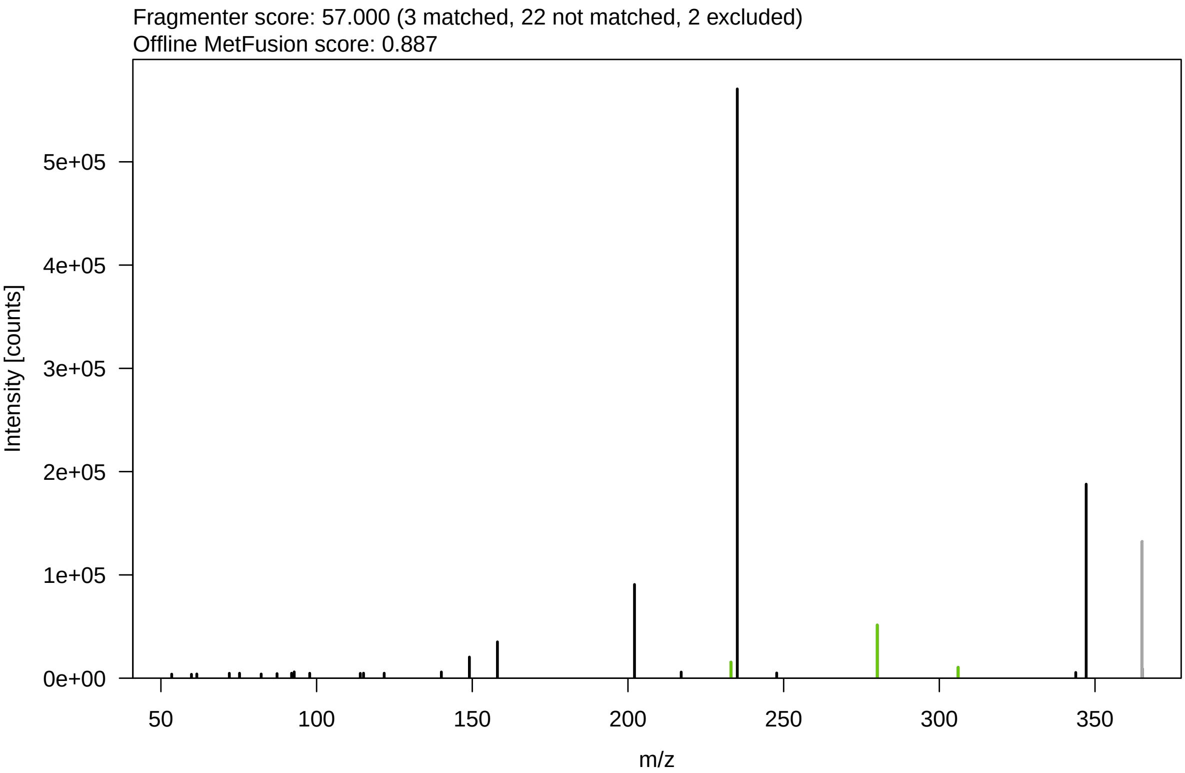

Figure SI-D276: Measured MS2 spectrum. Matching fragments with torasemide metabolite M3 predicted by MetFrag are highlighted in green. The molecular ion in gray is not considered.

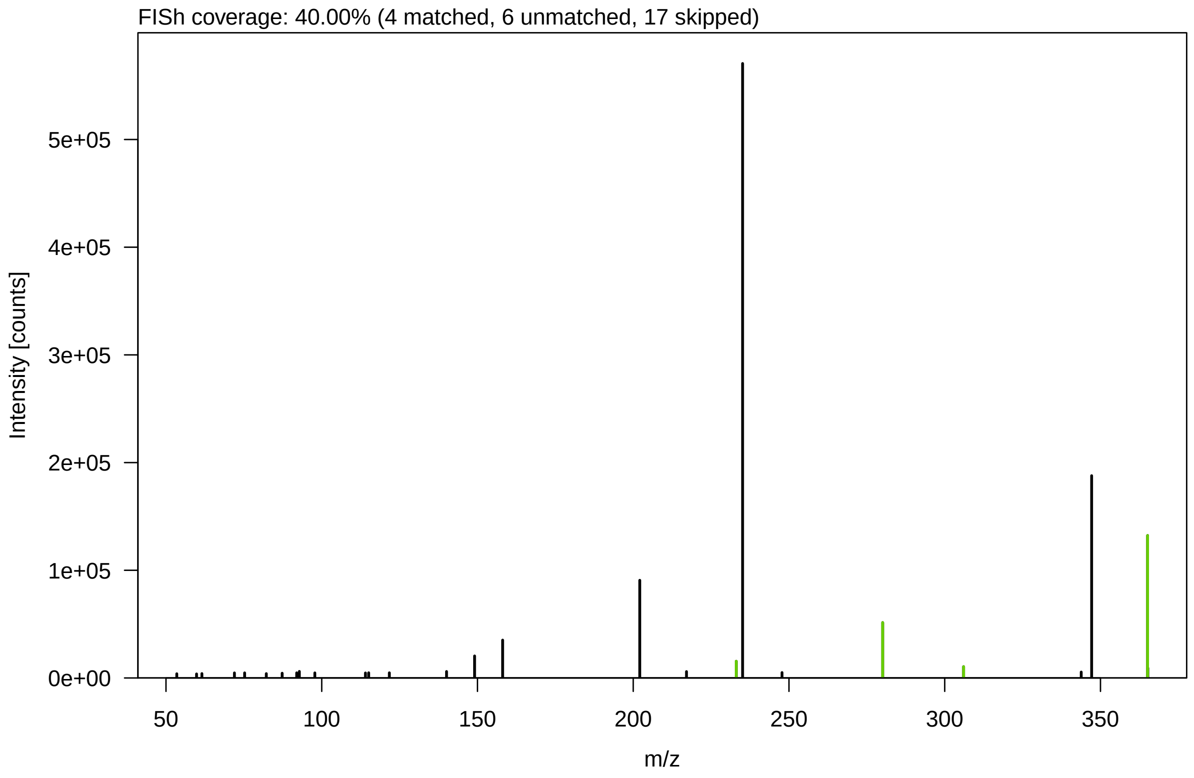

Figure SI-D277: Measured MS2 spectrum. Matching fragments with torasemide metabolite M3 predicted by FISh Scoring are highlighted in green.

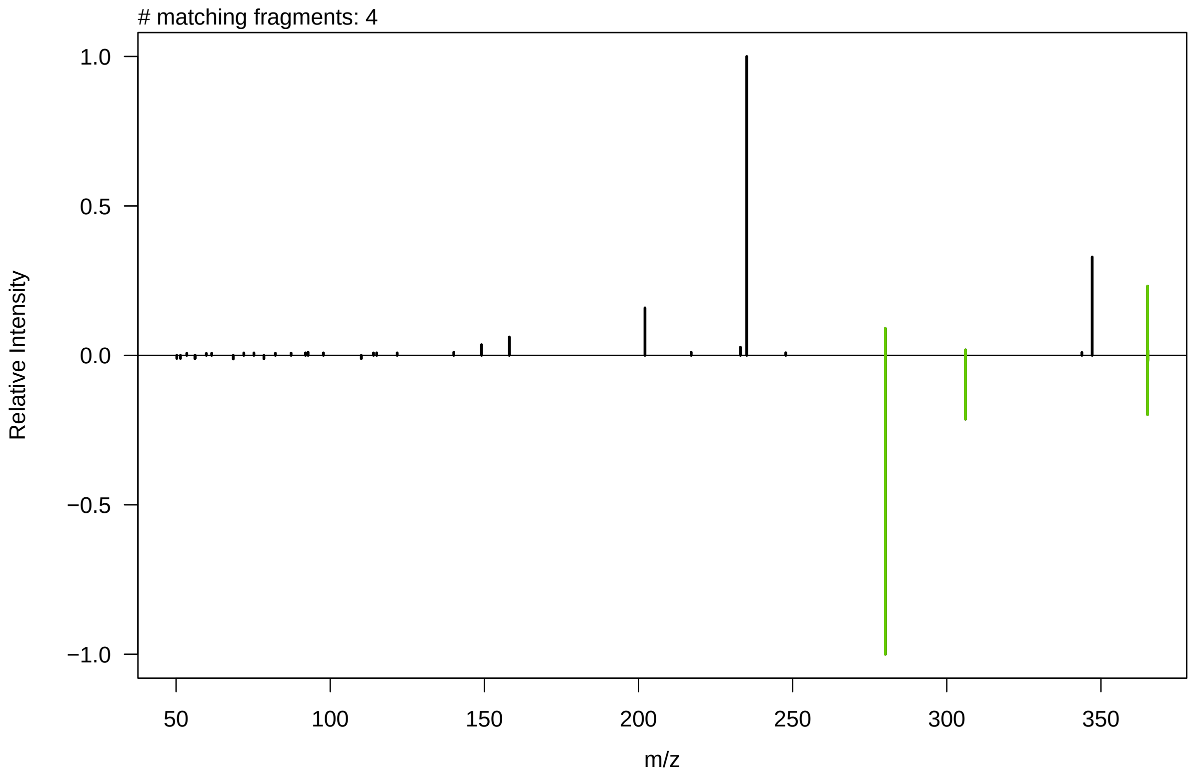

Figure SI-D278: Head to tail plot of the isomeric compounds torasemide metabolite M3 and torasemide metabolite M1. Matching fragments are highlighted in green.

Table SI-D137: Molecular network results and retention time prediction of torasemide metabolite M3.

Comparison with	Torasemide metabolite M1
MSn Score	51
Forward coverage	71
Reverse coverage	30
Forward match	5
Reverse match	3
ΔMass [g/mol]	0
Measured retention time [min]	14.0
Predicted $logD_{OW}$ (pH = 2.7)	0.78
Predicted retention time [min]	15.8
Predicted retention time range (95% confidence interval) [min]	11.2-20.3
Predicted retention time range (99% confidence interval) [min]	9.7-21.8

Table SI-D138: Annotated MS2 spectrum of torasemide metabolite M3.

m/z	Relative Intensity	Annotation
53.4673	6.85	
59.8153	6.58	
61.5326	7.12	
71.9712	8.24	
75.2495	8.33	
82.2093	7.06	
87.2988	7.73	
91.9692	8.38	
92.8135	10.64	
97.7858	8.28	
114.0362	8.12	
115.0748	8.46	
121.7050	8.40	
140.0701	10.51	$C_7H_9NO_2 + H^+$
149.0743	35.80	$C_5H_{12}N_2OS + H^+$
158.0814	61.58	$C_7H_{11}NO_3 + H^+$
202.0974	158.82	$C_{11}H_{11}N_3O + H^+$
217.1014	10.38	$C_9H_{16}N_2O_2S + H^+$
233.0695	27.18	$C_7H_{12}N_4O_3S + H^+$
235.1109	999.00	$C_9H_{18}N_2O_3S + H^+$
247.7709	8.80	
280.0754	90.05	$C_{12}H_{13}N_3O_3S + H^+$
306.0524	18.39	$C_{13}H_{11}N_3O_4S + H^+$

Continued on next page

Table SI-D138: Annotated MS2 spectrum of torasemide metabolite M3.(Continued)

343.8203	9.65	
347.1630	328.90	
365.1269	231.62	$C_{16}H_{20}N_4O_4S + H^+$
365.2036	16.22	

No reference standard of troasemide metabolite M3 was purchasable. Therefore, a human liver S9 incubation experiment with torasemide was performed, to generate torasemide metabolites *in vitro*. However, no compound with a precursor mass matching the one of torasemide metabolite M3 and lying within a retention time window of ±3 minutes of the suspect was detected. Since *in vitro* experiments cannot be translated one to one into *in vivo* experiments, the suspected compound can neither be confirmed nor rejected. As a consequence, the final identification confidence remains at level 3.

SI-D2.14 Other Phase I Metabolites

SI-D2.14.1 2-Hydroxytrimipramine

2-Hydroxytrimipramine is a metabolite of trimitramine, an antidepressant.[2] Figure SI-D279 shows the metabolism of trimipramine to 2-hydroxytrimipramine.

Table SI-D139: Information on identifiers, chemical properties, detection and confidence of identification of 2-hydroxytrimipramine.

IUPAC Name	11-[3-(dimethylamino)-2-methylpropyl]-5,6-dihydrobenzo[b][1]benzazepin-3-ol
Molecular formula	$C_{20}H_{26}N_2O$
Monoisotopic mass [g/mol]	310.2045
Adduct	$[M+H]^+$
Retention time [min]	15.5
SMILES	CC(CN1C2=C(CCC3=CC=CC=C31)C=C(C=C2)O)CN(C)C
InChI	InChI=1S/C20H26N2O/c1-15(13-21(2)3)14-22-19-7-5-4-6-16(19)8-9-17-12-18(23)10-11-20(17)22/h4-7,10-12,15,23H,8-9,13-14H2,1-3H3
InChI-Key	FQJSSUOYVSEYPF-UHFFFAOYSA-N
CAS RN	2064-15-5
Metabolite of	Trimipramine
Detection frequency	100% (15/15 samples)
Detected in	Neugut, Monday
Intensity	E7
Initial confidence level	level 3
Initial confidence score	0.34
Final confidence level	level 1

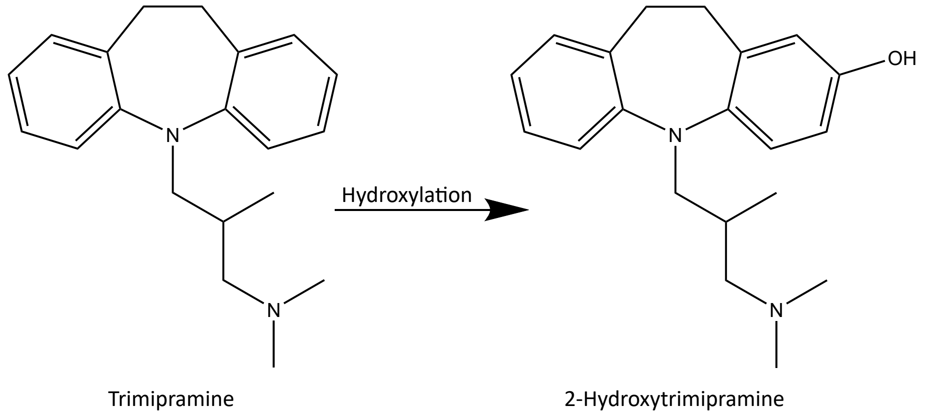

Figure SI-D279: Metabolism of trimipramine to 2-hydroxytrimipramine.

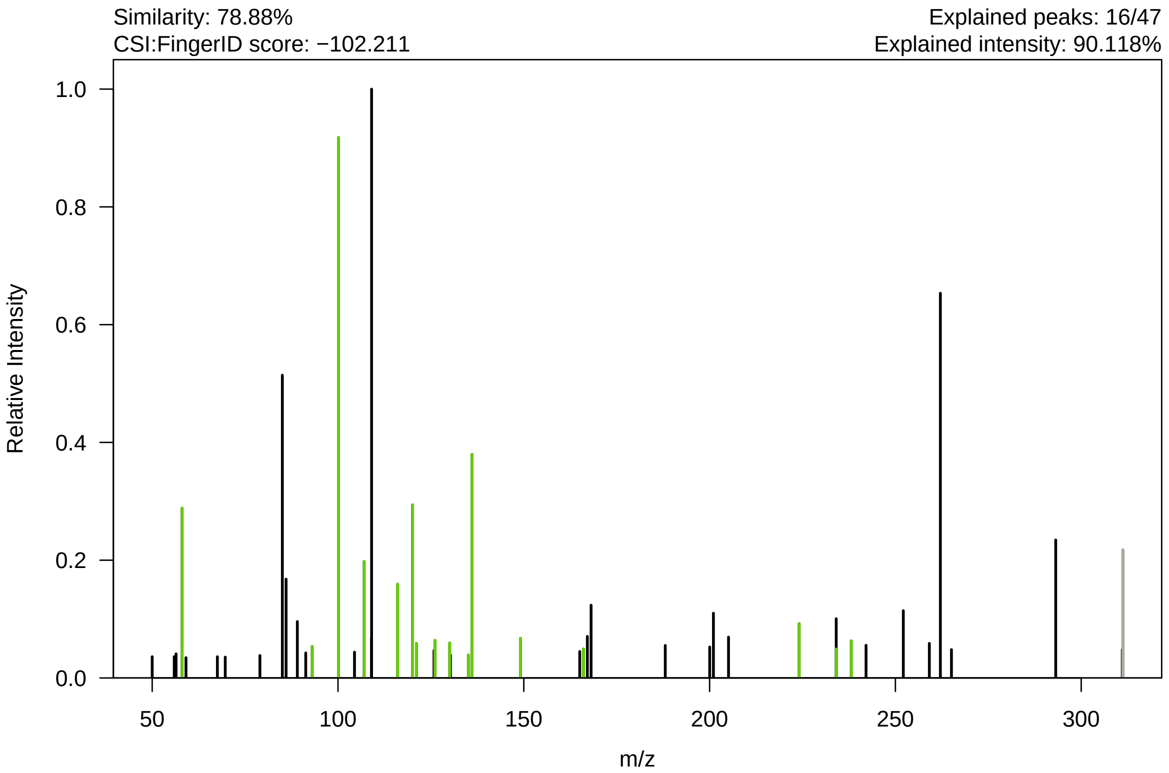

Figure SI-D280: Measured MS2 spectrum. Matching fragments with 2-hydroxytrimipramine predicted by SIRIUS/CSI:FingerID are highlighted in green. The molecular ion in gray is not considered.

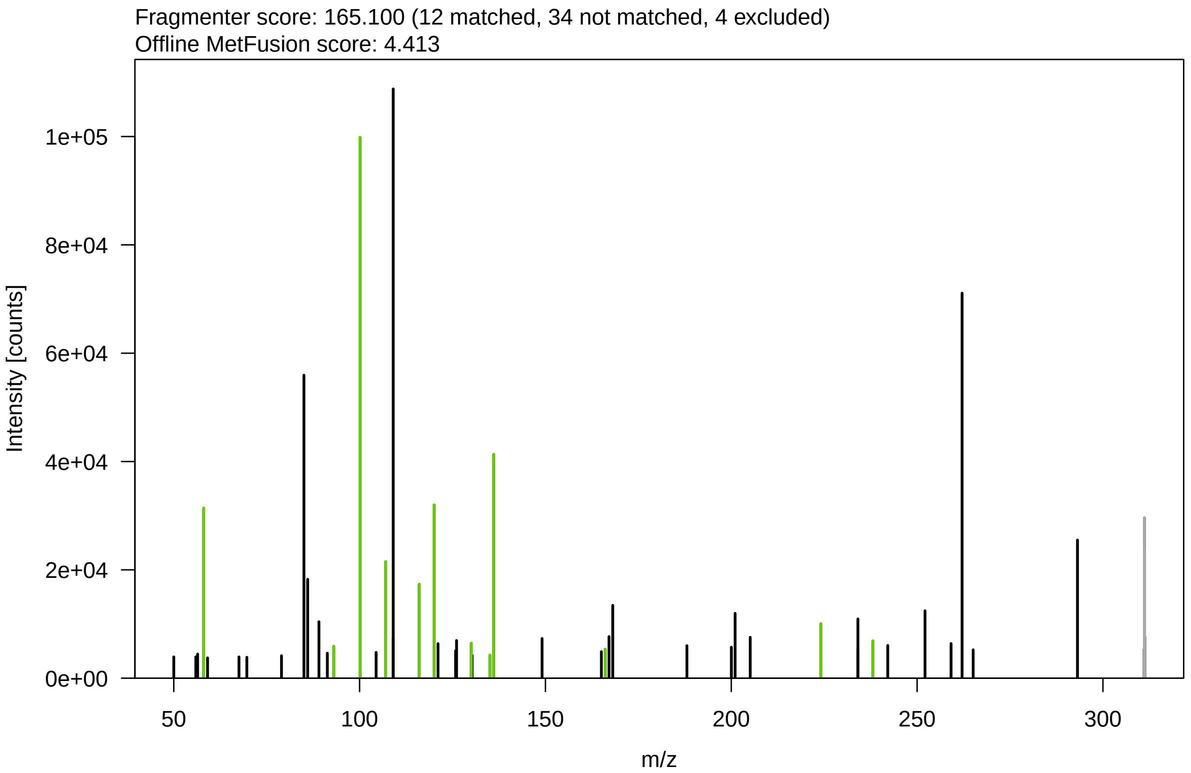

Figure SI-D281: Measured MS2 spectrum. Matching fragments with 2-hydroxytrimipramine predicted by MetFrag are highlighted in green. The molecular ion in gray is not considered.

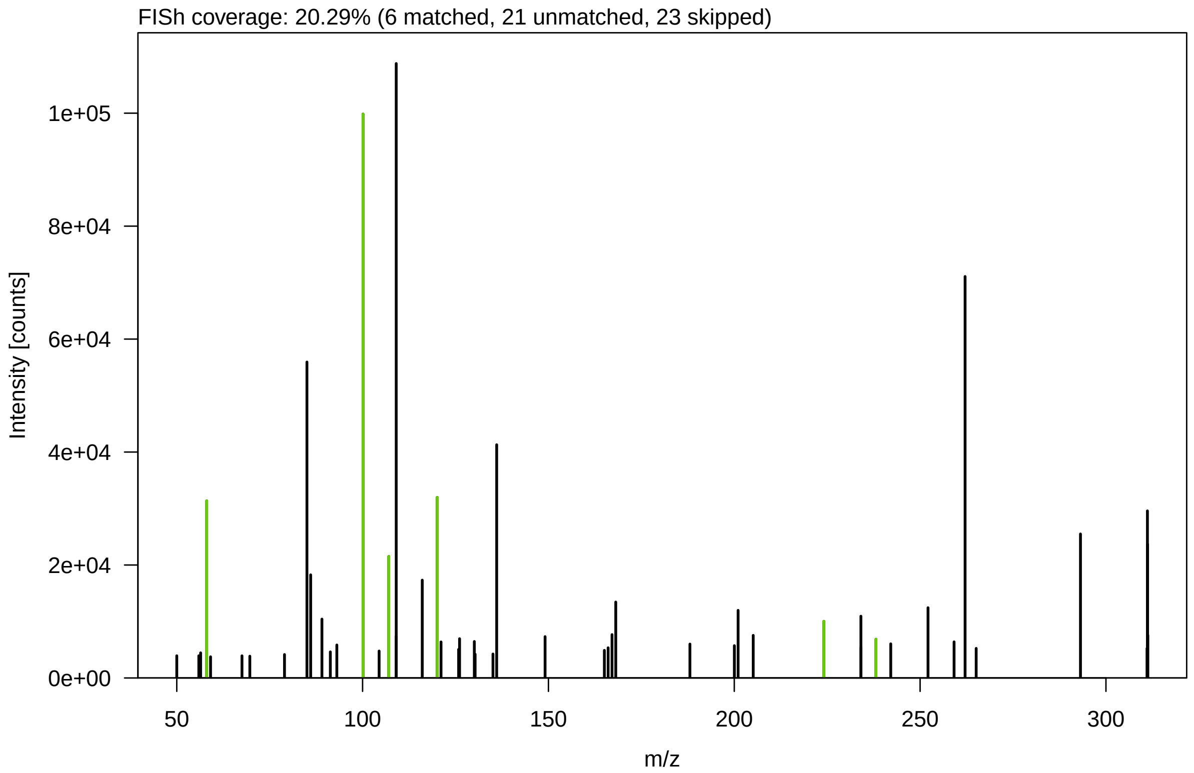

Figure SI-D282: Measured MS2 spectrum. Matching fragments with 2-hydroxytrimipramine predicted by FISh Scoring are highlighted in green. Low intensity fragments are not considered and skipped.

Table SI-D140: Retention time prediction of 2-hydroxytrimipramine.

Measured retention time [min]	15.5
Predicted $\log D_{OW}$ (pH = 2.7)	0.91
Predicted retention time [min]	15.9
Predicted retention time range (95% confidence interval) [min]	11.3-20.5
Predicted retention time range (99% confidence interval) [min]	9.9-22.0

Table SI-D141: Annotated MS2 spectrum of 2-hydroxytrimipramine.

m/z	Relative Intensity	Annotation
50.0073	36.11	
55.9362	36.12	
56.4247	40.87	
58.0655	288.09	$C_3H_7N + H^+$
59.0960	34.44	
67.5476	36.06	
69.6634	35.45	
78.9882	38.10	

Continued on next page

Table SI-D141: Annotated MS2 spectrum of 2-hydroxytrimipramine.(Continued)

85.0283	513.67	
86.0316	167.69	
89.0595	95.72	
91.3204	42.42	
93.0697	53.64	$C_7H_8 + H^+$
100.1120	916.82	$C_6H_{13}N + H^+$
104.4448	43.83	
107.0491	197.61	$C_7H_6O + H^+$
109.0377	67.60	
109.0448	999.00	
116.0492	159.07	$C_8H_5N + H^+$
120.0809	293.62	$C_8H_9N + H^+$
121.1006	58.61	$C_9H_{12} + H^+$
125.8404	46.51	
126.0908	63.87	$C_7H_{11}NO + H^+$
130.0652	59.29	$C_9H_7N + H^+$
130.2743	38.67	
135.0800	38.97	$C_9H_{10}O + H^+$
136.0756	379.23	$C_8H_9NO + H^+$
149.0960	67.24	$C_{10}H_{12}O + H^+$
165.0550	44.89	
166.0648	49.11	$C_{12}H_7N + H^+$
167.1056	70.50	
168.1104	123.46	
188.0693	55.14	
200.0287	52.55	
201.0318	109.89	
205.0965	69.29	
224.1080	92.18	$C_{15}H_{13}NO + H^+$
234.0705	100.48	
234.0915	49.29	$C_{16}H_{11}NO + H^+$
238.1229	63.04	$C_{16}H_{15}NO + H^+$
242.1013	55.50	
252.1301	114.25	
259.1346	58.69	
262.1027	652.72	
265.0859	48.08	

Continued on next page

Table SI-D141: Annotated MS2 spectrum of 2-hydroxytrimipramine.(Continued)

293.1450	234.17	
311.0530	48.17	
311.1583	271.67	
311.2070	217.22	$C_{20}H_{26}N_2O + H^+$
311.2683	69.51	

The human liver S9 incubation of trimipramine led to the formation of 2-hydroxytrimipramine. Considering the spectral match of 0.191 (see Figure SI-D283) and the retention times of 15.5 and 15.6 minutes in the wastewater and the human liver S9 sample, respectively, further confidence could be gained that the detected feature in wastewater is 2-hydroxytrimipramine. Due to this diagnostic evidence, the final identification confidence can be increased from level 3 to level 2b.

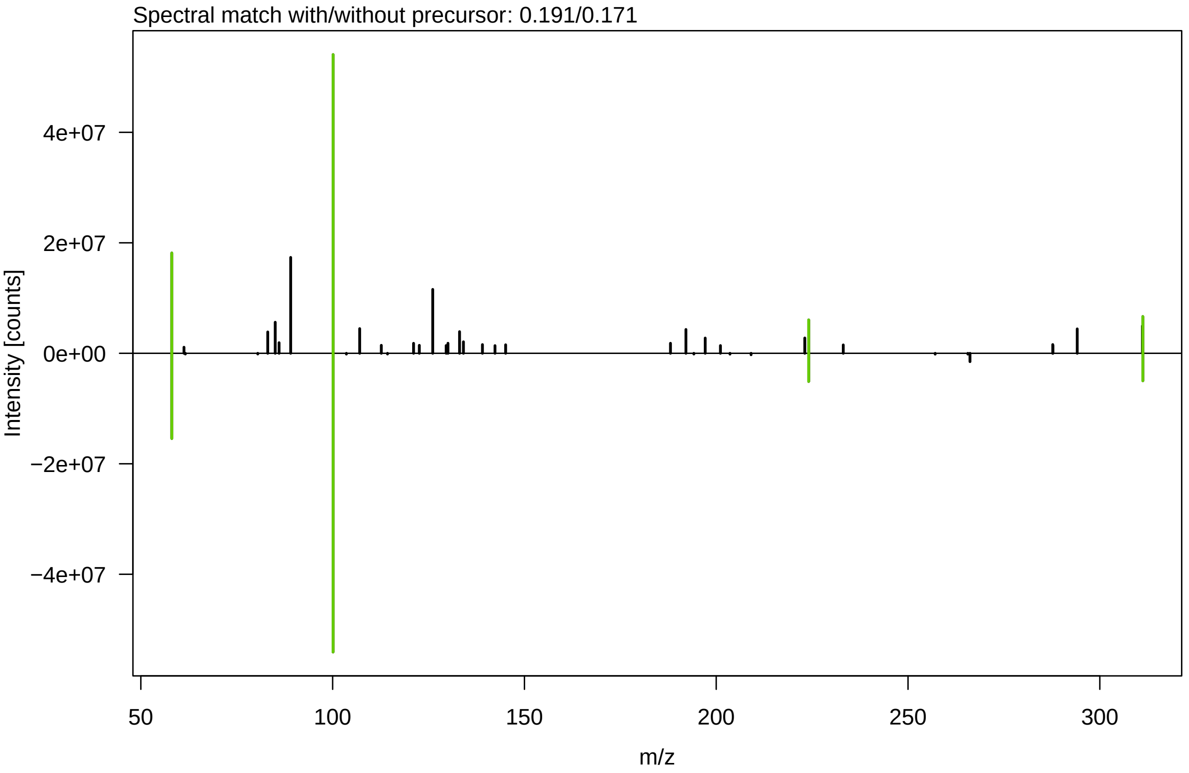

Figure SI-D283: Head to tail plot of 2-hydroxytrimipramine in wastewater (top) and from human liver S9 incubation (bottom). Matching fragments are highlighted in green.

In addition, a reference standard of 2-hydroxytrimipramine was purchased. Figure SI-D284 shows the extracted ion chromatograms of this standard, the sample and the spiked sample, as well as a head to tail plot of the MS2 spectra of the standard and the sample. In addition, the most intense MS2 fragments in the sample and in the standard are displayed. It becomes visible that the retention times of the sample and the spiked sample are identical and the spectra similarity score between sample and standard is equal to 0.932. The three most intense MS2 fragments in the sample can be explained by the reference standard. It can therefore be concluded that the suspected compound is indeed 2-hydroxytrimipramine. Correspondingly, the identification confidence can be increased to level 1.

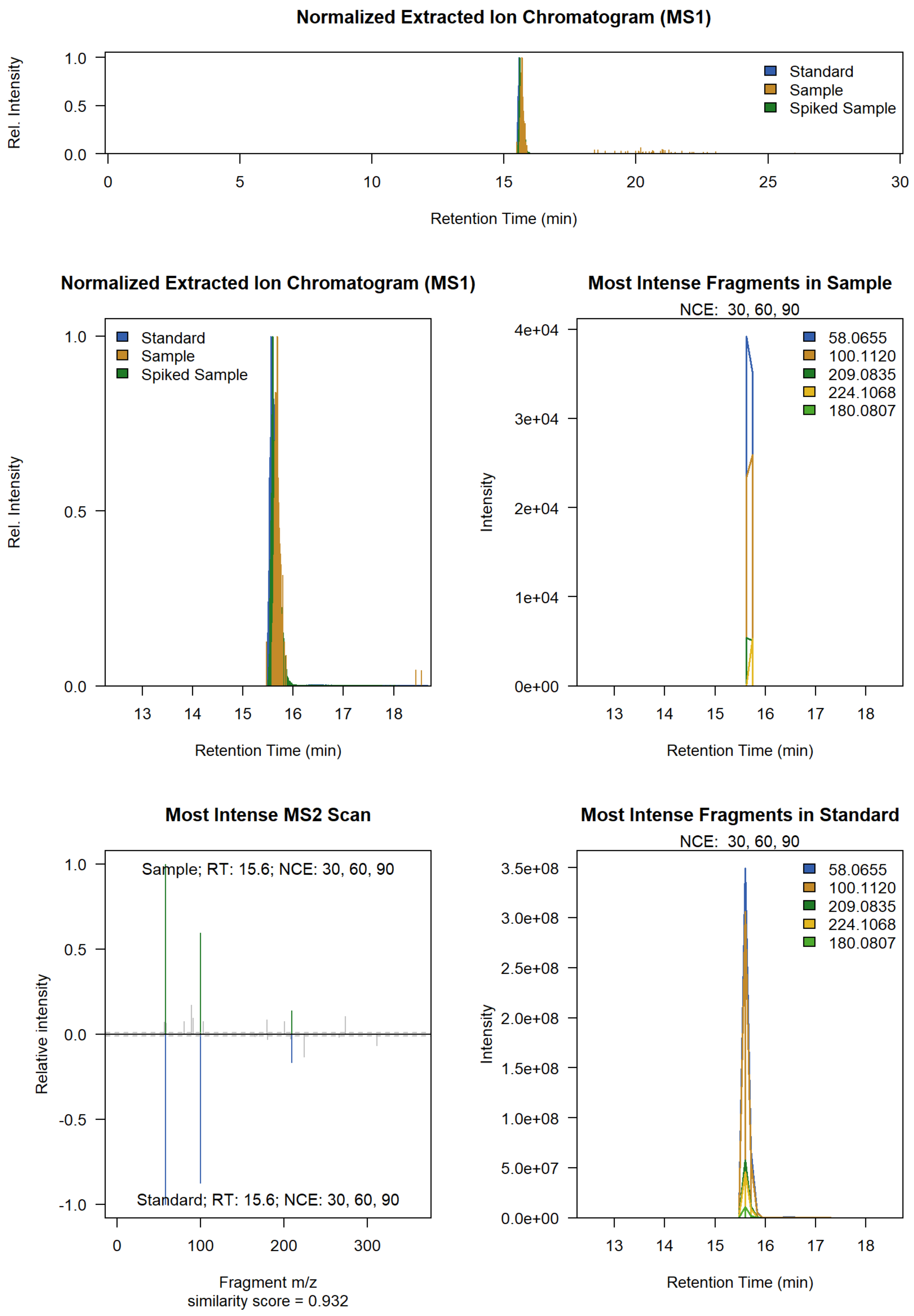

Figure SI-D284: Extracted ion chromatograms of 2-hydroxytrimipramine in the reference standard, the sample and the spiked sample, as well as MS2 head to tail plot and most intense MS2 fragments in standard and sample.

SI-D2.14.2 3-Desmethyltrimethoprim and 4-Desmethyltrimethoprim

3-Desmethyltrimethoprom and 4-desmethyltrimethoprim are metabolites of trime-thoprim. Trimethoprim is an antifolate antibiotic often used in combination with sulfamethoxazole to treat a number of infections, including those of the urinary tract, respiratory tract, and gastrointestinal tract.[2] Based on the MS2 fragments, no differentiation between 3- and 4-desmethyltrimethoprim was possible. Figure SI-D285 shows the metabolism scheme and Figure SI-D286 the trimethoprim cluster.

Table SI-D142: Information on identifiers, chemical properties, detection and confidence of identification of 3-desmethyltrimethoprim and 4-desmethyltrimethoprim.

IUPAC Name	5-[(2,4-diaminopyrimidin-5-yl)methyl]-2,3-dimethoxyphenol
	4-[(2,4-diaminopyrimidin-5-yl)methyl]-2,6-dimethoxyphenol
Molecular formula	$C_{13}H_{16}N_4O_3$
Monoisotopic mass [g/mol]	276.1222
Adduct	$[M+H]^+$
Retention time [min]	10.4
SMILES	COC1=CC(=CC(=C1OC)O)CC2=CN=C(N=C2N)N
	COC1=CC(=CC(=C1O)OC)CC2=CN=C(N=C2N)N
InChI	InChI=1S/C13H16N4O3/c1-19-10-5-7(4-9(18)11(10)20-2)3-8-6-16-13(15)17-12(8)14/h4-6,18H,3H2,1-2H3,(H4,14,15,16,17)
	InChI=1S/C13H16N4O3/c1-19-9-4-7(5-10(20-2)11(9)18)3-8-6-16-13(15)17-12(8)14/h4-6,18H,3H2,1-2H3,(H4,14,15,16,17)
InChI-Key	HWBPOLWLLFXEJY-UHFFFAOYSA-N
	HPOCGNHBIFZCAN-UHFFFAOYSA-N
CAS RN	27653-69-6
	21253-58-7
Metabolite of	Trimethoprim
Detection frequency	100% (15/15 samples)
Detected in	Altenrhein, Monday-Friday
	Neugut, Monday-Friday
	Werdhölzli, Monday-Friday
Intensity	E6-E7
Initial confidence level	level 3
Initial confidence score	0.36
Final confidence level	level 1 (3-desmethyltrimethoprim)

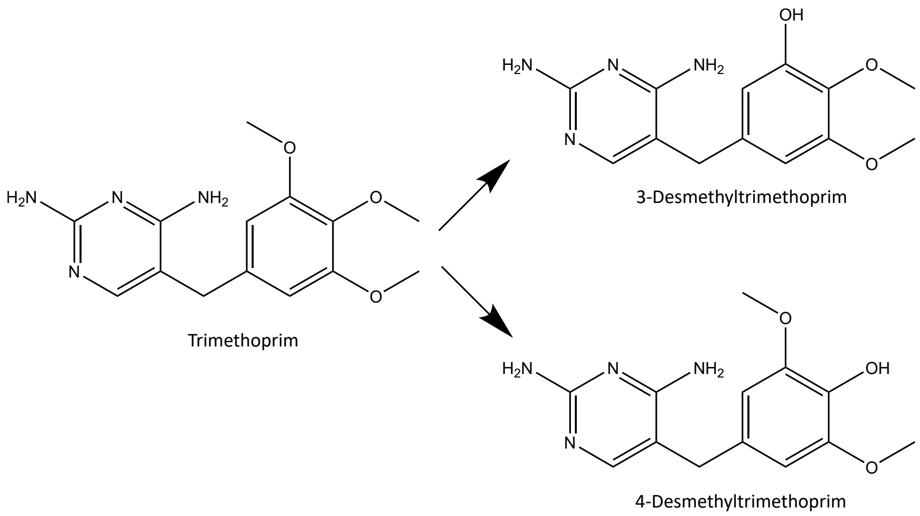

Figure SI-D285: Metabolism of trimethoprim to 3-desmethyltrimethoprim and 4-desmethlytrimethoprim.

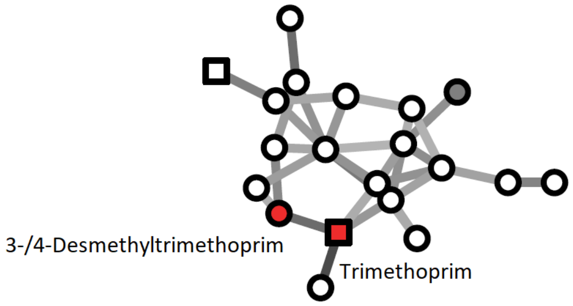

Figure SI-D286: Excerpt of the molecular network showing the trimethoprim cluster.

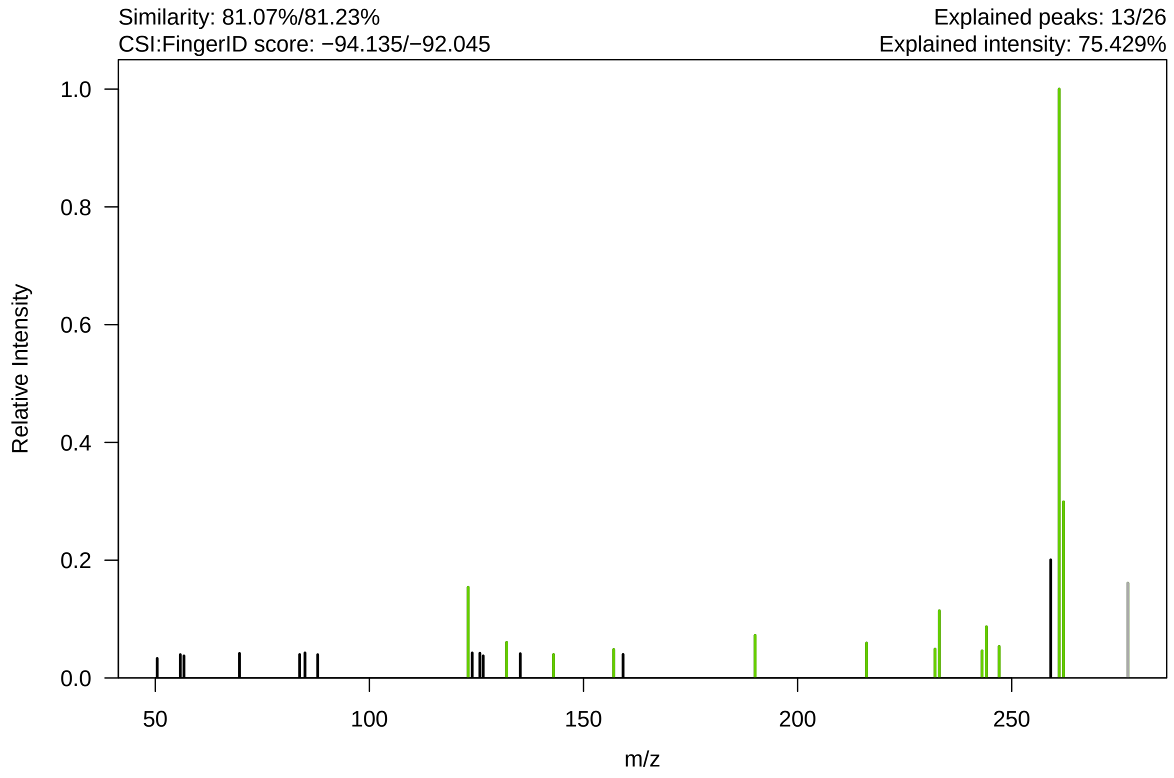

Figure SI-D287: Measured MS2 spectrum. Matching fragments with 3/4-desmethyltrimethoprim predicted by SIRIUS/CSI:FingerID are highlighted in green. The molecular ion in gray is not considered.

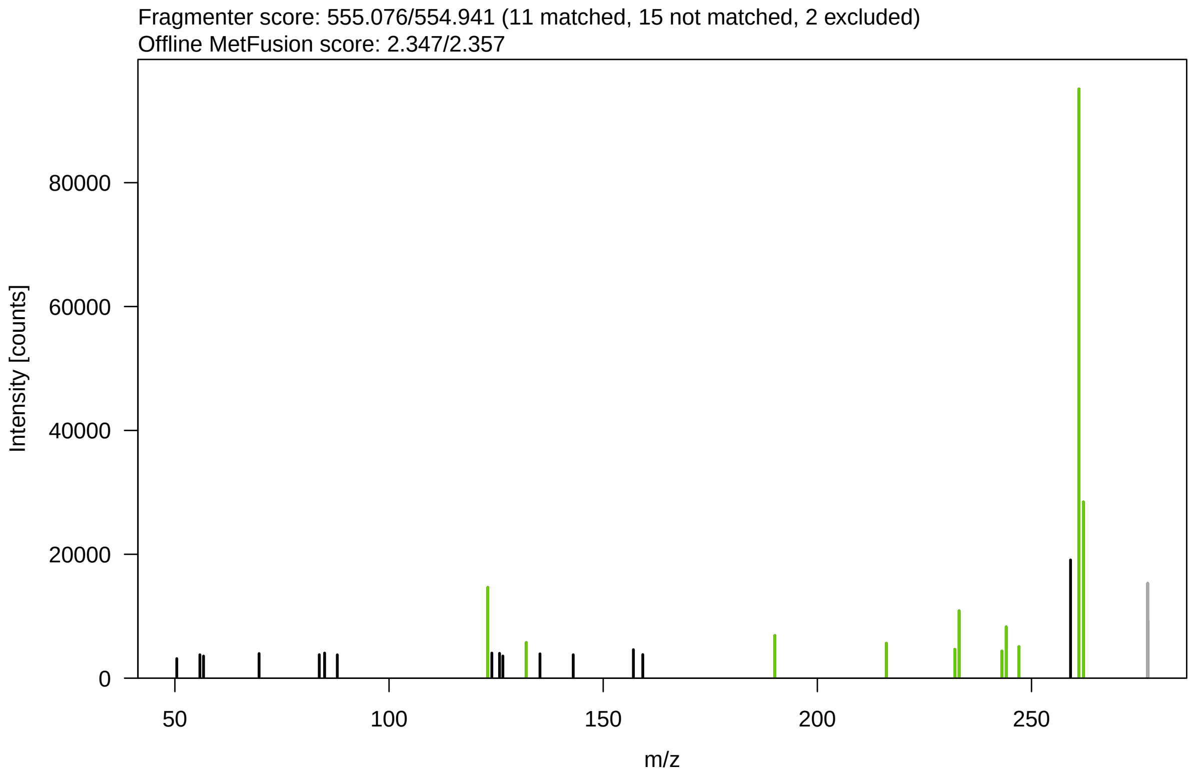

Figure SI-D288: Measured MS2 spectrum. Matching fragments with 3/4-desmethyltrimethoprim predicted by MetFrag are highlighted in green. The molecular ion in gray is not considered.

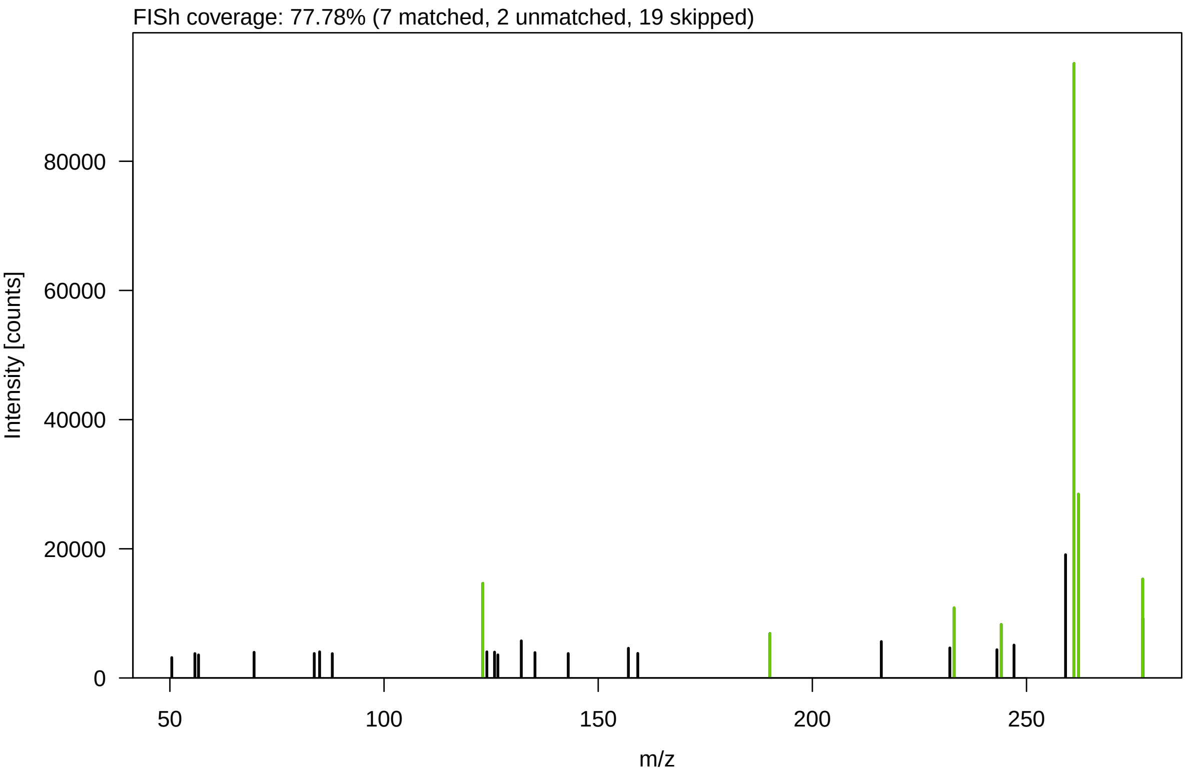

Figure SI-D289: Measured MS2 spectrum. Matching fragments with 3-desmethyltrimethoprim predicted by FISh Scoring are highlighted in green. Low intensity fragments are not considered and skipped.

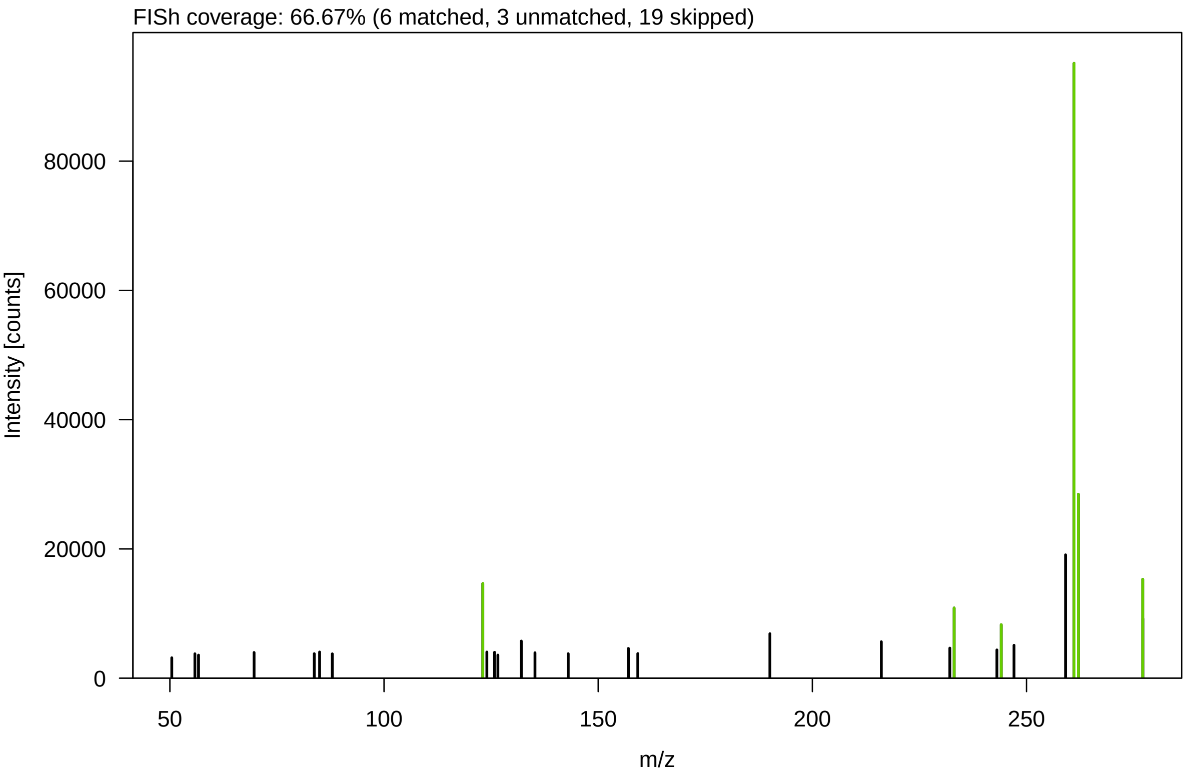

Figure SI-D290: Measured MS2 spectrum. Matching fragments with 4-desmethyltrimethoprim predicted by FISh Scoring are highlighted in green. Low intensity fragments are not considered and skipped.

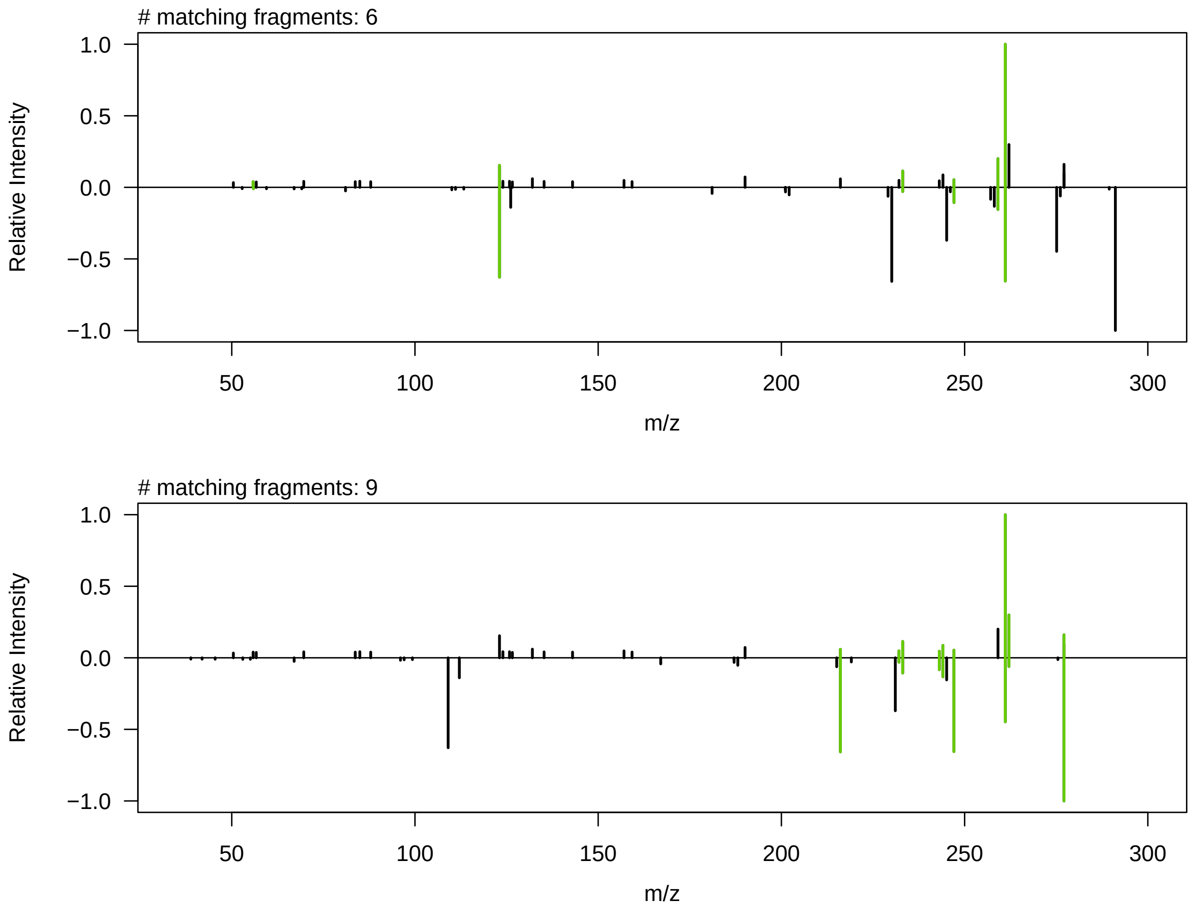

Figure SI-D291: Head to tail plots of 3/4-desmethyltrimethoprim and trimethoprim. In the bottom plot, the mass spectrum of trimethoprim is shifted by the mass difference. Matching fragments are highlighted in green.

Table SI-D143: Molecular network results and retention time prediction of 3-desmethyltrimethoprim and 4-desmethyltrimethoprim.

Comparison with	Trimethoprim
MSn Score	58
Forward coverage	78
Reverse coverage	38
Forward match	7
Reverse match	15
ΔMass [g/mol]	14.0157
Measured retention time [min]	10.4
Predicted $logD_{OW}$ (pH = 2.7)	-0.34, -0.34
Predicted retention time [min]	14.3, 14.3
Predicted retention time range (95% confidence interval) [min]	9.7-18.9, 9.7-18.9
Predicted retention time range (99% confidence interval) [min]	8.2-20.4, 8.2-20.4

Table SI-D144: Annotated MS2 spectrum of 3/4-desmethyltrimethoprim.

m/z	Relative Intensity	Annotation
50.4413	33.15	
55.8385	39.64	
56.6878	37.51	
69.6552	41.74	
83.7053	39.80	
84.9546	42.56	
87.9211	39.59	
123.0664	153.87	$C_7H_8NO + H^+$
124.0058	42.53	
125.7960	42.06	
126.5792	37.58	
132.0445	60.36	
135.2364	41.28	
143.0012	39.76	$C_8NO_2 + H^+$
157.0499	48.31	$C_8H_4N_4 + H^+$
159.2418	39.99	
190.0865	72.28	$C_{11}H_{11}NO_2 + H^+$
216.1001	59.22	$C_{11}H_{11}N_4O + H^+$
232.0962	48.90	$C_{13}H_{13}NO_3 + H^+$
233.1020	114.09	$C_{11}H_{13}N_4O_2 + H^+$
243.0896	45.94	$C_{12}H_{10}N_4O_2 + H^+$
244.0960	86.92	$C_{12}H_{11}N_4O_2 + H^+$
247.0826	53.47	$C_{11}H_{10}N_4O_3 + H^+$
259.1148	200.44	
261.0979	999.00	$C_{12}H_{12}N_4O_3 + H^+$
262.1060	298.80	$C_{12}H_{13}N_4O_3 + H^+$
277.1281	160.72	$C_{13}H_{16}N_4O_3 + H^+$
277.1566	96.65	

Reference standards of 3-desmethyltrimethoprim and 4-desmethyltrimethoprim were purchased, since it was not possible to differentiate between the two compounds based on the previously shown results. Figure SI-D292 shows the extracted ion chromatograms of 3-desmethyltrimethoptrim and 4-desmethyltrimethoprim, respectively, overlain by the extracted ion chromatogram of the suspected compound. Based on the retention time, one can conclude that the suspected compound is 3-desmethyltrimethoprim, since 4-desmethyltrimethoprim elutes about half a mintute too early.

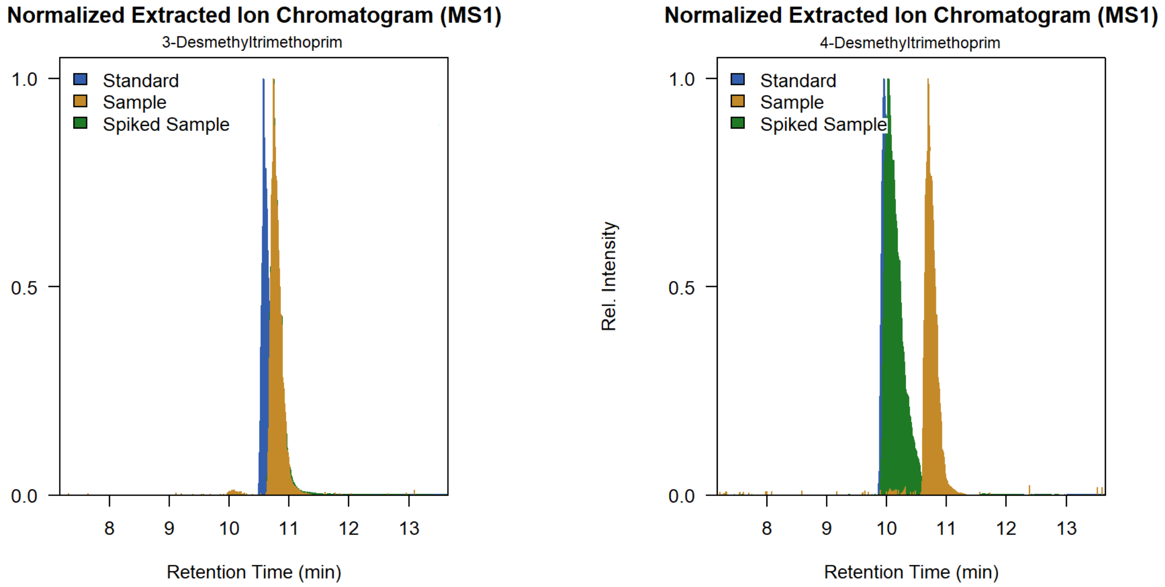

Figure SI-D292: Extracted ion chromatograms of 3-desmethyltrimethoprim and 4-desmethyltrimethoprim in the reference standard and the suspected compound in the sample and the spiked sample.

Figure SI-D293 shows the extracted ion chromatograms of the 3-desmethyltrimethoprim reference standard, the sample and the spiked sample, as well as a head to tail plot of the MS2 spectra of the standard and the sample. In addition, the most intense MS2 fragments in the sample and in the standard are displayed. It becomes visible that the retention times of the sample and the spiked sample are identical and the spectra similarity score between sample and standard is equal to 0.699. Several MS2 fragments in the sample can be explained by the reference standard. It can therefore be concluded that the suspected compound is indeed 3-desmethyltrimethoprim. Correspondingly, the identification confidence can be increased to level 1.

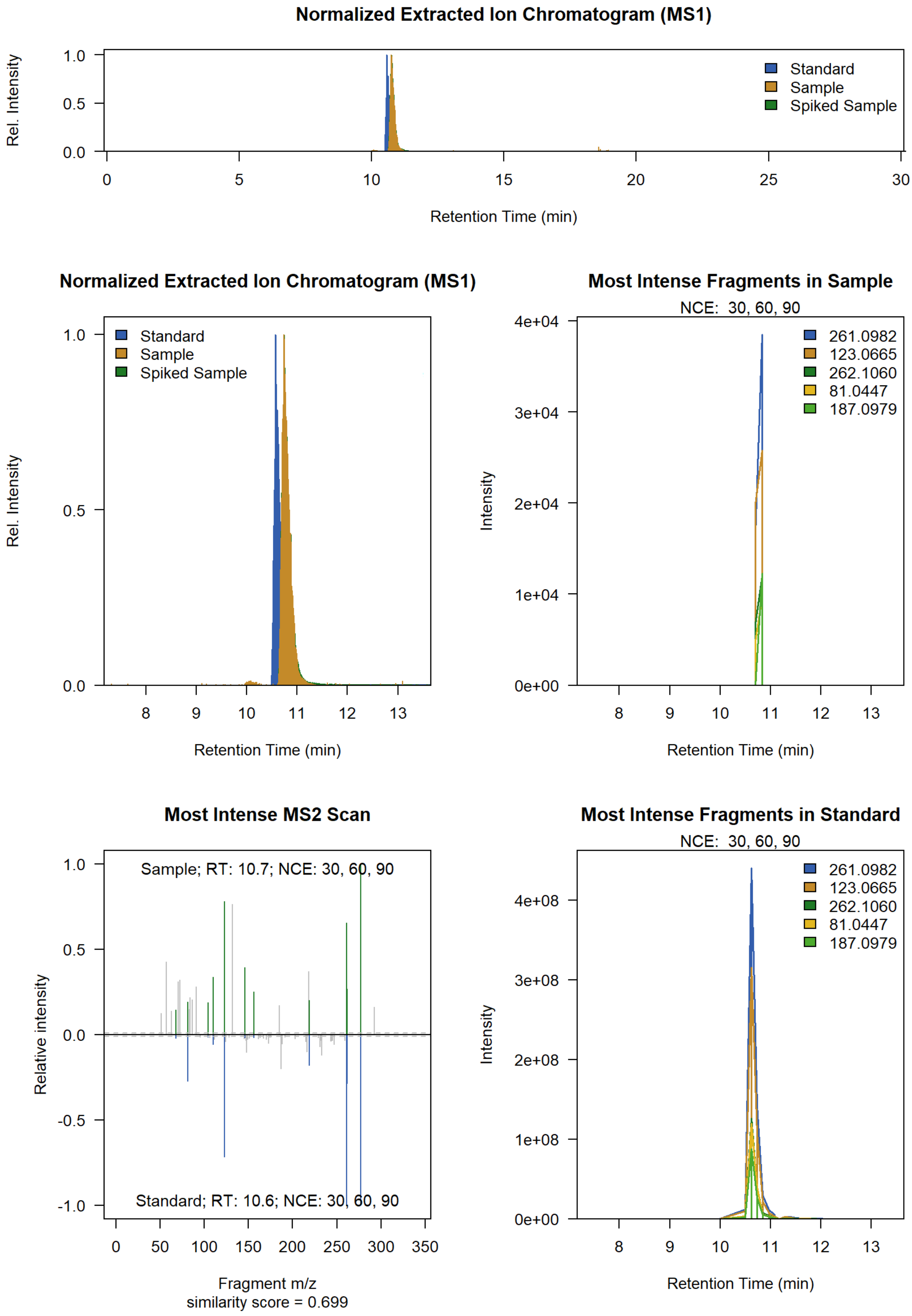

Figure SI-D293: Extracted ion chromatograms of 3-desmethyltrimethoprim in the reference standard, the sample and the spiked sample, as well as MS2 head to tail plot and most intense MS2 fragments in standard and sample.

SI-D2.14.3 4-Desmethylpantoprazole

4-Desmethylpantoprazole is a metabolite of pantoprazole, a proton pump inhibitor used for the treatment of gastroesophageal reflux disease.[2] Figure SI-D296 shows the metabolism scheme and Figure SI-D295 the pantoprazole cluster.

Table SI-D145: Information on identifiers, chemical properties, detection and confidence of identification of 4-desmethylpantoprazole.

IUPAC Name	2-[[6-(difluoromethoxy)-1*H*-benzimidazol-2-yl]sulfinylmethyl]-3-methoxy-1*H*-pyridin-4-one
Molecular formula	$C_{15}H_{13}F_2N_3O_4S$
Monoisotopic mass [g/mol]	369.0595
Adduct	$[M+H]^+$
Retention time [min]	14.6
SMILES	COC1=C(NC=CC1=O)CS(=O)C2=NC3=C(N2)C=C(C=C3)OC(F)F
InChI	InChI=1S/C15H13F2N3O4S/c1-23-13-11(18-5-4-12(13)21)7-25(22)15-19-9-3-2-8(24-14(16)17)6-10(9)20-15/h2-6,14H,7H2,1H3,(H,18,21)(H,19,20)
InChI-Key	LVQDPJBMSVUBTN-UHFFFAOYSA-N
CAS RN	141854-24-2
Metabolite of	Pantoprazole
Detection frequency	100% (15/15 samples)
Detected in	Altenrhein, Monday-Friday
	Neugut, Monday-Friday
	Werdhölzli, Monday-Friday
Intensity	E7
Initial confidence level	level 3
Initial confidence score	0.39
Final confidence level	level 2b

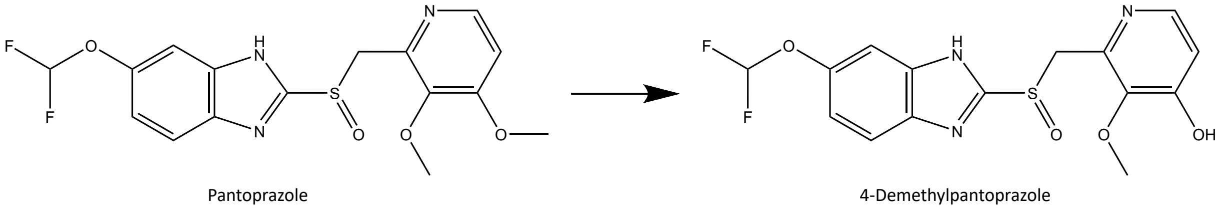

Figure SI-D294: Metabolism of pantoprazole to 4-desmethylpantoprazole.

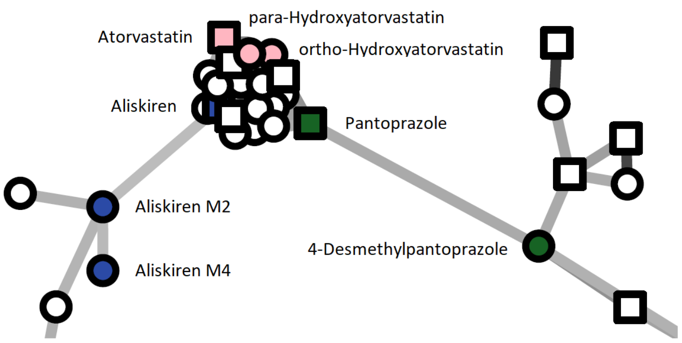

Figure SI-D295: Excerpt of the molecular network showing the pantoprazole cluster.

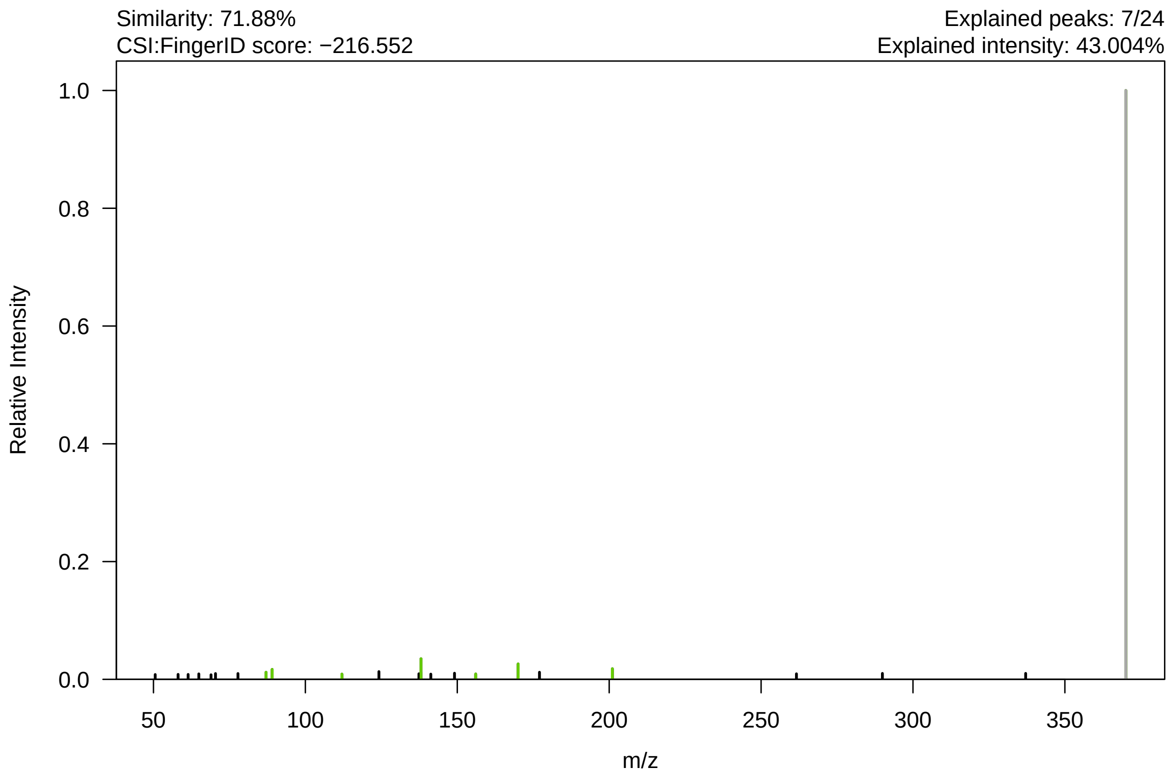

Figure SI-D296: Measured MS2 spectrum. Matching fragments with 4-desmethylpantoprazole predicted by SIRIUS/CSI:FingerID are highlighted in green. The molecular ion in gray is not considered.

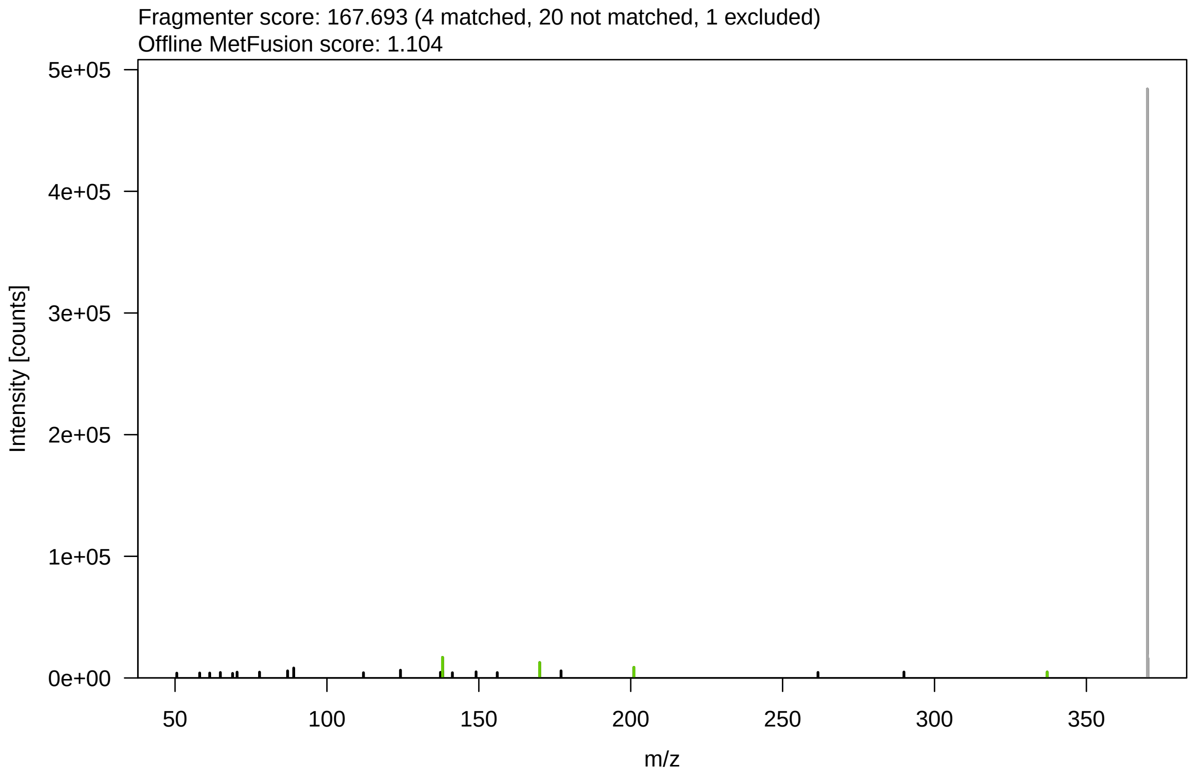

Figure SI-D297: Measured MS2 spectrum. Matching fragments with 4-desmethylpantoprazole predicted by MetFrag are highlighted in green. The molecular ion in gray is not considered.

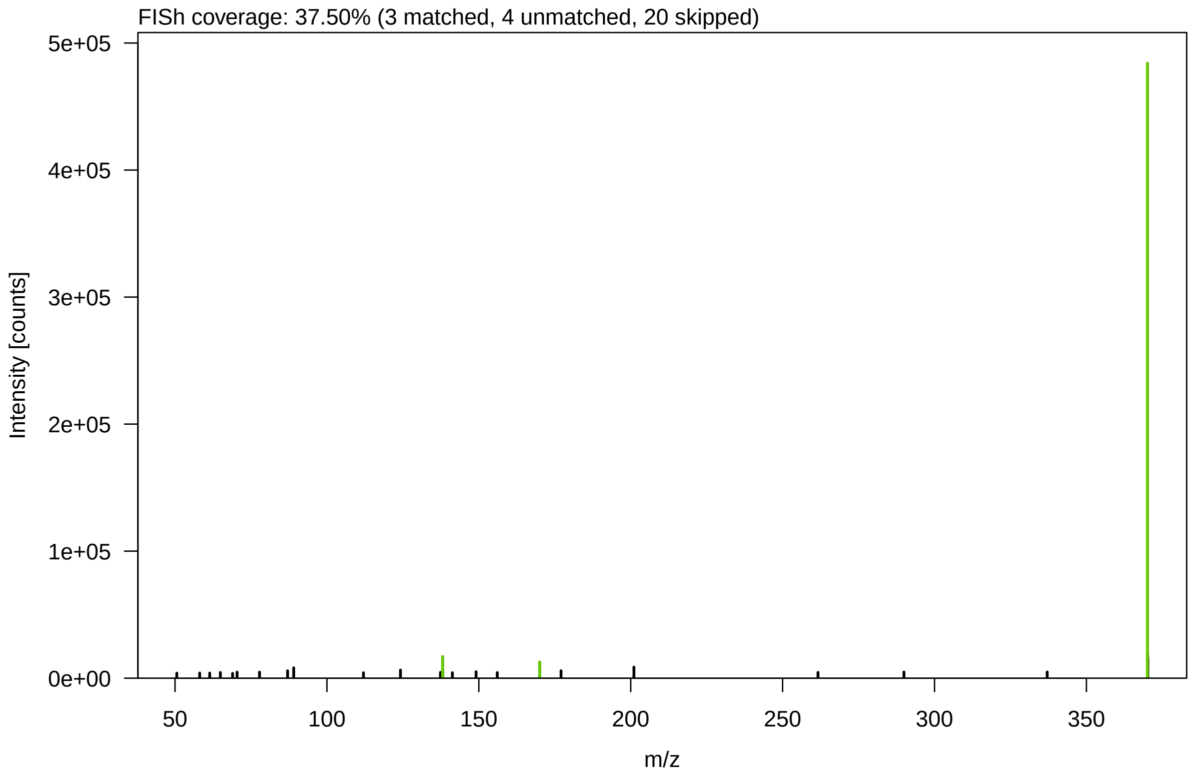

Figure SI-D298: Measured MS2 spectrum. Matching fragments with 4-desmethylpantoprazole predicted by FISh Scoring are highlighted in green. Low intensity fragments are not considered and skipped.

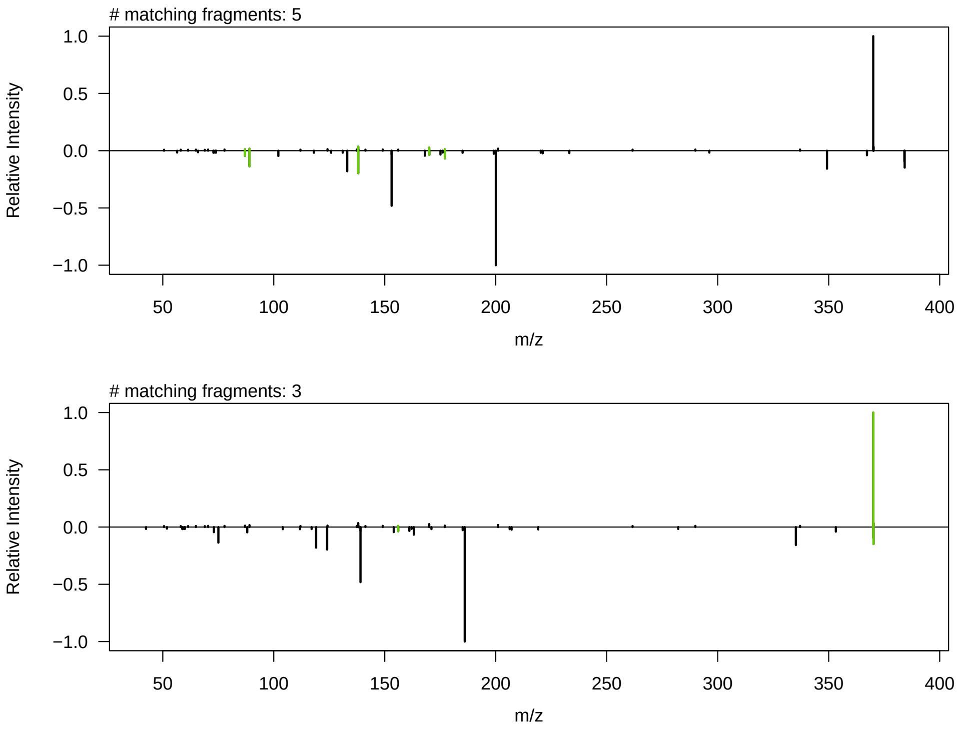

Figure SI-D299: Head to tail plots of 4-desmethylpantoprazole and pantoprazole. In the bottom plot, the mass spectrum of pantoprazole is shifted by the mass difference. Matching fragments are highlighted in green.

Table SI-D146: Molecular network results and retention time prediction of 4-desmethylpantoprazole.

Comparison with	Pantoprazole
MSn Score	34
Forward coverage	19
Reverse coverage	50
Forward match	18
Reverse match	8
ΔMass [g/mol]	14.0156
Measured retention time [min]	14.6
Predicted logD_{OW} (pH = 2.7)	1.39
Predicted retention time [min]	16.6
Predicted retention time range (95% confidence interval) [min]	12.0-21.2
Predicted retention time range (99% confidence interval) [min]	10.5-22.6

Table SI-D147: Annotated MS2 spectrum of 4-demethylpantoprazole.

m/z	Relative Intensity	Annotation
50.5689	8.17	
58.1035	8.44	
61.4050	8.33	

Continued on next page

Table SI-D147: Annotated MS2 spectrum of 4-demethylpantoprazole.(Continued)

64.9188	9.27	
68.9406	7.76	
70.4157	9.84	
77.8076	9.87	
87.0442	12.05	$C_4H_6O_2 + H^+$
89.0594	16.89	$C_4H_8O_2 + H^+$
112.0185	8.87	
124.2049	13.19	
137.3713	9.51	
138.0296	10.23	
138.0552	34.98	$C_7H_7NO_2 + H^+$
138.0910	11.53	
141.2907	8.88	
149.0859	10.40	
156.0647	9.11	$C_7H_9NO_3 + H^+$
170.0273	26.14	$C_7H_3F_2N_2O + H^+$
177.0544	12.08	
201.0469	18.05	$C_8H_6F_2N_2O_2 + H^+$
261.6392	9.41	
289.9359	10.08	
337.0869	10.12	
370.0666	999.00	$C_{15}H_{13}F_2N_3O_4S + H^+$
370.1263	39.27	
370.2152	33.56	

The human liver S9 incubation of pantoprazole led to the formation of a demethylated metabolite. Considering the spectral match of 0.678 (see Figure SI-D300) and the retention times of 14.6 and 14.8 minutes in the wastewater and the human liver S9 sample, respectively, further confidence could be gained that the detected feature in wastewater is 4-demethylpantoprazole. Due to this diagnostic evidence, the confidence level can be increased from level 3 to level 2b. A demethylation at the 3 position is also reported in literature[18] and a second feature corresponding to the mass of 4-desmethylpantoprazole but eluting later and with lower intensity was detected in the human liver S9 samples. The predicted logK_{OW} values are both equal to 1.92[15] and can therefore not be used to differentiate between the two isomers. However, demethylation at position 4 enables the formation of a stable amine tautomer, which is not possible when demethylation occurs at position 3.

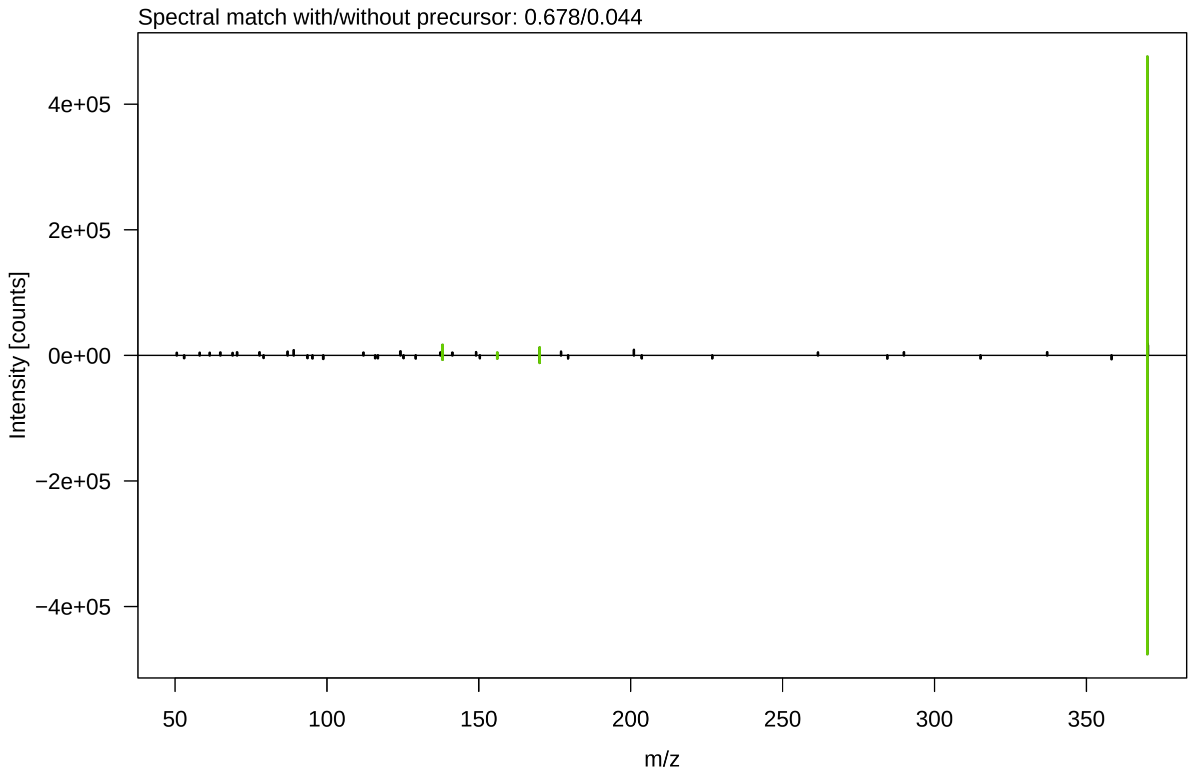

Figure SI-D300: Head to tail plot of 4-desmethylpantoprazole in wastewater (top) and from human liver S9 incubation (bottom). Matching fragments are highlighted in green.

SI-D2.14.4 5-Fluorocytosine

5-Fluorocytosine is a metabolite of capecitabine and emtricitabine. Capecitabine is a nucleoside metabolic inhibitor used in the treatment of metastatic breast and colorectal cancers.[2] Emtricitabine is a nucleoside reverse transcriptase inhibitor used in the treatment of HIV.[2] Figure SI-D301 shows the metabolism scheme.

Table SI-D148: Information on identifiers, chemical properties, detection and confidence of identification of 5-fluorocytosine.

IUPAC Name	6-amino-5-fluoro-1*H*-pyrimidin-2-one
Molecular formula	$C_4H_4FN_3O$
Monoisotopic mass [g/mol]	129.0338
Adduct	$[M+H]^+$
Retention time [min]	10.3
SMILES	C1=NC(=O)NC(=C1F)N
InChI	InChI=1S/C4H4FN3O/c5-2-1-7-4(9)8-3(2)6/h1H,(H3,6,7,8,9)
InChI-Key	XRECTZIEBJDKEO-UHFFFAOYSA-N
CAS RN	2022-85-7
Metabolite of	Emtricitabine, capecitabine
Detection frequency	100% (15/15 samples)
Detected in	Altenrhein, Monday-Friday
	Neugut, Monday-Friday
	Werdhölzli, Monday-Friday
Intensity	E7-E8
Initial confidence level	level 2a
Initial confidence score	0.74
Final confidence level	level 4

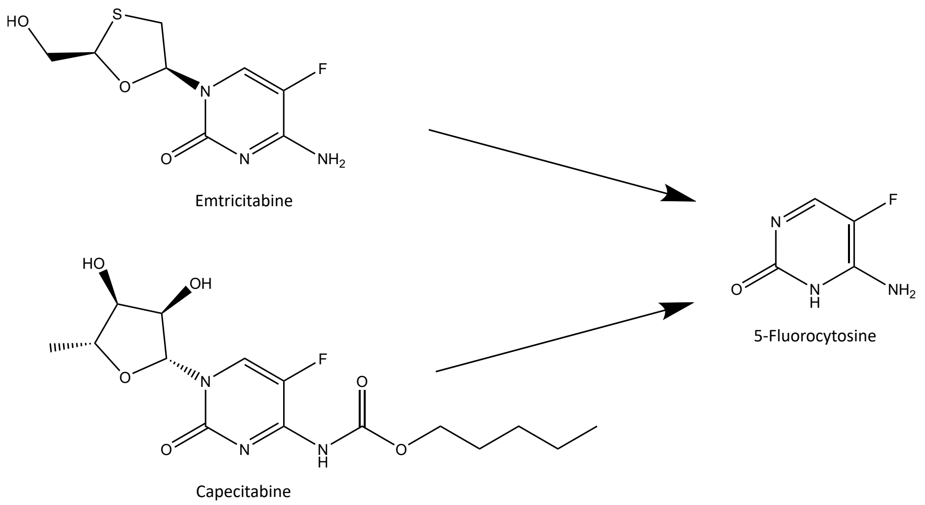

Figure SI-D301: Metabolism of emtricitabine and capecitabine to 5-fluorocytosine.

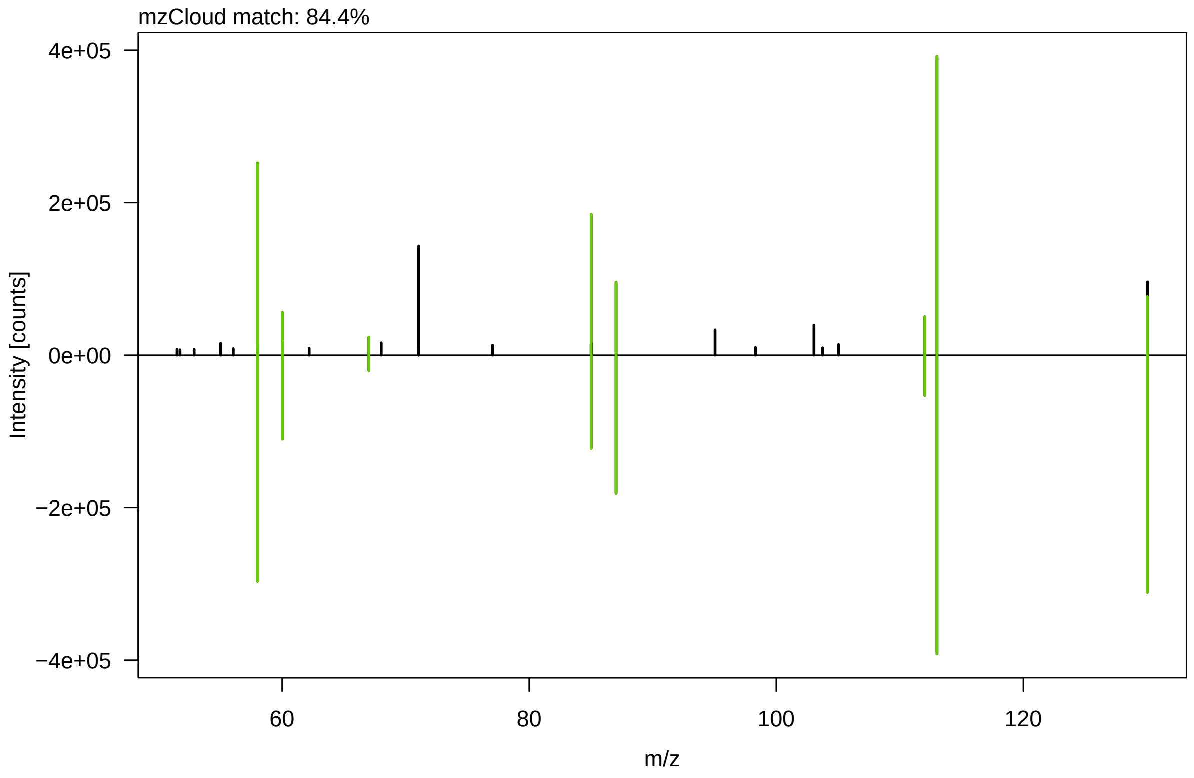

Figure SI-D302: Head to tail plot of measured MS2 spectrum against mzCloud library spectrum of 5-fluorocytosine. Matching fragments are highlighted in green.

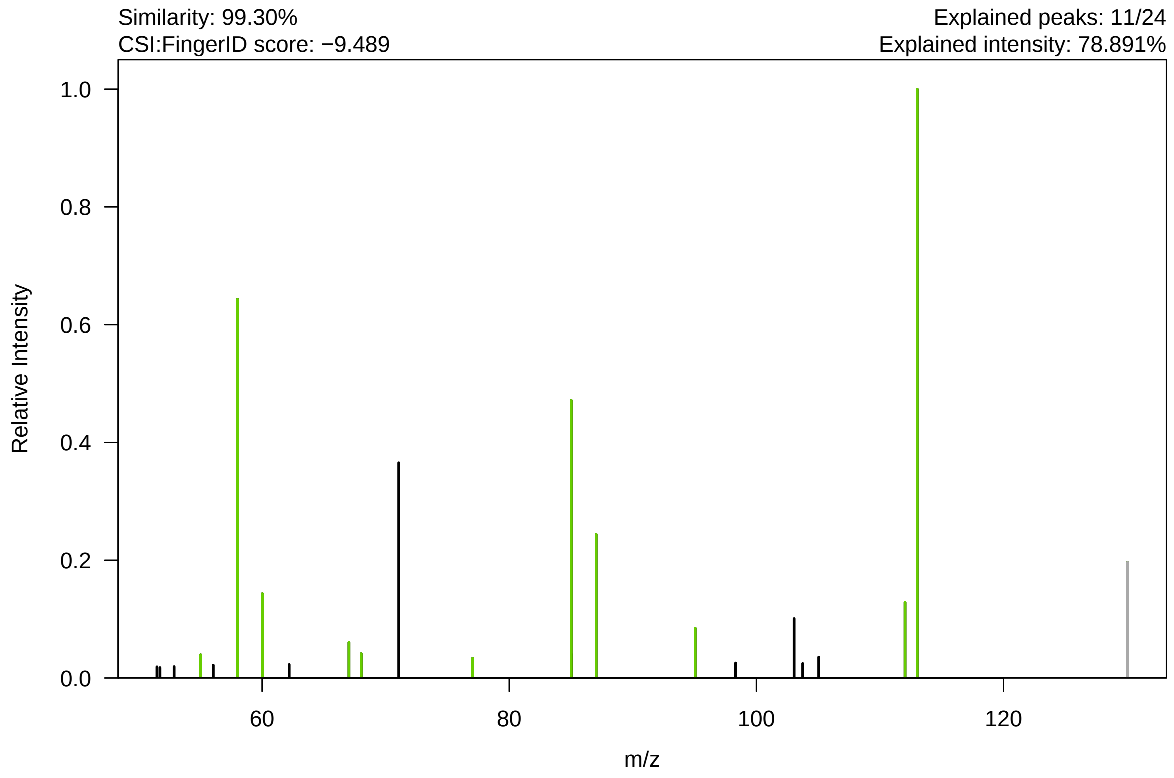

Figure SI-D303: Measured MS2 spectrum. Matching fragments with 5-fluorocytosine predicted by SIRIUS/CSI:FingerID are highlighted in green. The molecular ion in gray is not considered.

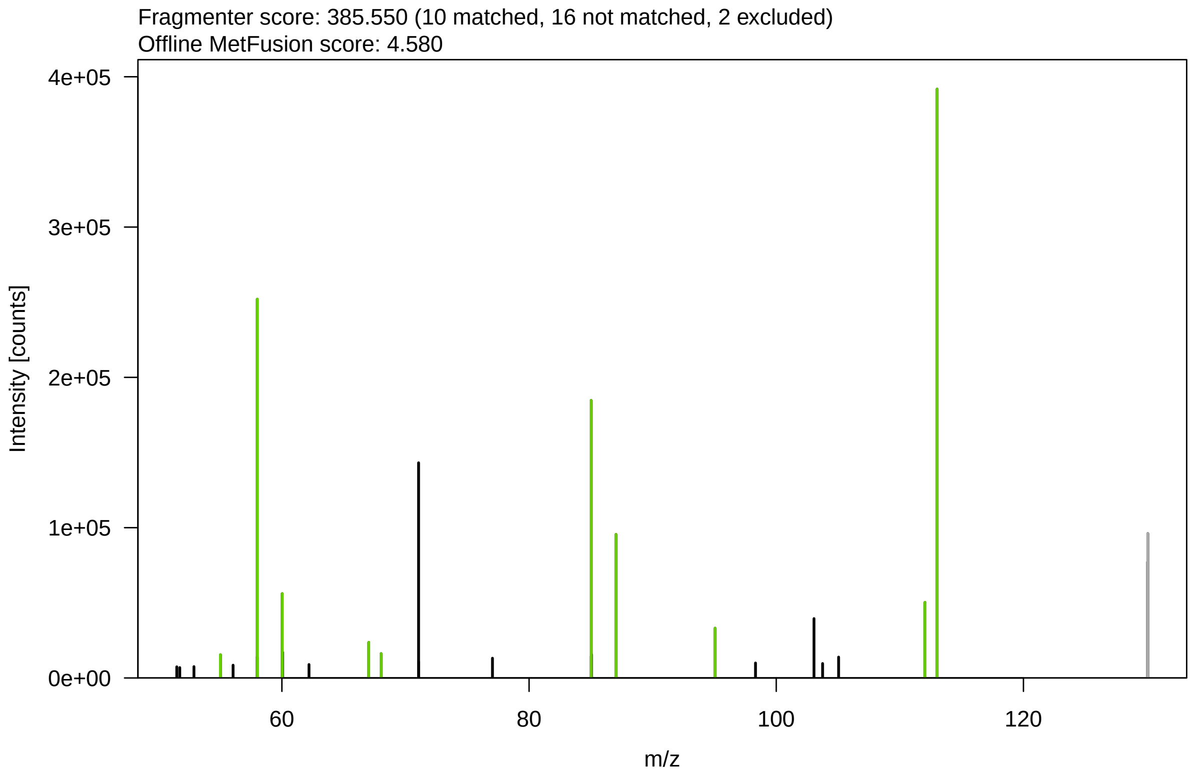

Figure SI-D304: Measured MS2 spectrum. Matching fragments with 5-fluorocytosine predicted by MetFrag are highlighted in green. The molecular ion in gray is not considered.

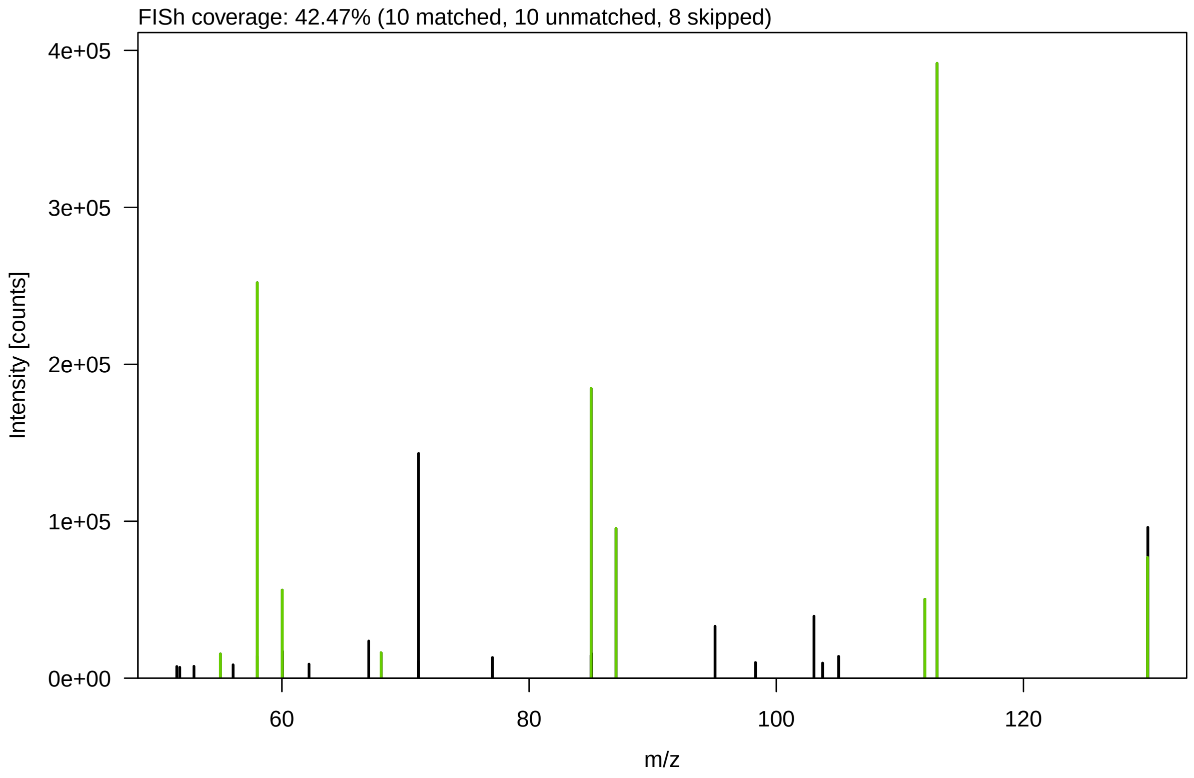

Figure SI-D305: Measured MS2 spectrum. Matching fragments with 5-fluorocytosine predicted by FISh Scoring are highlighted in green. Low intensity fragments are not considered and skipped.

Table SI-D149: Retention time prediction of 5-fluorocytosine.

Measured retention time [min]	10.3
Predicted $logD_{OW}$ (pH = 2.7)	-0.95
Predicted retention time [min]	13.5
Predicted retention time range (95% confidence interval) [min]	8.9-18.1
Predicted retention time range (99% confidence interval) [min]	7.5-19.6

Table SI-D150: Annotated MS2 spectrum of 5-fluorocytosine.

m/z	Relative Intensity	Annotation
51.4947	18.95	
51.7362	17.59	
52.8833	19.27	
55.0294	39.43	$C_2H_2N_2 + H^+$
56.0497	21.69	
58.0063	35.74	
58.0091	642.56	$C_2FN + H^+$
60.0247	143.09	$C_2H_2FN + H^+$
60.0558	43.81	
62.1919	22.81	
67.0292	60.38	$C_3H_2N_2 + H^+$
68.0244	41.33	$C_2HN_3 + H^+$
71.0568	27.54	
71.0604	365.08	
77.0384	33.54	$CH_3FN_3 + H^+$
85.0148	37.10	
85.0196	470.82	$C_3HFN_2 + H^+$
85.0507	39.91	
87.0354	243.53	$C_3H_3FN_2 + H^+$
95.0490	84.47	$C_4H_4N_3 + H^+$
98.3207	25.40	
103.0542	100.73	
103.7493	24.51	
105.0447	35.43	
112.0305	128.23	$C_4H_2FN_3 + H^+$
113.0145	999.00	$C_4FHN_2O + H^+$
130.0410	196.28	$C_4H_4FNO_3 + H^+$
130.0651	245.00	

A reference standard of 5-fluorocytosine was purchased. Figure SI-D306 shows the extracted ion chromatograms of this standard, the sample and the spiked sample. It becomes visible that the suspected compound elutes about five minutes later than the reference standard of 5-fluorocytosine. The suspected compound is therefore not 5-fluorocytosine and the confidence level is decreased to level 4 due to the unequivocal molecular formula.

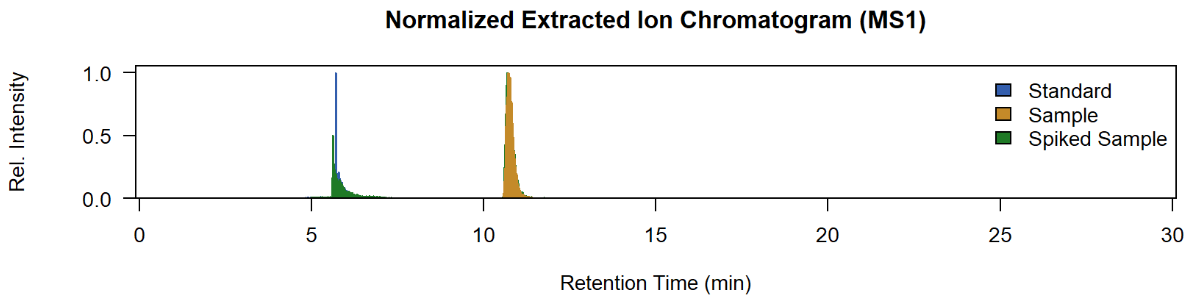

Figure SI-D306: Extracted ion chromatograms of 5-fluorocytosine in the reference standard and of the suspected compound in the sample and the spiked sample.

SI-D2.14.5 5-Hydroxyomeprazole-Sulfone

5-Hydroxyomeprazole-sulfone is a metabolite of omeprazole. Omeprazole is a proton-pump inhibitor used to treat gastric acid-related disorders.[2] Figure SI-D307 shows the metabolism scheme.

Table SI-D151: Information on identifiers, chemical properties, detection and confidence of identification of 5-hydroxyomeprazole-sulfone.

IUPAC Name	[4-methoxy-6-[(6-methoxy-1*H*-benzimidazol-2-yl)sulfonylmethyl]-5-methylpyridin-3-yl]methanol
Molecular formula	$C_{17}H_{19}N_3O_5S$
Monoisotopic mass [g/mol]	377.1045
Adduct	$[M+H]^+$
Retention time [min]	16.5
SMILES	CC1=C(C(=CN=C1CS(=O)(=O)C2=NC3=C(N2)C=C (C=C3)OC)CO)OC
InChI	InChI=1S/C17H19N3O5S/c1-10-15(18-7-11(8-21)16(10)25-3)9-26(22,23)17-19-13-5-4-12(24-2)6-14(13)20-17/h4-7,21H,8-9H2,1-3H3,(H,19,20)
InChI-Key	NXSJDCMGNOAVHM-UHFFFAOYSA-N
CAS RN	151602-51-6
Metabolite of	Omeprazole
Detection frequency	100% (15/15 samples)
Detected in	Altenrhein, Monday-Friday
	Neugut, Monday-Friday
	Werdhölzli, Monday-Friday
Intensity	E6-E7
Initial confidence level	level 3
Initial confidence score	0.42
Final confidence level	level 2b

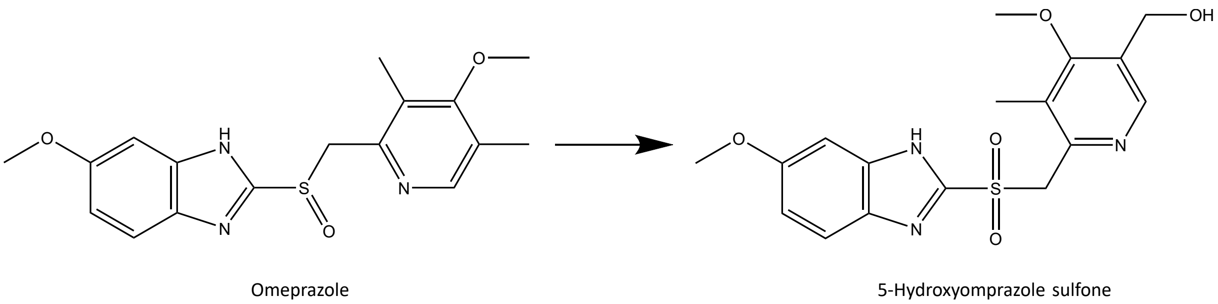

Figure SI-D307: Metabolism of omeprazole to 5-hydroxyomeprazole-sulfone.

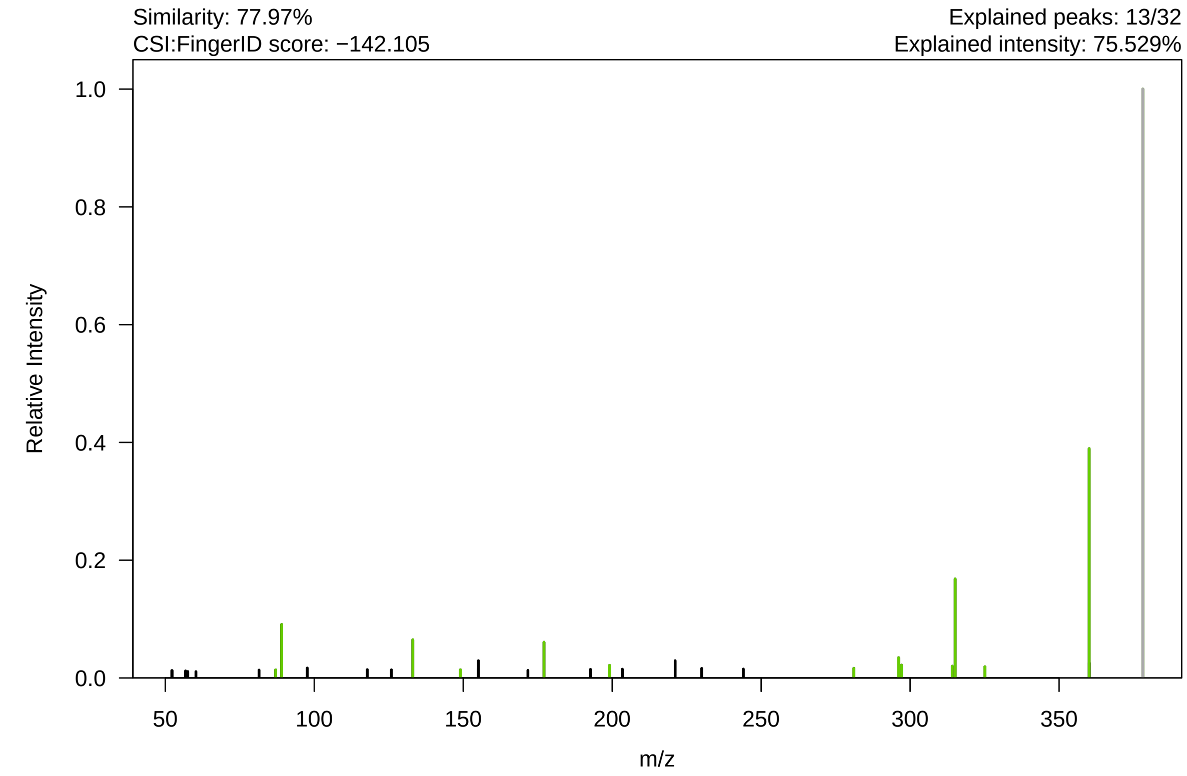

Figure SI-D308: Measured MS2 spectrum. Matching fragments with 5-hydroxyomeprazole-sulfone predicted by SIRIUS/CSI:FingerID are highlighted in green. The molecular ion in gray is not considered.

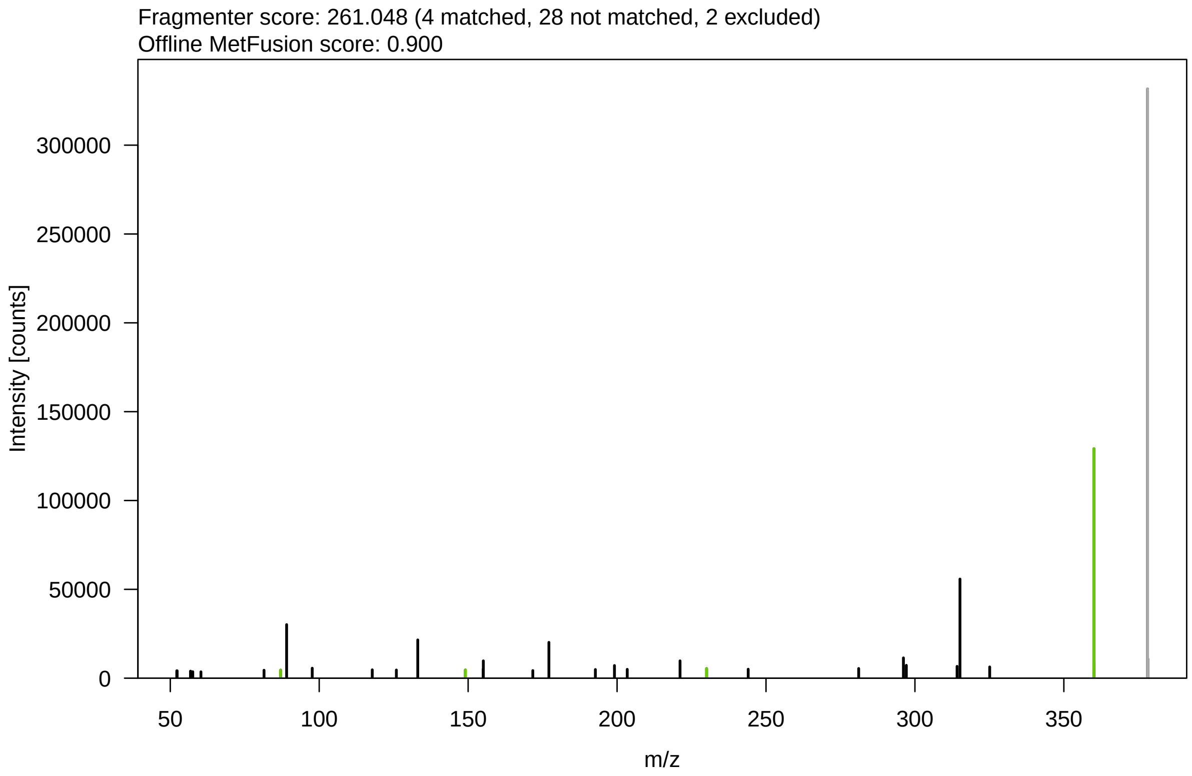

Figure SI-D309: Measured MS2 spectrum. Matching fragments with 5-hydroxyomeprazole-sulfone predicted by MetFrag are highlighted in green. The molecular ion in gray is not considered.

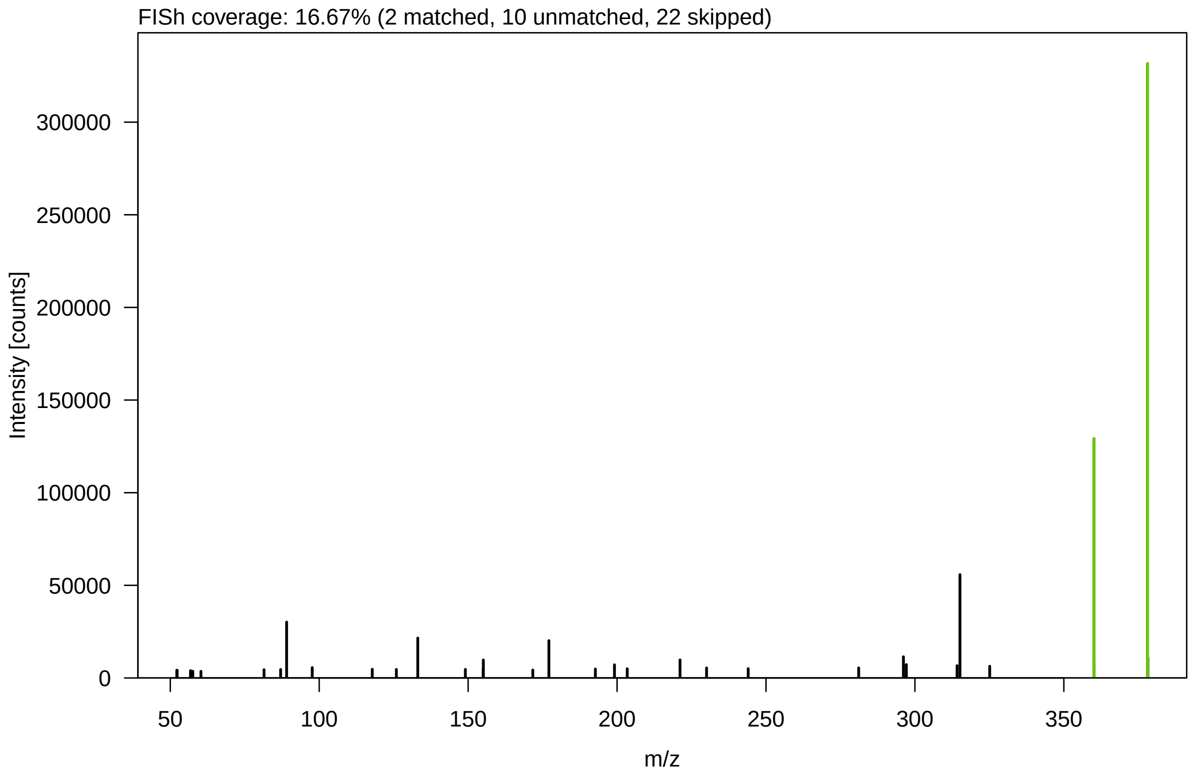

Figure SI-D310: Measured MS2 spectrum. Matching fragments with 5-hydroxyomeprazole-sulfone predicted by FISh Scoring are highlighted in green. Low intensity fragments are not considered and skipped.

Table SI-D152: Retention time prediction of 5-hydroxyomeprazole-sulfone.

Measured retention time [min]	16.5
Predicted logD_{OW} (pH = 2.7)	0.40
Predicted retention time [min]	15.3
Predicted retention time range (95% confidence interval) [min]	10.7-19.9
Predicted retention time range (99% confidence interval) [min]	9.2-21.3

Table SI-D153: Annotated MS2 spectrum of 5-hydroxyomeprazole sulfone.

m/z	Relative Intensity	Annotation
52.1802	12.55	
52.2966	12.59	
56.7980	11.93	
56.8227	11.69	
57.5520	10.99	
60.2836	10.79	
81.4704	13.47	
87.0437	13.74	$C_4H_6O_2 + H^+$
89.0596	90.84	$C_4H_8O_2 + H^+$
97.6554	16.95	
117.8081	14.18	
125.9009	13.89	
133.0857	64.89	$C_6H_{12}O_3 + H^+$
149.0715	13.90	$C_8H_8N_2O + H^+$
155.0557	15.24	
155.0992	29.36	
171.7125	12.93	
177.1121	60.73	$C_8H_{16}O_4 + H^+$
192.7178	14.63	
199.1245	21.44	$C_{13}H_{14}N_2 + H^+$
203.4160	14.99	
221.1377	29.39	
230.0482	16.31	$C_9H_{11}NO_4S + H^+$
244.0240	15.21	
281.1152	16.40	$C_{16}H_{14}N_3O_2 + H^+$
296.1384	34.44	$C_{17}H_{17}N_3O_2 + H^+$
297.1137	21.79	$C_{16}H_{14}N_3O_3 + H^+$
314.1497	20.05	$C_{17}H_{19}N_3O_3 + H^+$
315.1227	168.05	$C_{16}H_{16}N_3O_4 + H^+$
325.1060	19.08	$C_{17}H_{14}N_3O_4 + H^+$
360.1007	389.10	$C_{17}H_{17}N_3O_4S + H^+$
360.1440	25.69	$C_{17}H_{17}N_3O_4S + H^+$
378.1113	999.00	$C_{17}H_{19}N_3O_5S + H^+$
378.1949	32.91	

The human liver S9 incubation of omeprazole led to the formation of 5-hydroxyomeprazole-sulfone. Considering the spectral match of 0.625 (see Figure SI-D311) and the retention times of 16.5 and 16.9 minutes in the wastewater and the human liver S9 sample, respectively, further confidence could be gained that the detected feature in wastewater is 5-hydroxyomeprazole sulfone. Due to this diagnostic evidence, the final confidence level can be increased from level 3 to level 2b. Hydroxylation would also be feasible at position 3, however, 3-hydroxyomeprazole-sulfone is not reported in literature.[2]

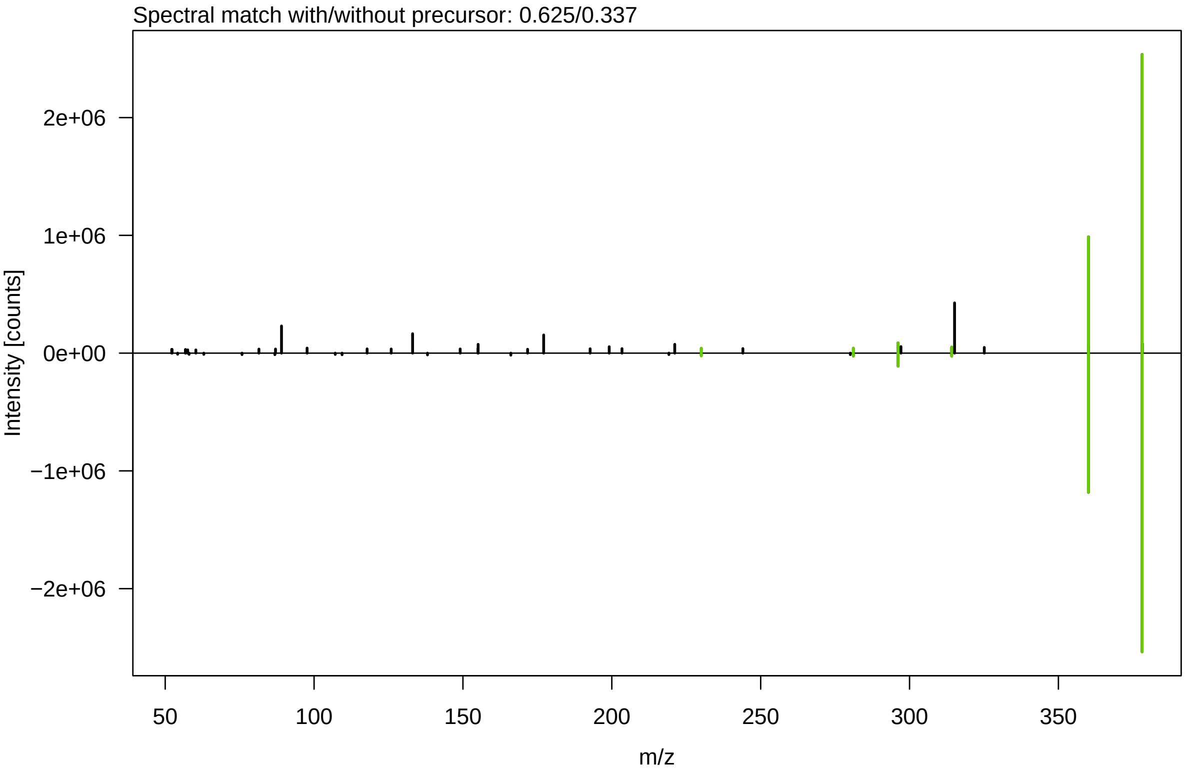

Figure SI-D311: Head to tail plot of 5-hydroxyomeprazole-sulfone in wastewater (top) and from human liver S9 incubation (bottom). Matching fragments are highlighted in green.

SI-D2.14.6 5-Hydroxysulfapyridine

5-Hydroxysulfapyridine is a metabolite of sulfasalazine, which is used to treat Crohn's disease, ulcerative colitis, and rheumatoid arthritis.[2] Figure SI-D312 shows the metabolism scheme and Figure SI-D313 the pantoprazole cluster.

Table SI-D154: Information on identifiers, chemical properties, detection and confidence of identification of 5-hydroxysulfapyridine.

IUPAC Name	4-amino-*N*-(5-hydroxypyridin-2-yl)benzenesulfonamide
Molecular formula	$C_{11}H_{11}N_3O_3S$
Monoisotopic mass [g/mol]	265.0521
Adduct	$[M+H]^+$
Retention time [min]	11.9
SMILES	C1=CC(=CC=C1N)S(=O)(=O)NC2=NC=C(C=C2)O
InChI	InChI=1S/C11H11N3O3S/c12-8-1-4-10(5-2-8)18(16,17)14-11-6-3-9(15)7-13-11/h1-7,15H,12H2,(H,13,14)
InChI-Key	XMCHHHBDBWYWSU-UHFFFAOYSA-N
CAS RN	50930-57-9
Metabolite of	Sulfasalazine
Detection frequency	100% (15/15 samples)
Detected in	Altenrhein, Monday-Friday
	Neugut, Monday-Friday
	Werdhölzli, Monday-Friday
Intensity	E6-E7
Initial confidence level	level 3
Initial confidence score	0.49
Final confidence level	level 1

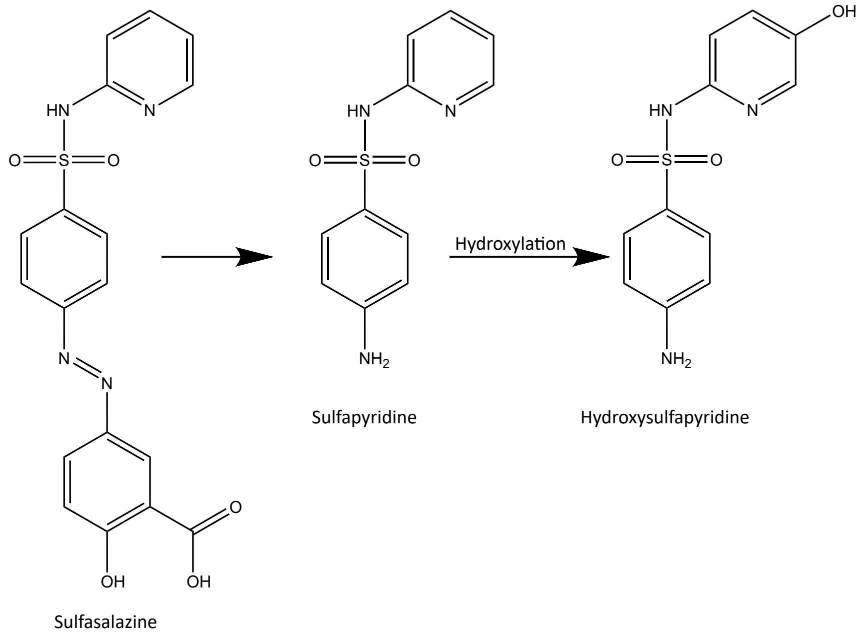

Figure SI-D312: Metabolism of sulfasalazine via sulfapyridine to 5-hydroxysulfapyridine.

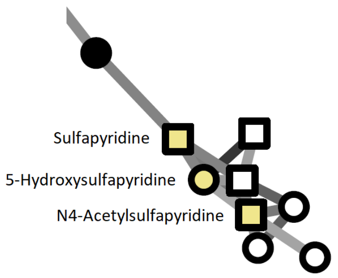

Figure SI-D313: Excerpt of the molecular network showing the sulfapyridine cluster.

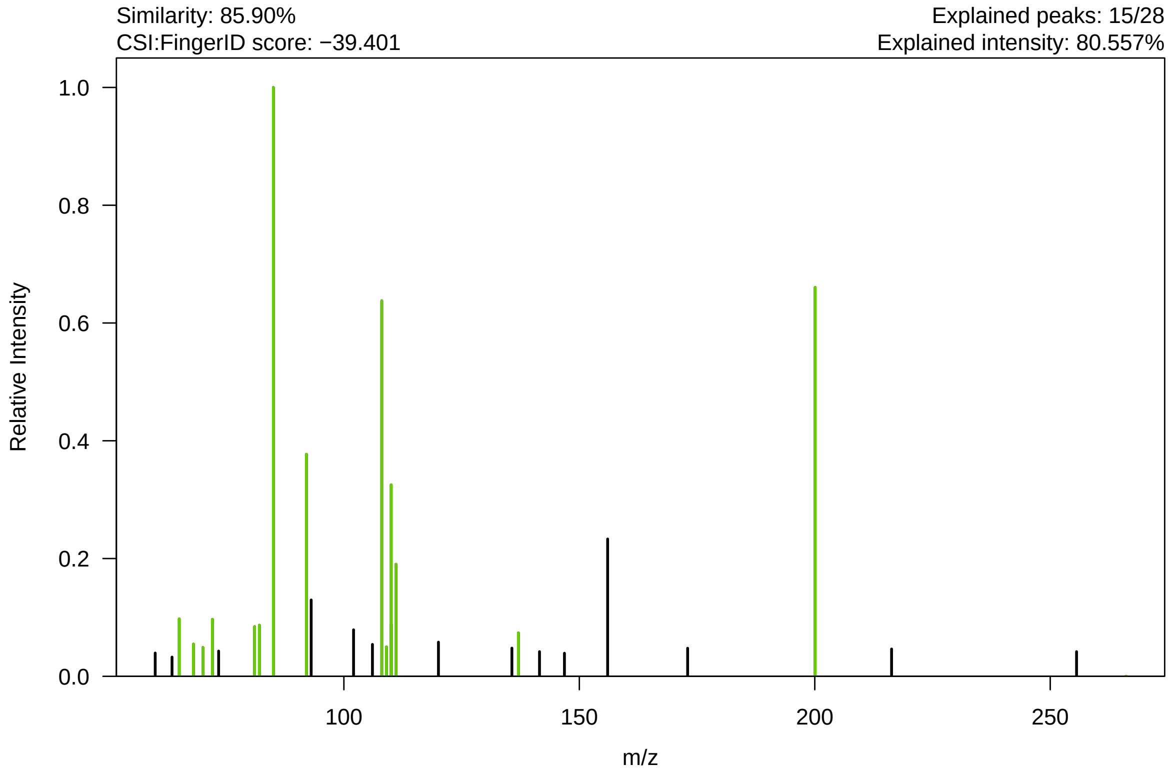

Figure SI-D314: Measured MS2 spectrum. Matching fragments with 5-hydroxysulfapyridine predicted by SIRIUS/CSI:FingerID are highlighted in green.

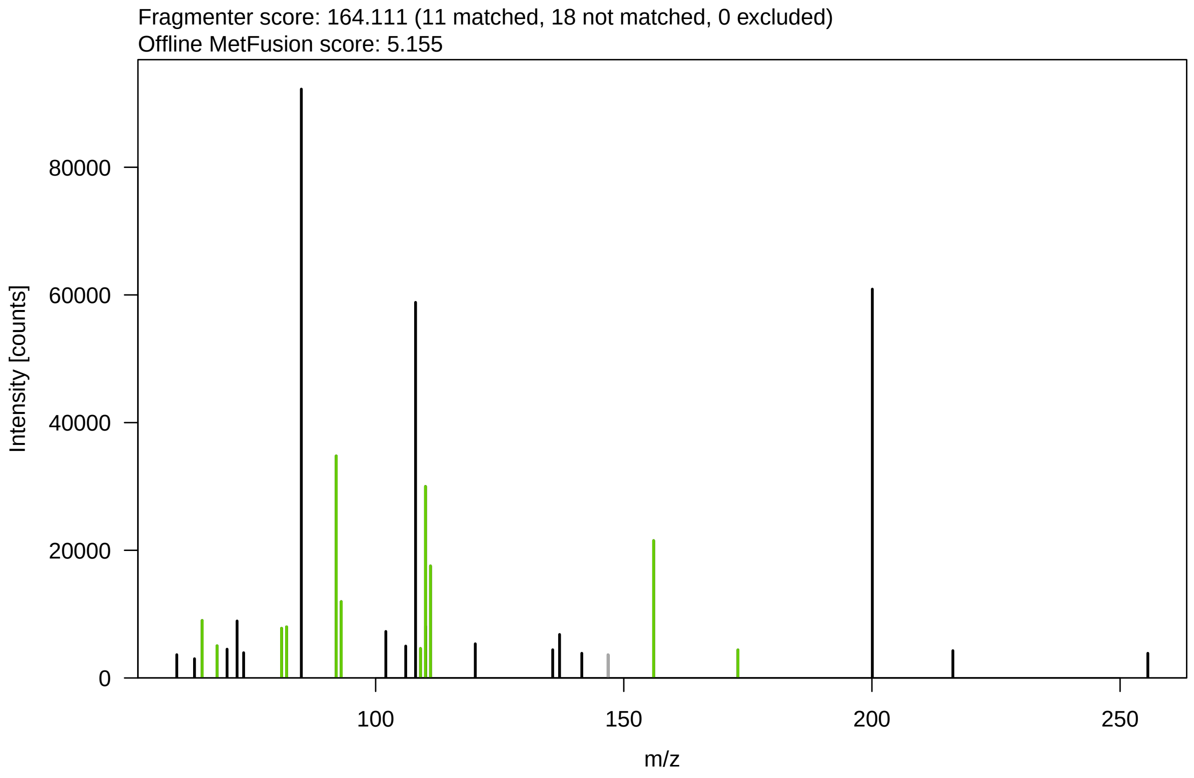

Figure SI-D315: Measured MS2 spectrum. Matching fragments with 5-hydroxysulfapyridine predicted by MetFrag are highlighted in green.

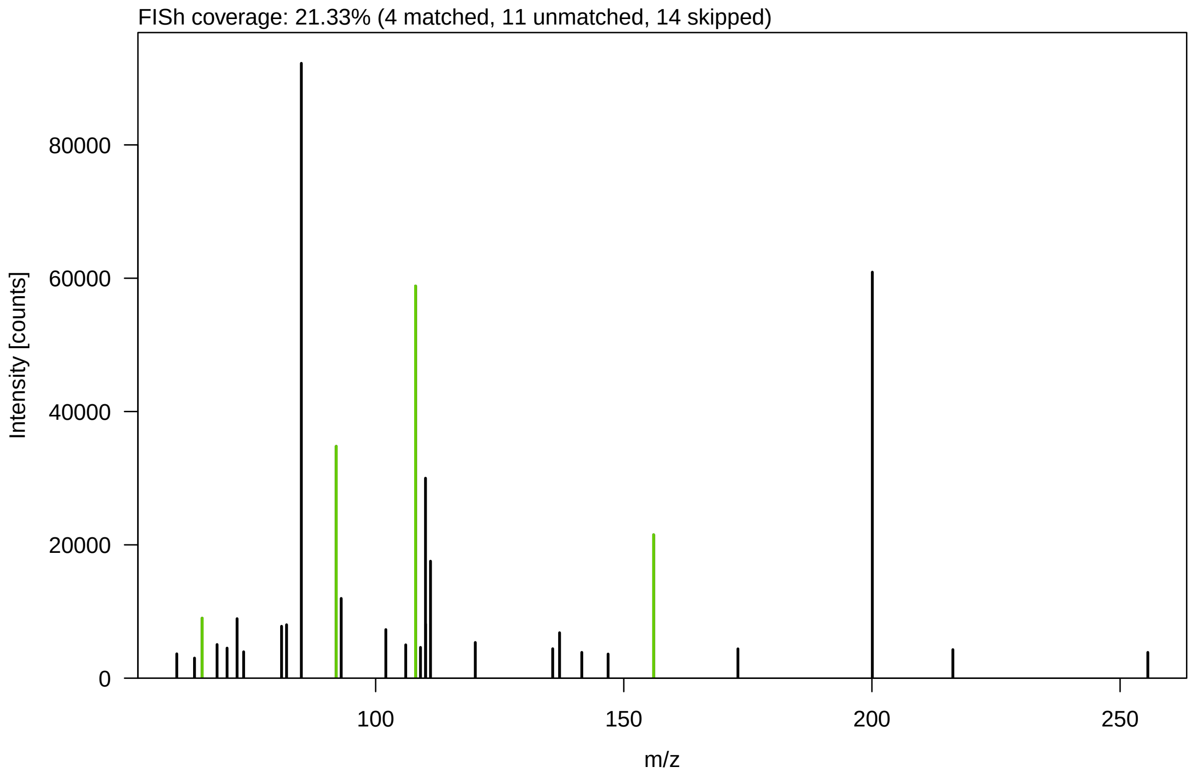

Figure SI-D316: Measured MS2 spectrum. Matching fragments with 5-hydroxysulfapyridine predicted by FISh Scoring are highlighted in green. Low intensity fragments are not considered and skipped.

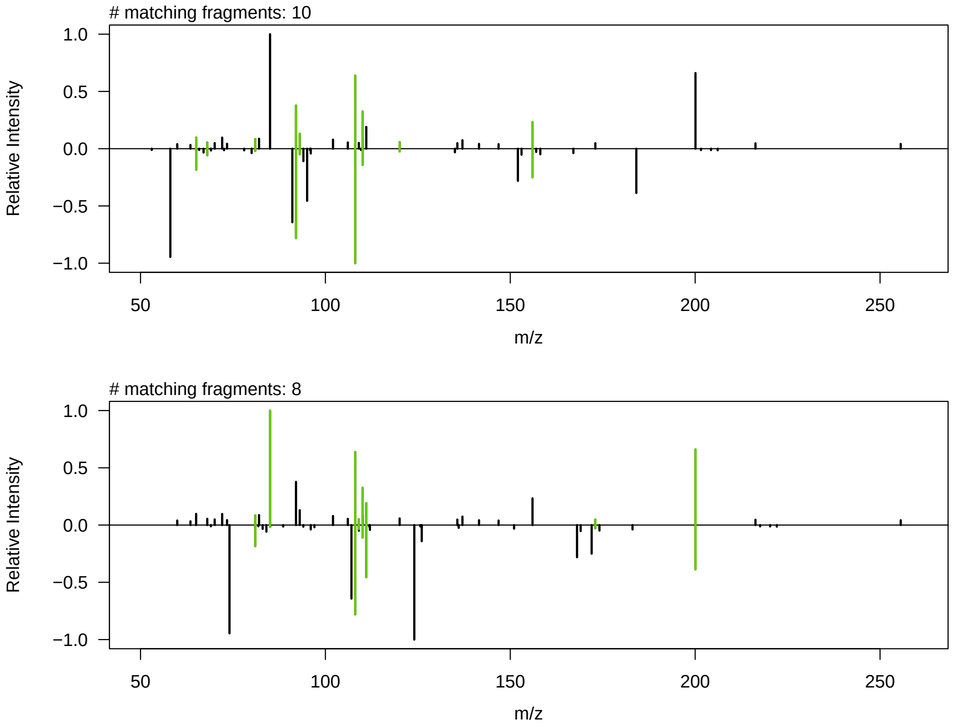

Figure SI-D317: Head to tail plots of 5-hydroxysulfapyridine and sulfapyridine. In the bottom plot, the mass spectrum of sulfapyridine is shifted by the mass difference. Matching fragments are highlighted in green.

Table SI-D155: Molecular network results and retention time prediction of 5-hydroxysulfapyridine.

Comparison with	Sulfapyridine
MSn Score	48
Forward coverage	48
Reverse coverage	48
Forward match	62
Reverse match	36
ΔMass [g/mol]	15.9949
Measured retention time [min]	11.9
Predicted logD_{OW} (pH = 2.7)	0.61
Predicted retention time [min]	15.5
Predicted retention time range (95% confidence interval) [min]	10.9-20.1
Predicted retention time range (99% confidence interval) [min]	9.5-21.6

Table SI-D156: Annotated MS2 spectrum of 5-hydroxysulfapyridine.

m/z	Relative Intensity	Annotation
59.9324	39.51	
63.5043	32.65	
65.0387	97.52	$C_5H_4 + H^+$
68.0495	54.66	$C_4H_5N + H^+$
70.0649	48.88	$C_4H_7N + H^+$
72.0807	96.68	$C_4H_9N + H^+$
73.4017	42.86	$C_4H_4N_2 + H^+$
81.0449	84.31	$C_4H_4N_2 + H^+$
82.0525	86.76	$C_4H_5N_2 + H^+$
85.0284	999.00	$C_4H_4O_2 + H^+$
92.0495	376.68	$C_6H_5N + H^+$
93.0572	129.58	C_6H_6N + H≡;
102.0548	78.92	$C_6H_5NO + H^+$
106.0646	54.14	$C_6H_5NO + H^+$
108.0444	637.20	$C_6H_5NO + H^+$
109.0395	50.00	$C_5H_4N_2O + H^+$
110.0476	324.91	$C_5H_5N_2O + H^+$
110.0597	87.80	$C_6H_7NO + H^+$
111.0550	190.09	$C_5H_6N_2O + H^+$
120.0804	58.00	
135.6791	47.85	
137.0594	73.81	$C_8H_8O_2 + H^+$
141.5426	41.91	$C_6H_5NO_2S + H^+$
146.8384	39.22	$C_6H_5NO_2S + H^+$
156.0111	233.01	$C_6H_5NO_2S + H^+$
173.0013	47.66	$C_5H_4N_2O_3S + H^+$
200.0817	659.68	$C_{11}H_9N_3O + H^+$
216.3088	46.37	
255.5922	41.87	

A reference standard of 5-hydroxysulfapyridine was purchased. Figure SI-D318 shows the extracted ion chromatograms of this standard, the sample and the spiked sample, as well as a head to tail plot of the MS2 spectra of the standard and the sample. In addition, the most intense MS2 fragments in the sample and in the standard are displayed. It becomes visible that the retention times of the sample and the spiked sample are identical and the spectra similarity score between sample and standard is equal to 0.617. Several MS2 fragments in the sample can be explained by the reference standard. It can therefore be concluded that the suspected compound is indeed 5-hydroxysulfapyridine. Correspondingly, the identification confidence can be increased to level 1.

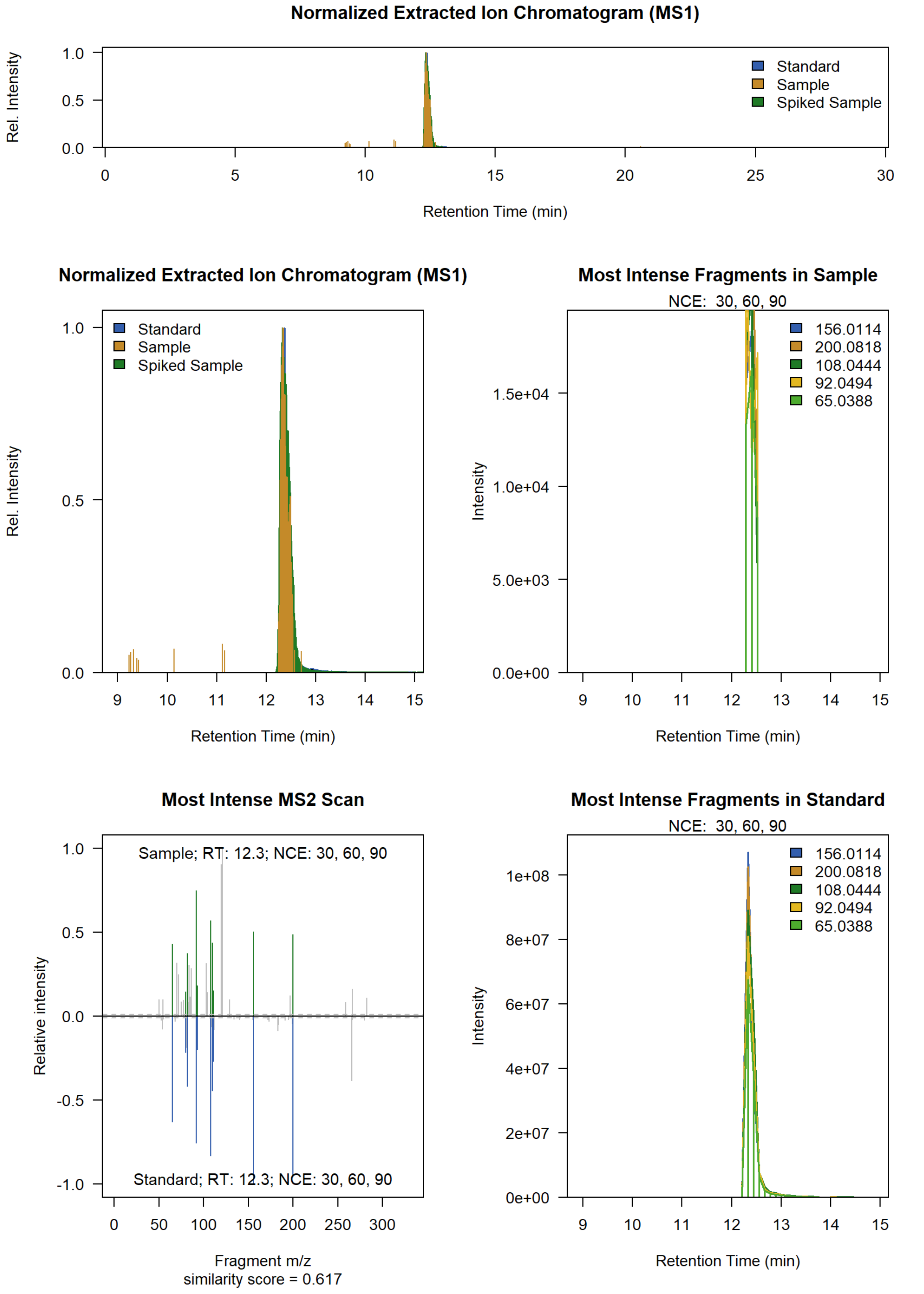

Figure SI-D318: Extracted ion chromatograms of 5-hydroxysulfapyridine in the reference standard, the sample and the spiked sample, as well as MS2 head to tail plot and most intense MS2 fragments in standard and sample.

SI-D2.14.7 6-O-Desmethylmycophenolic Acid

6-O-Desmethylmycophenolic acid is a metabolite of mycophenolic acid, the active pharmaceutical ingredient after splitting the prodrug mycophenolate mofetil. Mcophenolate mofetil is an inosine monophosphate dehydrogenase inhibitor used to prevent the rejection of kidney, heart, or liver transplants.[2] Figure SI-D319 shows the metabolism scheme.

Table SI-D157: Information on identifiers, chemical properties, detection and confidence of identification of 6-O-desmethylmycophenolic acid.

IUPAC Name	(*E*)-6-(4,6-dihydroxy-7-methyl-3-oxo-1*H*-2-benzofuran-5-yl)-4-methylhex-4-enoic acid
Molecular formula	$C_{16}H_{18}O_6$
Monoisotopic mass [g/mol]	306.1103
Adduct	$[M-H]^-$
Retention time [min]	17.0
SMILES	CC1=C2COC(=O)C2=C(C(=C1O)C/C=C(\\C)/CCC(=O)O)O
InChI	InChI=1S/C16H18O6/c1-8(4-6-12(17)18)3-5-10-14(19)9(2)11-7-22-16(21)13(11)15(10)20/h3,19-20H,4-7H2,1-2H3,(H,17,18)/b8-3+
InChI-Key	MHSRNZSBNXFMLF-FPYGCLRLSA-N
CAS RN	31858-65-8
Metabolite of	Mycophenolate mofetil, mycophenolic acid
Detection frequency	100% (15/15 samples)
Detected in	Altenrhein, Monday-Friday<br>Neugut, Monday-Friday<br>Werdhölzli, Monday-Friday
Intensity	E6-E7
Initial confidence level	level 3
Initial confidence score	0.48
Final confidence level	level 1

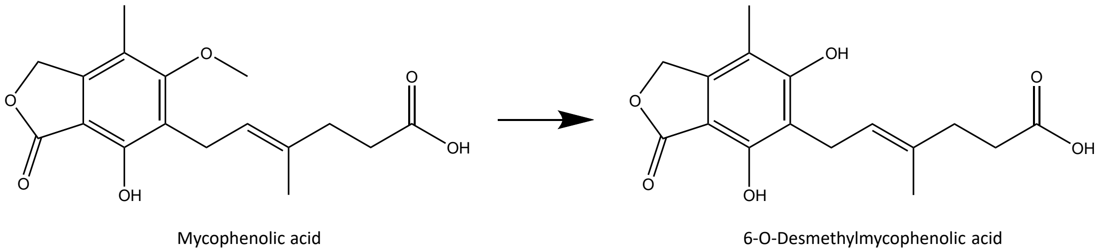

Figure SI-D319: Metabolism of mycophenolic acid to 6-O-desmethylmycophenolic acid.

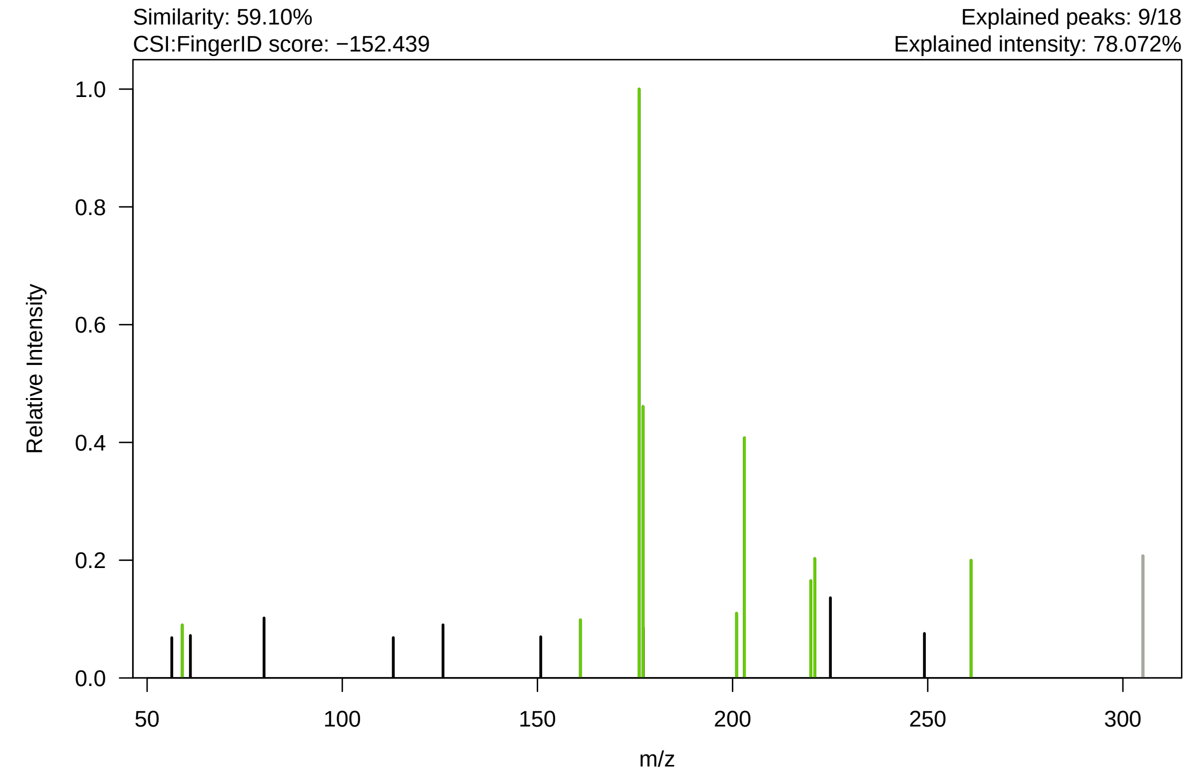

Figure SI-D320: Measured MS2 spectrum. Matching fragments with 6-O-desmethylmycophenolic acid predicted by SIRIUS/CSI:FingerID are highlighted in green. The molecular ion in gray is not considered.

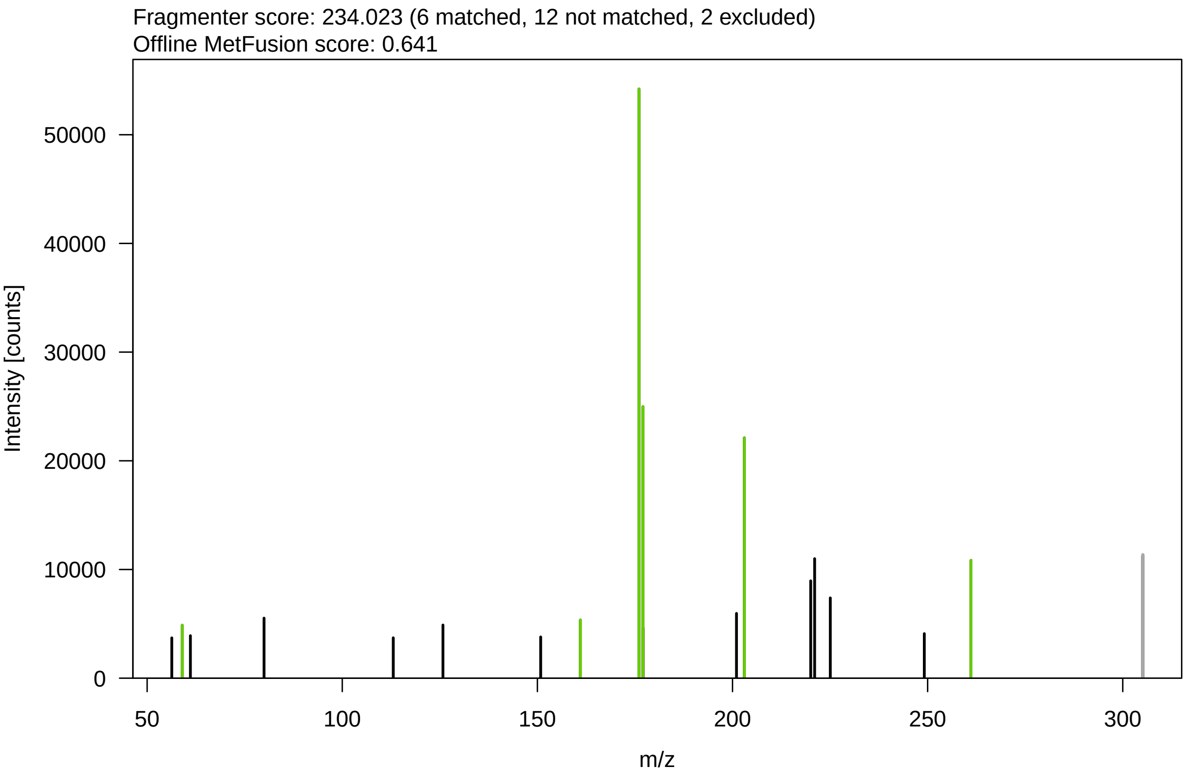

Figure SI-D321: Measured MS2 spectrum. Matching fragments with 6-O-desmethylmycophenolic acid predicted by MetFrag are highlighted in green. The molecular ion in gray is not considered.

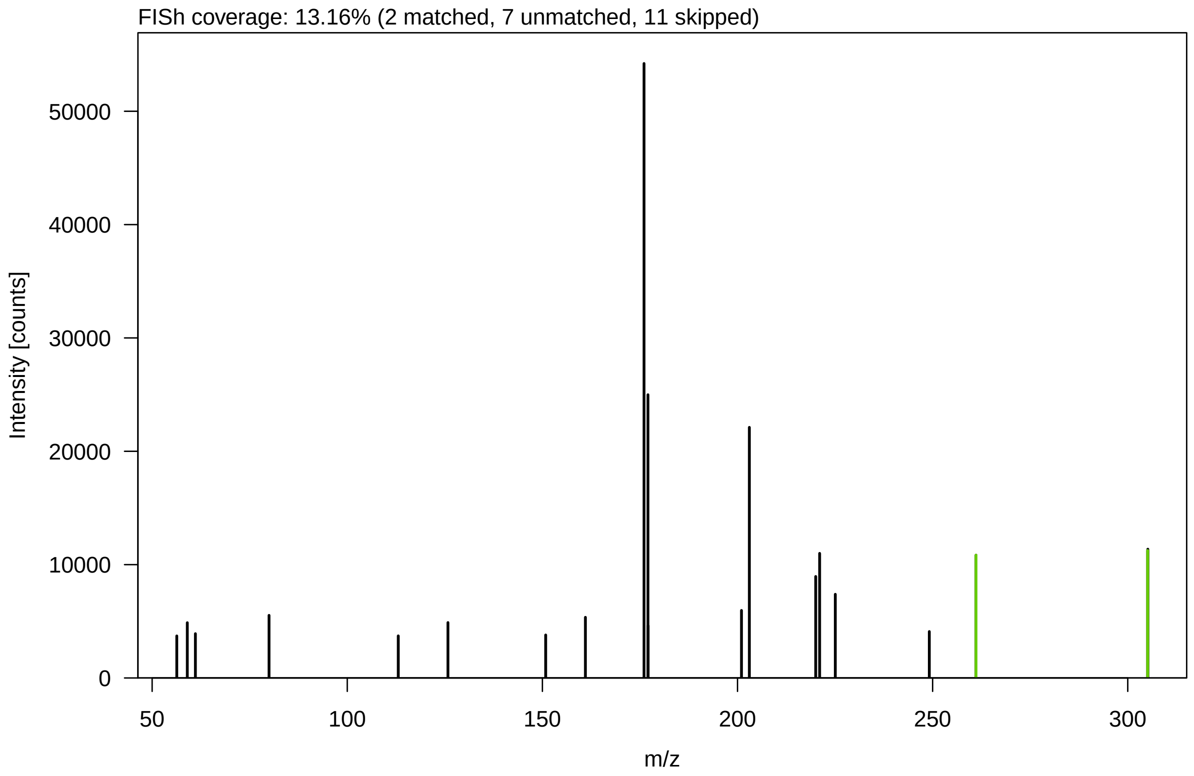

Figure SI-D322: Measured MS2 spectrum. Matching fragments with 6-O-desmethylmycophenolic acid predicted by FISh Scoring are highlighted in green. Low intensity fragments are not considered and skipped.

Table SI-D158: Retention time prediction of 6-O-desmethylmycophenolic acid.

Measured retention time [min]	17.0
Predicted $\log D_{OW}$ (pH = 4.8)	2.26
Predicted retention time [min]	18.5
Predicted retention time range (95% confidence interval) [min]	11.3-25.6
Predicted retention time range (99% confidence interval) [min]	9.0-27.9

Table SI-D159: Annotated MS2 spectrum of 6-O-desmethylmycophenolic acid.

m/z	Relative Intensity	Annotation
56.3184	68.35	
59.0130	89.85	$C_2H_4O_2 - H^-$
61.0879	72.01	
79.9571	101.79	
113.0710	68.46	
125.8039	90.09	
150.8455	69.89	
161.0251	98.60	$C_9H_6O_3 - H^-$
176.0479	999.00	$C_{10}H_9O_3 - H^-$
177.0557	460.49	$C_{10}H_{10}O_3 - H^-$
177.0865	85.30	
201.0188	109.79	$C_{11}H_6O_4 - H^-$
203.0349	407.44	$C_{11}H_8O_4 - H^-$
220.0385	165.00	$C_{11}H_9O_5 - H^-$
221.0452	202.57	$C_{11}H_{10}O_5 - H^-$
225.0575	136.06	
249.1483	75.50	
261.1131	199.66	$C_{15}H_{18}O_4 - H^-$
305.1041	207.27	$C_{16}H_{18}O_6 - H^-$
305.1426	209.39	

A reference standard of 6-O-desmethylmycophenolic acid was purchased. Figure SI-D323 shows the extracted ion chromatograms of this standard, the sample and the spiked sample, as well as a head to tail plot of the MS2 spectra of the standard and the sample. In addition, the most intense MS2 fragments in the sample and in the standard are displayed. It becomes visible that the retention times of the sample and the spiked sample are identical and the spectra similarity score between sample and standard is equal to 0.286. This low score can be explained by the low intensity signal, leading to a low quality and noisy MS2 spectrum. Nonetheless, several MS2 fragments in the sample can be explained by the reference standard. It can therefore be concluded that the suspected compound is indeed 6-O-desmethylmycophenolic acid. Correspondingly, the identification confidence can be increased to level 1.

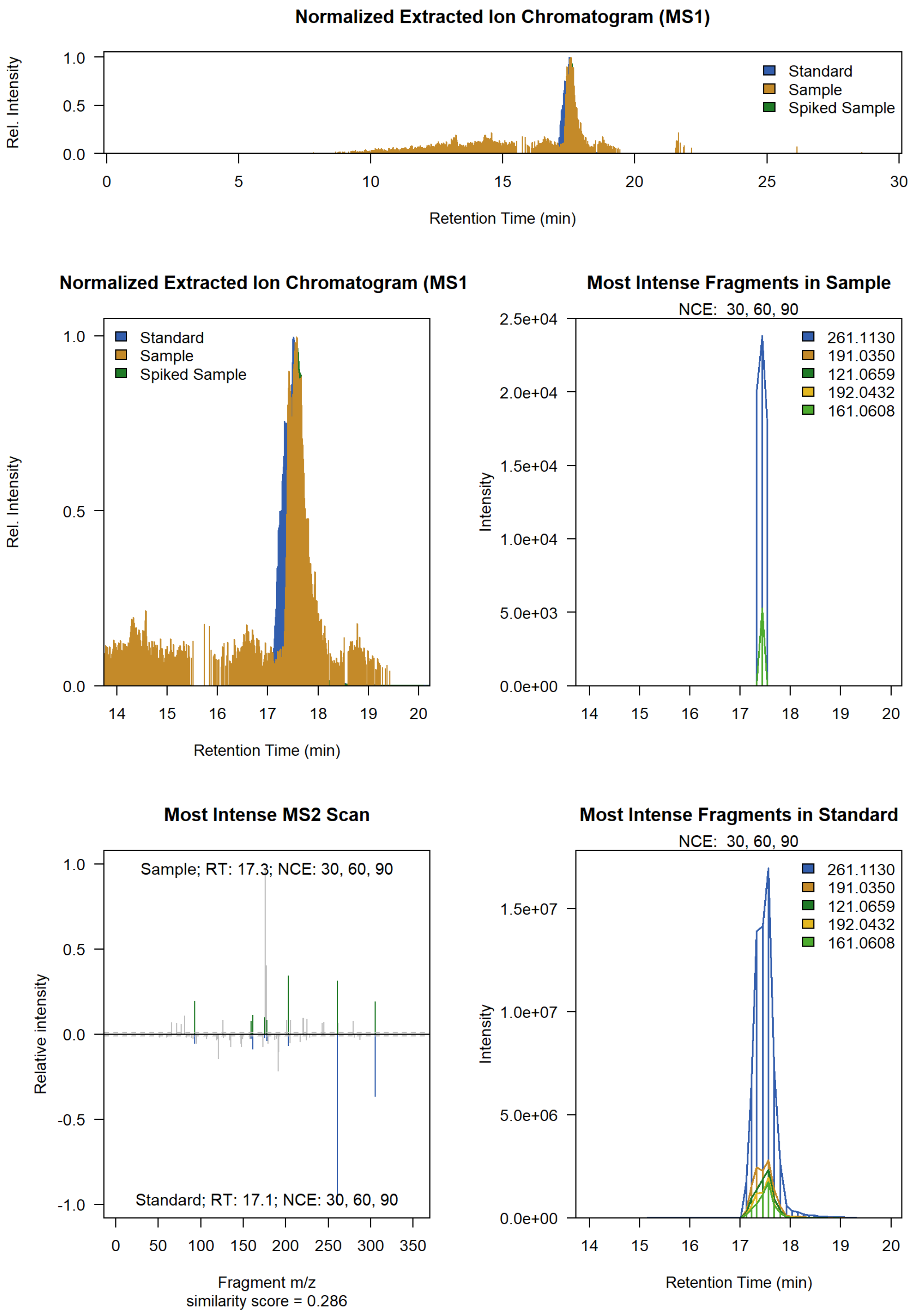

Figure SI-D323: Extracted ion chromatograms of 6-O-desmethylmycophenolic acid in the reference standard, the sample and the spiked sample, as well as MS2 head to tail plot and most intense MS2 fragments in standard and sample.

SI-D2.14.8 8-Hydroxymirtazapine

8-Hydroxymirtazapine is a metabolite of mirtazapine, which is a tetracyclic antidepressant used in the treatment of major depression.[2] Figure SI-D324 shows the metabolism scheme and Figure SI-D325 the mirtazapine cluster.

Table SI-D160: Information on identifiers, chemical properties, detection and confidence of identification of 8-hydroxymirtazapine.

IUPAC Name	5-methyl-2,5,19-triazatetracyclo[13.4.0.0^{2,7}.0^{8,13}]nonadeca-1(15),8,10,12,16,18-hexaen-17-ol
Molecular formula	$C_{17}H_{19}N_3O$
Monoisotopic mass [g/mol]	281.1528
Adduct	$[M+H]^+$
Retention time [min]	12.4
SMILES	CN1CCN2C(C1)C3=CC=CC=C3CC4=C2N=CC(=C4)O
InChI	InChI=1S/C17H19N3O/c1-19-6-7-20-16(11-19)15-5-3-2-4-12(15)8-13-9-14(21)10-18-17(13)20/h2-5,9-10,16,21H,6-8,11H2,1H3
InChI-Key	DAWYIZBOUQIVNX-UHFFFAOYSA-N
CAS RN	102335-57-9
Metabolite of	Mirtazapine
Detection frequency	100% (15/15 samples)
Detected in	Altenrhein, Monday-Friday<br>Neugut, Monday-Friday<br>Werdhölzli, Monday-Friday
Intensity	E6-E7
Initial confidence level	level 2a
Initial confidence score	0.50
Final confidence level	level 1

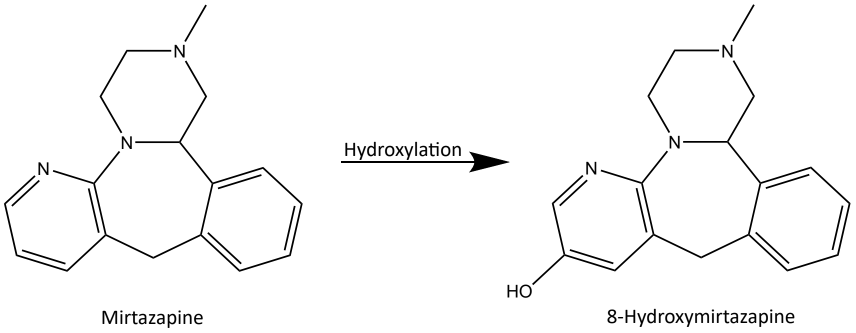

Figure SI-D324: Metabolism of mirtazapine to 8-hydroxymirtazapine.

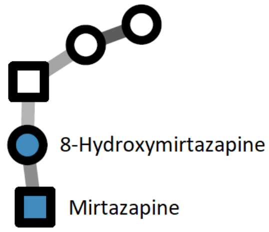

Figure SI-D325: Excerpt of the molecular network showing the mirtazapine cluster.

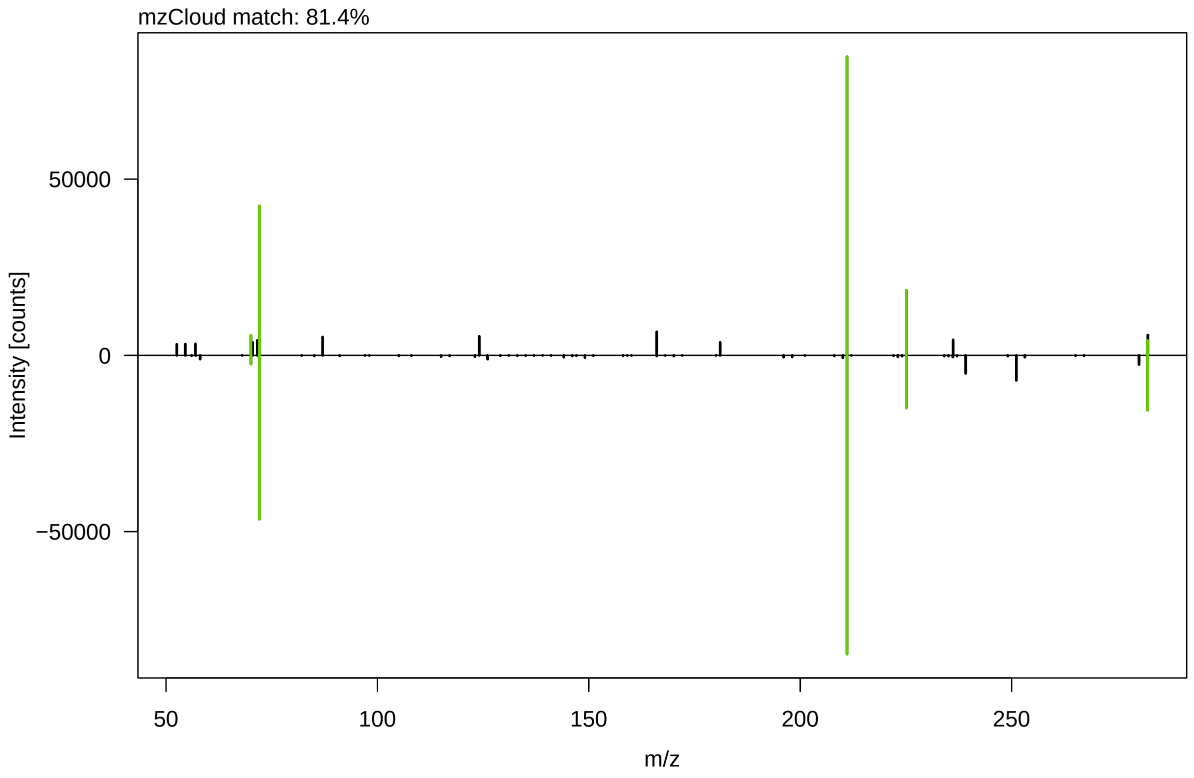

Figure SI-D326: Head to tail plot of measured MS2 spectrum against mzCloud library spectrum of 8-hydroxymirtazapine. Matching fragments are highlighted in green.

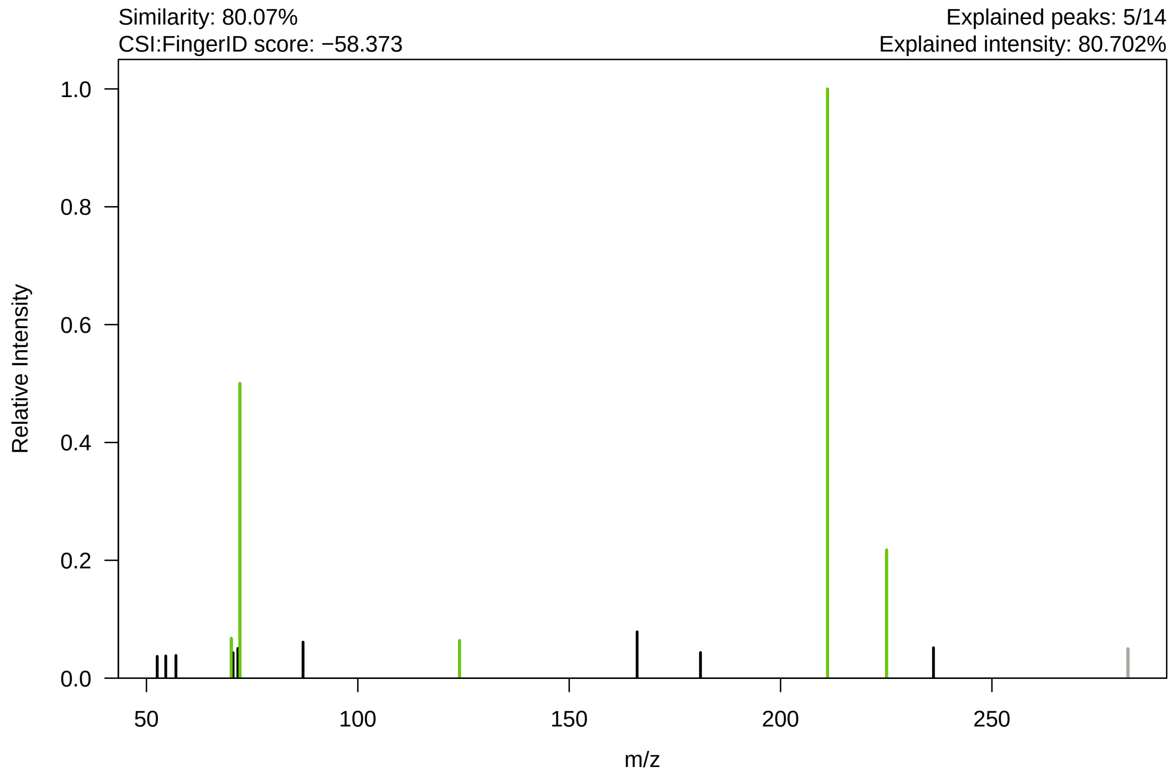

Figure SI-D327: Measured MS2 spectrum. Matching fragments with 8-hydroxymirtazapine predicted by SIRIUS/CSI:FingerID are highlighted in green. The molecular ion in gray is not considered.

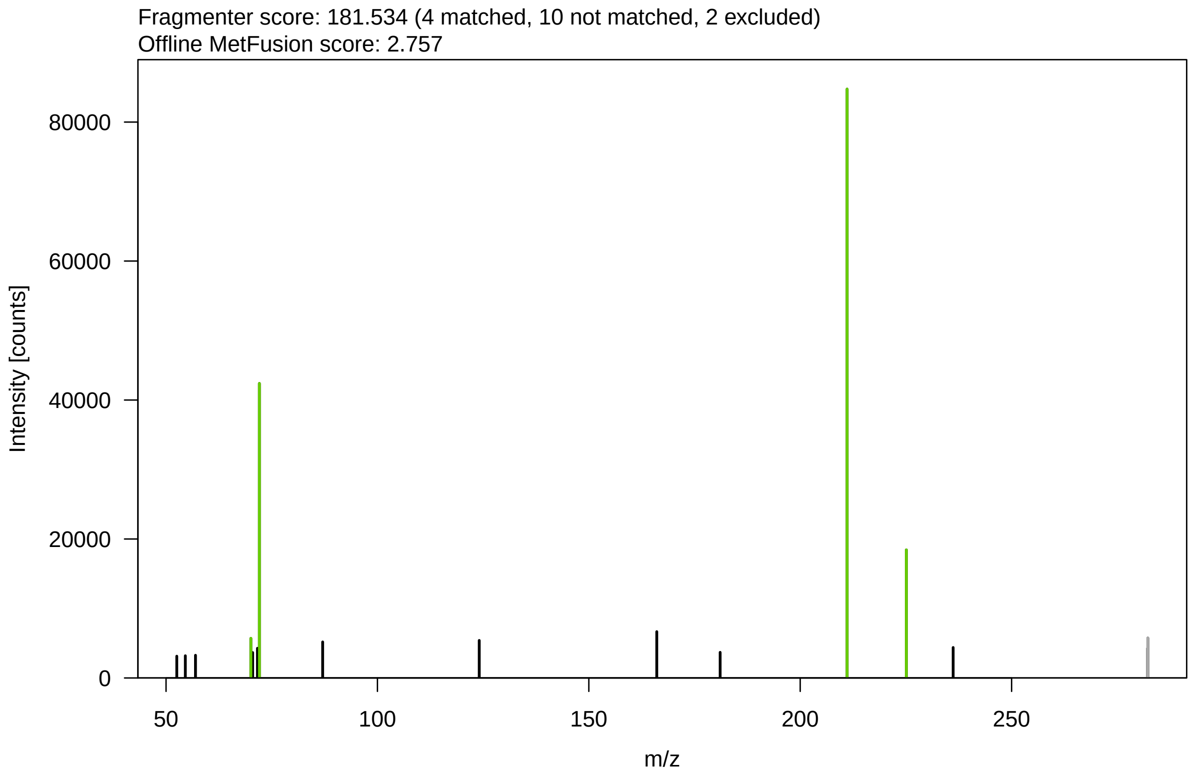

Figure SI-D328: Measured MS2 spectrum. Matching fragments with 8-hydroxymirtazapine predicted by MetFrag are highlighted in green. The molecular ion in gray is not considered.

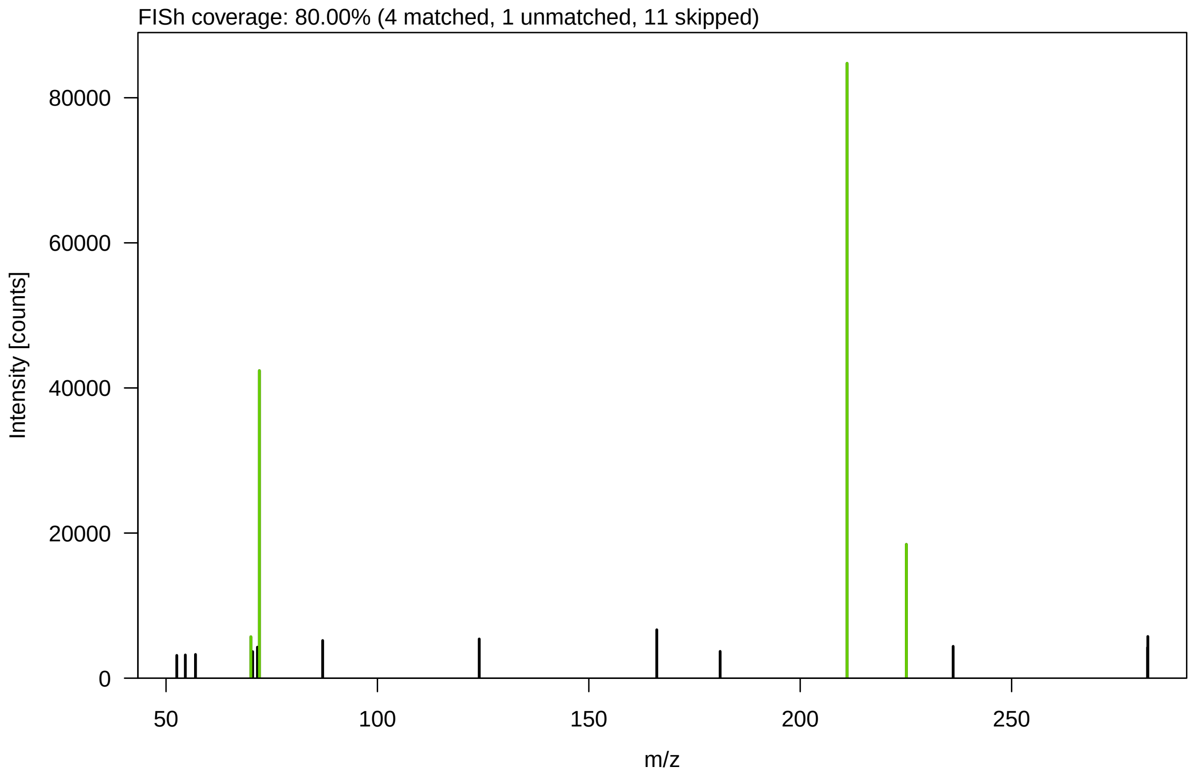

Figure SI-D329: Measured MS2 spectrum. Matching fragments with 8-hydroxymirtazapine predicted by FISh Scoring are highlighted in green. Low intensity fragments are not considered and skipped.

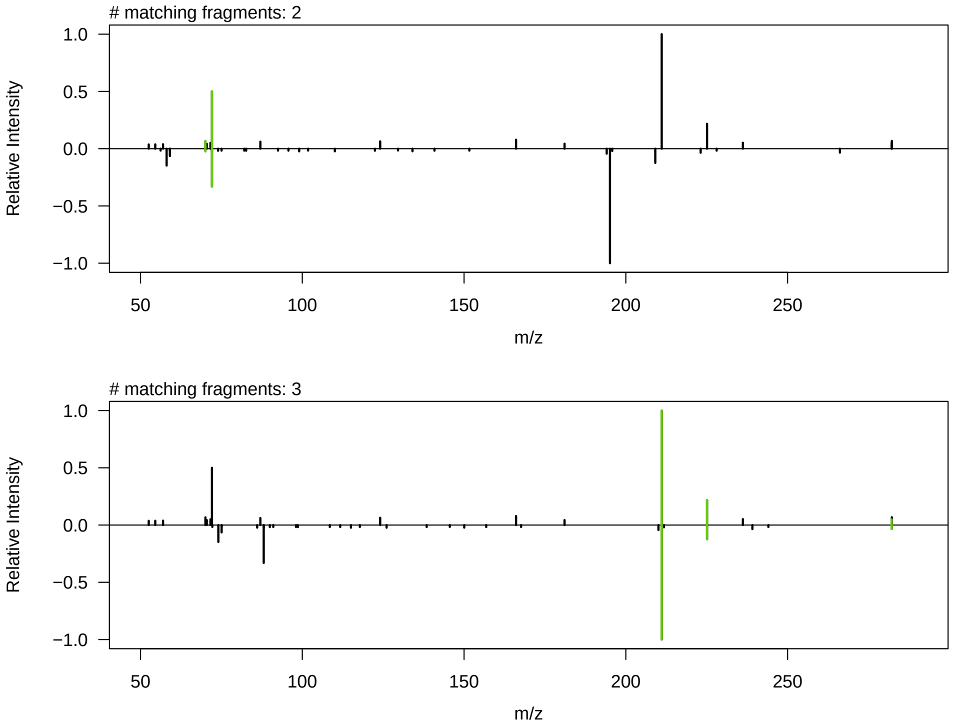

Figure SI-D330: Head to tail plots of 8-hydroxymirtazapine and mirtazapine. In the bottom plot, the mass spectrum of mirtazapine is shifted by the mass difference. Matching fragments are highlighted in green.

Table SI-D161: Molecular network results and retention time prediction of 8-hydroxymirtazapine.

Compared with	Mirtazapine
MSn Score	45%
Forward coverage	29%
Reverse coverage	60%
Forward match	5
Reverse match	3
ΔMass [g/mol]	15.9954
Measured retention time [min]	12.4
Predicted $\log D_{OW}$ (pH = 2.7)	-1.72
Predicted retention time [min]	12.5
Predicted retention time range (95% confidence interval) [min]	7.9 - 17.1
Predicted retention time range (99% confidence interval) [min]	6.4 - 18.6

Table SI-D162: Annotated MS2 spectrum of 8-hydroxymirtazapine.

m/z	Relative Intensity	Annotation
52.5386	36.96	
54.5598	37.68	
56.9623	38.52	
70.0652	67.28	$C_4H_7N + H^+$
70.4829	43.17	
71.6177	50.40	
72.0808	499.68	$C_4H_9N + H^+$
87.0438	61.15	
124.0759	63.69	$C_7H_9NO + H^+$
166.0722	78.60	
181.0593	43.50	
211.0864	999.00	$C_{13}H_{10}N_2O + H^+$
225.1020	217.34	$C_{14}H_{12}N_2O + H^+$
236.1739	51.68	
282.1567	49.89	$C_{17}H_{19}N_3O + H^+$
282.2208	67.70	

A reference standard of 8-hydroxymirtazapine was purchased. Figure SI-D331 shows the extracted ion chromatograms of this standard, the sample and the spiked sample, as well as a head to tail plot of the MS2 spectra of the standard and the sample. In addition, the most intense MS2 fragments in the sample and in the standard are displayed. It becomes visible that the retention times of the sample and the spiked sample are identical and the spectra similarity score between sample and standard is equal to 0.971. The majority of the MS2 fragments in the sample can be explained by the reference standard. It can therefore be concluded that the suspected compound is indeed 8-hydroxymirtazapine. Correspondingly, the identification confidence can be increased to level 1.

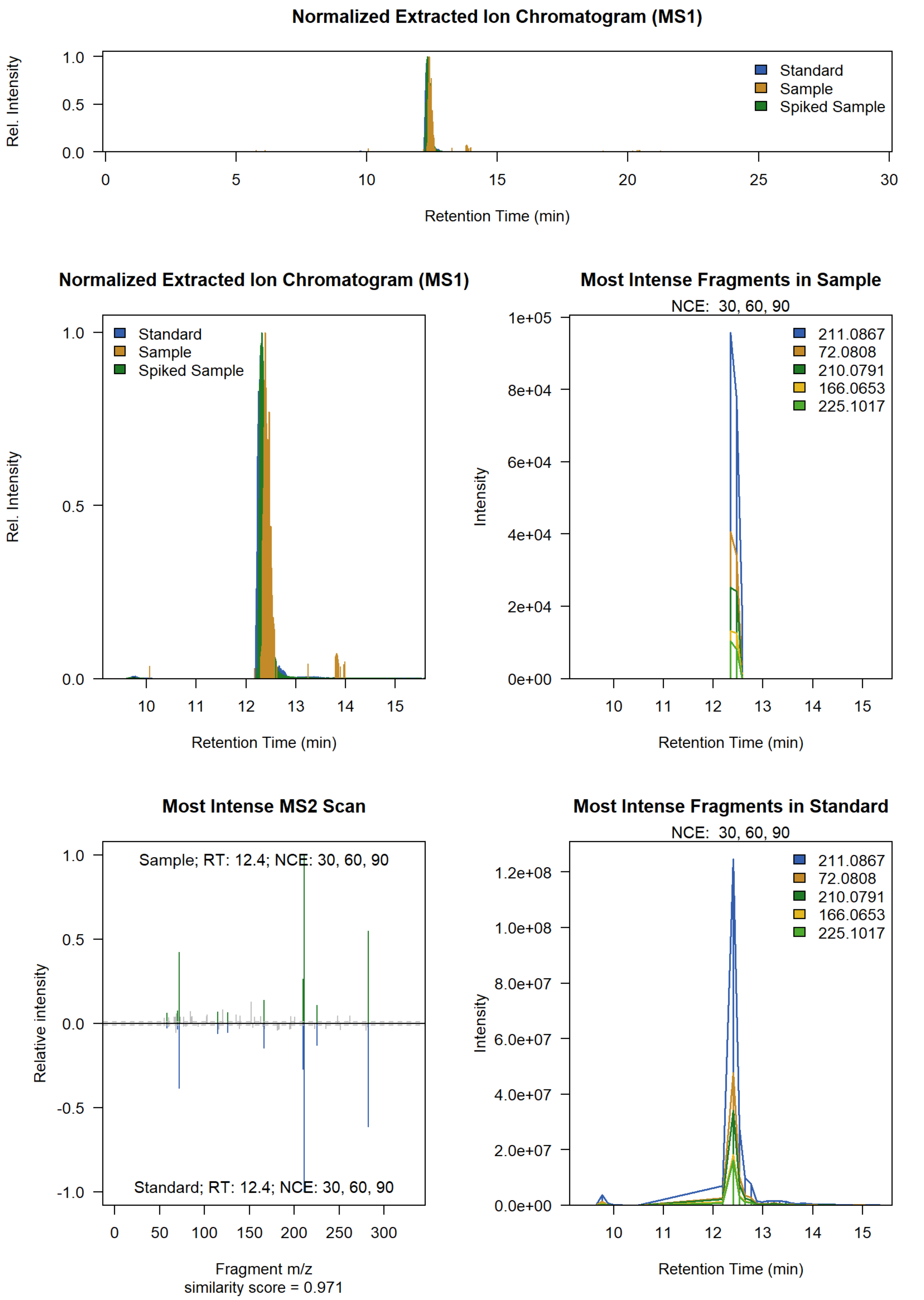

Figure SI-D331: Extracted ion chromatograms of 8-hydroxymirtazapine in the reference standard, the sample and the spiked sample, as well as MS2 head to tail plot and most intense MS2 fragments in standard and sample.

SI-D2.14.9 14-Hydroxyclarithromycin

14-Hydroxyclarithromycin is metabolite of clarithromycin, a macrolide antibiotic used for the treatment of a wide variety of bacterial infections.[2] Figure SI-D332 shows the metabolism scheme and Figure SI-D333 the clarithromycin cluster.

Table SI-D163: Information on identifiers, chemical properties, detection and confidence of identification of phenylethylmalonamide.

IUPAC Name	(3*R*,4*S*,5*S*,6*R*,7*R*,9*R*,11*R*,12*R*,13*S*,14*R*)-6-[(2*S*,3*R*,4*S*,6*R*)-4-(dimethylamino)-3-hydroxy-6-methyloxan-2-yl]oxy-12,13-dihydroxy-14-(1-hydroxyethyl)-4-[(2*R*,4*R*,5*S*,6*S*)-5-hydroxy-4-methoxy-4,6-dimethyloxan-2-yl]oxy-7-methoxy-3,5,7,9,11,13-hexamethyl-oxacyclotetradecane-2,10-dione
Molecular formula	$C_{38}H_{69}NO_{14}$
Monoisotopic mass [g/mol]	763.4718
Adduct	$[M+H]^+$
Retention time [min]	16.6
SMILES	C[C@@H]1C[C@@H]([C@H]([C@@H](O1)O[C@@H]2[C@H]([C@@H]([C@H](C(=O)O[C@@H]([C@@]([C@@H]([C@H](C(=O)[C@@H](C[C@@]2(C)OC)C)C)O)(C)O)C(C)O)C)O[C@H]3C[C@@]([C@H]([C@@H](O3)C)O)(C)OC)C)O)N(C)C
InChI	InChI=1S/C38H69NO14/c1-18-16-37(9,48-14)32(53-35-28(42)25(39(11)12)15-19(2)49-35)21(4)29(51-26-17-36(8,47-13)31(44)24(7)50-26)22(5)34(45)52-33(23(6)40)38(10,46)30(43)20(3)27(18)41/h18-26,28-33,35,40,42-44,46H,15-17H2,1-14H3/t18-,19-,20+,21+,22-,23?,24+,25+,26+,28-,29+,30-,31+,32-,33-,35+,36-,37-,38+/m1/s1
InChI-Key	BLPFDXNVUDZBII-KNPZYKNQSA-N
CAS RN	110671-78-8
Metabolite of	Clarithromycin
Detection frequency	20% (3/15 samples)
Detected in	Neugut, Monday
	Werdhölzli, Tuesday, Thursday
Intensity	E6-E7
Initial confidence level	level 3
Initial confidence score	0.39
Final confidence level	level 2b

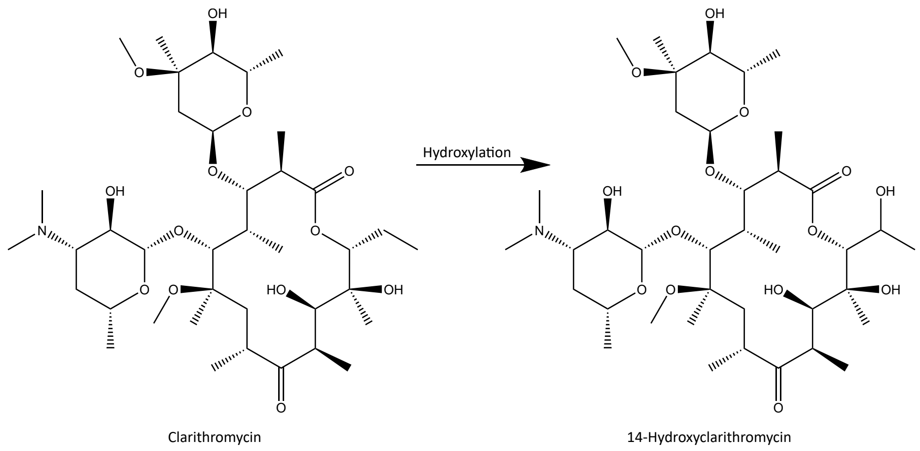

Figure SI-D332: Metabolism of clarithromycin to 14-hydroxyclarithromycin.

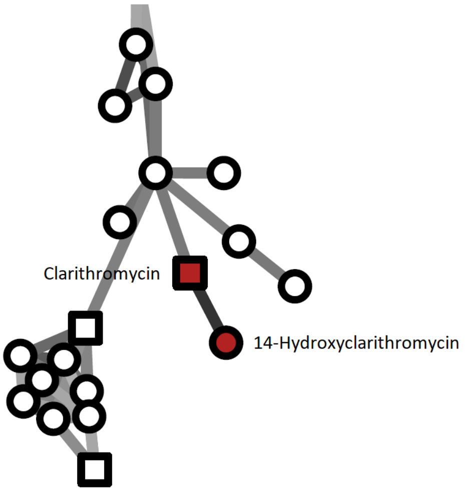

Figure SI-D333: Excerpt of the molecular network showing the clarithromycin cluster.

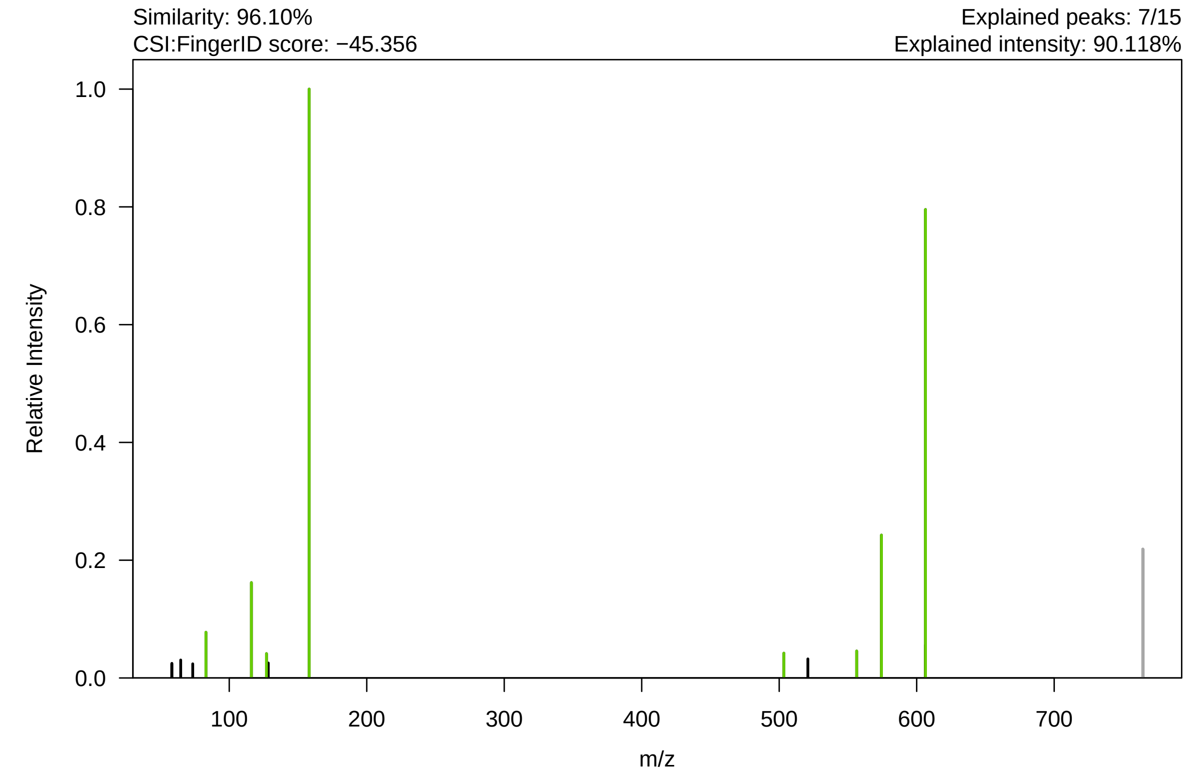

Figure SI-D334: Measured MS2 spectrum. Matching fragments with 14-hydroxyclarithromycin predicted by SIRIUS/CSI:FingerID are highlighted in green. The molecular ion in gray is not considered.

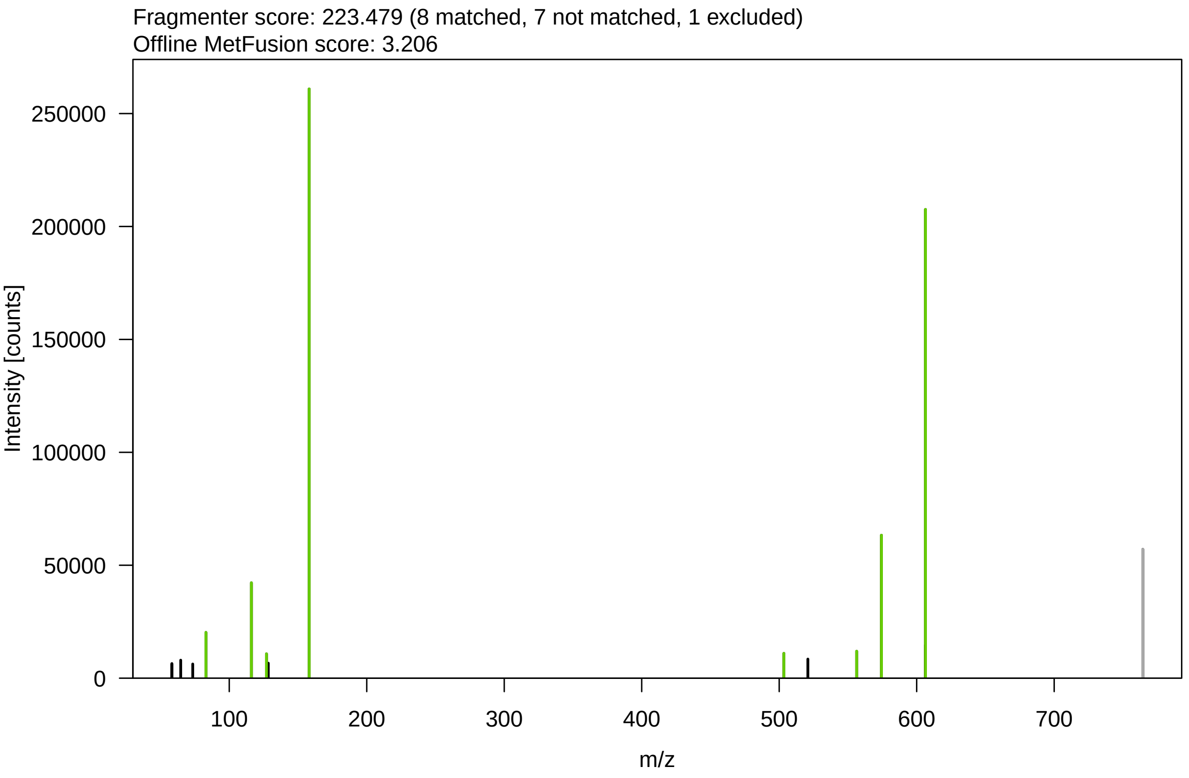

Figure SI-D335: Measured MS2 spectrum. Matching fragments with 14-hydroxyclarithromycin predicted by MetFrag are highlighted in green. The molecular ion in gray is not considered.

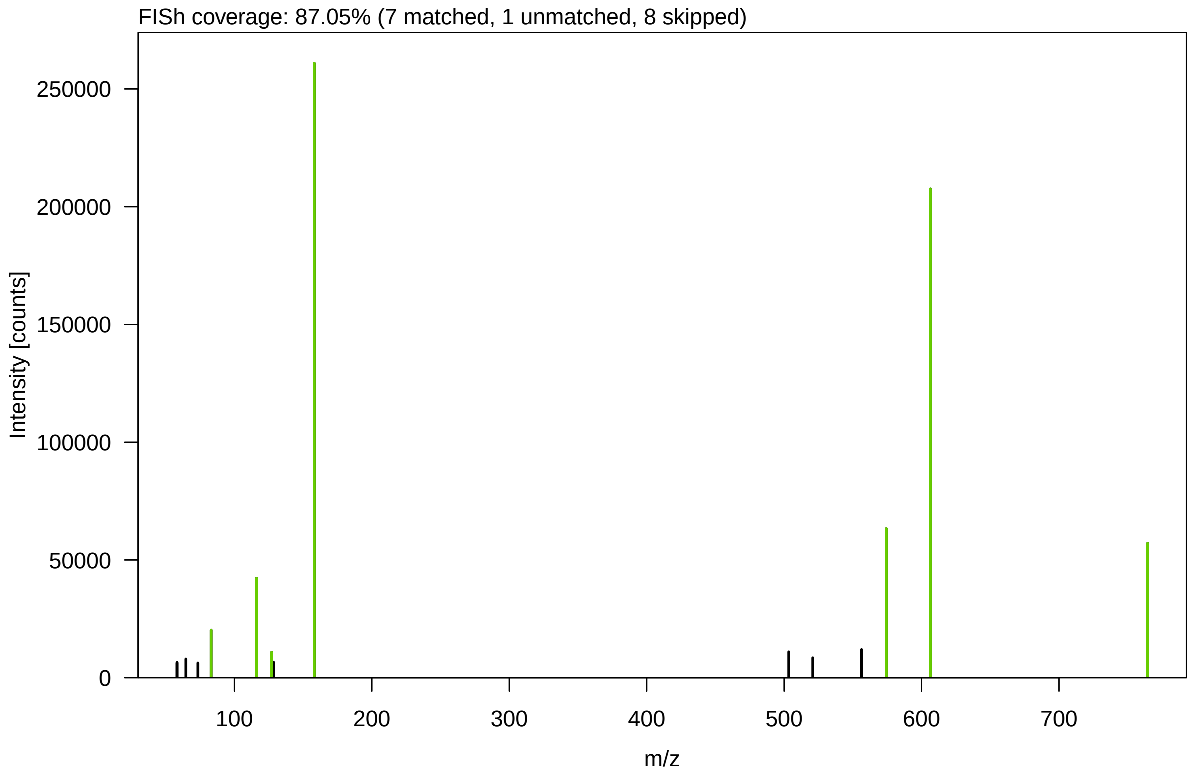

Figure SI-D336: Measured MS2 spectrum. Matching fragments with 14-hydroxyclarithromycin predicted by FISh Scoring are highlighted in green. Low intensity fragments are not considered and skipped.

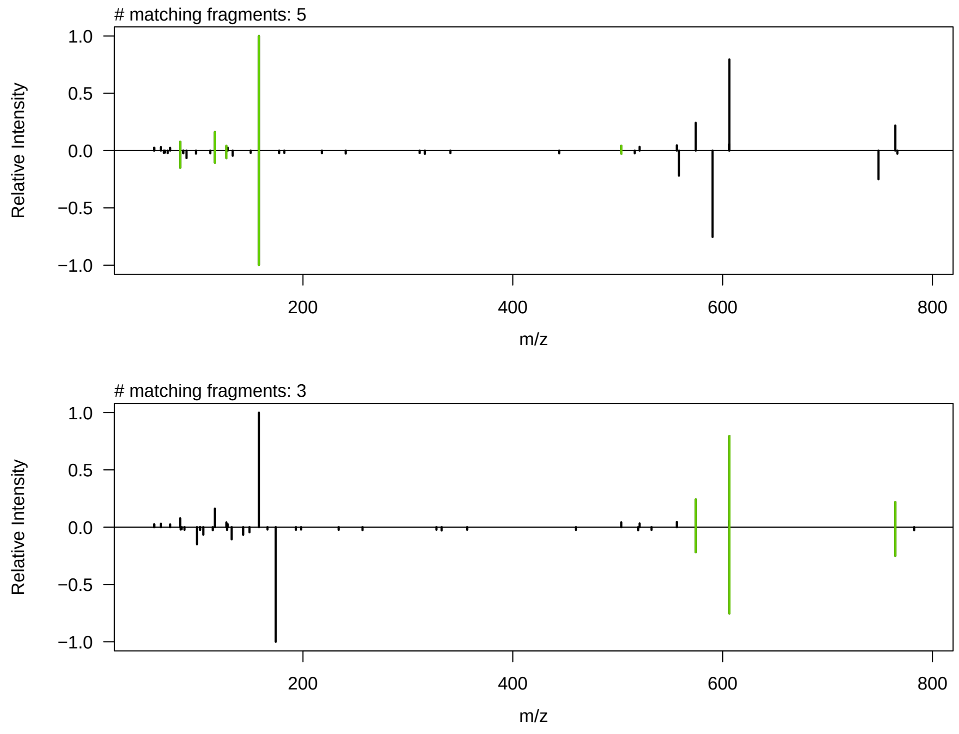

Figure SI-D337: Head to tail plots of 14-hydroxyclarithromycin and clarithromycin. In the bottom plot, the mass spectrum of clarithromycin is shifted by the mass difference. Matching fragments are highlighted in green.

Table SI-D164: Molecular network results and retention time prediction of 14-hydroxyclarithromycin.

Comparison with	Clarithromycin
MSn Score	80
Forward coverage	72
Reverse coverage	88
Forward match	13
Reverse match	7
ΔMass [g/mol]	15.9949
Measured retention time [min]	16.6
Predicted $logD_{OW}$ (pH = 2.7)	-1.41
Predicted retention time [min]	12.9
Predicted retention time range (95% confidence interval) [min]	8.3-17.5
Predicted retention time range (99% confidence interval) [min]	6.8-19.0

Table SI-D165: Annotated MS2 spectrum of 14-hydroxyclarithromycin.

m/z	Relative Intensity	Annotation
58.2006	24.08	
58.2848	24.79	
64.6932	30.52	
73.4462	24.01	
83.0489	77.56	$C_5H_6O + H^+$
116.1073	161.78	$C_6H_{13}NO + H^+$
127.0754	41.29	$C_7H_{10}O_2 + H^+$
128.3743	25.48	
158.1175	999.00	$C_8H_{15}NO_2 + H^+$
503.3844	42.07	$C_{27}H_{52}NO_7 + H^+$
520.8588	32.37	
556.3482	45.73	$C_{29}H_{49}NO_9 + H^+$
574.3592	242.41	$C_{29}H_{51}NO_{10} + H^+$
606.2904	55.75	
606.3840	794.74	$C_{30}H_{55}NO_{11} + H^+$
764.4782	218.48	$C_{38}H_{69}NO_{14} + H^+$

The human liver S9 incubation of clarithromycin led to the formation of a hydroxylated clarithromycin metabolite. Considering the spectral match of 0.845 (see Figure SI-D338) and the retention times of 16.6 and 16.7 minutes in the wastewater and the human liver S9 sample, respectively, further confidence could be gained that the detected feature in wastewater is 14-hydroxyclarithromycin. Due to this diagnostic evidence, the final confidence level can be increased from level 3 to level 2b. Several other positions for hydroxylations are conceivable. However, the only hydroxylated calrithromycin compound formed in considerable amount in the human body is 14-hydroxyclarithromycin.[19]

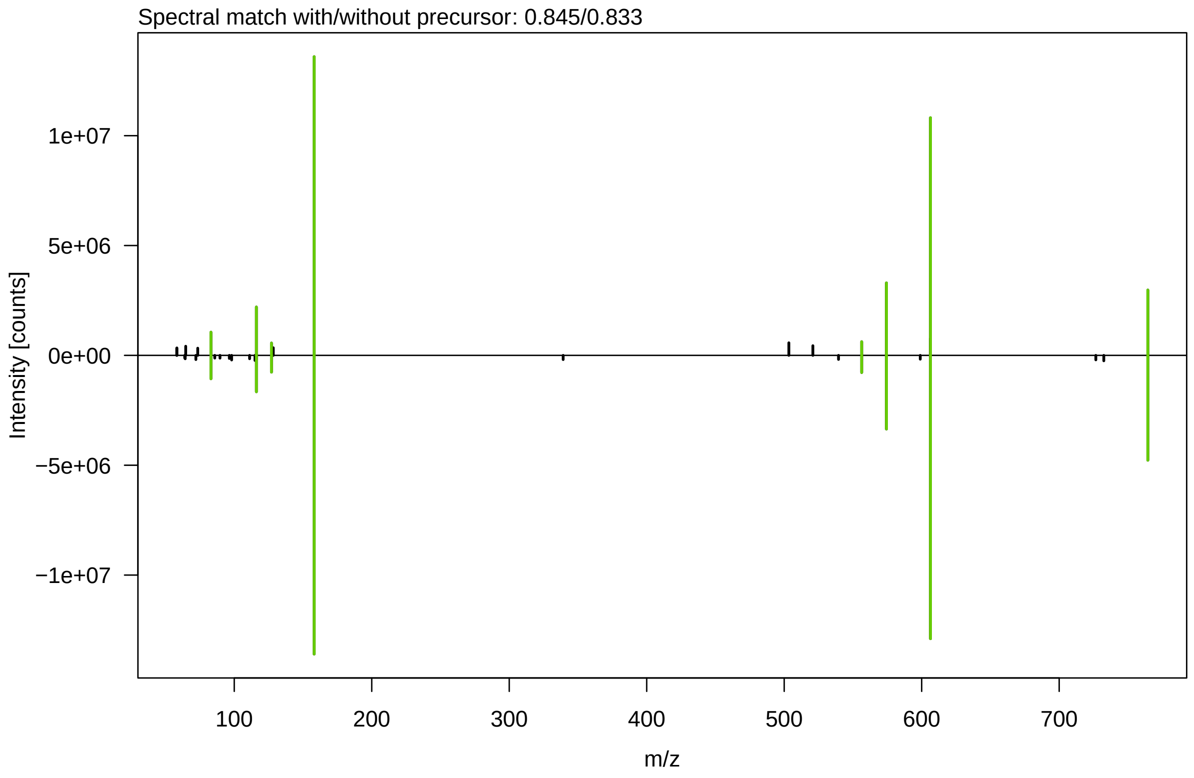

Figure SI-D338: Head to tail plot of 14-hydroxyclarithromycin in wastewater (top) and from human liver S9 incubation (bottom). Matching fragments are highlighted in green.

SI-D2.14.10 α-Hydroxymetoprolol

α-Hydroxymetoprolol is a metabolite of metoprolol, a beta-blocker used in the treatment of hypertension and angina.[2] Figure SI-D339 shows the metabolism scheme and Figure SI-D340 the metoprolol cluster.

Table SI-D166: Information on identifiers, chemical properties, detection and confidence of identification of α-hydroxymetoprolol.

IUPAC Name	1-[4-(1-hydroxy-2-methoxyethyl)phenoxy]-3-(propan-2-ylamino)propan-2-ol
Molecular formula	$C_{15}H_{25}NO_4$
Monoisotopic mass [g/mol]	283.1784
Adduct	$[M+H]^+$
Retention time [min]	10.8
SMILES	CC(C)NCC(COC1=CC=C(C=C1)C(COC)O)O
InChI	InChI=1S/C15H25NO4/c1-11(2)16-8-13(17)9-20-14-6-4-12(5-7-14)15(18)10-19-3/h4-7,11,13,15-18H,8-10H2,1-3H3
InChI-Key	OFRYBPCSEMMZHR-UHFFFAOYSA-N
CAS RN	56392-16-6
Metabolite of	Metoprolol
Detection frequency	100% (15/15 samples)
Detected in	Altenrhein, Monday-Friday
	Neugut, Monday-Friday
	Werdhölzli, Monday-Friday
Intensity	E7-E8
Initial confidence level	level 3
Initial confidence score	0.48
Final confidence level	level 3

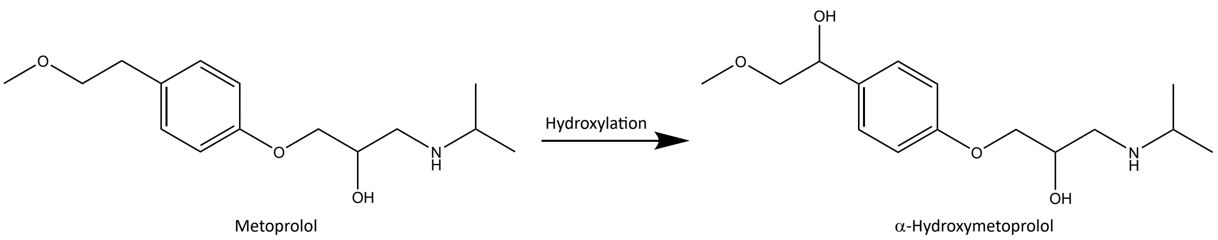

Figure SI-D339: Metabolism of metoprolol to α-hydroxymetoprolol.

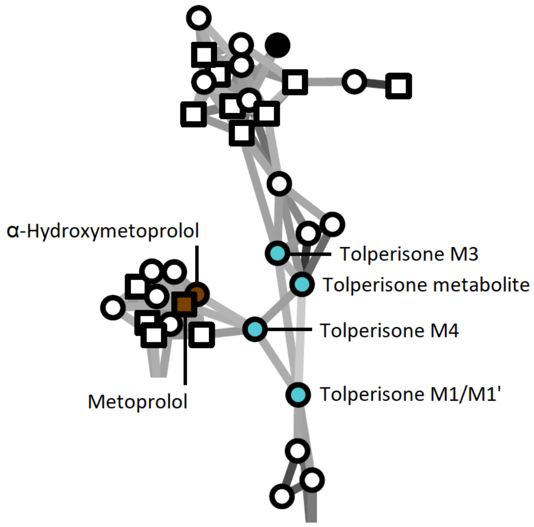

Figure SI-D340: Excerpt of the molecular network showing the metoprolol cluster.

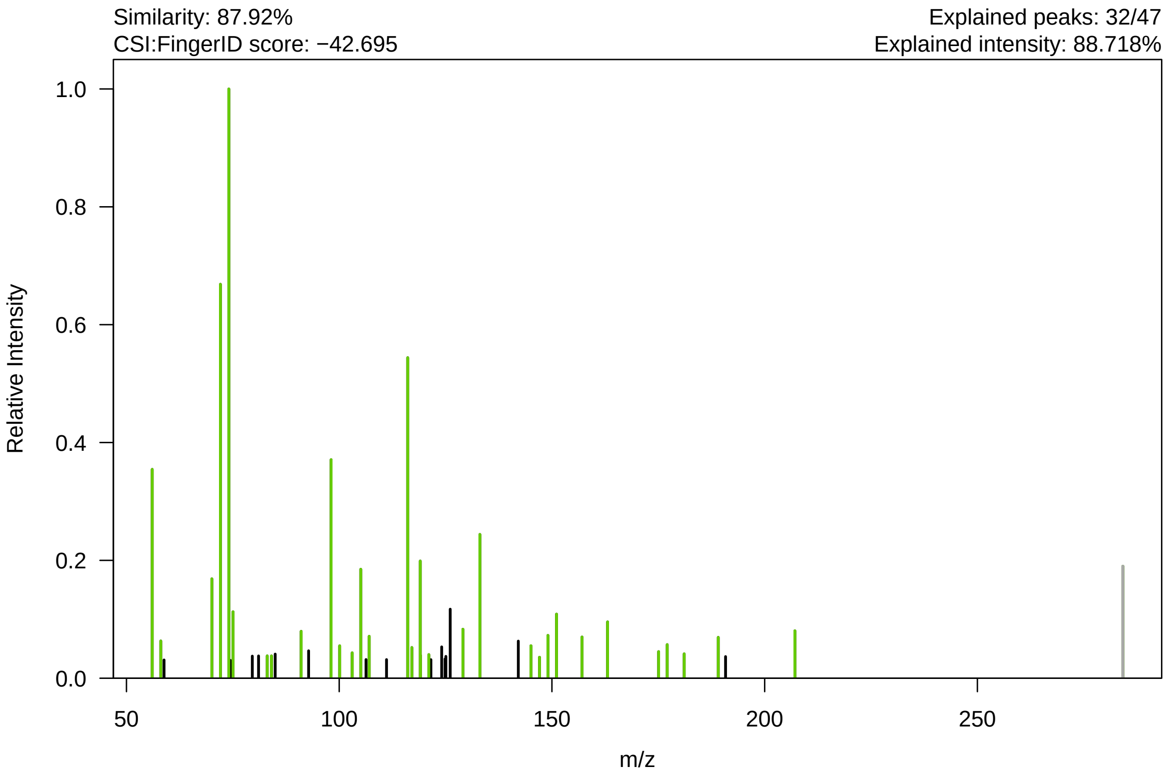

Figure SI-D341: Measured MS2 spectrum. Matching fragments with α-hydroxymetoprolol predicted by SIRIUS/CSI:FingerID are highlighted in green. The molecular ion in gray is not considered.

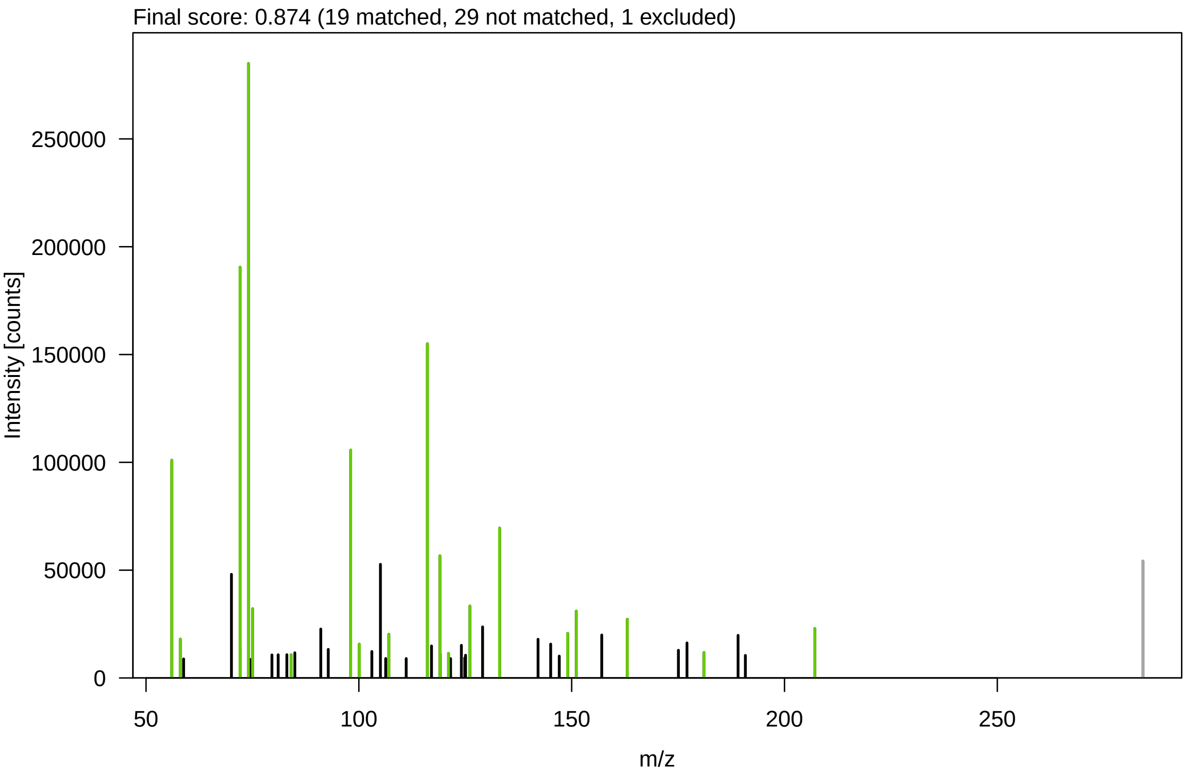

Figure SI-D342: Measured MS2 spectrum. Matching fragments with α-hydroxymetoprolol predicted by MetFrag are highlighted in green. The molecular ion in gray is not considered.

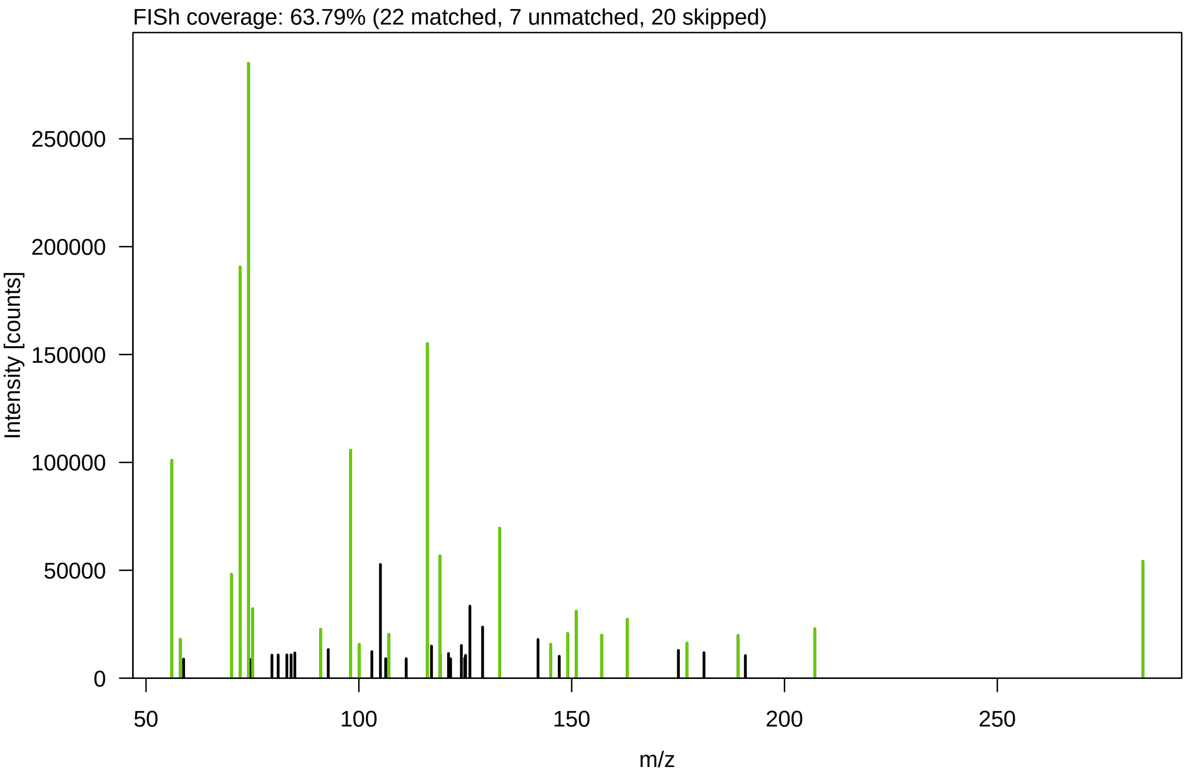

Figure SI-D343: Measured MS2 spectrum. Matching fragments with α-hydroxymetoprolol predicted by FISh Scoring are highlighted in green. Low intensity fragments are not considered and skipped.

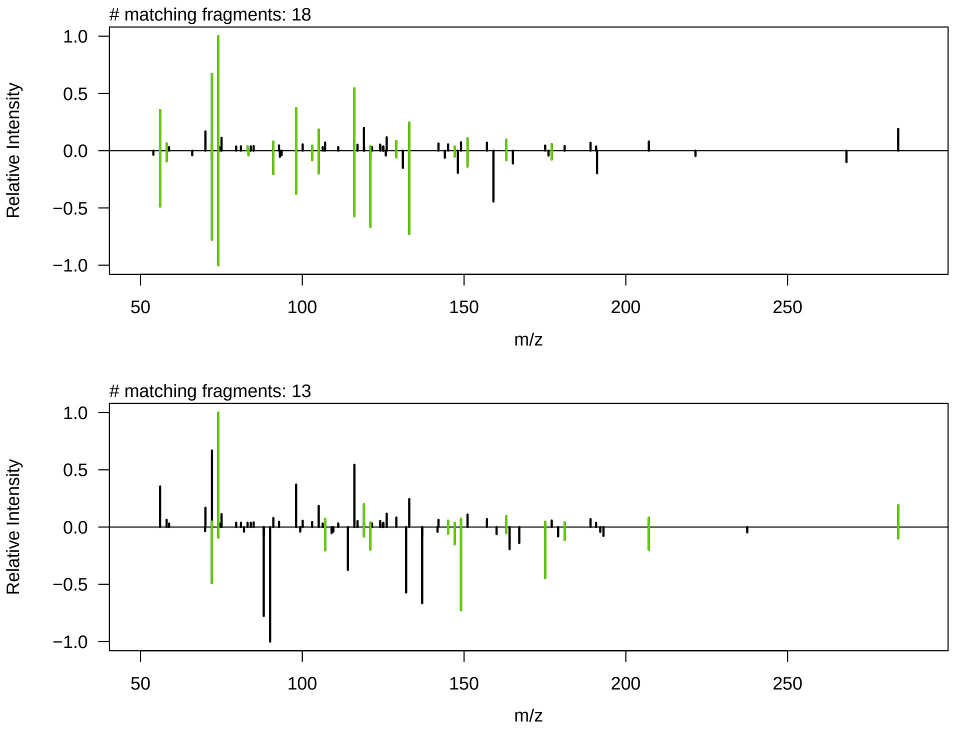

Figure SI-D344: Head to tail plots of α-hydroxymetoprolol and metoprolol. In the bottom plot, the mass spectrum of metoprolol is shifted by the mass difference. Matching fragments are highlighted in green.

Table SI-D167: Molecular network results and retention time prediction of α-hydroxymetoprolol.

Comparison with	Metoprolol
MSn Score	57
Forward coverage	61
Reverse coverage	53
Forward match	27
Reverse match	31
ΔMass [g/mol]	15.9949
Measured retention time [min]	10.8
Predicted $\log D_{OW}$ (pH = 2.7)	-2.40
Predicted retention time [min]	11.6
Predicted retention time range (95% confidence interval) [min]	7.0-16.2
Predicted retention time range (99% confidence interval) [min]	5.5-17.7

Table SI-D168: Annotated MS2 spectrum of α-hydroxymetoprolol.

m/z	Relative Intensity	Annotation
56.0498	354.06	$C_3H_5N + H^+$
58.0654	63.16	$C_3H_7N + H^+$
58.8304	30.94	
70.0652	168.53	$C_4H_7N + H^+$
72.0772	49.01	
72.0808	667.87	$C_4H_9N + H^+$
74.0600	999.00	$C_3H_7NO + H^+$
74.4109	30.26	
75.0440	112.66	$C_3H_6O_2 + H^+$
79.5768	37.46	
81.0445	37.77	
83.0851	37.84	$C_6H_{10} + H^+$
84.0801	37.81	$C_5H_9N + H^+$
84.9592	40.91	
91.0542	79.54	$C_7H_6 + H^+$
92.8086	46.51	
98.0965	370.48	$C_6H_{11}N + H^+$
100.1122	54.99	$C_6H_{13}N + H^+$
103.0540	43.08	$C_8H_6 + H^+$
105.0698	184.74	$C_8H_8 + H^+$
106.3058	31.67	
107.0492	71.14	$C_7H_6O + H^+$
111.1208	31.59	
116.1069	543.44	$C_6H_{13}NO + H^+$
117.0698	52.04	$C_9H_8 + H^+$
119.0493	198.70	$C_8H_6O + H^+$
119.0858	38.66	$C_9H_{10} + H^+$
121.0646	40.04	$C_8H_8O + H^+$
121.5508	31.47	
124.0864	53.11	
124.8824	32.71	
125.0712	37.04	
126.0909	117.05	$C_7H_{11}NOH$≡;
129.0697	83.06	$C_{10}H_8 + H^+$
133.0645	243.74	$C_9H_8O + H^+$
142.0977	62.93	

Continued on next page

Table SI-D168: Annotated MS2 spectrum of α-hydroxymetoprolol.(Continued)

145.0647	55.07	$C_{10}H_8O + H^+$
147.0802	35.55	$C_{10}H_{10}O + H^+$
149.0592	72.55	$C_9H_8O_2 + H^+$
151.0753	108.69	$C_9H_{10}O_2 + H^+$
157.0643	69.86	$C_{11}H_8O + H^+$
163.0750	95.48	$C_{10}H_{10}O_2 + H^+$
175.0748	45.08	$C_{11}H_{10}O_2 + H^+$
177.0913	56.98	$C_{11}H_{12}O_2$
181.0867	41.33	$C_{10}H_{12}O_3 + H^+$
189.0908	69.28	$C_{12}H_{12}O_2 + H^+$
190.8073	36.68	
207.1012	80.40	$C_{12}H_{14}O_3 + H^+$
284.1857	189.93	$C_{15}H_{25}NO_4 + H^+$

A reference standard of α-hydroxymetoprolol was not purchased and no human liver S9 incubation experiment was conducted. Correspondingly, the identification confidence remains at level 3.

SI-D2.14.11 Benzophenone

Benzophenone is a metabolite of cinnarizine, which is an anti-histaminic drug used for the control of vestibular disorders and motion sickness. It treats the symptoms vertigo, tinnitus, nystagmus, nausea and vomiting. Moreoever, benzophenone is contained in many every-day life products. It is an additive in plastics, coatings and adhesive formualtions, is a flvouring agent, fragrance anhancer and perfume fixative and prevents other product ingredients from degradation by UV light.[20] Figure SI-D345 shows the metabolism of cinnarizine to benzophenone.

Table SI-D169: Information on identifiers, chemical properties, detection and confidence of identification of benzophenone.

IUPAC Name	diphenylmethanone
Molecular formula	$C_{13}H_{10}O$
Monoisotopic mass [g/mol]	182.0732
Adduct	$[M+H]^+$
Retention time [min]	20.5
SMILES	C1=CC=C(C=C1)C(=O)C2=CC=CC=C2
InChI	InChI=1S/C13H10O/c14-13(11-7-3-1-4-8-11)12-9-5-2-6-10-12/h1-10H
InChI-Key	RWCCWEUUXYIKHB-UHFFFAOYSA-N
CAS RN	119-61-9
Metabolite of	Cinnarizine
Detection frequency	100% (15/15 samples)
Detected in	Altenrhein, Monday-Friday
	Neugut, Monday-Friday
	Werdhölzli, Monday-Friday
Intensity	E6-E7
Initial confidence level	level 2a
Initial confidence score	0.43
Final confidence level	level 1

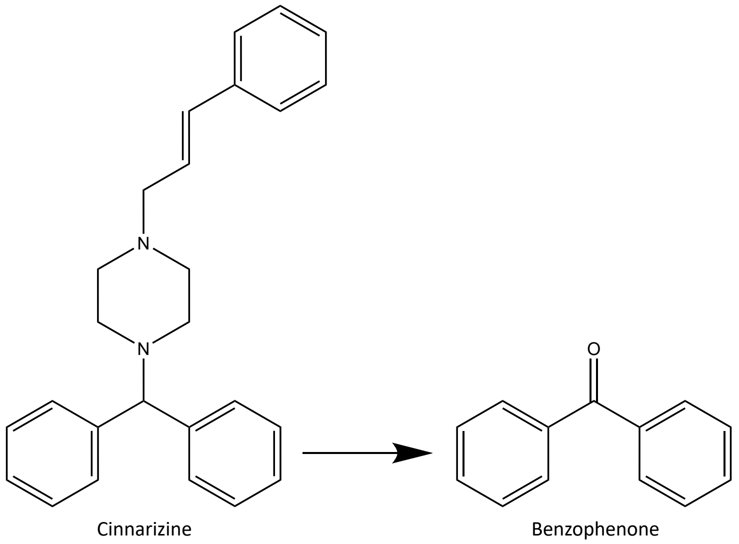

Figure SI-D345: Metabolism of cinnarizine to benzophenone.

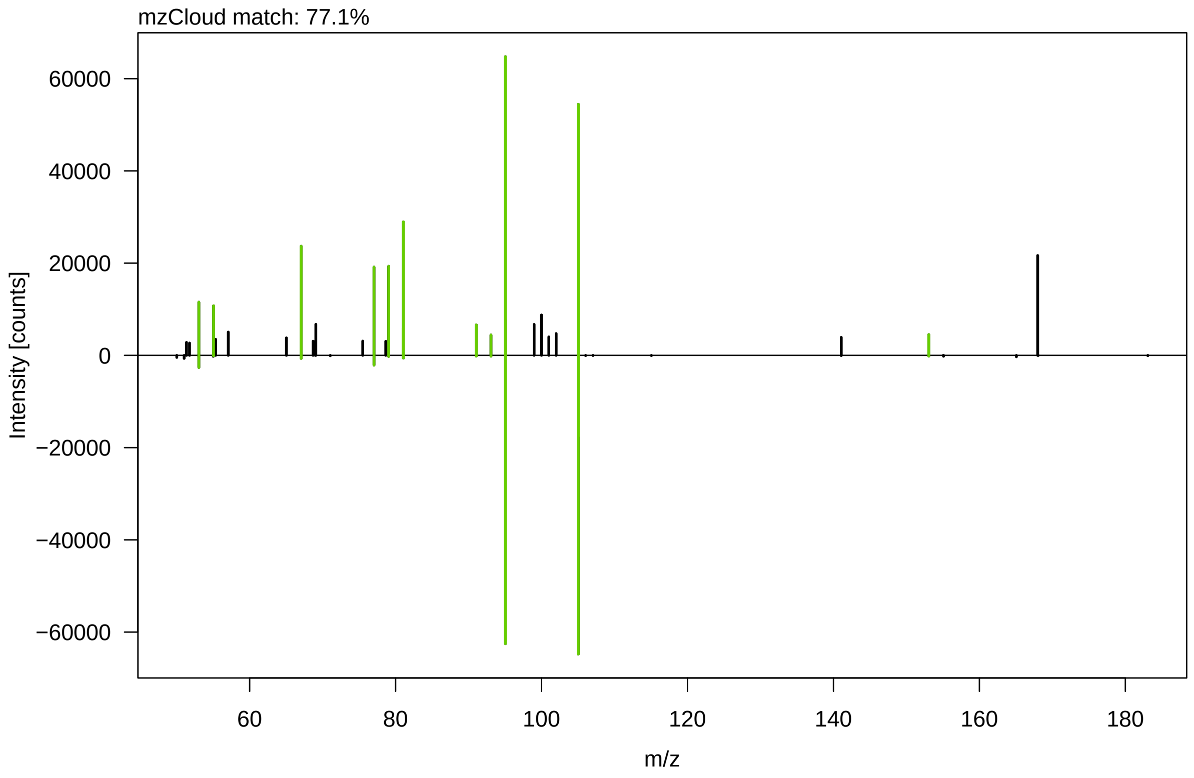

Figure SI-D346: Head to tail plot of measured MS2 spectrum against mzCloud library spectrum of benzophenone. Matching fragments are highlighted in green.

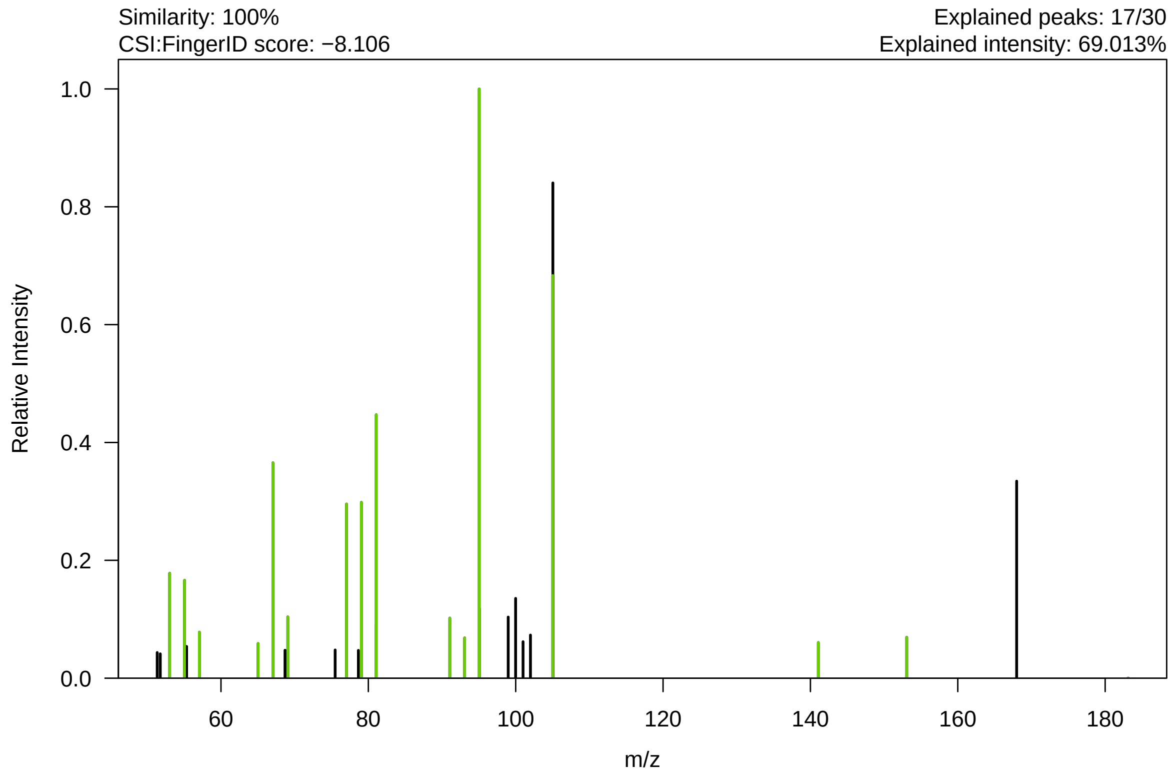

Figure SI-D347: Measured MS2 spectrum. Matching fragments with benzophenone predicted by SIRIUS/CSI:FingerID are highlighted in green.

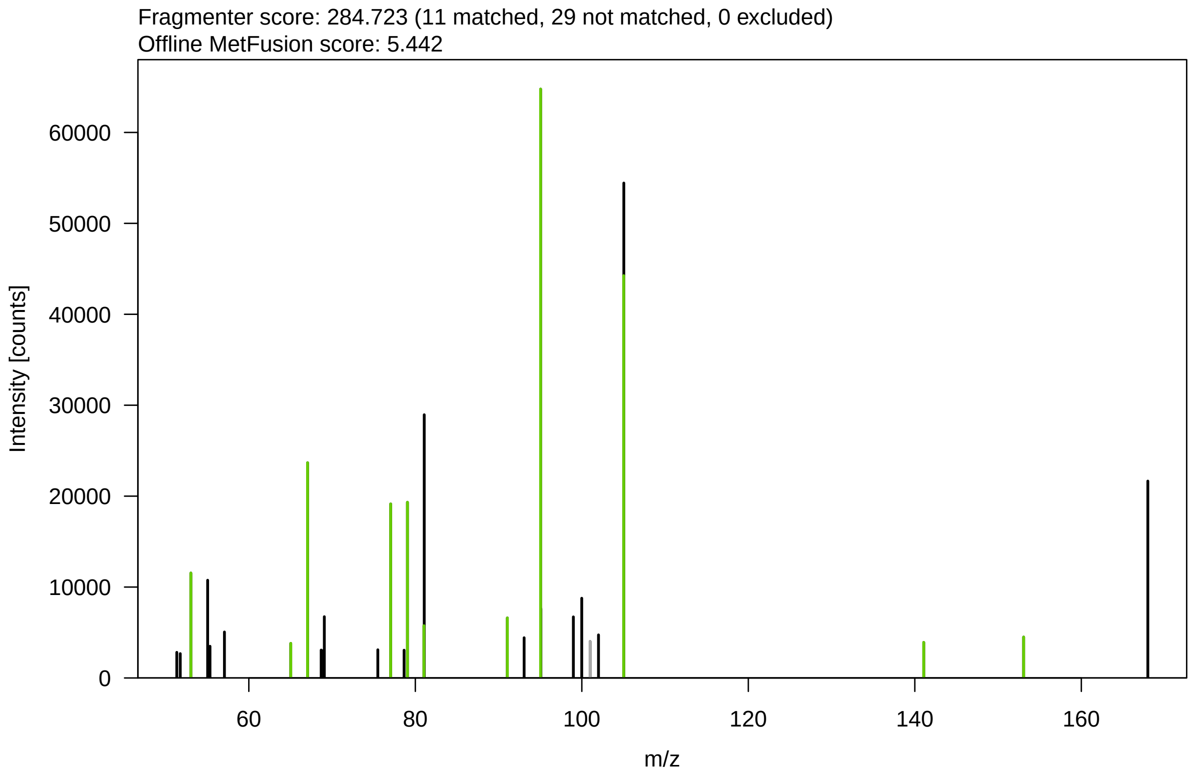

Figure SI-D348: Measured MS2 spectrum. Matching fragments with benzophenone predicted by MetFrag are highlighted in green.

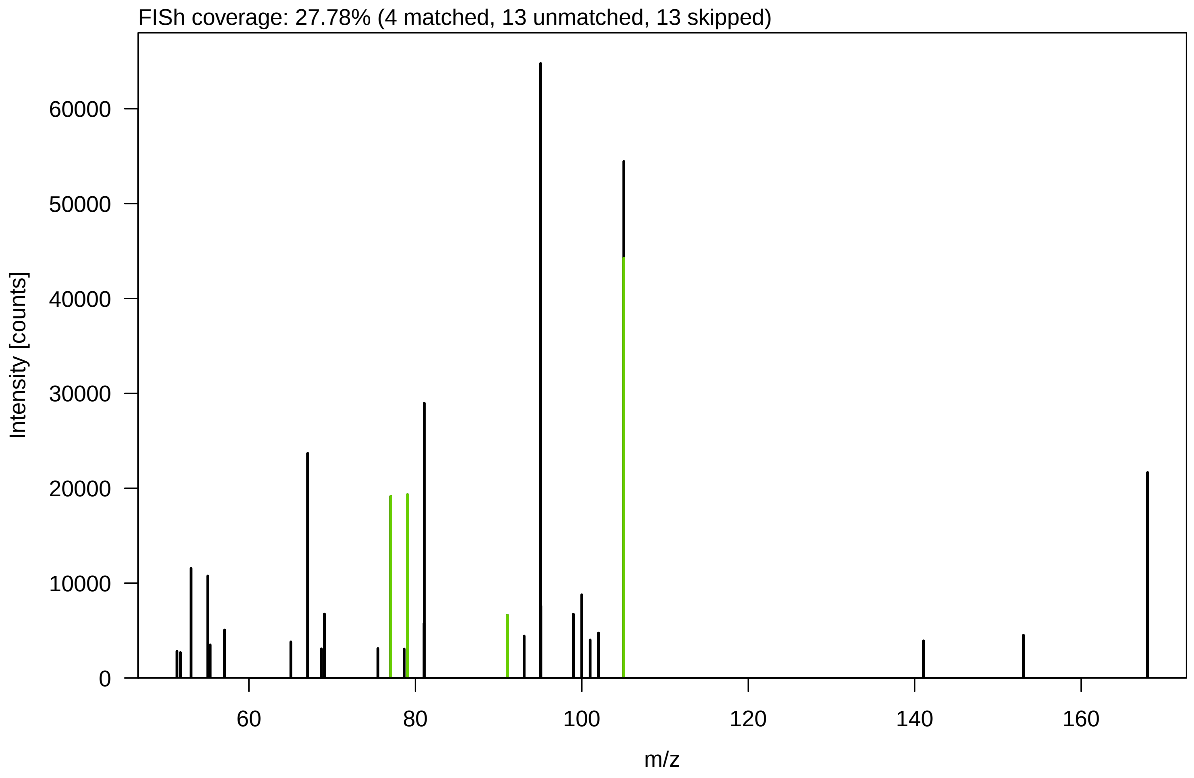

Figure SI-D349: Measured MS2 spectrum. Matching fragments with benzophenone predicted by FISh Scoring are highlighted in green. Low intensity fragments are not considered and skipped.

Table SI-D170: Retention time prediction of benzophenone.

Measured retention time [min]	20.5
Predicted $logD_{OW}$ (pH = 2.7)	3.43
Predicted retention time [min]	19.2
Predicted retention time range (95% confidence interval) [min]	14.6-23.8
Predicted retention time range (99% confidence interval) [min]	13.1-25.3

Table SI-D171: Annotated MS2 spectrum of benzophenone.

m/z	Relative Intensity	Annotation
51.3466	43.50	
51.7633	41.29	
53.0389	178.03	$C_4H_4 + H^+$
55.0545	165.84	$C_4H_6 + H^+$
55.3358	53.80	
57.0700	78.00	$C_4H_8 + H^+$
65.0388	58.71	$C_5H_4 + H^+$
67.0545	365.16	$C_5H_6 + H^+$
68.6935	47.38	
68.9971	47.23	
69.0698	103.95	$C_5H_8 + H^+$
75.4961	47.83	
77.0385	295.26	$C_6H_4 + H^+$
78.6506	47.12	
79.0543	298.09	$C_6H_6 + H^+$
81.0335	88.81	$C_5H_4O + H^+$
81.0699	446.57	$C_6H_8 + H^+$
91.0542	101.88	$C_7H_6 + H^+$
93.0697	68.25	$C_7H_8 + H^+$
95.0491	999.00	$C_6H_6O + H^+$
95.0855	117.47	$C_7H_{10} + H^+$
98.9839	103.64	
99.9905	135.26	
100.9966	61.85	
102.0028	73.08	
105.0336	682.64	$C_7H_4O + H^+$
105.0446	839.65	
141.0702	60.37	$C_{11}H_8 + H^+$
153.0703	69.41	$C_{12}H_8 + H^+$
167.9889	334.05	

A reference standard of benzophenone was purchased. Figure SI-D350 shows the extracted ion chromatograms of this standard, the sample and the spiked sample, as well as a head to tail plot of the MS2 spectra of the standard and the sample. In addition, the most intense MS2 fragments in the sample and in the standard are displayed. It becomes visible that the retention times of the sample and the spiked sample are identical and the spectra similarity score between sample and standard is equal to 0.394. This low score is a result of the low intensity signal of benzophenone, leading to a low qualtiy and noisy MS2 spectrum. Nonetheless, several MS2 fragments in the sample can be explained by the reference standard. It can therefore be concluded that the suspected compound is indeed benzophenone. Correspondingly, the identification confidence can be increased to level 1.

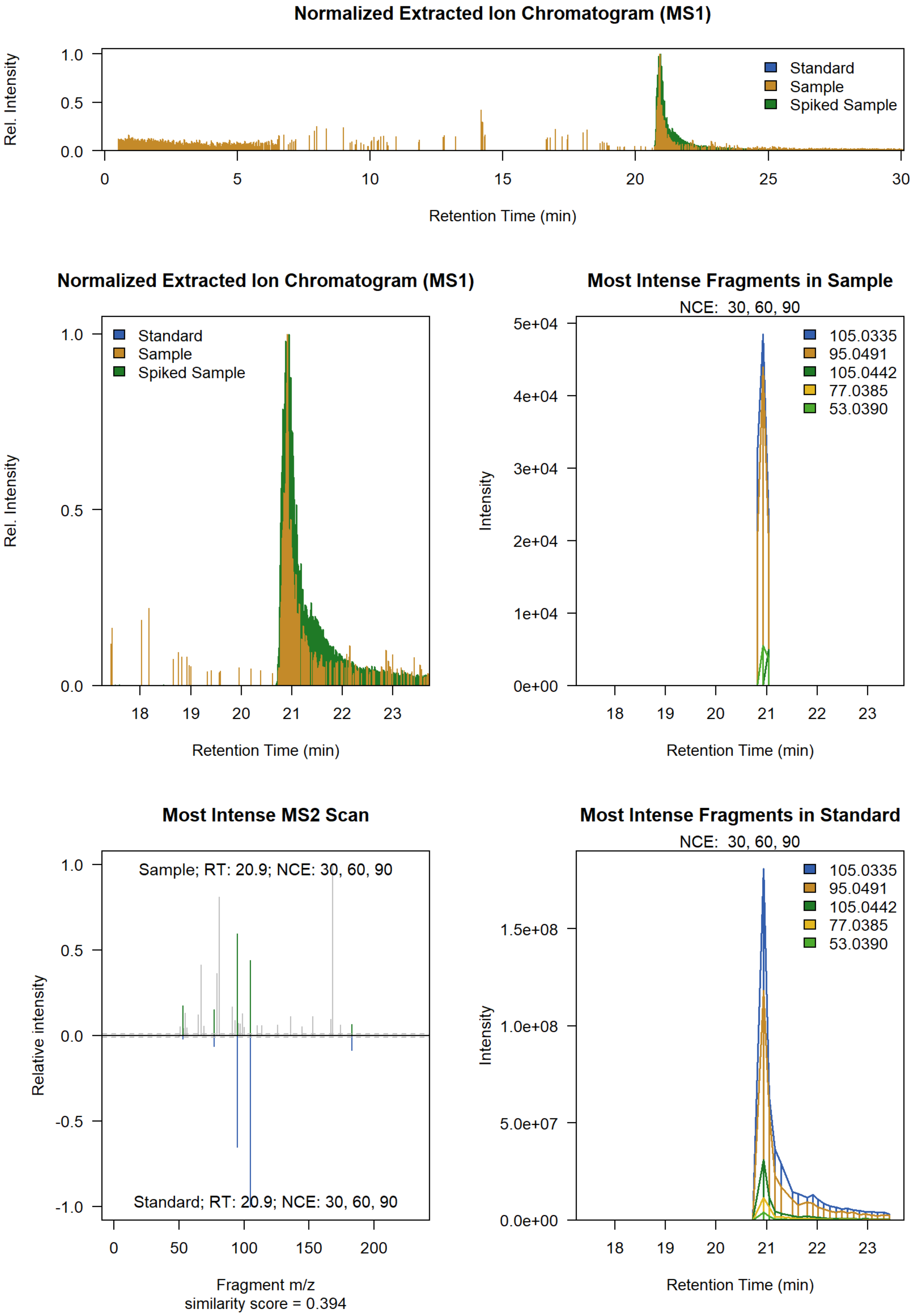

Figure SI-D350: Extracted ion chromatograms of benzophenone in the reference standard, the sample and the spiked sample, as well as MS2 head to tail plot and most intense MS2 fragments in standard and sample.

SI-D2.14.12 Clindamycin-Sulfoxide

Clindamycin-sulfoxide is a metabolite of clindamycin, a lincosamide antibiotic used to treat infections caused by susceptible anaerobic, streptococcal, staphylococcal, and pneumococcal bacteria.[2] Figure SI-D351 shows the metabolism scheme.

Table SI-D172: Information on identifiers, chemical properties, detection and confidence of identification of clindamycin sulfoxide.

IUPAC Name	*N*-[2-chloro-1-(3,4,5-trihydroxy-6-methylsulfinyloxan-2-yl)propyl]-1-methyl-4-propylpyrrolidine-2-carboxamide
Molecular formula	$C_{18}H_{33}ClN_2O_6S$
Monoisotopic mass [g/mol]	440.1748
Adduct	$[M+H]^+$
Retention time [min]	13.9
SMILES	CCCC1CC(N(C1)C)C(=O)NC(C2C(C(C(C(O2)S(=O)C)O)O)O)C(C)Cl
InChI	InChI=1S/C18H33ClN2O6S/c1-5-6-10-7-11(21(3)8-10)17(25)20-12(9(2)19)16-14(23)13(22)15(24)18(27-16)28(4)26/h9-16,18,22-24H,5-8H2,1-4H3,(H,20,25)
InChI-Key	XSLGFIQRVCXUEU-UHFFFAOYSA-N
CAS RN	22431-46-5
Metabolite of	Clindamycin
Detection frequency	100% (15/15 samples)
Detected in	Altenrhein, Monday-Friday
	Neugut, Monday-Friday
	Werdhölzli, Monday-Friday
Intensity	E6-E7
Initial confidence level	level 3
Initial confidence score	0.21
Final confidence level	level 1

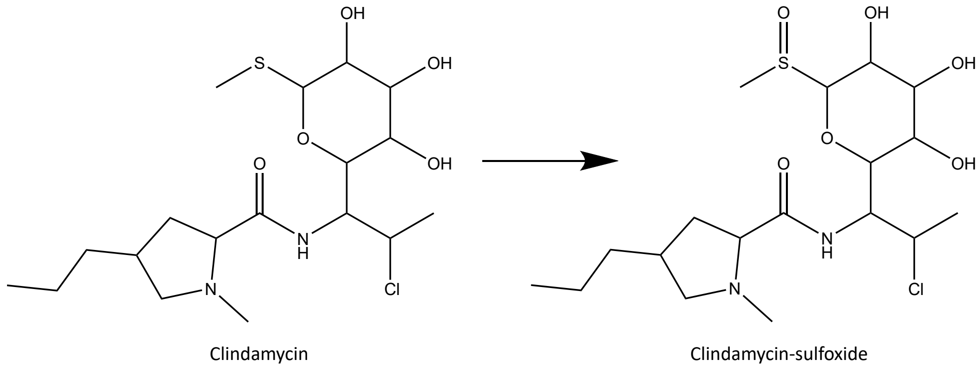

Figure SI-D351: Metabolism of clindamycin to clindamycin-sulfoxide.

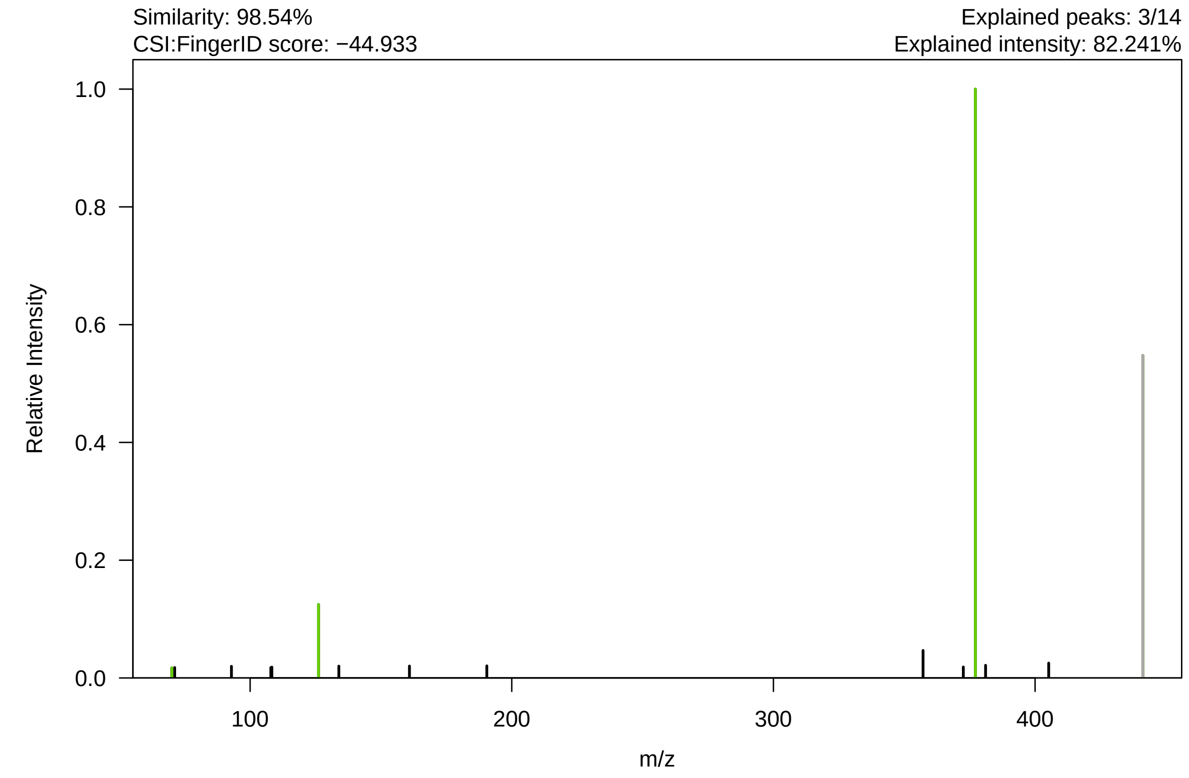

Figure SI-D352: Measured MS2 spectrum. Matching fragments with clindamycin-sulfoxide predicted by SIRIUS/CSI:FingerID are highlighted in green. The molecular ion in gray is not considered.

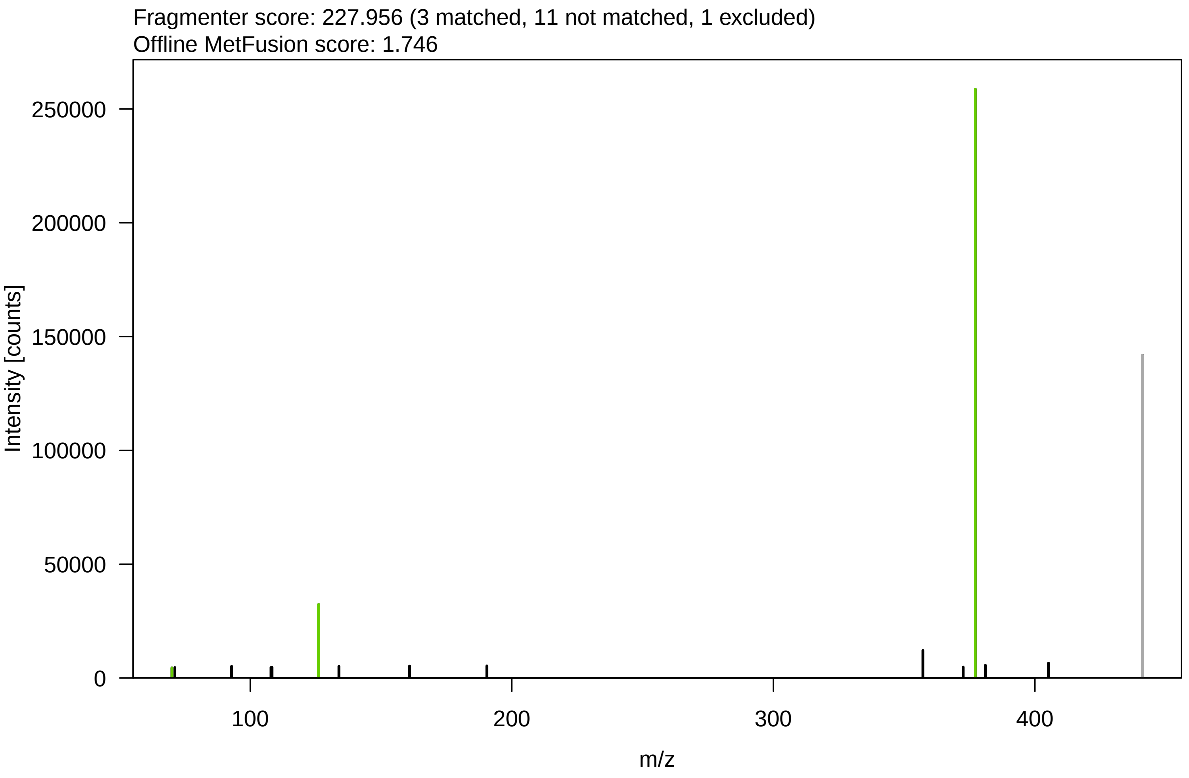

Figure SI-D353: Measured MS2 spectrum. Matching fragments with clindamycin-sulfoxide predicted by MetFrag are highlighted in green. The molecular ion in gray is not considered.

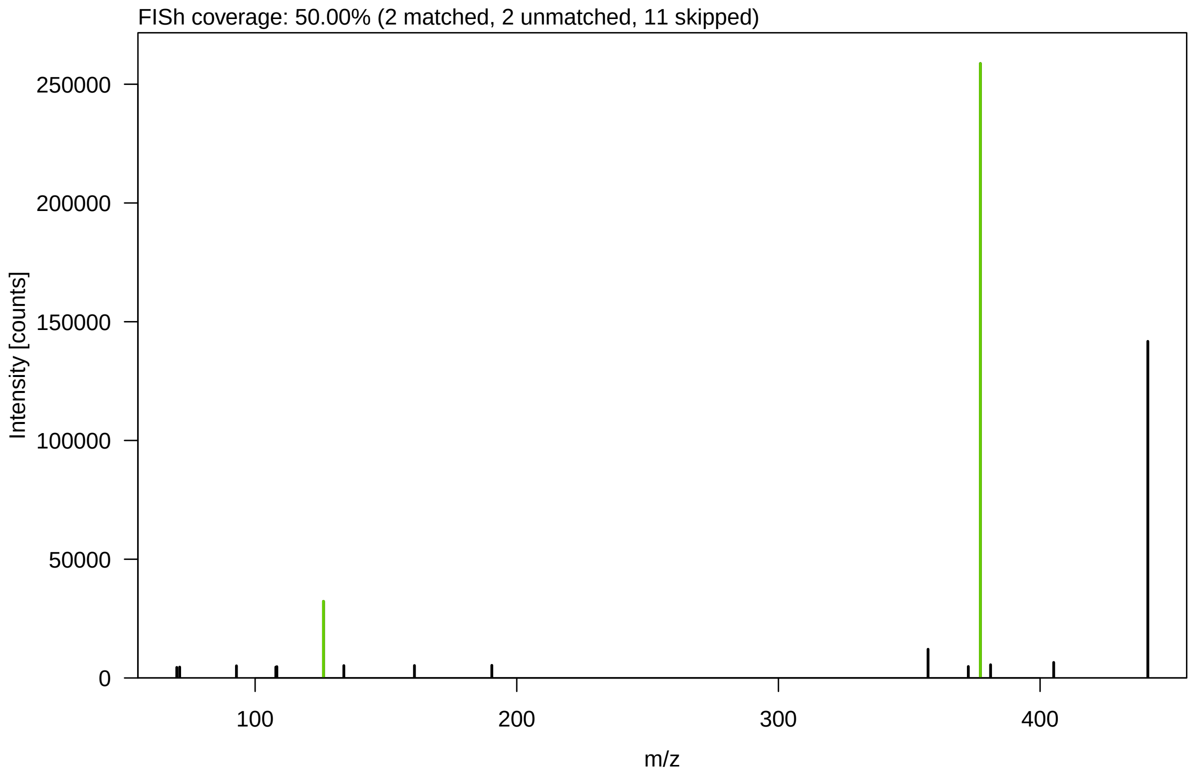

Figure SI-D354: Measured MS2 spectrum. Matching fragments with clindamycin-sulfoxide predicted by FISh Scoring are highlighted in green. Low intensity fragments are not considered and skipped.

Table SI-D173: Retention time prediction of clindamycin-sulfoxide.

Measured retention time [min]	13.9
Predicted $\log D_{OW}$ (pH = 2.7)	-4.46
Predicted retention time [min]	8.9
Predicted retention time range (95% confidence interval) [min]	4.3-13.6
Predicted retention time range (99% confidence interval) [min]	2.8-15.0

Table SI-D174: Annotated MS2 spectrum of clindamycin-sulfoxide.

m/z	Relative Intensity	Annotation
70.0639	17.15	$C_4H_7N + H^+$
71.1709	17.66	
92.8678	19.74	
107.9142	17.71	
108.3098	18.38	
126.1272	124.63	$C_8H_{15}N + H^+$
133.9124	20.09	
160.8969	20.22	
190.4701	20.51	
357.1824	46.67	
372.5734	18.63	
377.1836	999.00	$C_{17}H_{29}ClN_2O_5 + H^+$
381.0776	21.50	
405.1985	25.22	
441.1785	547.26	$C_{18}H_{33}ClN_2O_6S + H^+$

A reference standard of clindamycin-sulfoxide was purchased. Figure SI-D355 shows the extracted ion chromatograms of this standard, the sample and the spiked sample, as well as a head to tail plot of the MS2 spectra of the standard and the sample. In addition, the most intense MS2 fragments in the sample and in the standard are displayed. It becomes visible that the retention times of the sample and the spiked sample are identical and the spectra similarity score between sample and standard is equal to 0.935. The majority of the MS2 fragments in the sample can be explained by the reference standard. It can therefore be concluded that the suspected compound is indeed clindamycin-sulfoxide. Correspondingly, the identification confidence can be increased to level 1. The extracted ion chromatogram of the standard shows a double peak, which is caused by the fact that the reference standard was purchased as a mixture of diastereomers. In the sample, only one of the diastereomers can be detected.

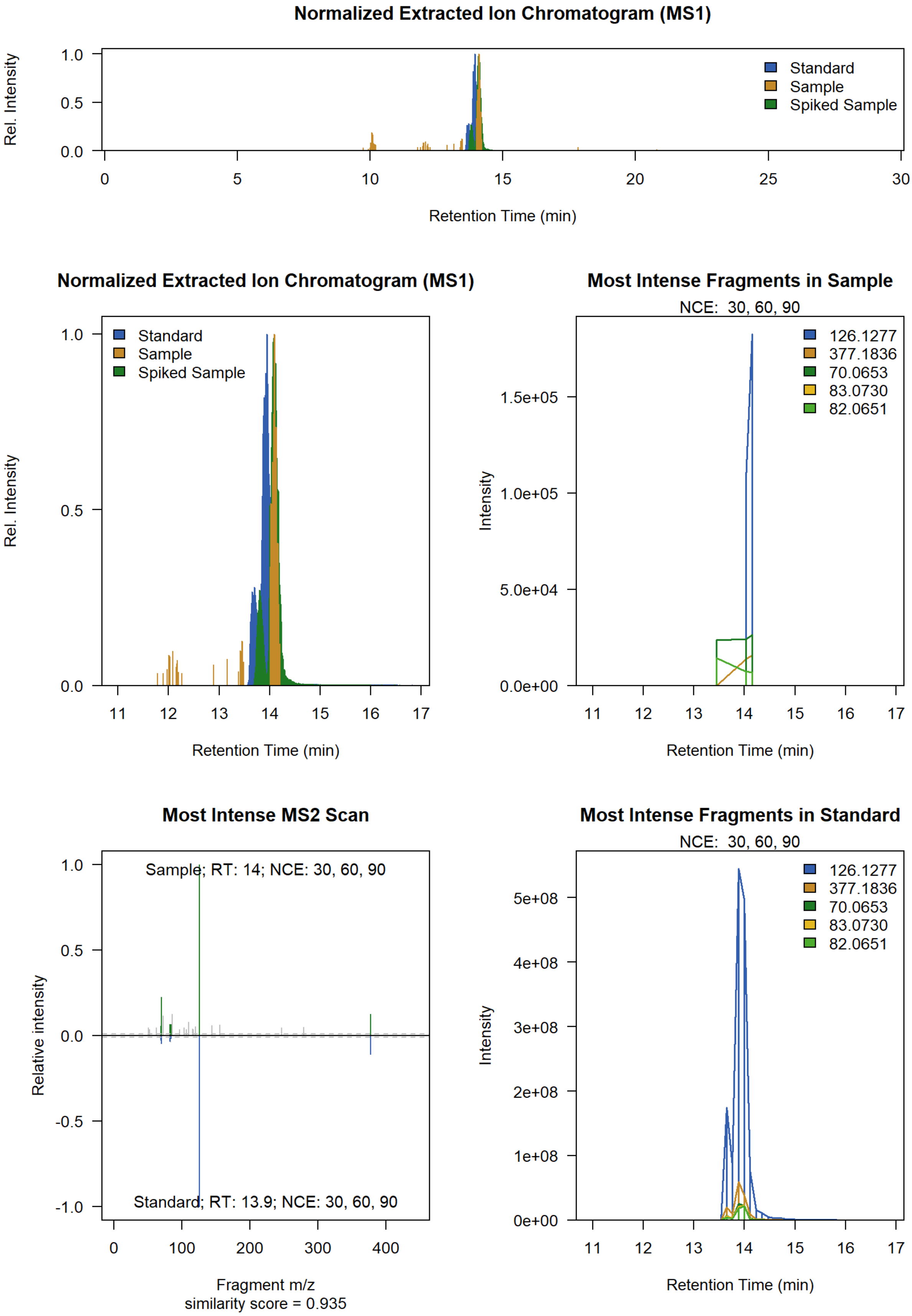

Figure SI-D355: Extracted ion chromatograms of clindamycin sulfoxide in the reference standard, the sample and the spiked sample, as well as MS2 head to tail plot and most intense MS2 fragments in standard and sample.

SI-D2.14.13 Desacetylbisacodyl

Desacetylbisacodyl is a metbaolite of bisacodyl, which is a stimulant laxative used for the temporary relief of occasional constipation and cleansing of the colon as a preparation for colonoscopy in adults.[2] Figure SI-D356 shows the metabolism scheme.

Table SI-D175: Information on identifiers, chemical properties, detection and confidence of identification of desacetylbisacodyl.

IUPAC Name	4-[(4-hydroxyphenyl)-pyridin-2-ylmethyl]phenol
Molecular formula	$C_{18}H_{15}NO_2$
Monoisotopic mass [g/mol]	277.1103
Adduct	$[M+H]^+$
Retention time [min]	12.6
SMILES	C1=CC=NC(=C1)C(C2=CC=C(C=C2)O)C3=CC=C (C=C3)O
InChI	InChI=1S/C18H15NO2/c20-15-8-4-13(5-9-15)18(17-3-1-2-12-19-17)14-6-10-16(21)11-7-14/h1-12,18,20-21H
InChI-Key	LJROKJGQSPMTKB-UHFFFAOYSA-N
CAS RN	603-41-8
Metabolite of	Bisacodyl
Detetction frequency	100% (15/15 samples)
Detected in	Altenrhein, Monday-Friday
	Neugut, Monday-Friday
	Werdhölzli, Monday-Friday
Intensity	E6-E7
Initial confidence level	level 3
Initial confidence score	0.31
Final confidence level	level 1

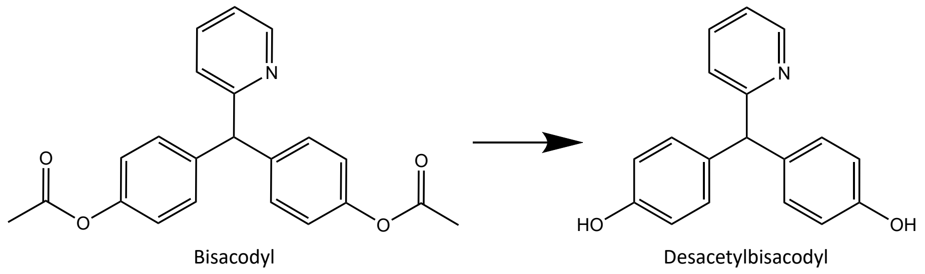

Figure SI-D356: Metabolism of bisacodyl to desacetylbisacodyl.

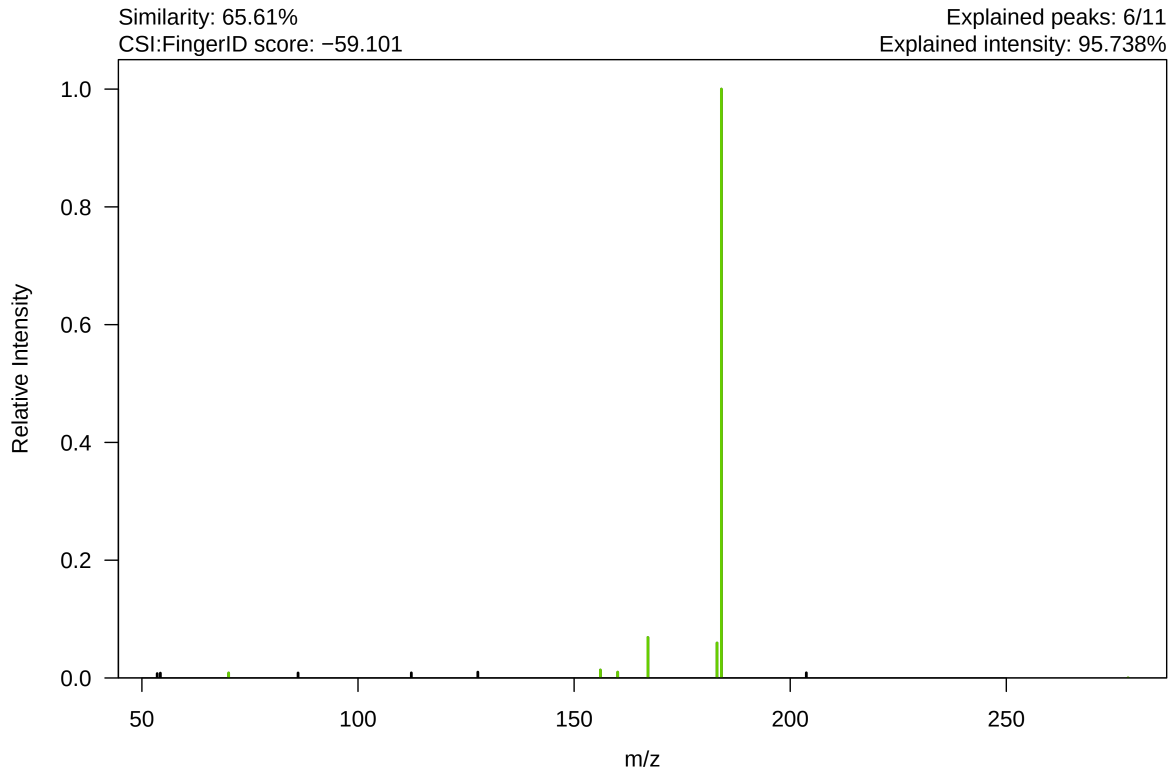

Figure SI-D357: Measured MS2 spectrum. Matching fragments with desacetylbisacodyl predicted by SIRIUS/CSI:FingerID are highlighted in green.

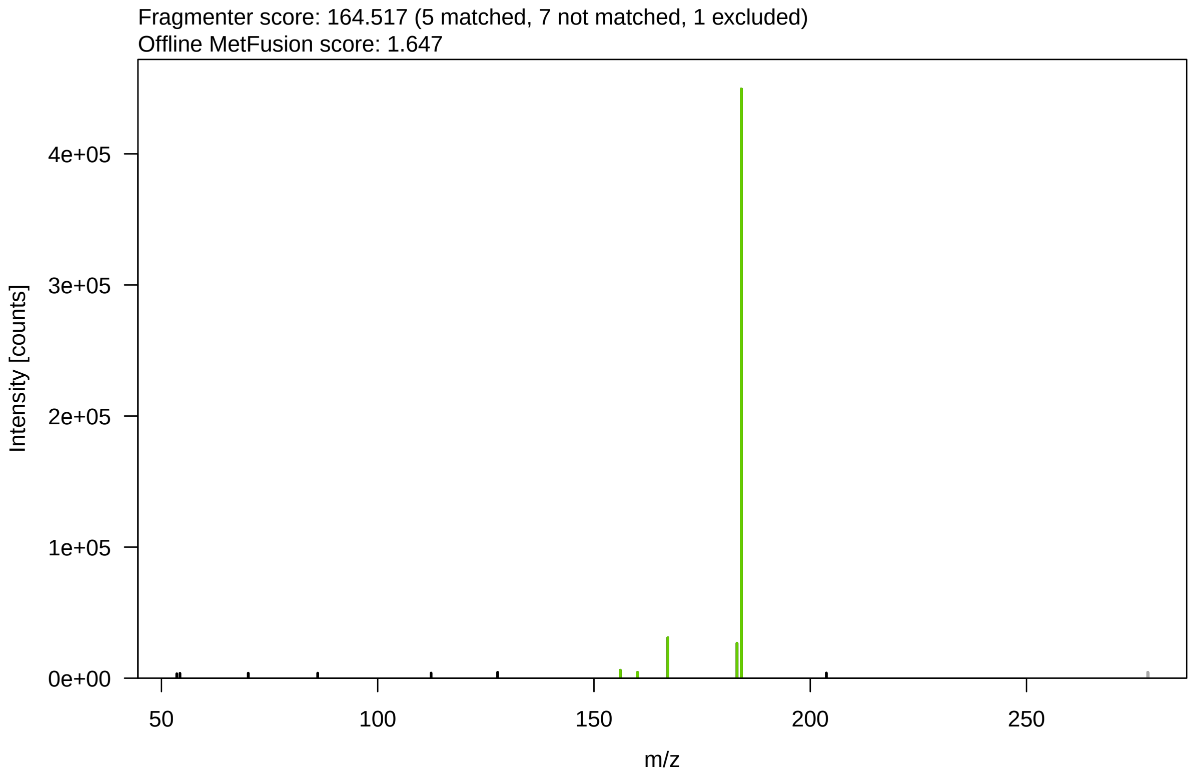

Figure SI-D358: Measured MS2 spectrum. Matching fragments with desacetylbisacodyl predicted by MetFrag are highlighted in green.

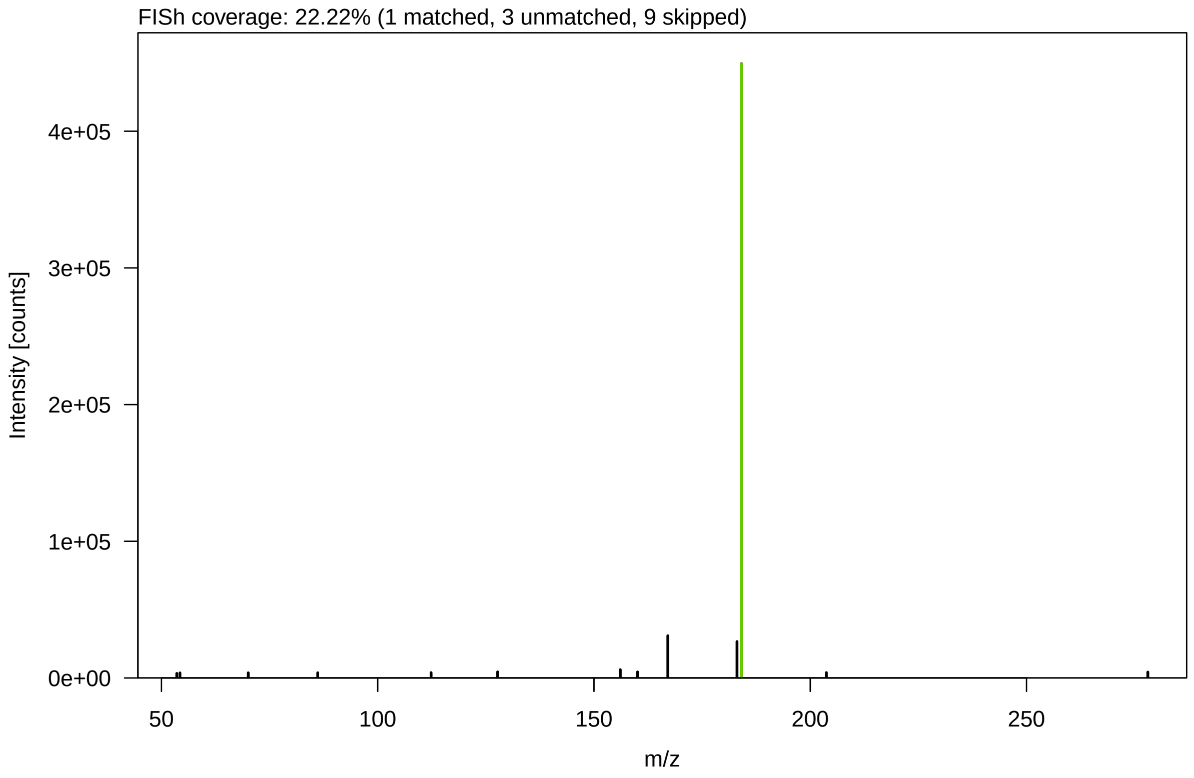

Figure SI-D359: Measured MS2 spectrum. Matching fragments with desacetylbisacodyl predicted by FISh Scoring are highlighted in green. Low intensity fragments are not considered and skipped.

Table SI-D176: Retention time prediction of desacetylbisacodyl.

Measured retention time [min]	12.6
Predicted logD_{OW} (pH = 2.7)	2.66
Predicted retention time [min]	18.2
Predicted retention time range (95% confidence interval) [min]	13.6-22.8
Predicted retention time range (99% confidence interval) [min]	12.1-24.3

Table SI-D177: Annotated MS2 spectrum of desacetylbisacodyl.

m/z	Relative Intensity	Annotation
53.5425	7.45	
54.2743	8.24	
70.0649	8.43	$C_4H_7N + H^+$
86.1323	8.51	
112.3376	8.67	
127.7267	9.94	
156.0808	13.50	$C_{11}H_9N + H^+$
160.0753	9.81	$C_{10}H_9NO + H^+$
167.0729	68.67	$C_{12}H_8N + H^+$
183.0675	59.26	$C_{12}H_8NO + H^+$
184.0756	999.00	$C_{12}H_9NO + H^+$
203.7260	8.77	
278.0493	9.56	

A reference standard of desacetylbisacodyl was purchased. Figure SI-D360 shows the extracted ion chromatograms of this standard, the sample and the spiked sample, as well as a head to tail plot of the MS2 spectra of the standard and the sample. In addition, the most intense MS2 fragments in the sample and in the standard are displayed. It becomes visible that the retention times of the sample and the spiked sample are identical and the spectra similarity score between sample and standard is equal to 0.995. The vast majority of the MS2 fragments in the sample can be explained by the reference standard. It can therefore be concluded that the suspected compound is indeed desacetylbisacodyl. Correspondingly, the identification confidence can be increased to level 1.

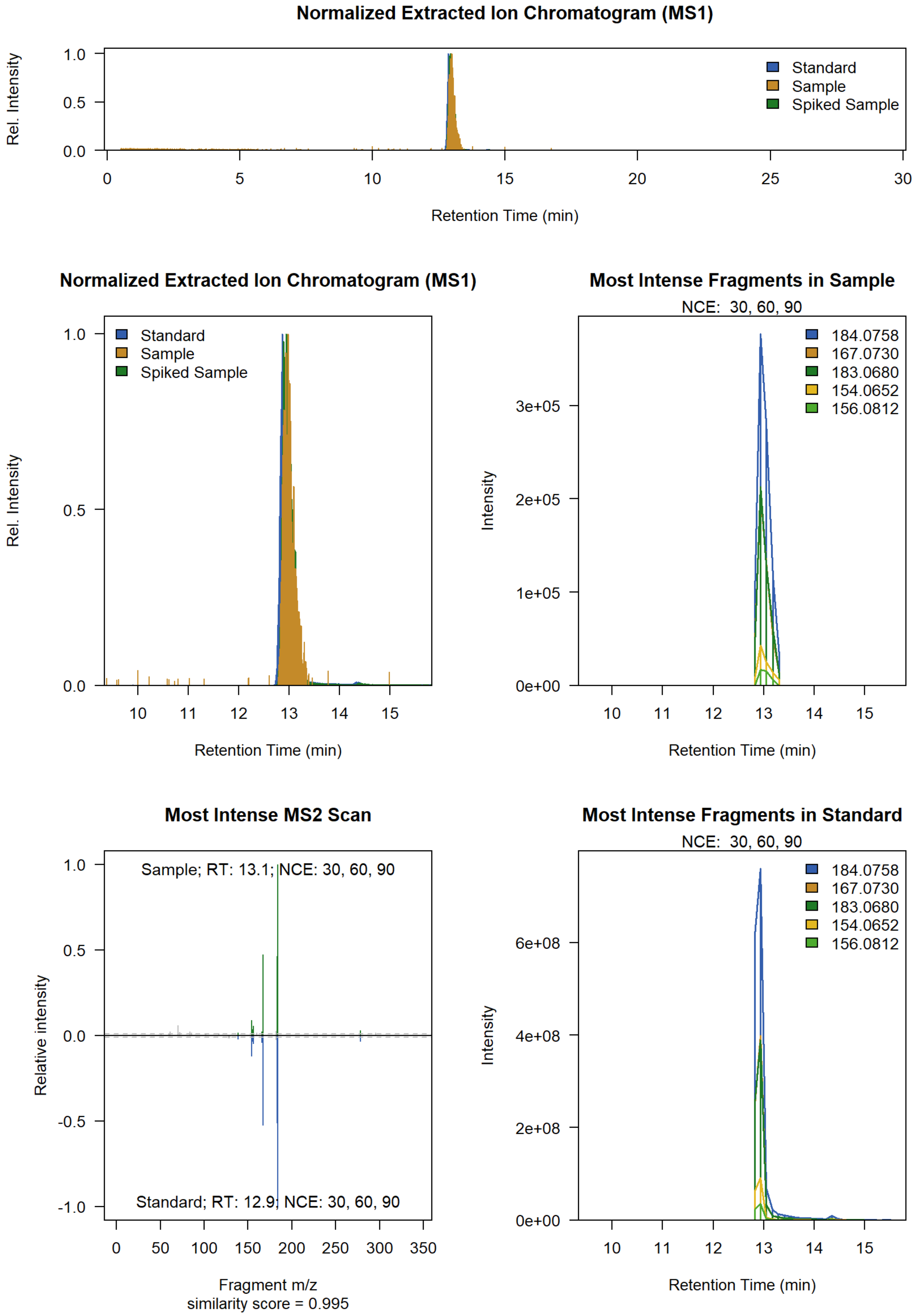

Figure SI-D360: Extracted ion chromatograms of desacetylbisacodyl in the reference standard, the sample and the spiked sample, as well as MS2 head to tail plot and most intense MS2 fragments in standard and sample.

SI-D2.14.14 Hydroxylevetiracetam

Hydroxylevetiracetam is a metabolite of levetiracetam, which belongs to the pyrrolidine class and is used to treat various types of seizures stemming from epileptic disorders.[2] Figure SI-D361 shows the metabolism scheme.

Table SI-D178: Information on identifiers, chemical properties, detection and confidence of identification of hydroxylevetiracetam.

IUPAC Name	2-(2-hydroxy-5-oxopyrrolidin-1-yl)butanamide
Molecular formula	$C_8H_{14}N_2O_3$
Monoisotopic mass [g/mol]	186.1004
Adduct	$[M+H]^+$
Retention time [min]	10.5
SMILES	CCC(C(=O)N)N1C(CCC1=O)O
InChI	InChI=1S/C8H14N2O3/c1-2-5(8(9)13)10-6(11)3-4-7(10)12/h5-6,11H,2-4H2,1H3,(H2,9,13)
InChI-Key	TZEPAZLOURDLKF-UHFFFAOYSA-N
CAS RN	-
Metabolite of	Levetiracetam
Detection frequency	100% (15/15 samples)
Detected in	Altenrhein, Monday-Friday
	Neugut, Monday-Friday
	Werdhölzli, Monday-Friday
Intensity	E8
Initial confidence level	level 3
Initial confidence score	0.50
Final confidence level	level 3

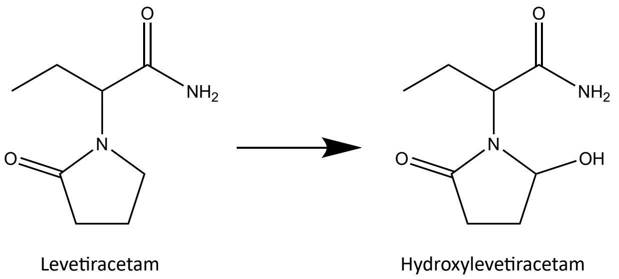

Figure SI-D361: Metabolism of levetiracetam to hydroxylevetiracetam.

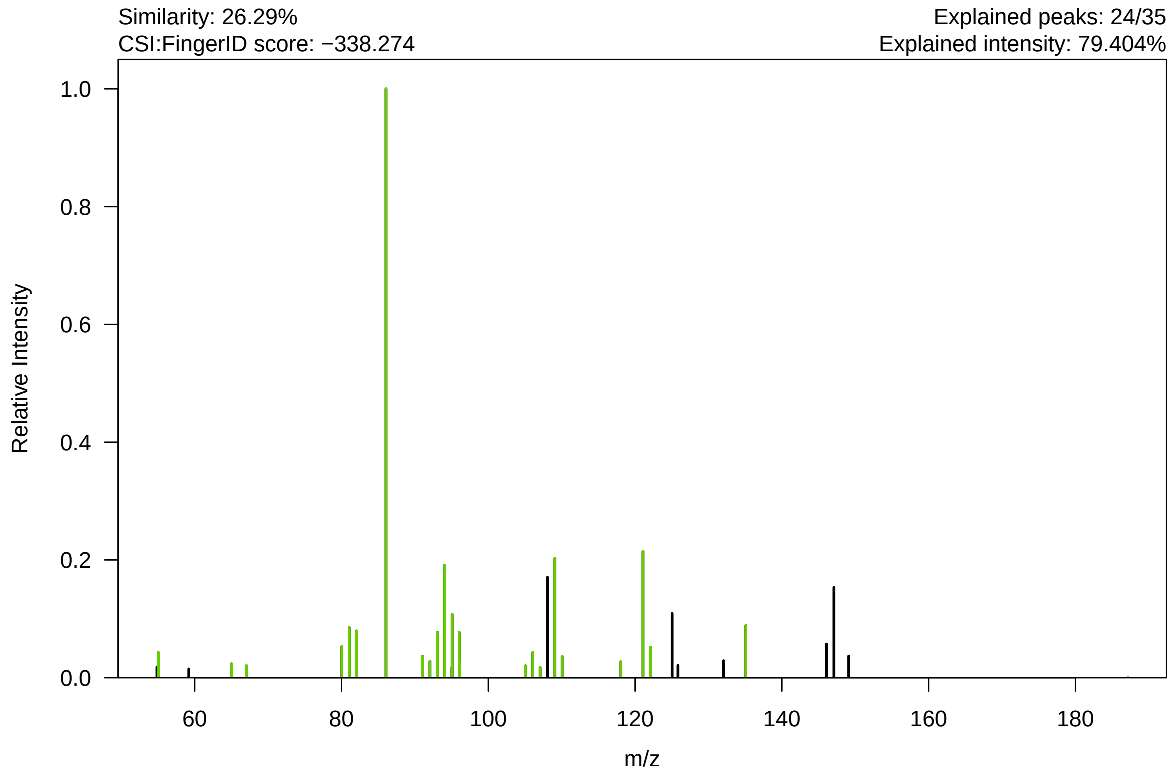

Figure SI-D362: Measured MS2 spectrum. Matching fragments with hydroxylevetiracetam predicted by SIRIUS/CSI:FingerID are highlighted in green. The molecular ion in gray is not considered.

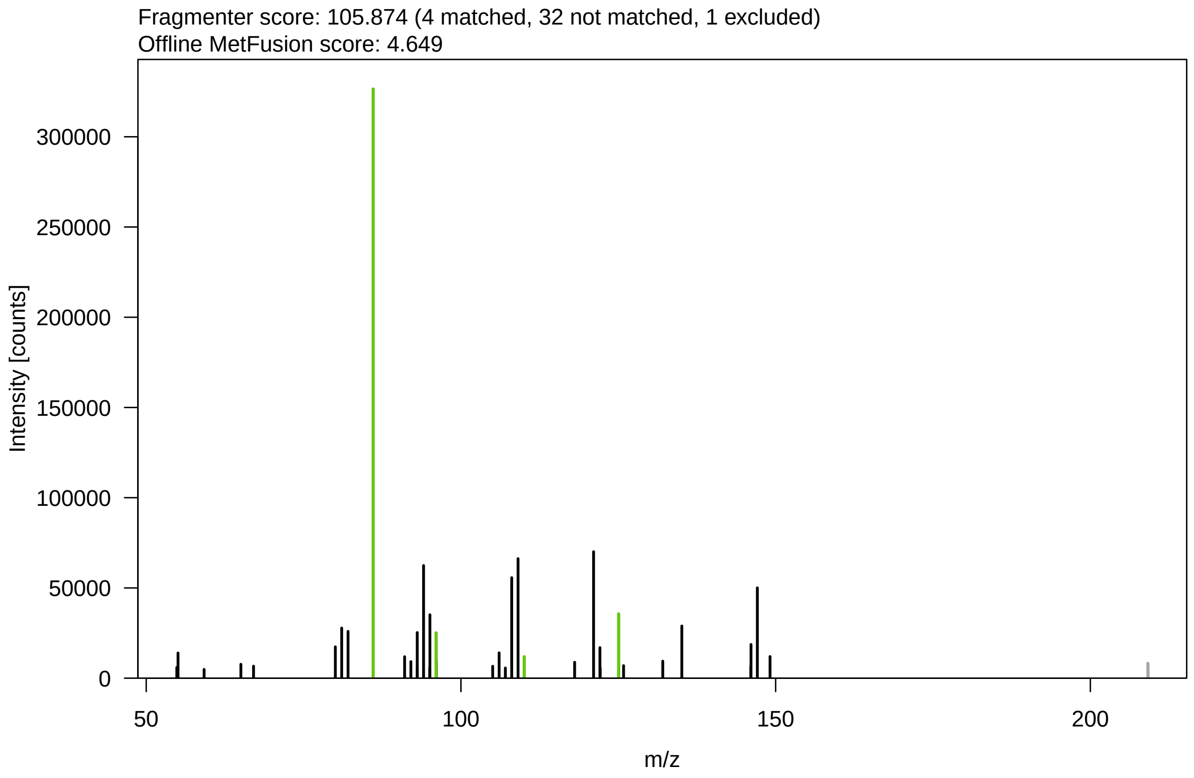

Figure SI-D363: Measured MS2 spectrum. Matching fragments with hydroxylevetiracetam predicted by MetFrag are highlighted in green. The molecular ion in gray is not considered.

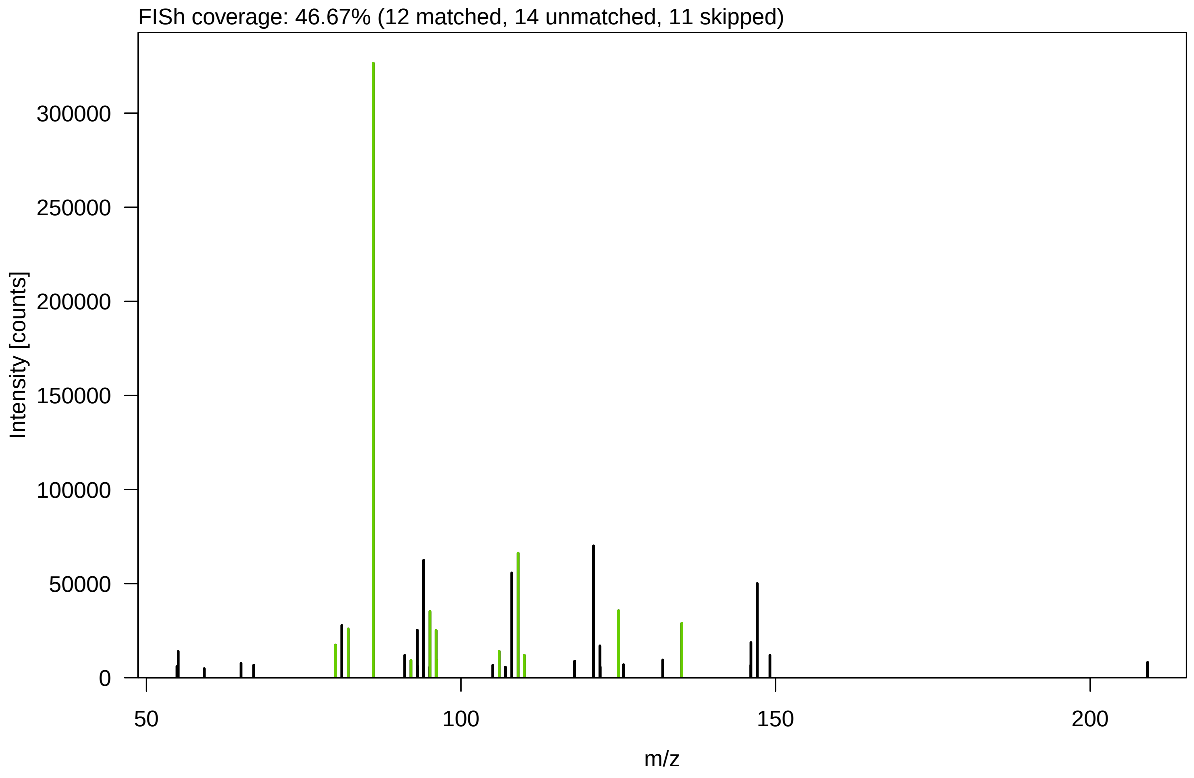

Figure SI-D364: Measured MS2 spectrum. Matching fragments with hydroxylevetiracetam predicted by FISh Scoring are highlighted in green. Low intensity fragments are not considered and skipped.

Table SI-D179: Retention time prediction of hydroxylevetiracetam.

Measured retention time [min]	10.5
Predicted logD_{OW} (pH = 2.7)	-1.19
Predicted retention time [min]	13.2
Predicted retention time range (95% confidence interval) [min]	8.6-17.8
Predicted retention time range (99% confidence interval) [min]	7.1-19.3

Table SI-D180: Annotated MS2 spectrum of hydroxylevetiracetam.

m/z	Relative Intensity	Annotation
54.8573	18.22	
55.0546	42.73	$C_4H_6 + H^+$
59.1963	14.85	
65.0386	23.56	$C_5H_4 + H^+$
67.0540	20.43	$C_5H_6 + H^+$
80.0492	53.14	$C_5H_5N + H^+$
81.0572	85.00	$C_5H_6N + H^+$
82.0648	79.34	$C_5H_7N + H^+$

Continued on next page

Table SI-D180: Annotated MS2 spectrum of hydroxylevetiracetam.(Continued)

86.0599	999.00	$C_4H_7NO + H^+$
91.0540	36.38	$C_7H_6 + H^+$
92.0493	28.11	$C_6H_5N + H^+$
93.0571	77.39	$C_6H_6N + H^+$
93.0698	18.87	
94.0651	190.98	$C_6H_7N + H^+$
95.0489	17.41	$C_6H_8N + H^+$
95.0728	107.57	$C_6H_8N + H^+$
96.0442	76.85	$C_5H_5NO + H^+$
96.0807	28.98	$C_6H_9N + H^+$
105.0445	20.22	$C_6H_4N_2 + H^+$
106.0650	42.95	$C_7H_7N + H^+$
107.0605	17.29	$C_6H_6N_2 + H^+$
108.0680	170.39	
109.0759	202.69	$C_6H_8N_2 + H^+$
110.0600	36.44	$C_6H_7NO + H^+$
118.0649	26.96	$C_8H_7N + H^+$
121.0758	214.41	$C_7H_8N_2 + H^+$
122.0836	51.80	$C_7H_9N_2 + H^+$
122.1214	17.43	
125.0468	109.00	$C_6H_6NO_2 + H^+$
125.8397	21.21	
132.0680	28.85	
135.0916	88.47	$C_8H_{10}N_2 + H^+$
146.0594	20.24	
146.0835	57.15	
147.0913	153.17	
149.1066	36.74	
209.1278	24.98	

No reference standard of hydroxylevetiracetam was purchasable. Therefore, a human liver S9 incubation experiment with levetiracetam was performed, to generate levetiracetam metabolites *in vitro*. However, no compound with a precursor matching the one of hydroxylevetiracetam was detected. Since *in vitro* experiments cannot be one to one translated into *in vivo* experiments, the suspected compound can neither be confirmed as hydroxylevetiracetam nor rejected. As a consequence, the final identification confidence remains at level 3.

SI-D2.14.15 Licarbazepine

Licarbazepine is a metabolite of carbamazepine, an antioconvulsant and analgesic drug used to control seizures and to treat pain resulting from trigeminal neuralgia.[2] Figure SI-D365 shows the metabolism scheme and Figure SI-D366 the carbamazepine cluster.

Table SI-D181: Information on identifiers, chemical properties, detection and confidence of identification of licarbazepine.

IUPAC Name	5-hydroxy-5,6-dihydrobenzo[b][1]benzazepine-11-carboxamide
Molecular formula	$C_{15}H_{14}N_2O_2$
Monoisotopic mass [g/mol]	254.1055
Adduct	$[M+H]^+$
Retention time [min]	16.5
SMILES	C1C(C2=CC=CC=C2N(C3=CC=CC=C31)C(=O)N)O
InChI	InChI=1S/C15H14N2O2/c16-15(19)17-12-7-3-1-5-10(12)9-14(18)11-6-2-4-8-13(11)17/h1-8,14,18H,9H2,(H2,16,19)
InChI-Key	BMPDWHIDQYTSHX-UHFFFAOYSA-N
CAS RN	29331-92-8
Metabolite of	Carbamezepine
Detection frequency	100% (15/15 samples)
Detected in	Altenrhein, Monday-Friday<br>Neugut, Monday-Friday<br>Werdhölzli, Monday-Friday
Intensity	E6-E7
Initial confidence level	level 2a
Initial confidence score	0.67
Final confidence level	level 1

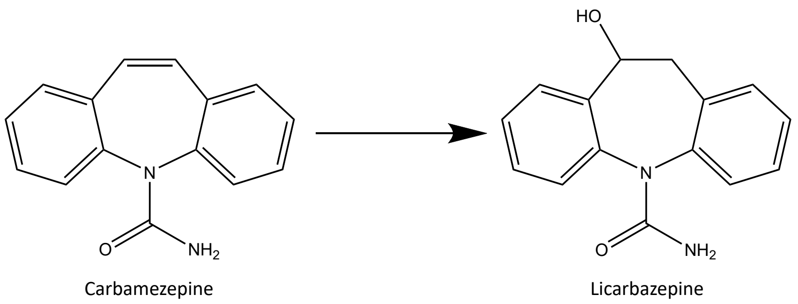

Figure SI-D365: Metabolism of carbamazepine to licarbazepine.

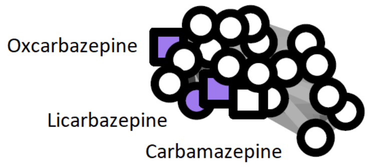

Figure SI-D366: Excerpt of the molecular network showing the carbamazepine cluster.

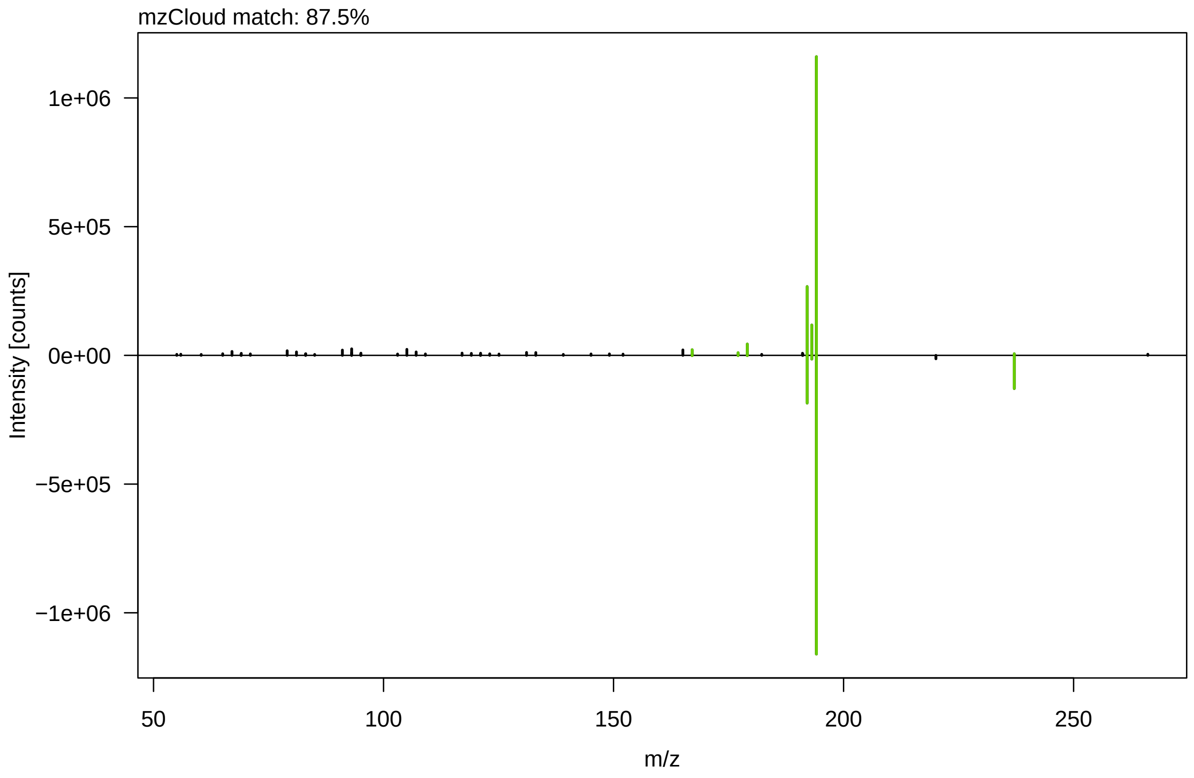

Figure SI-D367: Head to tail plot of measured MS2 spectrum against mzCloud library spectrum of licarbazepine. Matching fragments are highlighted in green.

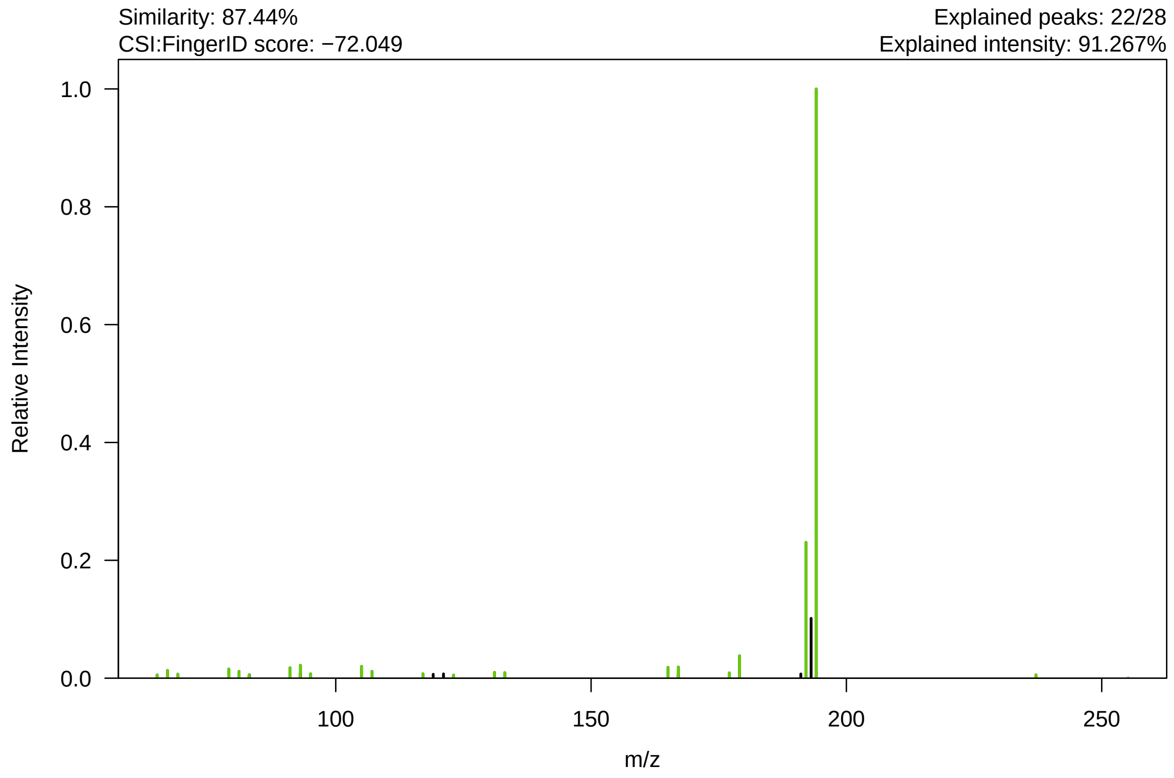

Figure SI-D368: Measured MS2 spectrum. Matching fragments with licarbazepine predicted by SIRIUS/CSI:FingerID are highlighted in green. The molecular ion in gray is not considered.

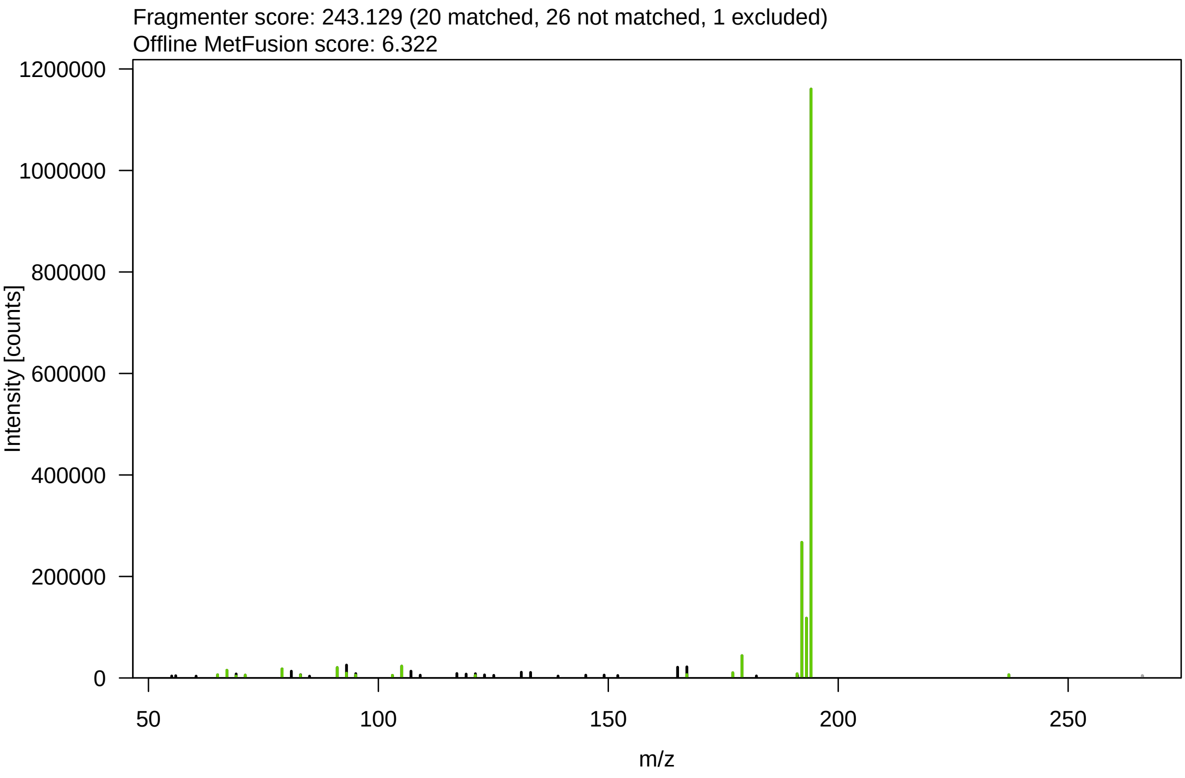

Figure SI-D369: Measured MS2 spectrum. Matching fragments with licarbazepine predicted by MetFrag are highlighted in green. The molecular ion in gray is not considered.

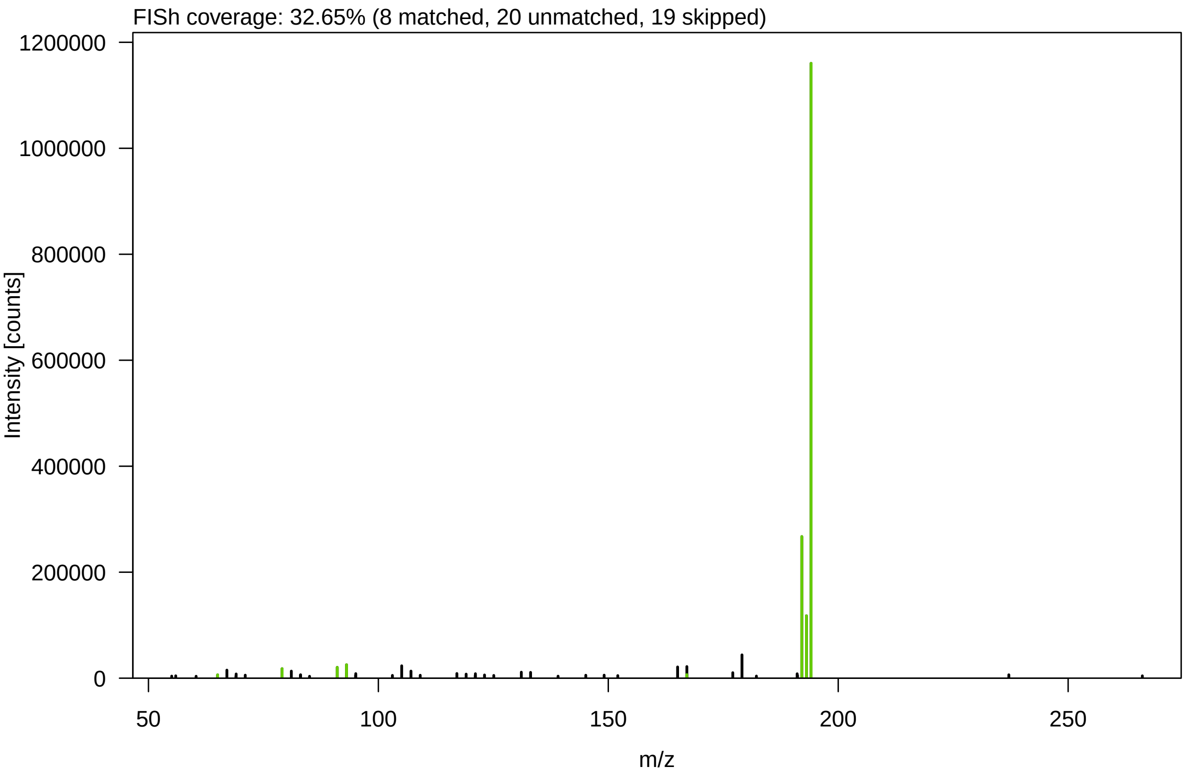

Figure SI-D370: Measured MS2 spectrum. Matching fragments with licarbazepine predicted by FISh Scoring are highlighted in green. Low intensity fragments are not considered and skipped.

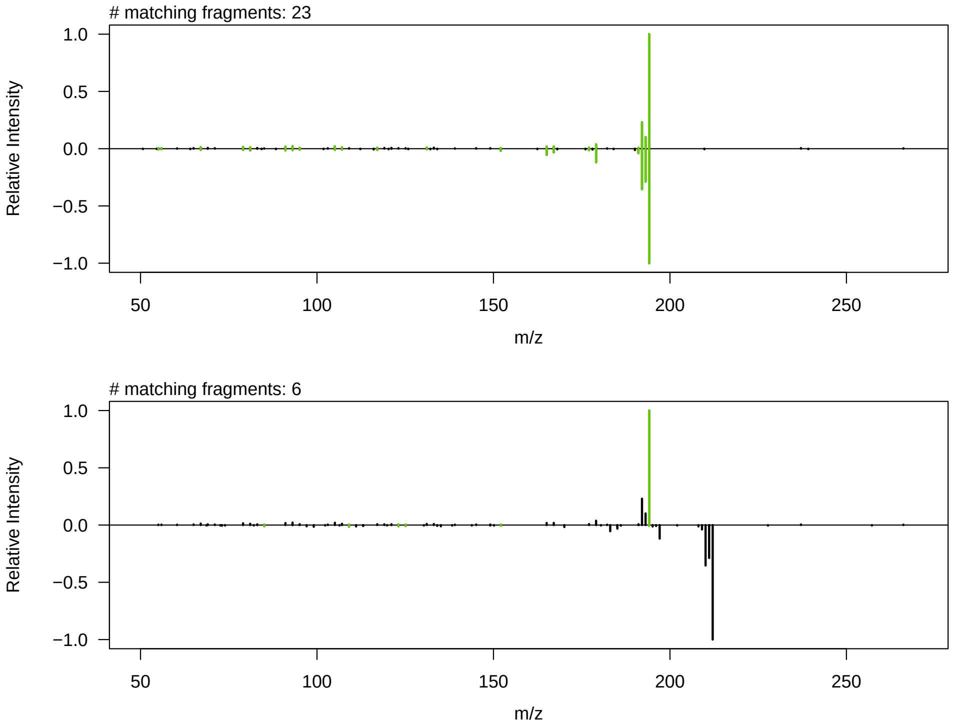

Figure SI-D371: Head to tail plots of licarbazepine and carbamazepine. In the bottom plot, the mass spectrum of carbamazepine is shifted by the mass difference. Matching fragments are highlighted in green.

Table SI-D182: Molecular network results and retention time prediction of licarbazepine.

Comparison with	Carbamazepine
MSn Score	76
Forward coverage	74
Reverse coverage	78
Forward match	59
Reverse match	38
ΔMass [g/mol]	18.0106
Measured retention time [min]	16.5
Predicted $logD_{OW}$ (pH = 2.7)	1.73
Predicted retention time [min]	17.0
Predicted retention time range (95% confidence interval) [min]	12.4-21.6
Predicted retention time range (99% confidence interval) [min]	10.9-23.1

Table SI-D183: Annotated MS2 spectrum of licarbazepine.

m/z	Relative Intensity	Annotation
55.0546	3.36	
55.9264	3.82	
60.3606	3.10	
65.0385	5.46	$C_5H_4 + H^+$
67.0542	13.08	$C_5H_6 + H^+$
69.0334	3.33	$C_4H_4O + H^+$
69.0699	6.82	$C_5H_8 + H^+$
71.0491	4.88	$C_4H_6O + H^+$
79.0541	15.47	$C_6H_6 + H^+$
81.0697	11.48	$C_6H_8 + H^+$
83.0492	5.03	$C_5H_6O + H^+$
83.0852	5.54	
85.0281	3.08	
91.0542	17.67	$C_7H_6 + H^+$
93.0572	8.37	$C_6H_6N + H^+$
93.0698	21.88	$C_7H_8 + H^+$
95.0494	4.96	$C_6H_6O + H^+$
95.0853	7.37	$C_7H_{10} + H^+$
103.0539	4.48	$C_8H_6 + H\equiv^+$
105.0699	20.10	$C_8H_8 + H^+$
107.0854	11.51	$C_8H_{10} + H^+$
109.1010	4.72	
117.0697	7.55	$C_9H_8 + H^+$
119.0858	6.60	
121.0640	4.31	$C_8H_8O + H^+$
121.1010	7.26	
123.0802	5.18	$C_8H_{10}O + H^+$
125.0963	4.42	
131.0856	9.74	$C_{10}H_{10} + H^+$
133.1012	9.40	$C_{10}H_{12} + H^+$
139.0744	3.24	
145.1013	4.76	
149.0959	4.88	
152.0617	4.17	
165.0696	18.21	$C_{13}H_8 + H^+$
167.0727	5.86	$C_{12}H_8N + H^+$

Continued on next page

Table SI-D183: Annotated MS2 spectrum of licarbazepine.(Continued)

167.0848	18.85	$C_{13}H_{10} + H^+$
177.0697	8.90	$C_{14}H_8 + H^+$
179.0727	37.84	$C_{13}H_8N + H^+$
182.2084	3.44	
191.0729	7.13	$C_{14}H_8N$ + H≡;
191.1422	4.70	
192.0807	230.11	$C_{14}H_9N + H^+$
193.0886	101.45	$C_{14}H_{10}N + H^+$
194.0964	999.00	$C_{14}H_{11}N + H^+$
237.1022	5.51	$C_{15}H_{12}N_2O + H^+$
266.1404	3.79	

A reference standard of licarbazepine was purchased. Figure SI-D372 shows the extracted ion chromatograms of this standard, the sample and the spiked sample, as well as a head to tail plot of the MS2 spectra of the standard and the sample. In addition, the most intense MS2 fragments in the sample and in the standard are displayed. It becomes visible that the retention times of the sample and the spiked sample are identical and the spectra similarity score between sample and standard is equal to 0.992. The majority of the MS2 fragments in the sample can be explained by the reference standard. It can therefore be concluded that the suspected compound is indeed licarbazepine. Correspondingly, the identification confidence can be increased to level 1.

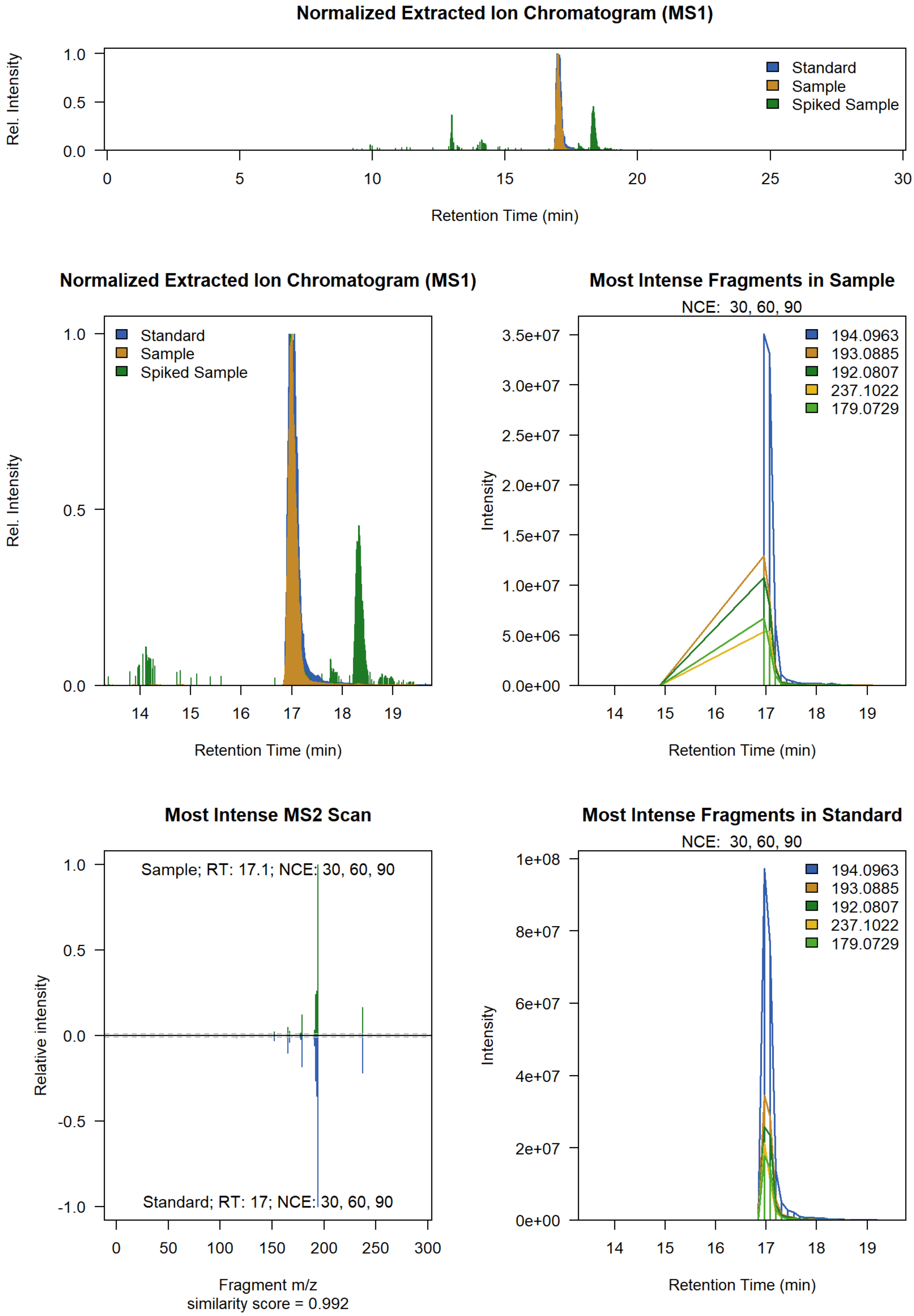

Figure SI-D372: Extracted ion chromatograms of licarbazepine in the reference standard, the sample and the spiked sample, as well as MS2 head to tail plot and most intense MS2 fragments in standard and sample.

SI-D2.14.16 N-Desmethylrosuvastatin

N-Desmethylrosuvastatin is a metabolite of rosuvastatin, which is an HMG-CoA reductase inhibitor used to lower lipid levels and reduce the risk of cardiovascular disease including myocardial infarction and stroke.[2] Figure SI-D373 shows the metabolism scheme.

Table SI-D184: Information on identifiers, chemical properties, detection and confidence of identification of N-desmethylrosuvastatin.

IUPAC Name	(*E*,3*R*,5*S*)-7-[4-(4-fluorophenyl)-2-(methanesulfonamido)-6-propan-2-ylpyrimidin-5-yl]-3,5-dihydroxyhept-6-enoic acid
Molecular formula	$C_{21}H_{26}FN_3O_6S$
Monoisotopic mass [g/mol]	467.1526
Adduct	$[M-H]^-$
Retention time [min]	15.5
SMILES	CC(C)C1=NC(=NC(=C1/C=C/[C@H](C[C@H](CC(=O)O)O)O)C2=CC=C(C=C2)F)NS(=O)(=O)C
InChI	InChI=1S/C21H26FN3O6S/c1-12(2)19-17(9-8-15(26)10-16(27)11-18(28)29)20(13-4-6-14(22)7-5-13)24-21(23-19)25-32(3,30)31/h4-9,12,15-16,26-27H,10-11H2,1-3H3,(H,28,29)(H,23,24,25)/b9-8+/t15-,16-/m1/s1
InChI-Key	DJUKMHIJCDJSIJ-GUFYHEMZSA-N
CAS RN	371775-74-5
Metabolite of	Rosuvastatin
Detection frequency	100% (15/15 samples)
Detected in	Altenrhein, Monday-Friday<br>Neugut, Monday-Friday<br>Werdhölzli, Monday-Friday
Intensity	E5-E6
Initial confidence level	level 3
Initial confidence score	0.32
Final confidence level	level 1

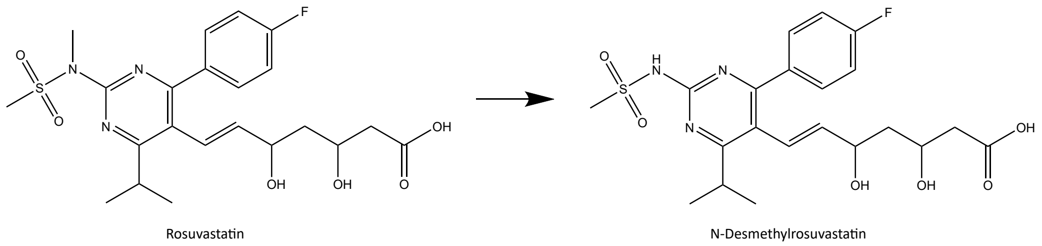

Figure SI-D373: Metabolism of rosuvastatin to N-desmethylrosuvastatin.

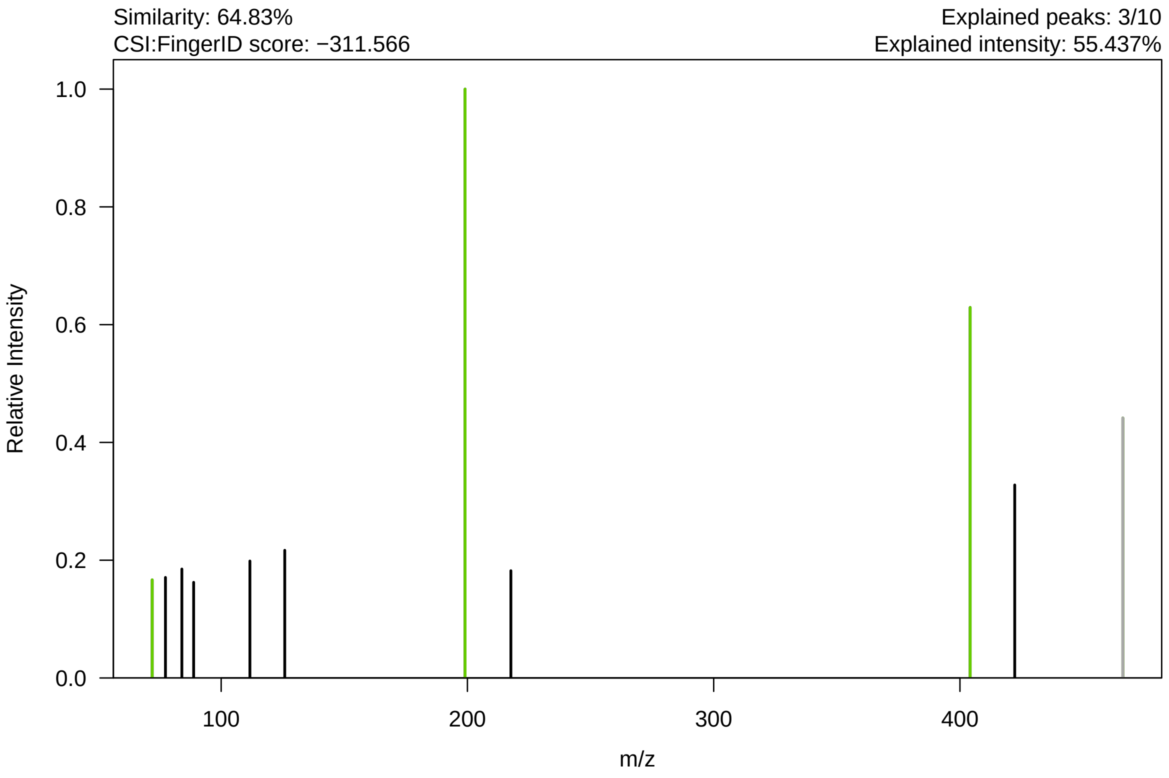

Figure SI-D374: Measured MS2 spectrum. Matching fragments with N-desmethylrosuvastatin predicted by SIRIUS/CSI:FingerID are highlighted in green. The molecular ion in gray is not considered.

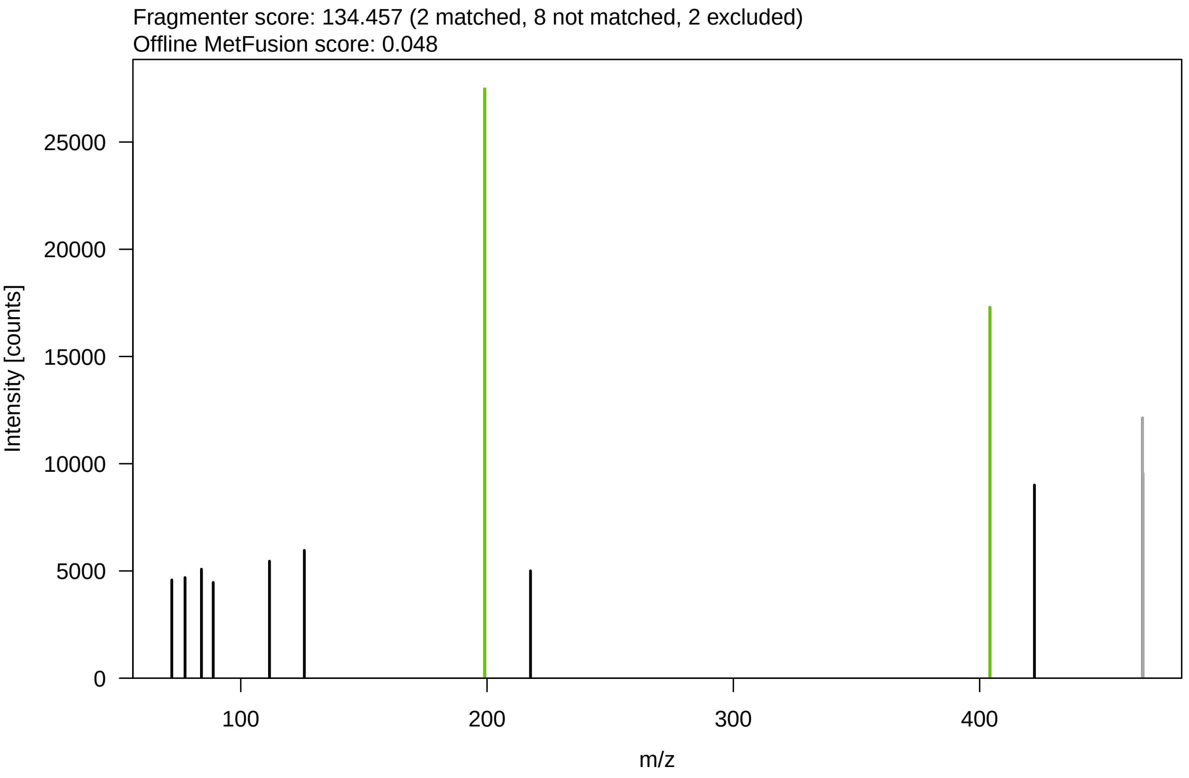

Figure SI-D375: Measured MS2 spectrum. Matching fragments with N-desmethylrosuvastatin predicted by MetFrag are highlighted in green. The molecular ion in gray is not considered.

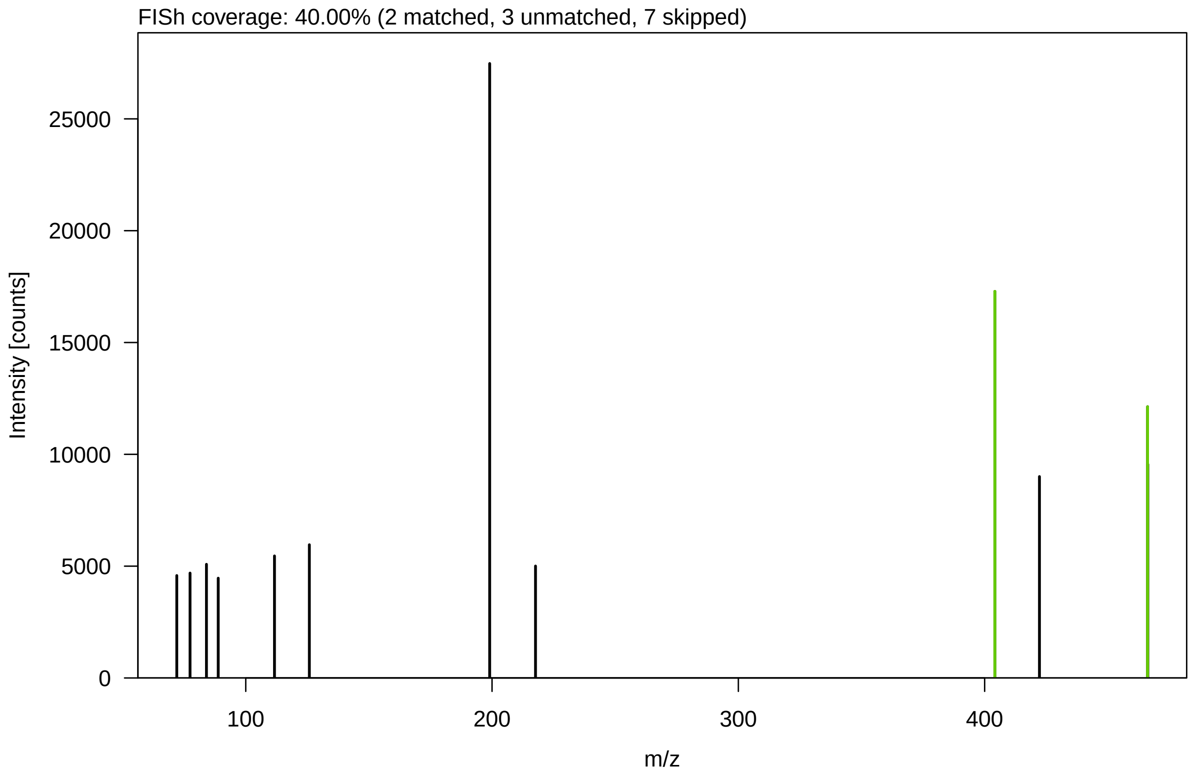

Figure SI-D376: Measured MS2 spectrum. Matching fragments with N-desmethylrosuvastatin predicted by FISh Scoring are highlighted in green. Low intensity fragments are not considered and skipped.

Table SI-D185: Retention time prediction of N-desmethylrosuvastatin.

Measured retention time [min]	15.5
Predicted $\log D_{OW}$ (pH = 4.8)	0.87
Predicted retention time [min]	16.0
Predicted retention time range (95% confidence interval) [min]	8.9-23.1
Predicted retention time range (99% confidence interval) [min]	6.6-25.4

Table SI-D186: Annotated MS2 spectrum of N-desmethylrosuvastatin.

m/z	Relative Intensity	Annotation
72.0038	166.52	$C_3H_5S - H^-$
77.3871	170.56	
84.0499	184.81	
88.8276	162.27	
111.6718	198.41	
125.8244	216.63	
199.0435	999.00	$C_9H_{12}O_3S - H^-$
217.6489	181.98	
404.1449	628.54	$C_{20}H_{24}FN_3O_3S - H^-$
422.2457	327.45	
466.1445	441.07	$C_{21}H_{26}FN_3O_6S - H^-$
466.2496	347.48	

A reference standard of N-desmethylrosuvastatin was purchased. Figure SI-D377 shows the extracted ion chromatograms of this standard, the sample and the spiked sample, as well as a head to tail plot of the MS2 spectra of the standard and the sample. In addition, the most intense MS2 fragments in the sample and in standard are displayed. It becomes visible that the retention times of the sample and the spiked sample are identical. The spectra similarity score between sample and standard is only equal to 0.264, which is a result of the low quality and noisy MS2 spectrum of N-desmethylrosuvastatin in the sample, resulting from a low intensity signal. Nonetheless, three MS2 fragments in the sample can be explained. Correspondingly, the suspected compound is confirmed as N-desmethylrosuvastatin and the confidence level is increased to level 1.

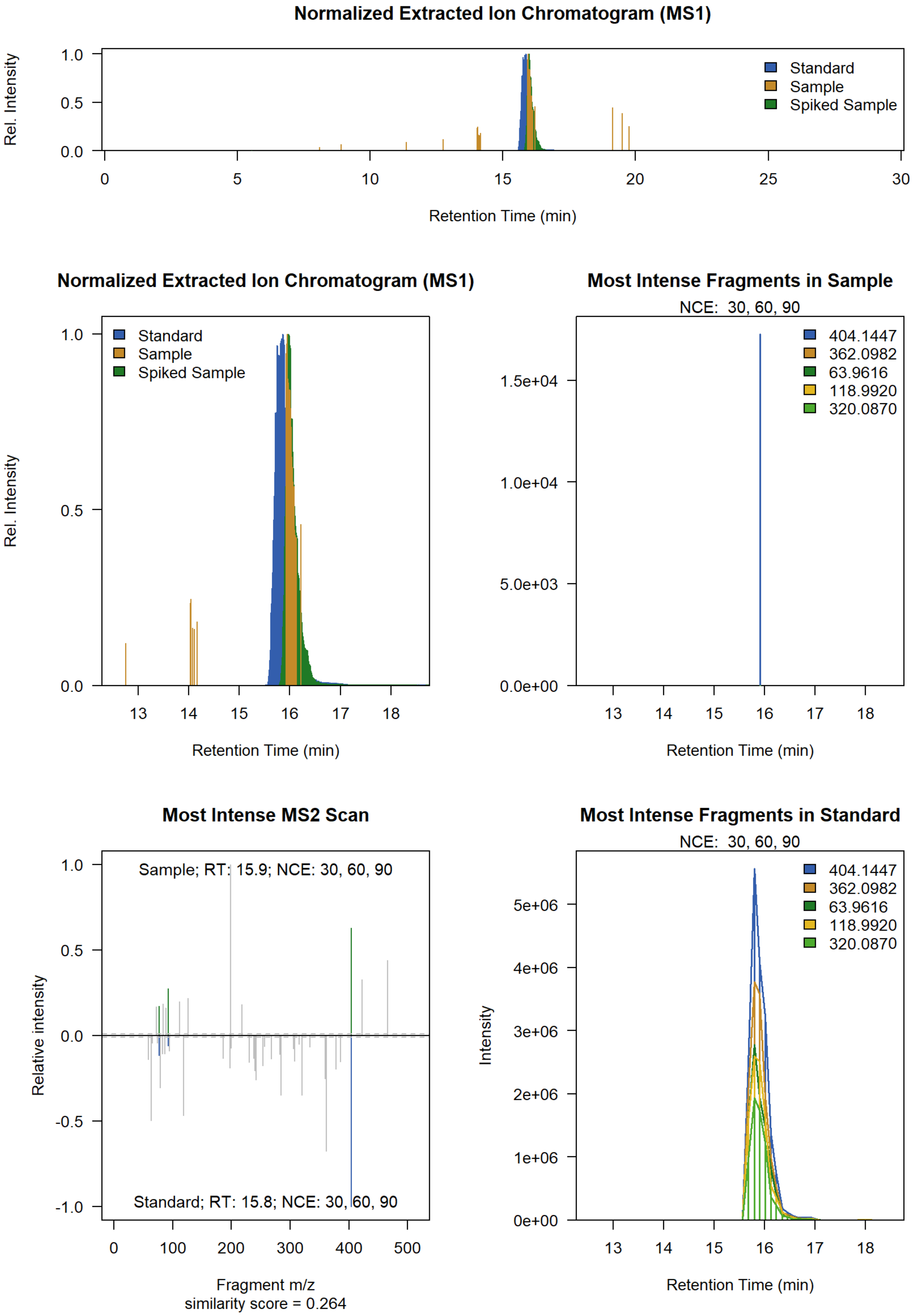

Figure SI-D377: Extracted ion chromatograms of N-desmethylrosuvastatin in the reference standard, the sample and the spiked sample, as well as MS2 head to tail plot and most intense MS2 fragments in sample and standard.

SI-D2.14.17 N-Methylpregabalin

N-Methylpregabalin is a metabolite of pregabalin. The structure of pregabalin is similar to gamma-aminobutyric acid, an inhibitory neurotransmitter. Pregabalin is used to treat neuropathic pain and fibromyalgia.[2] Figure SI-D378 shows the metabolism scheme and Figure SI-D379 the pregabalin cluster.

Table SI-D187: Information on identifiers, chemical properties, detection and confidence of identification of N-methylpergabalin.

IUPAC Name	(3*S*)-5-methyl-3-(methylaminomethyl)hexanoic acid
Molecular formula	$C_9H_{19}NO_2$
Monoisotopic mass [g/mol]	173.1416
Adduct	$[M+H]^+$
Retention time [min]	5.5
SMILES	CC(C)C[C@@H](CC(=O)O)CNC
InChI	InChI=1S/C9H19NO2/c1-7(2)4-8(6-10-3)5-9(11)12/h7-8,10H,4-6H2,1-3H3,(H,11,12)/t8-/m0/s1
InChI-Key	MADUVMLGQASXOK-QMMMGPOBSA-N
CAS RN	1155843-61-0
Metabolite of	Pregabalin
Detection frequency	33% (5/15 samples)
Detected in	Altenrhein, Monday-Friday
Intensity	E8-E9
Initial confidence level	level 3
Initial confidence score	0.39
Final confidence level	level 4

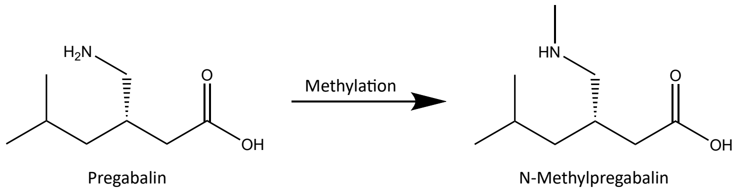

Figure SI-D378: Metabolism of pregabalin to N-methylpregabalin.

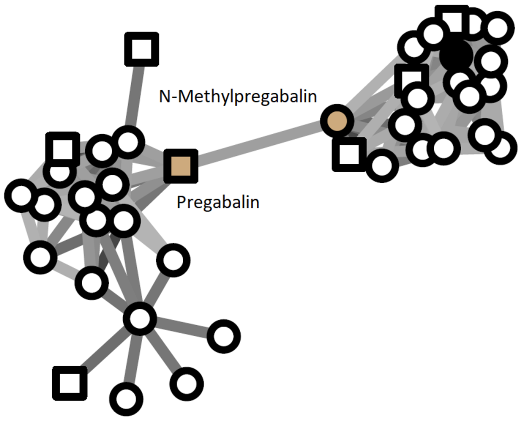

Figure SI-D379: Excerpt of the molecular network showing the pregabalin cluster.

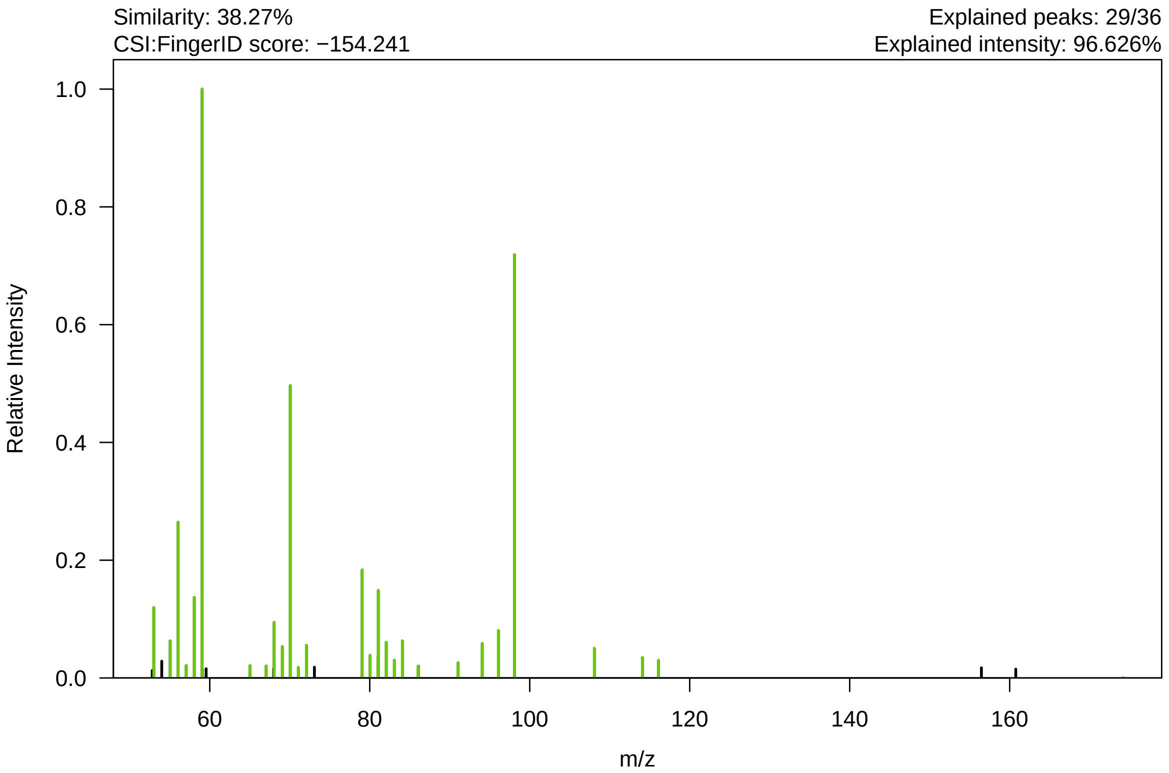

Figure SI-D380: Measured MS2 spectrum. Matching fragments with N-methylpregabalin predicted by SIRIUS/CSI:FingerID are highlighted in green.

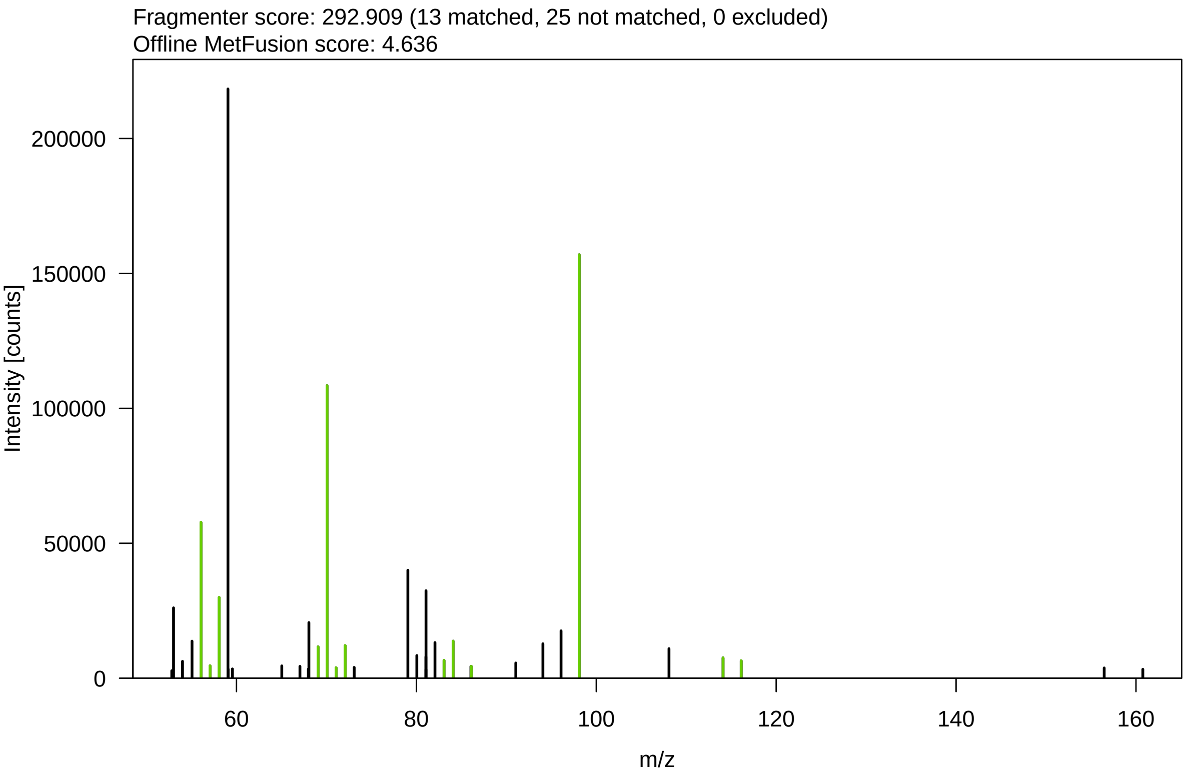

Figure SI-D381: Measured MS2 spectrum. Matching fragments with N-methylpregabalin predicted by MetFrag are highlighted in green.

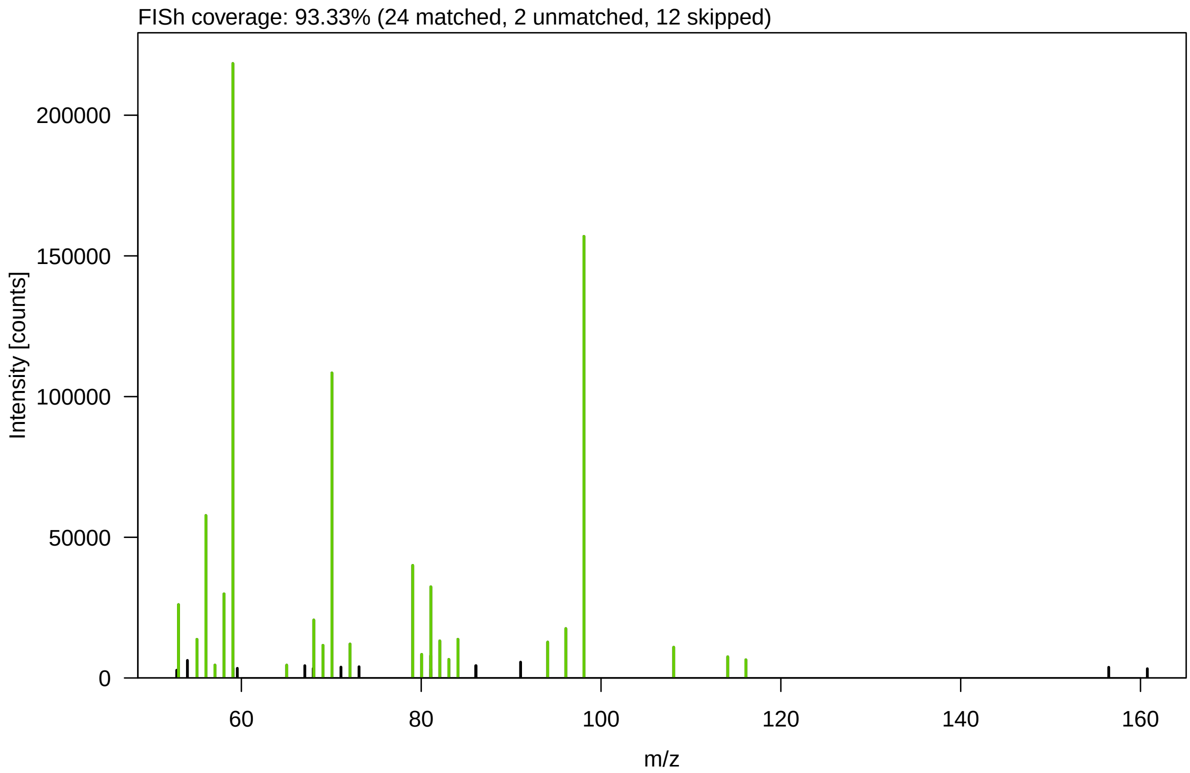

Figure SI-D382: Measured MS2 spectrum. Matching fragments with N-methylpregabalin predicted by FISh Scoring are highlighted in green. Low intensity fragments are not considered and skipped.

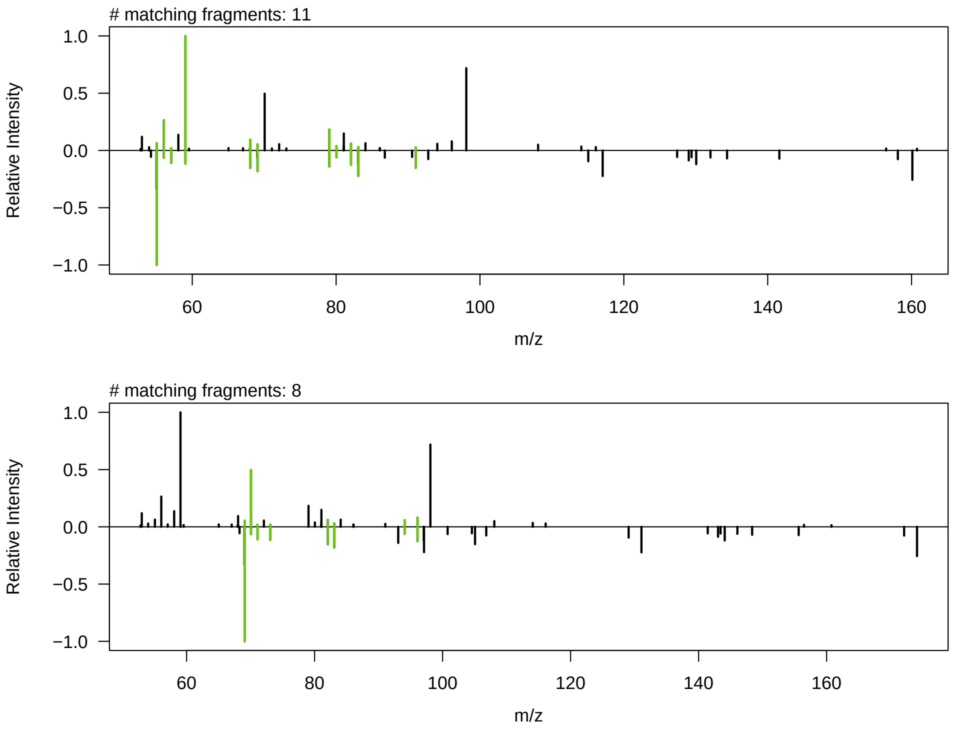

Figure SI-D383: Head to tail plots of N-methylpregabalin and pregabalin. In the bottom plot, the mass spectrum of pregabalin is shifted by the mass difference. Matching fragments are highlighted in green.

Table SI-D188: Molecular network results and retention time prediction of N-methylpregabalin.

Comparison with	Pregabalin
MSn Score	52
Forward coverage	46
Reverse coverage	58
Forward match	12
Reverse match	11
ΔMass [g/mol]	14.0157
Measured retention time [min]	5.5
Predicted $logD_{OW}$ (pH = 2.7)	-1.87
Predicted retention time [min]	12.3
Predicted retention time range (95% confidence interval) [min]	7.7-16.9
Predicted retention time range (99% confidence interval) [min]	6.2-18.4

Table SI-D189: Annotated MS2 spectrum of N-methylpregabalin.

m/z	Relative Intensity	Annotation
52.8055	12.65	
53.0026	119.25	$C_3O + H^+$
53.9976	28.55	
55.0544	62.83	$C_4H_6 + H^+$
56.0498	264.13	$C_3H_5N + H^+$
57.0576	20.95	$C_3H_6N + H^+$
58.0655	136.80	$C_3H_7N + H^+$
59.0494	999.00	$C_3H_6O + H^+$
59.0686	15.21	
59.5473	15.70	
65.0386	20.87	$C_5H_4 + H^+$
67.0544	20.04	$C_5H_6 + H^+$
67.9893	15.36	
68.0495	94.23	$C_4H_5N + H^+$
69.0698	53.10	$C_5H_8 + H^+$
70.0652	495.88	$C_4H_7N + H^+$
71.0728	17.67	$C_4H_8N + H^+$
72.0807	55.18	$C_4H_9N + H^+$
73.0839	18.26	
79.0541	183.08	$C_6H_6 + H^+$
80.0494	38.39	$C_5H_5N + H^+$
81.0570	36.22	$C_5H_6N + H^+$
81.0662	14.47	

Continued on next page

Table SI-D189: Annotated MS2 spectrum of N-methylpregabalin.(Continued)

81.0699	148.27	$C_6H_8 + H^+$
82.0649	60.35	$C_5H_7N + H^+$
83.0728	30.16	$C_5H_8N + H^+$
84.0805	62.90	$C_5H_9N + H^+$
86.0601	19.85	
86.0963	20.10	$C_5H_{11}N + H^+$
91.0540	25.78	$C_7H_6 + H^+$
94.0649	58.35	$C_6H_7N + H^+$
96.0808	80.29	$C_6H_9N + H^+$
98.0964	717.94	$C_6H_{11}N + H^+$
108.0806	50.00	$C_7H_9N + H^+$
114.0911	34.47	$C_6H_{11}NO + H^+$
116.1067	29.58	$C_6H_{13}NO + H^+$
156.4667	17.35	
160.7618	15.03	

A reference standard of N-methylpregabalin was purchased. Figure SI-D384 shows the extracted ion chromatograms of this standard, the sample and the spiked sample. It becomes visible that the suspected compound elutes about five minutes earlier than the reference standard of N-methylpregabalin. The suspected compound is therefore not N-methylpregabalin and the confidence level is decreased to level 4 due to the unequivocal molecular formula.

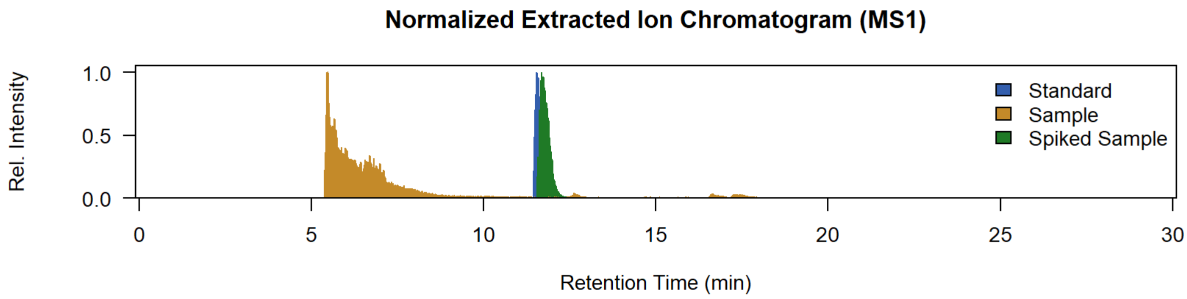

Figure SI-D384: Extracted ion chromatograms of N-methylpregabalin in the reference standard and of the suspected compound in the sample and the spiked sample.

SI-D2.14.18 O-Desarylranolazine

O-Desarylranolazine is a metabolite of ranolazine, which is used as an anti-anginal drug for the treatment of chronic angina.[2] Figure SI-D385 shows the metabolism scheme.

Table SI-D190: Information on identifiers, chemical properties, detection and confidence of identification of O-desaryl ranolazine.

IUPAC Name	2-[4-(2,3-dihydroxypropyl)piperazin-1-yl]-*N*-(2,6-dimethylphenyl)acetamide
Molecular formula	$C_{17}H_{27}N_3O_3$
Monoisotopic mass [g/mol]	321.2052
Adduct	$[M+H]^+$
Retention time [min]	10.9
SMILES	CC1=C(C(=CC=C1)C)NC(=O)CN2CCN(CC2)CC(CO)O
InChI	InChI=1S/C17H27N3O3/c1-13-4-3-5-14(2)17(13)18-16(23)11-20-8-6-19(7-9-20)10-15(22)12-21/h3-5,15,21-22H,6-12H2,1-2H3,(H,18,23)
InChI-Key	LDEJNELBMSFPDJ-UHFFFAOYSA-N
CAS RN	172430-46-5
Metabolite of	Ranolazine
Detection frequency	100% (15/15 samples)
Detected in	Altenrhein, Monday-Friday
	Neugut, Monday-Friday
	Werdhölzli, Monday-Friday
Intensity	E6-E7
Initial confidence level	level 3
Initial confidence score	0.43
Final confidence level	level 1

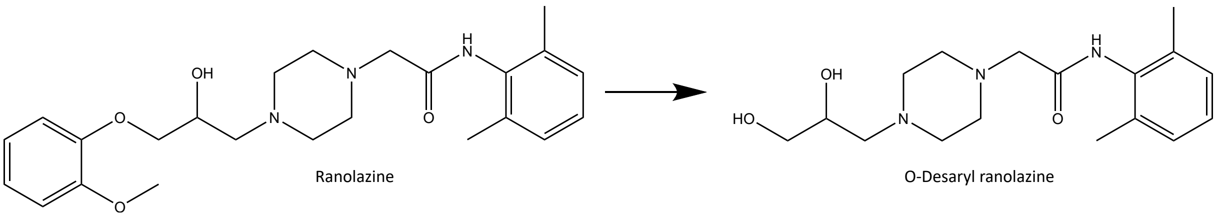

Figure SI-D385: Metabolism of ranolazine to O-desarylranolazine.

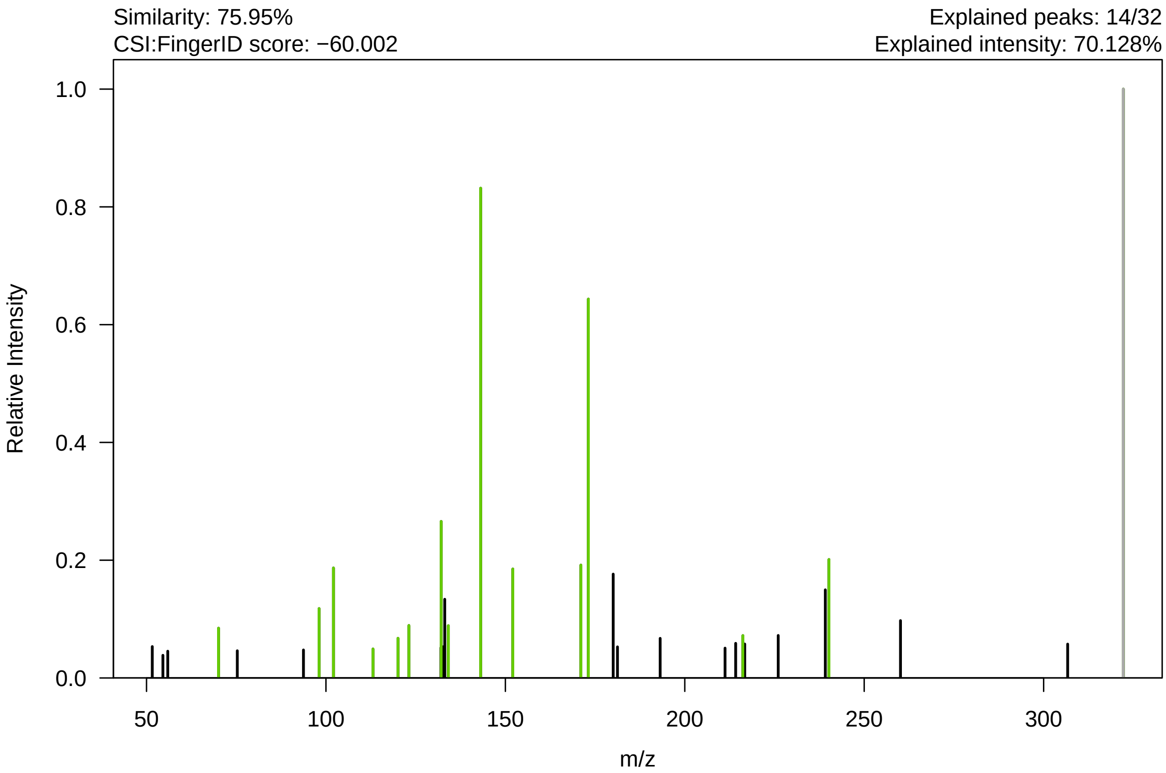

Figure SI-D386: Measured MS2 spectrum. Matching fragments with O-desarylranolazine predicted by SIRIUS/CSI:FingerID are highlighted in green. The molecular ion in gray is not considered.

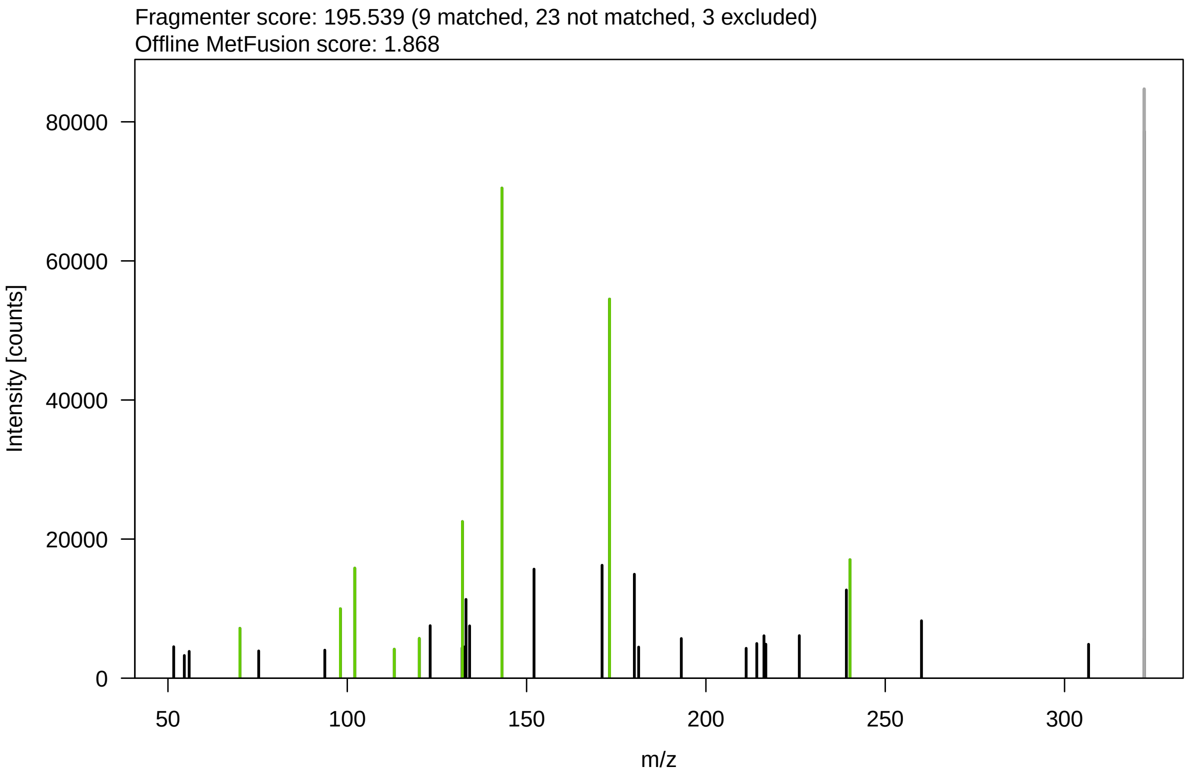

Figure SI-D387: Measured MS2 spectrum. Matching fragments with O-desarylranolazine predicted by MetFrag are highlighted in green. The molecular ion in gray is not considered.

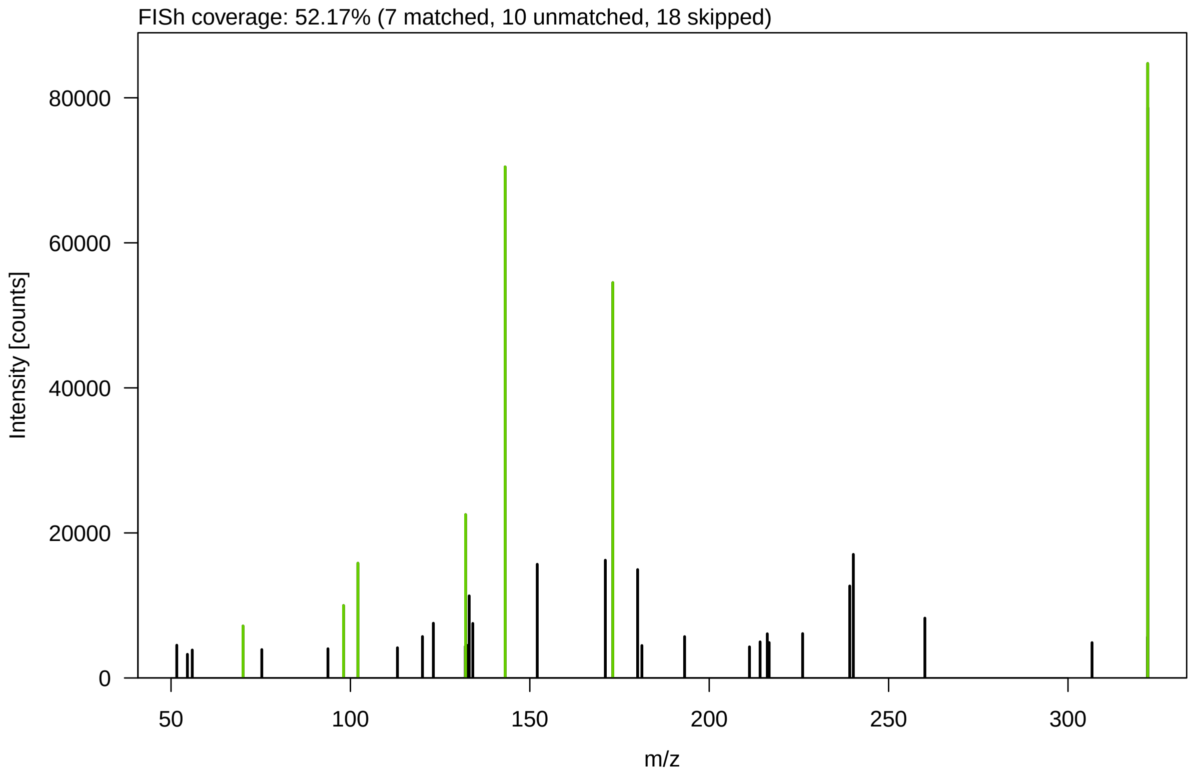

Figure SI-D388: Measured MS2 spectrum. Matching fragments with O-desarylranolazine predicted by FISh Scoring are highlighted in green. Low intensity fragments are not considered and skipped.

Table SI-D191: Retention time prediction of O-desarylranolazine.

Measured retention time [min]	10.9
Predicted logD_{OW} (pH = 2.7)	-2.79
Predicted retention time [min]	11.1
Predicted retention time range (95% confidence interval) [min]	6.5-15.7
Predicted retention time range (99% confidence interval) [min]	5.0-17.2

Table SI-D192: Annotated MS2 spectrum of O-desarylranolazine.

m/z	Relative Intensity	Annotation
51.6036	53.26	
54.5655	38.45	
55.9163	45.43	
70.0652	84.48	$C_4H_7N + H^+$
75.2994	46.14	
93.7335	47.52	
98.0839	117.74	$C_5H_9N_2 + H^+$
102.0913	186.60	$C_5H_{11}NO + H^+$

Continued on next page

Table SI-D192: Annotated MS2 spectrum of O-desarylranolazine.(Continued)

113.1072	49.14	$C_6H_{12}N_2 + H^+$
120.0804	67.35	$C_8H_9N + H^+$
123.1172	88.99	$C_9H_{14} + H^+$
132.0239	51.47	
132.1019	265.55	$C_6H_{13}NO_2 + H^+$
132.6389	52.97	
133.1054	133.53	
134.1081	88.69	$C_{10}H_{13} + H^+$
143.1178	830.90	$C_7H_{14}N_2O + H^+$
152.0705	184.85	$C_8H_9NO_2 + H^+$
171.0440	191.44	$C_{11}H_6O_2 + H^+$
173.1282	642.73	$C_8H_{16}N_2O_2 + H^+$
180.0496	176.19	
181.2403	52.74	
193.1385	67.22	
211.2193	50.60	
214.1817	58.74	
216.1868	71.89	$C_{16}H_{23} + H^+$
216.7014	57.61	
226.0383	72.11	
239.1684	149.53	
240.1703	200.93	$C_{12}H_{21}N_3O_2 + H^+$
260.1116	97.32	
306.6987	57.44	
322.1693	66.44	
322.2116	999.00	$C_{17}H_{27}N_3O_3 + H^+$
322.2487	926.96	

A reference standard of O-desarylranolazine was purchased. Figure SI-D389 shows the extracted ion chromatograms of this standard, the sample and the spiked sample, as well as a head to tail plot of the MS2 spectra of the standard and the sample. In addition, the most intense MS2 fragments in the standard are displayed. It becomes visible that the retention times of the sample and the spiked sample are identical. The spectra similarity score between sample and standard is only equal to 0.372, which is a result of the low quality and noisy MS2 spectrum of o-desarylranolazine in the sample, resulting from a low intensity signal. Nonetheless, the most intense MS2 fragment in the sample can be explained. Correspondingly, the suspected compound is confirmed as O-desarylranolazine and the confidence level is increased to level 1.

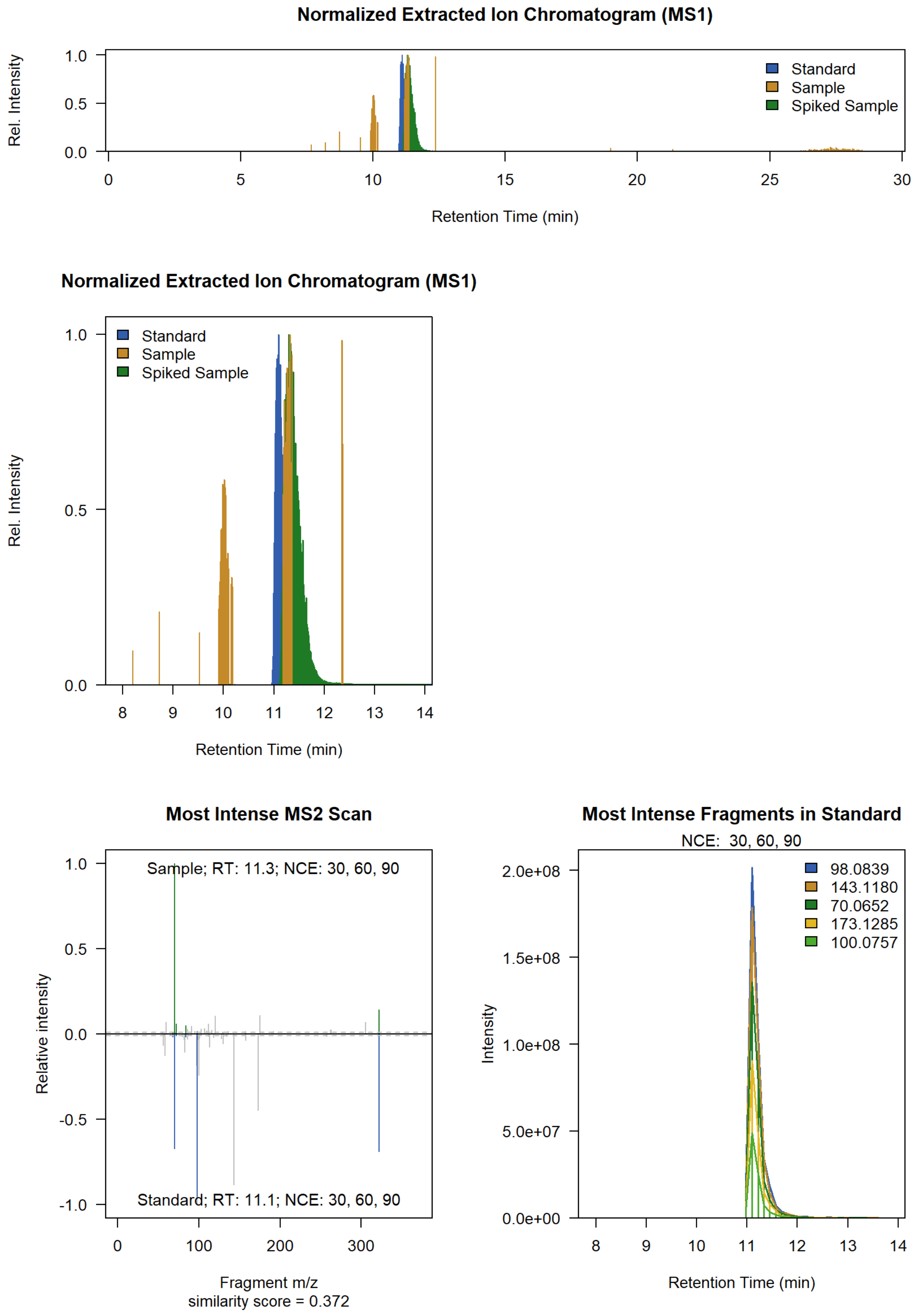

Figure SI-D389: Extracted ion chromatograms of O-desarylranolazine in the reference standard, the sample and the spiked sample, as well as MS2 head to tail plot and most intense MS2 fragments in standard.

SI-D2.14.19 Phenylethylmalonamide (PEMA)

Phenylethylmalonamide (PEMA) is a metabolite of primidone, which is an antiepileptic used to treat grand mal, psychomotor and focal epileptic seizures.[2] Figure SI-D390 shows the metabolism scheme.

Table SI-D193: Information on identifiers, chemical properties, detection and confidence of identification of phenylethylmalonamide.

IUPAC Name	2-ethyl-2-phenylpropanediamide
Molecular formula	$C_{11}H_{14}N_2O_2$
Monoisotopic mass [g/mol]	206.1055
Adduct	$[M+H]^+$
Retention time [min]	13.0
SMILES	CCC(C1=CC=CC=C1)(C(=O)N)C(=O)N
InChI	InChI=1S/C11H14N2O2/c1-2-11(9(12)14,10(13)15)8-6-4-3-5-7-8/h3-7H,2H2,1H3,(H2,12,14)(H2,13,15)
InChI-Key	JFZHPFOXAAIUMB-UHFFFAOYSA-N
CAS RN	7206-76-0
Metabolite of	Primidone
Detection frequency	100% (15/15 samples)
Detected in	Altenrhein, Monday-Friday<br>Neugut, Monday-Friday<br>Werdhölzli, Monday-Friday
Intensity	E6
Initial confidence level	level 2a
Initial confidence score	0.57
Final confidence level	level 1

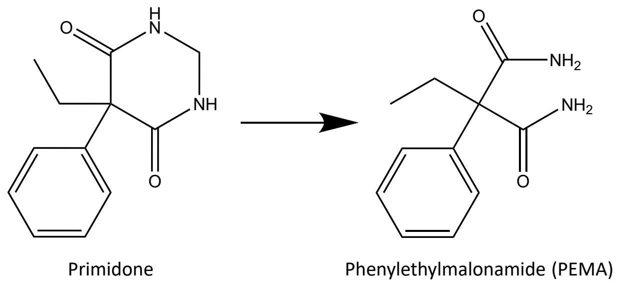

Figure SI-D390: Metabolism of primidone to phenylethylmalonamide.

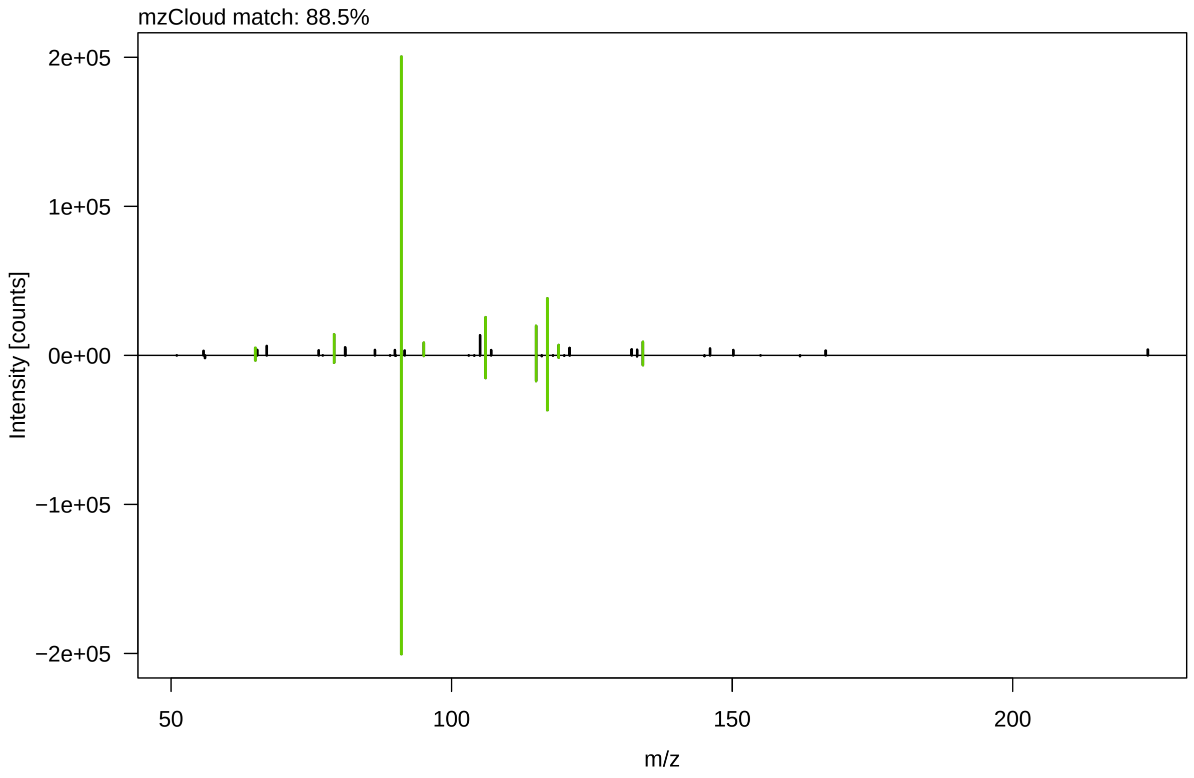

Figure SI-D391: Head to tail plot of measured MS2 spectrum against mzCloud library spectrum of phenylethylmalonamide. Matching fragments are highlighted in green.

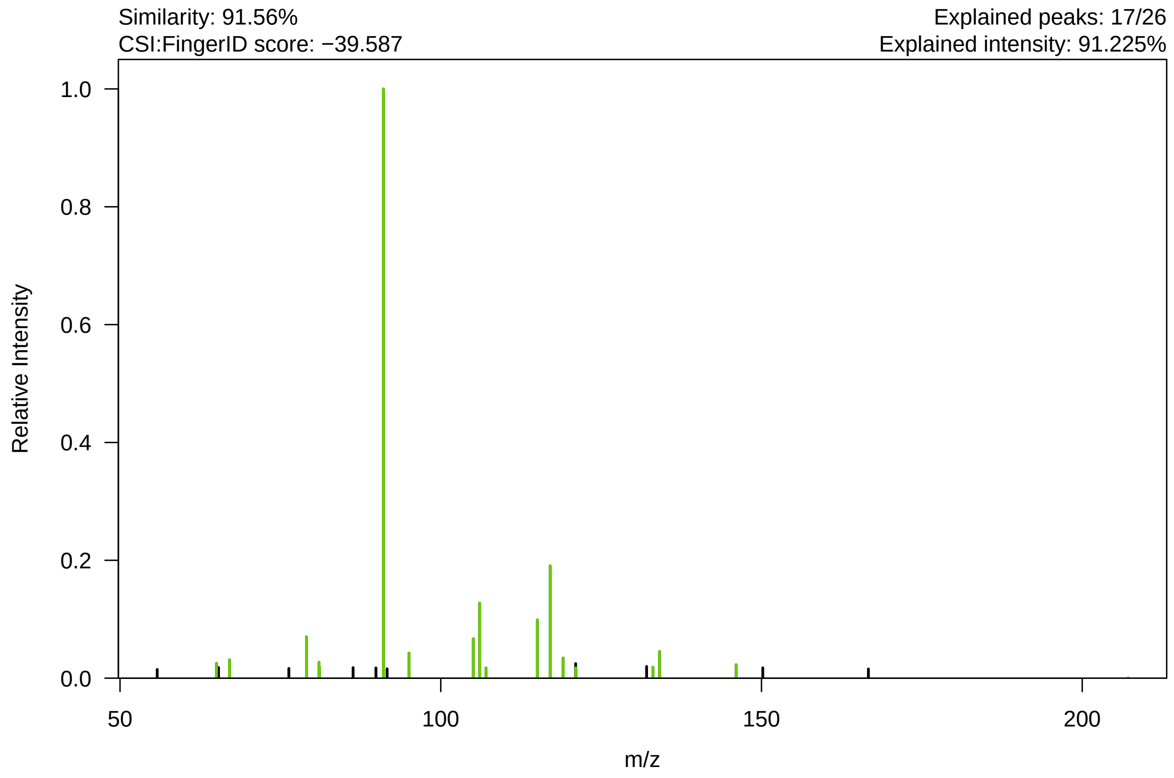

Figure SI-D392: Measured MS2 spectrum. Matching fragments with phenylethylmalonamide predicted by SIRIUS/CSI:FingerID are highlighted in green. The molecular ion in gray is not considered.

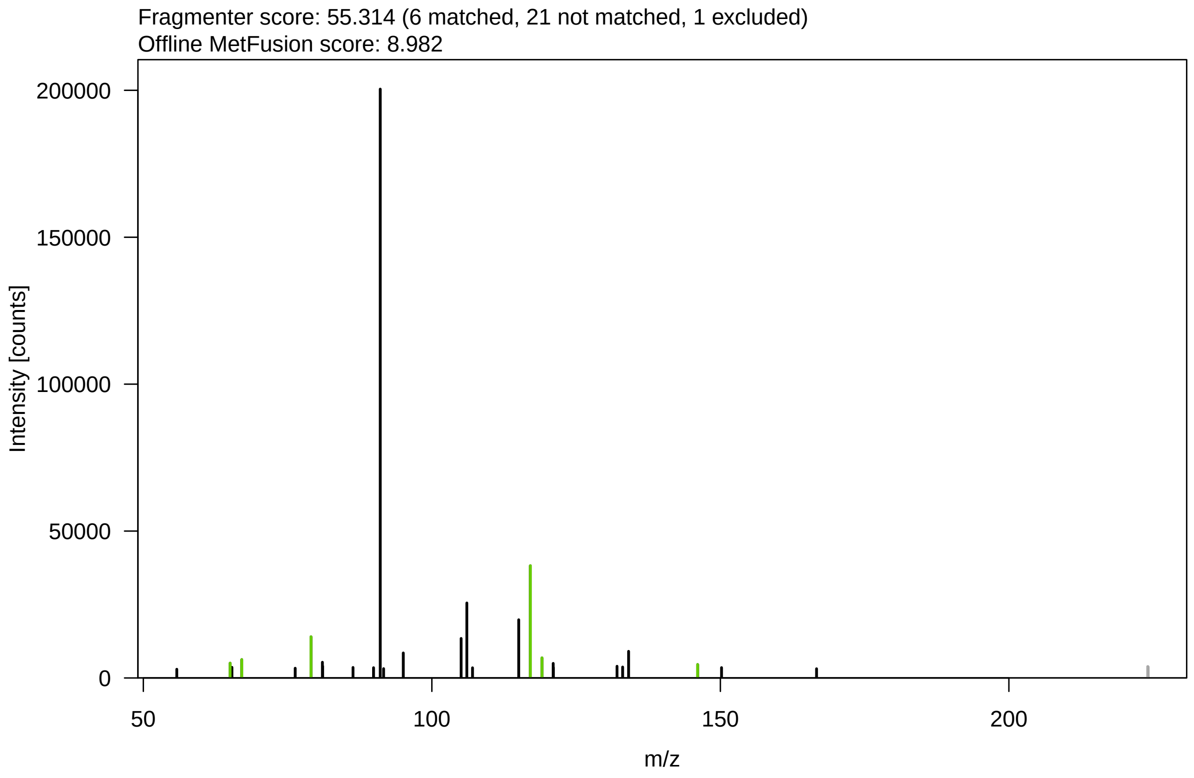

Figure SI-D393: Measured MS2 spectrum. Matching fragments with phenylethylmalonamide predicted by MetFrag are highlighted in green. The molecular ion in gray is not considered.

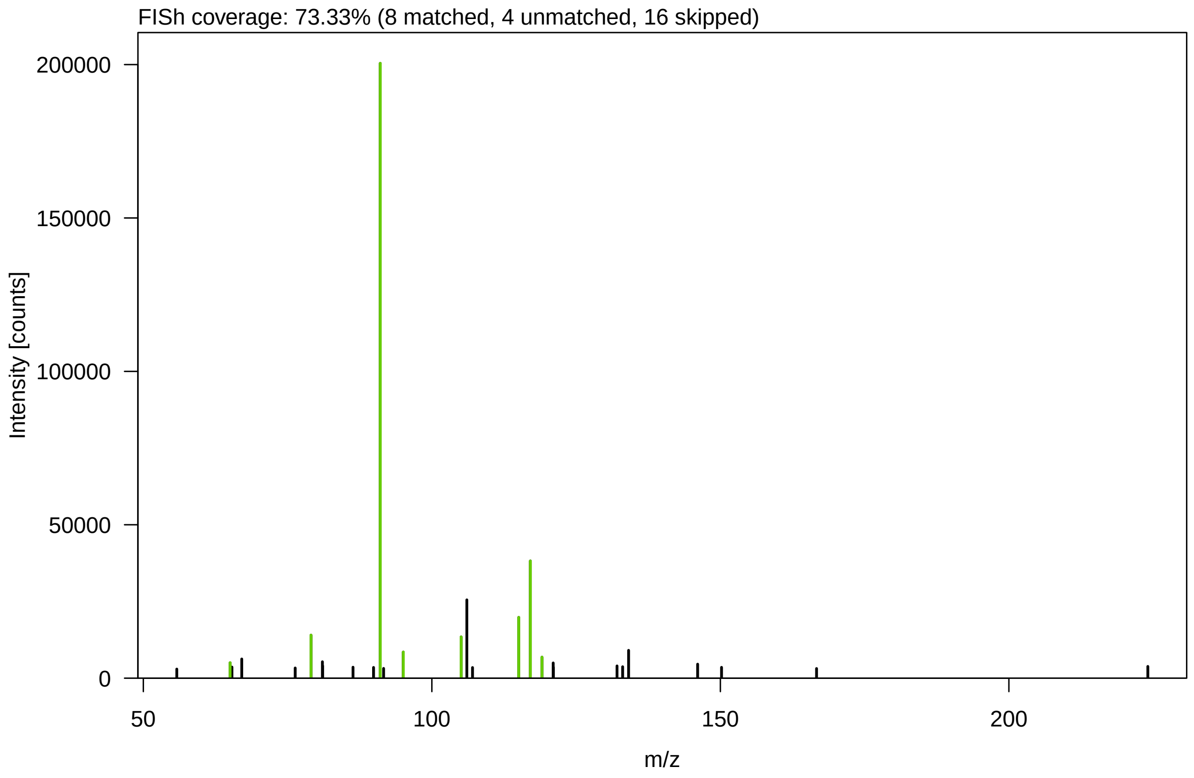

Figure SI-D394: Measured MS2 spectrum. Matching fragments with phenylethylmalonamide predicted by FISh Scoring are highlighted in green. Low intensity fragments are not considered and skipped.

Table SI-D194: Retention time prediction of phenylethylmalonamide.

Measured retention time [min]	13.0
Predicted logD_{OW} (pH = 2.7)	0.73
Predicted retention time [min]	15.7
Predicted retention time range (95% confidence interval) [min]	11.1-20.3
Predicted retention time range (99% confidence interval) [min]	9.6-21.7

Table SI-D195: Annotated MS2 spectrum of phenylethylmalonamide.

m/z	Relative Intensity	Annotation
55.7964	14.78	
65.0387	25.13	$C_5H_4 + H^+$
65.3529	18.05	
67.0544	31.18	$C_5H_6 + H^+$
76.3161	16.54	
79.0541	69.92	$C_6H_6 + H^+$
81.0333	26.66	$C_5H_4O + H^+$
81.0701	19.96	$C_6H_8 + H^+$
86.3364	17.83	
89.8945	17.42	
91.0543	999.00	$C_7H_6 + H^+$
91.6356	15.83	
95.0490	42.43	$C_6H_6O + H^+$
105.0698	67.05	$C_8H_8 + H^+$
106.0652	127.23	$C_7H_7N + H^+$
107.0487	17.27	$C_7H_6O + H^+$
115.0544	98.76	$C_9H_6 + H^+$
117.0698	190.44	$C_9H_8 + H^+$
119.0855	34.18	$C_9H_{10} + H^+$
121.0286	24.74	
121.0646	17.52	$C_8H_8O + H^+$
132.0895	19.87	
133.0651	18.56	$C_9H_8O + H^+$
134.0962	45.19	$C_9H_{11}N + H^+$
146.0604	22.77	$C_9H_7NO + H^+$
150.2011	17.56	
166.6686	15.65	
224.0782	19.07	

A reference standard of phenylethylmalonamide was purchased. Figure SI-D395 shows the extracted ion chromatograms of this standard, the sample and the spiked sample, as well as a head to tail plot of the MS2 spectra of the standard and the sample. In addition, the most intense MS2 fragments in the sample and in the standard are displayed. It becomes visible that the signal of phenylethylmalonamide in the unspiked sample is didtributed over the entire retention time range. Nonetheless, the maximal intensity is found ar the retention of the standard and the spiked sample. Moreover, the spectra similarity score between sample and standard is equal to 0.919. The majority of the MS2 fragments in the sample can be explained by the reference standard. It can therefore be concluded that the suspected compound is indeed phenylethylmalonamide. Correspondingly, the identification confidence can be increased to level 1.

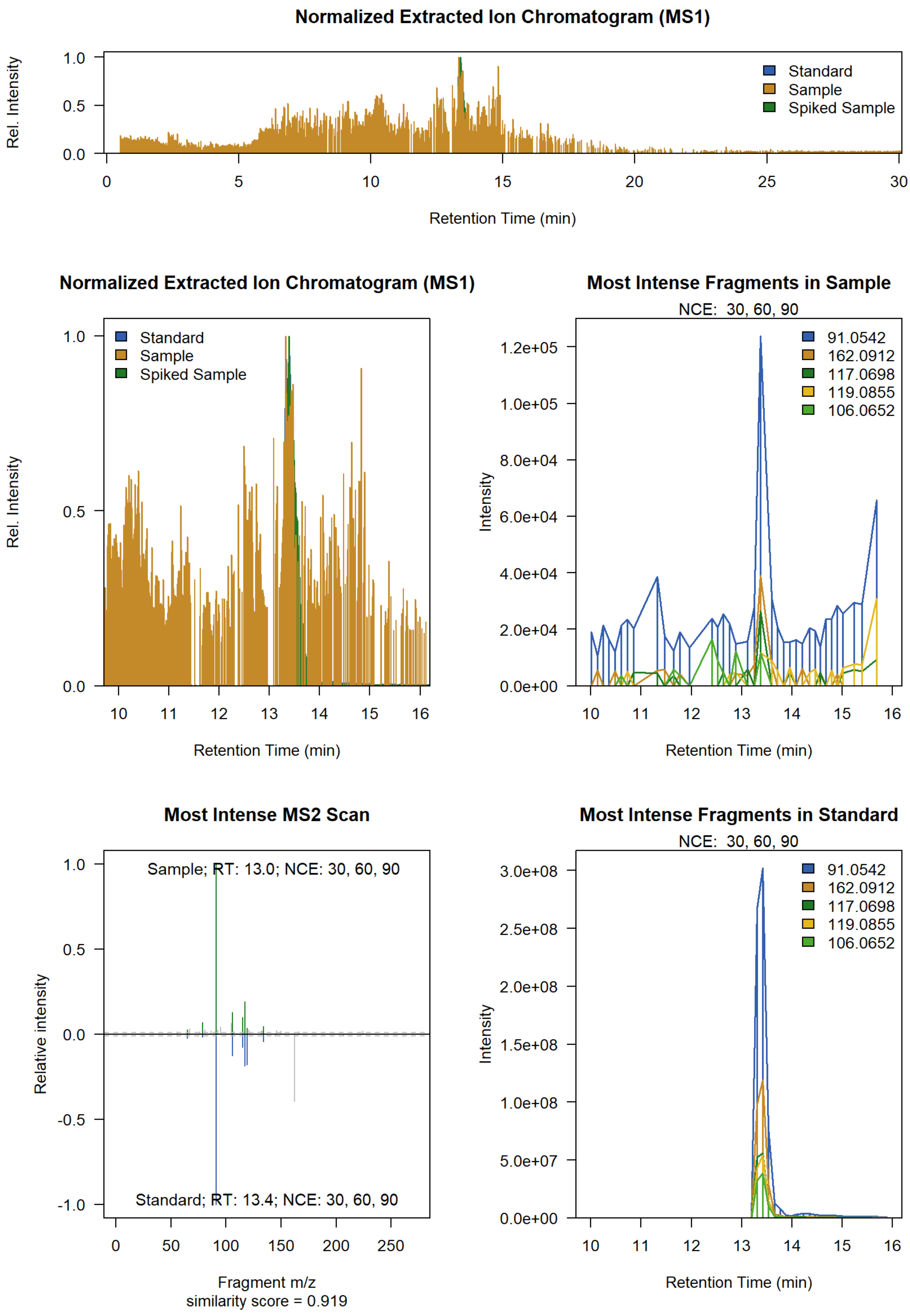

Figure SI-D395: Extracted ion chromatograms of phenylethylmalonamide in the reference standard, the sample and the spiked sample, as well as MS2 head to tail plot and most intense MS2 fragments in standard and sample.

SI-D2.14.20 Sacubitrilat (LBQ-657)

Sacubitrilat is a metabolite of sacubitril, a neprilysin inhibitor used in combination with valsartan to reduce the risk of cardiovascular events in patients with chronic heart failure.[2] Figure SI-D396 shows the metabolism scheme.

Table SI-D196: Information on identifiers, chemical properties, detection and confidence of identification of sacubitrilat.

IUPAC Name	(2*R*,4*S*)-4-(3-carboxypropanoylamino)-2-methyl-5-(4-phenylphenyl)pentanoic acid
Molecular formula	$C_{22}H_{25}NO_5$
Monoisotopic mass [g/mol]	383.1733
Adduct	$[M-H]^-$
Retention time [min]	18.9
SMILES	C[C@H](C[C@@H](CC1=CC=C(C=C1)C2=CC=CC=C2)NC(=O)CCC(=O)O)C(=O)O
InChI	InChI=1S/C22H25NO5/c1-15(22(27)28)13-19(23-20(24)11-12-21(25)26)14-16-7-9-18(10-8-16)17-5-3-2-4-6-17/h2-10,15,19H,11-14H2,1H3,(H,23,24)(H,25,26)(H,27,28)/t15-,19+/m1/s1
InChI-Key	DOBNVUFHFMVMDB-BEFAXECRSA-N
CAS RN	149709-44-4
Metabolite of	Sacubitril
Detection frequency	100% (15/15 samples)
Detected in	Altenrhein, Monday-Friday<br>Neugut, Monday-Friday<br>Werdhölzli, Monday-Friday
Intensity	E7
Initial confidence level	level 3
Initial confidence score	0.33
Final confidence level	level 1

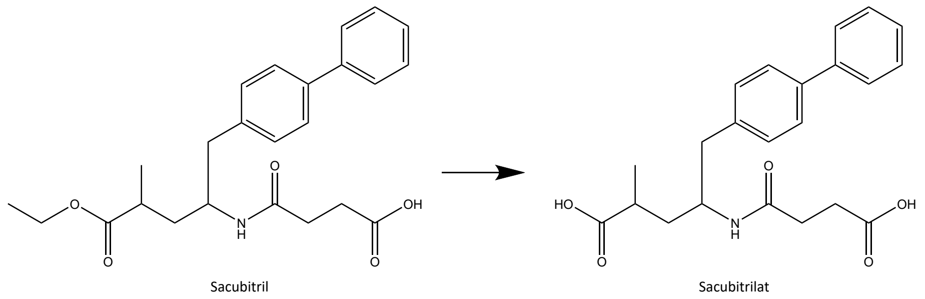

Figure SI-D396: Metabolism of sacubitril to sacubitrilat.

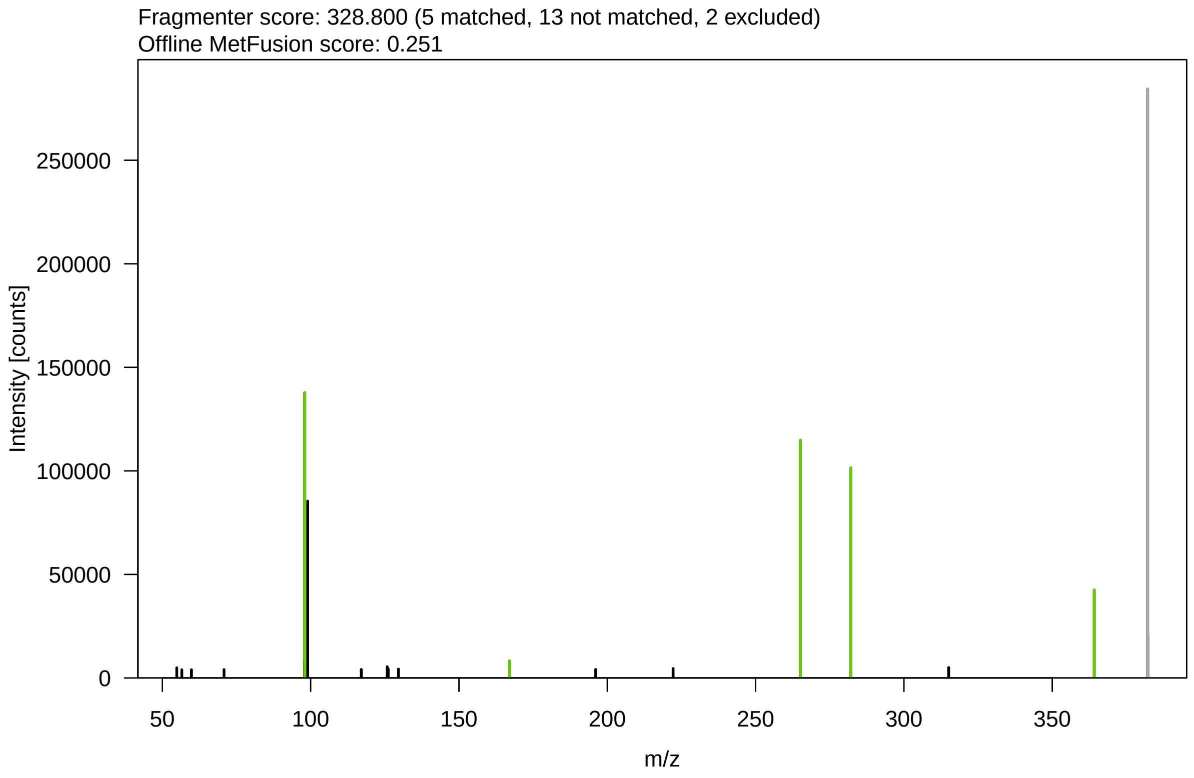

Figure SI-D397: Measured MS2 spectrum. Matching fragments with sacubitrilat predicted by SIRIUS/CSI:FingerID are highlighted in green. The molecular ion in gray is not considered.

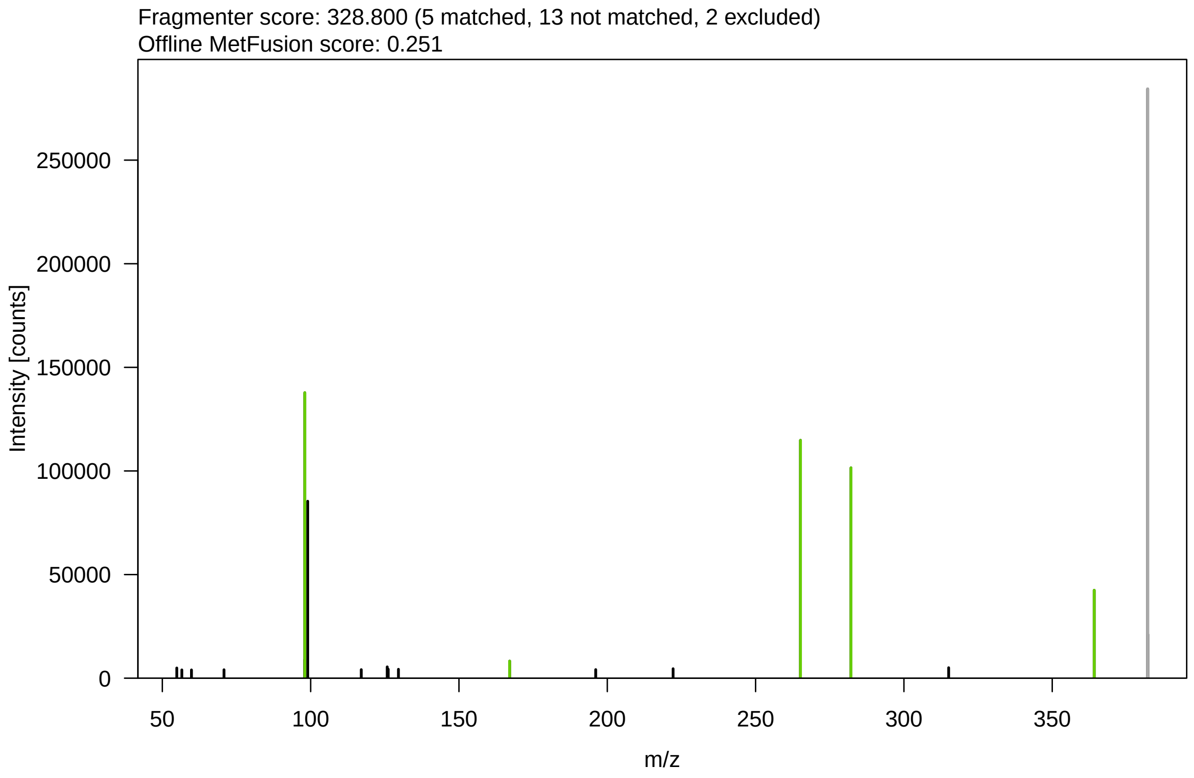

Figure SI-D398: Measured MS2 spectrum. Matching fragments with sacubitrilat predicted by MetFrag are highlighted in green. The molecular ion in gray is not considered.

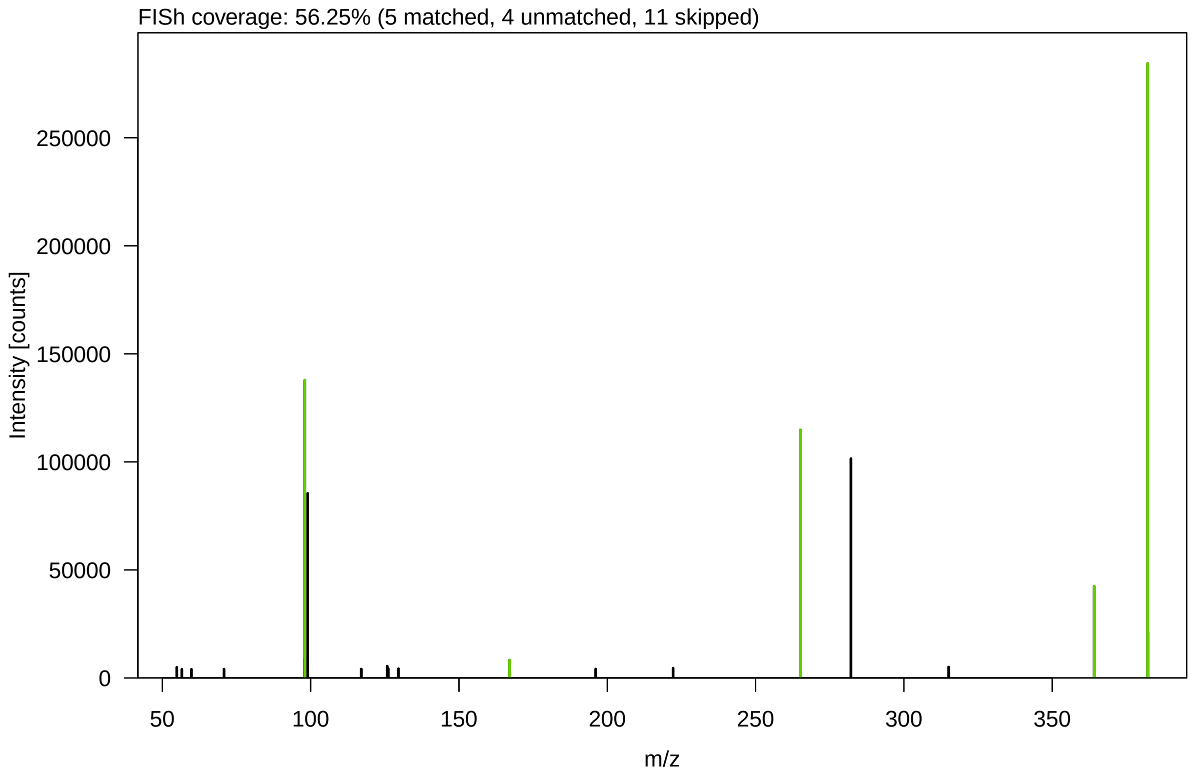

Figure SI-D399: Measured MS2 spectrum. Matching fragments with sacubitrilat predicted by FISh Scoring are highlighted in green. Low intensity fragments are not considered and skipped.

Table SI-D197: Retention time prediction of sacubitrilat.

Measured retention time [min]	18.9
Predicted logD_{OW} (pH = 4.8)	2.17
Predicted retention time [min]	18.3
Predicted retention time range (95% confidence interval) [min]	11.2-25.4
Predicted retention time range (99% confidence interval) [min]	8.9-27.7

Table SI-D198: Annotated MS2 spectrum of sacubitrilat.

m/z	Relative Intensity	Annotation
54.8706	17.27	
56.5680	14.00	
59.8220	13.95	
70.7831	14.26	
98.0190	31.42	
98.0249	484.04	$C_4H_5NO_2 - H^-$
99.0089	300.01	
117.0619	14.43	
125.8116	19.17	
126.1434	15.19	
129.5957	15.05	
167.0862	28.99	$C_{13}H_{12} - H^-$
196.1008	14.51	
222.1859	15.98	
265.1233	403.31	$C_{18}H_{18}O_2 - H^-$
282.1498	356.58	$C_{18}H_{21}NO_2 - H^-$
315.0809	17.70	
364.1553	149.10	$C_{22}H_{23}NO_4 - H^-$
382.1662	999.00	$C_{22}H_{25}NO_5 - H^-$
382.2252	73.98	

A reference standard of sacubitrilat was purchased. Figure SI-D400 shows the extracted ion chromatograms of this standard, the sample and the spiked sample, as well as a head to tail plot of the MS2 spectra of the standard and the sample. In addition, the most intense MS2 fragments in the sample and in the standard are displayed. It becomes visible that the retention times of the sample and the spiked sample are identical and the spectra similarity score between sample and standard is equal to 0.618. The four most intense MS2 fragments in the sample can be explained by the reference standard. It can therefore be concluded that the suspected compound is indeed sacubitrilat. Correspondingly, the identification confidence can be increased to level 1.

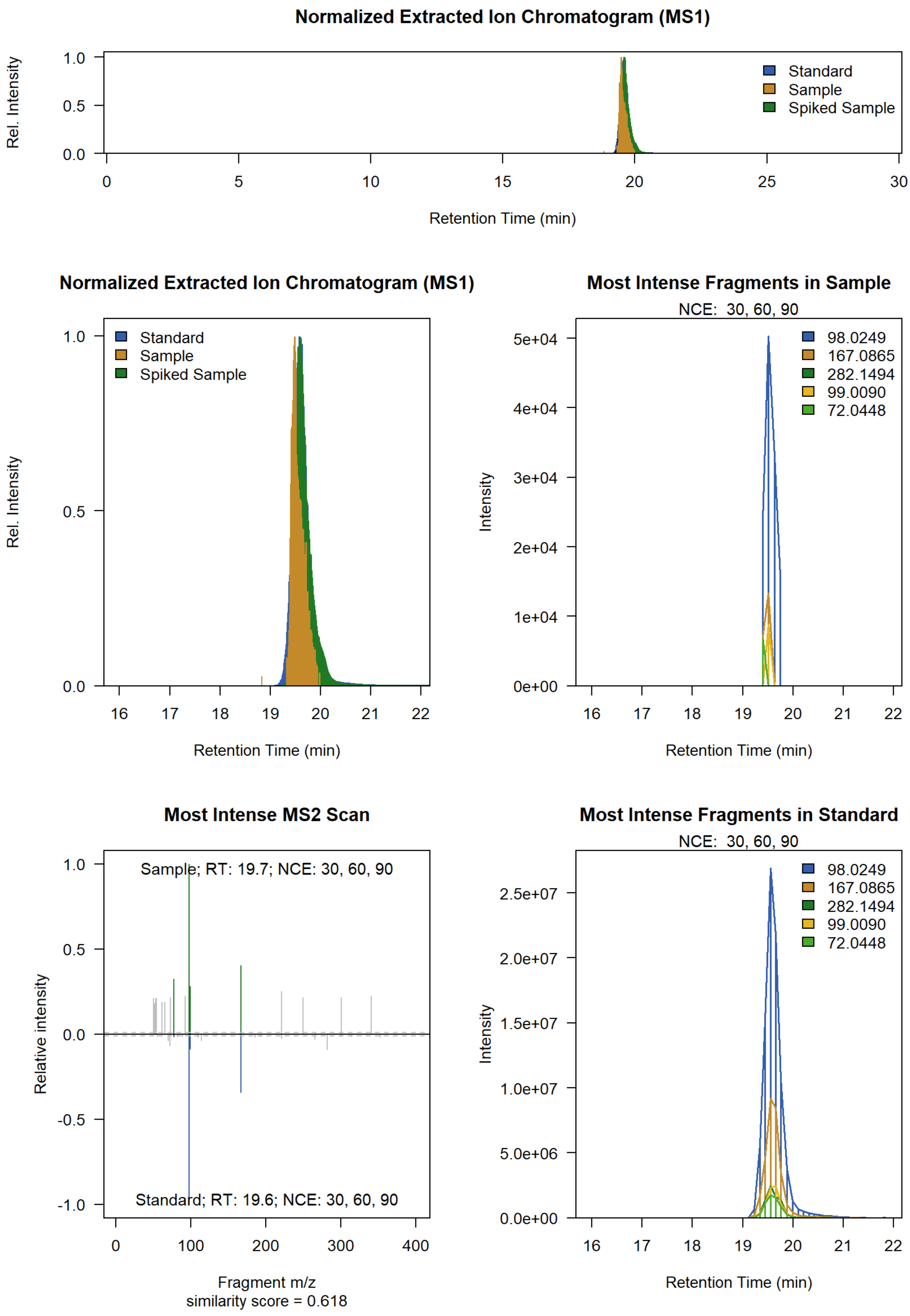

Figure SI-D400: Extracted ion chromatograms of sacubitrilat in the reference standard, the sample and the spiked sample, as well as MS2 head to tail plot and most intense MS2 fragments in standard and sample.

SI-D2.14.21 Succinic acid

Succinic acid is a metabolite of sodium oxybate, which is a central nervous system depressant used to treat cataplexy and excessive daytime sleepiness associated with narcolepsy.[2] Figure SI-D401 shows the metabolism scheme. Succinic acid is also an endogenous metabolite in cellular respiration.[21]

Table SI-D199: Information on identifiers, chemical properties, detection and confidence of identification of succinic acid.

IUPAC Name	butanedioic acid
Molecular formula	$C_4H_6O_4$
Monoisotopic mass [g/mol]	118.0266
Adduct	$[M-H]^-$
Retention time [min]	4.1
SMILES	C(CC(=O)O)C(=O)O
InChI	InChI=1S/C4H6O4/c5-3(6)1-2-4(7)8/h1-2H2,(H,5,6)(H,7,8)
InChI-Key	KDYFGRWQOYBRFD-UHFFFAOYSA-N
CAS RN	110-15-6
Metabolite of	Sodium oxybate
Detection frequency	100% (15/15 samples)
Detected in	Altenrhein, Monday-Friday
	Neugut, Monday-Friday
	Werdhölzli, Monday-Friday
Intensity	E6-E7
Initial confidence level	level 2a
Initial confidence score	0.52
Final confidence level	level 2a

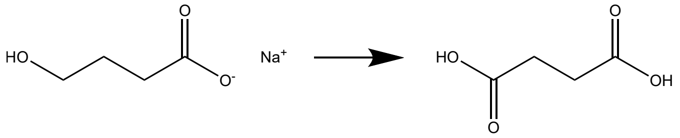

Figure SI-D401: Metabolism of sodium oxybate to succinic acid.

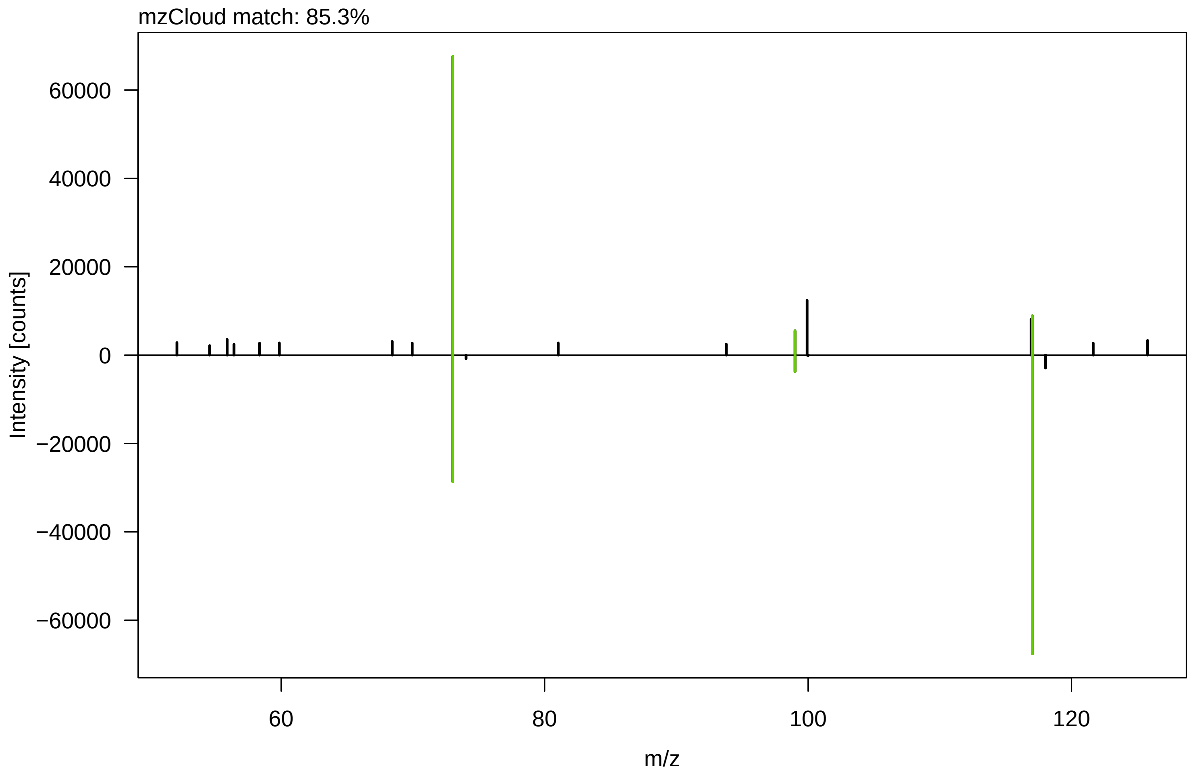

Figure SI-D402: Head to tail plot of measured MS2 spectrum against mzCloud library spectrum of succinic acid. Matching fragments are highlighted in green.

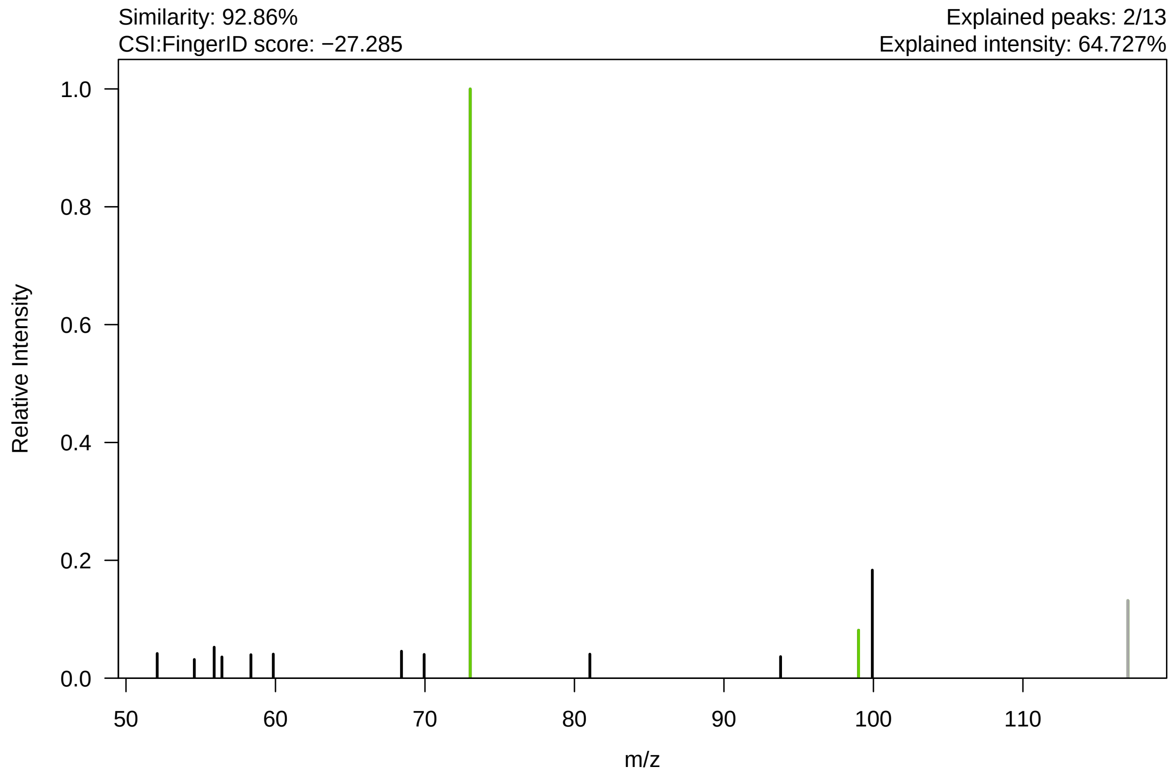

Figure SI-D403: Measured MS2 spectrum. Matching fragments with succinic acid predicted by SIRIUS/CSI:FingerID are highlighted in green. The molecular ion in gray is not considered.

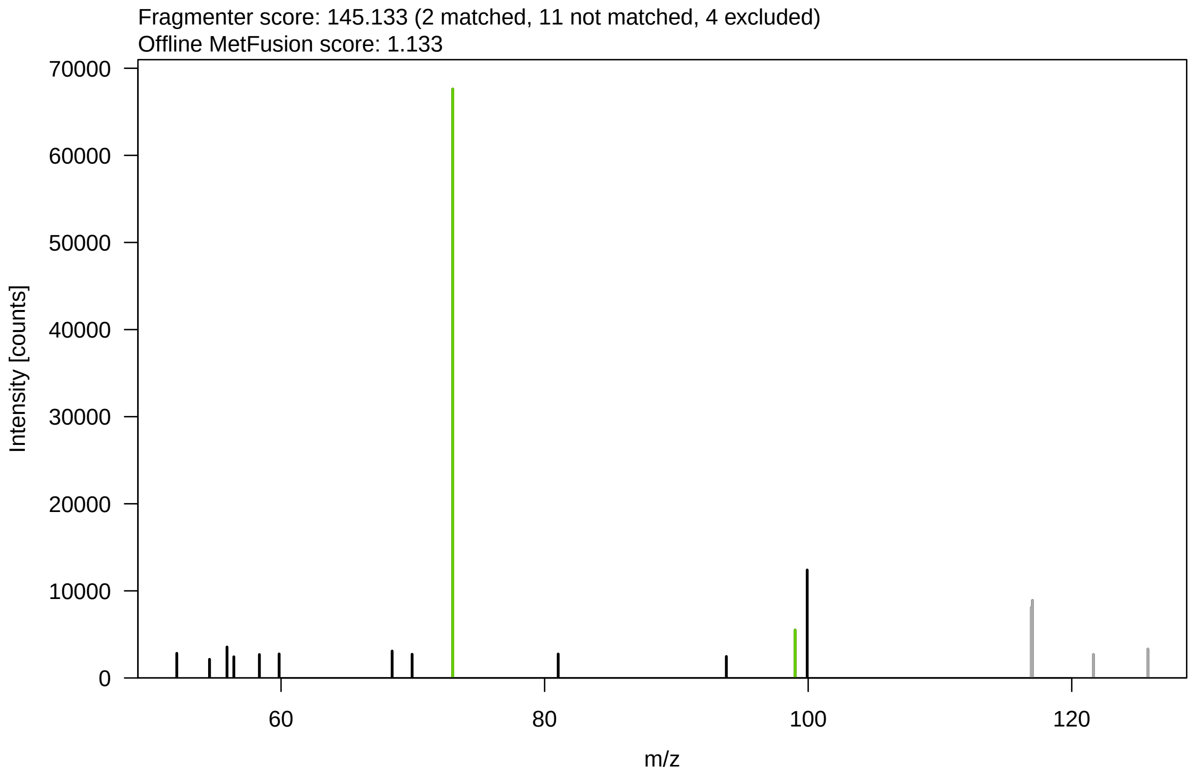

Figure SI-D404: Measured MS2 spectrum. Matching fragments with succinic acid predicted by MetFrag are highlighted in green. Fragments in gray were not considered.

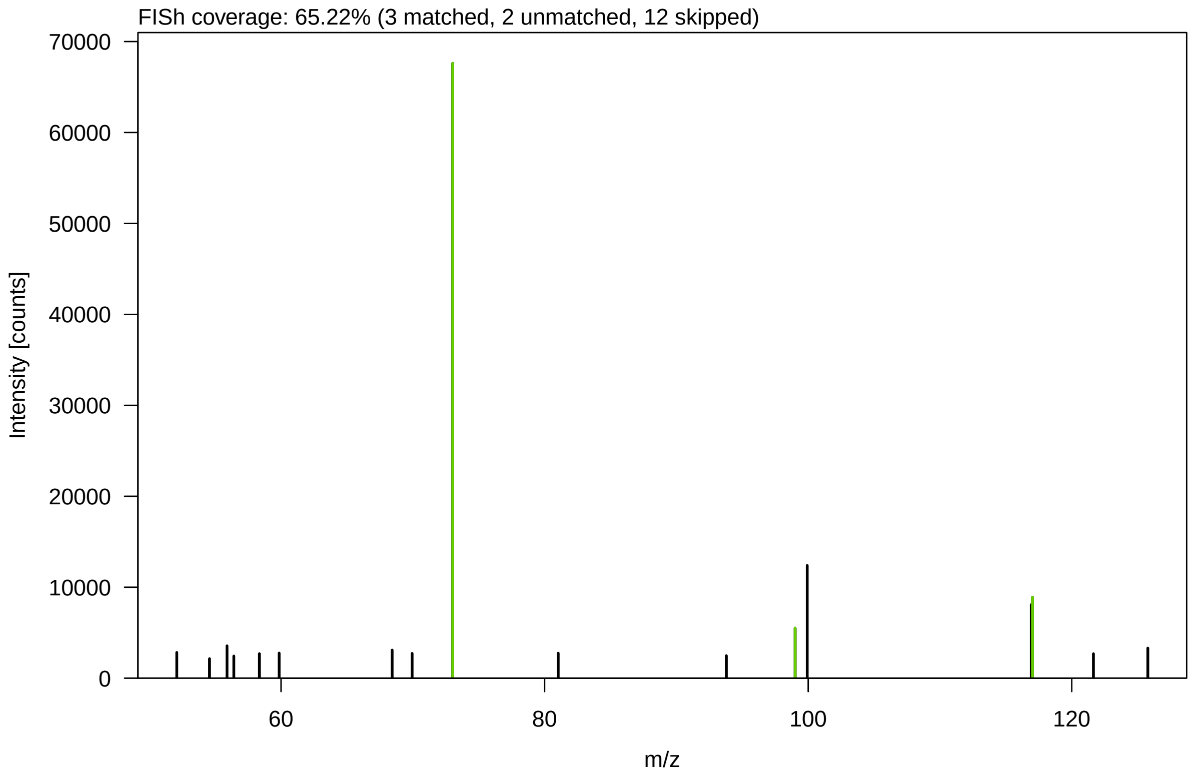

Figure SI-D405: Measured MS2 spectrum. Matching fragments with succinic acid predicted by FISh Scoring are highlighted in green. Low intensity fragments are not considered and skipped.

Table SI-D200: Retention time prediction of succinic acid.

Measured retention time [min]	4.1
Predicted $logD_{OW}$ (pH = 4.8)	-1.66
Predicted retention time [min]	11.4
Predicted retention time range (95% confidence interval) [min]	4.3-18.6
Predicted retention time range (99% confidence interval) [min]	2.0-20.9

Table SI-D201: Annotated MS2 spectrum of succinic acid.

m/z	Relative Intensity	Annotation
52.0883	41.80	
54.5723	31.64	
55.8991	52.50	
56.4169	35.97	
58.3571	39.76	
59.8542	40.80	
68.4293	45.66	
69.9451	40.24	
73.0289	999.00	$C_3H_6O_2 - H^-$
81.0301	40.68	
93.7892	36.50	
99.0090	81.31	$C_4H_4O_3 - H^-$
99.9258	183.18	
116.9287	119.39	
117.0194	131.48	$C_4H_6O_4 - H^-$
121.6452	39.63	
125.7728	48.88	

A reference standard of succinic acid was purchased. Figure SI-D406 shows the extracted ion chromatograms of this standard, the sample and the spiked sample. It becomes visible that the suspected compound elutes at the same time than the reference standard of succinic acid. Despite being on the inclusion list, no MS2 spectra of succinic acid in the sample or the standard were acquired in the second measurement. Correspondingly, the suspected compound can neither be confirmed as succinic acid nor rejected. As a consequence, the confidence level remains level 2a due to the library match obtained during the first measurement.

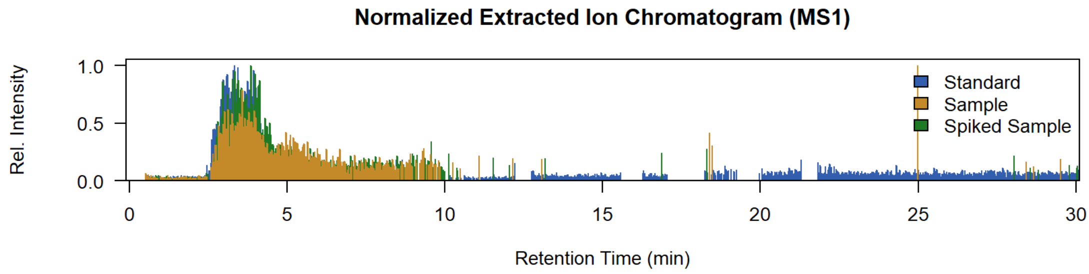

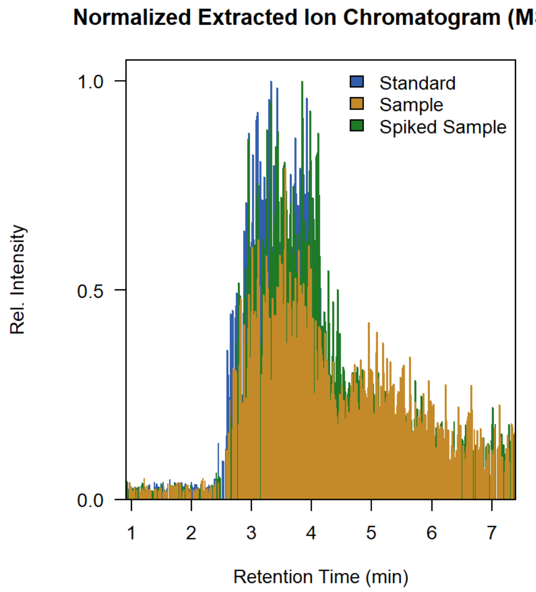

Figure SI-D406: Extracted ion chromatograms of succinic acid in the reference standard and in the sample and the spiked sample.

SI-D2.14.22 Valeryl-4-Hydroxyvalsartan

Valeryl-4-hydroxyvalsartan is a metabolite of valsartan. Valsartan is an aniotensin-receptor blocker used to treat hypertension.[2] Figure SI-D407 shows the metabolism scheme and Figure SI-D408 the valsartan cluster.

Table SI-D202: Information on identifiers, chemical properties, detection and confidence of identification of valeryl-4-hydroxyvalsartan.

IUPAC Name	(2*S*)-2-[4-hydroxypentanoyl-[[4-[2-(2*H*-tetrazol-5-yl)phenyl]phenyl]methyl]amino]-3-methylbutanoic acid
Molecular formula	$C_{24}H_{29}N_5O_4$
Monoisotopic mass [g/mol]	451.2220
Adduct	$[M+H]^+$
Retention time [min]	18.4
SMILES	CC(C)[C@@H](C(=O)O)N(CC1=CC=C(C=C1)C2=CC=CC=C2C3=NNN=N3)C(=O)CCC(C)O
InChI	InChI=1S/C24H29N5O4/c1-15(2)22(24(32)33)29(21(31)13-8-16(3)30)14-17-9-11-18(12-10-17)19-6-4-5-7-20(19)23-25-27-28-26-23/h4-7,9-12,15-16,22,30H,8,13-14H2,1-3H3,(H,32,33)(H,25,26,27,28)/t16?,22-/m0/s1
InChI-Key	ICSQZMPILLPFKC-XLDIYJRPSA-N
CAS RN	188259-69-0
Metabolite of	Losartan
Detection frequency	100% (15/15 samples)
Detected in	Altenrhein, Monday-Friday<br>Neugut, Monday-Friday<br>Werdhölzli, Monday-Friday
Intensity	E7
Initial confidence level	level 3
Initial confidence score	0.45
Final confidence level	level 1

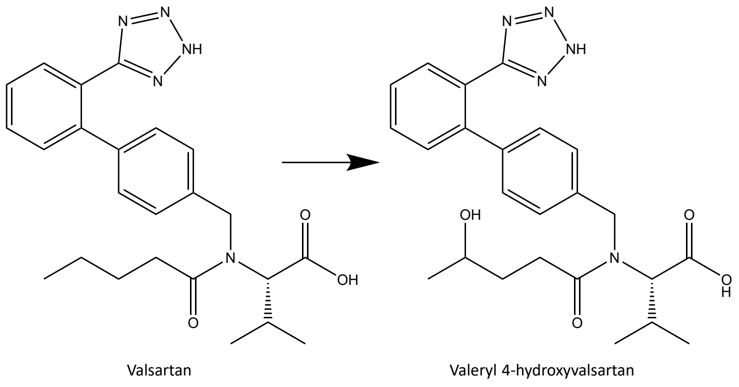

Figure SI-D407: Metabolism of losartan to valeryl-4-hydroxyvalsartan.

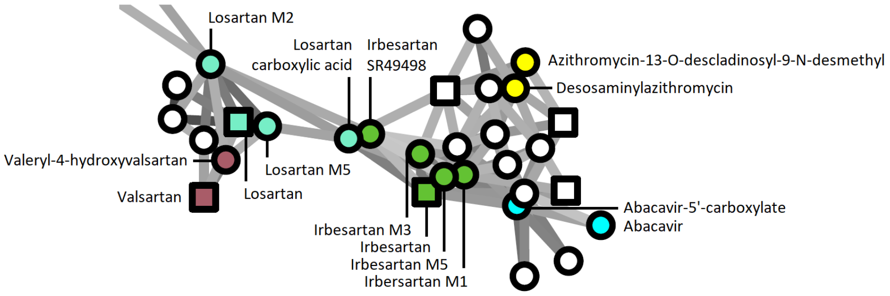

Figure SI-D408: Excerpt of the molecular network showing the valsartan cluster.

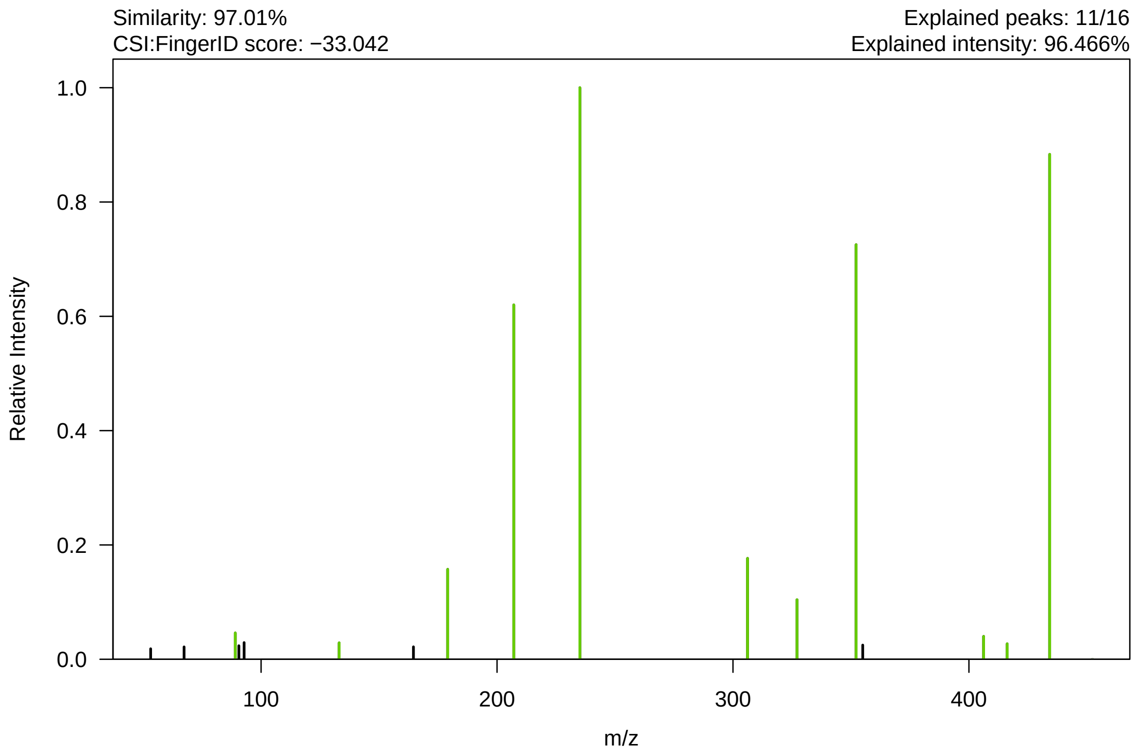

Figure SI-D409: Measured MS2 spectrum. Matching fragments with valeryl-4-hydroxyvalsartan predicted by SIRIUS/CSI:FingerID are highlighted in green.

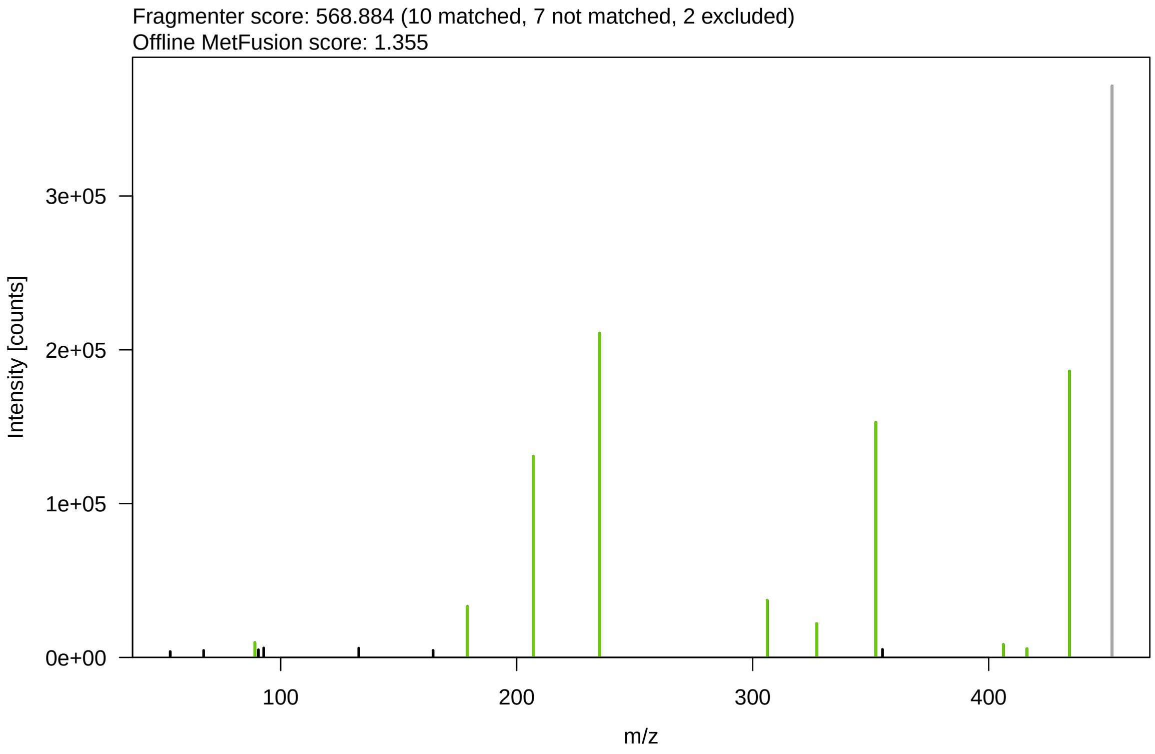

Figure SI-D410: Measured MS2 spectrum. Matching fragments with valeryl-4-hydroxyvalsartan predicted by MetFrag are highlighted in green. The molecular ion in gray is not considered.

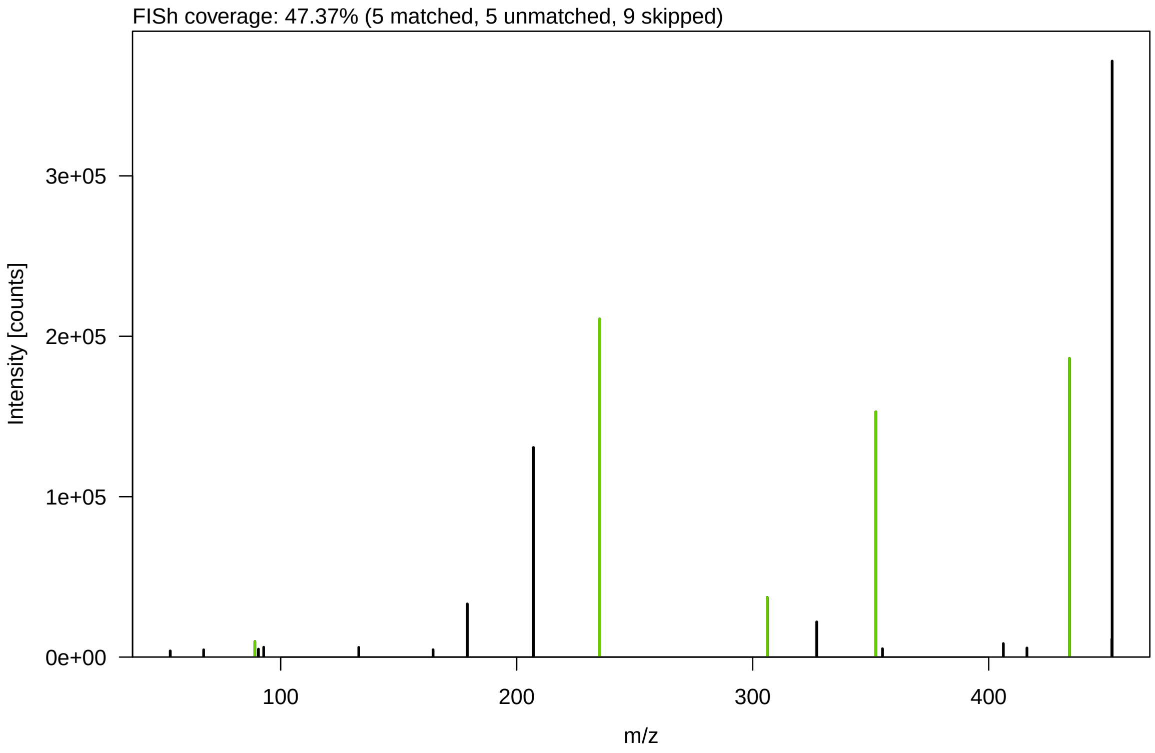

Figure SI-D411: Measured MS2 spectrum. Matching fragments with valeryl-4-hydroxyvalsartan predicted by FISh Scoring are highlighted in green. Low intensity fragments are not considered and skipped.

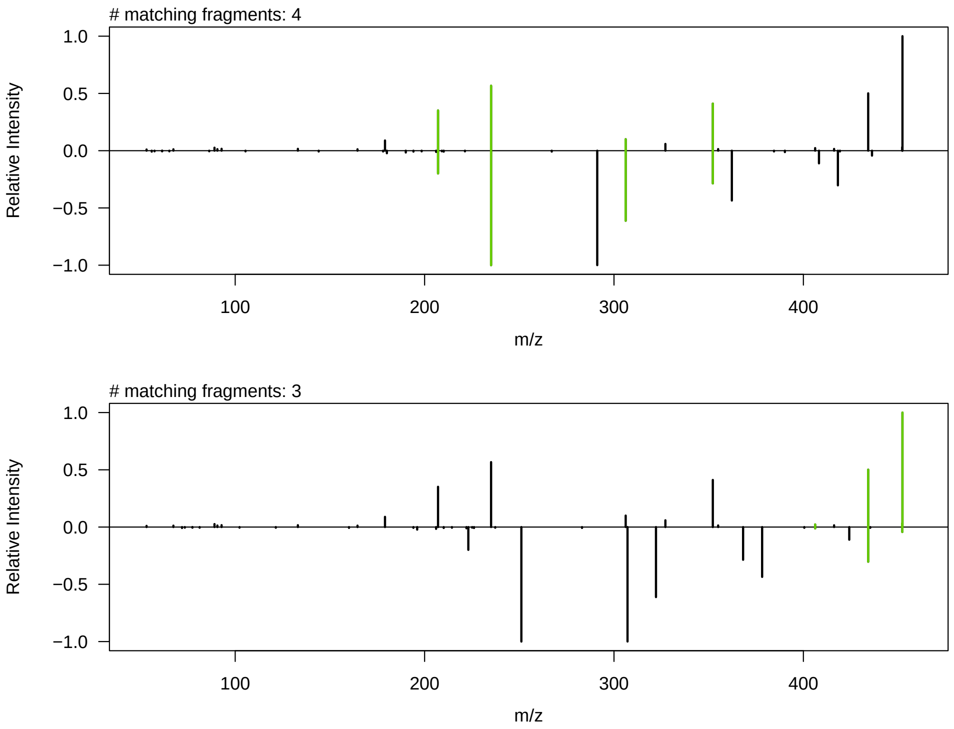

Figure SI-D412: Head to tail plots of valeryl-4-hydroxyvalsartan and valsartan. In the bottom plot, the mass spectrum of valsartan is shifted by the mass difference. Matching fragments are highlighted in green.

Table SI-D203: Molecular network results and retention time prediction of valeryl-4-hydroxyvalsartan.

Comparison with	Valsartan
MSn Score	48
Forward coverage	44
Reverse coverage	53
Forward match	15
Reverse match	10
ΔMass [g/mol]	15.9949
Measured retention time [min]	18.4
Predicted $\log D_{OW}$ (pH = 2.7)	3.84
Predicted retention time [min]	19.7
Predicted retention time range (95% confidence interval) [min]	15.1-24.4
Predicted retention time range (99% confidence interval) [min]	13.7-25.8

Table SI-D204: Annotated MS2 spectrum of valeryl-4-hydroxyvalsartan.

m/z	Relative Intensity	Annotation
53.1948	10.40	
67.3632	12.28	
89.0594	26.09	$C_4H_8O_2 + H^+$
90.5789	13.42	
92.8075	16.56	
133.0861	16.28	$C_6H_{12}O_3 + H^+$
164.5699	12.33	
179.0850	89.17	$C_{14}H_{10} + H^+$
207.0916	351.36	$C_{14}H_{10}N_2 + H^+$
235.0979	566.72	$C_{13}H_{14}O_4 + H^+$
306.1709	100.05	$C_{18}H_{19}N_5 + H^+$
327.1345	59.05	$C_{18}H_{18}N_2O_4 + H^+$
352.1768	411.08	$C_{19}H_{21}N_5O_2 + H^+$
354.9911	14.09	
406.2136	22.69	$C_{24}H_{27}N_3O_3 + H^+$
416.2097	15.34	$C_{24}H_{25}N_5O_2 + H^+$
434.2182	500.63	$C_{24}H_{27}N_5O_3 + H^+$
452.1727	30.13	
452.3118	999.00	$C_{24}H_{29}N_5O_4 + H^+$

A reference standard of valeryl-4-hydroxyvalsartan was purchased. Figure SI-D413 shows the extracted ion chromatograms of this standard, the sample and the spiked sample, as well as a head to tail plot of the MS2 spectra of the standard and the sample. In addition, the most intense MS2 fragments in the sample and in the standard are displayed. It becomes visible that the retention times of the sample and the spiked sample are identical and the spectra similarity score between sample and standard is equal to 0.977. The majority of the MS2 fragments in the sample can be explained by the reference standard. It can therefore be concluded that the suspected compound is indeed valeryl-4-hydroxyvalsartan. Correspondingly, the identification confidence can be increased to level 1.

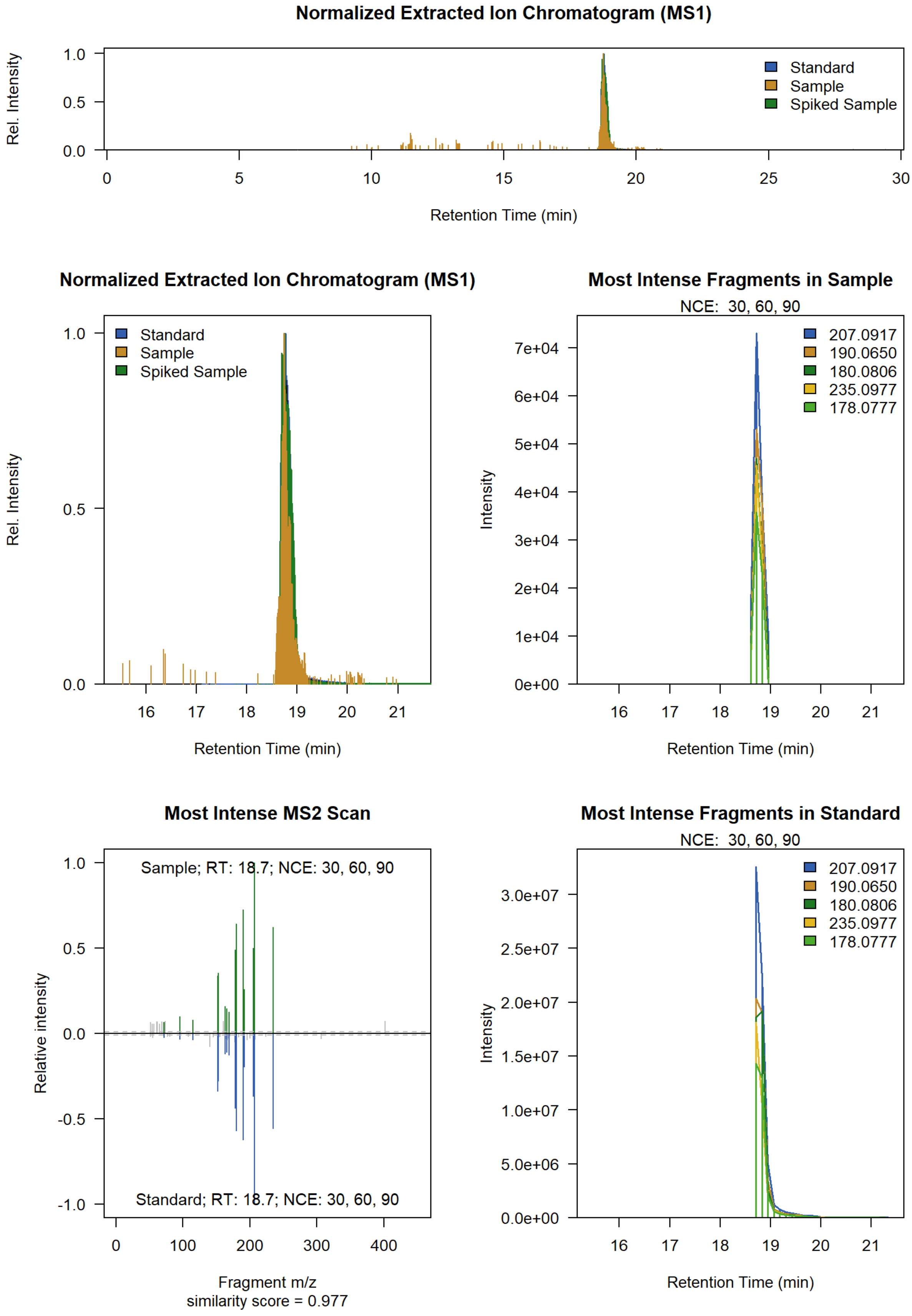

Figure SI-D413: Extracted ion chromatograms of valeryl-4-hydroxyvalsartan in the reference standard, the sample and the spiked sample, as well as MS2 head to tail plot and most intense MS2 fragments in standard and sample.

SI-D2.14.23 Zolpidem Carboxylic Acid

Zolpidem carboxylic acid is a metabolite of zolpidem, which is a sedative hypnotic used for the short-term treatment of insomnia with the aim to improve sleep latency.[2] Figure SI-D414 shows the metabolism scheme.

Table SI-D205: Information on identifiers, chemical properties, detection and confidence of identification of zolpidem carboxylic acid.

IUPAC Name	4-[3-[2-(dimethylamino)-2-oxoethyl]-6-methylimidazo[1,2-a]pyridin-2-yl]benzoic acid
Molecular formula	$C_{19}H_{19}N_3O_3$
Monoisotopic mass [g/mol]	337.1426
Adduct	$[M+H]^+$
Retention time [min]	12.1
SMILES	CC1=CN2C(=NC(=C2CC(=O)N(C)C)C3=CC=C(C=C3)C(=O)O)C=C1
InChI	InChI=1S/C19H19N3O3/c1-12-4-9-16-20-18(13-5-7-14(8-6-13)19(24)25)15(22(16)11-12)10-17(23)21(2)3/h4-9,11H,10H2,1-3H3,(H,24,25)
InChI-Key	FELZONDEFBLTSP-UHFFFAOYSA-N
CAS RN	109461-65-6
Metabolite of	Zolpidem
Detection frequency	100% (15/15 samples)
Detected in	Altenrhein, Monday-Friday<br>Neugut, Monday-Friday<br>Werdhölzli, Monday-Friday
Intensity	E7
Initial confidence level	level 3
Initial confidence score	0.36
Final confidece level	level 1

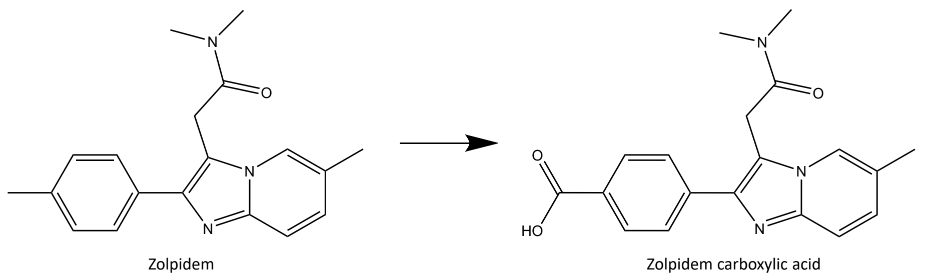

Figure SI-D414: Molecular structure of zolpidem carboxylic acid.

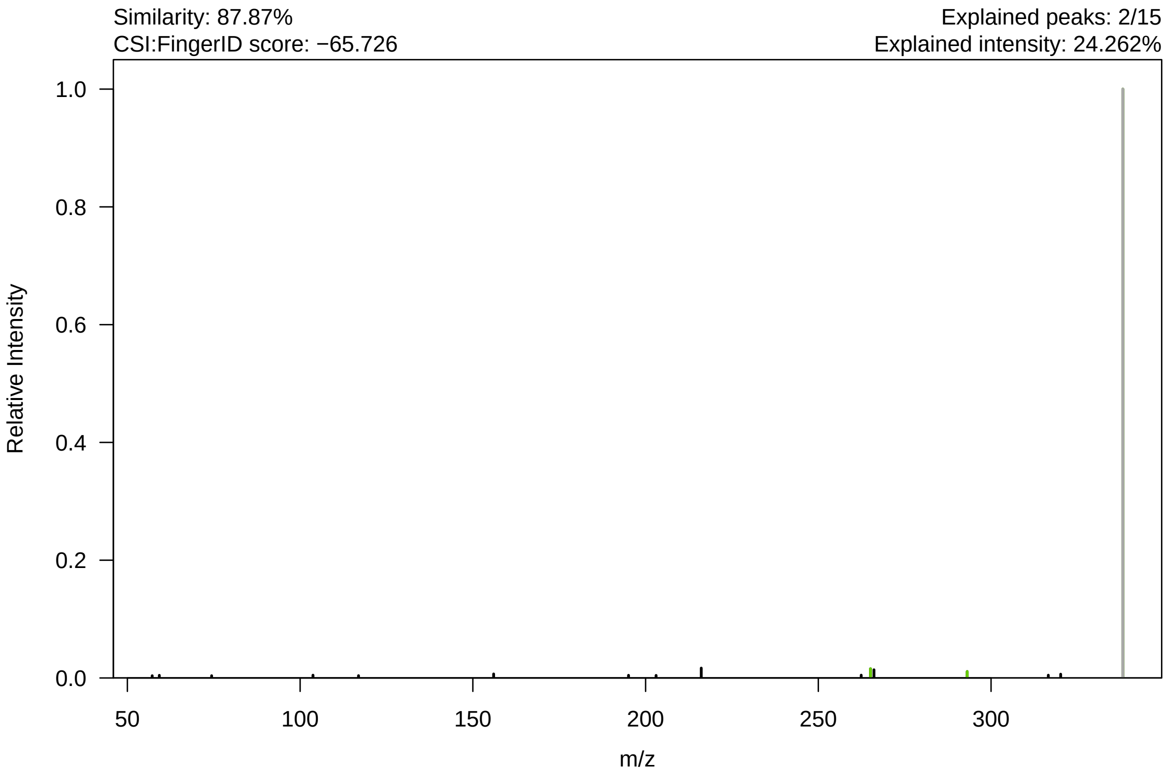

Figure SI-D415: Measured MS2 spectrum. Matching fragments with zolpidem carboxylic acid predicted by SIRIUS/CSI:FingerID are highlighted in green. The molecular ion in gray is not considered.

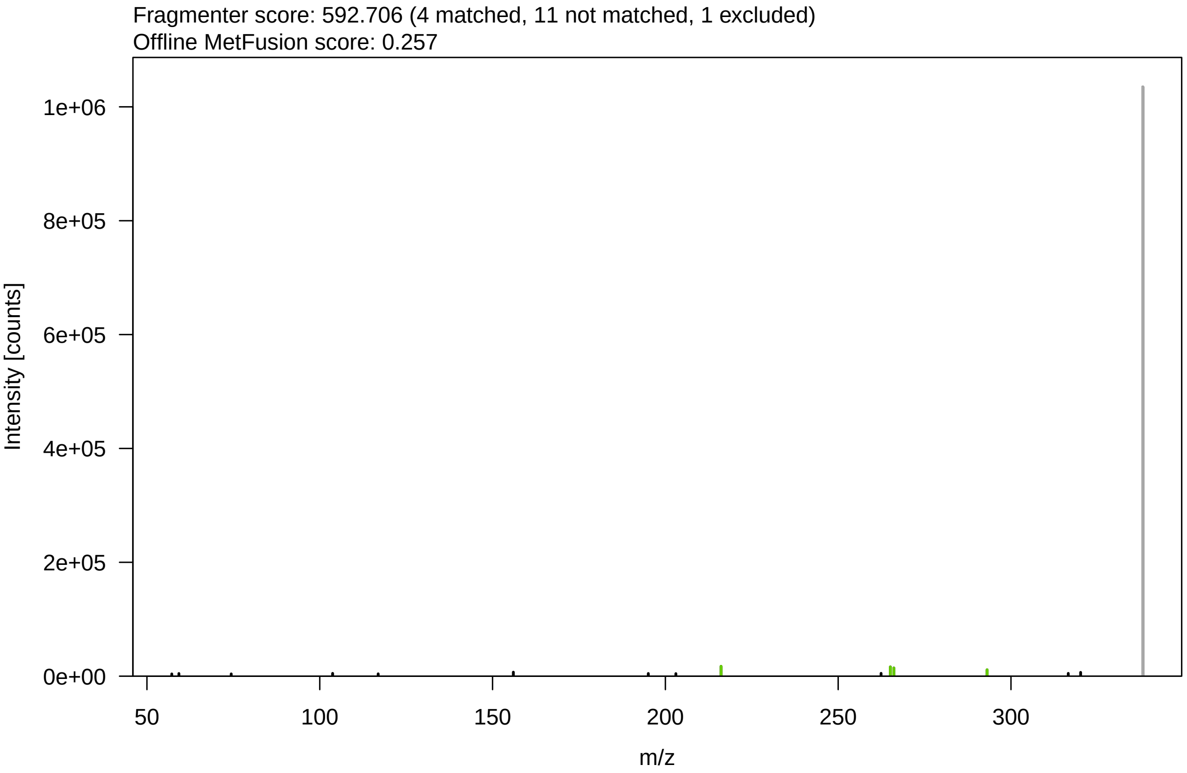

Figure SI-D416: Measured MS2 spectrum. Matching fragments with zolpidem carboxylic acid predicted by MetFrag are highlighted in green. The molecular ion in gray is not considered.

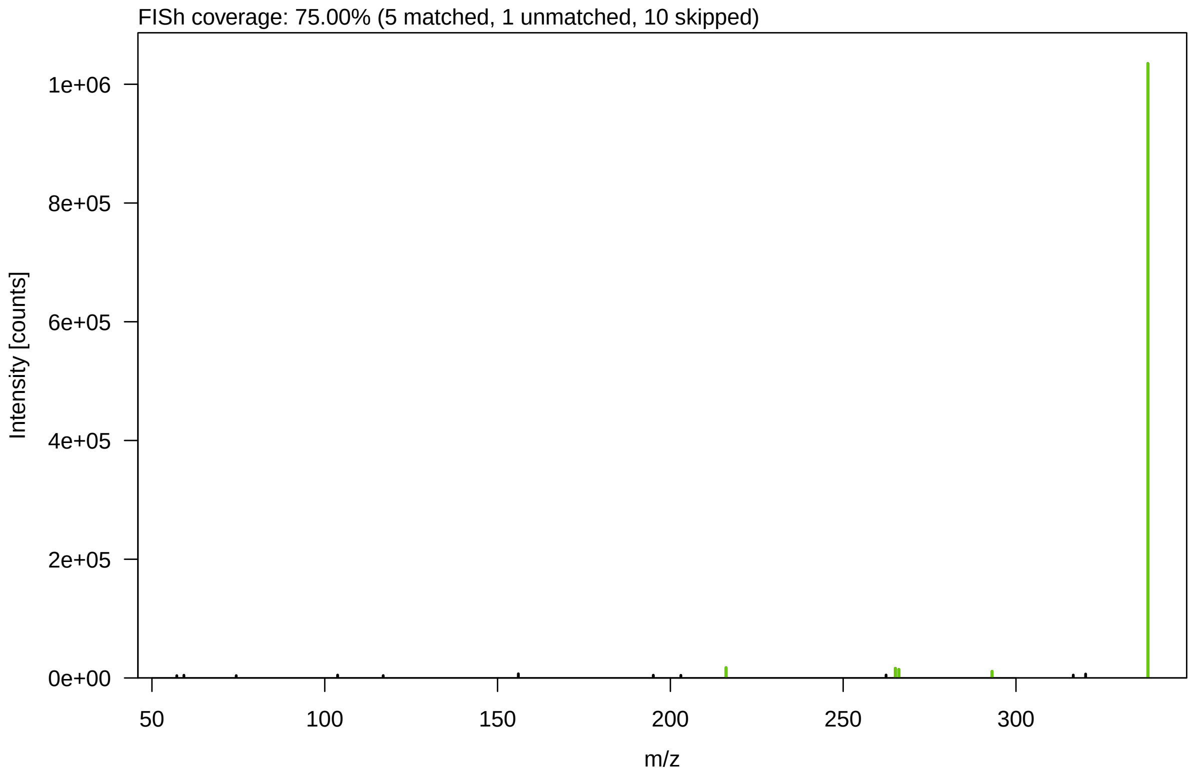

Figure SI-D417: Measured MS2 spectrum. Matching fragments with zolpidem carboxylic acid predicted by FISh Scoring are highlighted in green. Low intensity fragments are not considered and skipped.

Table SI-D206: Retention time prediction of zolpidem carboxylic acid.

Measured retention time [min]	12.1
Predicted $\log D_{OW}$ (pH = 2.7)	0.64
Predicted retention time [min]	15.6
Predicted retention time range (95% confidence interval) [min]	11.0-20.2
Predicted retention time range (99% confidence interval) [min]	9.5-21.6

Table SI-D207: Annotated MS2 spectrum of zolpidem carboxylic acid.

m/z	Relative Intensity	Annotation
57.1845	3.82	
59.2448	4.50	
74.3829	3.85	
103.7223	4.74	
116.9054	3.96	
156.0116	6.80	
195.0647	4.53	
203.0401	4.33	
216.1132	16.74	$C_{12}H_{13}N_3O + H^+$
262.4145	4.79	
265.0970	15.72	$C_{16}H_{12}N_2O_2 + H^+$
266.1050	13.90	$C_{16}H_{13}N_2O_2 + H^+$
293.0910	10.91	$C_{17}H_{12}N_2O_3 + H^+$
316.5768	4.72	
320.1519	6.43	
338.1497	999.00	$C_{19}H_{19}N_3O_3 + H^+$

A reference standard of zolpidem carboxylic acid was purchased. Figure SI-D418 shows the extracted ion chromatograms of this standard, the sample and the spiked sample, as well as a head to tail plot of the MS2 spectra of the standard and the sample. In addition, the most intense MS2 fragments in the sample and in the standard are displayed. It becomes visible that the retention times of the sample and the spiked sample are identical and the spectra similarity score between sample and standard is equal to 0.961. The majority of the MS2 fragments in the sample can be explained by the reference standard. It can therefore be concluded that the suspected compound is indeed zolpidem carboxylic acid. Correspondingly, the identification confidence can be increased to level 1.

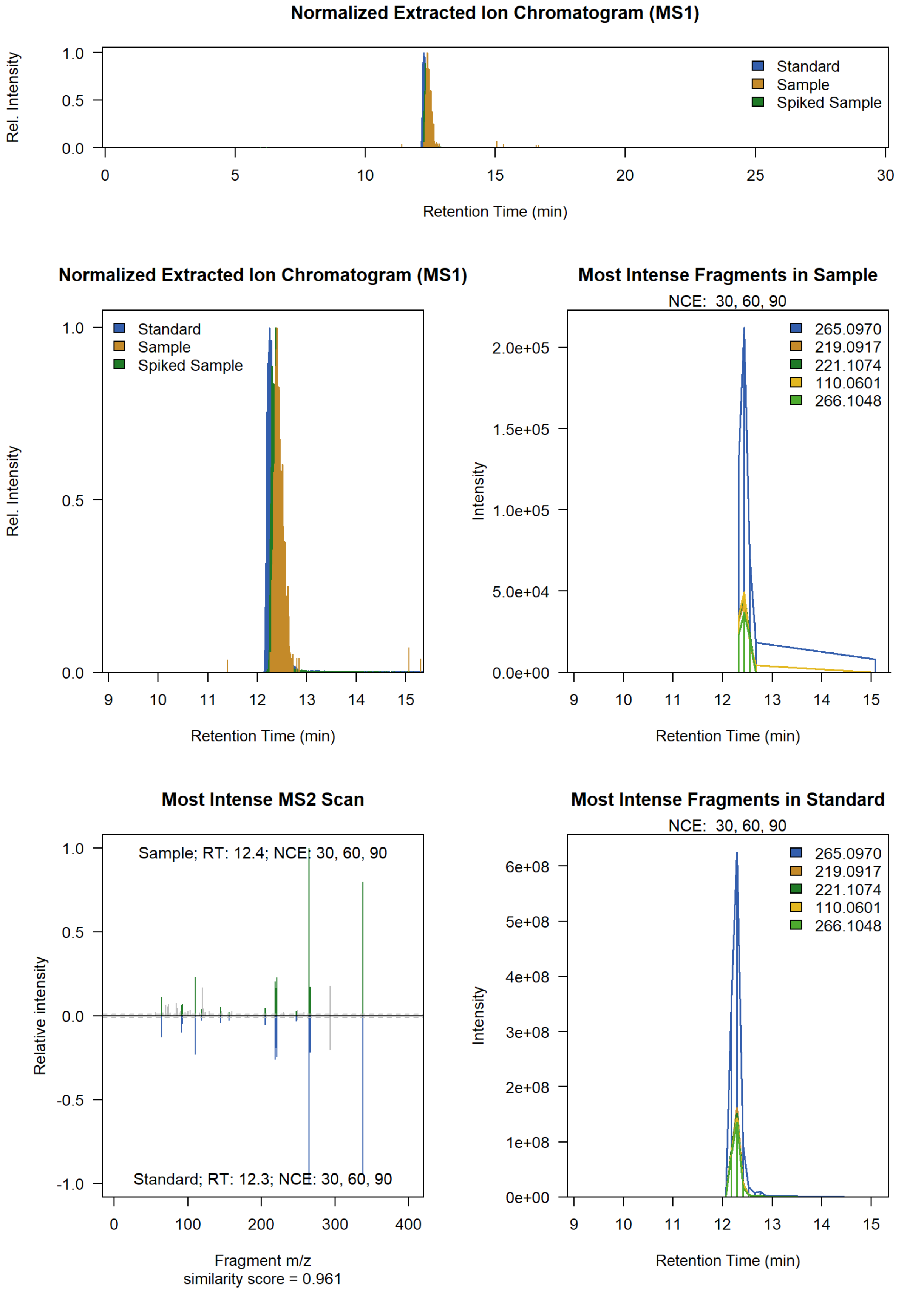

Figure SI-D418: Extracted ion chromatograms of zolpidem carboxylic acid in the reference standard, the sample and the spiked sample, as well as MS2 head to tail plot and most intense MS2 fragments in standard and sample.

SI-D2.15 Other Phase II Metabolites

SI-D2.15.1 4-Hydroxypropanolol-Sulfate (HOPS)

4-Hydroxypropanolol-sulfate is a metabolite of propanolol, which is non-selective beta adrenergic antagonist used to treat hypertension, angina, atrial fibrillation, myocardial infarction and migraine.[2] Figure SI-D419 shows the metabolism scheme.

Table SI-D208: Information on identifiers, chemical properties, detection and confidence of identification of 4-hydroxypropanolol-sulfate.

IUPAC Name	[4-[2-hydroxy-3-(propan-2-ylamino)propoxy]naphthalen-1-yl] hydrogen sulfate
Molecular formula	$C_{16}H_{21}NO_6S$
Monoisotopic mass [g/mol]	355.1090
Adduct	$[M-H]^-$
Retention time [min]	16.2
SMILES	CC(C)NCC(COC1=CC=C(C2=CC=CC=C21)OS(=O)(=O)O)O
InChI	InChI=1S/C16H21NO6S/c1-11(2)17-9-12(18)10-22-15-7-8-16(23-24(19,20)21)14-6-4-3-5-13(14)15/h3-8,11-12,17-18H,9-10H2,1-2H3,(H,19,20,21)
InChI-Key	ODCKICSDIPVTRM-UHFFFAOYSA-N
CAS RN	87075-33-0
Metabolite of	Propanolol
Detection frequency	100% (15/15 samples)
Detected in	Altenrhein, Monday-Friday<br>Neugut, Monday-Friday<br>Werdhölzli, Monday-Friday
Intensity	E7-E8
Initial confidence level	level 3
Initial confidence score	0.37
Final confidence level	level 4

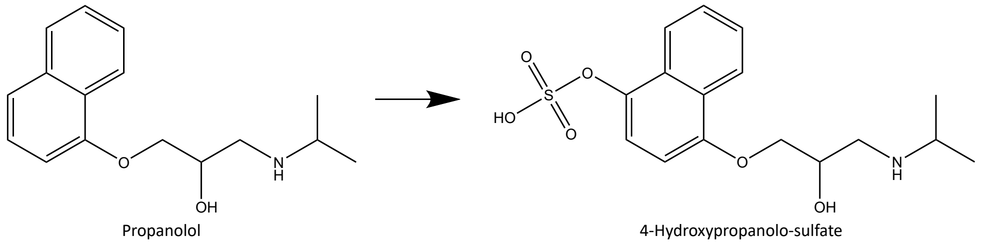

Figure SI-D419: Metabolism of propanolol to 4-hydroxypropanolol-sulfate.

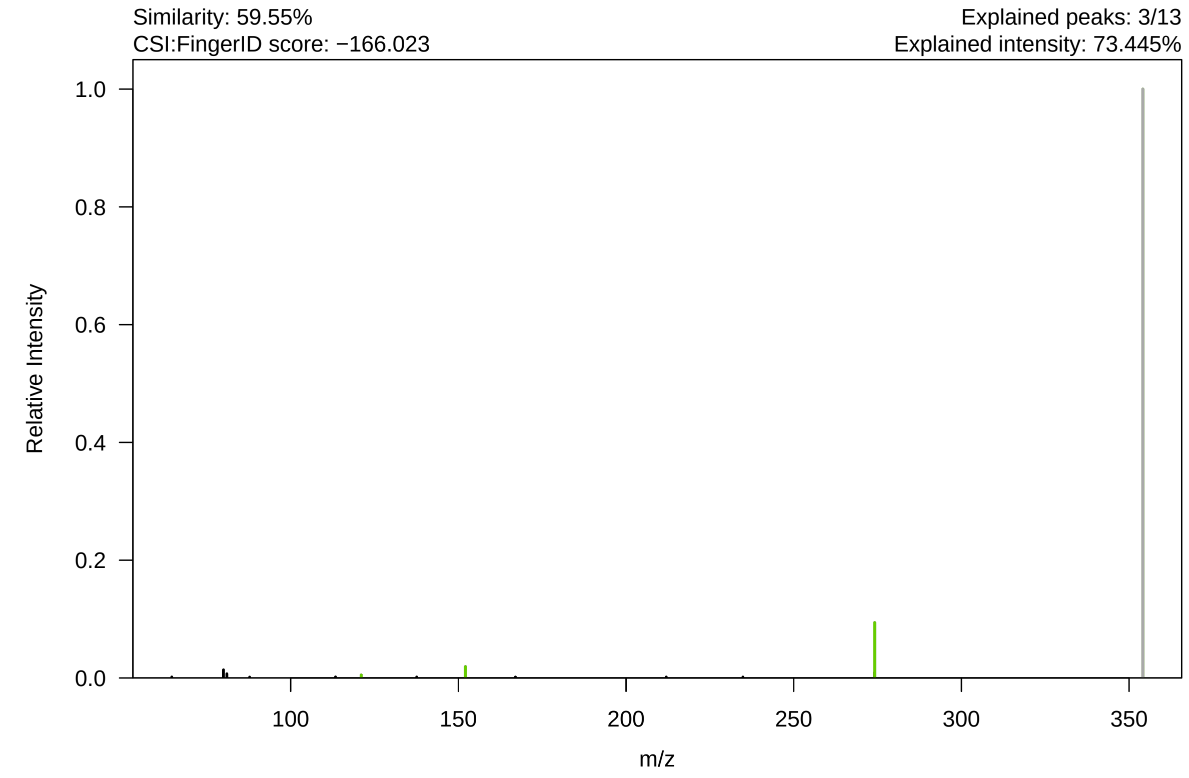

Figure SI-D420: Measured MS2 spectrum. Matching fragments with 4-hydroxypropanolol-sulfate predicted by SIRIUS/CSI:FingerID are highlighted in green. The molecular ion in gray is not considered.

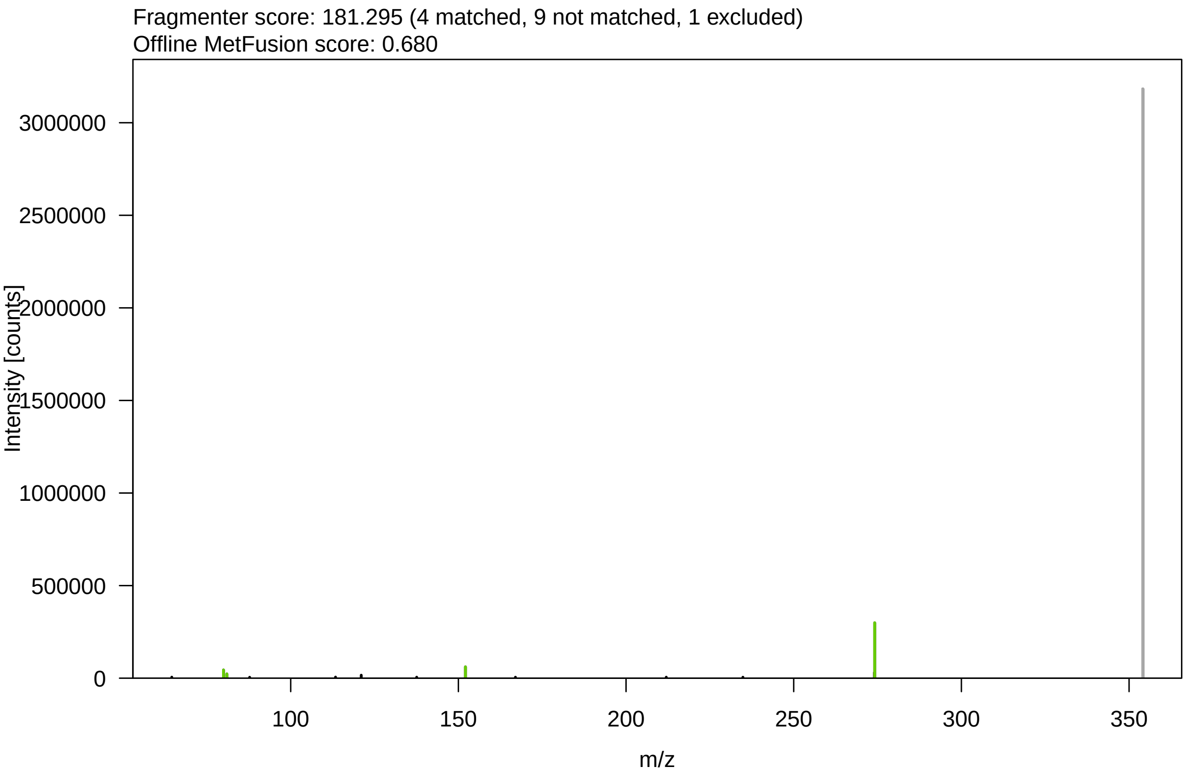

Figure SI-D421: Measured MS2 spectrum. Matching fragments with 4-hydroxypropanolol-sulfate predicted by MetFrag are highlighted in green. The molecular ion in gray is not considered.

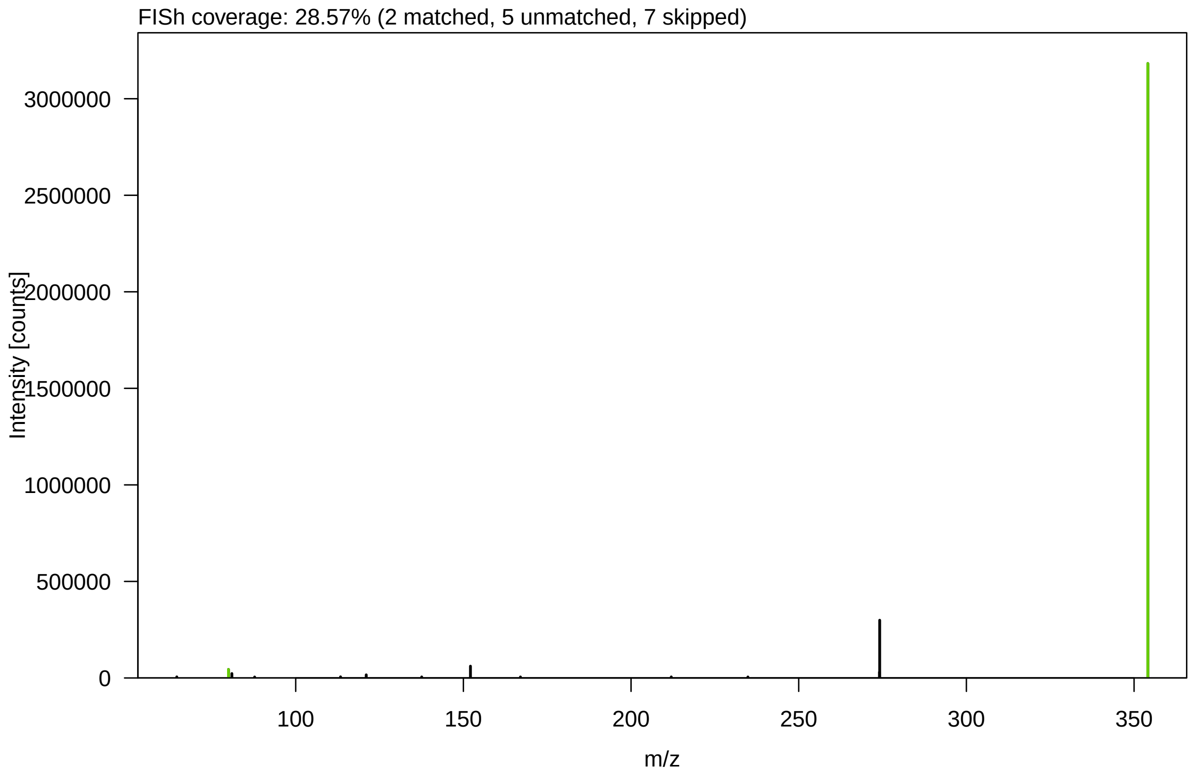

Figure SI-D422: Measured MS2 spectrum. Matching fragments with 4-hydroxypropanolol-sulfate predicted by FISh Scoring are highlighted in green. Low intensity fragments are not considered and skipped.

Table SI-D209: Retention time prediction of 4-hydroxypropanolol-sulfate.

Measured retention time [min]	16.2
Predicted $logD_{OW}$ (pH = 4.8)	0.59
Predicted retention time [min]	15.5
Predicted retention time range (95% confidence interval) [min]	8.4-22.6
Predicted retention time range (99% confidence interval) [min]	6.0-24.9

Table SI-D210: Annotated MS2 spectrum of 4-hydroxypropanolol-sulfate.

m/z	Relative Intensity	Annotation
64.5388	1.78	
79.9570	13.91	$HO_3S - H-$;
80.9650	7.18	HO_3S^-
87.7530	1.59	
113.3703	1.87	
121.0298	5.10	$C_7H_6O_2 - H^-$
137.5682	1.71	
152.1083	19.22	$C_9H_{15}NO - H^-$
167.0148	1.68	
211.9959	1.72	
234.8460	1.64	
274.0824	9.64	
274.1451	93.89	$C_{16}H_{21}NO_3 - H^-$
354.1018	999.00	$C_{16}H_{21}NO_6S - H^-$

A reference standard of 4-hydroxypropanolol-sulfate was purchased. Figure SI-D423 shows the extracted ion chromatograms of this standard, the sample and the spiked sample, as well as a head to tail plot of the MS2 spectra of the standard and the sample. In addition, the most intense MS2 fragments in the sample and in the standard are displayed. It becomes visible that the retention times of the sample and the spiked sample are not matching. The suspected compound elutes about five minutes later than the reference standard. Moroever, only a few of the MS2 fragments in the sample can be explained by the reference standard. Therefore, the suspect cannot be confirmed as 4-hydroxypropanolol-sulfate and the confidence level is decreased to level 4 due to the unequivocal molecular formula.

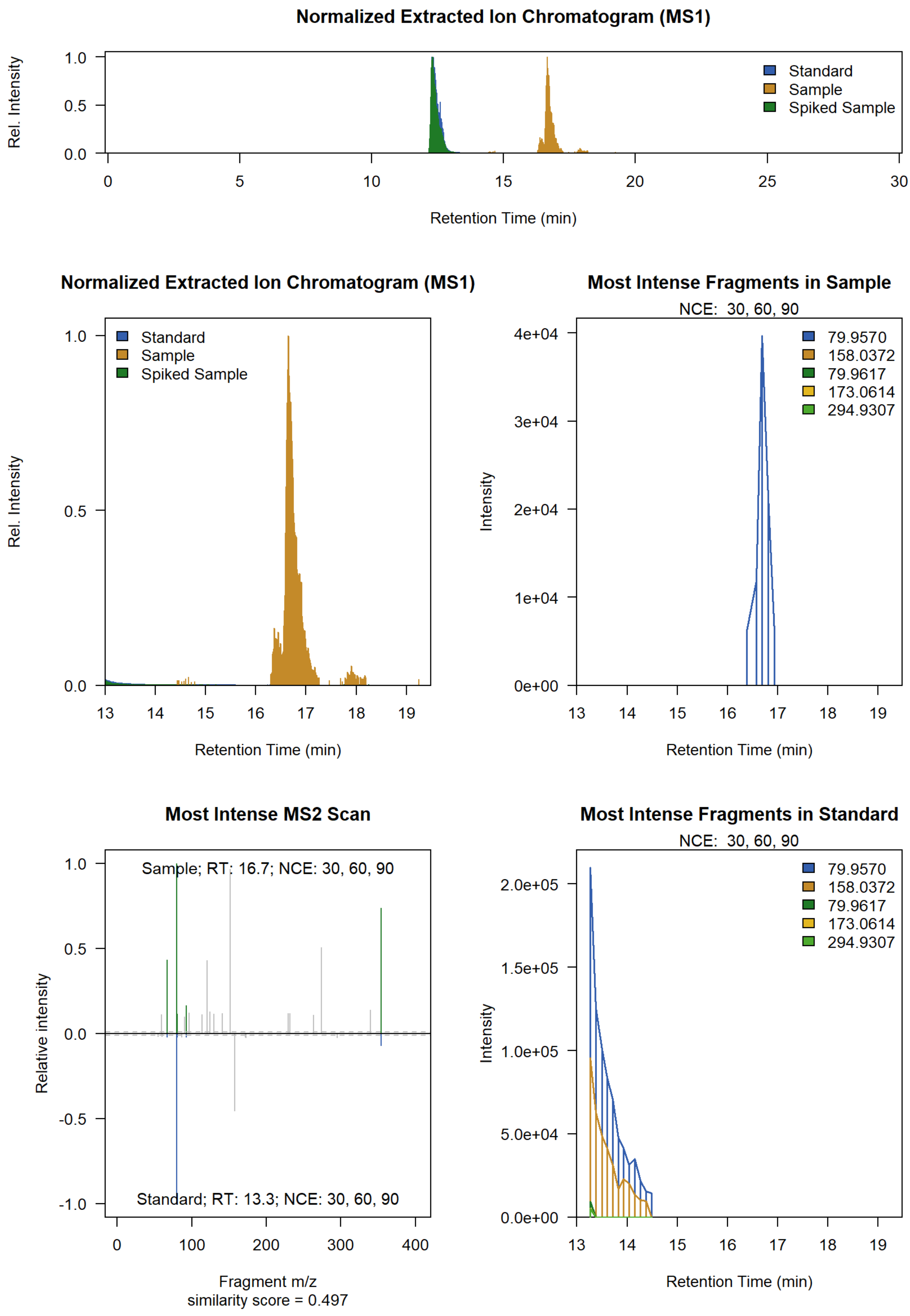

Figure SI-D423: Extracted ion chromatograms of 4-hydroxypropanolol-sulfate in the reference standard, the sample and the spiked sample, as well as MS2 head to tail plot and most intense MS2 fragments in standard and sample.

SI-D2.15.2 4-Quinol-Sulfate

4-Quinol-sulfate is a propofol metabolite, which is used for sedation and as anesthetic agent.[2] Figure SI-D424 shows the metabolism scheme.

Table SI-D211: Information on identifiers, chemical properties, detection and confidence of identification of 4-quinol-sulfate.

IUPAC Name	[4-hydroxy-3,5-di(propan-2-yl)phenyl] hydrogen sulfate
Molecular formula	$C_{12}H_{18}O_5S$
Monoisotopic mass [g/mol]	274.0875
Adduct	$[M-H]^-$
Retention time [min]	15.2
SMILES	CC(C)C1=CC(=CC(=C1O)C(C)C)OS(=O)(=O)O
InChI	InChI=1S/C12H18O5S/c1-7(2)10-5-9(17-18(14,15)16)6-11(8(3)4)12(10)13/h5-8,13H,1-4H3,(H,14,15,16)
InChI-Key	RKAQPQOYAJQIAA-UHFFFAOYSA-N
CAS RN	114991-27-4
Metabolite of	Propanolol
Detection frequency	100% (15/15 samples)
Detected in	Altenrhein, Monday-Friday
	Neugut, Monday-Friday
	Werdhölzli, Monday-Friday
Intensity	E6-E7
Initial confidence level	level 3
Initial confidence score	0.47
Final confidence level	level 3

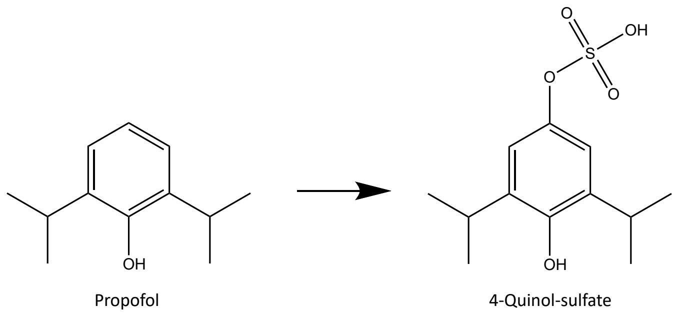

Figure SI-D424: Metabolism of propanolol to 4-quinol-sulfate.

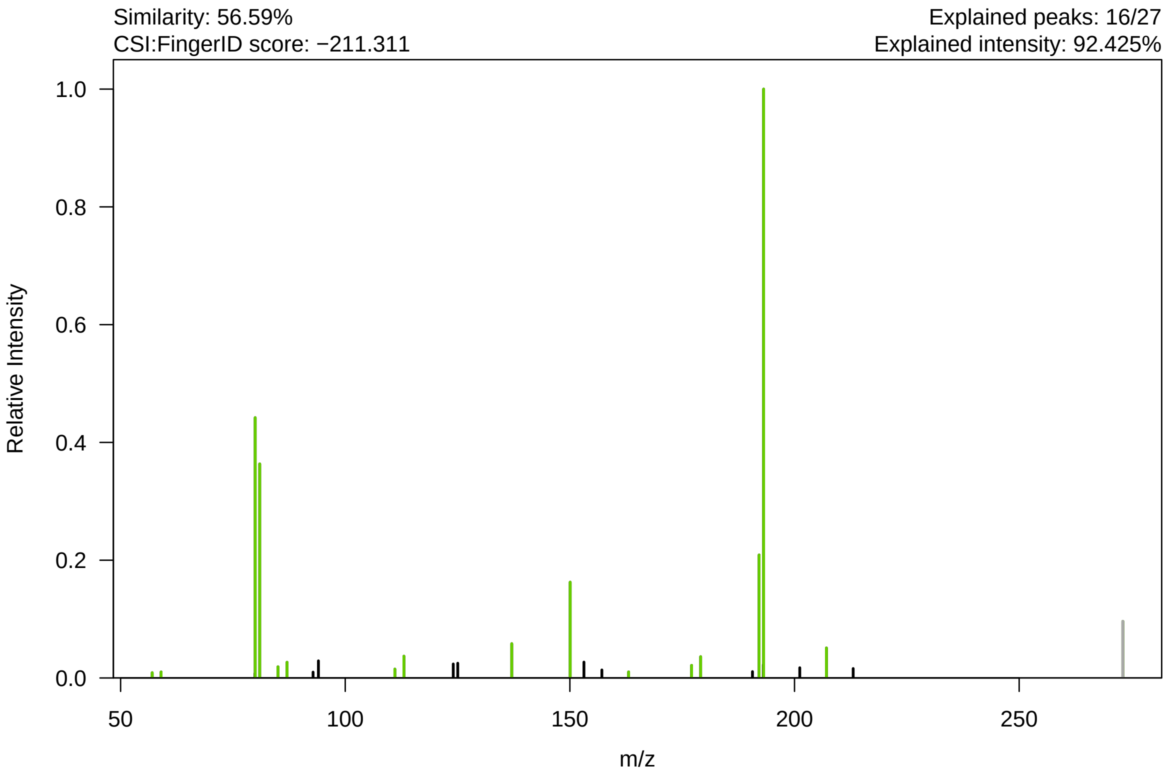

Figure SI-D425: Measured MS2 spectrum. Matching fragments with 4-quinol-sulfate predicted by SIRIUS/CSI:FingerID are highlighted in green. The molecular ion in gray is not considered.

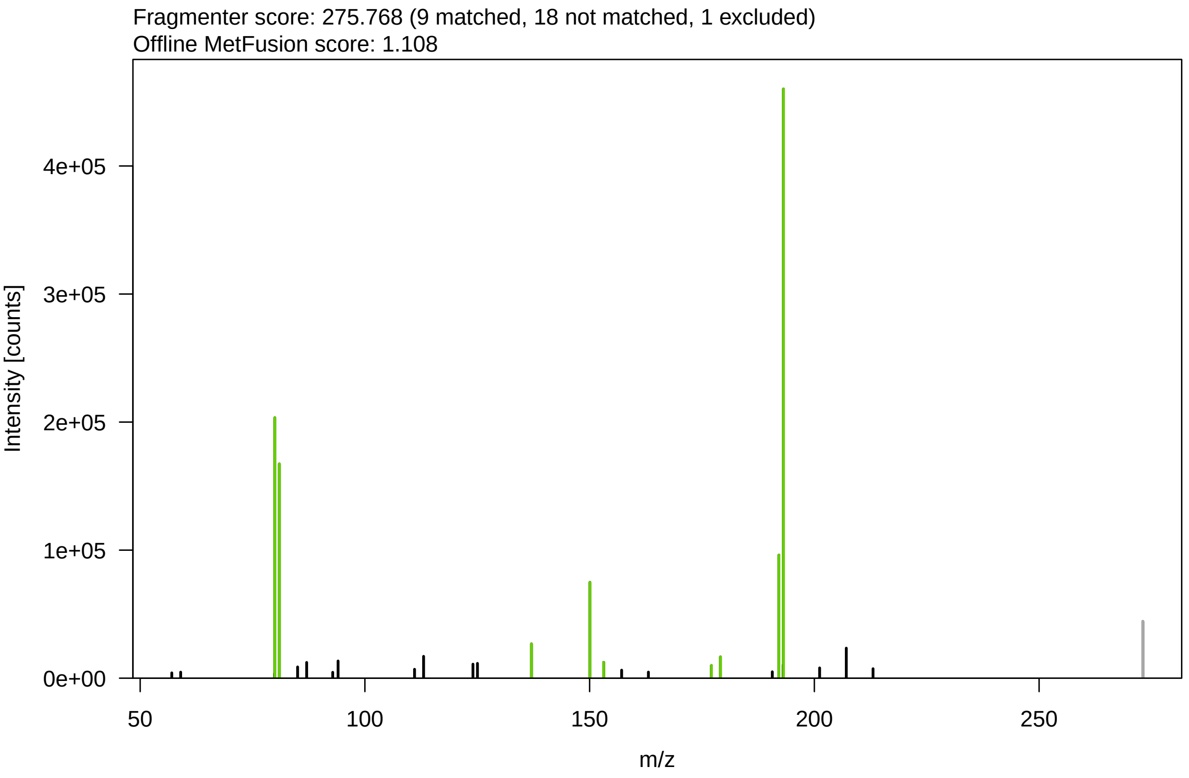

Figure SI-D426: Measured MS2 spectrum. Matching fragments with 4-quinol-sulfate predicted by MetFrag are highlighted in green. The molecular ion in gray is not considered.

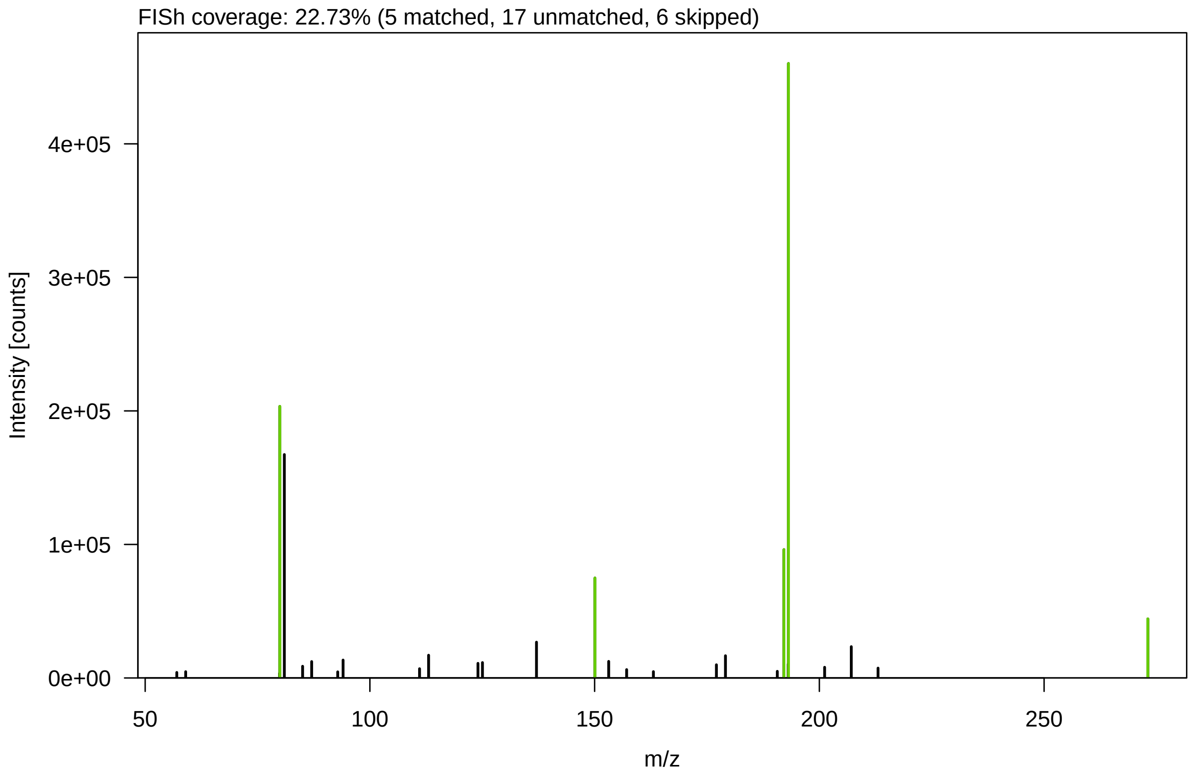

Figure SI-D427: Measured MS2 spectrum. Matching fragments with 4-quinol-sulfate predicted by FISh Scoring are highlighted in green. Low intensity fragments are not considered and skipped.

Table SI-D212: Retention time prediction of 4-quinol-sulfate.

Measured retention time [min]	15.2
Predicted logD_{OW} (pH = 4.8)	1.00
Predicted retention time [min]	16.2
Predicted retention time range (95% confidence interval) [min]	9.1-23.3
Predicted retention time range (99% confidence interval) [min]	6.8-25.6

Table SI-D213: Annotated MS2 spectrum of 4-quinol-sulfate.

m/z	Relative Intensity	Annotation
57.0338	8.97	$C_3H_6O - H^-$
59.0130	10.23	$C_2H_4O_2 - H^-$
79.8918	7.34	
79.9571	441.63	$HO_3S - H^-$
80.9650	363.25	$H_2O_3S - H^-$
85.0293	19.04	$C_4H_6O_2 - H^-$
87.0453	26.67	$C_4H_8O_2 - H^-$
92.8386	9.94	
94.0300	29.12	
111.0462	15.06	$C_6H_8O_2 - H^-$
113.0609	37.00	$C_6H_{10}O_2 - H^-$
124.0405	23.76	
125.0439	25.04	
137.0611	58.21	$C_8H_{10}O_2 - H^-$
150.0686	162.53	$C_9H_{11}O_2 - H^-$
153.1286	26.94	$C_{10}H_{18}O - H^-$
157.1231	13.61	
163.0762	10.33	$C_{10}H_{12}O_2 - H^-$
177.0917	21.45	$C_{11}H_{14}O_2 - H^-$
179.1078	36.15	$C_{11}H_{16}O_2 - H^-$
190.6421	10.81	
192.1158	208.69	$C_{12}H_{17}O_2 - H^-$
193.0502	22.34	
193.1233	999.00	$C_{12}H_{18}O_2 - H^-$
201.1688	17.40	
207.1029	50.93	$C_{12}H_{16}O_3 - H^-$
213.0554	16.04	
273.0802	96.12	$C_{12}H_{18}O_5S - H^-$

A reference standard of 4-quinol sulfate was not purchased and human liver S9 incubation experiments were only performed for phase I metabolites. Correspondingly, 4-quinol-sulfate is neither confirmed nor rejected, leading to an identical initial and final confidence level of 3.

SI-D2.15.3 Diphenhydramine-N-Glucuronide

Diphenhydramine-N-glucuronide is a metabolite of diphenhydramine, which is a H1 receptor antihistamine used in the treatment of seasonal allergies.[2] Figure SI-D428 shows the metabolism scheme.

Table SI-D214: Information on identifiers, chemical properties, detection and confidence of identification of diphenhydramine-N-glucuronide.

IUPAC Name	(2*S*,3*S*,4*S*,5*R*,6*R*)-6-[2-benzhydryloxyethyl(dimethyl)azaniumyl]-3,4,5-trihydroxyoxane-2-carboxylate
Molecular formula	$C_{23}H_{29}NO_7$
Monoisotopic mass [g/mol]	431.1944
Adduct	$[M+H]^+$
Retention time [min]	16.4
SMILES	C[N+](C)(CCOC(C1=CC=CC=C1)C2=CC=CC=C2)[C@H]3[C@@H]([C@H]([C@@H]([C@H](O3)C(=O)[O-])O)O)O
InChI	InChI=1S/C23H29NO7/c1-24(2,22-19(27)17(25)18(26)21(31-22)23(28)29)13-14-30-20(15-9-5-3-6-10-15)16-11-7-4-8-12-16/h3-12,17-22,25-27H,13-14H2,1-2H3/t17-,18-,19+,21-,22+/m0/s1
InChI-Key	OAIGZXXQYIJBLR-WJJPVSRMSA-N
CAS RN	137908-78-2
Metabolite of	Diphenhydramine
Detection frequency	100% (15/15 samples)
Detected in	Altenrhein, Monday-Friday<br>Neugut, Monday-Friday<br>Werdhölzli, Monday-Friday
Intensity	E6
Initial confidence level	level 3
Initial confidence score	0.31
Final confidence level	level 1

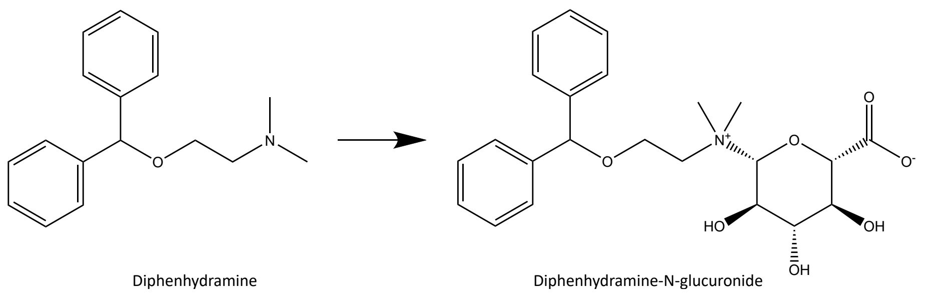

Figure SI-D428: Metabolism of diphenhydramine to diphenhydramine-N-glucuronide.

No FISh Scoring was possible, since it is not able to handle charged structures.

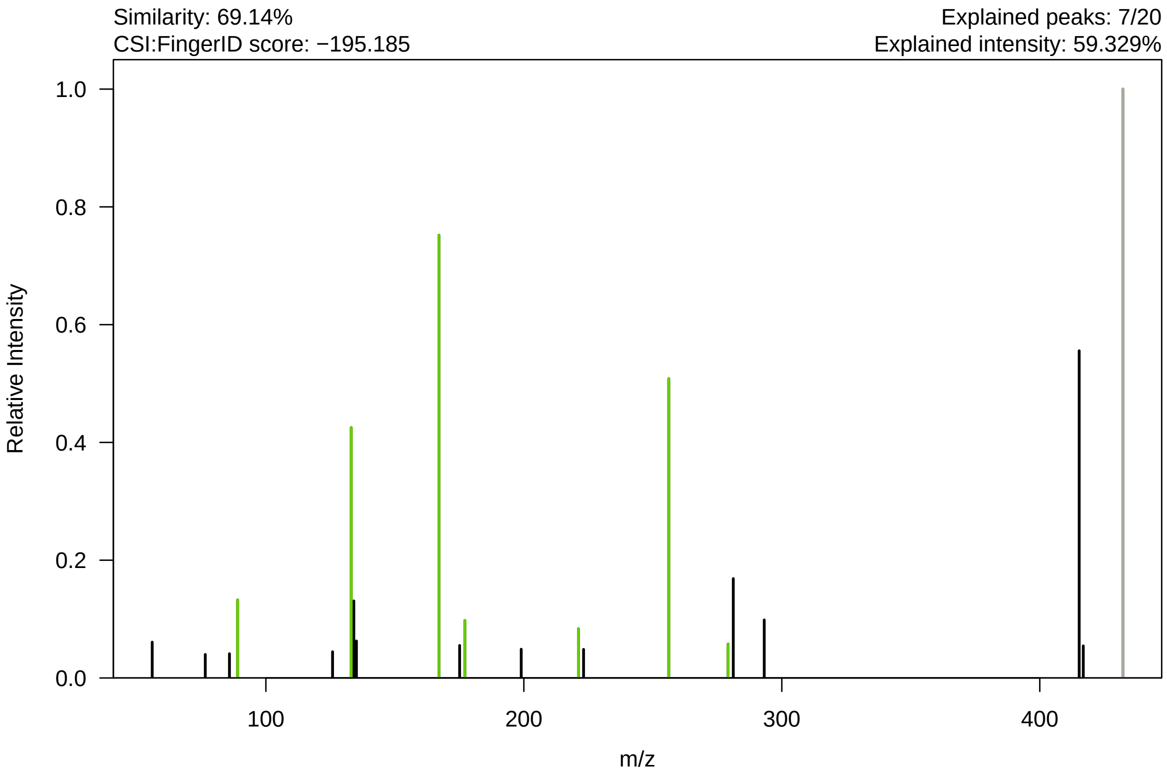

Figure SI-D429: Measured MS2 spectrum. Matching fragments with diphenhydramine-N-glucuronide predicted by SIRIUS/CSI:FingerID are highlighted in green. The molecular ion in gray is not considered.

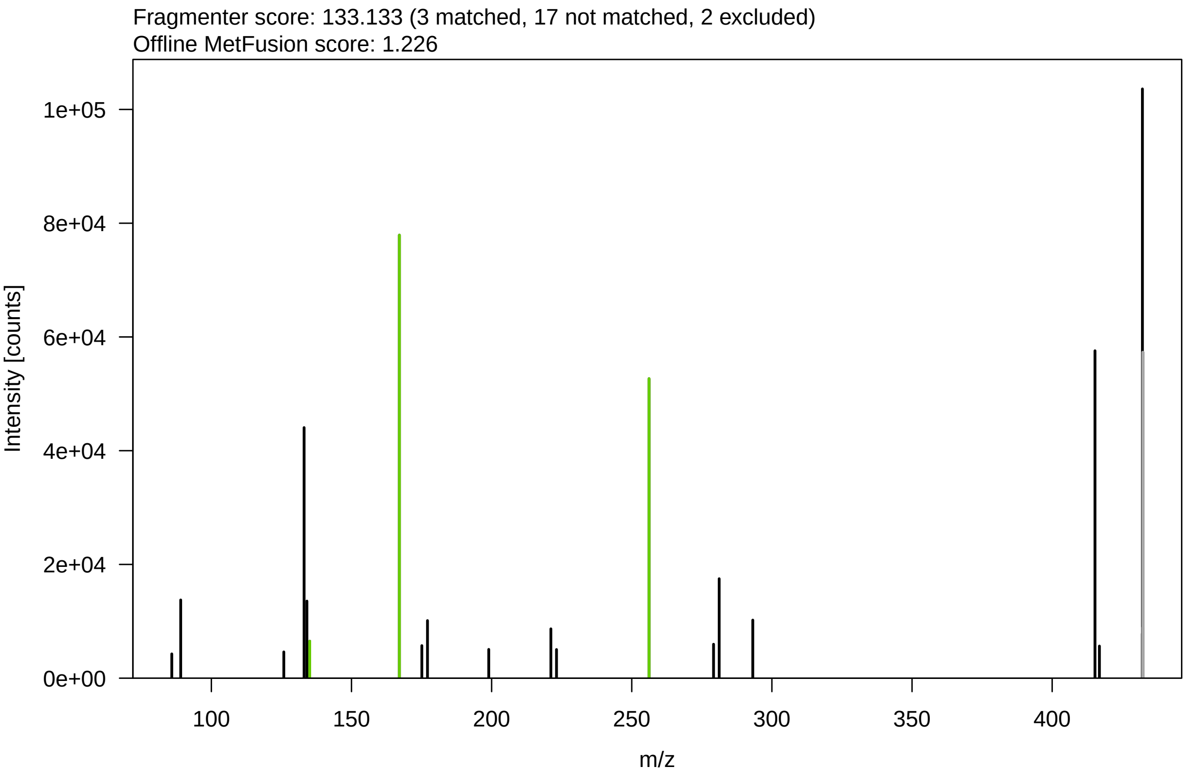

Figure SI-D430: Measured MS2 spectrum. Matching fragments with diphenhydramine-N-glucuronide predicted by MetFrag are highlighted in green. The molecular ion in gray is not considered.

Table SI-D215: Retention time prediction of diphenhydramine-N-glucuronide.

Measured retention time [min]	16.4
Predicted $logD_{OW}$ (pH = 4.8)	-2.31
Predicted retention time [min]	11.7
Predicted retention time range (95% confidence interval) [min]	7.1-16.3
Predicted retention time range (99% confidence interval) [min]	5.7-17.8

Table SI-D216: Annotated MS2 spectrum of diphenhydramine-N-glucuronide.

m/z	Relative Intensity	Annotation
85.8762	41.15	
89.0596	132.58	$C_4H_8O_2 + H^+$
125.8462	44.51	
133.0859	424.79	$C_6H_{12}O_3 + H^+$
134.0893	130.70	
135.0904	62.77	$C_5H_{12}NO_3 + H^+$
167.0853	751.18	$C_{13}H_{10} + H^+$
175.0986	55.18	
177.1118	97.61	$C_8H_{16}O_4 + H^+$
198.9756	48.77	
221.1390	83.60	$C_{10}H_{20}O_5 + H^+$
223.1441	48.51	
256.1695	507.83	$C_{17}H_{21}NO + H^+$
279.1802	57.52	$C_{13}H_{26}O_6 + H^+$
281.1858	168.58	
293.1837	98.45	
415.2775	555.10	
416.8592	54.43	
432.1508	75.27	
432.2017	999.00	$C_{23}H_{29}NO_7 + H^+$
432.2967	85.93	
432.3494	552.65	

A reference standard of diphenyhdarmine-N-glucuronide was purchased. Figure SI-D431 shows the extracted ion chromatograms of this standard, the sample and the spiked sample, as well as a head to tail plot of the MS2 spectra of the standard and the sample. In addition, the most intense MS2 fragments in the sample and in the standard are displayed. It becomes visible that the retention times of the sample and the spiked sample are identical and the spectra similarity score between sample and standard is equal to 0.922. The most intense of the sample MS2 fragments are explained by the reference standard. It can therefore be concluded that the suspected compound is indeed diphenyhdramine-N-glucuronide. Correspondingly, the identification confidence can be increased to level 1.

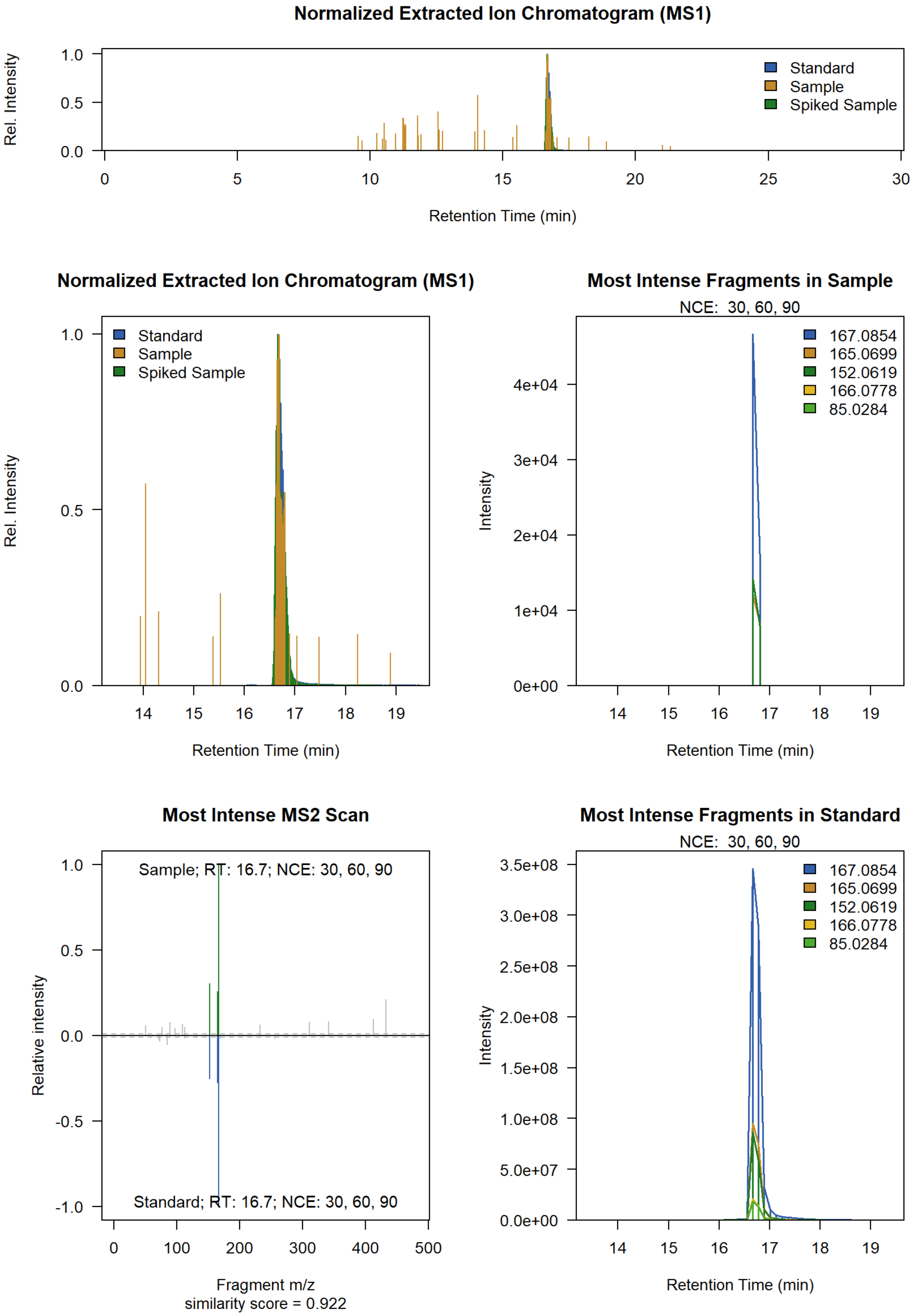

Figure SI-D431: Extracted ion chromatograms of diphenhydramine-N-glucuronide in the reference standard, the sample and the spiked sample, as well as MS2 head to tail plot and most intense MS2 fragments in standard and sample.

SI-D2.15.4 Lamotrigine-N2-Glucuronide

Lamotrigine-N2-glucuronide is a metabolite of lamotrigine, which is a phenyltriazine antiepileptic drug used to treat epilepsy and as a mood stabilizer in bipolar disorder.[2] Figure SI-D432 shows the metabolism scheme.

Table SI-D217: Information on identifiers, chemical properties, detection and confidence of identification of lamotrigine-N2-glucuronide.

IUPAC Name	(2*S*,3*S*,4*S*,5*R*,6*R*)-6-[3,5-diamino-6-(2,3-dichlorophenyl)-1,2,4-triazin-2-ium-2-yl]-3,4,5-trihydroxyoxane-2-carboxylic acid
Molecular formula	$C_{15}H_{16}Cl_2N_5O_6{}^+$
Monoisotopic mass [g/mol]	432.0478
Adduct	$[M]^+$
Retention time [min]	11.9
SMILES	C1=CC(=C(C(=C1)Cl)Cl)C2=C(N=C([N+](=N2)[C@H]3[C@@H]([C@H]([C@@H]([C@H](O3)C(=O)O)O)O)O)N)N
InChI	InChI=1S/C15H15Cl2N5O6/c16-5-3-1-2-4(6(5)17)7-12(18)20-15(19)22(21-7)13-10(25)8(23)9(24)11(28-13)14(26)27/h1-3,8-11,13,23-25H,(H4,18,19,20,26,27)/p+1/t8-,9-,10+,11-,13+/m0/s1
InChI-Key	IEVMENHZPOWVGO-XPORZQOISA-O
CAS RN	133310-19-7
Metabolite of	Lamotrigine
Detection frequency	100% (15/15 samples)
Detected in	Altenrhein, Monday-Friday<br>Neugut, Monday-Friday<br>Werdhölzli, Monday-Friday
Intensity	E8
Initial confidence level	level 3
Initial confidence score	0.26
Final confidence level	level 1

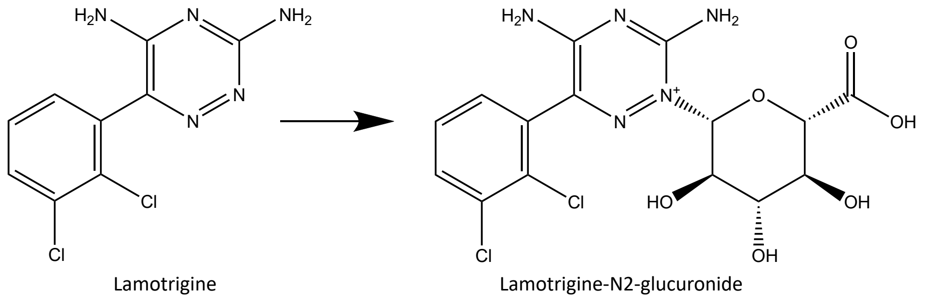

Figure SI-D432: Metabolism of lamotrigine to lamotrigine-N2-glucuronide.

Due to the low quality MS2 spectrum, no SIRIUS/CSI:FingerID evaluation was possible.

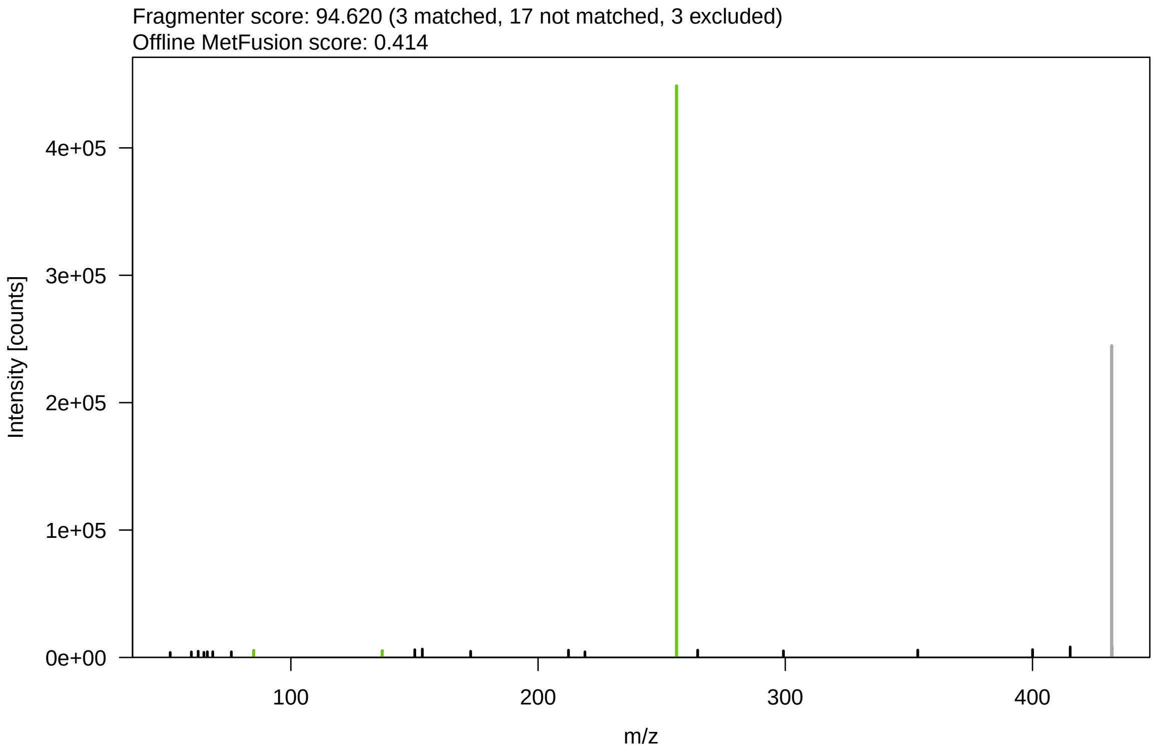

Figure SI-D433: Measured MS2 spectrum. Matching fragments with lamotrigine-N2-glucuronide predicted by MetFrag are highlighted in green. The molecular ion in gray is not considered.

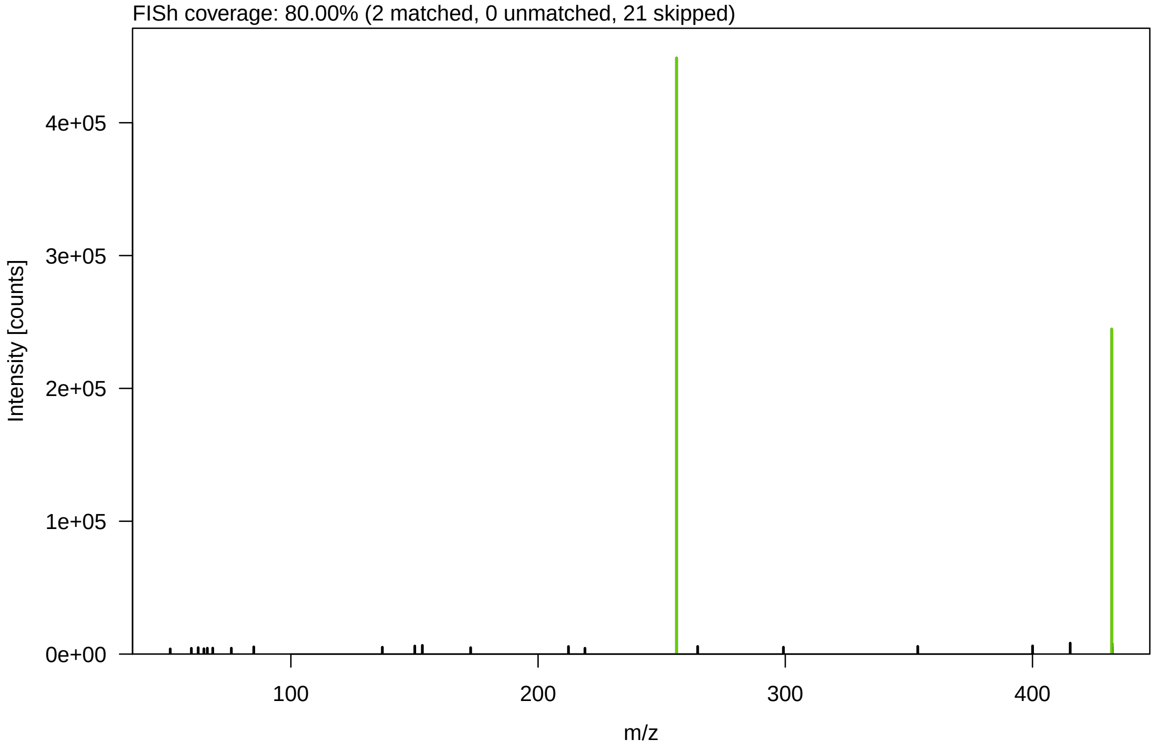

Figure SI-D434: Measured MS2 spectrum. Matching fragments with lamotrigine-N2-glucuronide predicted by FISh Scoring are highlighted in green. Low intensity fragments are not considered and skipped.

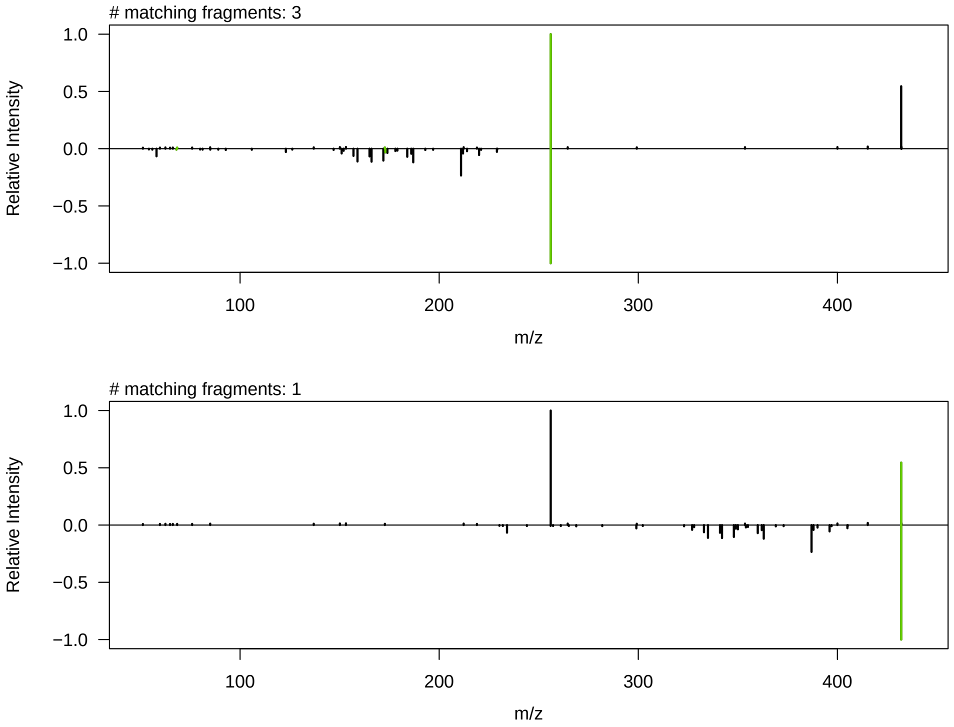

Figure SI-D435: Head to tail plots of lamotrigine-N2-glucuronide and lamotrigine. In the bottom plot, the mass spectrum of lamotrigine is shifted by the mass difference. Matching fragments are highlighted in green.

Table SI-D218: Molecular network results and retention time prediction of lamotrigine-N2-glucuronide.

Measured retention time [min]	11.9
Predicted logD_{OW} (pH = 2.7)	-3.07
Predicted retention time [min]	10.7
Predicted retention time range (95% confidence interval) [min]	6.1-15.3
Predicted retention time range (99% confidence interval) [min]	4.7-16.8

Table SI-D219: Annotated MS2 spectrum of lamotrigine-N2-glucuronide.

m/z	Relative Intensity	Annotation
51.2024	8.69	
59.7512	9.69	
62.5059	10.84	
64.8225	8.96	
66.2216	9.87	
68.4013	10.05	
75.9247	9.87	
84.9931	12.08	$C_3O_3 + H^+$
137.0015	11.50	$C_7H_3ClN + H^+$
150.1282	13.52	
153.1849	14.61	
172.7296	10.78	
212.3019	12.70	
218.9638	9.82	
256.0149	999.00	$C_9H_7Cl_2N_5 + H^+$
264.5625	12.81	
299.2472	11.53	
353.5987	12.90	
400.0262	13.72	
415.2543	18.36	
432.0470	544.69	$C_{15}H_{15}Cl_2N_5O_6 + H^+$
432.1706	17.73	
432.2260	11.79	

A reference standard of lamotrigine-N2-glucuronide was purchased. Figure SI-D436 shows the extracted ion chromatograms of this standard, the sample and the spiked sample, as well as a head to tail plot of the MS2 spectra of the standard and the sample. In addition, the most intense MS2 fragments in the sample and in the standard are displayed. It becomes visible that the retention times of the sample and the spiked sample are identical and the spectra similarity score between sample and standard is equal to 0.793. Only one fragment besides the molecular ion is present in the sample MS2 spectrum, which can be explained by the reference standard. Nonetheless, it can be concluded that the suspected compound is indeed lamotrigine-N2-glucuronide. Correspondingly, the identification confidence can be increased to level 1.

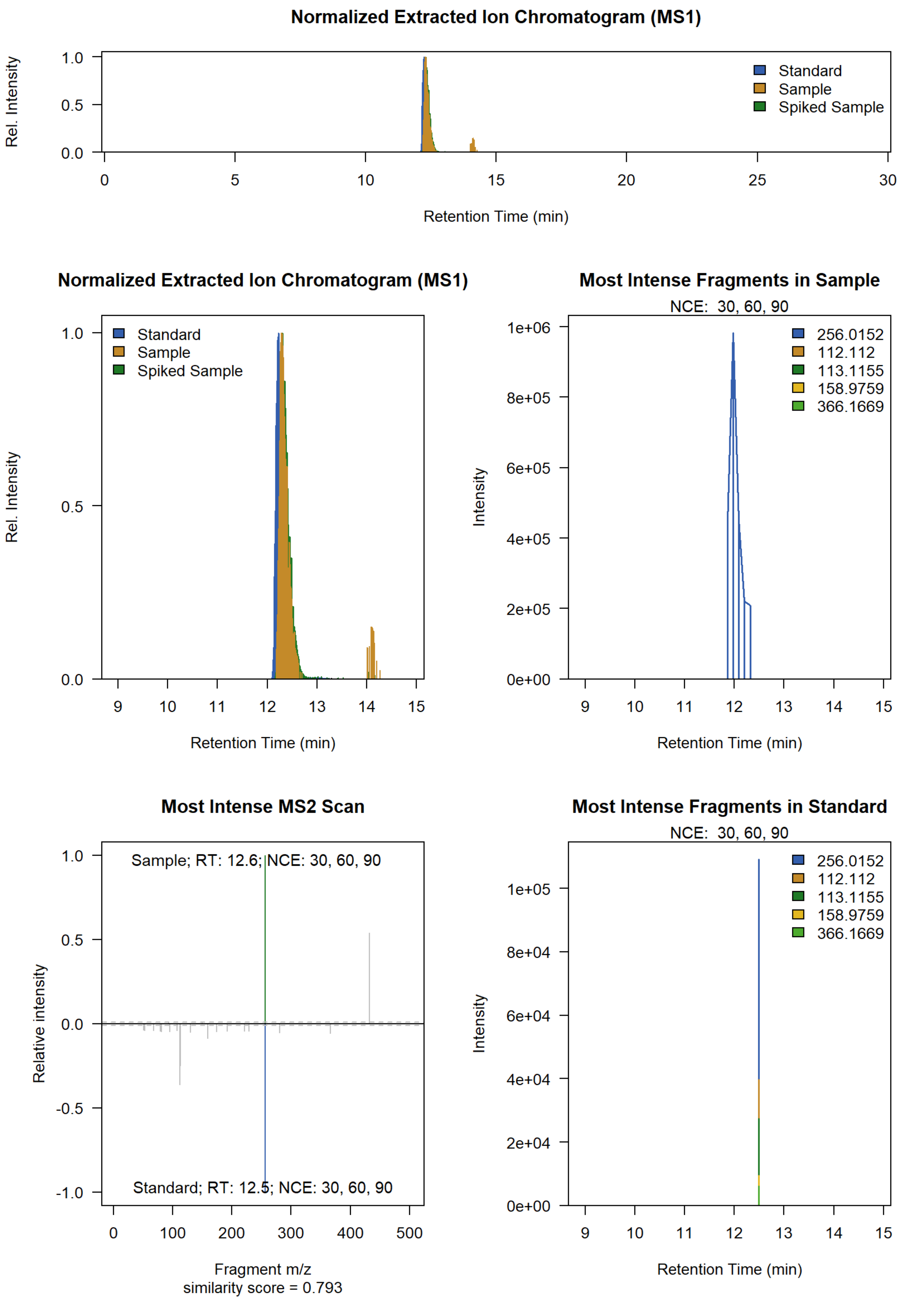

Figure SI-D436: Extracted ion chromatograms of lamotrigine-N2-glucuronide in the reference standard, the sample and the spiked sample, as well as MS2 head to tail plot and most intense MS2 fragments in standard and sample.

SI-D2.15.5 Paracetamol-Sulfate

Paracetamol sulfate is a metabolite of paracetamol, also known as acetaminophen, which is an analgesic drug used for the therapy of pain and as an antipyretic agent.[2] Figure SI-D437 shows the metabolism scheme.

Table SI-D220: Information on identifiers, chemical properties, detection and confidence of identification of paracetamol-sulfate.

IUPAC Name	(4-acetamidophenyl) hydrogen sulfate
Molecular formula	$C_8H_9NO_5S$
Monoisotopic mass [g/mol]	231.0201
Adduct	$[M+H]^+$
Retention time [min]	10.7
SMILES	CC(=O)NC1=CC=C(C=C1)OS(=O)(=O)O
InChI	InChI=1S/C8H9NO5S/c1-6(10)9-7-2-4-8(5-3-7)14-15(11,12)13/h2-5H,1H3,(H,9,10)(H,11,12,13)
InChI-Key	IGTYILLPRJOVFY-UHFFFAOYSA-N
CAS RN	10066-90-7
Metabolite of	Paracetamol
Detection frequency	100% (15/15 samples)
Detected in	Altenrhein, Monday-Friday<br>Neugut, Monday-Friday<br>Werdhölzli, Monday-Friday
Intensity	E7
Initial confidence level	level 3
Initial confidence score	0.42
Final confidence level	level 1

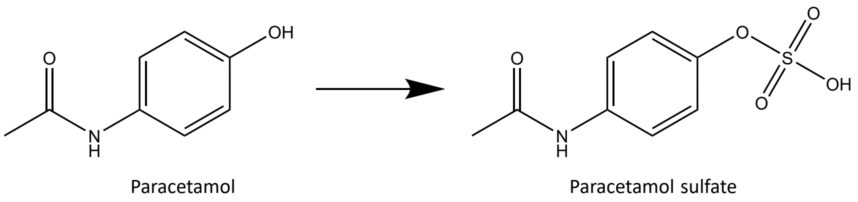

Figure SI-D437: Metabolism of paracetamol to paracetamol-sulfate.

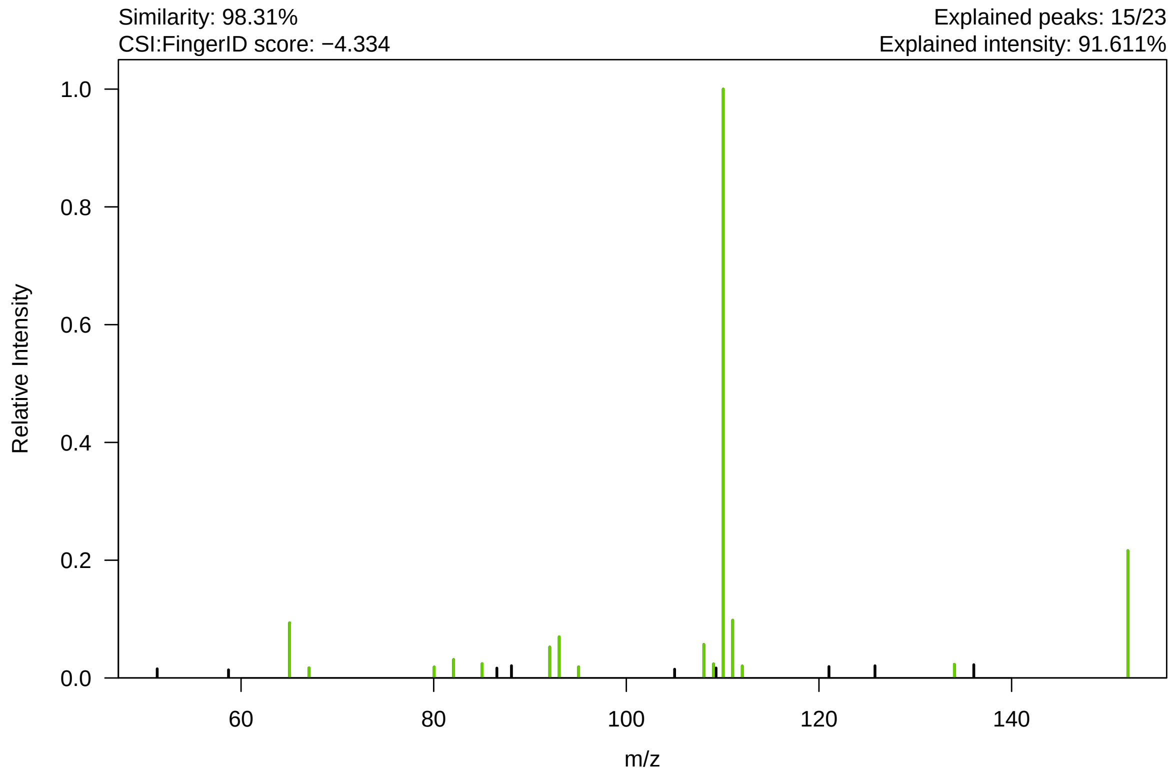

Figure SI-D438: Measured MS2 spectrum. Matching fragments with paracetamol-sulfate predicted by SIRIUS/CSI:FingerID are highlighted in green.

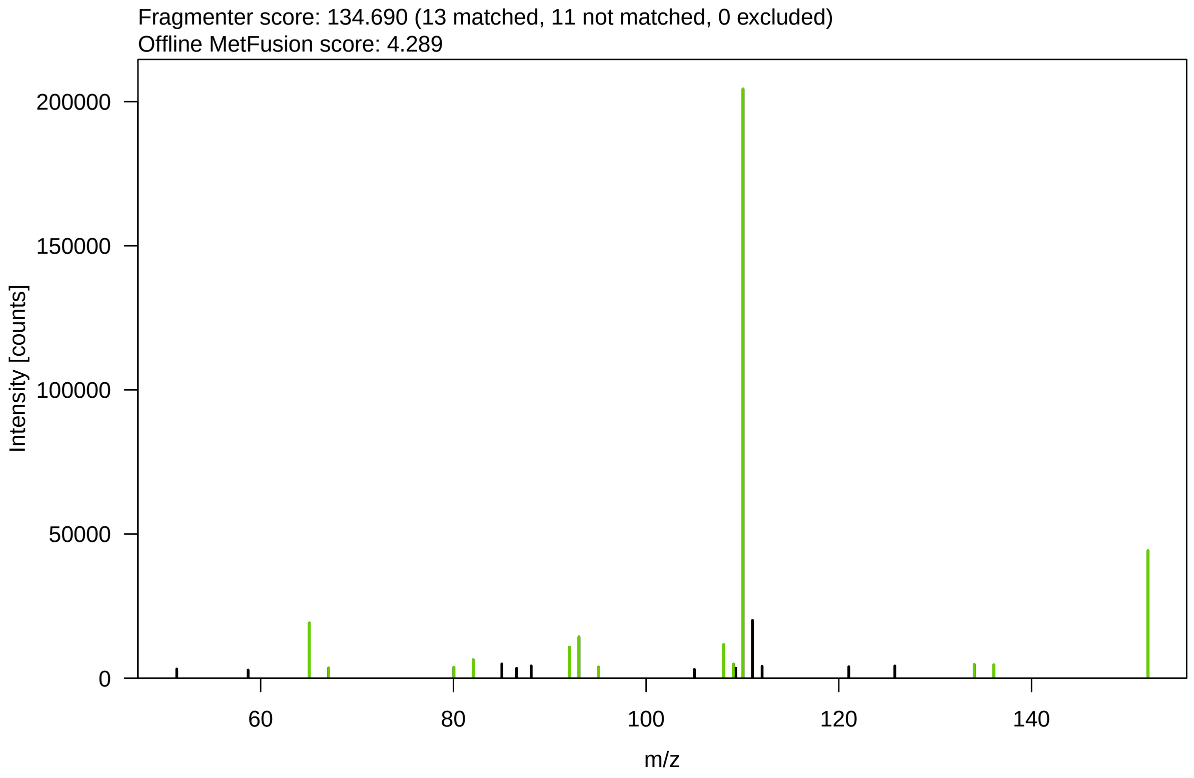

Figure SI-D439: Measured MS2 spectrum. Matching fragments with paracetamol-sulfate predicted by MetFrag are highlighted in green.

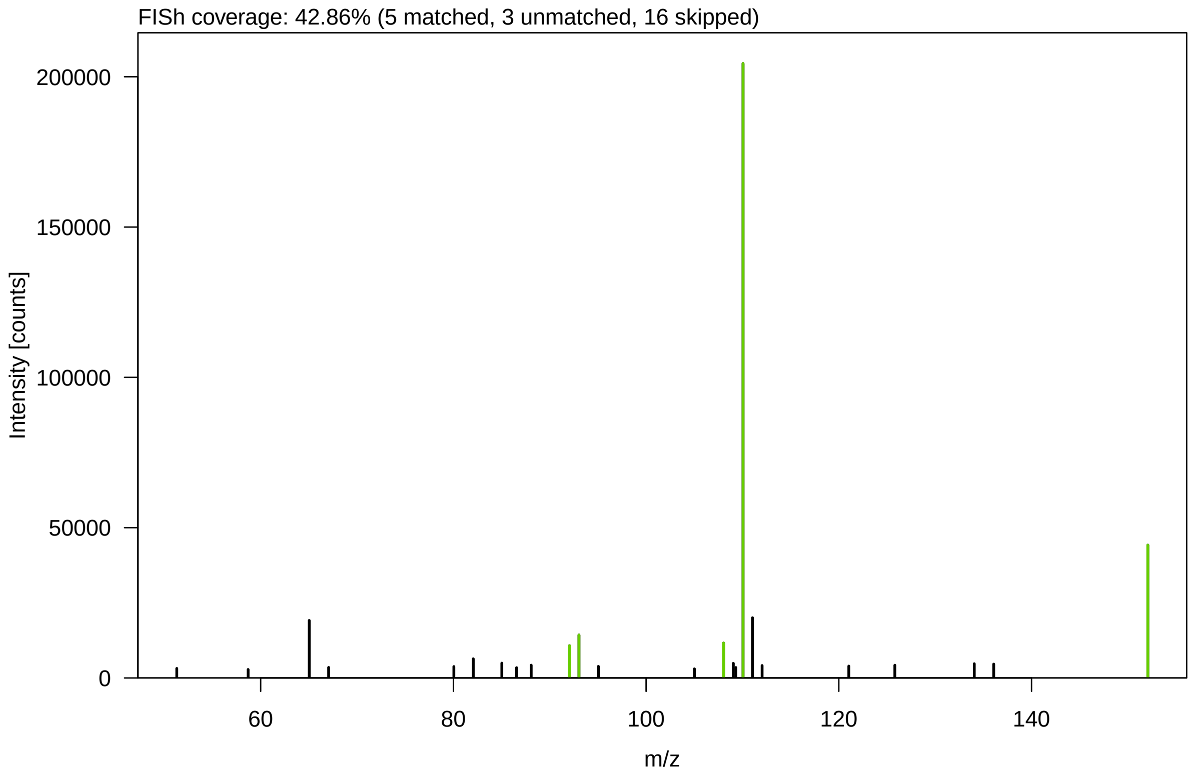

Figure SI-D440: Measured MS2 spectrum. Matching fragments with paracetamol-sulfate predicted by FISh Scoring are highlighted in green. Low intensity fragments are not considered and skipped.

Table SI-D221: Retention time prediction of paracetamol-sulfate.

Measured retention time [min]	10.7
Predicted $\log D_{OW}$ (pH = 2.7)	-1.94
Predicted retention time [min]	12.2
Predicted retention time range (95% confidence interval) [min]	7.6-16.8
Predicted retention time range (99% confidence interval) [min]	6.1-18.3

Table SI-D222: Annotated MS2 spectrum of paracetamol sulfate.

m/z	Relative Intensity	Annotation
51.2947	15.55	
58.6974	13.80	
65.0388	93.54	$C_5H_4 + H^+$
67.0542	17.13	$C_5H_6 + H^+$
80.0498	18.59	$C_5H_5N + H^+$
82.0651	31.21	$C_5H_7N + H^+$
85.0285	24.17	$C_4H_4O_2 + H^+$
86.5600	16.74	
88.0757	20.84	
92.0494	52.44	$C_6H_5N + H^+$
93.0334	69.86	$C_6H_4O + H^+$
95.0496	18.84	$C_6H_6O + H^+$
105.0148	14.86	
108.0442	56.82	$C_6H_5NO + H^+$
109.0522	23.83	$C_6H_6NO + H^+$
109.3138	16.99	
110.0600	999.00	$C_6H_7NO + H^+$
111.0439	97.95	$C_6H_6O_2 + H^+$
112.0391	20.25	$C_5H_5NO_2 + H^+$
121.0391	19.36	
125.8159	20.74	
134.0596	23.16	$C_8H_7NO + H^+$
136.0765	22.54	$C_8H_9NO + H^+$
152.0706	215.97	$C_8H_9NO_2 + H^+$

A reference standard of paracetamol-sulfate was purchased. Figure SI-D441 shows the extracted ion chromatograms of this standard, the sample and the spiked sample, as well as a head to tail plot of the MS2 spectra of the standard and the sample. In addition, the most intense MS2 fragments in the sample and in the standard are displayed. It becomes visible that the retention times of the sample and the spiked sample are identical and the spectra similarity score between sample and standard is equal to 0.950. The majority of the MS2 fragments in the sample can be explained by the reference standard. It can therefore be concluded that the suspected compound is indeed paracetamol-sulfate. Correspondingly, the identification confidence can be increased to level 1.

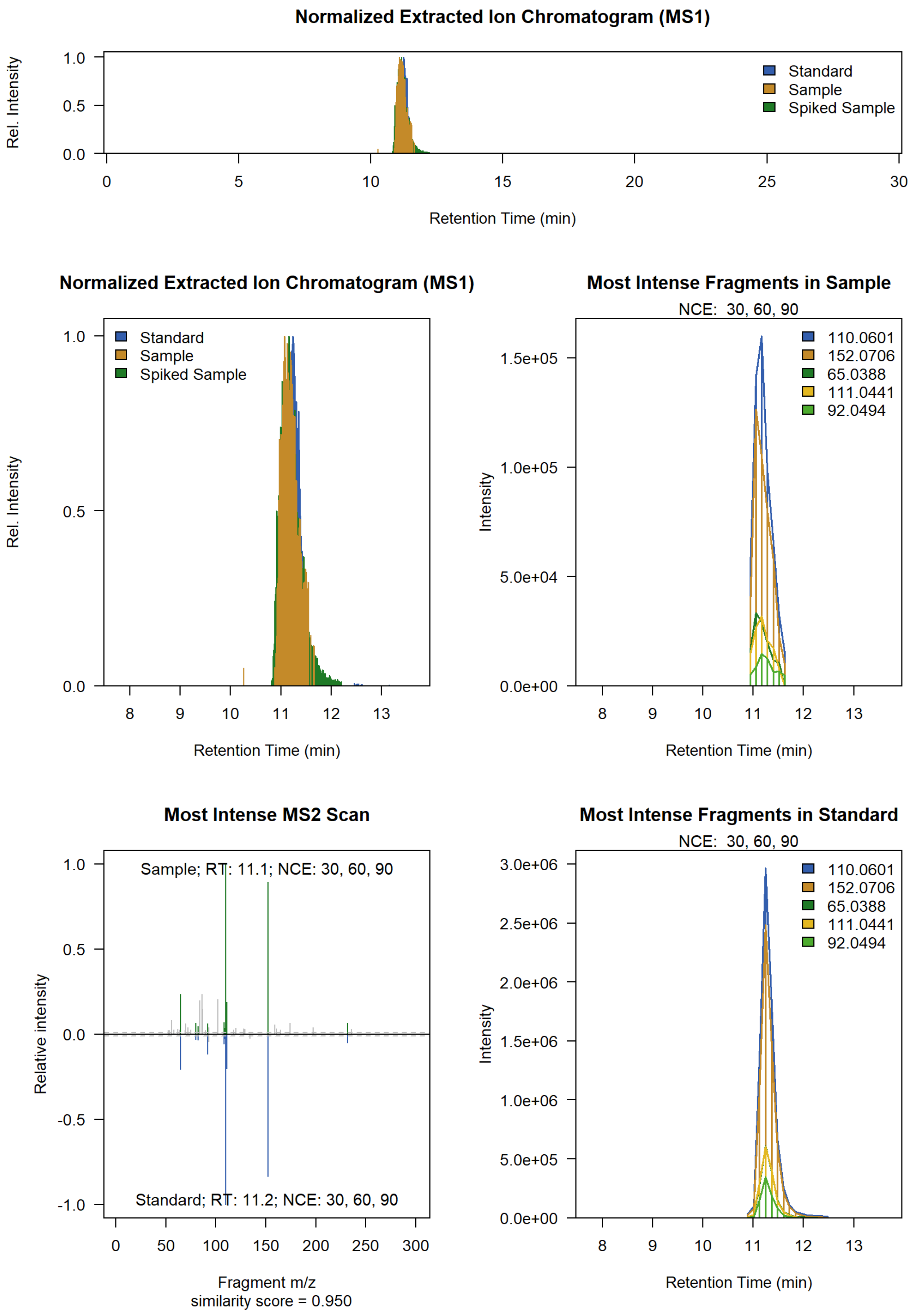

Figure SI-D441: Extracted ion chromatograms of paracetamol sulfate in the reference standard, the sample and the spiked sample, as well as MS2 head to tail plot and most intense MS2 fragments in standard and sample.

SI-D2.15.6 Phenolic-Glucuronide

Phenolic glucuronide is a metabolite of acetylsalicylic acid and carbasalate calcium. Acetylsalicylic acid is used to treat pain, fever, inflammation and migraines. Moreover, it inhibits platelet aggregation and is therefore used in the prevention of myocardial infarction. The same accounts for carbasalate calcium.[2] Figure SI-D442 shows the metabolism scheme.

Table SI-D223: Information on identifiers, chemical properties, detection and confidence of identification of phenolic glucuronide.

IUPAC Name	(2*S*,3*S*,4*S*,5*R*,6*S*)-3,4,5-trihydroxy-6-phenoxyoxane-2-carboxylic acid
Molecular formula	$C_{12}H_{14}O_7$
Monoisotopic mass [g/mol]	270.0740
Adduct	$[M+H]^+$
Retention time [min]	11.1
SMILES	C1=CC=C(C=C1)O[C@H]2[C@@H]([C@H]([C@@H]([C@H](O2)C(=O)O)O)O)O
InChI	InChI=1S/C12H14O7/c13-7-8(14)10(11(16)17)19-12(9(7)15)18-6-4-2-1-3-5-6/h1-5,7-10,12-15H,(H,16,17)/t7-,8-,9+,10-,12+/m0/s1
InChI-Key	WVHAUDNUGBNUDZ-GOVZDWNOSA-N
CAS RN	17685-05-1
Metabolite of	Acetylsalicylic acid, carbasalate calcium
Detection frequency	100% (15/15 samples)
Detected in	Altenrhein, Monday-Friday<br>Neugut, Monday-Friday<br>Werdhölzli, Monday-Friday
Intensity	E8
Initial confidence level	level 3
Initial confidence score	0.54
Final confidence level	level 5

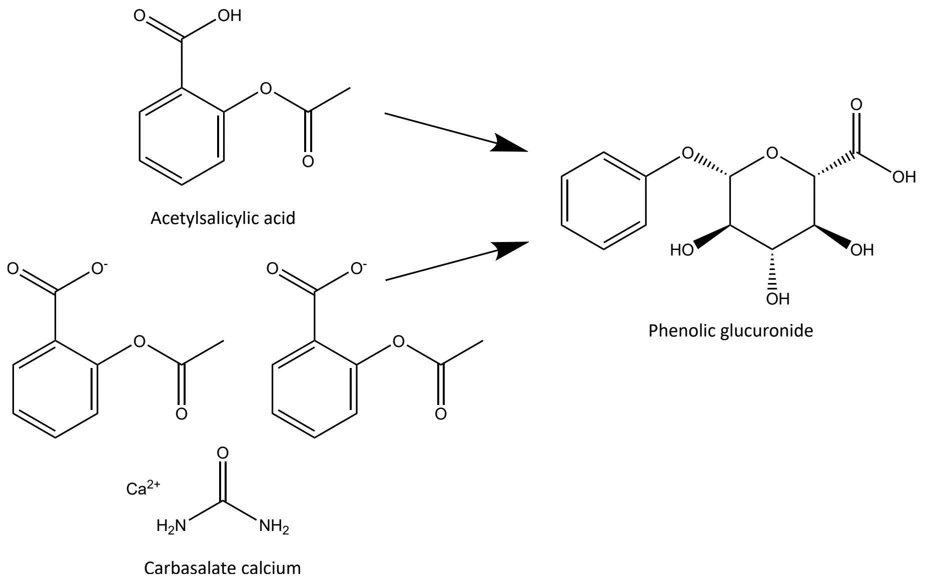

Figure SI-D442: Metabolism of acetylsalicylic acid and carbesalate calcium to phenolic glucuronide.

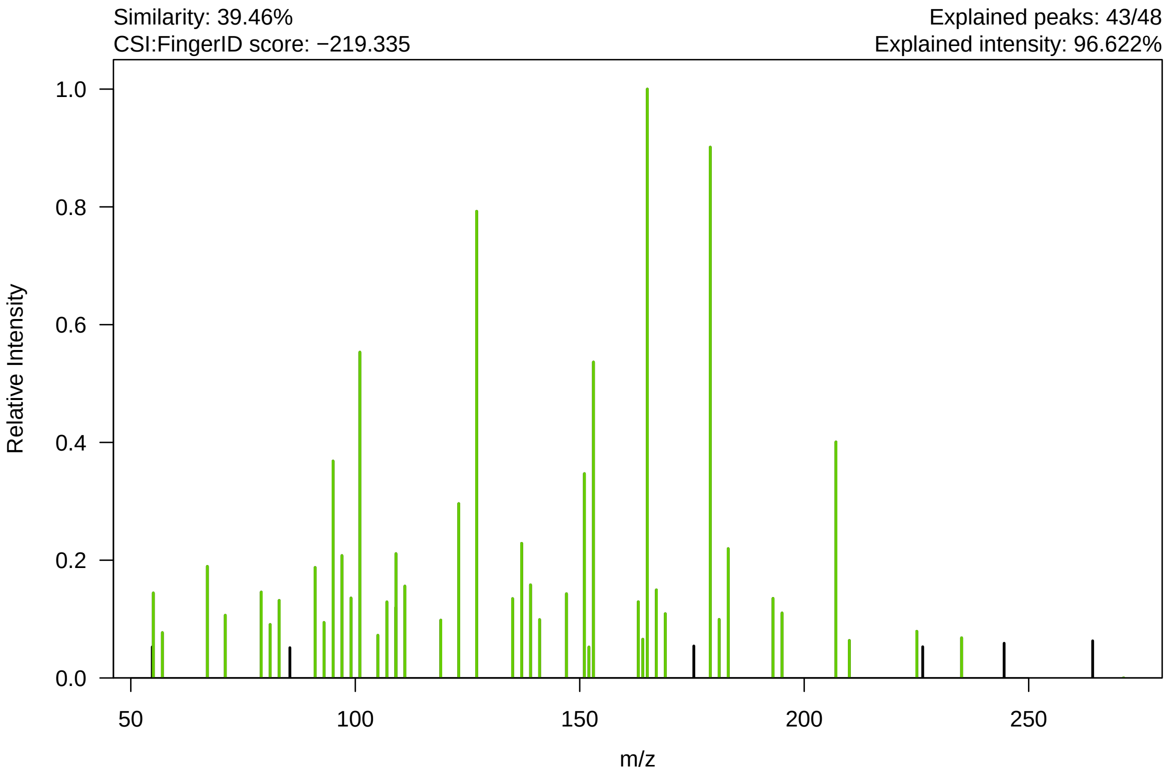

Figure SI-D443: Measured MS2 spectrum. Matching fragments with phenolic glucuronide predicted by SIRIUS/CSI:FingerID are highlighted in green.

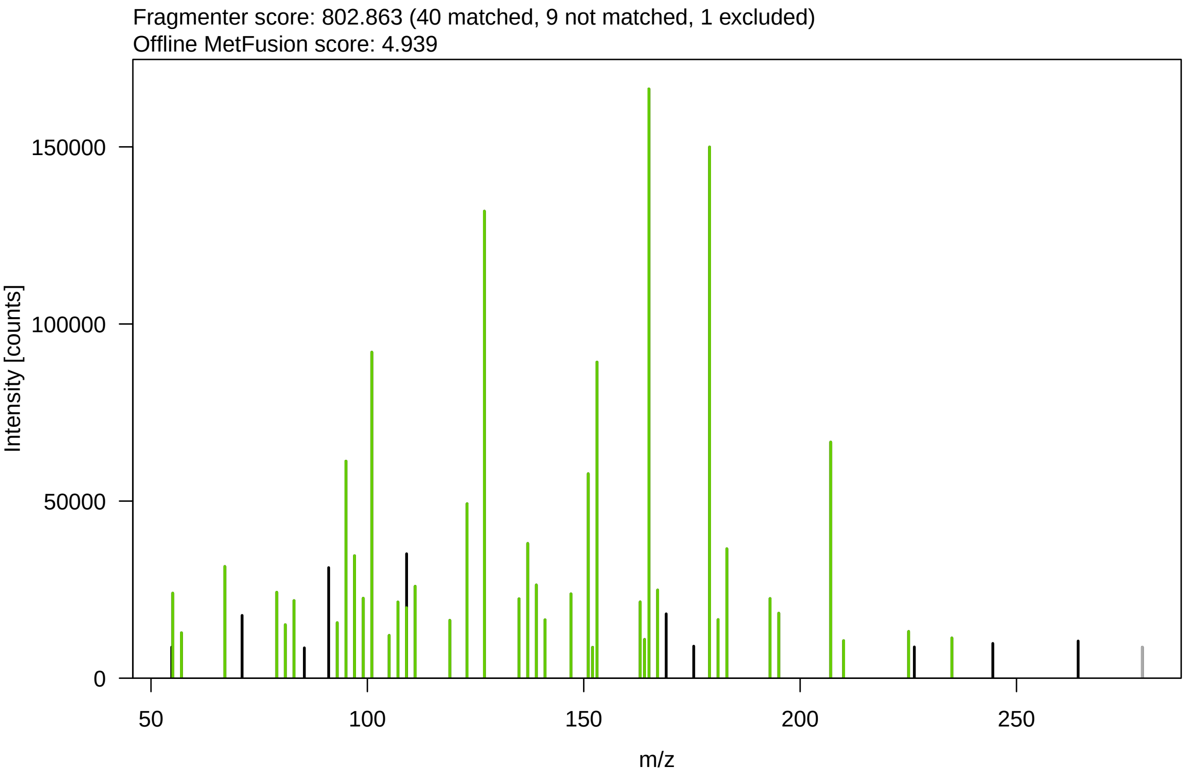

Figure SI-D444: Measured MS2 spectrum. Matching fragments with phenolic glucuronide predicted by MetFrag are highlighted in green. The molecular ion in gray is not considered.

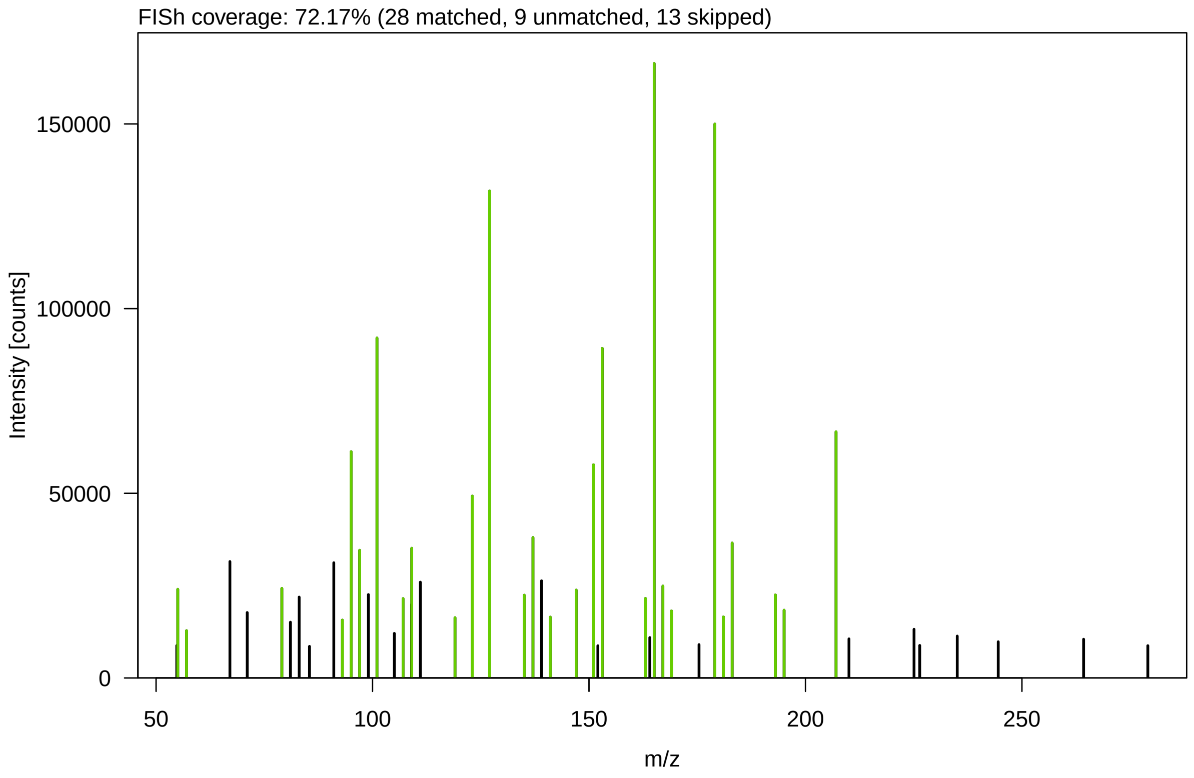

Figure SI-D445: Measured MS2 spectrum. Matching fragments with phenolic glucuronide predicted by FISh Scoring are highlighted in green. Low intensity fragments are not considered and skipped.

Table SI-D224: Retention time prediction of phenolic glucuronide.

Measured retention time [min]	11.1
Predicted logD_{OW} (pH = 2.7)	-0.36
Predicted retention time [min]	14.3
Predicted retention time range (95% confidence interval) [min]	9.7-18.9
Predicted retention time range (99% confidence interval) [min]	8.2-20.3

Table SI-D225: Annotated MS2 spectrum of phenolic glucuronide.

m/z	Relative Intensity	Annotation
54.7830	52.71	
55.0180	144.19	$C_3H_2O + H^+$
57.0338	76.92	$C_3H_4O + H^+$
67.0542	189.34	$C_5H_6 + H^+$
71.0489	106.42	$C_4H_6O + H^+$
79.0539	145.69	$C_6H_6 + H^+$
81.0334	90.69	$C_5H_4O + H^+$
83.0488	131.51	$C_5H_6O + H^+$
85.4335	51.38	
91.0541	187.39	$C_7H_6 + H^+$
93.0331	94.08	$C_6H_4O + H^+$
95.0489	367.96	$C_6H_6O + H^+$
97.0282	207.61	$C_5H_4O_2 + H^+$
99.0437	135.60	$C_5H_6O_2 + H^+$
101.0233	552.71	$C_4H_4O_3 + H^+$
105.0336	72.48	$C_7H_4O + H^+$
107.0489	129.08	$C_7H_6O + H^+$
109.0281	119.60	$C_6H_4O_2 + H^+$;
109.0646	210.91	$C_7H_8O + H^+$
111.0437	155.81	$C_6H_6O_2 + H^+$
119.0492	98.06	$C_8H_6O + H^+$
123.0440	295.73	$C_7H_6O_2 + H^+$
127.0389	791.73	$C_6H_6O_3 + H^+$
135.0439	134.61	$C_8H_6O_2 + H^+$
137.0594	228.37	$C_8H_8O_2 + H^+$
139.0388	158.00	$C_7H_6O_3 + H^+$
141.0541	98.97	$C_7H_8O_3 + H^+$
147.0435	142.89	$C_9H_6O_2 + H^+$
151.0390	346.60	$C_8H_6O_3 + H^+$
152.0463	52.42	$C_8H_7O_3 + H^+$
153.0547	535.89	$C_8H_8O_3 + H^+$
163.0387	129.29	$C_9H_6O_3 + H^+$
164.0462	65.72	$C_9H_7O_3 + H^+$
165.0544	999.00	$C_9H_8O_3 + H^+$
167.0697	149.42	$C_9H_{10}O_3 + H^+$
169.0493	109.03	$C_8H_8O_4 + H^+$

Continued on next page

Table SI-D225: Annotated MS2 spectrum of phenolic glucuronide.(Continued)

175.4113	54.26	
179.0700	900.52	$C_{10}H_{10}O_3 + H^+$
181.0490	99.27	$C_9H_8O_4 + H^+$
183.0650	219.39	$C_9H_{10}O_4 + H^+$
193.0504	135.00	$C_{10}H_8O_4 + H^+$
195.0649	110.20	$C_{10}H_{10}O_4 + H^+$
207.0652	400.32	$C_{11}H_{10}O_4 + H^+$
210.0524	63.60	$C_{10}H_9O_5 + H^+$
225.0770	79.18	$C_{11}H_{12}O_5 + H^+$
226.3949	52.92	
235.0589	68.14	$C_{12}H_{10}O_5 1\,H^+$
244.5292	58.90	
264.2503	62.99	
279.0832	52.53	

A reference standard of phenolic glucuronide was purchased. However, is was neither detectable in the positive nor in the negative ionization mode. This leads to the hypothesis, that phenolic glucuronide is not stable in aqueous solutions and dissociates into phenol and glucuornide. As a consequence, the suspected compound cannot be confirmed as phenolic glucuronide and the confidence level is decreased to level 5.

SI-D2.15.7 Phenylephrine-3-O-Sulfate

Phenylephrine-3-O-sulfate is a metabolite of phenylephrine, which is an α_1 adrenergic receptor agonist used to treat hypotension, to dilate the pupil and to induce local vasoconstriction.[2] Figure SI-D446 shows the metabolism scheme and Figure SI-D447 the phenylephrine cluster.

Table SI-D226: Information on identifiers, chemical properties, detection and confidence of identification of phenylephrine-3-O-sulfate.

IUPAC Name	[3-[(1*R*)-1-hydroxy-2-(methylamino)ethyl]phenyl] hydrogen sulfate
Molecular formula	$C_9H_{13}NO_5S$
Monoisotopic mass [g/mol]	247.0514
Adduct	$[M+H]^+$
Retention time [min]	12.3
SMILES	CNC[C@@H](C1=CC(=CC=C1)OS(=O)(=O)O)O
InChI	InChI=1S/C9H13NO5S/c1-10-6-9(11)7-3-2-4-8(5-7)15-16(12,13)14/h2-5,9-11H,6H2,1H3,(H,12,13,14)/t9-/m0/s1
InChI-Key	RNPCQQLCTVIPNU-VIFPVBQESA-N
CAS RN	1242184-39-9
Metabolite of	Phenylephrine
Detection frequency	100% (15/15 samples)
Detected in	Altenrhein, Monday-Friday<br>Neugut, Monday-Friday<br>Werdhölzli, Monday-Friday
Intensity	E7
Initial confidence level	level 3
Initial confidence score	0.49
Final confidence level	level 4

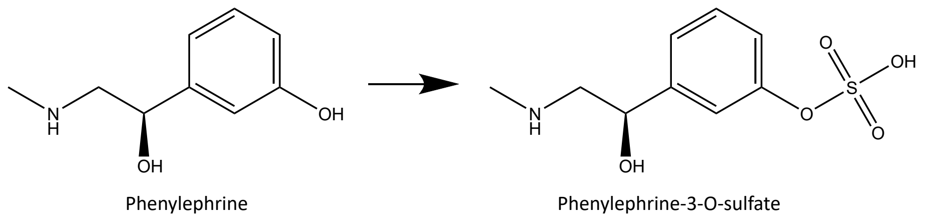

Figure SI-D446: Metabolism of phenylephrine to phenylephrine-3-O-sulfate.

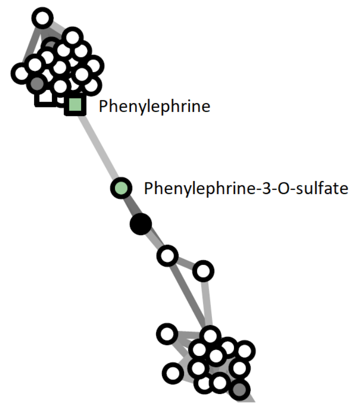

Figure SI-D447: Excerpt of the molecular network showing the phenylephrine cluster.

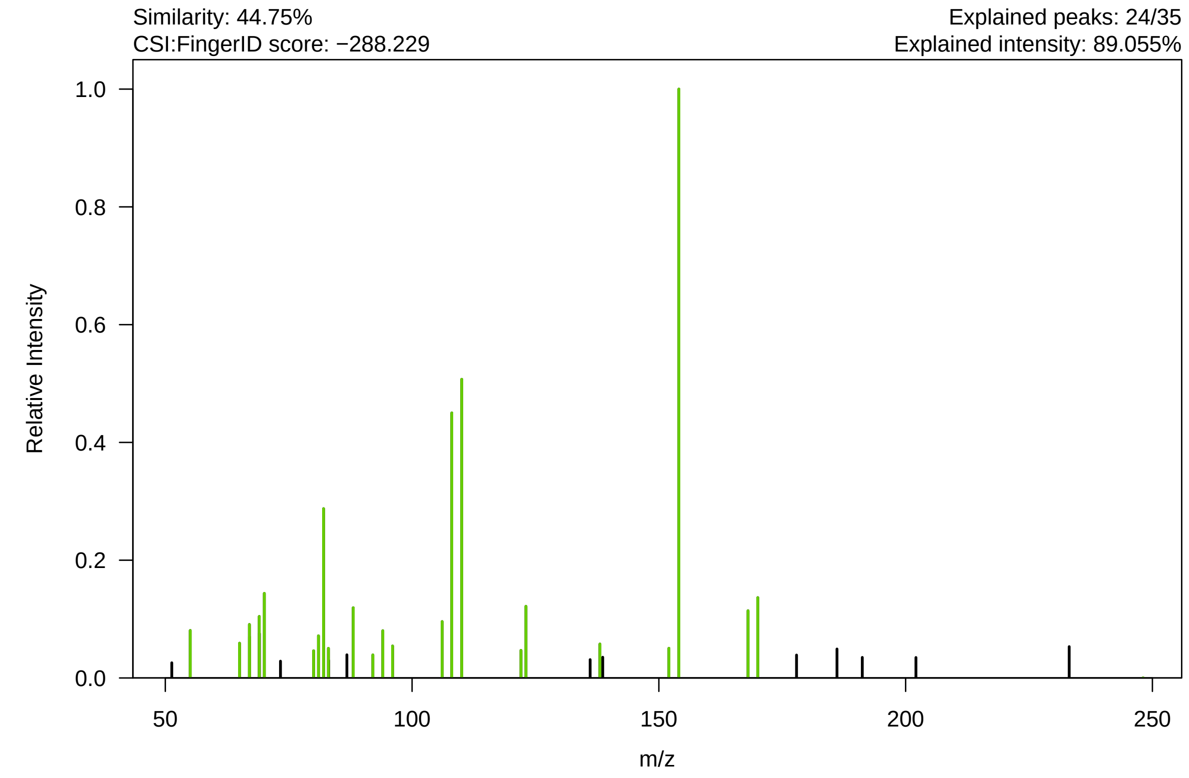

Figure SI-D448: Measured MS2 spectrum. Matching fragments with phenylephrine-3-O-sulfate predicted by SIRIUS/CSI:FingerID are highlighted in green.

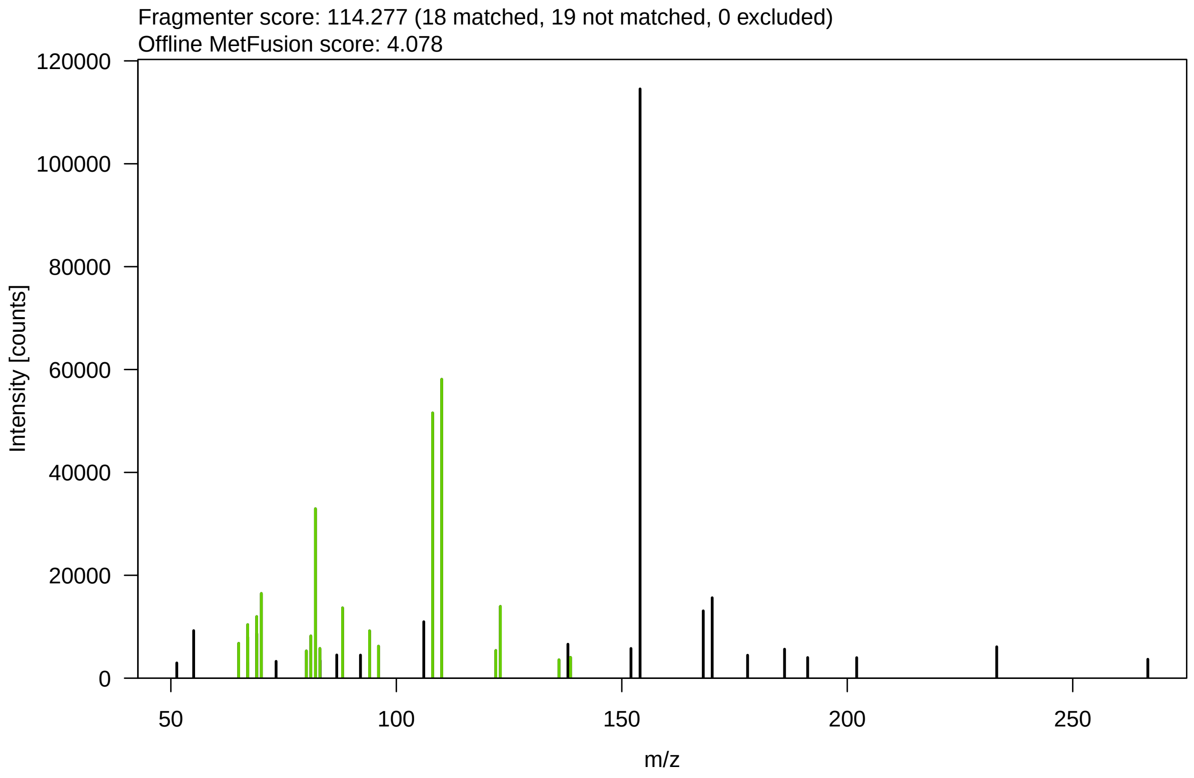

Figure SI-D449: Measured MS2 spectrum. Matching fragments with phenylephrine-3-O-sulfate predicted by MetFrag are highlighted in green.

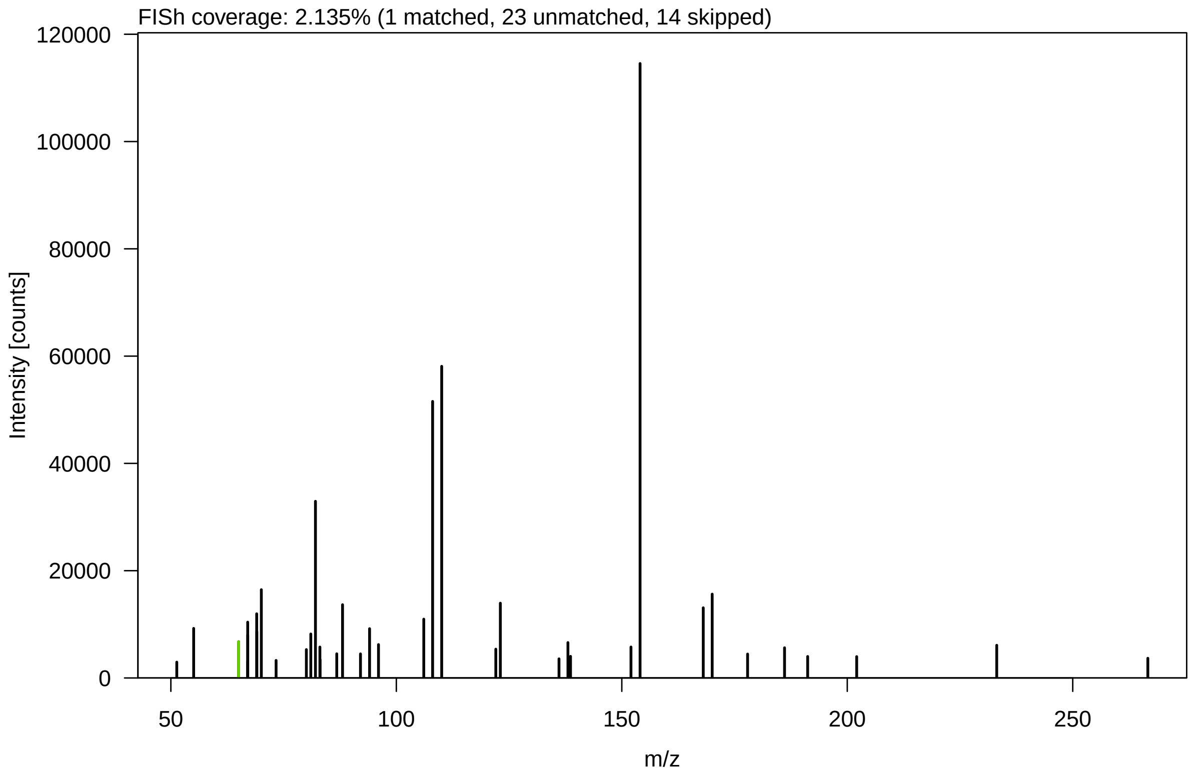

Figure SI-D450: Measured MS2 spectrum. Matching fragments with phenylephrine-3-O-sulfate predicted by FISh Scoring are highlighted in green. Low intensity fragments are not considered and skipped.

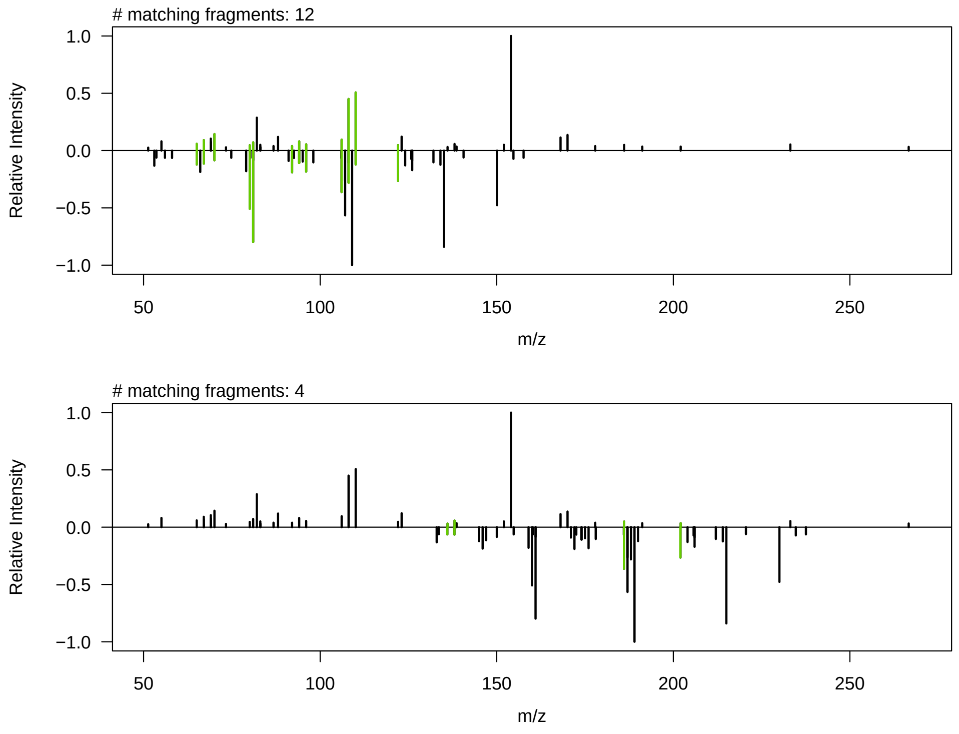

Figure SI-D451: Head to tail plots of phenylephrine-3-O-sulfate and phenylephrine. In the bottom plot, the mass spectrum of phenylephrine is shifted by the mass difference. Matching fragments are highlighted in green.

Table SI-D227: Molecular network results and retention time prediction of phenylephrine-3-O-sulfate.

Comparison with	Phenylephrine
MSn Score	26
Forward coverage	17
Reverse coverage	35
Forward match	8
Reverse match	17
ΔMass [g/mol]	79.9568
Measured retention time [min]	12.3
Predicted $logD_{OW}$ (pH = 2.7)	-1.10
Predicted retention time [min]	13.3
Predicted retention time range (95% confidence interval) [min]	8.7-17.9
Predicted retention time range (99% confidence interval) [min]	7.3-19.4

Table SI-D228: Annotated MS2 spectrum of phenylephrine-3-O-sulfate.

m/z	Relative Intensity	Annotation
51.3124	25.80	
55.0546	80.61	$C_4H_6 + H^+$
65.0386	58.99	$C_5H_4 + H^+$
67.0418	90.78	$C_4H_4N + H^+$
67.0454	26.65	
67.0544	69.80	$C_5H_6 + H^+$
69.0336	104.37	$C_4H_4O + H^+$
69.0698	75.48	
70.0651	143.58	$C_4H_7N + H^+$
73.3366	28.53	
80.0493	46.15	$C_5H_5N + H^+$
81.0334	71.53	$C_5H_4O + H^+$
82.0652	287.24	$C_5H_7N + H^+$
83.0490	50.29	$C_5H_6O + H^+$
83.0860	29.82	
86.7897	39.35	
88.0754	119.15	$C_4H_9NO + H^+$
92.0494	39.14	$C_6H_5N + H^+$
94.0650	80.19	$C_6H_7N + H^+$
96.0446	54.29	$C_5H_5NO + H^+$
106.0862	95.67	$C_4H_{11}NO_2 + H^+$
108.0443	449.67	$C_6H_5NO + H^+$
110.0601	506.69	$C_6H_7NO + H^+$

Continued on next page

Table SI-D228: Annotated MS2 spectrum of phenylephrine-3-O-sulfate.(Continued)

122.0603	46.86	$C_7H_7NO + H^+$
123.0677	121.61	$C_7H_8NO + H^+$
136.0744	31.14	$C_8H_9NO + H^+$
138.0547	57.58	$C_7H_7NO_2 + H^+$
138.6279	35.18	
152.0341	50.35	$C_7H_5NO_3 + H^+$
154.0498	999.00	$C_7H_7NO_3 + H^+$
168.0655	114.11	$C_8H_9NO_3 + H^+$
170.0449	136.32	$C_7H_7NO_4 + H^+$
177.8946	38.90	
186.0906	49.15	
191.2170	34.92	
202.0864	34.73	
233.1410	53.16	
266.6526	32.05	

A reference standard of phenylephrine-3-O-sulfate was purchased. Figure SI-D452 shows the extracted ion chromatograms of this standard, the sample and the spiked sample. It becomes visible that the suspectd compound elutes more than five minutes later than the reference standard of phenylephrine-3-O-sulfate. It can therefore be concluded that the suspected compound is not phenylephrine-3-O-sulfate. Correspondingly, the identification confidence has to be decreased to level 4 due to the unequivocal molecular formula.

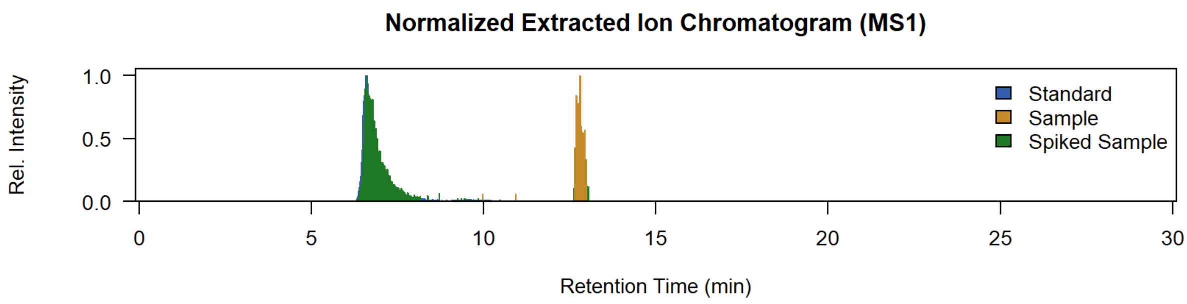

Figure SI-D452: Extracted ion chromatograms of phenylephrine-3-O-sulfate in the reference standard and the suspected compound in the sample and the spiked sample.

SI-D2.15.8 Tapentadol-O-Sulfate

Tapentadol-O-sulfate is metabolite of tapentadol, which is an opioid used to treat severe pain that has not repsonded to non-opioid medications.[2] Figure SI-D453 shows the metabolism scheme.

Table SI-D229: Information on identifiers, chemical properties, detection and confidence of identification of tapentadol-O-sulfate.

IUPAC Name	[3-[(2*R*,3*R*)-1-(dimethylamino)-2-methylpentan-3-yl]phenyl] hydrogen sulfate
Molecular formula	$C_{14}H_{23}NO_4S$
Monoisotopic mass [g/mol]	301.1348
Adduct	$[M+H]^+$
Retention time [min]	14.7
SMILES	CC[C@@H](C1=CC(=CC=C1)OS(=O)(=O)O)[C@@H](C)CN(C)C
InChI	InChI=1S/C14H23NO4S/c1-5-14(11(2)10-15(3)4)12-7-6-8-13(9-12)19-20(16,17)18/h6-9,11,14H,5,10H2,1-4H3,(H,16,17,18)/t11-,14+/m0/s1
InChI-Key	HPEFZESOUVTBSA-SMDDNHRTSA-N
CAS RN	1300037-87-9
Metabolite of	Tapentadol
Detection frequency	100% (15/15 samples)
Detected in	Altenrhein, Monday-Friday<br>Neugut, Monday-Friday<br>Werdhölzli, Monday-Friday
Intensity	E6-E7
Initial confidence level	level 3
Initial confidence score	0.35
Final confidence level	level 1

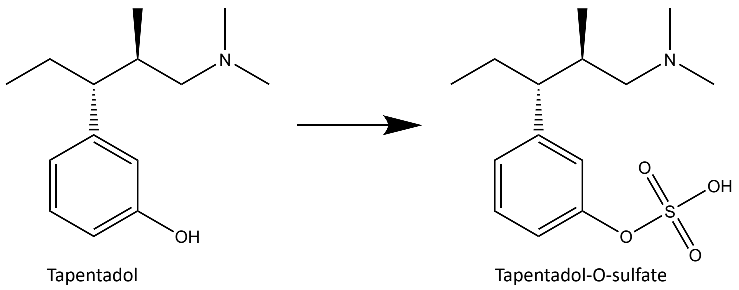

Figure SI-D453: Metabolism of tapentadol to tapentadol-O-sulfate.

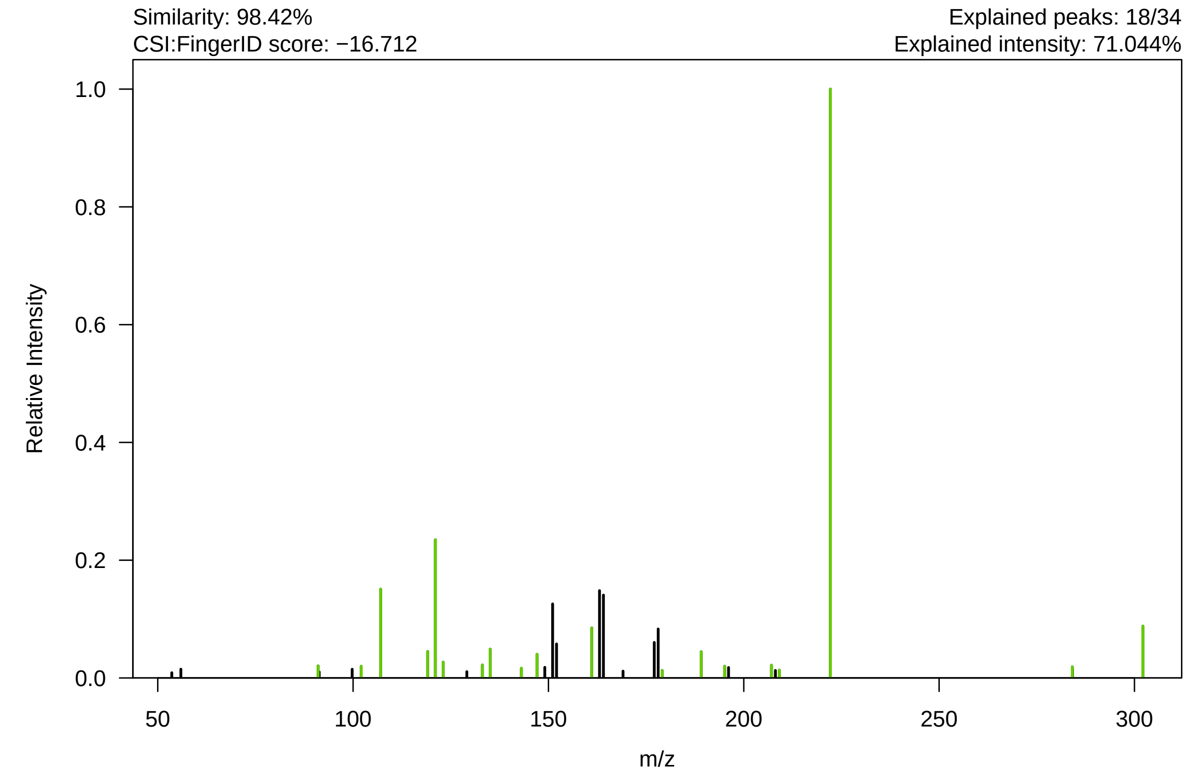

Figure SI-D454: Measured MS2 spectrum. Matching fragments with tapentadol-O-sulfate predicted by SIRIUS/CSI:FingerID are highlighted in green. The molecular ion in gray is not considered.

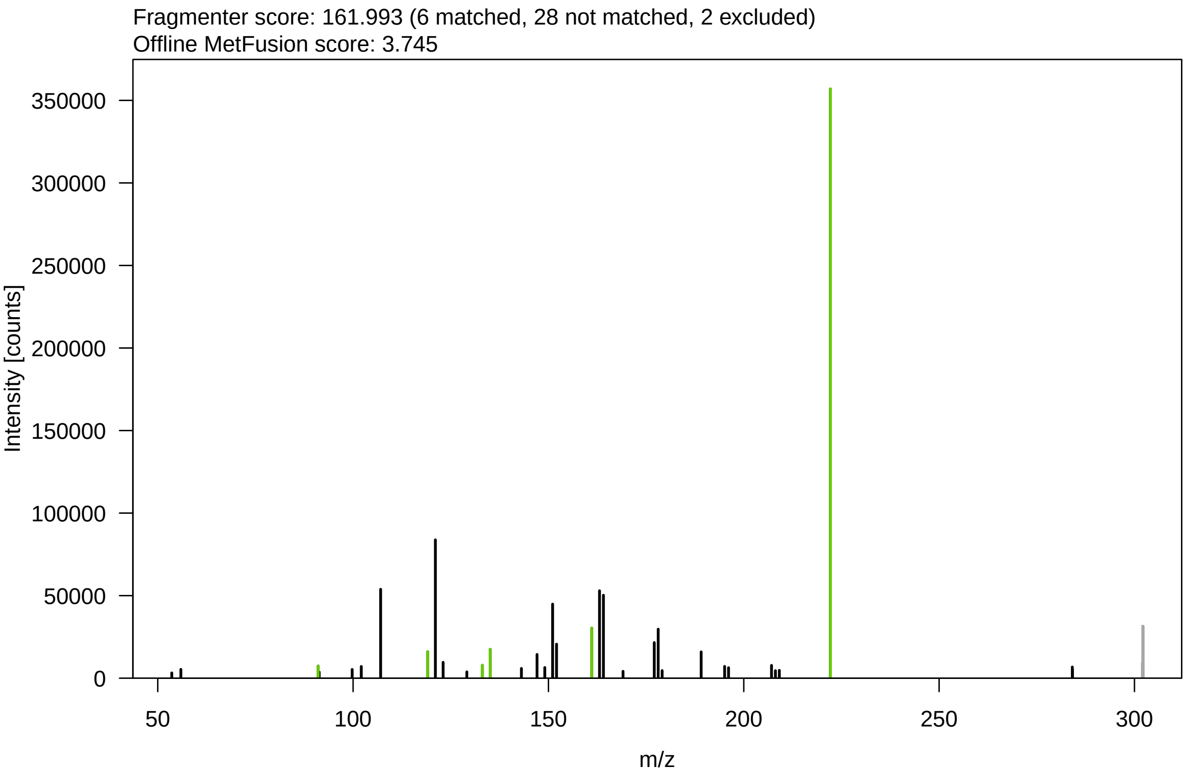

Figure SI-D455: Measured MS2 spectrum. Matching fragments with tapentadol-O-sulfate predicted by MetFrag are highlighted in green. The molecular ion in gray is not considered.

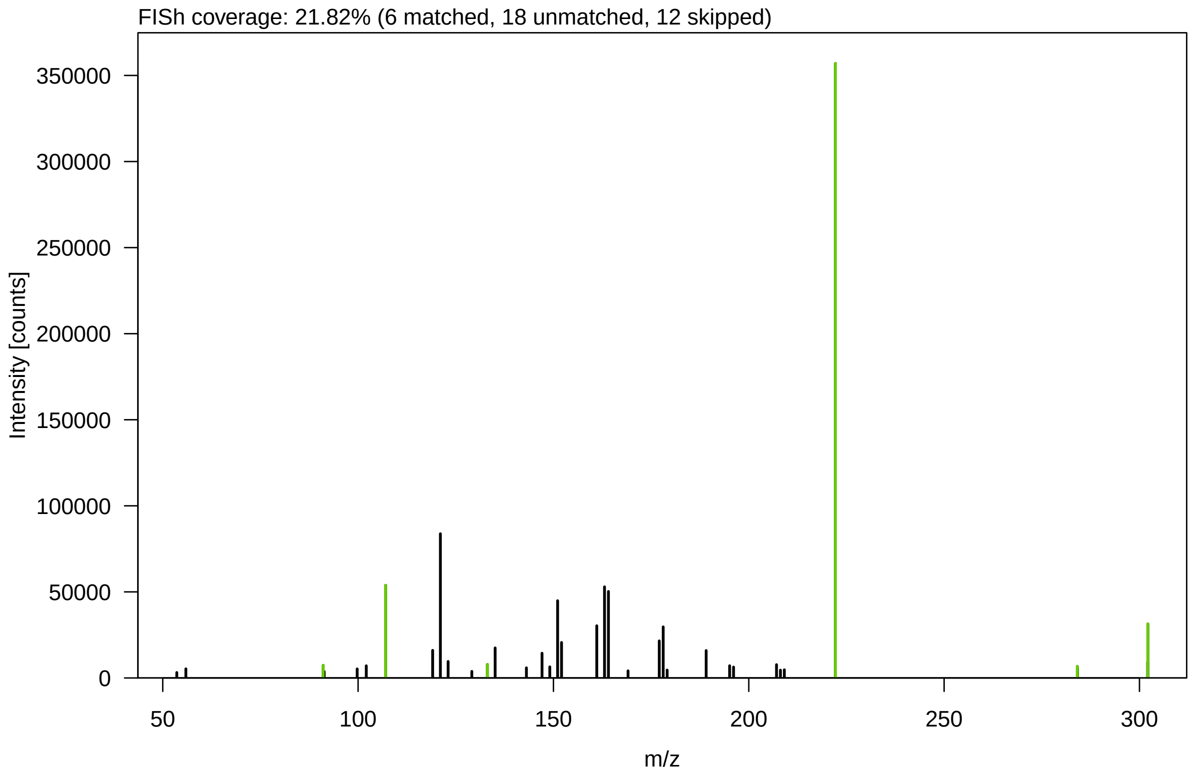

Figure SI-D456: Measured MS2 spectrum. Matching fragments with tapentadol-O-sulfate predicted by FISh Scoring are highlighted in green. Low intensity fragments are not considered and skipped.

Table SI-D230: Retention time prediction of tapentadol-O-sulfate.

Measured retention time [min]	14.7
Predicted $\log D_{OW}$ (pH = 2.7)	1.49
Predicted retention time [min]	16.7
Predicted retention time range (95% confidence interval) [min]	12.1-21.3
Predicted retention time range (99% confidence interval) [min]	10.6-22.7

Table SI-D231: Annotated MS2 spectrum of tapentadol-O-sulfate.

m/z	Relative Intensity	Annotation
53.5923	8.89	
55.9138	14.88	
91.0544	20.58	$C_7H_6 + H^+$
91.3122	10.40	
99.7658	14.79	
102.0914	19.82	$C_5H_{11}NO + H^+$
107.0492	150.65	$C_7H_6O + H^+$
119.0853	44.89	$C_9H_{10} + H^+$

Continued on next page

Table SI-D231: Annotated MS2 spectrum of tapentadol-O-sulfate.(Continued)

121.0646	234.51	$C_8H_8O + H^+$
123.0438	26.86	$C_7H_6O_2 + H^+$
129.1118	10.72	$C_{10}H_{12} + H^+$
133.1012	22.02	$C_{10}H_{12} + H^+$
135.0803	48.91	$C_9H_{10}O + H^+$
143.0854	16.49	$C_{11}H_{10} + H^+$
147.0803	40.23	$C_{10}H_{10}O + H^+$
149.0703	18.06	
151.0726	125.60	
152.0771	57.73	
161.0957	84.89	$C_{11}H_{12}O + H^+$
163.0721	148.17	
164.0773	140.63	
169.0965	11.58	
177.0882	60.32	
178.0919	83.07	
179.1064	12.85	$C_{11}H_{14}O_2 + H^+$
189.0911	44.59	$C_{12}H_{12}O_2 + H^+$
195.0994	19.91	$C_{11}H_{14}O_3 + H^+$
196.1018	17.73	
207.1001	21.67	$C_{12}H_{14}O_3 + H^+$
208.1013	12.72	
209.0982	13.30	$C_{12}H_{16}OS + H^+$
222.1851	999.00	$C_{14}H_{23}NO + H^+$
284.0933	18.92	$C_{13}H_{17}NO_4S + H^+$
284.1274	14.67	
302.1081	25.72	
302.1415	87.95	$C_{14}H_{23}NO_4S + H^+$

A reference standard of tapentadol-O-sulfate was purchased. Figure SI-D457 shows the extracted ion chromatograms of this standard, the sample and the spiked sample, as well as a head to tail plot of the MS2 spectra of the standard and the sample. In addition, the most intense MS2 fragments in the sample and in the standard are displayed. It becomes visible that the retention times of the sample and the spiked sample are identical and the spectra similarity score between sample and standard is equal to 0.648. The most intense of the MS2 fragments in the sample can be explained by the reference standard. It can therefore be concluded that the suspected compound is indeed tapentadol-O-sulfate. Correspondingly, the identification confidence can be increased to level 1.

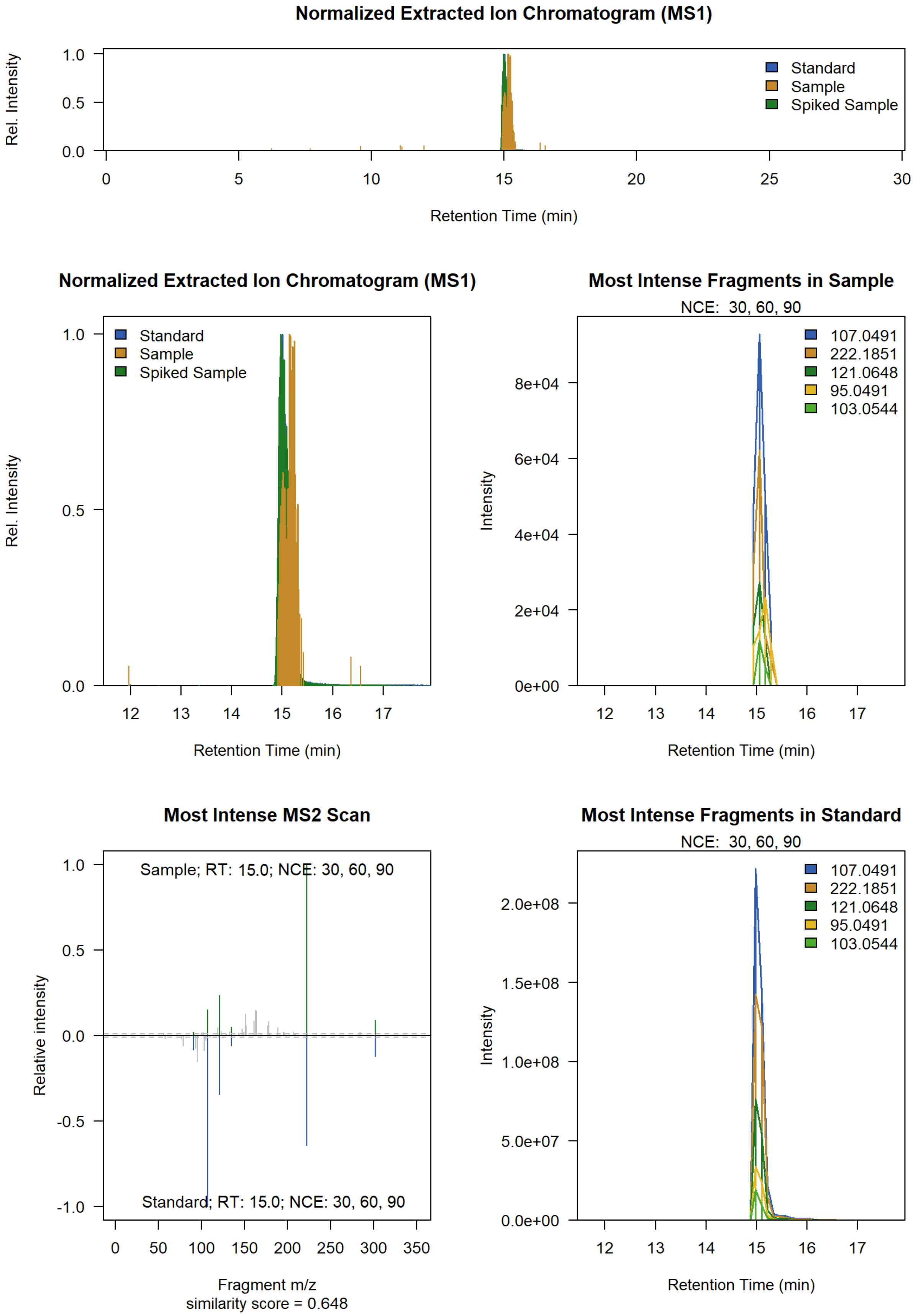

Figure SI-D457: Extracted ion chromatograms of tapentadol-O-sulfate in the reference standard, the sample and the spiked sample, as well as MS2 head to tail plot and most intense MS2 fragments in standard and sample.

SI-D2.15.9 Telmisartan-O-Acyl-Glucuronide

Telmisartan-O-acyl-glucuronide is a phase II metabolite of telmisartan, an angiotesin II receptor antagonist used to treat hypertension, diabetic nephropathy and congestive heart failure.[2] Figure SI-D458 shows the metabolism scheme.

Table SI-D232: Information on identifiers, chemical properties, detection and confidence of identification of telmisartan-O-acyl-glucuronide.

IUPAC Name	(2S,3*S*,4*S*,5*R*,6*S*)-3,4,5-trihydroxy-6-[2-[4-[[4-methyl-6-(1-methylbenzimidazol-2-yl)-2-propylbenzimidazol-1-yl]methyl]phenyl]benzoyl]oxyoxane-2-carboxylic acid
Molecular formula	$C_{39}H_{38}N_4O_8$
Monoisotopic mass [g/mol]	690.2690
Adduct	$[M\text{-}H]^-$
Retention time [min]	15.2
SMILES	CCCC1=NC2=C(N1CC3=CC=C(C=C3)C4=CC=CC=C4C(=O)O[C@H]5[C@@H]([C@H]([C@@H]([C@H](O5)C(=O)O)O)O)O)C=C(C=C2C)C6=NC7=CC=CC=C7N6C
InChI	InChI=1S/C39H38N4O8/c1-4-9-30-41-31-21(2)18-24(36-40-27-12-7-8-13-28(27)42(36)3)19-29(31)43(30)20-22-14-16-23(17-15-22)25-10-5-6-11-26(25)38(49)51-39-34(46)32(44)33(45)35(50-39)37(47)48/h5-8,10-19,32-35,39,44-46H,4,9,20H2,1-3H3,(H,47,48)/t32-,33-,34+,35-,39-/m0/s1
InChI-Key	RCOBUBSULFIXAR-QQPFWGBFSA-N
CAS RN	250780-40-6
Metabolite of	Telmisartan
Detection frequency	100% (15/15 samples)
Detected in	Altenrhein, Monday-Friday<br>Neugut, Monday-Friday<br>Werdhölzli, Monday-Friday
Intensity	E6-E7
Initial confidence level	level 3
Initial confidence score	0.36
Final confidence level	level 4

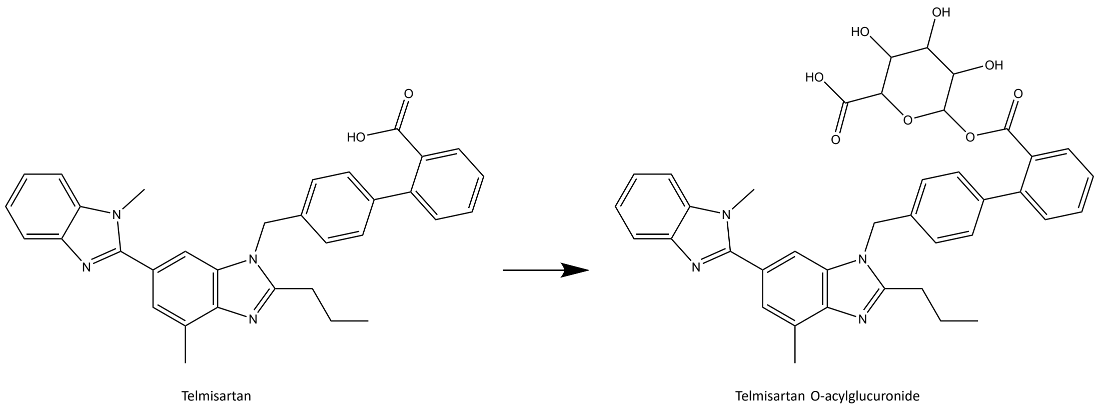

Figure SI-D458: Metabolism of tapentadol to telmisartan-O-acyl-glucuronide.

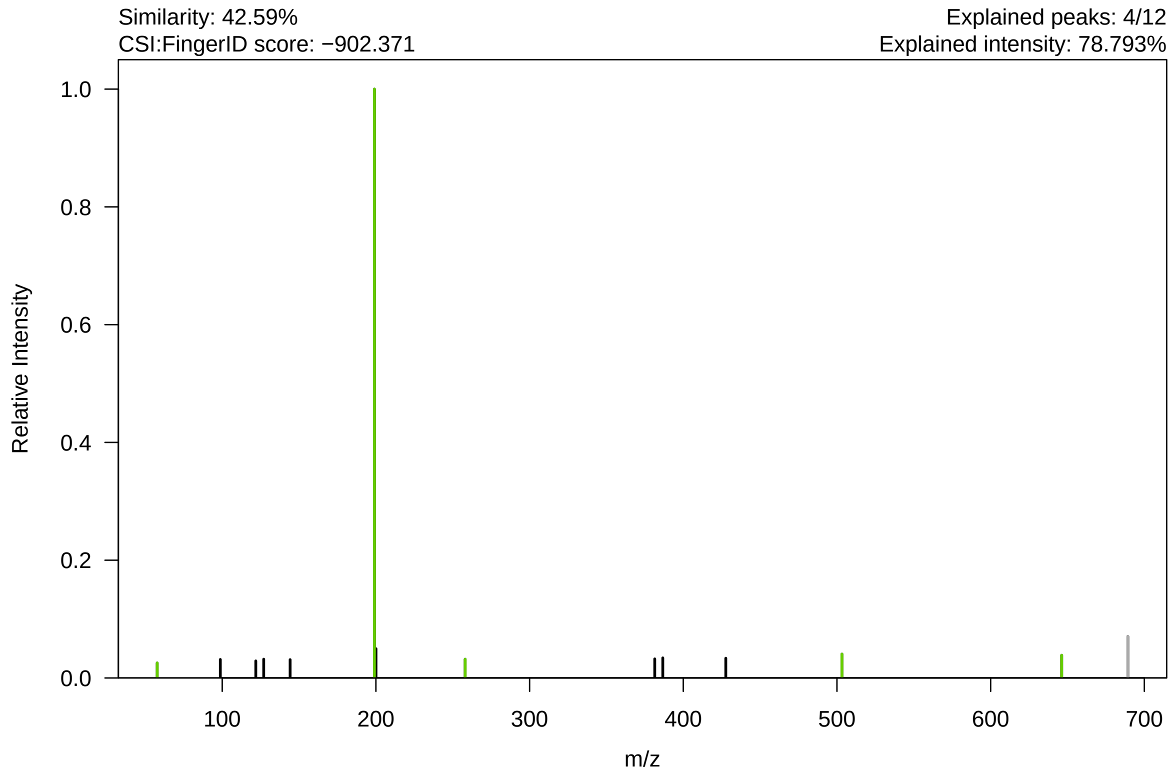

Figure SI-D459: Measured MS2 spectrum. Matching fragments with telmisartan-O-acyl-glucuronide predicted by SIRIUS/CSI:FingerID are highlighted in green. The molecular ion in gray is not considered.

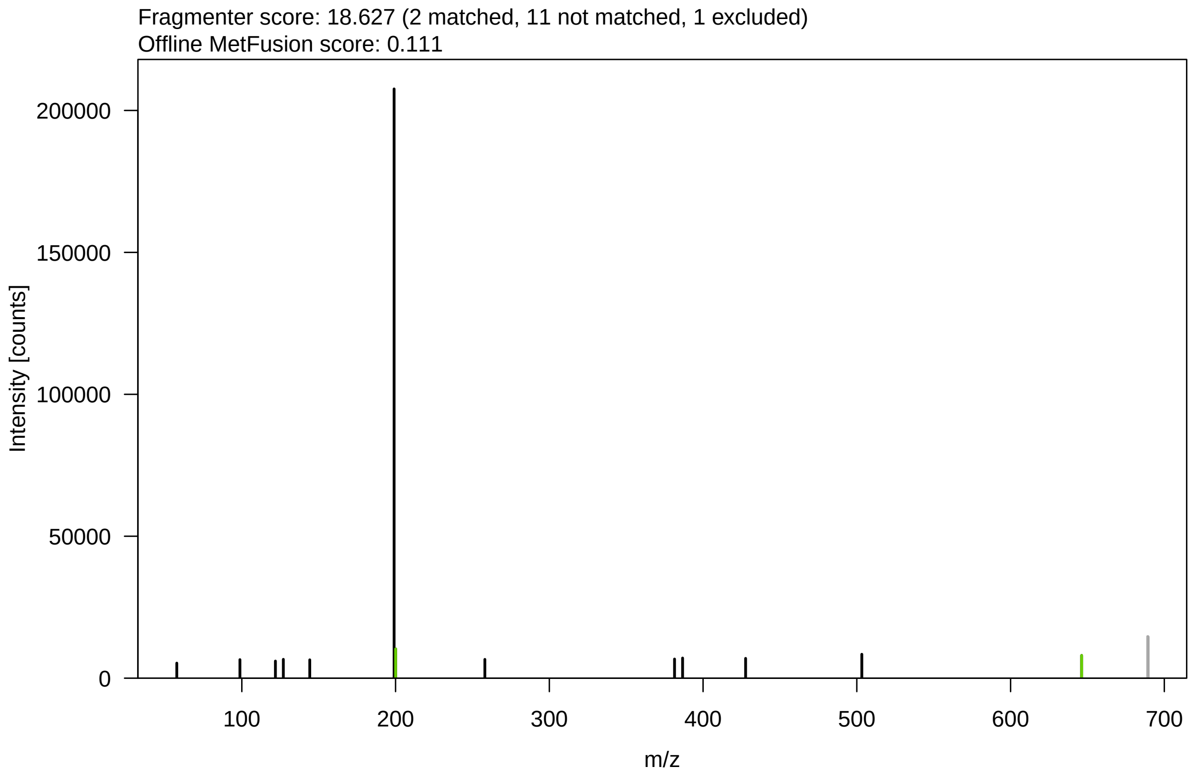

Figure SI-D460: Measured MS2 spectrum. Matching fragments with telmisartan-O-acyl-glucuronide predicted by MetFrag are highlighted in green. The molecular ion in gray is not considered.

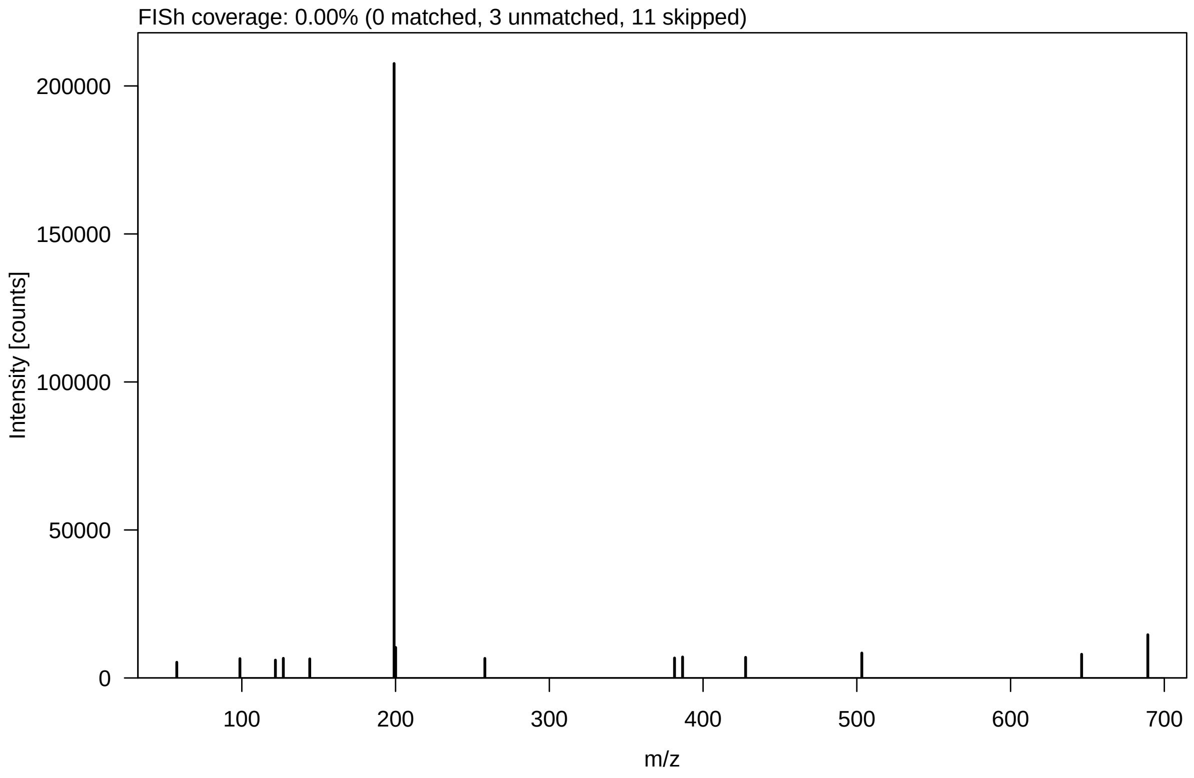

Figure SI-D461: Measured MS2 spectrum. None of the fragments could be rationalized by FISh Scoring. Low intensity fragments are not considered and skipped.

Table SI-D233: Retention time prediction of telmisartan-O-acyl-glucuronide.

Measured retention time [min]	15.2
Predicted $\log D_{OW}$ (pH = 4.8)	4.27
Predicted retention time [min]	22.1
Predicted retention time range (95% confidence interval) [min]	14.9-29.2
Predicted retention time range (99% confidence interval) [min]	12.5-31.6

Table SI-D234: Annotated MS2 spectrum of telmisartan-O-acyl-glucuronide.

m/z	Relative Intensity	Annotation
57.7005	25.54	
98.7603	31.42	
121.8591	29.02	
127.0076	31.92	
144.1901	31.09	
199.0434	999.00	$C_{15}H_6N - H^-$
200.0463	49.66	$C_{12}H_9O_3 - H^-$
258.0793	31.82	$C_{17}H_{11}N_2O^-H^-$
381.4364	32.44	
386.6786	34.13	
427.6693	33.53	
503.2514	40.55	$C_{25}H_{36}N_4O_7 - H^-$
646.2198	38.55	$C_{37}H_{33}N_3O_8 - H^-$
689.2707	70.31	$C_{39}H_{38}N_4O_8 - H^-$

A reference standard of telmisartan-O-acyl-glucuronide was purchased. Figure SI-D462 shows the extracted ion chromatograms of this standard, the sample and the spiked sample. It becomes visible that the suspected compound elutes more than five minutes later than the reference standard of telmisartan-O-acyl-glucuronide. It can therefore be concluded that the suspected compound is not telmisartan-O-acyl-glucuronide. Correspondingly, the identification confidence has to be decreased to level 4 due to the unequivocal molecular formula.

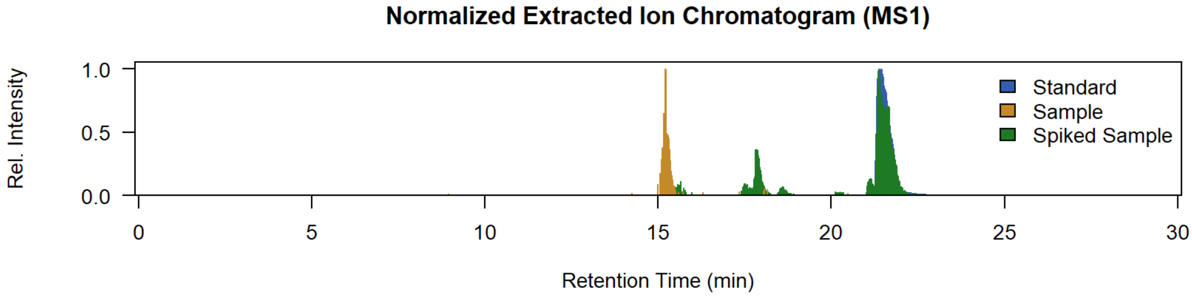

Figure SI-D462: Extracted ion chromatograms of telmisartan-O-acyl-glucuronide in the reference standard and the suspected compound in the sample and the spiked sample.

SI-D3 Metabolites of Non-Prioritized Compounds

The compounds discussed in this section were originally suspected to be pharmaceutical metabolites. However, detailed MS2 analysis revealed that they are human metabolites, but not of the prioritized pharmaceuticals.

SI-D3.1 3-Hydroxycotinine

3-hydroxycotinine is a metabolite of nicotine, which is a stimulatory alkaloid found in tobacco products. It is often used for the relief of nicotine withdrawal symptoms and as an aid to smoking cessation.[2] Figure SI-D463 shows the metabolism scheme.

Table SI-D235: Information on identifiers, chemical properties, detection and confidence of identification of 3-hydroxycotinine.

IUPAC Name	3-hydroxy-1-methyl-5-pyridin-3-ylpyrrolidin-2-one
Molecular formula	$C_{10}H_{12}N_2O_2$
Monoisotopic mass [g/mol]	192.0899
Adduct	$[M+H]^+$
Retention time [min]	5.6
SMILES	CN1C(CC(C1=O)O)C2=CN=CC=C2
InChI	InChI=1S/C10H12N2O2/c1-12-8(5-9(13)10(12)14)7-3-2-4-11-6-7/h2-4,6,8-9,13H,5H2,1H3
InChI-Key	XOKCJXZZNAUIQN-UHFFFAOYSA-N
CAS RN	79581-34-3
Metabolite of	Nicotine
Detection frequency	100% (15/15 samples)
Detected in	Altenrhein, Monday-Friday<br>Neugut, Monday-Friday<br>Werdhölzli, Monday-Friday
Intensity	E8
Initial confidence level	level 2a
Initial confidence score	0.55
Final confidence level	level 1

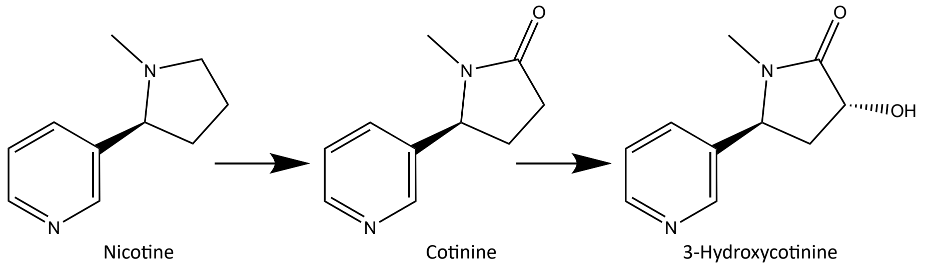

Figure SI-D463: Metabolism of nicotine, to cotinine and further to 3-hydroxycotinine.

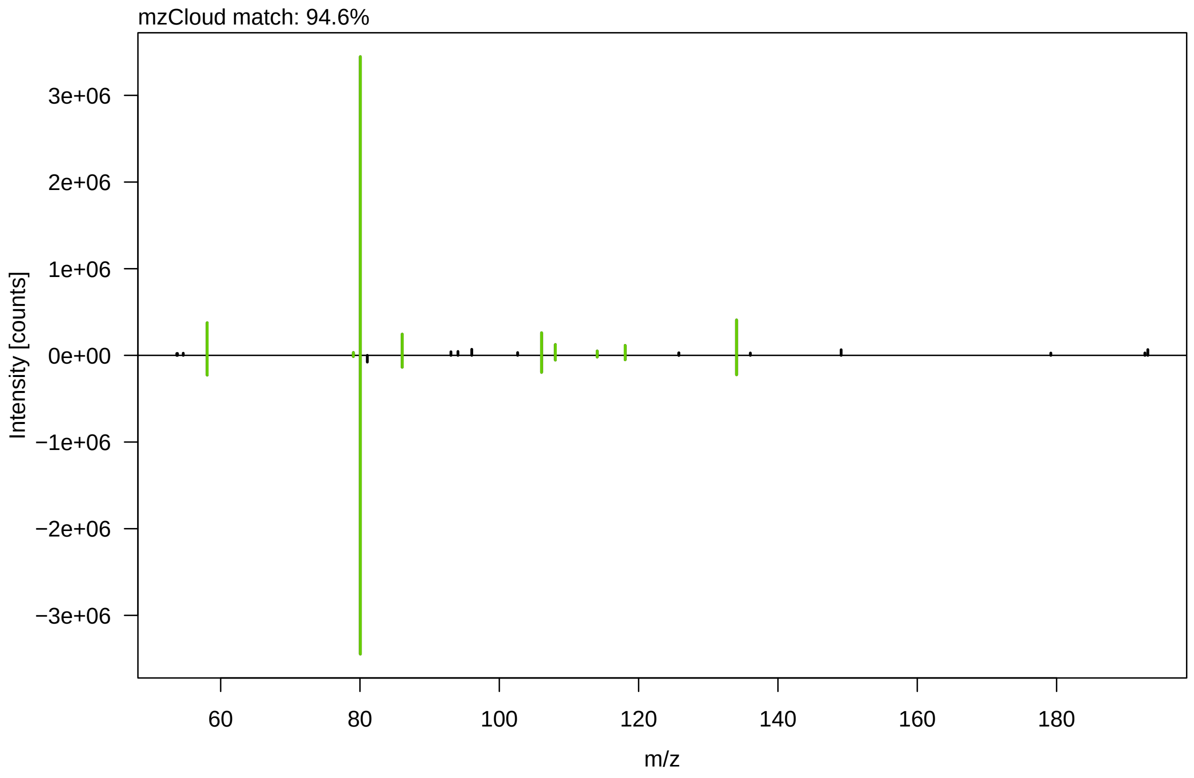

Figure SI-D464: Head to tail plot of measured MS2 spectrum against mzCloud library spectrum of 3-hydroxycotinine. Matching fragments are highlighted in green.

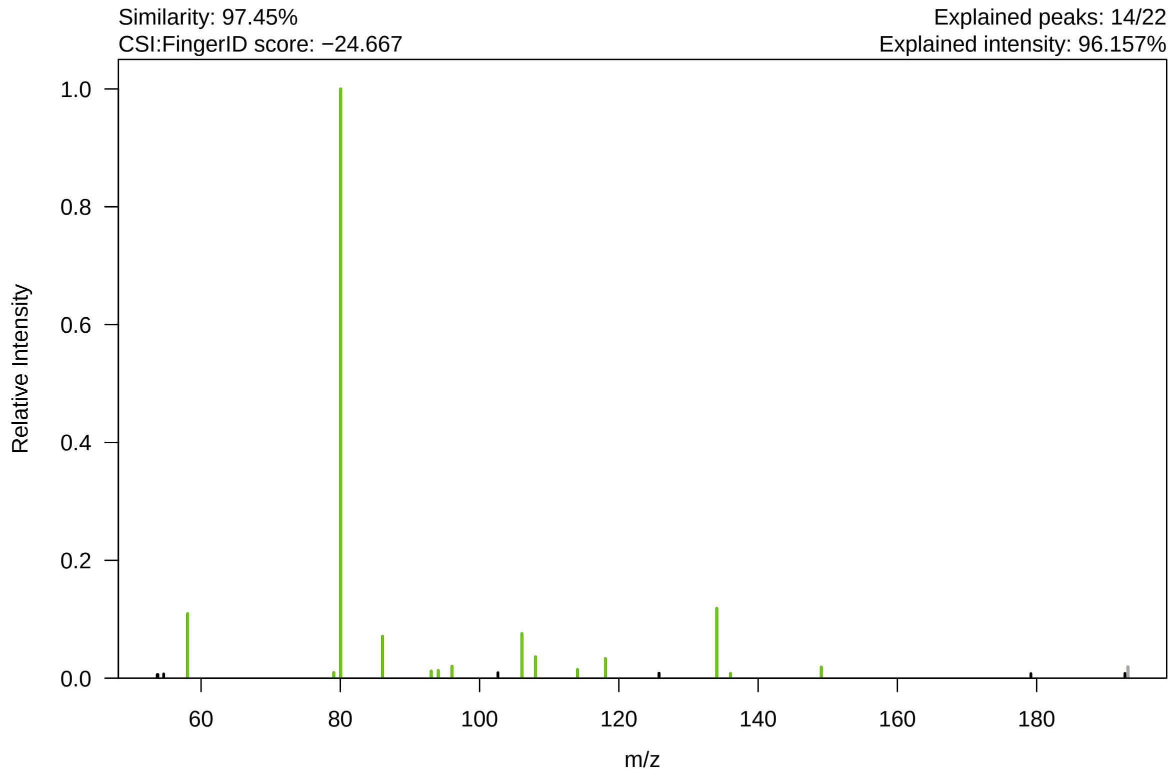

Figure SI-D465: Measured MS2 spectrum. Matching fragments with 3-hydroxycotinine predicted by SIRIUS/CSI:FingerID are highlighted in green. The molecular ion in gray is not considered.

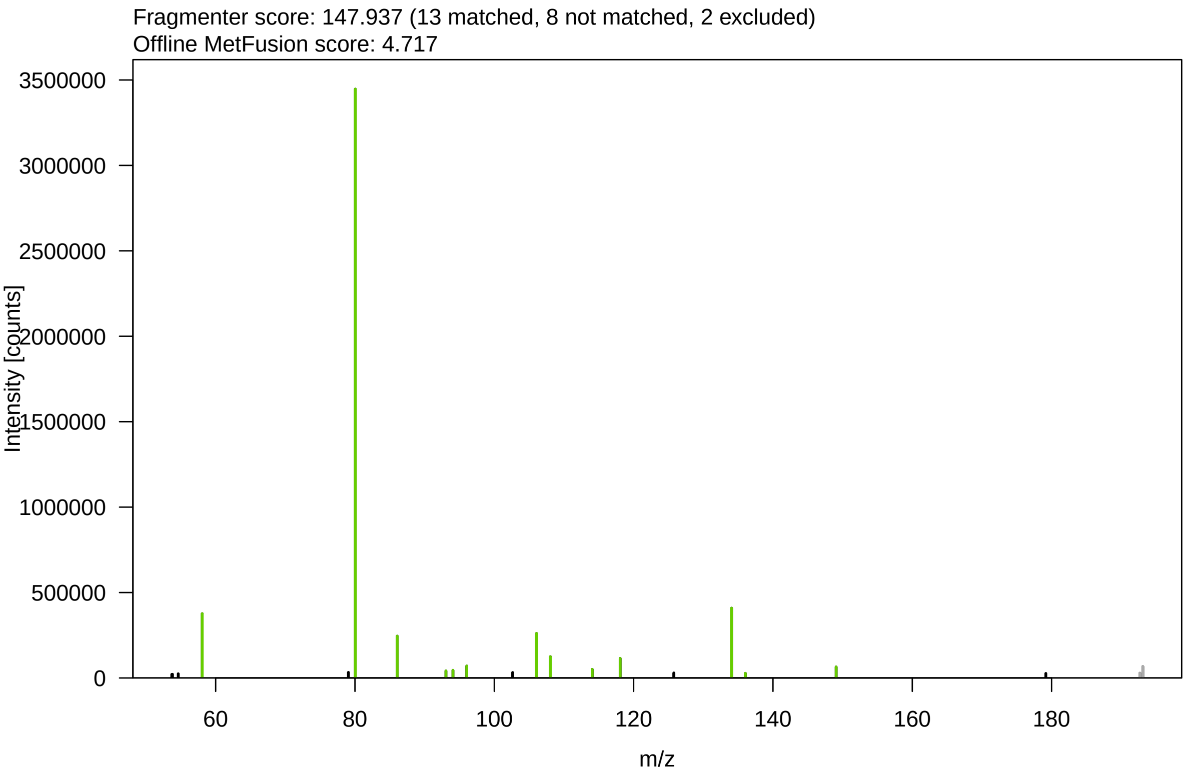

Figure SI-D466: Measured MS2 spectrum. Matching fragments with 3-hydroxycotinine predicted by MetFrag are highlighted in green. The molecular ion in gray is not considered.

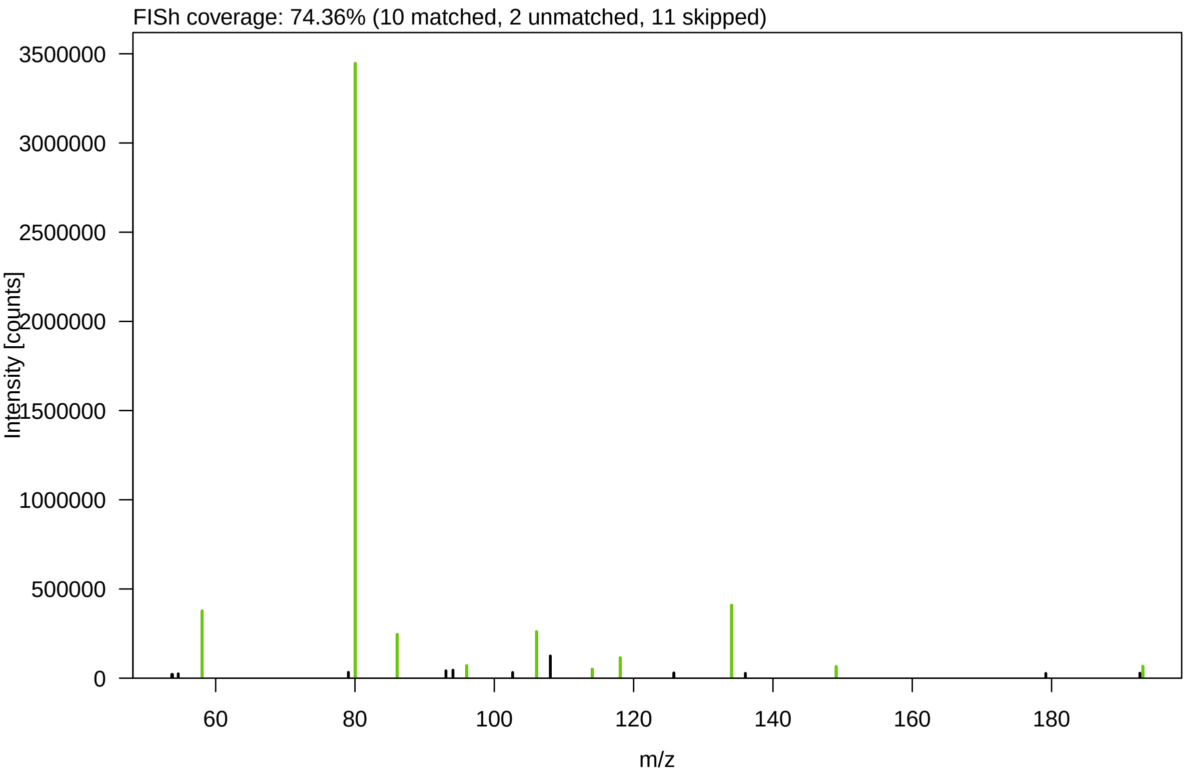

Figure SI-D467: Measured MS2 spectrum. Matching fragments with 3-hydroxycotinine predicted by FISh Scoring are highlighted in green. Low intensity fragments are not considered and skipped.

Table SI-D236: Retention time prediction of 3-hydroxycotinine.

Measured retention time [min]	5.6
Predicted $logD_{OW}$ (pH = 2.7)	-1.69
Predicted retention time [min]	12.5
Predicted retention time range (95% confidence interval) [min]	7.9-17.1
Predicted retention time range (99% confidence interval) [min]	6.5-18.6

Table SI-D237: Annotated MS2 spectrum of 3-hydroxycotinine.

m/z	Relative Intensity	Annotation
53.7014	6.14	
53.8021	6.23	
54.6284	7.17	
58.0654	109.11	$C_3H_7N + H^+$
79.0543	9.51	$C_6H_6 + H^+$
80.0494	999.00	$C_5H_5N + H^+$
86.0600	71.00	$C_4H_7NO + H^+$
93.0570	12.07	$C_6H_6N + H^+$
94.0651	13.06	$C_6H_7N + H^+$
96.0444	20.29	$C_5H_5NO + H^+$
102.6320	9.31	
106.0651	75.61	$C_7H_7N + H^+$
108.0442	36.13	$C_6H_5NO + H^+$
114.0544	14.61	$C_5H_7NO_2 + H^+$
118.0649	33.23	$C_8H_7N + H^+$
125.7726	8.49	
125.7819	8.28	
134.0599	118.46	$C_8H_7NO + H^+$
136.0382	7.93	$C_7H_5NO_2 + H^+$
149.0709	18.77	$C_8H_8N_2O + H^+$
179.1744	7.77	
192.6800	8.11	
193.0970	19.27	$C_{10}H_{12}N_2O_2 + H^+$

A reference standard of 3-hydroxycotinine was purchased. Figure SI-D468 shows the extracted ion chromatograms of this standard, the sample and the spiked sample, as well as a head to tail plot of the MS2 spectra of the standard and the sample. In addition, the most intense MS2 fragments in the sample and in the standard are displayed. It becomes visible that the retention times of the sample and the spiked sample are identical and the spectra similarity score between sample and standard is equal to 0.841. The vast majority of the sample fragments are explained by the reference standard. It can therefore be concluded that the suspected compound is indeed 3-hydroxycotinine. Correspondingly, the identification confidence can be increased to level 1.

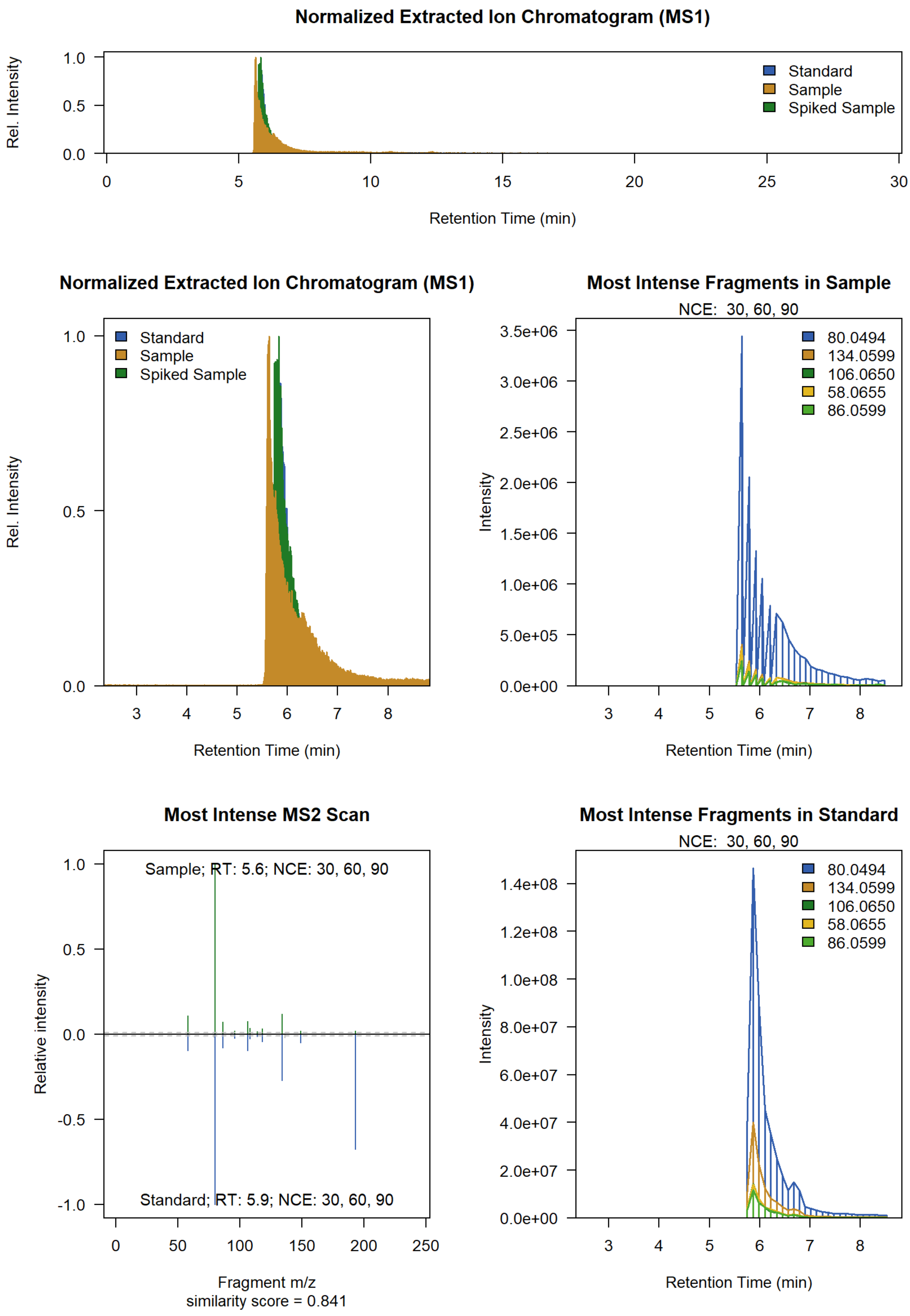

Figure SI-D468: Extracted ion chromatograms of 3-hydroxycotinine in the reference standard, the sample and the spiked sample, as well as MS2 head to tail plot and most intense MS2 fragments in standard and sample.

SI-D3.2 2',3'-Anhydroinosine

2',3'-Anhydroinosine is a metabolite of didanosine, which is a dideoxynucleoside compound that acts as a reverse transcriptase inhibitor. It is therefore used to treat HIV.[2] Figure SI-D469 shows the metabolism scheme.

Table SI-D238: Information on identifiers, chemical properties, detection and confidence of identification of 2,3-anhydroinosine.

IUPAC Name	9-[(2*R*,4*R*,5*R*)-4-(hydroxymethyl)-3,6-dioxabicyclo[3.1.0]hexan-2-yl]-1*H*-purin-6-one
Molecular formula	$C_{10}H_{10}N_4O_4$
Monoisotopic mass [g/mol]	250.0702
Adduct	$[M-H]^-$
Retention time [min]	8.7
SMILES	C1=NC2=C(C(=O)N1)N=CN2[C@H]3C4[C@H](O4)[C@H](O3)CO
InChI	InChI=1S/C10H10N4O4/c15-1-4-6-7(18-6)10(17-4)14-3-13-5-8(14)11-2-12-9(5)16/h2-4,6-7,10,15H,1H2,(H,11,12,16)/t4-,6-,7?,10-/m1/s1
InChI-Key	WDIGUIIOGAEQHN-CPTYKQRNSA-N
CAS RN	31766-13-9
Metabolite of	Didanosine
Detection frequency	100% (15/15 samples)
Detected in	Altenrhein, Monday-Friday<br>Neugut, Monday-Friday<br>Werdhölzli, Monday-Friday
Intensity	E7
Initial confidence level	level 3
Initial confidence score	0.52
Final confidence level	level 3

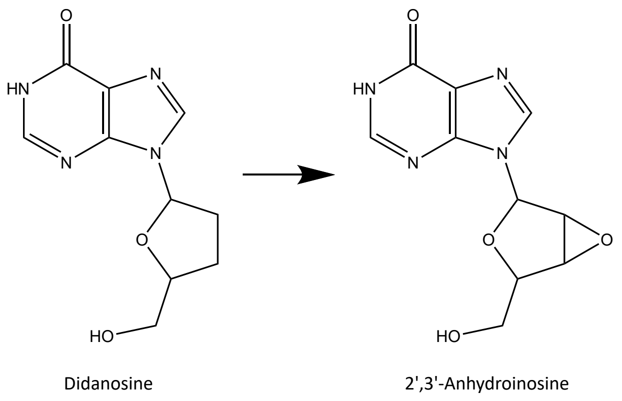

Figure SI-D469: Metabolism of didanosine to 2',3'-anhydroinosine.

No MetFrag analysis was conducted, since 2',3'-anhydroinosine was neither part of PubChemLite nor of the suspect list.

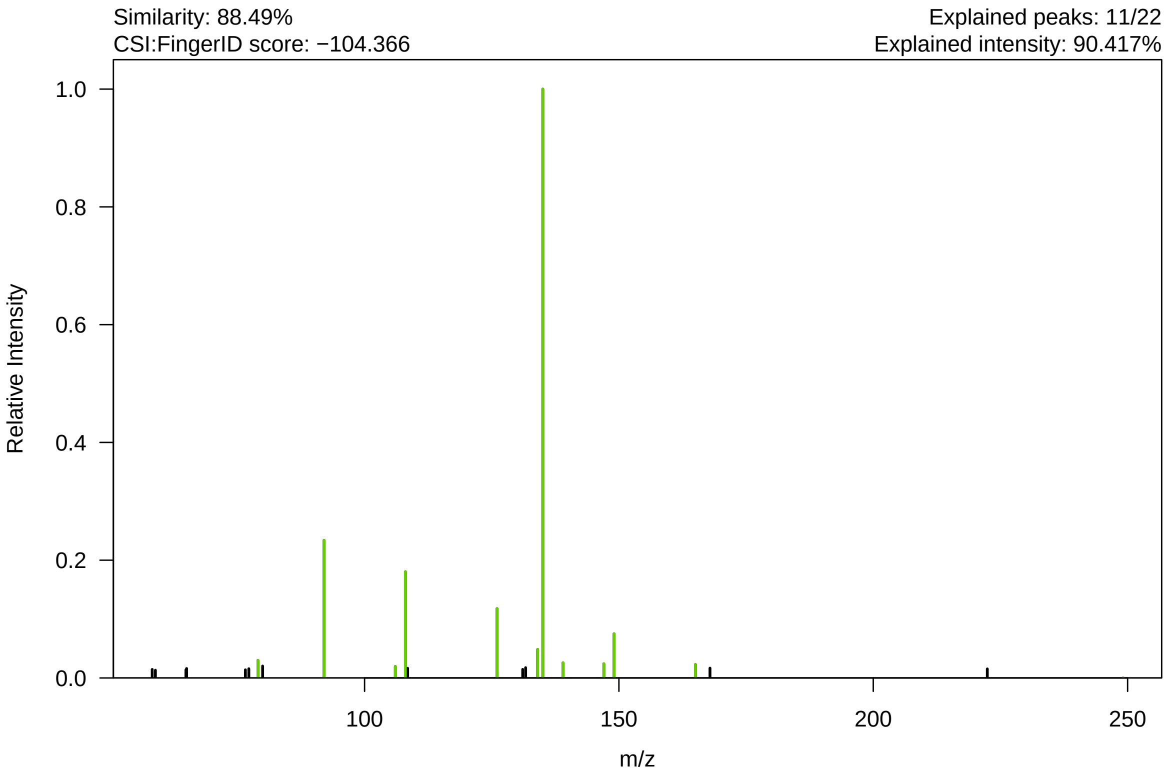

Figure SI-D470: Measured MS2 spectrum. Matching fragments with 2',3'-anhydroinosine predicted by SIRIUS/CSI:FingerID are highlighted in green.

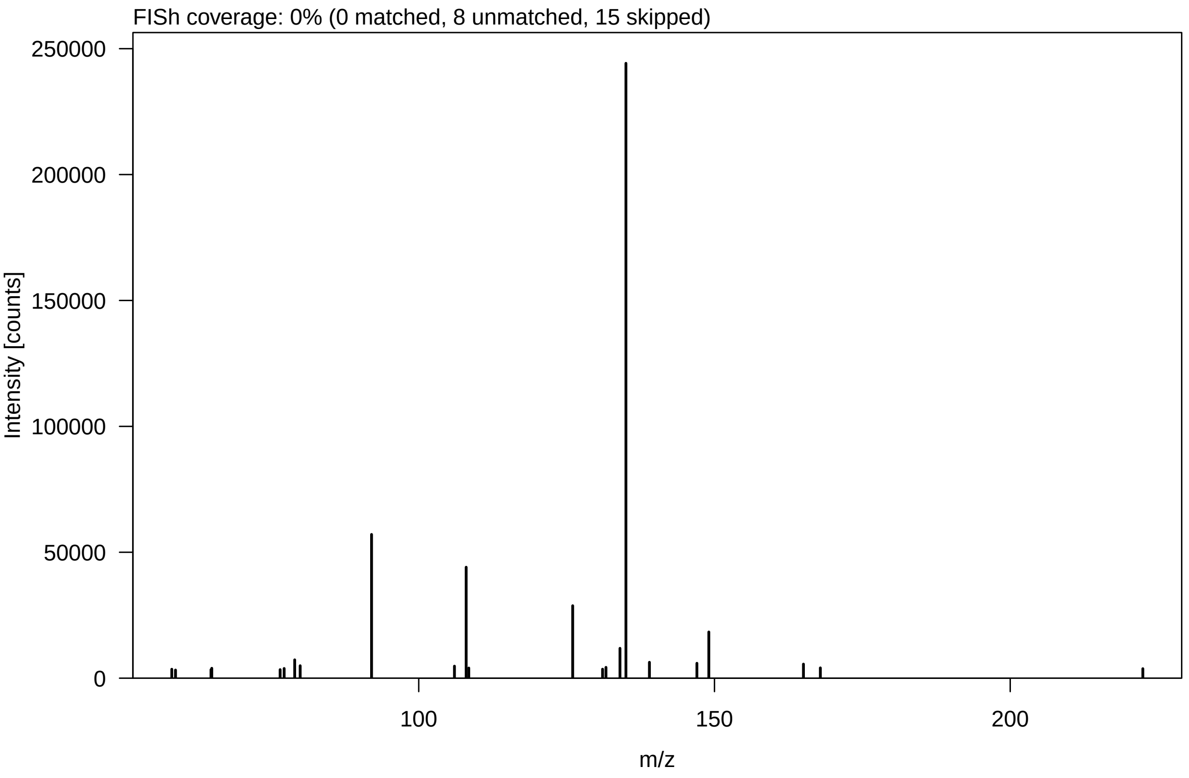

Figure SI-D471: Measured MS2 spectrum. None of the fragments could be rationalized by FISh Scoring. Low intensity fragments are not considered and skipped.

Table SI-D239: Retention time prediction of 2',3'-anhydroinosine.

Measured retention time [min]	8.7
Predicted $logD_{OW}$ (pH = 4.8)	-1.32
Predicted retention time [min]	12.0
Predicted retention time range (95% confidence interval) [min]	4.9-19.2
Predicted retention time range (99% confidence interval) [min]	2.6-21.5

Table SI-D240: Annotated MS2 spectrum of 2',3'-anhydroinosine.

m/z	Relative Intensity	Annotation
58.2514	14.53	
58.8814	13.19	
64.8782	13.80	
65.0135	16.09	
76.5682	13.89	
77.2475	15.75	
79.0297	29.78	$C_4H_4N_2 - H^-$
79.9567	20.22	
92.0255	233.51	$C_4H_3N_3 - H^-$
106.0410	19.55	$C_5H_5N_3{-}H^-$
108.0204	180.30	$C_4H_3N_3O - H^-$
108.4649	16.50	
126.0308	117.70	$C_4H_5N_3O_2 - H^-$
131.0816	14.62	
131.6571	17.54	
134.0233	48.49	$C_5H_3N_4O - H^-$
135.0313	999.00	$C_5H_4N_4O - H^-$
139.0039	25.79	
147.0313	24.19	$C_6H_4N_4O - H^-$
149.0465	75.04	$C_6H_6N_4O - H^-$
165.0416	22.87	$C_6H_6N_4O_2 - H^-$
167.8996	16.72	
222.4193	15.41	

No reference standard of 2',3'-anhydroinosine was purchased. Therefore, a human liver S9 incubation experiment with didanosine was performed, to generate didanosine metabolites *in vitro*. However, no compound with a precursor matching the one of 2',3'-anhydroinosine was detected within a retention time window of ±3 minutes of the suspect. Since *in vitro* experiments cannot be translated one to one into *in vivo* experiments, the suspected compound can neither be confirmed nor rejected. As a consequence, the final confidence of identification remains at level 3.

SI-D3.3 Methylecgonine

Methylecgonine is a metabolite of cocaine, which has a similar effect than amphethamines and is a drug of abuse. In some countries it used as local anesthetic during diagnostic procedures and surgeries in the eye, ear, nose and throat.[2] Figure SI-D472 shows the metabolism scheme.

Table SI-D241: Information on identifiers, chemical properties, detection and confidence of identification of methylecgonine.

IUPAC Name	methyl 3-hydroxy-8-methyl-8-azabicyclo[3.2.1]octane-2- carboxylate
Molecular formula	$C_{10}H_{17}NO_3$
Monoisotopic mass [g/mol]	199.1208
Adduct	$[M+H]^+$
Retention time [min]	5.3
SMILES	CN1C2CCC1C(C(C2)O)C(=O)OC
InChI	InChI=1S/C10H17NO3/c1-11-6-3-4-7(11)9(8(12)5-6)10(13)14-2/h6-9,12H,3-5H2,1-2H3
InChI-Key	QIQNNBXHAYSQRY-UHFFFAOYSA-N
CAS RN	7143-09-1
Metabolite of	Cocaine
Detection frequency	100% (15/15 samples)
Detected in	Altenrhein, Monday-Friday
	Neugut, Monday-Friday
	Werdhölzli, Monday-Friday
Intensity	E7
Initial confidence level	level 2a
Initial confidece score	0.62
Final confidence level	level 1

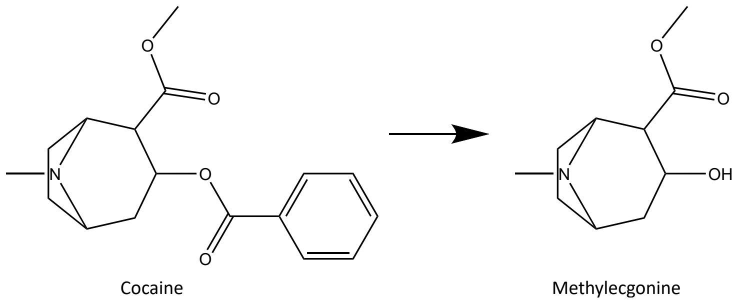

Figure SI-D472: Metabolism of cocaine to methylecgonine.

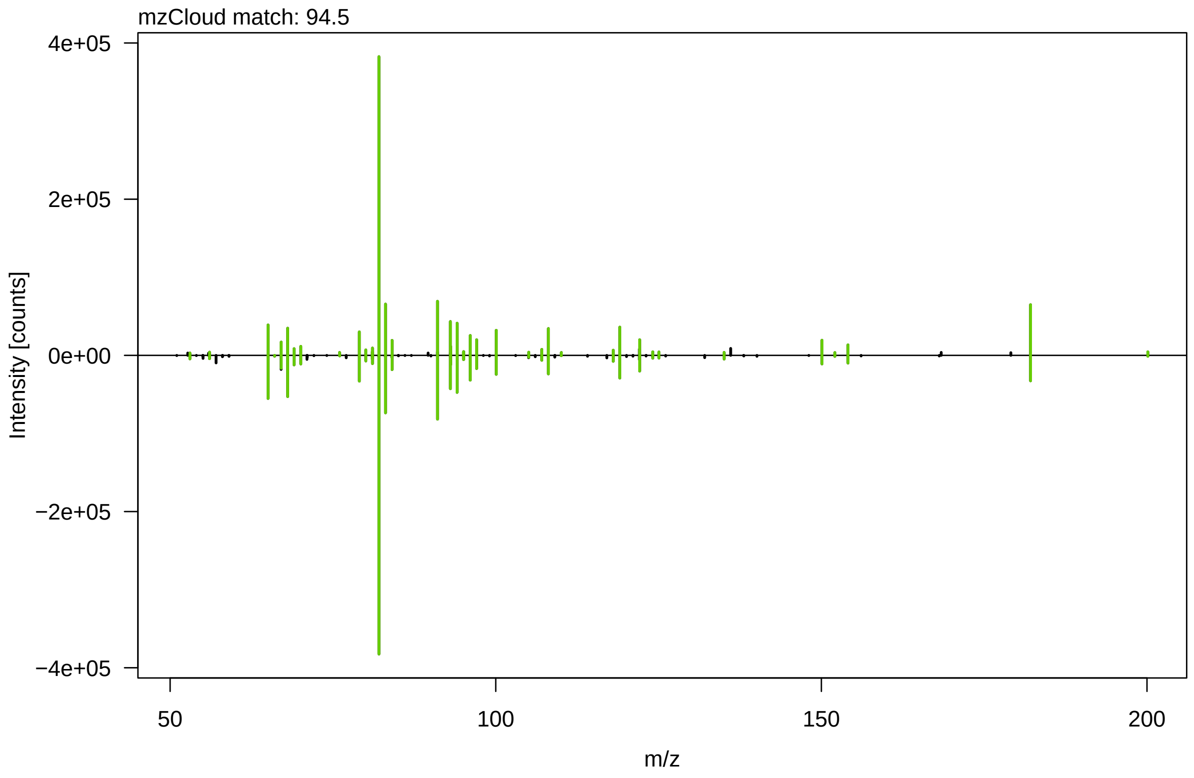

Figure SI-D473: Head to tail plot of measured MS2 spectrum against mzCloud library spectrum of methylecgonine. Matching fragments are highlighted in green.

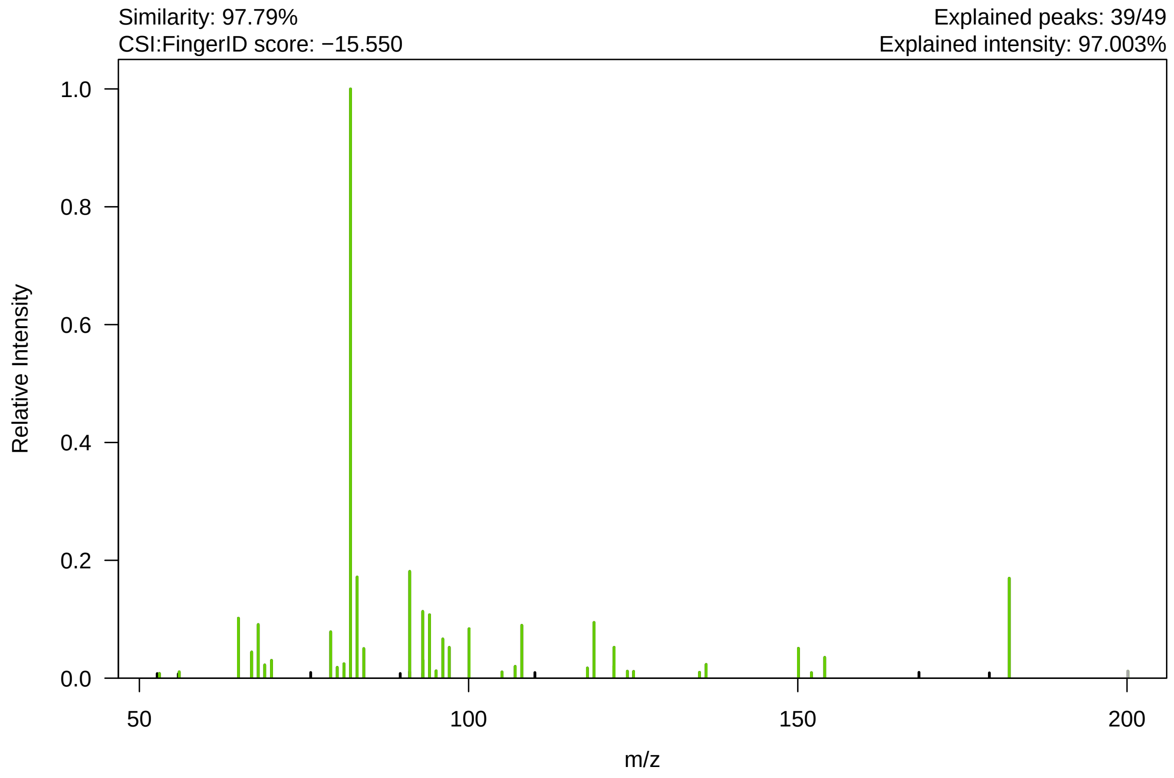

Figure SI-D474: Measured MS2 spectrum. Matching fragments with methylecgonine predicted by SIRIUS/CSI:FingerID are highlighted in green. The molecular ion in gray is not considered.

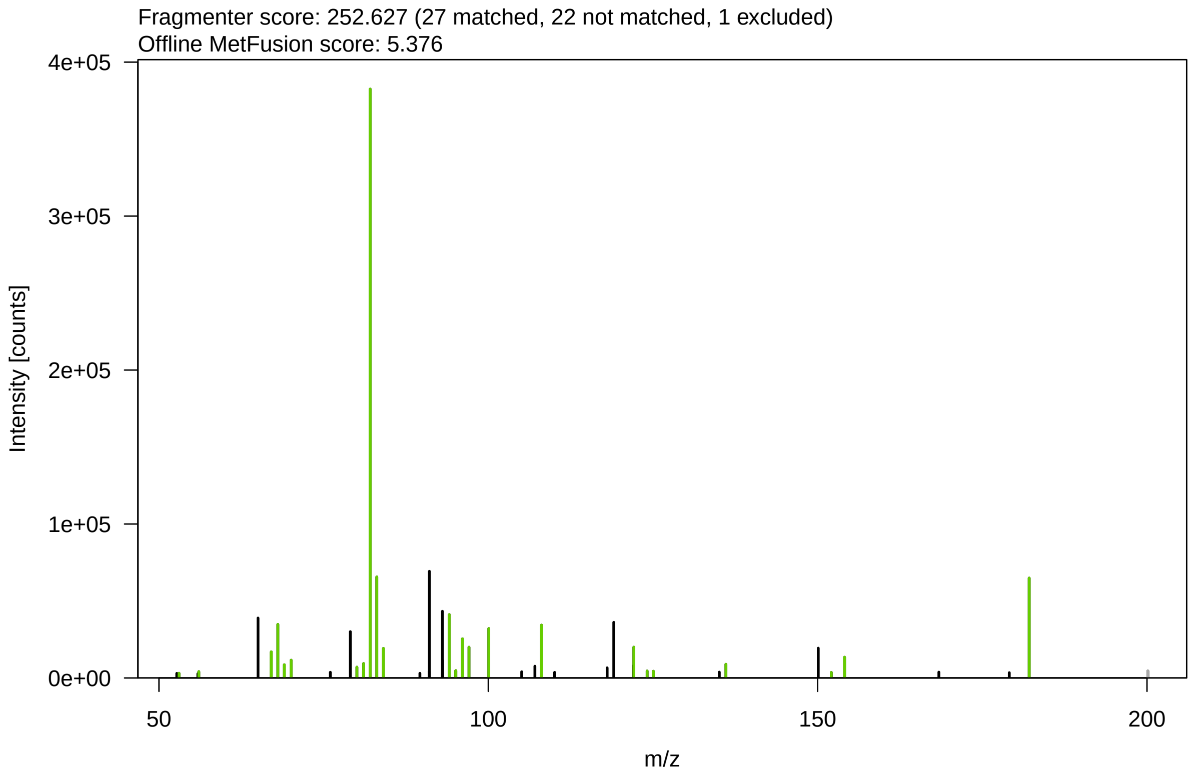

Figure SI-D475: Measured MS2 spectrum. Matching fragments with methylecgonine predicted by MetFrag are highlighted in green. The molecular ion in gray is not considered.

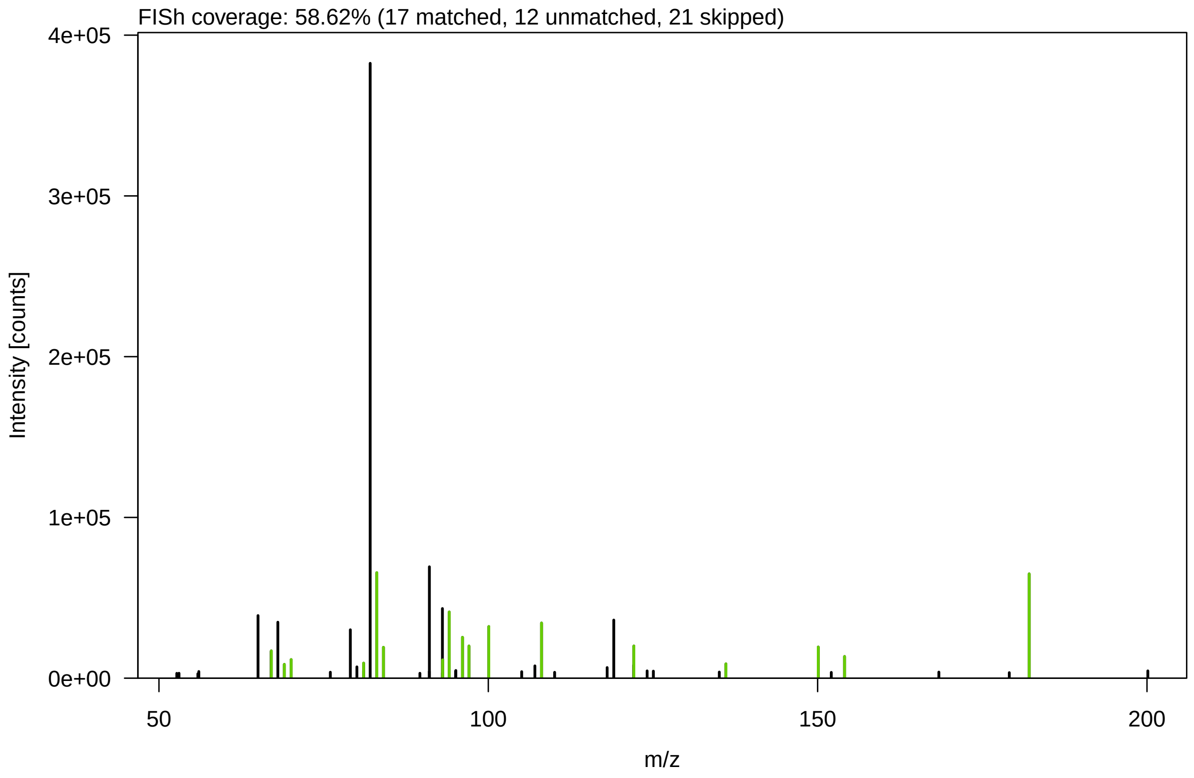

Figure SI-D476: Measured MS2 spectrum. Matching fragments with methylecgonine predicted by FISh Scoring are highlighted in green. Low intensity fragments are not considered and skipped.

Table SI-D242: Retention time prediction of methylecgonine.

Measured retention time [min]	5.3
Predicted $logD_{OW}$ (pH = 2.7)	-3.71
Predicted retention time [min]	9.9
Predicted retention time range (95% confidence interval) [min]	5.3-14.5
Predicted retention time range (99% confidence interval) [min]	3.8-16.0

Table SI-D243: Annotated MS2 spectrum of methylecgonine.

m/z	Relative Intensity	Annotation
52.7055	7.83	
53.0388	7.99	$C_4H_4 + H^+$
55.9021	7.15	
56.0495	10.81	$C_3H_5N + H^+$
65.0388	101.70	$C_5H_4 + H^+$
67.0416	43.06	$C_4H_4N + H^+$
67.0543	44.38	$C_5H_6 + H^+$
68.0496	90.94	$C_4H_5N + H^+$
69.0335	22.44	$C_4H_4O + H^+$
70.0650	30.19	$C_4H_7N + H^+$
76.0188	9.67	
79.0543	78.61	$C_6H_6 + H^+$
80.0494	18.37	$C_5H_5N + H^+$
81.0568	11.82	$C_5H_6N + H^+$
81.0695	24.48	$C_6H_8 + H^+$
82.0651	999.00	$C_5H_7N + H^+$
83.0729	171.42	$C_5H_8N + H^+$
84.0807	50.16	$C_5H_9N + H^+$
89.6146	7.95	
91.0487	11.06	
91.0542	180.97	$C_7H_6 + H^+$
93.0335	113.13	$C_6H_4O + H^+$
93.0453	8.41	
93.0700	29.87	$C_7H_8 + H^+$
94.0652	107.54	$C_6H_7N + H^+$
95.0491	11.36	$C_6H_6O + H^+$
95.0599	11.87	
95.0728	12.49	$C_6H_8N + H^+$
96.0805	66.44	$C_6H_9N + H^+$

Continued on next page

Table SI-D243: Annotated MS2 spectrum of methylecgonine.(Continued)

97.0647	52.23	$C_6H_8O + H^+$
100.0757	83.87	$C_5H_9NO + H^+$
105.0690	10.56	$C_8H_8 + H^+$
107.0726	19.98	$C_7H_8N + H^+$
108.0808	89.63	$C_7H_9N + H^+$
110.0709	9.54	
118.0412	17.24	$C_8H_5O + H^+$
119.0490	94.41	$C_8H_6O + H^+$
122.0599	20.95	$C_7H_7NO + H^+$
122.0962	52.26	$C_8H_{11}N + H^+$
124.1127	11.84	$C_8H_{13}N + H^+$
125.0599	11.45	$C_7H_8O_2 + H^+$
135.0682	9.96	$C_8H_8NO + H^+$
136.0761	23.25	$C_8H_9NO + H^+$
150.0914	50.60	$C_9H_{11}NO + H^+$
152.0710	9.38	$C_8H_9NO_2 + H^+$
154.0861	35.21	$C_8H_{11}NO_2 + H^+$
168.4047	9.91	
179.0951	8.98	
182.1176	169.47	$C_{10}H_{15}NO_2 + H^+$
200.1283	11.84	$C_{10}H_{17}NO_3 + H^+$

A reference standard of methylecgonine was purchased. Figure SI-D477 shows the extracted ion chromatograms of this standard, the sample and the spiked sample, as well as a head to tail plot of the MS2 spectra of the standard and the sample. In addition, the most intense MS2 fragments in the sample and in the standard are displayed. In the chromatogram, an abrupt increase in signal intensity is observed for the unspiked sample, the spiked sample and the standard. This increase occurs for the spiked and the unspiked sample at exactly the same retention time. In addition, the spectra similarity score between sample and standard is equal to 0.871. The vast majority of the sample fragments are explained by the reference standard. It can therefore be concluded that the suspected compound is indeed methylecgonine. Correspondingly, the identification confidence can be increased to level 1.

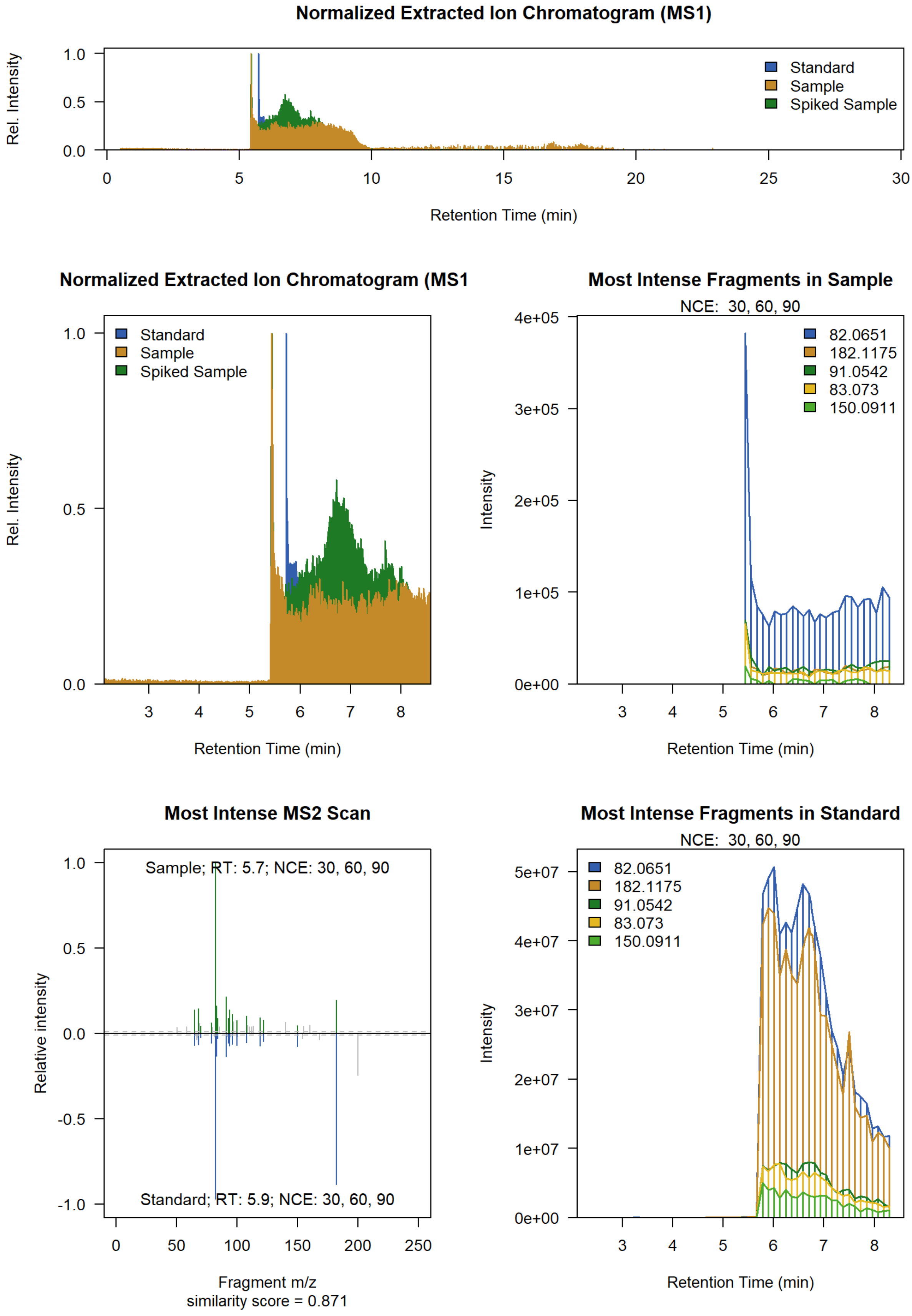

Figure SI-D477: Extracted ion chromatograms of methylecgonine in the reference standard, the sample and the spiked sample, as well as MS2 head to tail plot and most intense MS2 fragments in standard and sample.

SI-D4 Other Compounds

The compounds discussed in this section were originally suspected to be pharmaceutical metabolites. However, detailed MS2 analysis revealed that they are of different origin.

SI-D4.1 2-Acetylpyrazine

2-Acetylpyrazine is a fragrance and food additive. It has a savory, sweet corn, nut shell or dark bread crust taste.[22] The moelcular structure is shown in Figure SI-D478.

Table SI-D244: Information on identifiers, chemical properties, detection and confidence of identification of 2-acetylpyrazine.

IUPAC Name	1-pyrazin-2-ylethanone
Molecular formula	$C_6H_6N_2O$
Monoisotopic mass [g/mol]	122.0480
Adduct	$[M+H]^+$
Retention time [min]	12.1
SMILES	CC(=O)C1=NC=CN=C1
InChI	InChI=1S/C6H6N2O/c1-5(9)6-4-7-2-3-8-6/h2-4H,1H3
InChI-Key	DBZAKQWXICEWNW-UHFFFAOYSA-N
CAS RN	22047-25-2
Detection frequency	100% (15/15 samples)
Detected in	Altenrhein, Monday-Friday
	Neugut, Monday-Friday
	Werdhoelzli, Monday-Friday
Intensity	E6-E10
Initial confidence level	level 3
Initial confidence score	0.55
Final confidence level	level 1

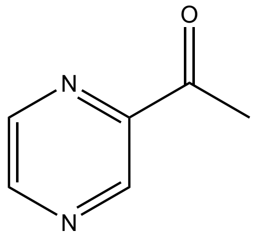

Figure SI-D478: Molecular structure of 2-acetylpyrazine.

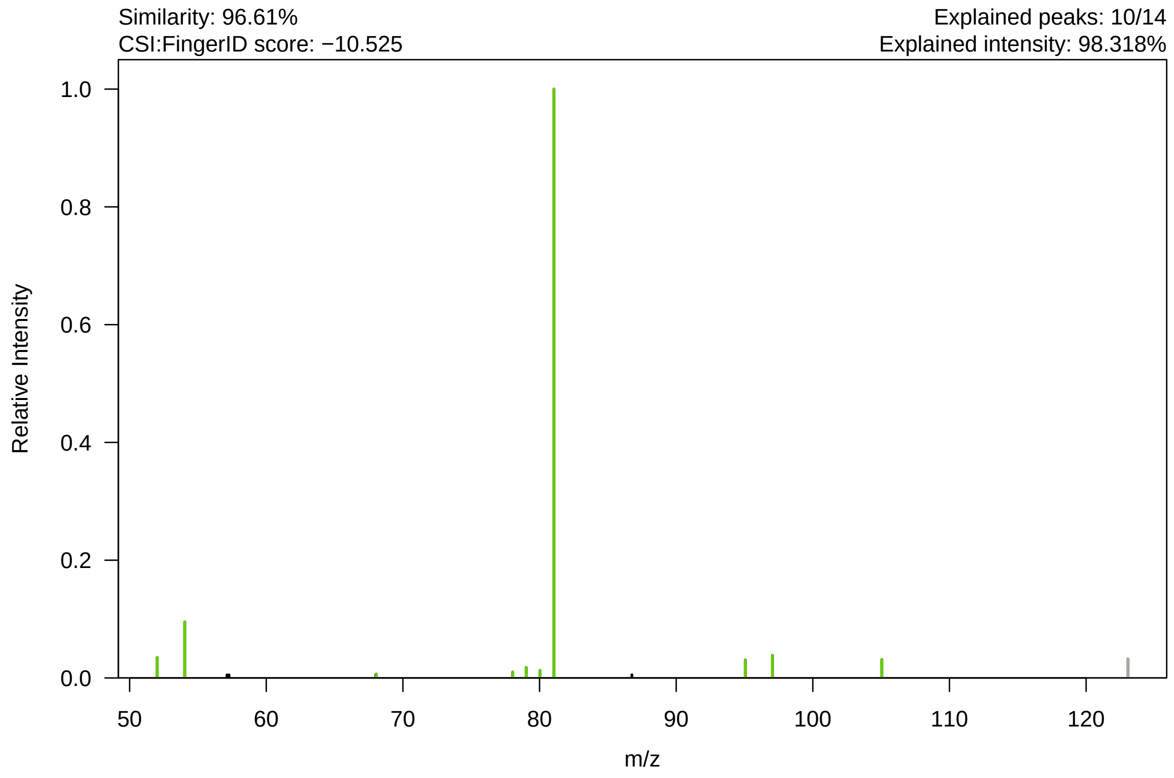

Figure SI-D479: Measured MS2 spectrum. Matching fragments with 2-acetylpyrazine predicted by SIRIUS/CSI:FingerID are highlighted in green. The molecular ion in gray is not considered.

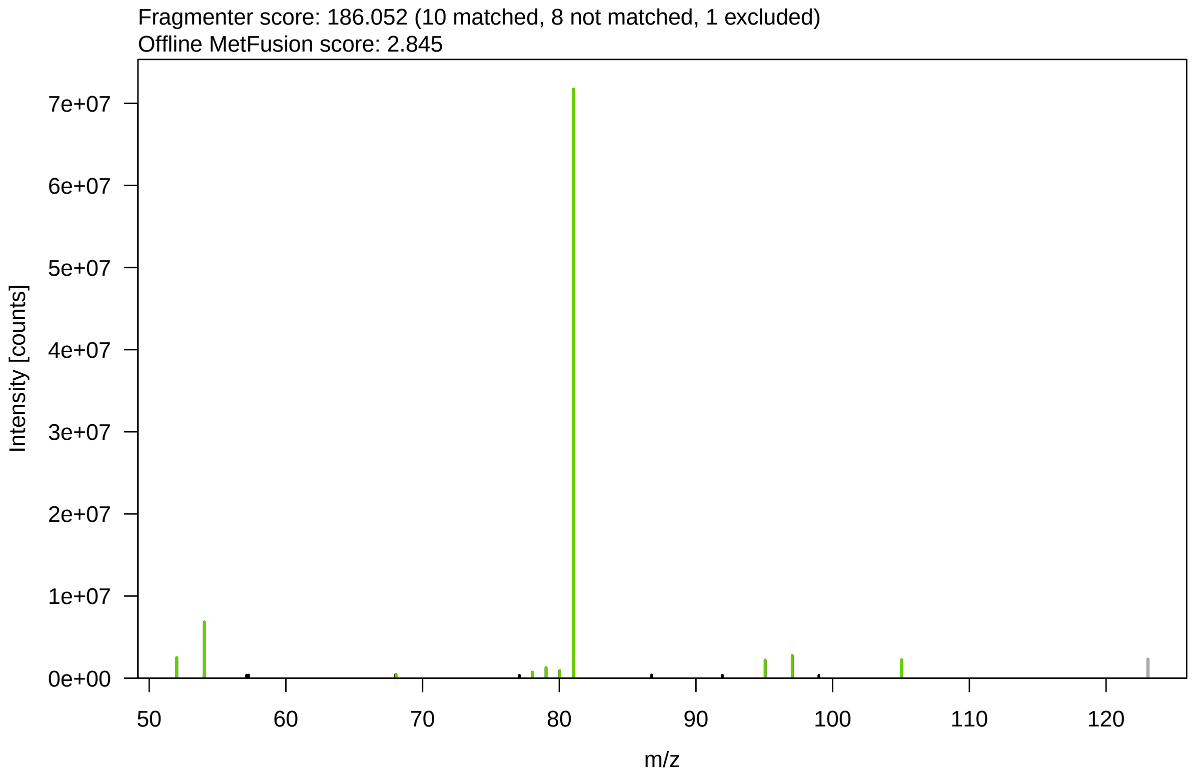

Figure SI-D480: Measured MS2 spectrum. Matching fragments with 2-acetylpyrazine predicted by MetFrag are highlighted in green. The molecular ion in gray is not considered.

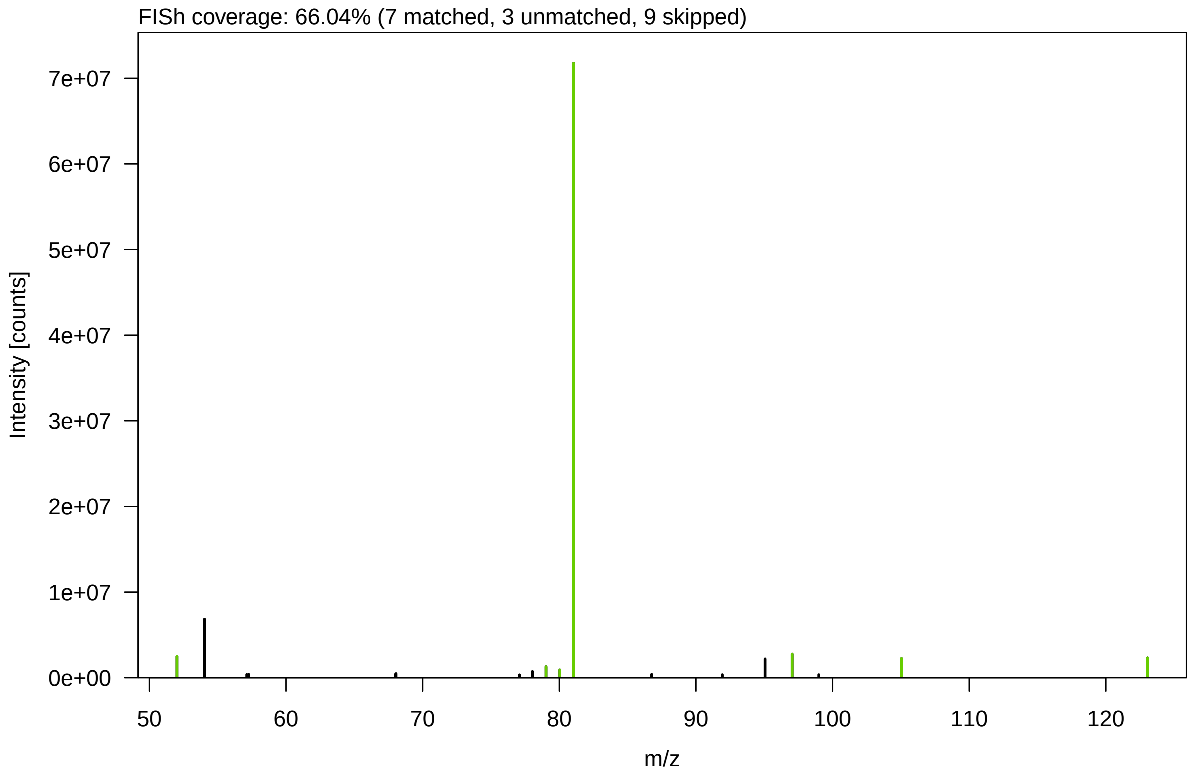

Figure SI-D481: Measured MS2 spectrum. Matching fragments with 2-acetylpyrazine predicted by FISh Scoring are highlighted in green. Low intensity fragments are not considered and skipped.

Table SI-D245: Retention time prediction of 2-acetylpyrazine.

Measured retention time [min]	12.1
Predicted $\log D_{OW}$ (pH = 2.7)	-0.52
Predicted retention time [min]	14.1
Predicted retention time range (95% confidence interval) [min]	9.5-18.7
Predicted retention time range (99% confidence interval) [min]	8.0-20.1

Table SI-D246: Annotated MS2 spectrum of 2-acetylpyrazine.

m/z	Relative Intensity	Annotation
52.0187	34.80	$C_3HN + H^+$
54.0319	5.98	
54.0343	95.25	$C_3H_3N + H^+$
57.1272	5.25	
57.2740	5.27	
68.0206	5.92	
68.0496	6.68	$C_4H_5N + H^+$
77.0843	4.72	
78.0341	10.14	$C_5H_3N + H^+$
79.0292	17.92	$C_4H_2N_2 + H^+$
80.0369	12.70	$C_4H_3N_2 + H^+$
81.0447	999.00	$C_4H_4N_2 + H^+$
86.7596	5.40	
91.9299	4.91	
95.0604	30.65	$C_5H_6N_2 + H^+$
97.0395	38.59	$C_4H_4N_2O + H^+$
98.9917	4.90	
105.0445	31.27	$C_6H_4N_2 + H^+$
123.0550	32.34	$C_6H_6N_2O + H^+$

A reference standard of 2-acetylpyrazine was purchased. Figure SI-D482 shows the extracted ion chromatograms of this standard, the sample and the spiked sample, as well as a head to tail plot of the MS2 spectra of the standard and the sample. In addition, the most intense MS2 fragments in the sample and in the standard are displayed. It becomes visible that the retention times of the sample and the spiked sample are identical and the spectra similarity score between sample and standard is equal to 1.0. All of the sample fragments are explained by the reference standard. It can therefore be concluded that the suspected compound is indeed 2-acetylpyrazine. Correspondingly, the identification confidence can be increased to level 1.

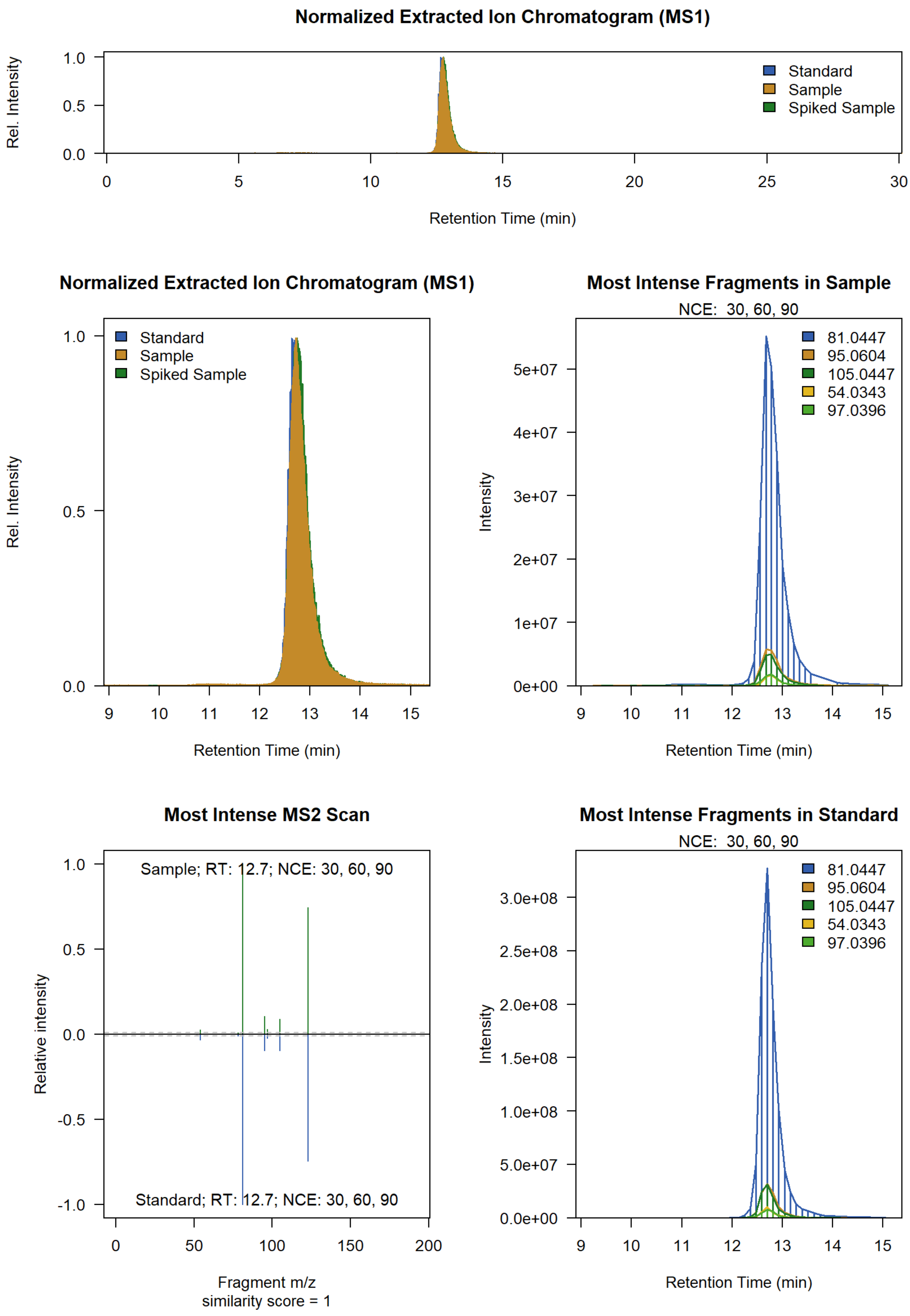

Figure SI-D482: Extracted ion chromatograms of 2-acetylpyrazine in the reference standard, the sample and the spiked sample, as well as MS2 head to tail plot and most intense MS2 fragments in standard and sample.

SI-D4.2 4-Hydroxybenzaldehyde

4-Hydroxybenzaldehyde is a food additive but is also a naturally occuring compound in humans and is found in vinegar, oats and beer.[23] Moreover, it is used in the synthesis of the food additive raspberry ketone.[24] The moelcular structure is shown in Figure SI-D483.

Table SI-D247: Information on identifiers, chemical properties, detection and confidence of identification of 4-hydroxybenzaldehyde.

IUPAC Name	4-hydroxybenzaldehyde
Molecular formula	$C_7H_6O_2$
Monoisotopic mass [g/mol]	122.0368
Adduct	$[M+H]^+$
Retention time [min]	13.6
SMILES	C1=CC(=CC=C1C=O)O
InChI	InChI=1S/C7H6O2/c8-5-6-1-3-7(9)4-2-6/h1-5,9H
InChI-Key	RGHHSNMVTDWUBI-UHFFFAOYSA-N
CAS RN	123-08-0
Detection frequency	60% (9/15 samples)
Detected in	Neugut, Monday-Friday
	Werdhölzli, Monday, Tuesday, Wednesday, Friday
Intensity	E6-E9
Initial confidence level	level 2a
Initial confidence score	0.39
Final confidence level	level 4

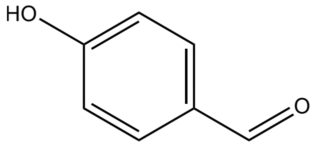

Figure SI-D483: Molecular structure of 4-hydroxybenzaldehyde.

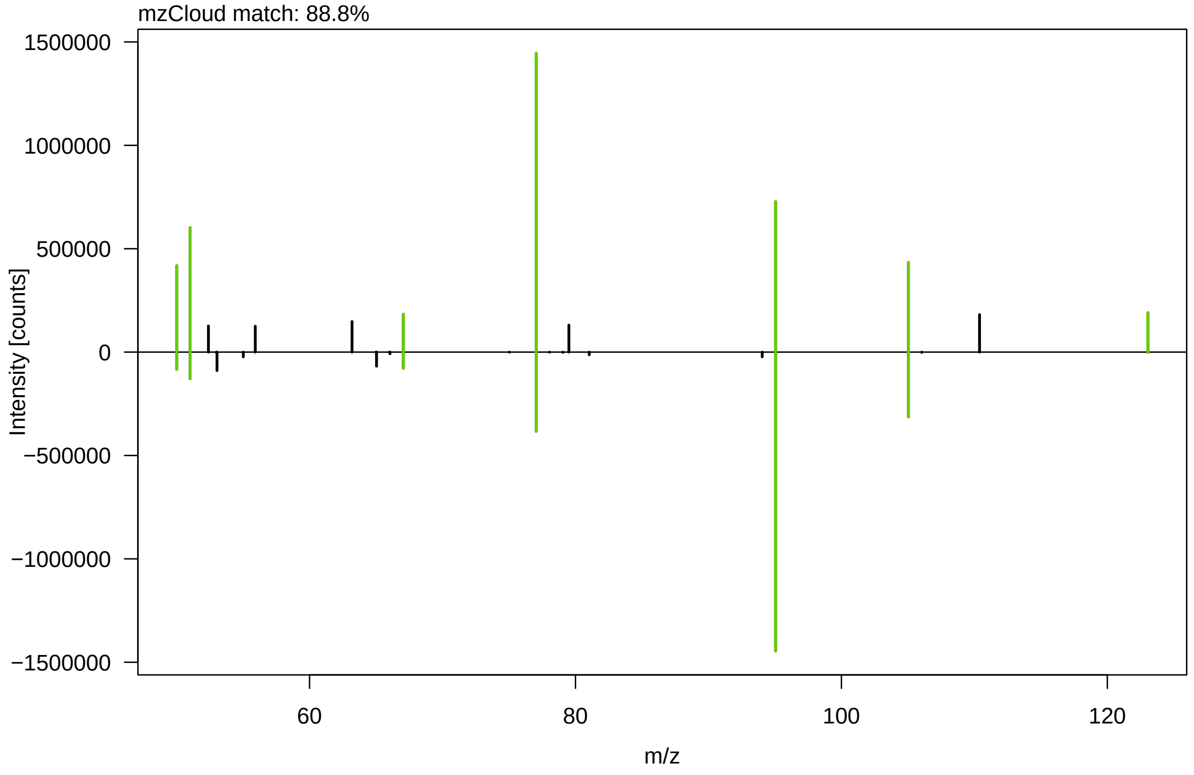

Figure SI-D484: Head to tail plot of measured MS2 spectrum against mzCloud library spectrum of 4-hydroxybenzaldehyde. Matching fragments are highlighted in green.

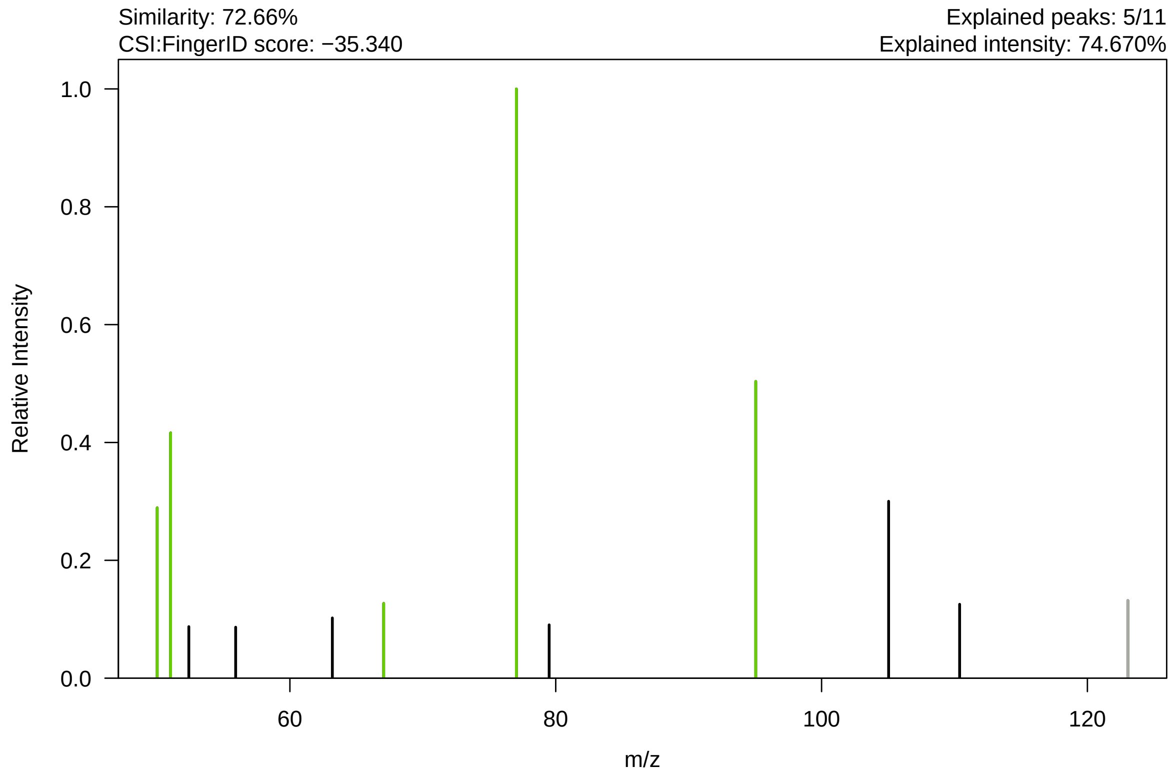

Figure SI-D485: Measured MS2 spectrum. Matching fragments with 4-hydroxybenzaldehyde predicted by SIRIUS/CSI:FingerID are highlighted in green. The molecular ion in gray is not considered.

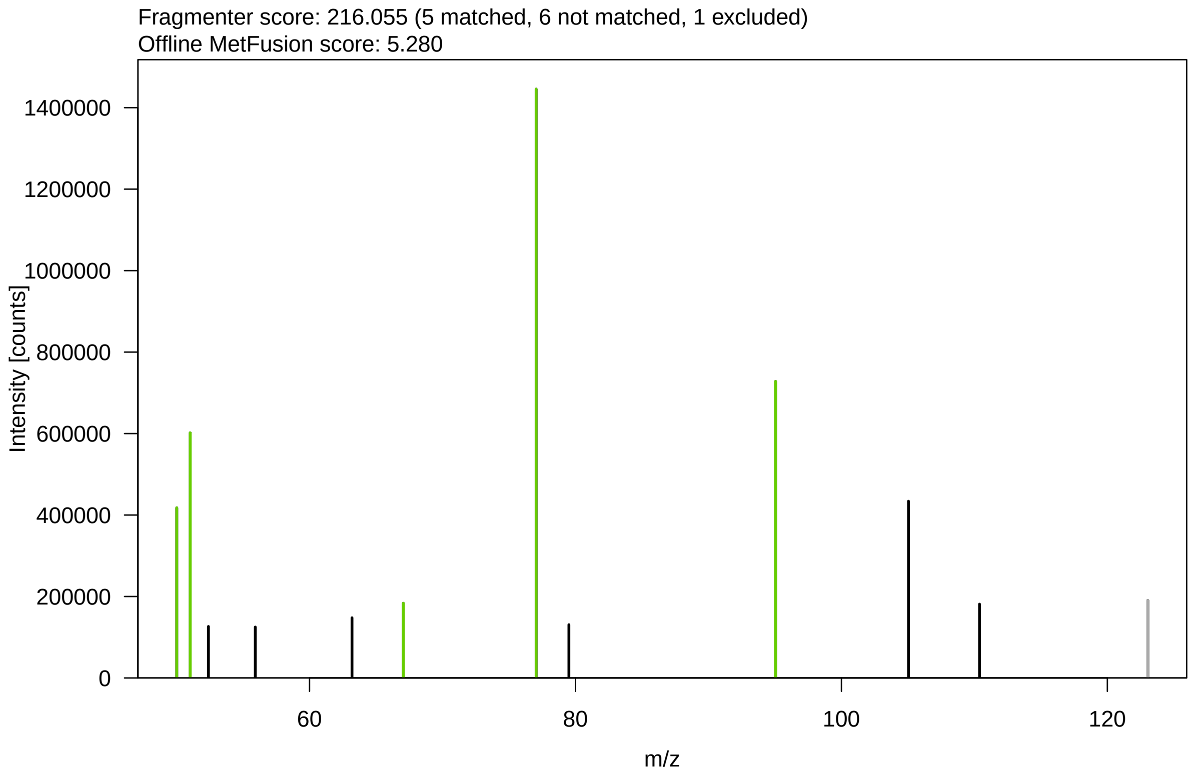

Figure SI-D486: Measured MS2 spectrum. Matching fragments with 4-hydroxybenzaldehyde predicted by MetFrag are highlighted in green. The molecular ion in gray is not considered.

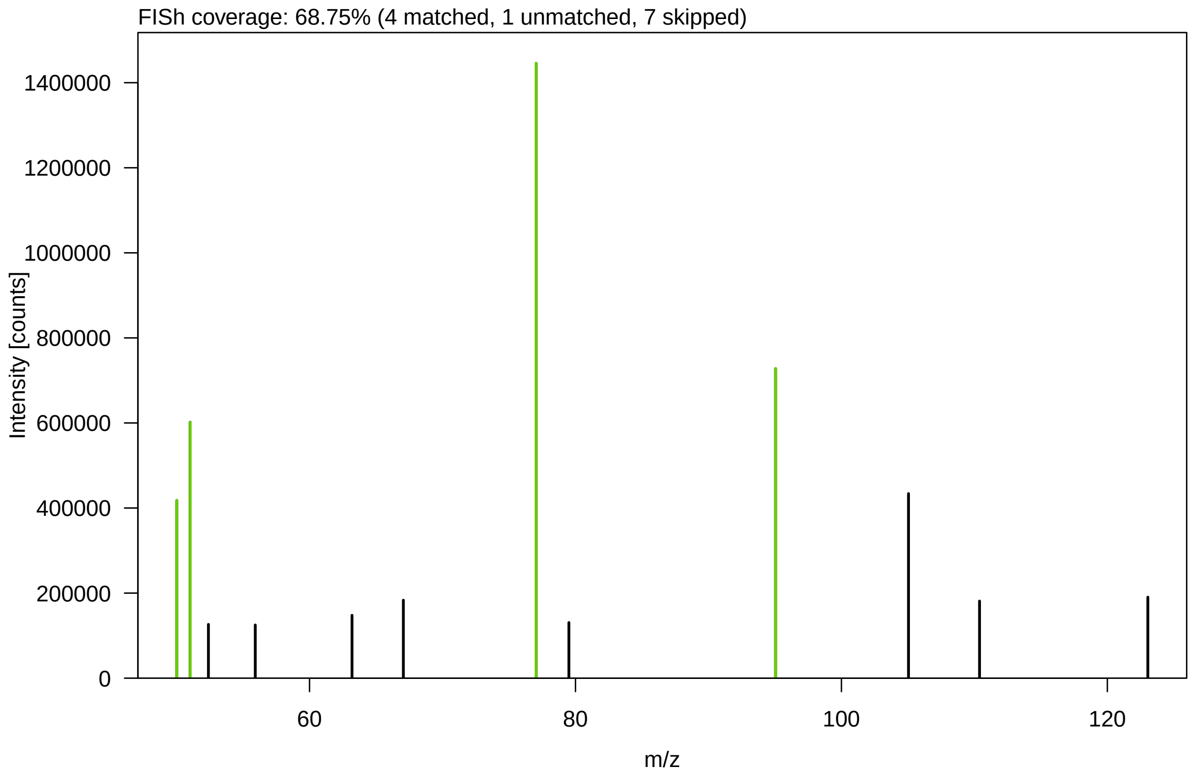

Figure SI-D487: Measured MS2 spectrum. Matching fragments with 4-hydroxybenzaldehyde predicted by FISh Scoring are highlighted in green. Low intensity fragments are not considered and skipped.

Table SI-D248: Retention time prediction of 4-hydroxybenzaldehyde.

Measured retention time [min]	13.6
Predicted $logD_{OW}$ (pH = 2.7)	1.38
Predicted retention time [min]	16.54
Predicted retention time range (95% confidence interval) [min]	11.9-21.1
Predicted retention time range (99% confidence interval) [min]	10.5-22.6

Table SI-D249: Annotated MS2 spectrum of 4-hydroxybenzaldehyde.

m/z	Relative Intensity	Annotation
50.0155	288.78	$C_4H + H^+$
51.0232	415.89	$C_4H_2 + H^+$
52.3950	87.40	
55.9176	86.45	
63.1934	102.15	
67.0544	126.71	$C_5H_6 + H^+$
77.0387	999.00	$C_6H_4 + H^+$
79.5049	90.37	
95.0490	503.09	$C_6H_6O + H^+$
105.0447	299.90	
110.3869	125.30	
123.0439	131.59	$C_7H_6O_2 + H^+$

A reference standard of 4-hydroxybenzaldehyde was purchased. Figure SI-D488 shows the extracted ion chromatograms of this standard, the sample and the spiked sample, as well as a head to tail plot of the MS2 spectra of the standard and the sample. In addition, the most intense MS2 fragments in the sample and in the standard are displayed. It becomes visible that the retention times of the sample and the spiked sample are not matching. The suspected compound elutes earlier than 4-hydroxybenzaldehyde. Nonetheless, the sample and reference standard spectra show a spectral similarity score of 0.519. Almost all fragments can be explained, but the intensity distribution is different. It is therefore hypothesized, that the suspected compound is an isomer of 4-hydroxybenzaldehyde. As a consequence, the confidence level is decreased to level 4.

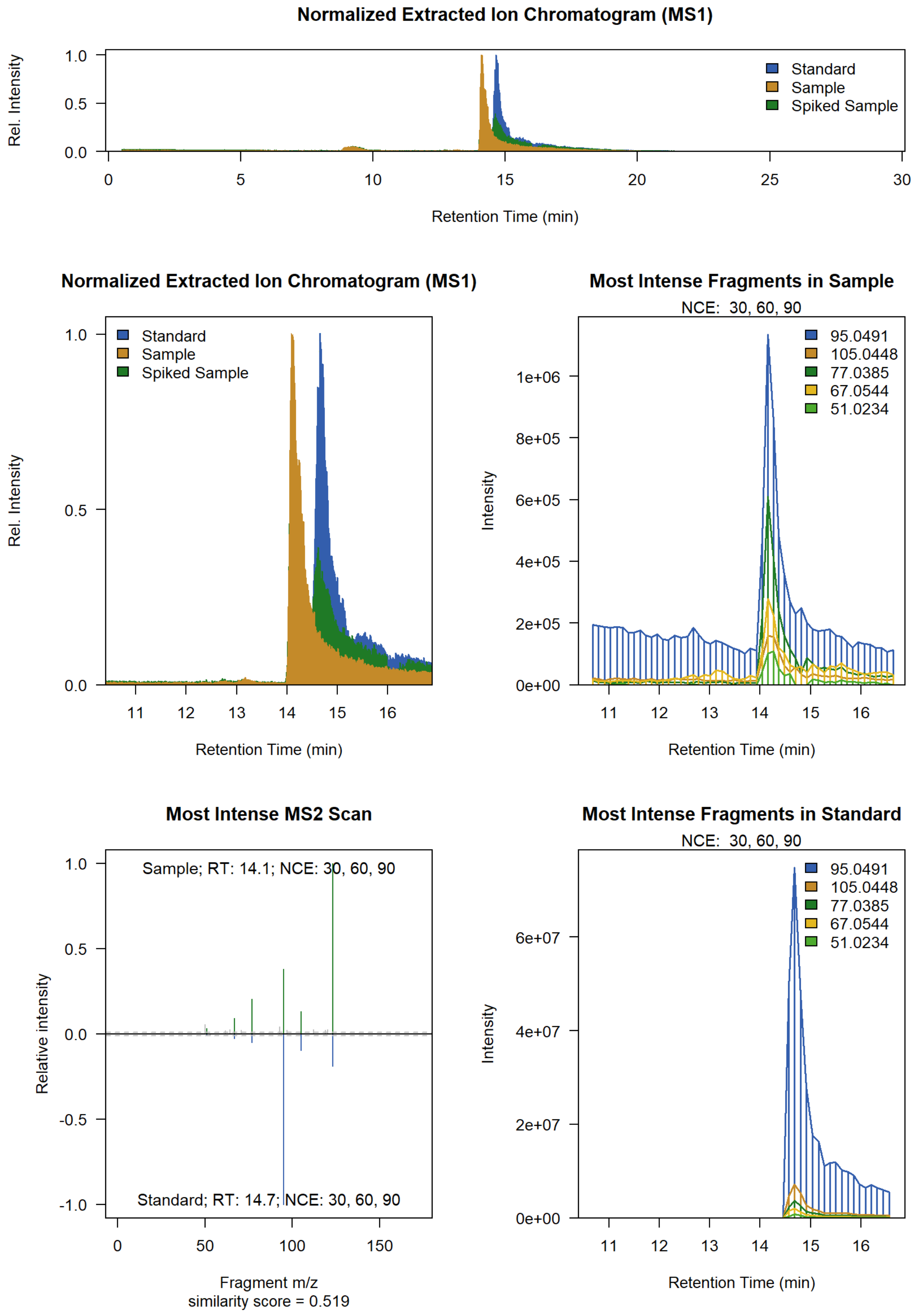

Figure SI-D488: Extracted ion chromatograms of 4-hydroxybenzaldehyde in the reference standard, the sample and the spiked sample, as well as MS2 head to tail plot and most intense MS2 fragments in standard and sample.

SI-D4.3 Hexa(methoxymethyl)melanine (HMMM)

Hexa(methoxymethyl)melanine (HMMM) is contained in resins that are used as a cross-linking agent in organic coatings and plastics.[25] It has been shown that HMMM is associated with acute toxic effects on daphnia[26] and that it is omnipresent is German rivers.[27] It is hypothesized that HMMM is discharged into the rivers with industrial wastewater related to automotive industry and related branches.[27] This hypothesis can be supported by this study, since the highest concentration of HMMM were found in the influent of the WWTP Altenrhein, which receives industrial watewater from a company, which produces railway vehicles. The molecular structure is shown in Figure SI-D489.

Table SI-D250: Information on identifiers, chemical properties, detection and confidence of identification of hexa(methoxymethyl)melanine (HMMM).

IUPAC Name	2-*N*,2-*N*,4-*N*,4-*N*,6-*N*,6-*N*-hexakis(methoxymethyl)-1,3,5-triazine-2,4,6-triamine
Molecular formula	$C_{15}H_{30}N_6O_6$
Monoisotopic mass [g/mol]	390.2227
Adduct	$[M+H]^+$
Retention time [min]	18.6
SMILES	COCN(COC)C1=NC(=NC(=N1)N(COC)COC)N(COC)COC
InChI	InChI=1S/C15H30N6O6/c1-22-7-19(8-23-2)13-16-14(20(9-24-3)10-25-4)18-15(17-13)21(11-26-5)12-27-6/h7-12H2,1-6H3
InChI-Key	BNCADMBVWNPPIZ-UHFFFAOYSA-N
CAS RN	3089-11-0
Detection frequency	33% (5/15 samples)
Detected in	Altenrhein, Monday-Friday
	Neugut, Monday-Friday
	Werdhoelzli, Monday-Friday
Intensity	E7-E10
Initial confidence level	level 2a
Initial confidence score	0.62
Final confidence level	level 1

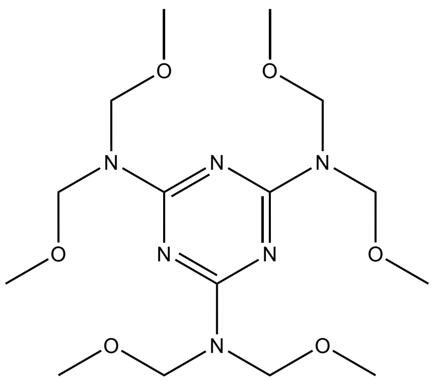

Figure SI-D489: Molecular structure of hexa(methoxymethyl)melanine.

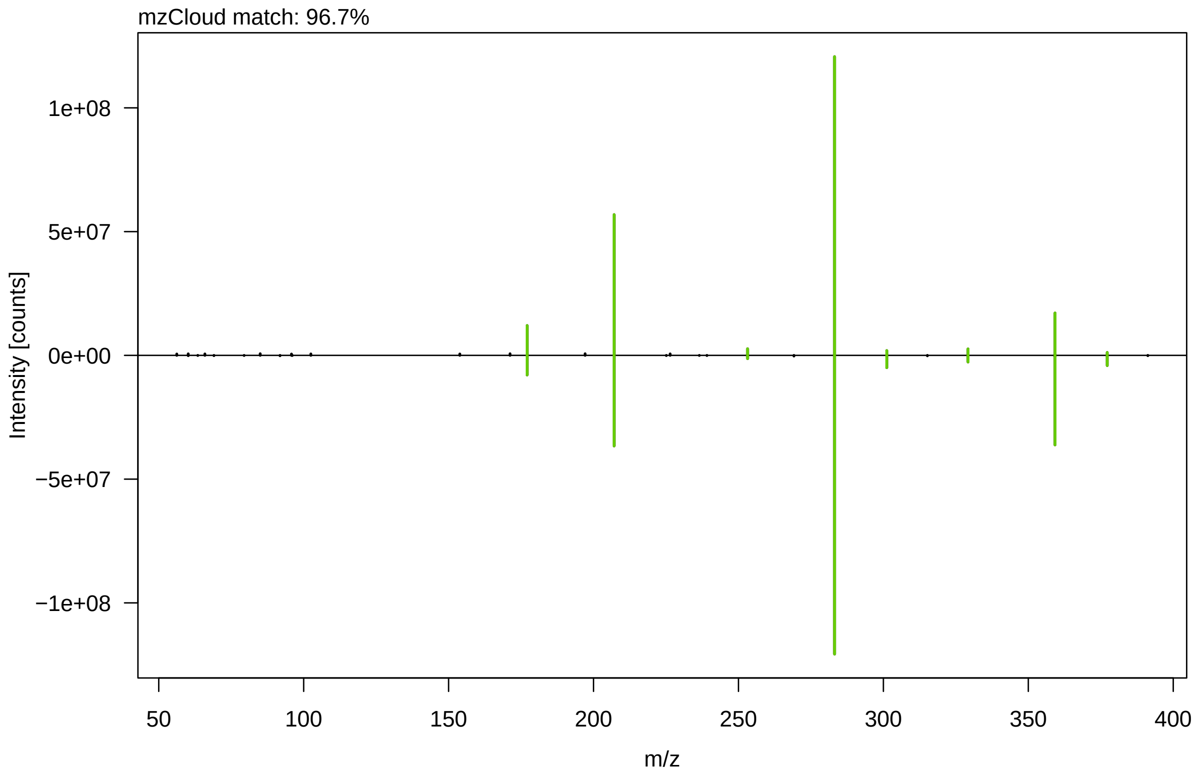

Figure SI-D490: Head to tail plot of measured MS2 spectrum against mzCloud library spectrum of hexa(methoxymethyl)melanine. Matching fragments are highlighted in green.

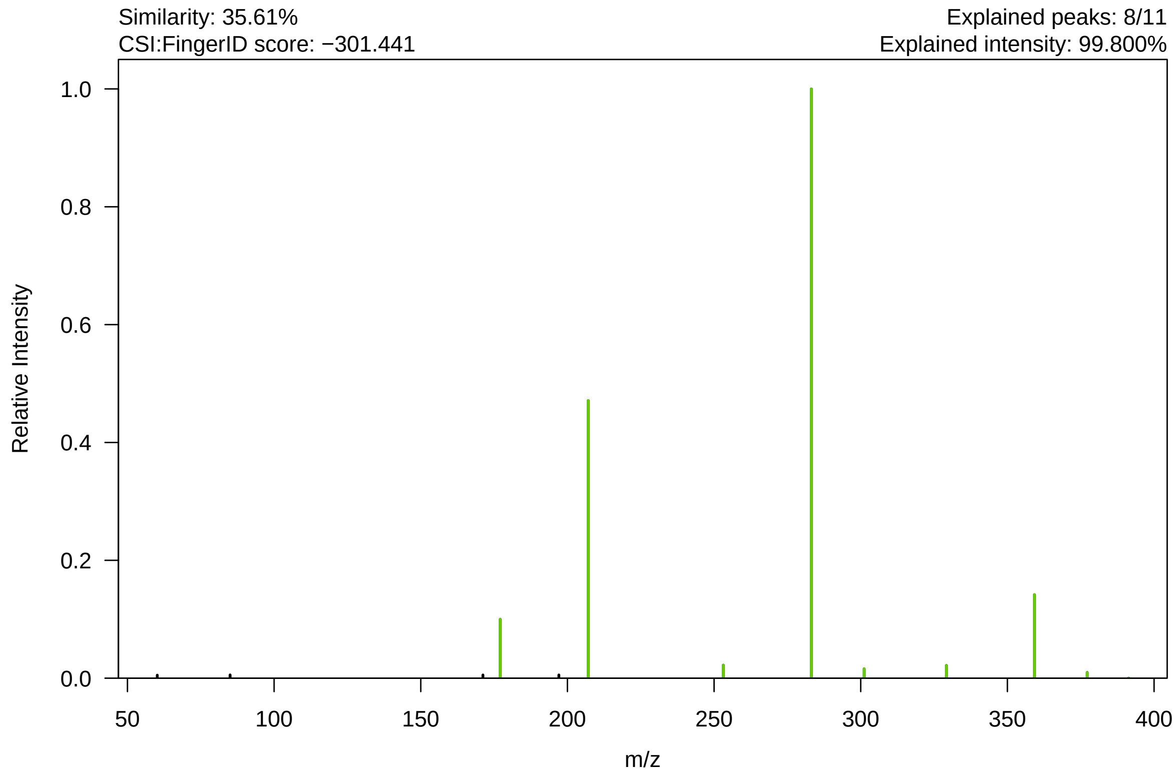

Figure SI-D491: Measured MS2 spectrum. Matching fragments with hexa(methoxymethyl)melanine predicted by SIRIUS/CSI:FingerID are highlighted in green.

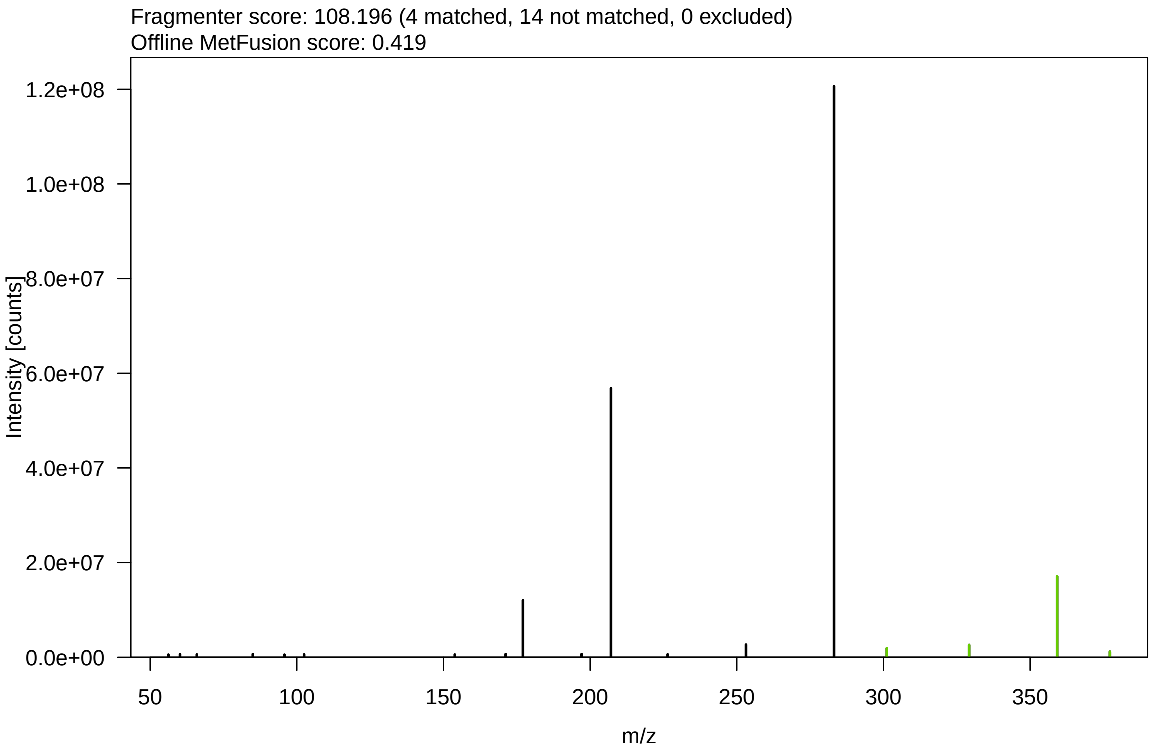

Figure SI-D492: Measured MS2 spectrum. Matching fragments with hexa(methoxymethyl)melanine predicted by MetFrag are highlighted in green.

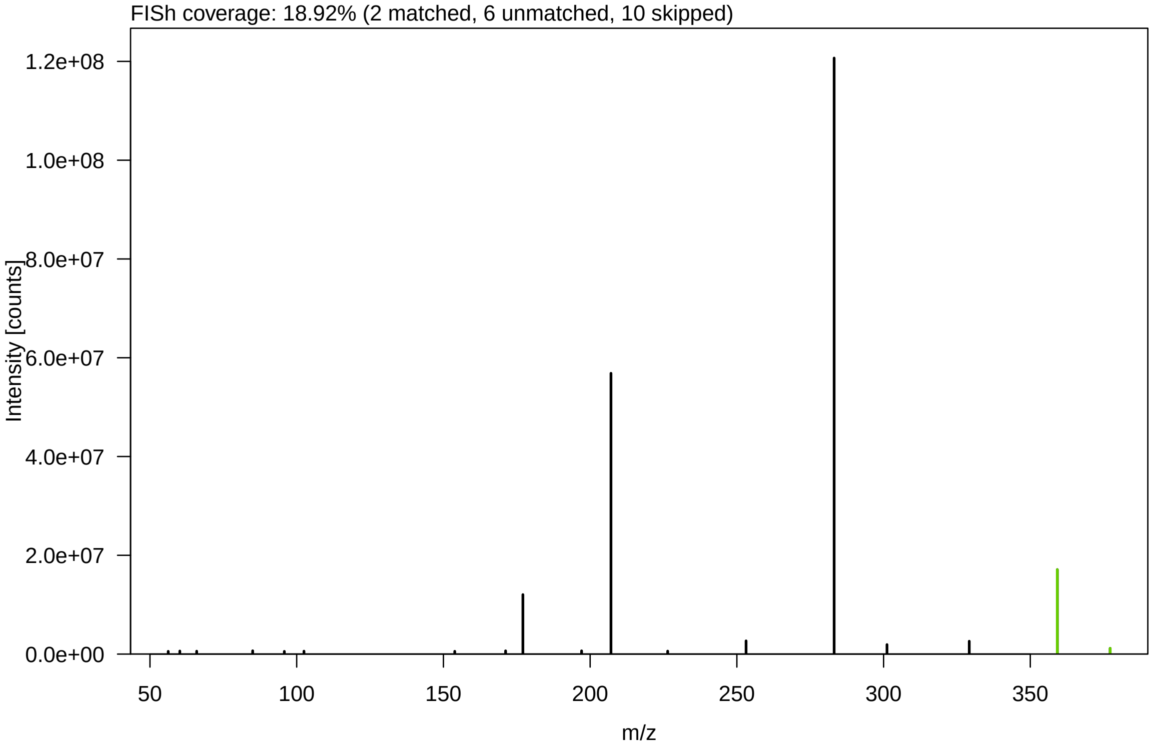

Figure SI-D493: Measured MS2 spectrum. Matching fragments with hexa(methoxymethyl)melanine predicted by FISh Scoring are highlighted in green. Low intensity fragments are not considered and skipped.

Table SI-D251: Retention time prediction of hexa(methoxymethyl)melamine.

Measured retention time [min]	18.6
Predicted $logD_{OW}$ (pH = 2.7)	0.64
Predicted retention time [min]	15.6
Predicted retention time range (95% confidence interval) [min]	11.0-20.1
Predicted retention time range (99% confidence interval) [min]	9.5-21.6

Table SI-D252: Annotated MS2 spectrum of hexa(methoxymethyl)melanine.

m/z	Relative Intensity	Annotation
56.2217	4.60	
60.1705	5.14	
65.9187	4.86	
84.9981	5.56	
95.7895	4.52	
102.4800	4.87	
153.8655	4.67	
171.1938	5.45	
177.0883	99.71	$C_7H_8N_6 + H^+$
197.0896	5.46	
207.0989	470.63	$C_8H_{10}N_6O + H^+$
226.4304	4.99	
253.1405	22.14	$C_{10}H_{16}N_6O_2 + H^+$
283.1512	999.00	$C_{11}H_{18}N_6O_3 + H^+$
301.1611	15.90	$C_{11}H_{20}N_6O_4 + H^+$
329.1932	21.59	$C_{13}H_{24}N_6O_4 + H^+$
359.2036	141.58	$C_{14}H_{26}N_6O_5 + H^+$
377.2138	9.68	$C_{14}H_{28}N_6O_6 + H^+$

A reference standard of HMMM was purchased. Figure SI-D494 shows the extracted ion chromatograms of this standard, the sample and the spiked sample, as well as a head to tail plot of the MS2 spectra of the standard and the sample. In addition, the most intense MS2 fragments in the sample and in the standard are displayed. It becomes visible that the retention times of the sample and the spiked sample are identical and the spectra similarity score between sample and standard is equal to 0.995. The vast majority of the sample fragments are explained by the reference standard. It can therefore be concluded that the suspected compound is indeed hexa(methoxymethyl)melamine. Correspondingly, the identification confidence can be increased to level 1.

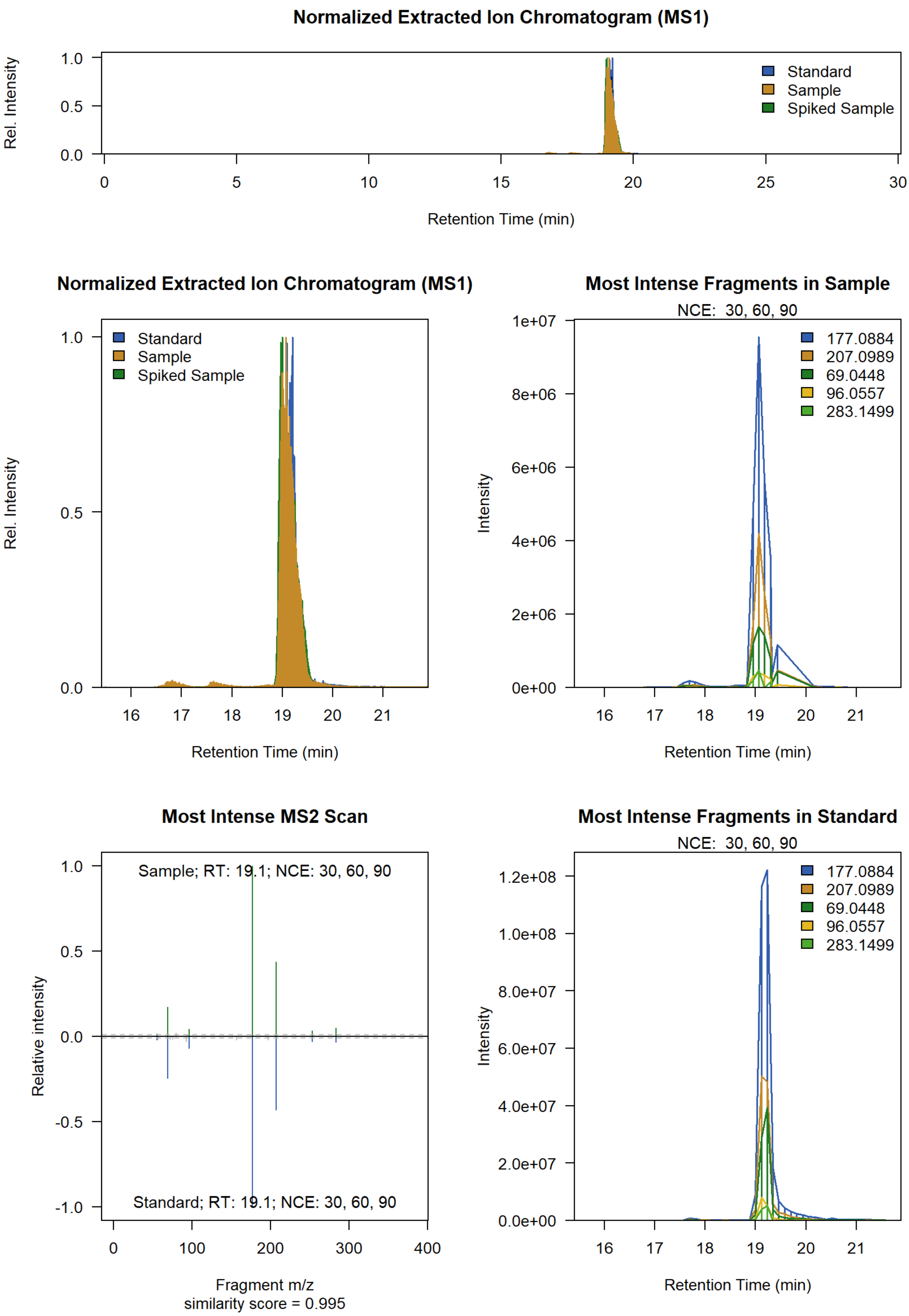

Figure SI-D494: Extracted ion chromatograms of HMMM in the reference standard, the sample and the spiked sample, as well as MS2 head to tail plot and most intense MS2 fragments in standard and sample.

SI-D4.4 Methyl Anthranilate

Methyl anthranilate, also known as Nevoli oil, is used in the fragrance industry to achieve wild fruit or orange blossom notes. It is naturally present in jasmine, grapes and citrus fruits.[28] The moelcular structure is shown in Figure SI-D495.

Table SI-D253: Information on identifiers, chemical properties, detection and confidence of identification of methyl anthranilate.

IUPAC Name	methyl 2-aminobenzoate
Molecular formula	$C_8H_9NO_2$
Monoisotopic mass [g/mol]	151.0633
Adduct	$[M+H]^+$
Retention time [min]	17.9
SMILES	COC(=O)C1=CC=CC=C1N
InChI	InChI=1S/C8H9NO2/c1-11-8(10)6-4-2-3-5-7(6)9/h2-5H,9H2,1H3
InChI-Key	VAMXMNNIEUEQDV-UHFFFAOYSA-N
CAS RN	134-20-3
Detection frequency	100% (15/15 samples)
Detected in	Altenrhein, Monday-Friday<br>Neugut, Monday-Friday<br>Werdhölzli, Monday-Friday
Intensity	E7-E8
Initial confidence level	level 2a
Initial confidence score	0.42
Final confidence level	level 1

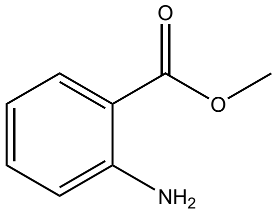

Figure SI-D495: Molecular structure of methyl anthranilate.

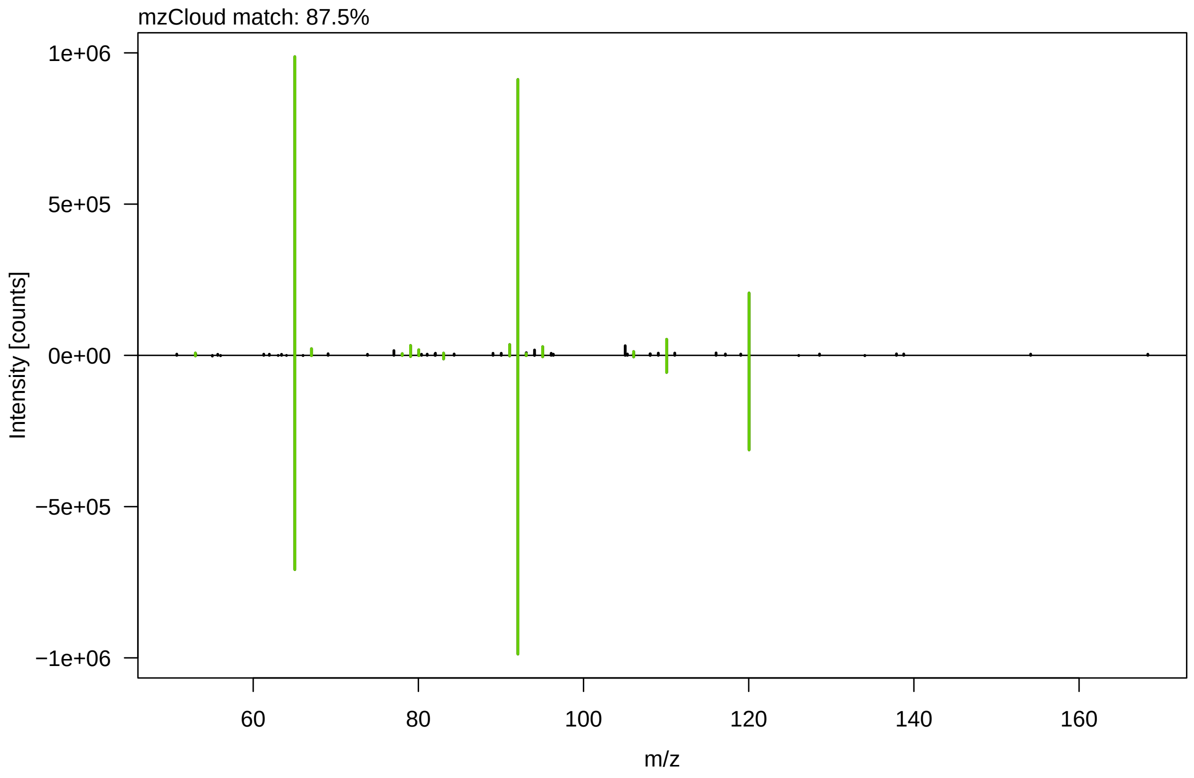

Figure SI-D496: Head to tail plot of measured MS2 spectrum against mzCloud library spectrum of methyl anthranilate. Matching fragments are highlighted in green.

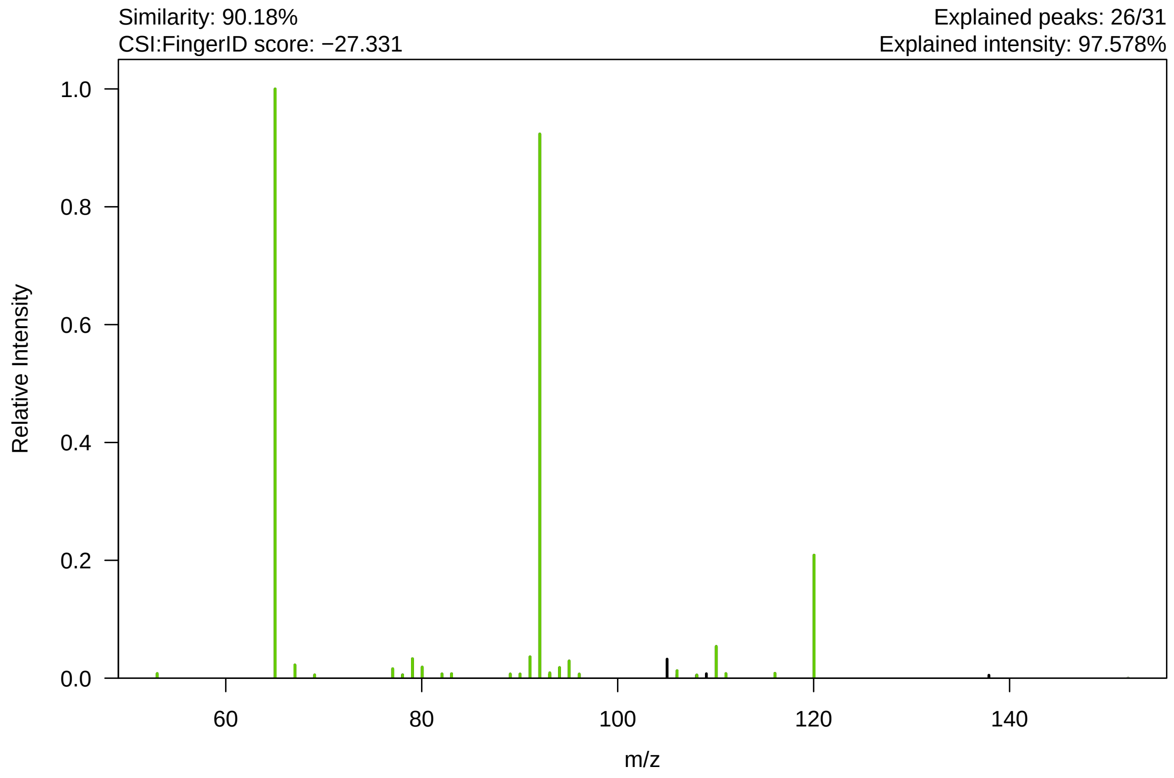

Figure SI-D497: Measured MS2 spectrum. Matching fragments with methyl anthranilate predicted by SIRIUS/CSI:FingerID are highlighted in green. The molecular ion in gray is not considered.

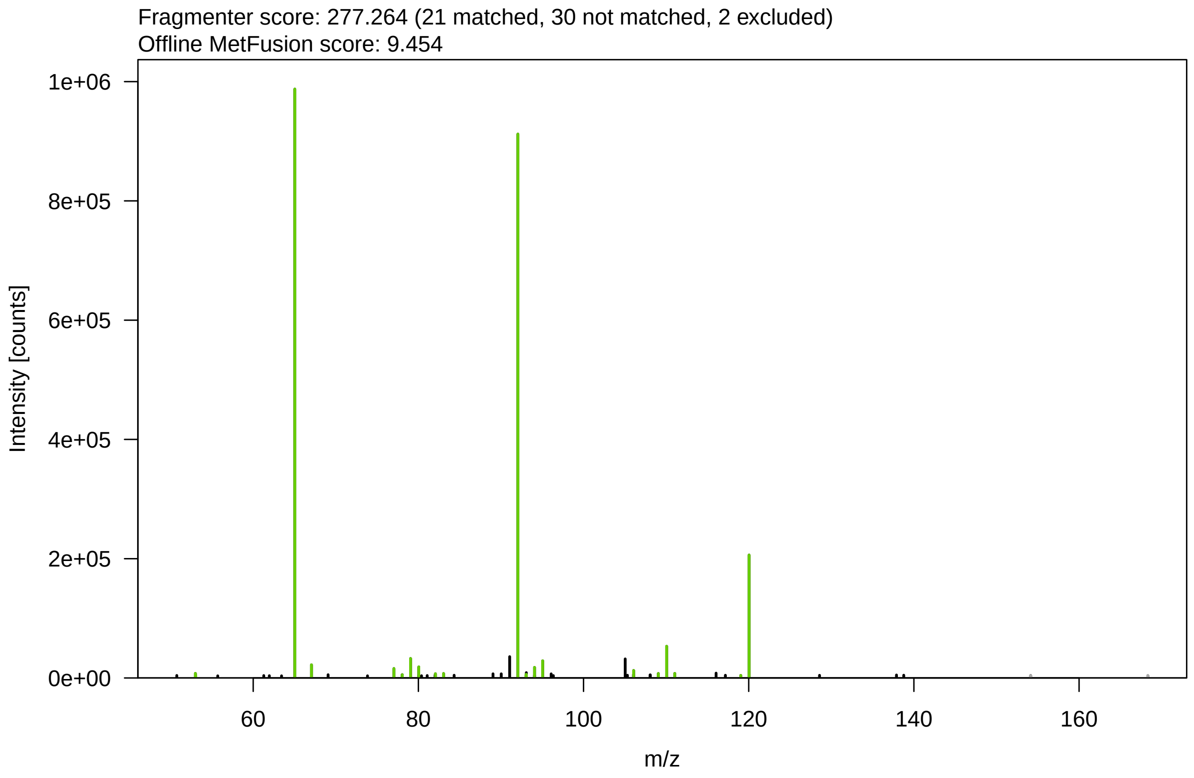

Figure SI-D498: Measured MS2 spectrum. Matching fragments with methyl anthranilate predicted by MetFrag are highlighted in green. The molecular ion in gray is not considered.

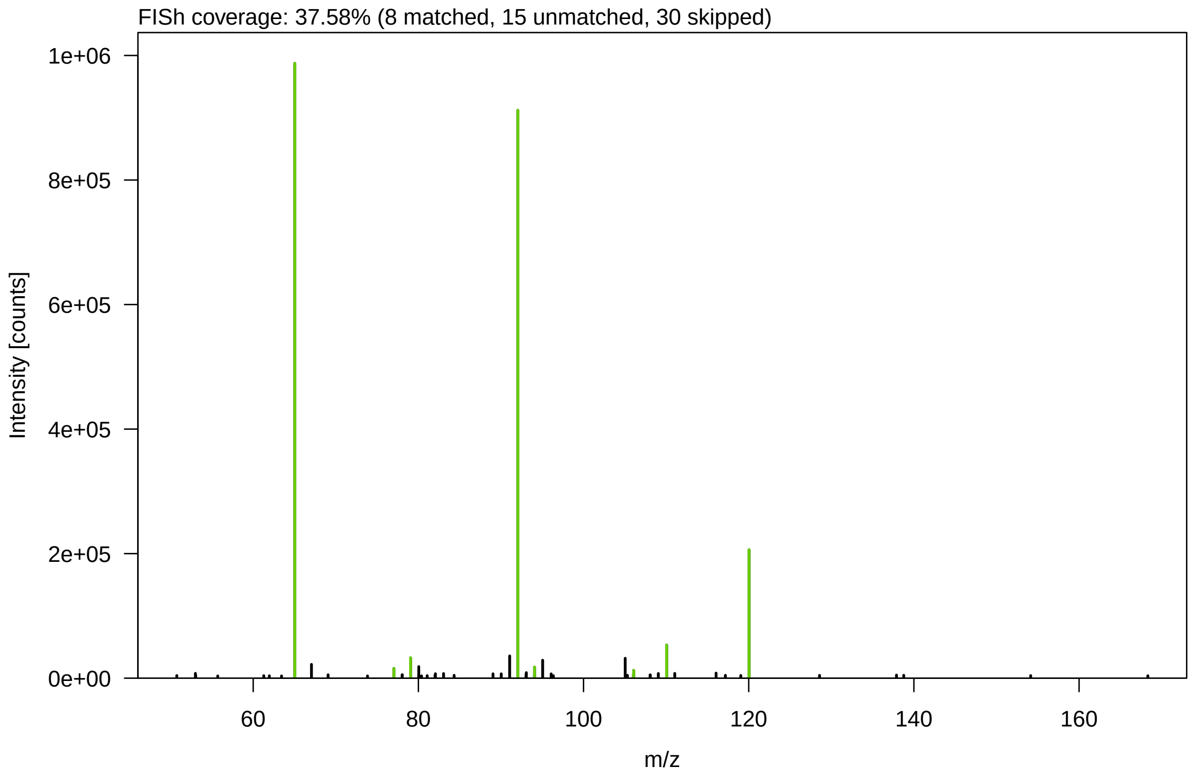

Figure SI-D499: Measured MS2 spectrum. Matching fragments with methyl anthranilate predicted by FISh Scoring are highlighted in green. Low intensity fragments are not considered and skipped.

Table SI-D254: Retention time prediction of methyl anthranilate.

Measured retention time [min]	17.9
Predicted $logD_{OW}$ (pH = 2.7)	1.70
Predicted retention time [min]	17.0
Predicted retention time range (95% confidence interval) [min]	12.4-21.5
Predicted retention time range (99% confidence interval) [min]	10.9-23.0

Table SI-D255: Annotated MS2 spectrum of methyl anthranilate.

m/z	Relative Intensity	Annotation
50.7479	4.25	
53.0026	7.80	$C_3O + H^+$
53.0391	4.32	$C_4H_4 + H^+$
55.7063	3.60	
61.2804	4.08	
61.9545	3.88	
63.4329	3.64	
65.0388	999.00	$C_5H_4 + H^+$
67.0544	22.37	$C_5H_6 + H^+$
69.0702	5.50	$C_5H_8 + H^+$
73.8397	3.63	
77.0386	15.98	$C_6H_4 + H^+$
78.0423	5.54	
78.0465	5.46	$C_6H_5 + H^+$
79.0542	33.01	$C_6H_6 + H^+$
80.0494	18.77	$C_5H_5N + H^+$
80.0570	4.72	
80.3823	3.79	
81.0702	4.13	
82.0166	4.59	
82.0650	7.23	$C_5H_7N + H^+$
83.0490	7.53	$C_5H_6O + H^+$
84.3308	4.63	
89.0384	7.06	$C_7H_4 + H^+$
90.0339	7.05	$C_6H_3N + H^+$
91.0542	36.17	$C_7H_6 + H^+$
92.0495	922.65	$C_6H_5N + H^+$
93.0337	5.60	$C_6H_4O + H^+$
93.0578	6.24	$C_6H_6N + H^+$

Continued on next page

Table SI-D255: Annotated MS2 spectrum of methyl anthranilate.(Continued)

93.0702	9.01	$C_7H_8 + H^+$
94.0594	4.54	
94.0652	17.94	$C_6H_7N + H^+$
95.0491	29.16	$C_6H_6O + H^+$
96.0809	6.97	$C_6H_9N + H^+$
96.3397	4.08	
105.0446	32.33	
105.3095	4.63	
106.0480	5.76	
106.0651	12.70	$C_7H_7N + H^+$
108.0523	5.00	
108.0803	5.32	$C_7H_9N + H^+$
109.0524	7.57	$C_6H_6NO + H^+$
110.0601	53.80	$C_6H_7NO + H^+$
111.0446	7.73	$C_8H_6O_2 + H^+$
116.0494	8.21	$C_8H_5N + H^+$
117.1742	4.59	
119.0372	4.37	$C_7H_4NO + H^+$
120.0443	208.57	$C_7H_5NO + H^+$
128.5728	4.49	
137.8911	5.03	
138.7711	4.67	
154.1395	4.19	
168.3252	3.93	

A reference standard of methyl anthranilate was purchased. Figure SI-D500 shows the extracted ion chromatograms of this standard, the sample and the spiked sample, as well as a head to tail plot of the MS2 spectra of the standard and the sample. In addition, the most intense MS2 fragments in the sample and in the standard are displayed. It becomes visible that the retention times of the sample and the spiked sample are identical and the spectra similarity score between sample and standard is equal to 0.988. The most intense sample fragments are explained by the reference standard. It can therefore be concluded that the suspected compound is indeed methyl anthranilate. Correspondingly, the identification confidence can be increased to level 1. A compound isobaric to methyl anthranilate eluting several minutes earlier is visible in the extracted ion chromatogram.

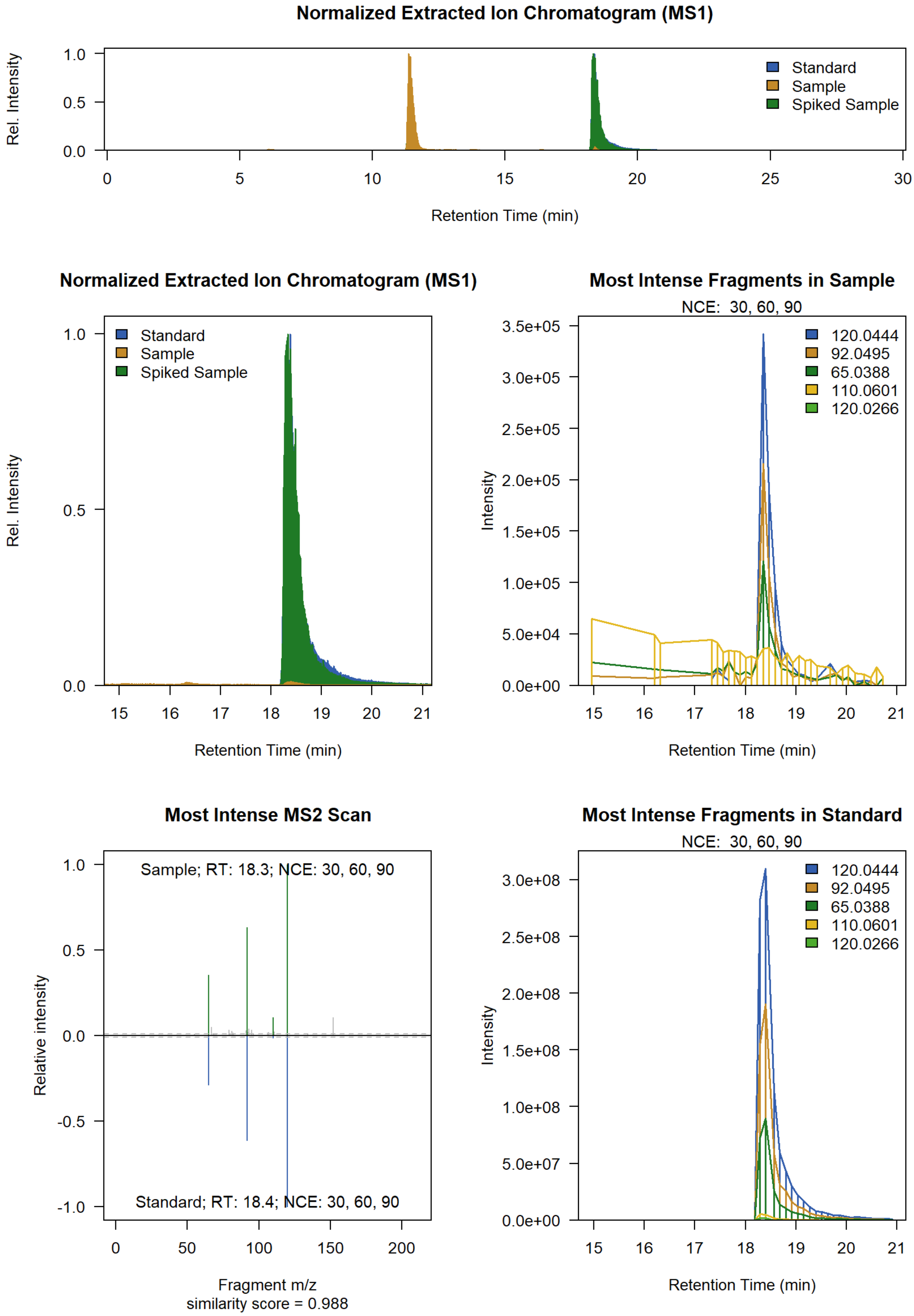

Figure SI-D500: Extracted ion chromatograms of methyl anthranilate in the reference standard, the sample and the spiked sample, as well as MS2 head to tail plot and most intense MS2 fragments in standard and sample. The earlier eluting signal originates from a compound isobaric to methyl anthranilate.

SI-D4.5 Piperine

Piperine is the main alkaloid in black pepper.[29] The molecular structure is shown in Figure SI-D501.

Table SI-D256: Information on identifiers, chemical properties, detection and confidence of identification of piperine.

IUPAC Name	(2*E*,4*E*)-5-(1,3-benzodioxol-5-yl)-1-piperidin-1-ylpenta-2,4-dien-1-one
Molecular formula	$C_{17}H_{19}NO_3$
Monoisotopic mass [g/mol]	285.1365
Adduct	$[M+H]^+$
Retention time [min]	20.9
SMILES	C1CCN(CC1)C(=O)/C=C/C=C/C2=CC3=C(C=C2)OCO3
InChI	InChI=1S/C17H19NO3/c19-17(18-10-4-1-5-11-18)7-3-2-6-14-8-9-15-16(12-14)21-13-20-15/h2-3,6-9,12H,1,4-5,10-11,13H2/b6-2+,7-3+
InChI-Key	MXXWOMGUGJBKIW-YPCIICBESA-N
CAS RN	94-62-2
Detection frequency	100% (15/15 samples)
Detected in	Altenrhein, Monday-Friday<br>Neugut, Monday-Friday<br>Werdhölzli, Monday-Friday
Intensity	E8-E9
Initial confidence level	level 2a
Initial confidence score	0.58
Final confidence level	level 1

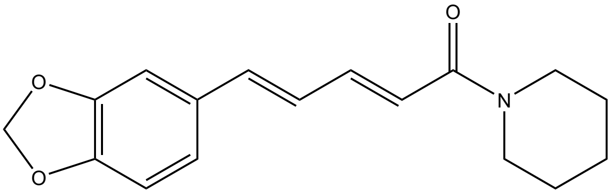

Figure SI-D501: Molecular structure of piperine.

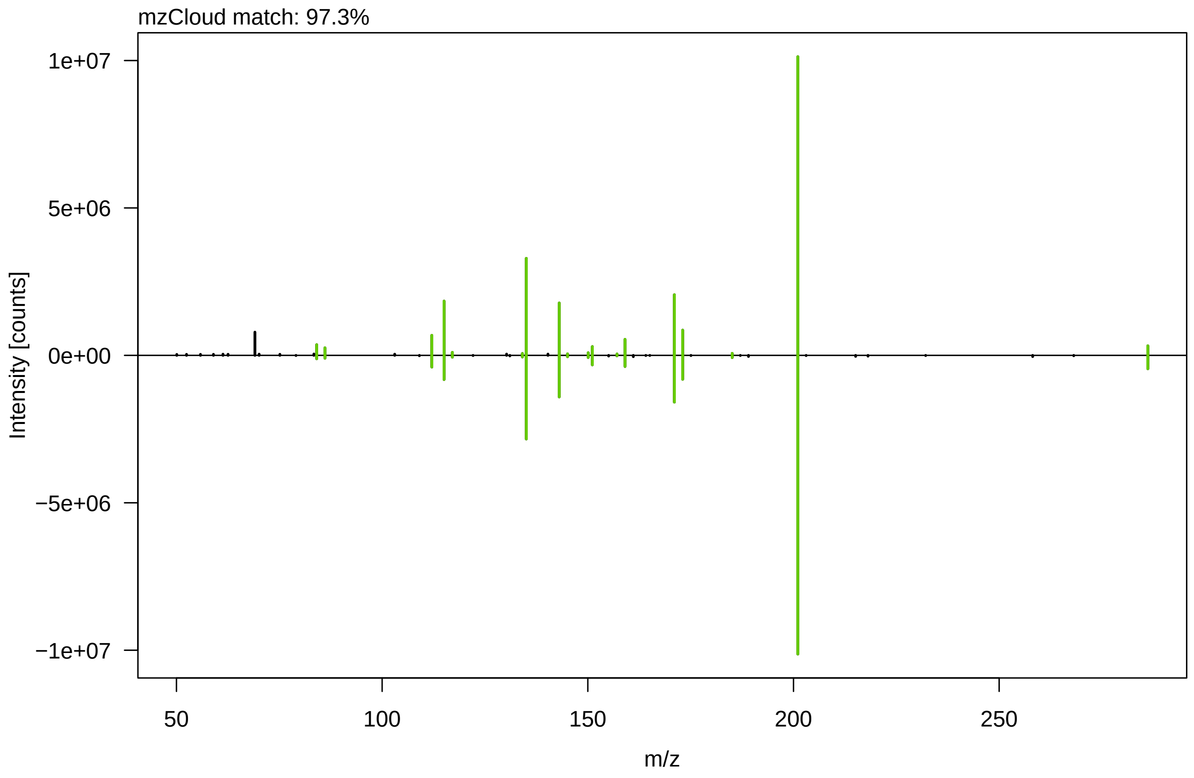

Figure SI-D502: Head to tail plot of measured MS2 spectrum against mzCloud library spectrum of piperine. Matching fragments are highlighted in green.

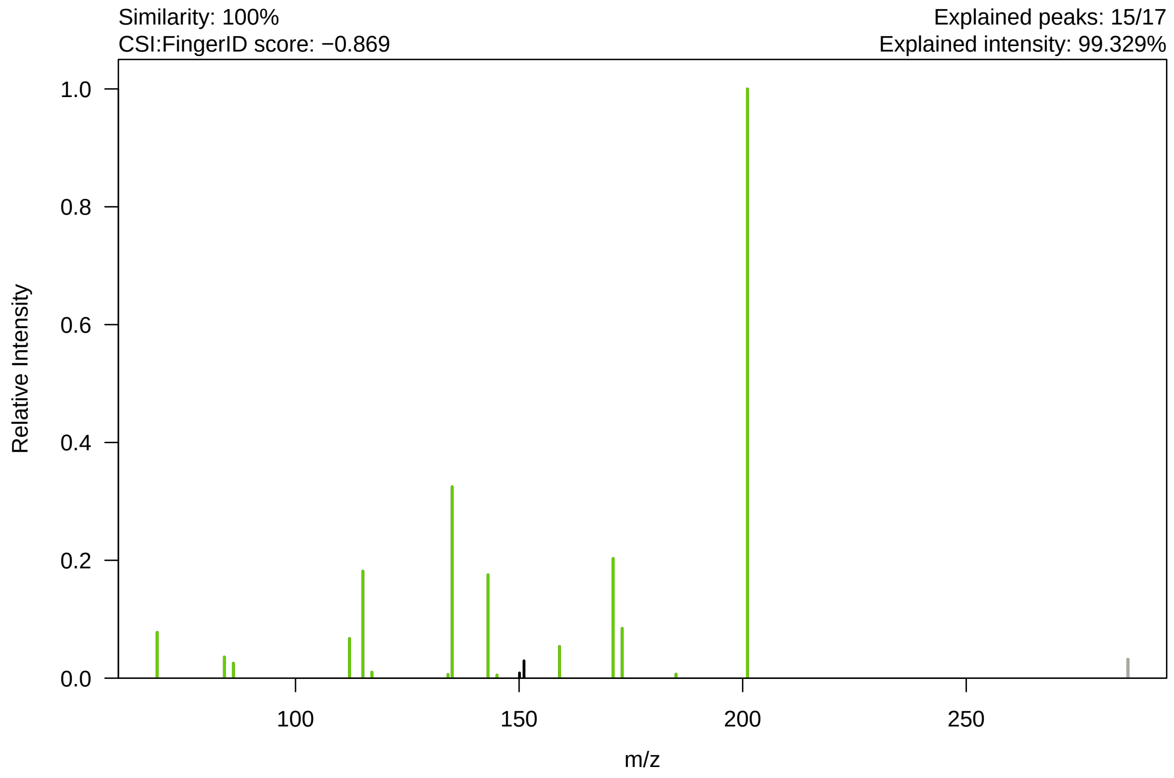

Figure SI-D503: Measured MS2 spectrum. Matching fragments with piperine predicted by SIRIUS/CSI:FingerID are highlighted in green. The molecular ion in gray is not considered.

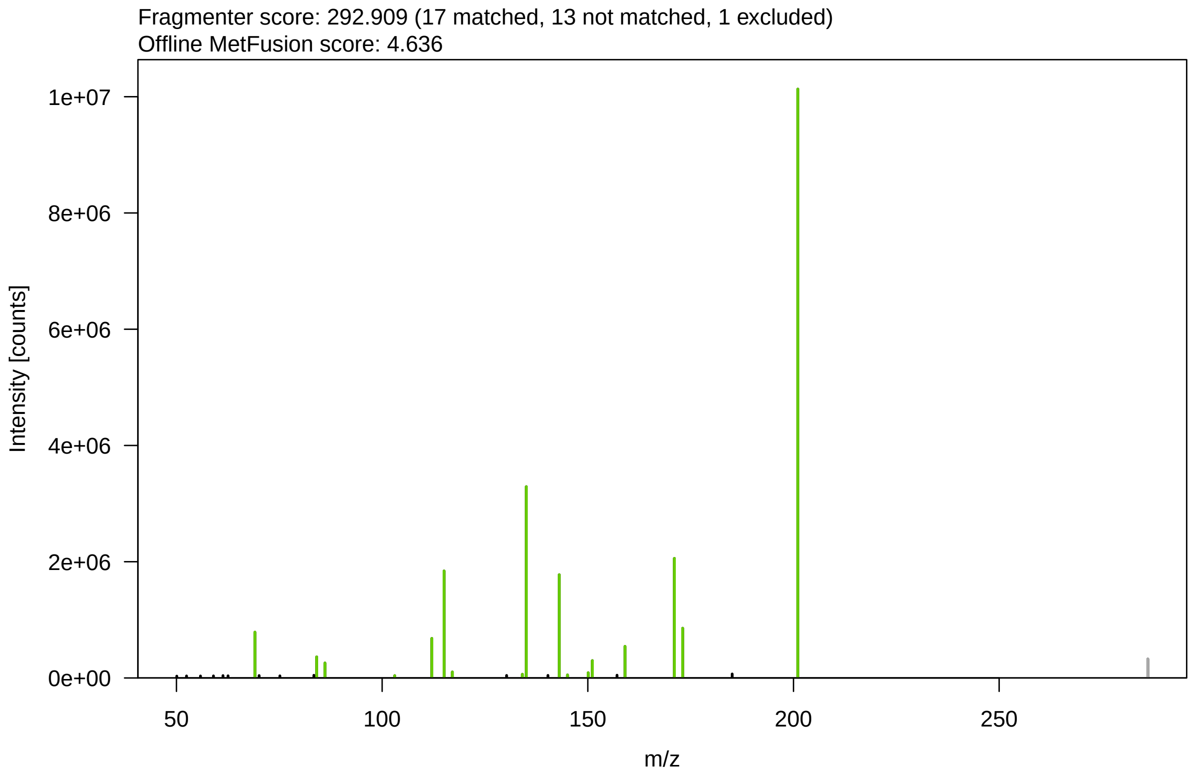

Figure SI-D504: Measured MS2 spectrum. Matching fragments with piperine predicted by MetFrag are highlighted in green. The molecular ion in gray is not considered.

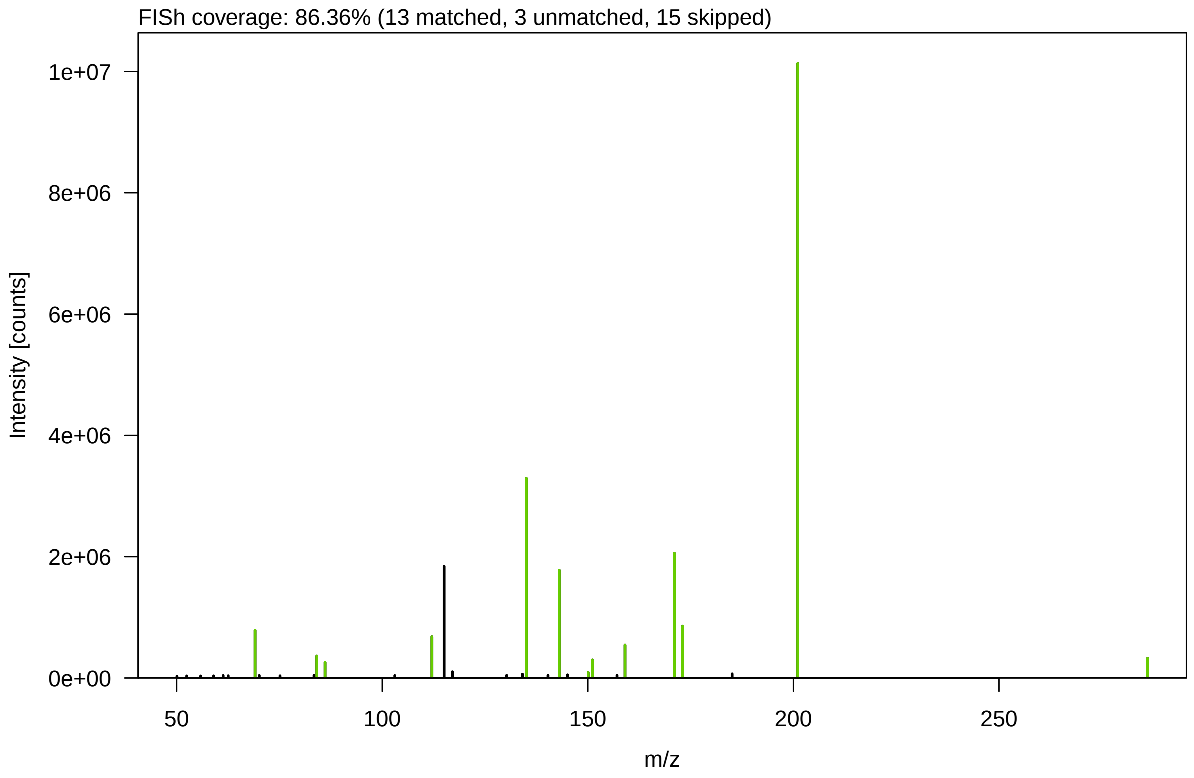

Figure SI-D505: Measured MS2 spectrum. Matching fragments with piperine predicted by FISh Scoring are highlighted in green. Low intensity fragments are not considered and skipped.

Table SI-D257: Retention time prediction of piperine.

Measured retention time [min]	20.9
Predicted $logD_{OW}$ (pH = 2.7)	2.78
Predicted retention time [min]	18.4
Predicted retention time range (95% confidence interval) [min]	13.8-23.0
Predicted retention time range (99% confidence interval) [min]	12.3-24.4

Table SI-D258: Annotated MS2 spectrum of piperine.

m/z	Relative Intensity	Annotation
50.0756	3.09	
52.4466	3.41	
55.8366	3.35	
58.9919	3.50	
61.3073	4.02	
62.5392	3.69	
69.0700	77.68	$C_5H_8 + H^+$
70.0917	4.08	
75.1378	3.57	
83.4044	4.57	
84.0809	35.80	$C_5H_9N + H^+$
86.0962	25.40	$C_5H_{11}N + H^+$
103.0544	3.97	$C_8H_6 + H^+$
112.0756	67.15	$C_6H_9NO + H^+$
115.0541	181.53	$C_9H_6 + H^+$
117.0694	10.19	$C_9H_8 + H^+$
130.2590	4.28	
134.0960	6.30	$C_9H_{11}N + H^+$
135.0439	324.55	$C_8H_6O_2 + H^+$
140.3100	4.33	
143.0491	175.18	$C_{10}H_6O + H^+$
145.0653	5.20	$C_{10}H_8O + H^+$
150.0909	8.90	$C_9H_{11}NO + H^+$
151.0990	29.43	$C_9H_{12}NO + H^+$
157.1026	4.69	
159.0438	53.67	$C_{10}H_6O_2 + H^+$
171.0438	202.87	$C_{11}H_6O_2 + H^+$
173.0595	84.40	$C_{11}H_8O_2 + H^+$
185.0960	6.79	$C_{13}H_{12}O + H^+$

Continued on next page

Table SI-D258: Annotated MS2 spectrum of piperine.(Continued)

201.0544	999.00	$C_{12}H_8O_3 + H^+$
286.1438	32.08	$C_{17}H_{19}NO_3 + H^+$

A reference standard of piperine was purchased. Figure SI-D506 shows the extracted ion chromatograms of this standard, the sample and the spiked sample, as well as a head to tail plot of the MS2 spectra of the standard and the sample. In addition, the most intense MS2 fragments in the sample and in the standard are displayed. It becomes visible that the retention times of the sample and the spiked sample are identical and the spectra similarity score between sample and standard is equal to 0.996. The vast majority of the sample fragments are explained by the reference standard. It can therefore be concluded that the suspected compound is indeed piperine. Correspondingly, the identification confidence can be increased to level 1.

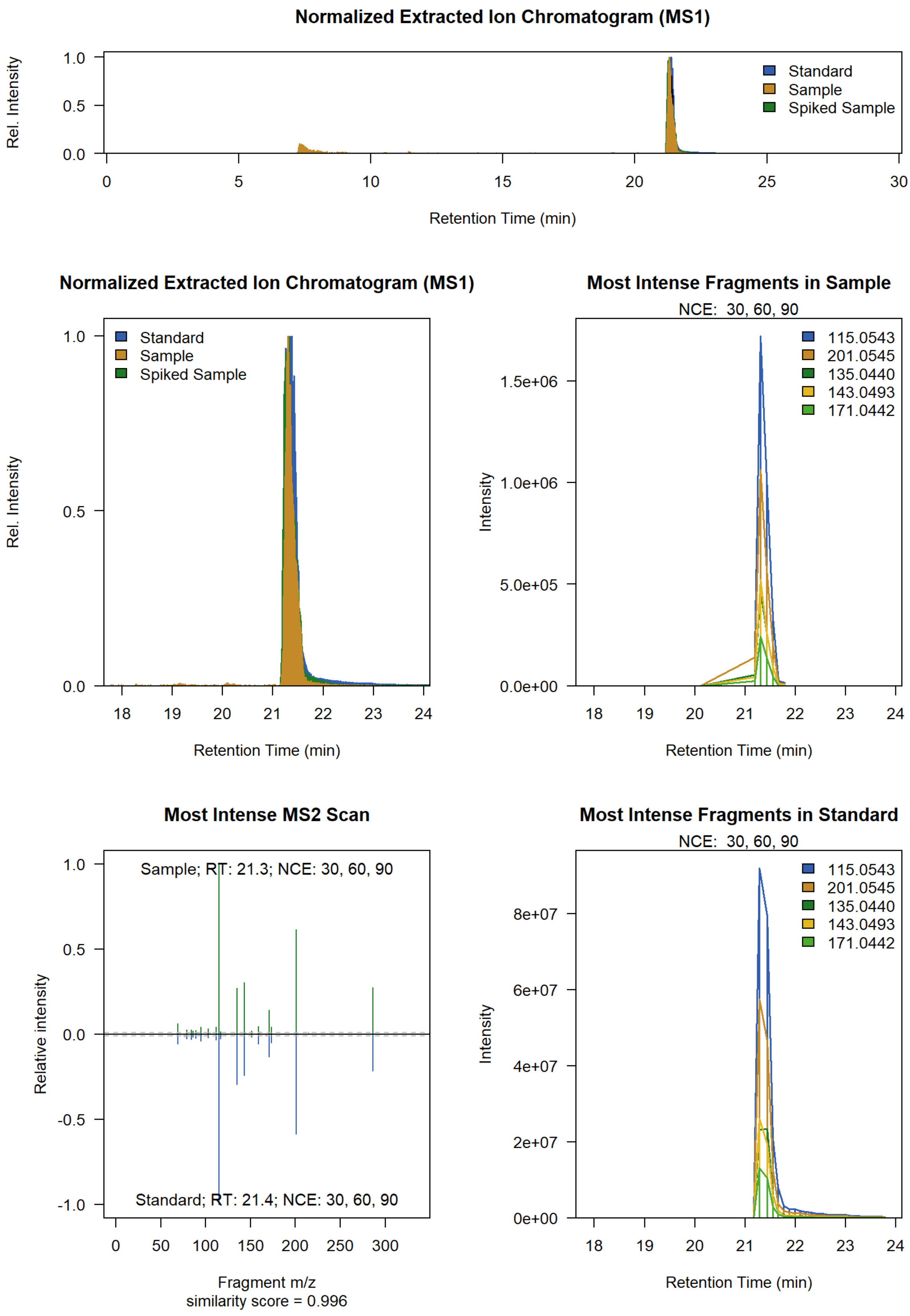

Figure SI-D506: Extracted ion chromatograms of piperine in the reference standard, the sample and the spiked sample, as well as MS2 head to tail plot and most intense MS2 fragments in standard and sample.

SI-D4.6 Sulfurol

Sulfurol is a food additive and fragrance. Its odour is described as fatty cooked beef juice in higher concentrations. When diluted, its odour becomes a milk-pudding effect. In the fragrance industry it is used as top-note modifier.[30,31] The moelcular structure is shown in Figure SI-D507.

Table SI-D259: Information on identifiers, chemical properties, detection and confidence of identification of sulfurol.

IUPAC Name	4-Methyl-5-thiazoleethanol
Molecular formula	C_6H_9NOS
Monoisotopic mass [g/mol]	143.0405
Adduct	$[M+H]^+$
Retention time [min]	9.4
SMILES	CC1=C(SC=N1)CCO
InChI	InChI=1S/C6H9NOS/c1-5-6(2-3-8)9-4-7-5/h4,8H,2-3H2,1H3
InChI-Key	BKAWJIRCKVUVED-UHFFFAOYSA-N
CAS RN	137-00-8
Detection frequency	100% (15/15 samples)
Detected in	Altenrhein, Monday-Friday
	Neugut, Monday-Friday
	Werdhölzli, Monday-Friday
Intensity	E8-E10
Initial confidence level	level 2a
Initial confidence score	0.56
Final confidence level	level 1

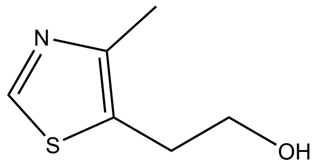

Figure SI-D507: Molecular structure of sulfurol.

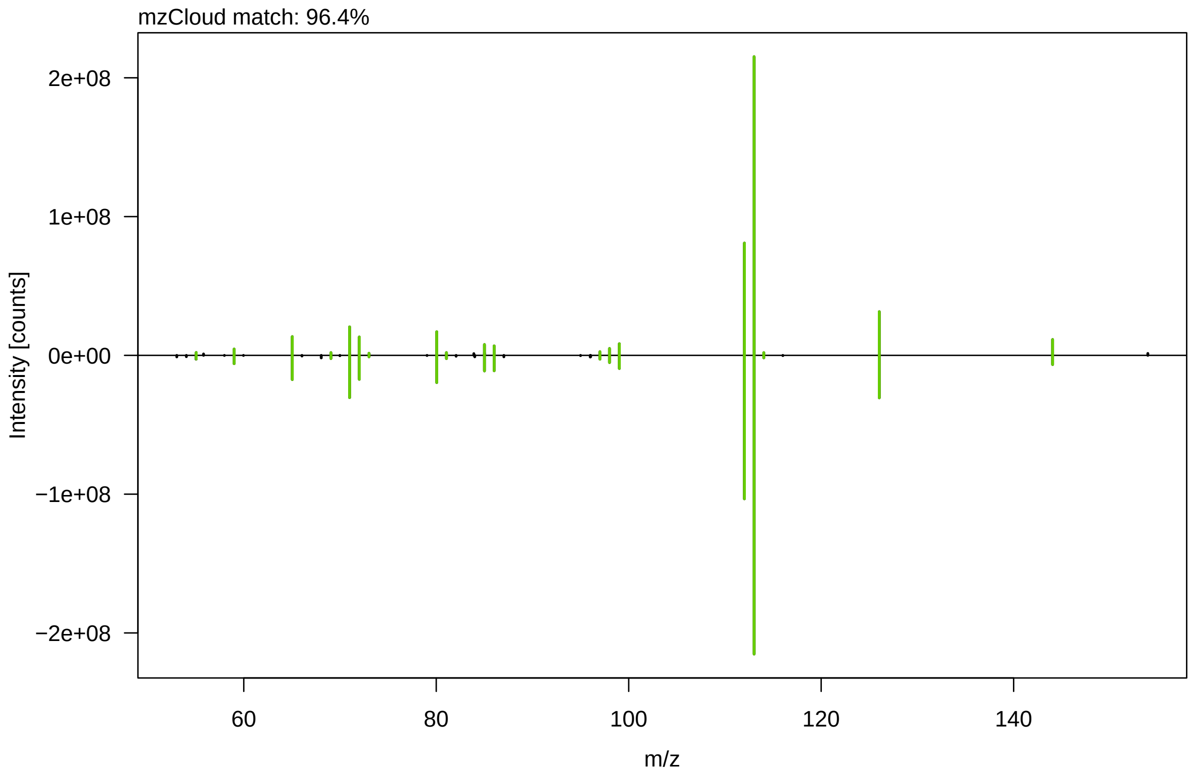

Figure SI-D508: Head to tail plot of measured MS2 spectrum against mzCloud library spectrum of sulfurol. Matching fragments are highlighted in green.

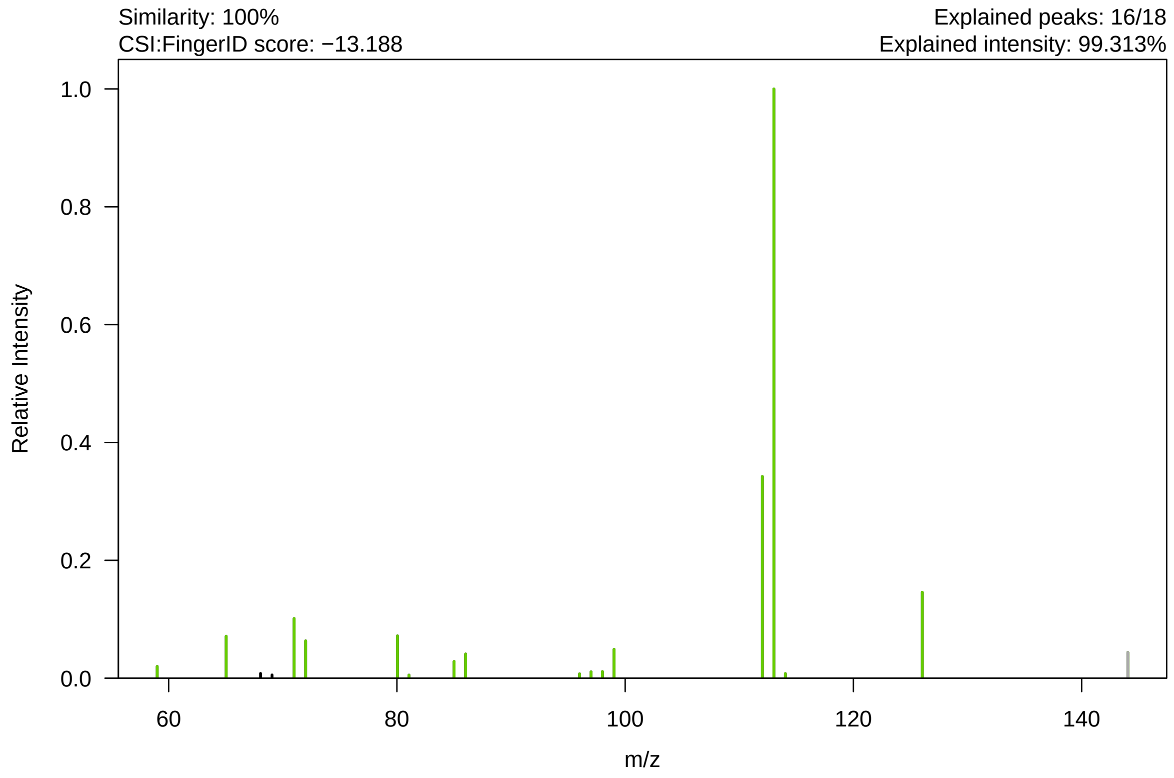

Figure SI-D509: Measured MS2 spectrum. Matching fragments with sulfurol predicted by SIRIUS/CSI:FingerID are highlighted in green. The molecular ion in gray is not considered.

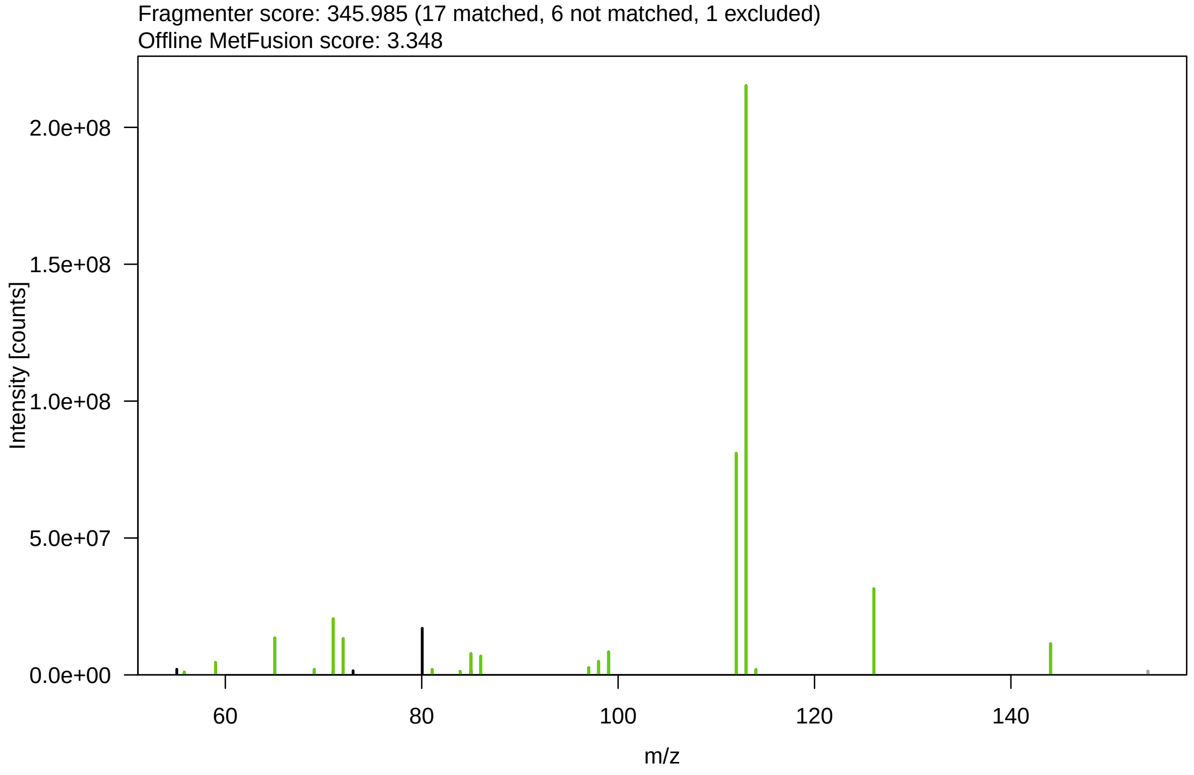

Figure SI-D510: Measured MS2 spectrum. Matching fragments with sulfurol predicted by MetFrag are highlighted in green. The molecular ion in gray is not considered.

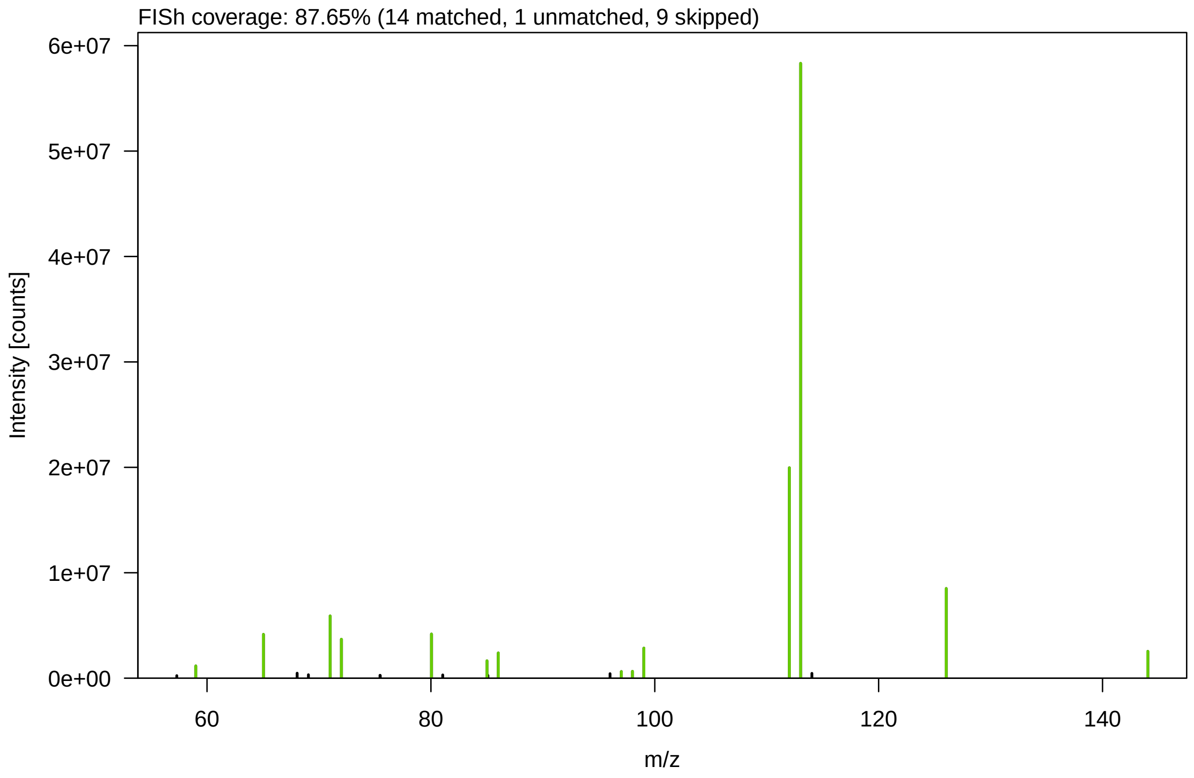

Figure SI-D511: Measured MS2 spectrum. Matching fragments with sulfurol predicted by FISh Scoring are highlighted in green. Low intensity fragments are not considered and skipped.

Table SI-D260: Retention time prediction of sulfurol.

Measured retention time [min]	9.4
Predicted $logD_{OW}$ (pH = 2.7)	0.10
Predicted retention time [min]	14.9
Predicted retention time range (95% confidence interval) [min]	10.3-19.5
Predicted retention time range (99% confidence interval) [min]	8.8-20.9

Table SI-D261: Annotated MS2 spectrum of sulfurol.

m/z	Relative Intensity	Annotation
55.0547	9.54	$C_4H_6 + H^+$
55.8200	4.75	
58.9952	21.09	$C_2H_2S + H^+$
65.0388	62.67	$C_5H_4 + H^+$
69.0572	9.20	$C_4H_6N + H^+$
70.9914	7.52	$C_3H_2S + H^+$
70.9950	95.27	$C_3H_3S + H^+$
72.0030	61.52	$C_3H_4S + H^+$
73.0108	7.08	$C_5H_5N + H^+$
80.0494	79.03	$C_5H_6N + H^+$
81.0572	9.16	$C_5H_7N + H^+$
83.9052	5.86	$C_4H_4S + H^+$
85.0108	36.12	$C_4H_5S + H^+$
85.0150	7.48	
86.0183	31.77	$C_4H_6S + H^+$
97.0108	12.32	$C_5H_4S + H^+$
98.0058	22.90	$C_4H_3NS + H^+$
99.0264	38.98	$C_5H_6S + H^+$
112.0215	375.59	$C_5H_5NS + H^+$
113.0293	999.00	$C_5H_6NS + H^+$
114.0371	9.03	$C_5H_7NS + H^+$
126.0371	146.10	$C_6H_7NS + H^+$
144.0479	52.95	$C_6H_9NOS + H^+$
153.9442	6.41	

A reference standard of sulfurol was purchased. Figure SI-D512 shows the extracted ion chromatograms of this standard, the sample and the spiked sample, as well as a head to tail plot of the MS2 spectra of the standard and the sample. In addition, the most intense MS2 fragments in the sample and in the standard are displayed. It becomes visible that the retention times of the sample and the spiked sample are identical and the spectra similarity score between sample and standard is equal to 1.0. All of the sample fragments are explained by the reference standard. It can therefore be concluded that the suspected compound is indeed sulfurol. Correspondingly, the identification confidence can be increased to level 1.

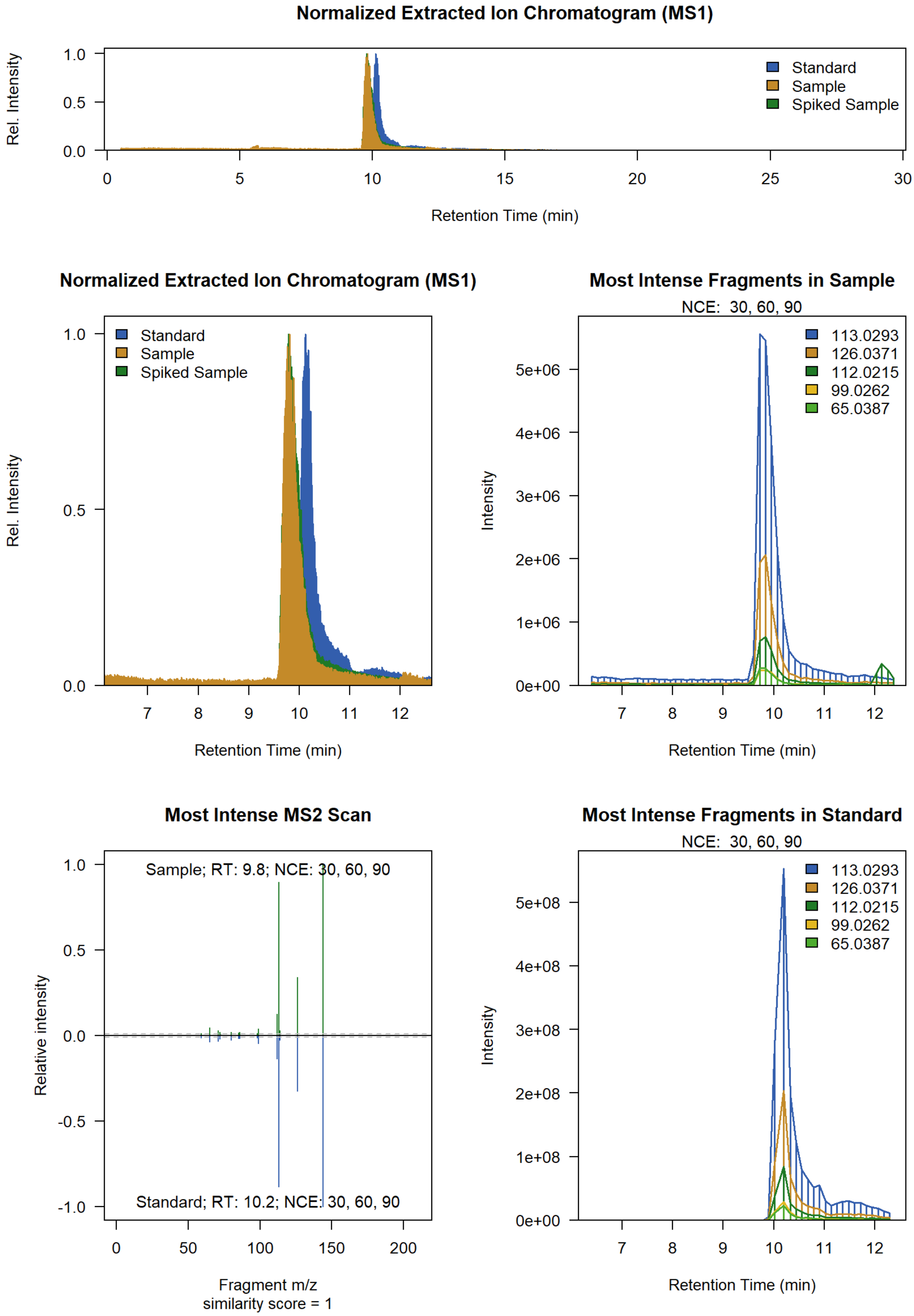

Figure SI-D512: Extracted ion chromatograms of sulfurol in the reference standard, the sample and the spiked sample, as well as MS2 head to tail plot and most intense MS2 fragments in standard and sample.

SI-D5 Non-Target Screening Compounds

SI-D5.1 Glucuronide Conjugates: Positive Ionization Mode

Neutral loss search of glucuronides in the positive ionization mode led to eight matches, three of them being diphenhydramine-N-glucuronide, lamotrigine-N-glucuronide and phenolic glucuronide, respectively, which were already covered by suspect screening. For three of the remaining five matches, the predicted molecular formula did not contain at least six oxygen atoms, which is the minimum required for a glucuronide conjugate. One of the two remaining candidates is isobaric with phenolic glucuronide. Besides phenolic glucuronide with significantly worse similarity and CSI:FigerID scores, no other glucuronide within the 238 candidates was proposed. The last neutral loss match does not reveal any compound with the same molecular formula on PubChem. It has to be noted that SIRIUS/CSI:FingerID and MetFrag only work with the given databases, but do not generate *de novo* structures. Since many phase metabolites are not contained in any database, the applied approach is biased.

SI-D5.2 Glucuronide Conjugates: Negative Ionization Mode

Neutral loss and specific fragment search of glucuroides in the negative ionization mode revealed four matches, one of them being phenolic glucuronide, which was already discovered in suspect screening in positive and negative ionization mode. For two of the other three matches, the predicted composition did not contain at least six oxygen atoms, which is the minimum required for a glucuronide conjugate. The remaining candidate with molecular formula $C_{20}H_{34}O_{10}$ leads to no match in SIRIUS/CSI:FingerID with a CSI:FingerID score better than -170 and none of the 51 proposed structures is a glucuronide.

SI-D5.3 Sulfate Conjugates: Positive Ionization Mode

Neutral loss search of sulfates in the positive ionization mode led to 43 matches, covering the previously identifed paracetamol-sulfate and tapentadol-O-sulfate. Ten components eluted after 30 minutes, leading to their exclusion. Additional seven components did not contain the required sulfur atom and additional five comopnents did not posses the required three oxygen atoms in the molecular formula. A compound with a structurally similar functional group to sulfate, a sulfonic acid, was identified as ensulizole, a sunscreen agent.[2] For the remaining 20 components, the best structural proposals did either not contain a sulfate group in the molecule or the compounds were sulfonic acids containing this group either naturally or by design but were unlikely to be the result of human phase II metabolism.

SI-D5.4 Sulfate Conjugates: Negative Ionization Mode

Neutral loss search of sulfates in the negative ionization mode revealed 474 matches. Of these, 79 contained no sulfur atom and 33 did not have enough oxygen atoms, while for further three components no molecular formula could be determined. Based on the retention time, no components could be excluded. One of the components is based on mzCloud and MassBank matches suspected to be hymecromone-sulfate, a metabolite of hymecromone, also known as 4-methylumbelliferyl. It is coumarin derivative used as choleretic and musculotropic antispasmodic. Moreoever, it is used as sunscreen agent and for fluoresence analyses.[32] A mirror plot of the MS2 spectrum from hymecromone-sulfate measured in wastewater and from mzCloud is shown in Figure SI-D513. Another identified compound is propachlor ESA, a transformation prodcut of propachlor, which is used as herbicide and biocide.[33] Ensulizole, the sunscreen agent containing

a sulfonic acid group,[2] which was previously identified in the positive ionization mode, was also detected in the negative ionization mode. A metabolite of dietary L-tryptophane, 3-indoxyl-sulfate, was detected.[34] A mirror plot of the MS2 spectrum measured in wastewater and of mzCloud are shown in Figure SI-D514. For the remaining 355 components, the best structural proposals did either not contain a sulfate group in the molecule or the compounds were sulfonic acids containing this group either naturally or by design but were unlikely to be the result of human phase II metabolism.

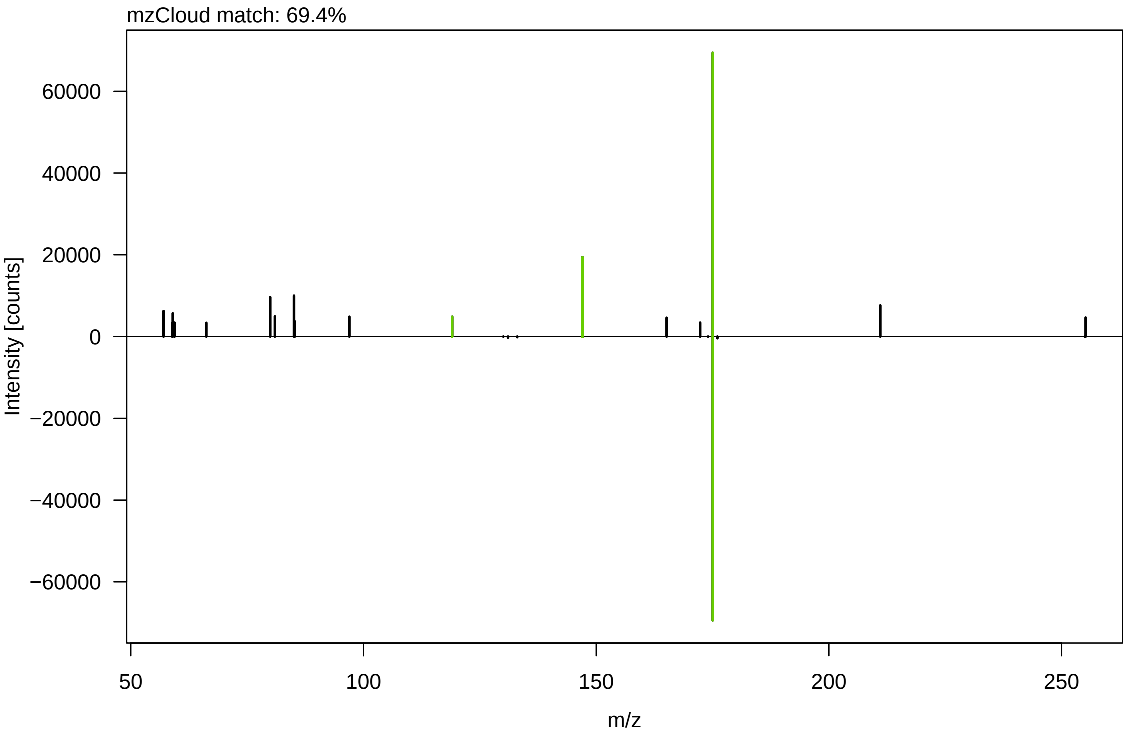

Figure SI-D513: Head to tail plot of measured MS2 spectrum against mzCloud library spectrum of hymecromone-sulfate. Matching fragments are highlighted in green.

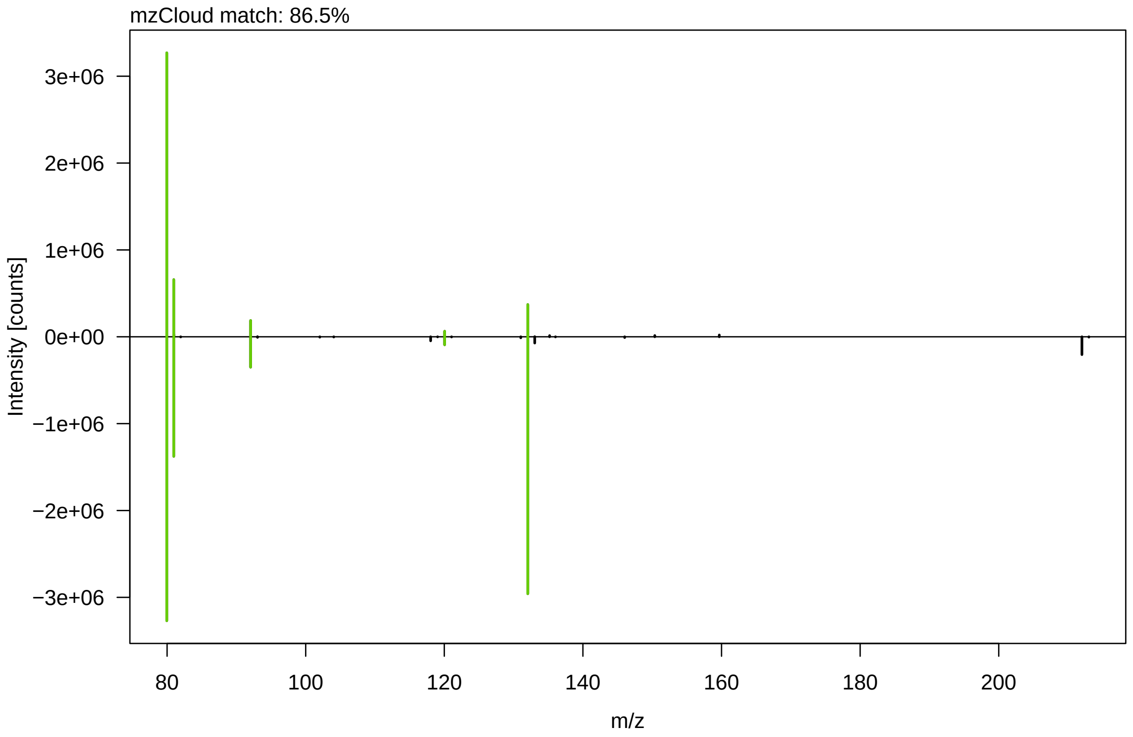

Figure SI-D514: Head to tail plot of measured MS2 spectrum against mzCloud library spectrum of 3-indoxyl-sulfate. Matching fragments are highlighted in green.

SI-D5.5 Glutathione Conjugates: Negative Ionization Mode

Specific fragment search of glutathiones in the negative ionization mode led to seven matches. None of them possessed the required types and numbers of atoms to be a glutathione conjugate.

References

[1] Swiss Compendium. https://compendium.ch/.

[2] DrugBank. https://go.drugbank.com/.

[3] Vögtli, A. PharmaWiki - Chinidin. 2013; https://www.pharmawiki.ch/wiki/index.php?wiki=chinidin.

[4] Grilo, N. M.; Antunes, A. M.; Caixas, U.; Marinho, A. T.; Charneira, C.; Conceição Oliveira, M.; Monteiro, E. C.; Matilde Marques, M.; Pereira, S. A. Monitoring abacavir bioactivation in humans: Screening for an aldehyde metabolite. *Toxicology Letters* **2013**, *219*, 59–64.

[5] Waldmeier, F.; Glaenzel, U.; Wirz, B.; Oberer, L.; Schmid, D.; Seiberling, M.; Valencia, J.; Riviere, G. J.; End, P.; Vaidyanathan, S. Absorption, distribution, metabolism, and elimination of the direct renin inhibitor aliskiren in healthy volunteers. *Drug Metabolism and Disposition* **2007**, *35*, 1418–1428.

[6] Zhu, Y.; Wang, F.; Li, Q.; Zhu, M.; Du, A.; Tang, W.; Chen, W. Amlodipine metabolism in human liver microsomes and roles of CYP3A4/5 in the dihydropyridine dehydrogenation. *Drug Metabolism and Disposition* **2014**, *42*, 245–249.

[7] Riedmaier, S.; Klein, K.; Winter, S.; Hofmann, U.; Schwab, M.; Zanger, U. M. Paraoxonase (PON1 and PON3) polymorphisms: Impact on liver expression and atorvastatin-lactone hydrolysis. *Frontiers in Pharmacology* **2011**, *JUL*, 1–20.

[8] Hunter, R. P.; Koch, D. E.; Coke, R. L.; Goatley, M. A.; Isaza, R. Azithromycin metabolite identification in plasma, bile, and tissues of the ball python (Python regius). *Journal of Veterinary Pharmacology and Therapeutics* **2003**, *26*, 117–121.

[9] Godos, J.; Pluchinotta, F. R.; Marventano, S.; Buscemi, S.; Volti, G. L.; Galvano, F.; Grosso, G. Coffee components and cardiovascular risk: Beneficial and detrimental effects. *International Journal of Food Sciences and Nutrition* **2014**, *65*, 925–936.

[10] Vögtli, A. Diosmin und Hesperidin. 2023; https://www.pharmawiki.ch/wiki/index.php?wiki=DiosminundHesperidin.

[11] Jin, M. J.; Kim, U.; Kim, I. S.; Kim, Y.; Kim, D. H.; Han, S. B.; Kwon, O. S.; Yoo, H. H. Effects of gut microflora on pharmacokinetics of hesperidin: A study on non-antibiotic and pseudo-germ-free rats. *Journal of Toxicology and Environmental Health - Part A: Current Issues* **2010**, *73*, 1441–1450.

[12] Chando, T. J.; Everett, D. W.; Kahle, A. D.; Starrett, A. M.; Vachharajani, N.; Shyu, W. C.; Kripalani, K. J.; Barbhaiya, R. H. Biotransformation of irbesartan in man. *Drug Metabolism and Disposition* **1998**, *26*, 408–417.

[13] Liu, Y.; Wang, H.; Cheng, Y.; Sun, J.; Qiao, J.; Lu, H.; Zhu, L.; Gong, L.; Ren, J. A 26-week repeated-dose toxicity study of allisartan isoproxil in Sprague-Dawley rats. *Drug and Chemical Toxicology* **2013**, *36*, 443–450.

[14] Fan-Havard, P.; Liu, Z.; Chou, M.; Ling, Y.; Barrail-Tran, A.; Haas, D. W.; Taburet, A. M. Pharmacokinetics of phase i nevirapine metabolites following a single dose and at steady state. *Antimicrobial Agents and Chemotherapy* **2013**, *57*, 2154–2160.

[15] US EPA Estimation Programs Interface Suite (EPI Suite). 2012.

[16] Quasthoff, S.; Möckel, C.; Zieglgänsberger, W.; Schreibmayer, W. Tolperisone: A typical representative of a class of centrally acting muscle relaxants with less sedative side effects. *CNS Neuroscience and Therapeutics* **2008**, *14*, 107–119.

[17] Begoña Barroso, M.; Alonso, R. M.; Jiménez, R. M. Simultaneous determination of torasemide and its major metabolite M5 in human urine by high-performance liquid chromatography-electrochemical detection. *Journal of Chromatographic Science* **2001**, *39*, 491–496.

[18] Roche, V. F. The chemically elegant proton pump inhibitors. *Am J Pharm Educ* **2006**, *70*.

[19] LeBel, M. Pharmacokinetic Properties of Clarithromycin: A Comparison with Erythromycin and Azithromycin. 1993.

[20] *Some Chemicals Present in Industrial and Consumer Products, Food and Drinking-Water.*; 2013.

[21] Tretter, L.; Patocs, A.; Chinopoulos, C. Succinate, an intermediate in metabolism, signal transduction, ROS, hypoxia, and tumorigenesis. *Biochimica et Biophysica Acta - Bioenergetics* **2016**, *1857*, 1086–1101.

[22] Perfumers World. 2021; `https://www.perfumersworld.com/view.php?pro_id=1EN13109`.

[23] Foodb. 2019; `https://foodb.ca/compounds/FDB010504`.

[24] Catalysts, S.; Ravasio, N.; Marelli, M.; Evangelisti, C. catalysts The Role of Support Hydrophobicity in the Selective The Hydrophobicity Selective and Unsaturated. **2020**,

[25] Dsikowitzky, L.; Schwarzbauer, J. Hexa(methoxymethyl)melamine: An Emerging Contaminant in German Rivers. *Water Environment Research* **2015**, *87*, 461–469.

[26] De Hoogh, C. J.; Wagenvoort, A. J.; Jonker, F.; Van Leerdam, J. A.; Hogenboom, A. C. HPLC-DAD and Q-TOF MS techniques identify cause of Daphnia biomonitor alarms in the River Meuse. *Environmental Science and Technology* **2006**, *40*, 2678–2685.

[27] Dsikowitzky, L.; Schwarzbauer, J. Hexa(methoxymethyl)melamine: An Emerging Contaminant in German Rivers. *Water Environment Research* **2015**, *87*, 461–469.

[28] Scentree - Methyl Anthranilate. `https://www.scentree.co/en/Methyl_Anthranilate.html`.

[29] Tiwari, A.; Mahadik, K. R.; Gabhe, S. Y. Piperine: A comprehensive review of methods of isolation, purification, and biological properties. *Medicine in Drug Discovery* **2020**, *7*, 100027.

[30] Perfumers World - Sulfurol. `https://www.perfumersworld.com/view.php?pro_id=4ED00423`.

[31] The Good Scents Company - Sulfurol. `http://www.thegoodscentscompany.com/data/rw1023221.html`.

[32] Heber, D.; Berger, M.; Imming, C. Hymecromon. 2021; `https://roempp.thieme.de/search;;searchterm=Hymecromon?search=Hymecromon`.

[33] Propachlor ESA. `https://pubchem.ncbi.nlm.nih.gov/compound/Propachlor-ESA`.

[34] Zhang, L. S.; Davies, S. S. Microbial metabolism of dietary components to bioactive metabolites: Opportunities for new therapeutic interventions. *Genome Medicine* **2016**, *8*, 1–18.
